# Predictions of Cleavability of Calpain Proteolysis by Quantitative Structure-Activity Relationship Analysis Using Newly Determined Cleavage Sites and Catalytic Efficiencies of an Oligopeptide Array* [Fn FN2]

**DOI:** 10.1074/mcp.M115.053413

**Published:** 2016-01-21

**Authors:** Fumiko Shinkai-Ouchi, Suguru Koyama, Yasuko Ono, Shoji Hata, Koichi Ojima, Mayumi Shindo, David duVerle, Mika Ueno, Fujiko Kitamura, Naoko Doi, Ichigaku Takigawa, Hiroshi Mamitsuka, Hiroyuki Sorimachi

**Affiliations:** From the ‡Calpain Project, Department of Advanced Science for Biomolecules, and; §The Advanced Technical Support Department, The Basic Technology Research Center, Tokyo Metropolitan Institute of Medical Science (IGAKUKEN), 2-1-6 Kamikitazawa, Setagaya-ku, Tokyo 156-8506, Japan;; ¶Graduate School of Frontier Sciences, The University of Tokyo, Kashiwa, Chiba 277-8561, Japan;; ‖Graduate School of Information Science and Technology, Hokkaido University, Kita 14, Nishi 9, Kita-ku, Sapporo, Hokkaido 060-0814, Japan;; **Bioinformatics Center, Institute for Chemical Research, Kyoto University, Uji, Kyoto 611-0011, Japan

## Abstract

Calpains are intracellular Ca^2+^-regulated cysteine proteases that are essential for various cellular functions. Mammalian conventional calpains (calpain-1 and calpain-2) modulate the structure and function of their substrates by limited proteolysis. Thus, it is critically important to determine the site(s) in proteins at which calpains cleave. However, the calpains' substrate specificity remains unclear, because the amino acid (aa) sequences around their cleavage sites are very diverse. To clarify calpains' substrate specificities, 84 20-mer oligopeptides, corresponding to P10-P10′ of reported cleavage site sequences, were proteolyzed by calpains, and the catalytic efficiencies (*k_cat_*/*K_m_*) were globally determined by LC/MS. This analysis revealed 483 cleavage site sequences, including 360 novel ones. The *k_cat_*/*K_m_*s for 119 sites ranged from 12.5–1,710 M^−1^s^−1^. Although most sites were cleaved by both calpain-1 and −2 with a similar *k_cat_*/*K_m_*, sequence comparisons revealed distinct aa preferences at P9-P7/P2/P5′. The aa compositions of the novel sites were not statistically different from those of previously reported sites as a whole, suggesting calpains have a strict implicit rule for sequence specificity, and that the limited proteolysis of intact substrates is because of substrates' higher-order structures. Cleavage position frequencies indicated that longer sequences N-terminal to the cleavage site (P-sites) were preferred for proteolysis over C-terminal (P′-sites). Quantitative structure-activity relationship (QSAR) analyses using partial least-squares regression and >1,300 aa descriptors achieved *k_cat_*/*K_m_* prediction with *r* = 0.834, and binary-QSAR modeling attained an 87.5% positive prediction value for 132 reported calpain cleavage sites independent of our model construction. These results outperformed previous calpain cleavage predictors, and revealed the importance of the P2, P3′, and P4′ sites, and P1-P2 cooperativity. Furthermore, using our binary-QSAR model, novel cleavage sites in myoglobin were identified, verifying our predictor. This study increases our understanding of calpain substrate specificities, and opens calpains to “next-generation,” *i.e.* activity-related quantitative and cooperativity-dependent analyses.

Calpains (Clan CA, family C02; EC 3.4.22.17) are major, Ca^2+^-regulated, intracellular proteases (1–3). The most-studied calpains are mammalian calpain-1 (C1)^1^ and calpain-2 (C2), which are called the “conventional” calpains (in this paper, “calpains” refers to the conventional calpains unless otherwise indicated). C1 and C2 each forms a heterodimer composed of a larger (∼80 kDa) catalytic subunit (CAPN1 or CAPN2) and a common smaller (∼28 kDa) regulatory subunit (CAPNS1). Because CAPN1 and CAPN2 have more than 60% aa sequence identity, C1 and C2 show highly similar, if not identical, substrate specificities (1, 4–6). They generally function by limited proteolysis, cleaving a few peptide bonds in their substrate protein, which changes the protein's function and/or structure to modulate cellular functions. Thus, calpains are called “modulator proteases.” To understand the calpains' physiological functions, it is essential to clarify their substrate specificity/selectivity, *i.e.* what proteins calpains proteolytically process and at which position(s).

There have been many attempts to define calpains' substrate specificities. The initial studies, focusing on whether specific proteins are proteolyzed or not (6–9), were followed by more detailed studies using substrate cleavage site amino acid (aa) sequence alignment and a position-specific scoring matrix (PSSM) method (10–12). Next, peptide libraries were used (13, 14). For example, Cuerrier and his colleagues used a peptide sequencing method to quantitatively determine calpains' preference for each aa residue (aar) at each position relative to the cleavage site (13), and developed a sensitive oligopeptidyl fluorescence substrate, H-E(EDANS)PLFAERK(DABCYL)-OH. More recently, machine-learning methods have been applied to the construction of calpain cleavage predictors (15–20).

However, PSSM-based and machine-learning methods have so far yielded rather limited accuracy in predicting calpain cleavage sites. This is because, unlike with caspases and granzymes (19), there appears to be no explicit rule for calpain specificity, and the number of known aa sequences for calpain cleavage sites is rather small (< 200, before this study). Furthermore, the cleavage efficiency of most of the reported calpain cleavage sites is unknown, and the cleavage patterns change depending on the reaction conditions.

Notably, the most important question in identifying cleavage specificity is not whether a protein is cleaved. Technically, all peptide bonds can be cleaved by calpains (or any protease) with some efficiency, *i.e. k_cat_/K_m_* > 0, which depends on the cleavage conditions. In other words, the apparent “cleavability” of a bond is defined by the threshold *k_cat_/K_m_* determined by both the proteolytic conditions and the detection sensitivity. Therefore, the ultimate cleavage predictor should predict a *k_cat_/K_m_* value for each peptide bond within a given protein sequence under given cleavage conditions.

To address the above points, here we sought to identify calpain cleavage-site sequences through literature searches and by performing *in vitro* digestions of a concentrated, synthesized oligopeptide library. Using the identified cleavage-site sequences, we performed quantitative structure-activity relationship (QSAR) analyses, which revealed the important P- and P′-site positions (the positions N- and C-terminal to the cleavage site, respectively) on which to focus. Although the reaction conditions used in this study were slightly different from those used in typical calpain kinetics studies, several verification analyses confirmed that our results successfully elucidated the calpains' substrate specificity.

## EXPERIMENTAL PROCEDURES

### 

#### 

##### Peptides and Calpains

From 116 reports, 147 calpain substrates, and their 420 cleavage-site sequences (after excluding two overlapping sequences from a total of 422) were collected (supplemental Table S1). The substrate proteins were numbered SB0001 to SB0150 (substrates reported multiple times under different conditions were assigned different SB numbers; see supplemental Table S1), among which SB0001-SB0090 were already reported in our previous paper (15)). Next, a database, CaMP DB (Calpain for modulatory proteolysis database (21), http://www.calpain.org/), was constructed from the collected information, including all the cleavage sites, secondary structures, and references.

From the above collected site sequences, 86 were selected according to their position in the substrate protein (to have 10 or more P and P′ site aars) and aa composition (to be not too hydrophobic), and the 20 aars surrounding the reported calpain cleavage site (10 on each side of the site) were selected for oligopeptide sequence preparation (there were several exceptions; see supplemental Table S2). Eight (ID031, 34, 36, 37, 55, 72, 73, and 84) of these 86 sequences were randomly selected, scrambled, and used as control peptides (ID087–94) (supplemental Table S2).

A total of 94 (93 20mer- and one 19mer-; 86 selected plus 8 scrambled sequences) oligopeptides were then synthesized with N-terminal acetylation (Ac) and C-terminal diketopiperadinylation (DKP), by the PepSets^TM^ Peptide Library synthesis service (Mimotopes, Victoria, Australia). Each peptide (2 mg) was independently dissolved in 0.4 ml sterile 0.1% acetic acid (AcOH) by sonication. For peptides that remained undissolved, 40 μl of AcOH and 110 μl of acetonitrile (MeCN) were successively added until the peptide dissolved. By these procedures, all but two of the peptides (ID051 and 89) were mostly dissolved (*ca*. 5 mg/ml [2 mm]). Next, 100 fmol of each peptide in Matrix solution (4 mg/ml α-cyano-4-hydroxycinnamate, 80 μg/ml ammonium citrate, 0.1% TFA, 70% MeCN) was spotted onto a MALDI target plate and subjected to MS using the 4800 MALDI-TOF/TOF system (Sciex, Framingham, MA). An equal mixture of all 94 peptides was then prepared, and the volume was adjusted to contain each peptide at 0.1 mm. This peptide library was named “P94mix.” No signals, however, corresponding to peptides ID001, 3, 23, 27, 51, 80, and 82 were detected in the preparatory experiments (see below). Therefore, 87 peptides (P94mix minus the seven nondetected peptides; named “P87mix”) were remixed, neutralized by 25% ammonia water, dried, dissolved to 0.1 mm (8.7 mm as the total peptide concentration) in 0.5% AcOH, 2% MeCN, and used for the kinetics study. Recombinant human C1 and C2 were produced using the baculovirus/Sf9 expression system, as previously described (22, 23). A commercially available C1 (Merck Millipore, Billerica, MA, #208712) was also used.

##### Preparatory Experiments

P94mix (0.5–20 μm each peptide) was digested with 50 nm–2.5 μm C1 or C2 in 50–100 mm HEPES (pH 7.5), 1 mm tris(2-carboxyethyl)phosphine (TCEP), and 1 mm or 5 mm CaCl_2_, respectively, (or 1 mm EDTA for negative controls for both calpains) at 30 °C for 0–20 min. The resulting reaction mixture was subjected to two-dimensional (2D)-LC-MALDI MS using the DiNa 2D nLC-spotting system (KYA Technologies Co., Tokyo, JAPAN) and a Sciex 4800 Proteomics Analyzer as previously reported (24, 25). MS and MS/MS spectra were acquired with 4000 series Explorer Ver. 3.5 software (Sciex).

In preliminary experiments, most of the peptides were detected as either or both of the following: (1) uncleaved (*i.e.* both N- and C termini capped with Ac and DKP, respectively [both-capped, BC]) peptides that were synthesized correctly and/or in truncated form; (2) fragments cleaved as previously reported (Rp), and/or not as reported (*i.e.* novel, Nv). The time course of the signals indicated that the optimal reaction time for most of the peptides was between 10 and 20 min (data not shown). Thus, the reaction time was set to 15 min for subsequent experiments. To maximize the number of cleaved peptides, the peptide concentration was increased to 1.7 mm (20 μm each) in the reaction mixture. After testing several combinations of peptides and calpains, we decided to use 0.3–1.7 mm (3.3–20 μm each) peptides and 2.5 μm calpains in the following kinetics study. The ratio of calpain to each peptide was high compared with typical calpain proteolysis experiments. The most likely reason for the high calpain requirement is that the calpain activity was inhibited by impurities derived from the peptide synthesis process and by the high ionic concentration of the reaction mixture, which was because of the need for excess buffer to neutralize acetic acid present in peptide solvents. Although these assay conditions may not have been optimal for peptides with high-end and low-end *k_cat_/K_m_* values, they appeared to be appropriate for most of the peptides (see supplemental Fig. S1).

Among the clearly detected proteolytic fragments obtained by cleavages at Rp sites, oligopeptides corresponding to 78 C-terminal and 26 N-terminal fragments were newly synthesized with C-terminal DKP or N-terminal Ac modification, respectively, as described above (supplemental Table S3, ID0XX-Rp-C or -N series). Peptides corresponding to 39 (C-terminal) and 15 (N-terminal) fragments obtained by cleavages at Nv sites were also synthesized (supplemental Table S3, ID0XX-Nv series). These peptides (158 total peptides, named “P158mix”) were used to quantify the generated calpain-cleaved peptides in the following kinetics experiments.

##### Peptide Proteolysis and MS Analysis

**Fig. 1.**
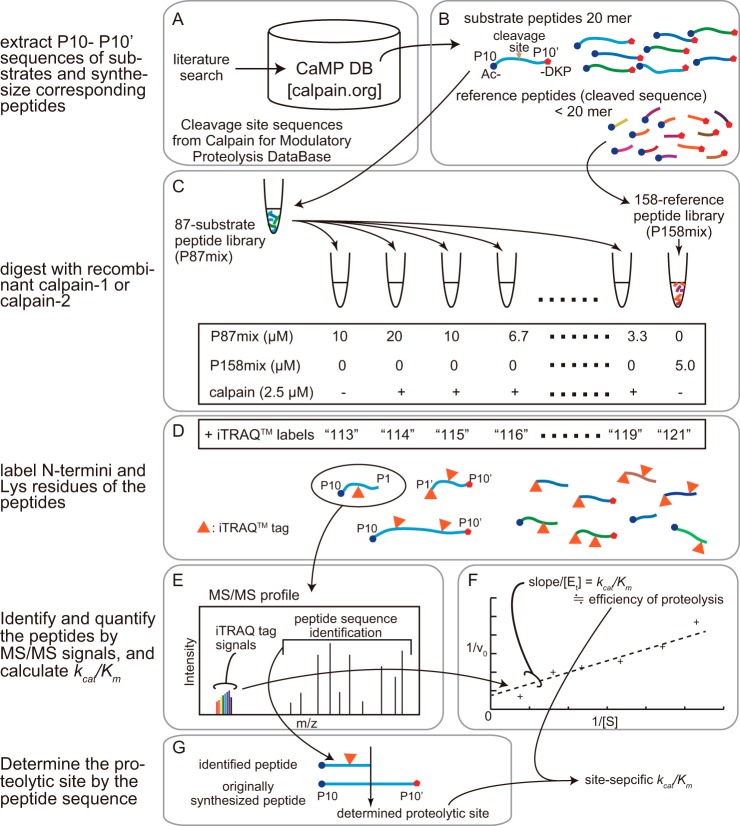
**Scheme of the experiments in this study.** First, 420 independent calpain cleavage site sequences of 143 substrate proteins were manually collected from 114 references by the authors, and their cleavage data were confirmed (see supplemental Table S1). These data were summarized in the CaMP (Cleavage site sequences from ***Ca***lpain for ***M***odulatory ***P***roteolysis) database (DB) web site (*A*). Next, 86 sequences corresponding to the P10-P10′ of some of the above cleavage sites and 8 control scrambled sequences were selected for oligopeptide synthesis (P94mix) with the N- and C terminus capped by acetyl- and -DKP modifications, respectively (*B*). Shorter reference peptides corresponding to segments created by calpain cleavage were also synthesized (P158mix). Next, varying amounts of P87mix (7 peptides were excluded from P94mix because of insolubility and other reasons) were incubated with or without C1 or C2 at 30 °C for 15 min (*C*). After the digestion, peptide solutions were labeled with iTRAQ^TM^ reagents (*D*), and peptides that were cleaved or uncleaved (*i.e.* with both terminals capped) were identified and quantified by liquid chromatography-combined with MS (*E*). Finally, the *v*_0_ (initial velocity of the cleavage reaction) values were calculated from the iTRAQ^TM^ signals, and 1/*v*_0_ was plotted against 1/[S] (where [S] was the substrate concentration) to determine the *k_cat_/K_m_* value for each cleavage (*F*). The identified peptide sequence was compared with the originally synthesized peptide sequence to determine the proteolytic site by calpains (*g*) associated with the determined *k_cat_/K_m_*.

P87mix (for final concentrations, see Table I) in 100 mm HEPES (pH 8.5) and 1 mm TCEP was denatured at 60 °C for 1 h, and digested with 2.5 μm C1 or C2 in the presence of 1 mm or 5 mm CaCl_2_, respectively, at 30 °C for 15 min in a 20-μl volume (see Fig. 1 for the overview of the experiments). As a standard for quantification of the cleaved peptides, P158mix (each peptide at 5 μm) was incubated under the same conditions, without calpains. After the reaction, TCEP, SDS, triethylammonium bicarbonate, and three control peptides for iTRAQ^TM^ standardization (C001: NH_2_-EFILRVFSEKRNL-COOH, *M*_r_ 1,649.93; C002: NH_2_-DFCIRVFSEKKAD-COOH, *M*_r_ 1,556.77; C003: NH_2_-DFVLRFFSEKSAG-COOH, *M*_r_ 1,501.76) were added to final concentrations of 4.36 mm, 0.0952%, 167 mm, and 0.5 μm each, respectively, and denatured at 60 °C for 1 h.

**Table I:** iTRAQ^TM^-8plex labeling mixtures for the kinetics study

iTRAQ^TM^ 8plex reagent	113	114	115	116	117	118	119	121
P87mix (μm each; μm total)	10; 870	20; 1,700	10; 870	6.7; 580	5.0; 440	4.0; 350	3.3; 290	0; 0
P158mix (μm each)	0	0	0	0	0	0	0	5.0
C1 or C2 (μm)	0	2.5	2.5	2.5	2.5	2.5	2.5	0

Next, methyl methanethiosulfonate was added to a concentration of 8.33 mm; the reaction mixture was then incubated at room temperature for 10 min, and labeled with the iTRAQ^TM^ 8-plex labeling kit (Sciex), according to the manufacturer's instructions (Table I). The resulting reaction mixture was subjected to 2D-LC-MALDI MS as described above. The same sample was also analyzed by 2D-LC/MS using the DiNa 2D nLC system and Sciex QSTAR Elite with NanoSpray^TM^ ESI. MS and MS/MS spectra were acquired with Analyst QS Ver. 2.0 software (Sciex), using the standard parameters recommended by the manufacturer. Peptides were identified using ProteinPilot^TM^ Ver.4.5 with the following Paragon parameters: Sample Type: iTRAQ 8plex (Peptide Labeled); Cys Alkylation: MMTS; Digestion: None; Instrument: QSTAR Elite ESI or 4800; Special Factors: “N-Ac and C-DKP” or “N-Ac and C-DKP, cleavable” (see below); Species: None; Specify Processing: check in Quantitate, Bias Correction, Background Correction, Biological modifications; Search Effort: Thorough ID; Results Quality: Detected Protein Threshold > 0.05 and Run False Discovery Rate Analysis (Threshold > 0.05 is recommended by the manufacturer, and the FDR was calculated automatically by ProteinPilot^TM^); Database: Hs4K DB (normal condition) or Hs50K DB (stringent condition). (For the database construction, see below.) Peak lists were generated by ProteinPilot^TM^ Ver.4.0 (Sciex).

“N-Ac and C-DKP” and “N-Ac and C-DKP, cleavable” were added by describing them in the ParameterTranslation.xml and ProteinPilot.DataDictionary.xml files of the ProteinPilot^TM^ software (see Supplemental Experimental Procedures for the description). The database was constructed as described below. A global false discovery rate (FDR) above 5% (normal condition) or 1% (stringent condition) was used to define significant data. Identified peptides were exported as PeptideSummary.txt for further data processing by Microsoft Excel Ver. 2010. Peptide structures and their proteolytic sites were assigned according to whether Ac and/or DKP was present (see supplemental Experimental Procedures).

##### Database Search

The core sequence database (“Core DB”) was constructed using the sequences in the P87mix, control peptides (C001–3), and human calpains (CAPN1 [38–714 aar], CAPN2 [30–700], and CAPNS1 [1–34, 55–268]; because sequences of parts of CAPN1, 2, and S1 [1–37, 1–29, and 35–54, respectively] were included in some of the P87mix entries [ID081, 83, 84, and 85], these sequence regions were deleted for the CAPN1, 2 and S1 entries), resulting in 93 entries and 3,460 aars. To identify peptides, the Core DB was combined with unrelated sequences retrieved from human proteome sequences (IPI_human protein database Ver.3.87, 91,464 entries, 36,355,611 aars) as follows, for reliable FDR selection (at least 500 [preferably 4,000] entries in the database are recommended by the manufacturer).

First, the C-terminal 20 aars were selected from the proteome database entries that had 20 or more aars, resulting in 90,858 entries (1,817,160 aars). Among these entries, those similar to Core DB entries when reversed, *i.e.* entries whose reverse sequence contained a four-aa block included among the Core DB sequences, were eliminated, to construct “Hs50K DB” (50,330 entries, 1,006,600 aars). Next, forward sequences containing a four-aa block included in the Core DB were also eliminated, reducing the number of entries to 30,317. From the remaining entries, 4,000 were randomly selected, resulting in “Hs4K DB” (4,000 entries, 800,000 aars). “Core DB + Hs50K DB and FDR < 1%”, and “Core DB + Hs4K DB and FDR < 5%” were used as the “stringent” and the “normal” condition, respectively. In this study, the reported results were obtained under the normal condition, because both conditions gave essentially the same results (see supplemental Fig. S5*C*).

##### Kinetics

A *k_cat_*/*K_m_* value for each cleavage was calculated using Lineweaver-Burk and Eadie-Hofstee plots. A comparison of the results revealed that the former gave much better estimations than the latter (data not shown), so the Lineweaver-Burk method was used. A *k_cat_*/*K_m_* value for each cleavage was calculated as 1/**b**/[E]_t_, where [E]_t_ was the concentration of calpain (2.5 μm); and **b** was the slope of the regression line obtained when 1/*v*_0_ (*y* axis) was plotted against 1/[S]_0_ (*x* axis), where [S]_0_ was the initial substrate concentration (3.3–20 μm) and *v*_0_ was the initial velocity of the cleavage reaction. For full-length peptides, *v*_0_ was calculated as ([S]_0(n)_ - I_n_/I_113_ × [S]_0(113)_)/900 s, where I_n_ and [S]_0(n)_, respectively, were the iTRAQ^TM^ signal intensities (standardized by those of control peptides) of iTRAQ^TM^-n (*n* = 113, 114, 115, 116, 117, 118, or 119) and [S]_0_ corresponding to the iTRAQ^TM^-n label (n: 113→1.0 × 10^−5^ m, 114→2.0 × 10^−5^ m, 115→1.0 × 10^−5^ m, 116→6.7 × 10^−6^ m, 117→5.0 × 10^−6^ m, 118→4.0 × 10^−6^ m, 119→3.3 × 10^−6^ m).

For cleaved fragments, *v*_0_ was calculated as I_n_/I_121_ × 5 × 10^−6^ m/900 s, where I_121_ was the iTRAQ^TM^ signal intensity (standardized by those of control peptides) of iTRAQ^TM^-121, which corresponded to 5 μm standard fragment peptides. In general, calculations using the full-length values showed considerably larger variance than those obtained using the fragments. This may have been due to the somewhat high variances among the iTRAQ^TM^ signals, and to their narrow dynamic range, as well as to unknown reasons. As verified in supplemental Fig. S1, *k_cat_/K_m_* values could be calculated with moderate errors, and the amounts of full-length peptides remaining after the reaction were smoothly distributed, supporting the appropriateness of the reaction time (15 min) in this study. For the rationale for calculating *k_cat_*/*K_m_*, see supplemental Experimental Procedures.

##### Determination of Cleavage Sites by N-terminal Sequencing and MS/MS Analysis

Human heart troponin T2 (Merck 648484–100UGA, *ca*. 30 pmol) and horse myoglobin (Sigma-Aldrich, M0630, *ca*. 60 pmol) were digested with C1 (Merck Millipore #208712, 0.9 pmol) in 50 μl of 100 mm Tris-HCl (pH 7.5), 1 mm DTT, and 5 mm CaCl_2_ at 30 °C for 20 min. The digested samples were directly separated by SDS-PAGE, and the proteolyzed fragments were then blotted onto a PVDF membrane and subjected to peptide sequencing analysis (AproScience Inc., Tokushima, Japan). For sequence analysis by MS, the same digestion reactions were performed, terminated by adding a 3-fold volume of 7% TCA followed by incubation on ice for 30 min, spun (20,000 × *g*, 2 °C, 10 min), and the supernatant was collected. An aliquot of the soluble fraction was desalted and concentrated to a few μl using Zip-Tip C-18, and analyzed by Sciex 5600^+^ with the Eksigent nanoLC system. The samples were analyzed in triplicate, the data were merged, and the peptide sequences were identified using ProteinPilot (Ver. 4.5) and Swiss-Prot DB (2015_08; 549,008 sequences; 195,692,017 aars) using the default parameters.

##### Determination of Cleavability of Synthetic Peptides by nLC

Peptides [tp1: Ac-QHLCGSHLVEALYLVCGERG (corresponding to ID014: INS); tp2: LEGNLYGSLFSVPSSKLLGN (ID040: GRIN2A), and tp3: GGGGYSASLHSEPPVYANLS (ID048: JUN)] for nLC analysis were synthesized and purified by Toray Research Center Inc. (Tokyo, Japan) with > 98% purity (determined by the manufacturer from the ratio of peak areas in HPLC), and were dissolved in distilled water. Each peptide (initial concentration: 6.7–20 μm) was incubated with 1 pmol of either C1 (Merck Millipore #208712) or C2 in 50 μl of 50 mm HEPES (pH 7.5), 1 mm TCEP, and 1 or 5 mm CaCl_2_ at 30 °C for 20 min. The digested sample was directly separated by DiNa nanoLC and monitored by a UV spectroscope MU701 (GL Sciences, Tokyo, Japan). Each peak sample was collected, and the contained peptide was determined by the Sciex 4800 MALDI MS system as described above. The areas of peaks were quantified using SmartChrom data analysis software Ver. 2.28J (KYA).

##### Statistics and QSAR Calculations

Statistical tests were performed using Excel 2010 (Microsoft), SAS Studio Release 3.1 of the SAS University Edition (SAS Institute Inc., Cary, NC), and Molecular Operating Environment (MOE, Ver. 2013.08, Chemical Computing Group Inc., Montoreal, Quebec, and Ryoka Systems Inc., Tokyo, Japan). Analyses for 3D structures and model constructions using the partial least squares (PLS) and binary-QSAR methods were performed by MOE.

A binary-QSAR model was constructed by Auto-QSAR (binary) of MOE software using default parameters and 812 aa descriptors at specific positions. The aa descriptors used were 3 secondary structure descriptors for each position (total of 3 × 20 = 60) and those that showed the largest *r*^2^ values between the measured *k_cat_/K_m_*s and the corresponding aa descriptor's values (see supplemental Tables S11-S13). In the binary QSAR analysis, all of the cleaved and uncleaved sequences without measured *k_cat_/K_m_* values were assigned values of 1 and 0 m^−1^s^−1^, respectively, and a cut-off value of 0.5 m^−1^s^−1^ was used so that all of the cleaved and uncleaved sequences were set as positive and negative samples, respectively. First, P10-P10′ aars, which contained many missing aars close to both ends, were used for the construction. This resulted in a classification that placed unusual emphasis on whether an aar was missing or not, which was considered artifactual. Thus, only cleavage sequences with no missing aars in the varying ranges (P10-P10′, P9-P9′, P8-P8′, …) were used and tested. The trajectory of backward variable selection was analyzed manually, and the most balanced model was selected as having a leave-one-out (LOO) cross-validated accuracy (XA) of more than 0.7 and the lowest number of descriptors. The best model was found using the range P6-P6′ with eight descriptors (see Table III), which achieved a LOO XA of 74.9% (sensitivity [TP/(TP+FN), where TP = true positive and FN = false negative]: 57.3%; specificity [TN/(TN+FP), where TN = true negative and FP = false positive]: 86.2%).

A PLS-QSAR model was constructed by Auto-QSAR (PLS) in the MOE software using default parameters and the same 812 aa descriptors at specific positions as above. After the first analysis, the calculated outliers were excluded by MOE, and the analysis was performed again. The trajectory of backward variable selection was analyzed manually, and the most balanced model, with eight descriptors, was selected as having an *r*^2^ value cross-validated with LOO (*Xr*^2^) of more than 0.6 and the lowest number of descriptors (see Table V).

For the standard aa compositions, the following values taken from Swiss-Prot DB release 2012_9 were used: Ala, 8.67; Cys, 1.26; Asp, 5.32; Glu, 6.17; Phe, 4.01; Gly, 7.10; His, 2.21; Ile, 5.96; Lys, 5.25; Leu, 9.92; Met, 2.46; Asn, 4.09; Pro, 4.71; Gln, 3.95; Arg, 5.46; Ser, 6.66; Thr, 5.57; Val, 6.77; Trp, 1.30; Tyr, 3.03 (%).

## RESULTS

### 

#### 

##### Literature Search and Peptide Library Digestion Followed by MS Detection Identified 420 and 483 Calpain Cleavage Sites, Respectively

One of the major reasons for the previously incomplete accuracy of calpain cleavage predictors (15–20) is the small number of positive (*i.e.* cleavage site sequence) samples. To increase the number of samples, we first searched the literature extensively for calpain cleavage site sequences, and picked up 420 sites from 147 substrates (supplemental Table S1).

To ensure that the reported (Rp) cleavage sites would be cleaved in the oligopeptide context, a mixture of oligopeptides (P87mix library), each of which corresponded to one of the above cleavage sites, was proteolyzed by either C1 or C2. The digests were then analyzed by LC/MS for the global identification of cleavage site sequences. In this analysis, most of the Rp sites (*i.e.* mostly the middle of each peptide) as well as many novel (Nv) sites were identified. Therefore, for the kinetics study (see below), peptides corresponding to some of the identified cleavage fragments (104 Rp and 54 Nv sites) were synthesized (P158mix library, supplemental Table S3).

Finally, 418 cleavage sites (106 Rp and 312 Nv) were identified for C1, 360 (107 Rp and 253 Nv) for C2, and a total of 483 (123 Rp and 360 Nv) for both combined (Tables II, supplemental Tables S7 and S8). In total, we found that 98 of the 131 Rp sites existing in the P87mix were proteolyzed by calpains (74 (out of 131) Rp sites were in the middle of the peptide [*i.e.* after position 10], and 70 of these were proteolyzed), even using oligopeptides (supplemental Table S4), indicating that the calpain substrate specificity was consistent and validating our experimental system.

**Table II:** Summary of sites and IDs in the P87mix library identified for C1 and/or C2 under the normal or stringent condition For ID numbers and more details about the identifications, see Tables S2 and S7, respectively.

	Normal			Stringent		
	C1	C2	C1+C2	C1	C2	C1+C2
**Site***^a^*	**418**	**360**	**483**	**253**	**257**	**317**
Spectrum = 1	119	103	120	74	96	88
≥2	299	257	363	179	161	229
≥3	240	189	300	145	117	191
**Rp***^b^*	106	107	123	83	87	97
Rp with *k_cat_/K_m_*	69	63	71	61	58	64
**Nv**	312	253	360	170	170	220
Nv with *k_cat_/Km*	47	44	48	39	40	44
**P87mix ID (max 87)**	**86**	**86**	**87**	**84**	**85**	**87**
Spectrum = 1	0	2	1	2	6	3
≥2	86	84	86	82	79	84
≥3	85	83	86	79	77	83

*^a^* Numbers in the top row of each section indicate the sum total, which is broken down in the numbers beneath.

*^b^* Some numbers of Rp sites are larger than the maximum ID number (87) because the peptides of some IDs had more than one Rp site. (ID004, 6, 11, 19, 24, *etc*., see supplemental Tables S2 and S8.)

##### All Cleavage Site Sequences Identified Using Oligopeptides Showed Similar Trends to Those Reported

**Fig. 2.**
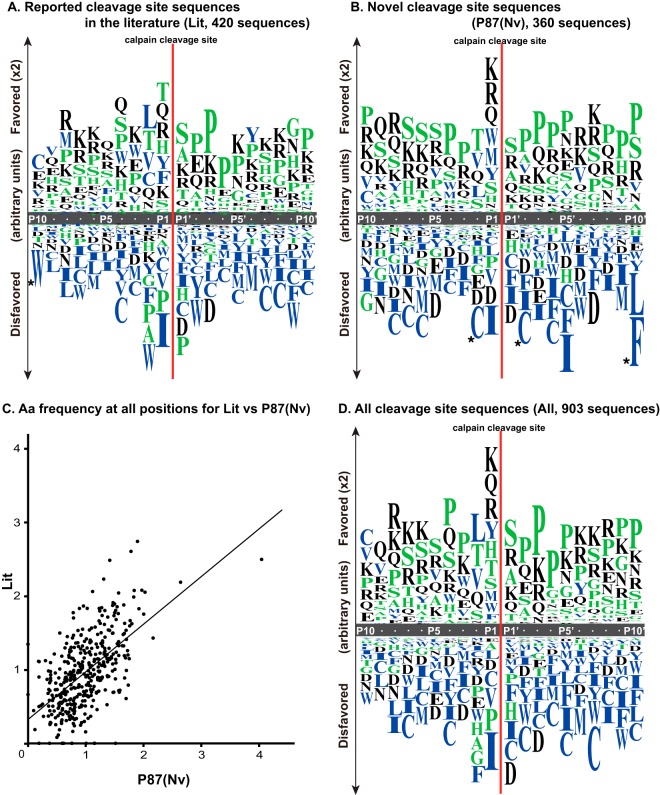
**Frequencies of amino acids proximal to the calpain cleavage sites.** P10-P10′ cleavage site sequences collected from the literature (*A*, “Lit”), novelly detected in our *in vitro* experiments (*B*, “P87(Nv)”), or the total identified in this study (*D*, “all”) were aligned. The occurrence of each aar (R) at each position was computed as follows: *r* = log([the ratio of the aar to all aars at each position]/[the standard composition ratio for that particular aar]). The total number of aars occurred at each positon was shown in supplemental Fig. S2*C*. Values are represented by the length of the letter abbreviation of each aar. The “favored” scale is doubled in length compared with the “disfavored” for easier visibility. The color of the aar letter indicates whether it is hydrophilic (Arg (R), Lys (K), Asp (D), Glu (E), Asn (N), or Gln (Q), black), neutral (Ser (S), Gly (G), His (H), Thr (T), Ala (A), or Pro (P), green), or hydrophobic (Tyr (Y), Val (V), Met (M), Cys (C), Leu (L), Phe (F), Ile (I), or Trp (W), blue). The aars marked by asterisks (Trp at P10 of Lit, and Cys at P2 and P2′, and Phe at P10′ of P87(Nv)) did not occur at all at these positions, and their height is not to scale. (*C*) The aa frequencies (standardized by the standard aa composition) at P10-P10′ of Lit and P87(Nv) were plotted. They showed significant correlation (*p* = 2.07 × 10^−38^, by *t* test for correlation coefficients) with an *r* of 0.587.

To examine whether the Nv site sequences were distinct from those of Rp sites, the P10-P10′ sequences for 420 sites from the literature (“Lit” sites) were compared with those of the 360 Nv sites identified above (Figs. 2*A*–2*C*). When the aa frequencies of all of the aars at all positions (P10-P10′) were compared for Lit and Nv, they showed significant correlation (*p* = 2.1 × 10^−38^), with a Pearson's correlation coefficient (*r*) of 0.59 (Fig. 2*C*). Although the *r* at each position varied from less than 0.2 to more than 0.8, they all showed significant correlation (*p* < 0.05, supplemental Fig. S2*A*(1)). In addition, 123 Rp sites and 360 Nv sites also showed significant correlation by the same analysis (supplemental Fig. S2*A*(2) and S2*B*).

Therefore, we concluded that the calpains' preference for the Nv sites was not significantly different from that of Rp sites as a whole, although small differences in several specific aars were observed (data not shown). The slight differences were probably because of the fact that the aa composition at each position of the P87mix peptides was somewhat different from the standard, because most of these peptides were selected to have a calpain cleavage site in the middle. The aa preference of all of the cleavage sites (Lit + Rp + Nv) is shown in Fig. 2*D*.

To test whether Nv sites were cleavable in the context of a whole protein, purified cardiac troponin T (TNNT2, corresponding to ID007) was digested by calpain. MS and peptide sequencing analyses revealed that two of the three identified Nv sites [C-terminal to Phe^80^ and Leu^84^ (corresponding to mouse Phe^73^ and Leu^77^, respectively)] were detected (supplemental Fig. S4). This experiment showed that some of the Nv sites, if not all, are cleaved by calpains in full-length proteins, and they have just not been reported yet.

These results strongly suggested that the calpains did not randomly proteolyze the oligopeptide mixture, but that all of the detected proteolytic sites strictly complied with an as-yet-unknown rule for calpain substrate specificity. Therefore, the limited proteolytic activity of calpains observed *in vivo* is likely to depend on secondary and/or higher-order structures.

##### C1 and C2 Showed Significantly Different Preferences at P9-P7, P2, and P5′

**Fig. 3.**
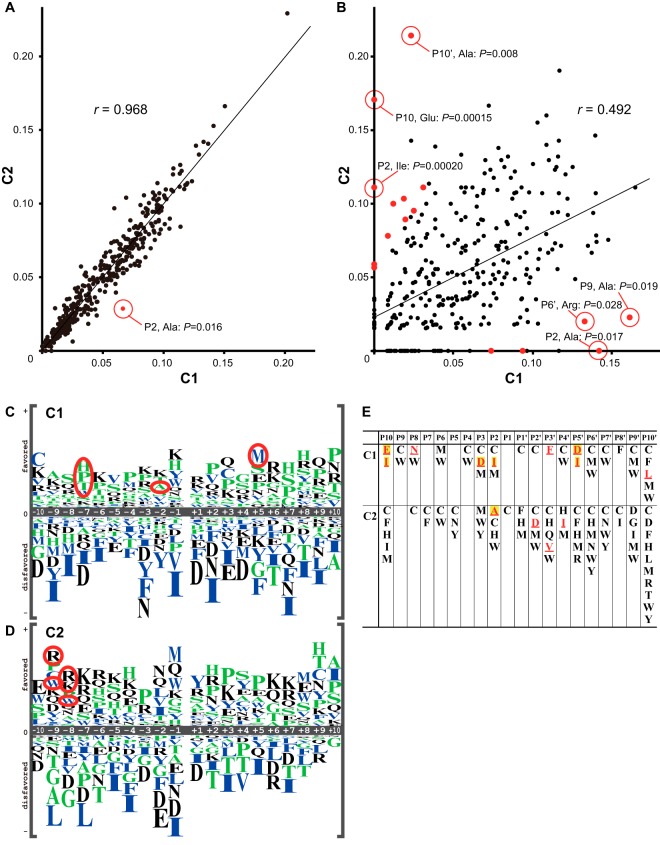
**Relationships between aa frequencies of the cleavage sites for C1 and C2.** The aa frequencies at P10-P10′ were plotted for C1 and C2 for all of the site sequences (*A*) or for those specific to each calpain (*B*). Only Ala at P2 in (*A*) and 15 others (Glu at P10, Ala at P9, Asn at P8, Arg at P7, Ile at P6, Ile and Asn at P2, Asn at P2′, Glu and Gln at P3′, Asp and Met at P5′, Arg and Thr at P6′, and Ala at P10′) in (*B*) were significantly different [*p* < 0.05, *Z*-test for the equality of two proportions (binomial distribution), see supplemental Table S6] between C1 and C2 (red dots; some are labeled with their position, aa, and *P*). For the *r* at each position, see supplemental Fig. S3*D*. *C*, *D*, The P10-P10′ cleavage site sequences specific for C1 (*C*, 123 sequences) or C2 (*D*, 65 sequences) were aligned, and the occurrence of each aar at each position was shown as in Fig. 2. Several aars that did not occur at some positions and are not shown in (*C*) and (*D*), are listed in (*E*). Red bold underlining indicates that the aa's absence represented a significant difference (*p* < 0.05; yellow marked: *p* < 0.01, binomial probability).

As previously reported (1, 4–6), our results also showed that C1 and C2 had highly similar aa preferences (*r* = 0.97 for all positions, and *r* > 0.93 for each position; Figs. 3*A* and supplemental Fig. S3). However, the frequency of Ala at P2 for C1 was significantly greater than that for C2 (6.7% *versus* 2.9%, *p* = 0.016; Fig. 3*A*, circle). Moreover, an analysis using 1,315 AAindex values showed that C2 preferred larger aars at P9 + P8 than did C1, and that C1 preferred His/Pro/Thr/Trp at P7, and Met at P5′ more than C1 did (supplemental Table S5).

There were 123 and 65 sites that were specifically cleaved by C1 and C2, respectively, and were uncleaved by the other (supplemental Fig. S3*C*). Comparison of the aa preferences of these C1- and C2-specific sequences showed that both had significantly lower correlation (*r* = 0.49, *p* < 0.001) than that for all sequences (Figs. 3*A versus* 3*B*), and that the above distinctive features at P9-P7, P2, and P5′ were emphasized in these sequences (Figs. 3*C*–3*E*, and supplemental Table S6). Although there appeared to be a much greater difference between the C1- and C2-specific sequences than among the total sequences, more samples are required to clarify this issue.

##### The k_cat_/K_m_ Values for 119 Calpain Cleavage Sites Ranged From 10 to 2000 M^−1^s^−1^

To shed further light on the calpain substrate specificity, the efficiency, *i.e.* the *k_cat_/K_m_*, for each cleavage site was determined. First, the decay of both-capped (“BC”; *i.e.* “uncleaved”) peptides was analyzed (because of the presence of truncated synthetic peptides, the number of BC peptides was much larger than 87; see supplemental Table S9). Although it was possible to calculate *k_cat_/K_m_*, the data were so variable that many signals could not be used for the calculation. There are several possible reasons for this variability, including the large variance in iTRAQ^TM^ 8-plex signals, the rapid degradation of efficiently cleaved peptides (making them inappropriate for quantification), and probably other unknown reasons. The calculated *k_cat_/K_m_* values ranged from 1 to 600 m^−1^s^−1^ (supplemental Table S9). These values correspond to the apparent *k_cat_/K_m_* of the total cleavages taking place in one peptide.

**Fig. 4.**
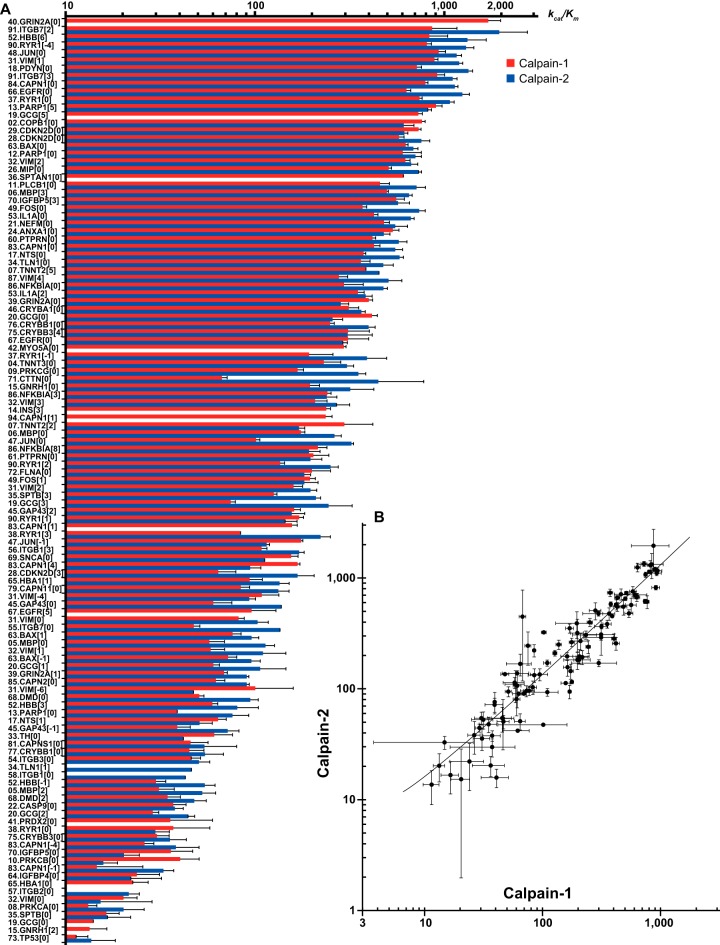
**Reaction efficiencies (*k_cat_*/*K_m_*) of 119 calpain cleavage sites.** Cleavage efficiencies (*k_cat_/K_m_* (M^−1^s^−1^)) of sites determined using quantified fragment peptides are shown for both C1 and C2 (*A*). The *k_cat_/K_m_* values of a peptide determined for the two calpains were plotted, and showed a high correlation coefficient (*r* = 0.916) (*B*). The numbers before the protein (gene product) names indicate the peptide ID No. (see supplemental Table S2), and the numbers in brackets represent the positions of cleavage sites from the middle (*e.g.* −2, −1, 0, and 1 indicate cleavage at the C terminus of positions, 8, 9, 10, and 11, respectively). Error bars: standard errors (S.E.). For data acquired under the stringent condition, see supplemental Figs. S5*A* and S5*B*.

To obtain data for each cleavage site with more confidence, the cleaved peptides generated in the P158mix were quantified. In this case, the deviations in the data were mostly small, and 71 and 48 *k_cat_/K_m_* values were calculated for Rp and Nv cleavage sites, respectively, with modest standard deviations (Fig. 4*A* and supplemental Table S8). The *k_cat_/K_m_* values for different sequences ranged widely, from 10 to 2,000 M^−1^s^−1^. To examine whether the *k_cat_/K_m_* values of Rp and Nv sites were distinct, those in the same peptides were compared (supplemental Table S10). The average *k_cat_/K_m_* values were 259.8 m^−1^s^−1^ and 189.4 m^−1^s^−1^ for the Rp and Nv sites, respectively, which were not significantly different (*p* = 0.33), supporting the above conclusion that the Nv sites are not essentially different from Rp sites.

Most of these sites were cut by both C1 and C2 with a similar *k_cat_/K_m_* value (*r* = 0.92; Fig. 4*B*), indicating that C1 and C2 share highly similar cleavage site efficiencies as well as highly similar sequence dependences. A few peptides, however, showed apparently different *k_cat_/K_m_* values for C1 and C2 (Fig. 4*A*). However, when we examined three peptides independently for their cleavability (tp1-tp3, see Experimental Procedures), no clear difference between C1 and C2 was observed (data not shown). It is possible that the relatively large deviations obtained using the iTRAQ^TM^-MS method were responsible for the apparent differences between C1 and C2. Thus, although C1 and C2 have distinct aa preferences, we have not yet observed a clear difference in their cleavage efficiency. Further studies are required to clarify the distinct substrate specificities of C1 and C2.

##### Calpains Significantly Prefer Longer P-site Sequences (N-terminal Side of the Cleavage Site) Than P′-site Sequences (C-terminal)

**Fig. 5.**
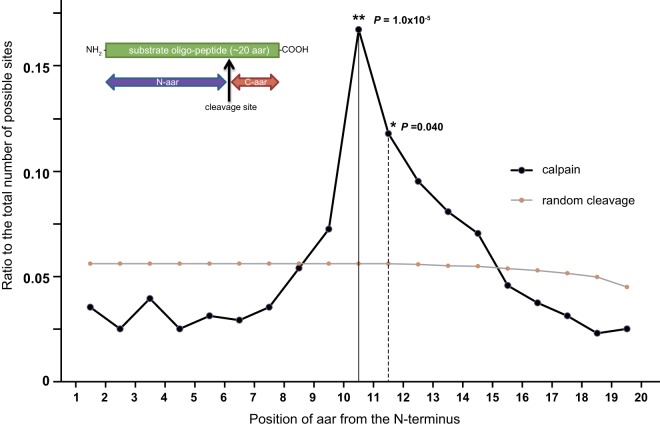
**Frequencies of cleavages by C1 and C2 at each position.** Occurrence rates of the number of cleavage sites detected at each position were plotted along with those expected by random cleavages. Cleavages before and after position 11 showed significantly increased occurrences (*P* was calculated by the *Z*-test for a proportion).

To investigate whether the P- and P′-sites have distinct features, the positions of calpain cleavage sites in the oligopeptides were analyzed statistically. If the peptides were randomly cleaved by calpains without specificity, all of the positions should show an ∼5% frequency (Fig. 5, gray line). However, the peptides were designed to contain a calpain cleavage site mostly in the middle (between positions 10 and 11), and, as expected, this site showed a significantly higher cleavage frequency (Fig. 5, black line between 10 and 11).

Unexpectedly, the site after position 11 showed a significantly higher cleavage frequency than expected (Fig. 5, dashed line between 11 and 12), and those after positions 12–14 had the same tendency as position 11, although the difference was not significant. On the other hand, sites N-terminal to position 8 and C-terminal to position 15 tended to be cleaved less frequently than expected. In summary, the sites between positions 10 and 14 are preferred by calpains, and those after the N-terminal 7 aars and before C-terminal 5 aars are cut poorly by calpains. These asymmetric features of cleavability suggest that calpains require a longer P-site sequence than P′-site sequence. In addition, there was no difference in these trends between C1 and C2 in this analysis.

##### Binary-QSAR Model Constructed with Cleavage Site Sequences Showed a Better Prediction Performance Than Previous Models

To predict calpain cleavage sites, we used a binary-QSAR model (see Discussion for advantages of this model) with the information gathered in the experiments above.

For aa descriptors, we used the AAindex (26), predicted secondary structures, and molecular descriptors in the MOE package (see supplemental Tables S11 and S12). Several ranges of sequences were tried, and P6-P6′ were used, because longer and shorter ranges did not perform well, probably because there were too many missing values and the sequences were too short, respectively. Of all the possible P87mix site sequences (1,703), 806 (314 cleaved and 492 uncleaved) sequences did not contain any missing values between P6 and P6', and were used for training data to construct a predictor. The best-balanced binary-QSAR model achieved was constructed with eight descriptors, associated with P6, P2, and P1 (Table III). This predictor performed with a leave-one-out (LOO) accuracy of 74.9% (Table IV, *versus* P87 P6-P6′).

**Table III:** Descriptors used in the binary-QSAR model For the values of aars for each descriptor, see supplemental Tables S11 and S12.

Position	Descriptor ID	Descriptor No.	Importance*^a^*	Attribute	Ref.
P6	SS_randomC	603	0.104	Probability of secondary structure other than α-helix or β-strand (random coil)	Predicted by “Jpred 3” (http://www.compbio.dundee.ac.uk/www-jpred/) (49)
P2	NADH010102	447	0.118	Hydropathy scale based on self-information values in the two-state model (9% accessibility)	(50)
P2	BIOV880101	10	0.102	Information value for accessibility with an average fraction of 35% (high if buried)	(51)
P2	BIOV880102	11	0.121	Information value for accessibility with an average fraction of 23% (high if buried)	(51)
P2	vsurf_W3	949	0.0973	Volumes of the interactions with the H_2_O probe at -1.0 kcal/mol	MOE
P2	GUOD860101	493	0.0971	Retention coefficient at pH 2	(52)
P2	vsurf_W2	948	0.100	Volumes of the interactions with the H_2_O probe at -0.5 kcal/mol	MOE
P1	ASA+	719	0.119	Water accessible surface area of all atoms with positive partial charge (strictly greater than 0)	MOE

*^a^* Importance was automatically calculated by the MOE software.

**Table IV:** Accuracy of our binary-QSAR model against the P87mix and Lit data sets [*vs* P87] All of the possible cleavage sequences of P87mix (*All*) or those having aars in all of the P6–P6′ positions [*P6–P6′*; *e.g.*, for a peptide ACDEFGHIKLMNPQRSTVWY, there are 19 possible cleavage sites. Among them, ACDEFG/HIKLMNPQRSTVWY (where/is the calpain cleavage site; for this cut, there are aars at P6–P14′) is included, but ACDEF/GHIKLMNPQRSTVWY (which is P5–P15′ and does not have aar at P6) is excluded] were tested using our binary-QSAR model. The accuracy and leave-one-out (LOO) accuracy rates for cleaved, uncleaved, and total sequences are shown. [*vs* Lit] Of 420 cleaved sequences in the literature, 132 P10–P10′ (20mer) sequences that were not used for training any of the predictors shown here (used as positive samples) and their reversed sequences (as negative samples) were tested (total *n* = 264). Various prediction rates are shown for the binary-QSAR model with a threshold of 0.5 or 0.95 (B-QSAR(0.5) or (0.95), respectively) in comparison with previously reported methods (GPS-H, -M, and -L: ccd.biocuckoo.org (16); SVL-R, -L, PSSM, and MKL: www.calpain.org (15); SP-C1, and -C2: www.dmbr.ugent.be/prx/bioit2-public/SitePrediction/ (18)). Bold numbers indicate the best scores. For each prediction result, see supplemental Table S14.

*vs* P87	P6–P6'			All	
	n*^a^*	Accuracy	LOO accuracy	n	Accuracy
Cleaved	314	0.576	0.573	483	0.582
Uncleaved	492	0.868	0.862	1,220	0.744
Total	806	0.754	0.749	1,703	0.698

*vs* Lit	B-QSAR (0.5)	B-QSAR (0.95)	GPS-H	GPS-M	GPS-L	SVL-R	SVL-L	PSSM	MKL	SP-C1	SP-C2
Sensitivity [TP/(TP+FN)]	**0.538**	0.053	0.242	0.348	0.394	0.258	0.205	0.288	0.288	0.197	0.220
Specificity [TN/(TN+FP)]	0.758	**0.992**	0.947	0.833	0.742	0.939	0.955	0.909	0.909	0.932	0.871
PPV, positive prediction value [TP/(TP+FP)]	0.689	**0.875**	0.821	0.676	0.605	0.810	0.818	0.760	0.760	0.743	0.630
NPV, negative prediction value [TN/(TN+FN)]	**0.621**	0.512	0.556	0.561	0.551	0.559	0.545	0.561	0.561	0.537	0.528
Total accuracy [(TP+TN)/n]	**0.648**	0.523	0.595	0.591	0.568	0.598	0.580	0.598	0.598	0.564	0.545

*^a^* Abbreviations used, GPS-H, -M, or -L: high-, medium-, or low-threshold mode of GPS-CCD Ver.1; SVL-R or -L: support vector machine using RBF or Linear kernels; PSSM: position-specific scoring matrix method; MKL: multiple kernel learning method; SP-C1, or -C2: Site Prediction for cleavage by calpain-1 or -2 (all species); n: number of samples used; TP, true positive; FN, false negative; TN, true negative; FP, false positive.

**Fig. 6.**
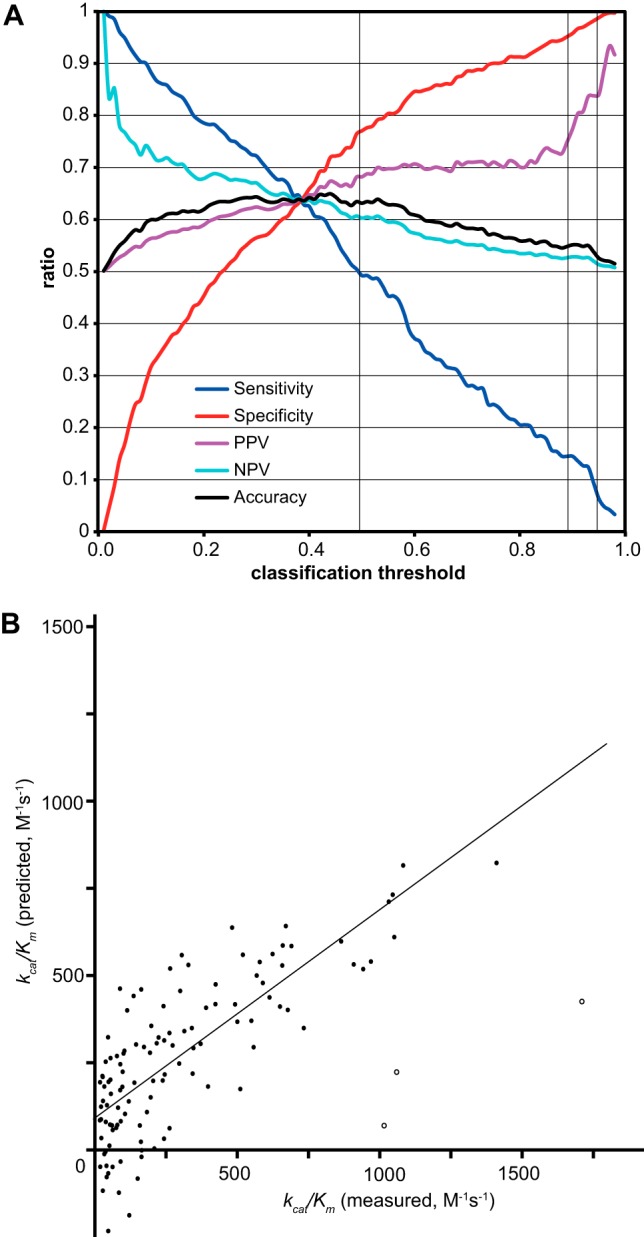
**QSAR analysis using binary QSAR and partial least square regression (PLS) of the 119 *k_cat_/K_m_* values obtained in this study.** *A*, Our best binary-QSAR model with eight descriptors achieved an XA of 74.9% (sensitivity [TP/(TP+FN)]: 57.3%; specificity [TN/(TN+FP)]: 86.2%). To evaluate this model, we used 331 P10-P10′ (20mer) sequences that were not used for training of the predictor among 420 Lit sequences and their reversed sequences as negative samples. Sensitivity, specificity, positive prediction value [PPV, TP/(TP+FP)], negative prediction value [NPV, TN/(TN+FN)], and accuracy [(TP+TN)/total number] are shown as a function of classification threshold values. Note that the PPV reached 68.2% (threshold = 0.5), 77.4% (0.9), and 84.0% (0.95) (vertical thin lines). *B*, Our best PLS-QSAR model showed an *Xr^2^* value of 0.604 with eight descriptors. *k_cat_/K_m_* values measured by our experiments (*x* axis) and predicted by this model (*y* axis) were plotted, generating the line y = 0.554 x +114 and *r* = 0.834 (*Xr* = 0.777). Open circles indicate three outliers excluded from the calculation by the MOE software. The coefficients determined for these models are shown in Tables III and V.

To test the real prediction performance of the binary-QSAR model, 331 cleavage site sequences from the literature (“Lit” data set) that were not used in its construction were analyzed with our model. The 331 reversed sequences were used as negative control samples. The model had 63.1% total accuracy (Fig. 6*A*). It should be noted that our model achieved a positive prediction value (the ratio of true positives to those predicted as positive) of 84.0% when the classification threshold was set to 0.95 (Fig. 6*A*, thin line at threshold = 0.95 crossing the PPV line). This means that sites predicted by our binary-QSAR model with a threshold of 0.95 are very likely to be cleaved by calpains at the cost of sensitivity.

Next, using 132 cleavage site sequences that were not used for training any of previous calpain predictors, the predictors' performance was compared. The results showed that our model outperformed all other reported prediction methods (Tables IV (*versus* Lit) and S14; note that reversed sequences were not necessarily true negative samples, and might be cleavable, implying that the accuracy of our model would be better than the value shown).

**Fig. 7.**
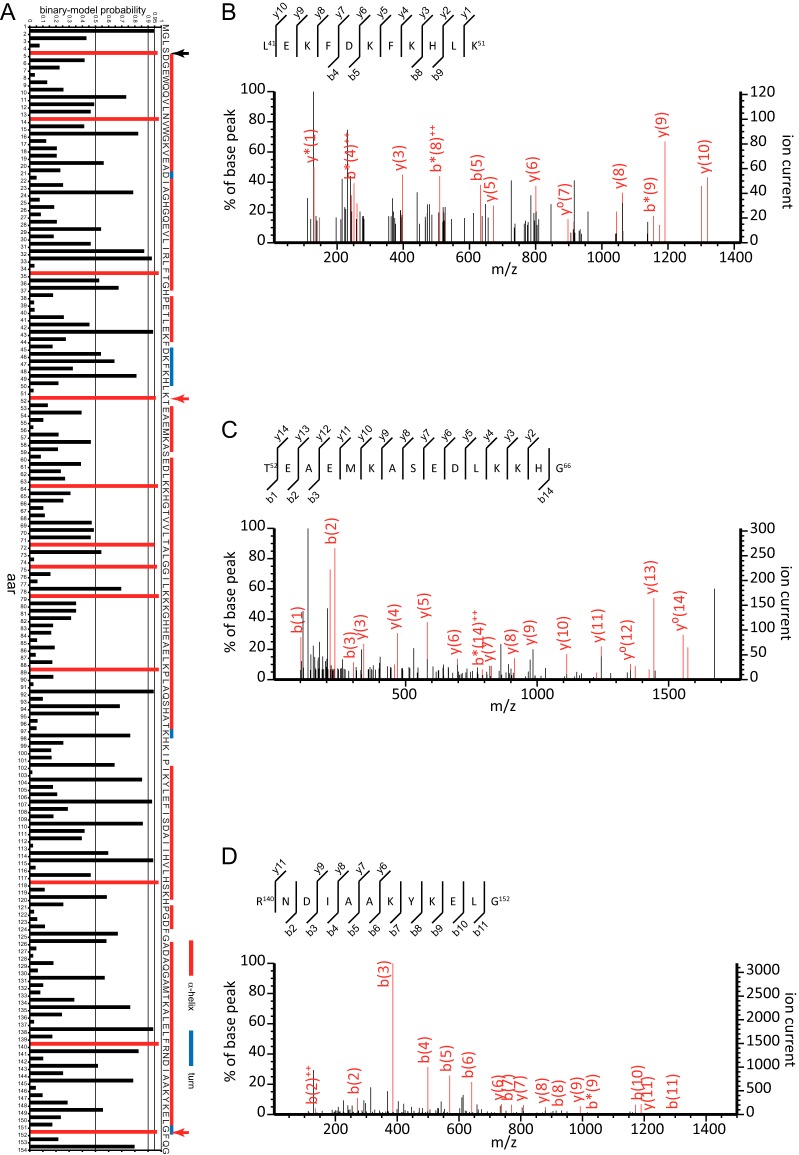
**Prediction and identification of two novel calpain cleavage sites in myoglobin.** The sequence of horse myoglobin (P68082) was subjected to our binary-QSAR prediction model, and the probability of calpain cleavage after each aar was plotted (*A*). Red horizontal bars indicate a probability > 0.95 (*i.e.* predicted as cleavable). The aa sequence is shown at right. Red and blue vertical bars indicate the secondary structures α-helices and turns, respectively, from the Protein Data Bank (PDB) entry for horse myoglobin, 3VM9 (no β-strand is found in the myoglobin structure). Arrows indicate predicted cleavage sites in exposed unstructured regions according to the protein's 3D structure (3VM9). Among these, red and black arrows indicate sites that were actually cleaved and uncleaved, respectively, in this study (*B–D*). Horse myoglobin was incubated with calpain-1, and the TCA-soluble supernatant of the reaction mixture was analyzed by LC-MS/MS. The MS/MS spectra of peptides corresponding to the Lys^51^/Thr^52^ (*B* and *C*) and Gly^152^/Phe^153^ (*D*) sites are shown.

Finally, to identify calpain cleavage sites in a novel substrate protein, the sequence of horse myoglobin (MYO) was subjected to our prediction analysis. Among 12 sites predicted (Fig. 7*A*, red horizontal bars), three sites (arrows) were in loop/unstructured regions according to the 3D structure of MYO. Identification of the fragments generated by the calpain digestion of MYO showed that two of these sites were cleaved by calpains in actuality (Fig. 7*A*, red arrows, 7*B*–7*D*).

##### The First PLS QSAR model for Calpain Cleavage Site Efficiency

Finally, to predict quantitatively the cleavage efficiency of calpains for any peptide bond, the QSAR analysis of 119 site sequences with *k_cat_/K_m_* values was performed using the partial least squares regression (PLS) method. Using the LOO method, the most balanced PLS model had eight descriptors associated with P10, P2, P1, P3′, and P4′ (Table V). This model showed a LOO *r* of 0.78 (total *r* = 0.83, after excluding three outliers) (Fig. 6*B*).

**Table V:** Descriptors used in the partial least squares regression (PLS) model For the values of aars for each descriptor, see supplemental Tables S11 and S12.

Position	Descriptor ID	Descriptor No.	Relative importance	Estimated coefficient	Δ*k_cat_/K_m_* (M^−1^s^−1^)		Attribute	Ref.
P10	JANJ780102	128	1	7.99	567	P10: 290	Percentage of buried residues (aar with an accessible surface area smaller than 20 Å^2^)	(53)
P10	GUYH850104	522	0.439	120	320		Apparent partition energies calculated by residues as exposed of buried as in (54)	(55)
P2–P1	PEOE_VSA-6	255 (2D)	0.545	-11.7	651	P2: 331 P1: 320	Sum of van der Waals surface area (v_i_ (Å^2^)) where atomic partial charges (q_i_) calculated by the Partial Equalization of Orbital Electronegativities (PEOE) method is less than -0.30	MOE
P3′	E_ang	763	0.628	62.6	448	P3′: 537 P4′: 475	Angle bend potential energy	MOE
P3′–P4′	Q_VSA_PNEG	282 (2D)	0.657	-8.60	478		Total negative polar van der Waals surface area. This is the sum of the v_i_ such that q_i_ is less than -0.2. The v_i_ were calculated using a connection table approximation, and q_i_ using the partial charges stored with each structure in the database	MOE
P4′	ROBB760111	349	0.592	-30.5	381		Information measure for C-terminal turn	(56)
P4′	PEOE_VSA+6	836	0.507	-11.5	395		Sum of the v_i_ where q_i_ is greater than 0.3 (see PEOE_VSA-6)	MOE
P4′	vsurf_Wp2	956	0.678	1.04	472		Volumes of the interactions with carbonyl probe at -0.5 kcal/mol	MOE

Because the PLS model was constructed using the data from only 119 sequences from the P87mix data set, all the rest of the P87mix data (364 “cleaved” and 1220 “uncleaved” data without *k_cat_/K_m_*) were evaluated by the model. As shown in Table VI (*versus* P87 unused), the average *k_cat_/K_m_* of the “cleaved” data set was significantly greater than that of “uncleaved” set (180.8 m^−1^s^−1^ *versus* 114.4 m^−1^s^−1^, *p* = 0.00049). These results indicated that our PLS model appropriately describes at least a portion of the calpain cleavage efficiencies. In other words, these findings indicate that the selections of aa descriptors and their weights by the MOE program are appropriate and reflect calpains' substrate specificity.

**Table VI:** Average predicted k_cat_/K_m_ values of our PLS model The k*_cat_/K_m_* values for all possible cleavage sequences of P87 [*vs* P87 all] or those except 119 sequences used for the PLS model construction [*vs* P87 unused] were calculated using our PLS model. The average and standard deviation (S.D.) of the *k_cat_/K_m_* values for cleaved, uncleaved, and total sequences are shown. The average *k_cat_/K_m_* values of the “Cleaved” versus the “Uncleaved” sequences were significantly different (*p* = 5.1×10^−8^ and 4.9×10^−4^, *t*-test for two population means with unknown and unequal variances).

	n	Average	S.D.	
*vs* P87 all				
Cleaved	483	204.6	303.4	
Uncleaved	1220	114.4	311.7	*p* = 5.1×10^−8^
Total	1703	140.0	311.9	
*vs* P87 unused				
Cleaved	364	180.8	318.7	
Uncleaved	1220	114.4	311.7	*p* = 4.9×10^−4^
Total	1584	129.6	314.4	

## DISCUSSION

### 

#### 

##### First Report of the Comprehensive Measurement of k_cat_/K_m_ values

In this study, using an oligopeptide library and the iTRAQ^TM^ proteomic method, 483 calpain cleavage sites were identified in addition to the 420 sites previously reported in the literature. Among the identified sites, 360 are novel, and the k_cat_/K*_m_* was determined for 119. These findings enabled us to analyze calpain substrate specificity not only precisely but also quantitatively. This is the first report to address calpain substrate specificity from the viewpoint of proteome-wide quantitative structure-activity relationships.

Proteases like caspases and granzymes have explicit sequence specificity for substrate cleavage (*e.g.* P1 = Asp), and thus, considerably precise predictors have been constructed for them using PSSM, SVM, and other methods, achieving total accuracy of more than 90% (27–32). For other important proteases such as matrix metalloproteinases and proteasomes, however, no significant predictor has been constructed, because these proteases lack clear selectivity on substrate sequences (32–37). The methods used in our study were effective for defining the specificity of calpains, one of the toughest examples of these “difficult” proteases; therefore, they will be applicable to solving the substrate specificities of the above-mentioned proteases.

To date, the *k_cat_/K_m_* values for fewer than 10 calpain substrates have been reported (6, 38), which range from 41.7 to 141 m^−1^s^−1^. These values are consistent with those obtained in this study. Because the proteolytic conditions used in this study were somewhat unusual because of the use of concentrated calpains and unpurified peptides, the *k_cat_/K_m_* values determined here may be underestimated compared with those obtained under more typical conditions. However, the smooth distribution of the *k_cat_/K_m_* values that we obtained (see Fig. 4*A*) indicates that at least the relative *k_cat_/K_m_* values among the 119 determined values hold true.

Calpains also show amidase-like activity, but surprisingly, the *k_cat_/K_m_* for hydrolysis of the NH_2_ group at the C terminus of substance P (RPKPQQFFGLM-NH_2_) is 10^6^ m^−1^s^−1^ (39). This activity is mainly achieved by an ∼10^4^-fold increase in the *k_cat_* without a significant change in the *K_m_* (39), by an unknown mechanism. Although this amidase-like calpain activity may be involved in as-yet-unknown physiological functions, there has been no further report on it. We did not detect any C-terminal DKP hydrolyzing activity in this study (data not shown; see supplemental Experimental Procedures).

##### Confirmation that the Substrate Sequence Selectivity of Calpains is Rather Weak

Consistent with all previous PSSM-type studies of calpain substrate sequences, both C1 and C2 showed weak sequence selectivity in this study (see supplemental Fig. S3). In terms of the 3D structure (40–42), the substrate recognition by calpains is mainly determined by relatively weak interactions between an atom in the peptide bonds of a substrate and an atom of calpains' subsite residues. For example, Gly^198^ of CAPN2 (supplemental Fig. S6*A*, corresponding to Gly^208^ of CAPN1 (supplemental Fig. S6*C*)) interacts with the O (-2.0 kcal/mol) and NH (-1.7 kcal/mol) of the P1-P2 and P2-P3 peptide bonds, respectively, whereas Gly^261^ of CAPN2 (S6A, corresponding to Gly^271^ of CAPN1 (S6C)) interacts with the NH (-4.7 kcal/mol) of P1-P2.

In other words, most of the side-chains of the substrate residues are exposed to the solvent without forming a strong interaction with calpain atoms. These features, which are common to both C1 and C2, are in sharp contrast to caspases, which strongly interact with P1 and P4 Asp side chains (supplemental Fig. S6*D*). These weak interactions contribute to the calpains' recognition of highly divergent substrate sequences. Exceptions are the P2 and P3′ positions, where the side-chains of Leu and Pro, respectively, are deeply encompassed by the active site cleft of the calpains (supplemental Fig. S7). This point will be discussed further, below.

##### Existence of Many Nv Sites Suggests that Substrate Protein Cleavages By Calpains are Regulated By Both Primary and Higher-order Structures

The literature contains reports of 420 unique calpain cleavage sites in 147 substrate proteins. Most of these sites are cleaved in the context of a whole protein or part of a protein that is expected to have a proper 3-D structure. On the other hand, the 483 sites identified in this study were in 20-mer peptides, which are unlikely to contain potential cleavable sites that were inaccessible by steric hindrance. Thus, the 360 Nv sites identified in this study are considered calpain-cleavable, not artifactual, sites that are not exposed in the context of a whole protein structure. The lack of significant differences in the aa preferences and *k_cat_/K_m_* values between the Rp and Nv sites supports this idea (see Fig. 2 and supplemental Table S10).

Therefore, most substrates have many sites that are potentially cleavable by calpains that escape cleavage when the substrate protein retains its higher-order structures. We thus conclude that the calpains' substrate specificity is defined by both primary and higher-order structures. The limited proteolysis by calpains that is often observed under physiological conditions probably reflects the fact that only extremely small amounts of calpains are activated *in vivo*.

##### Sequences Proximal to the Cleavage Sites Were Highly Similar for C1 and C2, and Both Preferred Longer Sequences in the P- than the P′-region

As in almost all previous reports, the aa sequence preferences around the cleavage sites for C1 and C2 were almost identical in this study, which is supported by the calpains' 3D-structural features, as described above. Surprisingly, however, detailed analysis revealed that the preferences for specific positions (P9-P7, P2, and P5′) were significantly different between C1 and C2 (Figs. 3*C* and 3*D*, and supplemental Table S5). Among them, the calpain aars most proximate to P8-P7 and P5′ are different between C1 and C2, *i.e.* Asp^256^, Ile^257^, and Leu^260^ of C1 are within 5 Å of Ser^169^-Thr^170^ (corresponding to P8-P7) of calpastatin, whereas the corresponding residues of C2 (Ser^246^, Ala^247^, and Ser^250^, respectively) are not (supplemental Fig. S8*A*); Glu^172^ of C2 and Met^329^ of C1 are close to Glu^185^ (P5′) of calpastatin, whereas the corresponding Gln^182^ of C1 and Gln^319^ of C2, respectively, are not (supplemental Fig. S8*B*). How these differences lead to distinct aa preferences is unknown at present. Moreover, there appears to be no significant difference in the P9- and P2-proximate aars between C1 and C2. To clarify the different substrate specificities of C1 and C2, further studies with more sample numbers are required.

The cleavage positions showed asymmetric frequencies (see Fig. 5), suggesting that calpains require a longer segment of P-site than P′-site residues. The P10-P5 sites are mainly recognized by the calpain CBSW domain (19, 40, 41), which may play a crucial role in substrate recognition (see supplemental Fig. S7*A*; the right side surface corresponds to CAPN2's CBSW domain). These results are in concert with calpains' amidase-like activity, for which only the P-site region plays a role (39).

##### Binary-QSAR Analyses of Calpain Substrate Cleavages Suggest That Discrete Positions (P6, P2, P1) Determine “Cleavability”

Many attempts have been made to predict calpain cleavage sites, including studies using PSSM, support vector machine (SVM), multiple kernel learning (MKL), a form of hierarchical clustering, and other methods (12–20), each of which has advantages and disadvantages. Here, we used the binary-QSAR model, which uses Bayes' theorem. It is a robust method that is low in computational cost and high in performance. In addition, it is easy to interpret the relative importance of various factors using a binary-QSAR model (43, 44).

**Fig. 8.**
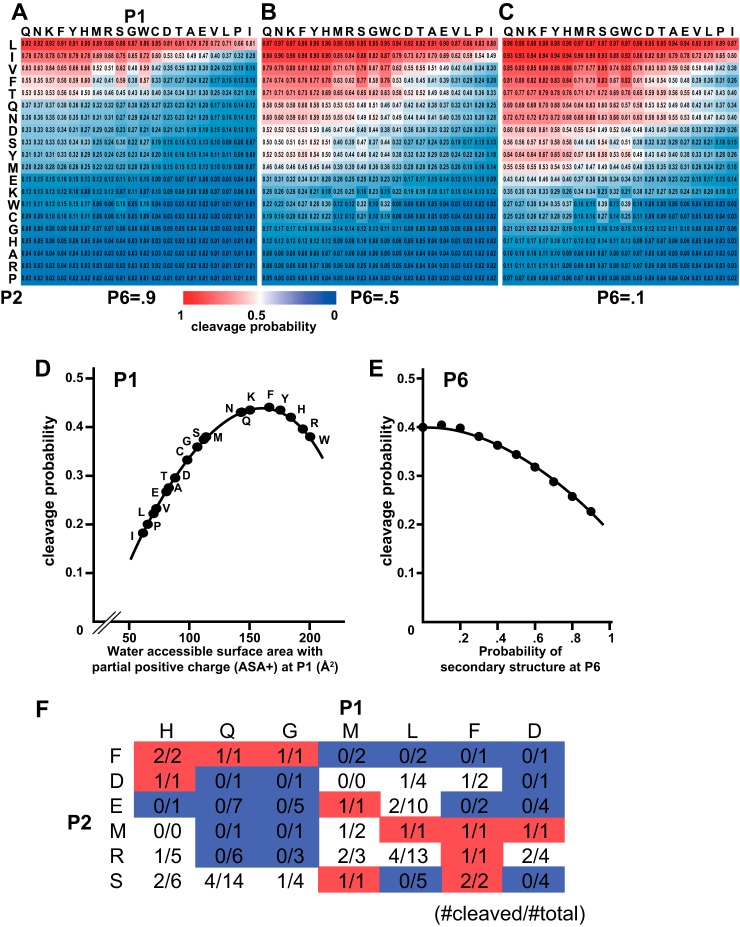
**Binary-QSAR analysis using 314 cleaved and 492 uncleaved sequences with no missing aars between P6 and P6′.** Because our binary-QSAR model (see Fig. 6*A* and Table III) refers only to P6, P2, and P1, all of the possible combinations of aars at these sites were analyzed for cleavage probability. The results are shown as a function of P2 and P1 aars when the probability of secondary structure at P6 was 0.9 (*A*), 0.5 (*B*), or 0.1 (*C*). Darker red indicates a greater probability of cleavage by calpains. (*D*) The relationship between ASA+ values at P1 (*x* axis) and cleavage probabilities (*y* axis) is shown as an average of all possible P2 and P6 aars. The regression curve is *y* = −3.0 × 10^−5^ *x*^2^ +8.8 × 10^−3^ *x* - 0.26 (*r* = 0.999). (*E*) Relationship between the probability of secondary structure at P6 (*x* axis) and cleavage probability (*y* axis) is shown as an average of all possible P1 and P2 aars. The regression curve is y = −2.0 × 10^−3^ x^2^ - 2.4 × 10^−3^ x +0.41 (*r* = 0.998). (*F*) P2 and P1 positions contributed to cleavability cooperatively. The numbers of cleaved sites (#cleaved) identified in this study among 1,703 all possible sites in P87mix (#total) were counted for P2 = Phe (F), Asp (D), Glu (E), Met (M), Arg (R), and Ser (S), and P1 = His (H), Gln (Q), Gly (G), Met (M), Leu (L), Phe (F), and Asp (D). For example, sequences with P2-P1 = Phe-Gln (total of one sequence) or Met-Leu (one) were cleaved, whereas those with Phe-Leu (two) or Met-Gln (one) were not cleaved.

Our binary-QSAR model showed that the aa properties of only sites P6, P2, and P1 could reasonably predict the macro “cleavability” of a substrate by calpains (Table III, Fig. 8). That is, these sites are primarily involved in the cleavage efficiency of substrates by calpains with a certain hierarchy. Consistent with previous studies, P2 was the most important, and in the binary-QSAR model, P2 was associated with six descriptors, which are all related to hydrophobicity (NADH010102, BIOV880101 and 102, vsurf_W2 and _W3, and GUOD860101) (Table III). In brief, the model predicts that sequences with Leu at P2 will always be cleaved, regardless of P1 or P6; those with Ile, Val, Phe, Thr, Gln, Asn, Asp, Ser, Tyr, or Met at P2 are dependent on P1 and P6; and those with Glu, Lys, Trp, Cys, Gly, His, Ala, Arg, or Pro at P2 are predicted to be uncleaved regardless of P1 or P6 (Figs. 8*A*–8*C*).

P6 and P1, which are associated with one descriptor each, contribute only moderately to the cleavability, compared with P2. At P1, a water-accessible surface area (probe radius of 1.4 Å) with a partial positive charge (ASA+) yields the maximum cleavage probability at 138 Å^2^ (Asn, Gln, Lys, Phe, and Tyr are close to this value; Fig. 8*D*). Larger and smaller ASA+ values decrease the probability (by about 0.26 at maximum), suggesting that the condition at the S1 subsite of calpains is not very flexible; thus, Ile, Pro, or Leu at P1 markedly decreases cleavability.

A lower probability of a random coil secondary structure at P6 slightly increased the cleavability (by less than 0.2, Figs. 8*A*–8*C*, 8*E*). The 3-D structures of C2/calpastatin co-crystals revealed that calpains' S6 subsite is on the surface of the CBSW domain, and S3-S10 are almost aligned (19, 40, 41) (supplemental Fig. S7*A*). Therefore, our results support the idea that the secondary structure in the middle of this region may decrease a substrate's affinity for the CBSW domain by reducing flexibility, resulting in lower cleavability.

It is noteworthy that a cooperative effect was observed on substrate cleavage efficiency between P2 and P1. For example, when the aars at P2-P1 were Phe-Gln, Phe-Gly, Met-Leu, or Met-Phe, they were cleaved; if they were Phe-Leu, Phe-Phe, Met-Gln, or Met-Gly, however, they were not cleaved (Fig. 8*F*). This cooperative effect has also been reported for various proteases (15, 19, 45–48), and involves local subsite structures. The precise structural factor(s) responsible for the observed cooperative effect of calpains, however, has not yet been determined.

##### PLS QSAR Analyses Suggest That P3′–P4′ Most Affects Cleavage Efficiency, Followed By P2, P1, and P10

**Fig. 9.**
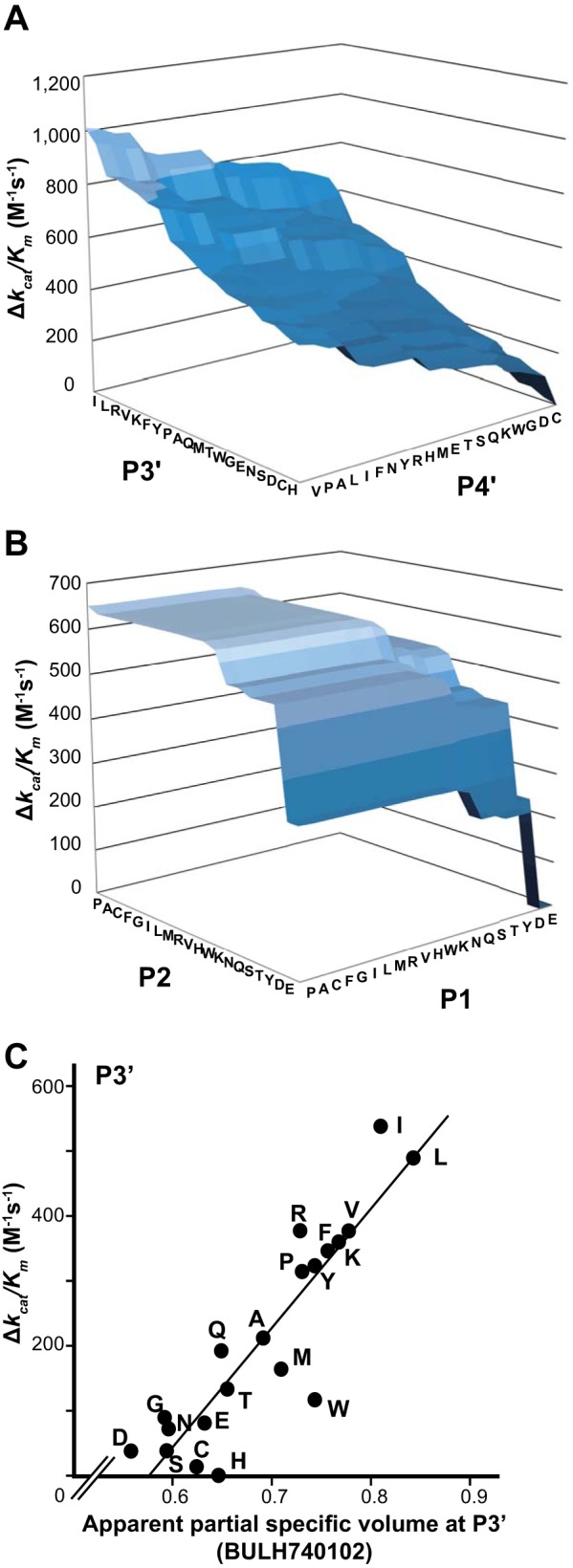
**Contribution of aars at positions P1, 2, 3′, or 4′ to *k_cat_/K_m_* values in our PLS-QSAR model.** *A*, *B*, Using our PLS-QSAR model (see Fig. 6*B* and Table V), the change in *k_cat_/K_m_* value (Δ*k_cat_/K_m_*) was calculated as a function of the aars at P3′ and P4′ (*A*), or P2 and P1 (*B*). *C*, Δ*k_cat_/K_m_* was plotted as a function of BULH740102 (see below) value for each aar at P3′. A *k_cat_/K_m_* value for each aar was calculated by entering each of the 20 aars for P3′ into our PLS-QSAR model equation assuming that all other positions are fixed; *i.e.* for each aar, aa_i_ (i = 1–20; aa_1_ = Ala (A), aa_2_ = Cys (C), … aa_20_ = Trp (W)), P3′(aa_i_) = 62.6·E_ang(aa_i_) + average[-8.60·Q_VSA_PNEG(aa_i_,aa_j_) (j = 1–20)]. The difference between the maximum (Ile) and minimum (His) values at the P3′ position was calculated to be 537 m^−1^s^−1^. Next, the most correlated aa descriptor was determined: first, *r* and ρ were calculated between the *k_cat_/K_m_* estimated above and each of the 1,315 aa descriptors; then, the descriptors were ranked independently for *r* and ρ, and the sums of the ranks of both were again ranked; the best descriptor was BULH740102 (*r* = 0.896, ρ = 0.869). ρ was used in addition to *r*, because ρ is robust against abnormal distributions with outliers, which are features of some aa descriptors, whereas *r* is greatly affected by the outliers. For the values of the aars of each descriptor, see supplemental Tables S11 and S12.

To our surprise, the P3′ and P4′ positions had the most effect on the *k_cat_/K_m_* values, which changed by *ca*. 1,000 M^−1^s^−1^, depending on the aars at P3′–P4′ (Fig. 9*A*).

The *k_cat_/K_m_* values predicted by our PLS QSAR model showed the best correlation with the partial specific volume and mass density of the aar at P3′ (Fig. 9*C*). This finding is consistent with the 3D-structural observations that the sidechain of P3′ has no specific interaction with calpain atoms, and is buried in a calpain surface cleft surrounded by a relatively hydrophobic environment (supplemental Fig. S7*B*).

P2 and P1 are also important (each *k_cat_/K_m_* change > 300 m^−1^s^−1^), and Leu, Ile, and Val at P2, which gave high cleavage probability in the binary-QSAR model, were also associated with high efficiency (Fig. 9*B*). On the other hand, Asn and Asp at P2, which moderately increased cleavability, showed rather low efficiency. The predicted *k_cat_/K_m_* values were dependent on the sum of the van der Waals surface area of aars at P2 and P1, where the atomic partial charge is less than −0.3 (Table V, PEOE_VSA-6). The preference of P2 site was also related to the 3D-structure; the P2 residue side-chain penetrates the cleft beside the calpain active site, making weak hydrophobic interactions with calpain atoms (supplemental Fig. S7*A*, green surfaces).

Notably, Pro at P1, which markedly lowered the cleavability, caused the greatest increase in efficiency, among the 20 aars. This result suggests that most substrates with a Pro at P1 are not easily cleaved, whereas they are rather efficiently cleaved if the aars at other positions are favorable for cleavage. The accessible surface area, which is related to hydrophilicity, of the aar at P10 also contributes to the calpain cleavage efficiency, by 290 m^−1^s^−1^.

Cuerrier and his colleagues developed a highly sensitive fluorescent oligopeptide substrate, H-E(EDANS)PLFAERK(DABCYL)-OH (13), which is cleaved after Phe (F) (4). Our PLS model predicted that PLFAER for P3-P3′ would have a *k_cat_/K_m_* of 763 m^−1^s^−1^, which is almost the maximum value (822 m^−1^s^−1^) for all possible P3-P3′ peptides, consistent with the sensitivity of the PLFAER substrate and supporting the effectiveness of our PLS QSAR approach. Indeed, Leu-Phe at P2-P1 and Arg at P3′ was one of the best combinations of these positions (see Figs. 9*A* and 9*B*). PSSM-based methods count cleavages equally, regardless of the sequences' cleavage efficiencies, whereas the peptide sequencing-based method used by Cuerrier *et al.* (13) as well as our PLS method take the cleavability of each peptide into account. Thus, further PLS studies with more *k_cat_/K_m_* data should eventually reveal the ultimate substrate specificities of calpains.

Taken together, our PLS QSAR analyses showed that substrates having (Leu or Ile) (Val, Pro, or Ala) at P3′–P4′ and P2-P1 are cleaved with high efficiency by calpains, and those with Glu or Asp at P3′, P2, and P1 are cleaved with the least efficiency. This information may be useful for mutation studies seeking to change calpain substrates to be uncleavable and/or to insert *de novo* calpain cleavage sites. Therefore, this study opens new avenues into the study of calpain substrates. Further elucidation of the context-dependent and quantitative structure-activity relationships of calpains and their substrates will improve our understanding of calpain substrate specificity.

## Supplementary Material

Supplemental Data

## Footnotes

^1^ The abbreviations used are:

aa: amino acid
aar: amino acid residue
BC: both-capped (uncleaved peptides)
C1: calpain-1 (CAPN1/S1, μ-calpain)
C2: calpain-2 (CAPN2/S1, m-calpain)
CAPN: calpain
CAPNS1: calpain small subunit 1
CBSW: calpain-type β-sandwich domain
DB: database
DKP: diketopiperadinylation
FN: false negative
FP: false positive
iTRAQ: isobaric tag for relative and absolute quantitation
*k_cat_*: catalytic reaction rate constant
*Km*: Michaelis-Menten constant
Lit: literature
MKL: multiple kernel learning
nLC: nano-scale LC
Nv: novel (cleavage site)
LOO: leave-one-out
NPV: negative prediction value
PLS: partial least squares regression
PPV: positive prediction value
PSSM: position-specific scoring matrix
QSAR: quantitative structure-activity relationship analysis
*r*: Pearson's correlation coefficient
ρ: Spearman's rank correlation coefficient
Rp: reported (cleavage site)
SVM: support vector machine
TCEP: tris (2-carboxyethyl) phosphine
TN: true negative
TP: true positive
XA: cross-validated accuracy
*Xr*: *r* value cross-validated with LOO.
