# Supplementary material for: Predictions of Cleavability of Calpain Proteolysis by Quantitative Structure-Activity Relationship Analysis Using Newly Determined Cleavage Sites and Catalytic Efficiencies of an Oligopeptide Array
Source: Mol Cell Proteomics. 2016 Jan 21;15(4):1262–80. doi: 10.1074/mcp.M115.053413 (PMC4824854; doi:10.1074/mcp.M115.053413)
Supplement: Supplemental Data [file supp_15_4_1262__index.html]

Predictions of Cleavability of Calpain Proteolysis by Quantitative Structure-Activity Relationship Analysis Using Newly Determined Cleavage Sites and Catalytic Efficiencies of an Oligopeptide Array — Predictions of Cleavability of Calpain Proteolysis by Quantitative Structure-Activity Relationship Analysis Using Newly Determined Cleavage Sites and Catalytic Efficiencies of an Oligopeptide Array — Proteomic QSAR Analysis of Calpain Substrate Specificity — Supplemental Data 

# Predictions of Cleavability of Calpain Proteolysis by Quantitative Structure-Activity Relationship Analysis Using Newly Determined Cleavage Sites and Catalytic Efficiencies of an Oligopeptide Array

## Supplemental Data

- Supplemental methods, Tables S1-S14, and Figures S1-S8 (.pdf, 35.0 MB) - Revised Supplemental methods, revised Supplemental Tables S1-S14, and revised Supplemental Figures S1-S8.
- Supplemental Table S15 (.xlsx, 9.1 MB) - All peptide lists identified in this study.
