# Supplementary material for: Predictions of Cleavability of Calpain Proteolysis by Quantitative Structure-Activity Relationship Analysis Using Newly Determined Cleavage Sites and Catalytic Efficiencies of an Oligopeptide Array
Source: Mol Cell Proteomics. 2016 Jan 21;15(4):1262–80. doi: 10.1074/mcp.M115.053413 (PMC4824854; doi:10.1074/mcp.M115.053413)
Supplement: Supplemental Data [file 10.1074_M115.053413_mcp.M115.053413-1.pdf]

SUPPLEMENTAL MATERIALS

Predictions of Cleavability of Calpain Proteolysis by Quantitative Structure-Activity Relationship Analysis Using Newly Determined Cleavage Sites and Catalytic Efficiencies of an Oligopeptide Array

Proteomic QSAR analysis of calpain substrate specificity

Fumiko Shinkai-Ouchi, Suguru Koyama, Yasuko Ono, Shoji Hata, Koichi Ojima, Mayumi Shindo, David duVerle, Mika Ueno, Fujiko Kitamura, Naoko Doi, Ichigaku Takigawa, Hiroshi Mamitsuka, and Hiroyuki Sorimachi

SUPPLEMENTAL EXPERIMENTAL PROCEDURES

Description of DKP modification for ProteinPilot™

Peptide identification

The rationale for calculating k_{cat}/K_m

SUPPLEMENTARY TABLE LEGENDS

Table S1. *List of reported substrates and their calpain cleavage sites.*

Table S2. *List of the 94 synthesized oligopeptides and their characteristics (P94mix library) The P87mix library excluded the shaded peptides.*

Table S3. *List of synthesized reference peptides used for quantification [P158mix library].*

Table S4. *Cleavability of all reported (Rp) sites.*

Table S5 *AAindex values showing a significant (*P*<0.05) difference between C1 and C2.*

Table S6 *Frequencies of aars significantly different between C1 and C2.*

Table S7. *Detailed summary of peptide and cleavage sites identified in this study.*

Table S8. *All calpain cleavage site sequences identified from fragment peptide spectra.*

Table S9. *All identified both-capped (uncleaved) peptides.*

Table S10. *Comparison of* k_{cat}/K_m *values of Rp and Nv sites in the same peptides.*

Table S11. *Amino acid descriptors (1D) used in this study.*

Table S12 *Amino acid descriptors (2D) used in this study.*

Table S13. *Aa descriptors used for QSAR model construction.*

Table S14. *Prediction results of calpain cleavage site predictors.*

SUPPLEMENTARY FIGURE LEGENDS

Figure S1. *Examples indicating that the proteolysis conditions used in this study were appropriate.*

Figure S2. *Relationships between the aa frequencies in Lit, Rp, and Nv.*

Figure S3. *Frequencies of aars proximal to the cleavage sites for C1 and C2.*

Figure S4. *Identification of Nv sites in human troponin T2.*

Figure S5. *Reaction efficiencies (*k_{cat}/K_m*) of calpain cleavage sites identified under the stringent condition.*

Figure S6. *Enzyme-substrate interaction maps of calpains or a caspase and their inhibitors.*

Figure S7. *Enzyme-substrate interaction of C2 and calpastatin.*

Figure S8. *Different interactions between calpastatin and CAPN1 versus CAPN2.*

SUPPLEMENTARY TABLES and FIGURES

SUPPLEMENTAL EXPERIMENTAL PROCEDURES

Description of DKP modification for ProteinPilot™

“N-Ac and C-DKP” and “N-Ac and C-DKP, cleavable” are added to the menu “Special Factors” of ProteinPilot™ by describing in ParameterTranslation.xml and ProteinPilot.DataDictionary.xml files of the software as follows:

ParameterTranslation.xml:

(1)<TRANSLATIONS>

<USER_INPUT_TRANSLATIONS>

<LIST name="Special factors">

At the end of the above section, the following two statements were added.

<ITEM name="N-Ac and C-DKP" value="MOD_FEATURE_SET:58"/>

<ITEM name="N-Ac and C-DKP, cleavable" value="MOD_FEATURE_SET:59"/>

(2)<!-- _______________ MODIFICATION SETS _________________ -->

<!-- SPECIAL FACTOR MODIFICATION SETS - USE SET NUMBERS IN RANGE 41-60 -->

At the end of the above section, the following statements were added.

<MOD_FEATURE_SET xml:id="MOD_FEATURE_SET:58" name="N-Ac and C-DKP">

<MOD_FEATURE mod="Terminal Acetyl">

<OCCURRENCE target="" term_spec="PepNTerm" prob="0.5"/>

</MOD_FEATURE>

<MOD_FEATURE mod="Protein Terminal Acetyl">

<OCCURRENCE target="" term_spec="ProtNTerm" prob="0.95"/>

</MOD_FEATURE>

<MOD_FEATURE mod="Terminal DKP">

<OCCURRENCE target="" term_spec="PepCTerm" prob="0.5"/>

</MOD_FEATURE>

```
    <MOD_FEATURE mod="Protein Terminal DKP">
        <OCCURRENCE target="" term_spec="ProtCTerm" prob="0.95"/>
    </MOD_FEATURE>
</MOD_FEATURE_SET>

<MOD_FEATURE_SET xml:id="MOD_FEATURE_SET:59" name="N-Ac and C-DKP, cleavable">
    <MOD_FEATURE mod="Terminal Acetyl">
        <OCCURRENCE target="" term_spec="PepNTerm" prob="0.5"/>
    </MOD_FEATURE>
    <MOD_FEATURE mod="Protein Terminal Acetyl">
        <OCCURRENCE target="" term_spec="ProtNTerm" prob="0.95"/>
    </MOD_FEATURE>
    <MOD_FEATURE mod="Terminal DKP">
        <OCCURRENCE target="" term_spec="PepCTerm" prob="0.4"/>
    </MOD_FEATURE>
    <MOD_FEATURE mod="Protein Terminal DKP">
        <OCCURRENCE target="" term_spec="ProtCTerm" prob="0.4"/>
    </MOD_FEATURE>
</MOD_FEATURE_SET>
```

ProteinPilot.DataDictionary.xml:

In the “XENOBIOTIC MODIFICATIONS” section, the following statements were added:

```
    <Mod rKey="0">
            <Nme>Terminal DKP</Nme>
            <DisplayName>DKP</DisplayName>
            <TLC>DKP</TLC>
```

<TS>10</TS>

<Fma>C11H18O2N3</Fma>

<RpF>OH</RpF>

<NLF></NLF>

<IIF></IIF>

<Chg>0</Chg>

</Mod>

<Mod rKey="0">

<Nme>Protein Terminal DKP</Nme>

<TLC>DKP</TLC>

<TS>8</TS>

<Fma>C11H18O2N3</Fma>

<RpF>OH</RpF>

<NLF></NLF>

<IIF></IIF>

<Chg>0</Chg>

</Mod>

Peptide identification

Several truncated peptides were included as by-products of peptide synthesis, and were detected by MS analysis. Considering that peptides were synthesized from the C-terminus starting with a DKP modified aa, the following criteria for identification of peptides were used:

Examples of ID005:MBP (the following samples include those not identified in actuality):

Sequence and modification identified by ProteinPilot™	Assignment
$_{\text{Ac-}}$SQRSK$_{\text{(-iT*)}}$YLASASTMDHARHGF$_{\text{-DKP}}$	full-length uncleaved (BC) peptide
$_{\text{Ac-}}$LASASTMDHARHGF$_{\text{-DKP}}$	BC peptide synthesized in N-terminally truncated form
$_{\text{Ac-}}$SQRSK$_{\text{(-iT)}}$YLASA$_{\text{-COOH}}$	cleaved fragment at the C-terminus (Rp site)
$_{\text{iT-}}$STMDHARHGF$_{\text{-DKP}}$	cleaved fragment at the N-terminus (Rp site)
$_{\text{Ac-}}$SQRSK$_{\text{(-iT)}}$YLASAST$_{\text{-COOH}}$	cleaved fragment at the C-terminus (Nv site)
$_{\text{iT-}}$MDHARHGF$_{\text{-DKP}}$	cleaved fragment at the N-terminus (Nv site)
$_{\text{Ac-}}$LASASTMDHAR$_{\text{-COOH}}$	cleaved fragment at the C-terminus of a peptide synthesized in N-terminally truncated form
$_{\text{iT-}}$RSK$_{\text{(-iT)}}$YLASAST$_{\text{-COOH}}$	cleaved fragment at the both N- and C-termini (Rp and/or Nv sites)
$_{\text{Ac-}}$SQRSK$_{\text{(-iT)}}$YLASASTMDHARHGF$_{\text{-COOH}}$	DKP-hydrolyzed peptide (For detection of possible amidase-like activity of calpains, this structure was enabled to be searched by selecting “N-Ac and C-DKP, cleavable” option. In actuality, this kind of peptides was not detected.)

The following structures are highly unlikely, and were considered mis-identification by ProteinPilot™, if any.

$_{\text{Ac-}}$SQRSK$_{\text{(-iT)}}$YLASASTMDHARHG$_{\text{-DKP}}$	BC peptide synthesized in C-terminally truncated form
$_{\text{iT-}}$ASTMDHARHG$_{\text{-DKP}}$	cleaved fragment at the N-terminus of a peptide synthesized in C-terminally truncated form

* -iT: iTRAQ modification.

The rationale for calculating k_{cat}/K_m

Suppose that N kinds of substrates (1, 2, …, N), at concentrations S_1, S_2, …, S_N, respectively, are digested by a proteolytic enzyme (E) at the same time. Each substrate (i) has Mi kinds of proteolytic sites (i_1, i_2, …,

i_{Mi}), producing 2Mi kinds of proteolytic products, *i.e.*, P_{i1}+P'_{i1}, P_{i2}+P'_{i2}, …, $P_{i_{Mi}}$+$P'_{i_{Mi}}$ after forming the intermediate enzyme-substrate complexes, $(ES_i)_j$ and EP_{ij} ($j_{(i)} = 1, 2, \dots, Mi$), respectively.

The reactions are presented as follows with apparent rate constants, $k_{1_{ij}}, k_{-1_{ij}}, k_{2_{ij}}, k_{3_{ij}}$ for each element reaction focusing on proteolytic site $j_{(i)}$:

$$E + S_i \underset{k_{-1_{ij}}}{\overset{k_{1_{ij}}}{\rightleftarrows}} (ES_i)_j \overset{k_{2_{ij}}}{\longrightarrow} EP_{ij}(+P'_{ij}) \overset{k_{3_{ij}}}{\longrightarrow} E + P_{ij} + P'_{ij}$$

$$(i = 1, 2, \dots, N;\ j_{(i)} = 1, 2, \dots, Mi)$$

$$\text{Let } v_{ij} = \frac{d[P_{ij}]}{dt} = \frac{d[P'_{ij}]}{dt} = k_{2_{ij}}[(ES_i)_j], \text{and Et} = \text{total enzyme concentration (constant).}$$

Since calpains' k_{cat}s for oligopeptides are known to be markedly lower than papain [*ca.* 1/100, Sasaki, T. *et al.* (1984) Comparative specificity and kinetic studies on porcine calpain I and calpain II with naturally occurring peptides and synthetic fluorogenic substrates. *J. Biol. Chem.* 259, 12489-12494], every reaction is supposed to be in a static state under the condition used (total [S] = 0.29 to 1.7 mM *vs.* Et =2.5 μM, and reaction time (T) of 15 min, which may not be ideal, but higher [S], lower Et, or shorter T was not feasible, because the observed initial velocities of some peptides were already at the minimum for accurate measurement). Then:

$$\forall i,j \quad \frac{d[EP_{ij}]}{dt} = k_{2_{ij}}[(ES_i)_j] - k_{3_{ij}}[EP_{ij}] = 0 \quad \therefore [EP_{ij}] = \frac{k_{2_{ij}}}{k_{3_{ij}}}[(ES_i)_j]$$

Let $K_{s_{ij}} = \frac{[E][S_i]}{[(ES_i)_j]}$ and then: $[E] = K_{s_{ij}} \frac{[(ES_i)_j]}{[S_i]}$

$$\therefore \forall i,j,i',j' \quad [E] = K_{s_{ij}} \frac{[(ES_i)_j]}{[S_i]} = K_{s_{i'j'}} \frac{[(ES_{i'})_{j'}]}{[S_{i'}]} = K_{s_{11}} \frac{[(ES_1)_1]}{[S_1]}$$

$$\therefore \forall i,j \quad [(ES_i)_j] = \frac{K_{s_{11}}}{K_{s_{ij}}} \frac{[S_i]}{[S_1]} [(ES_1)_1]$$

By definition,

$$Et = [E] + \sum_{i=1}^{N} \sum_{j=1}^{M_i} [(ES_i)_j] + \sum_{i=1}^{N} \sum_{j=1}^{M_i} [EP_{ij}]$$

$$= K_{s_{11}}\frac{[(\mathrm{ES}_1)_1]}{[\mathrm{S}_1]} + \sum_{i=1}^{\mathrm{N}}\sum_{j=1}^{\mathrm{M}_i}\frac{K_{s_{11}}}{K_{s_{ij}}}\frac{[\mathrm{S}_i]}{[\mathrm{S}_1]}[(ES_1)_1] + \sum_{i=1}^{\mathrm{N}}\sum_{j=1}^{\mathrm{M}_i}\frac{k_{2_{ij}}}{k_{3_{ij}}}(\frac{K_{s_{11}}}{K_{s_{ij}}}\frac{[\mathrm{S}_i]}{[\mathrm{S}_1]}[(ES_1)_1])$$

$$= [1 + \sum_{i=1}^{\mathrm{N}}\sum_{j=1}^{\mathrm{M}_i}\frac{[\mathrm{S}_i]}{\mathrm{K}_{s_{ij}}}\left(1 + \frac{k_{2_{ij}}}{k_{3_{ij}}}\right)]K_{s_{11}}\frac{[(\mathrm{ES}_1)_1]}{[\mathrm{S}_1]}$$

$$= [1 + \sum_{i=1}^{\mathrm{N}}[\mathrm{S}_i]\sum_{j=1}^{\mathrm{M}_i}\frac{1}{K_{s_{ij}}}(\frac{k_{2_{ij}} + k_{3_{ij}}}{k_{3_{ij}}})]K_{s_{11}}\frac{[(\mathrm{ES}_1)_1]}{[\mathrm{S}_1]}$$

$$= \left(1 + \sum_{i=1}^{\mathrm{N}}[\mathrm{S}_i]\sum_{j=1}^{\mathrm{M}_i}\frac{1}{K_{m_{ij}}}\right)K_{s_{11}}\frac{[(\mathrm{ES}_1)_1]}{[\mathrm{S}_1]}$$

$$= \left(1 + \sum_{i=1}^{\mathrm{N}}\frac{[\mathrm{S}_i]}{K_{m_i}}\right)K_{s_{11}}\frac{[(\mathrm{ES}_1)_1]}{[\mathrm{S}_1]}\ ,$$

where $K_{m_{ij}} = \frac{k_{3_{ij}}}{k_{2_{ij}} + k_{3_{ij}}}K_{s_{ij}}$ and $K_{m_i} = (\sum_{j=1}^{\mathrm{M}_i}\frac{1}{K_{m_{ij}}})^{-1}$

$$\therefore\ [(\mathrm{ES}_1)_1] = \frac{\mathrm{Et}[\mathrm{S}_1]}{(1 + \sum_{i=1}^{\mathrm{N}}\frac{[\mathrm{S}_i]}{K_{m_i}})K_{s_{11}}} = \frac{\mathrm{Et}[\mathrm{S}_1]/K_{s_{11}}}{1 + \sum_{i=1}^{\mathrm{N}}\frac{1}{K_{m_i}}[\mathrm{S}_i]}$$

Then, $v_{ij} = k_{2_{ij}}[(\mathrm{ES}_i)_j] = k_{2_{ij}}\frac{K_{s_{11}}}{K_{s_{ij}}}\frac{[\mathrm{S}_i]}{[\mathrm{S}_1]}[(\mathrm{ES}_1)_1] = k_{2_{ij}}(\frac{[\mathrm{S}_i]\mathrm{Et}}{K_{s_{ij}}})\frac{1}{1+\sum_{i'=1}^{\mathrm{N}}\frac{1}{K_{m_{i'}}}[\mathrm{S}_{i'}]}$

Let $[\mathrm{S}_1] = \mathrm{r}_1[\mathrm{S}_0], [\mathrm{S}_2] = \mathrm{r}_2[\mathrm{S}_0], \ldots, [\mathrm{S}_i] = \mathrm{r}_i[\mathrm{S}_0], \ldots, [\mathrm{S}_\mathrm{N}] = \mathrm{r}_\mathrm{N}[\mathrm{S}_0]$ and $k_{\mathrm{cat}_{ij}} = k_{2_{ij}}\frac{K_{m_{ij}}}{K_{s_{ij}}} = \frac{k_{2_{ij}}k_{3_{ij}}}{k_{2_{ij}}+k_{3_{ij}}}$,

where $[\mathrm{S}_0]$ is the average of all $[\mathrm{S}_i]$. Then:

$$v_{ij} = (\frac{k_{\mathrm{cat}_{ij}}\mathrm{Et}}{K_{m_{ij}}})\frac{\mathrm{r}_i[\mathrm{S}_0]}{1 + [\mathrm{S}_0]\sum_{i'=1}^{\mathrm{N}}\frac{\mathrm{r}_{i'}}{K_{m_{i'}}}}$$

$$= (\frac{k_{\mathrm{cat}_{ij}}\mathrm{Et}}{K_{m_{ij}}})\frac{\mathrm{r}_i}{\frac{1}{[\mathrm{S}_0]} + \frac{1}{K_{m_t}}}$$

where $Km_t = (\sum_{i'=1}^{\mathrm{N}}\frac{\mathrm{r}_{i'}}{K_{m_{i'}}})^{-1} = (\sum_{i'=1}^{\mathrm{N}}\sum_{j'=1}^{\mathrm{M}_{i'}}\frac{\mathrm{r}_{i'}}{K_{m_{i'j'}}})^{-1}$

$$\therefore\ v_{ij} = \frac{r_i k_{\text{cat}_{ij}}}{K_{m_{ij}}} \text{Et} \frac{1}{\frac{1}{K_{m_t}} + \frac{1}{[\text{S}_0]}}$$

$$\therefore\ \frac{1}{v_{ij}} = \frac{K_{m_{ij}}}{\text{r}_i k_{\text{cat}_{ij}}} \frac{1}{\text{Et}} (\frac{1}{K_{m_t}} + \frac{1}{[\text{S}_0]})$$

In our experiments, $\text{r}_1 = \text{r}_2 = \cdots = \text{r}_i = \cdots = \text{r}_\text{N} = 1$, thus:

$$\frac{1}{v_{ij}} = \frac{K_{m_{ij}}}{k_{\text{cat}_{ij}}} \frac{1}{\text{Et}} \left(\frac{1}{K_{m_t}} + \frac{1}{[\text{S}_0]} \right) \leftrightarrow v_{ij} = \frac{k_{\text{cat}_{ij}}}{K_{\text{m}_{ij}}} \text{Et} K_{m_t} - K_{m_t} \frac{v_{ij}}{[\text{S}_0]}$$

$$\text{where } Km_t = (\sum_{i'=1}^{\text{N}} \frac{1}{K_{m_{i'}}})^{-1} = (\sum_{i'=1}^{\text{N}} \sum_{j'=1}^{\text{M}_{i'}} \frac{1}{K_{m_{i'j'}}})^{-1}$$

Therefore, in the case of this study, $\frac{k_{\text{cat}_{ij}}}{K_{\text{m}_{ij}}} = -\frac{\mathbf{b}_{ij}}{\mathbf{a}_{ij}\text{Et}}$ where $\mathbf{a}_{ij}$ and $\mathbf{b}_{ij}$ are the intercept and the slope, respectively, of the regression line of a v_{ij} (y-axis) *vs* $\frac{v_{ij}}{[\text{S}_0]}$ (x-axis) plot (Eadie-Hofstee plot), or $\frac{k_{\text{cat}_{ij}}}{K_{\text{m}_{ij}}} = \frac{1}{\mathbf{b}_{ij}\text{Et}}$ where $\mathbf{b}_{ij}$ is the slope of the regression line of a $\frac{1}{v_{ij}}$ (y-axis) *vs* $\frac{1}{[\text{S}_0]}$ (x-axis) plot (Lineweaver-Burk plot).

SUPPLEMENTARY TABLE LEGENDS

Table S1. List of reported substrates and their calpain cleavage sites.

Notes: *1 This chicken sequence is incorrectly referred to as CAPN1 in the NCBI database, but it has been evolutionarily shown to be the homolog of human CAPN11 (117).

*2 CAPN1 cuts after G311 and K320, while CAPN2 cuts only after G311.

*3 SB0042 and SB0120 are the same protein (and site 115 is identical), but the calpains used for proteolysis differed.

*4 SB0052 and SB0078 are different isoforms of the same protein, but different cleavages are reported.

*5 CAPN1 cuts after K316 and R324, while CAPN2 cuts only after K309.

*6 SB0063 and SB0079 are different isoforms of the same protein, but different cleavages are reported.

*7 The CysPc domain of CAPN1 was used for cleavage.

*8 SB0100 and SB0147 are the same protein, but the calpains used for proteolysis differed.

*9 SB0101 and SB0148 are the same protein, but the calpains used for proteolysis and their sites differed.

*10 In this report, the cleavage experiments used chicken ACTN1, but the sequence numbers reported (243 and 246) correspond to those of the human protein (NP_001123476, 98% identical to chicken NP_989458; His is inserted between Asp2 and His3 of the human sequence). Here, the correct chicken sequence numbers are shown (244 and 247). Cleavage at 244 was only shown for calpain-2, and was described as being very minor.

*11 The species for STEP was not given; judging from the sequence, it is probably human, and the human sequence is referred to here.

*12 Abbreviations for species: *B. taurus, Bos taurus* (bovine); *C. harengus, Clupea harengus* (Atlantic herring); *C. pallasii, Clupea pallasii* (Pacific herring); *G. gallus, Gallus gallus* (chicken); *H. sapiens, Homo sapiens* (human); *M. musculus, Mus musculus* (mouse); *O. aries, Ovis aries* (ovine); *O. cuniculus, Oryctolagus cuniculus* (rabbit); *R. norvegicus, Rattus norvegicus* (rat); *S. scrofa, Sus scrofa* (porcine).

Table S2. List of 94 synthesized oligopeptides and their characteristics (P94mix library). The P87mix library excluded the shaded peptides.

Notes: *1 "SB No." is the substrate number used in the CaMP DB web site (http://www.calpain.org/browse.rb?cls=substrate).

*2 For species abbreviations, see Table S1.

*3 Numbers indicate the positions of cleavage sites in the peptides relative to the N-terminus, with the 1st aar being 1. Although most oligopeptides were designed so the cleavage site was 10, some were displaced from 10 to reduce the peptide's hydrophobicity. Note that about half of the peptides were designed to have only one cleavage site, and the rest have plural sites.

*4 The calpain cleavage site is the C-terminus of the aar indicated by number, which is based on the full-length sequence indicated in "gi#."

*5 "unclear" indicates that the calpain used in the study was not clear. 1, 2, and 3 indicate calpain-1 (C1, CAPN1/S1, μ-calpain), calpain-2 (C2, CAPN2/S1, m-calpain), and calpain-3 (CAPN3, p94), respectively.

*6 Peptides indicated by shaded rows were not included in the kinetics experiments, because our preliminary MS analysis could not detect any spectra corresponding to them.

*7 "sc" indicates a scrambled sequence of the peptide ID listed under "Definition."

Table S3. List of synthesized reference peptides used for quantification (P158mix library).

Peptides corresponding to the same site (*i.e.*, the N- and C-terminal peptides of the same site) are indicated under "Site identical to." Nos. 1 to 104, and 105 to 158 correspond to Rp and Nv sites, respectively.

Table S4. Cleavability of all reported (Rp) sites.

All (133) Rp sites in the P87mix peptides are listed by ID and the position ("site") of each site. N, C, and M indicate the number of independent peptide(s) identified as N-terminally Ac-capped, C-terminally DKP-capped, and both-cut (no capped) proteolyzed fragments that contained the Rp site uncleaved (*e.g.*, if a peptide

spanning positions 6 to 20 (a cleaved C-terminal fragment) was detected for a peptide with an Rp site after position 10, the count for "C" increased one). FL indicates the ratio of the iTRAQ^{TM}-115 signal to that of iTRAQ^{TM}-113, if the full-length both-capped peptide was identified for that ID (see the legend for Fig. S1E, F). The "cleaved" and "uncleaved" values are the numbers of independent peptides cleaved and uncleaved at the site, respectively. The number in "cleaved" and "uncleaved" is sometimes >1, because different peptides for the same site were counted independently. Site 10 of ID43 and site 4 of ID54 did not exist due to truncated synthesis of the peptide library (indicated by gray shading).

Table S5. AAindex values showing a significant (*P*<0.05) difference between C1 and C2.

All of the P10-P10' cleavage site sequences of C1 or C2 were aligned, and the frequency of each aar at each position was multiplied by each aa descriptor value (weighted average), and compared between C1 and C2. "C1", "C2", and "Ave." indicate the weighted averages of aa descriptors for the cleavage site sequences of C1 and C2, and for the standard (Sprot) aa composition, respectively. *P*, by *Z*-test for the equality of two proportions (binomial distribution).

Table S6. Frequencies of aars that were significantly different between C1 and C2.

The P10-P10' cleavage site sequences specific for C1 or C2 were aligned, and the frequency of each aar at each position was compared between C1 and C2 (see Figs. 3C-E). The significantly different aars are shown with their frequencies (%) and significance. *P*, *Z*-test for the equality of two proportions (binomial distribution).

Table S7. Summary of the peptides and cleavage sites identified in this study.

Notes: *1 Total number is the sum of the sites identified by 4800 and QSTAR Elite after eliminating shared and discordant peptides.

*2 Peptides with the same sequence but different modifications are identified as distinct peptides.

*3 Theoretically, the maximum number of possible sites is 1,652 (87 reported, and 1,565 novel sites); however, several other sites were identified from the cleavages of truncated synthetic peptides.

*4 Maximum number of sites is 80 and 50 for reported and novel sites, respectively, according to the number of reference peptides (see Table S3).

*5 Abbreviations used in this table: Rp, reported sites; Nv, novel sites; BC, both-side capped peptides; FL, full-length peptides.

Table S8. All calpain cleavage site sequences identified from fragment peptide spectra.

Notes: *1 Full-length peptides were not identified for P87 ID Nos. 38, 43, and 54 (A) and 38, 43, 54, and 55 (B), so the estimated longest sequence (38: TRKISQTAQTYDPRE, 43: LCIGNH, and 54: PLYKEATSTFTNI for A; 38: TRKISQTAQTYDPRE, 43: LCIGNH, 54: PLYKEATSTFTNI, and 55: PLYKSAITTTINP for B) was used to estimate the cleavage site sequences instead of the full-length sequence.

*2 Numbers indicate the position of the P1 aar in the whole protein sequence.

*3 1: cleaved fragment detected; 0: cleaved fragment not detected.

*4 Abbreviations used in this table: Ave, the average of the k_{cat}/K_m values for C1 and C2; Rp, reported site; Nv, novel site; N, C, M, and T, number of spectra identified for N-terminal, C-terminal, or internal sites [*e.g.*, cleavage sites in a both-side cleaved (uncapped) peptide fragment], and sum of them all, respectively; for protein names, see Table S2.

Table S9. All identified both-capped (uncleaved) peptides.

Notes: *1 Numbers 1-20 correspond to the positions of the originally designed full-length (FL) oligopeptides, which are indicated in boldface type.

*2 Abbreviations used: Ave., average; #sp., number of spectra identified; for protein names, see Table S2.

*3 "-" indicates that the value could not be calculated regardless of the existence of one or more spectra, whereas blank means no spectrum was available.

Table S10. Comparison of k_{cat}/K_m values of Rp and Nv sites in the same peptides.

Averages, standard deviations, and the total numbers of sites are indicated below the table.

Notes: *: Vertical lines (|) indicate Rp sites, and colons (:) indicate Nv sites, which are indicated in the "site positions" column (k_{cat}/K_m values are listed in the order corresponding to that of the site positions).

**: The average k_{cat}/K_m values of Rp sites that had Nv site(s) in the same peptide were not significantly different from those of the Nv sites (P=0.330; t-test for two population means by the method of paired comparisons).

Table S11. Amino acid descriptors (1D) used in this study.

Ref: AAindex, from the amino acid index database, http://www.genome.jp/aaindex/; Jpred 3: predicted by the web site, http://www.com/pbio.dundee.ac.uk/www-jpred/; MOE: calculated for 20 aas by the molecular descriptor calculation of the MOE software Ver.2013.08; all missing values were replaced by the average values.

Table S12. Amino acid descriptors (2D) used in this study.

Ref: AAindex: from the amino acid index database, http://www.genome.jp/aaindex/; MOE: calculated for 400 aa combinations by the molecular descriptor calculation of the MOE software Ver.2013.08; all missing values were replaced by the averages.

Table S13. Aa descriptors used for QSAR model construction.

For the aa descriptors, see Tables S11 (1D) and S12 (2D).

Table S14. Results of calpain cleavage site predictors.

Notes: *1: Names are presented as: "SB No."_"protein name"_["aar number of the proteolytic site in the original sequence, which corresponds to the middle of the following sequence"]

*2: Abbreviations for predictors, see Table 4.

*3: 1 and 0 indicate that they were not used and used in the construction of the GPS or SVL/PSSM/MKL predictors.

*4: 1 and 0 indicate that they were predicted to be cleaved and uncleaved, respectively.

*5: Probability predicted by our binary-QSAR model.

SUPPLEMENTARY FIGURE LEGENDS

FIGURE S1. Examples indicating that the proteolysis conditions used in this study were appropriate.

(*A*-*D*) Examples of the apparent reaction efficiency (k_{cat}/K_m) of peptide cleavages by calpain determined by the Lineweaver-Burk plots. The Lineweaver-Burk plots and regression lines are shown for the data acquired for LPENNVLSPL$_{\text{-DKP}}$ [*A*, ID073; TP53; low efficiency (k_{cat}/K_m ~ 10 $M^{-1}s^{-1}$)], KVNGAPREDA$_{\text{-DKP}}$ [*B*, ID064; IGFBP4; low efficiency (k_{cat}/K_m ~ 10 $M^{-1}s^{-1}$)], SEPPVYANLS$_{\text{-DKP}}$ [C, ID048; JUN; medium efficiency (k_{cat}/K_m ~ 800 $M^{-1}s^{-1}$)], and SVPSSKLLGN$_{\text{-DKP}}$ [*D*, ID040; GRIN2A; high efficiency (k_{cat}/K_m ~ 1700 $M^{-1}s^{-1}$)] cleaved by C1. Each sample set shown by the different markers and line colors corresponds to an independently identified spectrum of the same peptide. The apparent cleavage efficiency (k_{cat}/K_m, $M^{-1}s^{-1}$) of each peptide was calculated by the 1/slope/$[E]_t$ ($[E]_t$ = 2.5x10^{-6} M) of the regression line (each k_{cat}/K_m value is shown in the graphs). For all of the determined k_{cat}/K_m values, see Figs. 4 and S8.

(*E*, *F*) Degree of the degradation of full-length peptides by calpains under the condition used. After MS analysis of P87mix peptides proteolyzed by C1 or C2 under the final conditions used in the manuscript (20-3.3 μM of each peptide and 2.5 μM of C1 or C2 at 30°C for 15 min), 55 and 56 were identified as full-length, both-capped peptides for C1 (*E*) and C2 (*F*), respectively. Next, the ratio of the iTRAQ^{TM}-115 signal (corresponding to 10 μM peptide proteolyzed by calpain for 15 min) to the iTRAQ^{TM}-113 signal (corresponding to undigested 10 μM peptide) was calculated and plotted. "n" under the "ID" numbers indicates the number of spectra identified for each ID. Error bars: standard error (for n > 1).

FIGURE S2. Relationships between the aa frequencies in Lit, Rp, and Nv.

(*A*) The aa frequencies were compared at each position between Lit (420 sites) and the Nv subset of P87 (360 sites in the P87 cleaved sequences) standardized by the standard aa compositions (1), or between the Rp and Nv subsets of P87 (123 and 360 sites, respectively) (2), and their *r*'s were plotted. All of the positions showed significant correlation ($P < 0.0001$) by the Kendall rank correlation test, and none of the positions showed a significant difference between (1) and (2) by a *Z*-test for correlation coefficients ($P > 0.05$). (*B*) The aa frequencies at all positions (P10-P10') were plotted for P87-Rp and -Nv. They showed an *r* of 0.640 (significantly correlated ($P = 2.19\times10^{-47}$) by a *t*-test for correlation coefficients). (*C*) The number of aars

available for each position. Since the oligopeptides in the P87 library were only 20-mers, not all of the positions P10-P10’ for Nv and Rp had aars for some sites, other than those in the center of the peptide. For example, if a cleavage site was after position 4 of a 20-mer peptide, P4-P16’ would have aars, but P10-P5 would not. For Lit, much longer peptide sequences were used, and, thus, the aars were almost uniformly available for all the positions; however, if a cleavage site was close to the N- or C-terminus of the protein, a similar aar unavailability at the positions distal to the site occurred infrequently. The maximum number is the number of sequences, *i.e.*, 420, 360, 123, and 903 for Lit, Nv, Rp, and all, respectively. Note that both Nv and Rp showed fewer aars at the P10’ position than at P10, meaning that there were more missing aars at the C-terminus than at the N-terminus. In other words, more Nv and Rp sites were in the C-terminal half than in the N-terminal half.

FIGURE S3. Frequencies of aars proximal to the cleavage sites for C1 and C2.

The P10-P10’ cleavage site sequences identified in our *in vitro* experiments were aligned for C1 (*A*, 418 sites) or C2 (*B*, 360 sites), and their occurrence rates were represented by WebLogo 3 (http://weblogo.threeplusone.com). The numbers of identified sites are summarized in (*C*). The aa frequencies at each position (P10-P10’) were compared for C1 and C2 for all of the site sequences (black bars) or for those specific for each calpain (blue bars), and their *r*‘s were plotted (*D*). P9 and P9’ of the sites specific for each calpain showed no significant correlation (P>0.05) by the Kendall rank correlation test (*).

FIGURE S4. Identification of Nv sites in human troponin T2.

(*A*) In this experiment, mouse troponin T2 (TNNT2, ID007) was cleaved at three Nv sites (blue arrows) but not at a Rp site (black arrow). Human heart TNNT2 (Merck 648484-100UGA) was commercially available, and human and mouse TNNT2 are highly similar. Thus, human TNNT2 was incubated with calpain-1, and the digest was subjected to N-terminal peptide sequencing and MS analysis. The N-terminal sequencing detected NH_2-VPPKI (data not shown, underlined), indicating cleavage at site (2). MS analysis detected two sites (red arrows with (1) and (2) in *A*). MS/MS data for sites (1) and (2) are shown in (*B*)+(*C*) and (*D*)+(*E*), respectively.

FIGURE S5. Reaction efficiencies (k_{cat}/K_m) of calpain cleavage sites identified under the stringent condition.

Cleavage efficiencies (k_{cat}/K_m ($M^{-1}s^{-1}$)) of sites determined using fragment peptide data identified under the stringent condition are shown for both C1 and C2 (*A*). The k_{cat}/K_m values of a peptide determined for the both calpains were plotted, and showed high correlation (r = 0.919) (*B*). Results obtained using identifications made under normal (see Fig. 4) and stringent (this figure) conditions were compared (*C*), and showed good correlation (r = 0.850). The numbers before the protein (gene product) names are the peptide ID No., and the numbers in brackets are the positions of cleavage sites (*e.g.*, -2, -1, 0, and 1 indicate cleavage at position 8, 9, 10, and 11, respectively). Error bars: SE.

FIGURE S6. Enzyme-substrate interaction maps of calpains or a caspase and their inhibitors.

Interaction maps were drawn by Ligand Interaction in the MOE software using default parameters for rat CAPN2-calpastatin:M167–G176 (*A*) or CAPN2-calpastatin:T181–L190 (*B*) from the Protein Data Bank (PDB) entry 3df0, rat CAPN1-leupeptin from 1tl9 (*C*), and human caspase 8-Z-DEVD-CHO from 1f9e (*D*). In *A* and *B*, the atom interactions (green dotted and blue lines) observed in both 3df0 and 3bow are shown, and the distance and energy are the average of both structures. In *C* and *D*, all of the interactions are shown.

FIGURE S7. Enzyme-substrate interaction of C2 and calpastatin.

The surface of the CAPN2 molecule near calpastatin M167 (P10) to G176 (P1) (*A*) or T181 (P1’) to L190 (P10’) (*B*) was drawn by Surfaces and Maps/Electrostatic Map in the MOE software using the PDB entry, 3df0. Hydrophobic and hydrophilic interactions were predicted by the Surfaces and Maps/Contact Preference in the MOE software using default parameters. Red and blue on the surface indicate negative and positive regions, respectively. Transparent green and magenta surfaces indicate hydrophobic and hydrophilic contacts, respectively, between CAPN2 and the L177 (P2) (*A*) or P183 (P3’) of calpastatin (*B*) atoms. Stereograms are presented in cross-eye mode.

FIGURE S8. Different interactions between calpastatin and CAPN1 versus CAPN2.

Ribbon schemes of the 3D structures of the C2+calpasitatin complex (3df0) superposed on CAPN1:CysPc+leupeptin (1tl9) were drawn by MOE Ver.2014.0901 (the CysPc domains of CAPN1 and CAPN2 were superposed) focusing on S169–T170 (corresponding to P8-P7, *A*) and E185 (P5', *B*) of calpastatin. Red, blue, and pink represent CAPN1, CAPN2, and calpastatin, respectively (leupeptin is invisible here). Stereograms are presented in cross-eye mode.

Table S1. List of reported substrates and their calpain cleavage sites.

SB No.	Gene product	Description	Species*12	gi#	Reference sequence	length	# of sites	Sites	Calpain	Ref.	Note
SB0001	CAPN2	CAPN2, m-calpain catalytic subunit, mCL	*H. sapiens*	4502563	NP_001739	700	2	9, 19	2	1	
SB0002	ITGB2	Integrin β2	*H. sapiens*	89191865	NP_000202	769	4	744, 754, 760, 762	2	2	
SB0003	MIP	Aquaporin-0	*R. norvegicus*	34862195	XP_343138	263	1	238	unclear	3	
SB0004	PDE1A	Calcium/calmodulin-dependent 3',5'-cyclic nucleotide phosphodiesterase1A	*B. taurus*	162879	AAA74560	530	1	126	unclear	4	
SB0005	SPTBN1	β-II-spectrin, non-erythrocytic 1 isoform 1	*H. sapiens*	112382250	NP_003119	2364	1	2066	1	5	
SB0006	CAMK4	Calcium/calmodulin-dependent protein kinase IV	*M. musculus*	6753252	NP_033923	469	2	23, 199	2	6	
SB0007	GRM1	Glutamate receptor, metabotropic 1	*R. norvegicus*	8393487	NP_058707	1199	1	936	1	7	
SB0008	PLCB1	Phosphoinositide-specific phospholipase Cβ1	*B. taurus*	27807357	NP_777242	1216	1	880	2	8	
SB0009	NF2	Neurofibromin 2, Merlin	*H. sapiens*	4557795	NP_000259	595	2	294, 298	1 and 2	9	
SB0010	NEFM	Neurofilament medium polypeptide, Neurofilament 3	*B. taurus*	119923501	XP_582135	931	2	467, 516	1	10	
SB0011	NFKBIA	IkB-α, NFkB inhibitor α	*H. sapiens*	10092619	NP_065390	317	1	50	2	11	
SB0012	HIST1H2BC	Histone H2B type 1	*B. taurus*	119936491	ABM06136	126	14	6, 20, 40, 42, 46, 53, 64, 81, 96, 101, 105, 106, 107, 116	unclear	12	
SB0013	SLC6A5	Solute carrier family 6, Gly transporter 2	*R. norvegicus*	42627863	NP_976079	799	2	156, 164	1 and/or 2	13	
SB0014	ATG5	ATG5-like	*H. sapiens*	4757798	NP_004840	275	1	193	1 and/or 2	14	
SB0015	RYR1	Ryanodine receptor	*O. cuniculus*	1714	CAA33279	5037	2	1400, 2843	2	15	
SB0016	GJA8	Connexin-49, Gap junction α-8	*O. aries*	6014503	AAF01367	440	2	290, 300	unclear	16	
SB0017	CAPNS1	CAPNS1, 30K, Calpain regulatory subunit	*G. gallus*	2506056	BAA22638	214	2	32, 35	unclear	17	
SB0018	PHKG	Phosphorylase b kinase γ catalytic chain	*O. cuniculus*	1660	CAA68682	387	1	303	2	18	
SB0019	IL1A	IL-1 α, Interleukin 1 α	*H. sapiens*	27894330	NP_000566	271	1	118	1	19	
SB0020	CAPN1	CAPN1, μ-calpain catalytic subunit, μCL	*H. sapiens*	12408656	NP_005177	714	2	15, 27	1	20	
SB0021	CAPN11	CAPN11, μ/m-calpain catalytic subunit, μ/mCL	*G. gallus*	45384280	NP_990634	705	1	17	unclear	17	*1
SB0022	CTTN	Cortactin	*H. sapiens*	20357552	NP_005222	550	4	336, 346, 351, 358	2	21	
SB0023	IGFBP5	IGFBP-5, Insulin-like growth factor binding protein 5	*H. sapiens*	10834982	NP_000590	272	3	22, 161, 172	1	22	
SB0024	PTPRN	Protein tyrosine phosphatase, receptor type, N	*H. sapiens*	4506321	NP_002837	979	2	608, 658	1	23	
SB0025	SAG	S-arrestin	*B. taurus*	162670	AAA30377	404	2	377, 385	unclear	24	
SB0026	BAX	BAX, BCL2-associated X protein	*H. sapiens*	20631958	NP_620116	192	1	28	1 or 2	25	
SB0027	AGT	Angiotensin	*R. norvegicus*	19705570	NP_602308	477	1	28	1 and 2	26	
SB0028	(PRTMY)	Protamine-YII	*C. harengus*	59800156	P69008	30	2	14, 22	unclear	27	
SB0029	TNNT3	Troponin T3, fast skeletal muscle, TnTf	*O. cuniculus*	1717774	P02641	279	1	65	11	28	
SB0030	VWF	von Willebrand factor	*M. musculus*	115511022	NP_035838	2813	2	763, 1913	2	29	
SB0031	ACTB	β-Actin	*H. sapiens*	4501885	NP_001092	375	1	37	1 and/or 2	30	
SB0032	HBB	β-Globin	*H. sapiens*	4504349	NP_000509	147	1	9	1	31	
SB0033	MYO5A	Myosin 5A, heavy chain 12	*G. gallus*	46048699	NP_990631	1829	1	1140	1 or 2	32	
SB0034	NTS	Neurotensin	*B. taurus*	85719294	NP_776370	170	2	153, 158	1 and 2	26	
SB0035	GNG12	G-protein γ-12 subunit	*H. sapiens*	51036603	NP_061329	72	1	4	1	33	
SB0036	SNCA	α-Synuclein	*H. sapiens*	4507109	NP_000336	140	4	57, 73, 74, 83	1	34	
SB0037	VIM	Vimentin	*M. musculus*	31982755	NP_035831	466	10	18, 20, 32, 37, 40, 52, 63, 70, 91, 264	2	35	
SB0038	RHOA	RhoA, ras homolog gene family, member A	*H. sapiens*	10835049	NP_001655	193	1	180	1	36	
SB0039	PRKCB	Protein kinase Cβ	*R. norvegicus*	76880457	NP_036845	673	2	311, 320	1 and 2	37	*2
SB0040	HBA1	α1-Globin	*H. sapiens*	4504347	NP_000549	142	1	11	1	31	
SB0041	GNRH1	Gonadotropin-releasing hormone 1	*R. norvegicus*	6980968	NP_036899	92	2	28, 29	1 and 2	26	
SB0042	CASP9	Caspase 9	*H. sapiens*	14790124	NP_001220	416	2	115, 330	2	38	*3
SB0043	ITGB3	Integrin β3	*H. sapiens*	47078292	NP_000203	788	5	761, 767, 773, 780, 785	1	39	
SB0044	COPB1	β-COP, β-subunit of coatomer protein complex	*H. sapiens*	7705369	NP_057535	953	1	528	1	40	
SB0045	MBP	MBP, Myelin basic protein	*B. taurus*	126796	P02687	169	4	16, 68, 93, 96	2	41	
SB0046	MIP	Aquaporin-0	*H. sapiens*	6912506	NP_036196	263	5	237, 238, 252, 259, 260	2	42	
SB0047	(PRTMZ)	Protamine-Z	*C. harengus*	59800149	P69010	31	2	15, 23	unclear	27	
SB0048	ANXA1	Annexin I	*H. sapiens*	4502101	NP_000691	346	1	26	1	43	
SB0049	PDYN	β-Neoendorphin-dynorphin	*M. musculus*	148747390	NP_061351	248	2	207, 214	1 and 2	26	
SB0050	EGFR	EGFR, ErbB-1, Epidermal growth factor receptor	*H. sapiens*	29725609	NP_005219	1210	7	683, 733, 1030, 1059, 1086, 1151, 1185	2	44	
SB0051	HTT	Huntingtin	*H. sapiens*	90903231	NP_002102	3144	1	536	2	45	
SB0052	SPTB	β-I-Spectrin, isoform 1/b	*H. sapiens*	67782319	NP_000338	2137	1	2061	1	5	*4
SB0053	TP53	p53	*H. sapiens*	120407068	NP_000537	393	1	25	unclear	46	
SB0054	GCG	Glucagon	*R. norvegicus*	6978882	NP_036839	180	5	67, 69, 74, 77, 79	1 and 2	26	
SB0055	PRKCA	Protein kinase Cα	*R. norvegicus*	109492115	XP_001081588	672	3	309, 316, 324	1 and 2	37	*5
SB0056	ATP2B2	Plasma membrane calcium ATPase 2	*H. sapiens*	48255951	NP_001001331	1243	2	1124, 1135	1	47	
SB0057	MAP2	MAP2c	*R. norvegicus*	56623	CAA35667	467	1	99	unclear	48	
SB0058	(PRTMZ)	Protamine-Z	*C. pallasii*	59800150	P69011	31	2	15, 23	unclear	27	
SB0059	TH	Tyrosine hydroxylase	*B. taurus*	27806937	NP_776309	491	2	22, 30	11	49	
SB0060	INS	Insulin	*B. taurus*	27806865	NP_776351	105	5	29, 32, 37, 40, 50	11	50	
SB0061	SPTAN1	α-II-Spectrin, α-fodrin, non-erythrocytic	*H. sapiens*	4507191	NP_003118	2472	1	1176	1	51	
SB0062	ITGB7	Integrin β7	*H. sapiens*	4504777	NP_000880	798	10	760, 765, 766, 769, 770, 773, 774, 778, 784, 785	2	2	
SB0063	ITGB1	Integrin β1 isoform 1A	*H. sapiens*	19743813	NP_002202	798	5	771, 777, 778, 789, 791	2	2	*6
SB0064	FOS	c-fos	*R. norvegicus*	63147420	NP_071533	380	1	90	2	52	
SB0065	PARP1	ADP-ribosyltransferase (NAD+) poly(ADP-ribose) polymerase	*B. taurus*	27807449	NP_777176	1016	4	384, 480, 502, 658	1	53	
SB0066	TNNT2	Troponin T2, cardiac, TnTc	*M. musculus*	6755843	NP_035749	288	1	71	1	54	
SB0067	GAP43	GAP-43, Growth associated protein 43	*R. norvegicus*	8393415	NP_058891	226	2	4, 40	2	55	
SB0068	CRYBB3	Crystallin, βB3	*B. taurus*	27806955	NP_776952	211	2	5, 10	2	56	
SB0069	CRYBA1	Crystallin, βA1/A3	*B. taurus*	27806255	NP_776948	215	2	11, 17	2	56	
SB0070	IGFBP4	IGFBP4, Insulin-like growth factor binding protein 4	*H. sapiens*	62243290	NP_001543	258	4	23, 107, 143, 159	1	22	
SB0071	ACTA1	α-Actin-1	*H. sapiens*	4501881	NP_001091	377	1	39	1 and/or 2	30	
SB0072	GRIN2A	Glutamate receptor, ionotropic, NMDA 2A	*R. norvegicus*	140971205	NP_036705	1464	2	1278, 1329	1	57	
SB0073	DMD	Dystrophin	*H. sapiens*	5032283	NP_003997	3685	2	690, 1992	2	58	
SB0074	CAPN3	CAPN3, p94, calpain-3	*O. cuniculus*	126723116	NP_001075528	709	3	274, 296, 322	3	59	
SB0075	BAK1	BAK, BCL2-antagonist/killer 1	*H. sapiens*	4502363	NP_001179	211	1	15	1 and 2	60	*7
SB0076	CDK5R1	p35, cyclin-dependent kinase 5, regulatory subunit 1	*H. sapiens*	4502737	NP_003876	307	1	98	2	61	
SB0077	LOC618824	Histone H2A type 1	*B. taurus*	76631269	XP_876240	130	10	20, 24, 33, 34, 57, 82, 92, 110, 114, 123	unclear	12	
SB0078	SPTB	β-I-Spectrin, isoform 2/a	*H. sapiens*	67782321	NP_001020029	2328	1	2058	1	5	*4
SB0079	ITGB1	Integrin β1 isoform 1D	*H. sapiens*	19743819	NP_391988	801	11	767, 771, 772, 777, 778, 783, 788, 789, 790, 791, 795	2	2	*6
SB0080	CRYBA4	Crystallin, βA4	*R. norvegicus*	13928956	NP_113877	196	1	4	2	62	
SB0081	TLN1	Talin 1	*G. gallus*	6755809	NP_035732	2541	1	433	1	63	
SB0082	CRYBB2	Crystallin, βB2	*B. taurus*	27807379	NP_777232	205	1	8	2	56	
SB0083	CDKN2D	p19INK4d, Cyclin-dependent kinase inhibitor 2D	*H. sapiens*	4502753	NP_001791	166	6	25, 29, 47, 64, 113, 127	1	64	
SB0084	BID	BID, BH3 interacting domain death agonist	*H. sapiens*	4557361	NP_001187	195	1	70	2	65	
SB0085	(PRTMY)	Protamine-YII	*C. pallasii*	59800157	P69009	30	2	14, 22	unclear	27	
SB0086	JUN	c-jun	*R. norvegicus*	11177864	NP_068607	334	4	42, 62, 90, 164	2	52	

Table S1. List of reported substrates and their calpain cleavage sites.

SB No.	Gene product	Description	Species*12	gi#	Reference sequence	length	# of sites	Sites	Calpain	Ref.	Note
SB0087	LOC619141	Histone H3.2	*B. taurus*	119889445	XP_876553	136	8	10, 23, 28, 33, 34, 49, 119, 129	unclear	12	
SB0088	CRYBB1	Crystallin, βB1	*R. norvegicus*	6978711	NP_037068	249	1	49	2	62	
SB0089	PRKCG	Protein kinase Cγ	*R. norvegicus*	6981400	NP_036760	697	2	321, 338	1 and 2	37	
SB0090	FLNA	Filamin A	*H. sapiens*	116063573	NP_001447	2639	1	1761	1 or 2	66	
SB0091	PRDX2	Thioredoxin peroxidase 1	*H. sapiens*	32189392	NP_005800	198	5	181, 182, 183, 193, 194	1	67	
SB0092	FLNC	Filamin C	*H. sapiens*	116805322	NP_001449	2725	1	2626	1	68	
SB0093	AMPH	Amphiphysin I	*H. sapiens*	1351924	NP_001626	695	9	333, 377, 392, 454, 478, 527, 531, 593, 609	2	69	
SB0094	CRYBB1	Crystallin, βB1	*B. taurus*	290	CAA25951	253	2	12, 15	2	56	
SB0095	CRYBB3	Crystallin, βB3	*R. norvegicus*	13928958	NP_113878	211	3	5, 15, 17	2	62	
SB0096	CRYBA1	Crystallin, βA1/A3	*R. norvegicus*	109491386	XP_001080813	215	1	11	2	62	
SB0097	IGFBP2	Insulin-like growth factor binding protein 2	*H. sapiens*	55925576	NP_000588	328	1	202	2	70	
SB0098	IGFBP3	Insulin-like growth factor binding protein 3	*H. sapiens*	62243248	NP_001013416	297	1	175	2	70	
SB0099	MYOC	myocilin	*H. sapiens*	4557779	NP_000252	504	1	226	1 and 2	71	
SB0100	TTN	connectin/titin	*H. sapiens*	110349719	NP_596869	33423	3	8563, 8651, 8652	1	72	*8
SB0101	CTBP1	C-terminal binding protein 1	*H. sapiens*	4557497	NP_001319	440	2	375, 387	1	73	*9
SB0102	ANKRD2	MARP2, Ankrd2	*H. sapiens*	39812133	NP_065082	360	1	103	1	72	
SB0103	AP2B1	β2-Adaptin, AP2B1	*R. norvegicus*	18034787	NP_542150	951	1	691	1 and 2	74	
SB0104	CAPN2	CAPN2, m-calpain catalytic subunit, mCL	*O. cuniculus*	307611990	NP_001182651	709	1	19	2	75	
SB0105	EZR	Ezrin	*R. norvegicus*	52138521	NP_062230	586	1	467	1	76	
SB0106	AGC1	Aggrecan	*B. taurus*	27806761	NP_776406	2327	3	365, 474, 719	2	77	
SB0107	AGC1	Aggrecan	*H. sapiens*	129886	P16112	2415	1	709	2	77	
SB0108	AGC1	Aggrecan	*R. norvegicus*	11990616	NP_071526	2162	1	715	2	77	
SB0109	PDLIM1	PDLIM1	*H. sapiens*	13994151	NP_066272	329	1	271	3	78	
SB0110	CASP7	Caspase-7	*H. sapiens*	4502581	NP_001218	303	1	36	1	79	
SB0111	TOP1	DNA topoisomerase I	*H. sapiens*	11225260	NP_003277	765	2	158, 183	2	80	
SB0112	SMN1	Survival motor neuron protein 1, SMN1	*H. sapiens*	4507091	NP_000335	294	2	192, 193	1	81	
SB0113	PXN	Paxillin	*G. gallus*	45384484	NP_990315	559	1	96	1 or 2	82	
SB0114	FLG2	Filaggrin	*H. sapiens*	62122917	NP_001014364	2391	4	1713, 1741, 1771, 1788	1	83	
SB0115	SELK	Selenoprotein K	*M. musculus*	111119001	NP_064363	94	1	81	1	84	
SB0116	SLC17A6	Vesicular glutamate transporter 2	*R. norvegicus*	16758166	NP_445879	582	2	533, 541	1	85	
SB0117	ITPR1	Type 1 inositol 1,4,5-triphosphate receptor, InsP3R1	*R. norvegicus*	55925610	NP_001007236	2748	1	1917	1	86	
SB0118	RAD21	Rad21	*H. sapiens*	5453994	NP_006256	631	1	192	1	87	
SB0119	CASP3	Caspase-3	*H. sapiens*	14790115	NP_116786	277	1	7	1	88	
SB0120	CASP9	Caspase-9	*H. sapiens*	14790124	NP_001220	416	3	115, 120, 143	1	88	*3
SB0121	PPP3CA	Calcineurin	*R. norvegicus*	8394030	NP_058737	521	4	421, 422, 423, 425	2	89	
SB0122	PTK2	Focal adhesion kinase, FAK	*M. musculus*	194353972	NP_032008	1052	1	745	2	90	
SB0123	NCS1	Neuronal calcium sensor-1, NCS-1	*R. norvegicus*	13242261	NP_077342	190	1	36	1	91	
SB0124	DBNL	Actin-binding protein-1, Drebrin-like protein	*M. musculus*	226423871	NP_001139780	436	2	283, 290	2	92	
SB0125	TRPC6	Transient receptor potential canonical 6, TRPC6	*M. musculus*	160333370	NP_038866	930	1	16	1	93	
SB0126	ZNF175	OTK18	*H. sapiens*	6005970	NP_009078	711	1	359	1	94	
SB0127	MBP	Myelin basic protein	*H. sapiens*	68509940	NP_001020272	304	11	152, 157, 161, 183, 193, 204, 228, 231, 244, 279, 290	2	95	
SB0128	MEF2D	MEF2D	*H. sapiens*	5174545	NP_005911	521	1	78	2	96	
SB0129	CAPN2	CAPN2, m-calpain catalytic subunit, mCL	*R. norvegicus*	8393038	NP_058812	700	45	1, 2, 3, 7, 9, 10, 11, 12, 13, 14, 23, 60, 82, 116, 201, 207, 209, 247, 249, 255, 267, 359, 360, 381, 383, 387, 390, 392, 407, 409, 410, 423, 449, 461, 462, 468, 469, 502, 509, 513, 517, 524, 525, 611, 618	2	97	
SB0130	MAPT	tau	*R. norvegicus*	149054483	EDM06300	432	3	120, 209, 380	2	98	
SB0131	SLC32A1	Vesicular GABA transporter	*R. norvegicus*	13929106	NP_113970	525	2	51, 59	1	99	
SB0132	DUT	dUTPase	*H. sapiens*	4503423	NP_001939	164	3	4, 7, 31	2	100	
SB0133	CAPNS1	CAPNS1, 30K, Calpain regulatory subunit	*O. cuniculus*	126723197	NP_001075733	266	1	89	2	75	
SB0134	GSK3B	Glycogen synthase kinase-3β (GSK-3β)	*R. norvegicus*	14091770	NP_114469	420	2	38, 384	2	101	
SB0135	RXRA	Retinoid X receptor α	*H. sapiens*	4506755	NP_002948	462	2	90, 118	2	102	
SB0136	CTNNB1	β-Catenin	*M. musculus*	260166642	NP_001159374	781	4	28, 29, 30, 95	1	103	
SB0137	IGBP1	Protein Phosphatase 2A (PP2A) Regulatory Subunit α4, immunoglobulin-binding protein 1	*H. sapiens*	4557663	NP_001542	339	1	255	?	104	
SB0138	BCL2A1	Antiapoptotic Bfl-1 (BCL2A1)	*H. sapiens*	168480072	NP_001108207	163	3	21, 22, 71	1	105	
SB0139	CPEB3	Cytoplasmic polyadenylation element-binding protein 3 (CPEB3)	*H. sapiens*	296011035	NP_001171608	684	1	427	2	106	
SB0140	TARDBP	TAR DNA-binding protein 43, TDP-43	*H. sapiens*	6678271	NP_031401	414	10	229, 243, 286, 295, 297, 302, 303, 304, 324, 346	1	107	
SB0141	ATX3	Ataxin-3, Machado-Joseph disease (spinocerebellar ataxia 3)	*H. sapiens*	833928	AAB33571	360	6	55, 62, 187, 208, 256, 317	2	108	
SB0142	ACTN4	α-actinin-4	*H. sapiens*	12025678	NP_004915	911	2	13, 283	2	109	
SB0143	ACTN1	α-actinin-1	*G. gallus*	4501891	NP_989458	892	2	(244)*, 247	1 and 2	110	*10
SB0144	PTPN5	Striatal-enriched protein tyrosine phosphatase (STEP)	*H. sapiens*	503774431	NP_001265167	541	1	224	1	111	*11
SB0145	TNNT3	Troponin T3, fast skeletal muscle, TnTf	*S. scrofa*	46389775	BAD15378	271	3	22, 38, 52	2	112	
SB0146	JPH2	Junctophilin-2	*M. musculus*	10947010	NP_067541	696	1	201	in vivo	113	
SB0147	TTN	connectin/titin	*H. sapiens*	110349719	NP_596869	33423	4	8563, 33017, 33305, 33354	3	73, 114	*8
SB0148	CTBP1	C-terminal binding protein 1	*H. sapiens*	4557497	NP_001319	440	1	409	3	115	*9
SB0149	CAST	Calpastatin	*H. sapiens*	298919189	NP_001177371	778	7	195, 203, 209, 210, 228, 253, 256	3	73	
SB0150	CAPN3	CAPN3, p94, calpain-3	*H. sapiens*	4557405	NP_000061	821	4	14, 30, 33, 412	3	73, 116	

*1 This chicken sequence is incorrectly referred to as CAPN1 in the NCBI database, but it has been evolutionarily shown to be the homolog of human CAPN11 (117).

*2 CAPN1 cuts after G311 and K320, while CAPN2 cuts only after G311.

*3 SB0042 and SB0120 are the same protein (and site 115 is identical), but the calpains used for proteolysis differed.

*4 SB0052 and SB0078 are different isoforms of the same protein, but different cleavages are reported.

*5 CAPN1 cuts after K316 and R324, while CAPN2 cuts only after K309.

*6 SB0063 and SB0079 are different isoforms of the same protein, but different cleavages are reported.

*7 The CysPc domain of CAPN1 was used for cleavage.

*8 SB0100 and SB0147 are the same protein, but the calpains used for proteolysis differed.

*9 SB0101 and SB0148 are the same protein, but the calpains used for proteolysis and their sites differed.

*10 In this report, the cleavage experiments used chicken ACTIN1, but the sequence numbers reported (243 and 246) correspond to those of the human protein (NP_001123476, 98% identical to chicken NP_989458; His is inserted between Asp2 and His3 of the human sequence). Here, the correct chicken sequence numbers are shown (244 and 247). Cleavage at 244 was only shown for calpain-2, and was described as being very minor.

*11 The species for STEP was not given; judging from the sequence, it is probably human, and the human sequence is referred to here.

*12 Abbreviations for species: *B. taurus*, *Bos taurus* (bovine); *C. harengus*, *Clupea harengus* (Atlantic herring); *C. pallasii*, *Clupea pallasii* (Pacific herring); *G. gallus*, *Gallus gallus* (chicken); *H. sapiens*, *Homo sapiens* (human); *M. musculus*, *Mus musculus* (mouse); *O. aries*, *Ovis aries* (ovine); *O. cuniculus*, *Oryctolagus cuniculus* (rabbit); *R. norvegicus*, *Rattus norvegicus* (rat); *S. scrofa*, *Sus scrofa* (porcine).

1. SB0001: Brown, N., and Crawford, C. (1993) Structural modifications associated with the change in Ca2+ sensitivity on activation of m-calpain. *FEBS Lett.* **322**, 65-68

2. SB0002, SB0062, SB0063, SB0079: Pfaff, M., Du, X., and Ginsberg, M. H. (1999) Calpain cleavage of integrin beta cytoplasmic domains. *FEBS Lett.* **460**, 17-22

3. SB0003: Schey, K. L., Fowler, J. G., Shearer, T. R., and David, L. (1999) Modifications to rat lens major intrinsic protein in selenite-induced cataract. *Invest. Ophthalmol. Vis. Sci.* **40**, 657-667

4. SB0004: Kakkar, R., Raju, R. V., and Sharma, R. K. (1998) In vitro generation of an active calmodulin-independent phosphodiesterase from brain calmodulin-dependent phosphodiesterase (PDE1A2) by m-calpain. *Arch. Biochem. Biophys.* **358**, 320-328

5. SB0005, SB0052, SB0078: Lofvenberg, L., and Backman, L. (1999) Calpain-induced proteolysis of beta-spectrins. *FEBS Lett.* **443**, 89-92

6. SB0006: McGinnis, K. M., Whitton, M. M., Gnegy, M. E., and Wang, K. K. (1998) Calcium/calmodulin-dependent protein kinase IV is cleaved by caspase-3 and calpain in SH-SY5Y human neuroblastoma cells undergoing apoptosis. *J. Biol. Chem.* **273**, 19993-20000

7. SB0007: Xu, W., Wong, T. P., Chery, N., Gaertner, T., Wang, Y. T., and Baudry, M. (2007) Calpain-mediated mGluR1alpha truncation: a key step in excitotoxicity. *Neuron* **53**, 399-412

8. SB0008: Park, D., Jhon, D. Y., Lee, C. W., Ryu, S. H., and Rhee, S. G. (1993) Removal of the carboxyl-terminal region of phospholipase C-beta 1 by calpain abolishes activation by G alpha q. *J. Biol. Chem.* **268**, 3710-3714

9. SB0009: Kimura, Y., Koga, H., Araki, N., Mugita, N., Fujita, N., Takeshima, H., Nishi, T., Yamashima, T., Saido, T. C., Yamasaki, T., Moritake, K., Saya, H., and Nakao, M. (1998) The involvement of calpain-dependent proteolysis of the tumor suppressor NF2 (merlin) in schwannomas and meningiomas. *Nat. Med.* **4**, 915-922

10. SB0010: Shaw, G., Yang, C., Zhang, L., Cook, P., Pike, B., and Hill, W. D. (2004) Characterization of the bovine neurofilament NF-M protein and cDNA sequence, and identification of in vitro and in vivo calpain cleavage sites. *Biochem. Biophys. Res. Commun.* **325**, 619-625

11. SB0011: Schaecher, K., Goust, J. M., and Banik, N. L. (2004) The effects of calpain inhibition on IkB alpha degradation after activation of PBMCs: identification of the calpain cleavage sites. *Neurochem. Res.* **29**, 1443-1451

12. SB0012, SB077, SB0087: Sakai, K., Akanuma, H., Imahori, K., and Kawashima, S. (1987) A unique specificity of a calcium activated neutral protease indicated in histone hydrolysis. *J. Biochem.* **101**, 911-918

13. SB0013: Baliova, M., Betz, H., and Jursky, F. (2004) Calpain-mediated proteolytic cleavage of the neuronal glycine transporter, GlyT2. *J. Neurochem.* **88**, 227-232

14. SB0014: Yousefi, S., Perozzo, R., Schmid, I., Ziemiecki, A., Schaffner, T., Scapozza, L., Brunner, T., and Simon, H. U. (2006) Calpain-mediated cleavage of Atg5 switches autophagy to apoptosis. *Nature cell biology* **8**, 1124-1132

15. SB0015: Wu, Y., Aghdasi, B., Dou, S. J., Zhang, J. Z., Liu, S. Q., and Hamilton, S. L. (1997) Functional interactions between cytoplasmic domains of the skeletal muscle Ca2+ release channel. *J. Biol. Chem.* **272**, 25051-25061

16. SB0016: Lin, J. S., Fitzgerald, S., Dong, Y., Knight, C., Donaldson, P., and Kistler, J. (1997) Processing of the gap junction protein connexin50 in the ocular lens is accomplished by calpain. *Eur. J. Cell Biol.* **73**, 141-149

17. SB0017, SB0021: Crawford, C., Willis, A. C., and Gagnon, J. (1987) The effects of autolysis on the structure of chicken calpain II. *Biochem. J.* **248**, 579-588

18. SB0018: Pete, M. J., Liao, C. X., Bartleson, C., and Graves, D. J. (1999) A recombinant form of the catalytic subunit of phosphorylase kinase that is soluble, monomeric, and includes key C-terminal residues. *Arch. Biochem. Biophys.* **367**, 104-114

19. SB0019: Kobayashi, Y., Yamamoto, K., Saido, T., Kawasaki, H., Oppenheim, J. J., and Matsushima, K. (1990) Identification of calcium-activated neutral protease as a processing enzyme of human interleukin 1 alpha. *Proc. Natl. Acad. Sci. U. S. A.* **87**, 5548-5552

20. SB0020: Zimmerman, U. J., and Schlaepfer, W. W. (1991) Two-stage autolysis of the catalytic subunit initiates activation of calpain I. *Biochim. Biophys. Acta* **1078**, 192-198

21. SB0022: Perrin, B. J., Amann, K. J., and Huttenlocher, A. (2006) Proteolysis of cortactin by calpain regulates membrane protrusion during cell migration. *Mol. Biol. Cell* **17**, 239-250

22. SB0023, SB0070: Ghosh, M., Shanker, S., Siwanowicz, I., Mann, K., Machleidt, W., and Holak, T. A. (2005) Proteolysis of insulin-like growth factor binding proteins (IGFBPs) by calpain. *Biol. Chem.* **386**, 85-93

23. SB0024: Ort, T., Voronov, S., Guo, J., Zawalich, K., Froehner, S. C., Zawalich, W., and Solimena, M. (2001) Dephosphorylation of b2-syntrophin and Ca^{2+}/m-calpain-mediated cleavage of ICA512 upon stimulation of insulin secretion. *EMBO J.* **20**, 4013-4023

24. SB0025: Azarian, S. M., King, A. J., Hallett, M. A., and Williams, D. S. (1995) Selective proteolysis of arrestin by calpain. Molecular characteristics and its effect on rhodopsin dephosphorylation. *J. Biol. Chem.* **270**, 24375-24384

25. SB0026: Toyota, H., Yanase, N., Yoshimoto, T., Moriyama, M., Sudo, T., and Mizuguchi, J. (2003) Calpain-induced Bax-cleavage product is a more potent inducer of apoptotic cell death than wild-type Bax. *Cancer Lett.* **189**, 221-230

26. SB0027, SB0034, SB0041, SB0049, SB0054: Sasaki, T., Kikuchi, T., Yumoto, N., Yoshimura, N., and Murachi, T. (1984) Comparative specificity and kinetic studies on porcine calpain I and calpain II with naturally occurring

peptides and synthetic fluorogenic substrates. *J. Biol. Chem.* **259**, 12489-12494

27. SB0028, SB0047, SB0058, SB0085: Hayashi, M., Inomata, M., Nakamura, M., Imahori, K., and Kawashima, S. (1985) Hydrolysis of protamine by calcium-activated neutral protease (CANP). *J. Biochem.* **97**, 1363-1370

28. SB0029: Ohtsuki, I., Shiraishi, F., Suenaga, N., Miyata, T., and Tanokura, M. (1984) A 26K fragment of troponin T from rabbit skeletal muscle. *J. Biochem.* **95**, 1337-1342

29. SB0030: Nozaki, H. (1987) Amino acid analysis of human von Willebrand factor fragments cleaved by porcine calpain II. *Tokai J. Exp. Clin. Med.* **12**, 223-227

30. SB0031, SB0071: Villa, P. G., Henzel, W. J., Sensenbrenner, M., Henderson, C. E., and Pettmann, B. (1998) Calpain inhibitors, but not caspase inhibitors, prevent actin proteolysis and DNA fragmentation during apoptosis. *J. Cell Sci.* **111**, 713-722

31. SB0032, SB0040: Melloni, E., Salamino, F., Sparatore, B., Michetti, M., and Pontremoli, S. (1984) Characterization of the single peptide generated from the amino-terminus end of alpha- and beta-hemoglobin chains by the Ca2+-dependent neutral proteinase. *Biochim. Biophys. Acta* **788**, 11-16

32. SB0033: Nascimento, A. A., Cheney, R. E., Tauhata, S. B., Larson, R. E., and Mooseker, M. S. (1996) Enzymatic characterization and functional domain mapping of brain myosin-V. *J. Biol. Chem.* **271**, 17561-17569

33. SB0035: Morishita, R., Nakayama, H., Isobe, T., Matsuda, T., Hashimoto, Y., Okano, T., Fukada, Y., Mizuno, K., Ohno, S., Kozawa, O., and et al. (1995) Primary structure of a gamma subunit of G protein, gamma 12, and its phosphorylation by protein kinase C. *J. Biol. Chem.* **270**, 29469-29475

34. SB0036: Mishizen-Eberz, A. J., Norris, E. H., Giasson, B. I., Hodara, R., Ischiropoulos, H., Lee, V. M., Trojanowski, J. Q., and Lynch, D. R. (2005) Cleavage of alpha-synuclein by calpain: potential role in degradation of fibrillized and nitrated species of alpha-synuclein. *Biochemistry.* **44**, 7818-7829

35. SB0037: Fischer, S., Vandekerckhove, J., Ampe, C., Traub, P., and Weber, K. (1986) Protein-chemical identification of the major cleavage sites of the Ca2+ proteinase on murine vimentin, the mesenchymal intermediate filament protein. *Biol. Chem. Hoppe. Seyler* **367**, 1147-1152

36. SB0038: Kulkarni, S., Goll, D. E., and Fox, J. E. (2002) Calpain cleaves RhoA generating a dominant-negative form that inhibits integrin-induced actin filament assembly and cell spreading. *J. Biol. Chem.* **277**, 24435-24441

37. SB0039, SB0055, SB0089: Kishimoto, A., Mikawa, K., Hashimoto, K., Yasuda, I., Tanaka, S., Tominaga, M., Kuroda, T., and Nishizuka, Y. (1989) Limited proteolysis of protein kinase C subspecies by calcium-dependent neutral protease (calpain). *J. Biol. Chem.* **264**, 4088-4092

38. SB0042: Chua, B. T., Guo, K., and Li, P. (2000) Direct cleavage by the calcium-activated protease calpain can lead to inactivation of caspases. *J. Biol. Chem.* **275**, 5131-5135

39. SB0043: Du, X., Saido, T. C., Tsubuki, S., Indig, F. E., Williams, M. J., and Ginsberg, M. H. (1995) Calpain cleavage of the cytoplasmic domain of the integrin beta 3 subunit. *J. Biol. Chem.* **270**, 26146-26151

40. SB0044: Hata, S., Koyama, S., Kawahara, H., Doi, N., Maeda, T., Toyama-Sorimachi, N., Abe, K., Suzuki, K., and Sorimachi, H. (2006) Stomach-specific calpain, nCL-2, localizes in mucus cells and proteolyzes the β-subunit of coatomer complex, β-COP. *J. Biol. Chem.* **281**, 11214-11224

41. SB0045: Tsubata, T., and Takahashi, K. (1989) Limited proteolysis of bovine myelin basic protein by calcium-dependent proteinase from bovine spinal cord. *J. Biochem.* **105**, 23-28

42. SB0046: Ma, H., Azuma, M., and Shearer, T. R. (2005) Degradation of human aquaporin 0 by m-calpain. *FEBS Lett.* **579**, 6745-6748

43. SB0048: Ando, Y., Imamura, S., Hong, Y. M., Owada, M. K., Kakunaga, T., and Kannagi, R. (1989) Enhancement of calcium sensitivity of lipocortin I in phospholipid binding induced by limited proteolysis and phosphorylation at the amino terminus as analyzed by phospholipid affinity column chromatography. *J. Biol. Chem.* **264**, 6948-6955

44. SB0050: Gregoriou, M., Willis, A. C., Pearson, M. A., and Crawford, C. (1994) The calpain cleavage sites in the epidermal growth factor receptor kinase domain. *Eur. J. Biochem.* **223**, 455-464

45. SB0051: Gafni, J., Hermel, E., Young, J. E., Wellington, C. L., Hayden, M. R., and Ellerby, L. M. (2004) Inhibition of calpain cleavage of huntingtin reduces toxicity: accumulation of calpain/caspase fragments in the nucleus. *J. Biol. Chem.* **279**, 20211-20220

46. SB0053: Pariat, M., Carillo, S., Molinari, M., Salvat, C., Debussche, L., Bracco, L., Milner, J., and Piechaczyk, M. (1997) Proteolysis by calpains: a possible contribution to degradation of p53. *Mol. Cell. Biol.* **17**, 2806-2815

47. SB0056: James, P., Vorherr, T., Krebs, J., Morelli, A., Castello, G., McCormick, D. J., Penniston, J. T., De Flora, A., and Carafoli, E. (1989) Modulation of erythrocyte Ca2+-ATPase by selective calpain cleavage of the calmodulin-binding domain. *J. Biol. Chem.* **264**, 8289-8296

48. SB0057: Tompa, P., Buzder-Lantos, P., Tantos, A., Farkas, A., Szilagyi, A., Banoczi, Z., Hudecz, F., and Friedrich, P. (2004) On the sequential determinants of calpain cleavage. *J. Biol. Chem.* **279**, 20775-20785

49. SB0059: Kiuchi, K., Kiuchi, K., Titani, K., Fujita, K., Suzuki, K., and Nagatsu, T. (1991) Limited proteolysis of tyrosine hydroxylase by Ca^{2+}-activated neutral protease (calpain). *Biochemistry.* **30**, 10416-10419

50. SB0060: Ishiura, S., Sugita, H., Suzuki, K., and Imahori, K. (1979) Studies of a calcium-activated neutral protease from chicken skeletal muscle. II. Substrate specificity. *J. Biochem.* **86**, 579-581

51. SB0061: Harris, A. S., Croall, D. E., and Morrow, J. S. (1988) The calmodulin-binding site in alpha-fodrin is near the calcium-dependent protease-I cleavage site. *J. Biol. Chem.* **263**, 15754-15761

52. SB0064, SB0086: Pariat, M., Salvat, C., Bebien, M., Brockly, F., Altieri, E., Carillo, S., Jariel-Encontre, I., and Piechaczyk, M. (2000) The sensitivity of c-Jun and c-Fos proteins to calpains depends on conformational determinants of the monomers and not on formation of dimers. *Biochem. J.* **345 Pt 1**, 129-138

53. SB0065: Buki, K. G., Bauer, P. I., and Kun, E. (1997) Isolation and identification of a proteinase from calf thymus that cleaves poly(ADP-ribose) polymerase and histone H1 (bovine CAPN1). *Biochim. Biophys. Acta* **1338**, 100-106

54. SB0066: Zhang, Z., Biesiadecki, B. J., and Jin, J. P. (2006) Selective deletion of the NH2-terminal variable region of cardiac troponin T in ischemia reperfusion by myofibril-associated mu-calpain cleavage. *Biochemistry.* **45**, 11681-11694

55. SB0067: Zakharov, V. V., and Mosevitsky, M. I. (2001) Site-specific calcium-dependent proteolysis of neuronal protein GAP-43. *Neurosci. Res.* **39**, 447-453

56. SB0068, SB0069, SB0082, SB0094: Shih, M., Lampi, K. J., Shearer, T. R., and David, L. L. (1998) Cleavage of beta crystallins during maturation of bovine lens. *Mol. Vis.* **4**, 4

57. SB0072: Guttmann, R. P., Baker, D. L., Seifert, K. M., Cohen, A. S., Coulter, D. A., and Lynch, D. R. (2001) Specific proteolysis of the NR2 subunit at multiple sites by calpain. *J. Neurochem.* **78**, 1083-1093

58. SB0073: Yoshida, M., Suzuki, A., Shimizu, T., and Ozawa, E. (1992) Proteinase-sensitive sites on isolated rabbit dystrophin. *J. Biochem.* **112**, 433-439

59. SB0074: Kinbara, K., Ishiura, S., Tomioka, S., Sorimachi, H., Jeong, S. Y., Amano, S., Kawasaki, H., Kolmerer, B., Kimura, S., Labeit, S., and Suzuki, K. (1998) Purification of native p94, a muscle-specific calpain, and characterization of its autolysis. *Biochem. J.* **335**, 589-596

60. SB0075: Moldoveanu, T., Liu, Q., Tocilj, A., Watson, M., Shore, G., and Gehring, K. (2006) The X-ray structure of a BAK homodimer reveals an inhibitory zinc binding site. *Mol. Cell* **24**, 677-688

61. SB0076: Lee, M. S., Kwon, Y. T., Li, M., Peng, J., Friedlander, R. M., and Tsai, L. H. (2000) Neurotoxicity induces cleavage of p35 to p25 by calpain. *Nature* **405**, 360-364

62. SB0080, SB0088, SB0095, SB0096: David, L. L., and Shearer, T. R. (1993) Beta-crystallins insolubilized by calpain II in vitro contain cleavage sites similar to beta-crystallins insolubilized during cataract. *FEBS Lett.* **324**, 265-270

63. SB0081: Rees, D. J., Ades, S. E., Singer, S. J., and Hynes, R. O. (1990) Sequence and domain structure of talin. *Nature* **347**, 685-689

64. SB0083: Joy, J., Nalabothula, N., Ghosh, M., Popp, O., Jochum, M., Machleidt, W., Gil-Parrado, S., and Holak, T. A. (2006) Identification of calpain cleavage sites in the G1 cyclin-dependent kinase inhibitor p19(INK4d). *Biol. Chem.* **387**, 329-335

65. SB0084: Mandic, A., Viktorsson, K., Strandberg, L., Heiden, T., Hansson, J., Linder, S., and Shoshan, M. C. (2002) Calpain-mediated Bid cleavage and calpain-independent Bak modulation: two separate pathways in cisplatin-induced apoptosis. *Mol. Cell. Biol.* **22**, 3003-3013

66. SB0090: Gorlin, J. B., Yamin, R., Egan, S., Stewart, M., Stossel, T. P., Kwiatkowski, D. J., and Hartwig, J. H. (1990) Human endothelial actin-binding protein (ABP-280, nonmuscle filamin): a molecular leaf spring. *J. Cell Biol.* **111**, 1089-1105

67. SB0091: Schroder, E., Willis, A. C., and Ponting, C. P. (1998) Porcine natural-killer-enhancing factor-B: oligomerisation and identification as a calpain substrate in vitro. *Biochim. Biophys. Acta* **1383**, 279-291

68. SB0092: Fabrice, F., Carole, J.-N., M., A., Dieter, F., and Yves, B. (2006) Calpain 1-gamma filamin interaction in muscle cells: a possible in situ regulation by PKC-alpha. *Int. J. Biochem. Cell Biol.* **38**, 404-413

69. SB0093: Wu, Y., Liang, S., Oda, Y., Ohmori, I., Nishiki, T., Takei, K., Matsui, H., and Tomizawa, K. (2007) Truncations of amphiphysin I by calpain inhibit vesicle endocytosis during neural hyperexcitation. *EMBO J.* **26**, 2981-2990

70. SB0097, SB0098: Berg, U., Bang, P., and Carlsson-Skwirut, C. (2007) Calpain proteolysis of insulin-like growth factor binding protein (IGFBP) -2 and -3, but not of IGFBP-1. *Biol. Chem.* **388**, 859-863

71. SB0099: Sanchez-Sanchez, F., Martinez-Redondo, F., Aroca-Aguilar, J. D., Coca-Prados, M., and Escribano, J. (2007) Characterization of the intracellular proteolytic cleavage of myocilin and identification of calpain II as a myocilin-processing protease. *J. Biol. Chem.* **282**, 27810-27824

72. SB0100, SB0102: Hayashi, C., Ono, Y. e. c. f. a., Doi, N., Kitamura, F., Tagami, M., Mineki, R., Arai, T., Taguchi, H., Yanagida, M., Hirner, S., Labeit, D., Labeit, S., and Sorimachi, H. (2008) Multiple molecular interactions implicate the connectin/titin N2A region as a modulating scaffold for p94/calpain 3 activity in skeletal muscle. *J. Biol. Chem.* **283**, 14801-14814

73. SB0101, SB0147, SB0149, SB0150: Ono, Y., Kakinuma, K., Torii, F., Irie, A., Nakagawa, K., Labeit, S., Abe, K., Suzuki, K., and Sorimachi, H. (2004) Possible regulation of the conventional calpain system by skeletal muscle-specific calpain, p94/calpain 3. *J. Biol. Chem.* **279**, 2761-2771

74. SB0103: Rudinskiy, N., Grishchuk, Y., Vaslin, A., Puyal, J., Delacourte, A., Hirling, H., Clarke, P. G., and Luthi-Carter, R. (2009) Calpain hydrolysis of alpha- and beta2-adaptins decreases clathrin-dependent endocytosis and

may promote neurodegeneration. *J. Biol. Chem.* **284**, 12447-12458

75. SB0104, SB0133: Imajoh, S., Kawasaki, H., and Suzuki, K. (1986) Limited autolysis of calcium-activated neutral protease (CANP): reduction of the Ca^{2+}-requirement is due to the NH_2-terminal processing of the large subunit. *J. Biochem.* **100**, 633-642

76. SB0105: Wang, F., Xia, P., Wu, F., Wang, D., Wang, W., Ward, T., Liu, Y., Aikhionbare, F., Guo, Z., Powell, M., Liu, B., Bi, F., Shaw, A., Zhu, Z., Elmoselhi, A., Fan, D., Cover, T. L., Ding, X., and Yao, X. (2008) Helicobacter pylori VacA disrupts apical membrane-cytoskeletal interactions in gastric parietal cells. *J. Biol. Chem.* **283**, 26714-26725

77. SB0106, SB0107, SB0108: Oshita, H., Sandy, J. D., Suzuki, K., Akaike, A., Bai, Y., Sasaki, T., and Shimizu, K. (2004) Mature bovine articular cartilage contains abundant aggrecan that is C-terminally truncated at Ala719-Ala720, a site which is readily cleaved by m-calpain. *Biochem. J.* **382**, 253-259

78. SB0109: Bertipaglia, I., Bourg, N., Richard, I., Pahlman, A. K., Andersson, L., James, P., and Carafoli, E. (2009) A proteomic study of calpain-3 and its involvement in limb girdle muscular dystrophy type 2a. *Cell Calcium* **46**, 356-363

79. SB0110: Gafni, J., Cong, X., Chen, S. F., Gibson, B. W., and Ellerby, L. M. (2009) Calpain-1 cleaves and activates caspase-7. *J. Biol. Chem.* **284**, 25441-25449

80. SB0111: Chou, S. M., Huang, T. H., Chen, H. C., and Li, T. K. (2011) Calcium-induced cleavage of DNA topoisomerase I involves the cytoplasmic-nuclear shuttling of calpain 2. *Cell. Mol. Life Sci.* **68**, 2769-2784

81. SB0112: Fuentes, J. L., Strayer, M. S., and Matera, A. G. (2010) Molecular determinants of survival motor neuron (SMN) protein cleavage by the calcium-activated protease, calpain. *PLoS One* **5**, e15769

82. SB0113: Cortesio, C. L., Boateng, L. R., Piazza, T. M., Bennin, D. A., and Huttenlocher, A. (2011) Calpain-mediated proteolysis of paxillin negatively regulates focal adhesion dynamics and cell migration. *J. Biol. Chem.* **286**, 9998-10006

83. SB0114: Hsu, C. Y., Henry, J., Raymond, A. A., Mechin, M. C., Pendaries, V., Nassar, D., Hansmann, B., Balica, S., Burlet-Schiltz, O., Schmitt, A. M., Takahara, H., Paul, C., Serre, G., and Simon, M. (2011) Deimination of human filaggrin-2 promotes its proteolysis by calpain 1. *J. Biol. Chem.* **286**, 23222-23233

84. SB0115: Huang, Z., Hoffmann, F. W., Norton, R. L., Hashimoto, A. C., and Hoffmann, P. R. (2011) Selenoprotein K is a novel target of m-calpain, and cleavage is regulated by Toll-like receptor-induced calpastatin in macrophages. *J. Biol. Chem.* **286**, 34830-34838

85. SB0116: Lobo, A. C., Gomes, J. R., Catarino, T., Mele, M., Fernandez, P., Inacio, A. R., Bahr, B. A., Santos, A. E., Wieloch, T., Carvalho, A. L., and Duarte, C. B. (2011) Cleavage of the vesicular glutamate transporters under excitotoxic conditions. *Neurobiol. Dis.* **44**, 292-303

86. SB0117: Kopil, C. M., Vais, H., Cheung, K. H., Siebert, A. P., Mak, D. O., Foskett, J. K., and Neumar, R. W. (2011) Calpain-cleaved type 1 inositol 1,4,5-trisphosphate receptor ($InsP_3R1$) has $InsP_3$-independent gating and disrupts intracellular Ca^{2+} homeostasis. *J. Biol. Chem.* **286**, 35998-36010

87. SB0118: Panigrahi, A. K., Zhang, N., Mao, Q., and Pati, D. (2011) Calpain-1 cleaves Rad21 to promote sister chromatid separation. *Mol. Cell. Biol.* **31**, 4335-4347

88. SB0119, SB0120: Wolf, B. B., Goldstein, J. C., Stennicke, H. R., Beere, H., Amarante-Mendes, G. P., Salvesen, G. S., and Green, D. R. (1999) Calpain functions in a caspase-independent manner to promote apoptosis-like events during platelet activation. *Blood* **94**, 1683-1692

89. SB0121: Wu, H. Y., Tomizawa, K., Oda, Y., Wei, F. Y., Lu, Y. F., Matsushita, M., Li, S. T., Moriwaki, A., and Matsui, H. (2004) Critical role of calpain-mediated cleavage of calcineurin in excitotoxic neurodegeneration. *J. Biol. Chem.* **279**, 4929-4940

90. SB0122: Chan, K. T., Bennin, D. A., and Huttenlocher, A. (2010) Regulation of adhesion dynamics by calpain-mediated proteolysis of focal adhesion kinase (FAK). *J. Biol. Chem.* **285**, 11418-11426

91. SB0123: Blachford, C., Celic, A., Petri, E. T., and Ehrlich, B. E. (2009) Discrete proteolysis of neuronal calcium sensor-1 (NCS-1) by mu-calpain disrupts calcium binding. *Cell Calcium* **46**, 257-262

92. SB0124: Cortesio, C. L., Perrin, B. J., Bennin, D. A., and Huttenlocher, A. (2010) Actin-binding protein-1 interacts with WASp-interacting protein to regulate growth factor-induced dorsal ruffle formation. *Mol. Biol. Cell* **21**, 186-197

93. SB0125: Du, W., Huang, J., Yao, H., Zhou, K., Duan, B., and Wang, Y. (2010) Inhibition of TRPC6 degradation suppresses ischemic brain damage in rats. *J. Clin. Invest.* **120**, 3480-3492

94. SB0126: Martinez, L. B., Walsh, S. M., Jacobsen, M. T., Sato, S., Wiederin, J., Ciborowski, P., and Ikezu, T. (2009) Calpain and proteasomal regulation of antiretroviral zinc finger protein OTK18 in human macrophages: visualization in live cells by intramolecular FRET. *Journal of neuroimmune pharmacology : the official journal of the Society on NeuroImmune Pharmacology* **4**, 116-128

95. SB0127: Banik, N. L., Chou, C. H., Deibler, G. E., Krutzch, H. C., and Hogan, E. L. (1994) Peptide bond specificity of calpain: proteolysis of human myelin basic protein. *J. Neurosci. Res.* **37**, 489-496

96. SB0128: Wei, G., Yin, Y., Li, W., Bito, H., She, H., and Mao, Z. (2012) Calpain-mediated degradation of myocyte enhancer factor 2D contributes to excitotoxicity by activation of extrasynaptic N-methyl-D-aspartate receptors. *J. Biol. Chem.* **287**, 5797-5805

97. SB0129: Chou, J. S., Impens, F., Gevaert, K., and Davies, P. L. (2011) m-Calpain activation in vitro does not require autolysis or subunit dissociation. *Biochim. Biophys. Acta* **1814**, 864-872

98. SB0130: Liu, M. C., Kobeissy, F., Zheng, W., Zhang, Z., Hayes, R. L., and Wang, K. K. (2011) Dual vulnerability of tau to calpains and caspase-3 proteolysis under neurotoxic and neurodegenerative conditions. *ASN Neuro* **3**, e00051

99. SB0131: Gomes, J. R., Lobo, A. C., Melo, C. V., Inacio, A. R., Takano, J., Iwata, N., Saido, T. C., de Almeida, L. P., Wieloch, T., and Duarte, C. B. (2011) Cleavage of the vesicular GABA transporter under excitotoxic conditions is followed by accumulation of the truncated transporter in nonsynaptic sites. *J. Neurosci.* **31**, 4622-4635

100. SB0132: Bozoky, Z., Rona, G., Klement, E., Medzihradszky, K. F., Merenyi, G., Vertessy, B. G., and Friedrich, P. (2011) Calpain-catalyzed proteolysis of human dUTPase specifically removes the nuclear localization signal peptide. *PLoS One* **6**, e19546

101. SB0134: Ma, S., Liu, S., Huang, Q., Xie, B., Lai, B., Wang, C., Song, B., and Li, M. (2012) Site-specific phosphorylation protects glycogen synthase kinase-3β from calpain-mediated truncation of its N and C termini. *J. Biol. Chem.* **287**, 22521-22532

102. SB0135: Gao, W., Liu, J., Hu, M., Huang, M., Cai, S., Zeng, Z., Lin, B., Cao, X., Chen, J., Zeng, J. Z., Zhou, H., and Zhang, X. K. (2013) Regulation of proteolytic cleavage of retinoid X receptor-alpha by GSK-3beta. *Carcinogenesis* **34**, 1208-1215

103. SB0136: Abe, K., and Takeichi, M. (2007) NMDA-receptor activation induces calpain-mediated beta-catenin cleavages for triggering gene expression. *Neuron* **53**, 387-397

104. SB0137: Watkins, G. R., Wang, N., Mazalouskas, M. D., Gomez, R. J., Guthrie, C. R., Kraemer, B. C., Schweiger, S., Spiller, B. W., and Wadzinski, B. E. (2012) Monoubiquitination promotes calpain cleavage of the protein phosphatase 2A (PP2A) regulatory subunit alpha4, altering PP2A stability and microtubule-associated protein phosphorylation. *J. Biol. Chem.* **287**, 24207-24215

105. SB0138: Valero, J. G., Cornut-Thibaut, A., Juge, R., Debaud, A. L., Gimenez, D., Gillet, G., Bonnefoy-Berard, N., Salgado, J., Salles, G., Aouacheria, A., and Kucharczak, J. (2012) micro-Calpain conversion of antiapoptotic Bfl-1 (BCL2A1) into a prodeath factor reveals two distinct alpha-helices inducing mitochondria-mediated apoptosis. *PLoS One* **7**, e38620

106. SB0139: Wang, C. F., and Huang, Y. S. (2012) Calpain 2 activated through N-methyl-D-aspartic acid receptor signaling cleaves CPEB3 and abrogates CPEB3-repressed translation in neurons. *Mol. Cell. Biol.* **32**, 3321-3332

107. SB0140: Yamashita, T., Hideyama, T., Hachiga, K., Teramoto, S., Takano, J., Iwata, N., Saido, T. C., and Kwak, S. (2012) A role for calpain-dependent cleavage of TDP-43 in amyotrophic lateral sclerosis pathology. *Nature communications* **3**, 1307

108. SB0141: Haacke, A., Hartl, F. U., and Breuer, P. (2007) Calpain inhibition is sufficient to suppress aggregation of polyglutamine-expanded ataxin-3. *J. Biol. Chem.* **282**, 18851-18856

109. SB0142: Shao, H., Travers, T., Camacho, C. J., and Wells, A. (2013) The carboxyl tail of alpha-actinin-4 regulates its susceptibility to m-calpain and thus functions in cell migration and spreading. *Int. J. Biochem. Cell Biol.* **45**, 1051-1063

110. SB0143: Sprague, C. R., Fraley, T. S., Jang, H. S., Lal, S., and Greenwood, J. A. (2008) Phosphoinositide binding to the substrate regulates susceptibility to proteolysis by calpain. *J. Biol. Chem.* **283**, 9217-9223

111. SB0144: Xu, J., Kurup, P., Zhang, Y., Goebel-Goody, S. M., Wu, P. H., Hawasli, A. H., Baum, M. L., Bibb, J. A., and Lombroso, P. J. (2009) Extrasynaptic NMDA receptors couple preferentially to excitotoxicity via calpain-mediated cleavage of STEP. *J. Neurosci.* **29**, 9330-9343

112. SB0145 (cf. SB0029): Kitamura, S., Muroya, S., Tanabe, S., Okumura, T., Chikuni, K., and Nishimura, T. (2005) Mechanism of production of troponin T fragments during postmortem aging of porcine muscle. *J. Agric. Food Chem.* **53**, 4178-4181

113. SB0146: Wu, C. Y., Chen, B., Jiang, Y. P., Jia, Z., Martin, D. W., Liu, S., Entcheva, E., Song, L. S., and Lin, R. Z. (2014) Calpain-dependent cleavage of junctophilin-2 and T-tubule remodeling in a mouse model of reversible heart failure. *Journal of the American Heart Association* **3**, e000527

114. SB0147: Charton, K., Sarparanta, J., Vihola, A., Milic, A., Jonson, P. H., Suel, L., Luque, H., Boumela, I., Richard, I., and Udd, B. (2015) CAPN3-mediated processing of C-terminal titin replaced by pathological cleavage in titinopathy. *Hum. Mol. Genet.*

115. SB0148: Ono, Y., Iemura, S., Novak, S. M., Doi, N., Kitamura, F., Natsume, T., Gregorio, C. C., and Sorimachi, H. (2013) PLEIAD/SIMC1/C5orf25, a novel autolysis regulator for a skeletal-muscle-specific calpain, CAPN3, scaffolds a CAPN3 substrate, CTBP1. *J. Mol. Biol.* **425**, 2955-2972

116. SB0150: Federici, C., Eshdat, Y., Richard, I., Bertin, B., Guillaume, J. L., Hattab, M., Beckmann, J. S., Strosberg, A. D., and Camoin, L. (1999) Purification and identification of two putative autolytic sites in human calpain 3 (p94) expressed in heterologous systems. *Arch. Biochem. Biophys.* **363**, 237-245

117. Macqueen, D. J., Delbridge, M. L., Manthri, S., and Johnston, I. A. (2010) A newly classified vertebrate calpain protease, directly ancestral to CAPN1 and 2, episodically evolved a restricted physiological function in placental mammals. *Mol. Biol. Evol.* **27**, 1886-1902

Table S2. List of 94 synthesized oligopeptides and their characteristics (P94mix library). The P87mix library excluded the shaded peptides.

ID No.	Peptide sequence	Gene product	SB No.[*1]	Definition	Species[*2]	gi#	aar length	Relative Cleavage site[*3]	Cleavage site[*4]	Seq. Position	calpain source[*5]	Calculated mono-isotopic MH^+/Z
1[*6]	SLSCANLSTFAQPPPAQPPA	**CDK5R1**	76	p35, Cyclin-dependent kinase 5 (CDK5) regulatory subunit 1	*B. taurus*	4502737	307	10	98	89-108	2	**2,246.1280**
2	GTYATQSALSSSRPTKKEED	**COPB1**	44	β-COP, β-subunit of coatomer protein complex	*H. sapiens*	7705369	953	10	528	519-538	1	**2,405.1948**
3	EEVRHFSPRGKFKVICLTVL	**PHKG**	18	Phosphorylase b kinase γ catalytic chain	*O. cuniculus*	1660	387	10	303	294-313	2	**2,607.4597**
4	EEEKPRPKLTAPKIPEGEKV	**TNNT3**	29	Troponin T3 (fast skeletal muscle), TnTf	*O. cuniculus*	1717774	279	10	65	56-75	1 or 2	**2,524.4139**
5	SQRSKYLASASTMDHARHGF	**MBP**	45	MBP, Myelin basic protein	*B. taurus*	126796	169	10	16	7-26	2	**2,499.2315**
6	PVVHFFKNIVTPRTPPPSQG	**MBP**	45	MBP, Myelin basic protein	*B. taurus*	126796	169	10, 13	93; 96	84-103	2	**2,467.3614**
7	VEDTKPKPSRLFMPNLVPPK	**TNNT2**	66	Troponin T2 (cardiac), TnTc	*M. musculus*	6755843	288	10	71	62-81	1	**2,542.4220**
8	KAKLGPAGNKVISPSEDRKQ	**PRKCA**	55	Protein kinase Cα	*R. norvegicus*	109492115	672	3, 10, 18	316; 309, 324	307-326	1 and 2	**2,372.3414**
9	YERVRMGPSSSPIPSPSPSP	**PRKCG**	89	Protein kinase Cγ	*R. norvegicus*	6981400	697	10	321	312-331	1 and 2	**2,377.1974**
10	RQKFERAKIGQGTKAPEEKT	**PRKCB**	39	Protein kinase Cβ	*R. norvegicus*	76880457	673	10, 19	311; 320	302-321	1 and 2	**2,551.4109**
11	TPKPPSQALHSQPAPGSVKA	**PLCB1**	8	Phosphoinositide-specific phospholipase Cβ1	*B. taurus*	27807357	1,216	10	880	871-890	2	**2,247.2250**
12	SAASAPAAVHSGPPDKPLSN	**PARP1**	65	ADP-ribosyltransferase (NAD^+) poly(ADP-ribose) polymerase	*B. taurus*	27807449	1,016	10	384	375-394	1	**2,123.0885**
13	QDEEAVKKLTVNPGTKSKLP	**PARP1**	65	ADP-ribosyltransferase (NAD^+) poly(ADP-ribose) polymerase	*B. taurus*	27807449	1,016	10	658	649-668	1	**2,431.3561**
14	QHLCGSHLVEALYLVCGERG	**INS**	60	Insulin	*B. taurus*	27806865	105	2, 5, 10, 13	37; 29, 32, 40	28-47	1 or 2	**2,433.2171**
15	EGCSSQHWSYGLRPGGKRNT	**GNRH1**	41	Gonadotropin-releasing hormone 1	*R. norvegicus*	6980968	92	10, 11	28; 29	19-38	1 and 2	**2,469.1846**
16	SLTAGDRVYIHPFHLLYYSK	**AGT**	27	Angiotensin	*R. norvegicus*	19705570	477	9	28	20-39	1 and 2	**2,629.3931**
17	IPYILKRQLYENKPRRPYIL	**NTS**	34	Neurotensin	*B. taurus*	85719294	170	10, 15	153; 158	144-163	1 and 2	**2,822.6561**
18	LYKRYGGFLRRIRPKLKWDN	**PDYN**	49	β-Neoendorphin-dynorphin	*M. musculus*	148747390	248	10, 17	207; 214	198-217	1 and 2	**2,828.6316**
19	YLDSRRAQDFVQWLMNTKRN	**GCG**	54	Glucagon	*R. norvegicus*	6978882	180	3, 5, 10, 13, 15	74; 67, 69, 77, 79	65-84	1 and 2	**2,790.4262**
20	RAQDFVQWLMNTKRNRNNIA	**GCG**	54	Glucagon	*R. norvegicus*	6978882	180	5, 8, 10	79; 74, 77	70-89	1 and 2	**2,724.4269**
21	VVAAKKSPVKATAPELKEEE	**NEFM**	10	Neurofilament medium polypeptide, Neurofilament 3	*B. taurus*	119923501	931	10	516	507-526	1	**2,373.3393**
22	LTPVVLRPEIRKPEVLRPET	**CASP9**	42	Caspase-9	*H. sapiens*	14790124	416	10, 15	115; 120	106-125	2	**2,591.5401**
23	CSSVTASTENLVPDYWIDGS	**CAMK4**	6	Calcium/calmodulin-dependent protein kinase IV	*M. musculus*	6753252	469	10	23	14-33	2	**2,393.0971**
24	EEQEYVQTVKSSKGGPGSAV	**ANXA1**	48	Annexin I	*H. sapiens*	4502101	346	10	26	17-36	1	**2,329.1676**
25	RAVFPSIVGRPRHQGVMVGM	**ACTB**	31	β-Actin	*H. sapiens*	4501885	375	10	37	28-47	1 and/or 2	**2,443.3331**
26	SISERLSVLKGAKPDVSNGQ	**MIP**	46	Aquaporin-0	*H. sapiens*	6912506	263	9, 10	238; 237	229-248	2	**2,334.2781**
27	DVSNGQPEVTGEPVELNTQA	**MIP**	46	Aquaporin-0	*H. sapiens*	6912506	263	10, 17, 18	252; 259, 260	243-262	2	**2,333.1261**
28	DVQEVRRLLHRELVHPDALN	**CDKN2D**	83	p19, INK4d, Cyclin-dependent kinase inhibitor 2D	*H. sapiens*	4502753	166	6, 10	29; 25	20-39	1	**2,658.4592**
29	AIALELLKQGASPNVQDTSG	**CDKN2D**	83	p19, INK4d, Cyclin-dependent kinase inhibitor 2D	*H. sapiens*	4502753	166	10	64	55-74	1	**2,261.2141**
30	YRRMFGGSGTSSRPSSNRSY	**VIM**	37	Vimentin	*M. musculus*	31982755	466	8, 10	20; 18	11-30	2	**2,502.2060**
31	YVTRSSAVRLRSSVPGVRLL	**VIM**	37	Vimentin	*M. musculus*	31982755	466	3, 10	70; 63	61-80	2	**2,465.4469**
32	QIDVDVSKPDLTAALRDVRQ	**VIM**	37	Vimentin	*M. musculus*	31982755	466	10	264	255-274	2	**2,488.3524**
33	GFRRAVSELDAKQAEAIMSP	**TH**	59	Tyrosine hydroxylase	*B. taurus*	27806937	491	10, 18	22; 30	13-32	1 or 2	**2,425.2662**
34	VSPKKSTVLQQQYNRVGKVE	**TLN1**	81	Talin 1	*G. gallus*	6755809	2,541	10	433	424-443	1	**2,537.4204**
35	KRHEAFEKSTASWAERFAAL	**SPTB**	78	β-I-Spectrin, isoform 2/a	*H. sapiens*	67782321	2,328	10, 13	2,058; 2,061	2049-2068	1	**2,584.3425**
36	VQAVQQQEVYGMMPRDETDS	**SPTAN1**	61	α-II-Spectrin, α-fodrin (non-erythrocytic)	*H. sapiens*	4507191	2,472	10	1,176	1167-1186	1	**2,560.1812**
37	FKAKKAAMMTQPPATPALPR	**RYR1**	15	Ryanodine receptor	*O. cuniculus*	1714	5,037	10	1,400	1391-1410	2	**2,404.3361**
38	TEKKKTRKISQTAQTYDPRE	**RYR1**	15	Ryanodine receptor	*O. cuniculus*	1714	5,037	10	2,843	2834-2853	2	**2,657.4375**
39	QDWSQNNALQFQKNKLRINR	**GRIN2A**	72	Glutamate receptor, ionotropic, NMDA 2A	*R. norvegicus*	140971205	1,464	10	1,278	1269-1288	1	**2,750.4603**
40	LEGNLYGSLFSVPSSKLLGN	**GRIN2A**	72	Glutamate receptor, ionotropic, NMDA 2A	*R. norvegicus*	140971205	1,464	10	1,329	1320-1339	1	**2,344.2553**
41	PAGWKPGSDTIKPNVDDSKE	**PRDX2**	91	Thioredoxin peroxidase 1	*S. scrofa*	32189392	198	9, 10, 11	182; 181, 183	173-192	1	**2,390.1992**
42	ITEAEDLPLRMEEPSEKKAP	**MYO5A**	33	Myosin 5A, heavy chain 12	*G. gallus*	46048699	1,829	10	1,140	1131-1150	1 or 2	**2,532.3020**
43	FNSSKLRVNKLILQLCIGNH	**NF2**	9	Neurofibromin 2, Merlin	*H. sapiens*	4557795	595	10	294	285-304	1 and 2	**2,546.4393**
44	FEMATRAALQARRGKKKSGC	**RHOA**	38	RhoA, ras homolog gene family, member A	*H. sapiens*	10835049	193	10	180	171-190	1	**2,458.3287**
45	AHKAATKIQASFRGHITRKK	**GAP43**	67	GAP-43, Growth associated protein 43	*R. norvegicus*	8393415	226	10	40	31-50	2	**2,498.4584**
46	ETQTVQQELKSLPTTKMAQT	**CRYBA1**	69	Crystallin, βA1/A3	*B. taurus*	27806255	215	10, 16	11; 17	2-21	2	**2,511.3129**
47	LRAKNSDLLTSPDVGLLKLA	**JUN**	86	c-jun	*R. norvegicus*	11177864	334	10	62	53-72	2	**2,373.3870**
48	GGGGYSASLHSEPPVYANLS	**JUN**	86	c-jun	*R. norvegicus*	11177864	334	10	164	155-174	2	**2,212.0675**
49	LVSSVAPSQTRAPHPYGLPT	**FOS**	64	c-fos	*R. norvegicus*	63147420	380	10	90	81-100	2	**2,327.2512**
50	SSPLSHRSKRSLSCRPPMVK	**VWF**	30	von Willebrand factor	*M. musculus*	115511022	2,813	10	763	754-773	2	**2,502.3550**
51	NTPPVGWVNMSQSTVVLGTD	**SLC6A5**	13	Solute carrier family 6, Gly transporter 2	*R. norvegicus*	42627863	799	10	156	147-166	1 and/or 2	**2,351.1706**
52	MVHLTPEEKSAVTALWGKV	**HBB**	32	β-Globin	*H. sapiens*	4504349	147	9	9	1-19	1	**2,345.2692**
53	IKPRSAPFSFLSNVKYNFMR	**IL1A**	19	IL-1 α, Interleukin 1 α	*H. sapiens*	27894330	271	10	118	109-128	1	**2,651.4284**
54	KWDTANNPLYKEATSTFTNI	**ITGB3**	43	Integrin β3	*H. sapiens*	47078292	788	4, 10, 17	773; 767, 780	764-783	1	**2,563.2833**
55	NWKQDSNPLYKSAITTTINP	**ITGB7**	62	Integrin β7	*H. sapiens*	4504777	798	1, 2, 5, 6, 10, 16, 17	778; 769, 770, 773, 774, 784, 785	769-788	2	**2,540.3149**

Table S2. List of 94 synthesized oligopeptides and their characteristics (P94mix library). The P87mix library excluded the shaded peptides.

ID No.	Peptide sequence	Gene product	SB No.*1	Definition	Species*2	gi#	aar length	Relative Cleavage site*3	Cleavage site*4	Seq. Position	calpain source*5	Calculated mono-isotopic MH^+/Z
56	ENPIYKSPINNFKNPNYGRK	**ITGB1**	79	Integrin β1 isoform 1D	*H. sapiens*	19743819	801	5, 10, 11, 12, 13, 17	788; 783, 789, 790, 791, 795	779-798	2	**2,642.3843**
57	SQWNNDNPLFKSATTTVMNP	**ITGB2**	2	Integrin β2	*H. sapiens*	89191865	769	10, 16, 18	754; 760, 762	745-764	2	**2,514.2087**
58	EFAKFEKEKMNAKWDTGENP	**ITGB1**	63	Integrin β1 isoform 1A	*H. sapiens*	19743813	798	10, 16, 17	771; 777, 778	762-781	2	**2,648.2819**
59	GQILWFRGLNRIQTQIRVVK	**ATP2B2**	56	Plasma membrane calcium ATPase 2	*H. sapiens*	48255951	1,243	10	1,135	1126-1145	1	**2,674.5786**
60	CVRQHARQQDKERLAALGPE	**PTPRN**	24	Protein tyrosine phosphatase, receptor type, N	*H. sapiens*	4506321	979	10	608	599-618	1	**2,554.3425**
61	PEPSRVSSVSSQFSDAAQAS	**PTPRN**	24	Protein tyrosine phosphatase, receptor type, N	*H. sapiens*	4506321	979	10	658	649-668	1	**2,286.1002**
62	GNRSSHSRLGRIEADSESQE	**BID**	84	BID, BH3 interacting domain death agonist	*H. sapiens*	4557361	195	10	70	61-80	2	**2,464.1929**
63	IMKTGALLLQGFIQDRAGRM	**BAX**	26	BAX, BCL2-associated X protein	*H. sapiens*	20631958	192	10	28	19-38	1 or 2	**2,468.3634**
64	RDRSTSGGKMKVNGAPREDA	**IGFBP4**	70	IGFBP4, Insulin-like growth factor binding protein 4	*H. sapiens*	62243290	258	13	159	147-166	1	**2,381.2108**
65	VLSPADKTNVKAAWGKVGAH	**HBA1**	40	α1-Globin	*H. sapiens*	4504347	142	10	11	2-21	1	**2,298.2723**
66	VRKRTLRRLLQERELVEPLT	**EGFR**	50	EGFR, ErbB-1, Epidermal growth factor receptor	*H. sapiens*	29725609	1,210	10	683	674-693	2	**2,754.6583**
67	VQPTCVNSTFDSPAHWAQKG	**EGFR**	50	EGFR, ErbB-1, Epidermal growth factor receptor	*H. sapiens*	29725609	1,210	10	1,151	1142-1161	2	**2,422.1614**
68	VMETVTTVTTREQILVKHAQ	**DMD**	73	Dystrophin	*H. sapiens*	5032283	3,685	10	690	681-700	2	**2,533.3812**
69	VVHGVATVAEKTKEQVTNVG	**SNCA**	36	α-Synuclein	*H. sapiens*	4507109	140	10	57	48-67	1	**2,315.2723**
70	AEAVKKDRRKKLTQSKFVGG	**IGFBP5**	23	IGFBP-5, Insulin-like growth factor binding protein 5	*H. sapiens*	10834982	272	13	161	149-168	1	**2,495.4574**
71	TVPVEAVTSKTSNIRANFEN	**CTTN**	22	Cortactin	*H. sapiens*	20357552	550	10, 15	346; 351	337-356	2	**2,426.2680**
72	LRSQQLAPQYTYAQGGQQTW	**FLNA**	90	Filamin A	*H. sapiens*	116063573	2,639	10	1,753	1744-1763	1 or 2	**2,573.2901**
73	QETFSDLWKLLPENNVLSPL	**TP53**	53	p53	*H. sapiens*	120407068	393	10	25	16-35	unclear	**2,592.3714**
74	VGMVEASPLSAKPFSQFEEK	**GJA8**	16	Connexin-49, Gap junction α-8	*O. aries*	6014503	440	10	300	291-310	unclear	**2,430.2379**
75	PEQAAANKSHGGLGGSYKVT	**CRYBB3**	95	Crystallin, βB3	*R. norvegicus*	13928958	211	8, 10	17; 15	8-27	2	**2,221.1365**
76	SVPRPAAKVGELPPGSYRLV	**CRYBB1**	88	Crystallin, βB1	*R. norvegicus*	6978711	249	10	49	40-59	2	**2,342.3349**
77	AKASATAAVNPGPDGKGKAG	**CRYBB1**	94	Crystallin, βB1	*B. taurus*	290	253	7, 10	15; 12	6-25	2	**2,017.0831**
78	VFEEFARQNLKDAGEYKEEK	**SAG**	25	S-arrestin	*B. taurus*	162670	404	2, 10	385; 377	376-395	unclear	**2,679.3418**
79	AARLQRDRLRAEGVGEHNNA	**CAPN11**	21	CAPN11, μ/m-calpain catalytic subunit, μ/mCL	*G. gallus*	45384280	705	10	17	8-27	unclear	**2,482.3140**
80	ISADLADEEEITEDDIEDGF	**CAPN11**	21	CAPN11, μ/m-calpain catalytic subunit, μ/mCL	*G. gallus*	45384280	705	10	529	520-539	unclear	**2,475.0939**
81	PPPPRSHVSMVDPNESEEVR	**CAPNS1**	17	CAPNS1, 30K, Calpain regulatory subunit	*G. gallus*	2506056	214	7, 10	35; 32	26-45	unclear	**2,508.2305**
82	RFRSIVHVVQAGIFVERMYR	**PDE1A**	4	Calcium/calmodulin-dependent 3',5'-cyclic nucleotide phosphodiesterase1A	*B. taurus*	162879	530	10	126	117-136	unclear	**2,712.5037**
83	ITPVYCTGVSAQVQKQRARE	**CAPN1**	20	CAPN1, μ-calpain catalytic subunit, μCL	*H. sapiens*	12408656	714	10	15	6-25	1	**2,483.3193**
84	VQKQRARELGLGRHENAIKY	**CAPN1**	20	CAPN1, μ-calpain catalytic subunit, μCL	*H. sapiens*	12408656	714	10	27	18-37	1	**2,615.4646**
85	KDREAAEGLGSHERAIKYLN	**CAPN2**	1	CAPN2, m-calpain catalytic subunit, mCL	*H. sapiens*	4502563	700	1, 2, 3, 4, 5, 10, 14	19; 10, 11, 12, 13, 14, 23	10-29	2	**2,506.3166**
86	EYEQMVKELQEIRLEPQEVP	**NFKBIA**	11	IkB-α, NFkB inhibitor α	*H. sapiens*	10092619	317	10	50	41-60	2	**2,736.3918**
87	LLLRRVVAGSPSVRSTSVRY	**VIMsc*7**	37	Vimentin (scramble of ID0031)	*M. musculus*	31982755	466	-	-	61-80	-	**2,465.4469**
88	EQVLKVGTVSRKNKYPQSQV	**TLN1sc**	81	Talin 1 (scramble of ID0034)	*G. gallus*	6755809	2,541	-	-	424-443	-	**2,537.4204**
89	SYDVTEEQDQRQPVMAMQGV	**SPTAN1sc**	61	α-II-Spectrin, α-fodrin, non-erythrocytic (scramble of ID0036)	*H. sapiens*	4507191	2,472	-	-	1167-1186	-	**2,560.1812**
90	RTPMLMAAPATKAKPAPKQF	**RYR1sc**	15	Ryanodine receptor (scramble of ID0037)	*O. cuniculus*	1714	5,037	-	-	1391-1410	-	**2,404.3361**
91	TDIQAKSWKNPYNLIPTNTS	**ITGB7sc**	62	Integrin β7 (scramble of ID0055)	*H. sapiens*	4504777	798	-	-	769-788	-	**2,540.3149**
92	GQQQASYRTLWYTQQPQAGL	**FLNAsc**	90	Filamin A (scramble of ID0072)	*H. sapiens*	116063573	2,639	-	-	1752-1771	-	**2,573.2901**
93	NSNFETPELQLLPKSWLLVD	**TP53sc**	53	p53 (scramble of ID0073)	*H. sapiens*	120407068	393	-	-	16-35	-	**2,592.3714**
94	ERHQRKGQLVYGKLIEARNA	**CAPN1sc**	20	CAPN1, μ-calpain catalytic subunit, μCL (scramble of ID0084)	*H. sapiens*	12408656	714	-	-	18-37	-	**2,615.4646**
	**average**											**2,488.0530**

*1 "SB No." is the substrate number used in the CaMP DB web site (http://www.calpain.org/browse.rb?cls=substrate).
*2 For species abbreviations, see Table S1.
*3 Numbers indicate the position of cleavage sites in the peptides relative to the N-terminus, with the 1st aar being 1. Although most oligopeptides were designed so the cleavage site was 10, some were displaced from 10 to reduce the peptide's hydrophobicity. Note that about half of the peptides were designed to have only one cleavage site, and the rest have plural sites.
*4 The calpain cleavage site is the C-terminus of the aar indicated by number, which is based on the full-length sequence indicated in "gi#."
*5 "unclear" indicates that the calpain used in the report was not clear. 1, 2, and 3 indicate calpain-1 (CAPN1/S1, μ-calpain), calpain-2 (CAPN2/S1, m-calpain), and calpain-3 (CAPN3, p94), respectively.
*6 Peptides indicated by shaded rows were not included in the kinetics experiments, because our preliminary MS analysis could not detect any spectra corresponding to them.
*7 "sc" indicates a scrambled sequence of the peptide ID listed under “Definition.”

Table S3. List of synthesized reference peptides used for quantification (P158mix library).

Peptides corresponding to the same site (*i.e.* , the N- and C-terminal peptides of the same site) are indicated under “Site identical to.” Nos. 1 to 104, and 105 to 158 correspond to Rp and Nv sites, respectively.

No.	Peptide ID	Sequence	Length	N-terminal modification	C-terminal modification	MH^+/Z	Site identical to
1	ID002-Rp-C	SSRPTKKEED	10	-	DKP	1,383.7232	
2	ID004-Rp-C	APKIPEGEKV	10	-	DKP	1,274.7472	ID004-Rp-N
3	ID005-Rp-C	STMDHARHGF	10	-	DKP	1,365.6486	
4	ID006-Rp-C	TPRTPPPSQG	10	-	DKP	1,244.6751	ID006-Rp-N
5	ID007-Rp-C	LFMPNLVPPK	10	-	DKP	1,362.7972	
6	ID008-Rp-C	VISPSEDRKQ	10	-	DKP	1,365.7490	
7	ID009-Rp-C	SPIPSPSPSP	10	-	DKP	1,172.6315	
8	ID010-Rp-C	QGTKAPEEKT	10	-	DKP	1,295.6959	ID010-Rp-N
9	ID011-Rp-C	SQPAPGSVKA	10	-	DKP	1,148.6428	ID011-Rp-N
10	ID012-Rp-C	SGPPDKPLSN	10	-	DKP	1,218.6483	
11	ID013-Rp-C	VNPGTKSKLP	10	-	DKP	1,247.7476	
12	ID014-Rp-C	LVCGERG	7	-	DKP	940.5038	
13	ID015-Rp-C	GLRPGGKRNT	10	-	DKP	1,262.7446	
14	ID017-Rp-C	ENKPRRPYIL	10	-	DKP	1,492.8752	ID017-Rp-N
15	ID018-Rp-C	RIRPKLKWDN	10	-	DKP	1,532.9178	ID018-Rp-N
16	ID019-Rp-C	VQWLMNTKRN	10	-	DKP	1,496.8160	
17	ID019-Rp-C2	LMNTKRN	7	-	DKP	1,083.6097	
18	ID019-Rp-C3	NTKRN	5	-	DKP	839.4852	
19	ID020-Rp-C	NTKRNRNNIA	10	-	DKP	1,407.7933	
20	ID021-Rp-C	ATAPELKEEE	10	-	DKP	1,323.6796	ID021-Rp-N
21	ID022-Rp-C	RKPEVLRPET	10	-	DKP	1,431.8436	
22	ID024-Rp-C	SSKGGPGSAV	10	-	DKP	1,053.5693	ID024-Rp-N
23	ID025-Rp-C	PRHQGVMVGM	10	-	DKP	1,318.6876	
24	ID026-Rp-C	GAKPDVSNGQ	10	-	DKP	1,179.6122	ID026-Rp-N
25	ID028-Rp-C	RELVHPDALN	10	-	DKP	1,370.7545	
26	ID029-Rp-C	ASPNVQDTSG	10	-	DKP	1,182.5755	ID029-Rp-N
27	ID031-Rp-C	RSSVPGVRLL	10	-	DKP	1,290.8010	
28	ID032-Rp-C	LTAALRDVRQ	10	-	DKP	1,349.8017	
29	ID033-Rp-C	AKQAEAIMSP	10	-	DKP	1,252.6724	
30	ID034-Rp-C	QQYNRVGKVE	10	-	DKP	1,427.7759	ID034-Rp-N
31	ID035-Rp-C	ASWAERFAAL	10	-	DKP	1,328.7115	ID035-Rp-N
32	ID035-Rp-C2	AERFAAL	7	-	DKP	984.5631	
33	ID036-Rp-C	GMMPRDETDS	10	-	DKP	1,345.5880	
34	ID037-Rp-C	QPPATPALPR	10	-	DKP	1,254.7323	ID037-Rp-N
35	ID038-Rp-C	QTAQTYDPRE	10	-	DKP	1,415.6919	
36	ID039-Rp-C	FQKNKLRINR	10	-	DKP	1,523.9287	
37	ID040-Rp-C	SVPSSKLLGN	10	-	DKP	1,208.7003	
38	ID041-Rp-C	IKPNVDDSKE	10	-	DKP	1,351.7222	
39	ID042-Rp-C	MEEPSEKKAP	10	-	DKP	1,352.6884	
40	ID043-Rp-C	LILQLCIGNH	10	-	DKP	1,330.7669	
41	ID044-Rp-C	ARRGKKKSGC	10	-	DKP	1,297.7639	
42	ID045-Rp-C	SFRGHITRKK	10	-	DKP	1,436.8603	ID045-Rp-N
43	ID046-Rp-C	SLPTTKMAQT	10	-	DKP	1,284.6986	ID046-Rp-N
44	ID047-Rp-C	SPDVGLLKLA	10	-	DKP	1,219.7414	ID047-Rp-N
45	ID048-Rp-C	SEPPVYANLS	10	-	DKP	1,283.6636	
46	ID049-Rp-C	RAPHPYGLPT	10	-	DKP	1,315.7275	
47	ID050-Rp-C	SLSCRPPMVK	10	-	DKP	1,324.7234	
48	ID052-Rp-C	SAVTALWGKV	10	-	DKP	1,238.7261	
49	ID053-Rp-C	LSNVKYNFMR	10	-	DKP	1,478.7942	ID053-Rp-N
50	ID054-Rp-C	KEATSTFTNI	10	-	DKP	1,318.7007	
51	ID055-Rp-C	KSAITTTINP	10	-	DKP	1,252.7265	
52	ID056-Rp-C	NFKNPNYGRK	10	-	DKP	1,444.7813	
53	ID057-Rp-C	KSATTTVMNP	10	-	DKP	1,256.6673	
54	ID058-Rp-C	NAKWDTGENP	10	-	DKP	1,338.6442	
55	ID059-Rp-C	RIQTQIRVVK	10	-	DKP	1,447.9225	
56	ID060-Rp-C	KERLAALGPE	10	-	DKP	1,290.7534	
57	ID061-Rp-C	SQFSDAAQAS	10	-	DKP	1,218.5755	
58	ID063-Rp-C	GFIQDRAGRM	10	-	DKP	1,357.7163	ID063-Rp-N
59	ID065-Rp-C	KAAWGKVGAH	10	-	DKP	1,231.7064	
60	ID066-Rp-C	QERELVEPLT	10	-	DKP	1,420.7800	ID066-Rp-N
61	ID067-Rp-C	DSPAHWAQKG	10	-	DKP	1,303.6547	
62	ID068-Rp-C	REQILVKHAQ	10	-	DKP	1,428.8439	
63	ID069-Rp-C	KTKEQVTNVG	10	-	DKP	1,310.7432	
64	ID070-Rp-C	QSKFVGG	7	-	DKP	929.5209	
65	ID071-Rp-C	TSNIRANFEN	10	-	DKP	1,372.6973	ID071-Rp-N
66	ID072-Rp-C	TYAQGGQQTW	10	-	DKP	1,346.6493	
67	ID073-Rp-C	LPENNVLSPL	10	-	DKP	1,302.7422	
68	ID074-Rp-C	AKPFSQFEEK	10	-	DKP	1,417.7480	
69	ID075-Rp-C	GGLGGSYKVT	10	-	DKP	1,145.6319	
70	ID076-Rp-C	ELPPGSYRLV	10	-	DKP	1,337.7581	ID076-Rp-N
71	ID077-Rp-C	PGPDGKGKAG	10	-	DKP	1,090.6009	ID077-Rp-N
72	ID078-Rp-C	KDAGEYKEEK	10	-	DKP	1,403.7171	
73	ID079-Rp-C	AEGVGEHNNA	10	-	DKP	1,204.5711	
74	ID081-Rp-C	VDPNESEEVR	10	-	DKP	1,380.6759	
75	ID083-Rp-C	AQVQKQRARE	10	-	DKP	1,420.8137	
76	ID084-Rp-C	LGRHENAIKY	10	-	DKP	1,407.7861	ID084-Rp-N
77	ID085-Rp-C	SHERAIKYLN	10	-	DKP	1,437.7966	
78	ID086-Rp-C	EIRLEPQEVP	10	-	DKP	1,416.7851	ID086-Rp-N

Table S3. List of synthesized reference peptides used for quantification (P158mix library).

No.	Peptide ID	Sequence	Length	N-terminal modification	C-terminal modification	MH$^+$/Z	Site identical to
79	ID004-Rp-N	EEEKPRPKLT	10	Ac	-	1,268.6850	ID004-Rp-C
80	ID006-Rp-N	PVVHFFKNIV	10	Ac	-	1,241.7047	ID006-Rp-C
81	ID006-Rp-N2	PVVHFFKNIVTPR	13	Ac	-	1,595.9062	
82	ID010-Rp-N	RQKFERAKIG	10	Ac	-	1,274.7333	ID010-Rp-C
83	ID011-Rp-N	TPKPPSQALH	10	Ac	-	1,117.6006	ID011-Rp-C
84	ID017-Rp-N	IPYILKRQLY	10	Ac	-	1,348.7993	ID017-Rp-C
85	ID018-Rp-N	LYKRYGGFLR	10	Ac	-	1,314.7322	ID018-Rp-C
86	ID021-Rp-N	VVAAKKSPVK	10	Ac	-	1,068.6781	ID021-Rp-C
87	ID024-Rp-N	EEQEYVQTVK	10	Ac	-	1,294.6167	ID024-Rp-C
88	ID026-Rp-N	SISERLSVLK	10	Ac	-	1,173.6843	ID026-Rp-C
89	ID029-Rp-N	AIALELLKQG	10	Ac	-	1,097.6570	ID029-Rp-C
90	ID034-Rp-N	VSPKKSTVLQ	10	Ac	-	1,128.6628	ID034-Rp-C
91	ID035-Rp-N	KRHEAFEKST	10	Ac	-	1,274.6493	ID035-Rp-C
92	ID037-Rp-N	FKAKKAAMMT	10	Ac	-	1,168.6222	ID037-Rp-C
93	ID045-Rp-N	AHKAATKIQA	10	Ac	-	1,080.6166	ID045-Rp-C
94	ID046-Rp-N	ETQTVQQELK	10	Ac	-	1,245.6327	ID046-Rp-C
95	ID047-Rp-N	LRAKNSDLLT	10	Ac	-	1,172.6639	ID047-Rp-C
96	ID053-Rp-N	IKPRSAPFSF	10	Ac	-	1,191.6526	ID053-Rp-C
97	ID056-Rp-N	ENPIYKSPINNFK	13	Ac	-	1,605.8277	
98	ID063-Rp-N	IMKTGALLLQ	10	Ac	-	1,129.6655	ID063-Rp-C
99	ID066-Rp-N	VRKRTLRRLL	10	Ac	-	1,352.8966	ID066-Rp-C
100	ID071-Rp-N	TVPVEAVTSK	10	Ac	-	1,072.5890	ID071-Rp-C
101	ID076-Rp-N	SVPRPAAKVG	10	Ac	-	1,023.5951	ID076-Rp-C
102	ID077-Rp-N	AKASATAAVN	10	Ac	-	945.5005	ID077-Rp-C
103	ID084-Rp-N	VQKQRARELG	10	Ac	-	1,226.6969	ID084-Rp-C
104	ID086-Rp-N	EYEQMVKELQ	10	Ac	-	1,338.6251	ID086-Rp-C
105	ID005-Nv (13-20)	MDHARHGF	8	-	DKP	1,177.5689	
106	ID015-Nv (13-20)	RPGGKRNT	8	-	DKP	1,092.6390	
107	ID016-Nv (11-20)	HPFHLLYYSK	10	-	DKP	1,511.8163	
108	ID017-Nv (12-20)	NKPRRPYIL	9	-	DKP	1,363.8326	ID017-Nv (1-11)
109	ID020-Nv (12-20)	TKRNRNNIA	9	-	DKP	1,293.7504	
110	ID020-Nv (13-20)	KRNRNNIA	8	-	DKP	1,192.7027	
111	ID028-Nv (14-20)	VHPDALN	7	-	DKP	972.5267	
112	ID031-Nv (12-20)	SSVPGVRLL	9	-	DKP	1,134.6999	
113	ID031-Nv (13-20)	SVPGVRLL	8	-	DKP	1,047.6679	
114	ID031-Nv (5-20)	SSAVRLRSSVPGVRLL	16	-	DKP	1,904.1558	
115	ID031-Nv (7-20)	AVRLRSSVPGVRLL	14	-	DKP	1,730.0917	
116	ID032-Nv (12-20)	TAALRDVRQ	9	-	DKP	1,236.7177	
117	ID032-Nv (13-20)	AALRDVRQ	8	-	DKP	1,135.6700	
118	ID032-Nv (14-20)	ALRDVRQ	7	-	DKP	1,064.6329	
119	ID034-Nv (12-20)	QYNRVGKVE	9	-	DKP	1,299.7173	
120	ID037-Nv (10-20)	TQPPATPALPR	11	-	DKP	1,355.7799	
121	ID038-Nv (14-20)	QTYDPRE	7	-	DKP	1,115.5485	
122	ID039-Nv (12-20)	QKNKLRINR	9	-	DKP	1,376.8603	
123	ID047-Nv (10-20)	TSPDVGLLKLA	11	-	DKP	1,320.7891	
124	ID049-Nv (12-20)	APHPYGLPT	9	-	DKP	1,159.6264	
125	ID052-Nv (15-20)	ALWGKV	6	-	DKP	880.5409	
126	ID053-Nv (13-20)	NVKYNFMR	8	-	DKP	1,278.6781	ID053-Nv (1-12)
127	ID063-Nv (10-20)	QGFIQDRAGRM	11	-	DKP	1,485.7749	
128	ID063-Nv (12-20)	FIQDRAGRM	9	-	DKP	1,300.6948	
129	ID064-Nv (11-20)	KVNGAPREDA	10	-	DKP	1,263.6810	
130	ID065-Nv (12-20)	AAWGKVGAH	9	-	DKP	1,103.6114	
131	ID067-Nv (16-20)	WAQKG	5	-	DKP	796.4470	
132	ID068-Nv (13-20)	QILVKHAQ	8	-	DKP	1,143.7002	
133	ID070-Nv (11-20)	KLTQSKFVGG	10	-	DKP	1,271.7476	
134	ID075-Nv (15-20)	GSYKVT	6	-	DKP	861.4834	
135	ID083-Nv (10-20)	SAQVQKQRARE	11	-	DKP	1,507.8457	
136	ID083-Nv (12-20)	QVQKQRARE	9	-	DKP	1,349.7766	
137	ID083-Nv (15-20)	KQRARE	6	-	DKP	994.5910	
138	ID083-Nv (7-20)	TGVSAQVQKQRARE	14	-	DKP	1,764.9833	
139	ID087-Nv (15-20)	STSVRY	6	-	DKP	919.5001	
140	ID090-Nv (12-20)	KAKPAPKQF	9	-	DKP	1,221.7472	ID090-Nv (1-12)
141	ID090-Nv (13-20)	AKPAPKQF	8	-	DKP	1,093.6522	
142	ID090-Nv (7-20)	AAPATKAKPAPKQF	14	-	DKP	1,632.9590	
143	ID094-Nv (12-20)	GKLIEARNA	9	-	DKP	1,178.7010	ID094-Nv (1-11)
144	ID007-Nv (1-12)	VEDTKPKPSRLF	12	Ac	-	1,458.7957	
145	ID007-Nv (1-15)	VEDTKPKPSRLFMPN	15	Ac	-	1,800.9318	
146	ID013-Nv (1-15)	QDEEAVKKLTVNPGT	15	Ac	-	1,670.8601	
147	ID017-Nv (1-11)	IPYILKRQLYE	11	Ac	-	1,477.8419	ID017-Nv (12-20)
148	ID020-Nv (1-13)	RAQDFVQWLMNTK	13	Ac	-	1,678.8375	
149	ID045-Nv (1-12)	AHKAATKIQASF	12	Ac	-	1,314.7170	
150	ID045-Nv (1-9)	AHKAATKIQ	9	Ac	-	1,009.5795	
151	ID052-Nv (1-16)	MVHLTPEEKSAVTALW	16	Ac	-	1,853.9471	
152	ID053-Nv (1-12)	IKPRSAPFSFLS	12	Ac	-	1,391.7687	ID053-Nv (13-20)
153	ID086-Nv (1-13)	EYEQMVKELQEIR	13	Ac	-	1,736.8529	
154	ID086-Nv (1-18)	EYEQMVKELQEIRLEPQE	18	Ac	-	2,333.1335	
155	ID090-Nv (1-12)	RTPMLMAAPATK	12	Ac	-	1,329.7023	ID090-Nv (13-20)
156	ID091-Nv (1-12)	TDIQAKSWKNPY	12	Ac	-	1,492.7436	
157	ID091-Nv (1-13)	TDIQAKSWKNPYN	13	Ac	-	1,606.7865	
158	ID094-Nv (1-11)	ERHQRKGQLVY	11	Ac	-	1,455.7821	ID094-Nv (12-20)

Table S4 Cleavability of all reported (Rp) sites

		Calpain-1						Calpain-2						Calpain-1 or -2	
		fragment			i115/i113			fragment			i115/i113				
ID	site	N	C	M	FL	cleaved	uncleaved	N	C	M	FL	cleaved	uncleaved	cleaved	uncleaved
2	10	0	1	1	0.115	1	0	1	1	0	0.318	1	0	1	0
4	10	0	1	0	0.684	2	0	0	2	0	0.633	1	1	2	0
5	10	1	5	0	0.556	1	0	0	6	0	0.765	1	0	1	0
6	10	3	0	1	0.228	1	0	2	1	1	0.284	1	0	1	0
6	13	0	2	1	0.228	1	0	0	3	1	0.284	1	0	1	0
7	10	4	0	0		0	1	3	0	0		0	1	0	1
8	3	0	1	0		0	1	0	1	0		0	1	0	1
8	10	0	4	0		1	0	0	2	0		1	0	1	0
8	18	0	5	0		0	1	0	3	0		0	1	0	1
9	10	1	2	1	0.549	1	0	1	2	0	0.188	1	0	1	0
10	10	0	2	0	0.786	1	0	0	0	0	0.624	1	0	1	0
10	19	0	7	0	0.786	1	0	0	5	0	0.624	0	1	1	0
11	10	2	2	0	0.508	1	1	0	0	0	0.663	2	0	2	0
12	10	4	5	0	0.630	1	0	2	3	1	0.762	1	0	1	0
13	10	2	0	1	0.038	1	0	2	0	1	0.279	1	0	1	0
14	2	1	0	0		0	1	0	0	0		0	1	0	1
14	5	1	0	0		0	1	0	0	0		0	1	0	1
14	10	1	0	0		0	1	0	0	0		0	1	0	1
14	13	0	1	0		3	0	0	0	0		0	3	3	0
15	10	0	1	0	0.565	1	0	0	0	0	0.419	1	0	1	0
15	11	0	2	0	0.565	0	1	0	1	0	0.419	1	0	1	0
16	9	1	0	0		1	0	5	0	0		0	1	1	0
17	10	1	1	0		1	0	1	0	0		1	0	1	0
17	15	0	5	0		0	1	0	4	0		0	1	0	1
18	10	0	0	0		1	0	0	0	0		1	0	1	0
18	17	0	1	0		1	0	0	1	0		1	0	1	0
19	3	3	0	0		0	1	4	0	0		0	1	0	1
19	5	3	0	0		0	1	4	0	0		0	1	0	1
19	10	2	0	0		1	0	4	0	0		0	1	1	0
19	13	2	1	0		1	0	2	1	0		1	0	1	0
19	15	0	2	0		1	0	0	2	0		1	0	1	0
20	5	0	0	0		0	1	0	0	0	0.298	0	1	0	1
20	8	0	0	0		1	0	0	0	0	0.298	1	0	1	0
20	10	0	1	0		1	0	0	1	0	0.298	1	0	1	0
21	10	1	1	0	0.472	1	0	1	0	0	0.643	1	0	1	0
22	10	2	1	0	0.691	1	0	1	0	0	0.881	1	0	1	0
22	15	0	2	0	0.691	1	0	0	1	0	0.881	1	0	1	0
24	10	0	1	0	0.197	2	1	0	0	0	0.080	3	0	3	0
25	10	2	1	0	0.000	0	1	1	1	0	0.000	0	1	0	1
26	9	1	0	1	0.285	0	1	1	1	2	0.350	0	1	0	1
26	10	0	0	0	0.285	3	0	0	1	0	0.350	3	0	3	0
28	6	1	0	0	0.187	0	1	1	0	0	0.000	0	1	0	1
28	10	0	0	0	0.187	1	0	0	0	0	0.000	1	0	1	0
29	10	6	5	0	0.162	2	0	5	4	0	0.437	2	0	2	0
30	8	1	3	0	0.000	0	1	0	3	0	0.000	0	1	0	1
30	10	1	5	0	0.000	1	0	0	5	0	0.000	1	0	1	0
31	3	3	0	0	0.000	1	0	2	0	0		1	0	1	0
31	10	2	3	4	0.000	2	0	1	2	4		2	0	2	0
32	10	3	1	0	0.312	1	0	4	0	0	0.297	1	0	1	0
33	10	0	0	0	0.469	1	0	0	0	0	0.689	1	0	1	0
33	18	0	4	0	0.469	0	2	0	2	0	0.689	2	0	2	0
34	10	0	0	0		1	0	0	0	0		1	0	1	0
35	10	0	1	1		1	0	0	2	0		1	0	1	0
35	13	0	3	1		2	1	0	4	0		2	1	3	0
36	10	0	0	0	0.029	1	0	0	0	0	0.331	1	0	1	0
37	10	0	1	0	0.129	1	0	0	2	0	0.249	1	0	1	0
38	10	0	2	0		1	0	0	2	0		1	0	1	0
39	10	1	1	0		1	0	0	1	0	0.315	1	0	1	0
40	10	0	0	0	0.058	1	0	0	0	0	0.119	0	1	1	0
41	9	0	1	0	0.717	0	1	0	1	0	0.909	0	1	0	1
41	10	0	1	0	0.717	1	0	0	1	0	0.909	0	1	1	0
41	11	0	2	0	0.717	0	1	0	1	0	0.909	0	1	0	1
42	10	0	0	0	0.523	1	0	1	0	0	0.604	1	0	1	0
43	10	0	0	0		0	0	0	0	0		0	0	0	0
44	10	3	0	2		1	0	3	0	2		1	0	1	0
45	10	3	0	0		1	0	3	0	1		1	0	1	0
46	10	0	0	0	0.007	3	0	0	0	0	0.011	1	2	3	0

		Calpain-1						Calpain-2						Calpain-1 or -2	
		fragment			i115/i113			fragment			i115/i113				
ID	site	N	C	M	FL	cleaved	uncleaved	N	C	M	FL	cleaved	uncleaved	cleaved	uncleaved
46	16	0	1	0	0.007	2	0	0	1	0	0.011	1	1	2	0
47	10	0	1	1	0.312	1	0	0	1	1	0.306	1	0	1	0
48	10	0	0	1	0.276	2	0	0	0	0	0.593	2	0	2	0
49	10	2	2	2	0.002	1	0	1	1	1	0.015	1	0	1	0
50	10	2	0	1		0	1	1	0	0		0	1	0	1
52	9	2	1	0		1	0	2	0	0	0.086	1	0	1	0
53	10	1	0	0		1	0	1	0	0		1	0	1	0
54	4	0	0	0		0	0	0	0	0		0	0	0	0
54	10	0	0	0		1	0	0	0	0		1	0	1	0
54	17	0	3	0		0	1	0	3	0		0	1	0	1
55	1	0	0	0		0	1	1	0	0		0	1	0	1
55	2	0	0	0		0	1	1	0	0		0	1	0	1
55	5	0	0	0		0	1	1	0	0		0	1	0	1
55	6	0	0	0		0	1	1	0	0		0	1	0	1
55	10	0	0	0		1	0	0	1	0		1	0	1	0
55	16	0	3	0		0	1	0	4	0		0	1	0	1
55	17	0	3	0		0	1	0	4	0		0	1	0	1
56	5	4	0	0		0	1	5	0	0		0	1	0	1
56	10	3	0	0		1	0	4	0	0		1	0	1	0
56	11	2	0	0		1	0	3	0	0		1	0	1	0
56	12	1	0	0		1	1	2	0	0		2	0	2	0
56	13	0	0	0		2	0	1	0	0		2	0	2	0
56	17	0	0	0		0	1	0	0	0		1	0	1	0
57	10	0	0	0		0	1	2	0	0		1	0	1	0
57	16	0	3	0		0	1	2	3	0		0	1	0	1
57	18	0	3	0		1	1	0	3	0		1	1	2	0
58	10	0	1	0	1.107	0	1	0	0	0	0.720	1	0	1	0
58	16	0	3	0	1.107	0	1	0	2	0	0.720	0	1	0	1
58	17	0	3	0	1.107	0	1	0	2	0	0.720	0	1	0	1
59	10	0	0	0		2	0	1	0	0		2	0	2	0
60	10	0	1	0		2	0	0	0	0		2	0	2	0
61	10	0	1	0	0.117	1	0	0	0	0	0.231	1	0	1	0
62	10	0	0	0	1.045	0	1	0	0	0	0.000	1	0	1	0
63	10	1	2	0		1	0	0	2	1	0.264	1	0	1	0
64	13	0	2	0	0.910	1	1	0	3	0	0.806	1	1	2	0
65	10	0	1	0	0.456	1	0	1	1	0	0.467	1	0	1	0
66	10	0	0	0		1	0	0	1	0		1	0	1	0
67	10	2	1	0	0.334	1	0	1	2	0	0.508	1	0	1	0
68	10	0	0	0	0.307	1	0	0	0	0		1	0	1	0
69	10	1	0	0	0.556	1	0	1	0	0	0.802	1	0	1	0
70	13	0	1	0		1	0	0	1	0		1	0	1	0
71	10	3	0	1	0.371	2	0	1	1	2	0.469	2	0	2	0
71	15	1	2	0	0.371	2	1	0	3	0	0.469	3	0	3	0
72	10	1	0	0	0.000	1	0	1	0	0	0.000	1	0	1	0
73	10	1	4	1		1	0	1	2	1		1	0	1	0
74	10	0	0	0		0	1	0	0	0		1	0	1	0
75	8	1	0	0	0.675	0	1	1	0	0	0.782	1	0	1	0
75	10	1	0	0	0.675	1	0	1	1	0	0.782	1	0	1	0
76	10	0	0	0	0.481	1	0	0	0	0	0.589	1	0	1	0
77	7	2	2	0	0.773	1	0	1	1	0	1.035	1	0	1	0
77	10	1	3	0	0.773	1	0	0	2	0	1.035	1	0	1	0
78	2	3	0	0		0	1	3	0	0		0	1	0	1
78	10	1	0	0		1	0	1	0	0		1	0	1	0
79	10	0	10	0	0.000	1	0	0	7	0	0.000	1	0	1	0
81	7	2	0	0	0.504	0	1	0	0	0	0.239	0	1	0	1
81	10	0	0	0	0.504	1	0	0	0	0	0.239	1	0	1	0
83	10	0	3	0	0.257	1	0	0	4	0	0.610	1	0	1	0
84	10	0	0	0	0.043	1	0	0	0	0	0.006	1	0	1	0
85	1	0	0	0		0	1	0	0	0		0	1	0	1
85	2	0	0	0		1	0	0	0	0		0	1	1	0
85	3	0	1	0		0	1	0	0	0		0	1	0	1
85	4	0	1	0		0	1	0	0	0		0	1	0	1
85	5	0	1	0		0	1	0	0	0		0	1	0	1
85	10	0	1	0		1	0	0	0	0		1	0	1	0
85	14	0	3	0		0	1	0	1	0		0	1	0	1
86	10	4	0	0		1	1	4	0	0	0.097	2	0	2	0

Table S5. AAindex values showing a significant (P <0.05) difference between C1 and C2.

All of the P10-P10' cleavage site sequences of C1 or C2 were aligned, and the frequency of each aar at each position was multiplied by each AAindex value (weighted average), and compared between C1 and C2. "C1", "C2", and "Ave." indicate the weighted averages of AAindex for the cleavage site sequences of C1 and C2, and for the standard (Sprot) aa composition, respectively. p, by Z-test for the equality of two proportions (binomial distribution).

Position	AAindex	attribute	*P*	C1	C2	Ave.
P9-P8	2D392	Radius of gyration.	0.0388	3.23	3.32	3.18
	2D324	Molecular weight (including implicit hydrogens) in atomic mass units with atomic weights taken from [CRC 1994].	0.0407	243	250	239
	2D391	Third diagonal element of diagonalized moment of inertia tensor.	0.0414	2,535	2,746	2,416
	2D312	Vertex adjacency information (magnitude): 1 + log2 m where m is the number of heavy-heavy bonds. If m is zero, then zero is returned.	0.0415	4.98	5.03	4.95
	2D333	x component of the principal moment of inertia (external coordinates).	0.0421	2,039	2,229	1,929
	2D326	Weiner polarity number	0.0437	20.28	21.02	20.07
	2D327	Zagreb index: the sum of di2 over all heavy atoms i.	0.0438	73.56	76.15	72.85
	2D188	Number of bonds between heavy atoms.	0.0444	16.10	16.66	15.91
	2D314	If m is the sum of the distance matrix entries then VDistMa is defined to be the sum of log2 m - Dij log2 Dij / m over all i and j.	0.0448	7.78	7.87	7.74
	2D388	Principal moment of inertia.	0.0448	2,720	2,935	2,595
	2D390	Second diagonal element of diagonalized moment of inertia tensor.	0.0454	2,225	2,418	2,108
	2D197	Atomic connectivity index (order 1) from [Hall 1991] and [Hall 1977]. This is calculated as the sum of 1/sqrt(didj) over all bonds between heavy atoms i and j where i < j.	0.0455	7.78	8.03	7.68
	2D456	Interaction field surface area	0.0460	393	402	390
	2D160	Number of heavy atoms #{Zi | Zi > 1}.	0.0462	16.79	17.30	16.57
	2D162	Atom information content (total). This is calculated to be a_ICM times n.	0.0464	57.50	59.14	56.26
	2D338	The electronic energy (kcal/mol) calculated using the AM1 Hamiltonian [MOPAC].	0.0469	-484,371	-504,011	-471,517
	2D198	Atomic valence connectivity index (order 1) from [Hall 1991] and [Hall 1977]. This is calculated as the sum of 1/sqrt(vivj) over all bonds between heavy atoms i and j where i < j.	0.0490	5.39	5.58	5.33
	2D457	Interaction field volume	0.0490	525	540	521
	2D374	The electronic energy (kcal/mol) calculated using the MNDO Hamiltonian [MOPAC].	0.0496	-473,847	-492,651	-461,507
	2D301	Molecular refractivity (including implicit hydrogens). This property is an atomic contribution model [Crippen 1999] that assumes the correct protonation state (washed structures). The model was trained on ~7000 structures and results may vary from the mr descriptor.	0.0497	5.55	5.75	5.49
	2D343	Water accessible surface area calculated using a radius of 1.4 A for the water molecule. A polyhedral representation is used for each atom in calculating the surface area.	0.0499	451	461	446
P7	1D074	Optical rotation*	0.0369	-7.89	-6.76	-6.89
P2	1D565	A, Ala	0.0134	0.07	0.06	0.09
	1D196	Normalized composition from fungi and plant	0.0251	0.14	0.17	0.05
	1D881	Standard dimension 3: the square root of the third largest eigenvalue of the covariance matrix of the atomic coordinates. A standard dimension is equivalent to the standard deviation along a principal component axis.	0.0272	0.86	0.87	0.84
	1D326	Relative preference value at N2	0.0291	0.80	0.79	0.98
	1D491	Hydrophobicity coefficient in RP-HPLC, C18 with 0.1%TFA/2-PrOH/MeCN/H2O	0.0319	0.96	1.08	0.96
	1D856	z component of the principal moment of inertia (external coordinates).	0.0376	52.76	53.75	52.68
	1D344	Information measure for pleated-sheet	0.0431	0.81	0.91	-0.29
	1D810	Normalized PMI ratio pmi2/pmi3.	0.0442	0.81	0.81	0.81
	1D343	Information measure for extended	0.0482	0.76	0.84	-0.08
	1D352	Hydration free energy	0.0493	0.57	0.61	0.52
P5'	1D839	Sum of vi where qi is in the range [-0.20,-0.15).	0.0260	0.57	0.50	0.82
	1D713	Number of sulfur atoms: #{Zi | Zi = 16}.	0.0279	0.03	0.02	0.04
	1D577	M, Met	0.0461	0.03	0.02	0.02

*optical rotation value for each aa is as follows:

P	H	F	W	T	C	L	M	Y	S	N	G	A	D	V	Q	E	I	R	K
-86	-39	-35	-34	-28	-17	-11	-10	-10	-7.5	-5.6	0.0	1.8	5.1	5.6	6.3	12	12	13	15

Table S6. Frequencies of aars that were significantly different between C1 and C2.

The P10-P10’ cleavage site sequences specific for C1 or C2 were aligned, and the frequency of each aar at each position was compared between C1 and C2 (see Figs. 3C-E). The significantly different aars are shown with their frequencies (%) and significance. *P*, *Z*-test for the equality of two proportions (binomial distribution).

position	P10	P9	P8	P7	P6	P2			P2'	P3'		P5'		P6'		P10'
aar	E	A	N	R	I	A	I	N	N	E	Q	D	M	R	T	A
C1 (%)	0.0	16.1	0.0	3.1	2.0	14.2	0.0	2.5	0.9	1.9	9.3	0.0	7.4	13.3	1.2	2.3
C2 (%)	17.1	2.3	5.9	11.1	8.9	0.0	11.1	9.5	7.8	10.3	0.0	5.7	0.0	2.0	10.0	21.4
P	0.0002	0.019	0.018	0.046	0.042	0.002	0.0002	0.037	0.013	0.016	0.016	0.019	0.043	0.028	0.018	0.008

Table S7. Summary of the peptides and cleavage sites identified in this study.

calpain	condition	4800			QSTAR Elite			Total identified		Total used*1		Number of distinct peptides identified*2				Number of cleavage sites identified*3			Number of cleavage sites identified with k_{cat}/K_m *4			
		total spectra	spectra identified	distinct peptide identified	total spectra	spectra identified	distinct peptide identified	spectra	distinct peptide	spectra	distinct peptide	BC-FL*5	BC-other	Rp	Nv	total	Rp	Nv	Rp	Rp, n>1	Nv	Nv, n>1
**1**	stringent	18,299	644	187	107,224	2,786	561	3,430	663	3,285	627	89	165	141	207	250	82	168	60	54	39	32
	normal		1,010	258		6,804	997	7,666	1,098	7,466	1,028	124	266	198	371	418	106	312	69	66	47	45
**2**	stringent	8,804	340	211	62,440	2,163	574	2,433	664	2,298	577	83	144	136	192	255	87	168	58	46	39	28
	normal		447	253		4,101	817	4,432	899	4,308	850	104	213	181	296	360	107	253	63	58	44	41
**1+2**	stringent	27,103	984	398	169,664	4,949	1,135	5,863	1,327	5,583	1,204	116	210	170	279	314	96	218	63	58	43	36
	normal		1,457	511		10,905	1,814	12,098	1,997	11,774	1,878	156	307	238	487	483	123	360	71	68	48	46

*1 Total number is the sum of the sites identified by 4800 and QSTAR Elite after eliminating shared and discordant peptides.

*2 Peptides with the same sequence but different modifications are identified as distinct peptides.

*3 Theoretically, the maximum number of possible sites is 1,652 (87 reported, and 1,565 novel sites); however, several other sites were identified from the cleavages of truncated synthetic peptides.

*4 Maximum number of sites is 80 and 50 for reported and novel sites, respectively, according to the number of reference peptides (see Table S3).

*5 Abbreviations used in this table: Rp, reported sites; Nv, novel sites; BC, both-side capped peptides; FL, full-length peptides.

Table S8 All calpain cleavage site sequences identified from fragment peptide spectra.

A. Normal condition

ID No.	SB No.	Protein	Site sequence, P19 to P19'[*2]		Site[*2]	Rp/Nv	Cleaved by[*3]			C1[*4]		C2		Ave.	C1+C2				C1				C2			
			19..15....10....5...1	1'..5'...10'...15'..19'			C1	C2	C1 or C2	k_{cat}/K_m	SE	k_{cat}/K_m	SE	k_{cat}/K_m	T	N	C	M	T	N	C	M	T	N	C	M
2	44	COPB1	--------GTYATQSALSS	SRPTKKEED----------	529	2	1	1	1	-	-	-	-		7	1	5	1	3	0	2	1	4	1	3	0
			---------GTYATQSALS	SSRPTKKEED---------	528	1	1	1	1	762.0	30.1	610.2	81.1	686.1	21	0	17	4	16	0	13	3	5	0	4	1
			----------------GTY	ATQSALSSSRPTKKEED--	521	2	1	1	1	-	-	-	-		7	0	2	5	5	0	1	4	2	0	1	1
4	29	TNNT3	-----EEEKPRPKLTAPKI	PEGEKV-------------	69	2	0	1	1			-	-		1	0	1	0	0	0	0	0	1	0	1	0
			---------EEEKPRPKLT	APKIPEGEKV---------	65	1	1	1	1	230.7	53.1	306.0	26.0	268.4	23	10	13	0	13	7	6	0	10	3	7	0
			-----------------EE	EKPRPKLTAPKIPEGEKV-	57	2	1	1	1	-	-	-	-		3	0	3	0	1	0	1	0	2	0	2	0
			-----------EKPRPKLT	APKIPEGEKV---------	65	1	1	0	1	-	-				1	1	0	0	1	1	0	0	0	0	0	0
5	45	MBP	--------LASASTMDHAR	HGF----------------	23	2	1	1	1	-	-	-	-		3	3	0	0	1	1	0	0	2	2	0	0
			----SQRSKYLASASTMDH	ARHGF--------------	21	2	1	0	1	-	-				2	0	2	0	2	0	2	0	0	0	0	0
			-----SQRSKYLASASTMD	HARHGF-------------	20	2	1	1	1	-	-	-	-		5	0	5	0	4	0	4	0	1	0	1	0
			------SQRSKYLASASTM	DHARHGF------------	19	2	1	1	1	-	-	-	-		2	0	2	0	1	0	1	0	1	0	1	0
			-------SQRSKYLASAST	MDHARHGF-----------	18	2	1	1	1	31.1	6.4	52.7	9.3	41.9	15	0	15	0	7	0	7	0	8	0	8	0
			--------SQRSKYLASAS	TMDHARHGF----------	17	2	1	0	1	-	-				3	1	2	0	3	1	2	0	0	0	0	0
			---------SQRSKYLASA	STMDHARHGF---------	16	1	1	1	1	57.4	11.7	113.6	13.0	85.5	36	0	36	0	20	0	20	0	16	0	16	0
			----------SQRSKYLAS	ASTMDHARHGF--------	15	2	1	1	1	-	-	-	-		6	0	6	0	5	0	5	0	1	0	1	0
			-----------SQRSKYLA	SASTMDHARHGF-------	14	2	1	1	1	-	-	-	-		14	0	14	0	9	0	9	0	5	0	5	0
			-------------SQRSKY	LASASTMDHARHGF-----	12	2	0	1	1			-	-		2	0	2	0	0	0	0	0	2	0	2	0
			------------------S	QRSKYLASASTMDHARHGF	7	2	1	1	1	-	-	-	-		4	0	4	0	1	0	1	0	3	0	3	0
6	45	MBP	PVVHFFKNIVTPRTPPPSQ	G------------------	102	2	1	1	1	-	-	-	-		3	0	0	3	2	0	0	2	1	0	0	1
			------PVVHFFKNIVTPR	TPPPSQG------------	96	1	1	1	1	497.7	8.3	651.1	25.1	574.4	248	207	36	5	174	148	22	4	74	59	14	1
			-------PVVHFFKNIVTP	RTPPPSQG-----------	95	2	1	1	1	-	-	-	-		3	0	3	0	2	0	2	0	1	0	1	0
			--------PVVHFFKNIVT	PRTPPPSQG----------	94	2	1	0	1	-	-				1	1	0	0	1	1	0	0	0	0	0	0
			---------PVVHFFKNIV	TPRTPPPSQG---------	93	1	1	1	1	174.8	8.6	263.1	22.4	219.0	92	72	17	3	59	50	7	2	33	22	10	1
			---------------PVVH	FFKNIVTPRTPPPSQG---	87	2	1	1	1	-	-	-	-		6	0	1	5	4	0	0	4	2	0	1	1
7	66	TNNT2	---VEDTKPKPSRLFMPNL	VPPK---------------	77	2	1	1	1	-	-	-	-		13	13	0	0	8	8	0	0	5	5	0	0
			----VEDTKPKPSRLFMPN	LVPPK--------------	76	2	1	1	1	443.8	-	452.2	-	448.0	3	3	0	0	2	2	0	0	1	1	0	0
			-------VEDTKPKPSRLF	MPNLVPPK-----------	73	2	1	1	1	296.4	122.9	170.6	12.5	233.5	7	7	0	0	2	2	0	0	5	5	0	0
8	55	PRKCA	---------KAKLGPAGNK	VISPSEDRKQ---------	316	1	1	1	1	13.2	1.5	20.2	5.8	16.7	14	0	14	0	10	0	10	0	4	0	4	0
			----------KAKLGPAGN	KVISPSEDRKQ--------	315	2	1	0	1	-	-				1	0	1	0	1	0	1	0	0	0	0	0
			--------------KAKLG	PAGNKVISPSEDRKQ----	311	2	1	1	1	-	-	-	-		3	0	3	0	2	0	2	0	1	0	1	0
			------------------K	AKLGPAGNKVISPSEDRKQ	307	2	1	1	1	-	-	-	-		2	0	2	0	1	0	1	0	1	0	1	0
9	89	PRKCG	YERVRMGPSSSPIPSPSPS	P------------------	330	2	1	1	1	-	-	-	-		4	4	0	0	3	3	0	0	1	1	0	0
			-------YERVRMGPSSSP	IPSPSPSP-----------	323	2	1	0	1	-	-				1	0	0	1	1	0	0	1	0	0	0	0
			---------YERVRMGPSS	SPIPSPSPSP---------	321	1	1	1	1	167.8	12.7	351.3	33.3	259.5	68	16	52	0	39	9	30	0	29	7	22	0
			----------YERVRMGPS	SSPIPSPSPSP--------	320	2	1	1	1	-	-	-	-		6	3	3	0	4	3	1	0	2	0	2	0
			-------------YERVRM	GPSSSPIPSPSPSP-----	317	2	1	0	1	-	-				1	0	0	1	1	0	0	1	0	0	0	0
			--------------YERVR	MGPSSSPIPSPSPSP----	316	2	1	1	1	-	-	-	-		5	0	5	0	3	0	3	0	2	0	2	0
10	39	PRKCB	RQKFERAKIGQGTKAPEEK	T------------------	320	1	1	0	1	-	-				1	0	0	1	1	0	0	1	0	0	0	0
			-----RQKFERAKIGQGTK	APEEKT-------------	315	2	0	1	1			-	-		2	0	2	0	0	0	0	0	2	0	2	0
			------RQKFERAKIGQGT	KAPEEKT------------	314	2	1	1	1	-	-	-	-		3	0	3	0	2	0	2	0	1	0	1	0
			-------RQKFERAKIGQG	TKAPEEKT-----------	313	2	1	1	1	-	-	-	-		4	0	4	0	2	0	2	0	2	0	2	0
			--------RQKFERAKIGQ	GTKAPEEKT----------	312	2	1	0	1	-	-				1	0	1	0	1	0	1	0	0	0	0	0
			---------RQKFERAKIG	QGTKAPEEKT---------	311	1	1	1	1	40.3	10.5	73.5	32.1	56.9	32	5	26	1	19	2	16	1	13	3	10	0
			-----------RQKFERAK	IGQGTKAPEEKT-------	309	2	1	0	1	-	-				1	0	1	0	1	0	1	0	0	0	0	0
			----------------RQK	FERAKIGQGTKAPEEKT--	304	2	1	0	1	-	-				2	0	2	0	2	0	2	0	0	0	0	0
11	8	PLCB1	----------PKPPSQALH	SQPAPGSVKA---------	880	1	0	1	1			-	-		1	1	0	0	0	0	0	0	1	1	0	0
			TPKPPSQALHSQPAPGSVK	A------------------	889	2	1	1	1	-	-	-	-		7	1	0	6	4	1	0	3	3	0	0	3
			-----TPKPPSQALHSQPA	PGSVKA-------------	884	2	1	1	1	-	-	-	-		4	0	4	0	2	0	2	0	2	0	2	0
			-------TPKPPSQALHSQ	PAPGSVKA-----------	882	2	1	1	1	-	-	-	-		2	0	2	0	1	0	1	0	1	0	1	0
			--------TPKPPSQALHS	QPAPGSVKA----------	881	2	1	1	1	-	-	-	-		11	1	10	0	8	1	7	0	3	0	3	0
			---------TPKPPSQALH	SQPAPGSVKA---------	880	1	1	1	1	457.2	56.4	713.3	81.8	585.3	102	33	63	6	58	20	35	3	44	13	28	3
			----------------TPK	PPSQALHSQPAPGSVKA--	873	2	1	0	1	-	-				6	0	6	0	6	0	6	0	0	0	0	0
12	65	PARP1	SAASAPAAVHSGPPDKPLS	N------------------	393	2	1	1	1	-	-	-	-		21	14	0	7	9	6	0	3	12	8	0	4
			---SAASAPAAVHSGPPDK	PLSN---------------	390	2	1	0	1	-	-				1	1	0	0	1	1	0	0	0	0	0	0
			----SAASAPAAVHSGPPD	KPLSN--------------	389	2	1	0	1	-	-				1	1	0	0	1	1	0	0	0	0	0	0
			---------SAASAPAAVH	SGPPDKPLSN---------	384	1	1	1	1	603.3	152.7	703.9	53.6	653.6	86	20	43	23	50	15	23	12	36	5	20	11
			--------------SAASA	PAAVHSGPPDKPLSN----	379	2	1	1	1	-	-	-	-		16	0	15	1	8	0	8	0	8	0	7	1
			---------------SAAS	APAAVHSGPPDKPLSN---	378	2	1	0	1	-	-				2	0	2	0	2	0	2	0	0	0	0	0
			----------------SAA	SAPAAVHSGPPDKPLSN--	377	2	1	1	1	-	-	-	-		25	0	10	15	15	0	8	7	10	0	2	8
			-----------------SA	ASAPAAVHSGPPDKPLSN-	376	2	1	0	1	-	-				3	0	1	2	3	0	1	2	0	0	0	0
			------------------S	AASAPAAVHSGPPDKPLSN	375	2	0	1	1			-	-		1	0	1	0	0	0	0	0	1	0	1	0
13	65	PARP1	----DEEAVKKLTVNPGTK	SKLP---------------	664	2	1	1	1	-	-	-	-		2	2	0	0	1	1	0	0	1	1	0	0
			-----DEEAVKKLTVNPGT	KSKLP--------------	663	2	1	1	1	-	-	-	-		7	7	0	0	5	5	0	0	2	2	0	0
			------EAVKKLTVNPGTK	SKLP---------------	664	2	1	0	1	-	-				1	1	0	0	1	1	0	0	0	0	0	0
			------EEAVKKLTVNPGT	KSKLP--------------	663	2	1	0	1	-	-				1	1	0	0	1	1	0	0	0	0	0	0
			---QDEEAVKKLTVNPGTK	SKLP---------------	664	2	1	1	1	-	-	-	-		9	6	0	3	5	4	0	1	4	2	0	2
			----QDEEAVKKLTVNPGT	KSKLP--------------	663	2	1	1	1	903.1	69.5	818.6	37.0	860.8	20	13	7	0	18	11	7	0	2	2	0	0
			---------QDEEAVKKLT	VNPGTKSKLP---------	658	1	1	1	1	38.9	-	76.1	16.7	57.5	3	0	3	0	1	0	1	0	2	0	2	0
			----------------QDE	EAVKKLTVNPGTKSKLP--	651	2	1	1	1	-	-	-	-		3	0	0	3	1	0	0	1	2	0	0	2
14	60	INS	-------HLCGSHLVEALY	LVCGERG------------	40	1	1	0	1	-	-				1	1	0	0	1	1	0	0	0	0	0	0
			--------LCGSHLVEALY	LVCGERG------------	40	1	1	0	1	-	-				5	5	0	0	5	5	0	0	0	0	0	0
			------QHLCGSHLVEALY	LVCGERG------------	40	1	1	0	1	237.9	12.3			237.9	66	18	48	0	66	18	48	0	0	0	0	0
			--------QHLCGSHLVEA	LYLVCGERG----------	38	2	1	0	1	-	-				1	0	1	0	1	0	1	0	0	0	0	0
15	41	GNRH1	-------EGCSSQHWSYGL	RPGGKRNT-----------	30	2	1	1	1	13.4	3.1	-	-	13.4	4	0	4	0	3	0	3	0	1	0	1	0
			--------EGCSSQHWSYG	LRPGGKRNT----------	29	1	0	1	1			-	-		1	0	1	0	0	0	0	0	1	0	1	0
			---------EGCSSQHWSY	GLRPGGKRNT---------	28	1	1	1	1	195.1	24.2	318.8	105.6	256.9	16	0	16	0	11	0	11	0	5	0	5	0
			----------------EGC	SSQHWSYGLRPGGKRNT--	21	2	1	0	1	-	-				3	0	3	0	3	0	3	0	0	0	0	0
16	27	AGT	---LTAGDRVYIHPFHLLY	YSK----------------	36	2	0	1	1			-	-		1	1	0	0	0	0	0	0	1	1	0	0
			--SLTAGDRVYIHPFHLLY	YSK----------------	36	2	1	1	1	-	-	-	-		31	30	0	1	11	10	0	1	20	20	0	0
			---SLTAGDRVYIHPFHLL	YYSK---------------	35	2	0	1	1			-	-		1	1	0	0	0	0	0	0	1	1	0	0
			-----SLTAGDRVYIHPFH	LLYYSK-------------	33	2	0	1	1			-	-		2	2	0	0	0	0	0	0	2	2	0	0
			----------SLTAGDRVY	IHPFHLLYYSK--------	28	1	1	0	1	-	-				2	1	0	1	2	1	0	1	0	0	0	0
			---------VYIHPFHLLY	YSK----------------	36	2	1	0	1	-	-				10	10	0	0	10	10	0	0	0	0	0	0
			------------VYIHPFH	LLYYSK-------------	33	2	1	0	1	-	-				1	1	0	0	1	1	0	0	0	0	0	0
17	34	NTS	--------IPYILKRQLYE	NKPRRPYIL----------	154	2	1	1	1	63.9	6.7	51.0	8.5	57.4	37	20	17	0	22	10	12	0	15	10	5	0
			---------IPYILKRQLY	ENKPRRPYIL---------	153	1	1	1	1	374.4	10.1	580.3	28.6	477.3	159	106	53	0	97	67	30	0	62	39	23	0
			----------IPYILKRQL	YENKPRRPYIL--------	152	2	0	1	1			-	-		1	1	0	0	0	0	0	0	1	1	0	0
			-------------IPYILK	RQLYENKPRRPYIL-----	149	2	1	1	1	-	-	-	-		15	7	5	3	9	3	5	1	6	4	0	2
			------------------I	PYILKRQLYENKPRRPYIL	144	2	1	1	1	-	-	-	-		3	0	0	3	1	0	0	1	2	0	0	2
18	49	PDYN	--LYKRYGGFLRRIRPKLK	WDN----------------	214	1	1	1	1	-	-	-	-		10	0	0	10	6	0	0	6	4	0	0	4
			---------LYKRYGGFLR	RIRPKLKWDN---------	207	1	1	1	1	716.3	41.2	1,340.0	69.2	1,028.2	46	24	9	13	33	18	7	8	13	6	2	5
			--------------LYKRY	GGFLRRIRPKLKWDN----	202	2	1	1	1	-	-	-	-		2	0	0	2	1	0	0	1	1	0	0	1
			-----------------LY	KRYGGFLRRIRPKLKWDN-	199	2	1	0	1	-	-				1	0	0	1	1	0	0	1	0	0	0	0
19	54	GCG	----YLDSRRAQDFVQWLM	NTKRN--------------	79	1	1	1	1	729.4	36.2	-	-	729.4	21	15	6	0	16	10	6	0	5	5	0	0
			------YLDSRRAQDFVQW	LMNTKRN------------	77	1	1	1	1	74.4	4.4	244.6	81.3	159.5	13	1	12	0	5	0	5	0	8	1	7	0
			-------YLDSRRAQDFVQ	WLMNTKRN-----------	76	2	0	1	1			-	-		3	2	1	0	0	0	0	0	3	2	1	0
			---------YLDSRRAQDF	VQWLMNTKRN---------	74	1	1	0	1	14.0	-			14.0	2	1	1	0	2	1	1	0	0	0	0	0
20	54	GCG	------RAQDFVQWLMNTK	RNRNNIA------------	82	2	1	1	1	-	-	-	-		6	0	6	0	5	0	5	0	1	0	1	0
			-------RAQDFVQWLMNT	KRNRNNIA-----------	81	2	1	1	1	28.8	2.2	44.3	3.7	36.6	15	0	15	0	10	0	10	0	5	0	5	0
			--------RAQDFVQWLMN	TKRNRNNIA----------	80	2	1	1	1	60.3	4.6	106.6	39.2	83.4	14	0	14	0	10	0	10	0	4	0	4	0
			---------RAQDFVQWLM	NTKRNRNNIA---------	79	1	1	1	1	414.8	28.1	256.9	33.8	335.8	20	0	20	0	15	0	15	0	5	0	5	0
			-----------RAQDFVQW	LMNTKRNRNNIA-------	77	1	1	1	1	-	-	-	-		2	0	2	0	1	0	1	0	1	0	1	0
21	10	NEFM	VVAAKKSPVKATAPELKEE	E------------------	525	2	1	0	1	-	-				2	0	0	2	2	0	0	2	0	0	0	0
			--VVAAKKSPVKATAPELK	EEE----------------	523	2	1	1	1	-	-	-	-		7	3	0	4	4	2	0	2	3	1	0	2
			--------VVAAKKSPVKA	TAPELKEEE----------	517	2	1	1	1	-	-	-	-		3	0	3	0	1	0	1	0	2	0	2	0
			---------VVAAKKSPVK	ATAPELKEEE---------	516	1	1	1	1	480.0	33.0	550.7	88.8	515.4	86	38	31	17	59	28	18	13	27	10	13	4
			----------------VVA	AKKSPVKATAPELKEEE--	509	2	1	1	1	-	-	-	-		13	0	2	11	11	0	2	9	2	0	0	2
22	42	CASP9	----LTPVVLRPEIRKPEV	LRPET--------------	120	1	1	1	1	-	-	-	-		5	5	0	0	1	1	0	0	4	4	0	0
			-----LTPVVLRPEIRKPE	VLRPET-------------	119	2	1	0	1	-	-				2	2	0	0	2	2	0	0	0	0	0	0
			---------LTPVVLRPEI	RKPEVLRPET---------	115	1	1	1	1	37.0	6.2	37.7	4.1	37.4	14	0	14	0	10	0	10	0	4	0	4	0
			------------------L	TPVVLRPEIRKPEVLRPET	106	2	1	0	1	-	-				6	0	6	0	6	0	6	0	0	0	0	0
24	48	ANXA1	EEQEYVQTVKSSKGGPGSA	V------------------	35	2	1	0	1	-	-				1	0	0	1	1	0	0	1	0	0	0	0
			-------EEQEYVQTVKSS	KGGPGSAV-----------	28	2	1	1	1	-	-	-	-		3	0	3	0	1	0	1	0	2	0	2	0

Table S8 All calpain cleavage site sequences identified from fragment peptide spectra.

A. Normal condition

ID No.	SB No.	Protein	Site sequence, P19 to P19'[*2]		Site[*2]	Rp/Nv	Cleaved by[*3] C1	C2	C1 or C2	C1[*4] k_{cat}/K_m	SE	C2 k_{cat}/K_m	SE	Ave. k_{cat}/K_m	C1+C2 T	N	C	M	C1 T	N	C	M	C2 T	N	C	M
			1 1 1 / 9...5....0....5...1	1'..5'...1 0'...1 5'..1 9'																						
			--------EEQEYVQTVKS	SKGGPGSAV----------	27	2	1	1	1	-	-	-	-		4	0	4	0	3	0	3	0	1	0	1	0
			---------EEQEYVQTVK	SSKGGPGSAV---------	26	1	1	1	1	533.7	42.2	478.8	37.7	506.3	74	42	31	1	46	24	21	1	28	18	10	0
			------------EEQEYVQ	TVKSSKGGPGSAV------	23	2	1	0	1	-	-				2	0	2	0	2	0	2	0	0	0	0	0
			----------EQEYVQTVK	SSKGGPGSAV---------	26	1	1	1	1	-	-	-	-		6	6	0	0	2	2	0	0	4	4	0	0
			------------EYVQTVK	SSKGGPGSAV---------	26	1	0	1	1			-	-		1	1	0	0	0	0	0	0	1	1	0	0
25	31	ACTB	---AVFPSIVGRPRHQGVM	VGM----------------	44	2	1	0	1	-	-				1	1	0	0	1	1	0	0	0	0	0	0
			--RAVFPSIVGRPRHQGVM	VGM----------------	44	2	1	1	1	-	-	-	-		6	6	0	0	5	5	0	0	1	1	0	0
			----RAVFPSIVGRPRHQG	VMVGM--------------	42	2	1	0	1	-	-				1	0	1	0	1	0	1	0	0	0	0	0
			-----RAVFPSIVGRPRHQ	GVMVGM-------------	41	2	1	1	1	-	-	-	-		9	0	9	0	5	0	5	0	4	0	4	0
			------RAVFPSIVGRPRH	QGVMVGM------------	40	2	1	1	1	-	-	-	-		16	0	16	0	11	0	11	0	5	0	5	0
			-------RAVFPSIVGRPR	HQGVMVGM-----------	39	2	1	0	1	-	-				2	2	0	0	2	2	0	0	0	0	0	0
			----------RAVFPSIVG	RPRHQGVMVGM--------	36	2	1	1	1	-	-	-	-		6	2	4	0	5	2	3	0	1	0	1	0
26	46	MIP	----------ISERLSVLK	GAKPDVSNGQ---------	238	1	1	1	1	-	-	-	-		7	7	0	0	3	3	0	0	4	4	0	0
			-----------SERLSVLK	GAKPDVSNGQ---------	238	1	1	1	1	-	-	-	-		2	2	0	0	1	1	0	0	1	1	0	0
			---------SISERLSVLK	GAKPDVSNGQ---------	238	1	1	1	1	508.1	18.7	730.9	27.0	619.5	175	152	17	6	99	90	8	1	76	62	9	5
			------------SISERLS	VLKGAKPDVSNGQ------	235	2	0	1	1			-	-		1	0	1	0	0	0	0	0	1	0	1	0
			-----------------SI	SERLSVLKGAKPDVSNGQ-	230	2	1	1	1	-	-	-	-		4	0	0	4	1	0	0	1	3	0	0	3
			------------------S	ISERLSVLKGAKPDVSNGQ	229	2	0	1	1			-	-		2	0	0	2	0	0	0	0	2	0	0	2
28	83	CDKN2D	-DVQEVRRLLHRELVHPDA	LN-----------------	37	2	0	1	1			-	-		1	0	0	1	0	0	0	0	1	0	0	1
			------DVQEVRRLLHREL	VHPDALN------------	32	2	1	1	1	64.0	15.3	167.9	37.3	116.0	9	0	9	0	4	0	4	0	5	0	5	0
			---------DVQEVRRLLH	RELVHPDALN---------	29	1	1	1	1	576.0	36.9	756.1	81.2	666.1	140	88	51	1	97	60	37	0	43	28	14	1
29	83	CDKN2D	AIALELLKQGASPNVQDTS	G------------------	73	2	1	0	1	-	-				1	1	0	0	1	1	0	0	0	0	0	0
			---AIALELLKQGASPNVQ	DTSG---------------	70	2	1	1	1	-	-	-	-		3	3	0	0	1	1	0	0	2	2	0	0
			-----AIALELLKQGASPN	VQDTSG-------------	68	2	1	1	1	-	-	-	-		2	2	0	0	1	1	0	0	1	1	0	0
			------AIALELLKQGASP	NVQDTSG------------	67	2	1	0	1	-	-				1	0	1	0	1	0	1	0	0	0	0	0
			-------AIALELLKQGAS	PNVQDTSG-----------	66	2	1	1	1	-	-	-	-		5	5	0	0	3	3	0	0	2	2	0	0
			--------AIALELLKQGA	SPNVQDTSG----------	65	2	1	1	1	-	-	-	-		4	4	0	0	2	2	0	0	2	2	0	0
			---------AIALELLKQG	ASPNVQDTSG---------	64	1	1	1	1	731.0	22.5	615.3	26.8	673.2	241	194	47	0	147	116	31	0	94	78	16	0
			----------AIALELLKQ	GASPNVQDTSG--------	63	2	1	1	1	-	-	-	-		5	5	0	0	4	4	0	0	1	1	0	0
			-----------AIALELLK	QGASPNVQDTSG-------	62	2	1	1	1	-	-	-	-		42	37	5	0	22	19	3	0	20	18	2	0
			------------AIALELL	KQGASPNVQDTSG------	61	2	1	1	1	-	-	-	-		4	0	4	0	2	0	2	0	2	0	2	0
			-------------AIALEL	LKQGASPNVQDTSG-----	60	2	1	0	1	-	-				1	0	1	0	1	0	1	0	0	0	0	0
			--------------AIALE	LLKQGASPNVQDTSG----	59	2	1	1	1	-	-	-	-		4	0	4	0	2	0	2	0	2	0	2	0
			------------------A	IALELLKQGASPNVQDTSG	55	2	1	1	1	-	-	-	-		7	0	7	0	4	0	4	0	3	0	3	0
			----------IALELLKQG	ASPNVQDTSG---------	64	1	1	1	1	-	-	-	-		9	9	0	0	5	5	0	0	4	4	0	0
30	37	VIM	-------YRRMFGGSGTSS	RPSSNRSY-----------	22	2	1	0	1	-	-				2	0	2	0	2	0	2	0	0	0	0	0
			--------YRRMFGGSGTS	SRPSSNRSY----------	21	2	1	1	1	-	-	-	-		18	1	17	0	13	1	12	0	5	0	5	0
			---------YRRMFGGSGT	SSRPSSNRSY---------	20	1	1	1	1	-	-	-	-		8	0	8	0	6	0	6	0	2	0	2	0
			----------YRRMFGGSG	TSSRPSSNRSY--------	19	2	1	1	1	-	-	-	-		28	0	28	0	11	0	11	0	17	0	17	0
			------------YRRMFGG	SGTSSRPSSNRSY------	17	2	1	0	1	-	-				1	0	1	0	1	0	1	0	0	0	0	0
			-------------YRRMFG	GSGTSSRPSSNRSY-----	16	2	1	1	1	-	-	-	-		2	0	2	0	1	0	1	0	1	0	1	0
			--------------YRRMF	GGSGTSSRPSSNRSY----	15	2	1	1	1	-	-	-	-		5	0	5	0	2	0	2	0	3	0	3	0
31	37	VIM	-----------RSSAVRLR	SSVPGVRLL----------	71	2	1	1	1	-	-	-	-		2	2	0	0	1	1	0	0	1	1	0	0
			------SAVRLRSSVPGVR	LL-----------------	78	2	1	1	1	-	-	-	-		6	6	0	0	4	4	0	0	2	2	0	0
			-----SSAVRLRSSVPGVR	LL-----------------	78	2	1	1	1	-	-	-	-		5	5	0	0	3	3	0	0	2	2	0	0
			------------SSAVRLR	SSVPGVRLL----------	71	2	1	1	1	-	-	-	-		12	12	0	0	7	7	0	0	5	5	0	0
			----------TRSSAVRLR	SSVPGVRLL----------	71	2	1	1	1	-	-	-	-		7	7	0	0	3	3	0	0	4	4	0	0
			---------VTRSSAVRLR	SSVPGVRLL----------	71	2	1	1	1	-	-	-	-		7	7	0	0	5	5	0	0	2	2	0	0
			----------VTRSSAVRL	RSSVPGVRLL---------	70	1	1	1	1	-	-	-	-		6	6	0	0	5	5	0	0	1	1	0	0
			-YVTRSSAVRLRSSVPGVR	LL-----------------	78	2	1	1	1	-	-	-	-		4	0	0	4	1	0	0	1	3	0	0	3
			--YVTRSSAVRLRSSVPGV	RLL----------------	77	2	0	1	1			-	-		1	0	0	1	0	0	0	0	1	0	0	1
			-----YVTRSSAVRLRSSV	PGVRLL-------------	74	2	1	1	1	-	-	-	-		44	0	44	0	32	0	32	0	12	0	12	0
			-------YVTRSSAVRLRS	SVPGVRLL-----------	72	2	1	1	1	159.7	18.6	196.2	15.8	178.0	50	0	49	1	29	0	28	1	21	0	21	0
			--------YVTRSSAVRLR	SSVPGVRLL----------	71	2	1	1	1	886.4	39.8	1,197.5	52.8	1,041.9	329	27	260	42	211	19	166	26	118	8	94	16
			---------YVTRSSAVRL	RSSVPGVRLL---------	70	1	1	1	1	81.8	5.9	103.1	14.4	92.5	137	37	100	0	89	18	71	0	48	19	29	0
			-------------YVTRSS	AVRLRSSVPGVRLL-----	66	2	1	1	1	108.6	25.1	93.3	7.4	101.0	13	0	10	3	10	0	8	2	3	0	2	1
			--------------YVTRS	SAVRLRSSVPGVRLL----	65	2	1	1	1	-	-	-	-		3	0	2	1	2	0	2	0	1	0	0	1
			---------------YVTR	SSAVRLRSSVPGVRLL---	64	2	1	1	1	100.3	58.9	47.4	-	73.9	30	0	8	22	21	0	7	14	9	0	1	8
			----------------YVT	RSSAVRLRSSVPGVRLL--	63	1	1	1	1	-	-	-	-		14	0	0	14	10	0	0	10	4	0	0	4
32	37	VIM	--------IDVDVSKPDLT	AALRDVRQ-----------	266	2	1	1	1	-	-	-	-		4	4	0	0	2	2	0	0	2	2	0	0
			---QIDVDVSKPDLTAALR	DVRQ---------------	270	2	1	1	1	-	-	-	-		18	18	0	0	5	5	0	0	13	13	0	0
			------QIDVDVSKPDLTA	ALRDVRQ------------	267	2	1	1	1	207.6	32.5	269.7	46.3	238.6	37	1	36	0	22	0	22	0	15	1	14	0
			-------QIDVDVSKPDLT	AALRDVRQ-----------	266	2	1	1	1	622.8	36.7	668.4	55.2	645.6	133	20	113	0	99	12	87	0	34	8	26	0
			--------QIDVDVSKPDL	TAALRDVRQ----------	265	2	1	1	1	58.3	10.8	110.2	35.4	84.2	21	5	16	0	11	3	8	0	10	2	8	0
			---------QIDVDVSKPD	LTAALRDVRQ---------	264	1	1	1	1	20.2	3.7	15.3	13.3	17.7	15	0	15	0	13	0	13	0	2	0	2	0
			-----------QIDVDVSK	PDLTAALRDVRQ-------	262	2	1	0	1	-	-				1	0	1	0	1	0	1	0	0	0	0	0
33	59	TH	-----AVSELDAKQAEAIM	SP-----------------	30	1	0	1	1			-	-		1	1	0	0	0	0	0	0	1	1	0	0
			-GFRRAVSELDAKQAEAIM	SP-----------------	30	1	0	1	1			-	-		1	0	0	1	0	0	0	0	1	0	0	1
			----GFRRAVSELDAKQAE	AIMSP--------------	27	2	1	0	1	-	-				2	0	2	0	2	0	2	0	0	0	0	0
			------GFRRAVSELDAKQ	AEAIMSP------------	25	2	1	0	1	-	-				1	0	1	0	1	0	1	0	0	0	0	0
			---------GFRRAVSELD	AKQAEAIMSP---------	22	1	1	1	1	61.0	15.1	51.2	-	56.1	9	0	8	1	6	0	6	0	3	0	2	1
			------------GFRRAVS	ELDAKQAEAIMSP------	19	2	0	1	1			-	-		1	1	0	0	0	0	0	0	1	1	0	0
34	81	TLN1	--------VSPKKSTVLQQ	QYNRVGKVE----------	434	2	0	1	1			46.2	-	46.2	1	0	1	0	0	0	0	0	1	0	1	0
			---------VSPKKSTVLQ	QQYNRVGKVE---------	433	1	1	1	1	362.6	42.8	476.1	61.3	419.3	28	8	20	0	19	7	12	0	9	1	8	0
35	78	SPTB	--------AFEKSTASWAE	RFAAL--------------	2063	2	1	0	1	-	-				1	1	0	0	1	1	0	0	0	0	0	0
			----------AFEKSTASW	AERFAAL------------	2061	1	1	0	1	-	-				1	1	0	0	1	1	0	0	0	0	0	0
			-------EAFEKSTASWAE	RFAAL--------------	2063	2	1	0	1	-	-				3	3	0	0	3	3	0	0	0	0	0	0
			---------EAFEKSTASW	AERFAAL------------	2061	1	0	1	1			-	-		2	2	0	0	0	0	0	0	2	2	0	0
			--KRHEAFEKSTASWAERF	AAL----------------	2065	2	1	0	1	-	-				3	0	0	3	3	0	0	3	0	0	0	0
			------KRHEAFEKSTASW	AERFAAL------------	2061	1	1	1	1	125.7	5.0	209.7	12.4	167.7	49	0	48	1	30	0	29	1	19	0	19	0
			--------KRHEAFEKSTA	SWAERFAAL----------	2059	2	1	1	1	-	-	-	-		2	0	2	0	1	0	1	0	1	0	1	0
			---------KRHEAFEKST	ASWAERFAAL---------	2058	1	1	1	1	16.4	2.8	16.6	5.4	16.5	22	0	22	0	14	0	14	0	8	0	8	0
			----------KRHEAFEKS	TASWAERFAAL--------	2057	2	1	1	1	-	-	-	-		6	0	6	0	3	0	3	0	3	0	3	0
			-----------KRHEAFEK	STASWAERFAAL-------	2056	2	0	1	1			-	-		3	0	3	0	0	0	0	0	3	0	3	0
			-----------------KR	HEAFEKSTASWAERFAAL-	2050	2	1	0	1	-	-				2	0	0	2	2	0	0	2	0	0	0	0
36	61	SPTAN1	--------VQAVQQQEVYG	MMPRDETDS----------	1177	2	1	1	1	-	-	-	-		2	0	2	0	1	0	1	0	1	0	1	0
			---------VQAVQQQEVY	GMMPRDETDS---------	1176	1	1	1	1	608.9	-	-	-	608.9	6	5	1	0	3	2	1	0	3	3	0	0
			--------VYGMMPRDETD	S------------------	1185	2	1	0	1	-	-				2	2	0	0	2	2	0	0	0	0	0	0
37	15	RYR1	--------FKAKKAAMMTQ	PPATPALPR----------	1401	2	1	1	1	-	-	-	-		16	0	16	0	9	0	9	0	7	0	7	0
			---------FKAKKAAMMT	QPPATPALPR---------	1400	1	1	1	1	738.9	24.8	1,070.5	57.5	904.7	162	7	155	0	119	7	112	0	43	0	43	0
			----------FKAKKAAMM	TQPPATPALPR--------	1399	2	1	1	1	193.1	65.3	390.0	106.3	291.5	9	0	9	0	6	0	6	0	3	0	3	0
38	15	RYR1	-----------TRKISQTA	QTYDPRE------------	2846	2	1	1	1	83.9	-	221.9	28.3	152.9	4	0	4	0	1	0	1	0	3	0	3	0
			------------TRKISQT	AQTYDPRE-----------	2845	2	1	1	1	-	-	-	-		3	0	3	0	1	0	1	0	2	0	2	0
			-------------TRKISQ	TAQTYDPRE----------	2844	2	1	0	1	-	-				1	0	1	0	1	0	1	0	0	0	0	0
			--------------TRKIS	QTAQTYDPRE---------	2843	1	1	1	1	37.0	20.7	29.8	5.5	33.4	8	0	8	0	3	0	3	0	5	0	5	0
			-----------------TR	KISQTAQTYDPRE------	2840	2	1	1	1	-	-	-	-		2	0	2	0	1	0	1	0	1	0	1	0
			------------------T	RKISQTAQTYDPRE-----	2839	2	1	1	1	-	-	-	-		2	0	2	0	1	0	1	0	1	0	1	0
39	72	GRIN2A	--QDWSQNNALQFQKNKLR	INR----------------	1285	2	1	1	1	-	-	-	-		3	1	0	2	2	1	0	1	1	0	0	1
			-------QDWSQNNALQFQ	KNKLRINR-----------	1280	2	1	0	1	-	-				1	0	1	0	1	0	1	0	0	0	0	0
			--------QDWSQNNALQF	QKNKLRINR----------	1279	2	1	1	1	68.6	2.9	90.4	2.4	79.5	12	0	11	1	7	0	7	0	5	0	4	1
			---------QDWSQNNALQ	FQKNKLRINR---------	1278	1	1	1	1	398.9	18.8	283.9	28.5	341.4	16	0	15	1	12	0	11	1	4	0	4	0
			----------QDWSQNNAL	QFQKNKLRINR--------	1277	2	1	1	1	-	-	-	-		5	0	5	0	4	0	4	0	1	0	1	0
40	72	GRIN2A	---------LEGNLYGSLF	SVPSSKLLGN---------	1329	1	1	0	1	1,707.1	274.2			1,707.1	9	1	8	0	9	1	8	0	0	0	0	0
41	91	PRDX2	------PAGWKPGSDTIKP	NVDDSKE------------	185	2	0	1	1			-	-		2	0	2	0	0	0	0	0	2	0	2	0
			---------PAGWKPGSDT	IKPNVDDSKE---------	182	1	1	0	1	35.7	23.9			35.7	3	0	3	0	3	0	3	0	0	0	0	0
			--------------PAGWK	PGSDTIKPNVDDSKE----	177	2	1	1	1	-	-	-	-		6	0	6	0	5	0	5	0	1	0	1	0
42	33	MYO5A	--------ITEAEDLPLRM	EEPSEKKAP----------	1141	2	0	1	1			-	-		3	2	1	0	0	0	0	0	3	2	1	0
			---------ITEAEDLPLR	MEEPSEKKAP---------	1140	1	1	1	1	294.8	7.9	-	-	294.8	13	11	2	0	10	8	2	0	3	3	0	0
44	38	RHOA	--------EMATRAALQAR	RGKKKSGC-----------	182	2	1	1	1	-	-	-	-		8	8	0	0	6	6	0	0	2	2	0	0
			---------EMATRAALQA	RRGKKKSGC----------	181	2	0	1	1			-	-		1	1	0	0	0	0	0	0	1	1	0	0
			-------FEMATRAALQAR	RGKKKSGC-----------	182	2	1	1	1	-	-	-	-		82	78	0	4	47	44	0	3	35	34	0	1
			--------FEMATRAALQA	RRGKKKSGC----------	181	2	1	1	1	-	-	-	-		26	24	0	2	13	12	0	1	13	12	0	1
			---------FEMATRAALQ	ARRGKKKSGC---------	180	1	1	1	1	-	-	-	-		24	15	0	9	18	12	0	6	6	3	0	3

Table S8 All calpain cleavage site sequences identified from fragment peptide spectra.

A. Normal condition

ID No.	SB No.	Protein	Site sequence, P19 to P19'[*2] (1 1 1 9...5....0....5...1)	(1' 1 1 1 ..5'...0'...5'..9')	Site[*2]	Rp/Nv	Cleaved by[*3] C1	C2	C1 or C2	C1[*4] k_{cat}/K_m	SE	C2 k_{cat}/K_m	SE	Ave. k_{cat}/K_m	C1+C2 T	N	C	M	C1 T	N	C	M	C2 T	N	C	M
			----------------FEM	ATRAALQARRGKKKSGC--	173	2	1	1	1	-	-	-	-		15	0	0	15	10	0	0	10	5	0	0	5
45	67	GAP43	-----AATKIQASFRGHIT	RKK----------------	47	2	1	1	1	-	-	-	-		3	3	0	0	2	2	0	0	1	1	0	0
			--AHKAATKIQASFRGHIT	RKK----------------	47	2	1	1	1	-	-	-	-		54	53	0	1	40	40	0	0	14	13	0	1
			-------AHKAATKIQASF	RGHITRKK-----------	42	2	1	1	1	161.2	12.4	156.5	26.7	158.8	13	13	0	0	9	9	0	0	4	4	0	0
			---------AHKAATKIQA	SFRGHITRKK---------	40	1	1	1	1	60.2	15.2	137.9	-	99.1	8	8	0	0	6	6	0	0	2	2	0	0
			----------AHKAATKIQ	ASFRGHITRKK--------	39	2	1	1	1	38.8	6.7	71.8	10.5	55.3	25	24	0	1	17	17	0	0	8	7	0	1
			--------HKAATKIQASF	RGHITRKK-----------	42	2	1	0	1	-	-				1	1	0	0	1	1	0	0	0	0	0	0
46	69	CRYBA1	---ETQTVQQELKSLPTTK	MAQT---------------	17	1	1	1	1	-	-	-	-		3	0	0	3	1	0	0	1	2	0	0	2
			---------ETQTVQQELK	SLPTTKMAQT---------	11	1	1	1	1	312.6	40.4	364.6	17.6	338.6	40	31	3	6	19	14	1	4	21	17	2	2
			----------------ETQ	TVQQELKSLPTTKMAQT--	4	2	1	0	1	-	-				3	0	0	3	3	0	0	3	0	0	0	0
			-----------QTVQQELK	SLPTTKMAQT---------	11	1	1	0	1	-	-				1	1	0	0	1	1	0	0	0	0	0	0
			----------TQTVQQELK	SLPTTKMAQT---------	11	1	1	0	1	-	-				3	3	0	0	3	3	0	0	0	0	0	0
			------TVQQELKSLPTTK	MAQT---------------	17	1	1	0	1	-	-				1	1	0	0	1	1	0	0	0	0	0	0
47	86	JUN	-LRAKNSDLLTSPDVGLLK	LA-----------------	70	2	1	1	1	-	-	-	-		18	0	0	18	9	0	0	9	9	0	0	9
			----LRAKNSDLLTSPDVG	LLKLA--------------	67	2	0	1	1			-	-		1	0	1	0	0	0	0	0	1	0	1	0
			-------LRAKNSDLLTSP	DVGLLKLA-----------	64	2	0	1	1			-	-		1	0	1	0	0	0	0	0	1	0	1	0
			---------LRAKNSDLLT	SPDVGLLKLA---------	62	1	1	1	1	101.3	4.8	323.1	8.5	212.2	167	5	152	10	78	2	71	5	89	3	81	5
			----------LRAKNSDLL	TSPDVGLLKLA--------	61	2	1	1	1	175.3	3.8	115.2	4.1	145.2	178	0	170	8	115	0	111	4	63	0	59	4
			------SDLLTSPDVGLLK	LA-----------------	70	2	1	0	1	-	-				1	1	0	0	1	1	0	0	0	0	0	0
48	86	JUN	---GGGGYSASLHSEPPVY	ANLS---------------	170	2	1	1	1	-	-	-	-		2	0	0	2	1	0	0	1	1	0	0	1
			----GGGGYSASLHSEPPV	YANLS--------------	169	2	1	0	1	-	-				1	0	0	1	1	0	0	1	0	0	0	0
			---------GGGGYSASLH	SEPPVYANLS---------	164	1	1	1	1	933.3	81.9	1,161.6	72.2	1,047.5	139	54	82	3	84	32	51	1	55	22	31	2
			-----------------GG	GGYSASLHSEPPVYANLS-	156	2	0	1	1			-	-		1	0	0	1	0	0	0	0	1	0	0	1
			------------------G	GGGYSASLHSEPPVYANLS	155	2	1	0	1	-	-				1	0	0	1	1	0	0	1	0	0	0	0
			----------GGGYSASLH	SEPPVYANLS---------	164	1	1	1	1	-	-	-	-		15	15	0	0	12	12	0	0	3	3	0	0
49	64	FOS	LVSSVAPSQTRAPHPYGLP	T------------------	99	2	1	0	1	-	-				1	1	0	0	1	1	0	0	0	0	0	0
			--LVSSVAPSQTRAPHPYG	LPT----------------	97	2	1	0	1	-	-				2	0	0	2	2	0	0	2	0	0	0	0
			--------LVSSVAPSQTR	APHPYGLPT----------	91	2	1	1	1	195.3	13.1	182.3	33.1	188.8	69	5	52	12	51	4	36	11	18	1	16	1
			---------LVSSVAPSQT	RAPHPYGLPT---------	90	1	1	1	1	369.8	19.1	736.0	57.0	552.9	212	1	205	6	131	1	125	5	81	0	80	1
			----------------LVS	SVAPSQTRAPHPYGLPT--	83	2	1	1	1	-	-	-	-		24	0	6	18	20	0	4	16	4	0	2	2
50	30	VWF	------SSPLSHRSKRSLS	CRPPMVK------------	766	2	1	1	1	-	-	-	-		6	5	0	1	4	3	0	1	2	2	0	0
			-------------SSPLSH	RSKRSLSCRPPMVK-----	759	2	1	0	1	-	-				1	0	0	1	1	0	0	1	0	0	0	0
52	32	HBB	---MVHLTPEEKSAVTALW	GKV----------------	16	2	1	1	1	833.0	208.4	1,324.3	339.3	1,078.7	5	5	0	0	3	3	0	0	2	2	0	0
			-----MVHLTPEEKSAVTA	LWGKV--------------	14	2	1	0	1	-	-				2	0	2	0	2	0	2	0	0	0	0	0
			------MVHLTPEEKSAVT	ALWGKV-------------	13	2	1	1	1	59.6	4.0	80.7	23.5	70.1	19	4	15	0	14	3	11	0	5	1	4	0
			----------MVHLTPEEK	SAVTALWGKV---------	9	1	1	1	1	30.0	3.8	54.3	7.2	42.2	72	0	72	0	38	0	38	0	34	0	34	0
			----------------MVH	LTPEEKSAVTALWGKV---	3	2	1	0	1	-	-				1	0	1	0	1	0	1	0	0	0	0	0
			--------PEEKSAVTALW	GKV----------------	16	2	1	1	1	-	-	-	-		9	9	0	0	5	5	0	0	4	4	0	0
53	19	IL1A	-----IKPRSAPFSFLSNV	KYNFMR-------------	122	2	1	0	1	-	-				1	0	1	0	1	0	1	0	0	0	0	0
			------IKPRSAPFSFLSN	VKYNFMR------------	121	2	1	1	1	-	-	-	-		6	0	6	0	3	0	3	0	3	0	3	0
			-------IKPRSAPFSFLS	NVKYNFMR-----------	120	2	1	1	1	349.2	28.3	384.2	31.5	366.7	55	10	45	0	35	5	30	0	20	5	15	0
			---------IKPRSAPFSF	LSNVKYNFMR---------	118	1	1	1	1	424.8	21.1	665.5	27.8	545.2	127	54	72	1	84	29	55	0	43	25	17	1
			-----------------IK	PRSAPFSFLSNVKYNFMR-	110	2	0	1	1			-	-		1	0	0	1	0	0	0	0	1	0	0	1
54	43	ITGB3	--------------PLYKE	ATSTFTNI-----------	775	2	1	1	1	-	-	-	-		2	0	2	0	1	0	1	0	1	0	1	0
			---------------PLYK	EATSTFTNI----------	774	2	1	1	1	-	-	-	-		12	0	12	0	4	0	4	0	8	0	8	0
			----------------PLY	KEATSTFTNI---------	773	1	1	1	1	46.1	5.4	50.6	7.1	48.4	8	0	8	0	4	0	4	0	4	0	4	0
55	62	ITGB7	-------NWKQDSNPLYKS	AITTTINP-----------	780	2	1	1	1	-	-	-	-		6	0	6	0	3	0	3	0	3	0	3	0
			--------NWKQDSNPLYK	SAITTTINP----------	779	2	1	1	1	-	-	-	-		21	0	21	0	13	0	13	0	8	0	8	0
			---------NWKQDSNPLY	KSAITTTINP---------	778	1	1	1	1	47.5	3.7	135.6	-	91.6	4	1	3	0	2	0	2	0	2	1	1	0
			-----------NWKQDSNP	LYKSAITTTINP-------	776	2	0	1	1			-	-		1	0	1	0	0	0	0	0	1	0	1	0
56	79	ITGB1	--ENPIYKSPINNFKNPNY	GRK----------------	795	1	0	1	1			-	-		1	1	0	0	0	0	0	0	1	1	0	0
			------ENPIYKSPINNFK	NPNYGRK------------	791	1	1	1	1	108.4	7.4	171.1	10.7	139.7	15	15	0	0	10	10	0	0	5	5	0	0
			-------ENPIYKSPINNF	KNPNYGRK-----------	790	1	1	1	1	-	-	-	-		6	6	0	0	1	1	0	0	5	5	0	0
			--------ENPIYKSPINN	FKNPNYGRK----------	789	1	1	1	1	-	-	-	-		13	13	0	0	3	3	0	0	10	10	0	0
			---------ENPIYKSPIN	NFKNPNYGRK---------	788	1	1	1	1	-	-	-	-		29	29	0	0	17	17	0	0	12	12	0	0
			--------NPIYKSPINNF	KNPNYGRK-----------	790	1	0	1	1			-	-		1	1	0	0	0	0	0	0	1	1	0	0
			--------PIYKSPINNFK	NPNYGRK------------	791	1	1	1	1	-	-	-	-		4	4	0	0	1	1	0	0	3	3	0	0
57	2	ITGB2	--------PLFKSATTTVM	NP-----------------	762	1	1	0	1	-	-				3	3	0	0	3	3	0	0	0	0	0	0
			-SQWNNDNPLFKSATTTVM	NP-----------------	762	1	0	1	1			-	-		3	3	0	0	0	0	0	0	3	3	0	0
			------SQWNNDNPLFKSA	TTTVMNP------------	757	2	1	0	1	-	-				2	0	2	0	2	0	2	0	0	0	0	0
			-------SQWNNDNPLFKS	ATTTVMNP-----------	756	2	1	1	1	-	-	-	-		14	0	14	0	9	0	9	0	5	0	5	0
			--------SQWNNDNPLFK	SATTTVMNP----------	755	2	1	1	1	-	-	-	-		8	0	8	0	6	0	6	0	2	0	2	0
			---------SQWNNDNPLF	KSATTTVMNP---------	754	1	0	1	1			21.5	3.0	21.5	2	0	2	0	0	0	0	0	2	0	2	0
58	63	ITGB1	------EFAKFEKEKMNAK	WDTGENP------------	774	2	1	0	1	-	-				1	0	1	0	1	0	1	0	0	0	0	0
			--------EFAKFEKEKMN	AKWDTGENP----------	772	2	1	1	1	-	-	-	-		2	0	2	0	1	0	1	0	1	0	1	0
			---------EFAKFEKEKM	NAKWDTGENP---------	771	1	0	1	1			42.9	-	42.9	1	0	1	0	0	0	0	0	1	0	1	0
			-------------EFAKFE	KEKMNAKWDTGENP-----	767	2	1	0	1	-	-				1	0	1	0	1	0	1	0	0	0	0	0
59	56	ATP2B2	--GQILWFRGLNRIQTQIR	VVK----------------	1142	2	1	1	1	-	-	-	-		11	0	0	11	6	0	0	6	5	0	0	5
			------GQILWFRGLNRIQ	TQIRVVK------------	1138	2	0	1	1			-	-		1	1	0	0	0	0	0	0	1	1	0	0
			---------GQILWFRGLN	RIQTQIRVVK---------	1135	1	1	1	1	-	-	-	-		65	54	0	11	41	35	0	6	24	19	0	5
			-----------ILWFRGLN	RIQTQIRVVK---------	1135	1	1	1	1	-	-	-	-		3	3	0	0	2	2	0	0	1	1	0	0
60	24	PTPRN	---------CVRQHARQQD	KERLAALGPE---------	608	1	1	1	1	417.4	16.8	574.6	60.0	496.0	36	0	33	3	24	0	23	1	12	0	10	2
			----------------CVR	QHARQQDKERLAALGPE--	601	2	1	0	1	-	-				1	0	0	1	1	0	0	1	0	0	0	0
			------------------C	VRQHARQQDKERLAALGPE	599	2	1	1	1	-	-	-	-		5	0	3	2	3	0	3	0	2	0	0	2
			-----VRQHARQQDKERLA	ALGPE--------------	613	2	0	1	1			-	-		1	1	0	0	0	0	0	0	1	1	0	0
			----------VRQHARQQD	KERLAALGPE---------	608	1	1	1	1	-	-	-	-		7	7	0	0	4	4	0	0	3	3	0	0
61	24	PTPRN	-----PEPSRVSSVSSQFS	DAAQAS-------------	662	2	1	0	1	-	-				1	0	1	0	1	0	1	0	0	0	0	0
			------PEPSRVSSVSSQF	SDAAQAS------------	661	2	1	1	1	-	-	-	-		9	0	9	0	6	0	6	0	3	0	3	0
			-------PEPSRVSSVSSQ	FSDAAQAS-----------	660	2	1	1	1	-	-	-	-		2	0	2	0	1	0	1	0	1	0	1	0
			--------PEPSRVSSVSS	QFSDAAQAS----------	659	2	0	1	1			-	-		3	0	3	0	0	0	0	0	3	0	3	0
			---------PEPSRVSSVS	SQFSDAAQAS---------	658	1	1	1	1	203.2	42.3	196.2	29.2	199.7	32	0	32	0	23	0	23	0	9	0	9	0
			------------PEPSRVS	SVSSQFSDAAQAS------	655	2	1	0	1	-	-				1	0	1	0	1	0	1	0	0	0	0	0
62	84	BID	---------GNRSSHSRLG	RIEADSESQE---------	70	1	0	1	1			-	-		2	1	1	0	0	0	0	0	2	1	1	0
63	26	BAX	-IMKTGALLLQGFIQDRAG	RM-----------------	36	2	1	0	1	-	-				2	0	0	2	2	0	0	2	0	0	0	0
			--IMKTGALLLQGFIQDRA	GRM----------------	35	2	1	1	1	-	-	-	-		3	0	0	3	1	0	0	1	2	0	0	2
			---IMKTGALLLQGFIQDR	AGRM---------------	34	2	1	1	1	-	-	-	-		6	0	0	6	4	0	0	4	2	0	0	2
			----IMKTGALLLQGFIQD	RAGRM--------------	33	2	1	0	1	-	-				1	0	1	0	1	0	1	0	0	0	0	0
			-------IMKTGALLLQGF	IQDRAGRM-----------	30	2	1	0	1	-	-				3	0	3	0	3	0	3	0	0	0	0	0
			--------IMKTGALLLQG	FIQDRAGRM----------	29	2	1	1	1	76.2	8.2	96.0	8.8	86.1	19	1	18	0	10	1	9	0	9	0	9	0
			---------IMKTGALLLQ	GFIQDRAGRM---------	28	1	1	1	1	624.8	18.0	685.9	40.1	655.4	255	133	110	12	155	69	78	8	100	64	32	4
			----------IMKTGALLL	QGFIQDRAGRM--------	27	2	1	1	1	72.1	8.1	95.7	11.0	83.9	32	2	30	0	17	1	16	0	15	1	14	0
			--------------IMKTG	ALLLQGFIQDRAGRM----	23	2	1	0	1	-	-				1	0	0	1	1	0	0	1	0	0	0	0
			----------------IMK	TGALLLQGFIQDRAGRM--	21	2	0	1	1			-	-		2	0	0	2	0	0	0	0	2	0	0	2
64	70	IGFBP4	-------DRSTSGGKMKVN	GAPREDA------------	159	1	1	0	1	-	-				1	1	0	0	1	1	0	0	0	0	0	0
			-----RDRSTSGGKMKVNG	APREDA-------------	160	2	0	1	1			-	-		1	0	1	0	0	0	0	0	1	0	1	0
			------RDRSTSGGKMKVN	GAPREDA------------	159	1	0	1	1			-	-		1	0	1	0	0	0	0	0	1	0	1	0
			---------RDRSTSGGKM	KVNGAPREDA---------	156	2	1	1	1	9.9	5.0	22.1	10.0	16.0	23	0	23	0	12	0	12	0	11	0	11	0
			-----------------RD	RSTSGGKMKVNGAPREDA-	148	2	0	1	1			-	-		1	0	1	0	0	0	0	0	1	0	1	0
65	40	HBA1	---------NVKAAWGKVG	AH-----------------	19	2	0	1	1			-	-		2	2	0	0	0	0	0	0	2	2	0	0
			----VLSPADKTNVKAAWG	KVGAH--------------	16	2	1	0	1	-	-				2	0	2	0	2	0	2	0	0	0	0	0
			-----VLSPADKTNVKAAW	GKVGAH-------------	15	2	1	0	1	-	-				2	0	2	0	2	0	2	0	0	0	0	0
			------VLSPADKTNVKAA	WGKVGAH------------	14	2	1	0	1	-	-				1	0	1	0	1	0	1	0	0	0	0	0
			-------VLSPADKTNVKA	AWGKVGAH-----------	13	2	1	0	1	-	-				1	0	1	0	1	0	1	0	0	0	0	0
			--------VLSPADKTNVK	AAWGKVGAH----------	12	2	1	1	1	93.7	15.6	134.8	16.7	114.3	31	2	29	0	21	0	21	0	10	2	8	0
			---------VLSPADKTNV	KAAWGKVGAH---------	11	1	1	1	1	22.6	4.6	-	-	22.6	27	0	27	0	18	0	18	0	9	0	9	0
			------------------V	LSPADKTNVKAAWGKVGAH	2	2	1	1	1	-	-	-	-		7	0	7	0	6	0	6	0	1	0	1	0
66	50	EGFR	-------VRKRTLRRLLQE	RELVEPLT-----------	685	2	1	1	1	-	-	-	-		3	0	3	0	2	0	2	0	1	0	1	0
			--------VRKRTLRRLLQ	ERELVEPLT----------	684	2	0	1	1			-	-		1	0	1	0	0	0	0	0	1	0	1	0
			---------VRKRTLRRLL	QERELVEPLT---------	683	1	1	1	1	630.9	34.3	1,245.6	112.0	938.2	91	23	68	0	57	19	38	0	34	4	30	0
			-------------VRKRTL	RRLLQERELVEPLT-----	679	2	0	1	1			-	-		1	0	1	0	0	0	0	0	1	0	1	0
67	50	EGFR	---VQPTCVNSTFDSPAHW	AQKG---------------	1157	2	1	0	1	-	-				1	1	0	0	1	1	0	0	0	0	0	0
			----VQPTCVNSTFDSPAH	WAQKG--------------	1156	2	1	1	1	95.9	33.1	-	-	95.9	7	3	4	0	5	1	4	0	2	2	0	0
			---------VQPTCVNSTF	DSPAHWAQKG---------	1151	1	1	1	1	310.1	88.5	291.0	16.2	300.6	96	0	96	0	59	0	59	0	37	0	37	0

Table S8 All calpain cleavage site sequences identified from fragment peptide spectra.

A. Normal condition

ID No.	SB No.	Protein	Site sequence, P19 to P19'*2		Site*2	Rp/Nv	Cleaved by*3			C1*4		C2		Ave.	C1+C2				C1				C2			
			1 1 1 9...5....0....5...1	1'..5'...0'...5'..9'			C1	C2	C1 or C2	k_{cat}/K_m	SE	k_{cat}/K_m	SE	k_{cat}/K_m	T	N	C	M	T	N	C	M	T	N	C	M
			-----------VQPTCVNS	TFDSPAHWAQKG-------	1149	2	0	1	1			-	-		1	1	0	0	0	0	0	0	1	1	0	0
			------------VQPTCVN	STFDSPAHWAQKG------	1148	2	1	1	1	-	-	-	-		4	0	4	0	3	0	3	0	1	0	1	0
			---------------VQPT	CVNSTFDSPAHWAQKG---	1145	2	0	1	1			-	-		1	0	1	0	0	0	0	0	1	0	1	0
68	73	DMD	----TVTTVTTREQILVKH	AQ-----------------	698	2	1	0	1	-	-				1	1	0	0	1	1	0	0	0	0	0	0
			-VMETVTTVTTREQILVKH	AQ-----------------	698	2	1	0	1	-	-				4	0	0	4	4	0	0	4	0	0	0	0
			-------VMETVTTVTTRE	QILVKHAQ-----------	692	2	1	1	1	34.5	5.8	47.8	7.5	41.2	16	0	15	1	9	0	8	1	7	0	7	0
			--------VMETVTTVTTR	EQILVKHAQ----------	691	2	1	0	1	-	-				5	0	5	0	5	0	5	0	0	0	0	0
			---------VMETVTTVTT	REQILVKHAQ---------	690	1	1	1	1	50.7	3.0	94.4	9.7	72.6	18	0	15	3	12	0	9	3	6	0	6	0
69	36	SNCA	-----VVHGVATVAEKTKE	QVTNVG-------------	61	2	1	0	1	-	-				1	0	1	0	1	0	1	0	0	0	0	0
			------VVHGVATVAEKTK	EQVTNVG------------	60	2	1	1	1	-	-	-	-		10	2	8	0	8	1	7	0	2	1	1	0
			---------VVHGVATVAE	KTKEQVTNVG---------	57	1	1	1	1	155.3	13.6	112.6	-	133.9	9	2	7	0	8	2	6	0	1	0	1	0
70	23	IGFBP5	-----AEAVKKDRRKKLTQ	SKFVGG-------------	162	2	0	1	1			-	-		1	0	1	0	0	0	0	0	1	0	1	0
			------AEAVKKDRRKKLT	QSKFVGG------------	161	1	1	1	1	557.8	57.7	569.9	85.8	563.9	36	6	30	0	24	5	19	0	12	1	11	0
			---------AEAVKKDRRK	KLTQSKFVGG---------	158	2	1	1	1	36.0	10.9	20.3	4.2	28.1	16	0	16	0	10	0	10	0	6	0	6	0
71	22	CTTN	------PVEAVTSKTSNIR	ANFEN--------------	351	1	1	1	1	-	-	-	-		4	4	0	0	3	3	0	0	1	1	0	0
			-----------PVEAVTSK	TSNIRANFEN---------	346	1	1	1	1	-	-	-	-		15	15	0	0	7	7	0	0	8	8	0	0
			--TVPVEAVTSKTSNIRAN	FEN----------------	353	2	1	0	1	-	-				1	1	0	0	1	1	0	0	0	0	0	0
			----TVPVEAVTSKTSNIR	ANFEN--------------	351	1	1	1	1	-	-	-	-		19	14	0	5	15	12	0	3	4	2	0	2
			-------TVPVEAVTSKTS	NIRANFEN-----------	348	2	1	1	1	-	-	-	-		5	2	2	1	3	2	1	0	2	0	1	1
			---------TVPVEAVTSK	TSNIRANFEN---------	346	1	1	1	1	66.9	4.4	448.0	330.3	257.5	38	26	8	4	19	14	3	2	19	12	5	2
			-----------------TV	PVEAVTSKTSNIRANFEN-	338	2	0	1	1			-	-		2	0	0	2	0	0	0	0	2	0	0	2
			------------------T	VPVEAVTSKTSNIRANFEN	337	2	1	1	1	-	-	-	-		5	0	1	4	1	0	0	1	4	0	1	3
			-----VPVEAVTSKTSNIR	ANFEN--------------	351	1	0	1	1			-	-		1	1	0	0	0	0	0	0	1	1	0	0
72	90	FLNA	-------LRSQQLAPQYTY	AQGGQQTW-----------	1755	2	1	1	1	-	-	-	-		12	3	9	0	8	2	6	0	4	1	3	0
			--------LRSQQLAPQYT	YAQGGQQTW----------	1754	2	1	1	1	-	-	-	-		2	0	2	0	1	0	1	0	1	0	1	0
			---------LRSQQLAPQY	TYAQGGQQTW---------	1753	1	1	1	1	199.9	50.4	182.3	14.3	191.1	32	11	21	0	25	8	17	0	7	3	4	0
73	53	TP53	-----ETFSDLWKLLPENN	VLSPL--------------	30	2	1	1	1	-	-	-	-		7	7	0	0	4	4	0	0	3	3	0	0
			----QETFSDLWKLLPENN	VLSPL--------------	30	2	1	1	1	-	-	-	-		14	5	7	2	10	3	6	1	4	2	1	1
			-----QETFSDLWKLLPEN	NVLSPL-------------	29	2	1	0	1	-	-				2	0	2	0	2	0	2	0	0	0	0	0
			---------QETFSDLWKL	LPENNVLSPL---------	25	1	1	1	1	11.4	1.7	13.7	4.6	12.5	20	0	20	0	12	0	12	0	8	0	8	0
			----------QETFSDLWK	LLPENNVLSPL--------	24	2	1	0	1	-	-				1	0	1	0	1	0	1	0	0	0	0	0
			-----------QETFSDLW	KLLPENNVLSPL-------	23	2	1	1	1	-	-	-	-		11	0	11	0	9	0	9	0	2	0	2	0
			---------------QETF	SDLWKLLPENNVLSPL---	19	2	1	1	1	-	-	-	-		8	0	6	2	3	0	2	1	5	0	4	1
74	16	GJA8	---------VGMVEASPLS	AKPFSQFEEK---------	300	1	0	1	1			-	-		1	1	0	0	0	0	0	0	1	1	0	0
75	95	CRYBB3	--------AAANKSHGGLG	GSYKVT-------------	21	2	1	1	1	-	-	-	-		3	3	0	0	2	2	0	0	1	1	0	0
			-----------AAANKSHG	GLGGSYKVT----------	18	2	1	0	1	-	-				1	1	0	0	1	1	0	0	0	0	0	0
			-----PEQAAANKSHGGLG	GSYKVT-------------	21	2	1	1	1	310.2	91.8	308.7	107.8	309.4	15	4	11	0	9	2	7	0	6	2	4	0
			--------PEQAAANKSHG	GLGGSYKVT----------	18	2	1	1	1	-	-	-	-		4	0	4	0	2	0	2	0	2	0	2	0
			---------PEQAAANKSH	GGLGGSYKVT---------	17	1	1	1	1	30.3	4.8	35.6	7.8	33.0	24	0	24	0	13	0	13	0	11	0	11	0
			-----------PEQAAANK	SHGGLGGSYKVT-------	15	1	0	1	1			-	-		3	2	1	0	0	0	0	0	3	2	1	0
76	88	CRYBB1	---------SVPRPAAKVG	ELPPGSYRLV---------	49	1	1	1	1	249.0	13.1	398.1	33.3	323.5	73	8	65	0	41	3	38	0	32	5	27	0
			-----------SVPRPAAK	VGELPPGSYRLV-------	47	2	1	0	1	-	-				1	1	0	0	1	1	0	0	0	0	0	0
77	94	CRYBB1	AKASATAAVNPGPDGKGKA	G------------------	24	2	1	0	1	-	-				1	1	0	0	1	1	0	0	0	0	0	0
			---------AKASATAAVN	PGPDGKGKAG---------	15	1	1	1	1	45.0	8.7	54.4	13.8	49.7	21	18	3	0	11	10	1	0	10	8	2	0
			------------AKASATA	AVNPGPDGKGKAG------	12	1	1	1	1	-	-	-	-		5	0	5	0	3	0	3	0	2	0	2	0
			---------------AKAS	ATAAVNPGPDGKGKAG---	9	2	1	0	1	-	-				1	0	1	0	1	0	1	0	0	0	0	0
			----------------AKA	SATAAVNPGPDGKGKAG--	8	2	1	1	1	-	-	-	-		3	0	3	0	2	0	2	0	1	0	1	0
78	25	SAG	--------VFEEFARQNLK	DAGEYKEEK----------	386	2	1	1	1	-	-	-	-		3	3	0	0	2	2	0	0	1	1	0	0
			---------VFEEFARQNL	KDAGEYKEEK---------	385	1	1	1	1	-	-	-	-		38	38	0	0	24	24	0	0	14	14	0	0
79	21	CAPN11	---------AARLQRDRLR	AEGVGEHNNA---------	17	1	1	1	1	84.1	9.5	132.8	18.7	108.5	35	1	34	0	23	1	22	0	12	0	12	0
			----------AARLQRDRL	RAEGVGEHNNA--------	16	2	1	0	1	-	-				1	0	1	0	1	0	1	0	0	0	0	0
			-----------AARLQRDR	LRAEGVGEHNNA-------	15	2	1	1	1	-	-	-	-		10	0	10	0	6	0	6	0	4	0	4	0
			------------AARLQRD	RLRAEGVGEHNNA------	14	2	1	1	1	-	-	-	-		14	0	14	0	9	0	9	0	5	0	5	0
			-------------AARLQR	DRLRAEGVGEHNNA-----	13	2	1	1	1	-	-	-	-		4	0	4	0	2	0	2	0	2	0	2	0
			--------------AARLQ	RDRLRAEGVGEHNNA----	12	2	1	1	1	-	-	-	-		18	0	18	0	14	0	14	0	4	0	4	0
			---------------AARL	QRDRLRAEGVGEHNNA---	11	2	1	0	1	-	-				2	0	2	0	2	0	2	0	0	0	0	0
81	17	CAPNS1	-----PPPPRSHVSMVDPN	ESEEVR-------------	39	2	1	1	1	-	-	-	-		5	0	5	0	4	0	4	0	1	0	1	0
			------PPPPRSHVSMVDP	NESEEVR------------	38	2	1	1	1	-	-	-	-		4	0	4	0	2	0	2	0	2	0	2	0
			-------PPPPRSHVSMVD	PNESEEVR-----------	37	2	1	1	1	-	-	-	-		2	0	2	0	1	0	1	0	1	0	1	0
			---------PPPPRSHVSM	VDPNESEEVR---------	35	1	1	1	1	45.7	10.9	54.1	25.9	49.9	7	0	7	0	4	0	4	0	3	0	3	0
			----------PPPPRSHVS	MVDPNESEEVR--------	34	2	1	0	1	-	-				9	9	0	0	9	9	0	0	0	0	0	0
83	20	CAPN1	----ITPVYCTGVSAQVQK	QRARE--------------	20	2	1	1	1	-	-	-	-		4	0	0	4	2	0	0	2	2	0	0	2
			-----ITPVYCTGVSAQVQ	KQRARE-------------	19	2	1	1	1	167.9	5.0	94.3	13.1	131.1	14	0	14	0	10	0	10	0	4	0	4	0
			-------ITPVYCTGVSAQ	VQKQRARE-----------	17	2	1	0	1	-	-				2	0	2	0	2	0	2	0	0	0	0	0
			--------ITPVYCTGVSA	QVQKQRARE----------	16	2	1	0	1	156.8	10.4			156.8	8	0	8	0	8	0	8	0	0	0	0	0
			---------ITPVYCTGVS	AQVQKQRARE---------	15	1	1	1	1	425.5	32.0	550.5	51.5	488.0	21	0	17	4	13	0	11	2	8	0	6	2
			----------ITPVYCTGV	SAQVQKQRARE--------	14	2	1	1	1	14.6	11.0	32.8	4.4	23.7	7	0	7	0	4	0	4	0	3	0	3	0
			-----------ITPVYCTG	VSAQVQKQRARE-------	13	2	0	1	1			-	-		1	0	1	0	0	0	0	0	1	0	1	0
			-------------ITPVYC	TGVSAQVQKQRARE-----	11	2	1	1	1	26.1	3.1	38.2	12.5	32.2	17	0	17	0	13	0	13	0	4	0	4	0
			-----------------IT	PVYCTGVSAQVQKQRARE-	7	2	0	1	1			-	-		1	0	1	0	0	0	0	0	1	0	1	0
84	20	CAPN1	-------VQKQRARELGLG	RHENAIKY-----------	29	2	0	1	1			-	-		2	0	2	0	0	0	0	0	2	0	2	0
			---------VQKQRARELG	LGRHENAIKY---------	27	1	1	1	1	794.3	25.6	1,136.3	44.7	965.3	147	11	136	0	100	7	93	0	47	4	43	0
85	1	CAPN2	---------KDREAAEGLG	SHERAIKYLN---------	19	1	1	1	1	62.1	7.2	90.4	2.9	76.3	7	0	7	0	4	0	4	0	3	0	3	0
			-----------------KD	REAAEGLGSHERAIKYLN-	11	1	1	0	1	-	-				1	0	1	0	1	0	1	0	0	0	0	0
86	11	NFKBIA	---EQMVKELQEIRLEPQE	VP-----------------	58	2	0	1	1			-	-		1	1	0	0	0	0	0	0	1	1	0	0
			-EYEQMVKELQEIRLEPQE	VP-----------------	58	2	1	1	1	214.9	25.1	192.9	27.7	203.9	36	19	0	17	22	13	0	9	14	6	0	8
			--EYEQMVKELQEIRLEPQ	EVP----------------	57	2	0	1	1			-	-		2	0	0	2	0	0	0	0	2	0	0	2
			------EYEQMVKELQEIR	LEPQEVP------------	53	2	1	1	1	241.3	10.9	239.4	31.4	240.3	88	51	34	3	61	36	23	2	27	15	11	1
			--------EYEQMVKELQE	IRLEPQEVP----------	51	2	0	1	1			-	-		1	1	0	0	0	0	0	0	1	1	0	0
			---------EYEQMVKELQ	EIRLEPQEVP---------	50	1	1	1	1	294.9	78.5	476.9	23.7	385.9	72	35	21	16	31	16	8	7	41	19	13	9
			-----MVKELQEIRLEPQE	VP-----------------	58	2	1	1	1	-	-	-	-		25	25	0	0	21	21	0	0	4	4	0	0
			----------MVKELQEIR	LEPQEVP------------	53	2	1	1	1	-	-	-	-		3	3	0	0	2	2	0	0	1	1	0	0
			------VKELQEIRLEPQE	VP-----------------	58	2	1	0	1	-	-				1	1	0	0	1	1	0	0	0	0	0	0
			-----------VKELQEIR	LEPQEVP------------	53	2	0	1	1			-	-		1	1	0	0	0	0	0	0	1	1	0	0
			-------YEQMVKELQEIR	LEPQEVP------------	53	2	1	1	1	-	-	-	-		3	3	0	0	2	2	0	0	1	1	0	0
			----------YEQMVKELQ	EIRLEPQEVP---------	50	1	0	1	1			-	-		1	1	0	0	0	0	0	0	1	1	0	0
87	37	VIMsc*7	LLLRRVVAGSPSVRSTSVR	Y------------------	79	2	0	1	1			-	-		1	0	0	1	0	0	0	0	1	0	0	1
			--LLLRRVVAGSPSVRSTS	VRY----------------	77	2	1	0	1	-	-				1	1	0	0	1	1	0	0	0	0	0	0
			---LLLRRVVAGSPSVRST	SVRY---------------	76	2	1	1	1	-	-	-	-		18	11	0	7	17	11	0	6	1	0	0	1
			-----LLLRRVVAGSPSVR	STSVRY-------------	74	2	1	1	1	277.4	31.4	507.9	88.7	392.6	210	144	43	23	136	93	27	16	74	51	16	7
			------LLLRRVVAGSPSV	RSTSVRY------------	73	2	1	1	1	-	-	-	-		23	19	4	0	19	16	3	0	4	3	1	0
			-------LLLRRVVAGSPS	VRSTSVRY-----------	72	2	1	0	1	-	-				2	2	0	0	2	2	0	0	0	0	0	0
			----------LLLRRVVAG	SPSVRSTSVRY--------	69	2	1	0	1	-	-				1	0	1	0	1	0	1	0	0	0	0	0
			-----------LLLRRVVA	GSPSVRSTSVRY-------	68	2	1	1	1	-	-	-	-		11	2	7	2	8	1	6	1	3	1	1	1
			--------------LLLRR	VVAGSPSVRSTSVRY----	65	2	1	0	1	-	-				1	0	1	0	1	0	1	0	0	0	0	0
			---------------LLLR	RVVAGSPSVRSTSVRY---	64	2	1	1	1	-	-	-	-		29	0	12	17	18	0	6	12	11	0	6	5
			----------------LLL	RRVVAGSPSVRSTSVRY--	63	2	1	0	1	-	-				1	0	0	1	1	0	0	1	0	0	0	0
			-----------------LL	LRRVVAGSPSVRSTSVRY-	62	2	1	0	1	-	-				2	0	1	1	2	0	1	1	0	0	0	0
			------------------L	LLRRVVAGSPSVRSTSVRY	61	2	1	1	1	-	-	-	-		9	0	1	8	8	0	1	7	1	0	0	1
			---LLRRVVAGSPSVRSTS	VRY----------------	77	2	1	0	1	-	-				2	2	0	0	2	2	0	0	0	0	0	0
			----LLRRVVAGSPSVRST	SVRY---------------	76	2	1	1	1	-	-	-	-		11	11	0	0	10	10	0	0	1	1	0	0
			------LLRRVVAGSPSVR	STSVRY-------------	74	2	1	1	1	-	-	-	-		73	73	0	0	43	43	0	0	30	30	0	0
			-----LRRVVAGSPSVRST	SVRY---------------	76	2	1	0	1	-	-				1	1	0	0	1	1	0	0	0	0	0	0
			-------LRRVVAGSPSVR	STSVRY-------------	74	2	1	1	1	-	-	-	-		29	29	0	0	17	17	0	0	12	12	0	0
			--------RRVVAGSPSVR	STSVRY-------------	74	2	1	0	1	-	-				1	1	0	0	1	1	0	0	0	0	0	0
88	81	TLN1sc	-----EQVLKVGTVSRKNK	YPQSQV-------------	437	2	1	0	1	-	-				2	0	2	0	2	0	2	0	0	0	0	0
			------EQVLKVGTVSRKN	KYPQSQV------------	436	2	1	0	1	-	-				2	1	1	0	2	1	1	0	0	0	0	0
			--------EQVLKVGTVSR	KNKYPQSQV----------	434	2	0	1	1			-	-		1	0	1	0	0	0	0	0	1	0	1	0
			---------EQVLKVGTVS	RKNKYPQSQV---------	433	2	1	1	1	-	-	-	-		10	3	7	0	6	1	5	0	4	2	2	0
			------------EQVLKVG	TVSRKNKYPQSQV------	430	2	1	1	1	-	-	-	-		6	0	6	0	2	0	2	0	4	0	4	0
			--------VLKVGTVSRKN	KYPQSQV------------	436	2	1	0	1	-	-				1	1	0	0	1	1	0	0	0	0	0	0
89	61	SPTAN1sc	----SYDVTEEQDQRQPVM	AMQGV--------------	1181	2	1	1	1	-	-	-	-		23	20	3	0	12	10	2	0	11	10	1	0
90	15	RYR1sc	-------RTPMLMAAPATK	AKPAPKQF-----------	1402	2	1	1	1	135.8	7.3	250.8	24.2	193.3	129	75	30	24	80	48	16	16	49	27	14	8

Table S8 All calpain cleavage site sequences identified from fragment peptide spectra.

A. Normal condition

ID No.	SB No.	Protein	Site sequence, P19 to P19'*2 (1 1 1 9...5....0....5...1)	(1' ..5'...0'...5'..9')	Site*2	Rp/Nv	Cleaved by*3 C1	C2	C1 or C2	C1*4 k_{cat}/K_m	SE	C2 k_{cat}/K_m	SE	Ave. k_{cat}/K_m	C1+C2 T	N	C	M	C1 T	N	C	M	C2 T	N	C	M
			--------RTPMLMAAPAT	KAKPAPKQF----------	1401	2	1	1	1	171.2	9.2	144.4	22.4	157.8	23	2	21	0	18	2	16	0	5	0	5	0
			------------RTPMLMA	APATKAKPAPKQF------	1397	2	1	1	1	-	-	-	-		3	0	3	0	1	0	1	0	2	0	2	0
			-------------RTPMLM	AAPATKAKPAPKQF-----	1396	2	1	1	1	809.2	45.6	1,302.5	129.9	1,055.9	101	0	82	19	64	0	52	12	37	0	30	7
			--------------RTPML	MAAPATKAKPAPKQF----	1395	2	1	1	1	-	-	-	-		6	0	4	2	2	0	1	1	4	0	3	1
			---------------RTPM	LMAAPATKAKPAPKQF---	1394	2	1	1	1	-	-	-	-		19	0	19	0	12	0	12	0	7	0	7	0
			----------------RTP	MLMAAPATKAKPAPKQF--	1393	2	1	1	1	-	-	-	-		4	0	1	3	3	0	0	3	1	0	1	0
			--------TPMLMAAPATK	AKPAPKQF-----------	1402	2	1	1	1	-	-	-	-		3	3	0	0	1	1	0	0	2	2	0	0
91	62	ITGB7sc	-TDIQAKSWKNPYNLIPTN	TS-----------------	786	2	1	0	1	-	-				1	1	0	0	1	1	0	0	0	0	0	0
			------TDIQAKSWKNPYN	LIPTNTS------------	781	2	1	1	1	916.5	87.0	1,107.2	59.4	1,011.8	119	37	82	0	68	20	48	0	51	17	34	0
			-------TDIQAKSWKNPY	NLIPTNTS-----------	780	2	1	1	1	862.7	303.2	1,951.3	798.3	1,407.0	15	12	3	0	7	7	0	0	8	5	3	0
			-----------TDIQAKSW	KNPYNLIPTNTS-------	776	2	0	1	1			-	-		2	0	2	0	0	0	0	0	2	0	2	0
92	90	FLNAsc	---------ASYRTLWYTQ	QPQAGL-------------	1765	2	1	1	1	-	-	-	-		3	3	0	0	1	1	0	0	2	2	0	0
			-----GQQQASYRTLWYTQ	QPQAGL-------------	1765	2	1	1	1	-	-	-	-		45	7	38	0	33	5	28	0	12	2	10	0
			------GQQQASYRTLWYT	QQPQAGL------------	1764	2	1	1	1	-	-	-	-		45	6	39	0	28	4	24	0	17	2	15	0
			-------GQQQASYRTLWY	TQQPQAGL-----------	1763	2	1	1	1	-	-	-	-		78	19	59	0	47	10	37	0	31	9	22	0
			--------GQQQASYRTLW	YTQQPQAGL----------	1762	2	1	1	1	-	-	-	-		41	12	29	0	27	6	21	0	14	6	8	0
			--------QASYRTLWYTQ	QPQAGL-------------	1765	2	1	1	1	-	-	-	-		6	6	0	0	4	4	0	0	2	2	0	0
			-------QQASYRTLWYTQ	QPQAGL-------------	1765	2	1	1	1	-	-	-	-		2	2	0	0	1	1	0	0	1	1	0	0
			---------QQASYRTLWY	TQQPQAGL-----------	1763	2	1	1	1	-	-	-	-		2	2	0	0	1	1	0	0	1	1	0	0
93	53	TP53sc	---------NSNFETPELQ	LLPKSWLLVD---------	25	2	1	1	1	-	-	-	-		5	0	5	0	3	0	3	0	2	0	2	0
			---------------NSNF	ETPELQLLPKSWLLVD---	19	2	1	0	1	-	-				1	0	1	0	1	0	1	0	0	0	0	0
94	20	CAPN1sc	-ERHQRKGQLVYGKLIEAR	NA-----------------	35	2	1	0	1	-	-				1	1	0	0	1	1	0	0	0	0	0	0
			----ERHQRKGQLVYGKLI	EARNA--------------	32	2	1	0	1	-	-				1	0	1	0	1	0	1	0	0	0	0	0
			-----ERHQRKGQLVYGKL	IEARNA-------------	31	2	1	0	1	-	-				1	0	1	0	1	0	1	0	0	0	0	0
			-------ERHQRKGQLVYG	KLIEARNA-----------	29	2	1	1	1	-	-	-	-		9	0	9	0	3	0	3	0	6	0	6	0
			--------ERHQRKGQLVY	GKLIEARNA----------	28	2	1	0	1	236.1	19.2			236.1	10	9	1	0	10	9	1	0	0	0	0	0
			---------ERHQRKGQLV	YGKLIEARNA---------	27	2	0	1	1			-	-		1	0	1	0	0	0	0	0	1	0	1	0
			----------ERHQRKGQL	VYGKLIEARNA--------	26	2	1	1	1	-	-	-	-		5	0	5	0	3	0	3	0	2	0	2	0
			-----------ERHQRKGQ	LVYGKLIEARNA-------	25	2	1	1	1	-	-	-	-		4	0	4	0	2	0	2	0	2	0	2	0
			------------------E	RHQRKGQLVYGKLIEARNA	18	2	1	0	1	-	-				1	0	1	0	1	0	1	0	0	0	0	0
			---------LVYGKLIEAR	NA-----------------	35	2	1	0	1	-	-				1	1	0	0	1	1	0	0	0	0	0	0

*1 Full-length peptides were not identified for P87 ID Nos. 38, 43, and 54, so the estimated longest sequence (38: TRKISQTAQTYDPRE, 43: LCIGNH, and 54: PLYKEATSTFTNI) was used to estimate the cleavage site sequences instead of the full-length sequences.

*2 Numbers indicate the positions of the P1 aar in the whole protein sequence.

*3 1: cleaved fragment detected; 0: cleaved fragment not detected.

*4 Abbreviations used in this table: Ave, the average of the k_{cat}/K_m values for C1 and C2; N, C, M, and T, number of spectra identified for N-terminal, C-terminal, or internal sites [*e.g.*, cleavage sites in a both-side cleaved (uncapped) peptide fragment], and sum of them all, respectively; for protein names, see Table S2.

Table S8 All calpain cleavage site sequences identified from fragment peptide spectra.

B. Stringent condition

			Site sequence, P19 to P19'[*1]			Cleaved by[*3]			C1[*4]		C2				C1+C2				C1				C2			
ID No.	SB No.	Protein	P19...P1	P1'...P19'	Site[*2]	C1	C2	C1 or C2	k_{cat}/K_m	SE	k_{cat}/K_m	SE	Ave.	Rp/Nv	T	N	C	M	T	N	C	M	T	N	C	M
2	44	COPB1	---------GTYATQSALS	SSRPTKKEED---------	528	1	1	1	779.0	34.4	746.7	50.3	762.9	Rp	13	0	13	0	9	0	9	0	4	0	4	0
			--------GTYATQSALSS	SRPTKKEED----------	529	0	1	1		-	-		-	Nv	1	0	1	0	0	0	0	0	1	0	1	0
4	29	TNNT3	-----------------EE	EKPRPKLTAPKIPEGEKV-	57	1	1	1	-		-		-	Nv	2	0	2	0	1	0	1	0	1	0	1	0
			---------EEEKPRPKLT	APKIPEGEKV---------	65	1	1	1	90.2	-	187.1	98.2	138.6	Rp	4	2	2	0	1	0	1	0	3	2	1	0
5	45	MBP	------------------S	QRSKYLASASTMDHARHGF	7	1	1	1	-		-		-	Nv	2	0	2	0	1	0	1	0	1	0	1	0
			-------------SQRSKY	LASASTMDHARHGF-----	12	0	1	1		-	-		-	Nv	1	0	1	0	0	0	0	0	1	0	1	0
			-----------SQRSKYLA	SASTMDHARHGF-------	14	1	1	1	-		-		-	Nv	10	0	10	0	5	0	5	0	5	0	5	0
			----------SQRSKYLAS	ASTMDHARHGF--------	15	1	1	1	-		-		-	Nv	3	0	3	0	2	0	2	0	1	0	1	0
			---------SQRSKYLASA	STMDHARHGF---------	16	1	1	1	43.1	4.7	97.3	10.7	70.2	Rp	25	0	25	0	14	0	14	0	11	0	11	0
			-------SQRSKYLASAST	MDHARHGF-----------	18	1	1	1	23.2	4.8	44.1	9.2	33.7	Nv	11	0	11	0	5	0	5	0	6	0	6	0
			------SQRSKYLASASTM	DHARHGF------------	19	0	1	1		-	-		-	Nv	1	0	1	0	0	0	0	0	1	0	1	0
			-----SQRSKYLASASTMD	HARHGF-------------	20	1	0	1	-			-	-	Nv	2	0	2	0	2	0	2	0	0	0	0	0
			--------LASASTMDHAR	HGF----------------	23	1	1	1	-		-		-	Nv	2	2	0	0	1	1	0	0	1	1	0	0
6	45	MBP	---------------PVVH	FFKNIVTPRTPPPSQG---	87	0	1	1		-	-		-	Nv	1	0	1	0	0	0	0	0	1	0	1	0
			---------PVVHFFKNIV	TPRTPPPSQG---------	93	1	1	1	148.5	16.1	244.1	36.0	196.3	Rp	39	26	12	1	17	13	4	0	22	13	8	1
			-------PVVHFFKNIVTP	RTPPPSQG-----------	95	0	1	1		-	-		-	Nv	1	0	1	0	0	0	0	0	1	0	1	0
			------PVVHFFKNIVTPR	TPPPSQG------------	96	1	1	1	508.8	10.8	584.9	27.3	546.9	Rp	126	122	4	0	88	86	2	0	38	36	2	0
			PVVHFFKNIVTPRTPPPSQ	G------------------	102	0	1	1		-	-		-	Nv	1	0	0	1	0	0	0	0	1	0	0	1
7	66	TNNT2	-------VEDTKPKPSRLF	MPNLVPPK-----------	73	0	1	1			168.8	-	168.8	Nv	1	1	0	0	0	0	0	0	1	1	0	0
			---VEDTKPKPSRLFMPNL	VPPK---------------	77	1	0	1	-			-	-	Nv	3	3	0	0	3	3	0	0	0	0	0	0
8	55	PRKCA	------------------K	AKLGPAGNKVISPSEDRKQ	307	1	0	1	-			-	-	Nv	1	0	1	0	1	0	1	0	0	0	0	0
			--------------KAKLG	PAGNKVISPSEDRKQ----	311	0	1	1		-	-		-	Nv	1	0	1	0	0	0	0	0	1	0	1	0
			---------KAKLGPAGNK	VISPSEDRKQ---------	316	1	1	1	14.2	2.0	18.0	6.6	16.1	Rp	5	0	5	0	2	0	2	0	3	0	3	0
9	89	PRKCG	--------------YERVR	MGPSSSPIPSPSPSP----	316	1	1	1	-		-		-	Nv	3	0	3	0	1	0	1	0	2	0	2	0
			----------YERVRMGPS	SSPIPSPSPSP--------	320	1	1	1	-		-		-	Nv	3	0	3	0	1	0	1	0	2	0	2	0
			---------YERVRMGPSS	SPIPSPSPSP---------	321	1	1	1	142.7	8.7	265.0	23.6	203.9	Rp	42	9	33	0	22	4	18	0	20	5	15	0
			YERVRMGPSSSPIPSPSPS	P------------------	330	1	1	1	-		-		-	Nv	2	2	0	0	1	1	0	0	1	1	0	0
10	39	PRKCB	----------------RQK	FERAKIGQGTKAPEEKT--	304	1	0	1	-			-	-	Nv	2	0	2	0	2	0	2	0	0	0	0	0
			---------RQKFERAKIG	QGTKAPEEKT---------	311	1	1	1	56.6	29.2	74.5	40.0	65.5	Rp	13	1	12	0	6	1	5	0	7	0	7	0
			-------RQKFERAKIGQG	TKAPEEKT-----------	313	0	1	1		-	-		-	Nv	2	0	2	0	0	0	0	0	2	0	2	0
			------RQKFERAKIGQGT	KAPEEKT------------	314	0	1	1		-	-		-	Nv	1	0	1	0	0	0	0	0	1	0	1	0
11	8	PLCB1	----------------TPK	PPSQALHSQPAPGSVKA--	873	1	0	1	-			-	-	Nv	5	0	5	0	5	0	5	0	0	0	0	0
			---------TPKPPSQALH	SQPAPGSVKA---------	880	1	1	1	567.7	103.8	533.2	39.3	550.4	Rp	49	10	36	3	26	8	18	0	23	2	18	3
			--------TPKPPSQALHS	QPAPGSVKA----------	881	1	1	1	-		-		-	Nv	7	0	7	0	4	0	4	0	3	0	3	0
			TPKPPSQALHSQPAPGSVK	A------------------	889	0	1	1		-	-		-	Nv	3	0	0	3	0	0	0	0	3	0	0	3
12	65	PARP1	------------------S	AASAPAAVHSGPPDKPLSN	375	0	1	1		-	-		-	Nv	1	0	1	0	0	0	0	0	1	0	1	0
			-----------------SA	ASAPAAVHSGPPDKPLSN-	376	1	0	1	-			-	-	Nv	2	0	1	1	2	0	1	1	0	0	0	0
			----------------SAA	SAPAAVHSGPPDKPLSN--	377	1	1	1	-		-		-	Nv	11	0	5	6	6	0	4	2	5	0	1	4
			---------------SAAS	APAAVHSGPPDKPLSN---	378	1	0	1	-			-	-	Nv	1	0	1	0	1	0	1	0	0	0	0	0
			--------------SAASA	PAAVHSGPPDKPLSN----	379	1	1	1	-		-		-	Nv	16	0	15	1	8	0	8	0	8	0	7	1
			---------SAASAPAAVH	SGPPDKPLSN---------	384	1	1	1	1,222.9	500.4	594.7	65.1	908.8	Rp	38	11	16	11	20	9	6	5	18	2	10	6
			----SAASAPAAVHSGPPD	KPLSN--------------	389	1	0	1	-			-	-	Nv	1	1	0	0	1	1	0	0	0	0	0	0
			---SAASAPAAVHSGPPDK	PLSN---------------	390	1	0	1	-			-	-	Nv	1	1	0	0	1	1	0	0	0	0	0	0
			SAASAPAAVHSGPPDKPLS	N------------------	393	1	1	1	-		-		-	Nv	13	8	0	5	5	3	0	2	8	5	0	3
13	65	PARP1	---------QDEEAVKKLT	VNPGTKSKLP---------	658	1	1	1	38.9	-	78.5	-	58.7	Rp	2	0	2	0	1	0	1	0	1	0	1	0
			----QDEEAVKKLTVNPGT	KSKLP--------------	663	1	1	1	774.0	58.2	722.1	-	748.1	Nv	5	5	0	0	4	4	0	0	1	1	0	0
			-----DEEAVKKLTVNPGT	KSKLP--------------	663	1	0	1	-			-	-	Nv	3	3	0	0	3	3	0	0	0	0	0	0
			----DEEAVKKLTVNPGTK	SKLP---------------	664	1	1	1	-		-		-	Nv	2	2	0	0	1	1	0	0	1	1	0	0
			---QDEEAVKKLTVNPGTK	SKLP---------------	664	1	0	1	-			-	-	Nv	3	3	0	0	3	3	0	0	0	0	0	0
14	60	INS	------QHLCGSHLVEALY	LVCGERG------------	40	1	0	1	234.6	21.6			234.6	Rp	13	2	11	0	13	2	11	0	0	0	0	0
			--------LCGSHLVEALY	LVCGERG------------	40	1	0	1	-			-	-	Rp	2	2	0	0	2	2	0	0	0	0	0	0
15	41	GNRH1	---------EGCSSQHWSY	GLRPGGKRNT---------	28	1	1	1	198.9	37.8	237.1	41.8	218.0	Rp	6	0	6	0	4	0	4	0	2	0	2	0
			-------EGCSSQHWSYGL	RPGGKRNT-----------	30	0	1	1			49.1	-	49.1	Nv	1	0	1	0	0	0	0	0	1	0	1	0
16	27	AGT	----------SLTAGDRVY	IHPFHLLYYSK--------	28	1	0	1	-			-	-	Rp	1	1	0	0	1	1	0	0	0	0	0	0
			-----SLTAGDRVYIHPFH	LLYYSK-------------	33	0	1	1		-	-		-	Nv	1	1	0	0	0	0	0	0	1	1	0	0
			--SLTAGDRVYIHPFHLLY	YSK----------------	36	1	1	1	-		-		-	Nv	14	14	0	0	3	3	0	0	11	11	0	0
			---------VYIHPFHLLY	YSK----------------	36	1	0	1	-			-	-	Nv	3	3	0	0	3	3	0	0	0	0	0	0
17	34	NTS	-------------IPYILK	RQLYENKPRRPYIL-----	149	1	0	1	-			-	-	Nv	4	0	4	0	4	0	4	0	0	0	0	0
			---------IPYILKRQLY	ENKPRRPYIL---------	153	1	1	1	532.5	21.6	521.0	48.2	526.8	Rp	58	23	35	0	34	10	24	0	24	13	11	0
			--------IPYILKRQLYE	NKPRRPYIL----------	154	1	1	1	64.2	7.1	32.8	7.0	48.5	Nv	11	5	6	0	7	2	5	0	4	3	1	0
18	49	PDYN	---------LYKRYGGFLR	RIRPKLKWDN---------	207	1	1	1	529.8	44.3	696.3	-	613.0	Rp	15	3	6	6	12	3	5	4	3	0	1	2
			--LYKRYGGFLRRIRPKLK	WDN----------------	214	1	1	1	-		-		-	Rp	6	0	0	6	4	0	0	4	2	0	0	2
19	54	GCG	-------YLDSRRAQDFVQ	WLMNTKRN-----------	76	0	1	1		-	-		-	Nv	1	1	0	0	0	0	0	0	1	1	0	0
			------YLDSRRAQDFVQW	LMNTKRN------------	77	1	1	1	59.6	0.0	126.1	-	92.9	Rp	3	0	3	0	2	0	2	0	1	0	1	0
			----YLDSRRAQDFVQWLM	NTKRN--------------	79	1	1	1	-		-		-	Rp	4	4	0	0	3	3	0	0	1	1	0	0
20	54	GCG	-----------RAQDFVQW	LMNTKRNRNNIA-------	77	1	0	1	-			-	-	Rp	1	0	1	0	1	0	1	0	0	0	0	0
			---------RAQDFVQWLM	NTKRNRNNIA---------	79	1	1	1	450.0	24.4	329.6	33.4	389.8	Rp	14	0	14	0	12	0	12	0	2	0	2	0
			--------RAQDFVQWLMN	TKRNRNNIA----------	80	1	1	1	52.7	3.5	108.3	17.7	80.5	Nv	5	0	5	0	3	0	3	0	2	0	2	0
			-------RAQDFVQWLMNT	KRNRNNIA-----------	81	1	1	1	24.8	2.7	50.6	3.0	37.7	Nv	7	0	7	0	4	0	4	0	3	0	3	0
21	10	NEFM	----------------VVA	AKKSPVKATAPELKEEE--	819	1	1	1	-		-		-	Nv	7	0	2	5	6	0	2	4	1	0	0	1
			---------VVAAKKSPVK	ATAPELKEEE---------	826	1	1	1	580.7	107.6	649.5	141.4	615.1	Rp	30	10	14	6	23	9	9	5	7	1	5	1
			--VVAAKKSPVKATAPELK	EEE----------------	833	1	1	1	-		-		-	Nv	3	2	0	1	2	1	0	1	1	1	0	0
22	42	CASP9	------------------L	TPVVLRPEIRKPEVLRPET	106	1	0	1	-			-	-	Nv	6	0	6	0	6	0	6	0	0	0	0	0
			---------LTPVVLRPEI	RKPEVLRPET---------	115	1	1	1	17.2	4.7	31.6	-	24.4	Rp	4	0	4	0	3	0	3	0	1	0	1	0
			-----LTPVVLRPEIRKPE	VLRPET-------------	119	1	0	1	-			-	-	Nv	1	1	0	0	1	1	0	0	0	0	0	0
			----LTPVVLRPEIRKPEV	LRPET--------------	120	1	0	1	-			-	-	Rp	1	1	0	0	1	1	0	0	0	0	0	0
24	48	ANXA1	------------EEQEYVQ	TVKSSKGGPGSAV------	23	1	0	1	-			-	-	Nv	1	0	1	0	1	0	1	0	0	0	0	0
			---------EEQEYVQTVK	SSKGGPGSAV---------	26	1	1	1	383.7	79.4	441.6	44.5	412.6	Rp	33	21	12	0	18	10	8	0	15	11	4	0
			----------EQEYVQTVK	SSKGGPGSAV---------	26	0	1	1		-	-		-	Rp	1	1	0	0	0	0	0	0	1	1	0	0
			-------EEQEYVQTVKSS	KGGPGSAV-----------	28	0	1	1		-	-		-	Nv	1	0	1	0	0	0	0	0	1	0	1	0
25	31	ACTB	----------RAVFPSIVG	RPRHQGVMVGM--------	36	1	0	1	-			-	-	Nv	3	0	3	0	3	0	3	0	0	0	0	0
			-------RAVFPSIVGRPR	HQGVMVGM-----------	39	1	0	1	-			-	-	Nv	1	1	0	0	1	1	0	0	0	0	0	0
			------RAVFPSIVGRPRH	QGVMVGM------------	40	0	1	1		-	-		-	Nv	1	0	1	0	0	0	0	0	1	0	1	0
			--RAVFPSIVGRPRHQGVM	VGM----------------	44	0	1	1		-	-		-	Nv	1	1	0	0	0	0	0	0	1	1	0	0
26	46	MIP	------------------S	ISERLSVLKGAKPDVSNGQ	229	0	1	1		-	-		-	Nv	1	0	0	1	0	0	0	0	1	0	0	1
			-----------------SI	SERLSVLKGAKPDVSNGQ-	230	1	0	1	-			-	-	Nv	1	0	0	1	1	0	0	1	0	0	0	0
			---------SISERLSVLK	GAKPDVSNGQ---------	238	1	1	1	550.8	28.1	677.4	37.9	614.1	Rp	55	47	6	2	29	25	3	1	26	22	3	1
			----------ISERLSVLK	GAKPDVSNGQ---------	238	0	1	1		-	-		-	Rp	2	2	0	0	0	0	0	0	2	2	0	0
28	83	CDKN2D	---------DVQEVRRLLH	RELVHPDALN---------	29	1	1	1	619.1	96.2	725.9	73.6	672.5	Rp	46	37	9	0	27	22	5	0	19	15	4	0
29	83	CDKN2D	------------------A	IALELLKQGASPNVQDTSG	55	1	1	1	-		-		-	Nv	2	0	2	0	1	0	1	0	1	0	1	0
			--------------AIALE	LLKQGASPNVQDTSG----	59	1	1	1	-		-		-	Nv	3	0	3	0	1	0	1	0	2	0	2	0
			------------AIALELL	KQGASPNVQDTSG------	61	1	1	1	-		-		-	Nv	2	0	2	0	1	0	1	0	1	0	1	0
			-----------AIALELLK	QGASPNVQDTSG-------	62	1	1	1	-		-		-	Nv	19	14	5	0	11	8	3	0	8	6	2	0
			----------AIALELLKQ	GASPNVQDTSG--------	63	1	1	1	-		-		-	Nv	4	4	0	0	2	2	0	0	2	2	0	0
			---------AIALELLKQG	ASPNVQDTSG---------	64	1	1	1	698.1	25.4	483.0	30.1	590.5	Rp	107	84	23	0	67	52	15	0	40	32	8	0
			----------IALELLKQG	ASPNVQDTSG---------	64	1	1	1	-		-		-	Rp	2	2	0	0	1	1	0	0	1	1	0	0
			-------AIALELLKQGAS	PNVQDTSG-----------	66	1	1	1	-		-		-	Nv	2	2	0	0	1	1	0	0	1	1	0	0
			-----AIALELLKQGASPN	VQDTSG-------------	68	1	1	1	-		-		-	Nv	2	2	0	0	1	1	0	0	1	1	0	0
			---AIALELLKQGASPNVQ	DTSG---------------	70	1	1	1	-		-		-	Nv	3	3	0	0	1	1	0	0	2	2	0	0
30	37	VIM	--------------YRRMF	GGSGTSSRPSSNRSY----	15	1	1	1	-		-		-	Nv	4	0	4	0	2	0	2	0	2	0	2	0
			-------------YRRMFG	GSGTSSRPSSNRSY-----	16	1	1	1	-		-		-	Nv	2	0	2	0	1	0	1	0	1	0	1	0
			----------YRRMFGGSG	TSSRPSSNRSY--------	19	1	1	1	-		-		-	Nv	15	0	15	0	7	0	7	0	8	0	8	0
			---------YRRMFGGSGT	SSRPSSNRSY---------	20	1	1	1	-		-		-	Rp	5	0	5	0	4	0	4	0	1	0	1	0
			--------YRRMFGGSGTS	SRPSSNRSY----------	21	1	1	1	-		-		-	Nv	4	0	4	0	1	0	1	0	3	0	3	0
31	37	VIM	----------------YVT	RSSAVRLRSSVPGVRLL--	63	1	1	1	-		-		-	Rp	6	0	0	6	3	0	0	3	3	0	0	3
			---------------YVTR	SSAVRLRSSVPGVRLL---	64	1	1	1	41.9	15.6	39.9	-	40.9	Nv	9	0	5	4	6	0	4	2	3	0	1	2
			-------------YVTRSS	AVRLRSSVPGVRLL-----	66	1	1	1	62.3	8.0	78.0	6.8	70.1	Nv	6	0	6	0	4	0	4	0	2	0	2	0
			---------YVTRSSAVRL	RSSVPGVRLL---------	70	1	1	1	72.2	6.5	100.3	21.8	86.2	Rp	78	5	73	0	49	2	47	0	29	3	26	0
			--------YVTRSSAVRLR	SSVPGVRLL----------	71	1	1	1	874.1	55.5	910.8	24.5	892.5	Nv	110	14	85	11	56	8	43	5	54	6	42	6
			------------SSAVRLR	SSVPGVRLL----------	71	1	1	1	-		-		-	Nv	4	4	0	0	2	2	0	0	2	2	0	0
			----------TRSSAVRLR	SSVPGVRLL----------	71	1	1	1	-		-		-	Nv	6	6	0	0	2	2	0	0	4	4	0	0
			---------VTRSSAVRLR	SSVPGVRLL----------	71	1	1	1	-		-		-	Nv	3	3	0	0	1	1	0	0	2	2	0	0

Table S8 All calpain cleavage site sequences identified from fragment peptide spectra.

B. Stringent condition

ID No.	SB No.	Protein	Site sequence, P19 to P19'[*1] (1 1 1 / 9...5....0....5...1 | 1'..5'...0'...5'..9' 1 1 1)	Site[*2]	Cleaved by[*3] C1	Cleaved by C2	Cleaved by C1 or C2	C1[*4] k_{cat}/K_m	C1 SE	C2 k_{cat}/K_m	C2 SE	Ave.	Rp/ Nv	C1+C2 T	C1+C2 N	C1+C2 C	C1+C2 M	C1 T	C1 N	C1 C	C1 M	C2 T	C2 N	C2 C	C2 M
			-------YVTRSSAVRLRSSVPGVRLL-----------	72	1	1	1	120.8	13.5	144.6	10.3	132.7	Nv	14	0	14	0	3	0	3	0	11	0	11	0
			-----YVTRSSAVRLRSSVPGVRLL-------------	74	1	1	1	-		-		-	Nv	7	0	7	0	5	0	5	0	2	0	2	0
			------SAVRLRSSVPGVRLL-----------------	78	1	1	1	-		-		-	Nv	5	5	0	0	3	3	0	0	2	2	0	0
			-----SSAVRLRSSVPGVRLL-----------------	78	1	1	1	-		-		-	Nv	4	4	0	0	2	2	0	0	2	2	0	0
			-YVTRSSAVRLRSSVPGVRLL-----------------	78	0	1	1		-	-		-	Nv	1	0	0	1	0	0	0	0	1	0	0	1
32	37	VIM	---------QIDVDVSKPDLTAALRDVRQ---------	264	1	0	1	17.5	6.2			17.5	Rp	5	0	5	0	5	0	5	0	0	0	0	0
			--------QIDVDVSKPDLTAALRDVRQ----------	265	1	1	1	31.6	0.2	83.4	49.6	57.5	Nv	7	2	5	0	4	2	2	0	3	0	3	0
			-------QIDVDVSKPDLTAALRDVRQ-----------	266	1	1	1	575.7	82.0	519.5	35.9	547.6	Nv	39	7	32	0	26	6	20	0	13	1	12	0
			--------IDVDVSKPDLTAALRDVRQ-----------	266	0	1	1		-	-		-	Nv	2	2	0	0	0	0	0	0	2	2	0	0
			------QIDVDVSKPDLTAALRDVRQ------------	267	1	1	1	252.1	82.2	238.3	82.1	245.2	Nv	10	1	9	0	7	0	7	0	3	1	2	0
			---QIDVDVSKPDLTAALRDVRQ---------------	270	1	1	1	-		-		-	Nv	9	9	0	0	1	1	0	0	8	8	0	0
34	81	TLN1	---------VSPKKSTVLQQQYNRVGKVE---------	433	1	1	1	236.2	3.6	476.4	54.6	356.3	Rp	10	2	8	0	4	2	2	0	6	0	6	0
35	78	SPTB	-----------KRHEAFEKSTASWAERFAAL-------	2056	1	1	1	-		-		-	Nv	3	0	3	0	1	0	1	0	2	0	2	0
			----------KRHEAFEKSTASWAERFAAL--------	2057	1	1	1	-		-		-	Nv	3	0	3	0	2	0	2	0	1	0	1	0
			---------KRHEAFEKSTASWAERFAAL---------	2058	1	1	1	13.1	5.6	11.1	2.4	12.1	Rp	11	0	11	0	5	0	5	0	6	0	6	0
			--------KRHEAFEKSTASWAERFAAL----------	2059	0	1	1		-	-		-	Nv	1	0	1	0	0	0	0	0	1	0	1	0
			------KRHEAFEKSTASWAERFAAL------------	2061	1	1	1	132.2	18.1	176.8	9.5	154.5	Rp	7	0	7	0	3	0	3	0	4	0	4	0
			---------EAFEKSTASWAERFAAL------------	2061	0	1	1		-	-		-	Rp	1	1	0	0	0	0	0	0	1	1	0	0
36	61	SPTAN1	---------VQAVQQQEVYGMMPRDETDS---------	1176	1	1	1	-		-		-	Rp	4	4	0	0	1	1	0	0	3	3	0	0
			--------VYGMMPRDETDS------------------	1185	1	0	1	-			-	-	Nv	2	2	0	0	2	2	0	0	0	0	0	0
37	15	RYR1	----------FKAKKAAMMTQPPATPALPR--------	1399	1	1	1	202.2	79.2	239.8	-	221.0	Nv	6	0	6	0	5	0	5	0	1	0	1	0
			---------FKAKKAAMMTQPPATPALPR---------	1400	1	1	1	796.2	46.4	933.0	49.2	864.6	Rp	53	1	52	0	35	1	34	0	18	0	18	0
			--------FKAKKAAMMTQPPATPALPR----------	1401	1	1	1	-		-		-	Nv	11	0	11	0	7	0	7	0	4	0	4	0
38	15	RYR1	-----------------TRKISQTAQTYDPRE------	2840	1	1	1	-		-		-	Nv	2	0	2	0	1	0	1	0	1	0	1	0
			--------------TRKISQTAQTYDPRE---------	2843	1	1	1	16.3	-	25.3	8.1	20.8	Rp	4	0	4	0	1	0	1	0	3	0	3	0
			-----------TRKISQTAQTYDPRE------------	2846	0	1	1			267.1	-	267.1	Nv	1	0	1	0	0	0	0	0	1	0	1	0
39	72	GRIN2A	----------QDWSQNNALQFQKNKLRINR--------	1277	1	1	1	-		-		-	Nv	2	0	2	0	1	0	1	0	1	0	1	0
			---------QDWSQNNALQFQKNKLRINR---------	1278	1	1	1	398.9	18.8	401.9	37.6	400.4	Rp	14	0	13	1	10	0	9	1	4	0	4	0
			--------QDWSQNNALQFQKNKLRINR----------	1279	1	1	1	68.6	2.9	104.1	9.4	86.4	Nv	11	0	11	0	7	0	7	0	4	0	4	0
			-------QDWSQNNALQFQKNKLRINR-----------	1280	1	0	1	-			-	-	Nv	1	0	1	0	1	0	1	0	0	0	0	0
			--QDWSQNNALQFQKNKLRINR----------------	1285	1	0	1	-			-	-	Nv	1	0	0	1	1	0	0	1	0	0	0	0
40	72	GRIN2A	---------LEGNLYGSLFSVPSSKLLGN---------	1329	1	0	1	1,831.6	373.5			1,831.6	Rp	5	0	5	0	5	0	5	0	0	0	0	0
41	91	PRDX2	--------------PAGWKPGSDTIKPNVDDSKE----	177	1	1	1	-		-		-	Nv	3	0	3	0	2	0	2	0	1	0	1	0
			---------PAGWKPGSDTIKPNVDDSKE---------	182	1	0	1	14.8	-			14.8	Rp	1	0	1	0	1	0	1	0	0	0	0	0
42	33	MYO5A	---------ITEAEDLPLRMEEPSEKKAP---------	1140	1	1	1	-		-		-	Rp	7	7	0	0	5	5	0	0	2	2	0	0
			--------ITEAEDLPLRMEEPSEKKAP----------	1141	0	1	1		-	-		-	Nv	3	2	1	0	0	0	0	0	3	2	1	0
44	38	RHOA	----------------FEMATRAALQARRGKKKSGC--	173	1	1	1	-		-		-	Nv	3	0	0	3	2	0	0	2	1	0	0	1
			---------FEMATRAALQARRGKKKSGC---------	180	1	1	1	-		-		-	Rp	13	13	0	0	10	10	0	0	3	3	0	0
			--------FEMATRAALQARRGKKKSGC----------	181	1	1	1	-		-		-	Nv	21	20	0	1	9	9	0	0	12	11	0	1
			--------EMATRAALQARRGKKKSGC-----------	182	1	1	1	-		-		-	Nv	7	7	0	0	5	5	0	0	2	2	0	0
			-------FEMATRAALQARRGKKKSGC-----------	182	1	1	1	-		-		-	Nv	64	62	0	2	34	32	0	2	30	30	0	0
45	67	GAP43	----------AHKAATKIQASFRGHITRKK--------	39	1	1	1	52.0	31.8	56.5	21.9	54.2	Nv	5	5	0	0	2	2	0	0	3	3	0	0
			-------AHKAATKIQASFRGHITRKK-----------	42	1	1	1	160.5	23.5	114.3	16.1	137.4	Nv	7	7	0	0	4	4	0	0	3	3	0	0
			-----AATKIQASFRGHITRKK----------------	47	1	0	1	-			-	-	Nv	1	1	0	0	1	1	0	0	0	0	0	0
			--AHKAATKIQASFRGHITRKK----------------	47	1	1	1	-		-		-	Nv	24	24	0	0	20	20	0	0	4	4	0	0
46	69	CRYBA1	---------ETQTVQQELKSLPTTKMAQT---------	11	1	1	1	330.0	68.7	341.3	37.0	335.6	Rp	15	15	0	0	6	6	0	0	9	9	0	0
47	86	JUN	----------LRAKNSDLLTSPDVGLLKLA--------	61	1	1	1	172.1	4.1	96.0	3.9	134.1	Nv	126	0	122	4	75	0	74	1	51	0	48	3
			---------LRAKNSDLLTSPDVGLLKLA---------	62	1	1	1	111.2	12.7	260.9	9.6	186.1	Rp	67	1	62	4	24	0	23	1	43	1	39	3
			-LRAKNSDLLTSPDVGLLKLA-----------------	70	1	1	1	-		-		-	Nv	8	0	0	8	2	0	0	2	6	0	0	6
			------SDLLTSPDVGLLKLA-----------------	70	1	0	1	-			-	-	Nv	1	1	0	0	1	1	0	0	0	0	0	0
48	86	JUN	---------GGGGYSASLHSEPPVYANLS---------	164	1	1	1	853.4	89.2	963.7	115.6	908.6	Rp	72	34	38	0	43	17	26	0	29	17	12	0
			----------GGGYSASLHSEPPVYANLS---------	164	1	1	1	-		-		-	Rp	9	9	0	0	7	7	0	0	2	2	0	0
49	64	FOS	----------------LVSSVAPSQTRAPHPYGLPT--	83	1	1	1	-		-		-	Nv	7	0	4	3	6	0	3	3	1	0	1	0
			---------LVSSVAPSQTRAPHPYGLPT---------	90	1	1	1	430.7	31.7	692.1	78.9	561.4	Rp	98	0	98	0	59	0	59	0	39	0	39	0
			--------LVSSVAPSQTRAPHPYGLPT----------	91	1	1	1	146.2	19.7	91.8	18.2	119.0	Nv	25	3	20	2	17	3	12	2	8	0	8	0
			--LVSSVAPSQTRAPHPYGLPT----------------	97	1	0	1	-			-	-	Nv	1	0	0	1	1	0	0	1	0	0	0	0
			LVSSVAPSQTRAPHPYGLPT------------------	99	1	0	1	-			-	-	Nv	1	1	0	0	1	1	0	0	0	0	0	0
50	30	VWF	------SSPLSHRSKRSLSCRPPMVK------------	766	1	1	1	-		-		-	Nv	2	2	0	0	1	1	0	0	1	1	0	0
52	32	HBB	----------MVHLTPEEKSAVTALWGKV---------	9	1	1	1	24.9	6.6	41.1	8.5	33.0	Rp	23	0	23	0	9	0	9	0	14	0	14	0
			------MVHLTPEEKSAVTALWGKV-------------	13	1	1	1	56.1	-	75.3	-	65.7	Nv	4	2	2	0	2	1	1	0	2	1	1	0
			---MVHLTPEEKSAVTALWGKV----------------	16	0	1	1			1,353.8	-	1,353.8	Nv	1	1	0	0	0	0	0	0	1	1	0	0
			--------PEEKSAVTALWGKV----------------	16	1	1	1	-		-		-	Nv	2	2	0	0	1	1	0	0	1	1	0	0
53	19	IL1A	---------IKPRSAPFSFLSNVKYNFMR---------	118	1	1	1	386.1	15.0	836.9	20.0	611.5	Rp	43	17	26	0	24	4	20	0	19	13	6	0
			-------IKPRSAPFSFLSNVKYNFMR-----------	120	1	1	1	361.0	74.0	282.0	32.5	321.5	Nv	9	0	9	0	6	0	6	0	3	0	3	0
54	43	ITGB3	------------------YKEATSTFTNI---------	773	0	1	1			28.9	-	28.9	Rp	1	0	1	0	0	0	0	0	1	0	1	0
			-----------------YKEATSTFTNI----------	774	1	1	1	-		-		-	Nv	7	0	7	0	2	0	2	0	5	0	5	0
55	62	ITGB7	------------------YKSAITTTINP---------	778	1	0	1	51.2	-			51.2	Rp	1	0	1	0	1	0	1	0	0	0	0	0
			-----------------YKSAITTTINP----------	779	1	1	1	-		-		-	Nv	9	0	9	0	5	0	5	0	4	0	4	0
			----------------YKSAITTTINP-----------	780	1	1	1	-		-		-	Nv	3	0	3	0	1	0	1	0	2	0	2	0
56	79	ITGB1	---------ENPIYKSPINNFKNPNYGRK---------	788	1	1	1	-		-		-	Rp	16	16	0	0	9	9	0	0	7	7	0	0
			--------ENPIYKSPINNFKNPNYGRK----------	789	0	1	1		-	-		-	Rp	4	4	0	0	0	0	0	0	4	4	0	0
			-------ENPIYKSPINNFKNPNYGRK-----------	790	0	1	1		-	-		-	Rp	1	1	0	0	0	0	0	0	1	1	0	0
			--------NPIYKSPINNFKNPNYGRK-----------	790	0	1	1		-	-		-	Rp	1	1	0	0	0	0	0	0	1	1	0	0
			------ENPIYKSPINNFKNPNYGRK------------	791	1	1	1	90.0	4.2	128.0	-	109.0	Rp	4	4	0	0	3	3	0	0	1	1	0	0
			--------PIYKSPINNFKNPNYGRK------------	791	0	1	1		-	-		-	Rp	1	1	0	0	0	0	0	0	1	1	0	0
57	2	ITGB2	---------SQWNNDNPLFKSATTTVMNP---------	754	0	1	1			20.7	-	20.7	Rp	1	0	1	0	0	0	0	0	1	0	1	0
			-------SQWNNDNPLFKSATTTVMNP-----------	756	0	1	1		-	-		-	Nv	2	0	2	0	0	0	0	0	2	0	2	0
			-SQWNNDNPLFKSATTTVMNP-----------------	762	0	1	1		-	-		-	Rp	3	3	0	0	0	0	0	0	3	3	0	0
59	56	ATP2B2	---------GQILWFRGLNRIQTQIRVVK---------	1135	1	1	1	-		-		-	Rp	31	24	0	7	18	14	0	4	13	10	0	3
			-----------ILWFRGLNRIQTQIRVVK---------	1135	1	0	1	-			-	-	Rp	1	1	0	0	1	1	0	0	0	0	0	0
			------GQILWFRGLNRIQTQIRVVK------------	1138	0	1	1		-	-		-	Nv	1	1	0	0	0	0	0	0	1	1	0	0
			--GQILWFRGLNRIQTQIRVVK----------------	1142	1	1	1	-		-		-	Nv	7	0	0	7	4	0	0	4	3	0	0	3
60	24	PTPRN	------------------CVRQHARQQDKERLAALGPE	599	1	1	1	-		-		-	Nv	2	0	1	1	1	0	1	0	1	0	0	1
			---------CVRQHARQQDKERLAALGPE---------	608	1	1	1	418.1	30.1	560.9	118.0	489.5	Rp	14	0	13	1	9	0	9	0	5	0	4	1
			----------VRQHARQQDKERLAALGPE---------	608	1	1	1	-		-		-	Rp	4	4	0	0	2	2	0	0	2	2	0	0
61	24	PTPRN	---------PEPSRVSSVSSQFSDAAQAS---------	658	1	1	1	196.0	51.1	172.8	32.5	184.4	Rp	23	0	23	0	17	0	17	0	6	0	6	0
			--------PEPSRVSSVSSQFSDAAQAS----------	659	0	1	1		-	-		-	Nv	1	0	1	0	0	0	0	0	1	0	1	0
			------PEPSRVSSVSSQFSDAAQAS------------	661	0	1	1		-	-		-	Nv	1	0	1	0	0	0	0	0	1	0	1	0
62	84	BID	---------GNRSSHSRLGRIEADSESQE---------	70	0	1	1		-	-		-	Rp	1	1	0	0	0	0	0	0	1	1	0	0
63	26	BAX	----------------IMKTGALLLQGFIQDRAGRM--	21	0	1	1		-	-		-	Nv	1	0	0	1	0	0	0	0	1	0	0	1
			----------IMKTGALLLQGFIQDRAGRM--------	27	1	1	1	49.3	11.5	67.4	12.5	58.4	Nv	11	0	11	0	4	0	4	0	7	0	7	0
			---------IMKTGALLLQGFIQDRAGRM---------	28	1	1	1	598.2	27.6	560.2	61.4	579.2	Rp	80	41	37	2	51	23	26	2	29	18	11	0
			--------IMKTGALLLQGFIQDRAGRM----------	29	1	1	1	59.0	4.6	72.1	8.4	65.6	Nv	8	0	8	0	4	0	4	0	4	0	4	0
			---IMKTGALLLQGFIQDRAGRM---------------	34	0	1	1		-	-		-	Nv	1	0	0	1	0	0	0	0	1	0	0	1
			-IMKTGALLLQGFIQDRAGRM-----------------	36	1	0	1	-			-	-	Nv	2	0	0	2	2	0	0	2	0	0	0	0
64	70	IGFBP4	-----------------RDRSTSGGKMKVNGAPREDA-	148	0	1	1		-	-		-	Nv	1	0	1	0	0	0	0	0	1	0	1	0
			---------RDRSTSGGKMKVNGAPREDA---------	156	1	1	1	38.4	-	21.4	13.0	29.9	Nv	12	0	12	0	5	0	5	0	7	0	7	0
65	40	HBA1	------------------VLSPADKTNVKAAWGKVGAH	2	0	1	1		-	-		-	Nv	1	0	1	0	0	0	0	0	1	0	1	0
			---------VLSPADKTNVKAAWGKVGAH---------	11	1	1	1	26.3	5.8	-		26.3	Rp	23	0	23	0	15	0	15	0	8	0	8	0
			--------VLSPADKTNVKAAWGKVGAH----------	12	1	1	1	116.8	35.4	107.8	14.7	112.3	Nv	13	1	12	0	8	0	8	0	5	1	4	0
66	50	EGFR	-------------VRKRTLRRLLQERELVEPLT-----	679	1	1	1	-		-		-	Nv	2	0	2	0	1	0	1	0	1	0	1	0
			---------VRKRTLRRLLQERELVEPLT---------	683	1	1	1	811.5	47.7	1,111.2	129.7	961.4	Rp	47	15	32	0	26	13	13	0	21	2	19	0
			--------VRKRTLRRLLQERELVEPLT----------	684	0	1	1		-	-		-	Nv	1	0	1	0	0	0	0	0	1	0	1	0
67	50	EGFR	---------------VQPTCVNSTFDSPAHWAQKG---	1145	0	1	1		-	-		-	Nv	1	0	1	0	0	0	0	0	1	0	1	0
			------------VQPTCVNSTFDSPAHWAQKG------	1148	1	1	1	-		-		-	Nv	3	0	3	0	2	0	2	0	1	0	1	0
			---------VQPTCVNSTFDSPAHWAQKG---------	1151	1	1	1	357.2	127.3	233.2	11.7	295.2	Rp	72	0	72	0	44	0	44	0	28	0	28	0
			----VQPTCVNSTFDSPAHWAQKG--------------	1156	0	1	1		-	-		-	Nv	2	2	0	0	0	0	0	0	2	2	0	0
68	73	DMD	---------VMETVTTVTTREQILVKHAQ---------	690	1	1	1	48.4	2.4	85.9	12.6	67.1	Rp	7	0	7	0	4	0	4	0	3	0	3	0
			--------VMETVTTVTTREQILVKHAQ----------	691	1	0	1	-			-	-	Nv	1	0	1	0	1	0	1	0	0	0	0	0
			-------VMETVTTVTTREQILVKHAQ-----------	692	1	1	1	40.1	7.8	31.6	4.2	35.8	Nv	9	0	9	0	5	0	5	0	4	0	4	0
69	36	SNCA	---------VVHGVATVAEKTKEQVTNVG---------	57	1	1	1	163.1	15.7	165.8	-	164.5	Rp	5	0	5	0	4	0	4	0	1	0	1	0
			------VVHGVATVAEKTKEQVTNVG------------	60	1	1	1	-		-		-	Nv	2	1	1	0	1	0	1	0	1	1	0	0

Table S8 All calpain cleavage site sequences identified from fragment peptide spectra.

B. Stringent condition

ID No.	SB No.	Protein	Site sequence, P19 to P19'[*1]<br>1 1 1<br>9...5....0....5...1	 1 1 1<br>1'..5'...0'...5'..9'	Site[*2]	Cleaved by[*3] C1	C2	C1 or C2	C1[*4] k_{cat}/K_m	SE	C2 k_{cat}/K_m	SE	Ave.	Rp/Nv	C1+C2 T	N	C	M	C1 T	N	C	M	C2 T	N	C	M
70	23	IGFBP5	---------AEAVKKDRRK	KLTQSKFVGG---------	158	1	1	1	23.1	-	15.0	4.2	19.1	Nv	6	0	6	0	2	0	2	0	4	0	4	0
			------AEAVKKDRRKKLT	QSKFVGG------------	161	1	1	1	613.9	114.3	718.0	289.2	665.9	Rp	13	4	9	0	10	4	6	0	3	0	3	0
71	22	CTTN	------------------T	VPVEAVTSKTSNIRANFEN	337	0	1	1		-	-		-	Nv	2	0	0	2	0	0	0	0	2	0	0	2
			-----------------TV	PVEAVTSKTSNIRANFEN-	338	0	1	1		-	-		-	Nv	1	0	0	1	0	0	0	0	1	0	0	1
			---------TVPVEAVTSK	TSNIRANFEN---------	346	1	1	1	69.1	7.4	87.2	9.0	78.1	Rp	24	15	8	1	10	7	3	0	14	8	5	1
			-----------PVEAVTSK	TSNIRANFEN---------	346	1	1	1	-		-		-	Rp	5	5	0	0	1	1	0	0	4	4	0	0
			-------TVPVEAVTSKTS	NIRANFEN-----------	348	0	1	1		-	-		-	Nv	1	0	0	1	0	0	0	0	1	0	0	1
			------PVEAVTSKTSNIR	ANFEN--------------	351	1	1	1	-		-		-	Rp	2	2	0	0	1	1	0	0	1	1	0	0
			----TVPVEAVTSKTSNIR	ANFEN--------------	351	1	1	1	-		-		-	Rp	10	9	0	1	7	7	0	0	3	2	0	1
			-----VPVEAVTSKTSNIR	ANFEN--------------	351	0	1	1		-	-		-	Rp	1	1	0	0	0	0	0	0	1	1	0	0
72	90	FLNA	---------LRSQQLAPQY	TYAQGGQQTW---------	1753	1	1	1	213.9	68.6	154.3	11.7	184.1	Rp	21	6	15	0	16	5	11	0	5	1	4	0
			-------LRSQQLAPQYTY	AQGGQQTW-----------	1755	1	1	1	-		-		-	Nv	5	3	2	0	3	2	1	0	2	1	1	0
73	53	TP53	---------------QETF	SDLWKLLPENNVLSPL---	19	0	1	1		-	-		-	Nv	1	0	1	0	0	0	0	0	1	0	1	0
			-----------QETFSDLW	KLLPENNVLSPL-------	23	1	0	1	-			-	-	Nv	2	0	2	0	2	0	2	0	0	0	0	0
			---------QETFSDLWKL	LPENNVLSPL---------	25	1	1	1	8.9	1.6	14.3	-	11.6	Rp	6	0	6	0	4	0	4	0	2	0	2	0
			-----QETFSDLWKLLPEN	NVLSPL-------------	29	1	0	1	-			-	-	Nv	1	0	1	0	1	0	1	0	0	0	0	0
			-----ETFSDLWKLLPENN	VLSPL--------------	30	1	1	1	-		-		-	Nv	5	5	0	0	2	2	0	0	3	3	0	0
			----QETFSDLWKLLPENN	VLSPL--------------	30	1	1	1	-		-		-	Nv	3	3	0	0	2	2	0	0	1	1	0	0
74	16	GJA8	---------VGMVEASPLS	AKPFSQFEEK---------	300	0	1	1		-	-		-	Rp	1	1	0	0	0	0	0	0	1	1	0	0
75	95	CRYBB3	---------PEQAAANKSH	GGLGGSYKVT---------	17	1	1	1	25.6	-	30.0	9.7	27.8	Rp	7	0	7	0	1	0	1	0	6	0	6	0
			-----PEQAAANKSHGGLG	GSYKVT-------------	21	1	1	1	119.3	-	-		119.3	Nv	5	4	1	0	3	2	1	0	2	2	0	0
			--------AAANKSHGGLG	GSYKVT-------------	21	0	1	1		-	-		-	Nv	1	1	0	0	0	0	0	0	1	1	0	0
76	88	CRYBB1	---------SVPRPAAKVG	ELPPGSYRLV---------	49	1	1	1	208.5	15.8	249.2	17.5	228.9	Rp	23	3	20	0	11	0	11	0	12	3	9	0
77	94	CRYBB1	----------------AKA	SATAAVNPGPDGKGKAG--	8	1	0	1	-			-	-	Nv	1	0	1	0	1	0	1	0	0	0	0	0
			---------------AKAS	ATAAVNPGPDGKGKAG---	9	1	0	1	-			-	-	Nv	1	0	1	0	1	0	1	0	0	0	0	0
			------------AKASATA	AVNPGPDGKGKAG------	12	1	1	1	-		-		-	Rp	4	0	4	0	2	0	2	0	2	0	2	0
			---------AKASATAAVN	PGPDGKGKAG---------	15	1	1	1	51.2	12.6	45.2	11.2	48.2	Rp	16	14	2	0	8	7	1	0	8	7	1	0
			AKASATAAVNPGPDGKGKA	G------------------	24	1	0	1	-			-	-	Nv	1	1	0	0	1	1	0	0	0	0	0	0
78	25	SAG	---------VFEEFARQNL	KDAGEYKEEK---------	385	1	1	1	-		-		-	Rp	26	26	0	0	16	16	0	0	10	10	0	0
79	21	CAPN11	---------------AARL	QRDRLRAEGVGEHNNA---	12	1	0	1	-			-	-	Nv	1	0	1	0	1	0	1	0	0	0	0	0
			--------------AARLQ	RDRLRAEGVGEHNNA----	13	1	1	1	-		-		-	Nv	10	0	10	0	8	0	8	0	2	0	2	0
			-------------AARLQR	DRLRAEGVGEHNNA-----	14	0	1	1		-	-		-	Nv	1	0	1	0	0	0	0	0	1	0	1	0
			------------AARLQRD	RLRAEGVGEHNNA------	15	1	1	1	-		-		-	Nv	8	0	8	0	4	0	4	0	4	0	4	0
			-----------AARLQRDR	LRAEGVGEHNNA-------	16	1	1	1	-		-		-	Nv	7	0	7	0	5	0	5	0	2	0	2	0
			----------AARLQRDRL	RAEGVGEHNNA--------	17	1	0	1	-			-	-	Nv	1	0	1	0	1	0	1	0	0	0	0	0
			---------AARLQRDRLR	AEGVGEHNNA---------	18	1	1	1	72.0	7.3	118.0	22.7	95.0	Rp	23	0	23	0	14	0	14	0	9	0	9	0
81	17	CAPNS1	----------PPPPRSHVS	MVDPNESEEVR--------	34	1	0	1	-			-	-	Nv	4	4	0	0	4	4	0	0	0	0	0	0
			---------PPPPRSHVSM	VDPNESEEVR---------	35	1	1	1	36.0	7.0	45.4	21.6	40.7	Rp	6	0	6	0	3	0	3	0	3	0	3	0
			------PPPPRSHVSMVDP	NESEEVR------------	38	1	1	1	-		-		-	Nv	2	0	2	0	1	0	1	0	1	0	1	0
83	20	CAPN1	-------------ITPVYC	TGVSAQVQKQRARE-----	11	1	1	1	24.3	4.0	103.5	-	63.9	Nv	8	0	8	0	7	0	7	0	1	0	1	0
			----------ITPVYCTGV	SAQVQKQRARE--------	14	0	1	1			47.0	-	47.0	Nv	1	0	1	0	0	0	0	0	1	0	1	0
			---------ITPVYCTGVS	AQVQKQRARE---------	15	1	1	1	468.5	22.0	630.4	60.1	549.4	Rp	9	0	9	0	7	0	7	0	2	0	2	0
			--------ITPVYCTGVSA	QVQKQRARE----------	16	1	0	1	162.7	9.9			162.7	Nv	7	0	7	0	7	0	7	0	0	0	0	0
			-----ITPVYCTGVSAQVQ	KQRARE-------------	19	1	0	1	171.7	0.0			171.7	Nv	2	0	2	0	2	0	2	0	0	0	0	0
84	20	CAPN1	---------VQKQRARELG	LGRHENAIKY---------	27	1	1	1	856.7	50.4	1,016.1	53.4	936.4	Rp	59	4	55	0	42	3	39	0	17	1	16	0
			-------VQKQRARELGLG	RHENAIKY-----------	29	0	1	1		-	-		-	Nv	2	0	2	0	0	0	0	0	2	0	2	0
85	1	CAPN2	---------KDREAAEGLG	SHERAIKYLN---------	19	0	1	1			73.9	-	73.9	Rp	1	0	1	0	0	0	0	0	1	0	1	0
86	11	NFKBIA	---------EYEQMVKELQ	EIRLEPQEVP---------	50	1	1	1	420.2	61.3	418.4	37.7	419.3	Rp	34	16	11	7	7	4	1	2	27	12	10	5
			--------EYEQMVKELQE	IRLEPQEVP----------	51	0	1	1		-	-		-	Nv	1	1	0	0	0	0	0	0	1	1	0	0
			------EYEQMVKELQEIR	LEPQEVP------------	53	1	1	1	248.2	15.0	197.2	18.5	222.7	Nv	33	26	7	0	22	19	3	0	11	7	4	0
			-----------VKELQEIR	LEPQEVP------------	53	0	1	1		-	-		-	Nv	1	1	0	0	0	0	0	0	1	1	0	0
			-EYEQMVKELQEIRLEPQE	VP-----------------	58	1	1	1	221.1	28.1	179.6	12.3	200.3	Nv	25	18	0	7	14	12	0	2	11	6	0	5
			---EQMVKELQEIRLEPQE	VP-----------------	58	0	1	1		-	-		-	Nv	1	1	0	0	0	0	0	0	1	1	0	0
			-----MVKELQEIRLEPQE	VP-----------------	58	1	1	1	-		-		-	Nv	19	19	0	0	17	17	0	0	2	2	0	0
87	37	VIM	------------------L	LLRRVVAGSPSVRSTSVRY	61	1	0	1	-			-	-	Nv	4	0	1	3	4	0	1	3	0	0	0	0
			----------------LLL	RRVVAGSPSVRSTSVRY--	63	1	0	1	-			-	-	Nv	1	0	0	1	1	0	0	1	0	0	0	0
			---------------LLLR	RVVAGSPSVRSTSVRY---	64	1	1	1	-		-		-	Nv	21	0	8	13	12	0	3	9	9	0	5	4
			--------------LLLRR	VVAGSPSVRSTSVRY----	65	1	0	1	-			-	-	Nv	1	0	1	0	1	0	1	0	0	0	0	0
			-----------LLLRRVVA	GSPSVRSTSVRY-------	68	1	1	1	-		-		-	Nv	7	0	7	0	6	0	6	0	1	0	1	0
			----------LLLRRVVAG	SPSVRSTSVRY--------	69	1	0	1	-			-	-	Nv	1	0	1	0	1	0	1	0	0	0	0	0
			------LLLRRVVAGSPSV	RSTSVRY------------	73	1	1	1	-		-		-	Nv	10	9	1	0	8	7	1	0	2	2	0	0
			-----LLLRRVVAGSPSVR	STSVRY-------------	74	1	1	1	321.6	85.2	347.0	-	334.3	Nv	122	105	4	13	69	57	3	9	53	48	1	4
			------LLRRVVAGSPSVR	STSVRY-------------	74	1	1	1	-		-		-	Nv	59	59	0	0	34	34	0	0	25	25	0	0
			-------LRRVVAGSPSVR	STSVRY-------------	74	1	1	1	-		-		-	Nv	24	24	0	0	15	15	0	0	9	9	0	0
			--------RRVVAGSPSVR	STSVRY-------------	74	1	0	1	-			-	-	Nv	1	1	0	0	1	1	0	0	0	0	0	0
			---LLLRRVVAGSPSVRST	SVRY---------------	76	1	0	1	-			-	-	Nv	5	1	0	4	5	1	0	4	0	0	0	0
			----LLRRVVAGSPSVRST	SVRY---------------	76	1	1	1	-		-		-	Nv	7	7	0	0	6	6	0	0	1	1	0	0
			---LLRRVVAGSPSVRSTS	VRY----------------	77	1	0	1	-			-	-	Nv	1	1	0	0	1	1	0	0	0	0	0	0
88	81	TLN1	------------EQVLKVG	TVSRKNKYPQSQV------	430	1	1	1	-		-		-	Nv	3	0	3	0	1	0	1	0	2	0	2	0
			---------EQVLKVGTVS	RKNKYPQSQV---------	433	1	1	1	-		-		-	Nv	4	1	3	0	2	0	2	0	2	1	1	0
			------EQVLKVGTVSRKN	KYPQSQV------------	436	1	0	1	-			-	-	Nv	1	1	0	0	1	1	0	0	0	0	0	0
89	61	SPTAN1	----SYDVTEEQDQRQPVM	AMQGV--------------	1181	1	1	1	-		-		-	Nv	17	17	0	0	10	10	0	0	7	7	0	0
90	15	RYR1	---------------RTPM	LMAAPATKAKPAPKQF---	1394	1	1	1	-		-		-	Nv	18	0	18	0	11	0	11	0	7	0	7	0
			--------------RTPML	MAAPATKAKPAPKQF----	1395	1	1	1	-		-		-	Nv	3	0	3	0	1	0	1	0	2	0	2	0
			-------------RTPMLM	AAPATKAKPAPKQF-----	1396	1	1	1	784.6	44.4	1,162.4	114.5	973.5	Nv	49	0	48	1	32	0	31	1	17	0	17	0
			------------RTPMLMA	APATKAKPAPKQF------	1397	1	1	1	-		-		-	Nv	3	0	3	0	1	0	1	0	2	0	2	0
			--------RTPMLMAAPAT	KAKPAPKQF----------	1401	1	1	1	172.4	12.1	129.1	16.8	150.7	Nv	14	0	14	0	11	0	11	0	3	0	3	0
			-------RTPMLMAAPATK	AKPAPKQF-----------	1402	1	1	1	133.5	10.6	178.9	29.9	156.2	Nv	39	31	7	1	25	21	3	1	14	10	4	0
			--------TPMLMAAPATK	AKPAPKQF-----------	1402	0	1	1		-	-		-	Nv	1	1	0	0	0	0	0	0	1	1	0	0
91	62	ITGB7	-------TDIQAKSWKNPY	NLIPTNTS-----------	780	1	1	1	16.3	6.4	23.7	1.6	20.0	Nv	4	4	0	0	2	2	0	0	2	2	0	0
			------TDIQAKSWKNPYN	LIPTNTS------------	781	1	1	1	914.5	123.0	943.3	46.1	928.9	Nv	49	25	24	0	23	14	9	0	26	11	15	0
92	90	FLNA	--------GQQQASYRTLW	YTQQPQAGL----------	1762	1	1	1	-		-		-	Nv	29	6	23	0	22	3	19	0	7	3	4	0
			-------GQQQASYRTLWY	TQQPQAGL-----------	1763	1	1	1	-		-		-	Nv	48	17	31	0	29	10	19	0	19	7	12	0
			---------QQASYRTLWY	TQQPQAGL-----------	1763	0	1	1		-	-		-	Nv	1	1	0	0	0	0	0	0	1	1	0	0
			------GQQQASYRTLWYT	QQPQAGL------------	1764	1	1	1	-		-		-	Nv	19	4	15	0	12	2	10	0	7	2	5	0
			-----GQQQASYRTLWYTQ	QPQAGL-------------	1765	1	1	1	-		-		-	Nv	15	6	9	0	10	4	6	0	5	2	3	0
			--------QASYRTLWYTQ	QPQAGL-------------	1765	1	1	1	-		-		-	Nv	3	3	0	0	2	2	0	0	1	1	0	0
			-------QQASYRTLWYTQ	QPQAGL-------------	1765	0	1	1		-	-		-	Nv	1	1	0	0	0	0	0	0	1	1	0	0
93	53	TP53	---------NSNFETPELQ	LLPKSWLLVD---------	25	1	0	1	-			-	-	Nv	1	0	1	0	1	0	1	0	0	0	0	0
94	20	CAPN1	-----------ERHQRKGQ	LVYGKLIEARNA-------	25	1	0	1	-			-	-	Nv	1	0	1	0	1	0	1	0	0	0	0	0
			----------ERHQRKGQL	VYGKLIEARNA--------	26	1	1	1	-		-		-	Nv	2	0	2	0	1	0	1	0	1	0	1	0
			--------ERHQRKGQLVY	GKLIEARNA----------	28	1	0	1	221.0	29.6			221.0	Nv	5	4	1	0	5	4	1	0	0	0	0	0
			-------ERHQRKGQLVYG	KLIEARNA-----------	29	1	0	1	-			-	-	Nv	2	0	2	0	2	0	2	0	0	0	0	0

*1 Full-length peptides were not identified for P87 ID Nos. 38, 43, 54, and 55, so the estimated longest sequence (38: TRKISQTAQTYDPRE, 43: LCIGNH, 54: PLYKEATSTFTNI, and 55: PLYKSAITTTINP) was used to estimate the cleavage site sequences instead of the full-length sequences.

*2 Numbers indicate the positions of the P1 aar in the whole protein sequence.

*3 1: cleaved fragment detected, 0: cleaved fragment not detected.

*4 Abbreviations used in this table: Ave, the average of the k_{cat}/K_m values for C1 and C2; N, C, M, and T, number of spectra identified for N-terminal, C-terminal, or internal sites [*e.g.* , cleavage sites in a both-side cleaved (uncapped) peptide fragment], and sum of them all, respectively; for protein names, see Table S2.

Table S9 All identified both-capped (uncleaved) peptides.

A Normal condition

ID No.	SB No.	Protein	Ac- 1 2 3 4 5 6 7 8 9 10	11 12 13 14 15 16 17 18 19 20 -DKP[1]	length	FL (F) or not	#sp.[2] total	C1	C2	C1[3] k_{cat}/K_m	SE	C2 k_{cat}/K_m	SE	Ave. of C1 and C2
2	44	COPB1	**G T Y A T Q S A L S**	**S S R P T K K E E D**	20	F	5	3	2	399.5	2.3	180.2	27.3	289.9
			S A L S	S S R P T K K E E D	14		5	3	2	150.2	27.1	-	-	150.2
4	29	TNNT3	**E E E K P R P K L T**	**A P K I P E G E K V**	20	F	15	13	2	143.7	27.7	-	-	143.7
			P K L T	A P K I P E G E K V	14		1	0	1			-	-	
5	45	MBP	**S Q R S K Y L A S A**	**S T M D H A R H G F**	20	F	14	11	3	221.4	66.4	-	-	221.4
			R S K Y L A S A	S T M D H A R H G F	18		3	2	1	6.3	-	-	-	6.3
			S K Y L A S A	S T M D H A R H G F	17		15	11	4	137.0	21.7	-	-	137.0
			L A S A	S T M D H A R H G F	14		27	18	9	-	-	-	-	
			A S A	S T M D H A R H G F	13		21	14	7	-	-	168.6	-	168.6
			S A	S T M D H A R H G F	12		3	1	2	-	-	-	-	
			A	S T M D H A R H G F	11		1	1	0	-	-			
6	45	MBP	**P V V H F F K N I V**	**T P R T P P P S Q G**	20	F	86	42	44	303.6	10.2	272.5	9.2	288.0
			F F K N I V	T P R T P P P S Q G	16		3	1	2	273.8	-	59.8	8.7	166.8
			F K N I V	T P R T P P P S Q G	15		1	0	1			-	-	
				P V V H F F K N I V	10		1	1	0	-	-			
8	55	PRKCA	A K L G P A G N K	V I S P S E D R K Q	19		6	4	2	-	-	-	-	
			P A G N K	V I S P S E D R K Q	15		3	3	0	21.1	-			21.1
			N K	V I S P S E D R K Q	12		1	1	0	14.8	-			14.8
9	89	PRKCG	**Y E R V R M G P S S**	**S P I P S P S P S P**	20	F	135	94	41	73.9	17.1	111.4	-	92.7
10	39	PRKCB	**R Q K F E R A K I G**	**Q G T K A P E E K T**	20	F	12	10	2	40.6	12.8	-	-	40.6
			Q K F E R A K I G	Q G T K A P E E K T	19		7	7	0	-	-			
			F E R A K I G	Q G T K A P E E K T	17		7	2	5	-	-	-	-	
			E R A K I G	Q G T K A P E E K T	16		2	1	1	-	-	-	-	
			A K I G	Q G T K A P E E K T	14		3	1	2	-	-	-	-	
			G	Q G T K A P E E K T	11		7	5	2	-	-	-	-	
				Q G T K A P E E K T	10		10	5	5	116.1	-	-	-	116.1
				G T K A P E E K T	9		15	8	7	28.5	17.1	-	-	28.5
				T K A P E E K T	8		2	1	1	-	-	-	-	
11	8	PLCB1	**T P K P P S Q A L H**	**S Q P A P G S V K A**	20	F	25	17	8	203.8	17.9	106.6	20.0	155.2
			P S Q A L H	S Q P A P G S V K A	16		7	5	2	168.8	58.7	51.3	-	110.1
			A L H	S Q P A P G S V K A	13		12	7	5	271.3	16.7	150.7	12.5	211.0
				P A P G S V K A	8		18	10	8	71.6	64.4	354.9	316.1	213.2
				P G S V K A	6		1	1	0	210.1	-			210.1
12	65	PARP1	**S A A S A P A A V H**	**S G P P D K P L S N**	20	F	130	94	36	183.3	5.4	41.7	10.8	112.5
			A A S A P A A V H	S G P P D K P L S N	19		1	1	0	56.3	-			56.3
			P A A V H	S G P P D K P L S N	15		1	1	0	149.7	-			149.7
			V H	S G P P D K P L S N	12		4	3	1	-	-	-	-	
13	65	PARP1	**Q D E E A V K K L T**	**V N P G T K S K L P**	20	F	3	2	1	418.4	4.2	-	-	418.4
			D E E A V K K L T	V N P G T K S K L P	19		1	0	1			-	-	
			E A V K K L T	V N P G T K S K L P	17		2	1	1	186.9	-	-	-	186.9
14	60	INS	L V E	A L Y L V C G E R G	13		8	8	0	75.5	-			75.5
			V E	A L Y L V C G E R G	12		7	7	0	-	-			
			E	A L Y L V C G E R G	11		2	2	0	-	-			
				A L Y L V C G E R G	10		2	2	0	-	-			
				L Y L V C G E R G	9		3	3	0	-	-			
				Y L V C G E R G	8		1	1	0	-	-			
15	41	GNRH1	**E G C S S Q H W S Y**	**G L R P G G K R N T**	20	F	9	7	2	248.4	6.5	-	-	248.4
			S S Q H W S Y	G L R P G G K R N T	17		2	2	0	-	-			
				P G G K R N T	7		1	1	0	-	-			
17	34	NTS	L Y	E N K P R R P Y I L	12		1	1	0	-	-			
19	54	GCG	D F	V Q W L M N T K R N	12		19	10	9	169.5	40.5	133.1	27.4	151.3
20	54	GCG	**R A Q D F V Q W L M**	**N T K R N R N N I A**	20	F	3	0	3			297.8	7.8	297.8
			D F V Q W L M	N T K R N R N N I A	17		2	0	2			271.4	20.9	271.4
			L M	N T K R N R N N I A	12		1	0	1			-	-	
				N R N N I A	6		2	1	1	-	-	-	-	
21	10	NEFM	**V V A A K K S P V K**	**A T A P E L K E E E**	20	F	15	12	3	233.0	32.0	-	-	233.0
22	42	CASP9	**L T P V V L R P E I**	**R K P E V L R P E T**	20	F	144	111	33	69.0	5.8	36.2	11.7	52.6
			P E I	R K P E V L R P E T	13		2	2	0	-	-			
24	48	ANXA1	**E E Q E Y V Q T V K**	**S S K G G P G S A V**	20	F	5	3	2	421.0	3.1	385.3	39.4	403.2
			E Y V Q T V K	S S K G G P G S A V	17		1	1	0	78.8	-			78.8
			Y V Q T V K	S S K G G P G S A V	16		3	2	1	77.6	-	-	-	77.6
			V Q T V K	S S K G G P G S A V	15		7	4	3	41.7	3.8	-	-	41.7
			Q T V K	S S K G G P G S A V	14		3	2	1	-	-	-	-	
			T V K	S S K G G P G S A V	13		6	3	3	2.5	-	-	-	2.5
			V K	S S K G G P G S A V	12		1	1	0	-	-			
25	31	ACTB	**R A V F P S I V G R**	**P R H Q G V M V G M**	20	F	10	8	2	-	-	-	-	
			A V F P S I V G R	P R H Q G V M V G M	19		16	13	3	-	-	-	-	
			R A V F P S I V	G R P R H Q G V M V	18		4	2	2	-	-	185.3	-	185.3
			A V F P S I V	G R P R H Q G V M V	17		1	0	1			-	-	
				P R H Q G V M V G M	10		8	5	3	-	-	-	-	
				H Q G V M V G M	8		1	0	1			-	-	
26	46	MIP	**S I S E R L S V L K**	**G A K P D V S N G Q**	20	F	18	12	6	254.0	12.1	164.5	26.7	209.3
			I S E R L S V L K	G A K P D V S N G Q	19		1	1	0	157.3	-			157.3
			L S V L K	G A K P D V S N G Q	15		2	1	1	125.5	-	7.3	-	66.4

A Normal condition

Table S9 All identified both-capped (uncleaved) peptides.

ID No.	SB No.	Protein	Ac- 1 2 3 4 5 6 7 8 9 10	11 12 13 14 15 16 17 18 19 20 -DKP*1	length	FL (F) or not	#sp.*2 total	#sp.*2 C1	#sp.*2 C2	C1*3 k_{cat}/K_m	C1*3 SE	C2 k_{cat}/K_m	C2 SE	Ave. of C1 and C2
			S V L K	G A K P D V S N G Q	14		1	1	0	105.0	-			105.0
28	83	CDKN2D	**D V Q E V R R L L H**	**R E L V H P D A L N**	20	F	5	3	2	583.6	-	-	-	583.6
				D V Q E V R R	7		22	12	10	-	-	-	-	
				V Q E V R R	6		6	4	2	-	-	-	-	
29	83	CDKN2D	**A I A L E L L K Q G**	**A S P N V Q D T S G**	20	F	61	40	21	342.5	4.9	155.7	11.2	249.1
			I A L E L L K Q G	A S P N V Q D T S G	19		20	12	8	87.6	21.6	15.1	-	51.3
			A L E L L K Q G	A S P N V Q D T S G	18		5	3	2	-	-	-	-	
			L E L L K Q G	A S P N V Q D T S G	17		2	1	1	-	-	-	-	
				I A L E L L	6		1	1	0	-	-			
30	37	VIM	**Y R R M F G G S G T**	**S S R P S S N R S Y**	20	F	14	12	2	-	-	-	-	
			R R M F G G S G T	S S R P S S N R S Y	19		4	4	0	-	-			
			R M F G G S G T	S S R P S S N R S Y	18		10	7	3	-	-	-	-	
			M F G G S G T	S S R P S S N R S Y	17		12	7	5	0.6	-	-	-	0.6
			F G G S G T	S S R P S S N R S Y	16		1	1	0	-	-			
			G S G T	S S R P S S N R S Y	14		2	1	1	-	-	-	-	
			S G T	S S R P S S N R S Y	13		1	0	1			-	-	
			T	S S R P S S N R S Y	11		1	0	1			-	-	
				S S R P S S N R S Y	10		1	1	0	-	-			
				P S S N R S Y	7		2	0	2			-	-	
31	37	VIM	**Y V T R S S A V R L**	**R S S V P G V R L L**	20	F	1	1	0	-	-			
			Y V T R S S A V R	L R S S V P G V R L	19		1	1	0	-	-			
			S S A V R L	R S S V P G V R L L	16		62	44	18	-	-	-	-	
			S A V R L	R S S V P G V R L L	15		72	50	22	-	-	-	-	
			A V R L	R S S V P G V R L L	14		1	0	1			-	-	
			L	R S S V P G V R L L	11		7	3	4	-	-	-	-	
				S S V P G V R L L	9		1	0	1			-	-	
32	37	VIM	**Q I D V D V S K P D**	**L T A A L R D V R Q**	20	F	16	6	10	225.2	31.8	239.2	31.0	232.2
			I D V D V S K P D	L T A A L R D V R Q	19		6	1	5	261.5	-	161.7	21.7	211.6
			P D	L T A A L R D V R Q	12		21	14	7	-	-	-	-	
			D	L T A A L R D V R Q	11		2	1	1	-	-	-	-	
				L T A A L R D V R Q	10		3	2	1	-	-	-	-	
				T A A L R D V R Q	9		7	4	3	-	-	-	-	
				A A L R D V R Q	8		5	3	2	-	-	-	-	
33	59	TH	**G F R R A V S E L D**	**A K Q A E A I M S P**	20	F	13	9	4	135.9	22.6	71.4	19.2	103.6
			A V S E L D	A K Q A E A I M S P	16		1	1	0	148.3	-			148.3
34	81	TLN1		Q Q Y N R V G K V E	10		3	0	3			-	-	
				Q Y N R V G K V E	9		2	1	1	87.2	-	-	-	87.2
				Y N R V G K V E	8		3	1	2	42.1	-	-	-	42.1
				N R V G K V E	7		3	2	1	15.3	8.8	-	-	15.3
35	78	SPTB	A F E K S T	A S W A E R F A A L	16		1	0	1			334.4	-	334.4
			S T	A S W A E R F A A L	12		25	16	9	-	-	-	-	
			T	A S W A E R F A A L	11		16	7	9	-	-	-	-	
				A S W A E R F A A L	10		12	5	7	-	-	-	-	
				S W A E R F A A L	9		14	10	4	119.6	-	-	-	119.6
				W A E R F A A L	8		8	4	4	-	-	-	-	
36	61	SPTAN1	**V Q A V Q Q Q E V Y**	**G M M P R D E T D S**	20	F	13	11	2	266.2	-	-	-	266.2
			E V Y	G M M P R D E T D S	13		2	1	1	-	-	-	-	
			V Y	G M M P R D E T D S	12		24	15	9	-	-	-	-	
37	15	RYR1	**F K A K K A A M M T**	**Q P P A T P A L P R**	20	F	73	46	27	362.1	6.3	290.9	7.8	326.5
			A K K A A M M T	Q P P A T P A L P R	18		8	5	3	98.3	18.0	-	-	98.3
			M M T	Q P P A T P A L P R	13		33	22	11	-	-	-	-	
				P P A T P A L P R	9		7	5	2	-	-	-	-	
38	15	RYR1	T R K I S	Q T A Q T Y D P R E	15		5	3	2	64.6	29.6	-	-	64.6
			R K I S	Q T A Q T Y D P R E	14		4	3	1	44.1	36.8	-	-	44.1
			S	Q T A Q T Y D P R E	11		2	0	2			-	-	
				T A Q T Y D P R E	9		1	0	1			-	-	
39	72	GRIN2A	**Q D W S Q N N A L Q**	**F Q K N K L R I N R**	20	F	1	0	1			57.1	-	57.1
40	72	GRIN2A	**L E G N L Y G S L F**	**S V P S S K L L G N**	20	F	9	3	6	387.9	6.4	368.6	7.2	378.3
				P S S K L L G N	8		6	4	2	101.8	29.4	-	-	101.8
41	91	PRDX2	**P A G W K P G S D T**	**I K P N V D D S K E**	20	F	37	26	11	57.0	9.0	-	-	57.0
			A G W K P G S D T	I K P N V D D S K E	19		1	1	0	87.2	-			87.2
42	33	MYO5A	**I T E A E D L P L R**	**M E E P S E K K A P**	20	F	10	4	6	212.0	131.6	42.6	-	127.3
			P L R	M E E P S E K K A P	13		3	2	1	-	-	-	-	
43	9	NF2		L C I G N H	6		3	2	1	-	-	-	-	
46	69	CRYBA1	**E T Q T V Q Q E L K**	**S L P T T K M A Q T**	20	F	3	1	2	432.2	-	428.0	5.0	430.1
			T V Q Q E L K	S L P T T K M A Q T	17		3	1	2	261.1	-	134.2	-	197.6
			V Q Q E L K	S L P T T K M A Q T	16		1	1	0	240.1	-			240.1
			Q E L K	S L P T T K M A Q T	14		4	2	2	201.2	-	72.8	-	137.0
			E L K	S L P T T K M A Q T	13		3	2	1	252.2	31.0	39.7	-	145.9
				P T T K M A Q T	8		1	0	1			-	-	
47	86	JUN	**L R A K N S D L L T**	**S P D V G L L K L A**	20	F	60	49	11	234.8	5.1	245.7	9.0	240.3
			N S D L L T	S P D V G L L K L A	16		6	4	2	118.4	28.5	68.9	-	93.7
			S D L L T	S P D V G L L K L A	15		10	7	3	113.8	16.4	26.9	2.8	70.3
			L L T	S P D V G L L K L A	13		2	1	1	187.6	-	81.9	-	134.7

Table S9 All identified both-capped (uncleaved) peptides.

A Normal condition

ID No.	SB No.	Protein	Ac- 1 2 3 4 5 6 7 8 9 10	11 12 13 14 15 16 17 18 19 20 -DKP[*1]	length	FL (F) or not	#sp.[*2] total	#sp.[*2] C1	#sp.[*2] C2	C1[*3] k_{cat}/K_m	C1[*3] SE	C2 k_{cat}/K_m	C2 SE	Ave. of C1 and C2
			L T	S P D V G L L K L A	12		2	1	1	-	-	-	-	
			T	S P D V G L L K L A	11		2	1	1	-	-	-	-	
				S P D V G L L K L A	10		5	2	3	-	-	0.9	-	0.9
				P D V G L L K L A	9		16	10	6	51.0	20.9	276.8	-	163.9
				V G L L K L A	7		2	1	1	151.4	-	-	-	151.4
48	86	JUN	**G G G G Y S A S L H**	**S E P P V Y A N L S**	20	F	37	25	12	271.6	11.8	77.3	-	174.5
			G G G Y S A S L H	S E P P V Y A N L S	19		1	1	0	-	-			
			L H	S E P P V Y A N L S	12		5	3	2	23.4	-	-	-	23.4
				V Y A N L S	6		1	1	0	-	-			
49	64	FOS	**L V S S V A P S Q T**	**R A P H P Y G L P T**	20	F	52	31	21	388.2	-	119.3	-	253.7
			V S S V A P S Q T	R A P H P Y G L P T	19		16	10	6	-	-	-	-	
			T	R A P H P Y G L P T	11		20	10	10	-	-	-	-	
52	32	HBB	**M V H L T P E E K**	**S A V T A L W G K V**	19	F	6	0	6			381.7	7.9	381.7
			P E E K	S A V T A L W G K V	14		1	1	0	341.8	-			341.8
				S A V T A L W G K V	10		6	4	2	98.9	-	-	-	98.9
				A V T A L W G K V	9		9	6	3	-	-	-	-	
				V T A L W G K V	8		6	2	4	-	-	-	-	
				A L W G K V	6		2	2	0	-	-			
53	19	IL1A	F	L S N V K Y N F M R	11		2	1	1	-	-	-	-	
				N V K Y N F M R	8		2	2	0	-	-			
				V K Y N F M R	7		2	1	1	97.0	-	-	-	97.0
54	43	ITGB3	P L Y	K E A T S T F T N I	13		4	1	3	80.1	-	93.9	53.2	87.0
55	62	ITGB7	P L Y	K S A I T T T I N P	13		7	4	3	124.9	46.0	134.4	-	129.7
			Y	K S A I T T T I N P	11		2	2	0	104.7	-			104.7
57	2	ITGB2	P L F	K S A T T T V M N P	13		4	2	2	346.5	-	337.3	-	341.9
			F	K S A T T T V M N P	11		1	1	0	-	-			
58	63	ITGB1	**E F A K F E K E K M**	**N A K W D T G E N P**	20	F	2	1	1	7.8	-	-	-	7.8
				N A K W D T G E N P	10		2	1	1	-	-	-	-	
				A K W D T G E N P	9		3	3	0	-	-			
60	24	PTPRN	V R Q H A R Q Q D	K E R L A A L G P E	19		19	14	5	162.7	109.8	-	-	162.7
			H A R Q Q D	K E R L A A L G P E	16		8	6	2	-	-	27.6	-	27.6
			Q Q D	K E R L A A L G P E	13		3	1	2	-	-	-	-	
			Q D	K E R L A A L G P E	12		8	5	3	38.5	-	-	-	38.5
			D	K E R L A A L G P E	11		12	6	6	-	-	-	-	
61	24	PTPRN	**P E P S R V S S V S**	**S Q F S D A A Q A S**	20	F	13	9	4	132.8	30.6	-	-	132.8
			P S R V S S V S	S Q F S D A A Q A S	18		1	1	0	-	-			
62	84	BID	**G N R S S H S R L G**	**R I E A D S E S Q E**	20	F	6	4	2	-	-	-	-	
63	26	BAX	**I M K T G A L L L Q**	**G F I Q D R A G R M**	20	F	2	0	2			318.8	61.6	318.8
			Q	G F I Q D R A G R M	11		1	1	0	-	-			
				G F I Q D R A G R M	10		24	14	10	-	-	-	-	
				D R A G R M	6		8	4	4	-	-	-	-	
64	70	IGFBP4	**R D R S T S G G K M**	**K V N G A P R E D A**	20	F	49	29	20	101.6	31.1	-	-	101.6
			D R S T S G G K M	K V N G A P R E D A	19		33	20	13	85.5	26.1	1.5	-	43.5
			R S T S G G K M	K V N G A P R E D A	18		43	31	12	52.5	25.6	-	-	52.5
			S T S G G K M	K V N G A P R E D A	17		9	5	4	-	-	-	-	
			T S G G K M	K V N G A P R E D A	16		5	4	1	-	-	-	-	
65	40	HBA1	**V L S P A D K T N V**	**K A A W G K V G A H**	20	F	23	17	6	188.5	15.7	77.4	23.2	133.0
			T N V	K A A W G K V G A H	13		5	3	2	236.7	9.8	103.5	-	170.1
			N V	K A A W G K V G A H	12		11	8	3	50.8	12.1	-	-	50.8
			V	K A A W G K V G A H	11		10	4	6	44.7	-	5.3	-	25.0
				A A W G K V G A H	9		6	3	3	-	-	-	-	
				A W G K V G A H	8		8	5	3	-	-	-	-	
				W G K V G A H	7		3	1	2	-	-	-	-	
66	50	EGFR	R R L L	Q E R E L V E P L T	14		1	0	1			-	-	
			R L L	Q E R E L V E P L T	13		2	1	1	-	-	-	-	
			L L	Q E R E L V E P L T	12		6	4	2	-	-	-	-	
			L	Q E R E L V E P L T	11		1	1	0	-	-			
				E R E L V E P L T	9		11	7	4	-	-	-	-	
67	50	EGFR	**V Q P T C V N S T F**	**D S P A H W A Q K G**	20	F	21	14	7	292.8	20.5	237.7	-	265.3
			P T C V N S T F	D S P A H W A Q K G	18		6	5	1	153.0	14.5	-	-	153.0
			V N S T F	D S P A H W A Q K G	15		6	4	2	-	-	-	-	
			N S T F	D S P A H W A Q K G	14		1	0	1			-	-	
			S T F	D S P A H W A Q K G	13		1	0	1			-	-	
			T F	D S P A H W A Q K G	12		2	2	0	-	-			
			F	D S P A H W A Q K G	11		1	1	0	-	-			
68	73	DMD	**V M E T V T T V T T**	**R E Q I L V K H A Q**	20	F	1	1	0	330.8	-			330.8
			T V T T	R E Q I L V K H A Q	14		1	0	1			-	-	
			V T T	R E Q I L V K H A Q	13		4	4	0	35.8	17.4			35.8
			T T	R E Q I L V K H A Q	12		5	1	4	-	-	-	-	
			T	R E Q I L V K H A Q	11		4	2	2	-	-	-	-	
				R E Q I L V K H A Q	10		3	1	2	-	-	-	-	
				E Q I L V K H A Q	9		2	0	2			-	-	
				Q I L V K H A Q	8		6	2	4	-	-	-	-	
				I L V K H A Q	7		12	5	7	138.9	-	-	-	138.9

Table S9 All identified both-capped (uncleaved) peptides.

A Normal condition

ID No.	SB No.	Protein	Ac- 1 2 3 4 5 6 7 8 9 10	11 12 13 14 15 16 17 18 19 20 -DKP[*1]	length	FL (F) or not	#sp.[*2] total	#sp. C1	#sp. C2	C1[*3] k_{cat}/K_m	C1 SE	C2 k_{cat}/K_m	C2 SE	Ave. of C1 and C2
69	36	SNCA	**V V H G V A T V A E**	**K T K E Q V T N V G**	20	F	64	41	23	92.5	6.3	33.8	13.4	63.1
			V A T V A E	K T K E Q V T N V G	16		1	1	0	55.6	-			55.6
			T V A E	K T K E Q V T N V G	14		1	1	0	-	-			
			V A E	K T K E Q V T N V G	13		2	1	1	-	-	-	-	
			A E	K T K E Q V T N V G	12		1	1	0	-	-			
70	23	IGFBP5	R K	K L T Q S K F V G G	12		1	1	0	-	-			
				L T Q S K F V G G	9		11	5	6	112.7	75.2	-	-	112.7
				T Q S K F V G G	8		2	2	0	-	-			
				Q S K F V G G	7		1	0	1			-	-	
				S K F V G G	6		6	5	1	43.3	39.5	-	-	43.3
71	22	CTTN	**T V P V E A V T S K**	**T S N I R A N F E N**	20	F	15	8	7	216.8	10.6	106.9	6.6	161.9
			V P V E A V T S K	T S N I R A N F E N	19		1	1	0	167.9	-			167.9
			P V E A V T S K	T S N I R A N F E N	18		21	11	10	130.3	13.9	51.4	19.1	90.8
			V E A V T S K	T S N I R A N F E N	17		5	2	3	89.0	-	-	-	89.0
			E A V T S K	T S N I R A N F E N	16		1	0	1			-	-	
				T S N I R A N F E N	10		3	2	1	-	-	-	-	
				N I R A N F E N	8		1	0	1			-	-	
				I R A N F E N	7		1	0	1			-	-	
72	90	FLNA	**L R S Q Q L A P Q Y**	**T Y A Q G G Q Q T W**	20	F	7	5	2	-	-	-	-	
			Y	T Y A Q G G Q Q T W	11		1	0	1			-	-	
73	53	TP53	E T F S D L W K L	L P E N N V L S P L	19		4	1	3	-	-	207.1	-	207.1
75	95	CRYBB3	**P E Q A A A N K S H**	**G G L G G S Y K V T**	20	F	15	8	7	156.3	27.1	17.9	-	87.1
			A A A N K S H	G G L G G S Y K V T	17		7	6	1	47.6	2.8	-	-	47.6
			A N K S H	G G L G G S Y K V T	15		2	1	1	111.5	-	-	-	111.5
			N K S H	G G L G G S Y K V T	14		5	4	1	-	-	-	-	
				G G L G G S Y K V T	10		20	13	7	57.8	13.4	-	-	57.8
				G L G G S Y K V T	9		5	2	3	12.1	-	-	-	12.1
				L G G S Y K V T	8		3	2	1	187.6	19.0	-	-	187.6
				G G S Y K V T	7		1	1	0	-	-			
76	88	CRYBB1	**S V P R P A A K V G**	**E L P P G S Y R L V**	20	F	73	40	33	146.6	8.3	45.1	5.0	95.9
			V P R P A A K V G	E L P P G S Y R L V	19		1	1	0	128.4	-			128.4
			P A A K V G	E L P P G S Y R L V	16		43	26	17	146.6	16.1	46.2	-	96.4
			A K V G	E L P P G S Y R L V	14		1	1	0	-	-			
				P G S Y R L V	7		1	0	1			-	-	
77	94	CRYBB1	**A K A S A T A A V N**	**P G P D G K G K A G**	20	F	76	49	27	64.2	18.2	424.3	-	244.3
			A S A T A A V N	P G P D G K G K A G	18		6	5	1	24.0	11.7	-	-	24.0
			S A T A A V N	P G P D G K G K A G	17		5	4	1	-	-	-	-	
			A T A A V N	P G P D G K G K A G	16		2	1	1	-	-	-	-	
79	21	CAPN11	**A A R L Q R D R L R**	**A E G V G E H N N A**	20	F	7	6	1	-	-	-	-	
			R D R L R	A E G V G E H N N A	15		1	0	1			-	-	
			D R L R	A E G V G E H N N A	14		9	5	4	-	-	-	-	
			R L R	A E G V G E H N N A	13		2	0	2			-	-	
			L R	A E G V G E H N N A	12		23	14	9	-	-	-	-	
			R	A E G V G E H N N A	11		12	8	4	223.0	-	188.5	-	205.8
81	17	CAPNS1	**P P P P R S H V S M**	**V D P N E S E E V R**	20	F	22	15	7	50.0	-	-	-	50.0
			P P P R S H V S M	V D P N E S E E V R	19		5	1	4	-	-	-	-	
			P P R S H V S M	V D P N E S E E V R	18		2	2	0	-	-			
			P R S H V S M	V D P N E S E E V R	17		1	1	0	146.4	-			146.4
83	20	CAPN1	**I T P V Y C T G V S**	**A Q V Q K Q R A R E**	20	F	15	14	1	299.7	34.3	9.9	-	154.8
			T G V S	A Q V Q K Q R A R E	14		1	1	0	-	-			
84	20	CAPN1	**V Q K Q R A R E L G**	**L G R H E N A I K Y**	20	F	15	11	4	427.4	9.0	472.5	24.5	449.9
			R A R E L G	L G R H E N A I K Y	16		8	6	2	229.4	135.6	-	-	229.4
			A R E L G	L G R H E N A I K Y	15		2	1	1	-	-	-	-	
			E L G	L G R H E N A I K Y	13		1	1	0	101.5	-			101.5
85	1	CAPN2	D R E A A E G L G	S H E R A I K Y L N	19		1	1	0	228.6	-			228.6
			R E A A E G L G	S H E R A I K Y L N	18		1	1	0	148.7	-			148.7
			E A A E G L G	S H E R A I K Y L N	17		2	1	1	254.1	-	-	-	254.1
			A A E G L G	S H E R A I K Y L N	16		2	2	0	-	-			
86	11	NFKBIA	**E Y E Q M V K E L Q**	**E I R L E P Q E V P**	20	F	3	0	3			378.8	6.8	378.8
			M V K E L Q	E I R L E P Q E V P	16		11	5	6	219.5	17.0	56.1	20.4	137.8
			V K E L Q	E I R L E P Q E V P	15		2	2	0	164.5	21.6			164.5
				E I R L E P Q E V P	10		2	2	0	-	-			
87	37	VIM	**L L L R R V V A G S**	**P S V R S T S V R Y**	20	F	14	9	5	-	-	-	-	
			L L R R V V A G S	P S V R S T S V R Y	19		33	28	5	51.5	9.4	44.4	-	47.9
			L R R V V A G S	P S V R S T S V R Y	18		1	1	0	166.8	-			166.8
			R V V A G S	P S V R S T S V R Y	16		3	3	0	-	-			
			V V A G S	P S V R S T S V R Y	15		4	3	1	-	-	-	-	
				P S V R S T S V R Y	10		3	2	1	-	-	-	-	
				R S T S V R Y	7		3	1	2	-	-	-	-	
88	81	TLN1	**E Q V L K V G T V S**	**R K N K Y P Q S Q V**	20	F	2	2	0	152.2	-			152.2
			V L K V G T V S	R K N K Y P Q S Q V	18		2	1	1	-	-	-	-	
			V G T V S	R K N K Y P Q S Q V	15		2	1	1	-	-	-	-	
			G T V S	R K N K Y P Q S Q V	14		2	1	1	-	-	-	-	
				N K Y P Q S Q V	8		1	1	0	37.9	-			37.9

Table S9 All identified both-capped (uncleaved) peptides.

A Normal condition

ID No.	SB No.	Protein	Ac- 1 2 3 4 5 6 7 8 9 10	11 12 13 14 15 16 17 18 19 20 -DKP[*1]	length	FL (F) or not	#sp.[*2] total	#sp.[*2] C1	#sp.[*2] C2	C1[*3] k_{cat}/K_m	C1[*3] SE	C2 k_{cat}/K_m	C2 SE	Ave. of C1 and C2
89	61	SPTAN1	**S Y D V T E E Q D Q**	**R Q P V M A M Q G V**	20	F	3	2	1	89.4	-	181.2	-	135.3
				R Q P V M A M Q G V	10		4	3	1	-	-	-	-	
90	15	RYR1	**R T P M L M A A P A**	**T K A K P A P K Q F**	20	F	128	89	39	314.2	5.1	301.5	5.6	307.8
			P M L M A A P A	T K A K P A P K Q F	18		2	2	0	391.4	2.2			391.4
			L M A A P A	T K A K P A P K Q F	16		5	2	3	-	-	-	-	
91	62	ITGB7	**T D I Q A K S W K N**	**P Y N L I P T N T S**	20	F	33	14	19	332.8	7.7	179.1	12.5	256.0
			A K S W K N	P Y N L I P T N T S	16		3	1	2	53.1	-	-	-	53.1
92	90	FLNA	**G Q Q Q A S Y R T L**	**W Y T Q Q P Q A G L**	20	F	15	10	5	364.5	-	223.9	-	294.2
			Q Q A S Y R T L	W Y T Q Q P Q A G L	18		2	1	1	-	-	-	-	
			Q A S Y R T L	W Y T Q Q P Q A G L	17		8	3	5	-	-	-	-	
			A S Y R T L	W Y T Q Q P Q A G L	16		9	5	4	-	-	-	-	
93	53	TP53	**N S N F E T P E L Q**	**L L P K S W L L V D**	20	F	7	1	6	-	-	170.2	68.4	170.2
				L L P K S W L L V D	10		12	8	4	81.2	-	-	-	81.2
				L P K S W L L V D	9		1	1	0	77.1	-			77.1
				P K S W L L V D	8		2	2	0	64.0	-			64.0
94	20	CAPN1	**E R H Q R K G Q L V**	**Y G K L I E A R N A**	20	F	9	9	0	218.0	10.8			218.0
			R H Q R K G Q L V	Y G K L I E A R N A	19		3	3	0	239.6	-			239.6
			E R H Q R K G Q	L V Y G K L I E A R	18		1	1	0	235.7	-			235.7
			R K G Q L V	Y G K L I E A R N A	16		3	3	0	128.1	32.9			128.1
			G Q L V	Y G K L I E A R N A	14		16	10	6	42.5	22.1	86.7	-	64.6
			L V	Y G K L I E A R N A	12		109	60	49	48.2	6.9	2.3	-	25.2
			V	Y G K L I E A R N A	11		96	49	47	61.8	-	-	-	61.8
				Y G K L I E A R N A	10		10	6	4	73.0	-	-	-	73.0
				G K L I E A R N A	9		10	7	3	135.0	53.6	-	-	135.0

*1 Numbers 1-20 correspond to the positions of the originally designed full-length (FL) oligopeptides, which are indicated in boldface type.
*2 Abbreviations used: Ave., average; #sp., number of spectra identified; for protein names, see Table S2.
*3 "-" indicates that the value could not be calculated regardless of the existence of one or more spectra, whereas blank means no spectrum was available.

B Stringent condition

Table S9 All identified both-capped (uncleaved) peptides.

ID No.	SB No.	Protein	Ac- 1 2 3 4 5 6 7 8 9 10	11 12 13 14 15 16 17 18 19 20 -DKP*1	length	FL (F) or not	#sp.*2 total	#sp.*2 C1	#sp.*2 C2	C1*3 k_{cat}/K_m	C1*3 SE	C2 k_{cat}/K_m	C2 SE	Ave. of C1 and C2
2	44	COPB1	**G T Y A T Q S A L S**	**S S R P T K K E E D**	20	F	4	2	2	397.2	-	180.2	27.3	288.7
			S A L S	S S R P T K K E E D	14		1	0	1			-	-	
4	29	TNNT3	**E E E K P R P K L T**	**A P K I P E G E K V**	20	F	14	12	2	143.7	27.7	-	-	143.7
5	45	MBP	**S Q R S K Y L A S A**	**S T M D H A R H G F**	20	F	8	7	1	162.7	29.9	-	-	162.7
			R S K Y L A S A	S T M D H A R H G F	18		1	1	0	6.3	-			6.3
			S K Y L A S A	S T M D H A R H G F	17		9	5	4	151.2	38.3	-	-	151.2
			L A S A	S T M D H A R H G F	14		18	11	7	-	-	-	-	
			A S A	S T M D H A R H G F	13		13	7	6	-	-	168.6	-	168.6
			S A	S T M D H A R H G F	12		3	2	1	-	-	-	-	
			A	S T M D H A R H G F	11		1	1	0	-	-			
6	45	MBP	**P V V H F F K N I V**	**T P R T P P P S Q G**	20	F	79	38	41	301.1	10.6	275.6	8.8	288.3
			F F K N I V	T P R T P P P S Q G	16		1	0	1			68.5	-	68.5
			F K N I V	T P R T P P P S Q G	15		1	0	1			-	-	
8	55	PRKCA	A K L G P A G N K	V I S P S E D R K Q	19		3	2	1	-	-	-	-	
			P A G N K	V I S P S E D R K Q	15		2	2	0	21.1	-			21.1
			N K	V I S P S E D R K Q	12		1	1	0	14.8	-			14.8
9	89	PRKCG	**Y E R V R M G P S S**	**S P I P S P S P S P**	20	F	72	47	25	56.5	-	111.4	-	83.9
10	39	PRKCB	**R Q K F E R A K I G**	**Q G T K A P E E K T**	20	F	10	8	2	40.6	12.8	-	-	40.6
			Q K F E R A K I G	Q G T K A P E E K T	19		3	3	0	-	-			
			F E R A K I G	Q G T K A P E E K T	17		5	0	5			-	-	
			E R A K I G	Q G T K A P E E K T	16		1	0	1			-	-	
			A K I G	Q G T K A P E E K T	14		2	1	1	-	-	-	-	
			G	Q G T K A P E E K T	11		1	0	1			-	-	
				Q G T K A P E E K T	10		3	2	1	-	-	-	-	
				G T K A P E E K T	9		3	2	1	45.6	-	-	-	45.6
11	8	PLCB1	**T P K P P S Q A L H**	**S Q P A P G S V K A**	20	F	11	7	4	184.4	42.1	122.9	-	153.7
			P S Q A L H	S Q P A P G S V K A	16		5	3	2	177.6	82.1	51.3	-	114.5
			A L H	S Q P A P G S V K A	13		9	5	4	281.7	21.6	155.4	16.3	218.6
				P A P G S V K A	8		2	2	0	133.1	131.7			133.1
12	65	PARP1	**S A A S A P A A V H**	**S G P P D K P L S N**	20	F	71	48	23	187.9	6.8	36.1	11.8	112.0
			P A A V H	S G P P D K P L S N	15		1	1	0	149.7	-			149.7
			V H	S G P P D K P L S N	12		4	3	1	-	-	-	-	
13	65	PARP1	**Q D E E A V K K L T**	**V N P G T K S K L P**	20	F	2	1	1	414.2	-	-	-	414.2
			E A V K K L T	V N P G T K S K L P	17		2	1	1	186.9	-	-	-	186.9
14	60	INS	L V E	A L Y L V C G E R G	13		4	4	0	-	-			
			V E	A L Y L V C G E R G	12		7	7	0	-	-			
			E	A L Y L V C G E R G	11		2	2	0	-	-			
				A L Y L V C G E R G	10		2	2	0	-	-			
				Y L V C G E R G	8		1	1	0	-	-			
15	41	GNRH1	**E G C S S Q H W S Y**	**G L R P G G K R N T**	20	F	5	4	1	111.6	38.6	-	-	111.6
			S S Q H W S Y	G L R P G G K R N T	17		1	1	0	-	-			
17	34	NTS	L Y	E N K P R R P Y I L	12		1	1	0	-	-			
19	54	GCG	D F	V Q W L M N T K R N	12		13	8	5	-	-	166.0	39.9	166.0
20	54	GCG	**R A Q D F V Q W L M**	**N T K R N R N N I A**	20	F	1	0	1			313.2	-	313.2
			D F V Q W L M	N T K R N R N N I A	17		1	0	1			292.3	-	292.3
21	10	NEFM	**V V A A K K S P V K**	**A T A P E L K E E E**	20	F	11	8	3	278.2	38.4	-	-	278.2
22	42	CASP9	**L T P V V L R P E I**	**R K P E V L R P E T**	20	F	79	53	26	71.0	8.8	42.2	14.0	56.6
			P E I	R K P E V L R P E T	13		1	1	0	-	-			
24	48	ANXA1	**E E Q E Y V Q T V K**	**S S K G G P G S A V**	20	F	2	2	0	421.0	3.1			421.0
			Y V Q T V K	S S K G G P G S A V	16		2	1	1	-	-	-	-	
			V Q T V K	S S K G G P G S A V	15		2	2	0	37.9	-			37.9
			Q T V K	S S K G G P G S A V	14		2	2	0	-	-			
			T V K	S S K G G P G S A V	13		1	0	1			-	-	
25	31	ACTB	**R A V F P S I V G R**	**P R H Q G V M V G M**	20	F	3	2	1	-	-	-	-	
			A V F P S I V G R	P R H Q G V M V G M	19		8	7	1	-	-	-	-	
			R A V F P S I V	G R P R H Q G V M V	18		1	0	1			-	-	
			A V F P S I V	G R P R H Q G V M V	17		1	0	1			-	-	
				P R H Q G V M V G M	10		1	1	0	-	-			
26	46	MIP	**S I S E R L S V L K**	**G A K P D V S N G Q**	20	F	12	8	4	270.0	10.6	148.3	25.1	209.1
			I S E R L S V L K	G A K P D V S N G Q	19		1	1	0	157.3	-			157.3
			L S V L K	G A K P D V S N G Q	15		1	0	1			7.3	-	7.3
28	83	CDKN2D	**D V Q E V R R L L H**	**R E L V H P D A L N**	20	F	3	2	1	-	-	-	-	
				D V Q E V R R	7		1	1	0	-	-			
				V Q E V R R	6		1	0	1			-	-	
29	83	CDKN2D	**A I A L E L L K Q G**	**A S P N V Q D T S G**	20	F	42	26	16	344.5	5.9	161.4	12.2	253.0
			I A L E L L K Q G	A S P N V Q D T S G	19		11	6	5	62.0	17.5	-	-	62.0
			A L E L L K Q G	A S P N V Q D T S G	18		5	3	2	-	-	-	-	
			L E L L K Q G	A S P N V Q D T S G	17		1	0	1			-	-	
30	37	VIM	**Y R R M F G G S G T**	**S S R P S S N R S Y**	20	F	5	5	0	-	-			
			R R M F G G S G T	S S R P S S N R S Y	19		2	2	0	-	-			
			R M F G G S G T	S S R P S S N R S Y	18		8	6	2	-	-	-	-	
			M F G G S G T	S S R P S S N R S Y	17		8	5	3	-	-	-	-	
			G S G T	S S R P S S N R S Y	14		1	1	0	-	-			

Table S9 All identified both-capped (uncleaved) peptides.

B Stringent condition

ID No.	SB No.	Protein	Ac- 1 2 3 4 5 6 7 8 9 10	11 12 13 14 15 16 17 18 19 20 -DKP[*1]	length	FL (F) or not	#sp.[*2] total	#sp. C1	#sp. C2	C1[*3] k_{cat}/K_m	C1 SE	C2 k_{cat}/K_m	C2 SE	Ave. of C1 and C2
				S S R P S S N R S Y	10		1	1	0	-	-			
				P S S N R S Y	7		1	0	1			-	-	
31	37	VIM	S S A V R L	R S S V P G V R L L	16		32	21	11	-	-	-	-	
			S A V R L	R S S V P G V R L L	15		46	28	18	-	-	-	-	
			A V R L	R S S V P G V R L L	14		1	0	1			-	-	
			L	R S S V P G V R L L	11		4	1	3	-	-	-	-	
				S S V P G V R L L	9		1	0	1			-	-	
32	37	VIM	**Q I D V D V S K P D**	**L T A A L R D V R Q**	20	F	8	2	6	184.2	92.5	236.1	45.2	210.2
			I D V D V S K P D	L T A A L R D V R Q	19		2	0	2			123.3	39.5	123.3
			P D	L T A A L R D V R Q	12		12	11	1	-	-	-	-	
			D	L T A A L R D V R Q	11		1	1	0	-	-			
				T A A L R D V R Q	9		5	2	3	-	-	-	-	
				A A L R D V R Q	8		1	0	1			-	-	
33	59	TH	**G F R R A V S E L D**	**A K Q A E A I M S P**	20	F	1	0	1			-	-	
34	81	TLN1		Q Q Y N R V G K V E	10		2	0	2			-	-	
				Q Y N R V G K V E	9		1	0	1			-	-	
				Y N R V G K V E	8		1	0	1			-	-	
35	78	SPTB	A F E K S T	A S W A E R F A A L	16		1	0	1			334.4	-	334.4
			S T	A S W A E R F A A L	12		23	15	8	-	-	-	-	
			T	A S W A E R F A A L	11		14	5	9	-	-	-	-	
				A S W A E R F A A L	10		11	4	7	-	-	-	-	
				S W A E R F A A L	9		14	10	4	119.6	-	-	-	119.6
				W A E R F A A L	8		3	2	1	-	-	-	-	
36	61	SPTAN1	**V Q A V Q Q Q E V Y**	**G M M P R D E T D S**	20	F	5	4	1	-	-	-	-	
			E V Y	G M M P R D E T D S	13		2	1	1	-	-	-	-	
			V Y	G M M P R D E T D S	12		15	7	8	-	-	-	-	
37	15	RYR1	**F K A K K A A M M T**	**Q P P A T P A L P R**	20	F	26	17	9	388.3	7.0	305.8	6.6	347.0
			A K K A A M M T	Q P P A T P A L P R	18		8	5	3	98.3	18.0	-	-	98.3
			M M T	Q P P A T P A L P R	13		26	16	10	-	-	-	-	
				P P A T P A L P R	9		2	1	1	-	-	-	-	
38	15	RYR1	T R K I S	Q T A Q T Y D P R E	15		4	2	2	64.6	29.6	-	-	64.6
			R K I S	Q T A Q T Y D P R E	14		2	1	1	-	-	-	-	
			S	Q T A Q T Y D P R E	11		1	0	1			-	-	
				T A Q T Y D P R E	9		1	0	1			-	-	
39	72	GRIN2A	**Q D W S Q N N A L Q**	**F Q K N K L R I N R**	20	F	1	0	1			57.1	-	57.1
40	72	GRIN2A	**L E G N L Y G S L F**	**S V P S S K L L G N**	20	F	2	1	1	377.7	-	375.7	-	376.7
				P S S K L L G N	8		2	1	1	-	-	-	-	
41	91	PRDX2	**P A G W K P G S D T**	**I K P N V D D S K E**	20	F	22	15	7	56.8	12.0	-	-	56.8
42	33	MYO5A	**I T E A E D L P L R**	**M E E P S E K K A P**	20	F	7	2	5	76.8	20.9	42.6	-	59.7
			P L R	M E E P S E K K A P	13		1	0	1			-	-	
43	9	NF2		L C I G N H	6		1	1	0	-	-			
46	69	CRYBA1	**E T Q T V Q Q E L K**	**S L P T T K M A Q T**	20	F	1	0	1			423.0	-	423.0
			E L K	S L P T T K M A Q T	13		3	2	1	252.2	31.0	39.7	-	145.9
				P T T K M A Q T	8		1	0	1			-	-	
47	86	JUN	**L R A K N S D L L T**	**S P D V G L L K L A**	20	F	53	42	11	240.9	5.0	245.7	9.0	243.3
			N S D L L T	S P D V G L L K L A	16		2	2	0	95.7	60.1			95.7
			S D L L T	S P D V G L L K L A	15		4	2	2	145.1	25.9	26.9	2.8	86.0
			L L T	S P D V G L L K L A	13		1	0	1			81.9	-	81.9
			T	S P D V G L L K L A	11		1	0	1			-	-	
				S P D V G L L K L A	10		2	1	1	-	-	0.9	-	0.9
				P D V G L L K L A	9		8	5	3	67.1	23.2	-	-	67.1
				V G L L K L A	7		1	0	1			-	-	
48	86	JUN	**G G G G Y S A S L H**	**S E P P V Y A N L S**	20	F	29	17	12	271.6	11.8	77.3	-	174.5
			L H	S E P P V Y A N L S	12		1	1	0	23.4	-			23.4
49	64	FOS	**L V S S V A P S Q T**	**R A P H P Y G L P T**	20	F	30	13	17	-	-	119.3	-	119.3
			V S S V A P S Q T	R A P H P Y G L P T	19		11	5	6	-	-	-	-	
			T	R A P H P Y G L P T	11		11	4	7	-	-	-	-	
52	32	HBB	**M V H L T P E E K**	**S A V T A L W G K V**	19	F	3	0	3			377.7	8.5	377.7
			P E E K	S A V T A L W G K V	14		1	1	0	341.8	-			341.8
				S A V T A L W G K V	10		3	2	1	98.9	-	-	-	98.9
				A V T A L W G K V	9		1	1	0	-	-			
				V T A L W G K V	8		2	1	1	-	-	-	-	
55	62	ITGB7	Y	K S A I T T T I N P	11		2	2	0	104.7	-			104.7
58	63	ITGB1	**E F A K F E K E K M**	**N A K W D T G E N P**	20	F	2	1	1	7.8	-	-	-	7.8
				A K W D T G E N P	9		1	1	0	-	-			
60	24	PTPRN	V R Q H A R Q Q D	K E R L A A L G P E	19		14	10	4	203.3	154.5	-	-	203.3
			H A R Q Q D	K E R L A A L G P E	16		4	2	2	-	-	27.6	-	27.6
			Q Q D	K E R L A A L G P E	13		1	0	1			-	-	
			Q D	K E R L A A L G P E	12		5	2	3	38.5	-	-	-	38.5
			D	K E R L A A L G P E	11		7	3	4	-	-	-	-	
61	24	PTPRN	**P E P S R V S S V S**	**S Q F S D A A Q A S**	20	F	11	7	4	132.8	30.6	-	-	132.8
			P S R V S S V S	S Q F S D A A Q A S	18		1	1	0	-	-			
62	84	BID	**G N R S S H S R L G**	**R I E A D S E S Q E**	20	F	4	3	1	-	-	-	-	

B Stringent condition

Table S9 All identified both-capped (uncleaved) peptides.

ID No.	SB No.	Protein	Ac- 1 2 3 4 5 6 7 8 9 10	11 12 13 14 15 16 17 18 19 20 -DKP[*1]	length	FL (F) or not	#sp.[*2] total	#sp.[*2] C 1	#sp.[*2] C 2	C1[*3] k_{cat}/K_m	C1[*3] SE	C2 k_{cat}/K_m	C2 SE	Ave. of C1 and C2
63	26	BAX	**IMKTGALLLQ**	**GFIQDRAGRM**	20	F	1	0	1			380.5	-	380.5
			Q	GFIQDRAGRM	11		1	1	0	-	-			
				GFIQDRAGRM	10		8	3	5	-	-	-	-	
64	70	IGFBP4	**RDRSTSGGKM**	**KVNGAPREDA**	20	F	17	7	10	145.2	-	-	-	145.2
			DRSTSGGKM	KVNGAPREDA	19		15	9	6	67.2	-	1.5	-	34.4
			RSTSGGKM	KVNGAPREDA	18		20	13	7	50.9	44.3	-	-	50.9
			STSGGKM	KVNGAPREDA	17		7	4	3	-	-	-	-	
			TSGGKM	KVNGAPREDA	16		2	1	1	-	-	-	-	
65	40	HBA1	**VLSPADKTNV**	**KAAWGKVGAH**	20	F	8	6	2	180.0	28.5	-	-	180.0
			TNV	KAAWGKVGAH	13		5	3	2	236.7	9.8	103.5	-	170.1
			NV	KAAWGKVGAH	12		9	6	3	34.3	5.5	-	-	34.3
			V	KAAWGKVGAH	11		7	2	5	-	-	5.3	-	5.3
				AAWGKVGAH	9		5	3	2	-	-	-	-	
				AWGKVGAH	8		5	2	3	-	-	-	-	
66	50	EGFR	LL	QERELVEPLT	12		6	4	2	-	-	-	-	
			L	QERELVEPLT	11		1	1	0	-	-			
				ERELVEPLT	9		7	4	3	-	-	-	-	
67	50	EGFR	**VQPTCVNSTF**	**DSPAHWAQKG**	20	F	6	2	4	298.1	6.3	64.0	-	181.0
			PTCVNSTF	DSPAHWAQKG	18		2	2	0	147.1	32.4			147.1
			NSTF	DSPAHWAQKG	14		1	0	1			-	-	
			F	DSPAHWAQKG	11		1	1	0	-	-			
68	73	DMD	**VMETVTTVTT**	**REQILVKHAQ**	20	F	1	1	0	330.8	-			330.8
			VTT	REQILVKHAQ	13		1	1	0	34.0	-			34.0
			TT	REQILVKHAQ	12		3	1	2	-	-	-	-	
			T	REQILVKHAQ	11		1	0	1			-	-	
				REQILVKHAQ	10		3	1	2	-	-	-	-	
				EQILVKHAQ	9		2	0	2			-	-	
				QILVKHAQ	8		4	1	3	-	-	-	-	
				ILVKHAQ	7		4	1	3	-	-	-	-	
69	36	SNCA	**VVHGVATVAE**	**KTKEQVTNVG**	20	F	17	13	4	100.9	9.7	-	-	100.9
			VAE	KTKEQVTNVG	13		1	1	0	-	-			
70	23	IGFBP5		LTQSKFVGG	9		2	1	1	187.8	-	-	-	187.8
				SKFVGG	6		2	1	1	-	-	-	-	
71	22	CTTN	**TVPVEAVTSK**	**TSNIRANFEN**	20	F	10	6	4	220.5	13.9	101.0	5.0	160.8
			PVEAVTSK	TSNIRANFEN	18		9	4	5	149.5	16.0	58.7	33.2	104.1
			VEAVTSK	TSNIRANFEN	17		3	1	2	89.0	-	-	-	89.0
				NIRANFEN	8		1	0	1			-	-	
72	90	FLNA	**LRSQQLAPQY**	**TYAQGGQQTW**	20	F	4	2	2	-	-	-	-	
73	53	TP53	ETFSDLWKL	LPENNVLSPL	19		2	0	2			207.1	-	207.1
75	95	CRYBB3	**PEQAAANKSH**	**GGLGGSYKVT**	20	F	7	3	4	174.3	58.5	17.9	-	96.1
			AAANKSH	GGLGGSYKVT	17		2	1	1	-	-	-	-	
			ANKSH	GGLGGSYKVT	15		1	1	0	111.5	-			111.5
			NKSH	GGLGGSYKVT	14		1	1	0	-	-			
				GGLGGSYKVT	10		9	3	6	68.7	-	-	-	68.7
				GLGGSYKVT	9		3	1	2	12.1	-	-	-	12.1
76	88	CRYBB1	**SVPRPAAKVG**	**ELPPGSYRLV**	20	F	59	30	29	144.9	9.0	45.1	5.0	95.0
			VPRPAAKVG	ELPPGSYRLV	19		1	1	0	128.4	-			128.4
			PAAKVG	ELPPGSYRLV	16		36	20	16	148.8	16.8	46.2	-	97.5
				PGSYRLV	7		1	0	1			-	-	
77	94	CRYBB1	**AKASATAAVN**	**PGPDGKGKAG**	20	F	61	39	22	64.2	18.2	424.3	-	244.3
			ASATAAVN	PGPDGKGKAG	18		4	3	1	23.5	20.2	-	-	23.5
			SATAAVN	PGPDGKGKAG	17		4	3	1	-	-	-	-	
			ATAAVN	PGPDGKGKAG	16		2	1	1	-	-	-	-	
79	21	CAPN11	**AARLQRDRLR**	**AEGVGEHNNA**	20	F	6	5	1	-	-	-	-	
			DRLR	AEGVGEHNNA	14		4	1	3	-	-	-	-	
			RLR	AEGVGEHNNA	13		1	0	1			-	-	
			LR	AEGVGEHNNA	12		18	10	8	-	-	-	-	
			R	AEGVGEHNNA	11		8	5	3	223.0	-	-	-	223.0
81	17	CAPNS1	**PPPPRSHVSM**	**VDPNESEEVR**	20	F	18	11	7	-	-	-	-	
			PPPRSHVSM	VDPNESEEVR	19		3	1	2	-	-	-	-	
83	20	CAPN1	**ITPVYCTGVS**	**AQVQKQRARE**	20	F	11	10	1	288.7	48.1	9.9	-	149.3
			TGVS	AQVQKQRARE	14		1	1	0	-	-			
84	20	CAPN1	**VQKQRARELG**	**LGRHENAIKY**	20	F	13	9	4	435.5	4.6	472.5	24.5	454.0
			RARELG	LGRHENAIKY	16		7	6	1	229.4	135.6	-	-	229.4
			ELG	LGRHENAIKY	13		1	1	0	101.5	-			101.5
85	1	CAPN2	DREAAEGLG	SHERAIKYLN	19		1	1	0	228.6	-			228.6
			AAEGLG	SHERAIKYLN	16		1	1	0	-	-			
86	11	NFKBIA	**EYEQMVKELQ**	**EIRLEPQEVP**	20	F	2	0	2			374.5	9.1	374.5
			MVKELQ	EIRLEPQEVP	16		2	1	1	264.0	-	-	-	264.0
			VKELQ	EIRLEPQEVP	15		1	1	0	142.9	-			142.9
87	37	VIM	**LLLRRVVAGS**	**PSVRSTSVRY**	20	F	8	3	5	-	-	-	-	
			LLRRVVAGS	PSVRSTSVRY	19		18	15	3	55.6	18.7	44.4	-	50.0
			LRRVVAGS	PSVRSTSVRY	18		1	1	0	166.8	-			166.8

B Stringent condition

Table S9 All identified both-capped (uncleaved) peptides.

ID No.	SB No.	Protein	Ac- 1 2 3 4 5 6 7 8 9 10	11 12 13 14 15 16 17 18 19 20 -DKP*1	length	FL (F) or not	#sp.*2 total	#sp.*2 C1	#sp.*2 C2	C1*3 k_{cat}/K_m	C1*3 SE	C2 k_{cat}/K_m	C2 SE	Ave. of C1 and C2
			RVVAGS	PSVRSTSVRY	16		2	2	0	-	-			
			VVAGS	PSVRSTSVRY	15		2	1	1	-	-	-	-	
				PSVRSTSVRY	10		2	1	1	-	-	-	-	
88	81	TLN1	**EQVLKVGTVS**	**RKNKYPQSQV**	20	F	1	1	0	150.4	-			150.4
			VLKVGTVS	RKNKYPQSQV	18		1	1	0	-	-			
			VGTVS	RKNKYPQSQV	15		1	0	1			-	-	
			GTVS	RKNKYPQSQV	14		2	1	1	-	-	-	-	
89	61	SPTAN1	**SYDVTEEQDQ**	**RQPVMAMQGV**	20	F	2	1	1	317.6	-	181.2	-	249.4
				RQPVMAMQGV	10		1	0	1			-	-	
90	15	RYR1	**RTPMLMAAPA**	**TKAKPAPKQF**	20	F	66	44	22	323.5	6.0	309.6	6.7	316.5
			PMLMAAPA	TKAKPAPKQF	18		1	1	0	393.7	-			393.7
			LMAAPA	TKAKPAPKQF	16		5	2	3	-	-	-	-	
91	62	ITGB7	**TDIQAKSWKN**	**PYNLIPTNTS**	20	F	21	9	12	342.5	9.2	179.4	15.1	260.9
			AKSWKN	PYNLIPTNTS	16		2	1	1	53.1	-	-	-	53.1
92	90	FLNA	**GQQQASYRTL**	**WYTQQPQAGL**	20	F	13	9	4	380.4	-	-	-	380.4
			QQASYRTL	WYTQQPQAGL	18		1	0	1			-	-	
			QASYRTL	WYTQQPQAGL	17		6	2	4	-	-	-	-	
			ASYRTL	WYTQQPQAGL	16		6	2	4	-	-	-	-	
93	53	TP53	**NSNFETPELQ**	**LLPKSWLLVD**	20	F	4	0	4			208.1	128.4	208.1
				LLPKSWLLVD	10		2	2	0	-	-			
94	20	CAPN1	**ERHQRKGQLV**	**YGKLIEARNA**	20	F	8	8	0	218.0	10.8			218.0
			RHQRKGQLV	YGKLIEARNA	19		1	1	0	185.5	-			185.5
			ERHQRKGQ	LVYGKLIEAR	18		1	1	0	235.7	-			235.7
			GQLV	YGKLIEARNA	14		7	4	3	-	-	86.7	-	86.7
			LV	YGKLIEARNA	12		73	33	40	41.5	9.6	2.3	-	21.9
			V	YGKLIEARNA	11		61	29	32	61.8	-	-	-	61.8
				YGKLIEARNA	10		4	2	2	73.0	-	-	-	73.0
				GKLIEARNA	9		1	1	0	15.6	-			15.6

*1 Numbers 1-20 correspond to the positions of the originally designed full-length (FL) oligopeptides, which are indicated in boldface type.
*2 Abbreviations used: Ave., average; #sp., number of spectra identified; for protein names, see Table S2.
*3 "-" indicates that the value could not be calculated regardless of the existence of one or more spectra, whereas blank means no spectrum was available.

Table S10 Comparison of k_{cat}/K_m values of plural sites in the same peptides

ID No.	Substrate	k_{cat}/K_m [$M^{-1}s^{-1}$] Rp site(s)	Nv site(s)	site positions Rp site(s)	Nv site(s)	1	2	3	4	5	6	7	8	9	10	11	12	13	14	15	16	17	18	19	20
31	VIM(61-80)	82	886, 160, 109, 100	10	11, 12, 6, 4	Y	V	T	R :	S	S :	A	V	R	L |	R :	S :	S	V	P	G	V	R	L	L
32	VIM(255-274)	20	623, 208, 58	10	12, 13, 11	Q	I	D	V	D	V	S	K	P	D |	L :	T :	A :	A	L	R	D	V	R	Q
38	RYR1(2834-2853)	37	84	10	13	T	E	K	K	K	T	R	K	I	S |	Q	T	A :	Q	T	Y	D	P	R	E
37	RYR1(1391-1410)	739	193	10	9	F	K	A	K	K	A	A	M	M :	T |	Q	P	P	A	T	P	A	L	P	R
13	PARP1(649-668)	39	903	10	15	Q	D	E	E	A	V	K	K	L	T |	V	N	P	G	T :	K	S	K	L	P
17	NTS(144-163)	374	64	10	11	I	P	Y	I	L	K	R	Q	L	Y |	E :	N	K	P	R	R	P	Y	I	L
86	NFKBIA(41-60)	295	241, 215	10	13, 18	E	Y	E	Q	M	V	K	E	L	Q |	E	I	R :	L	E	P	Q	E :	V	P
5	MBP(7-26)	57	31	10	12	S	Q	R	S	K	Y	L	A	S	A |	S	T :	M	D	H	A	R	H	G	F
47	JUN(53-72)	101	175	10	9	L	R	A	K	N	S	D	L	L :	T |	S	P	D	V	G	L	L	K	L	A
53	IL1A(109-128)	425	349	10	12	I	K	P	R	S	A	P	F	S	F |	L	S :	N	V	K	Y	N	F	M	R
70	IGFBP5(149-168)	558	36	13	10	A	E	A	V	K	K	D	R	R	K :	K	L	T |	Q	S	K	F	V	G	G
52	HBB(1-19)	30	833, 60	9	16, 13	M	V	H	L	T	P	E	E	K |	S	A	V	T :	A	L	W :	G	K	V	
65	HBA1(2-21)	23	94	10	11	V	L	S	P	A	D	K	T	N	V |	K :	A	A	W	G	K	V	G	A	H
39	GRIN2A(1269-1288)	399	69	10	11	Q	D	W	S	Q	N	N	A	L	Q |	F :	Q	K	N	K	L	R	I	N	R
15	GNRH1(19-38)	195	13	10	12	E	G	C	S	S	Q	H	W	S	Y |	G	L :	R	P	G	G	K	R	N	T
20	GCG(70-89)	415	60, 29	10	11, 12	R	A	Q	D	F	V	Q	W	L	M |	N :	T :	K	R	N	R	N	N	I	A
45	GAP43(31-50)	60	161, 39	10	12, 9	A	H	K	A	A	T	K	I	Q :	A |	S	F :	R	G	H	I	T	R	K	K
49	FOS(81-100)	370	195	10	11	L	V	S	S	V	A	P	S	Q	T |	R :	A	P	H	P	Y	G	L	P	T
67	EGFR(1142-1161)	310	96	10	15	V	Q	P	T	C	V	N	S	T	F |	D	S	P	A	H :	W	A	Q	K	G
68	DMD(681-700)	51	35	10	12	V	M	E	T	V	T	T	V	T	T |	R	E :	Q	I	L	V	K	H	A	Q
75	CRYBB3(8-27)	30	310	10	14	P	E	Q	A	A	A	N	K	S	H |	G	G	L	G :	G	S	Y	K	V	T
28	CDKN2D(20-39)	576	64	10	13	D	V	Q	E	V	R	R	L	L	H |	R	E	L :	V	H	P	D	A	L	N
83	CAPN1(6-25)	425	157, 168, 26, 15	10	11, 14, 6, 9	I	T	P	V	Y	C :	T	G	V :	S |	A :	Q	V	Q :	K	Q	R	A	R	E
63	BAX(19-38)	625	76, 72	10	11, 9	I	M	K	T	G	A	L	L	L :	Q |	G :	F	I	Q	D	R	A	G	R	M
	average**	259.8	189.4																						
	standard deviation	226.7	236.9																						
	n	24	37																						

*: Vertical line (|) indicates Rp sites, and colon (:) indicates Nv sites, which are indicated in the "site positions" column (k_{cat}/K_m values are listed in the order corresponding to that of the site positions).

**: The average k_{cat}/K_m values of Rp sites that had Nv site(s) in the same peptide were not significantly different from those of the Nv sites (P=0.330; t-test for two population means by the method of paired comparisons).

Table S11 Amino acid descriptors (1D) used in this study.

No.	Descriptor ID	Attribute	Ref*	A	C	D	E	F	G	H	I	K	L	M	N	P	Q	R	S	T	V	W	Y
1	ANDN920101	alpha-CH chemical shifts	AAindex	4.35	4.65	4.76	4.29	4.66	3.97	4.63	3.95	4.36	4.17	4.52	4.75	4.44	4.37	4.38	4.5	4.35	3.95	4.7	4.6
2	ARGP820101	Hydrophobicity index	AAindex	0.61	1.07	0.46	0.47	2.02	0.07	0.61	2.22	1.15	1.53	1.18	0.06	1.95	0	0.6	0.05	0.05	1.32	2.65	1.88
3	ARGP820102	Signal sequence helical potentia	AAindex	1.18	1.89	0.05	0.11	1.96	0.49	0.31	1.45	0.06	3.23	2.67	0.23	0.76	0.72	0.2	0.97	0.84	1.08	0.77	0.39
4	ARGP820103	Membrane-buried preference parameters	AAindex	1.56	1.23	0.14	0.23	2.03	0.62	0.29	1.67	0.15	2.93	2.96	0.27	0.76	0.51	0.45	0.81	0.91	1.14	1.08	0.68
5	BEGF750101	Conformational parameter of inner helix	AAindex	1	0.06	0.44	0.73	0.6	0.35	0.6	0.73	0.6	1	1	0.35	0.06	0.44	0.52	0.35	0.44	0.82	0.73	0.44
6	BEGF750102	Conformational parameter of beta-structure	AAindex	0.77	0.65	0.65	0.55	0.98	0.65	0.83	0.98	0.55	0.83	0.98	0.55	0.55	0.72	0.72	0.55	0.83	0.98	0.77	0.83
7	BEGF750103	Conformational parameter of beta-turn	AAindex	0.37	0.84	0.97	0.53	0.53	0.97	0.75	0.37	0.75	0.53	0.64	0.97	0.97	0.64	0.84	0.84	0.75	0.37	0.97	0.84
8	BHAR880101	Average flexibility indices	AAindex	0.357	0.346	0.511	0.497	0.314	0.544	0.323	0.462	0.466	0.365	0.295	0.463	0.509	0.493	0.529	0.507	0.444	0.386	0.305	0.42
9	BIGC670101	Residue volume	AAindex	52.6	68.3	68.4	84.7	113.9	36.3	91.9	102	105.1	102	97.7	75.7	73.6	89.7	109.1	54.9	71.2	85.1	135.4	116.2
10	BIOV880101	Information value for accessibility; average fraction 35%	AAindex	16	168	-78	-106	189	-13	50	151	-141	145	124	-74	-20	-73	-70	-70	-38	123	145	53
11	BIOV880102	Information value for accessibility; average fraction 23%	AAindex	44	90	-91	-139	148	-8	47	100	-188	108	121	-72	-36	-117	-68	-60	-54	117	163	22
12	BROC820101	Retention coefficient in TFA	AAindex	7.3	-9.2	-2.9	-7.1	19.2	-1.2	-2.1	6.6	-3.7	20	5.6	-5.7	5.1	-0.3	-3.6	-4.1	0.8	3.5	16.3	5.9
13	BROC820102	Retention coefficient in HFBA	AAindex	3.9	-14.3	-2.8	-7.5	14.7	-2.3	2	11	-2.5	15	4.1	-2.8	5.6	1.8	3.2	-3.5	1.1	2.1	17.8	3.8
14	BULH740101	Transfer free energy to surface	AAindex	-0.2	-0.45	-0.2	-0.3	-2.33	0	-0.12	-2.26	-0.35	-2.46	-1.47	0.08	-0.98	0.16	-0.12	-0.39	-0.52	-1.56	-2.01	-2.24
15	BULH740102	Apparent partial specific volume	AAindex	0.691	0.624	0.558	0.632	0.756	0.592	0.646	0.809	0.767	0.842	0.709	0.596	0.73	0.649	0.728	0.594	0.655	0.777	0.743	0.743
16	BUNA790101	alpha-NH chemical shifts	AAindex	8.249	8.312	8.41	8.368	8.228	8.391	8.415	8.195	8.408	8.423	8.418	8.747	0	8.411	8.274	8.38	8.236	8.436	8.094	8.183
17	BUNA790102	alpha-CH chemical shifts	AAindex	4.349	4.686	4.765	4.295	4.663	3.972	4.63	4.224	4.358	4.385	4.513	4.755	4.471	4.373	4.396	4.498	4.346	4.184	4.702	4.604
18	BUNA790103	Spin-spin coupling constants 3JHalpha-NH	AAindex	6.5	7.7	7	7	9.4	5.6	8	7	6.5	6.5	0	7.5	0	6	6.9	6.5	6.9	7	0	6.8
19	BURA740101	Normalized frequency of alpha-helix	AAindex	0.486	0.2	0.288	0.538	0.318	0.12	0.4	0.37	0.402	0.42	0.417	0.193	0.208	0.418	0.262	0.2	0.272	0.379	0.462	0.161
20	BURA740102	Normalized frequency of extended structure	AAindex	0.288	0.533	0.271	0.262	0.318	0.312	0.2	0.411	0.265	0.4	0.375	0.229	0.34	0.327	0.362	0.354	0.388	0.495	0.231	0.429
21	CHAM810101	Steric parameter	AAindex	0.52	0.62	0.76	0.68	0.7	0	0.7	1.02	0.68	0.98	0.78	0.76	0.36	0.68	0.68	0.53	0.5	0.76	0.7	0.7
22	CHAM820101	Polarizability parameter	AAindex	0.046	0.128	0.105	0.151	0.29	0	0.23	0.186	0.219	0.186	0.221	0.134	0.131	0.18	0.291	0.062	0.108	0.14	0.409	0.298
23	CHAM820102	Free energy of solution in water, kcal/mole	AAindex	-1.03	0.731	4.53	-0.525	-2.24	0	0.791	1.07	0.656	0	1.06	2.06	-0.524	1.77	0	0	1.6	0.8031579	4.91	0.401
24	CHAM830101	The Chou-Fasman parameter of the coil conformation	AAindex	0.71	1.19	1.21	0.84	0.71	1.52	1.07	0.66	0.99	0.69	0.59	1.37	1.61	0.87	1.06	1.34	1.08	0.63	0.76	1.07
25	CHAM830102	A parameter defined from the residuals obtained from the best correlation of the Chou-Fasman parameter of beta-sheet	AAindex	0.124	-0.105	0.083	0.104	0	0.138	0.23	-0.052	-0.258	0.032	0.015	0.048	0.225	-0.245	0.289	0.166	0.158	0.0820526	0.094	0.513
26	CHAM830103	The number of atoms in the side chain labelled 1+1	AAindex	0	1	1	1	1	0	1	2	1	1	1	1	0	1	1	1	2	2	1	1
27	CHAM830104	The number of atoms in the side chain labelled 2+1	AAindex	0	0	1	1	1	0	1	1	1	2	1	1	0	1	1	0	0	0	1	1
28	CHAM830105	The number of atoms in the side chain labelled 3+1	AAindex	0	0	0	1	1	0	1	0	1	0	1	0	0	1	1	0	0	0	1.5	1
29	CHAM830106	The number of bonds in the longest chain	AAindex	0	1	2	3	4	0	3	2	4	2	3	2	0	3	5	1	1	1	5	5
30	CHAM830107	A parameter of charge transfer capability	AAindex	0	0	1	1	0	1	0	0	0	0	0	1	0	0	0	0	0	0	0	0
31	CHAM830108	A parameter of charge transfer donor capability	AAindex	0	1	0	0	1	0	1	0	1	0	1	1	0	1	1	0	0	0	1	1
32	CHOC750101	Average volume of buried residue	AAindex	91.5	117.7	124.5	155.1	203.4	66.4	167.3	168.8	171.3	167.9	170.8	135.2	129.3	161.1	202	99.1	122.1	141.7	237.6	203.6
33	CHOC760101	Residue accessible surface area in tripeptide	AAindex	115	135	150	190	210	75	195	175	200	170	185	160	145	180	225	115	140	155	255	230
34	CHOC760102	Residue accessible surface area in folded protein	AAindex	25	19	50	49	24	23	43	18	97	23	31	63	50	71	90	44	47	18	32	60
35	CHOC760103	Proportion of residues 95% buried	AAindex	0.38	0.45	0.15	0.18	0.5	0.36	0.17	0.6	0.03	0.45	0.4	0.12	0.18	0.07	0.01	0.22	0.23	0.54	0.27	0.15
36	CHOC760104	Proportion of residues 100% buried	AAindex	0.2	0.22	0.04	0.03	0.14	0.18	0.02	0.19	0	0.16	0.11	0.03	0.04	0.01	0	0.08	0.08	0.18	0.04	0.03
37	CHOP780101	Normalized frequency of beta-turn	AAindex	0.66	1.19	1.46	0.74	0.6	1.56	0.95	0.47	1.01	0.59	0.6	1.56	1.52	0.98	0.95	1.43	0.96	0.5	0.96	1.14
38	CHOP780201	Normalized frequency of alpha-helix	AAindex	1.42	0.7	1.01	1.51	1.13	0.57	1	1.08	1.16	1.21	1.45	0.67	0.57	1.11	0.98	0.77	0.83	1.06	1.08	0.69
39	CHOP780202	Normalized frequency of beta-sheet	AAindex	0.83	1.19	0.54	0.37	1.38	0.75	0.87	1.6	0.74	1.3	1.05	0.89	0.55	1.1	0.93	0.75	1.19	1.7	1.37	1.47
40	CHOP780203	Normalized frequency of beta-turn	AAindex	0.74	0.96	1.52	0.95	0.66	1.56	0.95	0.47	1.19	0.5	0.6	1.46	1.56	0.96	1.01	1.43	0.98	0.59	0.6	1.14
41	CHOP780204	Normalized frequency of N-terminal helix	AAindex	1.29	0.66	2.02	2.44	0.61	0.76	0.73	0.67	0.66	0.58	0.71	0.81	2.01	1.22	0.44	0.74	1.08	0.61	1.47	0.68
42	CHOP780205	Normalized frequency of C-terminal helix	AAindex	1.2	1.11	0.61	1.24	1.1	0.42	1.77	0.98	1.83	1.13	1.57	0.59	0	1.22	1.25	0.96	0.75	1.25	0.4	0.73
43	CHOP780206	Normalized frequency of N-terminal non helical region	AAindex	0.7	0.65	0.98	1.04	0.93	1.41	1.22	0.78	1.01	0.85	0.83	1.42	1.1	0.75	0.34	1.55	1.09	0.75	0.62	0.99
44	CHOP780207	Normalized frequency of C-terminal non helical region	AAindex	0.52	0.94	1.06	0.59	1.04	1.64	1.86	0.87	1.49	0.84	0.52	1.64	1.58	0.7	1.24	0.93	0.86	0.32	0.16	0.96
45	CHOP780208	Normalized frequency of N-terminal beta-sheet	AAindex	0.86	0.87	0.38	0.35	1.5	0.63	0.54	1.94	1	1.3	1.43	0.66	0.66	1.65	0.9	0.63	1.17	1.69	1.49	1.07
46	CHOP780209	Normalized frequency of C-terminal beta-sheet	AAindex	0.75	1.11	0.85	0.55	1.5	0.74	0.9	1.35	0.74	1.27	0.95	1.21	0.4	0.65	0.9	0.79	0.75	1.79	1.19	1.96
47	CHOP780210	Normalized frequency of N-terminal non beta region	AAindex	0.67	1.34	1.39	0.92	0.3	1.46	0.78	0.59	1.09	0.46	0.52	1.86	1.58	1.09	0.89	1.41	1.09	0.42	0.48	1.23
48	CHOP780211	Normalized frequency of C-terminal non beta region	AAindex	0.74	0.53	1.32	0.85	0.44	1.68	0.96	0.53	0.82	0.59	0.85	1.13	1.69	0.77	1.05	1.49	1.16	0.59	1.59	1.01
49	CHOP780212	Frequency of the 1st residue in turn	AAindex	0.06	0.149	0.147	0.056	0.059	0.102	0.14	0.043	0.055	0.061	0.068	0.161	0.102	0.074	0.07	0.12	0.086	0.062	0.077	0.082
50	CHOP780213	Frequency of the 2nd residue in turn	AAindex	0.076	0.053	0.11	0.06	0.041	0.085	0.047	0.034	0.115	0.025	0.082	0.083	0.301	0.098	0.106	0.139	0.108	0.048	0.013	0.065
51	CHOP780214	Frequency of the 3rd residue in turn	AAindex	0.035	0.117	0.179	0.077	0.065	0.19	0.093	0.013	0.072	0.036	0.014	0.191	0.034	0.037	0.099	0.125	0.065	0.028	0.064	0.114
52	CHOP780215	Frequency of the 4th residue in turn	AAindex	0.058	0.128	0.081	0.064	0.065	0.152	0.054	0.056	0.095	0.07	0.055	0.091	0.068	0.098	0.085	0.106	0.079	0.053	0.167	0.125
53	CHOP780216	Normalized frequency of the 2nd and 3rd residues in turn	AAindex	0.64	0.92	1.61	0.8	0.62	1.63	0.77	0.29	1.13	0.36	0.51	1.56	2.04	0.84	1.05	1.52	0.98	0.43	0.48	1.08
54	CIDH920101	Normalized hydrophobicity scales for alpha-proteins	AAindex	-0.45	0.79	-1.52	-0.8	1.48	-1	1.07	0.76	-0.36	1.29	1.37	-0.2	-0.12	-0.99	-0.24	-0.98	-0.7	1.26	1.38	1.49
55	CIDH920102	Normalized hydrophobicity scales for beta-proteins	AAindex	-0.08	0.76	-0.71	-1.31	1.53	-0.84	0.43	1.39	-0.09	1.24	1.27	-0.7	-0.01	-0.4	-0.09	-0.93	-0.59	1.09	2.25	1.53
56	CIDH920103	Normalized hydrophobicity scales for alpha+beta-proteins	AAindex	0.36	0.7	-1.09	-0.83	1.01	-0.82	0.16	2.17	-0.56	1.18	1.21	-0.9	-0.06	-1.05	-0.52	-0.6	-1.2	1.21	1.31	1.05
57	CIDH920104	Normalized hydrophobicity scales for alpha/beta-proteins	AAindex	0.17	1.24	-1.05	-1.19	1.29	-0.57	-0.25	2.06	-0.62	0.96	0.6	-0.9	-0.21	-1.2	-0.7	-0.83	-0.62	1.21	1.51	0.66
58	CIDH920105	Normalized average hydrophobicity scales	AAindex	0.02	0.77	-1.04	-1.14	1.35	-0.8	0.26	1.81	-0.41	1.14	1	-0.77	-0.09	-1.1	-0.42	-0.97	-0.77	1.13	1.71	1.11
59	COHE430101	Partial specific volume	AAindex	0.75	0.61	0.6	0.66	0.77	0.64	0.67	0.9	0.82	0.9	0.75	0.61	0.76	0.67	0.7	0.68	0.7	0.86	0.74	0.71
60	CRAJ730101	Normalized frequency of middle helix	AAindex	1.33	0.93	0.97	1.66	1.15	0.58	1.49	0.99	1.03	1.29	1.4	0.72	0.49	1.42	0.79	0.83	0.94	0.96	1.33	0.49
61	CRAJ730102	Normalized frequency of beta-sheet	AAindex	1	0.99	0.89	0.37	1.26	0.56	0.36	1.75	1.18	1.53	1.4	0.75	0.36	0.87	0.74	0.65	1.15	1.61	0.84	1.41
62	CRAJ730103	Normalized frequency of turn	AAindex	0.6	1.29	1.24	0.64	1.05	1.38	0.95	0.67	1.1	0.7	0.67	1.42	1.47	0.92	0.79	1.26	1.05	0.48	1.23	1.35
63	DAWD720101	Size	AAindex	2.5	3	2.5	5	6.5	0.5	6	5.5	7	5.5	6	5	5.5	6	7.5	3	5	5	7	7
64	DAYM780101	Amino acid composition	AAindex	8.6	2.9	5.5	6	3.6	8.4	2	4.5	6.6	7.4	1.7	4.3	5.2	3.9	4.9	7	6.1	6.6	1.3	3.4
65	DAYM780201	Relative mutability	AAindex	100	20	106	102	41	49	66	96	56	40	94	134	56	93	65	120	97	74	18	41
66	DESM900101	Membrane preference for cytochrome b: MPH89	AAindex	1.56	1.8	0.23	0.19	1.42	1.03	1	1.27	0.15	1.38	1.93	0.51	0.27	0.39	0.59	0.96	1.11	1.58	0.91	1.1
67	DESM900102	Average membrane preference: AMP07	AAindex	1.26	1.6	0.27	0.23	1.46	1.08	1	1.44	0.33	1.36	1.52	0.59	0.54	0.39	0.38	0.98	1.01	1.33	1.06	0.89
68	EISD840101	Consensus normalized hydrophobicity scale	AAindex	0.25	0.04	-0.72	-0.62	0.61	0.16	-0.4	0.73	-1.1	0.53	0.26	-0.64	-0.07	-0.69	-1.76	-0.26	-0.18	0.54	0.37	0.02
69	EISD860101	Solvation free energy	AAindex	0.67	0.38	-1.2	-0.76	2.3	0	0.64	1.9	-0.57	1.9	2.4	-0.6	1.2	-0.22	-2.1	0.01	0.52	1.5	2.6	1.6
70	EISD860102	Atom-based hydrophobic moment	AAindex	0	0.17	1.9	3	1.1	0	0.99	1.2	5.7	1	1.9	1.3	0.18	1.9	10	0.73	1.5	0.48	1.6	1.8
71	EISD860103	Direction of hydrophobic moment	AAindex	0	0.76	-0.98	-0.89	0.92	0	-0.75	0.99	-0.99	0.89	0.94	-0.86	0.22	-1	-0.96	-0.67	0.09	0.84	0.67	-0.93
72	FASG760101	Molecular weight	AAindex	89.09	121.15	133.1	147.13	165.19	75.07	155.16	131.17	146.19	131.17	149.21	132.12	115.13	146.15	174.2	105.09	119.12	117.15	204.24	181.19
73	FASG760102	Melting point	AAindex	297	178	270	249	284	290	277	284	224	337	283	236	222	185	238	228	253	293	282	344
74	FASG760103	Optical rotation	AAindex	1.8	-16.5	5.05	12	-34.5	0	-38.5	12.4	14.6	-11	-10	-5.6	-86.2	6.3	12.5	-7.5	-28	5.63	-33.7	-10
75	FASG760104	pK-N	AAindex	9.69	8.35	9.6	9.67	9.18	9.78	9.17	9.68	9.18	9.6	9.21	8.8	10.64	9.13	8.99	9.21	9.1	9.62	9.44	9.11
76	FASG760105	pK-C	AAindex	2.34	1.92	1.88	2.1	2.16	2.35	1.82	2.36	2.16	2.36	2.28	2.02	1.95	2.17	1.82	2.19	2.09	2.32	2.43	2.2
77	FAUJ830101	Hydrophobic parameter p	AAindex	0.31	1.54	-0.77	-0.64	1.79	0	0.13	1.8	-0.99	1.7	1.23	-0.6	0.72	-0.22	-1.01	-0.04	0.26	1.22	2.25	0.96
78	FAUJ880101	Graph shape index	AAindex	1.28	1.77	1.6	1.56	2.94	0	2.99	4.19	1.89	2.59	2.35	1.6	2.67	1.56	2.34	1.31	3.03	3.67	3.21	2.94
79	FAUJ880102	Smoothed upsilon steric parameter	AAindex	0.53	0.66	0.59	0.72	0.71	0	0.64	0.96	0.78	0.92	0.77	0.58	0	0.71	0.69	0.55	0.63	0.89	0.84	0.71
80	FAUJ880103	Normalized van der Waals volume	AAindex	1	2.43	2.78	3.78	5.89	0	4.66	4	4.77	4	4.43	2.95	2.72	3.95	6.13	1.6	2.6	3	8.08	6.47
81	FAUJ880104	STERIMOL length of the side chain	AAindex	2.87	4.47	4.74	5.97	4.62	2.06	5.23	4.92	6.89	4.92	6.36	4.58	4.11	6.11	7.82	3.97	4.11	4.11	7.68	4.73
82	FAUJ880105	STERIMOL minimum width of the side chain	AAindex	1.52	1.52	1.52	1.52	1.52	1	1.52	1.9	1.52	1.52	1.52	1.52	1.52	1.52	1.52	1.52	1.73	1.9	1.52	1.52
83	FAUJ880106	STERIMOL maximum width of the side chain	AAindex	2.04	3.41	3.78	3.31	6.02	1	5.66	3.49	4.87	4.45	4.8	4.37	4.31	3.53	6.24	2.7	3.17	3.17	5.9	6.72
84	FAUJ880107	N.m.r. chemical shift of alpha-carbon	AAindex	7.3	14.4	9.2	11.4	13.9	0	10.2	16.1	10.9	10.1	10.4	8	17.8	10.6	11.1	13.1	16.7	17.2	13.2	13.9
85	FAUJ880108	Localized electrical effect	AAindex	-0.01	0.12	0.15	0.07	0.03	0	0.08	-0.01	0	-0.01	0.04	0.06	0	0.05	0.04	0.11	0.04	0.01	0	0.03
86	FAUJ880109	Number of hydrogen bond donors	AAindex	0	0	1	1	0	0	1	0	2	0	0	2	0	2	4	1	1	0	1	1
87	FAUJ880110	Number of full nonbonding orbitals	AAindex	0	0	4	4	0	0	1	0	1	0	0	3	0	3	3	2	2	0	0	2
88	FAUJ880111	Positive charge	AAindex	0	0	0	0	0	0	1	0	1	0	0	0	0	0	1	0	0	0	0	0
89	FAUJ880112	Negative charge	AAindex	0	0	1	1	0	0	0	0	0	0	0	0	0	0	0	0	0	0	0	0
90	FAUJ880113	pK-a(RCOOH)	AAindex	4.76	3.67	5.69	5.48	4.31	3.77	2.84	4.81	4.27	4.79	4.25	3.64	0	4.54	4.3	3.83	3.87	4.86	4.75	4.3
91	FINA770101	Helix-coil equilibrium constant	AAindex	1.08	0.95	0.85	1.15	1.1	0.55	1	1.05	1.15	1.25	1.15	0.85	0.71	0.95	1.05	0.75	0.75	0.95	1.1	1.1
92	FINA910101	Helix initiation parameter at posision i-1	AAindex	1	1	3.2	1.7	1	1	1	0.6	0.7	1	1	1.7	1	1	0.7	1.7	1.7	0.6	1	1
93	FINA910102	Helix initiation parameter at posision i,i+1,i+2	AAindex	1	1	1.7	1.7	1	1.3	1	1	0.7	1	1	1	13	1	0.7	1	1	1	1	1
94	FINA910103	Helix termination parameter at posision j-2,j-1,	AAindex	1.2	1	0.7	0.7	1	0.8	1.2	0.8	1.7	1	1	1.2	1	1	1.7	1.5	1	0.8	1	1
95	FINA910104	Helix termination parameter at posision j+1	AAindex	1	1	0.7	0.7	1	1.5	1	1	1.7	1	1	1	0.1	1	1.7	1	1	1	1	1
96	GARJ730101	Partition coefficient	AAindex	0.28	0.28	0.21	0.33	2.18	0.17	0.21	0.82	0.09	1	0.74	0.25	0.39	0.35	0.1	0.12	0.21	0.6	5.7	1.26
97	GEIM800101	Alpha-helix indices	AAindex	1.29	0.79	1.1	1.49	1.13	0.63	1.33	1.05	1.33	1.31	1.54	0.81	0.63	1.07	1	0.78	0.77	0.81	1.18	0.71
98	GEIM800102	Alpha-helix indices for alpha-proteins	AAindex	1.13	1.32	0.94	1.2	1.01	0.83	1.09	1.05	1.08	1.13	1.23	1.06	0.82	0.93	1.09	1.01	1.17	1.13	1.32	0.88
99	GEIM800103	Alpha-helix indices for beta-proteins	AAindex	1.55	1.44	1.55	1.67	0.4	0.59	1.21	1.27	1.2	1.25	1.37	1.2	0.21	1.13	0.2	1.01	0.55	0.64	1.86	1.08
100	GEIM800104	Alpha-helix indices for alpha/beta-proteins	AAindex	1.19	0.95	1.07	1.64	1.02	0.6	1.03	1.12	1.27	1.18	1.49	0.94	0.68	1.32	1	0.81	0.85	0.74	1.18	0.77
101	GEIM800105	Beta-strand indices	AAindex	0.84	1.27	0.59	0.57	1.15	0.94	0.81	1.29	0.86	1.1	0.88	0.66	0.8	1.02	1.04	1.05	1.2	1.56	1.15	1.39
102	GEIM800106	Beta-strand indices for beta-proteins	AAindex	0.86	0.91	0.66	0.37	1.34	0.86	1.07	1.17	1.01	1.28	1.15	0.6	0.61	1.11	1.15	0.91	1.14	1.31	1.13	1.37
103	GEIM800107	Beta-strand indices for alpha/beta-proteins	AAindex	0.91	1.12	0.74	0.41	1.26	0.91	1.01	1.29	0.86	1.23	0.96	0.72	0.65	0.9	0.99	0.93	1.05	1.58	1.15	1.21
104	GEIM800108	Aperiodic indices	AAindex	0.91	0.93	1.4	0.97	0.72	1.51	0.9	0.65	0.82	0.59	0.58	1.64	1.66	0.94	1	1.23	1.04	0.6	0.67	0.92
105	GEIM800109	Aperiodic indices for alpha-proteins	AAindex	0.8	0	1.6	0.4	1.2	2	0.96	0.85	0.94	0.8	0.39	1.1	2.1	1.6	0.96	1.3	0.6	0.8	0	1.8
106	GEIM800110	Aperiodic indices for beta-proteins	AAindex	1.1	1.05	1.41	1.4	0.6	1.3	0.85	0.67	0.94	0.52	0.69	1.57	1.77	0.81	0.93	1.13	0.88	0.58	0.62	0.41
107	GEIM800111	Aperiodic indices for alpha/beta-proteins	AAindex	0.93	0.92	1.22	1.05	0.71	1.45	0.96	0.58	0.91	0.59	0.6	1.36	1.67	0.83	1.01	1.25	1.08	0.62	0.68	0.98
108	GOLD730101	Hydrophobicity factor	AAindex	0.75	1	0	0	2.65	0	0	2.95	1.5	2.4	1.3	0.69	2.6	0.59	0.75	0	0.45	1.7	3	2.85
109	GOLD730102	Residue volume	AAindex	88.3	112.4	110.8	140.5	189	60	152.6	168.5	175.6	168.5	162.2	125.1	122.2	148.7	181.2	88.7	118.2	141.4	227	193

Table S11 Amino acid descriptors (1D) used in this study.

No.	Descriptor ID	Attribute	Ref*	A	C	D	E	F	G	H	I	K	L	M	N	P	Q	R	S	T	V	W	Y
110	GRAR740101	Composition	AAindex	0	2.75	1.38	0.92	0	0.74	0.58	0	0.33	0	0	1.33	0.39	0.89	0.65	1.42	0.71	0	0.13	0.2
111	GRAR740102	Polarity	AAindex	8.1	5.5	13	12.3	5.2	9	10.4	5.2	11.3	4.9	5.7	11.6	8	10.5	10.5	9.2	8.6	5.9	5.4	6.2
112	GRAR740103	Volume	AAindex	31	55	54	83	132	3	96	111	119	111	105	56	32.5	85	124	32	61	84	170	136
113	GUYH850101	Partition energy	AAindex	0.1	-1.42	0.78	0.83	-2.12	0.33	-0.5	-1.13	1.4	-1.18	-1.59	0.48	0.73	0.95	1.91	0.52	0.07	-1.27	-0.51	-0.21
114	HOPA770101	Hydration number	AAindex	1	0.1	6.5	6.2	1.4	1.1	2.8	0.8	5.3	0.8	0.7	2.2	0.9	2.1	2.3	1.7	1.5	0.9	1.9	2.1
115	HOPT810101	Hydrophilicity value	AAindex	-0.5	-1	3	3	-2.5	0	-0.5	-1.8	3	-1.8	-1.3	0.2	0	0.2	3	0.3	-0.4	-1.5	-3.4	-2.3
116	HUTJ700101	Heat capacity	AAindex	29.22	50.7	37.09	41.84	48.52	23.71	59.64	45	57.1	48.03	69.32	38.3	36.13	44.02	26.37	32.4	35.2	40.35	56.92	51.73
117	HUTJ700102	Absolute entropy	AAindex	30.88	53.83	40.66	44.98	51.06	24.74	65.99	49.71	63.21	50.62	55.32	41.7	39.21	46.62	68.43	35.65	36.5	42.75	60	51.15
118	HUTJ700103	Entropy of formation	AAindex	341.01	235.51	219.79	127.9	179.93	242.54	233.21	232.3	202.65	300.46	204.74	194.91	174.06	223.16	207.9	205.8	237.01	221.03316	229.15	207.6
119	ISOY800101	Normalized relative frequency of alpha-helix	AAindex	1.53	0.89	1	1.63	1.22	0.44	1.03	1.07	1.26	1.32	1.66	0.6	0.25	1.27	1.17	0.65	0.86	0.93	1.05	0.7
120	ISOY800102	Normalized relative frequency of extended structure	AAindex	0.86	1.39	0.69	0.66	1.16	0.7	1.06	1.31	0.77	1.01	1.06	0.74	1.16	0.89	0.98	1.09	1.24	1.4	1.17	1.28
121	ISOY800103	Normalized relative frequency of bend	AAindex	0.78	0.6	1.5	0.97	0.67	1.73	0.83	0.4	1.01	0.57	0.3	1.56	1.55	0.78	1.06	1.19	1.09	0.44	0.74	1.14
122	ISOY800104	Normalized relative frequency of bend R	AAindex	1.09	0.5	0.77	0.92	0.5	1.25	0.67	0.66	1.25	0.44	0.45	1.14	2.96	0.83	0.97	1.21	1.33	0.56	0.62	0.94
123	ISOY800105	Normalized relative frequency of bend S	AAindex	0.35	0.5	2.16	0.65	0.89	2.4	1.19	0.12	0.83	0.58	0.22	2.12	0.43	0.73	0.75	1.24	0.85	0.43	0.62	1.44
124	ISOY800106	Normalized relative frequency of helix end	AAindex	1.09	1.04	1.24	1.14	0.8	0.27	1.07	0.97	1.2	1.3	0.55	0.88	1.78	1.09	1.07	1.2	0.99	0.77	1.03	0.69
125	ISOY800107	Normalized relative frequency of double bend	AAindex	1.34	1.44	1.77	2.54	0.43	0.95	0	0.52	0.79	1.05	0	0.92	0.37	0.79	2.78	0.87	1.14	0	1.79	0.73
126	ISOY800108	Normalized relative frequency of coil	AAindex	0.47	0.41	1.15	0.64	0.61	3.03	0.89	0.62	0.98	0.53	0.68	2.16	0.63	0.95	0.52	1.03	0.39	0.76	0.63	0.83
127	JANJ780101	Average accessible surface area	AAindex	27.8	15.5	60.6	68.2	25.5	24.5	50.7	22.8	103	27.6	33.5	60.1	51.5	68.7	94.7	42	45	23.7	34.7	55.2
128	JANJ780102	Percentage of buried residues	AAindex	51	74	19	16	58	52	34	66	3	60	52	22	25	16	5	35	30	64	49	24
129	JANJ780103	Percentage of exposed residues	AAindex	15	5	50	55	10	10	34	13	85	16	20	49	45	56	67	32	32	14	17	41
130	JANJ790101	Ratio of buried and accessible molar fractions	AAindex	1.7	4.6	0.4	0.3	2.2	1.8	0.8	3.1	0.05	2.4	1.9	0.4	0.6	0.3	0.1	0.8	0.7	2.9	1.6	0.5
131	JANJ790102	Transfer free energy	AAindex	0.3	0.9	-0.6	-0.7	0.5	0.3	-0.1	0.7	-1.8	0.5	0.4	-0.5	-0.3	-0.7	-1.4	-0.1	-0.2	0.6	0.3	-0.4
132	JOND750101	Hydrophobicity	AAindex	0.87	1.52	0.66	0.67	2.87	0.1	0.87	3.15	1.64	2.17	1.67	0.09	2.77	0	0.85	0.07	0.07	1.87	3.77	2.67
133	JOND750102	pK (-COOH)	AAindex	2.34	1.65	2.01	2.19	1.83	2.34	1.82	2.36	2.18	2.36	2.28	2.02	1.99	2.17	1.18	2.21	2.1	2.32	2.38	2.2
134	JOND920101	Relative frequency of occurrence	AAindex	0.077	0.02	0.052	0.062	0.04	0.074	0.023	0.053	0.059	0.091	0.024	0.043	0.051	0.041	0.051	0.069	0.059	0.066	0.014	0.032
135	JOND920102	Relative mutability	AAindex	100	44	86	77	51	50	91	103	72	54	93	104	58	84	83	117	107	98	25	50
136	JUKT750101	Amino acid distribution	AAindex	5.3	1.3	3.6	3.3	2.3	4.8	1.4	3.1	4.1	4.7	1.1	3	2.5	2.4	2.6	4.5	3.7	4.2	0.8	2.3
137	JUNJ780101	Sequence frequency	AAindex	685	241	400	427	303	707	155	394	575	581	132	397	366	313	382	593	490	553	99	292
138	KANM800101	Average relative probability of helix	AAindex	1.36	0.82	1.04	1.48	1.05	0.63	1.11	1.08	1.22	1.21	1.45	0.89	0.52	1.14	1	0.74	0.81	0.94	0.97	0.79
139	KANM800102	Average relative probability of beta-sheet	AAindex	0.81	1.17	0.71	0.53	1.2	0.88	0.92	1.48	0.77	1.24	1.05	0.62	0.61	0.98	0.85	0.92	1.18	1.66	1.18	1.23
140	KANM800103	Average relative probability of inner helix	AAindex	1.45	0.7	0.91	1.29	1.2	0.53	1.13	1.23	1.27	1.56	1.83	0.64	0.21	1.14	1.15	0.48	0.77	1.1	1.17	0.74
141	KANM800104	Average relative probability of inner beta-sheet	AAindex	0.75	1.46	0.31	0.46	1.37	0.83	0.83	1.87	0.66	1.56	0.86	0.33	0.52	0.75	0.79	0.82	1.36	2	0.79	1.08
142	KARP850101	Flexibility parameter for no rigid neighbors	AAindex	1.041	0.96	1.033	1.094	0.93	1.142	0.982	1.002	1.093	0.967	0.947	1.117	1.055	1.165	1.038	1.169	1.073	0.982	0.925	0.961
143	KARP850102	Flexibility parameter for one rigid neighbor	AAindex	0.946	0.878	1.089	1.036	0.912	1.042	0.952	0.892	1.082	0.961	0.862	1.006	1.085	1.025	1.028	1.048	1.051	0.927	0.917	0.93
144	KARP850103	Flexibility parameter for two rigid neighbors	AAindex	0.892	0.925	0.932	0.933	0.914	0.923	0.894	0.872	1.057	0.921	0.804	0.93	0.932	0.885	0.901	0.923	0.934	0.913	0.803	0.837
145	KHAG800101	The Kerr-constant increments	AAindex	49.1	0	0	0	54.7	64.6	75.7	18.9	0	15.6	6.8	-3.6	43.8	20	133	44.4	31	29.5	70.5	0
146	KLEP840101	Net charge	AAindex	0	0	-1	-1	0	0	0	0	1	0	0	0	0	0	1	0	0	0	0	0
147	KRIW710101	Side chain interaction parameter	AAindex	4.6	-1	5.7	5.6	3.2	7.6	4.5	2.6	7.9	3.25	1.4	5.9	7	6.1	6.5	5.25	4.8	3.4	4	4.35
148	KRIW790101	Side chain interaction parameter	AAindex	4.32	1.73	6.04	6.17	2.59	6.09	5.66	2.31	7.92	3.93	2.44	6.24	7.19	6.13	6.55	5.37	5.16	3.31	2.78	3.58
149	KRIW790102	Fraction of site occupied by water	AAindex	0.28	0.11	0.33	0.37	0.1	0.28	0.23	0.12	0.59	0.16	0.08	0.31	0.46	0.39	0.34	0.27	0.26	0.22	0.15	0.25
150	KRIW790103	Side chain volume	AAindex	27.5	44.6	40	62	115.5	0	79	93.5	100	93.5	94.1	58.7	41.9	80.7	105	29.3	51.3	71.5	145.5	117.3
151	KYTJ820101	Hydropathy index	AAindex	1.8	2.5	-3.5	-3.5	2.8	-0.4	-3.2	4.5	-3.9	3.8	1.9	-3.5	-1.6	-3.5	-4.5	-0.8	-0.7	4.2	-0.9	-1.3
152	LAWE840101	Transfer free energy, CHP/water	AAindex	-0.48	-0.32	-0.75	-0.71	1.03	0	-0.51	0.81	-0.09	1.02	0.81	-0.87	2.03	-0.32	-0.06	0.05	-0.35	0.56	0.66	1.24
153	LEVM760101	Hydrophobic parameter	AAindex	-0.5	-1	2.5	2.5	-2.5	0	-0.5	-1.8	3	-1.8	-1.3	0.2	-1.4	0.2	3	0.3	-0.4	-1.5	-3.4	-2.3
154	LEVM760102	Distance between C-alpha and centroid of side chain	AAindex	0.77	1.38	1.99	2.63	2.97	0	2.76	1.83	2.94	2.08	2.34	1.98	1.42	2.58	3.72	1.28	1.43	1.49	3.58	3.36
155	LEVM760103	Side chain angle theta(AAR)	AAindex	121.9	113.7	121.2	118.2	118.2	0	118.2	118.9	122	118.1	113.1	117.5	81.9	118	121.4	117.9	117.1	121.7	118.4	110
156	LEVM760104	Side chain torsion angle phi(AAAR)	AAindex	243.2	209.4	215	213.6	203.7	300	219.9	217.9	210.9	205.6	204	207.1	237.4	205.4	206.6	232	226.7	220.3	203.7	195.6
157	LEVM760105	Radius of gyration of side chain	AAindex	0.77	1.22	1.43	1.77	1.9	0.58	1.78	1.56	2.08	1.54	1.8	1.45	1.25	1.75	2.38	1.08	1.24	1.29	2.21	2.13
158	LEVM760106	van der Waals parameter R0	AAindex	5.2	6.1	5	6	7.1	4.2	6	7	6	7	6.8	5	6.2	6	6	4.9	5	6.4	7.6	7.1
159	LEVM760107	van der Waals parameter epsilon	AAindex	0.025	0.1	0.1	0.1	0.39	0.025	0.1	0.19	0.2	0.19	0.19	0.1	0.17	0.1	0.2	0.025	0.1	0.15	0.56	0.39
160	LEVM780101	Normalized frequency of alpha-helix, with weights	AAindex	1.29	1.11	1.04	1.44	1.07	0.56	1.22	0.97	1.23	1.3	1.47	0.9	0.52	1.27	0.96	0.82	0.82	0.91	0.99	0.72
161	LEVM780102	Normalized frequency of beta-sheet, with weights	AAindex	0.9	0.74	0.72	0.75	1.32	0.92	1.08	1.45	0.77	1.02	0.97	0.76	0.64	0.8	0.99	0.95	1.21	1.49	1.14	1.25
162	LEVM780103	Normalized frequency of reverse turn, with weights	AAindex	0.77	0.81	1.41	0.99	0.59	1.64	0.68	0.51	0.96	0.58	0.41	1.28	1.91	0.98	0.88	1.32	1.04	0.47	0.76	1.05
163	LEVM780104	Normalized frequency of alpha-helix, unweighted	AAindex	1.32	0.92	1.03	1.44	1.02	0.61	1.31	0.93	1.25	1.31	1.39	0.95	0.58	1.1	0.98	0.76	0.79	0.93	0.97	0.73
164	LEVM780105	Normalized frequency of beta-sheet, unweighted	AAindex	0.86	1.04	0.69	0.66	1.21	0.89	0.85	1.47	0.77	1.04	0.93	0.73	0.68	1	0.97	1.02	1.27	1.43	1.26	1.31
165	LEVM780106	Normalized frequency of reverse turn, unweighted	AAindex	0.79	0.79	1.47	1.02	0.77	1.67	0.81	0.5	0.99	0.57	0.51	1.25	1.78	0.92	0.9	1.3	0.97	0.46	0.79	0.93
166	LEWP710101	Frequency of occurrence in beta-bends	AAindex	0.22	0.2	0.73	0.08	0.08	0.58	0.14	0.22	0.27	0.19	0.38	0.42	0.46	0.26	0.28	0.55	0.49	0.08	0.43	0.46
167	LIFS790101	Conformational preference for all beta-strands	AAindex	0.92	1.16	0.48	0.61	1.25	0.61	0.93	1.81	0.7	1.3	1.19	0.6	0.4	0.95	0.93	0.82	1.12	1.81	1.54	1.53
168	LIFS790102	Conformational preference for parallel beta-strands	AAindex	1	0.91	0.5	0.59	1.3	0.79	0.38	2.6	0.59	1.42	1.49	0.54	0.35	0.28	0.68	0.7	0.59	2.63	0.89	1.08
169	LIFS790103	Conformational preference for antiparallel beta-strands	AAindex	0.9	1.24	0.47	0.62	1.23	0.56	1.12	1.54	0.74	1.26	1.09	0.62	0.42	1.18	1.02	0.87	1.3	1.53	1.75	1.68
170	MANP780101	Average surrounding hydrophobicity	AAindex	12.97	14.63	10.85	11.89	14	12.43	12.16	15.67	11.36	14.9	14.39	11.42	11.37	11.76	11.72	11.23	11.69	15.71	13.93	13.42
171	MAXF760101	Normalized frequency of alpha-helix	AAindex	1.43	0.94	0.92	1.67	1.19	0.46	0.98	1.04	1.27	1.36	1.53	0.64	0.49	1.22	1.18	0.7	0.78	0.98	1.01	0.69
172	MAXF760102	Normalized frequency of extended structure	AAindex	0.86	1.17	0.72	0.62	1.16	0.97	1.06	1.24	0.79	0.98	1.08	0.74	1.22	0.89	0.94	1.04	1.18	1.33	1.07	1.25
173	MAXF760103	Normalized frequency of zeta R	AAindex	0.64	0.32	1.92	1.01	0.86	0.63	2.05	0.92	0.89	0.37	1.07	3.14	0.5	0.8	0.62	1.01	0.92	0.87	1	1.31
174	MAXF760104	Normalized frequency of left-handed alpha-helix	AAindex	0.17	0.95	1.08	0.28	0.28	5.02	0.57	0.26	1.17	0.21	0	2.62	0.12	0.91	0.76	0.57	0.23	0.24	0	0.97
175	MAXF760105	Normalized frequency of zeta L	AAindex	1.13	0.38	1.18	1.02	0.45	3.84	0.3	0.4	1.13	0.65	0	1.11	0	0.41	0.48	0.81	0.71	0.48	0.93	0.38
176	MAXF760106	Normalized frequency of alpha region	AAindex	1	1.09	1.39	1.04	0.65	0.46	0.71	0.68	1.05	1.01	0.36	0.87	1.95	1.13	1.18	1.56	1.23	0.58	1.1	0.87
177	MCMT640101	Refractivity	AAindex	4.34	35.77	12	17.26	29.4	0	21.81	19.06	21.29	18.78	21.64	13.28	10.93	17.56	26.66	6.35	11.01	13.92	42.53	31.53
178	MEEJ800101	Retention coefficient in HPLC, pH7.4	AAindex	0.5	-6.8	-8.2	-16.9	13.2	0	-3.5	13.9	0.1	8.8	4.8	0.8	6.1	-4.8	0.8	1.2	2.7	2.7	14.9	6.1
179	MEEJ800102	Retention coefficient in HPLC, pH2.1	AAindex	-0.1	-2.2	-2.8	-7.5	13.9	-0.5	0.8	11.8	-3.2	10	7.1	-1.6	8	-2.5	-4.5	-3.7	1.5	3.3	18.1	8.2
180	MEEJ810101	Retention coefficient in NaClO4	AAindex	1.1	7.1	-1.6	0.7	13.4	-0.2	-0.7	8.5	-1.9	11	5.4	-4.2	4.4	-2.9	-0.4	-3.2	-1.7	5.9	17.1	7.4
181	MEEJ810102	Retention coefficient in NaH2PO4	AAindex	1	4.6	-0.5	1.1	12.6	0.2	-2.2	7	-3	9.6	4	-3	3.1	-2	-2	-2.9	-0.6	4.6	15.1	6.7
182	MEIH800101	Average reduced distance for C-alpha	AAindex	0.93	0.88	1.01	1.02	0.78	1.01	0.89	0.79	1.05	0.85	0.84	0.98	1	1.02	0.98	1.02	0.99	0.81	0.83	0.93
183	MEIH800102	Average reduced distance for side chain	AAindex	0.94	0.84	1.08	1.12	0.73	1.01	0.92	0.76	1.23	0.82	0.83	1.04	1.04	1.11	1.09	1.04	1.02	0.81	0.87	1.03
184	MEIH800103	Average side chain orientation angle	AAindex	87	104	71	72	108	90	90	105	65	104	100	70	78	66	81	83	83	94	94	83
185	MIYS850101	Effective partition energy	AAindex	2.36	3.36	1.67	1.74	4.37	2.06	2.41	4.17	1.23	3.93	4.22	1.7	1.89	1.75	1.92	1.81	2.04	3.49	3.82	2.91
186	NAGK730101	Normalized frequency of alpha-helix	AAindex	1.29	0.94	1	1.54	1.23	0.72	1.29	0.94	1.23	1.23	1.23	0.77	0.7	1.1	0.83	0.78	0.87	0.97	1.06	0.63
187	NAGK730102	Normalized frequency of bata-structure	AAindex	0.96	1.13	0.9	0.33	1.37	0.9	0.87	1.54	0.81	1.26	1.29	0.72	0.75	1.18	0.67	0.77	1.23	1.41	1.13	1.07
188	NAGK730103	Normalized frequency of coil	AAindex	0.72	1.01	1.04	0.75	0.58	1.35	0.76	0.8	0.84	0.63	0.62	1.38	1.43	0.81	1.33	1.34	1.03	0.83	0.87	1.35
189	NAKH900101	AA composition of total proteins	AAindex	7.99	1.81	5.14	6.1	3.83	6.91	2.17	5.48	6.01	9.16	2.5	4.33	4.95	3.98	5.86	6.84	5.77	6.65	1.34	3.15
190	NAKH900102	SD of AA composition of total proteins	AAindex	3.73	2.3	2.23	3	1.94	3.36	1.55	2.52	3.36	3.4	1.37	2.33	3.18	2.36	3.34	2.83	2.63	2.53	1.15	1.76
191	NAKH900103	AA composition of mt-proteins	AAindex	5.74	1.03	2.11	2.63	6.51	5.66	2.3	9.12	3.2	15.36	5.3	5.25	4.79	2.3	1.92	7.55	7.51	5.12	2.51	4.08
192	NAKH900104	Normalized composition of mt-proteins	AAindex	-0.6	-0.34	-1.36	-1.16	1.38	-0.37	0.08	1.44	-0.84	1.82	2.04	0.39	-0.05	-0.71	-1.18	0.25	0.66	-0.6	1.02	0.53
193	NAKH900105	AA composition of mt-proteins from animal	AAindex	5.88	1.11	1.7	2.6	6.58	5.29	2.33	8.78	2.58	16.52	6	4.38	5.29	2.3	1.54	7.68	8.38	4.66	2.89	3.51
194	NAKH900106	Normalized composition from animal	AAindex	-0.57	-0.3	-1.54	-1.17	1.42	-0.48	0.1	1.31	-1.02	2.16	2.55	0.02	0.11	-0.71	-1.29	0.3	0.99	-0.79	1.35	0.2
195	NAKH900107	AA composition of mt-proteins from fungi and plant	AAindex	5.39	0.86	3.07	2.7	6.34	6.52	2.23	9.94	4.67	12.64	3.68	7.31	3.62	2.31	2.81	7.24	5.44	6.18	1.64	5.42
196	NAKH900108	Normalized composition from fungi and plant	AAindex	-0.7	-0.41	-0.93	-1.13	1.29	-0.12	0.04	1.77	-0.4	1.02	0.86	1.28	-0.42	-0.71	-0.91	0.14	-0.13	-0.19	0.26	1.29
197	NAKH900109	AA composition of membrane proteins	AAindex	9.25	1.07	3.89	4.8	6.36	8.51	1.88	6.47	3.5	10.94	3.14	3.71	4.36	3.17	3.96	6.26	5.66	7.55	2.22	3.28
198	NAKH900110	Normalized composition of membrane proteins	AAindex	0.34	-0.32	-0.56	-0.43	1.3	0.48	-0.19	0.39	-0.75	0.52	0.47	-0.27	-0.19	-0.34	-0.57	-0.2	-0.04	0.36	0.77	0.07
199	NAKH900111	Transmembrane regions of non-mt-proteins	AAindex	10.17	1.48	1.18	1.15	9.6	8.87	1.07	10.91	1.04	16.22	4.12	1.36	2.24	1.57	1.21	5.38	5.61	11.44	2.67	2.68
200	NAKH900112	Transmembrane regions of mt-proteins	AAindex	6.61	0.83	0.59	1.63	7.76	4.88	1.14	12.91	1.15	21.66	7.17	1.84	3.51	1.2	0.41	6.84	8.89	6.3	2.11	2.57
201	NAKH900113	Ratio of average and computed composition	AAindex	1.61	0.37	0.75	1.5	1.24	3.12	0.46	1.61	0.62	1.37	1.59	0.73	0.67	0.61	0.4	0.68	0.92	1.3	1.63	0.67
202	NAKH920101	AA composition of CYT of single-spanning proteins	AAindex	8.63	1.03	6.24	7.82	2.73	6.8	2.7	3.48	6.25	8.44	2.14	4.18	6.28	4.76	6.75	8.53	4.43	5.44	0.8	2.54
203	NAKH920102	AA composition of CYT2 of single-spanning proteins	AAindex	10.88	0.69	6.13	9.34	2.93	7.72	2.15	1.8	6.11	8.03	3.79	5.75	7.21	4.68	6.01	7.25	3.51	4.57	0.47	1.01
204	NAKH920103	AA composition of EXT of single-spanning proteins	AAindex	5.15	3.24	5.75	7.05	3.52	6.38	2.69	4.4	5.25	8.11	1.6	4.81	5.65	4.45	4.38	8.04	7.41	7	1.68	3.42
205	NAKH920104	AA composition of EXT2 of single-spanning proteins	AAindex	5.04	2.2	5.26	6.07	3.72	7.09	2.99	4.32	6.31	9.88	1.85	5.94	6.22	4.5	3.73	8.05	5.2	6.19	2.1	3.32
206	NAKH920105	AA composition of MEM of single-spanning proteins	AAindex	9.9	2.55	0.35	0.08	6.47	8.14	0.2	15.25	0.16	22.28	1.85	0.94	2.38	0.87	0.09	4.17	4.33	14.34	2.21	3.42
207	NAKH920106	AA composition of CYT of multi-spanning proteins	AAindex	6.69	1.7	4.97	7.76	3.59	6.32	2.11	4.51	8.36	8.23	2.46	4.49	5.2	5.39	6.65	7.4	5.18	5.27	1.06	2.75
208	NAKH920107	AA composition of EXT of multi-spanning proteins	AAindex	5.08	2.95	5.96	6.04	4.36	8.2	2.1	4.95	4.93	8.03	2.61	5.75	4.84	4.24	4.75	6.41	5.87	6.07	2.31	4.55
209	NAKH920108	AA composition of MEM of multi-spanning proteins	AAindex	9.36	2.56	0.94	0.94	10.99	6.17	0.47	13.73	0.58	16.64	3.93	2.31	1.96	1.14	0.27	5.58	4.68	12.43	2.2	3.13
210	NISK800101	8 A contact number	AAindex	0.23	1.78	-1.13	-0.75	0.48	-0.07	0.11	1.19	-1.05	1.03	0.66	-0.94	-0.76	-0.57	-0.26	-0.67	-0.36	1.24	0.9	0.59
211	NISK860101	14 A contact number	AAindex	-0.22	4.66	-4.12	-3.64	5.27	-1.62	1.28	5.58	-4.18	5.01	3.51	-2.65	-3.03	-2.76	-0.93	-2.84	-1.2	4.45	5.2	2.15
212	NOZY710101	Transfer energy, organic solvent/water	AAindex	0.5	0	0	0	2.5	0	0.5	1.8	0	1.8	1.3	0	0	0	0	0	0.4	1.5	3.4	2.3
213	OOBM770101	Average non-bonded energy per atom	AAindex	-1.475	-1.521	-2.035	-1.898	-1.699	-1.755	-1.951	-1.966	-1.963	-1.374	-1.864	-1.518	-1.753	-1.535	-1.56	-1.767	-1.869	-1.7457895	-1.686	-1.981
214	OOBM770102	Short and medium range non-bonded energy per atom	AAindex	-0.921	-1.116	-1.365	-1.364	-1.236	-1.215	-1.189	-1.315	-1.303	-1.074	-1.135	-1.162	-1.297	-1.163	-1.178	-1.252	-1.03	-1.1894211	-1.03	-1.254
215	OOBM770103	Long range non-bonded energy per atom	AAindex	-0.554	-0.405	-0.67	-0.534	-0.463	-0.54	-0.762	-0.65	-0.659	-0.3	-0.729	-0.356	-0.455	-0.371	-0.382	-0.515	-0.839	-0.5562105	-0.656	-0.728
216	OOBM770104	Average non-bonded energy per residue	AAindex	-16.225	-13.689	-12.21	-7.592	-11.893	-17.55	-15.608	-15.728	-15.704	-12.366	-20.504	-12.144	-10.518	-13.815	-12.48	-12.369	-26.166	-14.771579	-20.232	-13.867
217	OOBM770105	Short and medium range non-bonded energy per residue	AAindex	-10.131	-10.044	-8.19	-5.456	-8.652	-12.15	-9.512	-10.52	-10.424	-9.666	-12.485	-9.296	-7.782	-10.467	-9.424	-8.764	-14.42	-9.9221579	-12.36	-8.778
218	OOBM850101	Optimized beta-structure-coil equilibrium constant	AAindex	2.01	1.98	-2.05	0.93	2.68	0.12	-0.14	3.7	2.55	2.73	1.75	0.03	0.41	1.02	0.84	1.47	2.39	3.5	2.49	2.23
219	OOBM850102	Optimized propensity to form reverse turn	AAindex	1.34	1.07	3.32	2.2	0.8	2.07	1.27	0.66	0.61	0.54	0.7	2.49	2.12	1.49	0.95	0.94	1.09	1.32	-4.65	-0.17

Table S11 Amino acid descriptors (1D) used in this study.

No.	Descriptor ID	Attribute	Ref*	A	C	D	E	F	G	H	I	K	L	M	N	P	Q	R	S	T	V	W	Y
220	OOBM850103	Optimized transfer energy parameter	AAindex	0.46	0.2	-0.33	0.48	0.52	0.64	-1.31	3.28	-1.71	0.43	0.15	1.31	-0.58	-1.12	-1.54	-0.83	-1.52	0.54	1.25	-2.21
221	OOBM850104	Optimized average non-bonded energy per atom	AAindex	-2.49	-3.13	8.86	4.04	-6.64	-0.56	4.22	-10.87	-9.97	-7.16	-4.96	2.27	5.19	1.79	2.55	-1.6	-4.75	-3.97	-17.84	9.25
222	OOBM850105	Optimized side chain interaction parameter	AAindex	4.55	-0.78	2.85	5.16	4.37	9.14	4.48	2.1	10.68	3.24	2.18	5.56	5.14	4.15	5.97	6.78	8.6	3.81	1.97	2.4
223	PALJ810101	Normalized frequency of alpha-helix from LG	AAindex	1.3	0.92	1.02	1.43	1.09	0.63	1.33	0.87	1.23	1.3	1.32	0.9	0.63	1.04	0.93	0.78	0.8	0.95	1.03	0.71
224	PALJ810102	Normalized frequency of alpha-helix from CF	AAindex	1.32	0.7	0.97	1.48	1.1	0.59	1.06	1.01	1.13	1.22	1.47	0.74	0.57	1.25	1.04	0.77	0.86	1.05	1.02	0.72
225	PALJ810103	Normalized frequency of beta-sheet from LG	AAindex	0.81	1.12	0.71	0.59	1.13	0.94	0.85	1.47	0.77	1.03	0.96	0.81	0.75	1.03	1.03	1.02	1.19	1.44	1.24	1.35
226	PALJ810104	Normalized frequency of beta-sheet from CF	AAindex	0.9	1.12	0.75	0.44	1.41	0.83	0.86	1.59	0.75	1.24	0.94	0.82	0.46	0.95	0.75	0.7	1.2	1.73	1.28	1.45
227	PALJ810105	Normalized frequency of turn from LG	AAindex	0.84	0.69	1.28	0.78	0.88	1.76	0.53	0.55	0.95	0.49	0.52	1.48	1.47	1	0.91	1.29	1.05	0.51	0.88	1.28
228	PALJ810106	Normalized frequency of turn from CF	AAindex	0.65	1.43	1.47	0.75	0.72	1.53	0.96	0.57	0.95	0.56	0.71	1.45	1.51	0.94	0.93	1.46	0.96	0.55	0.9	1.12
229	PALJ810107	Normalized frequency of alpha-helix in all-alpha class	AAindex	1.08	1.22	0.86	1.09	0.96	0.85	1.02	0.98	1.01	1.04	1.11	1.05	0.91	0.95	0.93	0.95	1.15	1.03	1.17	0.8
230	PALJ810108	Normalized frequency of alpha-helix in alpha+beta class	AAindex	1.34	1.27	1.06	1.69	1.02	0.47	1.11	0.84	1.08	1.39	0.9	0.83	0.48	1.13	0.91	1.05	0.74	1.18	0.64	0.73
231	PALJ810109	Normalized frequency of alpha-helix in alpha/beta class	AAindex	1.15	1.03	1	1.37	0.92	0.64	0.95	0.99	1.2	1.22	1.45	0.87	0.72	1.43	1.06	0.84	0.97	0.82	1.11	0.72
232	PALJ810110	Normalized frequency of beta-sheet in all-beta class	AAindex	0.89	1.04	0.71	0.72	1.32	0.87	1.04	1.14	1	1.02	1.41	0.67	0.69	1.06	1.06	0.86	1.15	1.66	1.06	1.35
233	PALJ810111	Normalized frequency of beta-sheet in alpha+beta class	AAindex	0.82	0.71	0.98	0.54	1.56	0.94	1.26	1.67	0.73	0.94	1.3	1.27	0.69	1.01	0.99	0.65	0.98	1.22	1.25	1.26
234	PALJ810112	Normalized frequency of beta-sheet in alpha/beta class	AAindex	0.98	1.01	0.74	0.59	1.23	0.9	1.17	1.38	0.83	1.05	0.82	0.66	0.73	0.63	1.03	0.98	1.2	1.62	1.26	1.23
235	PALJ810113	Normalized frequency of turn in all-alpha class	AAindex	0.69	0	2.42	0.63	2.2	2.64	0.22	0.43	1.18	0	0.88	1.52	1.34	1.44	0	1.43	0.28	0.14	0	1.53
236	PALJ810114	Normalized frequency of turn in all-beta class	AAindex	0.87	0.83	1.24	0.91	0.47	1.69	0.91	0.27	0.66	0.67	0	1.36	1.54	1.06	1.3	1.08	1.12	0.69	1.24	0.54
237	PALJ810115	Normalized frequency of turn in alpha+beta class	AAindex	0.91	0.5	0.9	0.53	0.37	1.61	1.08	0.36	1.27	0.77	0.76	1.32	1.62	1.06	0.77	1.34	0.87	0.52	1.1	1.24
238	PALJ810116	Normalized frequency of turn in alpha/beta class	AAindex	0.92	0.62	1.22	0.92	0.96	1.61	0.39	0.79	0.86	0.5	0.5	1.57	1.3	0.66	0.9	1.4	1.11	0.5	0.57	1.78
239	PARJ860101	HPLC parameter	AAindex	2.1	1.4	10	7.8	-9.2	5.7	2.1	-8	5.7	-9.2	-4.2	7	2.1	6	4.2	6.5	5.2	-3.7	-10	-1.9
240	PLIV810101	Partition coefficient	AAindex	-2.89	-2.49	-3.38	-2.94	-1.63	-3.25	-2.84	-1.72	-3.31	-1.61	-1.84	-3.41	-2.5	-3.15	-3.3	-3.3	-2.91	-2.08	-1.75	-2.42
241	PONP800101	Surrounding hydrophobicity in folded form	AAindex	12.28	14.93	10.97	11.19	13.43	12.01	12.84	14.77	10.8	14.1	14.33	11	11.19	11.28	11.49	11.26	11.65	15.07	12.95	13.29
242	PONP800102	Average gain in surrounding hydrophobicity	AAindex	7.62	10.93	6.18	6.38	8.99	7.31	7.85	9.99	5.72	9.37	9.83	6.17	6.64	6.67	6.81	6.93	7.08	10.38	8.41	8.53
243	PONP800103	Average gain ratio in surrounding hydrophobicity	AAindex	2.63	3.36	2.29	2.31	3.02	2.55	2.57	3.08	2.12	2.98	3.18	2.27	2.46	2.45	2.45	2.6	2.55	3.21	2.85	2.79
244	PONP800104	Surrounding hydrophobicity in alpha-helix	AAindex	13.65	14.49	10.98	12.55	14.08	15.36	11.59	14.63	11.96	14.01	13.4	12.24	11.51	11.3	11.28	11.26	13	12.88	12.06	12.64
245	PONP800105	Surrounding hydrophobicity in beta-sheet	AAindex	14.6	15.9	13.78	13.59	14.18	14.18	15.35	14.1	13.28	16.49	16.23	11.79	14.1	12.02	13.24	13.36	14.5	16.3	13.9	14.76
246	PONP800106	Surrounding hydrophobicity in turn	AAindex	10.67	14.15	10.21	11.71	13.27	10.95	12.07	12.95	9.93	13.07	15	10.85	10.62	11.71	11.05	11.18	10.53	13.86	11.41	11.52
247	PONP800107	Accessibility reduction ratio	AAindex	3.7	3.03	2.6	3.3	6.6	3.13	3.57	7.69	1.79	5.88	5.21	2.12	2.12	2.7	2.53	2.43	2.6	7.14	6.25	3.03
248	PONP800108	Average number of surrounding residues	AAindex	6.05	7.86	4.95	5.1	6.62	6.16	5.8	7.51	4.88	7.37	6.39	5.04	5.65	5.45	5.7	5.53	5.81	7.62	6.98	6.73
249	PRAM820101	Intercept in regression analysis	AAindex	0.305	0.339	0.335	0.282	0.195	0.352	0.215	0.278	0.391	0.262	0.28	0.322	0.346	0.306	0.227	0.326	0.251	0.291	0.291	0.293
250	PRAM820102	Slope in regression analysis x 1.0E1	AAindex	0.175	0.074	0.14	0.135	0.104	0.201	0.125	0.1	0.058	0.104	0.054	0.09	0.136	0.093	0.083	0.155	0.152	0.096	0.092	0.081
251	PRAM820103	Correlation coefficient in regression analysis	AAindex	0.687	0.263	0.632	0.669	0.577	0.67	0.594	0.564	0.407	0.541	0.328	0.489	0.6	0.527	0.59	0.692	0.713	0.529	0.632	0.495
252	PRAM900101	Hydrophobicity	AAindex	-6.7	-8.4	38.5	34.3	-15.5	-4.2	12.6	-13	36.8	-11.7	-14.2	20.1	0.8	17.2	51.5	-2.5	-5	-10.9	-7.9	2.9
253	PRAM900102	Relative frequency in alpha-helix	AAindex	1.29	1.11	1.04	1.44	1.07	0.56	1.22	0.97	1.23	1.3	1.47	0.9	0.52	1.27	0.96	0.82	0.82	0.91	0.99	0.72
254	PRAM900103	Relative frequency in beta-sheet	AAindex	0.9	0.74	0.72	0.75	1.32	0.92	1.08	1.45	0.77	1.02	0.97	0.76	0.64	0.8	0.99	0.95	1.21	1.49	1.14	1.25
255	PRAM900104	Relative frequency in reverse-turn	AAindex	0.78	0.8	1.41	1	0.58	1.64	0.69	0.51	0.96	0.59	0.39	1.28	1.91	0.97	0.88	1.33	1.03	0.47	0.75	1.05
256	PTIO830101	Helix-coil equilibrium constant	AAindex	1.1	0.95	0.65	1	1.1	0.6	0.85	1.1	1	1.25	1.15	0.8	0.1	1	0.95	0.75	0.75	0.95	1.1	1.1
257	PTIO830102	Beta-coil equilibrium constant	AAindex	1	1.9	0.5	0.7	3.1	0.3	0.8	4	0.7	2	1.9	0.6	0.2	1	0.7	0.9	1.7	4	2.2	2.8
258	QIAN880101	Weights for alpha-helix at the window position of -6	AAindex	0.12	-0.25	0.01	-0.02	0.05	-0.02	-0.06	-0.07	0.26	0.05	0	-0.1	-0.19	-0.03	0.04	-0.19	-0.04	-0.03	-0.06	-0.14
259	QIAN880102	Weights for alpha-helix at the window position of -5	AAindex	0.26	-0.15	0.15	0.21	0.12	-0.37	0.1	-0.03	0.12	-0.02	0	-0.03	-0.08	-0.13	-0.14	0.01	-0.34	0.02	-0.01	-0.29
260	QIAN880103	Weights for alpha-helix at the window position of -4	AAindex	0.64	0.03	0.33	0.51	-0.03	-0.09	-0.23	-0.22	-0.17	0.41	0.13	0.09	-0.43	-0.23	-0.1	-0.1	-0.07	-0.01	-0.02	-0.38
261	QIAN880104	Weights for alpha-helix at the window position of -3	AAindex	0.29	-0.05	0.11	0.28	0.24	-0.67	-0.26	0	-0.19	0.47	0.27	-0.04	-0.34	0.26	-0.03	-0.17	-0.2	-0.01	0.25	-0.3
262	QIAN880105	Weights for alpha-helix at the window position of -2	AAindex	0.68	-0.15	-0.02	0.44	0.06	-0.73	-0.14	-0.08	0.03	0.61	0.39	-0.09	-0.76	-0.15	-0.22	-0.26	-0.1	0.12	0.2	-0.04
263	QIAN880106	Weights for alpha-helix at the window position of -1	AAindex	0.34	-0.18	0.06	0.2	0.15	-0.88	-0.09	-0.03	-0.11	0.2	0.43	-0.33	-0.81	0.01	0.22	-0.35	-0.37	0.13	0.07	-0.31
264	QIAN880107	Weights for alpha-helix at the window position of 0	AAindex	0.57	-0.15	-0.46	0.26	0.03	-0.71	-0.05	0	0.16	0.48	0.41	-0.36	-1.12	0.15	0.23	-0.47	-0.54	0.31	-0.1	-0.35
265	QIAN880108	Weights for alpha-helix at the window position of 1	AAindex	0.33	-0.03	-0.44	0.21	0.48	-0.46	0.27	-0.33	0.23	0.57	0.79	-0.19	-1.86	0.19	0.1	-0.23	-0.33	0.24	0.15	-0.19
266	QIAN880109	Weights for alpha-helix at the window position of 2	AAindex	0.13	-0.09	-0.71	0.13	0.15	-0.39	0.32	0	0.37	0.5	0.63	-0.07	-1.4	0.12	0.08	-0.28	-0.21	0.17	0.02	-0.1
267	QIAN880110	Weights for alpha-helix at the window position of 3	AAindex	0.31	-0.26	-0.81	-0.06	0.1	-0.42	0.51	-0.15	0.47	0.56	0.58	-0.1	-1.33	0.41	0.18	-0.49	-0.44	-0.01	0.14	-0.08
268	QIAN880111	Weights for alpha-helix at the window position of 4	AAindex	0.21	-0.12	-0.58	-0.23	-0.06	-0.15	0.37	0.31	0.28	0.7	0.61	-0.04	-1.03	0.13	0.07	-0.28	-0.25	0	0.21	0.16
269	QIAN880112	Weights for alpha-helix at the window position of 5	AAindex	0.18	-0.29	-0.32	-0.25	0.05	-0.4	0.28	-0.03	0.41	0.62	0.21	-0.03	-0.84	-0.27	0.21	-0.05	-0.16	0.06	0.32	0.11
270	QIAN880113	Weights for alpha-helix at the window position of 6	AAindex	-0.08	-0.25	-0.24	-0.19	0	-0.1	0.29	-0.01	0.45	0.28	0.11	-0.08	-0.42	-0.28	0.05	0.07	-0.33	-0.13	0.36	0
271	QIAN880114	Weights for beta-sheet at the window position of -6	AAindex	-0.18	-0.26	0.05	-0.06	-0.18	0.23	0.24	-0.42	0.03	-0.23	-0.42	0.28	-0.13	0.21	-0.13	0.41	0.33	-0.07	-0.1	-0.1
272	QIAN880115	Weights for beta-sheet at the window position of -5	AAindex	-0.01	-0.27	-0.09	0.09	-0.12	0.13	0.22	-0.27	0.08	-0.25	-0.57	0.41	0.26	0.01	0.02	0.44	0.35	-0.09	-0.15	0.15
273	QIAN880116	Weights for beta-sheet at the window position of -4	AAindex	-0.19	-0.29	-0.06	-0.1	-0.32	0.19	-0.16	-0.08	-0.09	-0.42	-0.38	0.02	0.05	0.02	0.03	0.25	0.22	-0.15	-0.19	0.05
274	QIAN880117	Weights for beta-sheet at the window position of -3	AAindex	-0.14	-0.64	-0.1	-0.39	0.08	0.46	-0.04	0.16	0.04	-0.57	0.24	-0.27	0.02	-0.11	0.14	-0.12	0	0.29	-0.1	0.18
275	QIAN880118	Weights for beta-sheet at the window position of -2	AAindex	-0.31	-0.06	-0.54	-0.52	0.24	0.37	-0.32	0.57	-0.29	0.09	0.29	-0.53	-0.31	0.07	0.25	0.11	0.03	0.48	0.15	0.29
276	QIAN880119	Weights for beta-sheet at the window position of -1	AAindex	-0.1	0.13	-0.89	-0.34	0.36	-0.45	-0.34	0.95	-0.46	0.32	0.43	-0.89	-0.91	-0.04	0.19	-0.12	0.49	0.76	0.34	0.42
277	QIAN880120	Weights for beta-sheet at the window position of 0	AAindex	-0.25	0.13	-1.01	-0.62	0.48	-0.72	-0.16	1.1	-0.59	0.23	0.32	-0.77	-1.24	-0.12	-0.02	-0.31	0.17	0.69	0.45	0.77
278	QIAN880121	Weights for beta-sheet at the window position of 1	AAindex	-0.26	0.47	-0.55	-0.75	0.2	-0.56	-0.04	0.94	-0.55	0.25	-0.05	-0.34	-1.28	-0.33	-0.09	-0.28	0.08	0.67	0.22	0.53
279	QIAN880122	Weights for beta-sheet at the window position of 2	AAindex	0.05	0.36	-0.11	-0.35	0.2	0.14	0.02	0.47	-0.51	0.32	-0.1	-0.4	-0.79	-0.67	-0.11	0.03	-0.15	0.58	0.09	0.34
280	QIAN880123	Weights for beta-sheet at the window position of 3	AAindex	-0.44	0.13	-0.2	-0.28	-0.13	0.08	0.09	-0.04	-0.33	-0.12	-0.21	0.05	-0.48	-0.58	-0.13	0.27	0.47	0.06	-0.22	-0.11
281	QIAN880124	Weights for beta-sheet at the window position of 4	AAindex	-0.31	-0.11	0.13	-0.05	-0.04	0.45	-0.06	-0.25	-0.44	-0.44	-0.28	0.06	-0.29	-0.47	-0.1	0.34	0.27	0.11	-0.08	0.06
282	QIAN880125	Weights for beta-sheet at the window position of 5	AAindex	-0.02	-0.02	0.11	0.1	-0.03	0.38	-0.09	-0.48	-0.39	-0.26	-0.14	0.03	-0.04	-0.17	0.04	0.41	0.36	-0.18	-0.01	-0.08
283	QIAN880126	Weights for beta-sheet at the window position of 6	AAindex	-0.06	-0.19	0.24	-0.04	-0.33	0.17	0.19	-0.2	-0.43	-0.46	-0.52	0.1	0.37	-0.04	0.02	0.43	0.5	0	-0.32	0.35
284	QIAN880127	Weights for coil at the window position of -6	AAindex	-0.05	0.3	0.15	-0.02	0.09	-0.14	-0.07	0.26	-0.42	0.04	0.25	0	0.31	-0.08	0.06	-0.11	-0.06	0.04	0.19	0.33
285	QIAN880128	Weights for coil at the window position of -5	AAindex	-0.19	0.41	0.09	-0.2	0.07	0.28	-0.19	-0.06	-0.2	0.34	0.45	-0.38	0.04	0.04	0.17	-0.23	-0.02	0.05	0.16	0.22
286	QIAN880129	Weights for coil at the window position of -4	AAindex	-0.43	0.19	-0.31	-0.41	0.25	-0.21	0.21	0.29	0.33	-0.1	-0.01	0	0.28	0.14	0.06	-0.23	-0.26	-0.1	0.15	0.09
287	QIAN880130	Weights for coil at the window position of -3	AAindex	-0.19	0.42	-0.27	-0.22	-0.31	0.17	0.17	-0.34	0	-0.22	-0.53	0.17	0.14	-0.29	-0.07	0.22	0.1	-0.33	-0.15	-0.02
288	QIAN880131	Weights for coil at the window position of -2	AAindex	-0.25	0.18	0.6	-0.12	-0.29	0.09	0.42	-0.54	0.14	-0.55	-0.47	0.61	0.89	0.09	0.12	0.24	0.16	-0.45	-0.44	-0.19
289	QIAN880132	Weights for coil at the window position of -1	AAindex	-0.27	0	0.54	-0.12	-0.47	1.14	0.18	-0.74	0.45	-0.54	-0.76	0.71	1.4	-0.08	-0.4	0.4	-0.1	-0.86	-0.46	-0.05
290	QIAN880133	Weights for coil at the window position of 0	AAindex	-0.42	-0.18	0.95	-0.09	-0.39	1.24	0.05	-1.17	0.09	-0.69	-0.86	0.81	1.77	-0.01	-0.23	0.63	0.29	-1.32	-0.37	-0.41
291	QIAN880134	Weights for coil at the window position of 1	AAindex	-0.24	-0.38	0.65	0.07	-0.61	0.85	-0.21	-0.65	0.17	-0.8	-0.71	0.45	2.27	0.01	-0.04	0.33	0.13	-0.99	-0.44	-0.49
292	QIAN880135	Weights for coil at the window position of 2	AAindex	-0.14	-0.09	0.66	0.06	-0.25	0.36	-0.31	-0.51	-0.14	-0.8	-0.56	0.35	1.59	0.11	0.21	0.32	0.21	-0.7	-0.17	-0.35
293	QIAN880136	Weights for coil at the window position of 3	AAindex	0.01	-0.31	0.78	0.09	-0.2	0.14	-0.56	-0.09	-0.43	-0.81	-0.49	-0.11	1.14	-0.13	-0.13	0.13	-0.02	-0.11	-0.2	0.1
294	QIAN880137	Weights for coil at the window position of 4	AAindex	-0.3	0.03	0.44	0.18	0.11	-0.12	-0.2	-0.07	0.06	-0.18	-0.44	-0.12	0.77	0.24	-0.09	-0.09	-0.27	-0.06	-0.09	-0.25
295	QIAN880138	Weights for coil at the window position of 5	AAindex	-0.23	0.19	0.34	0.28	-0.02	0.14	-0.22	0.42	-0.15	-0.36	-0.19	0.06	0.78	0.47	-0.2	-0.29	-0.3	0.29	-0.18	0.07
296	QIAN880139	Weights for coil at the window position of 6	AAindex	0.08	0.37	0.04	0.36	0.34	-0.02	-0.45	0.09	-0.27	0.24	0.16	-0.06	0.16	0.48	-0.01	-0.35	-0.04	0.18	-0.06	-0.2
297	RACS770101	Average reduced distance for C-alpha	AAindex	0.934	0.9	0.994	0.986	0.773	1.015	0.882	0.766	1.04	0.825	0.804	0.986	1.047	1.047	0.962	1.056	1.008	0.825	0.848	0.931
298	RACS770102	Average reduced distance for side chain	AAindex	0.941	0.866	1.071	1.1	0.723	1.055	0.911	0.742	1.232	0.798	0.781	1.038	1.093	1.15	1.112	1.082	1.043	0.817	0.867	1.05
299	RACS770103	Side chain orientational preference	AAindex	1.16	0.5	2.66	2.4	0.43	1.63	0.86	0.57	3.9	0.51	0.4	1.97	2.04	3.87	1.72	1.61	1.48	0.59	0.75	1.72
300	RACS820101	Average relative fractional occurrence in A0(i)	AAindex	0.85	0.9	1.5	1.79	0.85	1.54	1.59	0.67	0.88	1.03	1.17	0.88	1.47	1.71	2.02	1.5	1.96	0.89	0.83	1.34
301	RACS820102	Average relative fractional occurrence in AR(i)	AAindex	1.58	1.04	0.98	1.49	1	0.66	0.99	1.09	1.27	1.21	1.41	0.77	1.46	1.24	1.14	1.05	0.87	0.88	1.23	0.68
302	RACS820103	Average relative fractional occurrence in AL(i)	AAindex	0.82	0	2.64	2.62	0	1.63	0	2.32	2.86	0	0	2.07	0	0	2.6	1.23	2.48	1.62	0	1.9
303	RACS820104	Average relative fractional occurrence in EL(i)	AAindex	0.78	3.14	1.25	0.94	1.07	1.13	1.03	1.26	0.85	0.91	0.41	1.32	1.73	0.93	1.75	1.31	1.57	1.11	0.98	1.31
304	RACS820105	Average relative fractional occurrence in E0(i)	AAindex	0.88	1.14	1.16	1.01	1.52	0.7	1.87	1.61	0.83	1.09	1.71	1.02	0.87	0.93	0.99	1.14	0.96	1.56	1.96	1.68
305	RACS820106	Average relative fractional occurrence in ER(i)	AAindex	0.3	0.72	1.26	1.33	1.2	3.09	1.33	0.45	0.71	0.96	1.89	2.73	0.83	0.97	0.9	1.16	0.97	0.64	1.58	0.86
306	RACS820107	Average relative fractional occurrence in A0(i-1)	AAindex	0.4	2.98	1.59	1.26	1.27	1.89	2.71	1.31	0.87	0.57	0	1.24	0.38	0.5	1.2	0.92	1.38	0.95	1.53	1.79
307	RACS820108	Average relative fractional occurrence in AR(i-1)	AAindex	1.48	0.86	1.19	1.43	1.3	0.46	1.27	1.12	1.36	1.33	1.41	0.99	0.25	1.42	1.02	0.89	0.81	0.93	1.27	0.91
308	RACS820109	Average relative fractional occurrence in AL(i-1)	AAindex	0	0	2.15	0	2.11	6.49	0	0	0	0	0	4.14	1.99	0	0	0	1.24	0	0	1.9
309	RACS820110	Average relative fractional occurrence in EL(i-1)	AAindex	1.02	1.05	1.76	0.83	0.41	2.39	0.4	0.83	0.94	1.06	1.33	1.31	2.73	1.05	1	1.18	0.77	0.88	1.22	1.09
310	RACS820111	Average relative fractional occurrence in E0(i-1)	AAindex	0.93	1.08	0.6	0.73	1.51	0.78	1.08	1.74	1	1.03	1.31	0.92	1.37	0.94	1.52	0.97	1.38	1.7	1.12	1.65
311	RACS820112	Average relative fractional occurrence in ER(i-1)	AAindex	0.99	2.32	1.18	1.36	1.25	1.4	1.06	0.81	0.91	1.26	1	1.15	0	1.52	1.19	1.5	1.18	1.01	1.33	1.09
312	RACS820113	Value of theta(i)	AAindex	17.05	28.84	19.27	20.12	16.26	38.14	23.07	16.66	16.46	10.89	20.61	34.81	23.94	15.42	21.25	19.95	18.92	17.06	23.36	26.49
313	RACS820114	Value of theta(i-1)	AAindex	14.53	30.57	19.78	18.19	19.61	37.16	22.63	20.28	14.07	14.3	20.61	13.59	52.63	22.18	17.82	18.56	21.09	21.87	19.78	26.36
314	RADA880101	Transfer free energy from chx to wat	AAindex	1.81	1.28	-8.72	-6.81	2.98	0.94	-4.66	4.92	-5.55	4.92	2.35	-6.64	0	-5.54	-14.92	-3.4	-2.57	4.04	2.33	-0.14
315	RADA880102	Transfer free energy from oct to wat	AAindex	0.52	0	0	-0.79	2.09	0	0.95	2.04	0.08	1.76	1.32	-0.01	0	-0.07	-1.32	0.04	0.27	1.18	2.51	1.63
316	RADA880103	Transfer free energy from vap to chx	AAindex	0.13	-2.52	-2.23	-3.43	-3.74	1.45	-5.61	-2.77	-3.97	-2.64	-3.83	-3.04	0	-3.84	-5	-1.66	-2.31	-2.05	-8.21	-5.97
317	RADA880104	Transfer free energy from chx to oct	AAindex	1.29	0	0	-6.02	0.89	0.94	-5.61	2.88	-5.63	3.16	1.03	-6.63	0	-5.47	-13.6	-3.44	-2.84	2.86	-0.18	-1.77
318	RADA880105	Transfer free energy from vap to oct	AAindex	1.42	0	0	-9.45	-2.85	2.39	-11.22	0.11	-9.6	0.52	-2.8	-9.67	0	-9.31	-18.6	-5.1	-5.15	0.81	-8.39	-7.74
319	RADA880106	Accessible surface area	AAindex	93.7	135.2	142.6	182.9	228.6	52.6	188.1	182.2	215.2	173.7	197.6	146.3	0	177.7	250.4	109.5	142.1	157.2	271.6	239.9
320	RADA880107	Energy transfer from out to in(95%buried	AAindex	-0.29	0	-1.02	-0.9	0	-0.34	-0.94	0.24	-2.05	-0.12	-0.24	-1.18	0	-1.53	-2.71	-0.75	-0.71	0.09	-0.59	-1.02
321	RADA880108	Mean polarity	AAindex	-0.06	1.36	-0.8	-0.77	1.27	-0.41	0.49	1.31	-1.18	1.21	1.27	-0.48	0	-0.73	-0.84	-0.5	-0.27	1.09	0.88	0.33
322	RICJ880101	Relative preference value at N"	AAindex	0.7	0.6	1.4	1	1.2	1.6	1.2	0.9	1	0.9	0.3	1.2	0.7	1	0.4	1.6	0.3	0.7	1.1	1.9
323	RICJ880102	Relative preference value at N'	AAindex	0.7	0.6	1.4	1	1.2	1.6	1.2	0.9	1	0.9	0.3	1.2	0.7	1	0.4	1.6	0.3	0.7	1.1	1.9
324	RICJ880103	Relative preference value at N-cap	AAindex	0.5	0.6	2.1	0.4	0.2	1.8	1.1	0.2	0.7	0.2	0.8	3.5	0.8	0.4	0.4	2.3	1.6	0.1	0.3	0.8
325	RICJ880104	Relative preference value at N1	AAindex	1.2	0.8	0.8	2.2	0.5	0.3	0.7	0.9	0.6	0.9	0.3	0.7	2.6	0.7	0.7	0.7	0.8	1.1	2.1	1.8
326	RICJ880105	Relative preference value at N2	AAindex	1.6	1.2	2.6	2	0.9	0.9	0.7	0.7	1	0.3	1	0.7	0.5	0.8	0.9	0.8	0.7	0.6	1.7	0.4
327	RICJ880106	Relative preference value at N3	AAindex	1	0.6	2.2	3.3	0.6	0.6	0.7	0.4	0.8	0.6	1	0.7	0.4	1.5	0.4	0.4	1	1.1	1.4	1.2
328	RICJ880107	Relative preference value at N4	AAindex	1.1	1.1	0.3	0.5	1.9	0.4	1.5	1.1	0.8	2.6	1.7	0	0.1	1.3	1.5	0.4	0.5	1.5	3.1	0.6
329	RICJ880108	Relative preference value at N5	AAindex	1.4	1.6	0.6	0.9	1	0.6	0.9	0.9	1.9	1.1	1.7	1.2	0.3	1.4	1.2	1.1	0.6	0.8	1.4	0.2

Table S11 Amino acid descriptors (1D) used in this study.

No.	Descriptor ID	Attribute	Ref*	A	C	D	E	F	G	H	I	K	L	M	N	P	Q	R	S	T	V	W	Y
330	RICJ880109	Relative preference value at Mid	AAindex	1.8	0.7	1	0.8	1.3	0.5	1	1.2	1.1	1.2	1.5	0.9	0.3	1.3	1.3	0.6	1	1.2	1.5	0.8
331	RICJ880110	Relative preference value at C5	AAindex	1.8	0	0.7	1.1	1.9	0.5	2.4	1.3	1.4	1.2	2.7	0.6	0.3	1	1	0.5	0.5	0.4	1.1	1.3
332	RICJ880111	Relative preference value at C4	AAindex	1.3	0.7	0.5	0.7	2.9	0.5	1.9	1.6	1	1.4	2.8	0.6	0	0.2	0.8	0.5	0.6	1.4	2.1	0.8
333	RICJ880112	Relative preference value at C3	AAindex	0.7	0.2	0.6	1.6	1.8	0.1	1.1	1.4	2.2	1.9	1	0.8	0	1.3	0.8	0.6	0.7	1.3	0.4	1.1
334	RICJ880113	Relative preference value at C2	AAindex	1.4	1.2	0.7	1.7	0.3	0.2	1.8	0.4	1.9	0.8	1.3	0.9	0.2	1.6	2.1	1.6	0.9	0.7	0.4	0.3
335	RICJ880114	Relative preference value at C1	AAindex	1.1	1.6	0.4	0.8	0.7	0.2	3.4	0.7	2	0.7	1	1.2	0	2.1	1	1.7	1	0.7	0	1.2
336	RICJ880115	Relative preference value at C-cap	AAindex	0.8	0.4	0.7	0.3	0.5	3.9	1.3	0.7	1.3	0.7	0.8	1.6	0.7	0.9	0.9	0.8	0.3	0.2	0	0.8
337	RICJ880116	Relative preference value at C'	AAindex	1	0.8	1.4	0.8	0.1	1.2	1.2	1.1	1.2	0.9	0.8	0.9	1.9	1.4	1.4	0.7	0.8	0.6	0.4	0.9
338	RICJ880117	Relative preference value at C"	AAindex	0.7	0.4	1.4	0.7	1.2	0.6	1	0.7	1.3	0.5	0	1.5	1.5	1.1	1.1	0.9	2.1	1	2.7	0.5
339	ROBB760101	Information measure for alpha-helix	AAindex	6.5	-1.3	0.5	7.8	1.6	-8.6	1.2	0.6	2.3	3.2	5.3	-5.1	-7.7	1	-0.9	-3.9	-2.6	1.4	1.2	-4.5
340	ROBB760102	Information measure for N-terminal helix	AAindex	2.3	0.8	7.4	10.3	-1.1	-5.2	-2.8	-4	-4.1	-2.1	-3.5	0.3	8.1	-0.7	-5.2	-3.5	2.3	-4.4	-0.9	-3.7
341	ROBB760103	Information measure for middle helix	AAindex	6.7	-4.9	-3.1	2.2	2.8	-6.8	-1	3.2	0.5	5.5	7.2	-6.1	-22.8	0.6	0.3	-3	-4	2.5	4	-4.6
342	ROBB760104	Information measure for C-terminal helix	AAindex	2.3	6.1	-4.4	2.5	1.6	-8.3	5.9	-0.5	7.3	0.1	3.5	-3.3	-24.4	2.7	1.4	-1.9	-3.7	2.3	-0.9	-0.6
343	ROBB760105	Information measure for extended	AAindex	-2.3	4.4	-4.4	-5	2.6	-4.2	-2.5	6.7	-3.3	2.3	2.3	-4.1	-1.8	1.2	0.4	-1.7	1.3	6.8	-1	4
344	ROBB760106	Information measure for pleated-sheet	AAindex	-2.7	3.7	-4.4	-8.1	3	-3.9	-3	7.7	-2.9	3.7	3.7	-4.2	-6.6	0.8	0.4	-2.4	1.7	7.1	0.3	3.3
345	ROBB760107	Information measure for extended without H-bond	AAindex	0	5.4	-2.6	3.1	0.7	-3.4	0.8	-0.1	-3.1	-3.7	-2.1	-2	7.4	2.4	1.1	1.3	0	2.7	-3.4	4.8
346	ROBB760108	Information measure for turn	AAindex	-5	4.4	3.1	-4.7	-1.8	5.7	-0.3	-4.6	1	-5.6	-4.8	4.2	2.6	0.4	2.1	2.6	0.3	-6	3.4	2.9
347	ROBB760109	Information measure for N-terminal turn	AAindex	-3.3	-0.3	3.9	-1.8	0.8	-1.2	3	-0.5	-1.2	-2.3	-4.3	5.4	6.5	-0.4	0	1.8	-0.7	-3.5	-0.8	3.1
348	ROBB760110	Information measure for middle turn	AAindex	-4.7	6.2	1.9	-4.2	-3.7	5.7	-2.6	-7	2.8	-6.2	-4.8	3.9	3.6	-2	2	2.1	0.6	-6.2	3.3	3.8
349	ROBB760111	Information measure for C-terminal turn	AAindex	-3.7	4	-0.6	-4.3	1.6	5.9	-0.8	-0.5	1.3	-2.8	-1.6	-0.6	-6	3.4	1	1.5	1.2	-4.6	6.5	1.3
350	ROBB760112	Information measure for coil	AAindex	-2.5	-4.7	0	-4.4	-4.1	4.9	1.6	-3.3	-0.8	-2	-4.1	4.6	5.8	-0.5	-1.2	2.5	1.7	-3.5	1.2	-0.6
351	ROBB760113	Information measure for loop	AAindex	-5.1	3.8	3.1	-5.2	-2.4	5.6	-0.9	-4.5	1	-5.4	-5.3	4.7	3.5	0.2	2.6	3.2	0	-6.3	2.9	3.2
352	ROBB790101	Hydration free energy	AAindex	-1	2.1	-1.2	-0.7	2.8	0.3	1.1	4	-0.9	2	1.8	-0.7	0.4	-0.1	0.3	-1.2	-0.5	1.4	3	2.1
353	ROSG850101	Mean area buried on transfer	AAindex	86.6	132.3	97.8	113.9	194.1	62.9	155.8	158	115.5	164.1	172.9	103.3	92.9	119.2	162.2	85.6	106.5	141	224.6	177.7
354	ROSG850102	Mean fractional area loss	AAindex	0.74	0.91	0.62	0.62	0.88	0.72	0.78	0.88	0.52	0.85	0.85	0.63	0.64	0.62	0.64	0.66	0.7	0.86	0.85	0.76
355	ROSM880101	Side chain hydropathy, uncorrected for solvation	AAindex	-0.67	-0.34	8.72	7.35	-3.24	0	3.82	-3.02	6.13	-3.02	-1.3	7.23	-1.75	6.39	12.1	4.35	3.86	-2.18	-2.86	0.98
356	ROSM880102	Side chain hydropathy, corrected for solvation	AAindex	-0.67	-2	1.57	1.78	-3.24	0	1.09	-3.02	2.46	-3.02	-1.67	2.27	-1.75	2.12	3.89	0.1	-0.42	-2.18	-2.86	0.98
357	ROSM880103	Loss of Side chain hydropathy by helix formation	AAindex	0.4	0.5	0.8	1.3	0.7	0	1	0.4	0.4	0.6	0.3	0.9	0.9	0.7	0.3	0.4	0.4	0.4	0.6	1.2
358	SIMZ760101	Transfer free energy	AAindex	0.73	0.7	0.54	0.55	2.65	0	1.1	2.97	1.5	2.49	1.3	-0.01	2.6	-0.1	0.73	0.04	0.44	1.69	3	2.97
359	SNEP660101	Principal component I	AAindex	0.239	0.22	0.171	0.187	0.234	0.16	0.205	0.273	0.228	0.281	0.253	0.249	0.165	0.26	0.211	0.236	0.213	0.255	0.183	0.193
360	SNEP660102	Principal component II	AAindex	0.33	0.074	-0.371	-0.409	-0.011	0.37	-0.078	0.149	-0.075	0.129	-0.092	-0.233	0.37	-0.254	-0.176	0.022	0.136	0.245	-0.011	-0.138
361	SNEP660103	Principal component III	AAindex	0.079	-0.067	-0.184	-0.073	-0.016	0.32	0.001	-0.008	-0.041	0.049	0.438	-0.285	-0.153	-0.246	-0.136	-0.208	0.493	0.0099474	0.381	-0.155
362	SNEP660104	Principal component IV	AAindex	-0.167	-0.025	0.38	-0.017	-0.036	0.056	-0.309	-0.264	0.077	-0.371	0.074	-0.079	0.47	-0.184	0.166	0.348	0.05	0.0093158	0.22	-0.212
363	SUEM840101	Zimm-Bragg parameter s at 20 C	AAindex	1.071	0.922	0.68	0.97	1.086	0.591	0.85	1.14	0.939	1.14	1.2	0.784	0.659	0.977	1.033	0.76	0.817	0.95	1.107	1.02
364	SUEM840102	Zimm-Bragg parameter sigma x 1.0E4	AAindex	8	26	70	6	18	0.1	0.1	55	1	33	54	0.1	42	33	0.1	0.1	0.1	0.1	77	66
365	SWER830101	Optimal matching hydrophobicity	AAindex	-0.4	0.17	-1.31	-1.22	1.92	-0.67	-0.64	1.25	-0.67	1.22	1.02	-0.92	-0.49	-0.91	-0.59	-0.55	-0.28	0.91	0.5	1.67
366	TANS770101	Normalized frequency of alpha-helix	AAindex	1.42	0.73	1.01	1.63	1.16	0.5	1.2	1.12	1.24	1.29	1.21	0.71	0.65	1.02	1.06	0.71	0.78	0.99	1.05	0.67
367	TANS770102	Normalized frequency of isolated helix	AAindex	0.946	0.481	1.311	0.698	0.963	0.36	2.168	1.283	1.203	1.192	0	0.432	2.093	1.615	1.128	0.523	1.961	0.409	1.925	0.802
368	TANS770103	Normalized frequency of extended structure	AAindex	0.79	1.268	0.53	0.643	1.052	0.725	0.864	1.361	0.735	1.111	1.092	0.832	1.249	1.038	1.087	1.093	1.214	1.428	1.114	1.34
369	TANS770104	Normalized frequency of chain reversal R	AAindex	1.194	0.678	1.056	0.928	0.377	1.015	0.611	0.603	1.06	0.595	0.831	0.659	3.159	1.29	0.795	1.444	1.172	0.64	0.452	0.816
370	TANS770105	Normalized frequency of chain reversal S	AAindex	0.497	1.348	1.498	0.651	1.348	1.848	1.474	0.471	0.932	0.656	0.425	2.072	0.179	0.711	0.677	1.151	0.749	0.654	1.283	1.283
371	TANS770106	Normalized frequency of chain reversal D	AAindex	0.937	1.004	1.64	0.679	0.803	0.901	1.085	0.178	1.254	0.808	0.886	1.08	0.748	1.078	1.725	1.145	1.487	0.625	0.803	1.227
372	TANS770107	Normalized frequency of left-handed helix	AAindex	0.289	1.767	0.917	0.285	0.393	4.259	1.061	0.262	1.288	0	0	3.169	0	2.372	1.38	0.16	0.218	0.167	0	0.654
373	TANS770108	Normalized frequency of zeta R	AAindex	0.328	0	3.379	0	1.336	0.5	1.204	2.078	0.835	0.414	0.982	1.498	0.415	0	2.088	1.089	1.732	0.946	1.781	0
374	TANS770109	Normalized frequency of coil	AAindex	0.945	0.932	1.315	1.014	0.622	2.355	0.525	0.673	0.947	0.758	1.028	1.202	0.579	0.704	0.364	1.14	0.863	0.561	0.777	0.907
375	TANS770110	Normalized frequency of chain reversal	AAindex	0.842	1.032	1.366	0.758	0.881	1.349	1.079	0.459	1.045	0.665	0.668	1.352	1.385	0.998	0.936	1.257	1.055	0.643	0.881	1.101
376	VASM830101	Relative population of conformational state A	AAindex	0.135	0.159	0.289	0.184	0.087	0.051	0.223	0.173	0.17	0.215	0.239	0.196	0.151	0.236	0.296	0.01	0.1	0.285	0.166	0.066
377	VASM830102	Relative population of conformational state C	AAindex	0.507	0.592	0.223	0.445	0.077	0.39	0.31	0.111	0.559	0.619	0.431	0.287	0.739	0.383	0.459	0.689	0.785	0.356	0.16	0.06
378	VASM830103	Relative population of conformational state E	AAindex	0.159	0.187	0.283	0.206	0.682	0.049	0.233	0.581	0.159	0.083	0.198	0.385	0.366	0.236	0.194	0.15	0.074	0.301	0.463	0.737
379	VELV850101	Electron-ion interaction potential	AAindex	0.09593	0.07606	0.08292	0.00499	0.01979	0.02415	0	0	0.08226	0.0371	0.0946	0.1263	0.08292	0.0058	0.00359	0.09408	0.05481	0.0496095	0.05159	0.00569
380	VENT840101	Bitterness	AAindex	0	0	0	0	1	0	0	1	0	1	0	0	0	0	0	0	0	1	1	1
381	VHEG790101	Transfer free energy to lipophilic phase	AAindex	39.23	2.16	3.95	-7.85	5.82	6.28	-18.32	-17.79	-8.86	9.71	-21.98	23.22	-1.54	16.81	4.25	-4.15	-16.19	-0.1568421	-1.51	-16.22
382	WARP780101	Average interactions per side chain atom	AAindex	10.04	8.89	5.76	5.37	7.98	7.99	7.49	8.72	4.4	8.79	9.15	5.63	7.79	5.41	6.18	7.08	7	8.88	8.07	6.9
383	WEBA780101	RF value in high salt chromatography	AAindex	0.89	0.85	0.87	0.84	0.52	0.92	0.83	0.76	0.97	0.73	0.74	0.89	0.82	0.82	0.88	0.96	0.92	0.85	0.2	0.49
384	WERD780101	Propensity to be buried inside	AAindex	0.52	0.83	0.37	0.38	0.87	0.41	0.7	0.79	0.31	0.77	0.76	0.42	0.35	0.35	0.49	0.49	0.38	0.72	0.86	0.64
385	WERD780102	Free energy change of epsilon(i) to epsilon(ex	AAindex	0.16	-0.12	-0.24	-0.45	-0.25	-0.16	-0.18	-0.19	-0.12	-0.44	-0.79	1.03	-0.59	-0.55	-0.2	-0.01	0.05	-0.46	-0.33	-0.42
386	WERD780103	Free energy change of alpha(Ri) to alpha(Rh	AAindex	0.15	-0.19	-0.22	0.14	1.18	0.36	-0.25	0.02	-0.16	0.06	0.11	0.69	0.11	-0.06	-0.37	0.13	0.28	-0.08	-0.12	0.19
387	WERD780104	Free energy change of epsilon(i) to alpha(Rh	AAindex	-0.07	0.17	-0.8	-0.63	0.4	0.27	-0.49	0.06	-0.45	-0.17	0.03	-0.57	-0.47	-0.26	-0.4	-0.11	0.09	-0.11	-0.61	-0.61
388	WOEC730101	Polar requirement	AAindex	7	5.5	13	12.5	5	7.9	8.4	4.9	10.1	4.9	5.3	10	6.6	8.6	9.1	7.5	6.6	5.6	5.3	5.7
389	WOLR810101	Hydration potential	AAindex	1.94	-1.24	-10.95	-10.2	-0.76	2.39	-10.27	2.15	-9.52	2.28	-1.48	-9.68	-3.68	-9.38	-19.92	-5.06	-4.88	1.99	-5.88	-6.11
390	WOLS870101	Principal property value z1	AAindex	0.07	0.71	3.64	3.08	-4.92	2.23	2.41	-4.44	2.84	-4.19	-2.49	3.22	-1.22	2.18	2.88	1.96	0.92	-2.69	-4.75	-1.39
391	WOLS870102	Principal property value z2	AAindex	-1.73	-0.97	1.13	0.39	1.3	-5.36	1.74	-1.68	1.41	-1.03	-0.27	1.45	0.88	0.53	2.52	-1.63	-2.09	-2.53	3.65	2.32
392	WOLS870103	Principal property value z3	AAindex	0.09	4.13	2.36	-0.07	0.45	0.3	1.11	-1.03	-3.14	-0.98	-0.41	0.84	2.23	-1.14	-3.44	0.57	-1.4	-1.29	0.85	0.01
393	YUTK870101	Unfolding Gibbs energy in water, pH7.0	AAindex	8.5	11	8.5	8.8	11.2	7.1	10.1	16.8	7.9	15	13.3	8.2	8.2	6.3	0	7.4	8.8	12	9.9	8.8
394	YUTK870102	Unfolding Gibbs energy in water, pH9.0	AAindex	6.8	8.3	7	4.9	8.3	6.4	9.2	10	7.5	12.2	8.4	6.2	6.9	8.5	0	8	7	9.4	5.7	6.8
395	YUTK870103	Activation Gibbs energy of unfolding, pH7.0	AAindex	18.08	18.17	17.36	18.16	17.3	18.24	18.49	18.62	17.96	18.6	18.11	17.47	18.16	17.93	0	17.57	17.54	18.3	17.19	17.99
396	YUTK870104	Activation Gibbs energy of unfolding, pH9.0	AAindex	18.56	17.84	17.94	17.97	17.95	18.57	18.64	19.21	18.36	19.01	18.49	18.24	18.77	18.51	0	18.06	17.71	18.98	16.87	18.23
397	ZASB820101	Dependence of partition coefficient on ionic strength	AAindex	-0.089	-0.181	0	-0.19	-0.181	0	-0.086	-0.102	-0.107	-0.062	0.001	-0.355	-0.203	-0.411	-0.203	-0.17	0.275	-0.1152105	0	-0.125
398	ZIMJ680101	Hydrophobicity	AAindex	0.83	1.48	0.64	0.65	2.75	0.1	1.1	3.07	1.6	2.52	1.4	0.09	2.7	0	0.83	0.14	0.54	1.79	0.31	2.97
399	ZIMJ680102	Bulkiness	AAindex	11.5	13.46	11.68	13.57	19.8	3.4	13.69	21.4	15.71	21.4	16.25	12.82	17.43	14.45	14.28	9.47	15.77	21.57	21.67	18.03
400	ZIMJ680103	Polarity	AAindex	0	1.48	49.7	49.9	0.35	0	51.6	0.13	49.5	0.13	1.43	3.38	1.58	3.53	52	1.67	1.66	0.13	2.1	1.61
401	ZIMJ680104	Isoelectric point	AAindex	6	5.05	2.77	3.22	5.48	5.97	7.59	6.02	9.74	5.98	5.74	5.41	6.3	5.65	10.76	5.68	5.66	5.96	5.89	5.66
402	ZIMJ680105	RF rank	AAindex	9.9	2.8	2.8	3.2	18.8	5.6	8.2	17.1	3.5	17.6	14.9	5.4	14.8	9	4.6	6.9	9.5	14.3	17.1	15
403	AURR980101	Normalized positional residue frequency at helix termini N4'(Aurora-Rose, 199	AAindex	0.94	0.6	1.19	1.41	1.06	1.18	1.15	1.07	1.03	0.95	0.88	0.79	1.18	0.94	1.15	0.69	0.87	0.9	0.91	1.04
404	AURR980102	Normalized positional residue frequency at helix termini N'''	AAindex	0.98	0.41	1.05	1.04	1.12	1.25	1.01	0.88	1.06	0.8	1.12	1.05	1.31	0.9	1.14	1.02	0.8	0.87	0.9	1.12
405	AURR980103	Normalized positional residue frequency at helix termini N''	AAindex	1.05	0.6	1.39	1.11	0.95	1.26	1.43	0.95	0.97	0.96	0.99	0.91	1.05	0.87	0.81	0.96	1.03	0.62	1.06	0.94
406	AURR980104	Normalized positional residue frequency at helix termini N'(Aurora-Rose, 199	AAindex	0.75	0.66	1.72	1.1	0.88	1.14	0.96	0.8	0.66	1.01	1.02	1.24	1.33	1.08	0.9	1.2	1.13	0.58	0.68	0.8
407	AURR980105	Normalized positional residue frequency at helix termini Nc	AAindex	0.67	0.37	1.58	0.94	0.96	0.98	0.83	0.78	0.84	0.79	0.98	1.28	1.12	1.05	0.76	1.25	1.41	0.67	0.94	0.82
408	AURR980106	Normalized positional residue frequency at helix termini N1	AAindex	1.1	0.26	1.14	2.3	0.9	0.55	0.83	1.06	1.08	0.84	0.9	0.72	1.67	1.31	1.05	0.81	0.77	0.76	1.26	0.99
409	AURR980107	Normalized positional residue frequency at helix termini N2	AAindex	1.39	0.52	1.64	2.07	1	0.65	1.36	0.64	0.8	0.91	1.1	0.67	0.94	1.6	0.95	0.69	0.92	0.7	1.1	0.73
410	AURR980108	Normalized positional residue frequency at helix termini N3	AAindex	1.43	0.52	0.9	1.7	1.1	0.56	0.66	1.18	0.82	1.52	1.68	0.55	0.15	1.43	1.33	0.61	0.75	1.14	1.68	0.65
411	AURR980109	Normalized positional residue frequency at helix termini N4	AAindex	1.55	0.59	0.61	1.34	1.39	0.37	0.89	1.47	1.27	1.36	2.13	0.6	0.03	1.43	1.39	0.44	0.65	1.18	1.1	0.93
412	AURR980110	Normalized positional residue frequency at helix termini N5	AAindex	1.8	0.55	0.9	1.73	0.96	0.32	0.46	1.09	1.24	1.47	1.64	0.73	0.15	0.97	1.73	0.67	0.7	0.81	0.68	0.91
413	AURR980111	Normalized positional residue frequency at helix termini C5	AAindex	1.52	0.26	1.04	1.76	1.14	0.3	0.83	1.25	1.1	1.26	1.14	0.58	0.44	1.41	1.49	0.66	0.73	1.03	0.68	1.04
414	AURR980112	Normalized positional residue frequency at helix termini C4	AAindex	1.49	0.37	0.94	1.55	0.86	0.29	0.96	1.04	1.17	1.4	1.84	0.67	0.2	1.52	1.41	0.68	0.79	0.94	1.52	1.06
415	AURR980113	Normalized positional residue frequency at helix termini C3	AAindex	1.73	0.63	0.68	1.16	1.35	0.32	0.76	1.15	1.22	1.8	2.21	0.7	0.07	0.88	1.24	0.65	0.46	0.94	1.57	1.1
416	AURR980114	Normalized positional residue frequency at helix termini C2	AAindex	1.33	0.44	0.6	1.43	1.22	0.2	1.02	1.58	1.71	1.63	1.76	0.64	0.07	1.37	1.39	0.42	0.57	1.08	1	1.02
417	AURR980115	Normalized positional residue frequency at helix termini C1	AAindex	1.87	0.33	0.91	1.88	0.67	0.33	0.89	0.9	1.63	1.65	1.35	0.7	0.03	1.24	1.66	0.71	0.5	0.51	1	0.73
418	AURR980116	Normalized positional residue frequency at helix termini Cc	AAindex	1.19	0.44	0.72	1.27	1.2	0.74	1.55	0.61	1.45	1.36	1.35	1.33	0.1	1.43	1.45	1.02	0.82	0.46	0.58	1.06
419	AURR980117	Normalized positional residue frequency at helix termini C'	AAindex	0.77	0.44	0.79	0.92	1.04	2.74	1.65	0.64	1.19	0.66	0.74	1.39	0.66	0.95	1.11	0.64	0.82	0.53	0.58	0.93
420	AURR980118	Normalized positional residue frequency at helix termini C''	AAindex	0.93	0.67	1.15	1.07	1.05	1.08	1.4	1.14	1.27	1.16	1.11	0.82	1.01	1.02	0.96	0.71	0.84	0.74	1.06	1.15
421	AURR980119	Normalized positional residue frequency at helix termini C'''	AAindex	1.09	0.26	1.17	1.31	0.84	0.97	0.88	0.97	1.13	0.87	0.96	1.03	2.01	1.08	1.29	0.76	0.79	0.77	0.91	0.64
422	AURR980120	Normalized positional residue frequency at helix termini C4'	AAindex	0.71	0.65	1.43	1.19	0.95	1.07	1.13	1.05	1.1	0.84	0.8	0.95	1.7	0.87	1.09	0.65	0.086	1.12	1.25	0.85
423	ONEK900101	Delta G values for the peptides extrapolated to 0 M urea	AAindex	13.4	11.6	11.7	12.2	12.1	11.3	11.6	12	13	13	12.8	12	6.5	12.8	13.3	12.2	11.7	11.9	12.4	12.1
424	ONEK900102	Helix formation parameters (delta delta G)	AAindex	-0.77	-0.23	-0.15	-0.27	-0.41	0	-0.06	-0.23	-0.65	-0.62	-0.5	-0.07	3	-0.33	-0.68	-0.35	-0.11	-0.14	-0.45	-0.17
425	VINM940101	Normalized flexibility parameters (B-values), average	AAindex	0.984	0.906	1.068	1.094	0.915	1.031	0.95	0.927	1.102	0.935	0.952	1.048	1.049	1.037	1.008	1.046	0.997	0.931	0.904	0.929
426	VINM940102	Normalized flexibility parameters (B-values) for each residue surrounded by none rigid neighbour	AAindex	1.315	1.196	1.372	1.376	1.247	1.382	1.279	1.241	1.367	1.234	1.269	1.38	1.342	1.342	1.31	1.381	1.324	1.235	1.186	1.199
427	VINM940103	Normalized flexibility parameters (B-values) for each residue surrounded by one rigid neighbour	AAindex	0.994	0.939	1.022	1.052	0.934	1.018	0.967	0.977	1.029	0.982	0.963	1.022	1.05	1.041	1.026	1.025	0.998	0.968	0.938	0.981
428	VINM940104	Normalized flexibility parameters (B-values) for each residue surrounded by two rigid neighbour	AAindex	0.783	0.785	0.822	0.826	0.774	0.784	0.777	0.776	0.834	0.783	0.806	0.799	0.809	0.817	0.807	0.811	0.795	0.781	0.796	0.788
429	MUNV940101	Free energy in alpha-helical conformation	AAindex	0.423	0.877	0.87	0.167	0.706	1.162	0.802	0.566	0.615	0.494	0.444	0.906	1.945	0.594	0.503	0.928	0.884	0.706	0.69	0.778
430	MUNV940102	Free energy in alpha-helical region	AAindex	0.619	1.107	0.932	0.675	0.968	1.361	1.034	0.876	0.784	0.74	0.736	1.089	1.78	0.77	0.753	0.969	1.053	0.939	0.91	1.009
431	MUNV940103	Free energy in beta-strand conformation	AAindex	1.08	0.733	1.266	1.085	0.685	1.104	0.906	0.583	1.026	0.789	0.812	1.197	1.412	1.05	0.976	0.987	0.784	0.546	0.755	0.665
432	MUNV940104	Free energy in beta-strand region	AAindex	0.978	0.573	1.038	0.962	0.585	1.405	0.724	0.502	0.841	0.766	0.729	0.915	2.613	0.863	0.784	0.784	0.569	0.444	0.671	0.56
433	MUNV940105	Free energy in beta-strand region	AAindex	1.4	1.14	1.89	1.42	1.07	2.06	1.25	1.02	1.34	1.33	1.12	1.61	3.9	1.33	1.23	1.2	0.99	0.87	1.1	0.98
434	WIMW960101	Free energies of transfer of AcWl-X-LL peptides from bilayer interface to water	AAindex	4.08	4.49	3.02	2.23	5.38	4.24	4.08	4.52	3.77	4.81	4.48	3.83	3.8	3.67	3.91	4.12	4.11	4.18	6.1	5.19
435	KIMC930101	Thermodynamic beta sheet propensity	AAindex	-0.35	-0.47	-0.41	-0.41	-0.55	0	-0.46	-0.56	-0.41	-0.48	-0.46	-0.38	-0.23	-0.4	-0.44	-0.39	-0.48	-0.53	-0.48	-0.5
436	MONM990101	Turn propensity scale for transmembrane helices	AAindex	0.5	0.6	1.6	1.6	0.4	1.3	1.6	0.6	1.6	0.4	0.5	1.7	1.7	1.6	1.7	0.7	0.4	0.5	0.7	0.6
437	BLAM930101	Alpha helix propensity of position 44 in T4 lysozyme	AAindex	0.96	0.42	0.42	0.53	0.59	0	0.57	0.84	0.73	0.92	0.86	0.39	-2.5	0.8	0.77	0.53	0.54	0.63	0.58	0.72
438	PARS000101	p-Values of mesophilic proteins based on the distributions of B values	AAindex	0.343	0.319	0.429	0.405	0.292	0.389	0.307	0.296	0.429	0.287	0.293	0.409	0.432	0.395	0.353	0.416	0.362	0.307	0.268	0.22
439	PARS000102	p-Values of thermophilic proteins based on the distributions of B values	AAindex	0.32	0.198	0.424	0.514	0.314	0.374	0.299	0.306	0.446	0.34	0.313	0.384	0.354	0.436	0.327	0.376	0.339	0.294	0.291	0.287

Table S11 Amino acid descriptors (1D) used in this study.

No.	Descriptor ID	Attribute	Ref*	A	C	D	E	F	G	H	I	K	L	M	N	P	Q	R	S	T	V	W	Y
440	KUMS000101	Distribution of amino acid residues in the 18 non-redundant families of thermophilic protein	AAindex	8.9	0.6	6.3	6.9	3.3	9.4	2.2	7	6.1	7.4	2.3	4.4	4.2	2.8	4.6	4	5.7	8.2	1.3	4.5
441	KUMS000102	Distribution of amino acid residues in the 18 non-redundant families of mesophilic protein	AAindex	9.2	1	6	6	3.4	9.4	2.1	6	6.5	7.7	2.4	5.1	4.2	2.9	3.6	5.5	5.7	8.2	1.2	3.7
442	KUMS000103	Distribution of amino acid residues in the alpha-helices in thermophilic protein	AAindex	14.1	0.1	5.7	8.8	5	4.1	2	7.1	7.7	9.1	3.3	3.2	0.7	3.7	5.5	3.9	4.4	5.9	1.2	4.5
443	KUMS000104	Distribution of amino acid residues in the alpha-helices in mesophilic protein	AAindex	13.4	0.8	4.6	7.8	4.5	4.6	3.3	6.5	7.5	10.6	3	3.7	1.3	4.8	3.9	3.8	4.6	7.1	1	3.3
444	TAKK010101	Side-chain contribution to protein stability (kJ/mol	AAindex	9.8	3	4.9	4.4	23	0	11.9	17.2	10.5	17	11.9	3.6	15	2.4	7.3	2.6	6.9	15.3	24.2	17.2
445	FODM020101	Propensity of amino acids within pi-helices	AAindex	0.7	1.17	0.87	0.96	1.34	0.64	1.39	1.29	0.91	1.44	0.91	1.47	0.12	0.73	0.95	0.84	0.74	1.2	1.8	1.68
446	NADH010101	Hydropathy scale based on self-information values in the two-state model (5% accessibility	AAindex	58	116	-97	-131	92	-11	-73	107	-24	95	78	-93	-79	-139	-184	-34	-7	100	59	-11
447	NADH010102	Hydropathy scale based on self-information values in the two-state model (9% accessibility	AAindex	51	137	-78	-115	108	-13	-55	106	-205	103	73	-84	-79	-128	-144	-26	-3	108	69	11
448	NADH010103	Hydropathy scale based on self-information values in the two-state model (16% accessibility	AAindex	41	169	-47	-90	128	-18	-35	104	-148	103	77	-74	-81	-104	-109	-31	10	116	102	36
449	NADH010104	Hydropathy scale based on self-information values in the two-state model (20% accessibility	AAindex	32	182	-29	-74	132	-22	-25	106	-124	104	82	-73	-82	-95	-95	-34	20	113	118	44
450	NADH010105	Hydropathy scale based on self-information values in the two-state model (25% accessibility	AAindex	24	194	0	-57	131	-28	-31	102	-9	103	90	-76	-85	-87	-79	-36	34	111	116	43
451	NADH010106	Hydropathy scale based on self-information values in the two-state model (36% accessibility	AAindex	5	224	45	-8	117	-47	-50	83	-38	82	83	-77	-103	-67	-57	-41	79	117	130	27
452	NADH010107	Hydropathy scale based on self-information values in the two-state model (50% accessibility	AAindex	-2	329	248	117	120	-66	-70	28	115	36	62	-97	-132	-37	-41	-52	174	114	179	-7
453	MONM990201	Averaged turn propensities in a transmembrane heli	AAindex	0.4	0.7	1.5	1.3	0.3	1.1	1.4	0.5	1.4	0.3	0.5	1.6	1.6	1.4	1.5	0.9	0.7	0.4	0.9	0.9
454	KOEP990101	Alpha-helix propensity derived from designed sequences	AAindex	-0.04	0.57	0.27	-0.33	-0.01	1.24	-0.11	-0.26	-0.18	-0.38	-0.09	0.25	0	-0.02	-0.3	0.15	0.39	-0.06	0.21	0.05
455	KOEP990102	Beta-sheet propensity derived from designed sequences	AAindex	-0.12	-0.63	1.12	0.91	-0.67	0.76	1.34	-0.77	0.29	0.15	-0.71	1.05	0	1.67	0.34	1.45	-0.7	-0.7	-0.14	-0.49
456	CEDJ970101	Composition of amino acids in extracellular proteins (percent	AAindex	8.6	2.9	4.9	5.1	3.7	7.8	2.1	4.6	6.3	8.8	2.5	4.6	4.9	4	4.2	7.3	6	6.7	1.4	3.6
457	CEDJ970102	Composition of amino acids in anchored proteins (percent	AAindex	7.6	2.2	5.2	6.2	4	6.9	2.1	5.1	5.8	9.4	2.1	4.4	5.4	4.1	5	7.2	6.1	6.7	1.4	3.2
458	CEDJ970103	Composition of amino acids in membrane proteins (percent	AAindex	8.1	2	3.8	4.6	5.6	7	2	6.7	4.4	11	2.8	3.7	4.7	3.1	4.6	7.3	5.6	7.7	1.8	3.3
459	CEDJ970104	Composition of amino acids in intracellular proteins (percent	AAindex	7.9	1.9	5.5	7.1	3.9	7.1	2.1	5.2	6.7	8.6	2.4	4	5.3	4.4	4.9	6.6	5.3	6.8	1.2	3.1
460	CEDJ970105	Composition of amino acids in nuclear proteins (percent	AAindex	8.3	1.6	4.7	6.5	2.7	6.3	2.1	3.7	7.9	7.4	2.3	3.7	6.9	4.7	8.7	8.8	5.1	5.3	0.7	2.4
461	FUKS010101	Surface composition of amino acids in intracellular proteins of thermophiles (percent	AAindex	4.47	0.29	7.05	16.56	2.32	8.29	1.74	3.3	12.98	5.06	1.71	3.89	5.41	2.87	8.48	4.27	3.83	4.05	0.67	2.75
462	FUKS010102	Surface composition of amino acids in intracellular proteins of mesophiles (percent	AAindex	6.77	0.31	8.57	12.93	1.92	7.95	2.8	2.72	10.2	4.43	1.87	5.5	4.79	5.24	6.87	5.41	5.36	3.57	0.54	2.26
463	FUKS010103	Surface composition of amino acids in extracellular proteins of mesophiles (percent	AAindex	7.43	0.42	8.71	5.86	1.18	9.4	1.49	1.76	9.67	2.74	0.6	9.12	5.6	5.42	4.51	9.6	8.95	3.1	1.18	3.26
464	FUKS010104	Surface composition of amino acids in nuclear proteins (percent	AAindex	5.22	1.01	7.91	10.66	1.68	5.81	2.27	2.36	12.68	4.52	1.85	6.06	5.7	6	7.3	6.99	5.16	4.1	0.56	2.16
465	FUKS010105	Interior composition of amino acids in intracellular proteins of thermophiles (percen	AAindex	9.88	1.12	3.5	4.02	5.27	6.88	1.88	10.08	3.39	13.21	2.44	2.35	3.8	1.66	3.71	4.1	4.98	12.53	1.11	4.07
466	FUKS010106	Interior composition of amino acids in intracellular proteins of mesophiles (percen	AAindex	10.98	1.47	3.37	3.51	4.97	7.48	2.2	9.74	2.54	12.79	3.1	2.85	3.42	2.3	3.26	4.93	5.55	10.69	1.28	3.55
467	FUKS010107	Interior composition of amino acids in extracellular proteins of mesophiles (percent	AAindex	9.95	1.3	4.46	2.58	5.41	8.87	1.99	7.73	2	9.66	2.45	4.84	3.2	2.64	3.05	6.03	5.62	9.46	2.6	6.15
468	FUKS010108	Interior composition of amino acids in nuclear proteins (percent	AAindex	8.26	2.67	2.8	2.67	7.32	5.62	1.98	8.95	1.89	16.46	2.67	2.54	3.3	2.86	2.8	6	5	10.24	2.01	3.96
469	FUKS010109	Entire chain composition of amino acids in intracellular proteins of thermophiles (percen	AAindex	7.39	0.74	5.14	9.8	3.91	7.53	1.82	6.96	7.81	9.45	2.1	3.06	4.54	2.22	5.91	4.18	4.45	8.62	0.9	3.46
470	FUKS010110	Entire chain composition of amino acids in intracellular proteins of mesophiles (percen	AAindex	9.07	0.95	5.73	7.77	3.59	7.69	2.47	6.56	6.01	9	2.54	4.05	4.04	3.63	4.9	5.15	5.46	7.47	0.95	2.96
471	FUKS010111	Entire chain composition of amino acids in extracellular proteins of mesophiles (percen	AAindex	8.82	0.9	6.38	4.05	3.51	9.11	1.77	5.05	5.45	6.54	1.62	6.77	4.28	3.89	3.71	7.64	7.12	6.6	1.96	4.85
472	FUKS010112	Entire chain compositino of amino acids in nuclear proteins (percent	AAindex	6.65	1.79	5.5	6.89	4.34	5.72	2.13	5.47	7.59	10.15	2.24	4.4	4.56	4.52	5.17	6.52	5.08	7	1.24	3.01
473	AVBF000101	Screening coefficients gamma, loca	AAindex	0.163	0.316	0.212	0.212	0.41	0.08	0.315	0.474	0.255	0.315	0.356	0.124	0.2958947	0.274	0.22	0.29	0.412	0.515	0.325	0.354
474	AVBF000102	Screening coefficients gamma, non-loca	AAindex	0.236	0.259	0.168	0.306	0.328	-0.17	0.256	0.391	0.231	0.293	0.367	0.189	0.2508947	0.314	0.233	0.202	0.308	0.436	0.197	0.223
475	AVBF000103	Slopes tripeptide, FDPB VFF neutra	AAindex	-0.429	-0.422	-0.352	-0.647	-0.3700556	-0.357	-0.268	-0.45	-0.375	-0.409	-0.309	-0.375	-0.426	-0.382	-0.387	-0.24	-0.325	-0.3700556	-0.288	-0.22
476	AVBF000104	Slopes tripeptides, LD VFF neutra	AAindex	-0.727	-0.728	-0.666	-0.822	-0.7003333	-0.685	-0.617	-0.798	-0.717	-0.715	-0.649	-0.737	-0.679	-0.773	-0.741	-0.629	-0.669	-0.7003333	-0.655	-0.599
477	AVBF000105	Slopes tripeptide, FDPB VFF noside	AAindex	-0.317	-0.291	-0.222	-0.57	-0.2485556	-0.244	-0.144	-0.281	-0.274	-0.294	-0.189	-0.247	-0.28	-0.26	-0.268	-0.152	-0.206	-0.2485556	-0.155	-0.08
478	AVBF000106	Slopes tripeptide FDPB VFF all	AAindex	-0.369	-0.29	-0.206	-0.56	-0.2326667	-0.295	-0.134	-0.266	-0.26	-0.335	-0.187	-0.113	-0.251	-0.165	-0.245	-0.093	-0.188	-0.2326667	-0.147	-0.084
479	AVBF000107	Slopes tripeptide FDPB PARSE neutral	AAindex	-0.535	-0.492	-0.408	-0.86	-0.4807222	-0.519	-0.361	-0.462	-0.518	-0.508	-0.454	-0.545	-0.278	-0.532	-0.597	-0.367	-0.455	-0.4807222	-0.439	-0.323
480	AVBF000108	Slopes dekapeptide, FDPB VFF neutral	AAindex	-0.567	-0.559	-0.571	-0.679	-0.4873333	-0.508	-0.199	-0.527	-0.571	-0.581	-0.461	-0.626	-0.458	-0.572	-0.619	-0.233	-0.327	-0.4873333	-0.451	-0.263
481	AVBF000109	Slopes proteins, FDPB VFF neutral	AAindex	-0.28	-0.362	-0.441	-0.392	-0.3175556	-0.345	-0.194	-0.317	-0.312	-0.412	-0.237	-0.405	-0.374	-0.362	-0.403	-0.243	-0.111	-0.3175556	-0.171	-0.355
482	YANJ020101	Side-chain conformation by gaussian evolutionary methoc	AAindex	0.7922222	0.83	0.66	0.73	0.92	0.7922222	0.92	0.88	0.77	0.89	0.77	0.76	0.94	0.59	0.62	0.58	0.73	0.88	0.86	0.93
483	MITS020101	Amphiphilicity index	AAindex	0	0	0	1.27	0	0	1.45	0	3.67	0	0	0	0	1.25	2.45	0	0	0	6.93	5.06
484	TSAJ990101	Volumes including the crystallographic waters using the ProtO	AAindex	89.3	102.5	114.4	138.8	190.8	63.8	157.5	163	165.1	163.1	165.8	122.4	121.6	146.9	190.3	94.2	119.6	138.2	226.4	194.6
485	TSAJ990102	Volumes not including the crystallographic waters using the ProtO	AAindex	90	103.3	117.3	142.2	191.9	64.9	160	163.9	167.3	164	167	124.7	122.9	149.4	194	95.4	121.5	139	228.2	197
486	COSI940101	Electron-ion interaction potential values	AAindex	0.0959	0.0761	0.0829	0.005	0.0198	0.0242	0	0	0.0823	0.0371	0.0946	0.1263	0.0829	0.0058	0.0036	0.0941	0.0548	0.0496158	0.0516	0.0057
487	PONP930101	Hydrophobicity scales	AAindex	0.85	2.1	-1.1	-0.79	1.69	0	0.22	3.14	-1.19	1.99	1.42	-0.48	-1.14	-0.42	0.2	-0.52	-0.08	2.53	1.76	1.37
488	WILM950101	Hydrophobicity coefficient in RP-HPLC, C18 with 0.1%TFA/MeCN/H2O	AAindex	0.06	0.49	-0.2	-0.1	4.8	0.21	-2.24	3.48	-1.62	3.5	0.21	0.25	0.71	0.31	-0.85	-0.62	0.65	1.59	2.29	1.89
489	WILM950102	Hydrophobicity coefficient in RP-HPLC, C8 with 0.1%TFA/MeCN/H2O	AAindex	2.62	0.73	-2.84	-0.45	9.14	-1.15	-0.74	4.38	-2.78	6.57	-3.12	-1.27	-0.12	-1.69	1.26	-1.39	1.81	2.3	5.91	1.39
490	WILM950103	Hydrophobicity coefficient in RP-HPLC, C4 with 0.1%TFA/MeCN/H2O	AAindex	-1.64	9.3	0.7	1.18	-1.36	-1.85	7.17	3.02	-2.36	0.83	4.26	0.83	3.12	-0.04	-3.28	1.59	2.31	0.52	2.61	2.37
491	WILM950104	Hydrophobicity coefficient in RP-HPLC, C18 with 0.1%TFA/2-PrOH/MeCN/H2O	AAindex	-2.34	5.03	-0.48	1.3	2.57	-1.06	-3	7.26	1.56	1.09	0.62	2.81	-0.15	0.16	1.6	1.93	0.19	2.06	3.59	-2.58
492	KUHL950101	Hydrophilicity scale	AAindex	0.78	0.55	1.35	1.45	0.47	0.68	0.99	0.47	1.1	0.56	0.66	1.2	0.69	1.19	1.58	1	1.05	0.51	0.7	1
493	GUOD860101	Retention coefficient at pH 2	AAindex	25	32	2	14	100	-2	-26	91	-26	100	68	-7	25	0	-7	-2	7	62	109	56
494	JURD980101	Modified Kyte-Doolittle hydrophobicity scale	AAindex	1.1	2.5	-3.6	-3.2	2.8	-0.64	-3.2	4.5	-4.11	3.8	1.9	-3.5	-1.9	-3.68	-5.1	-0.5	-0.7	4.2	-0.46	-1.3
495	BASU050101	Interactivity scale obtained from the contact matrix	AAindex	0.0363	0.0325	0.2745	-0.0464	0.0019	0.0549	0.4172	0.4251	0.1747	-0.0101	0.4076	-0.1233	-0.0433	-0.0484	-0.0345	0.0589	0.2362	0.1336263	0.3167	0.4084
496	BASU050102	Interactivity scale obtained by maximizing the mean of correlation coefficient over single-domain globular proteins	AAindex	0.0394	0.0126	0.3557	-0.0589	-0.0492	0.0874	0.3805	0.3819	0.1613	-0.0053	0.4201	-0.0552	-0.0282	-0.0295	-0.039	0.0239	0.4114	0.1376263	0.3113	0.2947
497	BASU050103	Interactivity scale obtained by maximizing the mean of correlation coefficient over pairs of sequence sharing the TIM barrel fold	AAindex	-0.0103	0.0246	0.3222	0.0248	0.0844	0.1335	0.4238	0.3926	0.216	-0.0158	0.3455	0.0047	0.004	-0.0639	0.0381	0.1462	0.2657	0.1597684	0.2998	0.3997
498	SUYM030101	Linker propensity index	AAindex	0	-0.073	0.447	0.331	-0.478	0.195	0.06	0.138	0.275	-0.112	0.24	0.016	-0.177	-0.128	0.027	-0.163	0.564	0.0753684	0.322	-0.052
499	PUNT030101	Knowledge-based membrane-propensity scale from 1D_Helix in MPtopo databases	AAindex	-0.17	-0.06	0.37	0.15	-0.41	0.01	-0.02	-0.28	0.32	-0.28	-0.26	0.18	0.13	0.26	0.37	0.05	0.02	-0.17	-0.15	-0.09
500	PUNT030102	Knowledge-based membrane-propensity scale from 3D_Helix in MPtopo databases	AAindex	-0.15	-0.15	0.41	0.3	-0.22	0.08	0.06	-0.29	0.24	-0.36	-0.19	0.22	0.15	0.03	0.32	0.16	-0.08	-0.24	-0.28	-0.03
501	GEOR030101	Linker propensity from all dataset	AAindex	0.964	0.778	0.916	1.051	1.119	0.835	1.014	0.922	0.944	1.085	1.032	0.944	1.299	1.047	1.143	0.947	1.017	0.955	0.895	1
502	GEOR030102	Linker propensity from 1-linker dataset	AAindex	0.974	0.972	0.892	1.054	1.122	0.845	0.949	0.928	0.946	1.11	0.923	0.988	1.362	1.092	1.129	0.932	1.023	0.923	0.879	0.902
503	GEOR030103	Linker propensity from 2-linker dataset	AAindex	0.938	0.6856	0.857	1.139	1.11	0.892	1.109	0.986	0.952	1	1.077	0.902	1.266	0.916	1.137	0.956	1.018	0.959	0.971	1.157
504	GEOR030104	Linker propensity from 3-linker dataset	AAindex	1.042	0.5	0.97	0.992	0.981	0.743	1.034	0.852	0.979	1.193	0.998	0.828	1.332	1.111	1.069	0.984	0.992	1.001	0.96	1.12
505	GEOR030105	Linker propensity from small dataset (linker length is less than six residues	AAindex	1.065	1.015	0.836	0.736	1.368	1.022	0.973	1.189	0.478	1.192	1.369	0.762	1.241	0.861	1.131	1.097	0.822	1.14	1.017	0.836
506	GEOR030106	Linker propensity from medium dataset (linker length is between six and 14 residues	AAindex	0.99	0.644	0.915	1.053	1.121	0.785	1.054	0.95	1.003	1.106	1.093	0.873	1.314	0.999	1.132	0.911	0.988	0.957	0.939	1.09
507	GEOR030107	Linker propensity from long dataset (linker length is greater than 14 residues	AAindex	0.892	1.035	0.925	1.115	1.058	0.917	0.992	0.817	0.944	0.994	0.782	1.144	1.309	1.2	1.154	0.986	1.11	0.9	0.841	0.866
508	GEOR030108	Linker propensity from helical (annotated by DSSP) dataset	AAindex	1.092	0.662	0.919	1.199	1.09	0.698	1.012	0.912	1.008	1.276	1.171	0.927	0.8	1.124	1.239	0.886	0.832	0.908	0.981	1.075
509	GEOR030109	Linker propensity from non-helical (annotated by DSSP) dataset	AAindex	0.843	0.896	0.906	0.9	1.151	0.978	1.05	0.946	0.893	0.885	0.878	0.956	1.816	0.968	1.038	1.003	1.189	0.999	0.852	0.945
510	ZHOH040101	The stability scale from the knowledge-based atom-atom potential	AAindex	2.18	3.89	1.75	1.89	5.88	1.17	2.51	4.5	2.12	4.71	3.63	1.85	2.09	2.16	2.71	1.66	2.18	3.77	6.46	5.01
511	ZHOH040102	The relative stability scale extracted from mutation experiments	AAindex	1.79	2.22	2.33	2.52	4.84	0.7	3.06	4.59	2.5	4.72	3.91	2.83	2.45	2.37	3.2	1.82	2.45	3.67	5.64	4.46
512	ZHOH040103	Buriability	AAindex	13.4	22.6	8.2	7.3	23.9	7	11.3	20.3	6.1	20.8	15.7	7.6	9.9	8.5	8.5	8.2	10.3	19.5	24.5	19.5
513	BAEK050101	Linker index	AAindex	-0.0762	-0.1051	0.5724	-0.0442	-0.4188	0.1643	0.2758	0.2523	0.0197	-0.2134	0.3561	-0.1278	-0.1629	-0.1794	-0.0786	-0.0701	0.3836	0.0513632	0.25	0.1782
514	HARY940101	Mean volumes of residues buried in protein interiors	AAindex	90.1	113.2	117.1	140.8	193.5	63.8	159.3	164.9	170	164.6	167.7	127.5	123.1	149.4	192.8	94.2	120	139.1	197.1	231.7
515	PONJ960101	Average volumes of residues	AAindex	91.5	114.4	135.2	154.6	198.8	67.5	163.2	162.6	162.5	163.4	165.9	138.3	123.4	156.4	196.1	102	126	138.4	209.8	237.2
516	DIGM050101	Hydrostatic pressure asymmetry index, PA	AAindex	1.076	0.753	1.29	1.118	0.869	1.346	0.985	0.926	1.105	1.054	0.974	1.056	0.82	0.729	1.361	1.342	0.871	1.131	0.666	0.531
517	WOLR790101	Hydrophobicity index	AAindex	1.12	0.59	-0.83	-0.92	0.67	1.2	-0.93	1.16	-0.8	1.18	0.55	-0.83	0.54	-0.78	-2.55	-0.05	-0.02	1.13	-0.19	-0.23
518	OLSK800101	Average internal preferences	AAindex	1.38	1.43	0.52	0.71	1.72	1.34	0.66	2.32	0.15	1.47	1.78	0.37	0.85	0.22	0	0.86	0.89	1.99	0.82	0.47
519	KIDA850101	Hydrophobicity-related index	AAindex	-0.27	-1.05	0.81	1.17	-1.43	-0.16	0.28	-0.77	1.7	-1.1	-0.73	0.81	-0.75	1.1	1.87	0.42	0.63	-0.4	-1.57	-0.56
520	GUYH850102	Apparent partition energies calculated from Wertz-Scheraga index	AAindex	0.05	-0.84	0.41	0.38	-0.45	0.31	-0.41	-0.69	0.57	-0.62	-0.38	0.29	0.46	0.46	0.12	0.12	0.38	-0.46	-0.98	-0.25
521	GUYH850103	Apparent partition energies calculated from Robson-Osguthorpe index	AAindex	0.54	-1.13	0.65	0.38	-1.51	-0.4157895	-0.59	-2.15	0.48	-1.08	-0.97	0.38	-0.22	0.05	-0.16	0.65	0.27	-0.75	-1.61	-1.13
522	GUYH850104	Apparent partition energies calculated from Janin index	AAindex	-0.31	-0.87	0.58	0.68	-0.45	-0.33	0.13	-0.66	1.79	-0.53	-0.38	0.49	0.34	0.7	1.3	0.1	0.21	-0.62	-0.27	0.4
523	GUYH850105	Apparent partition energies calculated from Chothia index	AAindex	-0.27	-0.23	0.5	0.33	-0.55	-0.22	0.37	-0.8	1.17	-0.44	-0.31	0.61	0.36	1	2	0.17	0.18	-0.65	0.05	0.48
524	ROSM880104	Hydropathies of amino acid side chains, neutral form	AAindex	0.39	0.25	-0.71	-0.18	2.27	0	-0.6	1.82	0.32	1.82	0.96	-1.91	0.3216667	-1.3	0.3216667	-1.24	-1	1.3	2.13	1.47
525	ROSM880105	Hydropathies of amino acid side chains, pi-values in pH 7.0	AAindex	0.39	0.25	-3.81	-2.91	2.27	0	-0.64	1.82	-2.77	1.82	0.96	-1.91	-0.3747368	-1.3	-3.95	-1.24	-1	1.3	2.13	1.47
526	JACR890101	Weights from the IFH scale	AAindex	0.18	0.27	-2.36	-2.1	0.5	0.09	-1.48	0.37	-2.53	0.41	0.44	-1.3	-0.2	-1.22	-5.4	-0.4	-0.34	0.32	-0.01	-0.08
527	COWR900101	Hydrophobicity index, 3.0 pH	AAindex	0.42	0.84	-0.51	-0.37	1.74	0	-2.28	1.81	-2.03	1.8	1.18	-1.03	0.86	-0.96	-1.56	-0.64	-0.26	1.34	1.46	0.51
528	BLAS910101	Scaled side chain hydrophobicity values	AAindex	0.616	0.68	0.028	0.043	1	0.501	0.165	0.943	0.283	0.943	0.738	0.236	0.711	0.251	0	0.359	0.45	0.825	0.878	0.88
529	CASG920101	Hydrophobicity scale from native protein structures	AAindex	0.2	1.9	-1.4	-1.3	1	-0.1	0.4	1.4	-1.6	0.5	0.5	-0.5	-1	-1.1	-0.7	-0.7	-0.4	0.7	1.6	0.5
530	CORJ870101	NNEIG index	AAindex	50.76	58.74	43.17	43.48	53.45	50.27	49.33	57.3	42.92	53.89	52.75	45.8	45.39	46.09	48.66	47.24	49.26	56.12	53.59	51.79
531	CORJ870102	SWEIG index	AAindex	-0.584	-0.905	0.162	-0.684	-0.503	-0.63	1.237	1.215	1.02	-0.67	1.938	-1.31	-0.563	-1.218	-0.916	-0.289	0.514	0.0216842	1.699	0.899
532	CORJ870103	PRIFT index	AAindex	-0.96	4.54	-5.68	-3.86	5.06	-1.28	-0.62	5.54	-5.62	6.81	4.76	-1.94	-4.47	-5.3	0.75	-1.92	-3.99	5.39	0.21	3.34
533	CORJ870104	PRILS index	AAindex	-0.26	0.83	-1.3	-0.73	1.09	-0.4	-0.18	1.1	-1.01	1.52	1.09	-0.46	-0.62	-0.83	0.08	-0.55	-0.71	1.15	-0.13	0.69
534	CORJ870105	ALTFT index	AAindex	-0.73	0.64	-6.13	-2.9	5.2	-2.67	3.03	5.04	-5.99	4.91	3.34	-5.29	-4.32	-0.96	-1.03	-3	-1.91	3.98	0.51	2.87
535	CORJ870106	ALTLS index	AAindex	-1.35	4.37	-11.88	-4.56	11.35	-5.82	6.54	10.93	-11.92	9.88	7.47	-10.96	-10.86	-1.34	-3.89	-6.21	-4.83	8.2	1.8	7.61
536	CORJ870107	TOTFT index	AAindex	-0.56	1.78	-4.31	-2.35	3.67	-1.35	0.81	3.83	-4.08	4.09	3.11	-2.87	-3.22	-2.31	-0.26	-1.85	-1.97	3.31	-0.11	2.17
537	CORJ870108	TOTLS index	AAindex	1.37	-4.47	8.93	4.04	-7.96	3.39	-1.65	-7.92	7.7	-8.68	-7.13	6.29	6.25	3.88	1.33	4.08	4.02	-6.94	0.79	-4.73
538	MIYS990101	Relative partition energies derived by the Bethe approximation	AAindex	-0.02	-0.96	0.72	0.74	-2.22	0.38	0	-1.89	1.01	-2.29	-1.36	0.63	0.47	0.56	0.44	0.55	0.25	-1.34	-1.28	-0.88
539	MIYS990102	Optimized relative partition energies - method A	AAindex	0	-0.16	0.12	0.12	-0.36	0.06	0	-0.31	0.17	-0.37	-0.22	0.1	0.08	0.09	0.07	0.09	0.04	-0.22	-0.21	-0.14
540	MIYS990103	Optimized relative partition energies - method B	AAindex	-0.03	-0.36	0.17	0.23	-0.34	0.09	-0.04	-0.33	0.32	-0.38	-0.3	0.13	0.2	0.13	0.09	0.1	0.01	-0.29	-0.24	-0.23
541	MIYS990104	Optimized relative partition energies - method C	AAindex	-0.04	-0.38	0.19	0.23	-0.38	0.09	-0.04	-0.34	0.33	-0.37	-0.3	0.13	0.19	0.14	0.07	0.12	0.03	-0.29	-0.33	-0.29
542	MIYS990105	Optimized relative partition energies - method D	AAindex	-0.02	-0.32	0.19	0.21	-0.33	-0.02	-0.02	-0.28	0.3	-0.32	-0.25	0.1	0.11	0.15	0.08	0.11	0.05	-0.23	-0.27	-0.23
543	ENGD860101	Hydrophobicity index	AAindex	-1.6	-2	9.2	8.2	-3.7	-1	3	-3.1	8.8	-2.8	-3.4	4.8	0.2	4.1	12.3	-0.6	-1.2	-2.6	-1.9	0.7
544	FASG890101	Hydrophobicity index	AAindex	-0.21	-6.04	1.36	2.3	-4.65	0	-1.23	-4.81	3.88	-4.68	-3.66	0.96	0.75	1.52	2.11	1.74	0.78	-3.5	-3.32	-1.01
545	P87cut483AA-P10	AA composition of proteolyzed 483 peptides in P87mix, P10 position	this study	1.817	2.886	1.253	2.456	1.360	0.939	1.371	1.220	3.232	1.955	0.985	1.037	3.217	3.069	2.886	2.093	1.523	3.312	2.331	1.600
546	P87cut483AA-P9	AA composition of proteolyzed 483 peptides in P87mix, P9 position	this study	2.384	0.887	1.470	2.354	1.532	1.023	1.264	1.125	3.299	1.183	2.498	0.820	2.135	4.243	2.353	2.768	2.207	2.806	2.149	2.397
547	P87cut483AA-P8	AA composition of proteolyzed 483 peptides in P87mix, P8 position	this study	2.584	0.867	1.130	2.126	1.226	1.231	1.731	0.917	3.851	1.157	1.333	1.202	3.249	3.874	4.203	2.872	2.158	2.018	1.681	2.525

Table S11 Amino acid descriptors (1D) used in this study.

No.	Descriptor ID	Attribute	Ref*	A	C	D	E	F	G	H	I	K	L	M	N	P	Q	R	S	T	V	W	Y
548	P87cut483AA-P7	AA composition of proteolyzed 483 peptides in P87mix, P7 positior	this study	2.185	0.418	0.989	2.388	1.706	1.408	3.096	0.971	3.910	1.273	1.284	1.029	2.458	2.532	3.374	4.109	2.646	2.332	1.619	1.390
549	P87cut483AA-P6	AA composition of proteolyzed 483 peptides in P87mix, P6 positior	this study	2.484	0.814	1.253	1.829	2.174	1.156	2.320	1.119	3.712	1.292	0.834	1.505	2.504	3.116	3.099	3.619	2.578	2.272	1.578	2.708
550	P87cut483AA-P5	AA composition of proteolyzed 483 peptides in P87mix, P5 positior	this study	2.984	0.395	0.842	0.806	1.861	1.191	1.801	1.252	2.748	2.307	0.809	1.825	2.641	2.771	3.189	3.660	2.054	3.160	1.914	1.314
551	P87cut483AA-P4	AA composition of proteolyzed 483 peptides in P87mix, P4 positior	this study	2.318	0.778	1.198	1.748	0.978	1.726	2.218	0.905	2.801	1.680	2.192	2.277	5.100	3.847	2.873	3.165	2.200	1.593	2.262	1.132
552	P87cut483AA-P3	AA composition of proteolyzed 483 peptides in P87mix, P3 positior	this study	2.241	1.128	0.802	2.228	0.946	2.003	1.501	0.795	3.430	2.102	1.734	1.738	4.226	2.880	3.038	2.633	1.957	2.520	2.552	1.721
553	P87cut483AA-P2	AA composition of proteolyzed 483 peptides in P87mix, P2 positior	this study	1.469	0.000	0.887	0.917	1.059	1.063	1.067	1.583	2.336	4.470	1.726	2.076	1.803	3.583	1.469	3.116	3.811	3.762	1.814	1.712
554	P87cut483AA-P1	AA composition of proteolyzed 483 peptides in P87mix, P1 positior	this study	1.671	1.113	1.054	1.590	1.981	2.304	3.595	0.392	4.984	1.225	3.609	2.628	0.893	4.141	3.937	3.157	2.936	0.828	4.313	3.701
555	P87cut483AA-P1p	AA composition of proteolyzed 483 peptides in P87mix, P1' positior	this study	3.342	0.742	0.703	1.363	0.816	2.106	0.846	0.784	3.115	1.978	1.900	2.171	1.984	3.194	3.851	4.631	2.433	2.140	1.797	1.696
556	P87cut483AA-P2p	AA composition of proteolyzed 483 peptides in P87mix, P2' positior	this study	2.816	0.000	0.794	2.359	1.054	2.116	1.912	1.103	2.683	1.656	1.718	1.837	4.386	3.090	2.494	3.525	2.191	2.566	1.083	1.549
557	P87cut483AA-P3p	AA composition of proteolyzed 483 peptides in P87mix, P3' positior	this study	2.229	1.150	0.908	1.096	1.205	1.633	1.093	0.811	4.233	1.266	1.767	2.599	6.975	3.669	3.539	2.611	1.474	1.570	1.858	2.073
558	P87cut483AA-P4p	AA composition of proteolyzed 483 peptides in P87mix, P4' positior	this study	2.505	0.401	1.234	1.965	1.008	2.845	1.600	1.017	2.020	1.324	0.821	2.470	5.576	2.813	2.775	2.351	2.630	2.014	1.554	2.167
559	P87cut483AA-P5p	AA composition of proteolyzed 483 peptides in P87mix, P5' positior	this study	2.523	0.413	1.566	2.954	0.390	2.788	1.885	0.175	2.976	1.050	1.905	2.802	4.202	2.373	2.194	2.815	2.525	2.616	0.801	1.203
560	P87cut483AA-P6p	AA composition of proteolyzed 483 peptides in P87mix, P6' positior	this study	2.257	0.000	0.919	1.409	1.220	2.526	1.476	1.094	3.830	1.479	0.663	1.993	3.000	2.614	3.583	2.203	1.659	3.291	0.836	2.332
561	P87cut483AA-P7p	AA composition of proteolyzed 483 peptides in P87mix, P7' positior	this study	1.922	0.000	1.080	1.863	0.860	2.752	2.080	0.964	3.722	1.564	1.635	2.529	2.684	2.619	3.368	3.020	1.857	1.443	0.442	1.138
562	P87cut483AA-P8p	AA composition of proteolyzed 483 peptides in P87mix, P8' positior	this study	1.752	1.507	0.714	2.462	1.263	1.961	2.005	0.850	2.291	1.340	1.544	3.250	3.091	2.564	3.246	2.946	1.704	1.683	1.947	1.462
563	P87cut483AA-P9p	AA composition of proteolyzed 483 peptides in P87mix, P9' positior	this study	2.045	1.126	1.066	1.839	0.707	1.798	1.925	0.595	2.972	1.716	0.577	2.254	3.764	1.975	2.598	2.236	1.655	2.200	1.091	1.638
564	P87cut483AA-P10p	AA composition of proteolyzed 483 peptides in P87mix, P10' positior	this study	1.782	0.645	1.070	1.581	0.203	1.718	1.472	0.818	2.168	0.492	0.330	3.180	4.315	2.264	2.382	2.808	2.189	2.282	0.625	1.073
565	A_Ala	A, Ala	this study	1	0	0	0	0	0	0	0	0	0	0	0	0	0	0	0	0	0	0	0
566	B_Asx	B, Asx	this study	0	0	0	0	0	0	0	0	0	0	0	0	0	0	0	0	0	0	0	0
567	C_Cys	C, Cys	this study	0	1	0	0	0	0	0	0	0	0	0	0	0	0	0	0	0	0	0	0
568	D_Asp	D, Asp	this study	0	0	1	0	0	0	0	0	0	0	0	0	0	0	0	0	0	0	0	0
569	E_Glu	E, Glu	this study	0	0	0	1	0	0	0	0	0	0	0	0	0	0	0	0	0	0	0	0
570	F_Phe	F, Phe	this study	0	0	0	0	1	0	0	0	0	0	0	0	0	0	0	0	0	0	0	0
571	G_Gly	G, Gly	this study	0	0	0	0	0	1	0	0	0	0	0	0	0	0	0	0	0	0	0	0
572	H_His	H, His	this study	0	0	0	0	0	0	1	0	0	0	0	0	0	0	0	0	0	0	0	0
573	I_Ile	I, Ile	this study	0	0	0	0	0	0	0	1	0	0	0	0	0	0	0	0	0	0	0	0
574	J_Glx	J, Glx	this study	0	0	0	0	0	0	0	0	0	0	0	0	0	0	0	0	0	0	0	0
575	K_Lys	K, Lys	this study	0	0	0	0	0	0	0	0	1	0	0	0	0	0	0	0	0	0	0	0
576	L_Leu	L, Leu	this study	0	0	0	0	0	0	0	0	0	1	0	0	0	0	0	0	0	0	0	0
577	M_Met	M, Met	this study	0	0	0	0	0	0	0	0	0	0	1	0	0	0	0	0	0	0	0	0
578	N_Asn	N, Asn	this study	0	0	0	0	0	0	0	0	0	0	0	1	0	0	0	0	0	0	0	0
579	O_Orn	O, Orn	this study	0	0	0	0	0	0	0	0	0	0	0	0	0	0	0	0	0	0	0	0
580	P_Pro	P, Pro	this study	0	0	0	0	0	0	0	0	0	0	0	0	1	0	0	0	0	0	0	0
581	Q_Gln	Q, Gln	this study	0	0	0	0	0	0	0	0	0	0	0	0	0	1	0	0	0	0	0	0
582	R_Arg	R, Arg	this study	0	0	0	0	0	0	0	0	0	0	0	0	0	0	1	0	0	0	0	0
583	S_Ser	S, Ser	this study	0	0	0	0	0	0	0	0	0	0	0	0	0	0	0	1	0	0	0	0
584	T_Thr	T, Thr	this study	0	0	0	0	0	0	0	0	0	0	0	0	0	0	0	0	1	0	0	0
585	U_Sec	U, Sec	this study	0	0	0	0	0	0	0	0	0	0	0	0	0	0	0	0	0	0	0	0
586	V_Val	V, Val	this study	0	0	0	0	0	0	0	0	0	0	0	0	0	0	0	0	0	1	0	0
587	W_Trp	W, Trp	this study	0	0	0	0	0	0	0	0	0	0	0	0	0	0	0	0	0	0	1	0
588	X_Xaa	X, Xaa	this study	0	0	0	0	0	0	0	0	0	0	0	0	0	0	0	0	0	0	0	0
589	Y_Tyr	Y, Tyr	this study	0	0	0	0	0	0	0	0	0	0	0	0	0	0	0	0	0	0	0	1
601	SS_alpha	Probability of α-helix secondary structure (predicted by Jpred 3 web site, values: 0-9	Jpred 3	-	-	-	-	-	-	-	-	-	-	-	-	-	-	-	-	-	-	-	-
602	SS_beta	Probability of β-strand secondary structure (predicted by Jpred 3 web site, values: 0-9	Jpred 3	-	-	-	-	-	-	-	-	-	-	-	-	-	-	-	-	-	-	-	-
603	SS_randomC	Probability of secondary structure other than α-helix or β-strand (random coil) (predicted by Jpred 3 we site, values: 0-9)	Jpred 3	-	-	-	-	-	-	-	-	-	-	-	-	-	-	-	-	-	-	-	-
701	a_acc	Number of hydrogen bond acceptor atoms (not counting acidic atoms but counting atoms that are bot hydrogen bond donors and acceptors such as -OH)	MOE	0	0	0	0	0	0	2	0	0	0	0	1	0	1	0	1	1	0	0	1
702	a_aro	Number of aromatic atoms.	MOE	0	0	0	0	6	0	5	0	0	0	0	0	0	0	0	0	0	0	9	6
703	a_base	Number of basic atoms.	MOE	1	1	1	1	1	1	1	1	2	1	1	1	1	1	4	1	1	1	1	1
704	a_count	Number of atoms (including implicit hydrogens). This is calculated as the sum of (1 + hi) over all nor trivial atoms i.	MOE	13	14	15	18	23	10	20	22	25	22	20	17	17	20	27	14	17	19	27	24
705	a_don	Number of hydrogen bond donor atoms (not counting basic atoms but counting atoms that are bot hydrogen bond donors and acceptors such as -OH)	MOE	0	0	0	0	0	0	2	0	0	0	0	1	0	1	0	1	1	0	1	1
706	a_donacc	Number of hydrogen bond donor and hydrogen bond acceptor atoms	MOE	0	0	0	0	0	0	4	0	0	0	0	2	0	2	0	2	2	0	1	2
707	a_hyd	Number of hydrophobic atoms	MOE	2	2	2	3	8	1	4	5	5	5	5	2	4	3	4	2	3	4	10	8
708	a_ICM	Atom information content (mean). This is the entropy of the element distribution in the molecule (includir implicit hydrogens but not lone pair pseudo-atoms). Let ni be the number of occurrences of atomic number i in the molecule. Let pi = ni / n where n is the sum of the ni. The value of a_ICM is the negative of the sum over all i of pi log pi	MOE	1.6691804	1.9211855	1.8062389	1.7471672	1.5416893	1.760964	1.7822287	1.4769093	1.5193309	1.4769093	1.7387587	1.8077636	1.6087002	1.7427377	1.6395862	1.7244078	1.6589798	1.5288018	1.6040246	1.6125475
709	a_nC	Number of carbon atoms: #{Zi | Zi = 6}	MOE	3	3	4	5	9	2	6	6	6	6	5	4	5	5	6	3	4	5	11	9
710	a_nH	Number of hydrogen atoms (including implicit hydrogens). This is calculated as the sum of hi over all nor trivial atoms i plus the number of non-trivial hydrogen atoms	MOE	7	7	6	8	11	5	9	13	15	13	11	8	9	10	15	7	9	11	12	11
711	a_nN	Number of nitrogen atoms: #{Zi | Zi = 7}	MOE	1	1	1	1	1	1	3	1	2	1	1	2	1	2	4	1	1	1	2	1
712	a_nO	Number of oxygen atoms: #{Zi | Zi = 8}	MOE	2	2	4	4	2	2	2	2	2	2	2	3	2	3	2	3	3	2	2	3
713	a_nS	Number of sulfur atoms: #{Zi | Zi = 16}	MOE	0	1	0	0	0	0	0	0	0	0	1	0	0	0	0	0	0	0	0	0
714	AM1_dipole	The dipole moment calculated using the AM1 Hamiltonian [MOPAC]	MOE	10.997499	9.4121666	5.1613965	5.8671317	10.313684	11.393995	8.7984514	10.737055	7.1613135	10.577208	9.1572008	7.2916455	11.541834	7.2533221	5.8157797	10.42135	10.383177	10.587674	12.230232	9.1027718
715	AM1_E	The total SCF energy (kcal/mol) calculated using the AM1 Hamiltonian [MOPAC]	MOE	-30620.158	-35105.422	-48113.332	-51706.34	-49597.172	-27026.307	-49633.316	-41396.84	-46677.414	-41397.199	-42286.574	-46069.809	-37160.07	-49655.559	-56220.305	-38016.148	-41609.152	-37803.703	-60575.051	-56990.852
716	AM1_HF	The heat of formation (kcal/mol) calculated using the AM1 Hamiltonian [MOPAC]	MOE	-66.231247	-60.562611	-219.33864	-225.55504	-41.775398	-59.169762	-17.370291	-82.540443	47.348808	-82.898499	-71.204567	-114.14158	-62.38081	-113.31319	65.744041	-113.21797	-119.43003	-71.649353	-10.15979	-86.451332
717	ASA-	Water accessible surface area of all atoms with negative partial charge (strictly less than 0)	MOE	64.357323	113.32629	129.96368	125.61393	134.61758	70.318779	90.004013	58.571175	65.059944	57.150478	92.996788	94.460403	61.311695	109.06306	55.948147	82.018669	77.59301	56.437557	160.4474	138.25377
718	ASA_P	Water accessible surface area of all polar (|qi|>=0.2) atoms.	MOE	147.33368	193.86926	210.97888	199.99446	122.80965	176.98442	185.65038	120.19822	215.43085	127.49862	206.93777	230.15117	126.81903	248.19911	250.09781	194.0195	165.78256	129.09962	175.36607	172.44815
719	ASA+	Water accessible surface area of all atoms with positive partial charge (strictly greater than 0)	MOE	82.976357	98.322243	88.303322	80.992683	166.34024	106.66565	184.19031	61.627045	150.3709	70.348137	113.94099	142.75401	65.507332	143.53644	194.14966	112.00083	88.189552	72.662071	200.2625	175.53059
720	ast_fraglike_ext	Astex Fragment-like Test (Extended)	MOE	1	1	0	0	1	1	1	1	0	1	0	0	1	0	0	1	1	1	1	1
721	ast_violation_ext	Astex Fragment-like Violation Count (Extended	MOE	0	0	1	2	0	0	0	0	2	0	1	1	0	2	2	0	0	0	0	0
722	b_1rotN	Number of rotatable single bonds. Conjugated single bonds are not included (e.g. ester and peptid bonds).	MOE	1	2	3	4	3	1	3	3	5	3	4	3	1	4	5	2	2	2	3	3
723	b_1rotR	Fraction of rotatable single bonds: b_1rotN divided by b_heavy	MOE	0.2	0.3333333	0.375	0.4444445	0.25	0.25	0.2727273	0.375	0.5555556	0.375	0.5	0.375	0.125	0.4444445	0.4545455	0.3333333	0.2857143	0.2857143	0.1875	0.2307692
724	b_ar	Number of aromatic bonds.	MOE	0	0	0	0	6	0	5	0	0	0	0	0	0	0	0	0	0	0	10	6
725	b_double	Number of double bonds. Aromatic bonds are not considered to be double bonds	MOE	1	1	2	2	1	1	1	1	1	1	1	2	1	2	2	1	1	1	1	1
726	b_heavy	Number of bonds between heavy atoms	MOE	5	6	8	9	12	4	11	8	9	8	8	8	8	9	11	6	7	7	16	13
727	b_max1len	Code Class Description Maximum single-bond chain lengtł	MOE	4	5	4	5	4	3	4	6	8	6	7	4	2	5	7	5	5	5	4	4
728	b_rotN	Number of rotatable bonds. A bond is rotatable if it has order 1, is not in a ring, and has at least tw heavy neighbors.	MOE	1	2	3	4	3	1	3	3	5	3	4	3	1	4	6	2	2	2	3	3
729	b_rotR	Fraction of rotatable bonds: b_rotN divided by b_heavy	MOE	0.2	0.3333333	0.375	0.4444445	0.25	0.25	0.2727273	0.375	0.5555556	0.375	0.5	0.375	0.125	0.4444445	0.5454546	0.3333333	0.2857143	0.2857143	0.1875	0.2307692
730	b_single	Number of single bonds (including implicit hydrogens). Aromatic bonds are not considered to be singl bonds.	MOE	11	12	12	15	16	8	14	20	23	20	18	14	16	17	24	12	15	17	17	17
731	balabanJ	Balaban's connectivity topological index [Balaban 1982]	MOE	3.077395	3.1946843	3.41747	3.3270686	2.2183652	2.6693285	2.2013693	3.5997987	3.1423776	3.3980889	3.1746612	3.41747	2.2622292	3.3270686	3.2079518	3.1946843	3.499558	3.499558	1.8900248	2.2403944
732	BCUT_PEOE_0	The BCUT descriptors [Pearlman 1998] are calculated from the eigenvalues of a modified adjacency matrix. Each ij entry of the adjacency matrix takes the value 1/sqrt(bij) where bij is the formal bond order between bonded atoms i and j. The diagonal takes the value of the PEOE partial charges. The resulting eigenvalues are sorted and the smallest, 1/3-ile, 2/3-ile and largest eigenvalues are reportec	MOE	-2.4964128	-2.5028403	-2.5129702	-2.5742788	-2.5253208	-2.3790979	-2.50686	-2.6729925	-2.6539295	-2.6676149	-2.5793788	-2.5052786	-2.498919	-2.5679984	-2.6179581	-2.4986613	-2.5661302	-2.6353483	-2.5161142	-2.5250249
733	BCUT_PEOE_1	The BCUT descriptors [Pearlman 1998] are calculated from the eigenvalues of a modified adjacency matrix. Each ij entry of the adjacency matrix takes the value 1/sqrt(bij) where bij is the formal bond order between bonded atoms i and j. The diagonal takes the value of the PEOE partial charges. The resulting eigenvalues are sorted and the smallest, 1/3-ile, 2/3-ile and largest eigenvalues are reportec	MOE	-0.5439147	-0.5480231	-0.5604393	-0.5580304	-0.5842268	-0.5441136	-0.5781555	-0.4644188	-0.5439084	-0.5413597	-0.3868223	-0.5398188	-0.5341087	-0.5518995	-0.5532467	-0.5429108	-0.4984705	-0.4743183	-0.5623844	-0.6096899
734	BCUT_PEOE_2	The BCUT descriptors [Pearlman 1998] are calculated from the eigenvalues of a modified adjacency matrix. Each ij entry of the adjacency matrix takes the value 1/sqrt(bij) where bij is the formal bond order between bonded atoms i and j. The diagonal takes the value of the PEOE partial charges. The resulting eigenvalues are sorted and the smallest, 1/3-ile, 2/3-ile and largest eigenvalues are reportec	MOE	0.345441	0.5010937	0.4197896	0.4232548	0.5921377	0.3452275	0.5248433	0.3454546	0.3454478	0.3454479	0.3454481	0.4233415	0.5008471	0.4254572	0.4241196	0.5059683	0.4669236	0.3454545	0.5987654	0.6290757
735	BCUT_PEOE_3	The BCUT descriptors [Pearlman 1998] are calculated from the eigenvalues of a modified adjacency matrix. Each ij entry of the adjacency matrix takes the value 1/sqrt(bij) where bij is the formal bond order between bonded atoms i and j. The diagonal takes the value of the PEOE partial charges. The resulting eigenvalues are sorted and the smallest, 1/3-ile, 2/3-ile and largest eigenvalues are reportec	MOE	2.5073121	2.5303884	2.5413401	2.5933609	2.5477884	2.369566	2.5552154	2.6722596	2.6616106	2.6556058	2.5957134	2.5737336	2.7587705	2.6121252	2.6410496	2.5403628	2.6105087	2.641588	2.5523913	2.5483546
736	BCUT_SLOGP_0	The BCUT descriptors using atomic contribution to logP (using the Wildman and Crippen SlogP methoc instead of partial charge.	MOE	-3.3039858	-3.312979	-3.3072324	-3.306875	-3.3101819	-3.2889431	-3.310199	-3.3135793	-3.3097563	-3.3078585	-3.3078804	-3.3076026	-3.3800206	-3.3069363	-3.3072762	-3.3161137	-3.319597	-3.3131099	-3.3101063	-3.3101802
737	BCUT_SLOGP_1	The BCUT descriptors using atomic contribution to logP (using the Wildman and Crippen SlogP methoc instead of partial charge.	MOE	-0.406841	-0.6091809	-0.5892785	-0.5495883	-0.4989418	-0.4549814	-0.513395	-0.3704034	-0.4000589	-0.3965118	-0.2261044	-0.5165007	-0.5773922	-0.5097163	-0.494608	-0.6478223	-0.521123	-0.3745492	-0.5121304	-0.6118475
738	BCUT_SLOGP_2	The BCUT descriptors using atomic contribution to logP (using the Wildman and Crippen SlogP methoc instead of partial charge.	MOE	0.2142	0.3936764	0.3030679	0.3046041	0.6148952	0.2142	0.4665528	0.2142	0.2142	0.2142	0.3366406	0.3320352	0.39177	0.3334165	0.3046741	0.3906922	0.3630091	0.2142	0.6744286	0.5937324

Table S11 Amino acid descriptors (1D) used in this study.

No.	Descriptor ID	Attribute	Ref*	A	C	D	E	F	G	H	I	K	L	M	N	P	Q	R	S	T	V	W	Y
739	BCUT_SLOGP_3	The BCUT descriptors using atomic contribution to logP (using the Wildman and Crippen SlogP methoc instead of partial charge.	MOE	2.316818	2.3096085	2.3227482	2.5176799	2.4081538	1.9479774	2.3531401	2.6752872	2.6571643	2.6830802	2.5316074	2.3349774	2.6190667	2.5231216	2.5801501	2.2001691	2.4143913	2.6007802	2.4398394	2.4289186
740	BCUT_SMR_0	The BCUT descriptors using atomic contribution to molar refractivity (using the Wildman and Crippe SMR method) instead of partial charge	MOE	-2.30796	-2.2983494	-2.3157358	-2.370079	-2.3249383	-2.2018034	-2.3204968	-2.4583325	-2.4513316	-2.4473414	-2.3640416	-2.3214085	-2.3058555	-2.3731861	-2.412833	-2.3183911	-2.3775434	-2.4278007	-2.3212147	-2.3249815
741	BCUT_SMR_1	The BCUT descriptors using atomic contribution to molar refractivity (using the Wildman and Crippe SMR method) instead of partial charge	MOE	-1.022E-17	-0.2784052	-0.1524074	-0.15253	-0.4473805	1.456E-17	-0.381255	1.433E-16	5.524E-17	-5.295E-17	0.03209	-0.2313392	-0.2894175	-0.2363989	-0.1525171	-0.2970782	-0.2281964	-1.205E-16	-0.4288943	-0.4808459
742	BCUT_SMR_2	The BCUT descriptors using atomic contribution to molar refractivity (using the Wildman and Crippe SMR method) instead of partial charge	MOE	0.1057	0.5107423	0.3082684	0.3091198	0.7364776	0.1057	0.664115	0.1057	0.1057	0.1057	0.3380091	0.4476779	0.4964164	0.4603454	0.4003973	0.504243	0.4353208	0.1057	0.7459933	0.7759593
743	BCUT_SMR_3	The BCUT descriptors using atomic contribution to molar refractivity (using the Wildman and Crippe SMR method) instead of partial charge	MOE	2.6955085	2.7550433	2.7412763	2.8003836	2.7547119	2.5432687	2.7470851	2.8866551	2.8649819	2.8748624	2.8324771	2.7589712	2.9519591	2.8105457	2.8467581	2.7133796	2.7927809	2.8494208	2.7640889	2.7553606
744	bpol	Sum of the absolute value of the difference between atomic polarizabilities of all bonded atoms in th molecule (including implicit hydrogens) with polarizabilities taken from [CRC 1994	MOE	8.2484493	10.528449	9.0712423	11.257656	12.621277	6.0620351	12.414863	14.807691	15.674105	14.807691	14.901277	9.6396561	11.754863	11.82607	16.334105	8.2484493	10.434863	12.621277	14.374484	12.621277
745	CASA-	Negative charge weighted surface area, ASA- times max { qi < 0 } [Stanton 1990]	MOE	170.73997	347.11844	592.50446	572.67389	477.48856	186.55573	316.81412	155.38933	228.10017	151.62022	289.49899	380.01422	165.90945	438.76068	303.8544	273.36823	258.61749	149.72884	623.81952	543.19904
746	CASA+	Positive charge weighted surface area, ASA+ times max { qi > 0 } [Stanton 1990]	MOE	220.13626	301.16101	314.27151	288.25296	590.00885	282.98395	648.34985	163.49655	677.57123	186.63361	354.6983	574.29938	177.26285	577.44708	1248.5764	373.29877	293.93576	192.77248	778.42035	689.65973
747	chi0_C	Carbon connectivity index (order 0). This is calculated as the sum of 1/sqrt(di) over all carbon atoms with di > 0.	MOE	2.1547005	1.8618073	2.4391575	3.1462643	5.9746914	1.2844571	3.8533711	4.4391575	3.9831276	4.4391575	3.5689142	2.4391575	3.2760208	3.1462643	3.8533711	1.8618073	2.7320509	3.7320509	7.1293921	5.8449349
748	chi0v_C	Carbon valence connectivity index (order 0). This is calculated as the sum of 1/sqrt(vi) over all carbo atoms i with vi > 0.	MOE	2.0773504	1.7844571	2.284457	2.9915638	5.1712084	1.2071068	3.4391575	4.3618073	3.9057775	4.3618073	3.4915638	2.284457	3.1986706	2.9915638	3.6986706	1.7844571	2.6547005	3.6547005	6.1712084	5.0938582
749	chi1_C	Carbon connectivity index (order 1). This is calculated as the sum of 1/sqrt(didj) over all bonds betwee carbon atoms i and j where i < j	MOE	0.9106836	0.7415816	1.1498299	1.6498299	3.9663265	0.4082483	1.5580782	2.3593719	2.2415817	2.3045304	1.2415817	1.1498299	1.7415817	1.6498299	1.7415817	0.7415816	1.2440169	1.8213673	4.5412416	3.7828231
750	chi1v	Atomic valence connectivity index (order 1) from [Hall 1991] and [Hall 1977]. This is calculated as th sum of 1/sqrt(vivj) over all bonds between heavy atoms i and j where i < j	MOE	1.6825219	2.4621034	2.2752216	2.7752216	3.777657	1.2618017	3.2148044	3.1312103	3.5134201	3.0763688	4.1041903	2.3597727	2.8467534	2.8597727	3.6943238	1.8296478	2.2740543	2.5932057	4.7716742	3.9119475
751	chi1v_C	Carbon valence connectivity index (order 1). This is calculated as the sum of 1/sqrt(vivj) over all bond between carbon atoms i and j where i < j	MOE	0.8660254	0.6969234	1.0504768	1.5504768	2.9611604	0.3535534	1.339152	2.3147137	2.1969235	2.2598722	1.1969234	1.0504768	1.6969234	1.5504768	1.6969234	0.6969234	1.1993587	1.776709	3.4165022	2.8718441
752	chiral	The number of chiral centers	MOE	1	1	1	1	1	0	1	2	1	1	1	1	1	1	1	1	2	1	1	1
753	chiral_u	The number of unconstrained chiral centers	MOE	1	1	1	1	1	0	1	2	1	1	1	1	1	1	1	1	2	1	1	1
754	DASA	Absolute value of the difference between ASA+ and ASA-	MOE	18.619032	15.004052	41.66037	44.621246	31.722664	36.346867	94.186287	3.0558691	85.310944	13.197659	20.944204	48.29361	4.1956363	34.473381	138.20151	29.982159	10.59654	16.224514	39.815094	37.276829
755	DCASA	Absolute value of the difference between CASA+ and CASA- [Stanton 1990]	MOE	49.396294	45.957413	278.23294	284.42096	112.50229	96.42823	331.53574	8.1072206	449.47107	35.013393	65.19931	194.28519	11.353392	138.68642	944.72205	99.930534	35.318268	43.043636	154.60081	146.46065
756	dens	Mass density: molecular weight divided by van der Waals volume as calculated in the vol descriptor	MOE	1.0590669	1.1791728	1.2686194	1.2051299	1.0165662	1.1079959	1.1172421	0.9557377	0.98132	0.9618698	1.076386	1.1551387	1.0514338	1.1198927	1.0562893	1.1709527	1.1211294	0.9721826	1.0433154	1.0729296
757	density	Molecular mass density: Weight divided by vdw_vol (amu/Å3)	MOE	0.802303	0.9153205	0.9382713	0.8844372	0.7308679	0.866638	0.8154287	0.7116106	0.7255026	0.7116106	0.818557	0.8730686	0.7798904	0.8315262	0.7718104	0.8789243	0.8272268	0.7326046	0.7525867	0.7725248
758	dipole	Dipole moment calculated from the partial charges of the molecule	MOE	2.6949787	2.4869499	2.5577302	2.1984289	2.6985266	2.7023234	2.5358727	2.6972606	4.5510893	2.7054412	2.2939868	2.3947239	2.8837996	2.7283227	1.5963147	2.706418	2.7277513	2.6792622	2.9398508	2.2812843
759	dipoleX	The x component of the dipole moment (external coordinates)	MOE	-2.5852461	1.513617	-1.4681535	-0.7076395	1.082163	2.624505	1.803962	-1.855317	4.2441449	1.152613	0.401232	1.410008	2.7187991	-1.063055	0.565645	-0.436199	-0.15831	-1.0168	-2.1488676	0.649531
760	dipoleY	The y component of the dipole moment (external coordinates)	MOE	0.702361	-1.76036	-1.8496898	0.9937297	2.433346	-0.643836	-1.4017169	-1.614795	1.3799891	2.445528	-2.2572179	1.4290299	0.379129	2.4900391	1.4407099	-2.668473	2.6647179	2.3426421	-1.4537882	2.1158831
761	dipoleZ	The z component of the dipole moment (external coordinates)	MOE	-0.293433	-0.891637	-1.7033684	-1.8288895	0.435657	-0.00045	1.100709	1.1070009	-0.8917834	-0.101434	0.079721	1.305547	-0.883568	-0.336696	0.3906672	-0.116964	0.561108	0.810304	1.3826029	-0.552636
762	E	Value of the potential energy. The state of all term enable flags will be honored (in addition to the ter weights). This means that the current potential setup accurately reflects what will be calculate	MOE	4.8876524	6.5094767	-21.604616	-23.971395	24.661219	1.352649	-6.0437198	11.715302	7.9783694	11.020361	8.0214634	-12.507687	10.632032	1.3955681	-91.175972	7.9107552	10.313235	9.1572571	21.817936	22.047476
763	E_ang	Angle bend potential energy. In the Potential Setup panel, the term enable (Bonded) flag is ignored, b the term weight is applied	MOE	7.727118	8.1818638	8.6756573	9.3641195	9.8653498	5.7728901	7.429338	12.926147	10.233256	12.149486	9.6778145	8.2719927	9.2048426	10.186963	12.209574	6.0188293	7.5399628	10.354797	8.5105991	10.565646
764	E_ele	Electrostatic component of the potential energy. In the Potential Setup panel, the term enable flag ignored, but the term weight is applied	MOE	-20.224669	-18.728235	-69.513969	-74.139908	-19.402779	-18.948584	-36.423534	-20.508112	-18.037291	-19.00967	-26.400406	-45.240685	-13.366058	-33.226048	-148.2856	-20.162832	-20.12677	-22.609856	-20.016853	-23.91132
765	E_nb	Value of the potential energy with all bonded terms disabled. The state of the non-bonded term enabl flags will be honored (in addition to the term weights)	MOE	-6.2816792	-4.5597239	-34.713947	-36.266319	9.2681875	-7.349792	-16.591763	-1.785501	0.1774343	-1.240423	-7.6244392	-26.620544	-1.5749207	-15.185855	-108.53027	-2.8801441	-1.3472252	-3.6238415	7.9146581	5.9933128
766	E_oop	Out-of-plane potential energy. In the Potential Setup panel, the term enable (Bonded) flag is ignored, b the term weight is applied	MOE	0.0002171	0.0194288	0.1042913	0.0226755	0.0033496	0	0.0039981	0.0012079	0.0017684	0.0012399	0.0063088	0.0445131	6.403E-05	0.0038456	0.1163477	0.0993219	0.0840597	0.0032562	0.0013511	0.0042716
767	E_sol	Solvation energy. In the Potential Setup panel, the term enable parameter (Solvation menu) is ignore but the term weight is applied	MOE	-53.774998	-54.922871	-109.93316	-105.25087	-53.617027	-54.76273	-55.854012	-51.56992	-129.091	-52.566257	-50.920761	-54.360924	-51.789886	-66.37603	-78.890701	-54.19722	-52.978416	-51.557083	-62.645191	-58.146091
768	E_stb	Bond stretch-bend cross-term potential energy. In the Potential Setup panel, the term enable (Bonde flag is ignored, but the term weight is applied	MOE	-0.8133627	-0.5394427	-0.4307219	-0.09539	-0.5932542	-0.7307586	-0.4873628	-0.3946621	-0.4421255	-0.5526193	-0.9348769	-0.5879453	-1.199623	-0.747508	-0.8700019	-0.2127574	-0.0119193	-0.9037118	-1.40843	-0.6466895
769	E_str	Bond stretch potential energy. In the Potential Setup panel, the term enable (Bonded) flag is ignored, b the term weight is applied	MOE	2.0422475	1.8487482	3.0517566	3.0854433	3.4886303	1.6064154	1.9944655	2.9789698	2.4375918	2.701988	2.7633283	1.8142091	2.285665	2.4967604	5.0682406	1.7530596	2.0594392	3.1011229	3.7792215	3.5050592
770	E_strain	Local strain energy: the current energy minus the value of the energy at a near local minimum. Th current energy is calculated as for the E descriptor. The local minimum energy is the value of the E descriptor after first performing an energy minimization. Current chirality is preserved and charges are le undisturbed during minimization. The structure in the database is not modified (results of the minimizatio are discarded).	MOE	0.0009727	0.0011663	0.0017439	0.0028001	0.0023555	0.0010062	0.0013777	0.0042042	0.0045341	0.0022759	0.0036615	0.0019335	0.0011552	0.0023355	0.0035772	0.0013544	0.0013153	0.0010981	0.0041252	0.0017888
771	E_tor	Torsion (proper and improper) potential energy. In the Potential Setup panel, the term enable (Bonde flag is ignored, but the term weight is applied	MOE	2.2131114	1.5586022	1.7083483	-0.0819231	2.6289551	2.0538945	1.6076047	-2.0108585	-2.6095562	-2.0393097	4.1333275	4.5700889	1.9160051	4.6413622	0.8301417	3.1324456	1.9889183	0.225634	3.0205362	2.6258757
772	E_vdw	van der Waals component of the potential energy. In the Potential Setup panel, the term enable flag i ignored, but the term weight is applied	MOE	13.94299	14.16851	34.800026	37.873585	28.670965	11.598792	19.831772	18.72261	18.214725	17.769247	18.775967	18.62014	11.791138	18.040194	39.755322	17.282688	18.779545	18.986013	27.931511	29.904633
773	FASA-	Fractional ASA- calculated as ASA- / ASA.	MOE	0.2654549	0.4268201	0.4813104	0.4238326	0.3856339	0.3241969	0.2758911	0.188765	0.1852707	0.1811502	0.2776299	0.3292663	0.2214778	0.3409091	0.1520507	0.327675	0.2854073	0.1950958	0.4023773	0.3822605
774	FASA_H	Fractional ASA_H calculated as ASA_H / ASA.	MOE	0.3922923	0.2698315	0.2186561	0.3252009	0.6481918	0.1840332	0.4309222	0.6126216	0.3865191	0.5958669	0.3822139	0.1977483	0.5418884	0.2241797	0.3203075	0.2248674	0.3902085	0.5537227	0.5602091	0.5231948
775	FASA_P	Fractional ASA_P calculated as ASA_P / ASA.	MOE	0.6077077	0.7301685	0.7813439	0.6747991	0.3518082	0.8159668	0.5690778	0.3873785	0.6134809	0.4041332	0.6177861	0.8022517	0.4581116	0.7758203	0.6796925	0.7751326	0.6097915	0.4462773	0.4397909	0.4768052
776	FASA+	Fractional ASA+ calculated as ASA+ / ASA.	MOE	0.3422529	0.3703104	0.3270245	0.2732765	0.4765086	0.49177	0.5646022	0.1986135	0.4282101	0.2229829	0.3401562	0.4976062	0.2366338	0.4486659	0.5276418	0.4474576	0.3243842	0.2511815	0.5022273	0.4853279
777	FCASA-	Fractional CASA- calculated as CASA- / ASA.	MOE	0.7042518	1.3073499	2.1942942	1.9322526	1.3678435	0.8600942	0.9711367	0.5007935	0.6495593	0.4805916	0.864262	1.3246384	0.599319	1.3714772	0.8257872	1.0921409	0.9516255	0.5175892	1.5644428	1.5019015
778	FCASA+	Fractional CASA+ calculated as CASA+ / ASA.	MOE	0.9079968	1.1342608	1.1638801	0.9725911	1.6901761	1.3046657	1.9873998	0.5269216	1.9295148	0.5915737	1.0589062	2.0018699	0.6403311	1.804983	3.3932648	1.491376	1.0811723	0.6663843	1.9521576	1.9068534
779	GCUT_PEOE_0	The GCUT descriptors are calculated from the eigenvalues of a modified graph distance adjacenc matrix. Each ij entry of the adjacency matrix takes the value 1/sqr(dij) where dij is the (modified) graph distance between atoms i and j. The diagonal takes the value of the PEOE partial charges. The resulting eigenvalues are sorted and the smallest, 1/3-ile, 2/3-ile and largest eigenvalues are reportec	MOE	-0.8514708	-0.8492063	-0.8544343	-0.8551902	-0.8504111	-0.853658	-0.8510299	-0.8508659	-0.8631378	-0.8513591	-0.8510289	-0.8503171	-0.8682196	-0.8512043	-0.8511861	-0.8465216	-0.8463	-0.8507368	-0.8630247	-0.8503834
780	GCUT_PEOE_1	The GCUT descriptors are calculated from the eigenvalues of a modified graph distance adjacenc matrix. Each ij entry of the adjacency matrix takes the value 1/sqr(dij) where dij is the (modified) graph distance between atoms i and j. The diagonal takes the value of the PEOE partial charges. The resulting eigenvalues are sorted and the smallest, 1/3-ile, 2/3-ile and largest eigenvalues are reportec	MOE	-0.3700041	-0.432287	-0.644707	-0.5929813	-0.382702	-0.4635816	-0.3878678	-0.3127965	-0.3477126	-0.3398836	-0.3317405	-0.4593652	-0.4106306	-0.44241	-0.3719761	-0.4413698	-0.3988986	-0.31707	-0.4001663	-0.4340951
781	GCUT_PEOE_2	The GCUT descriptors are calculated from the eigenvalues of a modified graph distance adjacenc matrix. Each ij entry of the adjacency matrix takes the value 1/sqr(dij) where dij is the (modified) graph distance between atoms i and j. The diagonal takes the value of the PEOE partial charges. The resulting eigenvalues are sorted and the smallest, 1/3-ile, 2/3-ile and largest eigenvalues are reportec	MOE	0.185441	0.1854676	0.1576671	0.131387	0.1527641	0.1852275	0.1846436	0.1481909	0.1849345	0.0964658	0.1518195	0.15871	0.1526	0.1530514	0.1854478	0.185505	0.1839882	0.1685976	0.1854554	0.1854554
782	GCUT_PEOE_3	The GCUT descriptors are calculated from the eigenvalues of a modified graph distance adjacenc matrix. Each ij entry of the adjacency matrix takes the value 1/sqr(dij) where dij is the (modified) graph distance between atoms i and j. The diagonal takes the value of the PEOE partial charges. The resulting eigenvalues are sorted and the smallest, 1/3-ile, 2/3-ile and largest eigenvalues are reportec	MOE	1.9296426	1.9730446	2.0095885	2.1373527	2.2742889	1.7150419	2.1980834	2.3343248	2.371609	2.3139548	2.172781	2.0969279	2.3052645	2.2029221	2.3545918	1.9786209	2.129941	2.2301064	2.4128106	2.2935927
783	GCUT_SLOGP_1	The GCUT descriptors using atomic contribution to logP (using the Wildman and Crippen SlogP methoc instead of partial charge.	MOE	-0.4138166	-0.4531388	-0.5927106	-0.5044374	-0.3673723	-0.5359281	-0.4077922	-0.2894899	-0.3107408	-0.2919759	-0.2534065	-0.4887634	-0.4185046	-0.4235476	-0.374104	-0.56804	-0.4676987	-0.309298	-0.3636571	-0.3932202
784	GCUT_SLOGP_2	The GCUT descriptors using atomic contribution to logP (using the Wildman and Crippen SlogP methoc instead of partial charge.	MOE	0.0542	0.0567282	0.0551061	0.0554542	0.1115577	0.0542	0.0568517	0.0542	0.0542	0.0542	0.0566979	0.0542	0.0566221	0.0542	0.055465	0.0542	0.0542	0.0542	0.1296447	0.0935481
785	GCUT_SLOGP_3	The GCUT descriptors using atomic contribution to logP (using the Wildman and Crippen SlogP methoc instead of partial charge.	MOE	1.7438873	1.7977972	1.8010395	2.021374	2.2682526	1.4078366	2.0849307	2.2977819	2.2953796	2.2927029	2.1127141	1.8712965	2.1629338	2.0717413	2.2145157	1.6977891	1.9356897	2.1552346	2.4617586	2.3096726
786	GCUT_SMR_0	The GCUT descriptors using atomic contribution to molar refractivity (using the Wildman and Crippe SMR method) instead of partial charge	MOE	-0.6405566	-0.6404877	-0.640608	-0.6406886	-0.6407014	-0.6409748	-0.6406753	-0.6408896	-0.6432516	-0.6409208	-0.6406391	-0.6406809	-0.6387873	-0.640684	-0.6407812	-0.6404957	-0.6405997	-0.6407439	-0.6406944	-0.6407009
787	GCUT_SMR_1	The GCUT descriptors using atomic contribution to molar refractivity (using the Wildman and Crippe SMR method) instead of partial charge	MOE	-0.16	-0.1375034	-0.1846992	-0.1836855	-0.2040631	-0.16	-0.1996156	-0.16	-0.16	-0.16	-0.12791	-0.2032759	-0.2004611	-0.2035565	-0.1835209	-0.2070114	-0.1845193	-0.16	-0.2024306	-0.2254214
788	GCUT_SMR_2	The GCUT descriptors using atomic contribution to molar refractivity (using the Wildman and Crippe SMR method) instead of partial charge	MOE	-0.0543	0.0982205	0.0214492	0.0213919	0.1727876	-0.0543	0.1456479	-0.0543	-0.0543	-0.0543	0.0960807	0.0935821	0.0849256	0.0959554	0.0891153	0.0948941	0.064955	-0.0543	0.1722691	0.1837605
789	GCUT_SMR_3	The GCUT descriptors using atomic contribution to molar refractivity (using the Wildman and Crippe SMR method) instead of partial charge	MOE	2.0893912	2.1847334	2.2045603	2.3335552	2.5121033	1.8581887	2.4051452	2.5189342	2.531605	2.5002518	2.3960042	2.2723396	2.4726548	2.3853848	2.5308931	2.1315584	2.2929735	2.4088459	2.6871305	2.5307744
790	glob	Globularity, or inverse condition number (smallest eigenvalue divided by the largest eigenvalue) of th covariance matrix of atomic coordinates. A value of 1 indicates a perfect sphere while a value of 0 indicates a two- or one-dimensional object	MOE	0.2824544	0.4642475	0.3553334	0.3185492	0.2030571	0.193241	0.2088109	0.5397995	0.1219335	0.2778475	0.1286153	0.2738108	0.3720073	0.1668978	0.0683502	0.2095423	0.3713011	0.385729	0.1167546	0.1754128
791	Kier1	First kappa shape index: (n-1)2 / m2 [Hall 1991]	MOE	6	7	9	10	10.083333	5	9.090909	9	10	9	9	9	6.125	10	12	7	8	8	11.484375	11.076923
792	Kier2	Second kappa shape index: (n-1)2 / m2 [Hall 1991]	MOE	2.2222223	3.0612245	3.9200001	4.7603307	4.8888888	2.25	4.1326532	3.9200001	5.7600002	3.9200001	4.8395061	3.9200001	2.52	4.7603307	6.5088758	3.0612245	3.1111112	3.1111112	4.8884296	5.0242214
793	Kier3	Third kappa shape index: (n-1) (n-3)2 / p32 for odd n, and (n-3) (n-2)2 / p32 for even n [Hall 1991	MOE	3	2.6666667	4.5	5.5308642	3.515625	4	2.8444445	2.8800001	5.5308642	4.5	4.5	4.5	1.4876033	5.5308642	7.4380164	2.6666667	2.8125	2.8125	2.5714285	3.7037036
794	KierA1	First alpha modified shape index: s (s-1)2 / m2 where s = n + a [Hall 1991]	MOE	5.03652	7.6879997	7.1872501	8.1884441	7.4624853	4.0214376	6.6297121	8.0597343	9.0641108	8.0597343	9.6657495	6.7219682	5.4008284	7.7182059	9.2505112	6.0467501	7.054143	7.054143	8.2204742	8.4107523
795	KierA2	Second alpha modified shape index: s (s-1)2 / m2 where s = n + a [Hall 1991]	MOE	1.7873056	3.4213877	3.03104	3.8015206	3.5079942	1.6858125	2.9070535	3.4565301	5.1582298	3.4565301	5.2480001	2.8061726	2.1768301	3.5554211	4.8861599	2.569653	2.6874444	2.6874444	3.4068823	3.7183318
796	KierA3	Third alpha modified shape index: (s-1) (s-3)2 / p32 for odd n, and (s-3) (s-2)2 / p32 for even n where s n + a [Hall 1991].	MOE	2.3101876	3.0379999	3.3716249	4.3267159	2.4615855	2.67325	1.9367585	2.50173	4.9042344	3.9089532	4.9251251	3.090023	1.2620083	4.0204959	5.4677176	2.1675832	2.3859844	2.3859844	1.7530622	2.6816823
797	KierFlex	Kier molecular flexibility index: (KierA1) (KierA2) / n [Hall 1991]	MOE	1.5003	3.7576611	2.4205379	3.112854	2.1815295	1.3558779	1.7520844	3.0954127	4.675477	3.0954127	5.6362062	2.0958893	1.4695855	2.7441473	3.766623	2.2197213	2.3697021	2.3697021	1.8670791	2.4056897
798	lip_acc	The number of O and N atoms.	MOE	3	3	5	5	3	3	5	3	4	3	3	5	3	5	6	4	4	3	4	4
799	lip_don	The number of OH and NH atoms.	MOE	3	3	3	3	3	3	4	3	6	3	3	5	2	5	8	4	4	3	4	4
800	lip_violation	The number of violations of Lipinski's Rule of Five [Lipinski 1997]	MOE	0	0	0	0	0	0	0	0	0	0	0	0	0	0	0	0	0	0	0	0

Table S11 Amino acid descriptors (1D) used in this study.

No.	Descriptor ID	Attribute	Ref*	A	C	D	E	F	G	H	I	K	L	M	N	P	Q	R	S	T	V	W	Y
801	logP(o/w)	Log of the octanol/water partition coefficient (including implicit hydrogens). This property is calculate from a linear atom type model [LOGP 1998] with r2 = 0.931, RMSE=0.393 on 1,827 molecules	MOE	-0.538	-0.428	-1.165	-0.723	0.997	-1	-1.307	0.876	-0.469	0.876	0.117	-2.02	0.178	-1.5779999	-0.89	-1.573	-1.1109999	0.434	1.363	0.689
802	logS	Log of the aqueous solubility (mol/L). This property is calculated from an atom contribution linear atom type model [Hou 2004] with r2 = 0.90, ~1,200 molecules	MOE	0.15899	-0.50226	0.34799	0.14622	-1.34317	0.4862	-0.10031	-0.75977	0.21392	-1.07322	-0.70745	0.32572	-0.14176	0.12395	-0.21349	0.68874	0.36153	-0.24455	-1.63307	-0.98122
803	MNDO_dipole	The dipole moment calculated using the MNDO Hamiltonian [MOPAC]	MOE	4.9365287	10.246024	4.7324452	7.5357332	10.898481	4.704031	8.8683414	4.8517351	13.68906	11.144662	4.0135121	8.6196041	12.199814	5.7196245	11.405553	10.53647	4.0284972	4.690774	11.896894	9.7379045
804	MNDO_E	The total SCF energy (kcal/mol) calculated using the MNDO Hamiltonian [MOPAC]	MOE	-30792.805	-36011.754	-48306.023	-51910.949	-49711.781	-27188.607	-49777.227	-41606.09	-46878.137	-41552.258	-43284.094	-46222.332	-37292.648	-49881.516	-56417.855	-38170.043	-41828.875	-38001.906	-60699.336	-57150.109
805	MNDO_HF	The heat of formation (kcal/mol) calculated using the MNDO Hamiltonian [MOPAC]	MOE	-93.139107	-33.705299	-203.73164	-205.61893	-7.22577	-91.978622	-1.16733	-97.312599	68.274002	-43.48048	-99.941101	-72.029823	-40.18829	-128.31139	97.150169	-80.558517	-136.37608	-96.164719	15.58614	-55.758492
806	MNDO_HOMO	The energy (eV) of the Highest Occupied Molecular Orbital calculated using the MNDO Hamiltonia [MOPAC].	MOE	-10.70008	-9.1820002	-4.8850002	-4.6212201	-8.9765196	-10.95102	-8.7708597	-10.75197	-13.69352	-9.0292902	-9.8342199	-9.2730703	-8.86129	-10.86477	-12.51601	-9.2079802	-10.86853	-10.84703	-8.9769201	-8.9719696
807	MNDO_IP	The ionization potential (kcal/mol) calculated using the MNDO Hamiltonian [MOPAC	MOE	10.70008	9.1820002	4.8850002	4.6212201	8.9765196	10.95102	8.7708597	10.75197	13.69352	9.0292902	9.8342199	9.2730703	8.86129	10.86477	12.51601	9.2079802	10.86853	10.84703	8.9769201	8.9719696
808	MNDO_LUMO	The energy (eV) of the Lowest Unoccupied Molecular Orbital calculated using the MNDO Hamiltonia [MOPAC].	MOE	1.1576101	-0.12225	4.5859199	3.81356	-0.21289	1.0281	0.34722	1.15125	-4.3470502	0.00914	1.0989701	-0.08466	-0.07191	0.95942	-4.62286	-0.15392	0.9416	1.10621	-0.48383	-0.25313
809	npr1	Normalized PMI ratio pmi1/pmi3	MOE	0.4612954	0.4451444	0.3896995	0.3235536	0.3202083	0.2863152	0.3578709	0.55215	0.218494	0.3846345	0.2270933	0.4460634	0.3821661	0.1945262	0.2662311	0.4390575	0.5349492	0.5187139	0.2282162	0.2654018
810	npr2	Normalized PMI ratio pmi2/pmi3	MOE	0.6413249	0.8406363	0.8260843	0.895445	0.9021517	0.7517954	0.909336	0.9692915	0.8537893	0.891049	0.8766262	0.8155468	0.8249959	0.9291891	0.7744201	0.6264931	0.7406777	0.7861859	0.8788248	0.9234953
811	opr_brigid	The number of rigid bonds from [Oprea 2000]	MOE	0	0	0	0	6	0	5	0	0	0	0	0	5	0	1	0	0	0	10	6
812	opr_nring	The number of ring bonds from [Oprea 2000]	MOE	0	0	0	0	1	0	1	0	0	0	0	0	1	0	0	0	0	0	2	1
813	opr_nrot	The number of rotatable bonds from [Oprea 2000]	MOE	1	2	3	4	3	1	3	3	5	3	4	3	2	4	6	2	2	2	3	3
814	opr_violation	The number of violations of Oprea's lead-like test [Oprea 2000]	MOE	0	0	1	1	0	0	0	0	1	0	0	1	0	0	2	1	0	0	0	0
815	PEOE_PC-	Total negative partial charge: the sum of the negative qi. Q_PC- is identical to PC- which has bee retained for compatibility	MOE	-1.4483862	-1.6129148	-2.5405571	-2.5487123	-1.7841777	-1.4445146	-2.0325921	-1.6103809	-1.8475491	-1.607512	-1.624445	-2.0841365	-1.429723	-2.0849543	-2.3180566	-1.8284317	-1.8581457	-1.5551536	-2.0290475	-2.0176141
816	PEOE_PC+	Total positive partial charge: the sum of the positive qi. Q_PC+ is identical to PC+ which has been retained for compatibility	MOE	1.4483862	1.6129148	1.5405571	1.5487123	1.7841777	1.4445146	2.0325921	1.6103809	2.8475492	1.607512	1.624445	2.0841365	1.429723	2.0849543	3.3180566	1.8284317	1.8581457	1.5551536	2.0290475	2.0176141
817	PEOE_RPC-	Relative negative partial charge: the smallest negative qi divided by the sum of the negative qi. Q_RPC is identical to RPC- which has been retained for compatibility	MOE	0.3755316	0.3372095	0.2163296	0.2157238	0.3048471	0.3766757	0.2675898	0.3377475	0.2943945	0.3383541	0.334827	0.2609713	0.3804287	0.260873	0.2346398	0.297444	0.2926839	0.3497417	0.2680575	0.2695765
818	PEOE_RPC+	Relative positive partial charge: the largest positive qi divided by the sum of the positive qi. Q_RPC+ i identical to RPC+ which has been retained for compatibility	MOE	0.2385006	0.2141883	0.2242408	0.2230549	0.1936216	0.2389921	0.1699583	0.2145173	0.1213141	0.214896	0.2126561	0.1657554	0.2428319	0.1656861	0.1041115	0.1889625	0.1859447	0.2221353	0.170255	0.1712198
819	PEOE_VSA_FHYD	Fractional hydrophobic van der Waals surface area. This is the sum of the vi such that |qi| is less than c equal to 0.2 divided by the total surface area. The vi are calculated using a connection table approximation.	MOE	0.4979926	0.5824884	0.3995875	0.4674224	0.6965284	0.402435	0.6280831	0.6635829	0.5409982	0.6635829	0.6739947	0.3933851	0.6383091	0.4582774	0.3826968	0.3871461	0.4653611	0.6232039	0.7293931	0.6166088
820	PEOE_VSA_FNEG	Fractional negative van der Waals surface area. This is the sum of the vi such that qi is negative divide by the total surface area. The vi are calculated using a connection table approximatior	MOE	0.4360334	0.4214166	0.4092903	0.4200977	0.5145475	0.31194	0.2114138	0.4629064	0.2647105	0.4087941	0.4785012	0.336851	0.2211776	0.3008166	0.2462121	0.3056867	0.4055227	0.4578602	0.3972009	0.4633056
821	PEOE_VSA_FPNEG	Fractional negative polar van der Waals surface area. This is the sum of the vi such that qi is less than 0.2 divided by the total surface area. The vi are calculated using a connection table approximatior	MOE	0.2620571	0.2179488	0.4092903	0.3630484	0.1584178	0.31194	0.2114138	0.175616	0.1661667	0.175616	0.1701808	0.336851	0.2211776	0.3008166	0.2036877	0.3056867	0.2666737	0.1966945	0.1415883	0.1912325
822	PEOE_VSA_FPOL	Fractional polar van der Waals surface area. This is the sum of the vi such that |qi| is greater than 0. divided by the total surface area. The vi are calculated using a connection table approximatior	MOE	0.5020074	0.4175117	0.6004125	0.5325776	0.3034716	0.5975651	0.371917	0.3364171	0.4590018	0.3364171	0.3260053	0.6066149	0.3616909	0.5417226	0.6173032	0.6128539	0.5346389	0.3767961	0.2706069	0.3833912
823	PEOE_VSA_FPOS	Fractional positive van der Waals surface area. This is the sum of the vi such that qi is non-negativ divided by the total surface area. The vi are calculated using a connection table approximatior	MOE	0.5639666	0.5785835	0.5907098	0.5799022	0.4854525	0.68806	0.7885862	0.5370936	0.7352895	0.5912058	0.5214988	0.6631489	0.7788224	0.6991834	0.7537879	0.6943133	0.5944773	0.5421398	0.6027991	0.5366944
824	PEOE_VSA_FPPOS	Fractional positive polar van der Waals surface area. This is the sum of the vi such that qi is greater tha 0.2 divided by the total surface area. The vi are calculated using a connection table approximatior	MOE	0.2399503	0.1995629	0.1911222	0.1695292	0.1450538	0.285625	0.1605032	0.1608012	0.292835	0.1608012	0.1558245	0.2697639	0.1405133	0.240906	0.4136155	0.3071673	0.2679653	0.1801016	0.1290186	0.1921587
825	PEOE_VSA_HYD	Total hydrophobic van der Waals surface area. This is the sum of the vi such that |qi| is less than c equal to 0.2. The vi are calculated using a connection table approximatior	MOE	53.663681	75.472076	54.060429	71.292519	124.16192	36.431595	101.18411	106.70499	95.5392	106.70499	111.84059	56.589958	78.307114	73.822044	78.307114	45.60191	62.833996	89.4729	146.18004	116.10015
826	PEOE_VSA_NEG	Total negative van der Waals surface area. This is the sum of the vi such that qi is negative. The vi ar calculated using a connection table approximation	MOE	46.986954	54.602264	55.373119	64.074432	91.722336	28.239277	34.058743	74.435944	46.747337	65.734634	79.401009	48.457317	27.133842	48.457317	50.379719	36.006817	54.754494	65.734634	79.604324	87.23497
827	PEOE_VSA_PNEG	Total negative polar van der Waals surface area. This is the sum of the vi such that qi is less than -0.2 The vi are calculated using a connection table approximation	MOE	28.239277	28.239277	55.373119	55.373119	28.239277	28.239277	34.058743	28.239277	29.344711	28.239277	28.239277	48.457317	27.133842	48.457317	41.678406	36.006817	36.006817	28.239277	28.376167	36.006817
828	PEOE_VSA_POL	Total polar van der Waals surface area. This is the sum of the vi such that |qi| is greater than 0.2. The v are calculated using a connection table approximation	MOE	54.096313	54.096313	81.230156	81.230156	54.096313	54.096313	59.915783	54.096313	81.058784	54.096313	54.096313	87.263885	44.371868	87.263885	126.31208	72.188026	72.188026	54.096313	54.233208	72.188026
829	PEOE_VSA_POS	Total positive van der Waals surface area. This is the sum of the vi such that qi is non-negative. The v are calculated using a connection table approximation	MOE	60.773045	74.966125	79.917473	88.448242	86.535896	62.288635	127.04115	86.365356	129.85065	95.066673	86.535896	95.39653	95.545135	112.62861	154.23947	81.783119	80.267532	77.834587	120.80892	101.05321
830	PEOE_VSA_PPOS	Total positive polar van der Waals surface area. This is the sum of the vi such that qi is greater than 0.2 The vi are calculated using a connection table approximation	MOE	25.857038	25.857038	25.857038	25.857038	25.857038	25.857038	25.857038	25.857038	51.714077	25.857038	25.857038	38.806568	17.238026	38.806568	84.633675	36.181213	36.181213	25.857038	25.857038	36.181213
831	PEOE_VSA+0	Sum of vi where qi is in the range [0.00,0.05)	MOE	12.796158	19.208147	31.940584	40.471355	17.232086	0	33.978001	38.388474	34.293629	47.089787	38.559017	8.5307722	34.464172	34.464172	25.762857	0	12.796158	29.857702	42.886093	17.232086
832	PEOE_VSA+1	Sum of vi where qi is in the range [0.05,0.10)	MOE	14.708499	0	14.708499	14.708499	36.035431	23.239271	46.910362	14.708499	36.431595	14.708499	14.708499	8.701313	36.431595	14.708499	36.431595	8.5307722	4.2653861	14.708499	36.035431	40.228561
833	PEOE_VSA+2	Sum of vi where qi is in the range [0.10,0.15)	MOE	7.4113464	29.900944	7.4113464	7.4113464	7.4113464	13.192325	11.676732	7.4113464	7.4113464	7.4113464	7.4113464	22.119846	7.4113464	7.4113464	7.4113464	33.925179	23.878815	7.4113464	7.4113464	7.4113464
834	PEOE_VSA+3	Sum of vi where qi is in the range [0.15,0.20)	MOE	0	0	0	0	0	0	8.6190128	0	0	0	0	17.238026	0	17.238026	0	3.1459606	3.1459606	0	8.6190128	0
835	PEOE_VSA+4	Sum of vi where qi is in the range [0.20,0.25)	MOE	0	0	0	0	0	0	0	0	0	0	0	12.949531	0	12.949531	0	10.324173	10.324173	0	0	10.324173
836	PEOE_VSA+6	Sum of vi where qi is greater than 0.3	MOE	25.857038	25.857038	25.857038	25.857038	25.857038	25.857038	25.857038	25.857038	51.714077	25.857038	25.857038	25.857038	17.238026	25.857038	41.538612	25.857038	25.857038	25.857038	25.857038	25.857038
837	PEOE_VSA-0	Sum of vi where qi is in the range [-0.05,0.00)	MOE	18.747677	0	0	8.701313	2.2085397	0	0	8.701313	17.402626	0	31.401114	0	0	0	8.701313	0	18.747677	0	14.463444	26.718348
838	PEOE_VSA-1	Sum of vi where qi is in the range [-0.10,-0.05)	MOE	0	0	0	0	61.274521	0	0	37.495354	0	37.495354	0	0	0	0	0	0	0	37.495354	36.764713	24.509808
839	PEOE_VSA-3	Sum of vi where qi is in the range [-0.20,-0.15)	MOE	0	26.362989	0	0	0	0	0	0	0	0	19.760618	0	0	0	0	0	0	0	0	0
840	PEOE_VSA-4	Sum of vi where qi is in the range [-0.25,-0.20)	MOE	0	0	0	0	0	0	5.6825762	0	0	0	0	0	0	0	0	0	0	0	0	0
841	PEOE_VSA-5	Sum of vi where qi is in the range [-0.30,-0.25)	MOE	0	0	0	0	0	0	0	0	0	0	0	13.566921	0	13.566921	13.43913	0	0	0	0	0
842	PEOE_VSA-6	Sum of vi where qi is less than -0.30	MOE	28.239277	28.239277	55.373119	55.373119	28.239277	28.239277	28.376167	28.239277	29.344711	28.239277	28.239277	34.890396	27.133842	34.890396	28.239277	36.006817	36.006817	28.239277	28.376167	36.006817
843	petitjean	Value of (diameter - radius) / diameter	MOE	0.3333333	0.5	0.4	0.5	0.4285714	0.3333333	0.5	0.4	0.4285714	0.4	0.5	0.4	0.5	0.5	0.5	0.5	0.5	0.5	0.5	0.5
844	petitjeanSC	Petitjean graph Shape Coefficient as defined in [Petitjean 1992]: (diameter - radius) / radius	MOE	0.5	1	0.6666667	1	0.75	0.5	1	0.6666667	0.75	0.6666667	1	0.6666667	1	1	1	1	1	1	1	1
845	PM3_dipole	The dipole moment calculated using the PM3 Hamiltonian [MOPAC]	MOE	10.269957	8.7255383	5.2069073	5.624454	9.7426167	10.531272	10.195673	10.155876	5.5756879	10.03338	8.246892	8.0771103	10.651457	9.6434412	6.9711885	9.9868183	9.9128399	9.9678268	11.597596	8.6219454
846	PM3_E	The total SCF energy (kcal/mol) calculated using the PM3 Hamiltonian [MOPAC]	MOE	-27964.857	-32253.854	-43949.574	-47411.125	-45767.219	-24513.844	-44345.391	-38308.004	-42611.621	-38308.348	-39149.43	-41574.898	-34141.023	-45016.219	-50105.348	-34739.262	-38188.172	-34859.469	-55323.762	-52544.727
847	PM3_HF	The heat of formation (kcal/mol) calculated using the PM3 Hamiltonian [MOPAC]	MOE	-70.46209	-62.504662	-208.91849	-227.39375	-43.008709	-61.563869	-37.25885	-84.383781	66.828362	-82.123833	-71.932533	-111.29704	-67.634918	-109.50355	68.4356	-112.90531	-118.73889	-78.9235	-26.47459	-66.074982
848	PM3_HOMO	The energy (eV) of the Highest Occupied Molecular Orbital calculated using the PM3 Hamiltonia [MOPAC].	MOE	-9.4281797	-9.6815004	-5.5651202	-5.10636	-9.4233303	-9.4928703	-8.9935102	-9.3930101	-10.32884	-9.4065304	-9.15413	-9.4542799	-9.3266296	-9.6409597	-13.1213	-9.7769804	-9.73034	-9.3779898	-8.9980698	-9.4090405
849	PM3_IP	The ionization potential (kcal/mol) calculated using the PM3 Hamiltonian [MOPAC	MOE	9.4281797	9.6815004	5.5651202	5.10636	9.4233303	9.4928703	8.9935102	9.3930101	10.32884	9.4065304	9.15413	9.4542799	9.3266296	9.6409597	13.1213	9.7769804	9.73034	9.3779898	8.9980698	9.4090405
850	pmi	Principal moment of inertia	MOE	236.78682	427.23801	548.1225	733.58539	1016.7993	166.55258	806.12885	556.88367	1040.3356	610.70612	895.93256	536.99377	393.95789	1005.4482	1222.0095	325.39059	406.59518	432.23633	1814.7333	1327.8734
851	pmi1	First diagonal element of diagonalized moment of inertia tensor	MOE	103.89767	166.40494	192.80138	213.92912	293.01068	46.794849	254.48943	243.89484	219.37836	206.44226	193.42912	211.82541	136.42618	184.19232	318.85599	138.33133	191.16295	194.54814	393.11194	322.00693
852	pmi2	Second diagonal element of diagonalized moment of inertia tensor	MOE	144.44574	314.24866	408.70001	592.05566	825.52533	122.87211	646.64764	428.15399	857.24512	478.2467	746.67554	387.2847	294.50821	879.82745	927.49677	197.38559	264.67957	294.86584	1513.8126	1120.4591
853	pmi3	Third diagonal element of diagonalized moment of inertia tensor	MOE	225.23021	373.82242	494.74368	661.18597	915.06262	163.43822	711.12067	441.71851	1004.0476	536.72327	851.7605	474.87738	356.98138	946.87665	1197.6663	315.06427	357.34787	375.05865	1722.542	1213.2808
854	pmiX	x component of the principal moment of inertia (external coordinates)	MOE	132.62314	253.76964	355.3064	519.65515	723.76208	119.734	551.5885	312.97681	820.354	404.16589	701.69543	324.93027	257.4823	821.12958	903.14111	186.19058	214.64459	237.40164	1421.5148	1005.7178
855	pmiY	y component of the principal moment of inertia (external coordinates)	MOE	92.606102	114.88908	139.43689	141.48149	190.98949	43.704216	159.52396	127.63605	183.61076	132.39616	149.10228	149.93472	99.477585	125.73605	294.51141	128.87172	142.70319	137.64952	300.72763	207.36359
856	pmiZ	z component of the principal moment of inertia (external coordinates)	MOE	11.557572	58.579281	53.37923	72.448746	102.04774	3.1143682	95.016441	116.2708	36.370747	74.144035	45.13485	62.128761	36.998005	58.582615	24.356945	10.328295	49.247414	57.18515	92.490906	114.79205
857	radius	If ri is the largest matrix entry in row i of the distance matrix D, then the radius is defined as the smalle of the ri [Petitjean 1992].	MOE	2	2	3	3	4	2	3	3	4	3	3	3	2	3	4	2	2	2	4	4
858	reactive	Indicator of the presence of reactive groups. A non-zero value indicates that the molecule contains a reactive group. The table of reactive groups is based on the Oprea set [Oprea 2000] and includes metals phospho-, N/O/S-N/O/S single bonds, thiols, acyl halides, Michael Acceptors, azides, esters, etc.	MOE	0	1	0	0	0	0	0	0	0	0	0	0	0	0	0	0	0	0	0	0
859	rgyr	Radius of gyration.	MOE	1.6302512	1.8778259	2.0370216	2.2406166	2.4809792	1.4895365	2.2793791	2.0604246	2.6584945	2.1576977	2.4503767	2.0160532	1.8498089	2.6229281	2.6409206	1.7596067	1.8475184	1.9208488	2.980902	2.7071357
860	rings	The number of rings.	MOE	0	0	0	0	1	0	1	0	0	0	0	0	1	0	0	0	0	0	2	1
861	SlogP	Log of the octanol/water partition coefficient (including implicit hydrogens). This property is an atom contribution model [Crippen 1999] that calculates logP from the given structure; i.e. the correct protonation state (washed structures). Results may vary from the logP(o/w) descriptor. The training set for SlogP was ~7000 structures.	MOE	-2.6333001	-2.7234001	-4.5131998	-4.1230998	-1.41053	-3.0218	-2.6874299	-1.6071	-3.2409999	-1.6071	-1.9001	-3.7778001	-2.5379	-3.3877001	-5.2096	-3.6609001	-3.2723999	-1.9972	-0.92923	-1.7049299
862	SlogP_VSA0	Subdivided Surface Areas, Sum of vi such that Li <= -0.4	MOE	75.580528	75.580528	118.26387	118.26387	75.580528	75.580528	75.580528	75.580528	108.47771	75.580528	75.580528	108.47771	60.694096	108.47771	158.16043	100.96576	100.96576	75.580528	75.580528	100.96576
863	SlogP_VSA1	Subdivided Surface Areas, Sum of vi such that Li is in (-0.4,-0.2]	MOE	1.4640081	1.4640081	2.9280162	2.9280162	1.4640081	1.4640081	18.249538	1.4640081	1.4640081	1.4640081	1.4640081	6.7227917	1.4640081	6.7227917	3.3631008	1.4640081	1.4640081	1.4640081	1.4640081	1.4640081
864	SlogP_VSA2	Subdivided Surface Areas, Sum of vi such that Li is in (-0.2,0]	MOE	27.844185	27.844185	51.706402	51.706402	27.844185	23.862217	44.629715	27.844185	27.844185	27.844185	27.844185	51.706402	27.844185	51.706402	27.844185	27.844185	34.312836	27.844185	44.629715	27.844185
865	SlogP_VSA3	Subdivided Surface Areas, Sum of vi such that Li is in (0,0.1]	MOE	0	17.233564	0	0	0	18.439579	0	0	18.439579	0	17.233564	0	18.439579	0	18.439579	20.926258	0	0	0	0
866	SlogP_VSA4	Subdivided Surface Areas, Sum of vi such that Li is in (0.1,0.15]	MOE	0	0	0	0	3.185575	0	2.7567475	4.4107962	0	4.4107962	0	0	0	0	0	0	0	4.4107962	3.185575	3.185575
867	SlogP_VSA5	Subdivided Surface Areas, Sum of vi such that Li is in (0.15,0.20]	MOE	0	0	0	0	18.868406	0	18.868406	0	0	0	31.691174	0	0	0	0	0	0	0	18.868406	18.868406
868	SlogP_VSA7	Subdivided Surface Areas, Sum of vi such that Li is in (0.25,0.30]	MOE	0	0	0	0	88.215919	0	33.999886	0	0	0	0	0	0	0	0	0	0	0	92.504196	70.572739
869	SlogP_VSA8	Subdivided Surface Areas, Sum of vi such that Li is in (0.30,0.40]	MOE	0	46.107109	18.868406	37.736813	0	0	0	18.868406	56.605217	18.868406	18.868406	18.868406	37.736813	37.736813	37.736813	0	0	0	0	0
870	SlogP_VSA9	Subdivided Surface Areas, Sum of vi such that Li > 0.40	MOE	33.326015	0	0	0	0	0	0	66.652031	0	66.652031	32.21954	0	0	0	0	0	33.326015	66.652031	0	5.2434282
871	SMR_VSA0	Subdivided Surface Areas, Sum of vi such that Ri is in [0,0.11]	MOE	66.545563	66.545563	133.09113	133.09113	66.545563	66.545563	66.545563	66.545563	66.545563	66.545563	66.545563	90.407776	66.545563	90.407776	66.545563	66.545563	66.545563	66.545563	66.545563	66.545563
872	SMR_VSA1	Subdivided Surface Areas, Sum of vi such that Ri is in (0.11,0.26]	MOE	0	0	0	0	0	0	16.78553	0	0	0	0	0	18.01075	0	31.671967	25.385227	25.385227	0	0	25.385227
873	SMR_VSA2	Subdivided Surface Areas, Sum of vi such that Ri is in (0.26,0.35]	MOE	32.897186	32.897186	32.897186	32.897186	32.897186	32.897186	49.682716	37.307983	65.794373	37.307983	32.897186	32.897186	0	32.897186	50.90794	32.897186	32.897186	37.307983	49.682716	32.897186
874	SMR_VSA3	Subdivided Surface Areas, Sum of vi such that Ri is in (0.35,0.39]	MOE	3.9819686	3.9819686	3.9819686	3.9819686	7.1675434	0	6.7387161	3.9819686	3.9819686	3.9819686	3.9819686	3.9819686	3.9819686	3.9819686	3.9819686	3.9819686	10.450618	3.9819686	7.1675434	12.410972
875	SMR_VSA4	Subdivided Surface Areas, Sum of vi such that Ri is in (0.39,0.44]	MOE	0	0	0	0	0	0	0	0	0	0	0	32.897186	0	32.897186	32.897186	0	0	0	4.7171016	0
876	SMR_VSA5	Subdivided Surface Areas, Sum of vi such that Ri is in (0.44,0.485]	MOE	0	0	18.868406	37.736813	107.08433	0	52.868294	18.868406	56.605217	18.868406	18.868406	18.868406	37.736813	37.736813	37.736813	0	0	0	106.6555	89.441139
877	SMR_VSA6	Subdivided Surface Areas, Sum of vi such that Ri is in (0.485,0.56]	MOE	1.4640081	18.697573	2.9280162	2.9280162	1.4640081	19.903585	1.4640081	1.4640081	19.903585	1.4640081	18.697573	6.7227917	19.903585	6.7227917	21.802679	22.390266	1.4640081	1.4640081	1.4640081	1.4640081
878	SMR_VSA7	Subdivided Surface Areas, Sum of vi such that Ri > 0.56	MOE	33.326015	46.107109	0	0	0	0	0	66.652031	0	66.652031	63.910713	0	0	0	0	0	33.326015	66.652031	0	0

Table S11 Amino acid descriptors (1D) used in this study.

No.	Descriptor ID	Attribute	Ref*	A	C	D	E	F	G	H	I	K	L	M	N	P	Q	R	S	T	V	W	Y
879	std_dim1	Standard dimension 1: the square root of the largest eigenvalue of the covariance matrix of the atomi coordinates. A standard dimension is equivalent to the standard deviation along a principal component axis.	MOE	1.2639022	1.3181921	1.4089293	1.5810752	2.1451728	1.2644597	1.9645185	1.5039384	2.3608639	1.8501447	2.392482	1.7129019	1.415099	2.1361201	2.5107274	1.4151419	1.4450568	1.5898048	2.5781331	2.305696
880	std_dim2	Standard dimension 2: the square root of the second largest eigenvalue of the covariance matrix of th atomic coordinates. A standard dimension is equivalent to the standard deviation along a principal component axis.	MOE	1.1496997	1.1258773	1.1557728	1.2068236	1.3211806	0.8636494	1.2531821	1.3036216	1.1789515	1.1493287	1.0147955	1.0842022	1.1691204	1.0411174	1.4438196	1.099227	1.1631058	1.1311729	1.4915211	1.319312
881	std_dim3	Standard dimension 3: the square root of the third largest eigenvalue of the covariance matrix of th atomic coordinates. A standard dimension is equivalent to the standard deviation along a principal component axis.	MOE	0.671719	0.8981596	0.8398606	0.8923615	0.9666548	0.5558462	0.897703	1.1049594	0.8243896	0.9752343	0.8580151	0.896309	0.8631029	0.8726718	0.6564012	0.6477925	0.8805379	0.9873814	0.8809319	0.9656788
882	TPSA	Polar surface area (Å2) calculated using group contributions to approximate the polar surface area fror connection table information only. The parameterization is that of Ertl et al. [Ertl 2000	MOE	67.769997	106.57	107.9	107.9	67.769997	67.769997	96.449997	67.769997	95.410004	67.769997	67.769997	110.86	56.740002	110.86	131.41	88	88	67.769997	83.559998	88
883	VAdjMa	Vertex adjacency information (magnitude): 1 + log2 m where m is the number of heavy-heavy bonds. If r is zero, then zero is returned	MOE	3.321928	3.5849626	4	4.1699252	4.5849624	3	4.4594316	4	4.1699252	4	4	4	4	4.1699252	4.4594316	3.5849626	3.8073549	3.8073549	5	4.7004399
884	vsa_acc	Approximation to the sum of VDW surface areas (Å2) of pure hydrogen bond acceptors (not countin acidic atoms and atoms that are both hydrogen bond donors and acceptors such as -OH	MOE	27.133842	27.133842	54.267685	54.267685	27.133842	27.133842	27.133842	27.133842	27.133842	27.133842	27.133842	40.700764	27.133842	40.700764	27.133842	27.133842	27.133842	27.133842	27.133842	27.133842
885	vsa_base	Approximation to the sum of VDW surface areas of basic atoms (Å2)	MOE	17.742489	17.742489	17.742489	17.742489	17.742489	17.742489	17.742489	17.742489	35.484978	17.742489	17.742489	17.742489	5.6825762	17.742489	58.910046	17.742489	17.742489	17.742489	17.742489	17.742489
886	vsa_don	Approximation to the sum of VDW surface areas of pure hydrogen bond donors (not counting basi atoms and atoms that are both hydrogen bond donors and acceptors such as -OH) (Å2	MOE	17.742489	17.742489	17.742489	17.742489	17.742489	17.742489	17.742489	17.742489	35.484978	17.742489	17.742489	35.484978	5.6825762	35.484978	58.910046	17.742489	17.742489	17.742489	23.425066	17.742489
887	vsa_hyd	Approximation to the sum of VDW surface areas of hydrophobic atoms (Å2)	MOE	36.108002	24.484108	22.508045	38.31654	103.75906	20.299505	49.507103	83.533485	74.424538	83.533485	91.437729	22.508045	58.616047	38.31654	58.616047	28.758026	44.566521	67.724991	117.15816	96.409081
888	vsa_other	Approximation to the sum of VDW surface areas (Å2) of atoms typed as "other"	MOE	14.708499	46.09301	29.416998	29.416998	14.708499	14.708499	39.499016	14.708499	14.708499	14.708499	14.708499	27.65803	14.708499	27.65803	30.390074	14.708499	14.708499	14.708499	14.708499	14.708499
889	vsa_pol	Approximation to the sum of VDW surface areas (Å2) of polar atoms (atoms that are both hydrogen bon donors and acceptors), such as -OH	MOE	44.876331	44.876331	72.01017	72.01017	44.876331	44.876331	56.241482	44.876331	62.61882	44.876331	44.876331	76.185745	32.816418	76.185745	86.043884	58.443253	58.443253	44.876331	50.558907	58.443253
890	vsurf_A	Amphiphilic moment	MOE	3.6721015	3.7676389	2.2406704	2.4523268	4.8923903	2.6413789	3.4889321	4.5130277	4.3621249	4.5740662	5.7149134	2.7562652	4.6960983	1.5043832	3.5296052	1.7766691	2.3757088	4.6216974	5.4991913	3.7253404
891	vsurf_CP	Critical packing parameter	MOE	0.0552205	0.0561133	0.0250676	0.0345282	0.0947655	0.0429851	0.0609053	0.0903429	0.0614889	0.0731898	0.0918449	0.0307606	0.1434262	0.0339475	0.0695011	0.0286213	0.0331766	0.076972	0.0923609	0.0706744
892	vsurf_CW1	Capacity factor (8 descriptors)	MOE	3.4248042	3.2955275	3.4388947	3.3611701	3.0525806	3.6728156	3.2091298	3.077245	3.1609468	3.0991642	3.06793	3.390666	3.1408224	3.3338892	3.1795855	3.5857399	3.3837023	3.1739295	3.0277629	3.1169608
893	vsurf_CW2	Capacity factor (8 descriptors)	MOE	2.7565348	2.9188068	3.1139355	3.0492618	2.6304135	3.1706705	2.8097086	2.2417338	2.694793	2.2308877	2.3774447	3.0665622	2.3434031	3.03321	2.8359082	3.2036242	2.8995183	2.3858998	2.6778252	2.8203473
894	vsurf_CW3	Capacity factor (8 descriptors)	MOE	2.0491498	2.1666923	2.5451005	2.4528611	1.4411749	2.3506076	1.8673192	1.5357533	1.9417989	1.5549858	1.5008165	2.3300085	1.3464586	2.435483	1.985126	2.617697	2.2698362	1.638258	1.6850369	1.8237
895	vsurf_CW4	Capacity factor (8 descriptors)	MOE	1.2410361	1.1878362	1.7329692	1.5392878	0.8490802	1.4399523	1.1541911	0.9424276	1.0837979	0.961744	0.8668205	1.6085144	0.7409902	1.5066259	1.0416877	1.6898497	1.4964654	0.9847348	0.8731138	1.006951
896	vsurf_CW5	Capacity factor (8 descriptors)	MOE	0.6878935	0.6508936	1.0451936	0.8870574	0.4811191	0.7672885	0.6592016	0.5115678	0.5661371	0.5302794	0.4682475	0.9631407	0.3669246	0.8376591	0.5571612	0.9167538	0.837358	0.5371152	0.4765409	0.5454798
897	vsurf_CW6	Capacity factor (8 descriptors)	MOE	0.2721061	0.2712956	0.5165583	0.4187099	0.1995306	0.3007456	0.2850234	0.2136435	0.2472415	0.2142859	0.1845394	0.4662899	0.1515214	0.3699274	0.258713	0.3711286	0.3473225	0.2177926	0.1903704	0.2027869
898	vsurf_CW7	Capacity factor (8 descriptors)	MOE	0.0763298	0.0780183	0.192601	0.1617393	0.0632919	0.0790604	0.0808908	0.0606427	0.0868241	0.0596098	0.0556331	0.1610459	0.0540904	0.1170513	0.111237	0.0884001	0.0895185	0.0644584	0.0454338	0.056246
899	vsurf_CW8	Capacity factor (8 descriptors)	MOE	0.011474	0.0098103	0.0449503	0.0408942	0.010674	0.0044947	0.0070939	0.0086274	0.0187674	0.0073707	0.0072175	0.0142864	0.0049791	0.0152956	0.0608859	0.0046474	0.0019389	0.0124305	0.0083482	0.0067847
900	vsurf_D1	Contact distances of vsurf_EWmin (3 descriptors)	MOE	190	247.125	218.625	268.25	476.125	147.875	357.375	322.75	334.5	326.75	401.75	231.875	361.625	253	419.25	146	184	284.375	548.75	434.25
901	vsurf_D2	Contact distances of vsurf_EWmin (3 descriptors)	MOE	103.5	128.25	115.625	146.5	264.75	79.5	196.125	173	180.875	171.5	224.5	124.25	188.375	139.625	238.375	79.5	98	152.375	315.25	243.625
902	vsurf_D3	Contact distances of vsurf_EWmin (3 descriptors)	MOE	80.75	90.5	75.875	98.25	180.25	56.5	134.125	131.625	134	132.5	163.375	83.25	138.375	95	164.5	54	70.25	114.375	217.25	165.875
903	vsurf_D4	Contact distances of vsurf_EWmin (3 descriptors)	MOE	65.125	69.5	56.625	75.5	153.25	44	111	115.75	110.375	118.25	142.75	62.875	119.125	72.25	130.625	40.5	52.125	97.75	179.75	133.125
904	vsurf_D5	Contact distances of vsurf_EWmin (3 descriptors)	MOE	51.875	51.75	43.75	59.75	140	31.75	97.75	97	92.5	98.625	123.25	50.5	100	57.625	107.75	29.875	39.25	80.875	152.375	112.125
905	vsurf_D6	Contact distances of vsurf_EWmin (3 descriptors)	MOE	29.25	35.25	34.375	46.5	121.125	23.75	79.625	60.375	72.875	63.375	87.5	40.75	82.5	45.375	88.625	22.125	24.375	54.375	132.5	93.375
906	vsurf_D7	Contact distances of vsurf_EWmin (3 descriptors)	MOE	16.75	18.75	26.375	35.25	73.625	13.875	55.5	23.25	32.875	24.5	57.75	27.625	37.5	32.25	61.5	14.25	13.375	23.625	92.875	69.75
907	vsurf_D8	Contact distances of vsurf_EWmin (3 descriptors)	MOE	7.875	6.75	16.25	23.625	28.25	8.75	27.625	7.875	16.5	6.875	24.25	13.875	15	18.5	32	9	6	9.625	50.75	35.5
908	vsurf_DD12	Contact distances of vsurf_DDmin (3 descriptors)	MOE	0.7071068	0.8660254	1.2247449	0.5	6.9641943	6.5	0.5	0.5	1.8027756	0.7071068	6.6708322	0.5	1.2247449	0.5	0.5	0.5	6.5574384	6.8007355	0.5	6.9641943
909	vsurf_DD13	Contact distances of vsurf_DDmin (3 descriptors)	MOE	0.5	6.7268119	1.118034	0.5	7.0887237	6.5383482	0.5	1.8708287	1.118034	2.2912879	1.118034	0.7071068	0.7071068	1.5	0.7071068	0.5	6.745369	1.118034	0.7071068	6.8373971
910	vsurf_DD23	Contact distances of vsurf_DDmin (3 descriptors)	MOE	0.5	6.9641943	0.5	1	0.5	0.7071068	1	2.2912879	0.7071068	1.6583124	6.98212	0.5	1.8708287	1.2247449	0.5	1	0.7071068	6.7082038	0.5	0.5
911	vsurf_DW12	Contact distances of vsurf_EWmin (3 descriptors)	MOE	0.5	0.5	6.8007355	0.5	0.7071068	0.5	0.5	0.5	0.7071068	0.5	0.7071068	5.0249376	0.5	10.735456	0.5	0.5	0.8660254	0.7071068	0.5	0.5
912	vsurf_DW13	Contact distances of vsurf_EWmin (3 descriptors)	MOE	0.7071068	0.7071068	0.7071068	0.7071068	0.5	0.5	0.5	0.8660254	0.5	0.5	0.5	0.5	0.5	0.5	0.7071068	0.5	0.7071068	0.7071068	0.5	1
913	vsurf_DW23	Contact distances of vsurf_EWmin (3 descriptors)	MOE	0.5	0.8660254	7.1589108	0.5	0.8660254	1	1	0.7071068	0.5	0.7071068	0.5	4.8476801	0.7071068	10.793516	0.5	0.7071068	0.5	0.7071068	0.7071068	0.5
914	vsurf_EDmin1	Lowest hydrophobic energy (3 descriptors	MOE	-1.929827	-2.156132	-2.642678	-2.5816734	-2.2426505	-1.813659	-2.6442056	-1.9527595	-2.1130974	-1.8615128	-2.1951571	-2.6121171	-2.1275668	-2.0891349	-2.9354012	-2.0650859	-1.9657583	-1.9606552	-2.5392506	-2.2485213
915	vsurf_EDmin2	Lowest hydrophobic energy (3 descriptors	MOE	-1.9166979	-1.9105738	-2.5898318	-2.5665219	-2.1847408	-1.8113465	-2.6269531	-1.8923414	-2.0083787	-1.8487463	-2.121371	-2.5627165	-2.0915151	-2.0503764	-2.871573	-2.0615828	-1.9033506	-1.9486187	-2.5385182	-2.2476006
916	vsurf_EDmin3	Lowest hydrophobic energy (3 descriptors	MOE	-1.9035559	-1.8947275	-2.5497856	-2.5220451	-2.1546416	-1.8109114	-2.5527077	-1.8818467	-1.9885008	-1.8472666	-2.1193478	-2.4937985	-2.0857127	-2.0501132	-2.7994165	-2.0407784	-1.8953888	-1.8965871	-2.4961312	-2.219568
917	vsurf_EWmin1	Lowest hydrophilic energy (3 descriptors	MOE	-6.3190322	-6.4538536	-6.9587255	-7.0005994	-6.4505386	-6.3943577	-6.3452306	-6.4554691	-7.5683594	-6.4870186	-6.3814273	-6.442255	-6.3608994	-6.3902888	-9.1530809	-6.3181949	-6.189672	-6.4683208	-6.3357806	-6.3278689
918	vsurf_EWmin2	Lowest hydrophilic energy (3 descriptors	MOE	-6.2075033	-6.3090105	-6.8508482	-6.982841	-6.2939882	-6.3927712	-6.2659345	-6.391624	-7.0114608	-6.4064031	-6.2970629	-6.4161501	-6.0963936	-6.3703442	-9.1298199	-6.150836	-6.099391	-6.4028602	-6.3278856	-6.3236852
919	vsurf_EWmin3	Lowest hydrophilic energy (3 descriptors	MOE	-6.1626616	-6.2160001	-6.7902331	-6.9083457	-6.1922989	-5.9946656	-6.2130375	-6.3383279	-6.8077021	-6.3684154	-6.248589	-6.3625646	-6.0663266	-6.3021135	-8.9333773	-6.0287685	-5.9907207	-6.3664498	-6.2513776	-6.0576415
920	vsurf_G	Surface globularity	MOE	1.1213403	1.1390152	1.1539135	1.1604484	1.1554586	1.1157482	1.1526961	1.1448913	1.1943517	1.1495376	1.1800582	1.148249	1.1231549	1.2017094	1.177237	1.1348213	1.1376181	1.1306418	1.1929653	1.1748623
921	vsurf_HB1	H-bond donor capacity (8 descriptors	MOE	213.875	221.25	331.75	330.125	229.875	219	224	207	190.375	208.875	200.125	214.5	260.125	272.25	124.375	258.875	257.625	197.375	241.5	277.125
922	vsurf_HB2	H-bond donor capacity (8 descriptors	MOE	248.625	284.375	429.875	447.875	274.625	257.625	271.625	242.875	213.75	239	237.125	261.375	309.625	355.125	126.25	351.5	340.5	232.75	282.625	337
923	vsurf_HB3	H-bond donor capacity (8 descriptors	MOE	182.875	210.75	345.75	344.125	190.625	181.25	216.375	185.625	175.125	181.875	176.875	239.625	181.875	289.125	133.375	262.375	258.375	173	206.5	244
924	vsurf_HB4	H-bond donor capacity (8 descriptors	MOE	90.875	104	185.625	171.5	96.125	94.5	123	97	93.375	94.375	88.125	150.5	74.125	144.75	81.25	134.75	133.25	88.125	99.125	118.5
925	vsurf_HB5	H-bond donor capacity (8 descriptors	MOE	42.375	46.875	92.375	82.75	46.625	42.375	55.875	44	45.125	43.75	41.25	76.875	31.125	65.5	39.5	60.375	61.875	40.625	44.375	51.25
926	vsurf_HB6	H-bond donor capacity (8 descriptors	MOE	15.875	19.5	39.375	34.875	17.125	15.125	22.25	17.25	20.375	16.5	14.375	33.75	10.875	25.875	19.25	23	23.25	16.125	18.5	19.375
927	vsurf_HB7	H-bond donor capacity (8 descriptors	MOE	4.375	5	13.875	12.5	5.375	3.5	6.75	5.125	7.625	4.75	4.625	10.125	3.375	6.75	10	5.25	6	4.625	4.25	5.25
928	vsurf_HB8	H-bond donor capacity (8 descriptors	MOE	0.75	0.625	2.75	3	1	0.25	0.875	0.5	1.375	0.5	0.625	1.25	0.375	1.5	5.625	0.375	0.25	0.875	0.875	0.625
929	vsurf_HL1	Hydrophilic-Lipophilic (2 descriptors	MOE	0.6006192	0.5690608	1.2570016	0.8778626	0.2857143	0.8495575	0.4622554	0.3646724	0.4412313	0.3745283	0.289977	1.0345346	0.2249323	0.8973684	0.3799392	1.2314814	0.9661922	0.4087432	0.2577676	0.3526752
930	vsurf_HL2	Hydrophilic-Lipophilic (2 descriptors	MOE	0.2571977	0.2841727	0.7041943	0.4685431	0.1150082	0.3551136	0.2094595	0.150108	0.200453	0.1437632	0.1085814	0.5566601	0.0912907	0.449827	0.1990431	0.5925926	0.4580336	0.168798	0.1119611	0.1502347
931	vsurf_ID1	Hydrophobic integy moment (8 descriptors	MOE	1.2927741	1.1196035	1.0206002	1.1435314	1.7829218	1.0751889	1.3756183	1.613935	1.4980714	1.6918355	1.7880979	1.4173808	1.3724703	0.7777348	1.2360463	0.4479331	0.8404589	1.6153654	1.7831736	1.2325596
932	vsurf_ID2	Hydrophobic integy moment (8 descriptors	MOE	1.3640095	1.1694888	1.0104548	1.072763	1.6458893	1.0119992	1.3366879	1.5410742	1.3713907	1.6375362	1.7245948	1.4223287	1.241779	0.8137189	1.1763191	0.5659372	0.9131677	1.5774335	1.7098789	1.2767007
933	vsurf_ID3	Hydrophobic integy moment (8 descriptors	MOE	1.6566482	1.3239694	1.0208961	1.0343672	1.678586	1.1871421	1.3213913	1.759667	1.5687484	1.9752886	1.967576	1.440277	1.3408631	0.8249983	1.1865888	0.7625102	1.1658002	1.8274109	1.733384	1.2031568
934	vsurf_ID4	Hydrophobic integy moment (8 descriptors	MOE	1.9830475	1.3866696	1.1095145	1.144694	1.9349284	1.3644598	1.3786986	2.0002747	1.9003854	2.3462105	2.2615335	1.5972356	1.6787529	0.9192711	1.3737966	0.7143201	1.4889268	2.1310282	1.9846854	1.2667886
935	vsurf_ID5	Hydrophobic integy moment (8 descriptors	MOE	1.9575889	1.3603638	1.2328238	1.1428097	2.0587223	1.2752111	1.3954785	2.2379503	1.9498377	2.5320442	2.4440637	1.6137092	1.9398726	0.9081969	1.4738936	0.3940133	1.5040034	2.3357728	2.2530732	1.3051606
936	vsurf_ID6	Hydrophobic integy moment (8 descriptors	MOE	1.0684894	1.3433622	1.3799032	1.1980938	2.2171681	1.2574841	1.4981276	1.9730189	1.944307	1.8872706	1.9131247	1.7201328	2.0258739	0.9460418	1.5026221	0.4020748	0.7305692	1.9027239	2.3955822	1.3740791
937	vsurf_ID7	Hydrophobic integy moment (8 descriptors	MOE	0.6399929	1.7353233	1.285382	0.9794345	1.6751512	0.7066117	1.149026	1.2054232	0.2917835	1.8509108	1.5677818	1.5205214	1.3482927	0.8206695	1.0604022	0.7714148	0.5319948	1.3171892	1.8212439	1.423198
938	vsurf_ID8	Hydrophobic integy moment (8 descriptors	MOE	1.0316106	2.6030066	0.7734949	0.3263629	1.5198798	0.7776544	0.8334908	2.3190043	1.4521961	2.8268471	0.6563757	0.8384204	1.0443016	0.3702341	0.775374	1.3180709	1.3541106	1.8769691	1.3189813	1.4715383
939	vsurf_IW1	Hydrophilic integy moment (8 descriptors	MOE	0.4326166	0.5292854	0.3455172	0.3098901	0.4688822	0.1559261	0.3562233	0.5987728	0.3255429	0.5805813	0.8218076	0.3497057	0.7516017	0.1770994	0.3451845	0.065065	0.2412233	0.629606	0.4588686	0.3365589
940	vsurf_IW2	Hydrophilic integy moment (8 descriptors	MOE	0.8752698	0.7861584	0.5108265	0.4974263	1.4377763	0.3841011	0.7752194	1.5452375	0.9074929	1.5899732	2.0197802	0.5726161	1.9256111	0.2253088	0.7882624	0.177291	0.4138951	1.4951274	1.34886	0.7256267
941	vsurf_IW3	Hydrophilic integy moment (8 descriptors	MOE	1.2279043	1.2565292	0.7637758	0.810232	2.4990053	0.7096193	1.3078355	1.9862237	1.4116328	1.9488422	2.8870678	0.8799177	2.709094	0.3659446	1.3620603	0.3901218	0.6568684	1.9979221	2.4021292	1.4806267
942	vsurf_IW4	Hydrophilic integy moment (8 descriptors	MOE	1.6226574	2.0240901	1.0287898	1.1315622	2.9857814	1.0828966	1.7702838	2.3863366	2.1464188	2.3104031	3.3983054	1.1321228	3.0212338	0.5188791	1.9808642	0.7013703	0.990364	2.4330013	3.2607839	2.1031635
943	vsurf_IW5	Hydrophilic integy moment (8 descriptors	MOE	2.0377011	2.445456	1.2545469	1.4321584	3.2966192	1.5001767	2.1815982	2.8050604	2.8349819	2.665283	3.7794752	1.3760344	3.3723288	0.80454	2.4424102	1.0304607	1.3175149	2.8258581	3.7824566	2.5425105
944	vsurf_IW6	Hydrophilic integy moment (8 descriptors	MOE	2.7068658	2.9734089	1.4418916	1.7995622	3.8250175	2.2601917	2.6417749	3.2654994	3.2891526	3.3057268	4.3790202	1.6284567	4.1683488	1.1957053	3.2615957	1.664633	2.1396432	3.4248598	4.6734853	3.5014472
945	vsurf_IW7	Hydrophilic integy moment (8 descriptors	MOE	3.8601434	3.8500714	2.1648998	2.312068	4.530962	3.7949252	3.938885	4.4163656	3.264802	4.2615891	5.1233153	1.865643	4.500124	1.9681987	3.809953	3.6520517	3.5220408	4.3450003	6.3132286	4.7336326
946	vsurf_IW8	Hydrophilic integy moment (8 descriptors	MOE	4.0599051	4.4307504	2.6095479	3.0355508	4.9293504	3.7193062	4.3176923	4.6171966	2.9747627	4.5581818	5.1766071	2.3648663	4.4705968	2.0663614	4.0062418	4.0128999	4.1603022	4.392138	6.5663214	5.0919747
947	vsurf_W1	Hydrophilic volume (8 descriptors	MOE	644.875	713.625	853.375	899.5	866.125	638.75	862.875	726.875	892.5	743.875	802.875	831.75	650	958.125	950.25	750.375	755.625	695.375	1023.625	955.25
948	vsurf_W2	Hydrophilic volume (8 descriptors	MOE	444.875	502.625	638.625	663.25	524.625	454.375	572	451	602.5	457.25	488.375	611.25	366.5	715.75	636.625	565.25	551	437.875	655.625	636.875
949	vsurf_W3	Hydrophilic volume (8 descriptors	MOE	257	290.25	403.25	405.5	264.375	260.625	321.625	254.125	343.5	260.625	257	378.75	181.875	435.375	353.125	344.125	332.75	245.875	342.75	335.25
950	vsurf_W4	Hydrophilic volume (8 descriptors	MOE	111.625	121.25	197	185.75	113	115.375	146.375	112.125	142.75	114.625	110.125	184.625	74.125	195.375	144.75	155.875	152.625	108.25	134.5	140.5
951	vsurf_W5	Hydrophilic volume (8 descriptors	MOE	48.5	51.5	95.375	86.25	51.5	48	62	48	59.125	49.625	47.375	86.125	31.125	85.25	62.5	66.5	67.875	46.75	56	58.5
952	vsurf_W6	Hydrophilic volume (8 descriptors	MOE	16.75	19.75	39.875	35.375	17.625	15.625	23.25	17.375	22.125	17	15.5	35	10.875	32.5	26	24	23.875	16.5	20.125	20
953	vsurf_W7	Hydrophilic volume (8 descriptors	MOE	4.375	5	13.875	12.5	5.375	3.5	6.75	5.125	7.625	4.75	4.625	10.125	3.375	8.5	11.25	5.25	6	4.625	4.25	5.25
954	vsurf_W8	Hydrophilic volume (8 descriptors	MOE	0.75	0.625	2.75	3	1	0.25	0.875	0.5	1.375	0.5	0.625	1.25	0.375	1.5	5.625	0.375	0.25	0.875	0.875	0.625
955	vsurf_Wp1	Polar volume (8 descriptors)	MOE	431	492.375	521.625	569.375	636.25	419.75	638.875	519.875	702.125	535	602.75	617.25	389.875	685.875	825.875	491.5	498	498	782.125	678.125
956	vsurf_Wp2	Polar volume (8 descriptors)	MOE	196.25	218.25	208.75	215.375	250	196.75	300.375	208.125	388.75	218.25	251.25	349.875	56.875	360.625	510.375	213.75	210.5	205.125	373	299.875
957	vsurf_Wp3	Polar volume (8 descriptors)	MOE	74.125	79.5	57.5	61.375	73.75	79.375	105.25	68.5	168.375	78.75	80.125	139.125	0	146.25	219.75	81.75	74.375	72.875	136.25	91.25
958	vsurf_Wp4	Polar volume (8 descriptors)	MOE	20.75	17.25	11.375	14.25	16.875	20.875	23.375	15.125	49.375	20.25	22	34.125	0	50.625	63.5	21.125	19.375	20.125	35.375	22
959	vsurf_Wp5	Polar volume (8 descriptors)	MOE	6.125	4.625	3	3.5	4.875	5.625	6.125	4	14	5.875	6.125	9.25	0	19.75	23	6.125	6	6.125	11.625	7.25
960	vsurf_Wp6	Polar volume (8 descriptors)	MOE	0.875	0.25	0.5	0.5	0.5	0.5	1	0.125	1.75	0.5	1.125	1.25	0	6.625	6.75	1	0.625	0.375	1.625	0.625
961	vsurf_Wp7	Polar volume (8 descriptors)	MOE	0	0	0	0	0	0	0	0	0	0	0	0	0	1.75	1.25	0	0	0	0	0
962	weinerPath	Weiner path number	MOE	29	46	96	136	212	18	165	92	143	96	102	96	62	136	247	46	65	65	369	268
963	weinerPol	Weiner polarity number	MOE	4	6	8	9	13	2	10	10	9	8	8	8	6	9	11	6	8	8	20	15
964	zagreb	Zagreb index: the sum of di2 over all heavy atoms i	MOE	22	26	36	40	54	16	50	36	38	36	34	36	36	40	48	26	32	32	76	60

* AAindex: from the amino acid index database, http://www.genome.jp/aaindex/; Jpred 3: predicted by the web site, http://www.com/pbio.dundee.ac.uk/www-jpred/; MOE: calculated for 20 aas by the molecular descriptor calculation of the MOE software Ver.2013.08; all missing values were replaced by the average values.

Table S12 Amino acid descriptors (2D) used in this study.

			1st aa	A	A	A	A	A	A	A	A	A	A	A	A	A	A	A	A	A	A	A	A	C	C	C	C	C	C	C	C	C	C	C	C	C	C	C	C	C	C	C
			2nd aa	A	C	D	E	F	G	H	I	K	L	M	N	P	Q	R	S	T	V	W	Y	A	C	D	E	F	G	H	I	K	L	M	N	P	Q	R	S	T	V	W
No.	Descriptor ID	Attribute	Ref*	AA	AC	AD	AE	AF	AG	AH	AI	AK	AL	AM	AN	AP	AQ	AR	AS	AT	AV	AW	AY	CA	CC	CD	CE	CF	CG	CH	CI	CK	CL	CM	CN	CP	CQ	CR	CS	CT	CV	CW
1	ALTS910101	The PAM-120 matrix (Altschul, 1991)	AAindex	3	-3	0	6	-7	1	-2	0	3	1	-5	0	6	1	-3	0	-2	1	-5	-2	-3	1	-4	1	-1	-1	0	-2	-5	-3	-5	-2	-3	0	-2	-7	1	-3	-8
2	BENS940101	Log-odds scoring matrix collected in 6.4-8.7 PAM (Benner et al., 1994)	AAindex	2.5	-1.3	-0.7	5.1	-2.8	1.8	-1.3	0.1	4.4	1.2	-2.3	-0.6	5.3	1.4	-1.7	-0.7	-1.6	2.4	-5.7	0.9	-1.3	1.1	-3.2	2.5	0.9	-3	0	-2.5	-5.3	-2.4	-1.6	-0.2	-3.8	0.7	-1.9	-4.7	0.8	-2	-1.7
3	BENS940102	Log-odds scoring matrix collected in 22-29 PAM (Benner et al., 1994)	AAindex	2.5	-1.7	-0.3	5	-3.3	1.5	-0.7	0.5	3.9	1.1	-3	-0.2	4.2	1.3	-1.2	-0.3	-0.9	2.7	-5.8	0.8	-1.7	0.8	-3.1	2.2	0.3	-2.1	0	-2.7	-4.9	-2.3	-1.9	-0.8	-2.7	0.4	-1	-4.3	0.8	-2.3	-4.5
4	BENS940103	Log-odds scoring matrix collected in 74-100 PAM (Benner et al., 1994)	AAindex	2.4	-1.4	-0.1	4.8	-2.9	1	-0.3	0.7	2.9	0.9	-2.8	-0.3	3	1.1	-0.8	-0.1	-0.2	2.8	-5.4	0.6	-1.4	0.4	-2.6	1.6	0.1	-1.8	-0.2	-2.8	-4.2	-2.2	-1.8	-0.8	-2	0.1	-0.4	-3.2	0.6	-2.6	-4.1
5	BENS940104	Genetic code matrix (Benner et al., 1994)	AAindex	4	-2.3	1.3	2.9	-3.2	3.6	-0.6	0.4	3.8	-0.3	2.3	1	5.5	0.1	-1.6	1.3	-2.1	1.2	-1.9	0.6	-2.3	0.8	-2.4	0.3	1.5	-0.4	-1.7	0.9	-2.4	-2.2	-1.5	-2	-1.9	1	-1.9	-3	1.2	-1.6	1.4
6	CSEM940101	Residue replace ability matrix (Cserzo et al., 1994)	AAindex	1	0.2	0.08	1	-0.43	0.13	-0.32	0.2	0.37	0.14	-0.34	-0.08	1	0.14	-0.07	0.08	-0.27	0.6	-0.09	-0.09	0.2	-0.07	-0.25	0.57	0.1	-0.17	-0.14	-0.37	-0.55	-0.46	-0.14	0.15	-0.43	0.06	-0.07	-0.51	0.11	-0.19	-0.08
7	DAYM780301	Log odds matrix for 250 PAMs (Dayhoff et al., 1978)RA Dayhoff, M.O., Schwartz, R.M. and Orcutt, B.C.T A model of evolutionary change in proteins	AAindex	2	-2	0	6	-5	2	-1	1	3	1	-4	0	4	1	-2	0	0	2	-5	0	-2	1	-4	1	0	0	0	-2	-4	-2	-4	-1	-2	0	-1	-5	1	-2	-7
8	FEND850101	Structure-Genetic matrix (Feng et al., 1985)	AAindex	6	2	4	6	0	4	3	3	5	5	2	4	6	5	2	4	3	5	1	3	2	5	2	3	4	2	3	2	1	2	2	2	1	5	3	0	5	2	3
9	FITW660101	Mutation values for the interconversion of amino acid pairs (Fitch, 1966)	AAindex	0	2	1	0	3	1	1	2	1	1	1	1	0	1	2	1	2	1	2	2	2	1	2	1	1	1	2	1	2	2	2	2	3	1	2	3	1	2	1
10	GEOD900101	Hydrophobicity scoring matrix (George et al., 1990)	AAindex	10	8	5	10	4	5	5	10	10	10	3	5	10	9	5	5	5	10	6	5	8	9	7	6	8	3	9	7	3	7	8	9	7	8	5	5	9	9	5
11	GONG920101	The mutation matrix for initially aligning (Gonnet et al., 1992)	AAindex	2.4	-1.2	0	4.7	-2.8	0.6	-0.2	0.7	2.7	0.9	-2.8	-0.3	2.7	1.1	-0.6	0	-0.1	2.8	-5.2	0.6	-1.2	0.3	-2.3	1.5	0.1	-1.7	-0.3	-2.8	-4	-2.2	-1.8	-0.7	-1.9	0.1	-0.4	-3	0.5	-2.6	-4
12	GRAR740104	Chemical distance (Grantham, 1974)	AAindex	0	96	107	0	202	29	71	46	45	46	160	126	0	99	112	107	65	5	153	32	96	27	113	43	112	91	111	149	172	133	139	84	109	64	106	170	60	95	184
13	HENS920101	BLOSUM45 substitution matrix (Henikoff-Henikoff, 1992)	AAindex	5	-1	-1	7	-3	0	-1	0	2	1	-2	-2	6	1	-2	-1	-1	2	-3	-1	-1	-1	-2	1	-1	-1	-1	-2	-3	-3	-2	-1	-2	0	-1	-3	0	-2	-2
14	HENS920102	BLOSUM62 substitution matrix (Henikoff-Henikoff, 1992)	AAindex	6	-2	-1	8	-5	0	-2	0	2	1	-5	-3	8	2	-2	-1	-1	2	-5	-1	-2	-1	-3	1	-1	-2	-2	-5	-5	-4	-4	-1	-4	0	-1	-5	0	-4	-4
15	HENS920103	BLOSUM80 substitution matrix (Henikoff-Henikoff, 1992)	AAindex	7	-3	-2	9	-6	0	-2	0	2	1	-6	-3	9	2	-3	-2	-2	2	-6	-1	-3	-1	-4	1	-2	-3	-3	-6	-7	-5	-5	-2	-5	-1	-1	-7	0	-5	-6
16	JOHM930101	Structure-based amino acid scoring table (Johnson-Overington, 1993)	AAindex	6	-3.3	-0.7	10	-8.7	0.1	-1.4	-0.8	2.4	1	-3.8	-1.6	9	0	-1.6	-0.7	-0.5	2.6	-8.6	0.1	-3.3	-1	-3.2	2.1	-7.7	-4.2	-1.4	-4.7	-8	-5.7	-7.6	-1.5	-7	-0.5	-0.9	-6.9	-0.5	-5.7	-6.3
17	JOND920103	The 250 PAM PET91 matrix (Jones et al., 1992)	AAindex	2	-1	-1	5	-3	2	-1	0	4	1	-2	0	5	1	-1	-1	-1	2	-5	1	-1	1	-3	2	1	-2	0	-2	-4	-2	-1	-1	-3	1	-1	-4	1	-2	-2
18	JOND940101	The 250 PAM transmembrane protein exchange matrix (Jones et al., 1994)	AAindex	2	-2	0	7	-3	5	-1	3	8	2	-2	0	11	2	-1	0	-1	1	-4	4	-2	0	-2	6	1	0	-1	-3	-5	-3	-1	-1	-4	0	-2	-3	1	-3	-2
19	KOLA920101	Conformational similarity weight matrix (Kolaskar-Kulkarni-Kale, 1992)	AAindex	10	5	9	10	0	5	5	0	0	6.6	0	0	10	0	6.6	9	0	0	0	0	5	0	0	9	9	9	0	0	0	0	0	5	0	0	6.6	0	0	0	0
20	LEVJ860101	The secondary structure similarity matrix (Levin et al., 1986)	AAindex	2	0	1	2	0	0	0	1	1	0	-1	0	2	1	0	1	0	0	-1	0	0	-1	-1	0	0	-1	0	-1	-1	-1	0	0	-1	0	0	0	0	-1	-1
21	LUTR910101	Structure-based comparison table for outside other class (Luthy et al., 1991)	AAindex	11	1	3	32	-16	-1	-1	3	6	3	-10	1	16	4	0	3	1	19	-24	-8	1	1	-13	6	-10	0	2	-3	-10	-3	-7	1	-3	3	-1	-12	-5	-11	-12
22	LUTR910102	Structure-based comparison table for inside other class (Luthy et al., 1991)	AAindex	12	-10	7	9	-22	5	6	6	12	8	0	6	7	9	5	7	10	9	-52	6	-10	-5	-24	6	-11	-6	5	-3	-16	-3	-11	-6	-4	0	6	-16	5	-28	-23
23	LUTR910103	Structure-based comparison table for outside alpha class (Luthy et al., 1991)	AAindex	24	-18	-2	18	-4	0	2	2	6	4	-14	-2	13	1	-2	-2	0	13	-21	-6	-18	-2	-20	2	5	0	-1	-6	-18	-6	1	-3	-4	-5	-10	-3	1	-8	-21
24	LUTR910104	Structure-based comparison table for inside alpha class (Luthy et al., 1991)	AAindex	22	-14	4	38	-2	4	3	4	20	4	-8	-1	6	9	-2	4	4	0	-25	-9	-14	5	-18	9	10	2	4	0	-20	-1	4	-2	0	-1	1	-2	5	1	-57
25	LUTR910105	Structure-based comparison table for outside beta class (Luthy et al., 1991)	AAindex	27	-7	-3	32	-32	-7	2	1	10	0	-13	-4	30	5	-3	-3	0	16	-10	-19	-7	6	-4	2	6	2	-2	-8	-21	-9	6	2	-7	-2	-6	-30	9	8	-33
26	LUTR910106	Structure-based comparison table for inside beta class (Luthy et al., 1991)	AAindex	18	-6	8	60	2	11	10	18	29	13	7	4	42	12	1	8	9	11	-17	14	-6	4	-12	24	-1	3	6	-14	-27	-17	5	1	-14	-7	4	-1	14	-9	-32
27	LUTR910107	Structure-based comparison table for other class (Luthy et al., 1991)	AAindex	11	-3	3	28	-19	0	0	4	8	4	-7	2	14	4	1	3	2	19	-30	-6	-3	0	-16	7	-11	-2	2	-4	-14	-4	-9	-1	-4	2	1	-15	-4	-15	-15
28	LUTR910108	Structure-based comparison table for alpha helix class (Luthy et al., 1991)	AAindex	23	-19	0	19	-6	2	2	5	10	5	-12	0	11	3	-1	0	2	7	-26	-2	-19	1	-23	5	7	-2	1	-7	-21	-8	1	-4	-6	-6	-6	-5	2	-7	-46
29	LUTR910109	Structure-based comparison table for beta strand class (Luthy et al., 1991)	AAindex	23	-7	0	40	-23	2	6	7	17	6	-5	-1	31	7	-2	0	4	13	-15	-1	-7	5	-9	9	-10	2	1	-12	-22	-12	-5	1	-13	-5	-3	-21	12	-1	-33
30	MCLA710101	The similarity of pairs of amino acids (McLachlan, 1971)	AAindex	8	2	4	8	0	5	3	4	5	5	1	3	8	4	2	4	4	5	0	4	2	4	1	5	2	1	3	1	1	1	1	3	0	3	3	0	3	1	1
31	MCLA720101	Chemical similarity scores (McLachlan, 1972)	AAindex	5	0	0	6	0	0	0	2	2	1	0	0	5	1	0	0	0	3	0	0	0	1	0	0	2	0	0	0	0	0	0	0	0	0	0	0	3	0	0
32	MIYS930101	Base-substitution-protein-stability matrix (Miyazawa-Jernigan, 1993)	AAindex	0.34	-0.45	0.03	0.65	-0.81	0.11	0	0.2	0.32	-0.02	0.09	0.04	0.48	0.16	-0.08	0.03	-0.09	0.48	-0.52	0.14	-0.45	0.17	-0.47	0.15	-0.2	-0.83	-0.16	-0.83	-0.75	-0.69	-0.46	-0.47	-0.95	-0.06	-0.2	-0.64	0.19	-0.78	-0.15
33	MIYT790101	Amino acid pair distance (Miyata et al., 1979)	AAindex	0	2.76	2.46	0	3.27	0.82	2.03	0.99	0.9	1.31	3.95	2.37	0	0.51	2.92	2.46	1.83	0.14	4.14	0.79	2.76	0.06	3.23	1.13	1.84	2.29	1.78	3.37	4.1	2.76	2.83	2.42	2.57	1.85	2.96	3.26	0.91	2.62	5.13
34	MOHR870101	EMPAR matrix (Mohana Rao, 1987)	AAindex	16	11	10	16	9	10	9	11	11	11	7	9	16	10	8	10	8	10	7	11	11	6	10	10	10	6	9	5	6	5	9	11	6	9	10	9	8	3	8
35	NIEK910101	Structure-derived correlation matrix 1 (Niefind-Schomburg, 1991)	AAindex	1	0.51	0.7	1	-0.25	0.15	-0.12	-0.14	0.07	0.04	-0.19	0.05	1	-0.38	0.09	0.7	-0.5	0.25	-0.05	-0.23	0.51	0.1	-0.18	0.05	0.31	-0.1	-0.29	-0.06	0.01	-0.15	0.04	0.39	-0.13	-0.23	0.55	-0.34	-0.4	-0.53	-0.1
36	NIEK910102	Structure-derived correlation matrix 2 (Niefind-Schomburg, 1991)	AAindex	1	0.54	0.74	1	-0.31	0.19	-0.12	-0.16	0.09	0.06	-0.22	0.07	1	-0.4	0.11	0.74	-0.54	0.27	-0.05	-0.26	0.54	0.11	-0.19	0.1	0.35	-0.09	-0.31	-0.07	0.01	-0.16	0.04	0.43	-0.15	-0.25	0.6	-0.36	-0.41	-0.55	-0.11
37	OVEJ920101	STR matrix from structure-based alignments (Overington et al., 1992)	AAindex	4	-2	0	7	-4	0	-1	0	2	0	-3	-1	6	0	-1	0	0	2	-6	0	-2	-1	-3	1	-4	-4	-1	-3	-6	-4	-6	0	-5	0	-1	-3	0	-4	-4
38	QU_C930101	Cross-correlation coefficients of preference factors main chain (Qu et al., 1993)	AAindex	1	0.555	0.3	1	0.054	0.093	0.037	0.082	0.137	-0.051	0.32	0.299	1	0.193	0.39	0.3	0.22	0.57	0.455	0.072	0.555	0.183	0.628	-0.084	-0.178	0.374	0.37	0.452	0.261	0.512	0.105	0.207	0.372	0.565	0.398	0.341	0.407	0.065	0.136
39	QU_C930102	Cross-correlation coefficients of preference factors side chain (Qu et al., 1993)	AAindex	1	0.862	-0.116	1	0.002	0.392	0.427	0.222	0.851	0.661	0.225	-0.018	1	0.281	0.25	-0.116	0.287	0.96	0	0.488	0.862	0.656	0.874	0.243	0.104	0.058	0.138	-0.101	-0.249	-0.203	0.032	0.858	0.511	0.85	0.281	-0.167	0	0.572	0
40	QU_C930103	The mutant distance based on spatial preference factor (Qu et al., 1993)	AAindex	8	5	0	8	0	2	2	1	4	3	1	0	8	2	2	0	3	6	0	3	5	3	5	0	1	0	1	0	0	0	0	5	2	5	2	0	2	3	-1
41	RISJ880101	Scoring matrix (Risler et al., 1988)	AAindex	2.2	1.3	1.7	2.2	-1.6	-0.4	1.9	1.6	1	1.9	-0.4	0.2	2.2	2	1.5	1.7	1.6	2.1	-0.4	-1	1.3	-0.2	0.6	2	-1.3	1.1	1.3	0.9	-0.2	1.1	-1.6	1	1.4	2	1.4	-1.5	0.6	-0.6	-1.3
42	TUDE900101	isomorphicity of replacements (Tudos et al., 1990)	AAindex	100	-6	12	100	-14	0	-5	12	34	1	-22	3	100	10	-2	12	-12	46	-12	-4	-6	-2	-8	26	-6	0	-8	-32	-40	-30	-7	-2	-20	-12	2	-18	-3	-18	-13
43	AZAE970101	The single residue substitution matrix from interchanges of spatially neighbouring residues (Azarya-Sprinzak et al., 1997)	AAindex	14	-2	2	16	-11	4	5	13	15	7	-4	0	21	1	1	2	3	8	-4	3	-2	2	-4	16	-1	-1	1	-11	-11	-11	-4	2	-12	-5	1	-8	5	-14	-1
44	AZAE970102	The substitution matrix derived from spatially conserved motifs (Azarya-Sprinzak et al., 1997)	AAindex	8	0	1	11	-10	2	2	10	10	6	-5	0	17	0	1	1	1	8	-3	2	0	-1	-2	12	-2	-2	0	-11	-10	-11	-5	1	-12	-4	1	-6	1	-9	-1
45	RIER950101	Hydrophobicity scoring matrix (Riek et al., 1995)	AAindex	100	93	39	100	33	42	16	96	94	66	38	33	100	94	13	39	41	98	83	64	93	89	87	49	91	2	60	51	25	54	58	89	55	94	35	36	96	79	94
46	WEIL970101	WAC matrix constructed from amino acid comparative profiles (Wei et al., 1997)	AAindex	4	0	-3	4	-2	0	0	1	0	1	-3	-2	4	0	-1	-3	-1	1	-2	0	0	0	0	0	0	0	0	-1	-3	-1	0	1	0	1	-1	-2	-1	0	-2
47	WEIL970102	Difference matrix obtained by subtracting the BLOSUM62 from the WAC matrix (Wei et al., 1997)	AAindex	0	1	-2	-1	1	0	1	1	-2	0	0	0	-1	-1	0	-2	0	-1	1	1	1	1	2	-1	1	1	2	2	1	2	3	2	3	1	0	2	-1	3	0
48	MEHP950101	(Mehta et al., 1995)	AAindex	1.23	1.19	1.34	1.06	1.31	1.06	0.88	1.2	1.31	1.01	0.73	1.31	1.16	1.17	1.17	1.34	0.94	0.99	0.68	1.1	1.19	0.94	0.94	1.1	1.33	0.97	1.28	0.89	1	0.96	1.36	1.2	1.02	1.09	1.29	1.43	1.11	0.67	0.58
49	MEHP950102	(Mehta et al., 1995)	AAindex	0.75	0.97	0.63	0.9	0.79	0.92	1.08	0.71	0.58	0.84	1.12	0.56	0.8	0.8	0.8	0.63	1.11	1.21	1.3	0.82	0.97	0.77	1.16	0.83	0.81	1.14	0.71	1.12	0.96	1.05	0.72	0.97	1.1	1.05	0.65	0.69	0.83	1.3	1.28
50	MEHP950103	(Mehta et al., 1995)	AAindex	0.86	0.57	0.96	1.06	0.68	1.02	1.13	1.13	1.17	1.35	1.46	1.23	0.96	1.04	1.02	0.96	0.89 -		1.16	1.15	0.57	1.69	0.79	1.13	0.55	0.74	0.95	1	1.09	0.99	0.7	0.53	0.7	0.65	1.06	0.57	1.11	1.18	1.46
51	KAPO950101	(Kapp et al., 1995)	AAindex	23.4	13.6	18.1	29.5	9.83	13.7	20.6	18.6	22.8	20.9	14.9	18.2	96.5	20.4	16.8	18.1	18.8	21	9.84	15.5	13.6	17.7	11.9	19.6	17.7	15.5	17.1	14.8	12.5	14.8	14.7	16.2	15.8	16.7	17.1	12.2	19.9	9.35	7.2
52	VOGG950101	(Vogt et al., 1995)	AAindex	7.6	4	5.2	9.9	2.4	5.8	5	5.9	7.9	6.1	2.4	4.9	7.9	6.3	4.6	5.2	5.1	8	0	5.8	4	5.5	2.9	6.7	5.3	3.5	4.9	2.4	1.2	3	3.4	4.5	3.3	5.3	4.8	2.2	5.7	2.6	1.2
53	BLAJ010101	Matrix built from structural superposition data for identifying potential remote homologues (Blake-Cohen, 2001)	AAindex	9	-2	0	14	-23	2	0	1	5	3	-6	0	13	0	0	0	3	7	-8	1	-2	-1	-1	7	-18	-8	0	-8	-10	-4	-13	-2	-7	0	0	-20	1	-3	-10
54	PRLA000101	Structure derived matrix (SDM) for alignment of distantly related sequences (Prlic et al., 2000)	AAindex	2.09	-0.39	-0.12	2.87	-2.19	0.54	-0.15	0.33	1.2	0.4	-1.21	-0.73	2.6	0.34	-0.5	-0.12	-0.21	1.56	-2.67	0.31	-0.39	-0.53	-0.76	0.13	0.31	-0.63	-0.57	-2.43	-2.65	-2.17	-2.08	-0.04	-1.47	0.02	-0.38	-1.97	0.27	-2	-1.21
55	PRLA000102	Homologous structure dereived matrix (HSDM) for alignment of distantly related sequences (Prlic et al., 2000)	AAindex	5.5	-1.09	-0.47	8.59	-6.65	0.7	0.34	1.42	2.41	1.54	-3.85	-2.38	7.85	1.27	-2.24	-0.47	-0.61	3.86	-7.46	-0.17	-1.09	-1.11	-2.42	-0.74	1.08	-1.43	-1.77	-5.78	-7.41	-5.65	-6.53	0.16	-3.26	0.16	-1.22	-4.7	0.63	-4.04	-4.77
56	QUIB020101	STROMA score matrix for the alignment of known distant homologs (Qian-Goldstein, 2002)	AAindex	2.5	-0.2	1.2	5.2	-3	1.5	0.2	1.9	3.2	1.4	-2.9	1	3.6	1.7	0.2	1.2	1.6	3.4	-4.7	1.1	-0.2	0.7	-1.6	2	0.7	-1.4	1.1	-2.4	-3.4	-0.9	-1.9	-0.2	-0.9	1.9	-0.2	-2.4	1.4	-2.4	-4.2
57	NAOD960101	Substitution matrix derived from the single residue interchanges at spatially conserved regions of proteins (Naor et al., 1996)	AAindex	4	0	0	7	-7	3	1	5	7	4	-3	0	7	0	0	0	3	7	-3	1	0	0	-1	5	-3	-2	0	-9	-8	-8	-5	0	-7	-2	0	-6	0	-8	-1
58	RUSR970101	Substitution matrix based on structural alignments of analogous proteins (Russell et al., 1997)	AAindex	-2	3	2	0	-1	2	0	2	6	5	0	0	0	0	1	2	2	3	-1	2	3	2	-1	-2	-5	-5	0	-4	-1	-1	-4	4	1	-1	1	-8	1	2	0
59	RUSR970102	Substitution matrix based on structural alignments of remote homologous proteins (Russell et al., 1997)	AAindex	0	0	0	4	0	2	0	2	5	3	-2	-1	4	2	0	0	0	7	-6	1	0	0	0	3	-1	0	0	-8	-7	-3	0	0	-3	1	1	-4	1	-2	-2
60	RUSR970103	Substitution matrix based on structural alignments of analogous and remote homologous proteins (Russell et al., 1997)	AAindex	-2	2	1	0	2	3	0	3	4	4	-1	-1	0	0	1	1	1	5	-3	3	2	0	0	1	-3	0	0	-7	-3	0	-1	2	0	0	1	-8	0	0	-1
61	OGAK980101	Substitution matrix derived from structural alignments by maximizing entropy (Ogata et al., 1998)	AAindex	-4.8	-6.7	-6.7	-6.2	-8.5	-8.1	-6.8	-7.1	-6.4	-6.5	-7.2	-6.9	-6.5	-5.6	-7	-6.7	-6.8	-5.5	-8.1	-7.6	-6.7	-6.8	-7.9	-7.5	-8.2	-7.8	-7.1	-7.6	-7.1	-7.2	-8.6	-8	-7.9	-5.9	-6.7	-10.2	-5.8	-7.2	-7.2
62	KANM000101	Substitution matrix (OPTIMA) derived by maximizing discrimination between homologs and non-homologs (Kann et al., 2000)	AAindex	36	-7	-10	56	-29	3	-8	2	20	10	-20	-20	46	12	-9	-10	-6	28	-32	-7	-7	-5	-19	12	-10	-10	-19	-32	-43	-31	-30	-9	-30	6	-10	-39	4	-30	-19
63	NGPC000101	Substitution matrix (PHAT) built from hydrophobic and transmembrane regions of the Blocks database (Ng et al., 2000)	AAindex	5	-1	-5	9	-10	-4	-6	2	6	1	-4	-5	9	2	-6	-5	-5	2	-2	-5	-1	-3	-1	-2	1	-6	-2	-3	-5	-3	-2	-1	-3	1	-7	-7	1	-4	-5
64	MUET010101	Non-symmetric substitution matrix (SLIM) for detection of homologous transmembrane proteins (Mueller et al., 2001)	AAindex	5	0	-7	10	-7	-4.5	-5	-1	2	0.5	-5.5	-6.5	7	2	-5.5	-7	-6.5	3.5	-1	-5.5	0	-4	0	-2.5	3	-4	-3	-4	-6	-4.5	-2.5	0	-3.5	1.5	-5.5	-7	0.5	-4	-4
65	MUET020101	Substitution matrix (VTML160) obtained by maximum likelihood estimation (Mueller et al., 2002)	AAindex	5	-2	-1	7	-4	1	-1	0	3	1	-5	-1	6	1	-2	-1	-1	3	-6	0	-2	0	-3	2	1	-2	-1	-4	-6	-4	-3	-1	-4	0	-1	-5	0	-4	-5
66	MUET020102	Substitution matrix (VTML250) obtained by maximum likelihood estimation (Mueller et al., 2002)	AAindex	2	-1	0	5	-2	1	-1	1	3	1	-3	0	3	1	-1	0	0	3	-4	0	-1	0	-2	2	1	-1	0	-3	-4	-2	-1	-1	-2	0	-1	-3	1	-3	-4
67	CROG050101	Substitution matrix computed from the Dirichlet Mixture Model (Crooks-Brenner, 2005)	AAindex	4	-2	-1	6	-4	0	-1	0	1	0	-3	-2	6	0	-2	-1	-1	2	-5	-1	-2	-1	-2	1	-2	-2	-2	-5	-5	-4	-3	-1	-3	-1	-2	-4	-1	-4	-4
68	LINK010101	Substitution matrices from an neural network model (Lin et al., 2001)	AAindex	0.441	0.088	0.519	0.02	0.006	0.004	0	0.003	0.001	0.026	0.011	0.045	0.034	0.056	0.03	0.519	0.027	0	0.015	0.014	0.088	0.395	0.008	0.041	0	0.016	0.033	0.051	0.017	0.028	0.01	0.011	0.021	0.157	0.102	0.047	0.014	0.013	0.019
69	DOSZ010102	Normalised version of SM_SAUSAGE (Dosztanyi-Torda, 2001)	AAindex	0.56	0.24	0.78	-7	-11.35	-6.2	-7.23	-1.22	-9.95	1.4	-2.94	-6.26	-4.35	-4.66	-5.1	0.78	-1.07	-10.18	1.86	-0.94	0.24	-0.05	-10.42	-6.13	-11.62	-12.42	-7.73	-0.51	-6.7	2.19	-1.44	-7.82	-9.48	6.53	3.96	-0.76	-2.33	-2.89	-4.62
70	DOSZ010103	An amino acid similarity matrix based on the THREADER force field (Dosztanyi-Torda, 2001)	AAindex	10	1.6	8.1	-1.2	-1.3	-0.5	-1	0.5	-0.6	0.8	-1.6	-1	2.3	3	-0.2	8.1	-0.1	-2.3	1	-0.4	1.6	8.4	-4.2	1.6	-0.6	-1.8	-2.8	0.5	-0.4	7.4	3.9	-1.4	-1.1	9.8	2.7	2.9	1.8	-0.2	1.4
71	DOSZ010104	Normalised version of SM_THREADER (Dosztanyi-Torda, 2001)	AAindex	5.34	1.09	7.03	-2.01	-4.75	-2.34	-3.04	-2.08	-3.62	0.6	-1.2	-1.66	-1.67	-1.02	-2.01	7.03	1.55	-4.24	-1.78	1.12	1.09	5.96	-3.54	-0.96	-3.78	-4.97	-2.43	0.93	-3.14	2.88	-0.84	-3.58	-5.08	4.84	3.93	1.22	-0.2	-0.67	-2.56
72	GIAG010101	Residue substitutions matrix from thermo/mesophilic to psychrophilic enzymes (Gianese et al., 2001)	AAindex	0	-1.3	0	0.5	-0.8	0.2	-0.2	-0.4	0.2	1.9	0.2	-5.7	2.4	5.7	-2.4	0	0.4	-0.2	0.1	-0.6	-1.3	0	0	0.9	0.3	0.1	-1	-0.4	0.4	1.3	0.2	-0.1	-0.5	-0.3	-2.7	2	0.1	-0.3	0.2
73	HENS920104	BLOSUM50 substitution matrix (Henikoff-Henikoff, 1992)	AAindex	5	1	7	-1	-4	-3	-3	-3	-4	0	-2	-1	-2	-1	-2	7	0	-4	-3	-1	1	7	-3	0	-3	-4	-2	0	-2	2	-1	-3	-4	2	2	1	0	-1	-2
74	DAYM780302	Log odds matrix for 40 PAMs (Dayhoff et al., 1978)RA Dayhoff, M.O., Schwartz, R.M. and Orcutt, B.C.T A model of evolutionary change in proteins	AAindex	6	7	7	-5	-3	-8	-11	-6	-5	-4	-7	7	-4	6	8	7	-7	-2	-1	-8	7	8	9	2	-5	-3	7	-5	-2	-15	-12	11	-7	7	6	-8	6	-3	-3
75	DOSZ010101	Amino acid similarity matrix based on the sausage force field (Dosztanyi-Torda, 2001)	AAindex	15.4	5.3	6.7	-9	-11.4	9.8	18.4	8.9	-2.3	9.3	36.7	-6	-2.8	10.9	-5.3	6.7	19.1	8.5	10.1	22.4	5.3	-1.6	-10	-8.9	-7.3	-8.7	23.5	13	-7.3	24.8	27.4	39.2	-13.3	24.9	-8.8	-5.8	-7.1	7.6	-5.4
76	KOSJ950101	Context-dependent optimal substitution matrices for exposed helix (Koshi-Goldstein, 1995)	AAindex	47.2	0.8	5.3	62.7	0.1	4.7	0.3	2	8.6	1.6	0.1	3.9	47.5	18.1	0.9	5.3	0.2	3.4	0.1	1.2	0.8	1.7	1.7	5.7	0.9	4.3	0.2	0.1	0.1	0.7	1.5	5.2	0.4	6.2	4.1	0.1	2	0.8	0.1
77	KOSJ950102	Context-dependent optimal substitution matrices for exposed beta (Koshi-Goldstein, 1995)	AAindex	47.2	0.8	5.3	62.7	0.1	4.7	0.3	2	8.6	1.6	0.1	3.9	47.5	18.1	0.9	5.3	0.2	3.4	0.1	1.2	0.8	1.7	1.7	5.7	0.9	4.3	0.2	0.1	0.1	0.7	1.5	5.2	0.4	6.2	4.1	0.1	2	0.8	0.1
78	KOSJ950103	Context-dependent optimal substitution matrices for exposed turn (Koshi-Goldstein, 1995)	AAindex	47.2	1.5	3.9	5.3	1.7	2	0.3	0.1	4.1	0.8	5.2	0.2	1.7	8.4	0.9	18.1	7.5	6.2	0.3	0.5	1.5	73.6	1.6	0.1	1	0.1	0.1	0.1	0.1	0.1	0.5	1.5	0.1	0.3	0.1	0.9	0.1	0.1	0.1
79	KOSJ950104	Context-dependent optimal substitution matrices for exposed coil (Koshi-Goldstein, 1995)	AAindex	47.2	1.5	3.9	5.3	1.7	2	0.3	0.1	4.1	0.8	5.2	0.2	1.7	8.4	0.9	18.1	7.5	6.2	0.3	0.5	1.5	73.6	1.6	0.1	1	0.1	0.1	0.1	0.1	0.1	0.5	1.5	0.1	0.3	0.1	0.9	0.1	0.1	0.1
80	KOSJ950105	Context-dependent optimal substitution matrices for buried helix (Koshi-Goldstein, 1995)	AAindex	47.2	1.5	3.9	5.3	1.7	2	0.3	0.1	4.1	0.8	5.2	0.2	1.7	8.4	0.9	18.1	7.5	6.2	0.3	0.5	1.5	73.6	1.6	0.1	1	0.1	0.1	0.1	0.1	0.1	0.5	1.5	0.1	0.3	0.1	0.9	0.1	0.1	0.1
81	KOSJ950106	Context-dependent optimal substitution matrices for buried beta (Koshi-Goldstein, 1995)	AAindex	47.2	1.5	3.9	5.3	1.7	2	0.3	0.1	4.1	0.8	5.2	0.2	1.7	8.4	0.9	18.1	7.5	6.2	0.3	0.5	1.5	73.6	1.6	0.1	1	0.1	0.1	0.1	0.1	0.1	0.5	1.5	0.1	0.3	0.1	0.9	0.1	0.1	0.1
82	KOSJ950107	Context-dependent optimal substitution matrices for buried turn (Koshi-Goldstein, 1995)	AAindex	47.2	1.5	3.9	5.3	1.7	2	0.3	0.1	4.1	0.8	5.2	0.2	1.7	8.4	0.9	18.1	7.5	6.2	0.3	0.5	1.5	73.6	1.6	0.1	1	0.1	0.1	0.1	0.1	0.1	0.5	1.5	0.1	0.3	0.1	0.9	0.1	0.1	0.1
83	KOSJ950108	Context-dependent optimal substitution matrices for buried coil (Koshi-Goldstein, 1995)	AAindex	47.2	1.5	3.9	5.3	1.7	2	0.3	0.1	4.1	0.8	5.2	0.2	1.7	8.4	0.9	18.1	7.5	6.2	0.3	0.5	1.5	73.6	1.6	0.1	1	0.1	0.1	0.1	0.1	0.1	0.5	1.5	0.1	0.3	0.1	0.9	0.1	0.1	0.1
84	KOSJ950109	Context-dependent optimal substitution matrices for alpha helix (Koshi-Goldstein, 1995)	AAindex	47.2	1.5	3.9	5.3	1.7	2	0.3	0.1	4.1	0.8	5.2	0.2	1.7	8.4	0.9	18.1	7.5	6.2	0.3	0.5	1.5	73.6	1.6	0.1	1	0.1	0.1	0.1	0.1	0.1	0.5	1.5	0.1	0.3	0.1	0.9	0.1	0.1	0.1
85	KOSJ950110	Context-dependent optimal substitution matrices for beta sheet (Koshi-Goldstein, 1995)	AAindex	47.2	1.5	3.9	5.3	1.7	2	0.3	0.1	4.1	0.8	5.2	0.2	1.7	8.4	0.9	18.1	7.5	6.2	0.3	0.5	1.5	73.6	1.6	0.1	1	0.1	0.1	0.1	0.1	0.1	0.5	1.5	0.1	0.3	0.1	0.9	0.1	0.1	0.1
86	KOSJ950111	Context-dependent optimal substitution matrices for turn (Koshi-Goldstein, 1995)	AAindex	47.2	1.5	3.9	5.3	1.7	2	0.3	0.1	4.1	0.8	5.2	0.2	1.7	8.4	0.9	18.1	7.5	6.2	0.3	0.5	1.5	73.6	1.6	0.1	1	0.1	0.1	0.1	0.1	0.1	0.5	1.5	0.1	0.3	0.1	0.9	0.1	0.1	0.1
87	KOSJ950112	Context-dependent optimal substitution matrices for coil (Koshi-Goldstein, 1995)	AAindex	47.2	1.5	3.9	5.3	1.7	2	0.3	0.1	4.1	0.8	5.2	0.2	1.7	8.4	0.9	18.1	7.5	6.2	0.3	0.5	1.5	73.6	1.6	0.1	1	0.1	0.1	0.1	0.1	0.1	0.5	1.5	0.1	0.3	0.1	0.9	0.1	0.1	0.1
88	KOSJ950113	Context-dependent optimal substitution matrices for exposed residues (Koshi-Goldstein, 1995)	AAindex	47.2	1.5	3.9	5.3	1.7	2	0.3	0.1	4.1	0.8	5.2	0.2	1.7	8.4	0.9	18.1	7.5	6.2	0.3	0.5	1.5	73.6	1.6	0.1	1	0.1	0.1	0.1	0.1	0.1	0.5	1.5	0.1	0.3	0.1	0.9	0.1	0.1	0.1
89	KOSJ950114	Context-dependent optimal substitution matrices for buried residues (Koshi-Goldstein, 1995)	AAindex	47.2	1.5	3.9	5.3	1.7	2	0.3	0.1	4.1	0.8	5.2	0.2	1.7	8.4	0.9	18.1	7.5	6.2	0.3	0.5	1.5	73.6	1.6	0.1	1	0.1	0.1	0.1	0.1	0.1	0.5	1.5	0.1	0.3	0.1	0.9	0.1	0.1	0.1
90	KOSJ950115	Context-dependent optimal substitution matrices for all residues (Koshi-Goldstein, 1995)	AAindex	47.2	1.5	3.9	5.3	1.7	2	0.3	0.1	4.1	0.8	5.2	0.2	1.7	8.4	0.9	18.1	7.5	6.2	0.3	0.5	1.5	73.6	1.6	0.1	1	0.1	0.1	0.1	0.1	0.1	0.5	1.5	0.1	0.3	0.1	0.9	0.1	0.1	0.1
91	OVEJ920102	Environment-specific amino acid substitution matrix for alpha residues (Overington et al., 1992)	AAindex	0.355	0.901	0.315	0.305	0.415	0.37	0.571	0.325	0.336	0.415	0.197	0.195	0.424	0.183	0.348	0.264	0.266	0.35	0.588	0.377	0.901	0	0	0.011	0.004	0.017	0.003	0.017	0.029	0.225	0.01	0	0.013	0.029	0.004	0.096	0.037	0.012	0.064
92	OVEJ920103	Environment-specific amino acid substitution matrix for beta residues (Overington et al., 1992)	AAindex	0.355	0.901	0.315	0.305	0.415	0.37	0.571	0.325	0.336	0.415	0.197	0.195	0.424	0.183	0.348	0.264	0.266	0.35	0.588	0.377	0.901	0	0	0.011	0.004	0.017	0.003	0.017	0.029	0.225	0.01	0	0.013	0.029	0.004	0.096	0.037	0.012	0.064
93	OVEJ920104	Environment-specific amino acid substitution matrix for accessible residues (Overington et al., 1992)	AAindex	0.355	0.901	0.315	0.305	0.415	0.37	0.571	0.325	0.336	0.415	0.197	0.195	0.424	0.183	0.348	0.264	0.266	0.35	0.588	0.377	0.901	0	0	0.011	0.004	0.017	0.003	0.017	0.029	0.225	0.01	0	0.013	0.029	0.004	0.096	0.037	0.012	0.064
94	OVEJ920105	Environment-specific amino acid substitution matrix for inaccessible residues (Overington et al., 1992)	AAindex	0.355	0.901	0.315	0.305	0.415	0.37	0.571	0.325	0.336	0.415	0.197	0.195	0.424	0.183	0.348	0.264	0.266	0.35	0.588	0.377	0.901	0	0	0.011	0.004	0.017	0.003	0.017	0.029	0.225	0.01	0	0.013	0.029	0.004	0.096	0.037	0.012	0.064
101	TANS760101	Statistical contact potential derived from 25 x-ray protein structures	AAindex	-2.6	-4.2	-2.8	-3	-5.1	-3.8	-4	-5.9	-3.1	-4.8	-4.6	-3.1	-3.4	-3.5	-3.4	-2.9	-3.3	-4.3	-5.2	-4.7	-4.2	-7.1	-4.2	-4.4	-6.8	-5.4	-5.6	-7.3	-4.4	-6.2	-6.2	-4.9	-5.3	-5	-5.3	-4.6	-4.8	-6	-6.9
102	TANS760102	Number of contacts between side chains derived from 25 x-ray protein structures	AAindex	36	15	36	27	62	115	25	119	55	174	26	39	33	34	20	64	70	101	38	65	15	48	10	9	32	53	11	33	14	49	11	21	24	13	13	28	22	53	17
103	ROBB790102	Interaction energies derived from side chain contacts in the interiors of known protein structures	AAindex	-0.268	-0.18	0.926	0.902	-0.961	0	-0.187	-0.899	0.92	-0.865	-0.525	0.01	-1.47	0.017	0.529	0.022	-0.177	-0.617	-1.052	-0.899	-0.18	-20	10.31	9.05	-0.873	0	-0.099	-0.811	8.223	-0.777	-0.437	0.245	-1.382	0.296	5.014	0.376	-0.089	-0.529	-0.964
104	BRYS930101	Distance-dependent statistical potential (only energies of contacts within 0-5 Angstrooms are included)	AAindex	0.23	0.159	0.14	0.182	0.132	0.282	-0.079	-0.535	0.063	-0.245	-0.364	0.159	0.022	-0.008	0.237	0.115	0.002	-0.359	0.117	-0.03	0.159	-0.524	-0.137	0.121	-0.095	-0.187	-0.187	-0.415	1.115	-0.061	-0.032	0.105	-0.199	0.487	0.174	-0.164	0.021	-0.026	0.08
105	THOP960101	Mixed quasichemical and optimization-based protein contact potential	AAindex	-0.08	-0.3	0.1	0.03	-0.05	-0.09	-0.15	-0.64	0	-0.57	0.05	-0.14	0.41	-0.11	0.07	-0.01	-0.22	-0.6	-0.08	-0.37	-0.3	-1.79	0.55	0.04	-0.98	-0.42	-0.82	-0.48	0	-0.69	-1.23	-0.32	0.07	-0.49	-0.4	-0.2	-0.38	-0.94	-0.3
106	MIRL960101	Statistical potential derived by the maximization of the harmonic mean of Z scores	AAindex	-0.13	0	0.12	0.26	0.03	-0.07	0.34	-0.22	0.14	-0.01	0.25	0.28	0.1	0.08	0.43	-0.06	-0.09	-0.1	-0.09	0.09	0	-1.06	0.03	0.69	-0.23	-0.08	-0.19	0.16	0.71	-0.08	0.19	0.13	0	0.05	0.24	-0.02	0.19	0.06	0.08
107	VENM980101	Statistical potential derived by the maximization of the perceptron criterion	AAindex	-0.0281	0.0007	0.0167	0.094	0.0388	0.0025	0.0092	-0.1004	0.0472	-0.0079	0.0757	0.0908	0.0782	0.023	0.0144	0.0065	-0.0383	-0.0852	0.0095	0.0159	0.0007	-0.1816	0.0159	0.02	-0.0452	-0.0184	-0.037	-0.0026	0.0114	0.0747	0.1307	0.0094	-0.0151	0.0358	0.1005	0.0158	0.1422	-0.0607	0.0489
108	BASU010101	Optimization-based potential derived by the modified perceptron criterion	AAindex	-0.0479	-0.1085	0.1018	0.1346	-0.1119	0.1844	0.0266	-0.0737	0.0691	-0.1711	-0.0847	0.1049	0.1462	-0.0457	0.1049	0.0464	0.031	-0.1431	-0.088	-0.1408	-0.1085	-1.0442	0.1169	0.155	-0.3262	-0.0982	-0.0701	-0.2235	-0.033	-0.1305	-0.0557	-0.0844	0.0545	0.0715	0.0544	-0.0132	-0.0013	-0.2349	-0.1176
109	MIYS850102	Quasichemical energy of transfer of amino acids from water to the protein environment	AAindex	-2.51	-3.38	-1.57	-1.51	-4.36	-2.15	-2.09	-4.41	-1.1	-3.96	-3.99	-1.44	-1.81	-1.7	-1.5	-1.89	-2.15	-3.62	-3.93	-2.85	-3.38	-5.44	-2.66	-2.08	-5.63	-3.16	-3.63	-5.03	-1.54	-5.03	-5.05	-2.59	-2.92	-2.73	-2.7	-2.86	-2.88	-4.46	-4.76
110	MIYS850103	Quasichemical energy of interactions in an average buried environment	AAindex	-0.13	0	0.12	0.25	0.03	-0.07	0.34	-0.22	0.15	-0.01	0.25	0.28	0.1	0.07	0.44	-0.06	-0.09	-0.11	-0.09	0.08	0	-1.06	0.03	0.68	-0.24	-0.08	-0.2	0.16	0.71	-0.08	0.19	0.13	-0.01	0.04	0.24	-0.03	0.18	0.05	0.08
111	MIYS960101	Quasichemical energy of transfer of amino acids from water to the protein environment	AAindex	-2.72	-3.57	-1.7	-1.51	-4.81	-2.31	-2.41	-4.58	-1.31	-4.91	-3.94	-1.84	-2.03	-1.89	-1.83	-2.01	-2.32	-4.04	-3.82	-3.36	-3.57	-5.44	-2.41	-2.27	-5.8	-3.16	-3.6	-5.5	-1.95	-5.83	-4.99	-2.59	-3.07	-2.85	-2.57	-2.86	-3.11	-4.96	-4.95
112	MIYS960102	Quasichemical energy of interactions in an average buried environment	AAindex	-0.13	0.02	0.16	0.3	-0.03	-0.1	0.17	-0.14	0.23	-0.08	0	0.1	0.08	0.13	0.3	-0.01	-0.01	-0.13	0.01	0.07	0.02	-0.85	0.45	0.54	-0.02	0.05	-0.02	-0.06	0.59	0	-0.05	0.35	0.04	0.17	0.56	0.14	0.2	-0.05	-0.12
113	MIYS960103	Number of contacts between side chains derived from 1168 x-ray protein structures	AAindex	11789	4306	7836	5933	10516	17079	3600	16465	6019	24837	5014	6465	6368	5037	5205	11447	11323	21733	3453	7942	4306	5003	1584	1189	2573	4168	1209	3830	1200	5395	1247	1549	1983	1375	1120	3012	2475	4768	1168
114	MIYS990106	Quasichemical energy of transfer of amino acids from water to the protein environment	AAindex	-0.12	-0.33	0.27	0.38	-0.36	-0.08	0.07	-0.37	0.41	-0.38	-0.27	0.15	0.15	0.22	0.24	0.1	0.04	-0.32	-0.27	-0.2	-0.33	-1.19	0.12	0.2	-0.67	-0.31	-0.36	-0.64	0.33	-0.65	-0.61	-0.01	-0.18	-0.07	0.08	-0.13	-0.15	-0.59	-0.66
115	MIYS990107	Quasichemical energy of interactions in an average buried environment	AAindex	-0.08	0.01	0.1	0.19	-0.01	-0.04	0.11	-0.07	0.13	-0.04	0	0.07	0.06	0.09	0.18	0.01	0.01	-0.07	0.02	0.05	0.01	-0.55	0.25	0.31	-0.02	0.03	-0.02	-0.04	0.35	-0.01	-0.04	0.21	0.03	0.1	0.32	0.08	0.12	-0.04	-0.07
116	LIWA970101	Modified version of the Miyazawa-Jernigan transfer energy	AAindex	-3.28	-3.93	-2.52	-2.45	-4.04	-2.91	-2.95	-4.18	-2.28	-4.06	-3.93	-2.59	-2.81	-2.67	-2.56	-2.76	-2.97	-3.93	-3.81	-3.41	-3.93	-4.54	-3.12	-2.97	-4.94	-3.68	-3.87	-4.9	-2.71	-4.72	-4.72	-3.14	-3.5	-3.31	-3.02	-3.56	-3.73	-4.53	-4.6
117	KESO980101	Quasichemical transfer energy derived from interfacial regions of protein-protein complexes	AAindex	-3.51	-3.99	-2.74	-2.69	-4.43	-2.24	-3.29	-4.45	-1.91	-5.02	-4.42	-2.06	-1.78	-3.65	-3.26	-2.49	-2.38	-3.86	-4.66	-3.96	-3.99	-7.23	-3.56	-3.02	-4.81	-3.43	-4.79	-5.53	-2.28	-6.13	-5.07	-1.76	-2.97	-4.37	-4.41	-3.41	-2.92	-4.82	-4.7
118	KESO980102	Quasichemical energy in an average protein environment derived from interfacial regions of protein-protein complexes	AAindex	-0.38	0	0.02	0.2	0.01	0.08	0.01	-0.08	0.06	-0.09	-0.27	0.31	0.19	0.08	0.16	0.08	0.14	-0.11	-0.38	0	0	-2.38	0.06	0.72	0.49	-0.26	-0.64	-0.3	-0.5	-0.34	-0.08	1.46	-0.15	0.21	-0.12	0.02	0.46	-0.22	0.44
119	MOOG990101	Quasichemical potential derived from interfacial regions of protein-protein complexes	AAindex	-0.48	-0.12	-0.39	-0.39	0.13	-0.29	0.04	-0.1	-0.53	0.01	0.15	-0.21	-0.56	-0.16	-0.2	-0.15	-0.43	-0.01	0.08	0.15	-0.12	0	-0.49	-0.14	0.18	0.11	0	0	-0.03	0.09	0	-0.3	-0.28	0.23	0.17	0.15	-0.17	-0.09	0
120	BETM990101	Modified version of the Miyazawa-Jernigan transfer energy	AAindex	-0.2	-0.26	0.3	0.43	-0.33	-0.03	0.21	-0.35	0.2	-0.37	-0.23	0.24	0.07	0.21	0.27	0.15	0	-0.38	-0.4	-0.15	-0.26	-1.34	0.38	0.46	-0.53	-0.09	-0.19	-0.48	0.35	-0.5	-0.49	0.28	-0.18	0.04	0.32	0.09	0	-0.51	-0.74
121	TOBD000101	Optimization-derived potential obtained for small set of decoys	AAindex	-0.17	-0.48	0.58	0.29	-0.49	0.43	0.66	-0.79	0.18	-0.13	-0.82	0.33	0.87	-0.14	0.03	0.18	-0.45	-0.54	-0.04	-0.08	-0.48	-2.94	-0.32	0.16	-2.52	0.14	-2.34	-0.26	0.45	-0.25	-1.99	-1.89	-0.19	-0.84	0.8	0.47	-1.38	-0.46	-2.09
122	TOBD000102	Optimization-derived potential obtained for large set of decoys	AAindex	-0.15	-0.96	0.62	-0.02	-0.79	0.23	0.29	-0.85	0.96	-0.72	-0.85	1.01	0.08	0.16	-0.16	0.24	0.33	-0.36	0.24	-0.5	-0.96	-2.11	-0.5	0.65	-0.96	-0.74	-0.66	-1.02	-0.38	-0.24	-1.3	0.18	-0.45	-0.54	-0.25	-0.41	0	-0.58	-0.32

Table S12 Amino acid descriptors (2D) used in this study.

			1st aa	A	A	A	A	A	A	A	A	A	A	A	A	A	A	A	A	A	A	A	A	C	C	C	C	C	C	C	C	C	C	C	C	C	C	C	C	C	C	C
			2nd aa	A	C	D	E	F	G	H	I	K	L	M	N	P	Q	R	S	T	V	W	Y	A	C	D	E	F	G	H	I	K	L	M	N	P	Q	R	S	T	V	W
No.	Descriptor ID	Attribute	Ref*	AA	AC	AD	AE	AF	AG	AH	AI	AK	AL	AM	AN	AP	AQ	AR	AS	AT	AV	AW	AY	CA	CC	CD	CE	CF	CG	CH	CI	CK	CL	CM	CN	CP	CQ	CR	CS	CT	CV	CW
123	PARB960101	Statistical contact potential derived by the quasichemical approximation	AAindex	0.5	0.3	0.3	0.6	-0.8	0	0	-0.4	1	-0.4	-0.5	0.3	0.6	0	0.2	0.5	0	-0.3	-0.8	-0.7	0.3	-2.7	0	0.1	-1.2	0	-0.6	-0.8	0.5	-0.8	-0.8	0	0	-0.2	0	-0.1	-0.3	-0.5	-1.3
124	PARB960102	Modified version of the Miyazawa-Jernigan transfer energy	AAindex	-2.9	-3.1	-2.6	-1.9	-3.7	0	-2.6	-3.9	-1.9	-3.7	-3.8	-2.8	-2.3	-2.7	-2.4	-2.4	-3.2	-4.1	-3.8	-3.7	-3.1	-6.1	-2.9	-2.4	-4.1	0	-3.3	-4.2	-2.4	-4	-4.2	-3.2	-2.8	-2.9	-2.6	-3	-3.5	-4.3	-4.3
125	KOLA930101	Statistical potential derived by the quasichemical approximation	AAindex	-0.1	0.1	0.1	0.5	-0.6	0.1	-0.1	-0.6	1	-0.4	0.1	0.1	0.3	0	0.5	-0.1	-0.3	-0.6	-0.7	-0.5	0.1	-3.3	0.3	0.8	-1.5	0.1	-1.2	-1.1	1.6	-0.5	-1.8	0.3	0	0.1	0.4	-0.4	0	-0.8	-0.5
126	GODA950101	Quasichemical statistical potential derived from buried contacts	AAindex	0.1	0.1	0.2	0.3	-0.1	0	0.2	-0.1	0.3	-0.1	0	0.1	0	0	0.3	0.1	0	-0.1	0	-0.1	0.1	-0.9	0.3	0.4	0.1	0	0	0.2	0.4	0.1	0.1	-0.1	-0.1	0.1	0.4	0.1	0	0	0.2
127	SKOJ970101	Statistical potential derived by the quasichemical approximation	AAindex	0.8	0.6	1.2	1.2	-0.2	1.2	0.4	-0.6	1.3	-0.3	-0.3	0.9	0.6	0.6	0.6	0.9	0.5	-0.4	-0.6	-0.4	0.6	-1.3	0.8	0.9	-0.6	1.3	0.1	-0.5	1.3	-0.4	-0.3	0.6	0.4	0.3	0.7	0.6	0.5	-0.6	-0.7
128	SKOJ000101	Statistical quasichemical potential with the partially composition-corrected pair scale	AAindex	1	0.6	1.1	1.1	-0.2	1.4	0.5	-0.3	1	-0.1	-0.2	0.8	0.8	0.6	0.4	0.9	0.6	-0.1	-0.5	-0.2	0.6	-1.7	0.5	0.8	-0.8	0.9	-0.2	-0.5	0.9	-0.5	-0.5	0.5	0.4	0.2	0.2	0.4	0.1	-0.5	-0.7
129	SKOJ000102	Statistical quasichemical potential with the composition-corrected pair scale	AAindex	0.8	0	0.9	1	-0.2	1.2	0.2	-0.3	0.8	-0.1	-0.3	0.7	0.7	0.4	0.3	0.8	0.5	0	-0.6	-0.3	0	-2.4	-0.3	-0.2	-1.1	0.1	-1.4	-0.8	-0.3	-0.6	-1.3	-0.5	-0.4	-0.7	-0.6	-0.4	-0.5	-0.7	-1.5
130	BONM030101	Quasichemical statistical potential for the antiparallel orientation of interacting side groups	AAindex	-0.1	-0.3	1	1	-0.3	0.1	0.4	-0.4	1	-0.4	-0.2	0.7	0.4	0.6	0.6	0.3	0.2	-0.5	0.1	0	-0.3	-2.3	0.6	0.7	-1.4	-0.4	-1.2	-0.2	0.4	-0.4	-0.4	0.2	-0.1	0.1	-0.7	-0.1	-0.1	-0.3	-1.2
131	BONM030102	Quasichemical statistical potential for the intermediate orientation of interacting side groups	AAindex	0.1	-0.2	0.6	0.8	0	0.4	0.5	-0.1	0.8	-0.2	0	0.4	0.5	0.4	0.5	0.3	0.2	-0.2	0.3	0.2	-0.2	-1.7	0.3	0.8	-1.2	-0.3	-1.1	-0.2	0.8	-0.4	-0.3	0.3	0.1	0.3	-0.6	-0.1	0	-0.3	-1.1
132	BONM030103	Quasichemical statistical potential for the parallel orientation of interacting side groups	AAindex	-0.1	-0.5	0.1	0.4	-0.2	0.3	0	-0.4	0.4	-0.4	-0.3	0.1	0.4	0.2	0.3	-0.2	-0.1	-0.4	0.2	-0.1	-0.5	-1.6	0.2	0.6	-1.5	-0.2	-1.3	-0.8	0.5	-0.9	-0.7	-0.1	-0.1	0	-0.6	-0.5	-0.4	-0.9	-1.3
133	BONM030104	Distances between centers of interacting side chains in the antiparallel orientation	AAindex	4.7	5	4.9	5.2	5.1	4.6	5.2	5.2	5.2	5.2	5	5	5.2	5	5.5	4.7	5.2	4.9	5.8	5.5	5	4.4	5.2	5.7	5.4	5.5	5.4	5.2	5.3	5.5	5.3	5.7	5.9	5.5	5.9	5.1	5.6	5.4	5.3
134	BONM030105	Distances between centers of interacting side chains in the intermediate orientation	AAindex	4.7	5.3	5	5	5.5	4.7	5.2	5.5	5.2	5.5	5.3	5.2	5.3	5.4	5.5	4.7	5.1	5.4	5.9	5.6	5.3	4.3	5.4	5.1	5.8	5.3	5.9	5.5	6.2	5.7	5.8	5.7	5.7	5.4	5.6	5.3	5.5	5.7	6.1
135	BONM030106	Distances between centers of interacting side chains in the parallel orientation	AAindex	4.8	5.1	5	4.8	5.8	4.5	5.3	5.5	5.4	5.4	5.3	4.9	4.9	5.3	5.5	4.9	4.9	5.2	5.8	5.7	5.1	4.2	5.1	5.7	5.9	5.2	6	5.9	6.5	5.7	5.3	5.2	5.2	6.1	6.1	5.5	6	5.8	6.5
136	MICC010101	Optimization-derived potential	AAindex	0.0015	-0.0008	-0.0013	-0.0024	0.0051	-0.0031	-0.0024	-0.0021	0.0018	0.0009	-0.0015	0.0033	-0.0051	0.005	-0.0025	-0.0015	-0.0002	0.0036	-0.0097	-0.0007	-0.0008	-0.0025	0.0023	0.0028	-0.0139	0.0018	0.0546	0.003	-0.006	-0.0002	0.0143	0.0061	-0.0017	0.0014	-0.0061	0.0018	0.0026	0.0003	-0.0352
137	SIMK990101	Distance-dependent statistical potential (contacts within 0-5 Angstrooms)	AAindex	-0.1357	0.2308	0.334	0.2647	0.0814	-0.0147	0.3241	-0.2218	0.3989	-0.1503	0.0352	0.2594	0.0362	0.2658	0.3712	0.1048	-0.0468	-0.2713	0.2	0.1284	0.2308	-1.7924	0.5302	0.6636	0.0964	0.2042	0.2438	0.1253	0.7318	0.1075	0.0446	0.3248	-0.0448	0.252	0.491	0.1128	0.2754	0.0978	0.094
138	SIMK990102	Distance-dependent statistical potential (contacts within 5-7.5 Angstrooms)	AAindex	-0.0223	-0.0441	0.1331	0.1522	-0.1491	-0.0566	0.0214	-0.0319	0.1468	0.0187	-0.0863	0.0296	0.08	0.0889	0.0952	0.0035	0.0053	-0.0235	-0.1126	-0.0531	-0.0441	-0.3542	0.1663	0.4664	-0.0887	-0.1562	-0.0895	0.0828	0.3018	0.0331	-0.0316	0.0506	-0.0959	0.0607	0.2119	-0.062	-0.0289	-0.0185	-0.1733
139	SIMK990103	Distance-dependent statistical potential (contacts within 7.5-10 Angstrooms)	AAindex	-0.0671	0.0492	0.0869	0.1011	0.0258	0.0261	0.0646	-0.1003	0.0617	-0.0899	-0.0322	0.0926	0.0457	0.0719	0.0615	0.0536	0.0474	-0.0728	0.0873	0.039	0.0492	-0.1711	0.0827	0.1284	-0.0552	-0.0687	-0.0512	0.0085	0.104	-0.0073	-0.1068	0.0203	0.0197	0.027	0.1034	-0.0084	-0.044	0.0077	-0.0257
140	SIMK990104	Distance-dependent statistical potential (contacts within 10-12 Angstrooms)	AAindex	-0.0143	0.0138	0.0335	0.029	-0.0232	0.0337	0.0165	-0.0442	0.0222	-0.0285	0.0046	0.0385	0.0226	0.0202	0.0032	0.0028	0.0064	-0.0201	0.0105	0.0121	0.0138	-0.0375	0.0567	0.0323	-0.0625	-0.0242	-0.0659	0.0432	0.0881	0.0056	0.0033	0.0009	-0.022	0.0267	-0.0216	-0.016	-0.0133	-0.0156	-0.0989
141	SIMK990105	Distance-dependent statistical potential (contacts longer than 12 Angstrooms)	AAindex	-0.0035	0.0018	0.0041	0.0041	-0.0021	0.0011	0.0032	-0.0075	0.0037	-0.0048	-0.0018	0.0039	0.0026	0.0034	0.0034	0.0017	0.0012	-0.0059	0.0019	0.0011	0.0018	-0.1084	0.0149	0.0183	-0.0149	-0.0036	-0.0042	-0.0051	0.0187	-0.0065	-0.0092	0.008	0.003	0.0099	0.012	0.001	-5E-05	-0.0093	-0.0129
142	ZHAC000101	Environment-dependent residue contact energies (rows = helix, cols = helix)	AAindex	-1.65	-2.04	-0.06	0.29	-1.7	-1.74	-0.48	-2.09	0.08	-1.89	-1.28	-0.25	-0.26	0	0.02	-0.83	-0.78	-1.84	-1.4	-1.1	-2.04	-2.81	-1.04	-0.38	-2.87	-2.35	-1.21	-2.99	-0.46	-2.74	-2.17	-0.95	-1.12	-0.41	-0.54	-1.36	-1.6	-2.72	-2.02
143	ZHAC000104	Environment-dependent residue contact energies (rows = strand, cols = strand)	AAindex	-2.52	-3.4	-1.22	-0.77	-2.99	-2.84	-1.69	-3.27	-0.7	-3.29	-2.63	-1.22	-1.64	-1.17	-1.24	-1.74	-1.87	-3.07	-2.2	-2.57	-3.4	-3.74	-1.34	-0.83	-3.61	-3.17	-2.31	-3.73	-0.74	-3.63	-3.48	-1.72	-1.92	-1.97	-0.78	-2.23	-2.39	-3.33	-3.03
144	ZHAC000106	Environment-dependent residue contact energies (rows = coil, cols = coil)	AAindex	0.12	-0.74	0.65	1.32	-0.41	0.11	0.35	-0.45	1.06	-0.25	0.29	0.59	0.48	0.7	0.56	0.45	0.3	-0.18	-0.28	-0.07	-0.74	-2.42	-0.12	0.2	-1.44	-0.56	-0.83	-1.27	0.76	-1.25	-0.74	-0.07	-0.23	-0.05	-0.25	-0.47	-0.37	-1.22	-0.63
145	ZHAC000102	Environment-dependent residue contact energies (helix->strand)	AAindex	-0.94	-1.75	0.66	0.82	-2.12	-0.4	-0.75	-1.99	0.6	-2.02	-1.54	0.59	0.63	0.33	0.56	-0.41	-0.32	-1.74	-1.85	-0.88	-1.54	-3.64	-0.21	0.01	-2.3	-1	-1.37	-2.44	0.5	-2.29	-2.51	-0.9	-1.63	1.37	-0.67	-0.21	-1.44	-2.25	-1.64
146	ZHAC000103	Environment-dependent residue contact energies (helix->coil)	AAindex	0.12	-0.83	0.9	1.33	-0.33	-0.22	0.47	-0.58	1.07	-0.44	-0.22	0.69	0.78	1.13	0.98	0.46	0.5	-0.51	-0.41	-0.22	-0.81	-2.65	0.58	0.31	-1.9	-0.95	-1.56	-1.17	0.42	-1.56	-0.95	-0.06	-0.04	0.14	-0.21	-0.31	-0.26	-1.4	-0.79
147	ZHAC000105	Environment-dependent residue contact energies (strand->coil)	AAindex	-0.57	-1.19	0.15	0.97	-0.7	-0.64	-0.02	-0.94	1.02	-0.76	-0.16	-0.28	0.17	0.63	0.23	-0.06	0.26	-0.83	-0.03	-0.44	-1.6	-3.3	-0.46	0.3	-1.26	-1.37	-0.58	-1.7	0.08	-1.63	-1.63	-0.68	-1.31	-0.3	-0.41	-0.89	-0.78	-1.44	-1.5
148	apol	Sum of the atomic polarizabilities (including implicit hydrogens) with polarizabilities taken from [CRC 1994].	MOE	23.168	26.068	25.865	28.958	36.395	20.074	31.981	32.448	34.882	32.448	32.255	27.496	28.021	30.59	37.082	23.97	27.063	29.355	41.681	37.197	26.068	28.968	28.765	31.858	39.295	22.974	34.881	35.348	37.782	35.348	35.155	30.396	30.921	33.49	39.982	26.87	29.963	32.255	44.581
149	ast_fraglike	Astex Fragment-like Test	MOE	1	1	1	1	1	1	1	1	1	1	1	1	1	1	1	1	1	1	1	1	1	1	1	1	1	1	1	1	1	1	1	1	1	1	1	1	1	1	0
150	ast_fraglike_ext	Astex Fragment-like Test (Extended)	MOE	0	0	0	0	0	0	0	0	0	0	0	0	1	0	0	0	0	0	0	0	0	0	0	0	0	0	0	0	0	0	0	0	0	0	0	0	0	0	0
151	ast_violation	Astex Fragment-like Violation Count	MOE	0	0	0	0	0	0	0	0	0	0	0	0	0	0	0	0	0	0	0	0	0	0	0	0	0	0	0	0	0	0	0	0	0	0	0	0	0	0	1
152	ast_violation_ext	Astex Fragment-like Violation Count (Extended)	MOE	1	2	2	2	2	1	2	2	2	2	2	2	0	2	2	2	2	2	2	2	2	2	2	2	2	2	2	2	2	2	2	2	1	2	2	2	2	2	3
153	a_acc	Number of hydrogen bond acceptor atoms (not counting acidic atoms but counting atoms that are both hydrogen bond donors and acceptors such as -OH).	MOE	1	1	1	1	1	1	3	1	1	1	1	2	1	2	1	2	2	1	1	2	1	1	1	1	1	1	3	1	1	1	1	2	1	2	1	2	2	1	1
154	a_acid	Number of acidic atoms.	MOE	2	2	4	4	2	2	2	2	2	2	2	2	2	2	2	2	2	2	2	2	2	2	4	4	2	2	2	2	2	2	2	2	2	2	2	2	2	2	2
155	a_aro	Number of aromatic atoms.	MOE	0	0	0	0	6	0	5	0	0	0	0	0	0	0	0	0	0	0	9	6	0	0	0	0	6	0	5	0	0	0	0	0	0	0	0	0	0	0	9
156	a_base	Number of basic atoms.	MOE	1	1	1	1	1	1	1	1	2	1	1	1	1	1	4	1	1	1	1	1	1	1	1	1	1	1	1	1	2	1	1	1	1	1	4	1	1	1	1
157	a_count	Number of atoms (including implicit hydrogens). This is calculated as the sum of (1 + hi) over all non-trivial atoms i.	MOE	23	24	25	28	33	20	30	32	35	32	30	27	27	30	37	24	27	29	37	34	24	25	26	29	34	21	31	33	36	33	31	28	28	31	38	25	28	30	38
158	a_don	Number of hydrogen bond donor atoms (not counting basic atoms but counting atoms that are both hydrogen bond donors and acceptors such as -OH).	MOE	1	1	1	1	1	1	3	1	1	1	1	2	0	2	1	2	2	1	2	2	1	1	1	1	1	1	3	1	1	1	1	2	0	2	1	2	2	1	2
159	a_donacc	Number of hydrogen bond donor and hydrogen bond acceptor atoms.	MOE	2	2	2	2	2	2	6	2	2	2	2	4	1	4	2	4	4	2	3	4	2	2	2	2	2	2	6	2	2	2	2	4	1	4	2	4	4	2	3
160	a_heavy	Number of heavy atoms #{Zi | Zi > 1}.	MOE	11	12	14	15	17	10	16	14	15	14	14	14	13	15	17	12	13	13	20	18	12	13	15	16	18	11	17	15	16	15	15	15	14	16	18	13	14	14	21
161	a_hyd	Number of hydrophobic atoms.	MOE	4	4	4	5	10	3	6	7	7	7	7	4	6	5	6	4	5	6	12	10	4	4	4	5	10	3	6	7	7	7	7	4	6	5	6	4	5	6	12
162	a_IC	Atom information content (total). This is calculated to be a_ICM times n.	MOE	38.758	44.755	44.782	48.891	52.691	34.855	52.62	49.657	55.047	49.657	52.452	47.87	44.324	51.848	61.417	41.51	45.428	46.127	60.45	55.954	44.755	48.812	50.897	55.166	59.199	40.655	58.993	56.122	61.64	56.122	56.825	54.094	50.548	58.222	68.089	47.567	51.651	52.452	67.122
163	a_ICM	Atom information content (mean). This is the entropy of the element distribution in the molecule (including implicit hydrogens but not lone pair pseudo-atoms). Let ni be the number of occurrences of atomic number i in the molecule. Let pi = ni / n where n is the sum of the ni. The value of a_ICM is the negative of the sum over all i of pi log pi.	MOE	1.6851	1.8648	1.7913	1.7461	1.5967	1.7427	1.754	1.5518	1.5728	1.5518	1.7484	1.773	1.6416	1.7283	1.6599	1.7296	1.6825	1.5906	1.6338	1.6457	1.8648	1.9525	1.9576	1.9023	1.7412	1.9359	1.903	1.7007	1.7122	1.7007	1.8331	1.9319	1.8053	1.8781	1.7918	1.9027	1.8447	1.7484	1.7664
164	a_nC	Number of carbon atoms: #{Zi | Zi = 6}.	MOE	6	6	7	8	12	5	9	9	9	9	8	7	8	8	9	6	7	8	14	12	6	6	7	8	12	5	9	9	9	9	8	7	8	8	9	6	7	8	14
165	a_nH	Number of hydrogen atoms (including implicit hydrogens). This is calculated as the sum of hi over all non-trivial atoms i plus the number of non-trivial hydrogen atoms.	MOE	12	12	11	13	16	10	14	18	20	18	16	13	14	15	20	12	14	16	17	16	12	12	11	13	16	10	14	18	20	18	16	13	14	15	20	12	14	16	17
166	a_nN	Number of nitrogen atoms: #{Zi | Zi = 7}.	MOE	2	2	2	2	2	2	4	2	3	2	2	3	2	3	5	2	2	2	3	2	2	2	2	2	2	2	4	2	3	2	2	3	2	3	5	2	2	2	3
167	a_nO	Number of oxygen atoms: #{Zi | Zi = 8}.	MOE	3	3	5	5	3	3	3	3	3	3	3	4	3	4	3	4	4	3	3	4	3	3	5	5	3	3	3	3	3	3	3	4	3	4	3	4	4	3	3
168	a_nS	Number of sulfur atoms: #{Zi | Zi = 16}.	MOE	0	1	0	0	0	0	0	0	0	0	1	0	0	0	0	0	0	0	0	0	1	2	1	1	1	1	1	1	1	1	2	1	1	1	1	1	1	1	1
169	balabanJ	Balaban's connectivity topological index [Balaban 1982].	MOE	3.6848	3.8744	4.1103	4.0579	2.4259	3.3326	2.4112	4.2436	3.9402	4.1045	3.9676	4.1103	2.5828	4.0579	3.9178	3.8744	4.1215	4.1215	1.9142	2.4341	3.7702	3.9466	4.1724	4.1301	2.4398	3.4549	2.4241	4.2935	4.0233	4.1677	4.0446	4.1724	2.5798	4.1301	4.0026	3.9466	4.1748	4.1748	1.9079
170	BCUT_PEOE_0	The BCUT descriptors [Pearlman 1998] are calculated from the eigenvalues of a modified adjacency matrix. Each ij entry of the adjacency matrix takes the value 1/sqrt(bij) where bij is the formal bond order between bonded atoms i and j. The diagonal takes the value of the PEOE partial charges. The resulting eigenvalues are sorted and the smallest, 1/3-ile, 2/3-ile and largest eigenvalues are reported.	MOE	-2.5487	-2.5512	-2.5562	-2.5957	-2.5625	-2.5129	-2.553	-2.6835	-2.662	-2.6765	-2.5993	-2.5515	-2.5505	-2.5908	-2.6305	-2.5486	-2.5895	-2.6488	-2.5578	-2.5623	-2.5509	-2.5533	-2.558	-2.5964	-2.564	-2.5175	-2.555	-2.6835	-2.6621	-2.6765	-2.5998	-2.5536	-2.553	-2.5915	-2.6307	-2.5508	-2.5902	-2.6489	-2.5595
171	BCUT_PEOE_1	The BCUT descriptors [Pearlman 1998] are calculated from the eigenvalues of a modified adjacency matrix. Each ij entry of the adjacency matrix takes the value 1/sqrt(bij) where bij is the formal bond order between bonded atoms i and j. The diagonal takes the value of the PEOE partial charges. The resulting eigenvalues are sorted and the smallest, 1/3-ile, 2/3-ile and largest eigenvalues are reported.	MOE	-0.5092	-0.5416	-0.5497	-0.5498	-0.5536	-0.5135	-0.5533	-0.4659	-0.5085	-0.4998	-0.4695	-0.5355	-0.5149	-0.5455	-0.5477	-0.5381	-0.4911	-0.4705	-0.5477	-0.5584	-0.5461	-0.5477	-0.5491	-0.5491	-0.5477	-0.5759	-0.5477	-0.5005	-0.5358	-0.5289	-0.5314	-0.5477	-0.5477	-0.5477	-0.5417	-0.5476	-0.5476	-0.5041	-0.5482
172	BCUT_PEOE_2	The BCUT descriptors [Pearlman 1998] are calculated from the eigenvalues of a modified adjacency matrix. Each ij entry of the adjacency matrix takes the value 1/sqrt(bij) where bij is the formal bond order between bonded atoms i and j. The diagonal takes the value of the PEOE partial charges. The resulting eigenvalues are sorted and the smallest, 1/3-ile, 2/3-ile and largest eigenvalues are reported.	MOE	0.3764	0.4408	0.3914	0.3914	0.5582	0.3806	0.4474	0.3748	0.3761	0.3761	0.3608	0.4131	0.4075	0.4159	0.3914	0.4428	0.4276	0.3749	0.5646	0.5478	0.4442	0.5372	0.4839	0.4882	0.5843	0.4939	0.5493	0.4237	0.4398	0.4375	0.3909	0.4573	0.5271	0.4649	0.4888	0.5424	0.5008	0.4252	0.5899
173	BCUT_PEOE_3	The BCUT descriptors [Pearlman 1998] are calculated from the eigenvalues of a modified adjacency matrix. Each ij entry of the adjacency matrix takes the value 1/sqrt(bij) where bij is the formal bond order between bonded atoms i and j. The diagonal takes the value of the PEOE partial charges. The resulting eigenvalues are sorted and the smallest, 1/3-ile, 2/3-ile and largest eigenvalues are reported.	MOE	2.5869	2.5911	2.5932	2.6075	2.5942	2.5731	2.5961	2.6555	2.6507	2.6439	2.6083	2.6014	2.7195	2.6156	2.6332	2.5935	2.6155	2.6324	2.5952	2.5943	2.6031	2.6065	2.6082	2.6192	2.6089	2.5918	2.6104	2.6598	2.6543	2.6488	2.6198	2.6146	2.7228	2.6255	2.6394	2.6085	2.6259	2.6396	2.6097
174	BCUT_SLOGP_0	The BCUT descriptors using atomic contribution to logP (using the Wildman and Crippen SlogP method) instead of partial charge.	MOE	-3.3053	-3.3055	-3.3054	-3.3054	-3.3054	-3.3051	-3.3054	-3.3055	-3.3054	-3.3054	-3.3054	-3.3054	-3.3034	-3.3054	-3.3054	-3.3055	-3.3056	-3.3055	-3.3054	-3.3054	-3.3143	-3.3144	-3.3143	-3.3143	-3.3144	-3.3141	-3.3144	-3.3144	-3.3144	-3.3143	-3.3143	-3.3143	-3.3123	-3.3143	-3.3143	-3.3145	-3.3145	-3.3144	-3.3144
175	BCUT_SLOGP_1	The BCUT descriptors using atomic contribution to logP (using the Wildman and Crippen SlogP method) instead of partial charge.	MOE	-0.4343	-0.5379	-0.4662	-0.4635	-0.4884	-0.4559	-0.4968	-0.4222	-0.4319	-0.4307	-0.4009	-0.5002	-0.4779	-0.4969	-0.4632	-0.5452	-0.5037	-0.4234	-0.4636	-0.5381	-0.5342	-0.6685	-0.6133	-0.6004	-0.5381	-0.5493	-0.5627	-0.4828	-0.5195	-0.4822	-0.4557	-0.5739	-0.5484	-0.5643	-0.5881	-0.6774	-0.5844	-0.4918	-0.591
176	BCUT_SLOGP_2	The BCUT descriptors using atomic contribution to logP (using the Wildman and Crippen SlogP method) instead of partial charge.	MOE	0.2971	0.3849	0.3385	0.3385	0.5471	0.2976	0.4384	0.297	0.2971	0.297	0.3583	0.356	0.295	0.3567	0.3385	0.3836	0.3709	0.297	0.6576	0.4815	0.3864	0.4773	0.4294	0.4321	0.6785	0.4219	0.4863	0.3801	0.3851	0.3849	0.3979	0.391	0.4547	0.393	0.4322	0.4735	0.4357	0.3803	0.6911
177	BCUT_SLOGP_3	The BCUT descriptors using atomic contribution to logP (using the Wildman and Crippen SlogP method) instead of partial charge.	MOE	2.3832	2.3744	2.3858	2.5356	2.4322	2.3362	2.3965	2.683	2.6641	2.6891	2.5454	2.392	2.6848	2.5404	2.592	2.3485	2.4396	2.6127	2.456	2.447	2.3778	2.3685	2.3807	2.5351	2.4307	2.3269	2.3929	2.6829	2.664	2.689	2.545	2.3875	2.6842	2.54	2.5918	2.3394	2.4382	2.6125	2.4552
178	BCUT_SMR_0	The BCUT descriptors using atomic contribution to molar refractivity (using the Wildman and Crippen SMR method) instead of partial charge.	MOE	-2.3393	-2.3377	-2.3407	-2.3577	-2.3426	-2.3279	-2.3417	-2.4312	-2.4347	-2.4247	-2.355	-2.3418	-2.3426	-2.3594	-2.3896	-2.3413	-2.3615	-2.3989	-2.3418	-2.3426	-2.3319	-2.33	-2.3335	-2.3528	-2.3356	-2.3193	-2.3345	-2.4304	-2.4343	-2.4239	-2.3497	-2.3347	-2.335	-2.3547	-2.3878	-2.3341	-2.357	-2.3972	-2.3347
179	BCUT_SMR_1	The BCUT descriptors using atomic contribution to molar refractivity (using the Wildman and Crippen SMR method) instead of partial charge.	MOE	-0.1776	-0.3115	-0.2658	-0.2658	-0.4042	-0.1826	-0.3235	-0.1751	-0.1772	-0.1771	-0.0886	-0.2926	-0.1249	-0.2954	-0.2658	-0.3165	-0.2889	-0.1753	-0.4054	-0.4326	-0.317	-0.3964	-0.362	-0.362	-0.4204	-0.3335	-0.42	-0.2941	-0.3129	-0.31	-0.2637	-0.3463	-0.3593	-0.3545	-0.362	-0.4068	-0.3408	-0.2961	-0.4151
180	BCUT_SMR_2	The BCUT descriptors using atomic contribution to molar refractivity (using the Wildman and Crippen SMR method) instead of partial charge.	MOE	0.3607	0.5559	0.4874	0.4874	0.7056	0.3687	0.5894	0.3578	0.3602	0.3601	0.4653	0.5315	0.2911	0.5375	0.4874	0.554	0.525	0.358	0.7177	0.7073	0.5586	0.6841	0.6178	0.6189	0.7254	0.6065	0.6958	0.5294	0.5521	0.5477	0.4855	0.6193	0.6462	0.6373	0.619	0.6794	0.6052	0.5318	0.7482
181	BCUT_SMR_3	The BCUT descriptors using atomic contribution to molar refractivity (using the Wildman and Crippen SMR method) instead of partial charge.	MOE	2.793	2.8172	2.8105	2.8397	2.8149	2.758	2.8119	2.9034	2.8812	2.8909	2.8592	2.8179	2.9733	2.8454	2.8684	2.7994	2.8366	2.8748	2.8184	2.8151	2.8208	2.8369	2.8322	2.8528	2.8349	2.7989	2.833	2.9075	2.8862	2.8954	2.8679	2.8372	2.9769	2.8571	2.8754	2.825	2.8508	2.882	2.8371
182	bpol	Sum of the absolute value of the difference between atomic polarizabilities of all bonded atoms in the molecule (including implicit hydrogens) with polarizabilities taken from [CRC 1994].	MOE	15.332	17.612	16.155	18.342	19.705	13.146	19.499	21.892	22.758	21.892	21.985	16.724	18.839	18.91	23.418	15.332	17.519	19.705	21.459	19.705	17.612	19.892	18.435	20.622	21.985	15.426	21.779	24.172	25.038	24.172	24.265	19.004	21.119	21.19	25.698	17.612	19.799	21.985	23.739
183	b_1rotN	Number of rotatable single bonds. Conjugated single bonds are not included (e.g. ester and peptide bonds).	MOE	3	4	5	6	5	3	5	5	7	5	6	5	3	6	7	4	4	4	5	5	4	5	6	7	6	4	6	6	8	6	7	6	4	7	8	5	5	5	6
184	b_1rotR	Fraction of rotatable single bonds: b_1rotN divided by b_heavy.	MOE	0.3	0.3636	0.3846	0.4286	0.2941	0.3333	0.3125	0.3846	0.5	0.3846	0.4615	0.3846	0.2308	0.4286	0.4375	0.3636	0.3333	0.3333	0.2381	0.2778	0.3636	0.4167	0.4286	0.4667	0.3333	0.4	0.3529	0.4286	0.5333	0.4286	0.5	0.4286	0.2857	0.4667	0.4706	0.4167	0.3846	0.3846	0.2727
185	b_ar	Number of aromatic bonds.	MOE	0	0	0	0	6	0	5	0	0	0	0	0	0	0	0	0	0	0	10	6	0	0	0	0	6	0	5	0	0	0	0	0	0	0	0	0	0	0	10
186	b_count	Number of bonds (including implicit hydrogens). This is calculated as the sum of (di/2 + hi) over all non-trivial atoms i.	MOE	22	23	24	27	33	19	30	31	34	31	29	26	27	29	36	23	26	28	38	34	23	24	25	28	34	20	31	32	35	32	30	27	28	30	37	24	27	29	39
187	b_double	Number of double bonds. Aromatic bonds are not considered to be double bonds.	MOE	2	2	3	3	2	2	2	2	2	2	2	3	2	3	3	2	2	2	2	2	2	2	3	3	2	2	2	2	2	2	2	3	2	3	3	2	2	2	2
188	b_heavy	Number of bonds between heavy atoms.	MOE	10	11	13	14	17	9	16	13	14	13	13	13	13	14	16	11	12	12	21	18	11	12	14	15	18	10	17	14	15	14	14	14	14	15	17	12	13	13	22
189	b_max1len	Code Class Description<br>Maximum single-bond chain length	MOE	4	5	4	5	4	4	4	6	8	6	7	4	4	5	7	5	5	5	4	4	5	5	5	5	5	5	5	6	8	6	7	5	5	5	7	5	5	5	5
190	b_rotN	Number of rotatable bonds. A bond is rotatable if it has order 1, is not in a ring, and has at least two heavy neighbors.	MOE	4	5	6	7	6	4	6	6	8	6	7	6	3	7	9	5	5	5	6	6	5	6	7	8	7	5	7	7	9	7	8	7	4	8	10	6	6	6	7
191	b_rotR	Fraction of rotatable bonds: b_rotN divided by b_heavy.	MOE	0.4	0.4545	0.4615	0.5	0.3529	0.4444	0.375	0.4615	0.5714	0.4615	0.5385	0.4615	0.2308	0.5	0.5625	0.4545	0.4167	0.4167	0.2857	0.3333	0.4545	0.5	0.5	0.5333	0.3889	0.5	0.4118	0.5	0.6	0.5	0.5714	0.5	0.2857	0.5333	0.5882	0.5	0.4615	0.4615	0.3182
192	b_single	Number of single bonds (including implicit hydrogens). Aromatic bonds are not considered to be single bonds.	MOE	20	21	21	24	25	17	23	29	32	29	27	23	25	26	33	21	24	26	26	26	21	22	22	25	26	18	24	30	33	30	28	24	26	27	34	22	25	27	27
193	chi0	Atomic connectivity index (order 0) from [Hall 1991] and [Hall 1977]. This is calculated as the sum of 1/sqrt(di) over all heavy atoms i with di > 0.	MOE	9.0165	9.7236	11.301	12.008	12.836	8.1463	12.129	11.301	11.845	11.301	11.138	11.301	10.008	12.008	13.422	9.7236	10.594	10.594	14.698	13.707	9.7236	10.431	12.008	12.715	13.544	8.8534	12.836	12.008	12.552	12.008	11.845	12.008	10.715	12.715	14.129	10.431	11.301	11.301	15.405
194	chi0v	Atomic valence connectivity index (order 0) from [Hall 1991] and [Hall 1977]. This is calculated as the sum of 1/sqrt(vi) over all heavy atoms i with vi > 0.	MOE	6.5866	7.6353	7.6102	8.3173	9.6804	5.7163	8.8956	8.871	9.1221	8.871	9.2255	7.7793	7.6551	8.4864	9.8626	6.7409	7.6111	8.1639	11.18	10.05	7.6353	8.684	8.6589	9.366	10.729	6.7651	9.9443	9.9198	10.171	9.9198	10.274	8.828	8.7038	9.5351	10.911	7.7896	8.6599	9.2127	12.229
195	chi0v_C	Carbon valence connectivity index (order 0). This is calculated as the sum of 1/sqrt(vi) over all carbon atoms i with vi > 0.	MOE	4.1547	3.8618	4.3618	5.0689	7.2486	3.2845	5.5165	6.4392	5.9831	6.4392	5.5689	4.3618	5.276	5.0689	5.776	3.8618	4.7321	5.7321	8.2486	7.1712	3.8618	3.5689	4.0689	4.776	6.9557	2.9916	5.2236	6.1463	5.6902	6.1463	5.276	4.0689	4.9831	4.776	5.4831	3.5689	4.4392	5.4392	7.9557
196	chi0_C	Carbon connectivity index (order 0). This is calculated as the sum of 1/sqrt(di) over all carbon atoms i with di > 0.	MOE	4.3094	4.0165	4.5939	5.301	8.1294	3.4392	6.0081	6.5939	6.1378	6.5939	5.7236	4.5939	5.4307	5.301	6.0081	4.0165	4.8868	5.8868	9.2841	7.9996	4.0165	3.7236	4.301	5.0081	7.8365	3.1463	5.7152	6.301	5.8449	6.301	5.4307	4.301	5.1378	5.0081	5.7152	3.7236	4.5939	5.5939	8.9912
197	chi1	Atomic connectivity index (order 1) from [Hall 1991] and [Hall 1977]. This is calculated as the sum of 1/sqrt(didj) over all bonds between heavy atoms i and j where i < j.	MOE	4.9473	5.4853	6.3411	6.8411	8.0029	4.5366	7.5029	6.396	6.9853	6.3411	6.4853	6.3411	6.0366	6.8411	7.8411	5.4853	5.8579	5.8579	9.4861	8.3968	5.4853	6.0233	6.8791	7.3791	8.5409	5.0746	8.0409	6.934	7.5233	6.8791	7.0233	6.8791	6.5746	7.3791	8.3791	6.0233	6.396	6.396	10.024
198	chi1v	Atomic valence connectivity index (order 1) from [Hall 1991] and [Hall 1977]. This is calculated as the sum of 1/sqrt(vivj) over all bonds between heavy atoms i and j where i < j.	MOE	3.2913	4.0709	3.884	4.384	5.3865	2.8438	4.8236	4.74	5.1222	4.6852	5.713	3.9686	4.3816	4.4686	5.3031	3.4385	3.8829	4.202	6.3805	5.5208	4.0709	4.8505	4.6636	5.1636	6.1661	3.6233	5.6032	5.5196	5.9018	5.4648	6.4926	4.7482	5.1612	5.2482	6.0827	4.2181	4.6625	4.9816	7.1601
199	chi1v_C	Carbon valence connectivity index (order 1). This is calculated as the sum of 1/sqrt(vivj) over all bonds between carbon atoms i and j where i < j.	MOE	1.7321	1.5629	1.9165	2.4165	3.8272	1.2196	2.2052	3.1807	3.0629	3.1259	2.0629	1.9165	2.5629	2.4165	2.5629	1.5629	2.0654	2.6427	4.2825	3.7379	1.5629	1.3938	1.7474	2.2474	3.6581	1.0505	2.0361	3.0116	2.8938	2.9568	1.8938	1.7474	2.3938	2.2474	2.3938	1.3938	1.8963	2.4736	4.1134
200	chi1_C	Carbon connectivity index (order 1). This is calculated as the sum of 1/sqrt(didj) over all bonds between carbon atoms i and j where i < j.	MOE	1.8214	1.6523	2.0605	2.5605	4.877	1.3189	2.4688	3.2701	3.1523	3.2152	2.1523	2.0605	2.6523	2.5605	2.6523	1.6523	2.1547	2.7321	5.4519	4.6935	1.6523	1.4832	1.8914	2.3914	4.7079	1.1498	2.2997	3.101	2.9832	3.0461	1.9832	1.8914	2.4832	2.3914	2.4832	1.4832	1.9856	2.5629	5.2828
201	chiral	The number of chiral centers.	MOE	2	2	2	2	2	1	2	3	2	2	2	2	2	2	2	2	3	2	2	2	2	2	2	2	2	1	2	3	2	2	2	2	2	2	2	2	3	2	2
202	chiral_u	The number of unconstrained chiral centers.	MOE	2	2	2	2	2	1	2	3	2	2	2	2	2	2	2	2	3	2	2	2	2	2	2	2	2	1	2	3	2	2	2	2	2	2	2	2	3	2	2
203	density	Molecular mass density: Weight divided by vdw_vol (amu/Å3).	MOE	0.7937	0.8616	0.8775	0.8485	0.7459	0.824	0.805	0.7352	0.7433	0.7352	0.8068	0.8394	0.7812	0.8151	0.7751	0.8376	0.8102	0.7509	0.7603	0.7755	0.8616	0.9176	0.9303	0.8989	0.7937	0.8969	0.8543	0.7905	0.7948	0.7905	0.8573	0.8932	0.8405	0.8661	0.8209	0.899	0.868	0.81	0.8016
204	diameter	Largest value in the distance matrix [Petitjean 1992].	MOE	6	6	7	8	9	6	8	7	9	7	8	7	6	8	10	6	6	6	10	10	7	7	8	9	10	7	9	8	10	8	9	8	7	9	11	7	7	7	11
205	FCharge	Total charge of the molecule (sum of formal charges).	MOE	0	0	-1	-1	0	0	0	0	1	0	0	0	0	0	1	0	0	0	0	0	0	0	-1	-1	0	0	0	0	1	0	0	0	0	0	1	0	0	0	0

Table S12 Amino acid descriptors (2D) used in this study.

			1st aa	A	A	A	A	A	A	A	A	A	A	A	A	A	A	A	A	A	A	A	A	C	C	C	C	C	C	C	C	C	C	C	C	C	C	C	C	C	C	C
			2nd aa	A	C	D	E	F	G	H	I	K	L	M	N	P	Q	R	S	T	V	W	Y	A	C	D	E	F	G	H	I	K	L	M	N	P	Q	R	S	T	V	W
No.	Descriptor ID	Attribute	Ref*	AA	AC	AD	AE	AF	AG	AH	AI	AK	AL	AM	AN	AP	AQ	AR	AS	AT	AV	AW	AY	CA	CC	CD	CE	CF	CG	CH	CI	CK	CL	CM	CN	CP	CQ	CR	CS	CT	CV	CW
206	GCUT_PEOE_0	The GCUT descriptors are calculated from the eigenvalues of a modified graph distance adjacency matrix. Each ij entry of the adjacency matrix takes the value 1/sqr(dij) where dij is the (modified) graph distance between atoms i and j. The diagonal takes the value of the PEOE partial charges. The resulting eigenvalues are sorted and the smallest, 1/3-ile, 2/3-ile and largest eigenvalues are reported.	MOE	-0.8479	-0.8473	-0.8505	-0.8543	-0.8476	-0.8486	-0.8489	-0.8476	-0.863	-0.8478	-0.8477	-0.8474	-0.8518	-0.8478	-0.8478	-0.8467	-0.8467	-0.8476	-0.863	-0.8476	-0.8467	-0.8459	-0.8505	-0.8543	-0.8463	-0.8475	-0.8487	-0.8464	-0.863	-0.8466	-0.8464	-0.8461	-0.851	-0.8466	-0.8466	-0.8452	-0.8451	-0.8463	-0.863
207	GCUT_PEOE_1	The GCUT descriptors are calculated from the eigenvalues of a modified graph distance adjacency matrix. Each ij entry of the adjacency matrix takes the value 1/sqr(dij) where dij is the (modified) graph distance between atoms i and j. The diagonal takes the value of the PEOE partial charges. The resulting eigenvalues are sorted and the smallest, 1/3-ile, 2/3-ile and largest eigenvalues are reported.	MOE	-0.3747	-0.3997	-0.5272	-0.5166	-0.3885	-0.424	-0.3893	-0.3387	-0.3588	-0.3526	-0.3533	-0.4179	-0.3927	-0.4102	-0.3676	-0.3986	-0.3816	-0.3407	-0.3839	-0.3834	-0.4163	-0.4644	-0.5084	-0.501	-0.39	-0.464	-0.4013	-0.3778	-0.383	-0.3865	-0.3787	-0.4652	-0.4651	-0.4651	-0.4053	-0.4648	-0.4599	-0.3814	-0.4375
208	GCUT_PEOE_2	The GCUT descriptors are calculated from the eigenvalues of a modified graph distance adjacency matrix. Each ij entry of the adjacency matrix takes the value 1/sqr(dij) where dij is the (modified) graph distance between atoms i and j. The diagonal takes the value of the PEOE partial charges. The resulting eigenvalues are sorted and the smallest, 1/3-ile, 2/3-ile and largest eigenvalues are reported.	MOE	0.1467	0.1855	0.1291	0.0997	0.1147	0.1644	0.182	0.1062	0.1849	0.076	0.1074	0.1197	0.1424	0.11	0.1855	0.1855	0.1713	0.1191	0.1852	0.1855	0.1758	0.1856	0.1573	0.147	0.1707	0.1774	0.1812	0.1408	0.1849	0.1106	0.1705	0.171	0.1856	0.1706	0.1804	0.1856	0.176	0.1534	0.1805
209	GCUT_PEOE_3	The GCUT descriptors are calculated from the eigenvalues of a modified graph distance adjacency matrix. Each ij entry of the adjacency matrix takes the value 1/sqr(dij) where dij is the (modified) graph distance between atoms i and j. The diagonal takes the value of the PEOE partial charges. The resulting eigenvalues are sorted and the smallest, 1/3-ile, 2/3-ile and largest eigenvalues are reported.	MOE	2.25	2.2707	2.2892	2.3581	2.4397	2.1627	2.3912	2.4898	2.5049	2.4698	2.3789	2.334	2.493	2.3961	2.4939	2.273	2.3545	2.4182	2.5334	2.4518	2.2776	2.2972	2.3147	2.3799	2.4572	2.1953	2.411	2.5061	2.5195	2.4863	2.3996	2.3569	2.5113	2.4159	2.509	2.2994	2.3765	2.4373	2.5467
210	GCUT_SLOGP_0	The GCUT descriptors using atomic contribution to logP (using the Wildman and Crippen SlogP method) instead of partial charge.	MOE	-2.2349	-2.2349	-2.235	-2.235	-2.235	-2.2349	-2.235	-2.235	-2.2353	-2.235	-2.235	-2.235	-2.235	-2.235	-2.235	-2.2349	-2.235	-2.235	-2.235	-2.235	-2.2351	-2.2351	-2.2351	-2.2351	-2.2351	-2.2351	-2.2351	-2.2351	-2.2354	-2.2351	-2.2351	-2.2351	-2.2352	-2.2351	-2.2352	-2.2351	-2.2351	-2.2351	-2.2351
211	GCUT_SLOGP_1	The GCUT descriptors using atomic contribution to logP (using the Wildman and Crippen SlogP method) instead of partial charge.	MOE	-0.4097	-0.4361	-0.52	-0.4527	-0.404	-0.4725	-0.4125	-0.3699	-0.3657	-0.3674	-0.3724	-0.4509	-0.4086	-0.4266	-0.4126	-0.4812	-0.4416	-0.3771	-0.3976	-0.4078	-0.4475	-0.4505	-0.5063	-0.4631	-0.4387	-0.4772	-0.4417	-0.4294	-0.4092	-0.4202	-0.4369	-0.4615	-0.4419	-0.4495	-0.4414	-0.4682	-0.4611	-0.4428	-0.4257
212	GCUT_SLOGP_2	The GCUT descriptors using atomic contribution to logP (using the Wildman and Crippen SlogP method) instead of partial charge.	MOE	0.0542	0.0555	0.0542	0.0542	0.0848	0.0542	0.0555	0.0542	0.0542	0.0542	0.0555	0.0542	0.0542	0.0542	0.0542	0.0542	0.0542	0.0542	0.1232	0.0581	0.0557	0.058	0.0561	0.0567	0.1383	0.0683	0.0582	0.0546	0.0555	0.0555	0.058	0.0542	0.0542	0.0542	0.0568	0.0542	0.0542	0.0546	0.1361
213	GCUT_SLOGP_3	The GCUT descriptors using atomic contribution to logP (using the Wildman and Crippen SlogP method) instead of partial charge.	MOE	2.0683	2.0921	2.1006	2.2237	2.3862	1.9424	2.2578	2.4289	2.416	2.4161	2.2792	2.1387	2.3946	2.2575	2.3586	2.054	2.173	2.3199	2.5411	2.4163	2.0941	2.1162	2.1245	2.2403	2.3961	1.9786	2.2726	2.4388	2.4256	2.4258	2.2931	2.1603	2.4076	2.2727	2.3702	2.0814	2.1925	2.3329	2.5475
214	GCUT_SMR_0	The GCUT descriptors using atomic contribution to molar refractivity (using the Wildman and Crippen SMR method) instead of partial charge.	MOE	-0.6408	-0.6408	-0.6408	-0.6408	-0.6408	-0.6408	-0.6408	-0.6408	-0.6418	-0.6408	-0.6408	-0.6408	-0.6409	-0.6408	-0.6408	-0.6408	-0.6408	-0.6408	-0.6408	-0.6408	-0.6407	-0.6407	-0.6407	-0.6407	-0.6407	-0.6407	-0.6407	-0.6407	-0.6418	-0.6407	-0.6407	-0.6407	-0.6408	-0.6407	-0.6407	-0.6407	-0.6407	-0.6407	-0.6407
215	GCUT_SMR_1	The GCUT descriptors using atomic contribution to molar refractivity (using the Wildman and Crippen SMR method) instead of partial charge.	MOE	-0.1887	-0.1743	-0.1994	-0.1985	-0.2081	-0.1963	-0.2092	-0.1779	-0.1855	-0.1832	-0.1733	-0.2116	-0.1861	-0.2115	-0.1988	-0.2131	-0.1989	-0.1794	-0.2053	-0.2161	-0.1744	-0.16	-0.1864	-0.1859	-0.2042	-0.178	-0.2038	-0.1691	-0.1728	-0.1717	-0.16	-0.1982	-0.16	-0.198	-0.186	-0.1994	-0.1845	-0.1698	-0.1973
216	GCUT_SMR_2	The GCUT descriptors using atomic contribution to molar refractivity (using the Wildman and Crippen SMR method) instead of partial charge.	MOE	0.0578	0.1316	0.1121	0.1122	0.1624	0.0669	0.1323	0.0506	0.0567	0.0565	0.1311	0.1304	0.0189	0.1311	0.1122	0.1308	0.1167	0.0511	0.1673	0.1721	0.1313	0.1637	0.1458	0.1458	0.1664	0.1551	0.1644	0.1162	0.1285	0.1269	0.1626	0.1614	0.1567	0.1626	0.1458	0.162	0.1411	0.1174	0.1697
217	GCUT_SMR_3	The GCUT descriptors using atomic contribution to molar refractivity (using the Wildman and Crippen SMR method) instead of partial charge.	MOE	2.4416	2.4863	2.4999	2.5734	2.6803	2.338	2.6117	2.7026	2.6978	2.6813	2.6082	2.537	2.7059	2.6042	2.6965	2.4633	2.5529	2.627	2.803	2.6924	2.4898	2.5291	2.5416	2.608	2.7055	2.3989	2.6424	2.7282	2.7224	2.7075	2.6394	2.575	2.7349	2.6361	2.7211	2.5091	2.5897	2.6578	2.8202
218	Kier1	First kappa shape index: (n-1)2 / m2 [Hall 1991].	MOE	11	12	14	15	15.059	10	14.063	14	15	14	14	14	11.077	15	17	12	13	13	16.372	16.056	12	13	15	16	16.056	11	15.059	15	16	15	15	15	12.071	16	18	13	14	14	17.355
219	Kier2	Second kappa shape index: (n-1)2 / m2 [Hall 1991].	MOE	4.7929	5.6122	6.4775	7.3025	7.438	4.7603	6.6667	6.4775	8.1869	6.4775	7.3125	6.4775	4.4815	7.3025	9	5.6122	5.6719	5.6719	7.3199	7.5556	5.6122	6.4533	7.3025	8.144	8.2268	5.625	7.438	7.3025	9.0741	7.3025	8.1869	7.3025	5.1856	8.144	9.8685	6.4533	6.4775	6.4775	8.0222
220	Kier3	Third kappa shape index: (n-1) (n-3)2 / p32 for odd n, and (n-3) (n-2)2 / p32 for even n [Hall 1991].	MOE	4.4444	4.5918	6.1875	6.9758	5.4444	5.5309	4.8166	4.8889	6.9758	6.1875	6.1875	6.1875	2.4793	6.9758	8.687	4.5918	4.6875	4.6875	4.25	5.6805	4.5918	4.6875	6.2222	7.0582	5.6805	5.2893	5.0176	5.04	7.0582	6.2222	6.2222	6.2222	2.75	7.0582	8.7075	4.6875	4.8889	4.8889	4.4875
221	KierA1	First alpha modified shape index: s (s-1)2 / m2 where s = n + a [Hall 1991].	MOE	8.7152	11.161	10.889	11.882	11.163	7.7182	10.323	11.709	12.708	11.709	13.166	10.442	9.1264	11.43	12.963	9.7128	10.711	10.711	11.837	12.119	11.161	13.742	13.279	14.28	13.309	10.158	12.457	14.167	15.169	14.167	15.735	12.792	11.352	13.791	15.309	12.164	13.166	13.166	13.833
222	KierA2	Second alpha modified shape index: s (s-1)2 / m2 where s = n + a [Hall 1991].	MOE	3.6993	5.1829	4.9315	5.6798	5.4009	3.5554	4.7832	5.3369	6.8472	5.3369	6.842	4.7112	3.6273	5.4465	6.7365	4.4465	4.5908	4.5908	5.1938	5.6007	5.1829	6.8549	6.4043	7.2096	6.7389	5.1497	6.0744	6.8672	8.5704	6.8672	8.6184	6.1515	4.852	6.9454	8.308	6.0023	6.0608	6.0608	6.3164
223	KierA3	Third alpha modified shape index: (s-1) (s-3)2 / p32 for odd n, and (s-3) (s-2)2 / p32 for even n where s = n + a [Hall 1991].	MOE	3.3505	4.2168	4.6339	5.3449	3.8885	4.0205	3.3967	3.9819	5.7733	5.0396	5.7671	4.414	1.9767	5.1118	6.4062	3.5772	3.7372	3.7372	2.9743	4.1544	4.2168	4.9996	5.4153	6.2103	4.6122	4.8049	4.056	4.7229	6.4676	5.8307	6.5692	5.1889	2.5631	5.9713	7.2753	4.3379	4.5567	4.5567	3.5002
224	KierFlex	Kier molecular flexibility index: (KierA1) (KierA2) / n [Hall 1991].	MOE	2.9309	4.8206	3.8357	4.4992	3.5464	2.7441	3.086	4.4636	5.8009	4.4636	6.4342	3.5139	2.5465	4.1504	5.1369	3.599	3.7824	3.7824	3.0739	3.7709	4.8206	7.2462	5.6695	6.4344	4.9827	4.7557	4.451	6.486	8.1251	6.486	9.0404	5.2461	3.9342	5.9867	7.0661	5.6161	5.6995	5.6995	4.1607
225	lip_acc	The number of O and N atoms.	MOE	5	5	7	7	5	5	7	5	6	5	5	7	5	7	8	6	6	5	6	6	5	5	7	7	5	5	7	5	6	5	5	7	5	7	8	6	6	5	6
226	lip_don	The number of OH and NH atoms.	MOE	4	4	4	4	4	4	5	4	7	4	4	6	3	6	9	5	5	4	5	5	4	4	4	4	4	4	5	4	7	4	4	6	3	6	9	5	5	4	5
227	lip_druglike	One if and only if lip_violation < 2 otherwise zero.	MOE	1	1	1	1	1	1	1	1	1	1	1	1	1	1	1	1	1	1	1	1	1	1	1	1	1	1	1	1	1	1	1	1	1	1	1	1	1	1	1
228	lip_violation	The number of violations of Lipinski's Rule of Five [Lipinski 1997].	MOE	0	0	0	0	0	0	0	0	1	0	0	1	0	1	1	0	0	0	0	0	0	0	0	0	0	0	0	0	1	0	0	1	0	1	1	0	0	0	0
229	logP(o/w)	Log of the octanol/water partition coefficient (including implicit hydrogens). This property is calculated from a linear atom type model [LOGP 1998] with r2 = 0.931, RMSE=0.393 on 1,827 molecules.	MOE	-0.949	-0.839	-1.576	-1.134	0.586	-1.411	-1.718	0.465	-0.88	0.465	-0.294	-2.431	-0.561	-1.989	-1.301	-1.984	-1.522	0.023	0.952	0.278	-0.839	-0.729	-1.466	-1.024	0.696	-1.301	-1.608	0.575	-0.77	0.575	-0.184	-2.321	-0.451	-1.879	-1.191	-1.874	-1.412	0.133	1.062
230	logS	Log of the aqueous solubility (mol/L). This property is calculated from an atom contribution linear atom type model [Hou 2004] with r2 = 0.90, ~1,200 molecules.	MOE	-0.3408	-1.002	-0.1518	-0.3536	-1.843	-0.0136	-0.6001	-1.2596	-0.2859	-1.573	-1.2072	-0.1741	-0.46	-0.3758	-0.7133	0.189	-0.1382	-0.7443	-2.1328	-1.481	-1.002	-1.6633	-0.813	-1.0148	-2.5042	-0.6748	-1.2613	-1.9208	-0.9471	-2.2343	-1.8685	-0.8353	-1.1212	-1.0371	-1.3745	-0.4723	-0.7995	-1.4056	-2.7941
231	mr	Molecular refractivity (including implicit hydrogens). This property is calculated from an 11 descriptor linear model [MREF 1998] with r2 = 0.997, RMSE = 0.168 on 1,947 small molecules.	MOE	3.9234	4.673	4.5674	5.0477	6.4719	3.4722	5.6983	5.3254	5.6784	5.3154	5.6002	4.7536	4.6739	5.2319	6.1607	4.0797	4.5221	4.8471	7.5168	6.5984	4.673	5.3923	5.3188	5.8016	7.2294	4.2187	6.4538	6.0823	6.4372	6.0722	6.3244	5.5066	5.4269	5.9873	6.9207	4.8302	5.2752	5.6018	8.2769
232	opr_brigid	The number of rigid bonds from [Oprea 2000].	MOE	0	0	0	0	6	0	5	0	0	0	0	0	5	0	1	0	0	0	10	6	0	0	0	0	6	0	5	0	0	0	0	0	5	0	1	0	0	0	10
233	opr_leadlike	One if and only if opr_violation < 2 otherwise zero.	MOE	1	1	1	1	1	1	1	1	0	1	1	0	1	0	0	1	1	1	1	1	1	1	1	1	1	1	1	1	0	1	1	0	1	0	0	1	1	1	1
234	opr_nring	The number of ring bonds from [Oprea 2000].	MOE	0	0	0	0	1	0	1	0	0	0	0	0	1	0	0	0	0	0	2	1	0	0	0	0	1	0	1	0	0	0	0	0	1	0	0	0	0	0	2
235	opr_nrot	The number of rotatable bonds from [Oprea 2000].	MOE	3	4	5	6	5	3	5	5	7	5	6	5	3	6	8	4	4	4	5	5	4	5	6	7	6	4	6	6	8	6	7	6	4	7	9	5	5	5	6
236	opr_violation	The number of violations of Oprea's lead-like test [Oprea 2000].	MOE	0	0	1	1	0	1	0	0	2	0	0	2	0	2	2	1	1	0	0	0	0	0	1	1	0	1	0	0	2	0	0	2	0	2	2	1	1	0	0
237	PC+	Total positive partial charge	MOE	3.953	4.363	4.859	4.859	4.847	3.953	4.82	3.953	5.806	3.953	4.413	5.323	3.883	5.323	7.731	4.633	4.633	3.953	5.187	5.229	4.363	4.773	5.269	5.269	5.257	4.363	5.23	4.363	6.216	4.363	4.823	5.733	4.293	5.733	8.141	5.043	5.043	4.363	5.597
238	PC-	Total negative partial charge	MOE	-3.953	-4.363	-5.859	-5.859	-4.847	-3.953	-4.82	-3.953	-4.806	-3.953	-4.413	-5.323	-3.883	-5.323	-6.731	-4.633	-4.633	-3.953	-5.188	-5.229	-4.363	-4.773	-6.269	-6.269	-5.257	-4.363	-5.23	-4.363	-5.216	-4.363	-4.823	-5.733	-4.293	-5.733	-7.141	-5.043	-5.043	-4.363	-5.598
239	PEOE_PC+	Total positive partial charge: the sum of the positive qi. Q_PC+ is identical to PC+ which has been retained for compatibility.	MOE	2.0991	2.2344	2.1585	2.1923	2.4079	2.0627	2.6504	2.2539	3.4962	2.2541	2.2642	2.7021	2.1038	2.7216	3.9638	2.4497	2.4831	2.2011	2.6505	2.6413	2.267	2.4023	2.3264	2.3602	2.5758	2.2306	2.8183	2.4218	3.664	2.422	2.4321	2.87	2.2717	2.8895	4.1317	2.6176	2.651	2.369	2.8184
240	PEOE_PC-	Total negative partial charge: the sum of the negative qi. Q_PC- is identical to PC- which has been retained for compatibility.	MOE	-2.0991	-2.2344	-3.1585	-3.1923	-2.4079	-2.0627	-2.6504	-2.2539	-2.4962	-2.2541	-2.2642	-2.7021	-2.1038	-2.7216	-2.9638	-2.4497	-2.4831	-2.2011	-2.6505	-2.6413	-2.267	-2.4023	-3.3264	-3.3602	-2.5758	-2.2306	-2.8183	-2.4218	-2.664	-2.422	-2.4321	-2.87	-2.2717	-2.8895	-3.1317	-2.6176	-2.651	-2.369	-2.8184
241	PEOE_RPC+	Relative positive partial charge: the largest positive qi divided by the sum of the positive qi. Q_RPC+ is identical to RPC+ which has been retained for compatibility.	MOE	0.1646	0.1546	0.1601	0.1576	0.1435	0.1675	0.1304	0.1533	0.0988	0.1533	0.1526	0.1279	0.1642	0.127	0.0872	0.1411	0.1392	0.157	0.1304	0.1308	0.1524	0.1438	0.1485	0.1464	0.1342	0.1549	0.1226	0.1427	0.0943	0.1427	0.1421	0.1204	0.1521	0.1196	0.0836	0.132	0.1304	0.1459	0.1226
242	PEOE_RPC-	Relative negative partial charge: the smallest negative qi divided by the sum of the negative qi. Q_RPC- is identical to RPC- which has been retained for compatibility.	MOE	0.2609	0.2451	0.174	0.1722	0.2275	0.2657	0.2066	0.243	0.2194	0.243	0.2419	0.2027	0.2603	0.2012	0.1848	0.2235	0.2205	0.2488	0.2066	0.2074	0.2416	0.228	0.1653	0.1636	0.2126	0.2457	0.1943	0.2261	0.2056	0.2261	0.2252	0.1908	0.2411	0.1895	0.1749	0.2092	0.2066	0.2312	0.1943
243	PEOE_VSA+0	Sum of vi where qi is in the range [0.00,0.05).	MOE	25.592	32.004	44.737	44.566	21.327	12.796	46.774	51.185	38.388	51.185	42.654	21.327	51.581	38.559	29.858	12.796	25.592	42.654	46.981	21.327	21.327	27.739	40.471	40.301	17.062	8.5308	42.509	46.919	34.123	46.919	38.388	17.062	47.315	34.294	25.592	8.5308	21.327	38.388	42.716
244	PEOE_VSA+1	Sum of vi where qi is in the range [0.05,0.10).	MOE	22.12	22.12	22.12	22.12	43.447	36.432	54.322	22.12	43.843	22.12	22.12	30.821	22.12	22.12	43.843	45.602	31.29	22.12	43.447	47.64	32.797	32.797	32.797	32.797	54.124	47.109	64.999	32.797	54.52	32.797	32.797	41.499	32.797	32.797	54.52	56.279	41.968	32.797	54.124
245	PEOE_VSA+2	Sum of vi where qi is in the range [0.10,0.15).	MOE	4.2654	12.046	4.2654	4.2654	4.2654	4.2654	8.5308	4.2654	4.2654	4.2654	4.2654	4.2654	4.2654	4.2654	4.2654	4.2654	4.2654	4.2654	4.2654	4.2654	12.046	19.828	12.046	12.046	12.046	12.046	16.312	12.046	12.046	12.046	12.046	12.046	12.046	12.046	12.046	12.046	12.046	12.046	12.046
246	PEOE_VSA+3	Sum of vi where qi is in the range [0.15,0.20).	MOE	11.765	11.765	11.765	11.765	11.765	11.765	20.384	11.765	11.765	11.765	11.765	29.003	3.146	29.003	11.765	11.765	11.765	11.765	20.384	11.765	11.765	11.765	11.765	11.765	11.765	11.765	20.384	11.765	11.765	11.765	11.765	29.003	3.146	29.003	11.765	11.765	11.765	11.765	20.384
247	PEOE_VSA+4	Sum of vi where qi is in the range [0.20,0.25).	MOE	0	0	0	0	0	0	0	0	0	0	0	12.95	0	12.95	0	10.324	10.324	0	0	10.324	0	0	0	0	0	0	0	0	0	0	0	12.95	0	12.95	0	10.324	10.324	0	0
248	PEOE_VSA+5	Sum of vi where qi is in the range [0.25,0.30).	MOE	12.95	12.95	12.95	12.95	12.95	12.95	12.95	12.95	12.95	12.95	12.95	12.95	12.95	12.95	56.045	12.95	12.95	12.95	12.95	12.95	12.95	12.95	12.95	12.95	12.95	12.95	12.95	12.95	12.95	12.95	12.95	12.95	12.95	12.95	56.045	12.95	12.95	12.95	12.95
249	PEOE_VSA+6	Sum of vi where qi is greater than 0.3.	MOE	25.857	25.857	25.857	25.857	25.857	25.857	25.857	25.857	51.714	25.857	25.857	25.857	25.857	25.857	41.539	25.857	25.857	25.857	25.857	25.857	25.857	25.857	25.857	25.857	25.857	25.857	25.857	25.857	51.714	25.857	25.857	25.857	25.857	25.857	41.539	25.857	25.857	25.857	25.857
250	PEOE_VSA-0	Sum of vi where qi is in the range [-0.05,0.00).	MOE	37.495	18.748	18.748	36.15	29.658	18.748	18.748	18.748	44.852	27.449	58.85	18.748	36.15	27.449	36.15	18.748	37.495	18.748	41.912	54.167	18.748	0	0	17.403	10.91	0	0	0	26.104	8.7013	40.102	0	17.403	8.7013	17.403	0	18.748	0	23.165
251	PEOE_VSA-1	Sum of vi where qi is in the range [-0.10,-0.05).	MOE	0	0	0	0	61.275	0	0	46.197	0	37.495	0	0	0	0	0	0	0	37.495	36.765	24.51	0	0	0	0	61.275	0	0	46.197	0	37.495	0	0	0	0	0	0	0	37.495	36.765
252	PEOE_VSA-3	Sum of vi where qi is in the range [-0.20,-0.15).	MOE	0	26.363	0	0	0	0	0	0	0	0	0	19.761	0	0	0	0	0	0	0	0	26.363	52.726	26.363	26.363	26.363	26.363	26.363	26.363	26.363	26.363	26.363	46.124	26.363	26.363	26.363	26.363	26.363	26.363	26.363
253	PEOE_VSA-4	Sum of vi where qi is in the range [-0.25,-0.20).	MOE	0	0	0	0	0	0	5.6826	0	0	0	0	0	0	0	0	0	0	0	0	0	0	0	0	0	0	0	5.6826	0	0	0	0	0	0	0	0	0	0	0	0
254	PEOE_VSA-5	Sum of vi where qi is in the range [-0.30,-0.25).	MOE	13.567	13.567	13.567	13.567	13.567	13.567	13.567	13.567	13.567	13.567	13.567	27.134	13.567	27.134	27.006	13.567	13.567	13.567	13.567	13.567	13.567	13.567	13.567	13.567	13.567	13.567	13.567	13.567	13.567	13.567	13.567	27.134	13.567	27.134	27.006	13.567	13.567	13.567	13.567
255	PEOE_VSA-6	Sum of vi where qi is less than -0.30.	MOE	28.376	28.376	55.51	55.51	28.376	28.376	28.513	28.376	29.482	28.376	28.376	35.027	28.239	35.027	28.376	36.144	36.144	28.376	28.513	36.144	28.376	28.376	55.51	55.51	28.376	28.376	28.513	28.376	29.482	28.376	28.376	35.027	28.239	35.027	28.376	36.144	36.144	28.376	28.513
256	PEOE_VSA_FHYD	Fractional hydrophobic van der Waals surface area. This is the sum of the vi such that |qi| is less than or equal to 0.2 divided by the total surface area. The vi are calculated using a connection table approximation.	MOE	0.5563	0.6038	0.4851	0.5242	0.6802	0.5099	0.6321	0.6564	0.5706	0.6564	0.6638	0.4776	0.5926	0.5159	0.4514	0.4852	0.5276	0.6292	0.7055	0.6235	0.6038	0.6421	0.5336	0.566	0.7056	0.5672	0.6633	0.6856	0.6049	0.6856	0.6918	0.5251	0.6331	0.557	0.4912	0.5377	0.5722	0.663	0.7271
257	PEOE_VSA_FNEG	Fractional negative van der Waals surface area. This is the sum of the vi such that qi is negative divided by the total surface area. The vi are calculated using a connection table approximation.	MOE	0.4365	0.4272	0.4192	0.4641	0.5263	0.3684	0.2826	0.4548	0.3504	0.4548	0.502	0.371	0.394	0.3808	0.3283	0.3565	0.4168	0.4508	0.4397	0.4891	0.4272	0.4196	0.4126	0.454	0.5122	0.3661	0.2883	0.4458	0.3503	0.4458	0.4892	0.369	0.3895	0.3781	0.3298	0.3558	0.4104	0.4416	0.433
258	PEOE_VSA_FPNEG	Fractional negative polar van der Waals surface area. This is the sum of the vi such that qi is less than -0.2 divided by the total surface area. The vi are calculated using a connection table approximation.	MOE	0.2305	0.2058	0.3297	0.3046	0.1661	0.2546	0.203	0.1785	0.1716	0.1785	0.1746	0.285	0.2113	0.2642	0.1986	0.2589	0.2376	0.1926	0.1532	0.1894	0.2058	0.1859	0.2986	0.2779	0.1529	0.2248	0.1857	0.1633	0.1579	0.1633	0.1601	0.2591	0.1903	0.2418	0.1842	0.2325	0.2151	0.1751	0.1419
259	PEOE_VSA_FPOL	Fractional polar van der Waals surface area. This is the sum of the vi such that |qi| is greater than 0.2 divided by the total surface area. The vi are calculated using a connection table approximation.	MOE	0.4437	0.3962	0.5149	0.4758	0.3198	0.4901	0.3679	0.3436	0.4294	0.3436	0.3362	0.5224	0.4074	0.4841	0.5486	0.5148	0.4724	0.3708	0.2945	0.3765	0.3962	0.3579	0.4664	0.434	0.2944	0.4328	0.3367	0.3144	0.3951	0.3144	0.3082	0.4749	0.3669	0.443	0.5088	0.4623	0.4278	0.337	0.2729
260	PEOE_VSA_FPOS	Fractional positive van der Waals surface area. This is the sum of the vi such that qi is non-negative divided by the total surface area. The vi are calculated using a connection table approximation.	MOE	0.5635	0.5728	0.5808	0.5359	0.4737	0.6316	0.7174	0.5452	0.6496	0.5452	0.498	0.629	0.606	0.6192	0.6717	0.6435	0.5832	0.5492	0.5603	0.5109	0.5728	0.5804	0.5874	0.546	0.4878	0.6339	0.7117	0.5542	0.6497	0.5542	0.5108	0.631	0.6105	0.6219	0.6702	0.6442	0.5896	0.5584	0.567
261	PEOE_VSA_FPPOS	Fractional positive polar van der Waals surface area. This is the sum of the vi such that qi is greater than 0.2 divided by the total surface area. The vi are calculated using a connection table approximation.	MOE	0.2132	0.1904	0.1852	0.1711	0.1537	0.2355	0.1649	0.1651	0.2578	0.1651	0.1616	0.2373	0.1961	0.2199	0.35	0.2559	0.2348	0.1782	0.1413	0.1872	0.1904	0.172	0.1678	0.1561	0.1415	0.208	0.1509	0.1511	0.2372	0.1511	0.1481	0.2157	0.1766	0.2013	0.3246	0.2298	0.2126	0.162	0.1309
262	PEOE_VSA_HYD	Total hydrophobic van der Waals surface area. This is the sum of the vi such that |qi| is less than or equal to 0.2. The vi are calculated using a connection table approximation.	MOE	101.24	123.05	101.63	118.87	171.74	84.006	148.76	154.28	143.11	154.28	159.41	104.16	117.26	121.4	125.88	93.176	110.41	137.05	193.75	163.67	123.05	144.85	123.44	140.68	193.54	105.81	170.57	176.09	164.92	176.09	181.22	125.97	139.07	143.2	147.69	114.98	132.22	158.86	215.56
263	PEOE_VSA_NEG	Total negative van der Waals surface area. This is the sum of the vi such that qi is negative. The vi are calculated using a connection table approximation.	MOE	79.438	87.054	87.825	105.23	132.88	60.691	66.51	106.89	87.9	106.89	120.55	80.909	77.956	89.61	91.533	68.458	87.206	98.186	120.76	128.39	87.054	94.669	95.44	112.84	140.49	68.306	74.126	114.5	95.515	114.5	128.17	88.524	85.572	97.225	99.148	76.074	94.821	105.8	128.37
264	PEOE_VSA_PNEG	Total negative polar van der Waals surface area. This is the sum of the vi such that qi is less than -0.2. The vi are calculated using a connection table approximation.	MOE	41.943	41.943	69.077	69.077	41.943	41.943	47.763	41.943	43.049	41.943	41.943	62.161	41.806	62.161	55.382	49.711	49.711	41.943	42.08	49.711	41.943	41.943	69.077	69.077	41.943	41.943	47.763	41.943	43.049	41.943	41.943	62.161	41.806	62.161	55.382	49.711	49.711	41.943	42.08
265	PEOE_VSA_POL	Total polar van der Waals surface area. This is the sum of the vi such that |qi| is greater than 0.2. The vi are calculated using a connection table approximation.	MOE	80.75	80.75	107.88	107.88	80.75	80.75	86.569	80.75	107.71	80.75	80.75	113.92	80.613	113.92	152.97	98.841	98.841	80.75	80.887	98.841	80.75	80.75	107.88	107.88	80.75	80.75	86.569	80.75	107.71	80.75	80.75	113.92	80.613	113.92	152.97	98.841	98.841	80.75	80.887
266	PEOE_VSA_POS	Total positive van der Waals surface area. This is the sum of the vi such that qi is non-negative. The vi are calculated using a connection table approximation.	MOE	102.55	116.74	121.69	121.52	119.61	104.06	168.82	128.14	162.93	128.14	119.61	137.17	119.92	145.7	187.31	123.56	122.04	119.61	153.88	134.13	116.74	130.94	135.89	135.72	133.8	118.26	183.01	142.33	177.12	142.33	133.8	151.37	134.11	159.9	201.51	137.75	136.24	133.8	168.08
267	PEOE_VSA_PPOS	Total positive polar van der Waals surface area. This is the sum of the vi such that qi is greater than 0.2. The vi are calculated using a connection table approximation.	MOE	38.807	38.807	38.807	38.807	38.807	38.807	38.807	38.807	64.664	38.807	38.807	51.756	38.807	51.756	97.583	49.131	49.131	38.807	38.807	49.131	38.807	38.807	38.807	38.807	38.807	38.807	38.807	38.807	64.664	38.807	38.807	51.756	38.807	51.756	97.583	49.131	49.131	38.807	38.807
268	petitjean	Value of (diameter - radius) / diameter.	MOE	0.5	0.5	0.4286	0.5	0.4444	0.5	0.5	0.4286	0.4444	0.4286	0.5	0.4286	0.5	0.5	0.5	0.5	0.5	0.5	0.5	0.5	0.4286	0.4286	0.5	0.4444	0.5	0.4286	0.4444	0.5	0.5	0.5	0.4444	0.5	0.4286	0.4444	0.4545	0.4286	0.4286	0.4286	0.4545
269	petitjeanSC	Petitjean graph Shape Coefficient as defined in [Petitjean 1992]: (diameter - radius) / radius.	MOE	1	1	0.75	1	0.8	1	1	0.75	0.8	0.75	1	0.75	1	1	1	1	1	1	1	1	0.75	0.75	1	0.8	1	0.75	0.8	1	1	1	0.8	1	0.75	0.8	0.8333	0.75	0.75	0.75	0.8333
270	Q_PC+	Total positive partial charge: the sum of the positive qi. Q_PC+ is identical to PC+ which has been retained for compatibility.	MOE	3.953	4.363	4.859	4.859	4.847	3.953	4.82	3.953	5.806	3.953	4.413	5.323	3.883	5.323	7.731	4.633	4.633	3.953	5.187	5.229	4.363	4.773	5.269	5.269	5.257	4.363	5.23	4.363	6.216	4.363	4.823	5.733	4.293	5.733	8.141	5.043	5.043	4.363	5.597
271	Q_PC-	Total negative partial charge: the sum of the negative qi. Q_PC- is identical to PC- which has been retained for compatibility.	MOE	-3.953	-4.363	-5.859	-5.859	-4.847	-3.953	-4.82	-3.953	-4.806	-3.953	-4.413	-5.323	-3.883	-5.323	-6.731	-4.633	-4.633	-3.953	-5.188	-5.229	-4.363	-4.773	-6.269	-6.269	-5.257	-4.363	-5.23	-4.363	-5.216	-4.363	-4.823	-5.733	-4.293	-5.733	-7.141	-5.043	-5.043	-4.363	-5.598
272	Q_RPC+	Relative positive partial charge: the largest positive qi divided by the sum of the positive qi. Q_RPC+ is identical to RPC+ which has been retained for compatibility.	MOE	0.2292	0.2077	0.1865	0.1865	0.1869	0.2292	0.188	0.2292	0.156	0.2292	0.2053	0.1702	0.2333	0.1702	0.1552	0.1956	0.1956	0.2292	0.1747	0.1733	0.2077	0.1898	0.1719	0.1719	0.1723	0.2077	0.1732	0.2077	0.1458	0.2077	0.1878	0.158	0.211	0.158	0.1474	0.1797	0.1797	0.2077	0.1619
273	Q_RPC-	Relative negative partial charge: the smallest negative qi divided by the sum of the negative qi. Q_RPC- is identical to RPC- which has been retained for compatibility.	MOE	0.2277	0.2063	0.1536	0.1536	0.1857	0.2277	0.1867	0.2277	0.1873	0.2277	0.2039	0.1691	0.2318	0.1691	0.1437	0.1943	0.1943	0.2277	0.1735	0.1721	0.2063	0.1886	0.1436	0.1436	0.1712	0.2063	0.1721	0.2063	0.1725	0.2063	0.1866	0.157	0.2096	0.157	0.1354	0.1785	0.1785	0.2063	0.1608
274	Q_VSA_FHYD	Fractional hydrophobic van der Waals surface area. This is the sum of the vi such that |qi| is less than or equal to 0.2 divided by the total surface area. The vi are calculated using a connection table approximation.	MOE	0.4108	0.2921	0.2885	0.3426	0.5753	0.3492	0.4124	0.5438	0.4124	0.5438	0.3405	0.2772	0.4357	0.3301	0.3092	0.2695	0.3777	0.5077	0.5172	0.5226	0.2921	0.1964	0.1955	0.2513	0.4741	0.2267	0.3182	0.4383	0.3236	0.4383	0.254	0.1885	0.3231	0.2429	0.2361	0.1708	0.2761	0.3979	0.4278
275	Q_VSA_FNEG	Fractional negative van der Waals surface area. This is the sum of the vi such that qi is negative divided by the total surface area. The vi are calculated using a connection table approximation.	MOE	0.2305	0.3352	0.3712	0.343	0.4176	0.2546	0.2735	0.1785	0.1716	0.1785	0.2569	0.285	0.2113	0.2642	0.1986	0.2589	0.2376	0.1926	0.4246	0.3845	0.3352	0.4196	0.4502	0.419	0.4805	0.3661	0.3529	0.266	0.2546	0.266	0.3362	0.369	0.3103	0.3443	0.2719	0.3558	0.3292	0.2851	0.4823
276	Q_VSA_FPNEG	Fractional negative polar van der Waals surface area. This is the sum of the vi such that qi is less than -0.2 divided by the total surface area. The vi are calculated using a connection table approximation.	MOE	0.2305	0.3352	0.3297	0.3046	0.1661	0.2546	0.2735	0.1785	0.1716	0.1785	0.2569	0.285	0.2113	0.2642	0.1986	0.2589	0.2376	0.1926	0.2137	0.1894	0.3352	0.4196	0.4126	0.384	0.249	0.3661	0.3529	0.266	0.2546	0.266	0.3362	0.369	0.3103	0.3443	0.2719	0.3558	0.3292	0.2851	0.2869
277	Q_VSA_FPOL	Fractional polar van der Waals surface area. This is the sum of the vi such that |qi| is greater than 0.2 divided by the total surface area. The vi are calculated using a connection table approximation.	MOE	0.5892	0.7079	0.7115	0.6574	0.4247	0.6508	0.5876	0.4562	0.5876	0.4562	0.6595	0.7228	0.5643	0.6699	0.6908	0.7305	0.6223	0.4923	0.4828	0.4774	0.7079	0.8036	0.8045	0.7487	0.5259	0.7733	0.6818	0.5617	0.6764	0.5617	0.746	0.8115	0.6769	0.7571	0.7639	0.8292	0.7239	0.6021	0.5722
278	Q_VSA_FPOS	Fractional positive van der Waals surface area. This is the sum of the vi such that qi is non-negative divided by the total surface area. The vi are calculated using a connection table approximation.	MOE	0.7695	0.6648	0.6288	0.657	0.5824	0.7454	0.7265	0.8215	0.8284	0.8215	0.7431	0.715	0.7887	0.7358	0.8014	0.7411	0.7624	0.8074	0.5754	0.6155	0.6648	0.5804	0.5498	0.581	0.5195	0.6339	0.6471	0.734	0.7454	0.734	0.6638	0.631	0.6897	0.6557	0.7281	0.6442	0.6708	0.7149	0.5177
279	Q_VSA_FPPOS	Fractional positive polar van der Waals surface area. This is the sum of the vi such that qi is greater than 0.2 divided by the total surface area. The vi are calculated using a connection table approximation.	MOE	0.3587	0.3727	0.3818	0.3528	0.2585	0.3962	0.314	0.2778	0.4159	0.2778	0.4026	0.4378	0.353	0.4057	0.4922	0.4716	0.3848	0.2997	0.2691	0.288	0.3727	0.384	0.3919	0.3648	0.2769	0.4071	0.3289	0.2957	0.4218	0.2957	0.4098	0.4425	0.3666	0.4128	0.492	0.4734	0.3946	0.317	0.2853
280	Q_VSA_HYD	Total hydrophobic van der Waals surface area. This is the sum of the vi such that |qi| is less than or equal to 0.2. The vi are calculated using a connection table approximation.	MOE	74.764	59.532	60.453	77.685	145.26	57.532	97.057	127.81	103.45	127.81	81.78	60.453	86.216	77.685	86.216	51.751	79.03	110.57	142.05	137.2	59.532	44.3	45.221	62.453	130.03	42.3	81.825	112.57	88.216	112.57	66.548	45.221	70.984	62.453	70.984	36.519	63.798	95.342	126.82

Table S12 Amino acid descriptors (2D) used in this study.

			1st aa	A	A	A	A	A	A	A	A	A	A	A	A	A	A	A	A	A	A	A	A	C	C	C	C	C	C	C	C	C	C	C	C	C	C	C	C	C	C	C
			2nd aa	A	C	D	E	F	G	H	I	K	L	M	N	P	Q	R	S	T	V	W	Y	A	C	D	E	F	G	H	I	K	L	M	N	P	Q	R	S	T	V	W
No.	Descriptor ID	Attribute	Ref*	AA	AC	AD	AE	AF	AG	AH	AI	AK	AL	AM	AN	AP	AQ	AR	AS	AT	AV	AW	AY	CA	CC	CD	CE	CF	CG	CH	CI	CK	CL	CM	CN	CP	CQ	CR	CS	CT	CV	CW
281	Q_VSA_NEG	Total negative van der Waals surface area. This is the sum of the vi such that qi is negative. The vi are calculated using a connection table approximation.	MOE	41.943	68.306	77.778	77.778	105.43	41.943	64.372	41.943	43.049	41.943	61.704	62.161	41.806	62.161	55.382	49.711	49.711	41.943	116.62	100.94	68.306	94.669	104.14	104.14	131.79	68.306	90.735	68.306	69.412	68.306	88.067	88.524	68.169	88.524	81.745	76.074	76.074	68.306	142.98
282	Q_VSA_PNEG	Total negative polar van der Waals surface area. This is the sum of the vi such that qi is less than -0.2. The vi are calculated using a connection table approximation.	MOE	41.943	68.306	69.077	69.077	41.943	41.943	64.372	41.943	43.049	41.943	61.704	62.161	41.806	62.161	55.382	49.711	49.711	41.943	58.689	49.711	68.306	94.669	95.44	95.44	68.306	68.306	90.735	68.306	69.412	68.306	88.067	88.524	68.169	88.524	81.745	76.074	76.074	68.306	85.052
283	Q_VSA_POL	Total polar van der Waals surface area. This is the sum of the vi such that |qi| is greater than 0.2. The vi are calculated using a connection table approximation.	MOE	107.22	144.26	149.07	149.07	107.22	107.22	138.27	107.22	147.38	107.22	158.38	157.63	111.66	157.63	192.63	140.27	130.22	107.22	132.59	125.31	144.26	181.3	186.11	186.11	144.26	144.26	175.31	144.26	184.42	144.26	195.43	194.67	148.7	194.67	229.67	177.31	167.26	144.26	169.63
284	Q_VSA_POS	Total positive van der Waals surface area. This is the sum of the vi such that qi is non-negative. The vi are calculated using a connection table approximation.	MOE	140.04	135.49	131.74	148.97	147.06	122.81	170.96	193.09	207.78	193.09	178.46	155.92	156.07	173.15	223.46	142.31	159.54	175.85	158.02	161.58	135.49	130.94	127.19	144.42	142.51	118.26	166.4	188.53	203.22	188.53	173.91	151.37	151.51	168.6	218.91	137.75	154.98	171.3	153.47
285	Q_VSA_PPOS	Total positive polar van der Waals surface area. This is the sum of the vi such that qi is greater than 0.2. The vi are calculated using a connection table approximation.	MOE	65.28	75.957	79.989	79.989	65.28	65.28	73.899	65.28	104.33	65.28	96.681	95.468	69.853	95.468	137.25	90.556	80.509	65.28	73.899	75.604	75.957	86.635	90.666	90.666	75.957	75.957	84.576	75.957	115.01	75.957	107.36	106.14	80.531	106.14	147.93	101.23	91.187	75.957	84.576
286	radius	If ri is the largest matrix entry in row i of the distance matrix D, then the radius is defined as the smallest of the ri [Petitjean 1992].	MOE	3	3	4	4	5	3	4	4	5	4	4	4	3	4	5	3	3	3	5	5	4	4	4	5	5	4	5	4	5	4	5	4	4	5	6	4	4	4	6
287	reactive	Indicator of the presence of reactive groups. A non-zero value indicates that the molecule contains a reactive group. The table of reactive groups is based on the Oprea set [Oprea 2000] and includes metals, phospho-, N/O/S-N/O/S single bonds, thiols, acyl halides, Michael Acceptors, azides, esters, etc.	MOE	0	1	0	0	0	0	0	0	0	0	0	0	0	0	0	0	0	0	0	0	1	1	1	1	1	1	1	1	1	1	1	1	1	1	1	1	1	1	1
288	rings	The number of rings.	MOE	0	0	0	0	1	0	1	0	0	0	0	0	1	0	0	0	0	0	2	1	0	0	0	0	1	0	1	0	0	0	0	0	1	0	0	0	0	0	2
289	RPC+	Relative positive partial charge	MOE	0.2292	0.2077	0.1865	0.1865	0.1869	0.2292	0.188	0.2292	0.156	0.2292	0.2053	0.1702	0.2333	0.1702	0.1552	0.1956	0.1956	0.2292	0.1747	0.1733	0.2077	0.1898	0.1719	0.1719	0.1723	0.2077	0.1732	0.2077	0.1458	0.2077	0.1878	0.158	0.211	0.158	0.1474	0.1797	0.1797	0.2077	0.1619
290	RPC-	Relative negative partial charge	MOE	0.2277	0.2063	0.1536	0.1536	0.1857	0.2277	0.1867	0.2277	0.1873	0.2277	0.2039	0.1691	0.2318	0.1691	0.1437	0.1943	0.1943	0.2277	0.1735	0.1721	0.2063	0.1886	0.1436	0.1436	0.1712	0.2063	0.1721	0.2063	0.1725	0.2063	0.1866	0.157	0.2096	0.157	0.1354	0.1785	0.1785	0.2063	0.1608
291	SlogP	Log of the octanol/water partition coefficient (including implicit hydrogens). This property is an atomic contribution model [Crippen 1999] that calculates logP from the given structure; i.e. the correct protonation state (washed structures). Results may vary from the logP(o/w) descriptor. The training set for SlogP was ~7000 structures.	MOE	-3.1286	-3.2187	-5.0085	-4.6184	-1.9058	-3.5171	-3.1827	-2.1024	-3.7363	-2.1024	-2.3954	-4.2731	-2.6423	-3.883	-5.7049	-4.1562	-3.7677	-2.4925	-1.4245	-2.2002	-3.2187	-3.3088	-5.0986	-4.7085	-1.9959	-3.6072	-3.2728	-2.1925	-3.8264	-2.1925	-2.4855	-4.3632	-2.7324	-3.9731	-5.795	-4.2463	-3.8578	-2.5826	-1.5146
292	SlogP_VSA0	Subdivided Surface Areas, Sum of vi such that Li <= -0.4.	MOE	93.591	93.591	136.27	136.27	93.591	93.591	93.591	93.591	126.49	93.591	93.591	126.49	75.581	126.49	176.17	118.98	118.98	93.591	93.591	118.98	93.591	93.591	136.27	136.27	93.591	93.591	93.591	93.591	126.49	93.591	93.591	126.49	75.581	126.49	176.17	118.98	118.98	93.591	93.591
293	SlogP_VSA1	Subdivided Surface Areas, Sum of vi such that Li is in (-0.4,-0.2].	MOE	6.7228	6.7228	8.1868	8.1868	6.7228	6.7228	23.508	6.7228	6.7228	6.7228	6.7228	11.982	9.8471	11.982	8.6219	6.7228	6.7228	6.7228	6.7228	6.7228	6.7228	6.7228	8.1868	8.1868	6.7228	6.7228	23.508	6.7228	6.7228	6.7228	6.7228	11.982	9.8471	11.982	8.6219	6.7228	6.7228	6.7228	6.7228
294	SlogP_VSA2	Subdivided Surface Areas, Sum of vi such that Li is in (-0.2,0].	MOE	55.688	55.688	79.551	79.551	55.688	51.706	72.474	55.688	55.688	55.688	55.688	79.551	55.688	79.551	55.688	55.688	62.157	55.688	72.474	55.688	55.688	55.688	79.551	79.551	55.688	51.706	72.474	55.688	55.688	55.688	55.688	79.551	55.688	79.551	55.688	55.688	62.157	55.688	72.474
295	SlogP_VSA3	Subdivided Surface Areas, Sum of vi such that Li is in (0,0.1].	MOE	0	17.234	0	0	0	18.44	0	0	18.44	0	17.234	0	18.44	0	18.44	20.926	0	0	0	0	17.234	34.467	17.234	17.234	17.234	35.673	17.234	17.234	35.673	17.234	34.467	17.234	35.673	17.234	35.673	38.16	17.234	17.234	17.234
296	SlogP_VSA4	Subdivided Surface Areas, Sum of vi such that Li is in (0.1,0.15].	MOE	0	0	0	0	3.1856	0	2.7567	4.4108	0	4.4108	0	0	0	0	0	0	0	4.4108	3.1856	3.1856	0	0	0	0	3.1856	0	2.7567	4.4108	0	4.4108	0	0	0	0	0	0	0	4.4108	3.1856
297	SlogP_VSA5	Subdivided Surface Areas, Sum of vi such that Li is in (0.15,0.20].	MOE	0	0	0	0	18.868	0	18.868	0	0	0	31.691	0	0	0	0	0	0	0	18.868	18.868	0	0	0	0	18.868	0	18.868	0	0	0	31.691	0	0	0	0	0	0	0	18.868
298	SlogP_VSA7	Subdivided Surface Areas, Sum of vi such that Li is in (0.25,0.30].	MOE	0	0	0	0	88.216	0	34	0	0	0	0	0	0	0	0	0	0	0	92.504	70.573	0	0	0	0	88.216	0	34	0	0	0	0	0	0	0	0	0	0	0	92.504
299	SlogP_VSA8	Subdivided Surface Areas, Sum of vi such that Li is in (0.30,0.40].	MOE	0	46.107	18.868	37.737	0	0	0	18.868	56.605	18.868	18.868	18.868	37.737	37.737	37.737	0	0	0	0	0	46.107	92.214	64.976	83.844	46.107	46.107	46.107	64.976	102.71	64.976	64.976	64.976	83.844	83.844	83.844	46.107	46.107	46.107	46.107
300	SlogP_VSA9	Subdivided Surface Areas, Sum of vi such that Li > 0.40.	MOE	66.652	33.326	33.326	33.326	33.326	33.326	33.326	99.978	33.326	99.978	65.546	33.326	33.326	33.326	33.326	33.326	66.652	99.978	33.326	38.569	33.326	0	0	0	0	0	0	66.652	0	66.652	32.22	0	0	0	0	0	33.326	66.652	0
301	SMR	Molecular refractivity (including implicit hydrogens). This property is an atomic contribution model [Crippen 1999] that assumes the correct protonation state (washed structures). The model was trained on ~7000 structures and results may vary from the mr descriptor.	MOE	3.4962	4.3142	3.8912	4.3529	5.9434	3.0367	5.1579	4.8743	5.0894	4.8743	5.2288	4.3099	4.2092	4.7716	5.7644	3.6374	4.0969	4.4126	7.129	6.1099	4.3142	5.1322	4.7092	5.1709	6.7614	3.8547	5.9759	5.6923	5.9074	5.6923	6.0468	5.1279	5.0272	5.5896	6.5824	4.4554	4.9149	5.2306	7.9471
302	SMR_VSA0	Subdivided Surface Areas, Sum of vi such that Ri is in [0,0.11].	MOE	90.408	90.408	156.95	156.95	90.408	90.408	90.408	90.408	90.408	90.408	90.408	114.27	90.408	114.27	90.408	90.408	90.408	90.408	90.408	90.408	90.408	90.408	156.95	156.95	90.408	90.408	90.408	90.408	90.408	90.408	90.408	114.27	90.408	114.27	90.408	90.408	90.408	90.408	90.408
303	SMR_VSA1	Subdivided Surface Areas, Sum of vi such that Ri is in (0.11,0.26].	MOE	0	0	0	0	0	0	16.786	0	0	0	0	0	3.1243	0	31.672	25.385	25.385	0	0	25.385	0	0	0	0	0	0	16.786	0	0	0	0	0	3.1243	0	31.672	25.385	25.385	0	0
304	SMR_VSA2	Subdivided Surface Areas, Sum of vi such that Ri is in (0.26,0.35].	MOE	50.908	50.908	50.908	50.908	50.908	50.908	67.693	55.319	83.805	55.319	50.908	50.908	32.897	50.908	68.919	50.908	50.908	55.319	67.693	50.908	50.908	50.908	50.908	50.908	50.908	50.908	67.693	55.319	83.805	55.319	50.908	50.908	32.897	50.908	68.919	50.908	50.908	55.319	67.693
305	SMR_VSA3	Subdivided Surface Areas, Sum of vi such that Ri is in (0.35,0.39].	MOE	7.9639	7.9639	7.9639	7.9639	11.15	3.982	10.721	7.9639	7.9639	7.9639	7.9639	7.9639	7.9639	7.9639	7.9639	7.9639	14.433	7.9639	11.15	16.393	7.9639	7.9639	7.9639	7.9639	11.15	3.982	10.721	7.9639	7.9639	7.9639	7.9639	7.9639	7.9639	7.9639	7.9639	7.9639	14.433	7.9639	11.15
306	SMR_VSA4	Subdivided Surface Areas, Sum of vi such that Ri is in (0.39,0.44].	MOE	0	0	0	0	0	0	0	0	0	0	0	0	32.897	0	32.897	32.897	0	0	0	4.7171	0	0	0	0	0	0	0	0	0	0	0	0	32.897	0	32.897	32.897	0	0	4.7171
307	SMR_VSA5	Subdivided Surface Areas, Sum of vi such that Ri is in (0.44,0.485].	MOE	0	0	18.868	37.737	107.08	0	52.868	18.868	56.605	18.868	18.868	18.868	37.737	37.737	37.737	0	0	0	106.66	89.441	0	0	18.868	37.737	107.08	0	52.868	18.868	56.605	18.868	18.868	18.868	37.737	37.737	37.737	0	0	0	106.66
308	SMR_VSA6	Subdivided Surface Areas, Sum of vi such that Ri is in (0.485,0.56].	MOE	6.7228	23.956	8.1868	8.1868	6.7228	25.162	6.7228	6.7228	25.162	6.7228	23.956	11.982	25.162	11.982	27.061	27.649	6.7228	6.7228	6.7228	6.7228	23.956	41.19	25.42	25.42	23.956	42.396	23.956	23.956	42.396	23.956	41.19	29.215	42.396	29.215	44.295	44.883	23.956	23.956	23.956
309	SMR_VSA7	Subdivided Surface Areas, Sum of vi such that Ri > 0.56.	MOE	66.652	79.433	33.326	33.326	33.326	33.326	33.326	99.978	33.326	99.978	97.237	33.326	33.326	33.326	33.326	33.326	66.652	99.978	33.326	33.326	79.433	92.214	46.107	46.107	46.107	46.107	46.107	112.76	46.107	112.76	110.02	46.107	46.107	46.107	46.107	46.107	79.433	112.76	46.107
310	TPSA	Polar surface area (Å2) calculated using group contributions to approximate the polar surface area from connection table information only. The parameterization is that of Ertl et al. [Ertl 2000].	MOE	96.87	135.67	137	137	96.87	96.87	125.55	96.87	124.51	96.87	96.87	139.96	88.08	139.96	160.51	117.1	117.1	96.87	112.66	117.1	135.67	174.47	175.8	175.8	135.67	135.67	164.35	135.67	163.31	135.67	135.67	178.76	126.88	178.76	199.31	155.9	155.9	135.67	151.46
311	VAdjEq	Vertex adjacency information (equality): -(1-f)log2(1-f) - f log2 f where f = (n2 - m) / n2, n is the number of heavy atoms and m is the number of heavy-heavy bonds. If f is not in the open interval (0,1), then 0 is returned.	MOE	0.6468	0.6167	0.5647	0.542	0.5226	0.6801	0.5436	0.5647	0.542	0.5647	0.5647	0.5647	0.6194	0.542	0.5021	0.6167	0.5895	0.5895	0.4846	0.5033	0.6167	0.5895	0.542	0.5212	0.5033	0.6468	0.5226	0.542	0.5212	0.542	0.542	0.542	0.5917	0.5212	0.4845	0.5895	0.5647	0.5647	0.4683
312	VAdjMa	Vertex adjacency information (magnitude): 1 + log2 m where m is the number of heavy-heavy bonds. If m is zero, then zero is returned.	MOE	4.3219	4.4594	4.7004	4.8074	5.0875	4.1699	5	4.7004	4.8074	4.7004	4.7004	4.7004	4.7004	4.8074	5	4.4594	4.585	4.585	5.3923	5.1699	4.4594	4.585	4.8074	4.9069	5.1699	4.3219	5.0875	4.8074	4.9069	4.8074	4.8074	4.8074	4.8074	4.9069	5.0875	4.585	4.7004	4.7004	5.4594
313	VDistEq	If m is the sum of the distance matrix entries then VdistEq is defined to be the sum of log2 m - pi log2 pi / m where pi is the number of distance matrix entries equal to i.	MOE	2.5024	2.5377	2.712	2.8785	2.9863	2.5111	2.88	2.6564	2.9748	2.712	2.8148	2.712	2.4665	2.8785	3.1972	2.5377	2.5502	2.5502	3.1247	3.1051	2.6517	2.6888	2.8374	2.9843	3.0781	2.6622	2.9874	2.7867	3.0608	2.8374	2.9203	2.8374	2.6127	2.9843	3.2738	2.6888	2.7059	2.7059	3.207
314	VDistMa	If m is the sum of the distance matrix entries then VDistMa is defined to be the sum of log2 m - Dij log2 Dij / m over all i and j.	MOE	6.5948	6.8646	7.3244	7.5165	7.8838	6.2897	7.709	7.3324	7.5061	7.3244	7.3135	7.3244	7.1176	7.5165	7.8584	6.8646	7.1114	7.1114	8.3579	8.042	6.8502	7.0973	7.524	7.7045	8.0494	6.5745	7.8846	7.5306	7.6956	7.524	7.5149	7.524	7.3304	7.7045	8.0269	7.0973	7.3248	7.3248	8.4983
315	vdw_area	Area of van der Waals surface (Å2) calculated using a connection table approximation.	MOE	181.99	203.8	209.52	226.75	252.49	164.76	235.33	235.03	250.83	235.03	240.16	218.08	197.88	235.31	278.85	192.02	209.25	217.8	274.64	262.52	203.8	225.6	231.33	248.56	274.29	186.56	257.14	256.84	272.63	256.84	261.97	239.89	219.68	257.12	300.66	213.83	231.06	239.61	296.45
316	vdw_vol	van der Waals volume (Å3) calculated using a connection table approximation.	MOE	201.8	223.12	231.54	255.97	316.78	177.37	281.03	275.09	293.64	275.09	273.04	242.08	238.38	266.51	317.77	210.32	234.75	250.66	362.12	325.3	223.12	244.44	252.86	277.29	338.1	198.69	302.35	296.41	314.97	296.41	294.36	263.4	259.7	287.83	339.09	231.64	256.07	271.98	383.44
317	vsa_acc	Approximation to the sum of VDW surface areas (Å2) of pure hydrogen bond acceptors (not counting acidic atoms and atoms that are both hydrogen bond donors and acceptors such as -OH).	MOE	40.701	40.701	67.835	67.835	40.701	40.701	40.701	40.701	40.701	40.701	40.701	54.268	40.701	54.268	40.701	40.701	40.701	40.701	40.701	40.701	40.701	40.701	67.835	67.835	40.701	40.701	40.701	40.701	40.701	40.701	40.701	54.268	40.701	54.268	40.701	40.701	40.701	40.701	40.701
318	vsa_acid	Approximation to the sum of VDW surface areas of acidic atoms (Å2).	MOE	27.134	27.134	54.268	54.268	27.134	27.134	27.134	27.134	27.134	27.134	27.134	27.134	27.134	27.134	27.134	27.134	27.134	27.134	27.134	27.134	27.134	27.134	54.268	54.268	27.134	27.134	27.134	27.134	27.134	27.134	27.134	27.134	27.134	27.134	27.134	27.134	27.134	27.134	27.134
319	vsa_base	Approximation to the sum of VDW surface areas of basic atoms (Å2).	MOE	17.742	17.742	17.742	17.742	17.742	17.742	17.742	17.742	35.485	17.742	17.742	17.742	17.742	17.742	58.91	17.742	17.742	17.742	17.742	17.742	17.742	17.742	17.742	17.742	17.742	17.742	17.742	17.742	35.485	17.742	17.742	17.742	17.742	17.742	58.91	17.742	17.742	17.742	17.742
320	vsa_don	Approximation to the sum of VDW surface areas of pure hydrogen bond donors (not counting basic atoms and atoms that are both hydrogen bond donors and acceptors such as -OH) (Å2).	MOE	23.425	23.425	23.425	23.425	23.425	23.425	23.425	23.425	41.168	23.425	23.425	41.168	17.742	41.168	64.593	23.425	23.425	23.425	29.108	23.425	23.425	23.425	23.425	23.425	23.425	23.425	23.425	23.425	41.168	23.425	23.425	41.168	17.742	41.168	64.593	23.425	23.425	23.425	29.108
321	vsa_hyd	Approximation to the sum of VDW surface areas of hydrophobic atoms (Å2).	MOE	72.216	60.592	58.616	74.425	139.87	56.408	85.615	119.64	110.53	119.64	127.55	58.616	94.724	74.425	94.724	64.866	80.675	103.83	153.27	132.52	60.592	48.968	46.992	62.801	128.24	44.784	73.991	108.02	98.909	108.02	115.92	46.992	83.1	62.801	83.1	53.242	69.051	92.209	141.64
322	vsa_other	Approximation to the sum of VDW surface areas (Å2) of atoms typed as "other".	MOE	27.658	59.043	42.367	42.367	27.658	27.658	52.449	27.658	27.658	27.658	27.658	40.608	27.658	40.608	43.34	27.658	27.658	27.658	27.658	27.658	59.043	90.427	73.751	73.751	59.043	59.043	83.833	59.043	59.043	59.043	59.043	71.992	59.043	71.992	74.724	59.043	59.043	59.043	59.043
323	vsa_pol	Approximation to the sum of VDW surface areas (Å2) of polar atoms (atoms that are both hydrogen bond donors and acceptors), such as -OH.	MOE	64.126	64.126	91.26	91.26	64.126	64.126	75.491	64.126	81.868	64.126	64.126	95.435	58.443	95.435	105.29	77.693	77.693	64.126	69.808	77.693	64.126	64.126	91.26	91.26	64.126	64.126	75.491	64.126	81.868	64.126	64.126	95.435	58.443	95.435	105.29	77.693	77.693	64.126	69.808
324	Weight	Molecular weight (including implicit hydrogens) in atomic mass units with atomic weights taken from [CRC 1994].	MOE	160.17	192.24	203.17	217.2	236.27	146.15	226.24	202.25	218.28	202.25	220.29	203.2	186.21	217.22	246.29	176.17	190.2	188.23	275.31	252.27	192.24	224.31	235.24	249.27	268.34	178.21	258.3	234.32	250.34	234.32	252.36	235.26	218.28	249.29	278.36	208.24	222.27	220.29	307.37
325	weinerPath	Weiner path number	MOE	168	211	323	404	552	136	464	314	416	323	334	323	238	404	612	211	256	256	832	654	216	266	394	485	651	178	553	384	498	394	406	394	297	485	716	266	318	318	961
326	weinerPol	Weiner polarity number	MOE	12	14	16	17	21	9	18	18	17	16	16	16	17	17	19	14	16	16	28	23	14	16	18	19	23	11	20	20	19	18	18	18	19	19	21	16	18	18	30
327	zagreb	Zagreb index: the sum of di2 over all heavy atoms i.	MOE	46	50	60	64	78	40	74	60	62	60	58	60	62	64	72	50	56	56	100	84	50	54	64	68	82	44	78	64	66	64	62	64	66	68	76	54	60	60	104
328	dipoleX	The x component of the dipole moment (external coordinates).	MOE	4.8853	5.4379	-4.8703	-6.4082	2.3451	-4.7052	2.8615	5.5045	4.1477	2.3338	4.9363	2.5588	5.1643	-2.2806	-1.7541	-5.0736	2.156	-2.7082	4.2489	-1.2368	-4.1856	2.3773	5.8068	4.1711	-1.1481	-3.7004	1.8619	-5.6719	1.4109	-2.261	-2.2363	-4.9842	2.4606	3.5164	3.9815	2.7106	5.8127	5.7283	0.6561
329	dipoleY	The y component of the dipole moment (external coordinates).	MOE	-0.9914	0.5904	-1.4729	1.9917	-2.4611	-1.6806	4.058	-2.2417	6.3304	-2.1129	-1.5183	-0.1237	-2.2626	5.061	-1.5571	1.2702	-2.0206	-0.9911	3.8482	2.4998	1.5674	0.3219	-2.4412	0.9901	3.281	0.5371	5.8055	-0.6453	-3.6299	-1.4556	-1.4285	-0.1082	0.8944	-2.9209	-3.2028	0.2003	-0.903	1.4078	2.8781
330	dipoleZ	The z component of the dipole moment (external coordinates).	MOE	-1.2076	-1.7226	0.5713	0.6762	0.1696	-0.8949	0.2813	-0.8339	0.446	-0.9874	-1.467	0.9258	-0.8696	1.1137	1.3771	1.2823	1.4233	-1.6743	-0.3128	-1.975	0.9193	-1.0012	-0.6526	-0.1302	-0.373	0.3661	0.3831	0.2217	-2.5437	2.0704	0.8586	-1.5381	1.8085	1.7428	1.1747	-0.1038	-0.8694	0.2111	0.8362
331	E_rnb	Non-bonded interaction energy (external reference frame: x3d) between the stored molecule and a "receptor" currently loaded in MOE. It is similar to the other E_r* calls in that it is an interaction energy term.	MOE	0	0	0	0	0	0	0	0	0	0	0	0	0	0	0	0	0	0	0	0	0	0	0	0	0	0	0	0	0	0	0	0	0	0	0	0	0	0	0
332	E_rsol	Solvation free energy difference (external reference frame: x3d). Let L be the free energy of solvation of the stored molecule (ligand), R be the free energy of solvation of the atoms currently loaded (receptor), and G be the free energy of solvation of the RL complex. Consequently, the returned value is G - L - R. The term enable flag is ignored, but the term weight is applied. Partial charges are assumed to be correct in the database molecule as well as on the currently loaded atoms.	MOE	0	0	0	0	0	0	0	0	0	0	0	0	0	0	0	0	0	0	0	0	0	0	0	0	0	0	0	0	0	0	0	0	0	0	0	0	0	0	0
333	pmiX	x component of the principal moment of inertia (external coordinates).	MOE	836.95	877.68	777.16	1359.7	1288.4	633.93	989.76	1134.4	1527.3	1016.1	1000.1	1180.1	775.29	1353.2	2524.5	735.76	634.57	831.85	1522.6	2227.5	1014.8	1708.5	1156.7	1407.9	1532.1	717.45	1495.9	1359.7	2198.6	1429.2	2254.7	1560.6	1159.9	1508.1	3211.3	980	1121.5	1048.6	2589.6
334	pmiY	y component of the principal moment of inertia (external coordinates).	MOE	166.24	308.05	431.1	432.35	448.13	181.98	592.88	364.12	642.68	379.22	717.04	303.5	263.32	524.71	402.79	222.73	342.67	309.17	797.27	572.27	244.82	258.34	440.3	544.19	805.06	198.62	896.04	457.03	497.64	496.11	471.19	454	279.36	683.8	964.56	404.32	388.57	382.88	1023.8
335	pmiZ	z component of the principal moment of inertia (external coordinates).	MOE	62.733	152.93	142.35	111.58	212.53	57.347	142.5	139.41	104.22	156.65	158.72	120.51	130	146.94	60.443	115.62	168.48	113.16	293.06	158.13	177.06	116.82	243.78	179.89	284.07	141.93	107.43	239.45	263.61	176.94	112.79	215.76	172.04	209.17	117.47	115.08	213.04	285.44	346.76
336	AM1_dipole	The dipole moment calculated using the AM1 Hamiltonian [MOPAC].	MOE	19.935	21.275	11.519	16.624	5.8174	5.4081	14.477	23.333	20.763	5.2205	13.024	3.0017	8.2966	16.119	13.915	14.194	11.441	6.704	19.363	6.5264	4.3194	3.0652	12.133	11.528	6.1052	6.0578	22.067	22.573	13.272	6.0303	4.9299	13.82	6.0708	14.41	21.423	5.0739	21.925	21.781	6.3121
337	AM1_E	The total SCF energy (kcal/mol) calculated using the AM1 Hamiltonian [MOPAC].	MOE	-53202	-57690	-70727	-74313	-72233	-49674	-72234	-63980	-69266	-64043	-64888	-68707	-59759	-72256	-78828	-60618	-64204	-60449	-83166	-79632	-57744	-62227	-75209	-78807	-76722	-54156	-76693	-68458	-73747	-68522	-69410	-73147	-64282	-76736	-83328	-65138	-68675	-64866	-87699
338	AM1_Eele	The electronic energy (kcal/mol) calculated using the AM1 Hamiltonian [MOPAC].	MOE	-255117	-293149	-362675	-395164	-449151	-224224	-432927	-371123	-415577	-370306	-380565	-366547	-340769	-404854	-498763	-310252	-347721	-332464	-569106	-479163	-289981	-328355	-403140	-452824	-490724	-254476	-466865	-414169	-463883	-410890	-400847	-406380	-362883	-454831	-519741	-336640	-382306	-372349	-590324
339	AM1_HF	The heat of formation (kcal/mol) calculated using the AM1 Hamiltonian [MOPAC].	MOE	-70.522	-73.162	-257.86	-256.82	-101.71	-131.44	-41.518	-90.386	35.601	-152.34	-94.578	-176.02	-86.405	-136.59	33.318	-134.35	-139.48	-146.67	-25.692	-152	-126.93	-122	-251.54	-263.5	-104.01	-125.17	-13.27	-80.266	43.009	-144.67	-131.97	-126.98	-120.95	-128.45	21.687	-171.9	-122.36	-75.799	-70.592
340	AM1_HOMO	The energy (eV) of the Highest Occupied Molecular Orbital calculated using the AM1 Hamiltonian [MOPAC].	MOE	-7.7593	-8.1384	-5.0209	-4.6804	-9.3298	-10.29	-8.0297	-7.5611	-10.512	-10.099	-8.1654	-10.537	-9.0054	-8.3931	-11.375	-8.5033	-8.6064	-10.267	-7.977	-9.0439	-9.4258	-9.4404	-5.0547	-4.9821	-9.7438	-9.8848	-7.3229	-7.5157	-11.048	-9.3168	-8.8905	-8.8679	-9.6445	-8.8981	-11.227	-9.4667	-7.8997	-7.7046	-8.7517
341	AM1_IP	The ionization potential (kcal/mol) calculated using the AM1 Hamiltonian [MOPAC].	MOE	7.7593	8.1384	5.0209	4.6804	9.3298	10.29	8.0297	7.5611	10.512	10.099	8.1654	10.537	9.0054	8.3931	11.375	8.5033	8.6064	10.267	7.977	9.0439	9.4258	9.4404	5.0547	4.9821	9.7438	9.8848	7.3229	7.5157	11.048	9.3168	8.8905	8.8679	9.6445	8.8981	11.227	9.4667	7.8997	7.7046	8.7517
342	AM1_LUMO	The energy (eV) of the Lowest Unoccupied Molecular Orbital calculated using the AM1 Hamiltonian [MOPAC].	MOE	-1.5704	-1.475	4.2691	3.1068	0.5116	0.8019	-1.1136	-1.4764	-3.9482	0.8326	-0.5792	0.5	-0.3268	-0.6704	-3.6789	-0.5398	-0.737	0.9583	-1.0035	0.2619	0.2566	0.1576	3.0068	3.1833	-0.0404	-0.1086	-0.8771	-1.5459	-3.6872	0.5526	0.2944	-1.2005	0.0604	-1.1512	-4.2689	0.1812	-1.6238	-1.5935	-0.1449
343	ASA	Water accessible surface area calculated using a radius of 1.4 A for the water molecule. A polyhedral representation is used for each atom in calculating the surface area.	MOE	355.65	367.42	366.19	399.81	438.54	321.31	412.48	420.57	460.09	413.57	416.69	378.59	369.31	424.39	471.61	355.97	361.02	390.04	464.83	470.33	373.35	392.15	391.56	400.37	463.83	338.47	441.26	435.62	477.32	434.67	447.88	419.75	384.46	432.34	513.85	373.87	398.35	404.63	526.13
344	ASA+	Water accessible surface area of all atoms with positive partial charge (strictly greater than 0).	MOE	92.702	117.45	89.576	105.37	181.63	113.67	175.54	88.344	177.91	77.475	104.26	145.65	90.108	168.61	195.54	119.53	118.29	73.627	193.25	192.51	101.76	127.7	113.78	90.557	184.38	120.98	199.51	114.99	179.34	99.25	138.24	159.11	84.281	148.21	243.5	133.12	106.82	96.402	221.22
345	ASA-	Water accessible surface area of all atoms with negative partial charge (strictly less than 0).	MOE	87.542	144.38	148.44	143.66	125.53	108.51	104.47	72.794	72.438	73.034	124.94	110.33	77.383	113.33	79.089	93.992	95.54	71.054	167.8	156.29	146.53	175.42	188.16	182.66	192.51	126.87	161.62	112.69	132.81	109.58	156.85	184.54	132.58	172.95	137.62	148.11	149.84	132.02	218.13
346	ASA_H	Water accessible surface area of all hydrophobic (|qi|<0.2) atoms.	MOE	177.35	117.27	134.43	154.87	305.21	103.8	211.93	260.28	210.03	263.06	188.59	127.85	202.4	144.88	197.79	143.02	148.01	245.36	267.27	268.16	136.87	115.7	96.889	151.28	273.96	127.31	173.82	234.47	182.07	239.18	176.34	91.849	183.23	132.97	156.35	108.11	144.96	179.38	292.45
347	ASA_P	Water accessible surface area of all polar (|qi|>=0.2) atoms.	MOE	178.3	250.14	231.77	244.94	133.33	217.51	200.56	160.28	250.06	150.51	228.09	250.74	166.92	279.51	273.81	212.95	213.01	144.68	197.56	202.17	236.48	276.45	294.67	249.09	189.87	211.16	267.44	201.15	295.25	195.5	271.54	327.9	201.22	299.37	357.49	265.76	253.39	225.24	233.68
348	CASA+	Positive charge weighted surface area, ASA+ times max { qi > 0 } [Stanton 1990].	MOE	366.45	512.44	435.25	511.97	880.35	449.35	846.11	349.22	1033	306.26	460.09	775.3	349.89	897.5	1511.7	553.79	548.03	291.05	1002.4	1006.6	443.99	609.52	599.49	477.15	969.29	527.84	1043.5	501.7	1114.8	433.03	666.73	912.16	361.82	849.71	1982.4	671.31	538.68	420.6	1238.2
349	CASA-	Negative charge weighted surface area, ASA- times max { qi < 0 } [Stanton 1990].	MOE	346.05	629.93	869.68	841.7	608.46	428.92	503.55	287.75	348.14	288.7	551.38	587.27	300.48	603.25	532.35	435.46	442.64	280.87	870.56	817.23	639.3	837.26	1179.6	1145.1	1012	553.51	845.25	491.67	692.76	478.09	756.49	1058	569.15	991.5	982.73	746.9	755.64	576.02	1221.1
350	DASA	Absolute value of the difference between ASA+ and ASA-.	MOE	5.16	26.928	58.859	38.294	56.095	5.1679	71.072	15.55	105.47	4.441	20.687	35.324	12.725	55.279	116.45	25.54	22.748	2.5735	25.449	36.217	44.766	47.716	74.387	92.106	8.1337	5.8847	37.899	2.2992	46.531	10.328	18.612	25.432	48.295	24.731	105.89	14.99	43.021	35.621	3.088
351	DCASA	Absolute value of the difference between CASA+ and CASA- [Stanton 1990].	MOE	20.397	117.49	434.43	329.73	271.89	20.429	342.57	61.47	684.81	17.555	91.29	188.03	49.41	294.25	979.34	118.33	105.39	10.173	131.84	189.38	195.31	227.75	580.11	667.97	42.759	25.675	198.21	10.032	422.05	45.06	89.765	145.8	207.33	141.78	999.64	75.592	216.96	155.42	17.065
352	dens	Mass density: molecular weight divided by van der Waals volume as calculated in the vol descriptor.	MOE	1.0564	1.1283	1.1908	1.1462	1.0363	1.1051	1.1029	0.9878	0.9956	1.0025	1.0655	1.1496	1.0528	1.0854	1.0559	1.115	1.0753	1.0279	1.0473	1.0775	1.1367	1.2076	1.2316	1.2197	1.0875	1.1911	1.1429	1.0484	1.0453	1.0591	1.1279	1.1778	1.1332	1.1548	1.1019	1.1882	1.1479	1.0726	1.1032
353	dipole	Dipole moment calculated from the partial charges of the molecule.	MOE	5.1291	5.7347	5.1201	6.7446	3.4037	5.0759	4.9734	6.0017	7.5813	3.2994	5.3688	2.724	5.7049	5.6617	2.7198	5.385	3.2798	3.3346	5.741	3.4175	4.5631	2.5995	6.3328	4.289	3.496	3.757	6.1087	5.7128	4.6516	3.3938	2.789	5.2172	3.182	4.8923	5.2431	2.72	5.9463	5.9025	3.0681
354	E	Value of the potential energy. The state of all term enable flags will be honored (in addition to the term weights). This means that the current potential setup accurately reflects what will be calculated.	MOE	26.894	29.608	-1.886	15.934	31.049	27.965	12.153	30.257	33.992	9.4484	30.84	-25.637	38.298	25.927	-78.142	28.217	33.509	7.1326	45.995	21.579	36.592	1.0689	28.56	-5.1961	32.442	6.5191	29.391	39.219	38.543	14.139	13.819	29.006	26.059	30.283	-58.009	17.331	33.652	39.829	25.667
355	E_ang	Angle bend potential energy. In the Potential Setup panel, the term enable (Bonded) flag is ignored, but the term weight is applied.	MOE	4.7772	7.5873	7.5005	8.2115	10.781	8.8835	4.5187	7.3225	5.2493	5.4964	7.1302	10.188	10.125	6.9803	10.96	4.8222	13.1	4.9476	10.458	4.9349	6.2299	10.644	8.3612	13.831	4.9621	3.5964	5.3091	8.5492	11.469	6.3559	6.8142	8.1874	12.319	11.868	5.9318	6.6312	4.3716	7.1962	6.1071
356	E_ele	Electrostatic component of the potential energy. In the Potential Setup panel, the term enable flag is ignored, but the term weight is applied.	MOE	6.6973	3.8577	-63.155	-50.341	-51.618	7.7119	-19.92	6.3059	11.825	-52.762	4.3478	-97.484	11.168	-2.0735	-136.82	0.9346	0.4857	-52.278	1.4498	-56.217	11.48	-59.7	-10.164	-77.305	-49.473	-51.049	-5.4437	7.6113	4.7928	-51.831	-52.53	-5.268	-48.592	-11.653	-123.34	-48.073	0.7226	11.661	-50.866
357	E_nb	Value of the potential energy with all bonded terms disabled. The state of the non-bonded term enable flags will be honored (in addition to the term weights).	MOE	19.229	16.291	-23.997	-10.127	-2.0744	15.813	0.0599	23.296	29.067	-12.307	19.588	-47.9	23.583	14.066	-95.28	19.604	14.081	-13.385	26.739	-4.044	24.709	-18.064	11.477	-28.881	2.9611	-15.623	14.723	27.372	23.224	-8.738	-12.898	10.279	-11.433	9.2128	-80.962	-10.522	22.473	28.214	-0.5723
358	E_oop	Out-of-plane potential energy. In the Potential Setup panel, the term enable (Bonded) flag is ignored, but the term weight is applied.	MOE	0.1247	0.0023	0.812	1.7999	1.9038	0.1724	0.7837	0.0694	0.2648	1.9948	0.1243	0.1768	0.0141	0.1987	0.3406	0.0083	0.2197	1.7023	0.0555	1.6589	0.1596	0.0519	0.0887	1.2482	0.9894	1.0538	0.9877	0.2762	0.1057	1.3073	1.4183	0.3551	0.5237	0.2714	1.4377	1.7885	0.0789	0.1848	1.7843
359	E_sol	Solvation energy. In the Potential Setup panel, the term enable parameter (Solvation menu) is ignored, but the term weight is applied.	MOE	-97.171	-97.816	-141.91	-163.81	-64.05	-98.694	-92.521	-97.93	-177.56	-60.542	-98.08	-53.05	-103.01	-109.45	-89.214	-105.46	-82.291	-61.246	-103.29	-68.761	-99.296	-57.508	-171.22	-117.27	-65.734	-67.626	-115.2	-100.21	-142.1	-63.845	-63.209	-111.01	-66.623	-103.64	-105.64	-67.505	-103.13	-102.94	-71.447
360	E_stb	Bond stretch-bend cross-term potential energy. In the Potential Setup panel, the term enable (Bonded) flag is ignored, but the term weight is applied.	MOE	-0.0466	-0.0111	0.0872	0.2776	0.2372	-0.1556	0.1286	-0.0385	0.4046	0.0971	0.0604	0.2308	-0.1277	0.3327	-0.3783	0.3832	0.0681	0.1052	-0.9352	0.0172	-0.0204	0.2606	0.0989	0.1216	0.3096	0.235	0.3744	0.0814	0.0779	0.3867	0.4407	-0.1062	-0.0287	-0.0931	0.0728	0.2196	0.1655	0.1261	-0.627
361	E_str	Bond stretch potential energy. In the Potential Setup panel, the term enable (Bonded) flag is ignored, but the term weight is applied.	MOE	1.9261	2.0552	4.293	5.1901	6.4019	1.3575	2.6012	3.1875	2.5737	5.7091	2.4663	5.302	2.17	2.7272	5.9239	2.5885	2.7497	5.5607	4.298	6.6319	2.2614	4.8936	2.6628	5.409	6.5837	4.3866	3.0392	3.7811	3.742	5.9784	5.3611	2.5515	5.125	3.3381	6.4955	5.0964	3.3237	3.1191	7.0032
362	E_strain	Local strain energy: the current energy minus the value of the energy at a near local minimum. The current energy is calculated as for the E descriptor. The local minimum energy is the value of the E descriptor after first performing an energy minimization. Current chirality is preserved and charges are left undisturbed during minimization. The structure in the database is not modified (results of the minimization are discarded).	MOE	0.0034	0.0044	0.0031	0.007	0.0055	0.0018	0.0033	0.0061	28.411	0.0048	0.0051	0.0038	0.0063	0.0144	0.0051	0.0039	0.0042	0.0044	0.0059	0.0083	0.0024	0.0016	23.222	0.004	1.2825	0.0017	0.0055	0.0092	0.0086	0.0095	0.0083	0.0117	0.0041	0.0033	0.0075	3.5683	0.0089	0.0052	0.005
363	E_tor	Torsion (proper and improper) potential energy. In the Potential Setup panel, the term enable (Bonded) flag is ignored, but the term weight is applied.	MOE	0.8828	3.6828	9.4187	10.582	13.799	1.8945	4.0608	-3.5798	-3.5676	8.458	1.4702	6.3654	2.5335	1.6217	0.2924	0.8115	3.2911	8.2021	5.3799	12.38	3.2532	3.2824	5.8716	3.0752	16.636	12.871	4.9577	-0.8408	-0.0754	8.8487	12.682	7.7398	19.553	5.6856	9.0146	14.117	3.2385	0.9888	11.971
364	E_vdw	van der Waals component of the potential energy. In the Potential Setup panel, the term enable flag is ignored, but the term weight is applied.	MOE	12.532	12.433	39.158	40.214	49.543	8.1013	19.98	16.99	17.242	40.455	15.241	49.584	12.415	16.14	41.541	18.669	13.595	38.893	25.289	52.174	13.228	41.636	21.641	48.423	52.434	35.425	20.167	19.761	18.431	43.093	39.632	15.547	37.159	20.866	42.376	37.551	21.751	16.553	50.293

Table S12 Amino acid descriptors (2D) used in this study.

			1st aa	A	A	A	A	A	A	A	A	A	A	A	A	A	A	A	A	A	A	A	A	C	C	C	C	C	C	C	C	C	C	C	C	C	C	C	C	C	C	C
			2nd aa	A	C	D	E	F	G	H	I	K	L	M	N	P	Q	R	S	T	V	W	Y	A	C	D	E	F	G	H	I	K	L	M	N	P	Q	R	S	T	V	W
No.	Descriptor ID	Attribute	Ref*	AA	AC	AD	AE	AF	AG	AH	AI	AK	AL	AM	AN	AP	AQ	AR	AS	AT	AV	AW	AY	CA	CC	CD	CE	CF	CG	CH	CI	CK	CL	CM	CN	CP	CQ	CR	CS	CT	CV	CW
365	FASA+	Fractional ASA+ calculated as ASA+ / ASA.	MOE	0.2607	0.3197	0.2446	0.2635	0.4142	0.3538	0.4256	0.2101	0.3867	0.1873	0.2502	0.3847	0.244	0.3973	0.4146	0.3358	0.3277	0.1888	0.4157	0.4093	0.2726	0.3256	0.2906	0.2262	0.3975	0.3574	0.4521	0.264	0.3757	0.2283	0.3087	0.379	0.2192	0.3428	0.4739	0.3561	0.2682	0.2382	0.4205
366	FASA-	Fractional ASA- calculated as ASA- / ASA.	MOE	0.2461	0.393	0.4053	0.3593	0.2863	0.3377	0.2533	0.1731	0.1574	0.1766	0.2999	0.2914	0.2095	0.267	0.1677	0.264	0.2646	0.1822	0.361	0.3323	0.3925	0.4473	0.4806	0.4562	0.4151	0.3748	0.3663	0.2587	0.2783	0.2521	0.3502	0.4396	0.3448	0.4	0.2678	0.3961	0.3761	0.3263	0.4146
367	FASA_H	Fractional ASA_H calculated as ASA_H / ASA.	MOE	0.4987	0.3192	0.3671	0.3874	0.696	0.3231	0.5138	0.6189	0.4565	0.6361	0.4526	0.3377	0.548	0.3414	0.4194	0.4018	0.41	0.6291	0.575	0.5702	0.3666	0.295	0.2474	0.3779	0.5907	0.3761	0.3939	0.5382	0.3814	0.5502	0.3937	0.2188	0.4766	0.3076	0.3043	0.2892	0.3639	0.4433	0.5558
368	FASA_P	Fractional ASA_P calculated as ASA_P / ASA.	MOE	0.5013	0.6808	0.6329	0.6126	0.304	0.6769	0.4862	0.3811	0.5435	0.3639	0.5474	0.6623	0.452	0.6586	0.5806	0.5982	0.59	0.3709	0.425	0.4298	0.6334	0.705	0.7526	0.6221	0.4093	0.6239	0.6061	0.4618	0.6186	0.4498	0.6063	0.7812	0.5234	0.6924	0.6957	0.7108	0.6361	0.5567	0.4442
369	FCASA+	Fractional CASA+ calculated as CASA+ / ASA.	MOE	1.0304	1.3947	1.1886	1.2805	2.0075	1.3985	2.0513	0.8304	2.2451	0.7405	1.1042	2.0479	0.9474	2.1148	3.2054	1.5557	1.518	0.7462	2.1565	2.1402	1.1892	1.5543	1.531	1.1918	2.0897	1.5595	2.3647	1.1517	2.3356	0.9962	1.4886	2.1731	0.9411	1.9654	3.8579	1.7956	1.3523	1.0395	2.3534
370	FCASA-	Fractional CASA- calculated as CASA- / ASA.	MOE	0.973	1.7145	2.3749	2.1053	1.3875	1.3349	1.2208	0.6842	0.7567	0.6981	1.3232	1.5512	0.8136	1.4214	1.1288	1.2233	1.2261	0.7201	1.8729	1.7376	1.7123	2.1351	3.0126	2.8602	2.1819	1.6354	1.9155	1.1287	1.4514	1.0999	1.6891	2.5204	1.4804	2.2933	1.9125	1.9978	1.8969	1.4236	2.3209
371	glob	Globularity, or inverse condition number (smallest eigenvalue divided by the largest eigenvalue) of the covariance matrix of atomic coordinates. A value of 1 indicates a perfect sphere while a value of 0 indicates a two- or one-dimensional object.	MOE	0.1545	0.1988	0.1868	0.1395	0.228	0.2524	0.1435	0.1769	0.0904	0.1586	0.202	0.1253	0.2008	0.1438	0.0438	0.1693	0.2562	0.1435	0.2574	0.1141	0.1839	0.1291	0.2256	0.1849	0.1746	0.2288	0.1133	0.2086	0.1245	0.1588	0.0889	0.1592	0.2618	0.2435	0.0817	0.1614	0.1729	0.2791	0.1861
372	MNDO_dipole	The dipole moment calculated using the MNDO Hamiltonian [MOPAC].	MOE	19.193	16.534	11.841	19.626	5.8828	5.6459	14.888	23.764	21.823	6.3808	5.0584	2.7377	20.576	15.076	20.768	20.805	2.7449	7.0488	18.085	7.2511	13.921	3.7903	21.419	11.878	6.3798	6.0927	22.786	21.587	16.97	6.0823	5.2228	15.557	7.0156	16.8	24.671	4.5813	22.269	20.93	5.8663
373	MNDO_E	The total SCF energy (kcal/mol) calculated using the MNDO Hamiltonian [MOPAC].	MOE	-53398	-58677	-70994	-74594	-72450	-49876	-72460	-64212	-69504	-64301	-65972	-68964	-59951	-72496	-79114	-60833	-64518	-60692	-83369	-79894	-58682	-64025	-76185	-79875	-77728	-55149	-77702	-69480	-74761	-69571	-71244	-74163	-65303	-77767	-84399	-66179	-69706	-65877	-88720
374	MNDO_Eele	The electronic energy (kcal/mol) calculated using the MNDO Hamiltonian [MOPAC].	MOE	-254925	-294475	-358401	-389414	-442912	-220332	-417151	-366930	-408838	-365055	-359194	-361386	-327387	-400419	-477853	-301376	-337114	-328644	-546320	-475509	-294572	-325629	-406327	-439719	-481569	-250489	-460063	-408948	-450231	-406124	-399110	-402131	-362816	-444572	-513067	-336371	-373371	-365958	-586132
375	MNDO_HF	The heat of formation (kcal/mol) calculated using the MNDO Hamiltonian [MOPAC].	MOE	-39.329	-41.76	-234.48	-231.97	-88.675	-122.94	-32.217	-45.751	103.7	-133.76	-130.8	-153.42	-41.897	-82.225	58.549	-85.378	-168.66	-130.14	2.6863	-143.03	-44.629	-111.63	-147.5	-234.71	-56.333	-117.62	4.197	-35.444	120.51	-126.38	-124.6	-74.796	-115.7	-76.304	50.884	-153.7	-78.152	-35.004	-70.374
376	MNDO_HOMO	The energy (eV) of the Highest Occupied Molecular Orbital calculated using the MNDO Hamiltonian [MOPAC].	MOE	-7.6083	-8.0349	-4.7124	-4.1971	-9.2844	-10.497	-7.842	-7.3526	-10.156	-10.404	-9.8578	-10.546	-7.3233	-8.132	-11.761	-7.4922	-10.543	-10.483	-7.6447	-9.0529	-8.157	-10.161	-4.4196	-4.8562	-9.6544	-10.502	-7.1865	-7.4241	-10.201	-10.051	-9.9477	-8.4172	-10.14	-8.3962	-11.888	-10.287	-7.5265	-7.4859	-8.7544
377	MNDO_IP	The ionization potential (kcal/mol) calculated using the MNDO Hamiltonian [MOPAC].	MOE	7.6083	8.0349	4.7124	4.1971	9.2844	10.497	7.842	7.3526	10.156	10.404	9.8578	10.546	7.3233	8.132	11.761	7.4922	10.543	10.483	7.6447	9.0529	8.157	10.161	4.4196	4.8562	9.6544	10.502	7.1865	7.4241	10.201	10.051	9.9477	8.4172	10.14	8.3962	11.888	10.287	7.5265	7.4859	8.7544
378	MNDO_LUMO	The energy (eV) of the Lowest Unoccupied Molecular Orbital calculated using the MNDO Hamiltonian [MOPAC].	MOE	-1.7325	-1.5899	4.0329	3.4677	0.2138	0.7461	-1.5976	-1.8829	-4.1739	0.9015	0.6495	0.678	-1.4649	-1.0826	-4.5961	-1.5965	0.8196	0.9032	-1.5447	-0.0675	-1.2084	0.5021	1.6523	3.6621	-0.1357	0.7751	-1.5205	-1.8422	-3.8734	0.7154	0.7157	-1.4051	0.3923	-1.331	-4.6891	0.5499	-1.9949	-1.7607	-0.2561
379	npr1	Normalized PMI ratio pmi1/pmi3.	MOE	0.2282	0.3825	0.4744	0.3035	0.3804	0.2931	0.4639	0.3359	0.3442	0.384	0.4927	0.2858	0.3786	0.3576	0.1582	0.3529	0.5227	0.37	0.4694	0.2608	0.3228	0.1856	0.422	0.3618	0.4609	0.353	0.4105	0.376	0.2774	0.3423	0.2139	0.3237	0.3114	0.4024	0.2527	0.3666	0.393	0.4519	0.3655
380	npr2	Normalized PMI ratio pmi2/pmi3.	MOE	0.8967	0.8723	0.7609	0.821	0.8643	0.8472	0.716	0.8501	0.7518	0.8405	0.6909	0.8766	0.8716	0.7988	0.8831	0.8879	0.8216	0.8283	0.7832	0.8521	0.9315	0.927	0.8737	0.8224	0.7721	0.9281	0.6778	0.8777	0.9081	0.8328	0.867	0.8771	0.925	0.782	0.8035	0.7947	0.8786	0.9018	0.8203
381	PM3_dipole	The dipole moment calculated using the PM3 Hamiltonian [MOPAC].	MOE	10.798	19.876	11.922	23.29	6.1604	19.269	16.933	22.126	21.518	5.9092	17.995	3.6693	20.999	15.043	10.733	13.085	11.04	6.0342	19.066	6.6694	9.0554	3.0149	12.782	16.023	5.7753	5.734	22.336	20.347	19.48	5.4814	3.9451	17.42	5.921	12.16	22.476	4.8753	22.099	19.754	4.9378
382	PM3_E	The total SCF energy (kcal/mol) calculated using the PM3 Hamiltonian [MOPAC].	MOE	-48456	-52724	-64458	-67884	-66280	-44979	-64820	-58774	-63087	-58832	-59614	-62096	-54605	-65504	-70594	-55230	-58669	-55383	-75794	-73066	-52743	-57065	-68746	-72165	-70576	-49325	-69093	-63057	-67367	-63116	-63957	-66325	-58950	-69788	-74895	-59542	-62938	-59614	-80131
383	PM3_Eele	The electronic energy (kcal/mol) calculated using the PM3 Hamiltonian [MOPAC].	MOE	-256927	-287030	-352060	-388399	-443935	-215001	-420385	-365017	-407702	-365151	-363324	-358380	-322407	-396818	-480754	-299071	-338729	-329272	-558209	-471568	-289054	-320364	-390647	-445695	-475401	-250042	-452892	-405823	-442378	-403759	-394154	-390484	-360835	-444406	-507426	-331190	-369040	-361214	-583414
384	PM3_HF	The heat of formation (kcal/mol) calculated using the PM3 Hamiltonian [MOPAC].	MOE	-102.21	-77.307	-260.36	-242.72	-97.108	-72.268	-58.822	-94.584	52.722	-149.78	-79.981	-177.12	-76.026	-137.66	35.813	-144.52	-143.87	-145.18	-40.974	-152.56	-92.415	-121.63	-249.55	-229.15	-99.307	-118.66	-33.943	-80.771	64.869	-137.56	-124.26	-106.03	-123.95	-123.8	34.402	-161.08	-116.51	-80.963	-79.572
385	PM3_HOMO	The energy (eV) of the Highest Occupied Molecular Orbital calculated using the PM3 Hamiltonian [MOPAC].	MOE	-8.9445	-8.4979	-4.9725	-4.1604	-9.2846	-8.3201	-7.9657	-7.8745	-10.573	-10.011	-8.3163	-9.6979	-7.4033	-9.0113	-11.848	-9.1241	-8.9775	-10.019	-8.1424	-9.068	-9.1022	-9.7273	-5.1127	-4.888	-9.9549	-10.157	-7.3111	-8.0113	-10.681	-9.792	-9.0853	-8.636	-9.7817	-9.3009	-11.556	-9.9789	-8.0033	-8.0152	-8.6183
386	PM3_IP	The ionization potential (kcal/mol) calculated using the PM3 Hamiltonian [MOPAC].	MOE	8.9445	8.4979	4.9725	4.1604	9.2846	8.3201	7.9657	7.8745	10.573	10.011	8.3163	9.6979	7.4033	9.0113	11.848	9.1241	8.9775	10.019	8.1424	9.068	9.1022	9.7273	5.1127	4.888	9.9549	10.157	7.3111	8.0113	10.681	9.792	9.0853	8.636	9.7817	9.3009	11.556	9.9789	8.0033	8.0152	8.6183
387	PM3_LUMO	The energy (eV) of the Lowest Unoccupied Molecular Orbital calculated using the PM3 Hamiltonian [MOPAC].	MOE	-0.6564	-1.5076	3.8268	2.6886	0.3319	-2.0705	-0.7626	-1.7781	-4.6051	0.2677	-1.9488	0.2346	-1.6341	-0.8973	-3.9977	-0.9693	-0.8123	0.2719	-1.3803	0.2257	-1.1829	-0.2991	2.4688	1.6981	-0.4794	-0.6338	-1.1904	-1.7243	-4.191	-0.216	-0.4281	-2.1087	-0.3589	-1.4392	-5.0769	-0.5264	-2.1723	-1.8087	-0.3204
388	pmi	Principal moment of inertia.	MOE	1065.9	1338.7	1350.6	1903.6	1949	873.26	1725.1	1637.9	2274.2	1552	1875.9	1604.1	1168.6	2024.9	2987.7	1074.1	1145.7	1254.2	2612.9	2957.9	1436.7	2083.6	1840.7	2132	2621.2	1058	2499.4	2056.2	2959.9	2102.3	2838.7	2230.4	1611.3	2401	4293.3	1499.4	1723.1	1716.9	3960.1
389	pmi1	First diagonal element of diagonalized moment of inertia tensor.	MOE	228.91	454.13	573.26	543.9	660.54	239.21	734.3	503.38	746.89	535.77	846.52	424	393.29	671.59	463.08	338.33	510.9	422.22	1089.1	730.23	411.42	366.07	676.68	706.36	1082	327.42	982.63	686.08	751.4	661.6	583.46	656.08	448.7	884.59	1055.4	508.62	596.15	659.29	1324.5
390	pmi2	Second diagonal element of diagonalized moment of inertia tensor.	MOE	899.64	1035.8	919.52	1471.3	1500.9	691.3	1133.2	1273.9	1631.5	1172.9	1187.1	1300.6	905.27	1500.2	2585.1	851.2	803.1	945.1	1816.9	2385.8	1187.3	1828.6	1401.1	1605.5	1812.7	860.95	1622.5	1601.5	2459.7	1609.9	2365.6	1777.8	1332.9	1719.2	3355.4	1102.6	1333	1315.6	2972.4
391	pmi3	Third diagonal element of diagonalized moment of inertia tensor.	MOE	1003.3	1187.4	1208.4	1792.1	1736.6	816.01	1582.8	1498.6	2170.1	1395.4	1718.2	1483.7	1038.7	1878	2927.3	958.69	977.44	1141	2319.9	2799.8	1274.7	1972.5	1603.7	1952.2	2347.7	927.62	2393.6	1824.7	2708.6	1933	2728.3	2026.9	1441	2198.3	4175.9	1387.6	1517.1	1458.9	3623.5
392	rgyr	Radius of gyration.	MOE	2.5797	2.6388	2.5783	2.9605	2.8721	2.4444	2.7614	2.8458	3.2278	2.7701	2.9181	2.8097	2.5051	3.0531	3.4829	2.4692	2.4543	2.5813	3.0807	3.4242	2.7338	3.0478	2.7973	2.9246	3.1254	2.4365	3.1107	2.9623	3.4385	2.9953	3.3539	3.079	2.717	3.1035	3.9273	2.6834	2.7843	2.7917	3.5894
393	std_dim1	Standard dimension 1: the square root of the largest eigenvalue of the covariance matrix of the atomic coordinates. A standard dimension is equivalent to the standard deviation along a principal component axis.	MOE	2.3926	2.2796	2.1001	2.544	2.7015	1.9348	2.3899	2.7579	3.0984	2.7186	2.3254	2.6161	2.3448	2.7918	3.5609	2.3616	2.1327	2.5641	2.3463	3.1718	2.2045	2.474	2.2656	2.3151	2.6996	2.0294	2.4148	2.5753	3.2737	2.7374	3.0599	2.6337	2.1398	2.3332	3.489	2.2069	2.5842	2.3538	2.9307
394	std_dim2	Standard dimension 2: the square root of the second largest eigenvalue of the covariance matrix of the atomic coordinates. A standard dimension is equivalent to the standard deviation along a principal component axis.	MOE	1.0984	1.1886	1.4006	1.3282	1.4121	1.1188	1.6529	1.3052	1.6338	1.2838	1.7203	1.2241	1.2418	1.4603	1.4431	1.1949	1.3042	1.2858	1.9483	1.5451	1.3457	1.2249	1.3021	1.4215	1.6729	1.1667	1.8541	1.4723	1.259	1.4024	1.2943	1.2883	1.3346	1.5542	1.6879	1.4087	1.2033	1.3066	1.8623
395	std_dim3	Standard dimension 3: the square root of the third largest eigenvalue of the covariance matrix of the atomic coordinates. A standard dimension is equivalent to the standard deviation along a principal component axis.	MOE	0.9405	1.0165	0.9076	0.9501	1.2901	0.9721	0.9052	1.1601	0.9315	1.0826	1.045	0.926	1.0507	1.0587	0.745	0.9717	1.0794	0.9712	1.1903	1.0712	0.9454	0.889	1.0761	0.9956	1.1281	0.9707	0.8127	1.1761	1.1553	1.091	0.9122	1.0507	1.095	1.1513	0.9971	0.8867	1.0746	1.2436	1.2642
396	vol	van der Waals volume calculated using a grid approximation (spacing 0.75 A).	MOE	151.63	170.38	170.63	189.5	228	132.25	205.13	204.75	219.25	201.75	206.75	176.75	176.88	200.13	233.25	158	176.88	183.13	262.88	234.13	169.13	185.75	191	204.38	246.75	149.63	226	223.5	239.5	221.25	223.75	199.75	192.63	215.88	252.63	175.25	193.63	205.38	278.63
397	VSA	van der Waals surface area. A polyhedral representation is used for each atom in calculating the surface area.	MOE	191.61	208.22	204.33	227.65	261.57	166.8	240.1	249.19	269.29	242.6	252.38	210	212.23	246.36	277.67	196.59	214.08	221.78	298.05	274.59	212.31	218.59	225.04	237.42	281.57	179.25	265.88	265.31	289.01	259	267.35	245.91	222.06	257.35	304.47	211.65	234.65	246.64	317.48
398	vsurf_A	Amphiphilic moment	MOE	2.7357	2.6829	2.4596	3.2288	4.2824	3.2183	1.5496	1.8119	3.7496	4.7373	3.1277	2.5142	4.3512	3.2233	1.2025	3.2493	1.6633	3.9962	4.2024	4.0852	3.2494	3.4905	2.3965	1.8249	5.2729	3.8387	2.4462	2.8016	2.6547	4.4752	4.481	3.4586	4.2061	2.8507	2.2382	2.3328	1.9198	2.8079	4.9292
399	vsurf_CP	Critical packing parameter	MOE	0.048	0.0528	0.0302	0.0329	0.0926	0.046	0.0526	0.0751	0.0535	0.071	0.0703	0.0396	0.0698	0.0382	0.0546	0.033	0.0318	0.0641	0.0836	0.0622	0.0503	0.0612	0.0314	0.0306	0.0974	0.0483	0.0511	0.067	0.0583	0.0811	0.0652	0.035	0.071	0.0382	0.0435	0.0312	0.0397	0.066	0.0807
400	vsurf_CW1	Capacity factor (8 descriptors)	MOE	3.0778	3.0519	3.1054	3.098	2.8991	3.2269	3.008	2.8825	3.0387	2.8865	2.8711	3.1698	3.0625	3.0675	2.9676	3.156	3.1214	2.9366	2.8935	2.9416	3.0489	3.014	3.0681	3.0112	2.8473	3.1608	2.9694	2.8807	2.9426	2.854	2.8757	3.0325	3.0131	3.004	2.9982	3.1091	3.032	2.9688	2.8358
401	vsurf_CW2	Capacity factor (8 descriptors)	MOE	2.5114	2.6337	2.6914	2.6904	2.4173	2.7027	2.5614	2.1566	2.6824	2.0472	2.2799	2.7295	2.5116	2.6986	2.5943	2.6648	2.7516	2.0956	2.4578	2.5232	2.6905	2.7248	2.8129	2.7705	2.4813	2.7935	2.702	2.3374	2.7067	2.1481	2.4046	2.8009	2.6292	2.7713	2.7464	2.8494	2.6402	2.5014	2.5383
402	vsurf_CW3	Capacity factor (8 descriptors)	MOE	1.9395	2.0232	2.2226	2.238	1.4077	2.1366	1.8685	1.5427	1.9446	1.499	1.5779	2.3025	1.6864	2.1278	1.904	2.0397	2.2247	1.5725	1.6777	1.7655	2.0631	2.0709	2.3276	2.1984	1.581	2.1461	1.9236	1.6713	2.036	1.5939	1.6444	2.2586	1.833	2.2773	2.0727	2.2853	2.1101	1.8259	1.6839
403	vsurf_CW4	Capacity factor (8 descriptors)	MOE	1.1296	1.1214	1.476	1.4424	0.7813	1.2944	1.1236	0.8975	1.0904	0.9144	0.9105	1.3784	1.0178	1.3045	1.0417	1.3451	1.3742	0.9989	0.9	0.9965	1.0951	0.9913	1.4098	1.3268	0.8296	1.2415	1.0892	0.9207	1.0044	0.8467	0.8729	1.3436	0.9904	1.2646	1.1255	1.3464	1.2756	0.9804	0.8683
404	vsurf_CW5	Capacity factor (8 descriptors)	MOE	0.5995	0.6259	0.8892	0.7985	0.4458	0.7216	0.6216	0.4648	0.494	0.5011	0.4921	0.7747	0.5193	0.7269	0.553	0.8175	0.726	0.543	0.4954	0.5347	0.5844	0.5042	0.8552	0.8386	0.4288	0.6259	0.5832	0.5241	0.4512	0.4412	0.4738	0.748	0.493	0.6854	0.6164	0.7605	0.6533	0.5356	0.4705
405	vsurf_CW6	Capacity factor (8 descriptors)	MOE	0.2434	0.3282	0.5168	0.376	0.2173	0.361	0.2843	0.2166	0.2022	0.212	0.208	0.3693	0.2356	0.3461	0.2577	0.3906	0.3451	0.226	0.2559	0.2039	0.2453	0.2425	0.4905	0.4246	0.1824	0.229	0.2654	0.2623	0.1847	0.1745	0.2184	0.3597	0.2276	0.3122	0.2794	0.3273	0.2984	0.2315	0.2033
406	vsurf_CW7	Capacity factor (8 descriptors)	MOE	0.097	0.1599	0.2445	0.1293	0.0801	0.1367	0.1082	0.0939	0.0707	0.062	0.0807	0.1621	0.0834	0.1478	0.1086	0.1267	0.188	0.0617	0.1322	0.0569	0.0815	0.1176	0.247	0.1404	0.0604	0.0706	0.0774	0.0951	0.0723	0.0573	0.0651	0.1372	0.0654	0.1028	0.0845	0.0968	0.1041	0.0891	0.061
407	vsurf_CW8	Capacity factor (8 descriptors)	MOE	0.0393	0.0872	0.092	0.0276	0.0246	0.0711	0.0343	0.0326	0.0181	0.018	0.0323	0.0515	0.0281	0.0353	0.0516	0.0262	0.0935	0.0181	0.0693	0.0144	0.0263	0.0421	0.099	0.0495	0.0166	0.0227	0.0186	0.0331	0.0272	0.019	0.0169	0.0435	0.0205	0.0202	0.0138	0.0201	0.0276	0.0315	0.0157
408	vsurf_D1	Contact distances of vsurf_EWmin (3 descriptors)	MOE	308.13	351.88	315.25	348.5	583.63	264.25	420.5	456.75	423.75	436.25	477.25	346.5	392.5	374.25	523.25	266.88	244.63	372	601.38	537	349.38	446.5	364.13	392.13	626.63	328.13	479.5	485.25	497.88	523.13	532.38	364.38	430.63	414	531.25	280	338.88	427.25	696.5
409	vsurf_D2	Contact distances of vsurf_EWmin (3 descriptors)	MOE	168.75	195.88	166.63	192.63	330.38	146.25	235.75	247.88	237	236.13	263	202.38	213.75	210.75	296.25	145.75	131.88	202.88	355.13	314.5	190.25	241.38	193.63	201.25	355.88	169.5	263.63	265	267	276.75	294.88	198.88	233.63	226.88	301	150	174.63	234	405.38
410	vsurf_D3	Contact distances of vsurf_EWmin (3 descriptors)	MOE	125.88	142.38	114.75	137	235.75	107.88	168.25	191.25	170.5	188.75	192.25	149.25	152.63	152.75	211.63	106.38	97.875	165.75	258.25	225.63	134.88	169.88	128.75	139.13	243.75	117.13	180.75	191.25	186.63	205.5	214.5	138.38	166.5	155.25	214.75	102.75	125.75	172.25	285
411	vsurf_D4	Contact distances of vsurf_EWmin (3 descriptors)	MOE	99.375	113.13	86.875	103.63	198	85.25	134.88	162.75	131.13	163.63	157	112	128.88	116.38	169.38	85.875	72.5	141.63	213.38	180.75	102.75	123.38	93.875	100.5	198	90.875	139.38	157.75	140	175.25	174.63	102.38	133	112.38	159.75	73.875	95.5	137.38	222.13
412	vsurf_D5	Contact distances of vsurf_EWmin (3 descriptors)	MOE	79.125	83.75	68.375	82.25	169.63	68.5	110.25	132.88	99	136.13	126.25	84.25	107.88	85.875	132.5	67.5	48.25	117	177.75	145.88	75.875	89.25	69	73.75	168.5	69.75	109.25	130.75	99	142.13	138.63	70.875	103.13	78.875	114.88	49.375	68.625	110.38	177.75
413	vsurf_D6	Contact distances of vsurf_EWmin (3 descriptors)	MOE	50.125	53	48.375	55.375	134.5	44.75	78.75	83.625	68.875	78.75	86.75	56.25	87.125	60.5	97.875	44.875	28.125	64.875	140.75	107	51.5	63.125	51.875	52.75	139.13	53.375	80.75	90.875	70.125	86.5	97.375	50.75	75	54.25	86.75	32.5	40	79.625	142.75
414	vsurf_D7	Contact distances of vsurf_EWmin (3 descriptors)	MOE	27.625	28	31.5	36.75	77.75	24.75	52.25	38.875	43.125	32.125	47.75	38	47.125	39.25	62.25	25	14	24.375	93.125	71.625	24.25	39.75	37.625	34.25	79.375	30.5	50.875	44.875	36.375	36.625	56.5	28.5	38.125	31.125	54.25	16.625	19.25	40.25	93.375
415	vsurf_D8	Contact distances of vsurf_EWmin (3 descriptors)	MOE	16.125	16.125	20.625	22.5	31.375	16.5	27.5	16.5	21.375	15.875	20.5	26	20.75	26.375	35.625	10.25	9.375	11.125	52.625	37.875	13	24.25	22.375	16.625	31.875	14	23.875	19.75	16.5	15.75	20.75	13.375	16.5	14.875	34.625	5.75	8.375	18.875	49.375
416	vsurf_DD12	Contact distances of vsurf_DDmin (3 descriptors)	MOE	0.5	0.5	0.5	0.5	0.7071	1	0.7071	0.5	0.5	0.5	7.3824	0.5	0.5	0.7071	0.5	7.1239	0.7071	0.5	0.7071	0.7071	7.1239	0.5	0.7071	0.5	0.5	6.5383	6.5383	1.5	0.5	6.1644	0.7071	0.866	7.3485	0.7071	1.2247	1.118	6.9821	0.5	0.7071
417	vsurf_DD13	Contact distances of vsurf_DDmin (3 descriptors)	MOE	0.5	0.866	0.5	5.831	1.118	6.5192	0.5	0.5	1.2247	0.5	0.7071	1.118	0.5	0.5	0.7071	7.0356	0.5	6.8739	0.7071	1.118	0.5	0.5	0.5	1.118	0.5	7.0356	6.9821	0.7071	0.5	6.364	0.5	0.7071	6.6895	1	0.5	0.5	7.1764	0.5	6.8007
418	vsurf_DD23	Contact distances of vsurf_DDmin (3 descriptors)	MOE	0.7071	0.7071	0.7071	6.1033	0.5	6.6708	0.5	1	0.866	0.7071	7.3485	0.7071	0.7071	0.5	0.5	0.5	0.5	7.1764	0.7071	0.5	6.7454	0.7071	0.866	0.7071	1	6.1847	3	0.866	0.7071	0.7071	0.5	1.118	7.9215	1.2247	0.866	0.7071	0.5	1	6.5765
419	vsurf_DW12	Contact distances of vsurf_EWmin (3 descriptors)	MOE	0.5	1.118	7.5	0.7071	0.5	0.5	0.7071	7.5	0.5	0.7071	1.2247	7.7942	0.5	0.866	0.5	0.5	0.5	0.5	0.5	0.866	0.5	0.5	7.5829	0.7071	0.7071	0.5	0.5	0.5	0.5	0.5	0.5	0.7071	0.5	0.7071	0.5	0.5	0.5	0.7071	0.5
420	vsurf_DW13	Contact distances of vsurf_EWmin (3 descriptors)	MOE	1.118	0.866	7.2284	0.5	1.118	0.7071	0.7071	0.7071	0.866	0.5	0.866	0.5	0.5	0.7071	0.5	1.118	0.7071	0.5	0.7071	0.5	0.5	7.6322	0.5	0.7071	1.118	8.1394	0.866	0.5	0.5	0.7071	0.5	0.7071	0.7071	0.5	0.5	0.5	0.5	1.118	0.5
421	vsurf_DW23	Contact distances of vsurf_EWmin (3 descriptors)	MOE	0.7071	0.7071	0.7071	0.5	0.7071	0.5	1.2247	7.2284	1.2247	1.118	0.5	7.7136	1	0.5	1	0.7071	0.5	0.7071	0.5	0.7071	1	7.5333	7.5333	0.5	0.866	0.7071	8.0932	1.2247	0.7071	0.866	0.7071	1.2247	0.5	0.866	0.7071	1	1	0.5	1
422	vsurf_EDmin1	Lowest hydrophobic energy (3 descriptors)	MOE	-2.0855	-2.4599	-2.3417	-2.1027	-2.3636	-2.2612	-2.2514	-2.4658	-2.4318	-2.3198	-2.1652	-2.6944	-2.2387	-2.5834	-2.9386	-2.1074	-2.4357	-2.1007	-2.5449	-2.9379	-2.2936	-2.5506	-2.4275	-2.457	-3.0058	-2.3153	-2.2225	-2.5021	-2.6922	-2.4038	-2.348	-2.32	-2.2361	-2.4633	-2.5394	-1.9324	-2.3301	-2.4294	-2.6587
423	vsurf_EDmin2	Lowest hydrophobic energy (3 descriptors)	MOE	-2.0784	-2.3841	-2.3416	-2.0725	-2.3355	-2.1488	-2.2452	-2.4536	-2.3412	-2.3188	-2.1394	-2.6475	-2.2383	-2.5285	-2.9017	-1.9629	-2.3788	-2.0983	-2.5447	-2.8566	-2.1835	-2.5354	-2.3921	-2.4449	-2.9548	-2.2086	-2.2095	-2.4606	-2.6282	-2.3918	-2.3421	-2.3073	-2.2262	-2.3685	-2.4592	-1.8883	-2.2619	-2.3856	-2.5509
424	vsurf_EDmin3	Lowest hydrophobic energy (3 descriptors)	MOE	-2.0726	-2.3762	-2.3089	-2.0324	-2.3342	-2.1111	-2.1992	-2.431	-2.3354	-2.2717	-2.1345	-2.6336	-2.1789	-2.5155	-2.8174	-1.9583	-2.3476	-2.0914	-2.5261	-2.7617	-2.0893	-2.5052	-2.3521	-2.4023	-2.9365	-2.1186	-2.209	-2.4554	-2.4899	-2.3578	-2.3349	-2.2071	-2.2095	-2.2265	-2.4351	-1.8677	-2.2603	-2.3667	-2.5278
425	vsurf_EWmin1	Lowest hydrophilic energy (3 descriptors)	MOE	-8.3492	-9.8577	-8.2251	-6.9361	-7.0259	-10.38	-8.0772	-7.6066	-7.095	-7.1076	-8.218	-7.6318	-8.1147	-7.7111	-9.0489	-7.393	-8.6195	-7.1375	-10.107	-7.0189	-8.4808	-7.586	-8.1212	-10.467	-6.9974	-6.9438	-7.0637	-7.6585	-8.4451	-7.046	-6.9556	-8.1793	-7.0252	-7.3063	-6.9971	-7.0595	-8.3297	-8.0037	-7.1313
426	vsurf_EWmin2	Lowest hydrophilic energy (3 descriptors)	MOE	-8.056	-9.4937	-7.8535	-6.8648	-7.0228	-10.193	-7.6928	-7.4048	-7.0362	-6.8359	-8.1314	-7.4279	-7.9339	-7.3362	-8.834	-6.9811	-8.4617	-7.0092	-10.031	-6.8423	-7.6894	-7.3157	-7.8163	-9.4884	-6.9289	-6.8673	-6.9711	-7.3337	-8.3462	-6.8948	-6.9441	-7.8976	-7.0005	-7.0676	-6.9313	-6.8288	-8.201	-7.5713	-6.9185
427	vsurf_EWmin3	Lowest hydrophilic energy (3 descriptors)	MOE	-7.784	-9.4244	-7.5341	-6.8551	-6.9249	-9.9537	-7.3294	-7.2932	-6.9543	-6.8139	-7.8759	-7.3692	-7.5723	-7.062	-8.4125	-6.9226	-8.1938	-6.675	-9.6162	-6.7695	-7.6145	-7.1382	-7.7179	-9.0962	-6.9239	-6.6899	-6.6158	-7.1861	-8.3245	-6.8124	-6.9007	-7.8455	-6.9186	-7.0469	-6.9183	-6.6922	-7.4389	-7.4143	-6.8475
428	vsurf_G	Surface globularity	MOE	1.2162	1.2122	1.2084	1.2389	1.1895	1.1934	1.1982	1.2382	1.2564	1.2195	1.2394	1.2119	1.1707	1.2558	1.273	1.1964	1.1865	1.2031	1.2072	1.253	1.2218	1.2018	1.211	1.2191	1.2352	1.172	1.2275	1.2183	1.2843	1.2285	1.2415	1.2601	1.1781	1.2546	1.3026	1.2077	1.2238	1.1986	1.2731
429	vsurf_HB1	H-bond donor capacity (8 descriptors)	MOE	251.88	251.88	357	358.63	196.13	309	262.88	208.63	200.5	259	251	276.88	238.5	245.25	148.63	248.5	263.75	247.13	272.5	301.5	274.5	228.63	329.38	312.63	283.5	283.25	255	235.38	226.5	226.13	246	284.38	275.38	314.75	215.38	298.63	289.5	253.25	270.13
430	vsurf_HB2	H-bond donor capacity (8 descriptors)	MOE	333	341.5	450.13	500.25	258.75	331.25	338.25	263.5	256.5	292.13	330.88	346.5	302.75	330.38	182.5	342.13	387.5	292.13	321.38	389.38	393.5	358.38	456.13	487.88	383.13	358.25	378.63	292.5	332.25	285.13	334.25	422.75	382.5	452.5	310.38	439.63	409.88	347.88	388.75
431	vsurf_HB3	H-bond donor capacity (8 descriptors)	MOE	236.63	272.38	377.63	397.5	202.63	256.88	260.75	215.13	215.63	226.38	247	308.75	237.5	290.5	189.75	272.38	308.25	221.13	255	286.75	274.75	272.25	394.63	398.63	270.63	275.88	272.63	247.5	249.25	235.13	243.88	338.75	274.25	357.13	248.63	328.88	329.88	257.88	273.5
432	vsurf_HB4	H-bond donor capacity (8 descriptors)	MOE	113.13	138.75	211.63	200.88	102.88	133.38	135.13	113.5	110.75	112.75	118.25	164.63	113.63	162.38	113.88	155.38	158.25	114.25	131.25	137	122.63	116.38	215.13	212.5	119.88	126.13	135.88	132	111.38	110.63	116.13	173.63	121.75	169.75	122.25	160.25	162.38	122.38	129.25
433	vsurf_HB5	H-bond donor capacity (8 descriptors)	MOE	50.125	69.875	107.75	92	50.375	68.25	66	53	46.25	53.125	52.125	82.125	51.125	80.5	56	80.5	75.875	50	68.125	62.875	57	51.125	115	107.5	54	55.125	62.625	66.375	45.375	46.75	53.375	83.125	49.5	79.25	52	75.125	70.75	56.375	57.625
434	vsurf_HB6	H-bond donor capacity (8 descriptors)	MOE	20.875	37.25	58.375	39.875	23.875	35.375	30.5	24.75	22	20.5	20.875	35.5	23.125	39.5	26.875	35.875	36.75	20.75	35.125	22.5	21.5	24.875	59	46.5	22.625	20.25	27.875	30.875	20.125	18.75	22.5	38.625	22.375	35	22	29.125	28.875	24.25	23.125
435	vsurf_HB7	H-bond donor capacity (8 descriptors)	MOE	8.625	16.75	24.25	12.75	7.5	14	12.25	10.25	8.125	6.75	8.375	16.125	8.375	15.125	12.875	10.75	19.5	6.375	18.625	7	7.5	13.125	26.5	15.375	7.375	6.125	8	10.625	7.875	6.75	7.5	15.125	6.625	10.5	6.5	9.125	11.25	9.25	8.25
436	vsurf_HB8	H-bond donor capacity (8 descriptors)	MOE	3.125	9.375	8.75	3	2.5	6.875	3.375	3.25	1.75	1.75	3.5	4.125	2.375	3.875	6.25	2.125	8.75	1.5	9	1.625	2.25	3.5	9.75	5.625	1.875	1.75	1.875	3.5	3.5	2	2.125	4.375	1.875	2.625	1.125	2	3	3	1.75
437	vsurf_HL1	Hydrophilic-Lipophilic (2 descriptors)	MOE	0.5035	0.5698	1.0098	0.7527	0.2609	0.7358	0.4725	0.334	0.3878	0.3424	0.3466	0.6482	0.3898	0.6579	0.3828	0.8825	0.9119	0.3778	0.3088	0.3429	0.5134	0.3738	0.9505	0.823	0.2585	0.5358	0.4474	0.3856	0.3577	0.2835	0.3147	0.7308	0.3461	0.6119	0.454	0.854	0.663	0.3824	0.2605
438	vsurf_HL2	Hydrophilic-Lipophilic (2 descriptors)	MOE	0.2491	0.3425	0.6863	0.4041	0.1288	0.4296	0.241	0.159	0.1849	0.1406	0.1584	0.3415	0.1891	0.3641	0.1978	0.4469	0.531	0.1624	0.1705	0.1432	0.2421	0.2188	0.6312	0.4776	0.1206	0.2311	0.2269	0.1981	0.175	0.117	0.1496	0.4176	0.1805	0.3404	0.234	0.4281	0.3285	0.1874	0.1221
439	vsurf_ID1	Hydrophobic integy moment (8 descriptors)	MOE	0.7222	0.9248	1.433	1.3208	1.632	1.4574	0.519	0.7622	1.4232	1.9248	0.8091	1.0349	1.9183	1.4199	0.3417	1.6214	0.9027	1.7526	1.5314	1.6182	1.2944	1.2473	1.4156	0.4422	1.2282	1.5748	0.9686	1.2524	0.8259	1.4836	1.6884	1.4907	1.5863	1.3184	1.1429	1.6416	0.9101	1.2323	2.118
440	vsurf_ID2	Hydrophobic integy moment (8 descriptors)	MOE	0.7951	1.0263	1.334	1.4098	1.5634	1.3858	0.5127	0.6503	1.5761	1.8371	0.7176	1.152	1.8339	1.4999	0.4024	1.6318	0.8672	1.607	1.4778	1.6303	1.2598	1.3676	1.282	0.5055	2.0712	1.4108	1.1729	1.1426	0.7151	1.4995	1.6675	1.5857	1.5942	1.2829	1.3165	1.6982	0.8187	1.195	2.1249
441	vsurf_ID3	Hydrophobic integy moment (8 descriptors)	MOE	0.9309	1.0966	1.5509	1.6607	1.4571	1.6369	0.7472	0.6341	1.5251	2.0332	0.9375	1.4036	1.8743	1.6125	0.2058	1.7655	0.9211	1.7676	1.3424	1.508	1.3838	1.329	1.2541	0.6865	2.023	1.5584	1.1545	1.2246	0.7791	1.7914	1.6887	1.6688	1.5902	1.3362	1.3948	1.9086	0.8343	1.3333	2.0737
442	vsurf_ID4	Hydrophobic integy moment (8 descriptors)	MOE	0.9869	1.2547	1.7531	1.9229	1.6863	1.9331	0.9064	0.7686	1.6575	2.3467	1.2786	1.6134	2.0936	1.7473	0.2834	1.9102	1.1857	1.922	1.505	1.6042	1.4998	1.4906	1.2592	0.4781	2.3453	1.762	1.4587	1.5644	0.8412	2.1163	2.0758	1.727	1.7828	1.3688	1.4853	1.8469	0.9831	1.6316	2.4213
443	vsurf_ID5	Hydrophobic integy moment (8 descriptors)	MOE	1.0018	1.5689	1.9607	1.9449	1.9426	2.1174	0.8644	0.9309	1.9004	2.6025	1.5035	1.8302	2.4302	1.8862	0.2942	1.9387	1.4489	2.0614	1.6671	1.6554	1.6337	1.5036	1.3566	0.2018	2.6646	1.8614	1.958	1.7593	0.8186	2.391	2.6748	1.6402	2.0265	1.2774	1.7326	1.8063	1.264	1.9487	2.905
444	vsurf_ID6	Hydrophobic integy moment (8 descriptors)	MOE	0.9433	1.3339	1.4224	1.5635	2.5213	1.8088	0.9097	1.3751	2.371	2.3994	1.6723	1.5093	2.8219	1.9081	0.9604	1.9575	1.4134	2.0712	1.9759	2.1695	1.703	1.8524	1.3759	0.5407	2.9865	1.8169	2.613	1.7411	0.8974	2.1051	2.8768	1.7376	2.3443	1.2121	2.1421	1.8436	0.8179	2.0865	3.2493
445	vsurf_ID7	Hydrophobic integy moment (8 descriptors)	MOE	2.2533	1.3763	1.0872	1.4995	2.5148	2.1666	1.2514	1.1413	2.655	1.6684	1.5425	1.3073	2.6368	1.9742	1.4127	2.2111	2.8677	1.5493	1.824	2.4518	1.2241	1.747	1.28	1.16	2.7556	1.2168	2.9303	1.4736	1.373	1.5547	2.918	1.5717	1.9673	1.2689	2.6428	2.331	0.428	1.1671	3.0725
446	vsurf_ID8	Hydrophobic integy moment (8 descriptors)	MOE	2.6584	1.6	1.6631	2.2088	2.455	2.2797	0.9016	1.4164	2.3847	1.4712	1.669	1.1164	2.281	1.916	1.6379	2.773	2.9813	1.6197	1.5781	2.1937	1.8637	1.5081	1.7109	1.3891	2.0799	1.3218	3.4141	1.7169	2.0657	1.3568	1.9471	1.7339	1.1625	2.1059	2.8251	2.7655	1.0477	0.8469	2.7602
447	vsurf_IW1	Hydrophilic integy moment (8 descriptors)	MOE	0.4017	0.1339	0.3108	0.4237	0.417	0.4113	0.3794	0.3136	0.4282	0.615	0.4532	0.265	0.4619	0.3152	0.2418	0.3469	0.1726	0.5564	0.3997	0.1578	0.5437	0.3632	0.3599	0.405	0.4056	0.6857	0.228	0.4941	0.3775	0.4748	0.4609	0.4459	0.5283	0.5681	0.5246	0.404	0.3791	0.4507	0.325
448	vsurf_IW2	Hydrophilic integy moment (8 descriptors)	MOE	0.6674	0.2914	0.6645	0.747	1.1756	0.8457	0.5643	0.7038	0.6985	1.7112	0.9892	0.4572	1.2858	0.6094	0.2827	0.6484	0.2387	1.3523	1.0372	0.6113	0.8442	0.7128	0.573	0.4417	1.4226	1.1787	0.371	1.1161	0.4672	1.4039	1.3372	0.6866	1.1009	0.8632	0.43	0.5541	0.5522	0.9901	1.1699
449	vsurf_IW3	Hydrophilic integy moment (8 descriptors)	MOE	0.9294	0.7139	0.7284	0.9789	2.099	1.1172	0.6697	0.8922	1.0328	2.066	1.3396	0.6358	1.9219	0.932	0.3969	0.9727	0.3109	1.6294	1.7565	1.3164	1.219	1.175	0.7702	0.6584	2.3577	1.4882	0.8052	1.3993	0.5349	1.7737	1.9469	1.0808	1.6729	1.1989	0.5411	0.8908	0.63	1.2799	2.0394
450	vsurf_IW4	Hydrophilic integy moment (8 descriptors)	MOE	1.3288	1.302	0.8032	1.2132	2.6465	1.458	0.8032	0.9117	1.5881	2.4235	1.6415	0.8333	2.273	1.3623	0.6944	1.3018	0.5656	1.9215	2.464	2.0329	1.8289	1.9928	1.1645	1.0581	2.986	1.9965	1.2583	1.6654	1.0231	2.4088	2.4441	1.6719	2.3805	1.5349	0.9328	1.3159	0.9687	1.6081	2.6808
451	vsurf_IW5	Hydrophilic integy moment (8 descriptors)	MOE	1.8995	1.711	0.9183	1.5729	2.8853	1.7204	0.9483	1.2247	2.3268	2.7266	2.2188	1.1838	2.4953	1.6505	1.0565	1.5073	0.9346	2.2673	2.866	2.6031	2.1308	2.1833	1.2118	1.2798	3.3104	2.3155	1.3737	1.7347	2.0593	2.7957	2.8478	1.8488	2.7062	1.5589	0.9523	1.3894	1.2773	1.5945	2.9224
452	vsurf_IW6	Hydrophilic integy moment (8 descriptors)	MOE	3.0111	2.1895	0.9921	1.9875	3.4041	2.4292	1.235	1.4156	2.8874	3.1666	2.8764	1.5939	2.8084	2.1219	1.3828	1.7924	1.4429	2.8092	3.2194	3.4997	2.7246	2.4	1.3351	1.7753	3.8217	3.3077	1.7775	1.7941	3.0642	3.6662	3.3724	2.1306	3.3011	1.4757	1.0733	1.769	1.5778	1.601	3.4138
453	vsurf_IW7	Hydrophilic integy moment (8 descriptors)	MOE	3.4101	2.9195	1.2089	2.5968	3.7972	3.4796	1.4877	1.5865	2.8434	4.2816	3.8014	2.2643	3.3091	2.5561	2.1713	2.5918	2.1631	4.2562	3.6542	5.004	3.7718	2.5157	1.1108	3.0654	4.7224	4.6913	2.6804	2.171	3.8486	4.5917	4.5017	2.33	4.5408	1.6408	1.7473	3.1063	1.3858	1.114	4.0098
454	vsurf_IW8	Hydrophilic integy moment (8 descriptors)	MOE	3.1612	3.1905	1.6204	3.0354	4.1912	3.6815	2.3266	1.8564	2.6639	4.9827	3.737	2.4097	3.5081	2.7994	2.6307	3.2442	2.6888	4.6614	3.7928	5.9377	3.5409	2.6078	1.1149	3.6632	5.494	4.9227	2.8747	1.7748	4.1912	4.9975	5.1881	1.4193	5.1849	2.4449	4.2981	4.8587	2.7938	1.4883	5.2711
455	vsurf_R	Surface rugosity	MOE	1.2291	1.2795	1.2798	1.2838	1.3987	1.2127	1.3509	1.3127	1.3118	1.3293	1.3244	1.2787	1.3223	1.2859	1.3147	1.2524	1.2899	1.3093	1.4398	1.3558	1.2676	1.3159	1.3155	1.3229	1.3901	1.2619	1.3584	1.3646	1.3203	1.3504	1.3466	1.2908	1.3433	1.3143	1.3244	1.2763	1.3027	1.3561	1.3963
456	vsurf_S	Interaction field surface area	MOE	307.34	329.81	326.91	354.42	372.41	282.73	355.05	369.95	386.01	362.41	377.63	329.14	317.25	370.37	403.32	303.81	314.27	337.6	412.46	408.98	331.44	339.96	347.58	358.58	411.85	289.93	385.94	380.87	417.61	382.38	392.46	377.09	333.7	385.77	438.5	324.56	351.77	358.11	454.96
457	vsurf_V	Interaction field volume	MOE	377.75	422	418.38	455	520.88	342.88	479.63	485.63	506.38	481.75	500.13	420.88	419.5	476.25	530.25	380.5	405.38	442	593.88	554.5	420.13	447.38	457.25	474.38	572.5	365.88	524.25	519.75	551.38	516.38	528.5	486.75	448.25	507	580.75	414.25	458.25	485.63	635.25
458	vsurf_W1	Hydrophilic volume (8 descriptors)	MOE	878.25	961.38	1027.8	1120.4	1022.9	887.13	1034.1	953.5	1140.8	940.38	990.88	1068.6	910.5	1137.9	1168.8	931.63	951.63	891.75	1157.3	1167	967.75	999.25	1086.8	1084.5	1129	900.25	1131.6	1010.5	1197.9	997.88	1056.8	1153.5	965.38	1169	1340.8	998.5	1037.3	1002.1	1277.9
459	vsurf_W2	Hydrophilic volume (8 descriptors)	MOE	605.88	676.63	744.13	826.13	624.75	626	707.38	590.25	802	580.88	631.88	780.25	589	821.75	790.25	668.25	716	564.5	744.25	788	683.5	702.63	807.13	803.88	709.75	631.63	793.75	645.63	842.63	612.75	687	857.88	647.88	863.13	964.75	758.63	752	672.5	842.75
460	vsurf_W3	Hydrophilic volume (8 descriptors)	MOE	353.25	402.13	480.38	513.25	323.5	380.25	406.88	343.88	452.38	339	359.88	482.75	335.25	502.25	460.25	412.5	444.5	335.38	412.38	434.5	394.25	402.38	515.25	495.88	376.25	375.63	447.5	376.75	472	359.25	385	525.5	360.5	523.13	562.5	459.75	464.75	384.5	451
461	vsurf_W4	Hydrophilic volume (8 descriptors)	MOE	151.5	178.25	239.25	235.13	136.5	169.88	180	150.13	182.25	148.88	153.88	210.63	141.38	225.38	190.88	196.88	199.13	150.63	171.63	178.88	158.63	150.5	244.25	233.63	148.88	154.13	190.5	162.5	176.75	143	157.5	229.13	144.13	217.38	230	199.38	202	157.13	179.25
462	vsurf_W5	Hydrophilic volume (8 descriptors)	MOE	63.375	81.125	115.88	103.13	61.5	79.375	79.5	63.875	66.125	64.625	66.625	96.75	59.5	100.5	81	93.875	89.25	62.625	79.75	77.375	69.25	63.5	122.38	114.5	63	62.75	80.875	73.75	66.75	58.25	67.5	101.13	57.625	95	97.5	87.75	83.375	65.875	74.25
463	vsurf_W6	Hydrophilic volume (8 descriptors)	MOE	24.75	38.75	59.625	41.875	25.5	36.625	32.5	25.875	24.25	23	24.875	38.25	24.375	42.375	33.5	38.375	38.5	23	36.375	25.875	24.875	27	59.25	48	23.875	21	31.625	31.25	24.5	20.5	26.125	42.75	24	38.25	37.375	31.625	31.375	25.75	27.125
464	vsurf_W7	Hydrophilic volume (8 descriptors)	MOE	9	16.75	24.25	12.75	7.5	14	12.25	10.25	8.125	6.75	8.75	16.125	8.375	15.125	13.75	10.75	19.5	6.375	18.625	7.25	7.75	13.125	26.5	15.375	7.375	6.125	8	10.625	8.875	6.75	7.5	15.25	6.625	10.75	10.75	9.125	11.25	9.25	8.5
465	vsurf_W8	Hydrophilic volume (8 descriptors)	MOE	3.125	9.375	8.75	3	2.5	6.875	3.375	3.25	1.75	1.75	3.5	4.125	2.375	3.875	6.25	2.125	8.75	1.5	9	1.625	2.25	3.5	9.75	5.625	1.875	1.75	1.875	3.5	3.5	2	2.125	4.375	1.875	2.625	1.625	2	3	3	1.75
466	vsurf_Wp1	Polar volume (8 descriptors)	MOE	626.38	709.5	670.75	761.75	826.75	578.13	771.25	744.88	940.25	681.38	739.88	791.75	672	892.63	1020.1	683.13	687.88	644.63	884.75	865.5	693.25	770.63	757.38	771.88	845.5	617	876.63	775.13	971.38	771.75	810.75	869.13	690	854.25	1125.4	699.88	747.75	748.88	1007.8
467	vsurf_Wp2	Polar volume (8 descriptors)	MOE	272.88	335.13	294	325.88	366	294.75	369.13	326.75	545.5	288.75	301	433.75	286.25	491.38	607.75	326.13	328.5	272.38	422.88	398.63	290	344.25	351	316	326.63	273.38	415.13	353.13	510.38	327.63	352.75	435.13	265.38	410.63	654.38	319	342.13	324.63	454
468	vsurf_Wp3	Polar volume (8 descriptors)	MOE	116.63	129.75	102.75	115.75	120.88	123.38	146.13	128.75	236.75	112.63	112.88	174	97.75	211.75	270.5	140.13	136.25	114.25	157.38	147.75	119.5	130.13	120.63	97.25	105.63	99.75	174.88	129.25	222.75	124.13	141.13	186.75	86.25	166	313.88	130.88	134.88	126.63	177.5
469	vsurf_Wp4	Polar volume (8 descriptors)	MOE	38.375	39.5	27.625	34.25	33.625	36.5	44.875	36.625	71.5	36.125	35.625	46	27.75	63	77	41.5	40.875	36.375	40.375	41.875	36	34.125	29.125	21.125	29	28	54.625	30.5	65.375	32.375	41.375	55.5	22.375	47.625	107.75	39.125	39.625	34.75	50
470	vsurf_Wp5	Polar volume (8 descriptors)	MOE	13.25	11.25	8.125	11.125	11.125	11.125	13.5	10.875	19.875	11.5	14.5	14.625	8.375	20	25	13.375	13.375	12.625	11.625	14.5	12.25	12.375	7.375	7	9	7.625	18.25	7.375	21.375	11.5	14.125	18	8.125	15.75	45.5	12.625	12.625	9.5	16.625
471	vsurf_Wp6	Polar volume (8 descriptors)	MOE	3.875	1.5	1.25	2	1.625	1.25	2	1.125	2.25	2.5	4	2.75	1.25	2.875	6.625	2.5	1.75	2.25	1.25	3.375	3.375	2.125	0.25	1.5	1.25	0.75	3.75	0.375	4.375	1.75	3.625	4.125	1.625	3.25	15.375	2.5	2.5	1.5	4
472	vsurf_Wp7	Polar volume (8 descriptors)	MOE	0.375	0	0	0	0	0	0	0	0	0	0.375	0	0	0	0.875	0	0	0	0	0.25	0.25	0	0	0	0	0	0	0	1	0	0	0.125	0	0.25	4.25	0	0	0	0.25
473	vsurf_Wp8	Polar volume (8 descriptors)	MOE	0	0	0	0	0	0	0	0	0	0	0	0	0	0	0	0	0	0	0	0	0	0	0	0	0	0	0	0	0	0	0	0	0	0	0.5	0	0	0	0

* AAindex: from the amino acid index database, http://www.genome.jp/aaindex/; MOE: calculated for 400 aa combinations by the molecular descriptor calculation of the MOE software Ver.2013.08; all missing values were replaced by the averages.

Table S12 Amino acid descriptors (2D) used in this study.

	C	D	D	D	D	D	D	D	D	D	D	D	D	D	D	D	D	D	D	D	D	E	E	E	E	E	E	E	E	E	E	E	E	E	E	E	E	E	E	E	E	F	F	F	F	F	F	F	F	F	F	F	F	F	F
	Y	A	C	D	E	F	G	H	I	K	L	M	N	P	Q	R	S	T	V	W	Y	A	C	D	E	F	G	H	I	K	L	M	N	P	Q	R	S	T	V	W	Y	A	C	D	E	F	G	H	I	K	L	M	N	P	Q
No.	CY	DA	DC	DD	DE	DF	DG	DH	DI	DK	DL	DM	DN	DP	DQ	DR	DS	DT	DV	DW	DY	EA	EC	ED	EE	EF	EG	EH	EI	EK	EL	EM	EN	EP	EQ	ER	ES	ET	EV	EW	EY	FA	FC	FD	FE	FF	FG	FH	FI	FK	FL	FM	FN	FP	FQ
1	-2	0	-4	-1	-4	-3	2	-3	2	-3	-2	-4	-3	3	-4	1	3	-3	0	-1	-1	6	1	-4	-4	-8	-1	4	1	-4	-3	9	-3	0	-3	-1	5	0	0	-2	-5	-7	-1	-3	-8	-7	-1	-2	-1	0	1	1	-6	-5	-3
2	-0.9	-0.7	-3.2	0.1	-0.4	-2.7	4.3	-3.7	1.4	-4.2	-0.9	-0.4	-2.1	3.2	-4	0.8	4.4	-3	0	-0.5	-0.4	5.1	2.5	-0.4	-0.1	1.6	-1.3	3.6	1	-4.3	-1.5	12.1	-1.5	2.5	-0.4	-0.1	5.2	-0.1	0.7	-2.3	-2.8	-2.8	0.9	-2.7	1.6	-3.2	2.6	-3.1	-1.7	0.1	2.5	0.6	-0.1	-1.4	-1.5
3	-0.5	-0.3	-3.1	-0.4	-1.6	-3.1	3.9	-2.9	1.4	-4	-1.2	-1.6	-1.6	2.4	-3.5	0.8	3.9	-2.7	0.5	-0.7	-0.4	5	2.2	-1.6	-0.7	0.5	-1.2	3.3	1	-3.9	-1	12.6	-1	2.2	-0.1	0.4	4.6	0.4	0.3	-2.5	-2.7	-3.3	0.3	-3.1	0.5	-3.3	0.6	-1.7	-0.9	0.5	2.2	0.6	-0.1	-1.9	-1.1
4	-0.3	-0.1	-2.6	-0.8	-2.2	-3.1	2.9	-2.2	1.2	-3.9	-1.4	-2.2	-1	1.4	-2.6	0.6	2.9	-2.1	0.9	-1	-1	4.8	1.6	-2.2	-1	-0.9	-1	3.6	0.9	-3.2	-0.5	11.8	-0.5	1.7	0.3	0.3	3.7	0.4	-0.3	-3.1	-1	-2.9	0.1	-3.1	-0.9	-2.6	-0.4	-0.2	-0.3	0.7	1.6	0.8	-0.7	-1.8	-0.6
5	-1.6	1.3	-2.4	-1.8	0.7	-1.9	-0.2	-2.1	1.8	-2.1	2.5	0.7	-2.1	3.6	-2.4	1.2	3.8	1.3	1.3	-2.1	0.7	2.9	0.3	0.7	0.8	4.1	0.3	4.7	3.5	-2.5	-2.3	5.5	-2.3	2.2	-2	-1.5	5.7	-2.6	0.8	1.1	-2.1	-3.2	1.5	-1.9	4.1	-3.1	2.6	-2.2	-2.1	0.4	0.3	0.3	1.8	-0.8	-1.9
6	0.14	0.08	-0.25	-0.1	-0.2	0.02	0.47	-0.29	0.32	-0.54	-0.27	-0.2	-0.11	0.05	-0.36	0.11	0.37	-0.44	0.6	-0.02	0.05	1	0.57	-0.2	-0.11	0.18	-0.17	1	0.22	-0.25	-0.01	1	-0.01	0.52	0.34	0.12	1	0.22	0	-0.12	-0.11	-0.43	0.1	0.02	0.18	-0.29	0.21	0.33	-0.01	0.2	0.57	0.19	0.3	-0.28	0.14
7	-1	0	-4	-1	-4	-3	3	-2	2	-2	-2	-4	-1	3	-3	1	3	-2	1	0	0	6	1	-4	-3	-8	0	2	1	-3	-1	12	-1	1	-1	0	4	0	0	-1	-3	-5	0	-3	-8	-5	0	-2	0	1	1	2	-4	-4	-2
8	3	4	2	2	2	2	5	2	4	1	3	2	2	4	2	5	5	4	4	2	3	6	3	2	3	3	3	6	4	0	2	6	2	4	2	2	6	3	3	4	1	0	4	2	3	1	3	2	3	3	3	4	3	2	2
9	2	1	2	2	1	2	1	2	1	2	1	1	2	1	2	1	1	1	1	2	1	0	1	1	1	1	1	0	1	3	2	0	2	1	2	2	0	2	1	1	3	3	1	2	1	2	1	1	2	2	1	2	1	1	2
10	9	5	7	8	4	9	10	3	9	3	6	4	10	9	7	9	10	4	9	9	9	10	6	4	5	6	3	10	6	4	9	10	9	6	9	6	10	10	8	8	5	4	8	9	6	8	8	9	9	10	6	6	8	6	9
11	-0.2	0	-2.3	-0.8	-2.2	-3.1	2.7	-2	1.2	-3.8	-1.4	-2.2	-0.8	1.2	-2.2	0.5	2.7	-1.9	1	-1.1	-1.1	4.7	1.5	-2.2	-1	-1	-0.9	3.8	0.8	-3	-0.3	11.5	-0.3	1.5	0.4	0.3	3.6	0.4	-0.6	-3.3	-0.8	-2.8	0.1	-3.1	-1	-2.4	-0.5	0	-0.2	0.7	1.5	0.9	-0.8	-1.7	-0.5
12	89	107	113	94	180	169	26	96	68	168	143	180	86	24	112	60	45	121	21	59	77	0	43	180	125	215	103	0	94	160	96	0	96	53	54	86	0	80	89	109	115	202	112	169	215	154	194	192	91	46	43	61	205	99	149
13	-1	-1	-2	-1	-3	-4	3	-2	1	-4	-2	-3	-2	1	-2	0	2	-3	0	-2	-2	7	1	-3	-2	-5	-2	6	0	-3	-1	12	-1	1	0	0	6	0	-1	-3	-3	-3	-1	-4	-5	-3	-3	-1	-1	0	1	0	-2	-1	-1
14	-1	-1	-3	-2	-5	-4	3	-4	1	-5	-3	-5	-2	1	-3	0	2	-4	0	-2	-3	8	1	-5	-3	-3	-3	8	0	-5	-2	13	-2	2	0	-1	7	-1	-1	-5	-4	-5	-1	-4	-3	-4	-4	-1	-1	0	1	0	-4	-2	-1
15	-2	-2	-4	-3	-6	-6	3	-4	1	-7	-4	-6	-3	1	-4	0	2	-4	-1	-3	-4	9	1	-6	-4	-5	-3	9	0	-6	-3	13	-3	2	-1	-1	8	-1	-2	-6	-4	-6	-2	-6	-5	-5	-5	-2	-2	0	1	-1	-4	-3	-2
16	-2.6	-0.7	-3.2	-2.2	-5.6	-8.9	3.2	-4.9	1.7	-4.8	-1.3	-5.6	-3.1	1.4	-4	-0.5	2.4	-4.2	0.5	-3.8	-4.3	10	2.1	-5.6	-2.8	-9.1	-3.6	8	0.1	-5.9	-3.4	16.1	-3.4	1.1	-0.2	-1.5	8.6	-1.4	-3.2	-5.6	-4	-8.7	-7.7	-8.9	-9.1	-6.9	-7.7	-4.8	-0.6	-0.8	2.1	-1.1	-4.4	-5.1	-6
17	-1	-1	-3	0	-1	-2	4	-3	1	-3	-1	-1	-2	2	-3	1	4	-2	0	-1	0	5	2	-1	0	1	-1	3	1	-3	-1	11	-1	2	0	0	5	0	1	-2	-3	-3	1	-2	1	-3	2	-2	-1	0	2	1	0	-2	-1
18	-2	0	-2	0	-1	-4	9	-2	3	-3	-1	-1	-3	7	-3	1	8	-2	-1	0	-4	7	6	-1	0	1	-3	11	5	-3	1	6	1	6	2	2	13	-2	0	-1	-1	-3	1	-4	1	-3	3	0	-2	3	6	2	1	0	0
19	9	9	0	0	0	0	9	0	5	0	0	0	0	5	0	0	0	0	0	0	0	10	9	0	0	5	0	10	0	0	0	10	0	9	6.6	0	10	0	0	0	5	0	9	0	5	0	5	0	6.6	0	9	0	5	5	6.6
20	0	1	-1	0	0	0	1	-1	0	-1	-1	0	0	0	-1	0	1	-1	1	0	0	2	0	0	0	-1	0	3	1	-1	0	2	0	0	0	0	2	0	0	-1	-1	0	0	0	-1	0	-1	0	0	1	0	0	-1	-1	0
21	-4	3	-13	1	-6	-28	6	-3	2	-7	-4	-6	-4	2	-6	-5	6	-2	7	-12	-12	32	6	-6	-7	-3	-9	11	-1	-7	-2	109	-2	5	2	2	15	-4	0	-12	-3	-16	-10	-28	-3	-10	3	-7	2	3	6	2	-12	-9	-7
22	-1	7	-24	-1	-10	-30	10	-3	6	-6	0	-10	-2	4	-4	5	12	-4	-4	0	-23	9	6	-10	-2	0	-12	6	10	-13	8	93	8	10	8	5	15	5	-1	-20	-21	-22	-11	-30	0	-5	-7	-12	6	6	6	8	-36	0	1
23	0	-2	-20	-5	2	-1	5	-3	3	-9	-10	2	-5	2	-21	1	6	-6	10	3	-7	18	2	2	2	16	-2	16	-2	-7	0	9	0	-1	-1	2	24	4	3	-5	7	-4	5	-1	16	2	-7	5	-1	2	2	3	4	-12	4
24	-19	4	-18	-2	-1	2	22	-3	-3	-13	-4	-1	-30	13	-15	5	20	-3	-4	4	-14	38	9	-1	0	-45	2	4	6	-11	5	50	5	9	12	5	12	7	2	-10	-41	-2	10	2	-45	0	-8	1	2	4	9	10	-12	0	8
25	-11	-3	-4	-2	-28	-22	9	-4	14	-10	-2	-28	-11	4	-10	9	10	-8	7	-1	-9	32	2	-28	6	-34	-1	18	-1	-7	0	156	0	4	-1	0	25	-4	-3	-14	-36	-32	6	-22	-34	-29	-25	-11	-3	1	2	3	12	-14	-7
26	10	8	-12	-8	-7	-8	22	-11	13	-22	13	-7	2	13	0	14	29	-24	-10	8	-15	60	24	-7	-8	-43	-20	18	16	5	-1	66	-1	22	-1	10	59	6	-7	-9	18	2	-1	-8	-43	7	-9	-28	6	18	24	18	-40	9	-11
27	-3	3	-16	-1	-9	-30	7	-4	2	-9	-2	-9	-3	2	-5	-4	8	-3	5	-10	-12	28	7	-9	-6	-1	-8	10	2	-9	1	108	1	6	3	3	15	-2	-1	-14	-7	-19	-11	-30	-1	-11	0	-8	3	4	7	4	-19	-6	-6
28	0	0	-23	-6	1	-2	9	-6	5	-12	-7	1	-6	4	-19	2	10	-8	3	4	-4	19	5	1	3	-13	2	13	3	-9	3	51	3	4	4	5	23	6	1	-9	-17	-6	7	-2	-13	1	-8	3	1	5	5	6	-4	-8	6
29	3	0	-9	-7	-19	-12	14	-7	13	-17	4	-19	-4	8	-5	12	17	-12	-3	3	-10	40	9	-19	2	-53	-6	15	7	-5	2	97	2	12	2	4	31	0	-7	-12	7	-23	-10	-12	-53	-11	-19	-27	0	7	9	9	-31	-4	-18
30	3	4	1	2	1	0	5	2	4	0	2	1	3	4	1	3	5	2	3	2	3	8	5	1	3	2	3	8	4	2	1	9	1	4	3	3	8	3	3	2	3	0	2	0	2	0	1	1	3	4	5	4	0	1	2
31	0	0	0	0	0	0	3	0	1	0	0	0	0	2	0	3	2	0	1	0	0	6	0	0	0	0	0	5	0	0	0	6	0	1	0	0	5	0	1	0	1	0	2	0	0	0	0	0	0	2	0	1	0	0	0
32	-0.15	0.03	-0.47	-0.45	-0.35	-0.65	0.04	-0.54	0.21	-0.78	0.12	-0.35	-0.21	0.37	-0.34	0.19	0.32	-0.42	0.45	-0.09	0.14	0.65	0.15	-0.35	0.2	0.82	0.14	0.38	0.38	-0.9	-0.06	1.02	-0.06	0.3	-0.02	0.01	0.36	-0.11	-0.59	-0.15	-0.7	-0.81	-0.2	-0.65	0.82	-0.74	0.4	-0.19	-0.16	0.2	0.15	0.16	0.39	-0.02	-0.62
33	1.94	2.46	3.23	2.69	3.06	1.33	0.4	2.43	1.29	3.98	3.42	3.06	2.17	0.32	3.18	0.91	0.9	2.97	0.61	1.7	2.15	0	1.13	3.06	3.58	3.34	2.9	0	1.84	3.69	2.34	0	2.34	1.06	1.45	2.04	0	1.96	2.14	2.76	3.16	3.27	1.84	1.33	3.34	2.48	2.38	0.86	1.92	0.99	1.13	1.47	2.24	2.48	1.45
34	10	10	10	9	8	7	11	5	10	3	8	8	11	11	9	8	11	4	12	10	5	16	10	8	7	11	6	16	11	5	10	16	10	12	9	10	16	10	10	6	10	9	10	7	11	10	10	8	11	11	10	11	10	8	10
35	-0.05	0.7	-0.18	-0.25	-0.17	0.13	0.14	-0.03	0.04	-0.28	0.04	-0.17	-0.18	0.08	-0.59	-0.4	0.07	-0.09	0.27	0.1	-0.32	1	0.05	-0.17	-0.18	0	-0.18	1	-0.19	-0.09	-0.08	1	-0.08	0.16	0.09	-0.1	1	0.37	0.3	-0.24	-0.01	-0.25	0.31	0.13	0	-0.16	0.25	-0.1	0.32	-0.14	0.05	0.11	-0.05	-0.28	0.12
36	-0.02	0.74	-0.19	-0.26	-0.2	0.14	0.21	-0.04	0.04	-0.29	0.05	-0.2	-0.2	0.12	-0.63	-0.41	0.09	-0.09	0.32	0.11	-0.34	1	0.1	-0.2	-0.21	-0.02	-0.21	1	-0.22	-0.08	-0.08	1	-0.08	0.23	0.11	-0.12	1	0.4	0.32	-0.25	-0.02	-0.31	0.35	0.14	-0.02	-0.16	0.29	-0.1	0.36	-0.16	0.1	0.13	-0.07	-0.32	0.15
37	-2	0	-3	-2	-2	-8	2	-3	2	-3	-1	-2	-2	0	-3	0	2	-2	1	-3	-3	7	1	-2	-2	-6	-2	5	0	-4	-2	11	-2	1	0	-1	5	-1	-2	-4	-3	-4	-4	-8	-6	-3	-6	-4	0	0	1	0	-2	-3	-5
38	0.144	0.3	0.628	0.476	0.273	0.355	0.236	0.531	0.299	0.242	0.387	0.273	0.356	0.63	0.683	0.407	0.137	0.444	0.532	0.335	0.304	1	-0.084	0.273	0.394	0.174	-0.037	1	0.208	-0.295	-0.109	1	-0.109	0.323	0.375	0.266	1	0.293	0.385	0.639	0.232	0.054	-0.178	0.355	0.174	0.191	0.195	0.427	0.261	0.082	-0.084	0.321	0.55	0.207	0.087
39	0.568	-0.116	0.874	0.888	0.113	0.558	0.872	0.045	0.305	-0.185	0.338	0.113	0.523	0.614	0.772	0	0.851	-0.186	0.893	0	0.651	1	0.243	0.113	0	0.779	0.133	1	0.49	-0.109	-0.054	1	-0.054	0.377	0.037	0.558	1	0	0.382	0	0.46	0.002	0.104	0.558	0.779	0.418	0.817	0.755	0.55	0.222	0.243	0.257	0.857	0.55	0.326
40	3	0	5	5	0	4	6	0	2	0	2	0	4	4	4	2	4	0	5	1	4	8	0	0	2	5	1	8	2	0	-1	8	-1	1	0	2	8	2	1	2	3	0	1	4	5	3	4	5	2	1	0	2	6	2	1
41	-0.4	1.7	0.6	1.7	-1.5	-1.8	2.1	1.5	-0.3	0	-0.1	-1.5	-0.6	-0.5	0.2	0.6	1	1.6	1	0.2	-1.6	2.2	2	-1.5	0.1	-1.8	-0.3	2.2	1	-0.5	-0.1	2.2	-0.1	1.7	1.9	1.2	2.2	0.2	1.6	0.1	-1.7	-1.6	-1.3	-1.8	-1.8	-1.4	-1.1	-1.4	1.8	1.6	2	0.6	-1.6	0.5	-1.4
42	-3	12	-8	-17	-12	-3	24	-18	8	-39	-13	-12	-10	-4	-18	-3	34	-20	32	-13	-2	100	26	-12	-6	8	-4	100	17	-16	2	100	2	26	11	11	100	10	-5	-14	-6	-14	-6	-3	8	-8	5	8	0	12	26	8	11	-23	-4
43	3	2	-4	-6	-9	0	21	-11	5	-14	-3	-9	-2	6	-2	5	15	-12	6	1	4	16	16	-9	0	-1	2	15	12	-7	13	18	13	22	13	10	11	8	-4	-8	3	-11	-1	0	-1	-10	-1	3	0	13	16	16	2	-3	-5
44	2	1	-2	-3	-7	-2	14	-10	2	-12	-4	-7	-2	2	-1	1	10	-11	7	1	3	11	12	-7	0	0	1	11	10	-6	9	14	9	16	10	7	8	7	-2	-7	-1	-10	-2	-2	0	-9	0	5	1	10	12	13	3	-4	-3
45	78	39	87	91	11	86	78	7	89	23	66	11	71	93	94	96	94	33	96	99	83	100	49	11	17	99	24	100	75	21	81	100	81	71	74	53	100	64	88	90	69	33	91	86	99	62	92	96	64	96	49	68	89	70	95
46	-1	-3	0	0	-1	-1	2	-2	0	-4	0	-1	0	0	0	-1	0	-4	0	0	0	4	0	-1	-1	0	0	4	0	-1	-1	4	-1	0	-1	0	4	0	0	-2	0	-2	0	-1	0	0	0	0	0	1	0	-1	0	1	0
47	0	-2	2	1	2	2	0	1	-1	-1	2	2	2	0	2	-1	-2	-2	0	2	2	-1	-1	2	1	2	2	-2	0	2	1	-5	1	-1	-1	0	-1	0	1	1	2	1	1	2	2	3	2	1	1	1	-1	-1	2	2	1
48	1.03	1.34	0.94	1.08	1.21	1.3	1.11	0.98	1.13	0.93	0.83	1.21	1.17	1.22	0.86	1.11	1.31	1.04	0.74	0.86	0.8	1.06	1.1	1.21	0.99	0.78	0.69	1.02	1.14	0.94	1.18	1.04	1.18	1.21	1.15	1.03	1.19	0.96	-	0.95	0.92	1.31	1.33	1.3	0.78	1.49	1	1.33	1.31	1.2	1.1	1.25	1.16	0.85	1.18
49	0.85	0.63	1.16	1.11	0.84	0.67	0.84	1.08	0.78	1.06	1.08	0.84	0.79	0.79	1.23	0.83	0.58	1.08	1.54	1.03	0.83	0.9	0.83	0.84	0.84	1.47	1.03	0.8	0.72	0.93	0.63	1.07	0.63	0.75	0.82	0.88	0.74	0.71	1.27	1.09	1.18	0.79	0.81	0.67	1.47	0.65	1.22	0.91	0.68	0.71	0.83	0.64	1.04	1.11	0.93
50	1.29	0.96	0.79	0.51	0.82	0.96	1.08	0.86	1.18	1.04	1.27	0.82	1.04	0.92	0.84	1.11	1.17	0.71	0.42	1.3	1.96	1.06	1.13	0.82	1.41	0.47	1.76	1.36	1.3	1.34	1.41	0.71	1.41	1.02	1.02	1.19	1.01	1.8	0.79	0.9	0.78	0.68	0.55	0.96	0.47	0.51	0.48	0.32	0.92	1.13	1.13	1.17	0.46	1.14	0.68
51	13.8	18.1	11.9	17.2	14.4	5.43	21.9	14.4	18.9	13.7	20.1	14.4	11.5	21.2	15.3	19.9	22.8	15.1	18.4	18.2	11.7	29.5	19.6	14.4	16.1	9.56	15.7	27.4	17.7	15.5	18.2	31.9	18.2	22	18.7	17.1	26.5	18.1	17.5	14.5	0	9.83	17.7	5.43	9.56	13.8	17.3	21.4	17.2	18.6	19.6	22.2	13.5	14.6	16.2
52	5	5.2	2.9	4.4	3	2.1	7.9	3.2	6.4	1.4	3.8	3	4.4	6.4	3	5.7	7.9	3.3	6.2	4.1	4.1	9.9	6.7	3	4.2	4.2	4.3	9	6	2.2	4.9	16.7	4.9	6.7	5.6	5.5	8.8	5.6	4.6	1.9	4.4	2.4	5.3	2.1	4.2	2.8	4.7	5.2	5	5.9	6.7	6.1	4.4	3.5	4.7
53	0	0	-1	0	-16	-18	7	-4	5	-9	1	-16	-2	2	-3	1	5	-6	3	-4	-4	14	7	-16	-2	-24	-3	11	2	-7	-2	39	-2	4	3	1	12	1	-3	-5	-9	-23	-18	-18	-24	-14	-1	0	1	1	7	1	-12	-5	-19
54	-0.74	-0.12	-0.76	-0.97	-1.3	-2.19	1.42	-1.52	0.76	-2.77	-0.36	-1.3	-1.42	-0.46	-1.25	0.27	1.2	-1.84	0.76	-0.81	-1.44	2.87	0.13	-1.3	-0.96	-0.76	0.21	3.6	0.83	-2.58	0.13	6.99	0.13	0.92	0.99	0.6	2.97	0.79	-0.96	-1.96	-1.48	-2.19	0.31	-2.19	-0.76	-0.83	0.13	0.34	-0.75	0.33	0.13	0.34	1.13	-0.61	-0.59
55	-2.38	-0.47	-2.42	-1.72	-6.29	-6.7	3.89	-3.8	1.77	-6.18	-0.93	-6.29	-3.01	-2.24	-4.22	0.63	2.41	-4.23	2.3	-2.24	-3.55	8.59	-0.74	-6.29	-3.39	-3.01	1.31	10	1.64	-7.88	-0.33	19.05	-0.33	3.24	2.83	0.24	8.43	1.16	-3.03	-5.32	-5.71	-6.65	1.08	-6.7	-3.01	-2.47	-0.44	1.32	-2.16	1.42	-0.74	1.1	1.72	-1.73	-1.89
56	-0.1	1.2	-1.6	0.3	-1.3	-3.6	3.4	-2.8	1.4	-2.9	-0.8	-1.3	-1.4	1.4	-1.8	1.4	3.2	-1.3	2.2	-0.6	-2.1	5.2	2	-1.3	-0.2	-0.5	-0.6	2.5	1.6	-2.8	0.1	11.5	0.1	2.2	1.9	0.7	3.7	0.7	0	-1.4	-1.2	-3	0.7	-3.6	-0.5	-2.5	-0.3	0.7	-0.1	1.9	2	1.1	-0.8	-1.5	0.3
57	2	0	-1	-2	-5	-1	9	-6	2	-9	-2	-5	-1	1	0	0	7	-9	6	0	2	7	5	-5	0	0	1	8	6	-4	4	14	4	7	8	4	11	5	-3	-5	-1	-7	-3	-1	0	-4	0	4	0	5	5	6	3	-1	-2
58	1	2	-1	1	-3	-1	4	-4	7	-4	-3	-3	-1	0	1	1	6	-3	5	0	2	0	-2	-3	2	1	1	0	1	-1	3	12	3	3	2	2	-2	3	0	-3	2	-1	-5	-1	1	2	0	7	1	2	-2	3	-1	2	2
59	-4	0	0	0	-1	-1	6	-1	4	-7	0	-1	-2	4	0	1	5	-2	4	0	1	4	3	-1	0	2	-1	3	1	-6	0	13	0	4	2	0	3	1	0	-2	0	0	-1	-1	2	-6	4	2	-1	2	3	3	3	1	-2
60	-1	1	0	1	-4	0	3	0	3	-3	0	-4	0	0	0	0	4	-2	4	0	2	0	1	-4	1	2	0	0	0	-2	0	12	0	4	2	0	0	2	0	-1	0	2	-3	0	2	-4	1	5	0	3	1	3	3	0	0
61	-7	-6.7	-7.9	-7.1	-8.3	-8.3	-6.6	-7.4	-6.8	-8.3	-7	-8.3	-7.7	-7.7	-7.5	-5.8	-6.4	-7.3	-7.2	-6.7	-7.5	-6.2	-7.5	-8.3	-7.5	-12	-7.9	-4.8	-6.8	-8.2	-7.3	-3.2	-7.3	-6.7	-7.5	-7.3	-5.4	-6.8	-7.1	-7.3	-9.7	-8.5	-8.2	-8.3	-12	-8.3	-10.6	-8.1	-7	-7.1	-7.5	-7.3	-8.9	-7.3	-7.5
62	-9	-10	-19	-5	-29	-30	31	-32	12	-34	-19	-29	-19	3	-19	4	20	-28	8	-17	-18	56	12	-29	-18	-18	-18	59	1	-30	-18	99	-18	15	3	4	40	7	-8	-30	-18	-29	-10	-30	-18	-30	-18	-6	-3	2	12	2	-18	-9	-7
63	-2	-5	-1	0	-8	-8	-1	-7	4	-5	2	-8	-3	2	-3	1	6	-5	0	-1	-6	9	-2	-8	-5	-4	-7	11	-2	-5	-7	7	-7	-1	-6	-3	12	-1	-1	-2	-3	-10	1	-8	-4	-5	-1	-2	-3	2	-2	0	0	0	-1
64	-2.5	-7	0	0	-5	-9	-0.5	-6	2.5	-6.5	0	-5	-3	-0.5	-3.5	0.5	2	-6.5	1.5	-1.5	-8	10	-2.5	-5	-5.5	-0.5	-8	8	-3.5	-6	-9.5	11	-9.5	-2	-9	-4.5	7	-3	0.5	-2.5	-1	-7	3	-9	-0.5	-3.5	0.5	1	-4	-1	-2.5	-2.5	2.5	-2	1
65	-1	-1	-3	-1	-3	-3	4	-4	1	-6	-2	-3	-2	2	-3	0	3	-3	0	-2	-2	7	2	-3	-3	-7	-2	7	0	-5	-3	13	-3	2	-1	0	6	0	-1	-5	-1	-4	1	-3	-7	-4	-1	1	-1	0	2	1	-4	-4	0
66	0	0	-2	-1	-2	-2	3	-2	1	-4	-1	-2	-1	1	-2	1	3	-2	1	-1	-1	5	2	-2	-1	-4	-1	4	1	-3	-1	11	-1	2	0	0	4	0	0	-3	0	-2	1	-2	-4	-2	0	1	0	1	2	1	-2	-2	0
67	-1	-1	-2	-2	-4	-3	2	-3	0	-6	-2	-4	-2	0	-2	-1	1	-4	0	-2	-2	6	1	-4	-3	-2	-2	7	0	-4	-1	12	-1	1	0	-1	5	-1	-2	-5	0	-4	-2	-3	-2	-3	-2	0	-1	0	1	0	-2	-2	-1
68	0.01	0.519	0.008	0.034	0.032	0.014	0.042	0.015	0.015	0.015	0.014	0.032	0.013	0.036	0.044	0.014	0.001	0.039	0.055	0.036	0.005	0.02	0.041	0.032	0.02	0	0.121	0.029	0.008	0.012	0.064	0.029	0.064	0.036	0.026	0	0.022	0.029	0.016	0.001	0.038	0.006	0	0.014	0	0.055	0.006	0.011	0.069	0.003	0.041	0.009	0.023	0.011	0.06
69	-2.27	0.78	-10.42	-0.47	-6.17	-8.94	0.39	-9.26	-6.44	-2.2	-5.02	-6.17	-2.29	-1.19	-2.36	-2.33	-9.95	-6.99	-4.22	-0.67	-7.28	-7	-6.13	-6.17	-8.02	-5.61	0.5	-5.75	-2.59	-8.93	-4	-7.45	-4	-0.39	-0.16	0.83	-2.26	-7.13	-2.6	1.12	-2.7	-11.35	-11.62	-8.94	-5.61	-2.26	-2.48	-8.48	-5.11	-1.22	-6.13	-2.38	-8.27	-3.13	-2.72
70	1.6	8.1	-4.2	1.2	-1.4	1.2	-0.4	-4.7	1.7	2	2	-1.4	0.7	0.3	-0.2	1.8	-0.6	1.1	3.5	-1.3	-0.6	-1.2	1.6	-1.4	0.5	-1.7	3.3	0.6	0.2	-3	0.9	-0.9	0.9	-0.1	-0.4	3.8	-1.9	1.8	-1.3	1.6	1.1	-1.3	-0.6	1.2	-1.7	5.5	-0.2	-3.1	1.8	0.5	1.6	1.8	-0.4	-0.4	2
71	-0.47	7.03	-3.54	0.13	-1.99	-4.13	0.59	-5.08	-1.64	0.53	-1.24	-1.99	-1.15	-0.19	-0.08	-0.2	-3.62	-1.2	-1.3	1.48	-4.69	-2.01	-0.96	-1.99	-3.98	-1.86	2.62	-1.75	-0.65	-3.48	-0.02	-1.29	-0.02	1.13	-0.49	0.01	-2.07	-2.76	-1.25	-0.79	0.01	-4.75	-3.78	-4.13	-1.86	0.48	-1.23	-3.16	0.01	-2.08	-0.96	-2.19	-0.95	-2.21	-0.26
72	-0.4	0	0	-0.8	-1.8	-0.5	-1	-0.6	0.2	-0.3	-0.5	-1.8	1.3	-2.1	-0.4	0.1	0.2	0.8	0.1	1	-0.3	0.5	0.9	-1.8	0.2	-0.1	-1.5	-0.1	-0.2	-0.2	1.8	-2	1.8	-0.7	2.1	-0.4	0.8	0.5	0.5	-0.5	-0.1	-0.8	0.3	-0.5	-0.1	-3.9	0.3	0.2	-0.4	-0.4	0.9	-0.4	0	0	-1.8
73	0	7	-3	0	-1	-3	-1	-3	-1	1	-1	-1	-1	-1	0	0	-4	-1	-1	-1	-4	-1	0	-1	-4	-3	2	-1	-1	-4	0	-1	0	-1	-1	-1	-3	-3	-2	-3	0	-4	-3	-3	-3	0	-1	-3	1	-3	0	-2	-1	-2	0
74	-4	7	9	8	-12	-12	-1	-15	-5	0	-2	-12	9	-5	10	6	-5	-15	-5	-13	0	-5	2	-12	-8	-9	-1	-9	-12	-5	-12	-7	-12	-2	-3	2	-6	-9	-11	-15	-10	-3	-5	-12	-9	-7	-7	-15	8	-6	2	1	-4	-7	-12
75	12.3	6.7	-10	24.1	31	7.8	-11.6	31.8	13.2	-5.7	25.1	31	-5.7	-5.6	-3.9	-7.1	-2.3	166.7	-2.1	23.8	-3	-9	-8.9	31	-1.5	19.8	8.8	-9.2	36.3	-9.4	-7.1	26.1	-7.1	-10.3	7.4	-7.2	8.7	-7.3	-6.5	-1.7	-10.7	-11.4	-7.3	7.8	19.8	-9	14.8	-3.2	10	8.9	-8.9	9.2	9.1	-7.1	-5.5
76	1.1	5.3	1.7	0.1	0.1	0.1	8.3	1.4	7.5	0.1	0.1	0.1	0.3	4.4	0.5	2	8.6	0.3	0.7	1.8	1	62.7	5.7	0.1	1.2	0.1	1	48.2	3.5	0.1	1.4	73.6	1.4	3.7	1	0.5	56.8	2.3	2.4	0.3	0.1	0.1	0.9	0.1	0.1	0.3	0.1	0.1	8.4	2	5.7	4.5	1	0.1	0.1
77	1.1	5.3	1.7	0.1	0.1	0.1	8.3	1.4	7.5	0.1	0.1	0.1	0.3	4.4	0.5	2	8.6	0.3	0.7	1.8	1	62.7	5.7	0.1	1.2	0.1	1	48.2	3.5	0.1	1.4	73.6	1.4	3.7	1	0.5	56.8	2.3	2.4	0.3	0.1	0.1	0.9	0.1	0.1	0.3	0.1	0.1	8.4	2	5.7	4.5	1	0.1	0.1
78	0.1	3.9	1.6	52.1	8.6	0.5	3.3	1.8	0.1	1.3	0.1	0.1	7.3	3.1	4.5	1.4	2.7	0.7	0.1	0.1	0.1	5.3	0.1	8.6	56.8	0.5	0.1	0.7	0.1	5.8	0.1	5.4	1.5	0.6	4.9	1	2.4	0.2	0.3	0.1	1.1	1.7	1	0.5	0.5	67.6	0.1	0.1	0.7	0.1	1.5	0.4	0.3	0.1	0.8
79	0.1	3.9	1.6	52.1	8.6	0.5	3.3	1.8	0.1	1.3	0.1	0.1	7.3	3.1	4.5	1.4	2.7	0.7	0.1	0.1	0.1	5.3	0.1	8.6	56.8	0.5	0.1	0.7	0.1	5.8	0.1	5.4	1.5	0.6	4.9	1	2.4	0.2	0.3	0.1	1.1	1.7	1	0.5	0.5	67.6	0.1	0.1	0.7	0.1	1.5	0.4	0.3	0.1	0.8
80	0.1	3.9	1.6	52.1	8.6	0.5	3.3	1.8	0.1	1.3	0.1	0.1	7.3	3.1	4.5	1.4	2.7	0.7	0.1	0.1	0.1	5.3	0.1	8.6	56.8	0.5	0.1	0.7	0.1	5.8	0.1	5.4	1.5	0.6	4.9	1	2.4	0.2	0.3	0.1	1.1	1.7	1	0.5	0.5	67.6	0.1	0.1	0.7	0.1	1.5	0.4	0.3	0.1	0.8
81	0.1	3.9	1.6	52.1	8.6	0.5	3.3	1.8	0.1	1.3	0.1	0.1	7.3	3.1	4.5	1.4	2.7	0.7	0.1	0.1	0.1	5.3	0.1	8.6	56.8	0.5	0.1	0.7	0.1	5.8	0.1	5.4	1.5	0.6	4.9	1	2.4	0.2	0.3	0.1	1.1	1.7	1	0.5	0.5	67.6	0.1	0.1	0.7	0.1	1.5	0.4	0.3	0.1	0.8
82	0.1	3.9	1.6	52.1	8.6	0.5	3.3	1.8	0.1	1.3	0.1	0.1	7.3	3.1	4.5	1.4	2.7	0.7	0.1	0.1	0.1	5.3	0.1	8.6	56.8	0.5	0.1	0.7	0.1	5.8	0.1	5.4	1.5	0.6	4.9	1	2.4	0.2	0.3	0.1	1.1	1.7	1	0.5	0.5	67.6	0.1	0.1	0.7	0.1	1.5	0.4	0.3	0.1	0.8
83	0.1	3.9	1.6	52.1	8.6	0.5	3.3	1.8	0.1	1.3	0.1	0.1	7.3	3.1	4.5	1.4	2.7	0.7	0.1	0.1	0.1	5.3	0.1	8.6	56.8	0.5	0.1	0.7	0.1	5.8	0.1	5.4	1.5	0.6	4.9	1	2.4	0.2	0.3	0.1	1.1	1.7	1	0.5	0.5	67.6	0.1	0.1	0.7	0.1	1.5	0.4	0.3	0.1	0.8
84	0.1	3.9	1.6	52.1	8.6	0.5	3.3	1.8	0.1	1.3	0.1	0.1	7.3	3.1	4.5	1.4	2.7	0.7	0.1	0.1	0.1	5.3	0.1	8.6	56.8	0.5	0.1	0.7	0.1	5.8	0.1	5.4	1.5	0.6	4.9	1	2.4	0.2	0.3	0.1	1.1	1.7	1	0.5	0.5	67.6	0.1	0.1	0.7	0.1	1.5	0.4	0.3	0.1	0.8
85	0.1	3.9	1.6	52.1	8.6	0.5	3.3	1.8	0.1	1.3	0.1	0.1	7.3	3.1	4.5	1.4	2.7	0.7	0.1	0.1	0.1	5.3	0.1	8.6	56.8	0.5	0.1	0.7	0.1	5.8	0.1	5.4	1.5	0.6	4.9	1	2.4	0.2	0.3	0.1	1.1	1.7	1	0.5	0.5	67.6	0.1	0.1	0.7	0.1	1.5	0.4	0.3	0.1	0.8
86	0.1	3.9	1.6	52.1	8.6	0.5	3.3	1.8	0.1	1.3	0.1	0.1	7.3	3.1	4.5	1.4	2.7	0.7	0.1	0.1	0.1	5.3	0.1	8.6	56.8	0.5	0.1	0.7	0.1	5.8	0.1	5.4	1.5	0.6	4.9	1	2.4	0.2	0.3	0.1	1.1	1.7	1	0.5	0.5	67.6	0.1	0.1	0.7	0.1	1.5	0.4	0.3	0.1	0.8
87	0.1	3.9	1.6	52.1	8.6	0.5	3.3	1.8	0.1	1.3	0.1	0.1	7.3	3.1	4.5	1.4	2.7	0.7	0.1	0.1	0.1	5.3	0.1	8.6	56.8	0.5	0.1	0.7	0.1	5.8	0.1	5.4	1.5	0.6	4.9	1	2.4	0.2	0.3	0.1	1.1	1.7	1	0.5	0.5	67.6	0.1	0.1	0.7	0.1	1.5	0.4	0.3	0.1	0.8
88	0.1	3.9	1.6	52.1	8.6	0.5	3.3	1.8	0.1	1.3	0.1	0.1	7.3	3.1	4.5	1.4	2.7	0.7	0.1	0.1	0.1	5.3	0.1	8.6	56.8	0.5	0.1	0.7	0.1	5.8	0.1	5.4	1.5	0.6	4.9	1	2.4	0.2	0.3	0.1	1.1	1.7	1	0.5	0.5	67.6	0.1	0.1	0.7	0.1	1.5	0.4	0.3	0.1	0.8
89	0.1	3.9	1.6	52.1	8.6	0.5	3.3	1.8	0.1	1.3	0.1	0.1	7.3	3.1	4.5	1.4	2.7	0.7	0.1	0.1	0.1	5.3	0.1	8.6	56.8	0.5	0.1	0.7	0.1	5.8	0.1	5.4	1.5	0.6	4.9	1	2.4	0.2	0.3	0.1	1.1	1.7	1	0.5	0.5	67.6	0.1	0.1	0.7	0.1	1.5	0.4	0.3	0.1	0.8
90	0.1	3.9	1.6	52.1	8.6	0.5	3.3	1.8	0.1	1.3	0.1	0.1	7.3	3.1	4.5	1.4	2.7	0.7	0.1	0.1	0.1	5.3	0.1	8.6	56.8	0.5	0.1	0.7	0.1	5.8	0.1	5.4	1.5	0.6	4.9	1	2.4	0.2	0.3	0.1	1.1	1.7	1	0.5	0.5	67.6	0.1	0.1	0.7	0.1	1.5	0.4	0.3	0.1	0.8
91	0.027	0.315	0	0.006	0.048	0.009	0.022	0.022	0.076	0.059	0.033	0	0.089	0.016	0.03	0.048	0.028	0.016	0.043	0	0.009	0.305	0.011	0.048	0.026	0.039	0.029	0.005	0.107	0.073	0.016	0.018	0.053	0.039	0.045	0.017	0.005	0.034	0.162	0.009	0	0.415	0.004	0.009	0.039	0.025	0.097	0.042	0.013	0.006	0.011	0.009	0.009	0.011	0.041
92	0.027	0.315	0	0.006	0.048	0.009	0.022	0.022	0.076	0.059	0.033	0	0.089	0.016	0.03	0.048	0.028	0.016	0.043	0	0.009	0.305	0.011	0.048	0.026	0.039	0.029	0.005	0.107	0.073	0.016	0.018	0.053	0.039	0.045	0.017	0.005	0.034	0.162	0.009	0	0.415	0.004	0.009	0.039	0.025	0.097	0.042	0.013	0.006	0.011	0.009	0.009	0.011	0.041
93	0.027	0.315	0	0.006	0.048	0.009	0.022	0.022	0.076	0.059	0.033	0	0.089	0.016	0.03	0.048	0.028	0.016	0.043	0	0.009	0.305	0.011	0.048	0.026	0.039	0.029	0.005	0.107	0.073	0.016	0.018	0.053	0.039	0.045	0.017	0.005	0.034	0.162	0.009	0	0.415	0.004	0.009	0.039	0.025	0.097	0.042	0.013	0.006	0.011	0.009	0.009	0.011	0.041
94	0.027	0.315	0	0.006	0.048	0.009	0.022	0.022	0.076	0.059	0.033	0	0.089	0.016	0.03	0.048	0.028	0.016	0.043	0	0.009	0.305	0.011	0.048	0.026	0.039	0.029	0.005	0.107	0.073	0.016	0.018	0.053	0.039	0.045	0.017	0.005	0.034	0.162	0.009	0	0.415	0.004	0.009	0.039	0.025	0.097	0.042	0.013	0.006	0.011	0.009	0.009	0.011	0.041
101	-6.6	-2.8	-4.2	-2.7	-3.3	-4.9	-3.7	-4.3	-5.4	-3.2	-4.3	-4.3	-3.1	-3.3	-3.2	-3.9	-2.7	-3.1	-4	-5.1	-4.7	-3	-4.4	-3.3	-2.8	-5	-3.8	-4.5	-5.7	-3.8	-4.6	-4.6	-3.4	-3.5	-3.6	-4.2	-3.2	-3.3	-4.2	-5.2	-4.9	-5.1	-6.8	-4.9	-5	-7.1	-5.6	-6.4	-8	-4.9	-7	-6.6	-5	-5.2	-5.3
102	43	36	10	24	38	32	83	32	43	54	60	13	31	23	17	35	37	38	53	24	52	27	9	38	9	22	54	24	35	70	53	12	27	17	19	34	41	28	39	15	40	62	32	32	22	53	67	38	103	32	175	20	27	19	21
103	-0.811	0.926	10.31	6.271	6.052	1.098	0	3.336	3.643	5.256	8.76	14.958	1.921	0.322	2.021	5.172	1.66	1.89	2.1	7.299	8.069	0.902	9.05	6.052	5.832	1.021	0	3.09	3.248	5.139	7.54	12.775	1.835	0.449	1.926	4.911	1.603	1.792	1.944	6.288	6.961	-0.961	-0.873	1.098	1.021	-1.653	0	-0.879	-1.592	1.135	-1.557	-1.217	-0.638	-2.162	-0.609
104	0.069	0.14	-0.137	0.539	-0.178	0.118	-0.336	-0.27	0.229	-0.759	0.316	0.097	-0.534	0.175	-0.128	-0.527	-0.565	-0.433	0.442	1.287	0.694	0.182	0.121	-0.178	1.06	0.27	-0.041	0.072	0.385	-0.782	0.48	0.188	-0.262	-0.064	-0.728	-0.717	-0.207	-0.006	0.255	-0.089	-0.004	0.132	-0.095	0.118	0.27	-0.163	0.457	-0.141	-0.348	0.259	-0.46	-0.501	0.336	-0.083	0.283
105	-0.96	0.1	0.55	0.6	0.68	0.2	-0.06	-0.06	0.04	-0.09	0.5	1.07	-0.12	0.84	0.46	-0.15	-0.09	-0.03	0.25	0.24	0.11	0.03	0.04	0.68	0.21	0.19	0.4	-0.53	-0.2	-0.09	-0.05	0.02	-0.25	0.33	0.62	-0.26	0.3	0.15	-0.02	0.32	0.22	-0.05	-0.98	0.2	0.19	-0.61	0.21	-0.75	-0.66	-0.17	-1.02	-1.03	-0.02	-0.43	0.1
106	0.04	0.12	0.03	0.04	-0.15	0.39	-0.22	-0.39	0.59	-0.76	0.67	0.65	-0.3	0.04	-0.17	-0.72	-0.31	-0.29	0.58	0.24	0	0.26	0.69	-0.15	-0.03	0.27	0.25	-0.45	0.35	-0.97	0.43	0.44	-0.32	-0.1	-0.17	-0.74	-0.26	0	0.34	0.29	-0.1	0.03	-0.23	0.39	0.27	-0.44	-0.38	-0.16	-0.19	0.44	-0.3	-0.42	0.18	0.2	-0.29
107	0.0217	0.0167	0.0159	0.0877	-0.0095	0.0116	-0.0646	-0.0338	0.0181	-0.0931	0.0207	0.0611	0.0758	0.0438	-0.0876	-0.1196	0.0006	-0.0422	0.0684	-0.0061	0.0017	0.094	0.02	-0.0095	0.0865	0.0136	0.086	-0.1188	0.05	-0.1536	0.0173	0.1327	-0.0072	0.0724	-0.0372	-0.1873	-0.1229	0.0788	0.0595	0.1134	-0.0183	0.0388	-0.0452	0.0116	0.0136	-0.1154	-0.1882	-0.094	-0.116	0.0384	-0.0551	-0.0876	0.0029	0.066	-0.1336
108	-0.2444	0.1018	0.1169	0.084	0.1581	0.0827	0.1115	-0.0749	0.1892	-0.1154	0.2673	-0.0197	0.0192	0.2386	-0.0728	-0.1859	0.0424	0.1043	0.2728	-0.0124	-0.1165	0.1346	0.155	0.1581	0.1259	0.0885	0.2311	-0.0827	0.1103	-0.2403	0.0802	0.0637	0.1146	0.2241	-0.0413	-0.3511	0.0823	0.0675	0.101	-0.0967	-0.0522	-0.1119	-0.3262	0.0827	0.0885	-0.3088	0.0789	-0.125	-0.3791	-0.0416	-0.545	-0.1785	0.0018	-0.0604	-0.0171
109	-3.89	-1.57	-2.66	-0.96	-1.23	-3.31	-1.62	-2.14	-2.91	-1.32	-2.59	-2.9	-1.33	-1.19	-1.26	-1.98	-1.46	-1.66	-2.25	-2.91	-2.25	-1.51	-2.08	-1.23	-1.18	-3.51	-1.22	-2.27	-3.23	-1.6	-2.91	-3.19	-1.43	-1.4	-1.33	-2.07	-1.48	-1.45	-2.56	-2.94	-2.42	-4.36	-5.63	-3.31	-3.51	-6.85	-3.72	-4.61	-6.39	-2.83	-6.26	-6.68	-3.55	-3.73	-3.3
110	0.04	0.12	0.03	0.04	-0.16	0.39	-0.23	-0.4	0.59	-0.76	0.67	0.65	-0.3	0.03	-0.18	-0.73	-0.32	-0.29	0.57	0.24	-0.01	0.25	0.68	-0.16	-0.04	0.26	0.24	-0.46	0.34	-0.97	0.42	0.43	-0.33	-0.11	-0.18	-0.75	-0.27	-0.01	0.33	0.28	-0.11	0.03	-0.24	0.39	0.26	-0.45	0.37	-0.17	-0.19	0.43	-0.3	-0.43	0.18	0.19	0.48
111	-4.16	-1.7	-2.41	-1.21	-1.02	-3.48	-1.59	-2.32	-3.17	-1.68	-3.4	-2.57	-1.68	-1.33	-1.46	-2.29	-1.63	-1.8	-2.48	-2.84	-2.76	-1.51	-2.27	-1.02	-0.91	-3.56	-1.22	-2.15	-3.27	-1.8	-3.59	-2.89	-1.51	-1.26	-1.42	-2.27	-1.48	-1.74	-2.67	-2.99	-2.79	-4.81	-5.8	-3.48	-3.56	-7.26	-4.13	-4.77	-6.84	-3.36	-7.28	-6.56	-3.75	-4.25	-4.1
112	0.27	0.16	0.45	-0.08	0.06	0.57	-0.11	-0.47	0.54	-0.87	0.7	0.64	-0.47	0.05	-0.17	-0.89	-0.36	-0.22	0.7	0.26	-0.06	0.3	0.54	0.06	0.12	0.44	0.21	-0.35	0.39	-1.04	0.46	0.27	-0.35	0.07	-0.18	-0.92	-0.26	-0.21	0.46	0.06	-0.14	-0.03	-0.02	0.57	0.44	-0.29	0.27	0	-0.21	0.37	-0.26	-0.43	0.38	0.05	0.11
113	2045	7836	1584	2951	4001	3217	8632	3603	4318	9501	5729	1389	6075	3483	3550	8981	8327	7362	4606	1444	5105	5933	1189	4001	1884	3114	5248	2800	4498	10234	6706	1827	4550	2989	3096	8570	6343	6172	5241	1521	4601	10516	2573	3217	3114	4778	6523	2581	10183	3098	17228	4546	2940	3930	3056
114	-0.39	0.27	0.12	0.29	0.44	0.18	0.11	-0.1	0.22	-0.01	0.27	0.3	0.02	0.33	0.24	-0.24	0.1	0.11	0.36	0.07	-0.07	0.38	0.2	0.44	0.46	0.14	0.32	0	0.17	-0.06	0.17	0.12	0.12	0.37	0.27	-0.22	0.18	0.16	0.26	0	-0.08	-0.36	-0.67	0.18	0.14	-0.88	-0.19	-0.34	-0.73	0.19	-0.8	-0.83	-0.01	-0.19	-0.11
115	0.16	0.1	0.25	-0.09	0.04	0.32	-0.06	-0.27	0.31	-0.5	0.4	0.36	-0.27	0.03	-0.1	-0.51	-0.2	-0.13	0.4	0.15	-0.03	0.19	0.31	0.04	0.04	0.26	0.13	-0.19	0.24	-0.57	0.28	0.16	-0.19	0.05	-0.09	-0.51	-0.14	-0.1	0.28	0.06	-0.06	-0.01	-0.02	0.32	0.26	-0.22	0.16	0.01	-0.12	0.22	-0.15	-0.25	0.22	0.03	0.07
116	-4.08	-2.52	-3.12	-1.85	-1.77	-2.95	-2.34	-2.7	-3.09	-2.22	-2.84	-2.8	-2.21	-2.16	-2.09	-2.67	-2.31	-2.43	-2.82	-3.12	-2.84	-2.45	-2.97	-1.77	-1.6	-2.89	-2.12	-2.49	-3.11	-2.2	-2.85	-2.9	-2.14	-2.09	-1.93	-2.61	-2.22	-2.33	-2.84	-3.04	-2.71	-4.04	-4.94	-2.95	-2.89	-5.22	-3.52	-3.95	-5.25	-2.7	-5.18	-5.03	-3.17	-3.54	-3.25
117	-4.71	-2.74	-3.56	-2.47	-2.35	-3.52	-1.84	-2.93	-3.46	-2.47	-4.16	-3.69	-2.5	-1.24	-3.15	-3.92	-2.34	-2.41	-3.28	-3.81	-3.63	-2.69	-3.02	-2.35	-2.85	-4.1	-1.58	-3.15	-3.99	-2.83	-4.35	-3.76	-2.43	-1.7	-3.45	-4.05	-2.57	-2.34	-3.2	-3.84	-3.62	-4.43	-4.81	-3.52	-4.1	-6.45	-3.76	-3.82	-6.11	-2.7	-6.65	-5.58	-3.89	-3.46	-4.89
118	0.1	0.02	0.06	-0.07	0.17	0.55	0.11	0	0.54	-0.87	0.41	0.09	-0.51	0.36	0.21	-0.87	-0.13	-0.26	0.11	0.11	-0.04	0.2	0.72	0.17	-0.2	0.09	0.5	-0.1	0.13	-1.11	0.34	0.13	-0.3	0.01	0.03	-0.87	-0.25	-0.07	0.3	0.2	0.08	0.01	0.49	0.55	0.09	-0.71	-0.15	0.79	-0.44	0.57	-0.4	-0.14	-0.22	-0.19	0.14
119	0.25	-0.39	-0.49	-0.65	-0.35	-0.01	-0.24	0.06	-0.24	0.16	-0.21	0.02	-0.12	-0.22	-0.01	0.45	0	-0.24	-0.29	0.02	0.25	-0.39	-0.14	-0.35	-0.67	-0.11	-0.37	0.14	-0.28	0.16	-0.29	0.1	-0.01	-0.3	-0.05	0.37	-0.1	-0.23	-0.13	0	0.08	0.13	0.18	-0.01	-0.11	0.33	-0.12	0.34	0.59	-0.05	0.41	0.58	-0.1	0.08	0.16
120	-0.16	0.3	0.38	0.27	0.4	0.48	0.17	-0.22	0.54	-0.69	0.62	0.62	-0.12	0.25	0.12	-0.71	0.01	0	0.66	0.06	-0.07	0.43	0.46	0.4	0.45	0.34	0.48	-0.11	0.38	-0.87	0.37	0.24	-0.01	0.26	0.1	-0.75	0.1	0	0.41	-0.15	-0.16	-0.33	-0.53	0.48	0.34	-0.82	0.11	-0.19	-0.65	0.11	-0.78	-0.89	0.29	-0.19	-0.04
121	-0.65	0.58	-0.32	-0.24	0.18	0.5	-0.55	0.63	0.19	-0.71	0.41	0.08	0.28	0.2	-0.26	-0.4	0.32	0.49	0.31	-0.05	0.04	0.29	0.16	0.18	1.03	-0.12	0.88	-0.17	-0.47	-0.34	-0.05	0.06	0.78	-0.18	0.48	-0.91	0.15	0.81	0.34	-0.41	-0.16	-0.49	-2.52	0.5	-0.12	-2.38	0.9	-1.03	-0.72	0.32	-0.89	-1.37	0.36	1.41	-0.65
122	-0.23	0.62	-0.5	0.89	0.83	0.35	0.37	-0.07	-0.18	-0.55	1.12	0.42	-0.53	0.83	0.78	-0.57	0.29	-0.35	0.2	-1.41	-0.27	-0.02	0.65	0.83	1.59	-0.56	0.48	1.27	0.31	-0.36	0.99	0	-0.19	0.24	0.28	-0.7	1.14	-0.11	0.47	1.13	-0.84	-0.79	-0.96	0.35	-0.56	-1.39	-0.1	0.52	-0.76	-0.01	-1.24	-1.04	0.5	-0.09	-0.1

Table S12 Amino acid descriptors (2D) used in this study.

	C	D	D	D	D	D	D	D	D	D	D	D	D	D	D	D	D	D	D	D	D	E	E	E	E	E	E	E
	Y	A	C	D	E	F	G	H	I	K	L	M	N	P	Q	R	S	T	V	W	Y	A	C	D	E	F	G	H
No.	CY	DA	DC	DD	DE	DF	DG	DH	DI	DK	DL	DM	DN	DP	DQ	DR	DS	DT	DV	DW	DY	EA	EC	ED	EE	EF	EG	EH
123	-0.8	0.3	0	0	0	-0.3	0	-1.1	0	-1	0	0.1	-0.6	0.1	-0.3	-1.4	-0.3	-0.3	0.4	-0.6	-1	0.6	0.1	0	0.1	-0.5	0	-1
124	-3.8	-2.6	-2.9	-2.6	-2	-2.7	0	-3.3	-2.9	-3.5	-2.7	-2.7	-3.3	-2.3	-2.7	-3.7	-2.8	-3.2	-2.8	-3.2	-3.5	-1.9	-2.4	-2	-1.5	-2.4	0	-2.8
125	-0.1	0.1	0.3	0.3	0.3	0.4	-0.4	-1	0.6	-0.6	0.8	1	-0.6	0.6	0	-1.1	-0.9	-0.6	0.7	-0.2	-0.3	0.5	0.8	0.3	0.6	0.1	0.6	-0.9
126	0.1	0.2	0.3	-0.3	-0.3	0.3	0	-0.7	0.5	-0.9	0.6	0.5	-0.6	0	-0.3	-1	-0.5	-0.3	0.6	0	-0.2	0.3	0.4	-0.3	-0.4	0.3	0	-0.6
127	-0.1	1.2	0.8	0.9	0.9	0.5	0.8	-0.1	0.5	0.2	0.7	0.6	0.4	0.9	0.6	-0.5	0.7	0.5	1	0	-0.2	1.2	0.9	0.9	1.2	0.4	1.1	0.1
128	-0.3	1.1	0.5	0.6	0.7	0.4	0.8	-0.2	0.6	-0.2	0.6	0.4	0	0.9	0.2	-0.6	0.3	0.1	0.8	0	-0.1	1.1	0.8	0.7	1	0.2	1.1	-0.1
129	-1	0.9	-0.3	0.2	0.5	0.2	0.7	-0.4	0.5	-0.2	0.5	0	0	0.6	0	-0.6	0.2	0.1	0.6	-0.4	-0.2	1	-0.2	0.5	0.5	0.1	0.9	-0.3
130	-1.2	1	0.6	1.8	2.1	-0.1	0.5	-0.3	0.9	0.9	1	1.1	0.8	1	1.2	-0.2	0.7	0.9	1	-0.2	-0.2	1	0.7	2.1	2.3	-0.1	1	0
131	-1	0.6	0.3	0.6	1.1	-0.2	0.5	-0.6	0.9	0.1	0.8	0.9	0.2	0.8	0.6	-0.8	0.2	0.3	0.8	-0.5	-0.5	0.8	0.8	1.1	1.2	-0.1	0.9	-0.4
132	-1.1	0.1	0.2	-0.2	0.1	-0.3	0.2	-1	0.4	-0.8	0.5	0.4	-0.6	0.5	-0.3	-1.2	-0.5	-0.4	0.4	-0.5	-0.7	0.4	0.6	0.1	0.2	-0.4	0.4	-1
133	6.1	4.9	5.2	5.4	5.5	5.6	4.5	5.2	5.7	5.3	5.5	6.1	5.3	5.2	5.3	5.6	5.2	5.5	5.5	6.6	5.7	5.2	5.7	5.5	6.1	5.8	4.5	5.5
134	6.1	5	5.4	5.5	5.9	5.9	4.7	5.8	5.6	5.6	5.6	5.9	5.2	5.2	5.5	5.6	4.7	5.3	5.6	6	6.2	5	5.1	5.9	6.1	5.9	5.6	5.7
135	5.9	5	5.1	5	5	5.6	6.1	5.5	6	5.8	5.6	5.5	5	5.4	5.5	6	5.4	5.2	5.6	6.3	6.2	4.8	5.7	5	5.7	6	5.3	5.8
136	0.0026	-0.0013	0.0023	-0.0005	0.0022	0.0049	0.0015	-0.0025	0.0027	-0.0006	0.0006	0.0015	0.0079	0.0008	-0.0015	0.002	-0.0016	0.0022	9E-05	-0.0078	0.0002	-0.0024	0.0028	0.0022	0.0065	-0.0011	-0.0001	-0.0072
137	0.1646	0.334	0.5302	0.067	0.2457	0.5378	-0.2244	-0.474	0.4497	-0.9001	0.382	0.3232	-0.4183	-0.2145	-0.0006	-0.7824	-0.5029	-0.3658	0.4466	0.5038	0.4647	0.2647	0.6636	0.2457	0.511	0.2513	0.1492	-0.1067
138	0.002	0.1331	0.1663	-0.213	-0.0542	0.3338	-0.1345	-0.5108	0.6407	-0.8377	0.6711	0.345	-0.3678	-0.108	-0.2211	-0.7576	-0.1746	-0.0437	0.4962	-0.0089	-0.1298	0.1522	0.4664	-0.0542	-0.068	0.2954	-0.0146	-0.3858
139	-0.0139	0.0869	0.0827	-0.1721	-0.1444	0.1457	-0.018	-0.1223	0.1705	-0.2813	0.1488	0.0663	-0.1502	-0.0557	-0.122	-0.3095	9E-05	0.0076	0.1478	0.0226	-0.0585	0.1011	0.1284	-0.1444	-0.1701	0.1183	0.0457	-0.0847
140	0.0344	0.0335	0.0567	-0.0149	-0.007	0.0223	-0.021	0.0135	0.0087	-0.0562	0.0075	0.025	-0.0157	-0.0888	-0.0043	-0.0461	0.0167	-0.0128	0.0341	-0.0321	-0.0087	0.029	0.0323	-0.007	-0.0801	0.0101	0.0338	0.0162
141	-0.0026	0.0041	0.0149	-0.0079	-0.0073	0.0114	-0.0024	-0.0069	0.0145	-0.0164	0.0123	0.0074	-0.0083	-0.0059	-0.0064	-0.016	-0.0031	-0.0017	0.013	0.0034	0.0016	0.0041	0.0183	-0.0073	-0.0095	0.0103	0.0009	-0.0035
142	-2.05	-0.06	-1.04	0.53	1.32	-0.09	-0.67	-0.09	-0.65	0.09	-0.31	0.14	0.09	0.44	0.15	-0.16	0.11	0.08	-0.37	-0.04	-0.22	0.29	-0.38	1.32	1.24	-0.06	0.37	-0.09
143	-2.92	-1.22	-1.34	-0.07	-0.18	-1.43	-1.64	-1.44	-1.28	-1	-1.26	-1.04	-1.17	-0.27	-0.71	-1.51	-1.09	-1	-1.08	-1	-1.5	-0.77	-0.83	-0.18	0.72	-1.22	-1.07	-1.17
144	-0.58	0.65	-0.12	1.15	1.48	0.38	0.75	0.29	0.47	0.58	0.57	0.75	0.61	1.26	0.96	0.33	0.54	0.51	0.59	0.43	0.38	1.32	0.2	1.48	2.18	0.55	1.16	0.74
145	-1.31	0.76	0.32	1.19	2.11	0.17	0.14	0.68	1	1.06	0.13	1.76	0.77	1.31	1.26	0.79	1.04	0.43	0.72	0.8	0.14	1.57	0.87	2.22	2.58	0.26	0.99	1.13
146	-0.87	0.9	0.12	1.45	1.6	0.42	0.47	0.51	0.46	0.98	0.71	0.88	1.22	1.27	1.37	0.6	0.73	0.91	0.67	0.32	0.46	1.44	0.96	2.18	1.93	0.64	1.39	0.89
147	-1.26	0.62	0.51	0.69	1.89	0.15	0.31	0.14	0.26	0.59	0.25	0.92	0.87	0.67	1.37	0.39	0.86	0.87	0.62	0.66	0.2	0.61	0.2	1.34	2.34	0.35	0.98	0.84
148	40.097	25.865	28.765	28.562	31.656	39.092	22.771	34.678	35.145	37.579	35.145	34.952	30.194	30.718	33.287	39.779	26.667	29.76	32.052	44.379	39.894	28.958	31.858	31.656	34.749	42.185	25.865	37.772
149	1	1	1	1	1	1	1	1	1	1	1	1	1	1	1	1	1	1	1	0	1	1	1	1	1	1	1	1
150	0	0	0	0	0	0	0	0	0	0	0	0	0	0	0	0	0	0	0	0	0	0	0	0	0	0	0	0
151	0	0	0	0	0	0	0	0	0	0	0	0	0	0	0	0	0	0	0	1	0	0	0	0	0	0	0	0
152	2	2	2	2	2	2	2	2	2	2	2	2	2	2	2	2	2	2	2	3	2	2	2	2	2	2	2	2
153	2	1	1	1	1	1	1	3	1	1	1	1	2	1	2	1	2	2	1	1	2	1	1	1	1	1	1	3
154	2	4	4	6	6	4	4	4	4	4	4	4	4	4	4	4	4	4	4	4	4	4	4	6	6	4	4	4
155	6	0	0	0	0	6	0	5	0	0	0	0	0	0	0	0	0	0	0	9	6	0	0	0	0	6	0	5
156	1	1	1	1	1	1	1	1	1	2	1	1	1	1	1	4	1	1	1	1	1	1	1	1	1	1	1	1
157	35	25	26	27	30	35	22	32	34	37	34	32	29	29	32	39	26	29	31	39	36	28	29	30	33	38	25	35
158	2	1	1	1	1	1	1	3	1	1	1	1	2	0	2	1	2	2	1	2	2	1	1	1	1	1	1	3
159	4	2	2	2	2	2	2	6	2	2	2	2	4	1	4	2	4	4	2	3	4	2	2	2	2	2	2	6
160	19	14	15	17	18	20	13	19	17	18	17	17	17	16	18	20	15	16	16	23	21	15	16	18	19	21	14	20
161	10	4	4	4	5	10	3	6	7	7	7	7	4	6	5	6	4	5	6	12	10	5	5	5	6	11	4	7
162	62.505	44.782	50.897	49.511	54.006	59.206	40.459	59.065	56.658	62.455	56.658	59.258	53.597	50.637	57.903	68.981	46.996	51.266	52.838	67.163	61.898	48.891	55.166	54.006	58.291	63.022	44.782	63.258
163	1.7859	1.7913	1.9576	1.8337	1.8002	1.6916	1.839	1.8458	1.6664	1.688	1.6664	1.8518	1.8482	1.7461	1.8095	1.7688	1.8076	1.7678	1.7044	1.7221	1.7194	1.7461	1.9023	1.8002	1.7664	1.6585	1.7913	1.8074
164	12	7	7	8	9	13	6	10	10	10	10	9	8	9	9	10	7	8	9	15	13	8	8	9	10	14	7	11
165	16	11	11	10	12	15	9	13	17	19	17	15	12	13	14	19	11	13	15	16	15	13	13	12	14	17	11	15
166	2	2	2	2	2	2	2	4	2	3	2	2	3	2	3	5	2	2	2	3	2	2	2	2	2	2	2	4
167	4	5	5	7	7	5	5	5	5	5	5	5	6	5	6	5	6	6	5	5	6	5	5	7	7	5	5	5
168	1	0	1	0	0	0	0	0	0	0	0	1	0	0	0	0	0	0	0	0	0	0	1	0	0	0	0	0
169	2.4485	3.898	4.0497	4.2546	4.2293	2.4506	3.6439	2.4328	4.3541	4.1412	4.2515	4.1516	4.2546	2.5531	4.2293	4.1287	4.0497	4.2439	4.2439	1.8881	2.4609	3.8384	3.9854	4.1939	4.1846	2.4125	3.606	2.3909
170	-2.5639	-2.5555	-2.5576	-2.562	-2.5981	-2.5675	-2.5253	-2.5592	-2.6839	-2.6624	-2.6768	-2.6014	-2.5578	-2.5581	-2.5935	-2.6314	-2.5553	-2.5922	-2.6495	-2.5634	-2.5674	-2.5913	-2.5921	-2.5942	-2.6147	-2.5969	-2.5766	-2.5928
171	-0.6113	-0.5496	-0.549	-0.5496	-0.5497	-0.5533	-0.5496	-0.5524	-0.5477	-0.5477	-0.5477	-0.5455	-0.549	-0.549	-0.549	-0.5483	-0.5489	-0.5489	-0.5477	-0.5515	-0.5808	-0.5498	-0.5491	-0.5498	-0.5498	-0.552	-0.5498	-0.551
172	0.6504	0.3906	0.4874	0.4366	0.4387	0.5699	0.3965	0.4966	0.3884	0.3903	0.3902	0.3754	0.4347	0.4647	0.44	0.439	0.4909	0.4632	0.3885	0.5757	0.597	0.3908	0.4882	0.437	0.4391	0.5705	0.3967	0.4976
173	2.609	2.6103	2.6134	2.6148	2.6245	2.6155	2.6	2.6168	2.6618	2.656	2.6512	2.625	2.6206	2.7241	2.6301	2.6425	2.6152	2.6307	2.643	2.6161	2.6155	2.6445	2.6463	2.6471	2.6521	2.6474	2.6382	2.6482
174	-3.3144	-3.3086	-3.3087	-3.3086	-3.3086	-3.3086	-3.3084	-3.3086	-3.3087	-3.3086	-3.3086	-3.3086	-3.3086	-3.3066	-3.3086	-3.3086	-3.3087	-3.3088	-3.3087	-3.3086	-3.3086	-3.3082	-3.3083	-3.3082	-3.3082	-3.3083	-3.308	-3.3083
175	-0.6332	-0.4845	-0.6187	-0.5668	-0.5553	-0.5202	-0.4851	-0.5367	-0.4841	-0.4845	-0.4844	-0.4647	-0.5447	-0.5351	-0.5378	-0.5448	-0.6322	-0.5528	-0.4842	-0.5435	-0.5955	-0.462	-0.6017	-0.5427	-0.5336	-0.5127	-0.4669	-0.5292
176	0.6492	0.338	0.4308	0.3836	0.385	0.561	0.3387	0.4622	0.3379	0.3379	0.3379	0.3779	0.3733	0.3742	0.3747	0.385	0.4283	0.4031	0.3379	0.6199	0.521	0.3381	0.431	0.3838	0.3851	0.5635	0.3388	0.4623
177	2.446	2.3849	2.3764	2.3873	2.5357	2.4325	2.3412	2.3975	2.683	2.6641	2.6891	2.5454	2.3933	2.6848	2.5404	2.592	2.3526	2.4399	2.6127	2.4562	2.4472	2.5255	2.5249	2.5256	2.5483	2.5262	2.5231	2.5255
178	-2.3356	-2.3451	-2.3436	-2.3464	-2.3616	-2.348	-2.3347	-2.3472	-2.4318	-2.435	-2.4252	-2.3591	-2.3474	-2.3485	-2.3631	-2.391	-2.3468	-2.365	-2.4002	-2.3473	-2.348	-2.3886	-2.3878	-2.3891	-2.3956	-2.3899	-2.383	-2.3895
179	-0.4627	-0.2648	-0.355	-0.3159	-0.316	-0.4168	-0.2718	-0.3759	-0.2613	-0.2642	-0.2641	-0.1761	-0.319	-0.244	-0.3244	-0.316	-0.3639	-0.3147	-0.2615	-0.4137	-0.4513	-0.2647	-0.355	-0.3159	-0.316	-0.4168	-0.2718	-0.3759
180	0.8019	0.486	0.6178	0.5515	0.552	0.7127	0.4971	0.6409	0.4819	0.4852	0.4851	0.475	0.5744	0.466	0.5866	0.5521	0.6147	0.5639	0.4822	0.7312	0.7494	0.4861	0.6183	0.5518	0.5524	0.7131	0.4973	0.6415
181	2.835	2.8133	2.8312	2.8261	2.8489	2.8292	2.7887	2.827	2.9063	2.8847	2.894	2.8652	2.8316	2.9758	2.8536	2.8732	2.8179	2.8466	2.8798	2.8317	2.8293	2.8448	2.8552	2.852	2.8659	2.8536	2.8309	2.8524
182	21.985	16.155	18.435	16.978	19.164	20.528	13.969	20.322	22.715	23.581	22.715	22.808	17.546	19.662	19.733	24.241	16.155	18.342	20.528	22.281	20.528	18.342	20.622	19.164	21.351	22.715	16.155	22.508
183	6	5	6	7	8	7	5	7	7	9	7	8	7	5	8	9	6	6	6	7	7	6	7	8	9	8	6	8
184	0.3158	0.3846	0.4286	0.4375	0.4706	0.35	0.4167	0.3684	0.4375	0.5294	0.4375	0.5	0.4375	0.3125	0.4706	0.4737	0.4286	0.4	0.4	0.2917	0.3333	0.4286	0.4667	0.4706	0.5	0.381	0.4615	0.4
185	6	0	0	0	0	6	0	5	0	0	0	0	0	0	0	0	0	0	0	10	6	0	0	0	0	6	0	5
186	35	24	25	26	29	35	21	32	33	36	33	31	28	29	31	38	25	28	30	40	36	27	28	29	32	38	24	35
187	2	3	3	4	4	3	3	3	3	3	3	3	4	3	4	4	3	3	3	3	3	3	3	4	4	3	3	3
188	19	13	14	16	17	20	12	19	16	17	16	16	16	16	17	19	14	15	15	24	21	14	15	17	18	21	13	20
189	5	4	5	4	5	4	4	4	6	8	6	7	4	4	5	7	5	5	5	4	4	5	5	5	5	5	5	5
190	7	6	7	8	9	8	6	8	8	10	8	9	8	5	9	11	7	7	7	8	8	7	8	9	10	9	7	9
191	0.3684	0.4615	0.5	0.5	0.5294	0.4	0.5	0.4211	0.5	0.5882	0.5	0.5625	0.5	0.3125	0.5294	0.5789	0.5	0.4667	0.4667	0.3333	0.381	0.5	0.5333	0.5294	0.5556	0.4286	0.5385	0.45
192	27	21	22	22	25	26	18	24	30	33	30	28	24	26	27	34	22	25	27	27	27	24	25	25	28	29	21	27
193	14.414	11.301	12.008	13.585	14.293	15.121	10.431	14.414	13.585	14.129	13.585	13.422	13.585	12.293	14.293	15.707	12.008	12.878	12.878	16.983	15.991	12.008	12.715	14.293	15	15.828	11.138	15.121
194	11.099	7.6102	8.6589	8.6338	9.3409	10.704	6.7399	9.9192	9.8946	10.146	9.8946	10.249	8.8029	8.6787	9.51	10.886	7.7645	8.6347	9.1875	12.204	11.074	8.3173	9.366	9.3409	10.048	11.411	7.447	10.626
195	6.8783	4.3618	4.0689	4.5689	5.276	7.4557	3.4916	5.7236	6.6463	6.1902	6.6463	5.776	4.5689	5.4831	5.276	5.9831	4.0689	4.9392	5.9392	8.4557	7.3783	5.0689	4.776	5.276	5.9831	8.1628	4.1987	6.4307
196	7.7067	4.5939	4.301	4.8783	5.5854	8.4138	3.7236	6.2925	6.8783	6.4223	6.8783	6.0081	4.8783	5.7152	5.5854	6.2925	4.301	5.1712	6.1712	9.5685	8.2841	5.301	5.0081	5.5854	6.2925	9.121	4.4307	6.9996
197	8.9348	6.3411	6.8791	7.735	8.235	9.3968	5.9304	8.8968	7.7898	8.3791	7.735	7.8791	7.735	7.4304	8.235	9.235	6.8791	7.2518	7.2518	10.88	9.7906	6.8411	7.3791	8.235	8.735	9.8968	6.4304	9.3968
198	6.3004	3.884	4.6636	4.4767	4.9767	5.9792	3.4365	5.4163	5.3327	5.7149	5.2779	6.3057	4.5613	4.9743	5.0613	5.8958	4.0312	4.4756	4.7947	6.9732	6.1135	4.384	5.1636	4.9767	5.4767	6.4792	3.9365	5.9163
199	3.5688	1.9165	1.7474	2.101	2.601	4.0116	1.404	2.3896	3.3652	3.2474	3.3103	2.2474	2.101	2.7474	2.601	2.7474	1.7474	2.2498	2.8272	4.467	3.9223	2.4165	2.2474	2.601	3.101	4.5116	1.904	2.8896
200	4.5244	2.0605	1.8914	2.2997	2.7997	5.1162	1.5581	2.7079	3.5092	3.3914	3.4544	2.3914	2.2997	2.8914	2.7997	2.8914	1.8914	2.3938	2.9712	5.6911	4.9327	2.5605	2.3914	2.7997	3.2997	5.6162	2.0581	3.2079
201	2	2	2	2	2	2	1	2	3	2	2	2	2	2	2	2	2	3	2	2	2	2	2	2	2	2	1	2
202	2	2	2	2	2	2	1	2	3	2	2	2	2	2	2	2	2	3	2	2	2	2	2	2	2	2	1	2
203	0.8203	0.8775	0.9303	0.9422	0.9107	0.8059	0.9133	0.8664	0.8046	0.808	0.8046	0.8696	0.9057	0.8549	0.8784	0.8325	0.913	0.8817	0.8246	0.8123	0.8317	0.8485	0.8989	0.9107	0.8842	0.7907	0.8775	0.8451
204	11	8	8	9	10	11	8	10	9	11	9	10	9	8	10	12	8	8	8	12	12	9	9	10	11	12	9	11
205	0	-1	-1	-2	-2	-1	-1	-1	-1	0	-1	-1	-1	-1	-1	0	-1	-1	-1	-1	-1	-1	-1	-2	-2	-1	-1	-1

	E	E	E	E	E	E	E	E	E	E	E	E	E	F	F	F	F	F	F	F	F	F	F	F	F	F	F
	I	K	L	M	N	P	Q	R	S	T	V	W	Y	A	C	D	E	F	G	H	I	K	L	M	N	P	Q
No.	EI	EK	EL	EM	EN	EP	EQ	ER	ES	ET	EV	EW	EY	FA	FC	FD	FE	FF	FG	FH	FI	FK	FL	FM	FN	FP	FQ
123	-0.2	-1.1	-0.1	-0.3	-0.6	0.1	-0.4	-1.5	-0.2	-0.3	0	-0.8	-1	-0.8	-1.2	-0.3	-0.5	-2	0	-1.2	-1.7	-0.4	-1.6	-1.9	-0.6	-0.7	-0.8
124	-2.7	-3.2	-2.4	-2.7	-2.8	-1.8	-2.2	-3.2	-2.3	-2.7	-2.8	-2.8	-3	-3.7	-4.1	-2.7	-2.4	-4.3	0	-3.3	-4.5	-2.8	-4.2	-4.6	-3.2	-3.1	-3
125	0.4	-0.7	0.7	0	-0.4	0.6	0.2	-0.9	-0.2	-0.2	0.4	0.1	0	-0.6	-1.5	0.4	0.1	-1.5	0.1	-1	-0.8	0.3	-0.9	-1.1	0.2	-0.2	0
126	0.5	-0.8	0.5	0.3	-0.5	-0.1	-0.3	-1	-0.5	-0.2	0.4	0	-0.3	-0.1	0.1	0.3	0.3	-0.2	0	0.1	0	0.1	-0.1	-0.1	0.1	-0.1	0.1
127	0.4	0	0.6	0.4	0.5	0.7	0.8	-0.4	0.6	0.4	0.5	-0.1	-0.2	-0.2	-0.6	0.5	0.4	-1.5	0.3	-0.4	-1.3	0.4	-1.3	-1.3	0.1	-0.1	-0.1
128	0.3	-0.4	0.4	0.4	0.3	0.5	0.2	-0.5	0.4	0.2	0.5	-0.2	-0.2	-0.2	-0.8	0.4	0.2	-1.2	0.4	-0.3	-1.1	0.3	-1.1	-1.1	0	-0.2	-0.1
129	0.3	-0.4	0.4	0	0.2	0.4	0.1	-0.5	0.3	0.1	0.4	-0.5	-0.2	-0.2	-1.1	0.2	0.1	-1.3	0.3	-0.6	-1.1	0.1	-1.1	-1.2	-0.2	-0.3	-0.3
130	0.7	1.4	0.7	1.1	1.2	1	1.6	-0.1	0.9	1	0.8	-0.2	-0.1	-0.3	-1.4	-0.1	-0.1	-1.6	0.3	-0.8	-1.4	-0.2	-1.4	-1.4	-0.4	-0.7	-0.4
131	0.8	0.2	0.8	0.9	0.6	0.7	0.7	-0.7	0.5	0.5	0.9	-0.3	-0.4	0	-1.2	-0.2	-0.1	-1.4	0.2	-0.6	-1.1	-0.1	-1.2	-1.2	-0.4	-0.6	-0.3
132	0.2	-1	0.2	0.3	-0.2	0.4	-0.2	-1.4	-0.2	-0.4	0.2	-0.6	-0.7	-0.2	-1.5	-0.3	-0.4	-1.5	0.1	-0.9	-1.4	-0.2	-1.4	-1.4	-0.6	-0.6	-0.6
133	5.6	5.3	5.8	5.9	5.3	5.5	5.4	5.7	5.1	5.4	5.3	6.3	5.8	5.1	5.4	5.6	5.8	6.2	5.4	5.5	6	5.5	5.8	5.8	5.8	5.5	5.7
134	5.9	5.8	5.5	5.7	5.6	5.3	5.7	5.9	5.2	5.2	5.4	6.2	6.1	5.5	5.8	5.9	5.9	6.3	6	6	6.2	5.5	6.2	6.2	6.1	5.8	6.1
135	5.9	6.3	5.7	6.2	5	5.6	6.5	6.1	4.9	5.3	5.5	6.8	6.5	5.8	5.9	5.6	6	6.4	5.3	6.1	6.4	5.8	6.3	6	6.1	5.6	6.1
136	0.0076	-0.0096	-0.0005	0.0032	-0.0032	0.0054	-0.0052	-0.0046	0.0029	0.0009	0.0014	-0.0094	0.0033	0.0051	-0.0139	0.0049	-0.0011	-0.0131	0.0002	-0.0002	-0.0098	-0.0045	-0.0021	0.001	0.0035	-0.007	0.0038
137	0.2195	-0.9236	0.1989	0.1516	-0.1801	-0.1419	-0.0584	-0.7861	-0.3531	-0.2914	0.2276	0.1607	0.1888	0.0814	0.0964	0.5378	0.2513	-0.2465	0.2129	0.0392	-0.1911	0.0483	-0.2268	-0.2336	0.2661	-0.1048	0.1434
138	0.517	-0.849	0.4759	0.2154	-0.2905	-0.0851	-0.2264	-0.7311	-0.2114	-0.1265	0.4169	-0.098	-0.0382	-0.1491	-0.0887	0.3338	0.2954	-0.1993	0.0527	0.0943	-0.0925	0.3116	-0.1269	-0.12	0.2704	0.1612	0.1169
139	0.1435	-0.3221	0.1492	0.0714	-0.0928	-0.1063	-0.1063	-0.2898	0.007	-0.0213	0.119	-0.0492	-0.0819	0.0258	-0.0552	0.1457	0.1183	-0.1014	0.0702	0.037	-0.0888	0.1406	-0.1181	-0.114	0.0974	0.1237	0.0641
140	0.0302	-0.0928	-0.0286	0.0085	-0.0179	-0.0533	-0.0266	-0.0543	0.0472	-0.0025	0.0478	-0.0116	0.0011	-0.0232	-0.0625	0.0223	0.0101	0.0322	0.0028	-0.0011	-0.025	0.0152	-0.0042	0.0074	0.0071	0.0582	0.0194
141	0.0141	-0.0179	0.0104	0.0062	-0.0068	-0.0059	-0.0073	-0.0152	-0.0023	-0.0022	0.0125	0.002	0.0022	-0.0021	-0.0149	0.0114	0.0103	-0.0127	0.0038	0.0022	-0.0138	0.0105	-0.013	-0.0098	0.0084	0.0081	0.007
142	-0.52	0.09	-0.22	0.27	0.52	0.87	0.47	-0.11	0.5	0.31	-0.23	-0.43	-0.12	-1.7	-2.87	-0.09	-0.06	-2.45	-1.63	-1.21	-2.46	-0.21	-2.58	-2.25	-0.43	-0.42	-0.48
143	-1.48	-1.06	-1.23	-0.64	-0.67	0.38	-0.47	-1.24	-0.85	-1.17	-1.07	-1.21	-1.27	-2.99	-3.61	-1.43	-1.22	-3.1	-3.01	-1.83	-3.5	-0.92	-3.5	-3.13	-1.41	-1.75	-1.59
144	0.7	0.93	0.82	1.08	1.09	1.25	1.11	0.54	0.87	0.81	0.74	0.44	0.47	-0.41	-1.44	0.38	0.55	-1.09	0.09	-0.28	-1.09	0.74	-1.05	-0.6	0.46	-0.04	0.39
145	0.88	1.65	0.73	0.72	3.06	1.91	2.56	0.72	0.94	1.36	0.81	0.11	0.71	-1.49	-2.67	0.49	0.12	-2.68	-1.41	-1.01	-2.46	-0.21	-2.28	-1.87	0.63	0.15	-0.26
146	0.8	1.04	0.82	1.92	1.45	1.71	1.84	1.12	1.23	1.25	0.63	0.58	0.59	-0.67	-1.47	0.33	0.55	-1.77	-0.36	-0.41	-1.56	0.27	-1.55	-1.6	0.03	0.27	0.2
147	0.46	0.6	0.68	0.71	1	0.94	1.27	0.76	0.67	1.2	0.73	0.5	0.1	-1.55	-1.7	-0.38	0.58	-1.55	-1.38	-0.49	-1.82	-0.03	-1.76	-1.35	-0.17	-0.87	-0.3
148	38.239	40.673	38.239	38.045	33.287	33.812	36.381	42.873	29.76	32.854	35.145	47.472	42.987	36.395	39.295	39.092	42.185	49.622	33.301	45.208	45.675	48.109	45.675	45.482	40.723	41.248	43.817
149	1	1	1	1	1	1	1	0	1	1	1	0	0	1	1	1	1	0	1	0	1	1	1	1	1	1	1
150	0	0	0	0	0	0	0	0	0	0	0	0	0	0	0	0	0	0	0	0	0	0	0	0	0	0	0
151	0	0	0	0	0	0	0	1	0	0	0	1	1	0	0	0	0	1	0	1	0	0	0	0	0	0	0
152	2	2	2	2	2	2	2	3	2	2	2	3	3	2	2	2	2	3	2	3	2	2	2	2	2	1	2
153	1	1	1	1	2	1	2	1	2	2	1	1	2	1	1	1	1	1	1	3	1	1	1	1	2	1	2
154	4	4	4	4	4	4	4	4	4	4	4	4	4	2	2	4	4	2	2	2	2	2	2	2	2	2	2
155	0	0	0	0	0	0	0	0	0	0	0	9	6	6	6	6	6	12	6	11	6	6	6	6	6	6	6
156	1	2	1	1	1	1	1	4	1	1	1	1	1	1	1	1	1	1	1	1	1	2	1	1	1	1	1
157	37	40	37	35	32	32	35	42	29	32	34	42	39	33	34	35	38	43	30	40	42	45	42	40	37	37	40
158	1	1	1	1	2	0	2	1	2	2	1	2	2	1	1	1	1	1	1	3	1	1	1	1	2	0	2
159	2	2	2	2	4	1	4	2	4	4	2	3	4	2	2	2	2	2	2	6	2	2	2	2	4	1	4
160	18	19	18	18	18	17	19	21	16	17	17	24	22	17	18	20	21	23	16	22	20	21	20	20	20	19	21
161	8	8	8	8	5	7	6	7	5	6	7	13	11	10	10	10	11	16	9	12	13	13	13	13	10	12	11
162	60.376	66.22	60.376	63.209	57.903	54.568	62.041	72.966	51.266	55.358	56.658	71.054	65.831	52.691	59.199	59.206	63.022	65.077	49.095	66.46	63.012	68.981	63.012	66.381	62.402	57.633	66.109
163	1.6318	1.6555	1.6318	1.806	1.8095	1.7052	1.7726	1.7373	1.7678	1.7299	1.6664	1.6918	1.688	1.5967	1.7412	1.6916	1.6585	1.5134	1.6365	1.6615	1.5003	1.5329	1.5003	1.6595	1.6865	1.5577	1.6527
164	11	11	11	10	9	10	10	11	8	9	10	16	14	12	12	13	14	18	11	15	15	15	15	14	13	14	14
165	19	21	19	17	14	15	16	21	13	15	17	18	17	16	16	15	17	20	14	18	22	24	22	20	17	18	19
166	2	3	2	2	3	2	3	5	2	2	2	3	2	2	2	2	2	2	2	4	2	3	2	2	3	2	3
167	5	5	5	5	6	5	6	5	6	6	5	5	6	3	3	5	5	3	3	3	3	3	3	3	4	3	4
168	0	0	0	1	0	0	0	0	0	0	0	0	0	0	1	0	0	0	0	0	0	0	0	1	0	0	0
169	4.2809	4.1069	4.1913	4.1041	4.1939	2.4761	4.1846	4.1128	3.9854	4.1692	4.1692	1.8528	2.4265	2.2831	2.3324	2.3977	2.3797	1.7753	2.1968	1.769	2.439	2.3431	2.3967	2.3557	2.3977	1.8536	2.3797
170	-2.6866	-2.6655	-2.6794	-2.6167	-2.592	-2.5952	-2.6116	-2.6387	-2.5908	-2.6106	-2.6553	-2.5949	-2.5968	-2.5613	-2.5631	-2.567	-2.6003	-2.5719	-2.5349	-2.5645	-2.6842	-2.6628	-2.6771	-2.6034	-2.5632	-2.5643	-2.5959
171	-0.5477	-0.5477	-0.5477	-0.5448	-0.5491	-0.5491	-0.5491	-0.5484	-0.5491	-0.5491	-0.5477	-0.5511	-0.5809	-0.5539	-0.5477	-0.5515	-0.5497	-0.5635	-0.5818	-0.5627	-0.5294	-0.5482	-0.5406	-0.5477	-0.5477	-0.5477	-0.5477
172	0.3885	0.3904	0.3903	0.3754	0.4348	0.4664	0.4401	0.4395	0.4918	0.4636	0.3886	0.5764	0.5955	0.5611	0.581	0.5643	0.5666	0.5852	0.6192	0.5812	0.5227	0.5528	0.5481	0.54	0.569	0.5807	0.5423
173	2.6742	2.6681	2.6662	2.6524	2.6503	2.7306	2.655	2.661	2.6474	2.6559	2.6625	2.6477	2.6474	2.6133	2.6161	2.6175	2.6266	2.6181	2.6036	2.6194	2.6624	2.6566	2.652	2.6271	2.6229	2.7242	2.6318
174	-3.3083	-3.3083	-3.3083	-3.3083	-3.3082	-3.3062	-3.3082	-3.3082	-3.3084	-3.3084	-3.3083	-3.3083	-3.3083	-3.3115	-3.3116	-3.3115	-3.3115	-3.3116	-3.3113	-3.3116	-3.3116	-3.3116	-3.3115	-3.3116	-3.3115	-3.3095	-3.3115
175	-0.4605	-0.4617	-0.4614	-0.454	-0.5364	-0.5127	-0.5301	-0.5264	-0.6095	-0.5431	-0.4607	-0.5276	-0.5879	-0.4878	-0.5381	-0.5139	-0.5129	-0.5381	-0.4917	-0.5381	-0.4862	-0.4875	-0.4868	-0.4623	-0.5381	-0.5381	-0.5381
176	0.338	0.3381	0.3381	0.3779	0.3733	0.3749	0.3747	0.3852	0.4284	0.4031	0.338	0.6223	0.5224	0.5478	0.6763	0.5726	0.564	0.6786	0.6181	0.6763	0.5352	0.5451	0.5447	0.48	0.4857	0.5928	0.4797
177	2.6834	2.6646	2.6894	2.5535	2.5258	2.6872	2.5513	2.5944	2.5241	2.5265	2.6144	2.5267	2.5264	2.4245	2.422	2.425	2.536	2.4414	2.4152	2.4266	2.683	2.6641	2.6891	2.5456	2.4264	2.6847	2.5407
178	-2.4382	-2.4387	-2.4317	-2.3945	-2.3896	-2.3917	-2.3961	-2.4083	-2.3894	-2.3971	-2.4154	-2.3896	-2.3899	-2.352	-2.3507	-2.3531	-2.3664	-2.3546	-2.3426	-2.3538	-2.4325	-2.4354	-2.4259	-2.3642	-2.354	-2.3554	-2.3677
179	-0.2613	-0.2642	-0.2641	-0.1761	-0.319	-0.2441	-0.3244	-0.316	-0.3639	-0.3147	-0.2615	-0.4138	-0.4514	-0.4062	-0.4038	-0.4119	-0.4121	-0.4354	-0.4265	-0.4354	-0.3723	-0.4006	-0.3964	-0.3891	-0.3896	-0.3932	-0.3892
180	0.4821	0.4854	0.4852	0.4751	0.5746	0.4669	0.5867	0.5525	0.6151	0.5641	0.4823	0.7314	0.75	0.7087	0.7182	0.7075	0.7081	0.7331	0.7594	0.7213	0.6556	0.6978	0.6887	0.6906	0.7097	0.7176	0.6945
181	2.9113	2.8914	2.8998	2.8769	2.8552	2.9791	2.8689	2.8826	2.8474	2.8647	2.889	2.855	2.8537	2.8182	2.8345	2.8297	2.8509	2.8325	2.7964	2.8305	2.9067	2.8853	2.8945	2.8665	2.8348	2.976	2.8553
182	24.901	25.767	24.901	24.995	19.733	21.848	21.919	26.427	18.342	20.528	22.715	24.468	22.715	19.705	21.985	20.528	22.715	24.078	17.519	23.872	26.265	27.131	26.265	26.358	21.097	23.212	23.283
183	8	10	8	9	8	6	9	10	7	7	7	8	8	5	6	7	8	7	5	7	7	9	7	8	7	5	8
184	0.4706	0.5556	0.4706	0.5294	0.4706	0.3529	0.5	0.5	0.4667	0.4375	0.4375	0.32	0.3636	0.2941	0.3333	0.35	0.381	0.2917	0.3125	0.3043	0.35	0.4286	0.35	0.4	0.35	0.25	0.381
185	0	0	0	0	0	0	0	0	0	0	0	10	6	6	6	6	6	12	6	11	6	6	6	6	6	6	6
186	36	39	36	34	31	32	34	41	28	31	33	43	39	33	34	35	38	44	30	41	42	45	42	40	37	38	40
187	3	3	3	3	4	3	4	4	3	3	3	3	3	2	2	3	3	2	2	2	2	2	2	2	3	2	3
188	17	18	17	17	17	17	18	20	15	16	16	25	22	17	18	20	21	24	16	23	20	21	20	20	20	20	21
189	6	8	6	7	5	5	5	7	5	5	5	5	5	4	5	4	5	4	4	4	6	8	6	7	4	4	5
190	9	11	9	10	9	6	10	12	8	8	8	9	9	6	7	8	9	8	6	8	8	10	8	9	8	5	9
191	0.5294	0.6111	0.5294	0.5882	0.5294	0.3529	0.5556	0.6	0.5333	0.5	0.5	0.36	0.4091	0.3529	0.3889	0.4	0.4286	0.3333	0.375	0.3478	0.4	0.4762	0.4	0.45	0.4	0.25	0.4286
192	33	36	33	31	27	29	30	37	25	28	30	30	30	25	26	26	29	30	22	28	34	37	34	32	28	30	31
193	14.293	14.836	14.293	14.129	14.293	13	15	16.414	12.715	13.585	13.585	17.69	16.698	12.836	13.544	15.121	15.828	16.656	11.966	15.949	15.121	15.665	15.121	14.958	15.121	13.828	15.828
194	10.602	10.853	10.602	10.956	9.51	9.3858	10.217	11.593	8.4716	9.3418	9.8946	12.911	11.781	9.6804	10.729	10.704	11.411	12.774	8.8102	11.989	11.965	12.216	11.965	12.319	10.873	10.749	11.58
195	7.3534	6.8973	7.3534	6.4831	5.276	6.1902	5.9831	6.6902	4.776	5.6463	6.6463	9.1628	8.0854	7.2486	6.9557	7.4557	8.1628	10.342	6.3783	8.6104	9.533	9.077	9.533	8.6628	7.4557	8.3699	8.1628
196	7.5854	7.1294	7.5854	6.7152	5.5854	6.4223	6.2925	6.9996	5.0081	5.8783	6.8783	10.276	8.9912	8.1294	7.8365	8.4138	9.121	11.949	7.2591	9.8281	10.414	9.9578	10.414	9.5436	8.4138	9.2507	9.121
197	8.2898	8.8791	8.235	8.3791	8.235	7.9304	8.735	9.735	7.3791	7.7518	7.7518	11.38	10.291	8.0029	8.5409	9.3968	9.8968	11.059	7.5922	10.559	9.4516	10.041	9.3968	9.5409	9.3968	9.0922	9.8968
198	5.8327	6.2149	5.7779	6.8057	5.0613	5.4743	5.5613	6.3958	4.5312	4.9756	5.2947	7.4732	6.6135	5.3865	6.1661	5.9792	6.4792	7.4816	4.9389	6.9188	6.8352	7.2174	6.7803	7.8081	6.0637	6.4767	6.5637
199	3.8652	3.7474	3.8103	2.7474	2.601	3.2474	3.101	3.2474	2.2474	2.7498	3.3272	4.967	4.4223	3.8272	3.6581	4.0116	4.5116	5.9223	3.3147	4.3003	5.2759	5.1581	5.221	4.1581	4.0116	4.6581	4.5116
200	4.0092	3.8914	3.9544	2.8914	2.7997	3.3914	3.2997	3.3914	2.3914	2.8938	3.4712	6.1911	5.4327	4.877	4.7079	5.1162	5.6162	7.9327	4.3746	5.5244	6.3257	6.2079	6.2709	5.2079	5.1162	5.7079	5.6162
201	3	2	2	2	2	2	2	2	2	3	2	2	2	2	2	2	2	2	1	2	3	2	2	2	2	2	2
202	3	2	2	2	2	2	2	2	2	3	2	2	2	2	2	2	2	2	1	2	3	2	2	2	2	2	2
203	0.7875	0.7915	0.7875	0.8475	0.8784	0.8315	0.8552	0.8155	0.8817	0.8557	0.8046	0.7983	0.8151	0.7459	0.7937	0.8059	0.7907	0.7235	0.7602	0.7635	0.7136	0.7204	0.7136	0.7639	0.7822	0.7423	0.7689
204	10	12	10	11	10	9	11	13	9	9	9	13	13	10	10	11	12	13	10	12	11	13	11	12	11	10	12
205	-1	0	-1	-1	-1	-1	-1	0	-1	-1	-1	-1	-1	0	0	-1	-1	0	0	0	0	1	0	0	0	0	0

Table S12 Amino acid descriptors (2D) used in this study.

	C	D	D	D	D	D	D	D	D	D	D	D	D	D	D	D	D	D	D	D	D	E	E	E	E	E	E	E
	Y	A	C	D	E	F	G	H	I	K	L	M	N	P	Q	R	S	T	V	W	Y	A	C	D	E	F	G	H
No.	CY	DA	DC	DD	DE	DF	DG	DH	DI	DK	DL	DM	DN	DP	DQ	DR	DS	DT	DV	DW	DY	EA	EC	ED	EE	EF	EG	EH
206	-0.8463	-0.8513	-0.8513	-0.852	-0.8543	-0.8513	-0.8513	-0.8513	-0.8513	-0.863	-0.8513	-0.8513	-0.8513	-0.8518	-0.8513	-0.8513	-0.8513	-0.8513	-0.8513	-0.863	-0.8513	-0.8526	-0.8525	-0.853	-0.8543	-0.8526	-0.8526	-0.8526
207	-0.438	-0.5289	-0.5184	-0.6186	-0.5758	-0.4804	-0.55	-0.4844	-0.4828	-0.4132	-0.4423	-0.4736	-0.5261	-0.5014	-0.5145	-0.457	-0.5301	-0.5148	-0.4989	-0.4881	-0.53	-0.5166	-0.5071	-0.5742	-0.5436	-0.4734	-0.5238	-0.4773
208	0.1806	0.1175	0.1628	0.1205	0.1054	0.1047	0.1313	0.1595	0.1125	0.1849	0.0798	0.0994	0.1072	0.1337	0.102	0.14	0.1629	0.1493	0.1147	0.14	0.1401	0.0991	0.1567	0.1089	0.099	0.0925	0.1221	0.1532
209	2.4687	2.3013	2.32	2.3367	2.3988	2.4727	2.2229	2.4284	2.5204	2.5325	2.5009	2.4177	2.3769	2.5272	2.4331	2.5224	2.322	2.3957	2.454	2.5586	2.4836	2.3837	2.3994	2.4135	2.4657	2.5281	2.3181	2.4903
210	-2.2351	-2.2351	-2.2351	-2.2351	-2.2351	-2.2351	-2.2351	-2.2351	-2.2351	-2.2354	-2.2351	-2.2351	-2.2351	-2.2351	-2.2351	-2.2352	-2.2351	-2.2351	-2.2351	-2.2351	-2.2351	-2.2351	-2.2351	-2.2351	-2.2351	-2.2351	-2.2351	-2.2351
211	-0.4337	-0.5402	-0.5204	-0.6046	-0.5561	-0.4561	-0.5643	-0.5085	-0.4282	-0.3968	-0.4142	-0.4678	-0.5487	-0.4541	-0.5134	-0.4775	-0.6039	-0.5345	-0.4778	-0.4278	-0.4512	-0.4688	-0.4648	-0.5453	-0.5021	-0.4449	-0.5069	-0.453
212	0.129	0.0542	0.0567	0.0551	0.0555	0.0865	0.0542	0.0569	0.0542	0.0542	0.0542	0.0567	0.0542	0.0542	0.0542	0.0555	0.0542	0.0542	0.0542	0.1082	0.0723	0.0542	0.0568	0.0551	0.0555	0.0872	0.0542	0.0569
213	2.4252	2.1032	2.1251	2.1334	2.2478	2.4014	1.9877	2.2795	2.4443	2.4309	2.4311	2.2999	2.1689	2.4149	2.2799	2.3762	2.0907	2.2007	2.3395	2.5512	2.4301	2.2328	2.2479	2.2548	2.3371	2.4562	2.1545	2.36
214	-0.6407	-0.6408	-0.6408	-0.6408	-0.6408	-0.6408	-0.6408	-0.6408	-0.6408	-0.6418	-0.6408	-0.6408	-0.6408	-0.6409	-0.6408	-0.6408	-0.6408	-0.6408	-0.6408	-0.6408	-0.6408	-0.6409	-0.6409	-0.6409	-0.6409	-0.6409	-0.6409	-0.6409
215	-0.2121	-0.2007	-0.1872	-0.2112	-0.2103	-0.2114	-0.2104	-0.2139	-0.1858	-0.1963	-0.1932	-0.1854	-0.2222	-0.2072	-0.2224	-0.2104	-0.2235	-0.2106	-0.1879	-0.2156	-0.2243	-0.2001	-0.1868	-0.2104	-0.2096	-0.2112	-0.2093	-0.2137
216	0.1838	0.1126	0.1457	0.1282	0.1282	0.1627	0.1243	0.1464	0.1027	0.1111	0.1109	0.1449	0.1441	0.0858	0.1449	0.1282	0.1445	0.1278	0.1034	0.1664	0.174	0.1127	0.1457	0.1282	0.1282	0.1627	0.1244	0.1464
217	2.7167	2.5041	2.5425	2.5547	2.6196	2.7148	2.4153	2.653	2.7376	2.7314	2.717	2.6502	2.5873	2.7453	2.647	2.7302	2.523	2.6018	2.6685	2.8271	2.7257	2.5813	2.6129	2.6236	2.6789	2.7606	2.5075	2.7071
218	17.053	14	15	17	18	18.05	13	17.053	17	18	17	17	17	14.063	18	20	15	16	16	19.326	19.048	15	16	18	19	19.048	14	18.05
219	8.3232	6.4775	7.3025	8.1633	8.9917	9.1065	6.4533	8.3232	8.1633	9.8685	8.1633	9	8.1633	6.0744	8.9917	10.688	7.3025	7.35	7.35	8.9091	9.2092	7.3025	8.144	8.9917	9.8336	9.904	7.3125	9.1065
220	5.8776	6.1875	6.2222	7.84	8.7075	7.0255	7.1006	6.321	6.4793	8.7075	7.84	7.84	7.84	3.7692	8.7075	10.412	6.2222	6.37	6.37	5.5	7.2	6.9758	7.0582	8.7075	9.5207	7.7051	8.0816	7.0255
221	14.286	10.889	13.279	13.068	14.058	13.346	9.8973	12.504	13.871	14.866	13.871	15.28	12.627	11.317	13.613	15.146	11.882	12.876	12.876	13.989	14.305	11.882	14.28	14.058	15.048	14.305	10.889	13.461
222	6.8994	4.9315	6.4043	6.1631	6.912	6.617	4.79	5.9886	6.5694	8.0519	6.5694	8.0323	5.9405	4.8117	6.6788	7.9655	5.6798	5.8222	5.8222	6.3459	6.8091	5.6798	7.2096	6.912	7.6799	7.3228	5.5673	6.6775
223	4.8321	4.6339	5.4153	5.8343	6.6152	5.0401	5.1575	4.4809	5.1572	7.041	6.2402	6.9576	5.6123	2.9505	6.3818	7.67	4.7675	4.9863	4.9863	3.87	5.2599	5.3449	6.2103	6.6152	7.3542	5.6289	6.0525	5.0881
224	5.1875	3.8357	5.6695	4.7376	5.3981	4.4155	3.6468	3.9411	5.3601	6.6499	5.3601	7.2197	4.4124	3.4033	5.051	6.0323	4.4992	4.6854	4.6854	3.8596	4.6382	4.4992	6.4344	5.3981	6.0826	4.9882	4.3302	4.4942
225	6	7	7	9	9	7	7	9	7	8	7	7	9	7	9	10	8	8	7	8	8	7	7	9	9	7	7	9
226	5	4	4	4	4	4	4	5	4	7	4	4	6	3	6	9	5	5	4	5	5	4	4	4	4	4	4	5
227	1	1	1	1	1	1	1	1	1	1	1	1	1	1	1	1	1	1	1	1	1	1	1	1	1	1	1	1
228	0	0	0	0	0	0	0	0	0	1	0	0	1	0	1	1	0	0	0	0	0	0	0	0	0	0	0	0
229	0.388	-1.576	-1.466	-2.203	-1.761	-0.041	-2.038	-2.345	-0.162	-1.507	-0.162	-0.921	-3.058	-1.188	-2.616	-1.928	-2.611	-2.149	-0.604	0.325	-0.349	-1.134	-1.024	-1.761	-1.319	0.401	-1.596	-1.903
230	-2.1423	-0.1518	-0.813	0.0372	-0.1646	-1.654	0.1754	-0.4111	-1.0706	-0.0969	-1.384	-1.0182	0.0149	-0.271	-0.1868	-0.5243	0.378	0.0507	-0.5553	-1.9439	-1.292	-0.3536	-1.0148	-0.1646	-0.3663	-1.8557	-0.0264	-0.6129
231	7.3566	4.5674	5.3188	5.1911	5.6775	7.1235	4.1096	6.3489	5.9846	6.3439	5.9745	6.2564	5.3929	5.3223	5.8763	6.8286	4.7153	5.1632	5.5018	8.1715	7.2411	5.0477	5.8016	5.6775	6.1606	7.5992	4.5933	6.8305
232	6	0	0	0	0	6	0	5	0	0	0	0	0	5	0	1	0	0	0	10	6	0	0	0	0	6	0	5
233	1	1	1	0	0	1	1	0	1	0	1	1	0	1	0	0	1	1	1	1	1	1	1	0	0	1	1	0
234	1	0	0	0	0	1	0	1	0	0	0	0	0	1	0	0	0	0	0	2	1	0	0	0	0	1	0	1
235	6	5	6	7	8	7	5	7	7	9	7	8	7	5	8	10	6	6	6	7	7	6	7	8	9	8	6	8
236	0	1	1	2	2	1	1	2	1	2	1	1	3	1	3	3	1	1	1	0	1	1	1	2	2	0	1	2
237	5.639	4.859	5.269	5.765	5.765	5.753	4.859	5.726	4.859	6.712	4.859	5.319	6.229	4.789	6.229	8.637	5.539	5.539	4.859	6.093	6.135	4.859	5.269	5.765	5.765	5.753	4.859	5.726
238	-5.639	-5.859	-6.269	-7.765	-7.765	-6.753	-5.859	-6.726	-5.859	-6.712	-5.859	-6.319	-7.229	-5.789	-7.229	-8.637	-6.539	-6.539	-5.859	-7.094	-7.135	-5.859	-6.269	-7.765	-7.765	-6.753	-5.859	-6.726
239	2.8092	2.1948	2.3301	2.2542	2.288	2.5036	2.1584	2.7461	2.3496	3.5918	2.3498	2.3599	2.7978	2.1994	2.8173	4.0595	2.5453	2.5787	2.2968	2.7462	2.737	2.2028	2.3382	2.2623	2.296	2.5116	2.1665	2.7541
240	-2.8092	-3.1948	-3.3301	-4.2542	-4.288	-3.5036	-3.1584	-3.7461	-3.3496	-3.5918	-3.3498	-3.3599	-3.7978	-3.1994	-3.8173	-4.0595	-3.5453	-3.5787	-3.2968	-3.7462	-3.737	-3.2028	-3.3382	-4.2623	-4.296	-3.5116	-3.1665	-3.7541
241	0.123	0.1574	0.1483	0.1533	0.151	0.138	0.1601	0.1258	0.1471	0.0962	0.1471	0.1464	0.1235	0.1571	0.1227	0.0851	0.1358	0.134	0.1504	0.1258	0.1262	0.1569	0.1478	0.1527	0.1505	0.1376	0.1595	0.1255
242	0.195	0.172	0.165	0.1292	0.1282	0.1569	0.174	0.1467	0.1641	0.153	0.1641	0.1636	0.1447	0.1718	0.144	0.1354	0.155	0.1536	0.1667	0.1467	0.1471	0.1717	0.1647	0.129	0.128	0.1566	0.1736	0.1465
243	17.062	44.737	51.149	63.881	63.711	40.471	31.941	65.919	70.329	57.533	70.329	61.798	40.471	70.725	57.703	49.002	31.941	44.737	61.798	66.125	40.471	53.268	59.68	72.412	72.241	49.002	40.471	74.449
244	58.317	22.12	22.12	22.12	22.12	43.447	36.432	54.322	22.12	43.843	22.12	22.12	30.821	22.12	22.12	43.843	45.602	31.29	22.12	43.447	47.64	22.12	22.12	22.12	22.12	43.447	36.432	54.322
245	12.046	4.2654	12.046	4.2654	4.2654	4.2654	4.2654	8.5308	4.2654	4.2654	4.2654	4.2654	4.2654	4.2654	4.2654	4.2654	4.2654	4.2654	4.2654	4.2654	4.2654	4.2654	12.046	4.2654	4.2654	4.2654	4.2654	8.5308
246	11.765	11.765	11.765	11.765	11.765	11.765	11.765	20.384	11.765	11.765	11.765	11.765	29.003	3.146	29.003	11.765	11.765	11.765	11.765	20.384	11.765	11.765	11.765	11.765	11.765	11.765	11.765	20.384
247	10.324	0	0	0	0	0	0	0	0	0	0	0	12.95	0	12.95	0	10.324	10.324	0	0	10.324	0	0	0	0	0	0	0
248	12.95	12.95	12.95	12.95	12.95	12.95	12.95	12.95	12.95	12.95	12.95	12.95	12.95	12.95	12.95	56.045	12.95	12.95	12.95	12.95	12.95	12.95	12.95	12.95	12.95	12.95	12.95	12.95
249	25.857	25.857	25.857	25.857	25.857	25.857	25.857	25.857	25.857	51.714	25.857	25.857	25.857	25.857	25.857	41.539	25.857	25.857	25.857	25.857	25.857	25.857	25.857	25.857	25.857	25.857	25.857	25.857
250	35.42	18.748	0	0	17.403	10.91	0	0	0	26.104	8.7013	40.102	0	17.403	8.7013	17.403	0	18.748	0	23.165	35.42	27.449	8.7013	8.7013	26.104	19.611	8.7013	8.7013
251	24.51	0	0	0	0	61.275	0	0	46.197	0	37.495	0	0	0	0	0	0	0	37.495	36.765	24.51	0	0	0	0	61.275	0	0
252	26.363	0	26.363	0	0	0	0	0	0	0	0	19.761	0	0	0	0	0	0	0	0	0	0	26.363	0	0	0	0	0
253	0	0	0	0	0	0	0	5.6826	0	0	0	0	0	0	0	0	0	0	0	0	0	0	0	0	0	0	0	5.6826
254	13.567	13.567	13.567	13.567	13.567	13.567	13.567	13.567	13.567	13.567	13.567	13.567	27.134	13.567	27.134	27.006	13.567	13.567	13.567	13.567	13.567	13.567	13.567	13.567	13.567	13.567	13.567	13.567
255	36.144	55.51	55.51	82.644	82.644	55.51	55.51	55.647	55.51	56.615	55.51	55.51	62.161	55.373	62.161	55.51	63.278	63.278	55.51	55.647	63.278	55.51	55.51	82.644	82.644	55.51	55.51	55.647
256	0.6524	0.4851	0.5336	0.4304	0.469	0.6147	0.4389	0.5674	0.5891	0.5156	0.5891	0.597	0.4257	0.522	0.4634	0.4122	0.4262	0.468	0.5602	0.6425	0.5657	0.5242	0.566	0.469	0.5027	0.6371	0.4851	0.594
257	0.4783	0.4192	0.4126	0.4059	0.4468	0.5045	0.3592	0.2849	0.439	0.3459	0.439	0.4817	0.3636	0.3831	0.3728	0.3261	0.35	0.4037	0.4344	0.4274	0.4716	0.4257	0.419	0.4126	0.4505	0.5045	0.3712	0.2985
258	0.1748	0.3297	0.2986	0.4059	0.3784	0.2467	0.3592	0.2849	0.2631	0.2521	0.2631	0.258	0.3636	0.3058	0.3397	0.2693	0.35	0.3245	0.2816	0.2291	0.2649	0.3046	0.2779	0.3784	0.3544	0.2324	0.3297	0.2674
259	0.3476	0.5149	0.4664	0.5696	0.531	0.3853	0.5611	0.4326	0.4109	0.4844	0.4109	0.403	0.5743	0.478	0.5366	0.5878	0.5738	0.532	0.4398	0.3575	0.4343	0.4758	0.434	0.531	0.4973	0.3629	0.5149	0.406
260	0.5217	0.5808	0.5874	0.5941	0.5532	0.4955	0.6408	0.7151	0.561	0.6541	0.561	0.5183	0.6364	0.6169	0.6272	0.6739	0.65	0.5963	0.5656	0.5726	0.5284	0.5743	0.581	0.5874	0.5495	0.4955	0.6288	0.7015
261	0.1728	0.1852	0.1678	0.1637	0.1526	0.1386	0.2018	0.1476	0.1478	0.2323	0.1478	0.145	0.2107	0.1722	0.1969	0.3185	0.2238	0.2075	0.1582	0.1284	0.1694	0.1711	0.1561	0.1526	0.1429	0.1306	0.1852	0.1386
262	185.48	101.63	123.44	102.03	119.26	172.13	84.403	149.16	154.68	143.51	154.68	159.81	104.56	117.66	121.79	126.28	93.573	110.8	137.44	194.15	164.07	118.87	140.68	119.26	136.5	189.36	101.63	166.39
263	136	87.825	95.44	96.211	113.61	141.26	69.077	74.896	115.27	96.286	115.27	128.94	89.295	86.343	97.996	99.919	76.844	95.592	106.57	129.14	136.77	96.526	104.14	104.91	122.31	149.96	77.778	83.598
264	49.711	69.077	69.077	96.211	96.211	69.077	69.077	74.896	69.077	70.182	69.077	69.077	89.295	68.94	89.295	82.516	76.844	76.844	69.077	69.214	76.844	69.077	69.077	96.211	96.211	69.077	69.077	74.896
265	98.841	107.88	107.88	135.02	135.02	107.88	107.88	113.7	107.88	134.85	107.88	107.88	141.05	107.75	141.05	180.1	125.98	125.98	107.88	108.02	125.98	107.88	107.88	135.02	135.02	107.88	107.88	113.7
266	148.32	121.69	135.89	140.84	140.67	138.76	123.21	187.96	147.29	182.07	147.29	138.76	156.32	139.06	164.85	206.46	142.7	141.19	138.76	173.03	153.27	130.22	144.42	149.37	149.2	147.29	131.74	196.49
267	49.131	38.807	38.807	38.807	38.807	38.807	38.807	38.807	38.807	64.664	38.807	38.807	51.756	38.807	51.756	97.583	49.131	49.131	38.807	38.807	49.131	38.807	38.807	38.807	38.807	38.807	38.807	38.807
268	0.4545	0.5	0.5	0.4444	0.5	0.4545	0.5	0.5	0.4444	0.4545	0.4444	0.5	0.4444	0.5	0.5	0.5	0.5	0.5	0.5	0.5	0.5	0.4444	0.4444	0.5	0.4545	0.5	0.4444	0.4545
269	0.8333	1	1	0.8	1	0.8333	1	1	0.8	0.8333	0.8	1	0.8	1	1	1	1	1	1	1	1	0.8	0.8	1	0.8333	1	0.8	0.8333
270	5.639	4.859	5.269	5.765	5.765	5.753	4.859	5.726	4.859	6.712	4.859	5.319	6.229	4.789	6.229	8.637	5.539	5.539	4.859	6.093	6.135	4.859	5.269	5.765	5.765	5.753	4.859	5.726
271	-5.639	-5.859	-6.269	-7.765	-7.765	-6.753	-5.859	-6.726	-5.859	-6.712	-5.859	-6.319	-7.229	-5.789	-7.229	-8.637	-6.539	-6.539	-5.859	-7.094	-7.135	-5.859	-6.269	-7.765	-7.765	-6.753	-5.859	-6.726
272	0.1607	0.1865	0.1719	0.1572	0.1572	0.1575	0.1865	0.1582	0.1865	0.135	0.1865	0.1703	0.1454	0.1892	0.1454	0.1389	0.1636	0.1636	0.1865	0.1487	0.1477	0.1865	0.1719	0.1572	0.1572	0.1575	0.1865	0.1582
273	0.1596	0.1536	0.1436	0.1159	0.1159	0.1333	0.1536	0.1338	0.1536	0.1341	0.1536	0.1424	0.1245	0.1555	0.1245	0.112	0.1376	0.1376	0.1536	0.1269	0.1261	0.1536	0.1436	0.1159	0.1159	0.1333	0.1536	0.1338
274	0.429	0.2885	0.1955	0.1946	0.2492	0.4677	0.2248	0.3148	0.4323	0.3202	0.4323	0.252	0.1879	0.319	0.2411	0.2347	0.1705	0.2733	0.3924	0.4227	0.4237	0.3426	0.2513	0.2492	0.2969	0.4985	0.2885	0.3569
275	0.4477	0.3712	0.4502	0.4793	0.4468	0.5045	0.4045	0.3812	0.2962	0.2834	0.2962	0.3644	0.399	0.3445	0.3728	0.2977	0.3896	0.3613	0.317	0.5045	0.4716	0.343	0.419	0.4468	0.4184	0.4752	0.3712	0.3578
276	0.2676	0.3297	0.4126	0.4059	0.3784	0.2467	0.3592	0.3481	0.2631	0.2521	0.2631	0.3319	0.3636	0.3058	0.3397	0.2693	0.35	0.3245	0.2816	0.284	0.2649	0.3046	0.384	0.3784	0.3544	0.2324	0.3297	0.3267
277	0.571	0.7115	0.8045	0.8054	0.7508	0.5323	0.7752	0.6852	0.5677	0.6798	0.5677	0.748	0.8121	0.681	0.7589	0.7653	0.8295	0.7267	0.6076	0.5773	0.5763	0.6574	0.7487	0.7508	0.7031	0.5015	0.7115	0.6431
278	0.5523	0.6288	0.5498	0.5207	0.5532	0.4955	0.5955	0.6188	0.7038	0.7166	0.7038	0.6356	0.601	0.6555	0.6272	0.7023	0.6104	0.6387	0.683	0.4955	0.5284	0.657	0.581	0.5532	0.5816	0.5248	0.6288	0.6422
279	0.3035	0.3818	0.3919	0.3995	0.3724	0.2857	0.416	0.3371	0.3046	0.4276	0.3046	0.4161	0.4486	0.3752	0.4192	0.496	0.4795	0.4021	0.326	0.2932	0.3114	0.3528	0.3648	0.3724	0.3488	0.2691	0.3818	0.3164
280	121.97	60.453	45.221	46.141	63.373	130.95	43.221	82.745	113.49	89.136	113.49	67.468	46.141	71.904	63.373	71.904	37.44	64.718	96.262	127.74	122.89	77.685	62.453	63.373	80.605	148.18	60.453	99.977

	E	E	E	E	E	E	E	E	E	E	E	E	E	F	F	F	F	F	F	F	F	F	F	F	F	F	F
	I	K	L	M	N	P	Q	R	S	T	V	W	Y	A	C	D	E	F	G	H	I	K	L	M	N	P	Q
No.	EI	EK	EL	EM	EN	EP	EQ	ER	ES	ET	EV	EW	EY	FA	FC	FD	FE	FF	FG	FH	FI	FK	FL	FM	FN	FP	FQ
206	-0.8526	-0.863	-0.8526	-0.8526	-0.8526	-0.8526	-0.8526	-0.8526	-0.8525	-0.8525	-0.8526	-0.863	-0.8526	-0.8472	-0.8465	-0.8505	-0.8543	-0.8468	-0.848	-0.8488	-0.8469	-0.863	-0.8472	-0.847	-0.8467	-0.8513	-0.8471
207	-0.4823	-0.4132	-0.4423	-0.4683	-0.5183	-0.5002	-0.5077	-0.4541	-0.5146	-0.5022	-0.4971	-0.4841	-0.5201	-0.3906	-0.39	-0.48	-0.4735	-0.39	-0.4151	-0.39	-0.362	-0.3755	-0.3693	-0.3788	-0.39	-0.39	-0.39
208	0.0988	0.1849	0.0802	0.088	0.095	0.1123	0.09	0.1279	0.1567	0.1421	0.0989	0.1278	0.1279	0.1147	0.1855	0.1123	0.0929	0.0793	0.1428	0.1801	0.0948	0.1849	0.0797	0.0793	0.0866	0.1169	0.0793
209	2.5713	2.5794	2.553	2.4817	2.447	2.582	2.4946	2.5709	2.4011	2.4632	2.5133	2.6018	2.5373	2.4713	2.4841	2.4956	2.5384	2.5899	2.4181	2.5584	2.6277	2.6327	2.6111	2.5517	2.5229	2.64	2.5621
210	-2.2351	-2.2354	-2.2351	-2.2351	-2.2351	-2.2351	-2.2351	-2.2351	-2.2351	-2.2351	-2.2351	-2.2351	-2.2351	-2.2351	-2.2351	-2.2352	-2.2352	-2.2352	-2.2351	-2.2352	-2.2352	-2.2354	-2.2352	-2.2352	-2.2352	-2.2352	-2.2352
211	-0.4272	-0.396	-0.413	-0.4287	-0.5053	-0.4483	-0.4641	-0.4517	-0.5475	-0.4855	-0.4615	-0.4236	-0.4389	-0.4022	-0.4288	-0.4679	-0.4405	-0.3977	-0.44	-0.4079	-0.3297	-0.3372	-0.3297	-0.3515	-0.42	-0.3997	-0.4182
212	0.0542	0.0542	0.0542	0.0567	0.0542	0.0542	0.0542	0.0555	0.0542	0.0542	0.0542	0.1088	0.0727	0.0853	0.1382	0.0897	0.0871	0.1383	0.1105	0.1382	0.083	0.0848	0.0848	0.1382	0.0585	0.0777	0.0581
213	2.4977	2.4834	2.484	2.376	2.2805	2.4828	2.362	2.4392	2.2259	2.3031	2.4098	2.5854	2.4795	2.3967	2.4058	2.4108	2.4612	2.5397	2.3502	2.4755	2.5748	2.5617	2.5622	2.4859	2.4269	2.571	2.4778
214	-0.6409	-0.6419	-0.6409	-0.6409	-0.6409	-0.641	-0.6409	-0.6409	-0.6409	-0.6409	-0.6409	-0.6409	-0.6409	-0.6409	-0.6409	-0.6409	-0.6409	-0.6409	-0.6409	-0.6409	-0.6409	-0.6419	-0.6409	-0.6409	-0.6409	-0.641	-0.6409
215	-0.1855	-0.1958	-0.1928	-0.185	-0.2214	-0.2059	-0.2216	-0.2097	-0.2226	-0.2098	-0.1875	-0.2149	-0.2239	-0.2081	-0.2039	-0.2109	-0.2106	-0.2161	-0.2155	-0.2161	-0.1978	-0.205	-0.2029	-0.2012	-0.2161	-0.2161	-0.2161
216	0.1027	0.1112	0.1109	0.1449	0.1441	0.0858	0.1449	0.1283	0.1445	0.1279	0.1034	0.1663	0.174	0.162	0.1673	0.1634	0.1633	0.1697	0.1695	0.1679	0.1491	0.16	0.1581	0.1662	0.1649	0.163	0.1662
217	2.7833	2.776	2.7638	2.7049	2.6513	2.7947	2.7025	2.7749	2.5972	2.6641	2.722	2.86	2.7701	2.6904	2.7134	2.722	2.7645	2.8281	2.6361	2.7858	2.849	2.8414	2.8319	2.7843	2.7432	2.8624	2.7829
218	18	19	18	18	18	15.059	19	21	16	17	17	20.314	20.045	15.059	16.056	18.05	19.048	19.326	14.063	18.34	18.05	19.048	18.05	18.05	18.05	15.39	19.048
219	8.9917	10.748	8.9917	9.8685	8.9917	6.8053	9.8336	11.552	8.144	8.1633	8.1633	9.6298	9.9881	7.438	8.2268	9.1065	9.904	10.096	7.35	9.3333	9.1065	10.68	9.1065	9.8496	9.1065	7.1358	9.904
220	7.259	9.5207	8.7075	8.7075	8.7075	4.3018	9.5207	11.25	7.0582	7.1111	7.1111	6.0464	7.9084	5.4444	5.6805	7.0255	7.7051	6.7901	5.7778	6.2041	6.12	7.7051	7.0255	7.0255	7.0255	3.9862	7.7051
221	14.866	15.862	14.866	16.281	13.613	12.287	14.6	16.132	12.876	13.871	13.871	14.92	15.267	11.163	13.309	13.346	14.305	13.884	10.211	13.064	14.045	15.013	14.045	15.264	12.96	11.805	13.914
222	7.3365	8.8765	7.3365	8.8701	6.6788	5.4765	7.4369	8.7483	6.4512	6.5694	6.5694	6.9702	7.5011	5.4009	6.7389	6.617	7.3228	7.1346	5.212	6.5312	6.9852	8.3108	6.9852	8.2513	6.4142	5.3864	7.1111
223	5.8697	7.7971	7.041	7.7904	6.3818	3.424	7.1108	8.4262	5.525	5.66	5.66	4.3306	5.8789	3.8885	4.6122	5.0401	5.6289	4.7388	4.0244	4.2858	4.6453	5.9365	5.3327	5.845	4.8792	2.9713	5.4594
224	6.0591	7.4104	6.0591	8.0229	5.051	3.9582	5.7148	6.7205	5.1916	5.3601	5.3601	4.3331	5.2056	3.5464	4.9827	4.4155	4.9882	4.3069	3.3262	3.8782	4.9054	5.9415	4.9054	6.2976	4.1564	3.3466	4.7115
225	7	8	7	7	9	7	9	10	8	8	7	8	8	5	5	7	7	5	5	7	5	6	5	5	7	5	7
226	4	7	4	4	6	3	6	9	5	5	4	5	5	4	4	4	4	4	4	5	4	7	4	4	6	3	6
227	1	1	1	1	1	1	1	1	1	1	1	1	1	1	1	1	1	1	1	1	1	1	1	1	1	1	1
228	0	1	0	0	1	0	1	1	0	0	0	0	0	0	0	0	0	0	0	0	0	1	0	0	1	0	1
229	0.28	-1.065	0.28	-0.479	-2.616	-0.746	-2.174	-1.486	-2.169	-1.707	-0.162	0.767	0.093	0.586	0.696	-0.041	0.401	2.121	0.124	-0.183	2	0.655	2	1.241	-0.896	0.974	-0.454
230	-1.2723	-0.2986	-1.5858	-1.22	-0.1868	-0.4727	-0.3886	-0.726	0.1762	-0.151	-0.7571	-2.1456	-1.4938	-1.843	-2.5042	-1.654	-1.8557	-3.3451	-1.5157	-2.1023	-2.7617	-1.788	-3.0752	-2.7094	-1.6762	-1.9621	-1.878
231	6.4588	6.8189	6.4488	6.7342	5.8763	5.7999	6.3571	7.307	5.1981	5.6432	5.9776	8.6485	7.7187	6.4719	7.2294	7.1235	7.5992	8.9962	6.0254	8.2453	7.8648	8.2268	7.8548	8.1486	7.3114	7.2126	7.7854
232	0	0	0	0	0	5	0	1	0	0	0	10	6	6	6	6	6	12	6	11	6	6	6	6	6	11	6
233	1	0	1	1	0	1	0	0	1	1	1	1	1	1	1	1	1	1	1	1	1	1	1	1	1	1	1
234	0	0	0	0	0	1	0	0	0	0	0	2	1	1	1	1	1	2	1	2	1	1	1	1	1	2	1
235	8	10	8	9	8	6	9	11	7	7	7	8	8	5	6	7	8	7	5	7	7	9	7	8	7	5	8
236	1	2	1	1	3	1	3	4	1	1	1	0	1	0	0	1	0	0	0	0	0	1	0	0	1	0	1
237	4.859	6.712	4.859	5.319	6.229	4.789	6.229	8.637	5.539	5.539	4.859	6.093	6.135	4.847	5.257	5.753	5.753	5.741	4.847	5.714	4.847	6.7	4.847	5.307	6.217	4.777	6.217
238	-5.859	-6.712	-5.859	-6.319	-7.229	-5.789	-7.229	-8.637	-6.539	-6.539	-5.859	-7.094	-7.135	-4.847	-5.257	-6.753	-6.753	-5.741	-4.847	-5.714	-4.847	-5.7	-4.847	-5.307	-6.217	-4.777	-6.217
239	2.3576	3.5999	2.3578	2.3679	2.8058	2.2075	2.8253	4.0676	2.5534	2.5868	2.3049	2.7542	2.745	2.4383	2.5736	2.4977	2.5314	2.7471	2.4019	2.9896	2.5931	3.8353	2.5933	2.6034	3.0412	2.4429	3.0608
240	-3.3576	-3.5999	-3.3578	-3.3679	-3.8058	-3.2075	-3.8253	-4.0676	-3.5534	-3.5868	-3.3049	-3.7542	-3.745	-2.4383	-2.5736	-3.4977	-3.5314	-2.7471	-2.4019	-2.9896	-2.5931	-2.8353	-2.5933	-2.6034	-3.0412	-2.4429	-3.0608
241	0.1466	0.096	0.1465	0.1459	0.1232	0.1565	0.1223	0.0849	0.1353	0.1336	0.1499	0.1255	0.1259	0.1417	0.1343	0.1383	0.1365	0.1258	0.1439	0.1156	0.1333	0.0901	0.1332	0.1327	0.1136	0.1414	0.1129
242	0.1638	0.1527	0.1637	0.1633	0.1445	0.1714	0.1437	0.1352	0.1547	0.1533	0.1664	0.1465	0.1468	0.2246	0.2128	0.1572	0.1557	0.1994	0.2281	0.1832	0.2112	0.1932	0.2112	0.2104	0.1801	0.2242	0.1789
243	78.86	66.064	78.86	70.329	49.002	79.256	66.234	57.533	40.471	53.268	70.329	74.656	49.002	30.028	36.44	49.173	49.002	25.763	17.232	51.21	55.621	42.824	55.621	47.09	25.763	56.017	42.995
244	22.12	43.843	22.12	22.12	30.821	22.12	22.12	43.843	45.602	31.29	22.12	43.447	47.64	43.447	43.447	43.447	43.447	64.774	57.759	75.649	43.447	65.17	43.447	43.447	52.148	43.447	43.447
245	4.2654	4.2654	4.2654	4.2654	4.2654	4.2654	4.2654	4.2654	4.2654	4.2654	4.2654	4.2654	4.2654	4.2654	12.046	4.2654	4.2654	4.2654	4.2654	8.5308	4.2654	4.2654	4.2654	4.2654	4.2654	4.2654	4.2654
246	11.765	11.765	11.765	11.765	29.003	3.146	29.003	11.765	11.765	11.765	11.765	20.384	11.765	11.765	11.765	11.765	11.765	11.765	11.765	20.384	11.765	11.765	11.765	11.765	29.003	3.146	29.003
247	0	0	0	0	12.95	0	12.95	0	10.324	10.324	0	0	10.324	0	0	0	0	0	0	0	0	0	0	0	12.95	0	12.95
248	12.95	12.95	12.95	12.95	12.95	12.95	12.95	56.045	12.95	12.95	12.95	12.95	12.95	12.95	12.95	12.95	12.95	12.95	12.95	12.95	12.95	12.95	12.95	12.95	12.95	12.95	12.95
249	25.857	51.714	25.857	25.857	25.857	25.857	25.857	41.539	25.857	25.857	25.857	25.857	25.857	25.857	25.857	25.857	25.857	25.857	25.857	25.857	25.857	51.714	25.857	25.857	25.857	25.857	25.857
250	8.7013	34.805	17.403	48.804	8.7013	26.104	17.403	26.104	8.7013	27.449	8.7013	31.866	44.121	20.956	2.2085	2.2085	19.611	13.118	2.2085	2.2085	2.2085	28.312	10.91	42.311	2.2085	19.611	10.91
251	46.197	0	37.495	0	0	0	0	0	0	0	37.495	36.765	24.51	61.275	61.275	61.275	61.275	122.55	61.275	61.275	107.47	61.275	98.77	61.275	61.275	61.275	61.275
252	0	0	0	19.761	0	0	0	0	0	0	0	0	0	0	26.363	0	0	0	0	0	0	0	0	19.761	0	0	0
253	0	0	0	0	0	0	0	0	0	0	0	0	0	0	0	0	0	0	0	5.6826	0	0	0	0	0	0	0
254	13.567	13.567	13.567	13.567	27.134	13.567	27.134	27.006	13.567	13.567	13.567	13.567	13.567	13.567	13.567	13.567	13.567	13.567	13.567	13.567	13.567	13.567	13.567	13.567	27.134	13.567	27.134
255	55.51	56.615	55.51	55.51	62.161	55.373	62.161	55.51	63.278	63.278	55.51	55.647	63.278	28.376	28.376	55.51	55.51	28.376	28.376	28.513	28.376	29.482	28.376	28.376	35.027	28.239	35.027
256	0.6144	0.5438	0.6144	0.6214	0.4634	0.5559	0.4964	0.4435	0.468	0.5041	0.5891	0.6618	0.59	0.6802	0.7056	0.6147	0.6371	0.75	0.6568	0.7169	0.7357	0.6648	0.7357	0.7401	0.6052	0.6996	0.6275
257	0.4431	0.3552	0.4431	0.4831	0.3728	0.3917	0.381	0.3357	0.3613	0.4106	0.439	0.4316	0.4734	0.4918	0.4805	0.4734	0.5045	0.5499	0.4481	0.3638	0.4963	0.4128	0.4963	0.5321	0.4354	0.4572	0.4393
258	0.2469	0.2374	0.2469	0.2424	0.3397	0.2841	0.3188	0.255	0.3245	0.3025	0.2631	0.2167	0.2501	0.1661	0.1529	0.2467	0.2324	0.1299	0.1783	0.1562	0.1373	0.134	0.1373	0.135	0.2154	0.1558	0.2033
259	0.3856	0.4562	0.3856	0.3786	0.5366	0.4441	0.5036	0.5565	0.532	0.4959	0.4109	0.3382	0.41	0.3198	0.2944	0.3853	0.3629	0.25	0.3432	0.2831	0.2643	0.3352	0.2643	0.2599	0.3948	0.3004	0.3725
260	0.5569	0.6448	0.5569	0.5169	0.6272	0.6083	0.619	0.6643	0.6387	0.5894	0.561	0.5684	0.5266	0.5082	0.5195	0.5266	0.4955	0.4501	0.5519	0.6362	0.5037	0.5872	0.5037	0.4679	0.5646	0.5428	0.5607
261	0.1387	0.2188	0.1387	0.1362	0.1969	0.1599	0.1848	0.3015	0.2075	0.1934	0.1478	0.1215	0.1599	0.1537	0.1415	0.1386	0.1306	0.1202	0.165	0.1269	0.127	0.2012	0.127	0.1249	0.1793	0.1446	0.1692
262	171.91	160.74	171.91	177.04	121.79	134.89	139.03	143.51	110.8	128.04	154.68	211.38	181.3	171.74	193.54	172.13	189.36	242.23	154.5	219.26	224.78	213.61	224.78	229.91	174.66	187.76	191.89
263	123.97	104.99	123.97	137.64	97.996	95.044	106.7	108.62	85.546	104.29	115.27	137.84	145.48	124.17	131.79	132.56	149.96	177.61	105.43	111.25	151.62	132.64	151.62	165.29	125.64	122.69	134.35
264	69.077	70.182	69.077	69.077	89.295	68.94	89.295	82.516	76.844	76.844	69.077	69.214	76.844	41.943	41.943	69.077	69.077	41.943	41.943	47.763	41.943	43.049	41.943	41.943	62.161	41.806	62.161
265	107.88	134.85	107.88	107.88	141.05	107.75	141.05	180.1	125.98	125.98	107.88	108.02	125.98	80.75	80.75	107.88	107.88	80.75	80.75	86.569	80.75	107.71	80.75	80.75	113.92	80.613	113.92
266	155.82	190.6	155.82	147.29	164.85	147.59	173.38	214.99	151.23	149.72	147.29	181.56	161.8	128.31	142.51	147.46	147.29	145.37	129.83	194.58	153.9	188.69	153.9	145.37	162.94	145.68	171.47
267	38.807	64.664	38.807	38.807	51.756	38.807	51.756	97.583	49.131	49.131	38.807	38.807	49.131	38.807	38.807	38.807	38.807	38.807	38.807	38.807	38.807	64.664	38.807	38.807	51.756	38.807	51.756
268	0.5	0.5	0.5	0.4545	0.5	0.4444	0.4545	0.4615	0.4444	0.4444	0.4444	0.4615	0.4615	0.5	0.5	0.4545	0.5	0.4615	0.5	0.5	0.4545	0.4615	0.4545	0.5	0.4545	0.5	0.5
269	1	1	1	0.8333	1	0.8	0.8333	0.8571	0.8	0.8	0.8	0.8571	0.8571	1	1	0.8333	1	0.8571	1	1	0.8333	0.8571	0.8333	1	0.8333	1	1
270	4.859	6.712	4.859	5.319	6.229	4.789	6.229	8.637	5.539	5.539	4.859	6.093	6.135	4.847	5.257	5.753	5.753	5.741	4.847	5.714	4.847	6.7	4.847	5.307	6.217	4.777	6.217
271	-5.859	-6.712	-5.859	-6.319	-7.229	-5.789	-7.229	-8.637	-6.539	-6.539	-5.859	-7.094	-7.135	-4.847	-5.257	-6.753	-6.753	-5.741	-4.847	-5.714	-4.847	-5.7	-4.847	-5.307	-6.217	-4.777	-6.217
272	0.1865	0.135	0.1865	0.1703	0.1454	0.1892	0.1454	0.1389	0.1636	0.1636	0.1865	0.1487	0.1477	0.1869	0.1723	0.1575	0.1575	0.1578	0.1869	0.1586	0.1869	0.1352	0.1869	0.1707	0.1457	0.1897	0.1457
273	0.1536	0.1341	0.1536	0.1424	0.1245	0.1555	0.1245	0.112	0.1376	0.1376	0.1536	0.1269	0.1261	0.1857	0.1712	0.1333	0.1333	0.1568	0.1857	0.1575	0.1857	0.1579	0.1857	0.1696	0.1448	0.1884	0.1448
274	0.4672	0.3599	0.4672	0.2973	0.2411	0.3674	0.2878	0.2754	0.2309	0.3226	0.4323	0.4539	0.456	0.5753	0.4741	0.4677	0.4985	0.668	0.5442	0.5479	0.6491	0.5413	0.6491	0.4902	0.4538	0.5839	0.4846
275	0.278	0.2669	0.278	0.3423	0.3728	0.32	0.3499	0.2819	0.3613	0.3368	0.2962	0.4773	0.4451	0.4176	0.4805	0.5045	0.4752	0.523	0.4481	0.4181	0.3451	0.3315	0.3451	0.403	0.4354	0.3923	0.4109
276	0.2469	0.2374	0.2469	0.3118	0.3397	0.2841	0.3188	0.255	0.3245	0.3025	0.2631	0.2687	0.2501	0.1661	0.249	0.2467	0.2324	0.1299	0.1783	0.2105	0.1373	0.134	0.1373	0.1986	0.2154	0.1558	0.2033
277	0.5328	0.6401	0.5328	0.7027	0.7589	0.6326	0.7122	0.7246	0.7691	0.6774	0.5677	0.5461	0.544	0.4247	0.5259	0.5323	0.5015	0.332	0.4558	0.4521	0.3509	0.4587	0.3509	0.5098	0.5462	0.4161	0.5154
278	0.722	0.7331	0.722	0.6577	0.6272	0.68	0.6501	0.7181	0.6387	0.6632	0.7038	0.5227	0.5549	0.5824	0.5195	0.4955	0.5248	0.477	0.5519	0.5819	0.6549	0.6685	0.6549	0.597	0.5646	0.6077	0.5891
279	0.2859	0.4027	0.2859	0.3909	0.4192	0.3485	0.3934	0.4696	0.4446	0.3749	0.3046	0.2774	0.2939	0.2585	0.2769	0.2857	0.2691	0.2021	0.2775	0.2416	0.2137	0.3247	0.2137	0.3112	0.3308	0.2603	0.3122
280	130.73	106.37	130.73	84.7	63.373	89.136	80.605	89.136	54.672	81.95	113.49	144.97	140.12	145.26	130.03	130.95	148.18	215.76	128.03	167.56	198.3	173.95	198.3	152.28	130.95	156.71	148.18

Table S12 Amino acid descriptors (2D) used in this study.

	C	D	D	D	D	D	D	D	D	D	D	D	D	D	D	D	D	D	D	D	D	E	E	E	E	E
	Y	A	C	D	E	F	G	H	I	K	L	M	N	P	Q	R	S	T	V	W	Y	A	C	D	E	F
No.	CY	DA	DC	DD	DE	DF	DG	DH	DI	DK	DL	DM	DN	DP	DQ	DR	DS	DT	DV	DW	DY	EA	EC	ED	EE	EF
281	127.3	77.778	104.14	113.61	113.61	141.26	77.778	100.21	77.778	78.884	77.778	97.539	97.996	77.641	97.996	91.217	85.546	85.546	77.778	152.45	136.77	77.778	104.14	113.61	113.61	141.26
282	76.074	69.077	95.44	96.211	96.211	69.077	69.077	91.505	69.077	70.182	69.077	88.838	89.295	68.94	89.295	82.516	76.844	76.844	69.077	85.823	76.844	69.077	95.44	96.211	96.211	69.077
283	162.36	149.07	186.11	190.91	190.91	149.07	149.07	180.11	149.07	189.22	149.07	200.23	199.47	153.5	199.47	234.47	182.11	172.06	149.07	174.43	167.16	149.07	186.11	190.91	190.91	149.07
284	157.02	131.74	127.19	123.44	140.67	138.76	114.51	162.65	184.78	199.47	184.78	170.16	147.62	147.76	164.85	215.16	134	151.23	167.55	149.72	153.27	148.97	144.42	140.67	157.9	155.99
285	86.282	79.989	90.666	94.697	94.697	79.989	79.989	88.608	79.989	119.04	79.989	111.39	110.18	84.562	110.18	151.96	105.26	95.218	79.989	88.608	90.313	79.989	90.666	94.697	94.697	79.989
286	6	4	4	5	5	6	4	5	5	6	5	5	5	4	5	6	4	4	4	6	6	5	5	5	6	6
287	1	0	1	0	0	0	0	0	0	0	0	0	0	0	0	0	0	0	0	0	0	0	1	0	0	0
288	1	0	0	0	0	1	0	1	0	0	0	0	0	1	0	0	0	0	0	2	1	0	0	0	0	1
289	0.1607	0.1865	0.1719	0.1572	0.1572	0.1575	0.1865	0.1582	0.1865	0.135	0.1865	0.1703	0.1454	0.1892	0.1454	0.1389	0.1636	0.1636	0.1865	0.1487	0.1477	0.1865	0.1719	0.1572	0.1572	0.1575
290	0.1596	0.1536	0.1436	0.1159	0.1159	0.1333	0.1536	0.1338	0.1536	0.1341	0.1536	0.1424	0.1245	0.1555	0.1245	0.112	0.1376	0.1376	0.1536	0.1269	0.1261	0.1536	0.1436	0.1159	0.1159	0.1333
291	-2.2903	-5.0085	-5.0986	-6.8884	-6.4983	-3.7857	-5.397	-5.0626	-3.9823	-5.6162	-3.9823	-4.2753	-6.153	-4.5222	-5.7629	-7.5848	-6.0361	-5.6476	-4.3724	-3.3044	-4.0801	-4.6184	-4.7085	-6.4983	-6.1082	-3.3956
292	118.98	136.27	136.27	178.96	178.96	136.27	136.27	136.27	136.27	169.17	136.27	136.27	169.17	118.26	169.17	218.85	161.66	161.66	136.27	136.27	161.66	136.27	136.27	178.96	178.96	136.27
293	6.7228	8.1868	8.1868	9.6508	9.6508	8.1868	8.1868	24.972	8.1868	8.1868	8.1868	8.1868	13.446	11.311	13.446	10.086	8.1868	8.1868	8.1868	8.1868	8.1868	8.1868	8.1868	9.6508	9.6508	8.1868
294	55.688	79.551	79.551	103.41	103.41	79.551	75.569	96.336	79.551	79.551	79.551	79.551	103.41	79.551	103.41	79.551	79.551	86.019	79.551	96.336	79.551	79.551	79.551	103.41	103.41	79.551
295	17.234	0	17.234	0	0	0	18.44	0	0	18.44	0	17.234	0	18.44	0	18.44	20.926	0	0	0	0	0	17.234	0	0	0
296	3.1856	0	0	0	0	3.1856	0	2.7567	4.4108	0	4.4108	0	0	0	0	0	0	0	4.4108	3.1856	3.1856	0	0	0	0	3.1856
297	18.868	0	0	0	0	18.868	0	18.868	0	0	0	31.691	0	0	0	0	0	0	0	18.868	18.868	0	0	0	0	18.868
298	70.573	0	0	0	0	88.216	0	34	0	0	0	0	0	0	0	0	0	0	0	92.504	70.573	0	0	0	0	88.216
299	46.107	18.868	64.976	37.737	56.605	18.868	18.868	18.868	37.737	75.474	37.737	37.737	37.737	56.605	56.605	56.605	18.868	18.868	18.868	18.868	18.868	37.737	83.844	56.605	75.474	37.737
300	5.2434	33.326	0	0	0	0	0	0	66.652	0	66.652	32.22	0	0	0	0	0	33.326	66.652	0	5.2434	33.326	0	0	0	0
301	6.9279	3.8912	4.7092	4.2862	4.7479	6.3384	3.4317	5.5529	5.2693	5.4844	5.2693	5.6238	4.7049	4.6042	5.1666	6.1594	4.0324	4.4919	4.8076	7.5241	6.5049	4.3529	5.1709	4.7479	5.2096	6.8001
302	90.408	156.95	156.95	223.5	223.5	156.95	156.95	156.95	156.95	156.95	156.95	156.95	180.82	156.95	180.82	156.95	156.95	156.95	156.95	156.95	156.95	156.95	156.95	223.5	223.5	156.95
303	25.385	0	0	0	0	0	0	16.786	0	0	0	0	0	3.1243	0	31.672	25.385	25.385	0	0	25.385	0	0	0	0	0
304	50.908	50.908	50.908	50.908	50.908	50.908	50.908	67.693	55.319	83.805	55.319	50.908	50.908	32.897	50.908	68.919	50.908	50.908	55.319	67.693	50.908	50.908	50.908	50.908	50.908	50.908
305	16.393	7.9639	7.9639	7.9639	7.9639	11.15	3.982	10.721	7.9639	7.9639	7.9639	7.9639	7.9639	7.9639	7.9639	7.9639	7.9639	14.433	7.9639	11.15	16.393	7.9639	7.9639	7.9639	7.9639	11.15
306	0	0	0	0	0	0	0	0	0	0	0	0	32.897	0	32.897	32.897	0	0	0	4.7171	0	0	0	0	0	0
307	89.441	18.868	18.868	37.737	56.605	125.95	18.868	71.737	37.737	75.474	37.737	37.737	37.737	56.605	56.605	56.605	18.868	18.868	18.868	125.52	108.31	37.737	37.737	56.605	75.474	144.82
308	23.956	8.1868	25.42	9.6508	9.6508	8.1868	26.626	8.1868	8.1868	26.626	8.1868	25.42	13.446	26.626	13.446	28.525	29.113	8.1868	8.1868	8.1868	8.1868	8.1868	25.42	9.6508	9.6508	8.1868
309	46.107	33.326	46.107	0	0	0	0	0	66.652	0	66.652	63.911	0	0	0	0	0	33.326	66.652	0	0	33.326	46.107	0	0	0
310	155.9	137	175.8	177.13	177.13	137	137	165.68	137	164.64	137	137	180.09	128.21	180.09	200.64	157.23	157.23	137	152.79	157.23	137	175.8	177.13	177.13	137
311	0.4855	0.5647	0.542	0.5021	0.4845	0.469	0.5895	0.4855	0.5021	0.4845	0.5021	0.5021	0.5021	0.5436	0.4845	0.4529	0.542	0.5212	0.5212	0.4389	0.4537	0.542	0.5212	0.4845	0.4681	0.4537
312	5.2479	4.7004	4.8074	5	5.0875	5.3219	4.585	5.2479	5	5.0875	5	5	5	5	5.0875	5.2479	4.8074	4.9069	4.9069	5.585	5.3923	4.8074	4.9069	5.0875	5.1699	5.3923
313	3.1854	2.8758	2.9058	3.0396	3.1568	3.2484	2.8693	3.1686	2.9996	3.2205	3.0396	3.1044	3.0396	2.8527	3.1568	3.3998	2.9058	2.92	2.92	3.3633	3.3366	3.0302	3.0576	3.1778	3.2811	3.3642
314	8.1993	7.2974	7.5087	7.8816	8.0431	8.3507	7.0665	8.2025	7.886	8.0365	7.8816	7.8754	7.8816	7.7091	8.0431	8.3336	7.5087	7.7051	7.7051	8.7568	8.4867	7.4883	7.6856	8.0375	8.1921	8.4845
315	284.32	209.52	231.33	237.05	254.28	280.02	192.29	262.86	262.56	278.36	262.56	267.7	245.61	225.41	262.84	306.38	219.55	236.78	245.33	302.17	290.05	226.75	248.56	254.28	271.51	297.25
316	346.62	231.54	252.86	261.28	285.71	346.51	207.11	310.77	304.83	323.38	304.83	302.78	271.82	268.12	296.25	347.51	240.06	264.49	280.4	391.86	355.04	255.97	277.29	285.71	310.14	370.94
317	40.701	67.835	67.835	94.968	94.968	67.835	67.835	67.835	67.835	67.835	67.835	67.835	81.402	67.835	81.402	67.835	67.835	67.835	67.835	67.835	67.835	67.835	67.835	94.968	94.968	67.835
318	27.134	54.268	54.268	81.402	81.402	54.268	54.268	54.268	54.268	54.268	54.268	54.268	54.268	54.268	54.268	54.268	54.268	54.268	54.268	54.268	54.268	54.268	54.268	81.402	81.402	54.268
319	17.742	17.742	17.742	17.742	17.742	17.742	17.742	17.742	17.742	35.485	17.742	17.742	17.742	17.742	17.742	58.91	17.742	17.742	17.742	17.742	17.742	17.742	17.742	17.742	17.742	17.742
320	23.425	23.425	23.425	23.425	23.425	23.425	23.425	23.425	23.425	41.168	23.425	23.425	41.168	17.742	41.168	64.593	23.425	23.425	23.425	29.108	23.425	23.425	23.425	23.425	23.425	23.425
321	120.89	58.616	46.992	45.016	60.825	126.27	42.808	72.015	106.04	96.933	106.04	113.95	45.016	81.124	60.825	81.124	51.266	67.075	90.233	139.67	118.92	74.425	62.801	60.825	76.633	142.08
322	59.043	42.367	73.751	57.075	57.075	42.367	42.367	67.157	42.367	42.367	42.367	42.367	55.316	42.367	55.316	58.048	42.367	42.367	42.367	42.367	42.367	42.367	73.751	57.075	57.075	42.367
323	77.693	91.26	91.26	118.39	118.39	91.26	91.26	102.62	91.26	109	91.26	91.26	122.57	85.577	122.57	132.43	104.83	104.83	91.26	96.942	104.83	91.26	91.26	118.39	118.39	91.26
324	284.34	203.17	235.24	246.17	260.2	279.27	189.15	269.24	245.25	261.28	245.25	263.29	246.2	229.21	260.23	289.29	219.17	233.2	231.23	318.31	295.27	217.2	249.27	260.2	274.23	293.3
325	764	338	404	568	681	887	286	767	556	696	568	582	568	445	681	962	404	472	472	1263	1024	424	500	686	812	1042
326	25	16	18	20	21	25	13	22	22	21	20	20	20	21	21	23	18	20	20	32	27	17	19	21	22	26
327	88	60	64	74	78	92	54	88	74	76	74	72	74	76	78	86	64	70	70	114	98	64	68	78	82	96
328	-3.5829	1.3855	2.6527	4.0301	-0.8792	-0.6055	-2.878	0.057	-0.0806	0.6408	2.3046	-3.571	0.6944	-1.1338	0.5823	6.9965	1.2532	-0.9387	2.2284	2.6051	0.6621	-0.5272	-1.4527	4.359	3.0014	-1.3361
329	-3.678	-2.7102	-0.1924	2.5006	0.9275	-1.0318	-2.6818	-0.5552	4.627	-3.2638	0.2632	-0.8548	-2.2391	1.6196	-0.1046	0.028	3.0301	-1.0497	1.8165	-0.3027	1.7814	-2.173	-1.8754	-1.0457	-0.5777	3.4312
330	-0.5849	0.714	2.7636	-0.5093	-1.6863	-0.0425	-1.6867	2.1424	0.6496	-1.3302	-2.4032	0.009	-0.2403	0.0794	1.9027	2.0075	-0.1193	-0.2865	1.8223	1.4212	0.7424	0.3042	-0.7491	2.4842	0.5388	0.3566
331	0	0	0	0	0	0	0	0	0	0	0	0	0	0	0	0	0	0	0	0	0	0	0	0	0	0
332	0	0	0	0	0	0	0	0	0	0	0	0	0	0	0	0	0	0	0	0	0	0	0	0	0	0
333	3274.3	1717.5	1341.2	1671.8	1769.6	1831.8	1225.6	1283.5	1954.7	1957.9	1739.4	1454.5	2032.4	1457.8	2516.9	3190.1	1722.8	1692.7	1333.6	3366.9	3387.3	1545.4	2186.9	2794.3	3004.3	2633.7
334	780.64	277.74	430.42	401.32	799.56	868.08	254.04	790.8	359.22	917.56	478.56	1046	439.93	350.68	501.67	712	334.29	268.25	447.12	732.91	554.7	293.52	464.28	430.25	610.95	1103.7
335	121.72	60.258	254.53	214.31	135.32	258.88	95.902	247.65	222.44	225.87	294.4	90.587	83.471	152.27	123.04	123.84	135.95	149.88	191.63	308.19	178.19	93.77	72.701	157.27	178.58	174.92
336	21.342	12.566	8.1986	9.7253	8.7142	7.7322	11.84	8.511	8.7978	13.503	8.6491	6.2906	17.245	5.7017	5.2504	11.22	11.716	5.9947	7.9405	18.611	15.79	9.957	8.4942	17.658	19.321	12.616
337	-84055	-70729	-75213	-88130	-91737	-89695	-67136	-89728	-81498	-86829	-81503	-82361	-86138	-77243	-89773	-96402	-78131	-81699	-77874	-100668	-97094	-74323	-78805	-91715	-95316	-93299
338	-518842	-349205	-396080	-471440	-511889	-565732	-314075	-549642	-495785	-531258	-485195	-497105	-470356	-437883	-518378	-624550	-397042	-453099	-451674	-677992	-603094	-397355	-432942	-508272	-548879	-594545
339	-87.749	-259.82	-255.52	-320.44	-325.13	-223.44	-253.88	-197.05	-266.86	-189.18	-271.73	-228.36	-267.35	-230.15	-315.9	-200.82	-312.4	-292.31	-228.79	-187.13	-264.61	-265.35	-260.05	-319.2	-332.67	-240.99
340	-7.858	-5.0348	-5.3703	-2.7197	-2.6766	-4.9892	-5.1215	-5.2303	-5.1384	-8.6292	-5.1327	-5.612	-4.8721	-5.7056	-5.6156	-9.9878	-5.2194	-6.0994	-5.6082	-4.7702	-5.2902	-4.9632	-5.1824	-1.4599	-2.1153	-5.0483
341	7.858	5.0348	5.3703	2.7197	2.6766	4.9892	5.1215	5.2303	5.1384	8.6292	5.1327	5.612	4.8721	5.7056	5.6156	9.9878	5.2194	6.0994	5.6082	4.7702	5.2902	4.9632	5.1824	1.4599	2.1153	5.0483
342	-1.5919	3.2867	3.5378	6.939	6.0739	3.3335	3.6632	3.4649	4.0877	-0.5405	3.5827	2.7743	2.5993	3.1437	3.7372	0.4875	3.2387	2.9569	2.9015	1.9629	2.1653	3.4376	3.3454	5.4325	5.3896	2.9463
343	496.64	389.29	395.1	398.38	422.61	466.22	360.46	435.53	449.67	477.74	452.47	443.71	407.42	397.53	435.6	495.04	394.41	395.1	419.15	524.68	484.72	392.77	421.92	443.1	466.14	502.16
344	216.68	103.64	103.63	106.42	106.78	195.94	114.3	193.62	92.24	171.07	95.233	121.14	157.58	79.616	147.08	201.63	126.43	101.16	99.943	211.93	199.15	89.517	117.68	108.54	117.86	188.72
345	203.42	153.79	204.08	210.97	208.72	195.68	160.95	157.63	134.83	146.7	143.96	172	166.13	142.08	187.17	146.78	175.56	146.34	129.13	224.68	207.46	137.66	180.42	217.63	222.22	204.84
346	236.28	138.48	99.18	91.976	117	238.92	99.805	168.89	230.04	162.6	221.5	156.98	93.633	181.87	113.69	151.33	98.692	152.2	195.98	275	228.72	173.48	152.09	129.76	137.81	284.06
347	260.36	250.81	295.92	306.41	305.61	227.3	260.66	266.64	219.62	315.14	230.97	286.73	313.79	215.65	321.91	343.72	295.72	242.91	223.18	249.68	255.99	219.28	269.83	313.34	328.33	218.09
348	1221.9	503.58	546.01	613.52	615.57	1127.2	555.38	1108.7	448.19	1148.2	462.74	644.32	981.57	381.28	916.17	1741.5	700.32	560.35	485.62	1291.3	1221.8	434.97	620.03	625.75	679.44	1085.7
349	1147.1	901.06	1279.4	1638.2	1620.7	1321.5	943.01	1060.2	789.96	984.63	843.45	1086.9	1201	822.51	1353.1	1267.8	1148	956.91	756.55	1593.8	1480.2	806.53	1131.1	1689.9	1725.6	1383.3
350	13.263	50.151	100.45	104.55	101.95	0.2557	46.652	35.992	42.589	24.374	48.725	50.863	8.5522	62.466	40.09	54.849	49.13	45.175	29.182	12.747	8.3062	48.14	62.744	109.09	104.37	16.124
351	74.791	397.47	733.36	1024.6	1005.2	194.21	387.64	48.462	341.77	163.6	380.71	442.54	219.4	441.23	436.89	473.73	447.7	396.56	270.92	302.57	258.41	371.57	511.02	1064.2	1046.1	297.6
352	1.1101	1.1838	1.2316	1.283	1.2578	1.1204	1.2272	1.1946	1.0888	1.087	1.0961	1.158	1.2349	1.1777	1.1992	1.1384	1.2427	1.2178	1.1123	1.1313	1.1506	1.1607	1.21	1.2281	1.1942	1.0949
353	5.1679	3.1265	3.8355	4.7701	2.1158	1.1971	4.2801	2.2139	4.6731	3.5823	3.3401	3.6719	2.3566	1.9786	1.9926	7.2789	3.2812	1.4371	3.4038	2.9829	2.0403	2.2566	2.4877	5.1249	3.1036	3.6994
354	47.571	-14.488	-12.53	-7.3535	-32.162	13.779	-17.911	-17.284	5.0238	-22.645	-7.6366	3.2964	-38.514	-14.488	-27.547	-104.75	-16.202	-21.104	8.4664	0.3683	-0.7075	-25.454	-24.143	-4.0261	-0.1942	3.8294
355	6.3425	6.0316	8.1765	9.6444	14.41	9.9696	6.2501	7.6581	13.605	7.3616	8.9865	5.8197	6.3884	10.792	12.05	25.108	4.2963	6.8524	9.5818	7.4273	8.2232	7.1432	8.2457	12.146	16.01	7.2318
356	0.3978	-65.588	-70.559	-86.133	-120.04	-55.536	-64.708	-93.482	-55.455	-74.021	-64.817	-39.237	-124.08	-101.91	-103.96	-202.73	-74.276	-118.63	-34.101	-80.602	-100.26	-109.52	-110.65	-68.555	-68.67	-67.486
357	32.356	-27.597	-28.577	-42.683	-57.484	-5.5787	-27.455	-48.905	-12.098	-32.258	-20.889	-11.054	-69.348	-50.006	-51.883	-141.84	-28.11	-53.314	-5.5424	-27.321	-37.064	-50.941	-51.828	-23.473	-25.434	-11.2
358	0.0478	0.0739	0.1678	2.4767	0.0691	0.192	0.1793	2.1355	0.4232	0.0544	0.223	0.0415	2.2677	1.7342	0.2285	0.205	0.0352	1.9547	0.0818	2.555	2.0139	1.5897	1.7507	0.3121	0.2293	0.0849
359	-97.506	-102.64	-127.31	-246.41	-208.36	-121.45	-128.56	-127.72	-145.18	-116.6	-127.17	-149.22	-93.587	-96.776	-93.746	-77.753	-121.59	-98.792	-151.59	-118.07	-116.24	-98.576	-88.815	-215.48	-183.08	-92.656
360	-0.1085	0.0841	-0.0142	-0.1423	0.1976	0.606	-0.0677	0.1329	0.3724	0.2452	-0.0938	0.452	0.0453	0.1181	0.4457	-0.208	0.2094	0.4971	0.4191	-0.8373	0.0502	0.4118	0.5602	-0.0265	0.7138	0.293
361	3.8838	4.1914	4.2494	6.2425	6.3962	5.6641	3.3486	5.7699	5.7352	4.6729	4.9238	3.0542	6.0588	5.1707	5.4241	7.9953	4.433	6.6264	3.9258	7.2888	7.6877	6.2766	6.031	4.9002	5.0016	5.2524
362	0.4355	0.0024	0.004	0.0057	0.0049	0.0045	0.0025	0.1699	0.0063	0.0093	0.0125	0.0087	0.0035	0.0024	0.0052	0.0076	0.0033	2.3014	0.0101	0.0173	0.0045	0.0043	1.8386	0.0046	0.0066	0.0053
363	5.0498	2.7277	3.4679	17.108	4.2486	2.926	-0.1666	15.924	-3.0133	-2.7208	-0.787	4.9827	16.074	17.703	6.1882	3.989	2.9344	16.279	0.0003	11.256	18.381	10.066	11.097	2.1149	3.2851	2.1674
364	31.958	37.991	41.982	43.45	62.556	49.957	37.253	44.577	43.357	41.763	43.928	28.183	54.735	51.904	52.081	60.896	46.167	65.311	28.559	53.281	63.198	58.578	58.825	45.083	43.236	56.286

	E	E	E	E	E	E	E	E	E	E	E	E	E	E	E	F	F	F	F	F	F	F	F	F	F	F	F	F	F
	G	H	I	K	L	M	N	P	Q	R	S	T	V	W	Y	A	C	D	E	F	G	H	I	K	L	M	N	P	Q
No.	EG	EH	EI	EK	EL	EM	EN	EP	EQ	ER	ES	ET	EV	EW	EY	FA	FC	FD	FE	FF	FG	FH	FI	FK	FL	FM	FN	FP	FQ
281	77.778	100.21	77.778	78.884	77.778	97.539	97.996	77.641	97.996	91.217	85.546	85.546	77.778	152.45	136.77	105.43	131.79	141.26	141.26	168.91	105.43	127.85	105.43	106.53	105.43	125.19	125.64	105.29	125.64
282	69.077	91.505	69.077	70.182	69.077	88.838	89.295	68.94	89.295	82.516	76.844	76.844	69.077	85.823	76.844	41.943	68.306	69.077	69.077	41.943	41.943	64.372	41.943	43.049	41.943	61.704	62.161	41.806	62.161
283	149.07	180.11	149.07	189.22	149.07	200.23	199.47	153.5	199.47	234.47	182.11	172.06	149.07	174.43	167.16	107.22	144.26	149.07	149.07	107.22	107.22	138.27	107.22	147.38	107.22	158.38	157.63	111.66	157.63
284	131.74	179.88	202.01	216.7	202.01	187.39	164.85	165	182.08	232.39	151.23	168.47	184.78	166.95	170.5	147.06	142.51	138.76	155.99	154.07	129.83	177.97	200.1	214.79	200.1	185.48	162.94	163.08	180.17
285	79.989	88.608	79.989	119.04	79.989	111.39	110.18	84.562	110.18	151.96	105.26	95.218	79.989	88.608	90.313	65.28	75.957	79.989	79.989	65.28	65.28	73.899	65.28	104.33	65.28	96.681	95.468	69.853	95.468
286	5	6	5	6	5	6	5	5	6	7	5	5	5	7	7	5	5	6	6	7	5	6	6	7	6	6	6	5	6
287	0	0	0	0	0	0	0	0	0	0	0	0	0	0	0	0	1	0	0	0	0	0	0	0	0	0	0	0	0
288	0	1	0	0	0	0	0	1	0	0	0	0	0	2	1	1	1	1	1	2	1	2	1	1	1	1	1	2	1
289	0.1865	0.1582	0.1865	0.135	0.1865	0.1703	0.1454	0.1892	0.1454	0.1389	0.1636	0.1636	0.1865	0.1487	0.1477	0.1869	0.1723	0.1575	0.1575	0.1578	0.1869	0.1586	0.1869	0.1352	0.1869	0.1707	0.1457	0.1897	0.1457
290	0.1536	0.1338	0.1536	0.1341	0.1536	0.1424	0.1245	0.1555	0.1245	0.112	0.1376	0.1376	0.1536	0.1269	0.1261	0.1857	0.1712	0.1333	0.1333	0.1568	0.1857	0.1575	0.1857	0.1579	0.1857	0.1696	0.1448	0.1884	0.1448
291	-5.0069	-4.6725	-3.5922	-5.2261	-3.5922	-3.8852	-5.7629	-4.1321	-5.3728	-7.1947	-5.646	-5.2575	-3.9823	-2.9143	-3.69	-1.9058	-1.9959	-3.7857	-3.3956	-0.6831	-2.2943	-1.96	-0.8796	-2.5135	-0.8796	-1.1726	-3.0503	-1.4195	-2.6602
292	136.27	136.27	136.27	169.17	136.27	136.27	169.17	118.26	169.17	218.85	161.66	161.66	136.27	136.27	161.66	93.591	93.591	136.27	136.27	93.591	93.591	93.591	93.591	126.49	93.591	93.591	126.49	75.581	126.49
293	8.1868	24.972	8.1868	8.1868	8.1868	8.1868	13.446	11.311	13.446	10.086	8.1868	8.1868	8.1868	8.1868	8.1868	6.7228	6.7228	8.1868	8.1868	6.7228	6.7228	23.508	6.7228	6.7228	6.7228	6.7228	11.982	9.8471	11.982
294	75.569	96.336	79.551	79.551	79.551	79.551	103.41	79.551	103.41	79.551	79.551	86.019	79.551	96.336	79.551	55.688	55.688	79.551	79.551	55.688	51.706	72.474	55.688	55.688	55.688	55.688	79.551	55.688	79.551
295	18.44	0	0	18.44	0	17.234	0	18.44	0	18.44	20.926	0	0	0	0	0	17.234	0	0	0	18.44	0	0	18.44	0	17.234	0	18.44	0
296	0	2.7567	4.4108	0	4.4108	0	0	0	0	0	0	0	4.4108	3.1856	3.1856	3.1856	3.1856	3.1856	3.1856	6.3712	3.1856	5.9423	7.5964	3.1856	7.5964	3.1856	3.1856	3.1856	3.1856
297	0	18.868	0	0	0	31.691	0	0	0	0	0	0	0	18.868	18.868	18.868	18.868	18.868	18.868	37.737	18.868	37.737	18.868	18.868	18.868	50.56	18.868	18.868	18.868
298	0	34	0	0	0	0	0	0	0	0	0	0	0	92.504	70.573	88.216	88.216	88.216	88.216	176.43	88.216	122.22	88.216	88.216	88.216	88.216	88.216	88.216	88.216
299	37.737	37.737	56.605	94.342	56.605	56.605	56.605	75.474	75.474	75.474	37.737	37.737	37.737	37.737	37.737	0	46.107	18.868	37.737	0	0	0	18.868	56.605	18.868	18.868	18.868	37.737	37.737
300	0	0	66.652	0	66.652	32.22	0	0	0	0	0	33.326	66.652	0	5.2434	33.326	0	0	0	0	0	0	66.652	0	66.652	32.22	0	0	0
301	3.8934	6.0146	5.731	5.9461	5.731	6.0855	5.1666	5.0659	5.6283	6.6211	4.4941	4.9536	5.2693	7.9858	6.9666	5.9434	6.7614	6.3384	6.8001	8.3906	5.4839	7.6052	7.3215	7.5366	7.3215	7.676	6.7571	6.6564	7.2188
302	156.95	156.95	156.95	156.95	156.95	156.95	180.82	156.95	180.82	156.95	156.95	156.95	156.95	156.95	156.95	90.408	90.408	156.95	156.95	90.408	90.408	90.408	90.408	90.408	90.408	90.408	114.27	90.408	114.27
303	0	16.786	0	0	0	0	0	3.1243	0	31.672	25.385	25.385	0	0	25.385	0	0	0	0	0	0	16.786	0	0	0	0	0	3.1243	0
304	50.908	67.693	55.319	83.805	55.319	50.908	50.908	32.897	50.908	68.919	50.908	50.908	55.319	67.693	50.908	50.908	50.908	50.908	50.908	50.908	50.908	67.693	55.319	83.805	55.319	50.908	50.908	32.897	50.908
305	3.982	10.721	7.9639	7.9639	7.9639	7.9639	7.9639	7.9639	7.9639	7.9639	7.9639	14.433	7.9639	11.15	16.393	11.15	11.15	11.15	11.15	14.335	7.1675	13.906	11.15	11.15	11.15	11.15	11.15	11.15	11.15
306	0	0	0	0	0	0	32.897	0	32.897	32.897	0	0	0	4.7171	0	0	0	0	0	0	0	0	0	0	0	0	32.897	0	32.897
307	37.737	90.605	56.605	94.342	56.605	56.605	56.605	75.474	75.474	75.474	37.737	37.737	37.737	144.39	127.18	107.08	107.08	125.95	144.82	214.17	107.08	159.95	125.95	163.69	125.95	125.95	125.95	144.82	144.82
308	26.626	8.1868	8.1868	26.626	8.1868	25.42	13.446	26.626	13.446	28.525	29.113	8.1868	8.1868	8.1868	8.1868	6.7228	23.956	8.1868	8.1868	6.7228	25.162	6.7228	6.7228	25.162	6.7228	23.956	11.982	25.162	11.982
309	0	0	66.652	0	66.652	63.911	0	0	0	0	0	33.326	66.652	0	0	33.326	46.107	0	0	0	0	0	66.652	0	66.652	63.911	0	0	0
310	137	165.68	137	164.64	137	137	180.09	128.21	180.09	200.64	157.23	157.23	137	152.79	157.23	96.87	135.67	137	137	96.87	96.87	125.55	96.87	124.51	96.87	96.87	139.96	88.08	139.96
311	0.5647	0.469	0.4845	0.4681	0.4845	0.4845	0.4845	0.5226	0.4681	0.4388	0.5212	0.5021	0.5021	0.4257	0.4395	0.5226	0.5033	0.469	0.4537	0.4389	0.5436	0.4531	0.469	0.4537	0.469	0.469	0.469	0.5023	0.4537
312	4.7004	5.3219	5.0875	5.1699	5.0875	5.0875	5.0875	5.0875	5.1699	5.3219	4.9069	5	5	5.6439	5.4594	5.0875	5.1699	5.3219	5.3923	5.585	5	5.5236	5.3219	5.3923	5.3219	5.3219	5.3219	5.3219	5.3923
313	3.0203	3.293	3.1423	3.3343	3.1778	3.2339	3.1778	3.0181	3.2811	3.4959	3.0576	3.0711	3.0711	3.4701	3.4432	3.1394	3.1732	3.2814	3.3812	3.4564	3.128	3.3915	3.2499	3.4262	3.2814	3.331	3.2814	3.1323	3.3812
314	7.2753	8.3428	8.0406	8.1871	8.0375	8.033	8.0375	7.8714	8.1921	8.4712	7.6856	7.8695	7.8695	8.8727	8.6151	7.8548	8.0284	8.3417	8.4809	8.7463	7.6687	8.6175	8.3445	8.4765	8.3417	8.3378	8.3417	8.1943	8.4809
315	209.52	280.09	279.79	295.59	279.79	284.93	262.84	242.64	280.08	323.61	236.78	254.01	262.56	319.4	307.28	252.49	274.29	280.02	297.25	322.98	235.25	305.83	305.53	321.32	305.53	310.66	288.58	268.37	305.81
316	231.54	335.2	329.26	347.81	329.26	327.21	296.25	292.55	320.68	371.93	264.49	288.92	304.83	416.29	379.46	316.78	338.1	346.51	370.94	431.75	292.35	396	390.06	408.62	390.06	388.02	357.05	353.35	381.48
317	67.835	67.835	67.835	67.835	67.835	67.835	81.402	67.835	81.402	67.835	67.835	67.835	67.835	67.835	67.835	40.701	40.701	67.835	67.835	40.701	40.701	40.701	40.701	40.701	40.701	40.701	54.268	40.701	54.268
318	54.268	54.268	54.268	54.268	54.268	54.268	54.268	54.268	54.268	54.268	54.268	54.268	54.268	54.268	54.268	27.134	27.134	54.268	54.268	27.134	27.134	27.134	27.134	27.134	27.134	27.134	27.134	27.134	27.134
319	17.742	17.742	17.742	35.485	17.742	17.742	17.742	17.742	17.742	58.91	17.742	17.742	17.742	17.742	17.742	17.742	17.742	17.742	17.742	17.742	17.742	17.742	17.742	35.485	17.742	17.742	17.742	17.742	17.742
320	23.425	23.425	23.425	41.168	23.425	23.425	41.168	17.742	41.168	64.593	23.425	23.425	23.425	29.108	23.425	23.425	23.425	23.425	23.425	23.425	23.425	23.425	23.425	41.168	23.425	23.425	41.168	17.742	41.168
321	58.616	87.824	121.85	112.74	121.85	129.75	60.825	96.933	76.633	96.933	67.075	82.883	106.04	155.47	134.73	139.87	128.24	126.27	142.08	207.52	124.06	153.27	187.29	178.18	187.29	195.2	126.27	162.38	142.08
322	42.367	67.157	42.367	42.367	42.367	42.367	55.316	42.367	55.316	58.048	42.367	42.367	42.367	42.367	42.367	27.658	59.043	42.367	42.367	27.658	27.658	52.449	27.658	27.658	27.658	27.658	40.608	27.658	40.608
323	91.26	102.62	91.26	109	91.26	91.26	122.57	85.577	122.57	132.43	104.83	104.83	91.26	96.942	104.83	64.126	64.126	91.26	91.26	64.126	64.126	75.491	64.126	81.868	64.126	64.126	95.435	58.443	95.435
324	203.17	283.26	259.28	275.3	259.28	277.32	260.23	243.24	274.25	303.32	233.2	247.23	245.25	332.34	309.3	236.27	268.34	279.27	293.3	312.37	222.24	302.33	278.35	294.38	278.35	296.39	279.3	262.31	293.32
325	363	909	673	828	686	701	686	548	812	1122	500	578	578	1457	1193	582	676	902	1052	1326	505	1169	887	1070	902	919	902	736	1052
326	14	23	23	22	21	21	21	22	22	24	19	21	21	33	28	21	23	25	26	30	18	27	27	26	25	25	25	26	26
327	58	92	78	80	78	76	78	80	82	90	68	74	74	118	102	78	82	92	96	110	72	106	92	94	92	90	92	94	96
328	1.4347	1.9124	-1.7634	5.7095	-2.0958	-1.4823	-0.0273	-1.8138	0.1812	7.2114	-1.4193	-0.0009	-0.5592	-3.7933	-2.842	3.201	4.7332	9.7178	3.9378	-2.6864	2.5782	3.4343	-1.6479	-3.1986	2.9349	-1.3809	3.9553	2.8413	-2.5036
329	2.4384	-2.4018	0.2978	1.1741	1.7336	-0.8869	0.492	-2.209	2.9449	0.105	0.6651	3.8642	4.6355	4.1538	-1.157	4.7232	-2.9995	2.8981	-2.9141	-0.0369	-0.8547	3.7243	4.9468	-2.6006	-1.4471	4.7823	5.2647	-4.9536	-0.6301
330	-0.1044	2.191	-1.2267	-0.9879	-1.616	0.6283	-1.6114	-0.4074	0.2062	-0.7884	-0.4203	-1.3796	-1.4776	-1.2692	-0.2646	0.0423	0.3961	0.9319	-0.7987	0.0128	0.074	-0.0751	1.424	0.208	0.5223	-1.7296	0.0844	-0.6015	0.7218
331	0	0	0	3E-14	0	0	0	0	0	0	0	0	0	0	0	0	0	0	0	0	0	6E-14	0	4E-14	0	0	0	0	0
332	0	0	0	3E-14	0	0	0	0	0	0	0	0	0	0	0	0	0	0	0	0	0	6E-14	0	4E-14	0	0	0	0	0
333	1814.6	2613.7	2252	2824	1830.6	2795.8	1993.5	1833.4	2995.2	3831.6	1718.6	2143.2	1863.7	4523.1	4462.1	1569.4	3385	3656.4	2264.7	1928.8	1836	3517.1	2111.4	3566.8	3327.2	3012.2	2386.1	1898.9	3674
334	314.94	732.23	554.56	610.77	517.16	769.11	396.82	436.6	688.98	976.35	414.96	410.98	453.87	787.65	637.53	494.09	290.87	515.75	837.85	1552.4	361.48	564.04	692.2	672.15	521.97	640.71	807	539.24	645.64
335	34.567	173.33	204.28	233.42	397.03	169.39	282.49	107.1	90.586	212.06	135.44	115.52	155.88	221.14	189.75	138.9	196.88	132.25	292.27	321.89	136.73	376.28	304.63	266.08	190.89	260.46	189.69	195.64	309.12
336	13.791	4.834	15.613	17.376	8.193	6.9826	8.2961	6.4335	12.224	10.833	14.901	9.924	10.403	9.5425	21.498	18.554	15.774	18.556	11.153	4.9413	5.4371	20.342	19.665	24.155	6.3604	16.988	21.952	15.76	4.5622
337	-70732	-93329	-85082	-90425	-85101	-85993	-89772	-80863	-93364	-99993	-81708	-85311	-81498	-104244	-100677	-72186	-76681	-89702	-93299	-91213	-68650	-91189	-82955	-88271	-83017	-83848	-87625	-78722	-91272
338	-344428	-564309	-528679	-584646	-533876	-519097	-524656	-480308	-553205	-713081	-441612	-494201	-493233	-734497	-639765	-449028	-484884	-550522	-611056	-671336	-388750	-619555	-587552	-623309	-562004	-579159	-565128	-534916	-588282
339	-263.36	-210.87	-265.23	-188.4	-284.15	-273.91	-314.39	-262.98	-320	-204.91	-303.18	-318.12	-267.7	-176.87	-270.25	-53.761	-60.528	-231.2	-241.39	-81.301	-106.56	3.6664	-47.493	30.033	-125.64	-55.638	-92.65	-47.588	-153.09
340	-5.069	-5.0861	-4.7647	-8.3033	-5.3037	-5.1642	-5.5323	-5.2477	-5.3614	-9.3275	-4.6699	-5.1947	-4.8643	-5.8481	-4.7842	-7.7117	-8.097	-4.812	-4.9867	-9.6264	-9.85	-7.6394	-7.83	-11.277	-9.8019	-8.2027	-7.9786	-7.9696	-9.9656
341	5.069	5.0861	4.7647	8.3033	5.3037	5.1642	5.5323	5.2477	5.3614	9.3275	4.6699	5.1947	4.8643	5.8481	4.7842	7.7117	8.097	4.812	4.9867	9.6264	9.85	7.6394	7.83	11.277	9.8019	8.2027	7.9786	7.9696	9.9656
342	3.1666	3.1859	3.1167	-0.9212	3.8897	3.0993	3.3675	3.899	2.9583	-0.7453	2.9567	3.6787	3.9895	2.1635	2.0325	-0.8995	-0.7989	2.0356	2.6686	0.1763	-0.0268	-1.2067	-1.5124	-4.2798	0.0129	-1.3642	-1.5001	-0.4949	-0.0832
343	387.01	484.81	464.36	491.26	466.44	481.79	425.54	421.46	470.01	538.04	410.41	429.25	442.79	549.5	524.03	444.68	487.2	500	496.35	537	429.06	540.8	509.52	548.55	527.31	525.13	486.49	467.54	532.25
344	111.91	211.46	91.381	170.53	84.435	131.55	136.2	77.582	147.17	237.57	128.21	108.07	90.696	209.24	206.08	175.13	208.36	199.56	166.18	281.32	203.93	276.49	157.43	227.59	167.39	184.56	238.94	160.33	238.56
345	158.34	164.77	134.56	148.92	141.82	186.13	167.52	131.06	176.87	144.85	168.72	149.83	133.02	233.5	221.59	141.95	207.1	232.68	200.6	199.95	165.16	186.33	152.54	149.89	149.49	219.17	183.74	143.29	202.62
346	129.75	196.82	242.7	177.77	247.07	171.48	136.35	219.96	158.74	159.55	119.63	176.85	224.3	279.39	247.91	291.34	266.4	252.56	301.46	388.24	252.8	332.29	369.36	349.52	392.58	293.9	232.19	325.81	279.57
347	257.25	287.98	221.66	313.48	219.37	310.32	289.18	201.5	311.26	378.49	290.78	252.4	218.48	270.12	276.12	153.34	220.81	247.44	194.89	148.76	176.26	208.51	140.17	199.03	134.73	231.22	254.31	141.72	252.68
348	543.76	1210.8	444.02	1144.6	410.27	699.7	848.39	371.54	916.74	2051.9	710.18	598.58	440.69	1274.9	1264.3	848.88	1095.3	1148.1	956.02	1615	988.43	1579.9	763.05	1524.8	811.35	979.46	1485.5	765.88	1483.1
349	927.7	1108.2	788.36	999.54	830.93	1176.1	1211	758.73	1278.6	1251.1	1103.3	979.74	779.36	1656.5	1581	688.05	1088.7	1571.3	1354.6	1147.9	800.53	1064.7	739.36	854.38	724.6	1163.1	1142.3	684.49	1259.7
350	46.43	46.693	43.175	21.607	57.385	54.579	31.319	53.482	29.696	92.714	40.509	41.763	42.324	24.26	15.506	33.18	1.2553	33.116	34.42	81.363	38.768	90.165	4.8871	77.697	17.897	34.611	55.207	17.038	35.941
351	383.94	102.6	344.34	145.02	420.66	476.43	362.6	387.19	361.84	800.77	393.1	381.16	338.67	381.55	316.72	160.83	6.5993	423.19	398.62	467.11	187.91	515.21	23.688	670.46	86.746	183.68	343.22	81.388	223.45
352	1.1864	1.1538	1.0714	1.0744	1.0759	1.1279	1.2097	1.14	1.1683	1.108	1.1944	1.1662	1.0858	1.1018	1.1268	1.0212	1.0728	1.1006	1.0964	1.0097	1.0508	1.0484	0.9728	0.9949	0.9853	1.0391	1.065	1.0332	1.0671
353	2.8311	3.7718	2.1687	5.912	3.1637	1.8381	1.6851	2.8872	2.9576	7.2551	1.6228	4.1031	4.8974	5.7666	3.0799	5.7058	5.6175	10.183	4.9635	2.6867	2.7172	5.0666	5.405	4.1277	3.3137	5.2696	6.5855	5.7422	2.6807
354	-21.084	17.725	11.595	-11.63	-27.988	-9.2536	-36.974	-30.948	-25.993	-67.677	-1.6309	-8.3749	2.1733	39.186	14.97	52.075	51.37	54.15	18.498	35.919	9.1038	48.184	57.168	28.271	30.436	63.476	41.975	59.044	20.752
355	6.0349	7.0069	13.781	17.662	15.857	12.719	9.146	15.269	13.283	11.685	6.1493	9.3337	11.194	8.8004	8.0784	6.1112	4.9479	8.7335	12.651	9.5429	7.7149	7.8076	8.0065	20.125	6.6634	12.631	7.3342	11.359	5.8971
356	-68.591	-26.167	-62.466	-82.188	-118.86	-75.337	-118.25	-116.37	-94.658	-141.13	-68.872	-79.065	-58.32	-2.3505	-72.417	14.841	8.0371	6.1061	-67.659	-54.339	-55.947	3.8039	12.206	-56.004	-49.756	11.866	-6.6687	11.782	-64.383
357	-28.682	2.7924	-17.932	-37.508	-51.851	-30.878	-60.636	-52.103	-45.613	-96.974	-27.609	-24.756	-12.756	26.067	-14.721	40.979	38.055	37.927	-5.4242	12.499	-7.074	33.258	43.426	0.461	4.8704	38.016	22.807	39.595	-9.8495
358	0.0355	0.0994	1.8571	0.1625	0.0852	0.2594	1.2338	0.0202	0.0695	1.7466	1.9239	0.2904	0.2184	0.2769	2.0976	0.1152	0.1872	0.2634	0.1463	0.1353	0.0616	0.1451	0.3787	0.3121	1.6946	0.0265	0.2883	0.0167	1.948
359	-95.129	-178.83	-103.11	-127.64	-98.423	-74.351	-84.743	-96.688	-82.792	-104.51	-101.26	-119.55	-141.14	-129.74	-91.872	-109.15	-94.004	-197.46	-140.14	-61.139	-56.909	-105.68	-102.87	-121.86	-56.694	-104.12	-115.93	-105.13	-63.132
360	0.2009	0.4801	0.5829	0.2135	0.7319	0.3386	0.5441	0.0873	0.0914	0.1949	0.3429	0.6646	0.5246	-0.4251	0.0605	0.5	0.4374	-0.2836	1.1314	0.1099	0.1709	-0.0876	0.177	0.3064	0.1553	0.1517	-0.1338	0.1027	0.0965
361	3.2318	3.8337	6.4486	5.128	6.423	4.7677	5.6286	6.4274	4.8823	7.2651	5.3395	5.4274	5.395	4.222	7.363	3.7449	4.2533	3.9453	7.0795	7.8346	5.1928	4.2399	4.9316	6.6495	7.565	3.9398	4.0175	4.0067	6.7919
362	0.003	2.3991	0.0065	26.515	0.003	17.967	0.0029	0.0042	0.0058	0.0145	0.0045	0.4377	0.0052	0.0189	0.0157	0.0036	0.0056	0.0058	13.153	0.0182	0.0031	0.0133	0.0201	2.2844	0.0093	0.0121	1.2353	0.0046	0.0287
363	-1.905	3.5126	6.8575	2.7116	0.7659	3.5399	7.1096	-0.6483	1.294	8.4058	12.222	0.665	-2.4029	0.2448	12.092	0.6248	3.4892	3.5646	2.914	5.7965	3.0376	2.821	0.2484	0.4172	9.4878	8.7111	7.662	3.964	15.868
364	39.91	28.959	44.534	44.68	67.004	44.459	57.618	64.264	49.045	44.161	41.263	54.309	45.565	28.418	57.696	26.138	30.018	31.821	62.235	66.838	48.873	29.454	31.22	56.465	54.627	26.151	29.476	27.814	54.533

Table S12 Amino acid descriptors (2D) used in this study.

	C	D	D	D	D	D	D	D	D	D	D	D	D	D	D	D	D	D	D	D	D	E	E	E	E	E	E	E	E	E	E	E	E	E	E	E	E	E	E	E	E	F	F	F	F	F	F	F	F	F	F	F	F	F	F
	Y	A	C	D	E	F	G	H	I	K	L	M	N	P	Q	R	S	T	V	W	Y	A	C	D	E	F	G	H	I	K	L	M	N	P	Q	R	S	T	V	W	Y	A	C	D	E	F	G	H	I	K	L	M	N	P	Q
No.	CY	DA	DC	DD	DE	DF	DG	DH	DI	DK	DL	DM	DN	DP	DQ	DR	DS	DT	DV	DW	DY	EA	EC	ED	EE	EF	EG	EH	EI	EK	EL	EM	EN	EP	EQ	ER	ES	ET	EV	EW	EY	FA	FC	FD	FE	FF	FG	FH	FI	FK	FL	FM	FN	FP	FQ
365	0.4363	0.2662	0.2623	0.2671	0.2527	0.4203	0.3171	0.4446	0.2051	0.3581	0.2105	0.273	0.3868	0.2003	0.3377	0.4073	0.3206	0.256	0.2384	0.4039	0.4109	0.2279	0.2789	0.245	0.2528	0.3758	0.2892	0.4362	0.1968	0.3471	0.181	0.273	0.3201	0.1841	0.3131	0.4415	0.3124	0.2518	0.2048	0.3808	0.3933	0.3938	0.4277	0.3991	0.3348	0.5239	0.4753	0.5113	0.309	0.4149	0.3174	0.3515	0.4912	0.3429	0.4482
366	0.4096	0.3951	0.5165	0.5296	0.4939	0.4197	0.4465	0.3619	0.2998	0.3071	0.3182	0.3876	0.4078	0.3574	0.4297	0.2965	0.4451	0.3704	0.3081	0.4282	0.428	0.3505	0.4276	0.4912	0.4767	0.4079	0.4091	0.3399	0.2898	0.3031	0.304	0.3863	0.3937	0.311	0.3763	0.2692	0.4111	0.3491	0.3004	0.4249	0.4229	0.3192	0.4251	0.4654	0.4041	0.3724	0.3849	0.3445	0.2994	0.2732	0.2835	0.4174	0.3777	0.3065	0.3807
367	0.4758	0.3557	0.251	0.2309	0.2769	0.5125	0.2769	0.3878	0.5116	0.3404	0.4895	0.3538	0.2298	0.4575	0.261	0.3057	0.2502	0.3852	0.4676	0.5241	0.4719	0.4417	0.3605	0.2928	0.2956	0.5657	0.3353	0.406	0.5227	0.3619	0.5297	0.3559	0.3204	0.5219	0.3377	0.2965	0.2915	0.412	0.5066	0.5084	0.4731	0.6552	0.5468	0.5051	0.6074	0.723	0.5892	0.6144	0.7249	0.6372	0.7445	0.5597	0.4773	0.6969	0.5253
368	0.5242	0.6443	0.749	0.7691	0.7231	0.4875	0.7231	0.6122	0.4884	0.6596	0.5105	0.6462	0.7702	0.5425	0.739	0.6943	0.7498	0.6148	0.5324	0.4759	0.5281	0.5583	0.6395	0.7072	0.7044	0.4343	0.6647	0.594	0.4773	0.6381	0.4703	0.6441	0.6796	0.4781	0.6623	0.7035	0.7085	0.588	0.4934	0.4916	0.5269	0.3448	0.4532	0.4949	0.3926	0.277	0.4108	0.3856	0.2751	0.3628	0.2555	0.4403	0.5227	0.3031	0.4747
369	2.4603	1.2936	1.382	1.54	1.4566	2.4179	1.5407	2.5455	0.9967	2.4034	1.0227	1.4521	2.4092	0.9591	2.1032	3.5179	1.7756	1.4182	1.1586	2.4611	2.5206	1.1074	1.4695	1.4122	1.4576	2.1621	1.405	2.4976	0.9562	2.3299	0.8796	1.4523	1.9937	0.8816	1.9505	3.8136	1.7304	1.3945	0.9953	2.3201	2.4127	1.909	2.2482	2.2962	1.9261	3.0075	2.3037	2.9214	1.4976	2.7798	1.5387	1.8652	3.0535	1.6381	2.7866
370	2.3097	2.3146	3.2381	4.112	3.8351	2.8344	2.6161	2.4343	1.7568	2.061	1.8641	2.4494	2.9478	2.0691	3.1062	2.5609	2.9107	2.4219	1.8049	3.0377	3.0537	2.0535	2.6807	3.8139	3.7018	2.7547	2.3971	2.2859	1.6977	2.0347	1.7814	2.4411	2.8458	1.8002	2.7204	2.3253	2.6882	2.2825	1.7601	3.0145	3.0171	1.5473	2.2347	3.1426	2.7292	2.1377	1.8658	1.9687	1.4511	1.5575	1.3742	2.215	2.348	1.464	2.3668
371	0.06	0.0783	0.2163	0.1438	0.1154	0.2677	0.1336	0.2604	0.1241	0.1263	0.1738	0.1034	0.0769	0.1299	0.0907	0.0533	0.1021	0.1047	0.1749	0.1517	0.057	0.1097	0.0873	0.1016	0.0955	0.1184	0.0732	0.0659	0.1176	0.0939	0.1976	0.0838	0.1948	0.1168	0.0782	0.0755	0.1276	0.1115	0.118	0.078	0.0688	0.1202	0.1031	0.069	0.1319	0.219	0.1334	0.1161	0.1645	0.0861	0.073	0.1206	0.0863	0.1233	0.0906
372	21.695	14.513	13.234	7.5894	7.0408	9.8444	16.037	13.561	10.573	21.091	12.019	9.7491	12.839	14.444	4.5818	8.6029	12.958	8.3855	4.0804	17.659	17.951	2.9514	11.846	25.85	22.315	14.616	15.856	7.7466	15.715	19.29	10.226	9.1882	10.639	7.1142	12.976	6.2079	16.945	11.929	9.8314	13.058	25.983	22.027	21.575	16.55	13.197	5.7289	6.3024	19.053	21.059	24.943	6.8339	18.623	24.994	21.115	3.6857
373	-85079	-70997	-76265	-88462	-92040	-89968	-67383	-90021	-81802	-87135	-81807	-83490	-86471	-77549	-90087	-96760	-78433	-82036	-78145	-100956	-97402	-74582	-79876	-92043	-95663	-93578	-70998	-93631	-85406	-90745	-85391	-87094	-90090	-81158	-93693	-100318	-82027	-85638	-81805	-104565	-101007	-72373	-77649	-89975	-93575	-91431	-68853	-91409	-83187	-88536	-83273	-84860	-87854	-78926	-91546
374	-512932	-345771	-395612	-455082	-502646	-555183	-311246	-535807	-483802	-519537	-477654	-462708	-470327	-426769	-506478	-599402	-392502	-437321	-435446	-672632	-596410	-388734	-427216	-492339	-536476	-585369	-339098	-554858	-519504	-570245	-517924	-507318	-508804	-468195	-540869	-703355	-433926	-479666	-481511	-715044	-626200	-438758	-471780	-539765	-603453	-661515	-382666	-612595	-571051	-607572	-552198	-563823	-556424	-521053	-580689
375	-32.94	-237.69	-227.46	-300.24	-274.88	-202.32	-226.96	-190.93	-232.53	-129	-239.04	-245.76	-223.32	-237.25	-274.59	-185.55	-284.72	-283.86	-178.38	-181.28	-247.65	-220.13	-234.01	-277.9	-295.41	-204.43	-238.92	-198.08	-234.97	-135.94	-219.09	-247.54	-276.99	-242.92	-276.85	-139.87	-274.39	-281.55	-236.08	-187.79	-241.55	-10.836	-6.4753	-208.83	-208.42	-64.56	-94.78	37.026	-15.569	72.307	-102.38	-14.149	-41.186	5.6406	-130.06
376	-7.4318	-4.7794	-4.8789	-2.8884	-0.8671	-4.8345	-4.6914	-4.7087	-4.8742	-7.4607	-4.779	-4.9585	-4.8276	-4.7534	-5.2678	-10.493	-4.9401	-5.1077	-5.3956	-4.7684	-4.783	-3.3927	-4.9051	-1.4467	-1.9887	-4.7922	-4.7407	-4.69	-4.5872	-7.7688	-3.6053	-4.9901	-5.1272	-4.9428	-5.0608	-8.1181	-4.5124	-4.8079	-4.689	-4.8732	-4.5545	-7.2731	-7.6218	-4.7427	-4.6163	-9.5522	-9.7704	-7.2208	-7.4508	-11.168	-9.7158	-7.8866	-7.5836	-7.2376	-9.755
377	7.4318	4.7794	4.8789	2.8884	0.8671	4.8345	4.6914	4.7087	4.8742	7.4607	4.779	4.9585	4.8276	4.7534	5.2678	10.493	4.9401	5.1077	5.3956	4.7684	4.783	3.3927	4.9051	1.4467	1.9887	4.7922	4.7407	4.69	4.5872	7.7688	3.6053	4.9901	5.1272	4.9428	5.0608	8.1181	4.5124	4.8079	4.689	4.8732	4.5545	7.2731	7.6218	4.7427	4.6163	9.5522	9.7704	7.2208	7.4508	11.168	9.7158	7.8866	7.5836	7.2376	9.755
378	-1.9115	3.0464	3.115	6.1687	6.1413	3.0911	3.3325	2.9246	3.8755	-1.9134	3.2675	3.0278	3.1123	3.1781	3.468	0.4479	3.0388	2.984	2.1688	1.7677	1.9365	3.8413	2.9759	4.6523	4.7691	2.4668	2.8211	3.0201	2.941	-1.5941	4.1057	3.1062	2.9942	3.2676	2.745	-0.8157	2.8131	3.1695	3.614	2.2515	1.4749	-1.4979	-1.3572	1.9077	2.2014	-0.053	-0.2627	-1.5125	-1.843	-4.5423	-0.2094	-1.7577	-1.9028	-1.4213	-0.2202
379	0.2202	0.1694	0.3829	0.2969	0.3638	0.4169	0.2364	0.5006	0.2513	0.3975	0.3484	0.4347	0.2117	0.2781	0.207	0.2142	0.2285	0.2132	0.3586	0.2539	0.1859	0.2106	0.197	0.1822	0.2184	0.3414	0.1641	0.2706	0.2704	0.2458	0.3893	0.2577	0.2841	0.2395	0.2116	0.2471	0.2579	0.2061	0.2631	0.1898	0.1622	0.3064	0.1317	0.1553	0.3642	0.5377	0.2267	0.2301	0.3555	0.2211	0.1852	0.2464	0.3118	0.3011	0.2208
380	0.8379	0.891	0.8974	0.9098	0.7415	0.7748	0.8932	0.7381	0.9407	0.7594	0.9169	0.6377	0.8558	0.8903	0.8745	0.8493	0.9036	0.9394	0.8564	0.8964	0.9045	0.8913	0.8569	0.9154	0.8804	0.7521	0.8684	0.833	0.8752	0.8901	0.9489	0.8362	0.9521	0.8548	0.8375	0.841	0.869	0.8843	0.8714	0.8934	0.9122	0.8281	0.9739	0.9081	0.8241	0.6472	0.8978	0.9541	0.8617	0.9041	0.914	0.8959	0.8069	0.8593	0.9222
381	18.412	11.004	11.467	7.2705	6.8993	7.2423	13.545	10.088	12.39	9.1488	10.001	12.221	13.822	6.8869	5.1374	13.058	11.372	5.3324	6.3	16.728	13.973	9.5279	8.1499	16.121	16.951	12.441	11.521	10.207	15.832	15.603	9.7222	6.462	7.788	8.897	9.5883	20.062	13.05	10.731	11.432	22.456	20.547	18.452	12.949	29.259	17.621	5.218	5.6637	16.995	18.662	23.199	6.2682	18.416	21.56	20.649	4.9338
382	-77299	-64462	-68747	-80374	-83836	-82239	-61008	-80828	-74788	-79188	-74802	-75599	-78059	-70628	-81526	-86689	-71241	-74673	-71332	-91812	-89042	-67912	-72188	-83814	-87273	-85713	-64463	-84234	-78235	-82634	-78259	-79104	-81523	-74094	-84969	-90077	-74675	-78133	-74797	-95204	-92482	-66237	-70547	-82197	-85672	-84095	-62847	-82615	-76574	-80924	-76634	-77414	-79842	-72409	-83342
383	-505886	-341877	-387866	-447915	-506447	-558259	-306634	-537689	-477231	-528524	-473682	-477588	-462800	-430378	-507548	-608360	-387826	-443632	-437205	-664880	-592408	-391082	-428025	-501246	-541227	-583908	-338337	-572764	-521472	-579254	-521138	-511843	-517120	-466556	-543197	-660526	-435753	-480776	-480201	-703646	-632005	-442049	-472522	-543473	-617875	-662794	-384021	-612720	-573022	-610987	-553588	-565108	-550434	-520190	-581894
384	-90.306	-266.09	-254.14	-331.34	-350.23	-210.02	-254.86	-220.12	-261.31	-207.58	-276.11	-218.14	-291.21	-253.03	-317.04	-213.5	-313.07	-297.93	-246.31	-210.27	-284.36	-269.39	-248.41	-328.09	-344.64	-243.85	-267.29	-180.52	-263.32	-210.41	-290.57	-281.51	-312.66	-276.04	-317.42	-152.94	-301.8	-315.36	-270.13	-162.13	-277.88	-55.786	-66.569	-171.07	-202.69	-85.472	-110.27	-24.277	-63.822	39.945	-122.8	-50.151	-71.508	-39.098	-147.97
385	-8.2468	-5.3002	-5.5767	-2.7795	-2.9505	-5.8572	-5.2338	-5.3433	-4.6694	-9.2939	-5.1762	-4.9291	-5.3585	-5.9907	-5.9944	-9.9415	-5.3852	-6.3433	-6.1003	-5.241	-5.5955	-5.2322	-6.3422	-1.7598	-2.3014	-5.1002	-5.3503	-5.2586	-4.7611	-9.1111	-5.3925	-5.5406	-5.72	-5.3395	-5.7046	-8.1454	-5.0763	-5.3046	-4.8968	-5.2913	-5.092	-7.8843	-8.6899	-4.4288	-4.6424	-9.794	-10.024	-8.1697	-8.0706	-11.578	-9.9602	-8.228	-8.3379	-7.4328	-9.7649
386	8.2468	5.3002	5.5767	2.7795	2.9505	5.8572	5.2338	5.3433	4.6694	9.2939	5.1762	4.9291	5.3585	5.9907	5.9944	9.9415	5.3852	6.3433	6.1003	5.241	5.5955	5.2322	6.3422	1.7598	2.3014	5.1002	5.3503	5.2586	4.7611	9.1111	5.3925	5.5406	5.72	5.3395	5.7046	8.1454	5.0763	5.3046	4.8968	5.2913	5.092	7.8843	8.6899	4.4288	4.6424	9.794	10.024	8.1697	8.0706	11.578	9.9602	8.228	8.3379	7.4328	9.7649
387	-1.7174	3.4922	3.4935	6.0076	6.2905	2.9236	3.7655	3.0485	3.7436	-0.471	3.7719	1.5536	2.9377	2.883	3.6071	-0.1927	3.3082	2.757	2.7082	1.8288	2.1913	3.7076	2.4918	5.6853	5.8358	2.632	3.4043	1.8782	2.7328	-0.3904	3.8478	2.5678	3.4115	3.7685	3.1974	-2.0064	3.0975	3.5888	3.7248	0.8153	1.9692	-1.4115	-0.9092	0.9345	1.8909	-0.0448	-0.2487	-1.25	-1.9041	-4.8192	-0.2113	-2.0792	-1.8692	-1.5253	-0.3345
388	4176.7	2055.5	2026.2	2287.4	2704.5	2958.7	1575.6	2321.9	2536.4	3101.3	2512.4	2591.1	2555.8	1960.8	3141.6	4026	2193.1	2110.9	1972.4	4408	4120.2	1932.7	2723.8	3381.8	3793.8	3912.3	2164.1	3519.2	3010.8	3668.2	2744.8	3734.3	2672.8	2377.1	3774.8	5020	2269	2669.7	2473.4	5531.9	5289.4	2202.4	3872.7	4304.4	3394.8	3803.1	2334.2	4457.4	3108.2	4505	4040	3913.4	3382.8	2633.8	4628.7
389	893.63	337.95	680.53	615.6	934.76	1125.6	349.8	1038.4	581.64	1143.1	772.86	1087.1	523.4	502.91	624.7	835.79	470.12	418.1	638.72	1040.8	732.83	387.27	522.64	587.44	789.51	1275.9	349.42	905.49	758.8	844.1	914.11	919.18	679.17	543.7	779.55	1188.3	550.32	526.44	609.69	1008.2	827.21	632.34	484.37	647.85	1130	1871.8	498.11	939.21	996.77	937.24	712.78	900.26	995.67	734.09	954
390	3400.9	1777.8	1594.7	1886.1	1905	2091.8	1321.7	1531.1	2176.9	2183.8	2033.8	1594.5	2115.8	1610.1	2639.9	3314	1858.8	1842.4	1525.2	3675.2	3565.5	1639.2	2272.7	2951.6	3182.9	2811.1	1849.3	2787	2456.3	3057.4	2227.7	2982.6	2276	1940.5	3085.7	4043.7	1854.1	2258.7	2019.5	4744.8	4651.9	1708.9	3582.5	3788.8	2556.9	2253.1	1972.8	3894.2	2416	3833.2	3518.1	3273.1	2576.8	2095.2	3983.7
391	4058.8	1995.3	1777.1	2073.1	2569.2	2700	1479.7	2074.4	2314.2	2875.7	2218.1	2500.6	2472.3	1808.5	3018.6	3902.1	2057.2	1961.2	1780.9	4099.9	3942.1	1839	2652.4	3224.5	3615.2	3737.6	2129.6	3346	2806.6	3434.8	2347.8	3566.8	2390.4	2270	3684.2	4808.1	2133.6	2554.2	2317.7	5310.8	5099.7	2063.6	3678.6	4172.1	3102.7	3481.3	2197.5	4081.4	2803.7	4239.6	3849.1	3653.3	3193.2	2438.3	4319.8
392	3.8326	3.1807	2.9348	3.0482	3.2239	3.2549	2.8862	2.9367	3.2159	3.4452	3.2006	3.1371	3.2219	2.9248	3.4745	3.7305	3.1632	3.0086	2.9206	3.7213	3.7355	2.983	3.3057	3.6051	3.7195	3.6523	3.2637	3.5248	3.4077	3.6502	3.2537	3.6696	3.2048	3.1261	3.71	4.0682	3.1193	3.2861	3.1757	4.0799	4.1354	3.0531	3.799	3.9259	3.4021	3.4893	3.2408	3.8397	3.3416	3.912	3.8097	3.6336	3.4802	3.1687	3.9724
393	3.4048	2.6869	2.3946	2.4474	2.4912	2.4461	2.3725	2.3354	3.0645	2.7956	2.9145	2.5539	2.8386	2.4975	2.9384	3.3446	2.6666	2.7346	2.6291	3.3038	3.5023	2.6436	2.8445	2.9463	3.1218	2.9592	2.62	3.3686	2.9019	3.3093	2.7764	3.1436	2.6168	2.8022	3.0971	3.6171	2.4904	2.8249	2.892	3.5907	3.733	2.8105	3.4406	3.508	3.0256	2.6098	2.89	3.6173	3.0896	3.8492	3.7553	3.2303	3.0248	2.9616	3.7674
394	1.6206	1.2643	1.2309	1.3412	1.564	1.8304	1.1965	1.6208	1.2599	1.845	1.3511	1.7997	1.3137	1.3494	1.3181	1.7156	1.2735	1.1946	1.365	1.5412	1.4146	1.1856	1.252	1.3145	1.2877	1.8973	1.3255	1.5135	1.5741	1.4242	1.4782	1.5117	1.2892	1.2915	1.5225	1.7588	1.4828	1.4072	1.407	1.6406	1.4501	1.5935	1.145	1.3971	1.6063	2.3531	1.2664	1.5418	1.6504	1.5434	1.4457	1.5955	1.882	1.565	1.3552
395	0.8342	0.752	1.1137	0.928	0.8463	1.2656	0.8671	1.1917	1.0796	0.9937	1.215	0.8214	0.7874	0.9003	0.8847	0.7725	0.852	0.8849	1.0995	1.2869	0.8365	0.8755	0.8405	0.9392	0.9646	1.0181	0.7088	0.8646	0.995	1.0138	1.2343	0.91	1.1551	0.9577	0.8661	0.9938	0.8897	0.9431	0.9935	1.003	0.9792	0.9742	1.1048	0.9217	1.099	1.2215	1.0556	1.2326	1.2531	1.1298	1.0147	1.1219	0.8886	1.0399	1.134
396	256.13	171.63	191	191.88	206.88	249.25	154.13	225.38	225.25	240.38	223.75	227.38	199.38	194.63	217	254.13	176.38	191.5	207.88	281.38	256.63	187.13	206	211.88	229.63	267.88	171.25	245.5	242	256.25	241	245.88	215.13	213.38	234.75	273.75	195.25	212	225.88	301.63	274.5	231.38	250.13	253.75	267.5	309.38	211.5	288.38	286.13	295.88	282.5	285.25	262.25	253.88	274.88
397	299.82	210.14	225.09	229.45	246.41	292.01	189.7	258.97	266.19	289.74	267.83	272.73	234.95	223.76	256.99	297.47	216.83	225.24	248.56	321.32	288.72	218.47	242.86	254.64	272.05	311.55	209.74	289.17	282.23	301.26	277.01	287.99	247.47	242.47	277.95	326.96	235	253	266.05	350.73	315.02	271.12	290.7	299.19	301.78	347.03	243.07	328.05	327.18	337.98	324.85	331.87	303.87	289.91	318.8
398	2.1249	1.4287	0.7836	1.2597	0.902	4.2083	1.2055	3.0105	5.3491	2.7122	2.6181	3.7205	0.8869	4.2571	2.9499	3.1328	0.9635	3.8382	3.1308	4.6459	3.688	3.78	4.0212	1.4894	1.5126	2.7263	1.1748	1.6874	3.7149	3.1922	4.5529	5.1096	1.1341	4.4461	1.493	2.6102	1.7279	2.4422	3.8963	3.5247	4.6337	2.3605	3.7531	5.491	2.3017	5.6941	5.198	1.4557	2.1201	2.7329	4.1279	1.5187	4.6509	3.3486	4.2756
399	0.0674	0.0312	0.0322	0.0185	0.0263	0.0453	0.0272	0.0336	0.0367	0.0333	0.0474	0.0476	0.0219	0.039	0.0277	0.0277	0.0183	0.0258	0.0391	0.0439	0.0398	0.038	0.0371	0.0224	0.0222	0.0515	0.0316	0.0349	0.0465	0.0332	0.0505	0.0498	0.0242	0.0544	0.0338	0.0327	0.0204	0.0261	0.0392	0.0409	0.0439	0.068	0.0775	0.0461	0.0588	0.1153	0.0748	0.0609	0.0767	0.0706	0.0773	0.0746	0.0537	0.0852	0.0484
400	2.9022	3.1375	3.0395	3.1551	3.0949	2.9326	3.2263	3.0776	2.9339	2.9525	2.8872	2.961	3.2033	3.0659	3.1173	2.9854	3.1916	3.0983	2.9887	2.9095	2.9955	3.1073	3.0529	3.0939	3.0483	2.8844	3.1868	3.0188	2.9327	3.0121	2.9094	2.9089	3.0772	3.0258	3.0624	3.0039	3.1246	3.0827	2.9604	2.9031	2.9555	2.8809	2.8566	2.8916	2.8804	2.7525	2.9744	2.8293	2.7657	2.7828	2.7489	2.7561	2.9156	2.8471	2.866
401	2.6537	2.7907	2.8067	2.9101	2.8535	2.6492	2.9495	2.7875	2.3511	2.6523	2.3309	2.6054	2.9495	2.6564	2.8847	2.7577	2.9325	2.6518	2.4757	2.6386	2.7558	2.7822	2.7895	2.8693	2.8231	2.5935	2.9171	2.7207	2.4141	2.7771	2.321	2.4467	2.8383	2.6637	2.822	2.7759	2.8777	2.7142	2.3895	2.6496	2.7214	2.4581	2.5421	2.5829	2.584	2.4218	2.5888	2.4819	2.1967	2.372	2.1584	2.2942	2.6131	2.4049	2.5568
402	1.8942	2.371	2.2738	2.58	2.4695	1.8605	2.4734	2.2778	1.7946	2.1557	1.8507	1.975	2.6533	2.0533	2.4664	2.1456	2.5433	2.2564	1.9938	1.9258	2.0718	2.1926	2.2624	2.5141	2.4229	1.8097	2.4125	2.1671	1.9477	2.177	1.833	1.8025	2.4136	1.9979	2.3415	2.2317	2.4848	2.2751	1.8714	2.0036	2.0754	1.4994	1.6676	1.8221	1.7253	1.321	1.5972	1.5484	1.3254	1.4562	1.2331	1.3214	1.8055	1.3546	1.7026
403	0.9056	1.5474	1.3977	1.8325	1.6177	1.2447	1.6009	1.4661	1.2268	1.3867	1.1958	1.2195	1.7331	1.3397	1.5827	1.3749	1.7988	1.5206	1.2667	1.2137	1.2873	1.313	1.2913	1.6553	1.609	1.086	1.4809	1.362	1.1678	1.3207	1.0995	1.1222	1.5362	1.1469	1.4155	1.3178	1.7027	1.5458	1.2613	1.1819	1.1993	0.8891	0.8135	1.1622	1.0309	0.7254	0.9096	0.8848	0.7849	0.8277	0.738	0.7958	1.0964	0.7829	1.0308
404	0.4685	0.8389	0.8474	1.0771	0.977	0.7318	0.9545	0.817	0.7354	0.777	0.6975	0.6619	0.9867	0.7935	0.9089	0.8143	1.0319	0.9554	0.7545	0.6693	0.688	0.7509	0.7192	0.9522	0.9025	0.6428	0.8351	0.7482	0.6585	0.7173	0.6507	0.6377	0.9452	0.6414	0.8121	0.7386	0.9232	0.8643	0.7228	0.6498	0.6235	0.4976	0.4072	0.6519	0.5869	0.3785	0.5012	0.5276	0.44	0.4454	0.4265	0.4413	0.6161	0.4416	0.5931
405	0.1938	0.4098	0.4329	0.57	0.5336	0.3437	0.4733	0.3919	0.3529	0.4091	0.35	0.3119	0.456	0.3743	0.4666	0.4262	0.5223	0.5116	0.3719	0.2988	0.3005	0.3893	0.3787	0.4618	0.4969	0.3449	0.4117	0.3559	0.3405	0.3768	0.3735	0.3458	0.5172	0.3199	0.4167	0.3704	0.4339	0.3999	0.3746	0.3076	0.2782	0.258	0.2016	0.3175	0.3063	0.1885	0.2513	0.2688	0.1994	0.2401	0.1998	0.2389	0.3032	0.2046	0.3006
406	0.084	0.1828	0.1917	0.2407	0.2424	0.1441	0.2046	0.1692	0.1613	0.1782	0.1684	0.1126	0.1983	0.1488	0.2058	0.192	0.1998	0.1874	0.1547	0.1056	0.1234	0.1221	0.1453	0.2012	0.2255	0.1523	0.1844	0.1485	0.131	0.1991	0.1771	0.1599	0.2229	0.1322	0.1764	0.1517	0.1686	0.1376	0.1714	0.1377	0.0952	0.0812	0.0788	0.127	0.1186	0.0838	0.1	0.1104	0.0607	0.117	0.0563	0.1149	0.1155	0.075	0.1175
407	0.0336	0.0761	0.0726	0.0756	0.0993	0.0535	0.0766	0.0512	0.0476	0.0724	0.0664	0.0242	0.0588	0.0482	0.0696	0.0869	0.0668	0.0357	0.0439	0.0274	0.0353	0.0382	0.0449	0.0712	0.0688	0.0626	0.0737	0.0498	0.0435	0.098	0.0553	0.0571	0.0847	0.0412	0.0663	0.0389	0.0497	0.0486	0.0554	0.047	0.0221	0.024	0.0126	0.0412	0.052	0.0328	0.034	0.0299	0.0262	0.05	0.0132	0.0519	0.0378	0.0232	0.0313
408	668	354	367.38	301.88	397.75	541.5	311.88	371	459.25	444.5	470.38	486.38	353	430.63	407.75	498.75	269.25	354	397.88	626	576.38	391.13	432.88	410.5	410	650.5	372.75	467.63	510.38	465.38	504	580	396.25	497.25	517.75	543.13	296.25	358.5	443.38	644.75	634.13	544.75	665.5	629	647.88	794.13	573.13	717.5	644.63	743.13	724.63	689.88	598	648.75	681.38
409	385.25	191.38	201	157.5	226.88	299.25	173.13	210.75	245.88	246.13	262	277.5	192.5	236.88	227.5	271.38	143	187.75	214.63	362	327.5	221.88	235.63	224.75	225	366	211.63	263.75	274.25	266.25	291.75	329.25	212.75	286.63	300.63	302.13	155.5	194.88	242.5	363.13	367.63	296.25	362.75	348.38	365.88	462.63	327.5	404.25	361.5	405.63	398.13	390.5	343.25	360.38	381.13
410	265.5	131.63	135.88	103.38	152.75	201.25	116.63	148.25	181.63	169	194.88	192.25	123.88	165.25	156.38	183.88	94.125	132.13	159.63	250.38	216.5	152.63	161.75	150.63	149.13	251.5	140.5	184.63	201.38	187.38	221.75	232.63	140.88	205.88	203.5	205.75	105.13	135.63	178.88	251.63	251.25	205.75	250.38	240.38	248	324.38	225.75	276.38	264	282.63	284.25	277.13	239.5	246.25	261.38
411	201.38	101.5	102.5	72.5	115.88	164.63	86.125	115.5	151.75	131.13	163.13	155.13	89.125	129.5	114.5	143.75	71.5	102.13	132.63	201	165.38	115.88	120.75	107.75	115	200.88	106.25	143	160.25	145.25	185.63	190.88	102.5	164.75	154	154.38	77.25	103	145.38	199.75	190	174	201.13	197	200.75	272.75	190.5	229	227.38	235.88	245.38	237.25	193.38	209.5	214.38
412	157.5	78.625	77.5	53.625	89	139.25	65	92.625	130	103.88	135	125	68	108.5	89.875	116.88	52.875	83.625	104.38	164.63	135.75	88.625	88.25	84	90.125	169.5	83.375	111.75	128.75	114.75	152.75	159.13	78.125	127.38	121.25	121.88	59.625	80.5	119.5	166.38	149.13	147.63	160.75	163.75	166.88	240.75	165.5	196.13	197.38	202.38	216.75	207.38	160.88	184.25	183
413	127.88	59	62.5	41.625	71.5	120	54	74.5	91.25	84.125	90.625	101.5	50.75	88	74.125	92.875	42	55.75	75.125	135.75	112.13	68.5	64.125	66.125	72.5	144	68.875	88.75	94.5	88.875	104.75	121.63	58.5	100	100.75	97.125	44.625	53.375	83.125	135.88	117.75	119.25	132.38	140.63	137.88	210.38	140.13	164.63	146.38	166.25	168.88	168.75	136.13	155.38	154.25
414	96.125	40.625	46.25	32	55.75	78	41	46.125	51.125	52.375	53.875	68.375	37.75	53.875	57.75	62.5	31.625	37.625	43.5	91.25	86.5	51.375	42.375	49.875	56.75	99.625	56.25	61	49.25	60.375	61.625	82	43.5	63.25	85	72.5	34	32.875	44.125	94.875	91.125	69.875	80.375	89.375	91.625	144.13	87.875	106	77.625	87.875	89.75	98.25	93.375	88.75	100.5
415	61.375	28.375	27.75	20.25	37.75	38.25	27.125	27.75	29.625	28.875	34.5	37.75	25.5	28	39.625	31.375	21.5	23.25	26.625	51.125	47.125	30.5	22.75	32	36.375	55.625	37.375	36.25	27.125	34.5	37.75	47	24.25	41.5	56.75	45	19.25	20.25	24.125	54.125	56.125	26.375	33.375	40.75	42.75	67.5	40.75	48.125	31.5	34.125	28.75	39.875	49.375	36.125	49.625
416	0.5	0.7071	0.5	0.5	3.0822	0.7071	0.5	0.866	0.7071	0.7071	0.5	0.5	0.5	0.5	0.5	0.5	0.5	1.118	0.5	0.5	1.5811	0.5	0.5	0.7071	6.6708	0.7071	0.5	0.5	6.5192	0.7071	0.5	0.866	6.5765	0.5	5.244	0.5	0.5	0.7071	0.866	6.6708	0.5	0.5	6.6895	0.5	0.7071	0.5	0.5	2.0616	0.5	0.5	0.5	3.6056	0.5	0.7071	0.5
417	0.5	0.5	1.118	2.0616	3.6742	0.5	1.2247	1.2247	0.5	0.7071	0.5	0.7071	0.7071	0.866	0.5	1.6583	0.5	0.7071	0.7071	0.5	2.7386	0.5	6.8191	0.5	0.7071	1.5	6.6895	0.5	0.7071	0.7071	1.118	0.5	0.5	0.5	7.2801	6.7268	0.866	6.5	0.7071	7.0711	1.5	0.5	0.5	1.118	0.7071	0.5	1.5811	1.5811	3	1.5	0.5	0.5	1.5811	0.7071	0.5
418	0.7071	0.866	1.5811	2	0.7071	1.118	0.866	0.5	0.866	0.7071	1	0.5	0.5	0.7071	1	1.2247	1	0.866	0.866	1	1.4142	0.7071	6.6895	0.866	6.6332	0.866	6.9642	0.7071	6.8191	1.2247	1.2247	1.2247	6.5192	0.7071	6.5955	6.7823	0.7071	6.5	0.5	0.7071	1.2247	0.7071	6.8191	0.7071	1.2247	0.7071	2.0616	0.5	2.6926	1.2247	0.7071	3.2016	1.118	0.7071	0.7071
419	0.5	0.5	0.5	1.2247	0.866	0.5	0.5	9.1378	0.866	7.433	0.5	0.5	0.5	0.7071	0.7071	0.7071	0.7071	0.5	0.5	7.3485	0.5	7.0887	6.7454	0.5	0.7071	7.1764	0.5	0.7071	0.7071	0.866	0.5	0.7071	6.5192	7.1764	0.7071	6.442	0.7071	0.5	0.7071	0.866	0.5	0.5	0.7071	1.2247	0.5	0.5	7.6485	0.5	0.5	0.7071	0.7071	0.7071	0.7071	0.5	7.7782
420	0.7071	0.7071	7.7942	0.5	0.5	0.7071	7.3824	9.1241	0.5	7.4498	7.6322	0.5	0.5	0.5	1.118	0.866	1.118	8.5	0.5	7.0356	5.9372	0.5	6.8739	4.717	0.5	0.5	0.7071	8.3815	0.5	0.5	8.1548	0.5	6.3443	0.5	0.5	0.7071	0.7071	1.118	1.118	0.5	0.5	0.5	0.5	0.866	0.5	0.5	7.2629	7.1589	0.5	0.7071	0.5	0.5	4.6904	0.5	0.5
421	0.5	0.5	7.5829	0.866	1.2247	0.5	7.1589	0.866	0.7071	0.5	7.8422	0.7071	0.7071	0.5	0.5	0.5	0.5	8.5147	0.7071	0.7071	6.1644	7.0711	0.5	4.4159	0.5	7.1239	0.866	8.8741	0.866	0.7071	8.2916	0.5	4.7697	6.6895	1.118	7	1.2247	0.7071	0.5	1.2247	0.7071	0.7071	1.118	0.5	0.7071	1	0.5	6.6708	1	0.7071	0.5	0.5	5.244	0.7071	7.7621
422	-2.5431	-2.3607	-2.3999	-2.3893	-2.5874	-2.5571	-2.8331	-2.4961	-2.3897	-2.6694	-2.9197	-3.0724	-2.5364	-2.4496	-2.4726	-2.5964	-2.297	-2.3009	-2.8818	-3.3529	-2.4941	-2.4109	-2.2437	-2.7197	-2.5775	-2.575	-2.4281	-2.7753	-2.3751	-2.6392	-3.3226	-2.5206	-2.3195	-2.8208	-2.4366	-2.2839	-2.49	-2.6252	-3.1582	-2.6171	-2.5506	-2.2077	-2.1605	-2.5356	-2.9281	-3.0562	-2.5011	-2.5095	-2.7953	-2.64	-2.4029	-2.2077	-2.5818	-2.2861	-2.5017
423	-2.4507	-2.327	-2.3276	-2.2869	-2.5769	-2.4418	-2.8065	-2.49	-2.3355	-2.5415	-2.8498	-3.0428	-2.4719	-2.3897	-2.4472	-2.474	-2.267	-2.2291	-2.8719	-3.1136	-2.2885	-2.3702	-2.2193	-2.6591	-2.546	-2.5478	-2.373	-2.6874	-2.1959	-2.4711	-3.1001	-2.4743	-2.3109	-2.7209	-2.4137	-2.273	-2.4373	-2.4061	-3.0507	-2.559	-2.4767	-2.2072	-2.1581	-2.5202	-2.8409	-2.9306	-2.4518	-2.4971	-2.7489	-2.5601	-2.3823	-2.1642	-2.5318	-2.2609	-2.3893
424	-2.3907	-2.3267	-2.2512	-2.2774	-2.5608	-2.4413	-2.7652	-2.4586	-2.3286	-2.3602	-2.8364	-2.9534	-2.464	-2.3647	-2.4107	-2.4356	-2.2572	-2.2041	-2.8266	-3.0355	-2.2505	-2.3633	-2.1976	-2.5569	-2.5321	-2.5301	-2.3559	-2.6553	-2.1933	-2.4509	-3.009	-2.4606	-2.2914	-2.709	-2.3614	-2.2041	-2.3184	-2.2726	-2.9634	-2.5168	-2.4649	-2.2069	-2.1366	-2.5161	-2.8316	-2.8427	-2.4303	-2.4856	-2.6658	-2.5233	-2.3536	-2.1615	-2.5035	-2.2097	-2.3748
425	-7.6899	-9.1304	-8.1601	-7.6232	-10.842	-8.7997	-8.0906	-7.2092	-8.223	-8.6363	-8.1713	-7.5158	-7.516	-7.2027	-8.3851	-8.9835	-8.2894	-7.1101	-7.5083	-7.1853	-7.3158	-7.1941	-7.1639	-7.9922	-8.7252	-8.6639	-8.5191	-7.2602	-8.0458	-10.288	-7.643	-7.771	-7.176	-7.1703	-8.6376	-7.297	-8.2141	-8.0772	-8.1824	-9.4767	-7.2138	-7.7686	-6.9113	-7.6248	-9.206	-7.8746	-7.0014	-7.399	-9.7789	-9.1242	-7.0639	-10.614	-7.8759	-8.6834	-7.0694
426	-7.5885	-8.288	-8.0098	-7.6046	-9.8272	-8.7755	-7.5772	-7.0913	-7.865	-8.5313	-8.1408	-7.0303	-7.4285	-7.1247	-8.2294	-8.7954	-8.1629	-6.8532	-7.4659	-7.1538	-7.1741	-7.0538	-7.0768	-7.7454	-8.6082	-8.5003	-8.2027	-7.1578	-7.9855	-10.265	-7.4124	-7.6364	-7.0121	-7.135	-8.4949	-7.1933	-8.0711	-8.0132	-8.1213	-9.3112	-7.119	-7.7522	-6.6892	-7.4316	-8.6324	-7.739	-6.9807	-7.2874	-9.1541	-9.0248	-6.8473	-10.556	-7.6162	-8.4525	-6.8012
427	-7.5524	-8.2326	-7.6611	-7.3725	-9.8245	-8.3809	-7.4886	-7.0214	-7.8005	-8.2451	-7.9391	-6.7979	-7.2956	-7.0927	-8.1828	-8.7378	-8.0533	-6.7897	-7.1439	-7.0616	-7.089	-7.0056	-7.0237	-7.7161	-8.5735	-8.2994	-7.9802	-7.0907	-7.7966	-10.131	-7.262	-7.5096	-7.0072	-7.1028	-8.0561	-7.1629	-7.6056	-7.8004	-7.7257	-8.7665	-7.0756	-7.6918	-6.5939	-7.1945	-8.5919	-7.1571	-6.8814	-7.1912	-8.5013	-8.6846	-6.8403	-10.492	-7.3769	-7.7406	-6.7676
428	1.285	1.2575	1.241	1.2536	1.2644	1.2409	1.2377	1.2091	1.2502	1.3078	1.2751	1.2436	1.2413	1.224	1.2629	1.2967	1.2681	1.2263	1.2331	1.2812	1.2736	1.2155	1.2372	1.2939	1.3061	1.2937	1.256	1.2687	1.2668	1.2838	1.2636	1.2828	1.244	1.2183	1.2937	1.3253	1.2664	1.2467	1.2345	1.305	1.3134	1.2109	1.2642	1.3166	1.2443	1.2446	1.2278	1.286	1.254	1.309	1.2845	1.2806	1.2635	1.214	1.3185
429	285.63	387.63	347.88	448.25	472.5	338.88	388.13	363.5	318.88	337.38	334.5	314.38	369.75	374.38	409.25	318.38	442.13	373.13	319	349.38	375.75	377.88	358.5	462	502.5	383.88	417.5	341	384.25	369.63	383.13	374	388.5	372.38	413.88	310	435.5	393	332.88	399	415	231.63	238.88	373.63	380.38	264.88	286.63	256.88	273.38	244.88	279.75	289.25	256	233.75	320.13
430	366.75	506.13	491.13	657.63	653	465.5	489.25	523.63	428.63	452.88	436.38	462.13	515.75	499.38	546.75	426.88	607	513	443.75	519	523.63	505.5	518.25	668.38	728	509.88	532.75	515.13	499.25	480.13	482.88	479.13	538.63	520.5	556.5	447.38	619.88	561.38	455.38	563.13	580	302.75	349.75	516.38	510	336.38	345.63	332.13	333	288.63	327.38	351	359.13	312.13	418.75
431	290.5	414	410.75	544.75	530.88	381.88	410.88	439.5	356.63	399.25	373.5	372.13	458.75	398.25	475.13	372.13	489.5	427.88	352.75	398.25	436.88	371.88	396.13	558	566.38	396.88	415.13	426.5	386.88	395.5	383.25	381.13	460.75	374.88	457.13	375.5	473.13	453.75	369.75	433	443.88	212.38	254.5	395.75	370.38	245.5	248.88	278.38	244.88	229.5	226.75	254.38	294	236.25	321.88
432	138.88	219.63	220.38	298	285	209.25	222.88	229.88	205	224.63	206.5	186	251.38	213.75	251.13	208.75	267.5	240.25	197.13	211.38	229.13	184.5	191	287.75	290.5	204	208.63	227.25	192.13	214.25	194.88	200.25	248.13	177.25	241.5	190.75	251.13	232.63	202.63	214	211.88	113.75	108.88	206.63	183.88	120	123.13	154.13	124.13	119.38	114	135.88	169.75	119.13	180.25
433	64	101.25	110.75	146.13	146	99.375	110.13	104.88	101	113	103.75	85.625	114.5	101.38	122.38	108.25	128.88	119.13	95.75	99.375	98.375	89.75	91	139	139.25	103.25	99.5	105.88	93	108.38	101.38	98.375	129.63	86	117.88	91.875	112.5	110	99	101.25	92.875	55.75	46.125	100.5	90.25	55.625	57.875	78	55.25	58.875	54.375	69.875	80.875	55.125	92.375
434	27.5	46.25	51.25	66.125	72.5	42.875	49.125	46.5	45	54.75	48.375	37	51.125	43.25	57.625	54.75	57.5	54.75	43	41.25	40.5	40.25	42.5	61.75	68.5	50.5	46.375	47.5	42.375	56.625	51.25	48.5	62.625	38	57.625	41.25	49	46.25	46.75	46	37.125	28.25	21.875	45.625	41.5	27.5	28.25	37.75	22.875	31.875	22.625	38.375	40.5	24	42.625
435	10	19.25	21	25.875	30.625	17.625	19.875	18.25	18.875	23.625	22	12.5	20.25	15.875	23.625	25.875	19.75	18.5	16.5	13.75	15.375	12.375	16.125	23.625	28.875	21.125	20.375	19.375	16	29	21.875	20.375	25	14.5	22	16.25	18	16.625	20.875	19	12	9.125	8.75	16.875	16.25	12.625	10.75	14.75	8.5	15.125	7.125	18.5	14.625	9.5	16.5
436	3.625	7.25	7.5	8.25	11.25	6.125	6.625	5.375	4.875	8.5	7.5	2.625	5.875	4.25	7.375	11.375	6	3	4.25	3.125	4.25	3.375	4.5	7.625	9.25	8	6.5	5.375	5	13	6.5	7.375	8.125	4.5	8.125	3.75	4.875	5	5.375	7	2.75	2.625	1.375	5.125	6.875	4.25	3.5	3.875	3.375	6	1.875	9	4.375	2.5	3.875
437	0.2806	0.8376	0.8583	1.4813	1.0057	0.5646	1.0011	0.7766	0.6111	0.7907	0.5632	0.4948	1.0525	0.6437	0.8697	0.7641	1.49	0.9688	0.6641	0.4528	0.4942	0.6413	0.6306	0.9527	1.0201	0.4503	0.7687	0.6445	0.5233	0.6651	0.4961	0.468	1.016	0.4426	0.6431	0.6513	1.1914	0.8885	0.6052	0.4839	0.4239	0.3414	0.2511	0.4602	0.3997	0.2092	0.3117	0.3265	0.2661	0.2937	0.2502	0.2986	0.4118	0.2629	0.4223
438	0.1409	0.4643	0.5037	0.931	0.6354	0.2794	0.5747	0.4177	0.3064	0.449	0.2996	0.2466	0.6115	0.3427	0.5317	0.4435	0.8409	0.552	0.3336	0.2152	0.2525	0.3614	0.3727	0.5777	0.6228	0.2614	0.4482	0.3453	0.2855	0.4036	0.2855	0.2652	0.6402	0.239	0.3872	0.3725	0.665	0.4648	0.3328	0.2616	0.2112	0.1803	0.1299	0.2392	0.2204	0.1095	0.1647	0.1747	0.1226	0.1733	0.1141	0.1718	0.2262	0.1253	0.2163
439	0.7636	0.2225	0.4092	0.9363	0.4047	1.9341	0.7948	1.9068	2.2711	1.202	0.9558	2.0794	0.7449	1.9158	1.3765	1.3747	0.6947	1.6976	1.7584	2.3881	1.669	1.4426	1.3094	0.9183	0.6451	1.2304	0.8313	1.2471	1.682	1.5643	1.8547	2.1184	0.2728	1.505	0.7269	1.0612	1.2276	1.2417	1.6993	1.5454	1.8007	1.3487	1.8704	2.7242	1.4297	2.0573	2.3056	0.872	1.0024	1.2461	1.6325	0.5437	2.0783	1.1968	2.0776
440	0.9241	0.3415	0.4257	0.9917	0.4683	2.0051	0.7106	1.873	2.4206	1.18	1.0093	1.8968	0.7957	1.9176	1.6436	1.5721	0.6353	1.7548	1.7539	2.3985	1.7495	1.4695	1.2658	1.0512	0.6728	1.1665	0.7356	1.1753	1.5714	1.6121	1.8344	2.0567	0.3918	1.4566	0.6862	1.1163	1.2331	1.3104	1.5839	1.5604	1.9048	1.298	1.9042	2.6803	1.2324	2.0021	2.1588	0.8006	1.0767	1.1937	1.6375	0.5768	2.0768	1.2088	2.0335
441	0.8267	0.4176	0.2754	0.9503	0.5711	1.9975	0.6741	1.996	2.7678	1.3639	1.4789	1.883	0.8429	2.0803	1.7073	1.5954	0.6052	2.0204	1.9414	2.4175	1.8538	1.4586	1.4327	0.9825	0.6547	1.1147	0.71	1.2016	1.8327	1.6181	2.1567	2.229	0.4958	1.5104	0.5962	1.1733	1.2879	1.3819	1.795	1.5701	1.9091	1.0866	1.8477	2.7432	1.2618	1.995	2.1283	0.7451	0.7333	1.1666	1.582	0.3901	2.0623	1.121	2.0022
442	0.9425	0.6273	0.3432	0.9633	0.5036	2.2458	0.734	2.2485	3.2291	1.7697	1.8429	2.2649	0.8275	2.4683	1.7705	1.9024	0.5289	2.3006	2.2761	2.7426	1.943	1.625	1.5432	1.0697	0.8509	1.4509	0.7101	1.3614	2.2617	1.7644	2.5017	2.5539	0.5947	1.6579	0.6264	1.3414	1.3507	1.552	2.2998	2.0235	2.0285	1.2708	2.4934	3.1695	1.6016	2.2299	2.4146	0.9699	0.7529	1.4227	1.6488	0.5186	2.3832	1.2212	2.3833
443	1.2731	0.7019	0.6005	1.1412	0.5892	2.4997	0.7996	2.4929	3.4636	2.0467	2.0699	2.6914	0.9205	2.7324	1.907	2.0712	0.7387	2.4177	2.555	3.102	2.1545	1.7237	1.5678	1.0315	0.9755	1.7395	0.733	1.5997	2.499	1.9224	2.845	2.8363	0.7543	1.7717	0.7638	1.6692	1.462	1.7052	2.6497	2.353	2.217	1.5462	3.2222	3.5735	1.9473	2.4068	2.6942	1.2345	0.9124	1.693	1.8002	0.6347	2.7921	1.3594	2.8336
444	1.4297	0.3973	0.8499	1.3347	0.5779	2.7138	0.8803	2.7356	2.7312	2.2024	1.2405	2.8563	1.0572	3.0123	2.0041	2.2661	0.9469	1.9711	2.3673	3.378	2.3218	1.7177	1.835	1.1563	0.9822	1.935	0.6982	1.8689	2.3852	2.0464	2.2757	2.7003	1.1073	1.9436	0.8491	1.8089	1.5984	1.6973	2.3482	2.662	2.3524	2.0677	3.7304	3.7679	2.2383	2.6036	2.9407	1.3635	1.8701	1.9946	2.3147	1.1101	3.0113	1.4797	3.0846
445	1.8581	0.6494	1.2674	1.5341	0.5604	2.4266	0.8105	2.4645	2.1015	0.8839	0.8395	2.2789	1.1363	2.3067	2.0965	1.9664	1.4344	1.6477	1.9184	2.9519	2.1804	1.7417	2.1178	1.3758	0.9269	1.3524	0.7748	1.7172	1.3145	2.1195	1.3235	2.2785	1.2721	1.6567	0.9004	1.8295	1.6911	2.0891	1.3287	2.001	2.3237	2.0583	3.0319	3.0402	1.4725	2.116	2.3816	1.1701	2.7619	1.9622	2.241	1.3899	2.5739	1.8438	2.5415
446	1.8873	0.9869	1.8663	1.8173	0.7841	1.9524	0.4529	2.2018	1.8613	0.486	0.9188	1.3832	1.3953	1.8416	1.9809	1.2371	1.802	1.6815	1.88	2.36	2.0448	1.5162	2.352	1.2392	0.7843	1.1582	0.4217	2.0761	1.8464	2.0035	1.3521	1.7981	1.5362	1.5042	0.4456	1.4419	1.9786	1.9336	1.0099	0.9359	1.8528	2.3437	2.1899	1.9851	0.5574	1.5156	2.1822	1.5116	2.7853	1.0515	2.9153	2.0334	1.6122	1.8884	1.6586
447	0.239	0.3905	0.318	0.1156	0.3025	0.5291	0.3176	0.2876	0.7167	0.3978	0.3925	0.4673	0.1665	0.5657	0.1412	0.4986	0.3329	0.6242	0.5355	0.5878	0.1705	0.6298	0.7087	0.0578	0.3575	0.5388	0.2168	0.1043	0.8008	0.1858	0.6065	0.6703	0.4418	0.7082	0.2287	0.5731	0.1974	0.472	0.7809	0.7121	0.4184	0.1917	0.2537	0.5397	0.2897	0.3445	0.5411	0.2206	0.2068	0.354	0.3732	0.2404	0.4153	0.3314	0.4831
448	0.3742	0.5107	0.2439	0.1214	0.3353	1.1854	0.3894	0.5698	1.7105	0.674	0.8741	1.155	0.2411	1.2198	0.2688	0.84	0.3874	0.9951	1.1099	1.316	0.5817	0.9147	0.9416	0.2367	0.466	1.1353	0.234	0.317	1.6056	0.469	1.4731	1.6342	0.4883	1.3151	0.3979	0.7139	0.2268	0.6912	1.6278	1.4299	0.943	0.5725	1.0246	1.6732	0.9048	1.5641	1.695	0.6313	0.3841	1.0304	1.1777	0.3242	1.2575	0.8982	1.3912
449	0.8472	0.6154	0.3235	0.2061	0.4081	1.7784	0.4492	0.8808	2.0977	0.9153	0.9877	1.5926	0.2123	1.6373	0.5538	1.2677	0.424	1.1745	1.2591	1.9831	1.1669	1.3432	1.2941	0.3711	0.5913	1.6084	0.3504	0.4402	1.8425	0.9156	1.801	2.3015	0.4745	1.8715	0.6158	1.0282	0.3026	0.8715	1.8638	1.9487	1.6053	1.1242	1.706	2.5399	1.3322	3.0173	2.6924	0.9592	0.8451	1.5783	2.0173	0.4772	2.0753	1.6664	2.05
450	1.1517	0.8224	0.5086	0.3026	0.4291	2.1219	0.5274	1.1244	2.4246	1.2185	1.1135	1.7931	0.1996	1.9884	1.0096	1.6233	0.5749	1.4701	1.3012	2.24	1.5815	1.9027	2.1776	0.5128	0.7299	1.6401	0.4165	0.5829	1.9823	1.3354	2.2098	2.6698	0.6419	2.5148	0.7369	1.3535	0.4803	1.0596	1.9714	2.0385	2.2865	1.3616	2.11	2.8254	1.2744	3.5343	3.0962	0.8573	1.1451	1.5258	2.4249	0.9373	2.4561	2.1021	2.2622
451	1.3461	1.0674	0.6766	0.3571	0.4896	2.3072	0.6236	1.1001	2.5827	1.4447	1.2834	1.8527	0.0897	2.3913	1.269	1.6409	0.6925	1.8709	1.2964	2.2728	1.8715	2.4487	2.7864	0.5669	0.9646	1.6445	0.5921	0.4859	2.0209	1.587	2.4632	2.8828	0.9061	3.0393	0.9614	1.7043	0.7268	1.1925	2.114	1.9853	2.7258	1.4116	2.1056	2.7754	1.2317	3.7032	3.0905	0.7268	1.4934	1.5774	2.5864	1.2875	2.6075	2.3201	2.3343
452	1.7011	1.3308	0.7808	0.733	0.5934	2.6127	0.7786	1.115	2.8231	1.5807	1.4616	2.0572	0.4923	2.7961	1.6735	1.7506	0.9361	2.2936	1.2873	2.1954	2.2111	3.1726	3.4467	0.7032	1.1434	1.7534	1.0524	0.6409	2.0121	1.6983	2.7773	3.1075	1.2818	3.7414	1.1982	2.0752	0.9898	1.7051	2.0607	2.0174	3.3489	1.5402	2.208	2.7954	1.2125	3.8023	3.227	0.7166	2.0619	1.6147	2.793	1.8361	2.8267	2.6019	2.6753
453	2.0693	1.7924	1.1143	1.0796	0.5394	2.943	1.0804	0.9835	3.081	1.979	1.4352	2.2234	0.8312	3.4752	2.2913	1.8836	1.4402	2.9124	1.1638	2.2349	2.2754	3.9455	4.1727	0.9721	1.1368	2.2736	1.5903	0.4636	1.9866	1.7898	2.8409	3.3172	1.8686	4.1677	2.0271	2.2363	0.9944	2.9633	2.2664	1.8721	3.9442	2.2601	2.9068	2.705	2.0549	3.9906	3.661	0.8151	3.481	1.6625	4.1287	2.5875	3.1691	2.7141	3.0269
454	2.0423	2.0591	1.3584	2.1785	1.3293	3.2179	0.8115	1.1466	3.5764	1.8353	1.3133	1.9889	1.8913	3.4676	2.9782	2.2683	2.1329	2.9971	1.0275	2.0922	2.5863	4.1958	4.2657	1.5198	1.8093	2.496	2.0922	0.9494	2.0352	2.016	2.4438	3.6728	1.7819	4.4512	2.6003	1.9701	1.2762	3.6928	2.9004	2.1379	3.9292	2.595	3.4608	2.4541	2.5827	3.9088	4.0152	1.4154	3.4833	2.2202	5.2983	3.2354	3.3215	2.6767	2.9163
455	1.358	1.2398	1.2934	1.2832	1.3027	1.3878	1.2201	1.377	1.3428	1.303	1.3227	1.351	1.2946	1.3037	1.3139	1.3333	1.2349	1.2995	1.3277	1.3953	1.3588	1.2988	1.3169	1.2852	1.3033	1.365	1.2376	1.3578	1.3443	1.3474	1.352	1.3513	1.3215	1.34	1.3098	1.3349	1.2656	1.317	1.3586	1.3986	1.3556	1.384	1.3651	1.3297	1.4118	1.4778	1.3378	1.4012	1.4289	1.3769	1.3927	1.4113	1.3839	1.4175	1.3544
456	442.49	345.71	361.56	366.86	387.95	416.2	319.23	379	398.52	429.49	410.23	397.01	362.55	352.46	393.3	438.38	351.75	352.25	373.85	463.06	431.37	342.63	371.42	404.7	427.96	456.31	343.2	425.78	415.47	434.45	417.06	435.97	380.17	367.17	420.09	469.13	367.99	380.12	392.76	491.72	470.84	384.66	425.8	456.39	434.27	476.22	374.6	472.26	455.34	480.97	464.91	473.06	436.9	406.6	475.5
457	600.88	428.63	467.63	470.75	505.38	577.63	389.5	521.88	535.13	559.63	542.63	536.38	469.38	459.5	516.75	584.5	434.38	457.75	496.38	646.13	586.13	445	489.13	520.13	557.75	622.88	424.75	578.13	558.5	585.38	563.88	589.13	502.38	492	550.25	626.25	465.75	500.63	533.63	687.75	638.25	532.38	581.25	606.88	613.13	703.75	501.13	661.75	650.63	662.25	647.5	667.63	604.63	576.38	644
458	1270.3	1114.9	1121	1251.9	1289.9	1242.5	1077.3	1226.1	1135.8	1300.4	1145.5	1180.4	1265.4	1104.5	1318.5	1363.6	1199.1	1117.6	1101.8	1403.4	1353.4	1084.9	1158.3	1342	1397.5	1329.9	1150.5	1338.8	1214.3	1355.6	1190.9	1262.1	1240.3	1123	1381.6	1491.4	1218.5	1207.8	1139.5	1481.4	1454.5	1044.3	1172.3	1335.3	1258.8	1269.9	1091.9	1310	1159.5	1283.8	1176.6	1223.9	1285.5	1092.1	1373.9
459	848.63	825.25	834.63	975.13	984.38	862	803.75	916	772.5	950.63	778.13	825.38	968.75	768.88	990.38	994.5	935.25	818.38	775	978.25	956.38	788.75	854.63	1024	1067.3	892.63	851.5	979.13	818.38	999	805.88	847.88	937.5	780.63	1007.4	1090.5	945.38	900.5	783.13	1039.1	1027.6	661.63	750.13	901	826.88	769.13	690.88	827.63	691.5	784.38	679.63	738.63	870.38	649.25	910.75
460	460.63	526.25	520.38	646.38	635.25	514.38	514.25	570.25	476.25	603	481.88	492.63	630.75	470.88	630.13	615.63	607.75	531.63	482.13	570.88	564.88	471.75	517.75	664.75	687.88	515.13	524.75	596	506.88	600	489.13	498.88	601.25	448.38	624.75	666.13	603.63	570.88	486.75	612.38	594.75	355.25	403.13	531.38	465.38	379.38	371	451	367.5	428.75	357.25	389.13	495.38	337.88	516.5
461	178.25	249.38	244.25	321.5	307.88	249.63	245.5	262.63	235	288	230.38	215.88	297.38	226.63	293.88	289.25	302.75	266.38	230.5	257.63	256.25	210.5	222.63	311	327.63	237.5	237.25	269.5	228.63	271.88	219.13	233.88	288.63	192.75	283	294.75	289.25	264.63	234.13	271.75	252.5	157.25	154.75	241.88	209.38	157.5	158	197.5	162.75	186.38	157.13	176.38	223.63	149	235.38
462	74.5	110.25	116.63	153.13	153.63	113.63	116.75	115.13	111	133.63	109.75	95.125	130.38	106.38	136	140.5	140.25	128	106	113.38	107	97.875	102	143.5	152.13	113.25	108	119	105.38	124.63	110	108.88	143.13	91.125	130.88	134	125.25	120.5	108.25	121.75	106.5	70.25	62.875	110.63	99.125	67.875	70.375	90.25	70.25	83	71.125	82.75	98.625	64.75	110.38
463	28.375	47.125	51.625	67.5	73.625	46	49.5	48.25	46.5	58.875	48.875	38.25	54.5	44.375	60.875	63.75	60.125	56.375	44.25	43.25	41.75	41.875	45	62.25	71.625	52.5	47.625	49.375	45.75	58.625	53	50.625	65.625	39.375	59.625	57.5	51.375	47.875	48.375	52.25	40.125	31.375	26.125	47.125	44.25	29.875	31.375	40	27.875	40.875	28	40.75	43.75	26.25	46.375
464	10	19.25	21	25.875	30.625	17.625	19.875	18.25	18.875	23.75	22	12.5	20.25	15.875	23.625	27.5	19.875	18.5	16.5	13.75	15.375	12.375	16.125	23.625	29	21.125	20.375	19.375	16.125	29	21.875	20.375	25.125	14.5	22	20.625	18	16.625	20.875	20.375	12	9.125	8.875	16.875	16.5	12.625	10.875	14.75	9.5	17.875	7.5	18.5	14.625	9.5	16.625
465	3.625	7.25	7.5	8.25	11.25	6.125	6.625	5.375	4.875	8.5	7.5	2.625	5.875	4.25	7.375	11.375	6	3	4.25	3.125	4.25	3.375	4.5	7.625	9.25	8	6.5	5.375	5	13	6.5	7.375	8.125	4.5	8.125	3.75	4.875	5	5.375	7	2.75	2.625	1.375	5.125	6.875	4.25	3.5	3.875	3.375	6	1.875	9	4.375	2.5	3.875
466	[illegible]	[illegible]	[illegible]	[illegible]	[illegible]	[illegible]	[illegible]	[illegible]	[illegible]	[illegible]	[illegible]	[illegible]	[illegible]	[illegible]	[illegible]	[illegible]	[illegible]	[illegible]	[illegible]	[illegible]	[illegible]	[illegible]	[illegible]	[illegible]	[illegible]	[illegible]	[illegible]	[illegible]	[illegible]	[illegible]	[illegible]	[illegible]	[illegible]	[illegible]	[illegible]	[illegible]	[illegible]	[illegible]	[illegible]	[illegible]	[illegible]	[illegible]	[illegible]	[illegible]	[illegible]	[illegible]	[illegible]	[illegible]	[illegible]	[illegible]	[illegible]	[illegible]	[illegible]	[illegible]	[illegible]
467	[illegible]	[illegible]	[illegible]	[illegible]	[illegible]	[illegible]	[illegible]	[illegible]	[illegible]	[illegible]	[illegible]	[illegible]	[illegible]	[illegible]	[illegible]	[illegible]	[illegible]	[illegible]	[illegible]	[illegible]	[illegible]	[illegible]	[illegible]	[illegible]	[illegible]	[illegible]	[illegible]	[illegible]	[illegible]	[illegible]	[illegible]	[illegible]	[illegible]	[illegible]	[illegible]	[illegible]	[illegible]	[illegible]	[illegible]	[illegible]	[illegible]	[illegible]	[illegible]	[illegible]	[illegible]	[illegible]	[illegible]	[illegible]	[illegible]	[illegible]	[illegible]	[illegible]	[illegible]	[illegible]	[illegible]
468	[illegible]	[illegible]	[illegible]	[illegible]	[illegible]	[illegible]	[illegible]	[illegible]	[illegible]	[illegible]	[illegible]	[illegible]	[illegible]	[illegible]	[illegible]	[illegible]	[illegible]	[illegible]	[illegible]	[illegible]	[illegible]	[illegible]	[illegible]	[illegible]	[illegible]	[illegible]	[illegible]	[illegible]	[illegible]	[illegible]	[illegible]	[illegible]	[illegible]	[illegible]	[illegible]	[illegible]	[illegible]	[illegible]	[illegible]	[illegible]	[illegible]	[illegible]	[illegible]	[illegible]	[illegible]	[illegible]	[illegible]	[illegible]	[illegible]	[illegible]	[illegible]	[illegible]	[illegible]	[illegible]	[illegible]
469	[illegible]	[illegible]	[illegible]	[illegible]	[illegible]	[illegible]	[illegible]	[illegible]	[illegible]	[illegible]	[illegible]	[illegible]	[illegible]	[illegible]	[illegible]	[illegible]	[illegible]	[illegible]	[illegible]	[illegible]	[illegible]	[illegible]	[illegible]	[illegible]	[illegible]	[illegible]	[illegible]	[illegible]	[illegible]	[illegible]	[illegible]	[illegible]	[illegible]	[illegible]	[illegible]	[illegible]	[illegible]	[illegible]	[illegible]	[illegible]	[illegible]	[illegible]	[illegible]	[illegible]	[illegible]	[illegible]	[illegible]	[illegible]	[illegible]	[illegible]	[illegible]	[illegible]	[illegible]	[illegible]	[illegible]
470	[illegible]	[illegible]	[illegible]	[illegible]	[illegible]	[illegible]	[illegible]	[illegible]	[illegible]	[illegible]	[illegible]	[illegible]	[illegible]	[illegible]	[illegible]	[illegible]	[illegible]	[illegible]	[illegible]	[illegible]	[illegible]	[illegible]	[illegible]	[illegible]	[illegible]	[illegible]	[illegible]	[illegible]	[illegible]	[illegible]	[illegible]	[illegible]	[illegible]	[illegible]	[illegible]	[illegible]	[illegible]	[illegible]	[illegible]	[illegible]	[illegible]	[illegible]	[illegible]	[illegible]	[illegible]	[illegible]	[illegible]	[illegible]	[illegible]	[illegible]	[illegible]	[illegible]	[illegible]	[illegible]	[illegible]
471	[illegible]	[illegible]	[illegible]	[illegible]	[illegible]	[illegible]	[illegible]	[illegible]	[illegible]	[illegible]	[illegible]	[illegible]	[illegible]	[illegible]	[illegible]	[illegible]	[illegible]	[illegible]	[illegible]	[illegible]	[illegible]	[illegible]	[illegible]	[illegible]	[illegible]	[illegible]	[illegible]	[illegible]	[illegible]	[illegible]	[illegible]	[illegible]	[illegible]	[illegible]	[illegible]	[illegible]	[illegible]	[illegible]	[illegible]	[illegible]	[illegible]	[illegible]	[illegible]	[illegible]	[illegible]	[illegible]	[illegible]	[illegible]	[illegible]	[illegible]	[illegible]	[illegible]	[illegible]	[illegible]	[illegible]
472	[illegible]	[illegible]	[illegible]	[illegible]	[illegible]	[illegible]	[illegible]	[illegible]	[illegible]	[illegible]	[illegible]	[illegible]	[illegible]	[illegible]	[illegible]	[illegible]	[illegible]	[illegible]	[illegible]	[illegible]	[illegible]	[illegible]	[illegible]	[illegible]	[illegible]	[illegible]	[illegible]	[illegible]	[illegible]	[illegible]	[illegible]	[illegible]	[illegible]	[illegible]	[illegible]	[illegible]	[illegible]	[illegible]	[illegible]	[illegible]	[illegible]	[illegible]	[illegible]	[illegible]	[illegible]	[illegible]	[illegible]	[illegible]	[illegible]	[illegible]	[illegible]	[illegible]	[illegible]	[illegible]	[illegible]
473	[illegible]	[illegible]	[illegible]	[illegible]	[illegible]	[illegible]	[illegible]	[illegible]	[illegible]	[illegible]	[illegible]	[illegible]	[illegible]	[illegible]	[illegible]	[illegible]	[illegible]	[illegible]	[illegible]	[illegible]	[illegible]	[illegible]	[illegible]	[illegible]	[illegible]	[illegible]	[illegible]	[illegible]	[illegible]	[illegible]	[illegible]	[illegible]	[illegible]	[illegible]	[illegible]	[illegible]	[illegible]	[illegible]	[illegible]	[illegible]	[illegible]	[illegible]	[illegible]	[illegible]	[illegible]	[illegible]	[illegible]	[illegible]	[illegible]	[illegible]	[illegible]	[illegible]	[illegible]	[illegible]	[illegible]

Table S12 Amino acid descriptors (2D) used in this study.

	F	F	F	F	F	F	G	G	G	G	G	G	G	G	G	G	G	G	G	G	G	G	G	G	G	G	H	H	H	H	H	H	H	H	H	H	H	H	H	H	H	H	H	H	H	H	I	I	I	I	I	I	I	I	I
	R	S	T	V	W	Y	A	C	D	E	F	G	H	I	K	L	M	N	P	Q	R	S	T	V	W	Y	A	C	D	E	F	G	H	I	K	L	M	N	P	Q	R	S	T	V	W	Y	A	C	D	E	F	G	H	I	K
No.	FR	FS	FT	FV	FW	FY	GA	GC	GD	GE	GF	GG	GH	GI	GK	GL	GM	GN	GP	GQ	GR	GS	GT	GV	GW	GY	HA	HC	HD	HE	HF	HG	HH	HI	HK	HL	HM	HN	HP	HQ	HR	HS	HT	HV	HW	HY	IA	IC	ID	IE	IF	IG	IH	II	IK
1	-6	-6	-4	-5	-1	-3	1	-1	2	-1	-1	-1	2	-3	-7	2	-7	-4	-1	-4	-2	-1	0	-7	-3	-1	-2	0	-3	4	-2	2	-5	-4	-2	5	-7	-6	-6	-1	1	-1	-5	-2	1	-3	0	-2	2	1	-1	-3	-4	-2	0
2	-3.7	-6.7	-3.4	-5.3	-0.2	-3.6	1.8	-3	4.3	-1.3	2.6	-0.9	2.5	-2.5	-5.7	2.5	-3.2	-3.2	-3.1	-4.9	-3.8	0.5	0.8	-5	-2.1	4.4	-1.3	0	-3.7	3.6	-3.1	2.5	-1.6	-3.5	-2.8	5.2	-4.7	-2.6	-4.4	-0.1	2	-0.2	-1.3	-3.3	1.8	-3.8	0.1	-2.5	1.4	1	-1.7	-2.5	-3.5	-1.1	-0.4
3	-2.5	-5.7	-3.8	-4.9	-0.2	-2.4	1.5	-2.1	3.9	-1.2	0.6	-0.5	2.4	-2.6	-5.4	2.4	-3.3	-2.9	-1.7	-4.3	-3.2	0.5	0.7	-4.4	-1.6	3.7	-0.7	0	-2.9	3.3	-1.7	2.4	-1.9	-3.5	-1.8	4.8	-4.3	-2.7	-3.6	0.4	-1.1	-0.2	-1.7	-2.2	1.5	-3	0.5	-2.7	1.4	1	-0.9	-2.6	-3.5	-1.1	0.1
4	-1.2	-4.3	-4.3	-4.2	0.2	-1.2	1	-1.8	2.9	-1	-0.4	-0.2	2.2	-2.2	-4.7	2.2	-2.6	-2.4	-1	-3.5	-2.6	-0.5	0.2	-2.3	-1	2.5	-0.3	-0.2	-2.2	3.6	-0.2	2.2	-1.8	-3.2	-1	4.8	-3.2	-2	-2.8	0.3	-1.6	0.2	-2	-1	1	-2.1	0.7	-2.8	1.2	0.9	-0.3	-2.2	-3.2	-1	0.4
5	-2.7	-2.9	-2.5	-2.4	0.3	-1.9	3.6	-0.4	-0.2	0.3	2.6	0.3	1.7	0.1	-1.7	1.7	-3.1	-0.4	-1.2	-1.5	-1.2	1.4	1.1	-2.2	-2.1	2.3	-0.6	-1.7	-2.1	4.7	-2.2	1.7	-1.5	-1.3	-2.2	4.8	-3	-1.9	-2.1	-1.5	1.8	0.3	1	-1.6	3.6	-2.1	0.4	0.9	1.8	3.5	-2.1	0.1	-1.3	-1.6	-2.1
6	0.1	-0.5	-0.22	-0.55	-0.19	0.26	0.13	-0.17	0.47	-0.17	0.21	-0.16	0.56	-0.11	-0.4	0.56	-0.29	-0.2	-0.18	-0.31	-0.29	-0.23	0.11	0.36	-0.11	0.08	-0.32	-0.14	-0.29	1	0.33	0.56	-0.14	-0.33	0.09	1	-0.51	-0.03	-0.44	0.12	-0.03	-0.19	0.25	0.43	0.13	-0.18	0.2	-0.37	0.32	0.22	-0.01	-0.11	-0.33	0.05	0.22
7	-5	-5	-3	-4	1	-2	2	0	3	0	0	0	2	-2	-6	2	-5	-3	-1	-4	-2	0	1	-5	-1	0	-1	0	-2	2	-2	2	-4	-4	-1	4	-5	-4	-5	0	2	1	-3	-1	2	-2	1	-2	2	1	0	-2	-4	-1	0
8	2	0	2	1	2	2	4	2	5	3	3	3	5	1	1	5	1	2	2	1	2	4	4	2	2	3	3	3	2	6	2	5	2	1	2	6	0	1	1	2	2	2	3	3	4	1	3	2	4	4	3	1	1	2	3
9	3	3	2	2	2	2	1	1	1	1	1	1	1	2	2	1	2	1	2	2	2	1	1	3	2	1	1	2	2	0	1	1	2	2	2	0	3	2	2	2	1	2	1	2	1	2	2	1	1	1	2	2	2	2	2
10	10	2	7	3	5	9	5	3	10	3	8	6	6	8	2	6	8	3	8	1	3	6	6	8	10	7	5	9	3	10	9	6	8	6	4	10	5	2	6	6	0	5	8	9	5	8	10	7	9	6	9	8	6	8	7
11	-0.9	-3.9	-4.5	-4	0.4	-1.1	0.6	-1.7	2.7	-0.9	-0.5	-0.2	2.2	-2.2	-4.5	2.2	-2.4	-2.2	-1	-3.2	-2.4	-0.8	0.1	-1.8	-0.8	2.2	-0.2	-0.3	-2	3.8	0	2.2	-1.8	-3.1	-0.7	4.7	-3	-1.8	-2.6	0.3	-1.6	0.4	-2	-0.7	0.6	-2	0.7	-2.8	1.2	0.8	-0.2	-2.2	-3.1	-0.9	0.5
12	196	140	135	172	40	198	29	91	26	103	194	110	23	142	177	23	154	102	101	97	97	98	94	61	86	83	71	111	96	0	192	23	139	158	108	0	170	77	116	86	101	40	159	33	29	84	46	149	68	94	91	142	158	91	65
13	-2	-3	-4	-3	0	-3	0	-1	3	-2	-3	-1	2	-2	-4	2	-3	-2	0	-2	-3	-2	-1	-2	-2	2	-1	-1	-2	6	-1	2	-2	-2	-1	7	-3	-1	-4	0	-2	0	-3	0	0	-3	0	-2	1	0	-1	-2	-2	-2	0
14	-2	-5	-6	-5	0	-2	0	-2	3	-3	-4	-1	2	-3	-5	2	-4	-3	-1	-4	-4	-3	-2	-4	-2	3	-2	-2	-4	8	-1	2	-4	-4	-2	9	-5	-3	-5	-1	-4	0	-4	-2	0	-5	0	-5	1	0	-1	-3	-4	-3	0
15	-3	-6	-7	-7	0	-2	0	-3	3	-3	-5	-2	2	-4	-6	2	-5	-4	-1	-5	-5	-4	-3	-5	-3	3	-2	-3	-4	9	-2	2	-5	-6	-3	10	-7	-4	-5	-1	-5	0	-6	-3	0	-5	0	-6	1	0	-2	-4	-6	-4	-1
16	-4.4	-6.4	-5.5	-8	-2.3	-7.7	0.1	-4.2	3.2	-3.6	-7.7	-0.6	2.6	-3.7	-7	2.6	-6.9	-3.7	-0.6	-6	-5.4	-2.5	-2.1	-3.3	-3.1	-0.4	-1.4	-1.4	-4.9	8	-4.8	2.6	-7.6	-3.8	-1	8.5	-6.9	-2.1	-6.4	-1.5	-3.8	-2.3	-8.2	-2.5	0.1	-3.9	-0.8	-4.7	1.7	0.1	-0.6	-3.7	-3.8	-2.4	-0.2
17	-2	-5	-3	-4	0	-2	2	-2	4	-1	2	-1	2	-2	-5	2	-3	-3	-2	-4	-3	0	1	-4	-2	4	-1	0	-3	3	-2	2	-1	-3	-2	5	-4	-2	-4	0	0	0	-1	-2	2	-3	0	-2	1	1	-1	-2	-3	-1	0
18	-1	-6	-2	-5	2	-1	5	0	9	-3	3	-1	6	-2	-6	6	-3	-3	-2	-4	-3	3	3	-3	-3	6	-1	-1	-2	11	0	6	-1	-4	-2	12	-3	-1	-4	2	5	2	-1	-4	5	-4	3	-3	3	5	-2	-2	-4	-2	0
19	0	0	0	0	0	0	5	9	9	0	5	5	9	0	0	9	0	6.6	5	9	0	0	0	0	0	6.6	5	0	0	10	0	9	0	0	0	10	0	5	6.6	0	6.6	0	0	0	5	0	0	0	5	0	6.6	0	0	0	6.6
20	0	-1	-1	-1	0	0	0	-1	1	0	-1	0	1	-1	-1	1	0	-1	-1	-1	-1	0	0	0	0	0	0	0	-1	3	0	1	0	-1	0	2	0	-1	-1	0	0	0	0	0	0	-1	1	-1	0	1	0	-1	-1	0	0
21	-3	-18	-16	-10	-3	-13	-1	0	6	-9	3	1	4	-1	-15	4	-10	-4	0	-14	-4	-6	-7	1	-4	2	-1	2	-3	11	-7	4	-7	-11	-9	29	-12	-3	-15	2	3	-3	-15	-3	-1	-9	3	-3	2	-1	2	-1	-11	-8	2
22	-17	-30	-20	-16	-3	-17	5	-6	10	-12	-7	6	8	-8	-25	8	-5	-10	-5	-11	-4	8	6	-19	-2	5	6	5	-3	6	-12	8	-11	-19	-7	17	-16	3	-20	5	2	-3	-36	0	5	-6	6	-3	6	10	6	-8	-19	-11	8
23	4	-24	-5	-18	-1	4	0	0	5	-2	-7	2	4	-5	-18	4	2	-7	0	-18	-3	1	2	-14	-5	-1	2	-1	-3	16	5	4	1	-18	1	23	-3	-10	-13	2	-8	-1	4	-14	0	-8	2	-6	3	-2	-1	-5	-18	-2	3
24	0	-21	-6	-20	-3	3	4	2	22	2	-8	4	11	0	-26	11	0	-3	-1	-14	-1	8	0	-27	-30	23	3	4	-3	4	1	11	4	-15	5	71	-2	-3	-14	5	-35	-3	3	-4	4	-27	4	0	-3	6	2	0	-15	2	3
25	-17	-12	-8	-21	-5	-26	-7	2	9	-1	-25	-4	9	-3	-17	9	-29	-8	-5	-9	-2	4	3	-25	-11	18	2	-2	-4	18	-11	9	6	-8	-2	38	-30	-13	-9	0	-31	-5	-33	-10	-7	-19	1	-8	14	-1	-3	-3	-8	5	0
26	-23	-7	-9	-27	16	-29	11	3	22	-20	-9	3	18	3	-24	18	7	-12	4	-10	-14	13	-4	-8	2	20	10	6	-11	18	-28	18	5	-6	-26	80	-1	7	-16	10	16	16	-10	-16	11	-19	18	-14	13	16	6	3	-6	-13	17
27	-7	-22	-18	-14	-2	-13	0	-2	7	-8	0	2	5	-3	-20	5	-11	-7	-2	-15	-6	-4	-5	-5	-3	3	0	2	-4	10	-8	5	-9	-14	-8	27	-15	-1	-18	3	2	-2	-21	-2	0	-9	4	-4	2	2	3	-3	-14	-7	3
28	3	-28	-8	-21	1	5	2	-2	9	2	-8	4	7	-5	-24	7	1	-10	-2	-22	-6	3	4	-25	-6	4	2	1	-6	13	3	7	1	-22	5	28	-5	-8	-19	5	-29	1	4	-11	2	-14	5	-7	5	3	1	-5	-22	3	5
29	-28	-13	-11	-22	5	-30	2	2	14	-6	-19	1	13	-2	-21	13	-11	-10	-3	-11	-9	6	1	-12	-4	21	6	1	-7	15	-27	13	-5	-9	-7	49	-21	-5	-12	4	-5	5	-18	-15	2	-18	7	-12	13	7	0	-2	-9	2	8
30	3	0	1	1	2	1	5	1	5	3	1	4	5	2	1	5	0	2	3	1	1	3	3	3	3	4	3	3	2	8	1	5	1	0	3	8	0	2	0	3	3	2	1	3	5	2	4	1	4	4	3	2	0	1	3
31	0	0	0	0	0	0	0	0	3	0	0	0	3	0	0	3	0	0	0	0	0	0	0	1	0	2	0	0	0	5	0	3	0	0	0	5	0	0	0	0	0	0	0	1	0	0	2	0	1	0	0	0	0	0	0
32	-0.29	-0.81	-0.58	-0.75	0.13	-0.13	0.11	-0.83	0.04	0.14	0.4	0.05	0.2	-0.97	-0.62	0.2	-0.74	-0.74	-0.97	-0.79	-0.82	0.13	0.15	-0.23	-0.21	0.35	0	-0.16	-0.54	0.38	-0.19	0.2	-0.46	-0.71	-0.15	0.36	-0.64	-0.31	-0.85	0.01	-0.26	0.13	-0.18	-0.35	0.11	-0.59	0.2	-0.83	0.21	0.38	-0.16	-0.97	-0.71	-0.12	-0.14
33	1.46	3.59	3.6	4.1	0.96	1.63	0.82	2.29	0.4	2.9	2.38	2.74	0.65	3.08	4.27	0.65	2.48	2.62	2.3	2.47	2.49	2.78	2.37	1.72	2.17	2.27	2.03	1.78	2.43	0	0.86	0.65	2.83	3.7	2.4	0	3.26	2.02	2.81	2.04	2.72	0.96	2.22	0.86	0.82	2.11	0.99	3.37	1.29	1.84	1.92	3.08	3.7	1.8	1.87
34	10	6	6	6	11	8	10	6	11	6	10	9	11	6	4	11	10	6	9	5	4	6	9	11	11	9	9	9	5	16	8	11	9	6	8	16	9	7	7	10	7	11	8	10	10	9	11	5	10	11	11	6	6	9	10
35	-0.25	-0.24	-0.23	0.01	-0.14	-0.15	0.15	-0.1	0.14	-0.18	0.25	-0.1	0.3	-0.13	-0.31	0.3	-0.16	0	0.22	0.15	0.05	-0.45	0.08	0.03	-0.18	0.13	-0.12	-0.29	-0.03	1	-0.1	0.3	0.04	-0.13	0.02	1	-0.34	-0.01	-0.25	-0.1	0	-0.14	0.12	0.13	0.15	-0.11	-0.14	-0.06	0.04	-0.19	0.32	-0.13	-0.13	-0.1	0.02
36	-0.29	-0.27	-0.24	0.01	-0.14	-0.16	0.19	-0.09	0.21	-0.21	0.29	-0.13	0.35	-0.13	-0.35	0.35	-0.16	0.03	0.22	0.2	0.06	-0.46	0.08	0.04	-0.2	0.14	-0.12	-0.31	-0.04	1	-0.1	0.35	0.04	-0.12	0.01	1	-0.36	-0.01	-0.26	-0.12	0.03	-0.14	0.13	0.15	0.19	-0.13	-0.16	-0.07	0.04	-0.22	0.36	-0.13	-0.12	-0.1	0.03
37	-5	-4	-5	-6	-2	-4	0	-4	2	-2	-6	0	2	-2	-5	2	-3	-3	1	-4	-3	-2	-1	-2	-2	0	-1	-1	-3	5	-4	2	-6	-3	-1	6	-3	-1	-4	-1	-2	-2	-6	-1	0	-2	0	-3	2	0	0	-2	-3	-2	0
38	-0.006	0.433	0.429	0.261	0.121	-0.029	0.093	0.374	0.236	-0.037	0.195	-0.043	0.037	0.438	0.475	0.037	0.191	0.446	0.211	0.552	0.305	0.164	0.25	0.357	0.356	0.083	0.037	0.37	0.531	1	0.427	0.037	0.105	0.278	0.008	1	0.341	0.201	0.364	0.266	-0.059	0.121	0.246	0.333	0.093	0.534	0.082	0.452	0.299	0.208	0.261	0.438	0.278	0.156	0.012
39	0.912	-0.187	0	-0.249	-0.102	0.717	0.392	0.058	0.872	0.133	0.817	0.804	0.452	-0.09	-0.107	0.452	0.418	0.049	0.45	0.108	0.108	0	0	0.792	0.523	0.492	0.427	0.138	0.045	1	0.755	0.452	0.032	-0.072	0.276	1	-0.167	0.266	0.482	0.558	0.169	-0.102	0	0.573	0.392	0.284	0.222	-0.101	0.305	0.49	0.55	-0.09	-0.072	0.265	0.245
40	5	0	0	0	0	4	2	0	6	1	4	3	3	0	0	3	3	0	2	0	0	-1	0	4	4	3	2	1	0	8	5	3	0	1	1	8	0	0	3	2	0	0	1	4	2	2	1	0	2	2	2	0	1	3	2
41	-1.6	0.6	0	-0.2	-0.6	-1.6	-0.4	1.1	2.1	-0.3	-1.1	2	0.8	0	-0.3	0.8	-1.4	1.2	1.2	0.4	1.4	0.3	-0.4	-0.7	-0.6	-0.8	1.9	1.3	1.5	2.2	-1.4	0.8	-1.6	0.4	-1.2	2.2	-1.5	0.8	0.7	1.2	-0.8	-0.6	-1.7	0.4	-0.4	-0.7	1.6	0.9	-0.3	1	1.8	0	0.4	-1	0.7
42	2	-40	-21	-40	-8	12	0	0	24	-4	5	-10	32	-12	-31	32	-8	-24	-12	-11	-25	-5	8	12	-10	12	-5	-8	-18	100	8	32	-7	-23	1	100	-18	0	-22	11	-7	-8	-1	29	0	-10	12	-32	8	17	0	-12	-23	8	7
43	0	-7	-10	-11	4	2	4	-1	21	2	-1	6	16	-3	-10	16	-10	-8	-2	-8	-11	1	7	4	-2	0	5	1	-11	15	3	16	-4	-9	11	26	-8	-1	-10	10	-3	4	1	2	4	-4	13	-11	5	12	0	-3	-9	11	8
44	1	-7	-8	-10	1	4	2	-2	14	1	0	4	12	-4	-9	12	-9	-7	-3	-8	-10	0	8	4	-2	0	2	0	-10	11	5	12	-5	-9	7	16	-6	-3	-10	7	-4	1	0	3	2	-3	10	-11	2	10	1	-4	-9	8	6
45	91	26	87	25	68	93	42	2	78	24	92	19	73	49	19	73	62	6	53	0	4	43	36	93	71	77	16	60	7	100	96	73	58	47	44	100	36	19	51	53	11	68	94	85	42	65	96	51	89	75	64	49	47	71	39
46	1	-4	-2	-3	-1	0	0	0	2	0	0	-1	0	0	-4	0	0	-1	1	-2	-2	-2	-2	0	0	0	0	0	-2	4	0	0	0	-1	-1	4	-2	-1	0	0	0	-1	0	0	0	-1	1	-1	0	0	0	0	-1	0	-1
47	2	-1	2	1	-1	1	0	1	0	2	2	0	-1	2	-1	-1	3	1	1	1	1	0	-1	3	2	-2	1	2	1	-2	1	-1	3	2	0	-2	2	1	3	0	3	-1	3	1	0	2	1	2	-1	0	1	2	2	2	-1
48	1.36	0.91	0.79	1	1.23	1.24	1.06	0.97	1.11	0.69	1	0.9	1.08	1.01	0.79	1.08	1.49	1.02	1.1	0.74	0.91	1.06	1.02	0.53	1.17	0.81	0.88	1.28	0.98	1.02	1.33	1.08	1.36	0.83	0.84	1.11	1.43	0.75	0.88	1.03	0.63	1.23	1.36	0.68	1.06	1.01	1.2	0.89	1.13	1.14	1.31	1.01	0.83	0.74	0.97
49	0.97	1.17	1.25	0.96	0.78	0.96	0.92	1.14	0.84	1.03	1.22	0.99	0.69	0.92	1.17	0.69	0.65	1.05	0.97	1.26	1.23	0.87	0.61	1.71	0.79	1.23	1.08	0.71	1.08	0.8	0.91	0.69	0.72	1.11	0.75	0.66	0.69	1.25	1.07	0.88	1.33	0.78	0.8	1.51	0.92	1.13	0.71	1.12	0.78	0.72	0.68	0.92	1.11	0.91	0.77
50	0.1	0.85	0.98	1.09	0.91	0.43	1.02	0.74	1.08	1.76	0.48	1.29	1.52	1.17	1.17	1.52	0.51	0.82	0.79	1.09	0.69	1.15	1.88	0.56	1.04	0.97	1.13	0.95	0.86	1.36	0.32	1.52	0.7	1.19	2.04	1.43	0.57	1.1	1.16	1.19	1.22	0.91	0.51	0.65	1.02	0.66	1.13	1	1.18	1.3	0.92	1.17	1.19	1.91	1.63
51	19.8	9.24	14.7	12.5	15.7	20.9	13.7	15.5	21.9	15.7	17.3	17.5	21.4	13.7	9.04	21.4	13.8	12.2	17.8	10.1	14.3	19.3	18.7	10.4	11.5	19.4	20.6	17.1	14.4	27.4	21.4	21.4	14.7	11.4	19.4	25.9	12.2	13.8	11.5	17.1	9.94	15.7	15	17.6	13.7	12.9	18.6	14.8	18.9	17.7	17.2	13.7	11.4	14.6	19.1
52	4.3	1.3	0.7	1.2	5.6	4.1	5.8	3.5	7.9	4.3	4.7	5	7.4	3	0.7	7.4	2.8	3	4.2	2	2.8	4.4	5.3	3.4	4.4	7.4	5	4.9	3.2	9	5.2	7.4	3.4	2.1	4.5	9.9	2.2	3.4	2.6	5.5	3.6	5.6	3.2	4.5	5.8	3.2	5.9	2.4	6.4	6	5	3	2.1	4.3	5.7
53	-9	-10	-3	-10	0	-3	2	-8	7	-3	-1	2	5	-6	-6	5	-14	-4	-8	-6	-7	0	2	-6	-2	1	0	0	-4	11	0	5	-13	-3	2	17	-20	-1	-4	1	-6	0	-15	-2	2	-6	1	-8	5	2	1	-6	-3	-1	0
54	1.04	-1.61	-2.93	-2.65	-0.62	0.13	0.54	-0.63	1.42	0.21	0.13	-0.06	1.78	-2.01	-2.19	1.78	-0.83	-1.19	-0.13	-1.4	-1.4	-0.41	-1.2	0.25	-1.42	-0.12	-0.15	-0.57	-1.52	3.6	0.34	1.78	-2.08	-2.25	0.72	4.02	-1.97	-0.75	-2.31	0.6	-0.04	-0.62	-2.11	0.08	0.54	-0.35	0.33	-2.43	0.76	0.83	-0.75	-2.01	-2.25	-1.1	0.71
55	3.5	-4.44	-8.58	-7.41	-1.29	-0.13	0.7	-1.43	3.89	1.31	-0.44	-0.5	4.07	-4.67	-5.06	4.07	-2.47	-2.83	-1.76	-4.36	-3.93	-1.8	-3.91	-0.26	-3.01	1.17	0.34	-1.77	-3.8	10	1.32	4.07	-6.53	-6.22	0.81	11.01	-4.7	-1.01	-5.54	0.24	1.02	-1.29	-5.7	-0.08	0.7	-1.63	1.42	-5.78	1.77	1.64	-2.16	-4.67	-6.22	-3.23	2.34
56	-1.3	-13.7	-3.7	-3.4	0.3	-3.2	1.5	-1.4	3.4	-0.6	-0.3	0.2	3.3	-2.1	-3.7	3.3	-2.5	-1.5	-0.6	-3.2	-1.9	0.5	0.9	-1.8	-1.4	2.9	0.2	1.1	-2.8	2.5	0.7	3.3	-1.9	-2.5	-0.2	5.3	-2.4	-0.9	-1.7	0.7	-1.5	0.3	-1.3	0.2	1.5	-2.5	1.9	-2.4	1.4	1.6	-0.1	-2.1	-2.5	-0.1	1.7
57	2	-6	-7	-8	2	4	3	-2	9	1	0	2	8	-3	-6	8	-4	-5	-2	-5	-7	0	4	4	-1	0	1	0	-6	8	4	8	-5	-6	6	11	-6	-2	-5	4	-3	2	0	2	3	-2	5	-9	2	6	0	-3	-6	6	4
58	3	-4	0	-1	-11	-1	2	-5	4	1	0	2	2	-1	-3	2	2	-2	-1	5	1	3	3	2	-1	3	0	0	-4	0	7	2	-4	1	3	-2	-8	3	-6	2	-5	-11	-1	3	2	0	2	-4	7	1	1	-1	1	0	3
59	1	-5	-4	-7	3	1	2	0	6	-1	4	0	5	-3	-6	5	-6	-2	0	-3	0	-1	1	3	-2	2	0	0	-1	3	2	5	0	-1	1	5	-4	2	-2	0	2	3	-1	0	2	-3	2	-8	4	1	-1	-3	-1	2	2
60	1	-5	-1	-3	-1	-4	3	0	3	0	1	0	1	-3	-4	1	-4	-2	0	-1	1	1	2	4	0	1	0	0	0	0	5	1	-1	1	3	0	-8	3	-3	0	0	-1	0	2	3	-1	3	-7	3	0	0	-3	1	1	2
61	-8.7	-8.2	-8	-7.1	-7.8	-9	-8.1	-7.8	-6.6	-7.9	-10.6	-6.9	-6.3	-7.4	-9.5	-6.3	-8.3	-7.9	-8.5	-8.1	-7.7	-7.4	-6.3	-7.7	-7.7	-6.8	-6.8	-7.1	-7.4	-4.8	-8.1	-6.3	-8.6	-8.2	-7.3	-5	-10.2	-8.4	-8.6	-7.3	-9.4	-7.8	-7.5	-7.4	-8.1	-7.5	-7.1	-7.6	-6.8	-6.8	-7	-7.4	-8.2	-8	-5.7
62	-8	-32	-41	-43	2	-6	3	-10	31	-18	-18	-11	18	-19	-33	18	-30	-20	1	-30	-28	-23	-10	-28	-19	20	-8	-19	-32	59	-6	18	-30	-29	-7	65	-39	-15	-29	4	-29	2	-29	-8	3	-29	2	-32	12	1	-3	-19	-29	-17	4
63	-2	-5	-2	-5	-1	-3	-4	-6	-1	-7	-1	-6	2	-2	-5	2	-5	-6	-1	-7	-6	-3	-2	-4	-3	3	-6	-2	-7	11	-2	2	-2	-1	-5	12	-7	-6	-2	-3	-7	-1	-2	-3	-4	-5	2	-3	4	-2	-3	-2	-1	-4	-4
64	1	-5.5	-2.5	-6	-3	-0.5	-4.5	-4	-0.5	-8	0.5	-6	-0.5	-3	-5.5	-0.5	-3.5	-4.5	-1.5	-5.5	-5.5	-6.5	-4.5	-1.5	-3	2.5	-5	-3	-6	8	1	-0.5	-2.5	-2	-7	9	-7	-5.5	-2	-4.5	-4.5	-3	-1.5	-2.5	-4.5	-4.5	-1	-4	2.5	-3.5	-4	-3	-2	-6	-5.5
65	-1	-6	-7	-6	-1	-1	1	-2	4	-2	-1	-1	3	-3	-7	3	-4	-3	-1	-5	-4	-2	-1	-2	-2	3	-1	-1	-4	7	1	3	-3	-5	-1	7	-5	-3	-4	0	-4	-1	-2	-2	1	-3	0	-4	1	0	-1	-3	-5	-2	0
66	0	-4	-4	-4	0	0	1	-1	3	-1	0	0	2	-2	-5	2	-2	-2	-1	-3	-2	-1	0	-1	-1	2	-1	0	-2	4	1	2	-1	-3	0	5	-3	-2	-2	0	-3	0	-1	-1	1	-2	1	-3	1	1	0	-2	-3	-1	0
67	-1	-4	-6	-5	-1	-1	0	-2	2	-2	-2	-1	1	-3	-5	1	-3	-3	-2	-3	-3	-2	-1	-1	-2	0	-1	-2	-3	7	0	1	-3	-4	-1	7	-4	-2	-3	-1	-2	-1	-3	-1	0	-3	0	-5	0	0	-1	-3	-4	-2	0
68	0.007	0.001	0.006	0.017	0.396	0.014	0.004	0.016	0.042	0.121	0.006	0.029	0.011	0.035	0.004	0.011	0.055	0.054	0.006	0.003	0.046	0.019	0.134	0.041	0.013	0.015	0	0.033	0.015	0.029	0.011	0.011	0.01	0.009	0.022	0.021	0.047	0.006	0.023	0	0.017	0.396	0.007	0.024	0.004	0.009	0.003	0.051	0.015	0.008	0.069	0.035	0.009	0.022	0.091
69	-11.04	1.05	-8	-6.7	2.69	-10.75	-6.2	-12.42	0.39	0.5	-2.48	-0.6	-5.12	-1.72	-10.05	-5.12	-2.26	-1.08	-12.59	2.17	0.31	-2.89	0.93	-1.65	-2.29	-6.44	-7.23	-7.73	-9.26	-5.75	-8.48	-5.12	-1.44	2.61	-2.34	-1.42	-0.76	-5.32	-7.21	0.83	-0.52	2.69	-5.59	-3.54	-6.2	1.5	-1.22	-0.51	-6.44	-2.59	-5.11	-1.72	2.61	-2.03	4.09
70	-2.4	-1.2	0.2	-0.4	6.4	-0.4	-0.5	-1.8	-0.4	3.3	-0.2	2.2	0.2	2.7	-3.6	0.2	5.5	1	-4.9	2.2	0.6	-2.1	3.6	0.8	0.7	0.3	-1	-2.8	-4.7	0.6	-3.1	0.2	3.9	2.7	2.7	5.1	2.9	2.1	0.5	3.8	-1.5	6.4	1.7	4.1	-0.5	4	0.5	0.5	1.7	0.2	1.8	2.7	2.7	-1	2.4
71	-1.98	-2.5	-3.28	-3.14	6.86	-4.39	-2.34	-4.97	0.59	2.62	-1.23	0.98	-2.48	-0.65	-2.65	-2.48	0.48	-0.27	-3.34	-1.22	1.27	-1.78	3.65	0.68	-1.15	-4.03	-3.04	-2.43	-5.08	-1.75	-3.16	-2.48	-0.84	-0.03	-0.1	-0.08	1.22	-1.9	-0.79	0.01	0.51	6.86	-2.34	-0.31	-2.34	0.26	-2.08	0.93	-1.64	-0.65	0.01	-0.65	-0.03	-1.93	3.37
72	-0.2	-0.1	-0.2	0.4	0	-0.2	0.2	0.1	-1	-1.5	0.3	-0.9	-0.2	0.4	0	-0.2	-3.9	-1.5	0.2	0	-1.9	0.5	3.4	0.3	1.3	0.1	-0.2	-1	-0.6	-0.1	0.2	-0.2	0.2	0	0.4	-0.8	2	0	-0.9	-0.4	-0.2	0	0.1	0.8	0.2	0	-0.4	-0.4	0.2	-0.2	-0.4	0.4	0	-0.5	2.7
73	-2	-5	-2	-2	7	-3	-3	-4	-1	2	-1	0	-2	-1	-4	-2	0	0	-4	-2	0	-3	3	1	-1	-4	-3	-2	-3	-1	-3	-2	-1	-2	1	-1	1	-1	-1	-1	-2	7	-2	-1	-3	-1	-3	0	-1	-1	1	-1	-2	-2	2
74	-3	-7	-2	-2	-3	-2	-8	-3	-1	-1	-7	1	-2	-7	-8	-2	-7	-7	-11	-9	-1	-8	-9	-8	9	-6	-11	7	-15	-9	-15	-2	-12	-6	-12	3	-8	-6	2	2	-4	-3	-6	-5	-8	-15	-6	-5	-5	-12	8	-7	-6	-3	-6
75	0.4	8.2	-8.2	-7.3	-11.3	-10.1	9.8	-8.7	-11.6	8.8	14.8	-10.1	-2.7	-12.5	12.5	-2.7	-9	-3	27.2	-6.2	35.5	6.8	8.4	-14.8	-5.7	48.4	18.4	23.5	31.8	-9.2	-3.2	-2.7	27.4	-1.2	-10	5.3	-5.8	22.1	-10.4	-7.2	-3.9	-11.3	9.8	-9.3	9.8	-10.1	8.9	13	13.2	36.3	10	-12.5	-1.2	7.9	11
76	0.5	0.5	0.1	0.1	0.7	0.1	4.7	4.3	8.3	1	0.1	1.2	7.3	3.2	0.5	7.3	0.3	0.6	2	0.1	0.1	0.1	3.3	0.1	0.3	3.4	0.3	0.2	1.4	48.2	0.1	7.3	1.5	0.3	3.1	52.1	0.1	0.1	0.8	0.5	0.1	0.7	0.1	2.4	4.7	0.1	2	0.1	7.5	3.5	8.4	3.2	0.3	0.5	2.7
77	0.5	0.5	0.1	0.1	0.7	0.1	4.7	4.3	8.3	1	0.1	1.2	7.3	3.2	0.5	7.3	0.3	0.6	2	0.1	0.1	0.1	3.3	0.1	0.3	3.4	0.3	0.2	1.4	48.2	0.1	7.3	1.5	0.3	3.1	52.1	0.1	0.1	0.8	0.5	0.1	0.7	0.1	2.4	4.7	0.1	2	0.1	7.5	3.5	8.4	3.2	0.3	0.5	2.7
78	0.1	0.1	0.1	1.6	4.3	8.7	2	0.1	3.3	0.1	0.1	6	0.1	0.1	1.1	0.2	1.5	2.3	0.1	0.4	1.2	5	1.8	0.3	0.1	0.1	0.3	0.1	1.8	0.7	0.1	0.1	56.9	0.1	1.2	0.2	0.1	7.5	1	4.4	4.7	1.1	0.8	0.1	0.1	3.4	0.1	0.1	0.1	0.1	0.7	0.1	0.1	5	0.3
79	0.1	0.1	0.1	1.6	4.3	8.7	2	0.1	3.3	0.1	0.1	6	0.1	0.1	1.1	0.2	1.5	2.3	0.1	0.4	1.2	5	1.8	0.3	0.1	0.1	0.3	0.1	1.8	0.7	0.1	0.1	56.9	0.1	1.2	0.2	0.1	7.5	1	4.4	4.7	1.1	0.8	0.1	0.1	3.4	0.1	0.1	0.1	0.1	0.7	0.1	0.1	5	0.3
80	0.1	0.1	0.1	1.6	4.3	8.7	2	0.1	3.3	0.1	0.1	6	0.1	0.1	1.1	0.2	1.5	2.3	0.1	0.4	1.2	5	1.8	0.3	0.1	0.1	0.3	0.1	1.8	0.7	0.1	0.1	56.9	0.1	1.2	0.2	0.1	7.5	1	4.4	4.7	1.1	0.8	0.1	0.1	3.4	0.1	0.1	0.1	0.1	0.7	0.1	0.1	5	0.3
81	0.1	0.1	0.1	1.6	4.3	8.7	2	0.1	3.3	0.1	0.1	6	0.1	0.1	1.1	0.2	1.5	2.3	0.1	0.4	1.2	5	1.8	0.3	0.1	0.1	0.3	0.1	1.8	0.7	0.1	0.1	56.9	0.1	1.2	0.2	0.1	7.5	1	4.4	4.7	1.1	0.8	0.1	0.1	3.4	0.1	0.1	0.1	0.1	0.7	0.1	0.1	5	0.3
82	0.1	0.1	0.1	1.6	4.3	8.7	2	0.1	3.3	0.1	0.1	6	0.1	0.1	1.1	0.2	1.5	2.3	0.1	0.4	1.2	5	1.8	0.3	0.1	0.1	0.3	0.1	1.8	0.7	0.1	0.1	56.9	0.1	1.2	0.2	0.1	7.5	1	4.4	4.7	1.1	0.8	0.1	0.1	3.4	0.1	0.1	0.1	0.1	0.7	0.1	0.1	5	0.3
83	0.1	0.1	0.1	1.6	4.3	8.7	2	0.1	3.3	0.1	0.1	6	0.1	0.1	1.1	0.2	1.5	2.3	0.1	0.4	1.2	5	1.8	0.3	0.1	0.1	0.3	0.1	1.8	0.7	0.1	0.1	56.9	0.1	1.2	0.2	0.1	7.5	1	4.4	4.7	1.1	0.8	0.1	0.1	3.4	0.1	0.1	0.1	0.1	0.7	0.1	0.1	5	0.3
84	0.1	0.1	0.1	1.6	4.3	8.7	2	0.1	3.3	0.1	0.1	6	0.1	0.1	1.1	0.2	1.5	2.3	0.1	0.4	1.2	5	1.8	0.3	0.1	0.1	0.3	0.1	1.8	0.7	0.1	0.1	56.9	0.1	1.2	0.2	0.1	7.5	1	4.4	4.7	1.1	0.8	0.1	0.1	3.4	0.1	0.1	0.1	0.1	0.7	0.1	0.1	5	0.3
85	0.1	0.1	0.1	1.6	4.3	8.7	2	0.1	3.3	0.1	0.1	6	0.1	0.1	1.1	0.2	1.5	2.3	0.1	0.4	1.2	5	1.8	0.3	0.1	0.1	0.3	0.1	1.8	0.7	0.1	0.1	56.9	0.1	1.2	0.2	0.1	7.5	1	4.4	4.7	1.1	0.8	0.1	0.1	3.4	0.1	0.1	0.1	0.1	0.7	0.1	0.1	5	0.3
86	0.1	0.1	0.1	1.6	4.3	8.7	2	0.1	3.3	0.1	0.1	6	0.1	0.1	1.1	0.2	1.5	2.3	0.1	0.4	1.2	5	1.8	0.3	0.1	0.1	0.3	0.1	1.8	0.7	0.1	0.1	56.9	0.1	1.2	0.2	0.1	7.5	1	4.4	4.7	1.1	0.8	0.1	0.1	3.4	0.1	0.1	0.1	0.1	0.7	0.1	0.1	5	0.3
87	0.1	0.1	0.1	1.6	4.3	8.7	2	0.1	3.3	0.1	0.1	6	0.1	0.1	1.1	0.2	1.5	2.3	0.1	0.4	1.2	5	1.8	0.3	0.1	0.1	0.3	0.1	1.8	0.7	0.1	0.1	56.9	0.1	1.2	0.2	0.1	7.5	1	4.4	4.7	1.1	0.8	0.1	0.1	3.4	0.1	0.1	0.1	0.1	0.7	0.1	0.1	5	0.3
88	0.1	0.1	0.1	1.6	4.3	8.7	2	0.1	3.3	0.1	0.1	6	0.1	0.1	1.1	0.2	1.5	2.3	0.1	0.4	1.2	5	1.8	0.3	0.1	0.1	0.3	0.1	1.8	0.7	0.1	0.1	56.9	0.1	1.2	0.2	0.1	7.5	1	4.4	4.7	1.1	0.8	0.1	0.1	3.4	0.1	0.1	0.1	0.1	0.7	0.1	0.1	5	0.3
89	0.1	0.1	0.1	1.6	4.3	8.7	2	0.1	3.3	0.1	0.1	6	0.1	0.1	1.1	0.2	1.5	2.3	0.1	0.4	1.2	5	1.8	0.3	0.1	0.1	0.3	0.1	1.8	0.7	0.1	0.1	56.9	0.1	1.2	0.2	0.1	7.5	1	4.4	4.7	1.1	0.8	0.1	0.1	3.4	0.1	0.1	0.1	0.1	0.7	0.1	0.1	5	0.3
90	0.1	0.1	0.1	1.6	4.3	8.7	2	0.1	3.3	0.1	0.1	6	0.1	0.1	1.1	0.2	1.5	2.3	0.1	0.4	1.2	5	1.8	0.3	0.1	0.1	0.3	0.1	1.8	0.7	0.1	0.1	56.9	0.1	1.2	0.2	0.1	7.5	1	4.4	4.7	1.1	0.8	0.1	0.1	3.4	0.1	0.1	0.1	0.1	0.7	0.1	0.1	5	0.3
91	0.053	0.02	0	0.009	0	0.001	0.37	0.017	0.022	0.029	0.097	0.015	0.043	0.007	0.094	0.028	0.003	0.049	0.009	0.009	0.021	0.054	0.055	0	0.006	0	0.571	0.003	0.022	0.005	0.042	0.043	0.006	0.007	0.163	0.029	0.01	0.017	0.002	0.037	0.009	0.043	0	0.003	0.006	0.001	0.325	0.017	0.076	0.107	0.013	0.007	0.007	0.015	0.041
92	0.053	0.02	0	0.009	0	0.001	0.37	0.017	0.022	0.029	0.097	0.015	0.043	0.007	0.094	0.028	0.003	0.049	0.009	0.009	0.021	0.054	0.055	0	0.006	0	0.571	0.003	0.022	0.005	0.042	0.043	0.006	0.007	0.163	0.029	0.01	0.017	0.002	0.037	0.009	0.043	0	0.003	0.006	0.001	0.325	0.017	0.076	0.107	0.013	0.007	0.007	0.015	0.041
93	0.053	0.02	0	0.009	0	0.001	0.37	0.017	0.022	0.029	0.097	0.015	0.043	0.007	0.094	0.028	0.003	0.049	0.009	0.009	0.021	0.054	0.055	0	0.006	0	0.571	0.003	0.022	0.005	0.042	0.043	0.006	0.007	0.163	0.029	0.01	0.017	0.002	0.037	0.009	0.043	0	0.003	0.006	0.001	0.325	0.017	0.076	0.107	0.013	0.007	0.007	0.015	0.041
94	0.053	0.02	0	0.009	0	0.001	0.37	0.017	0.022	0.029	0.097	0.015	0.043	0.007	0.094	0.028	0.003	0.049	0.009	0.009	0.021	0.054	0.055	0	0.006	0	0.571	0.003	0.022	0.005	0.042	0.043	0.006	0.007	0.163	0.029	0.01	0.017	0.002	0.037	0.009	0.043	0	0.003	0.006	0.001	0.325	0.017	0.076	0.107	0.013	0.007	0.007	0.015	0.041
101	-5.8	-4.7	-5.1	-6.5	-7.4	-6.6	-3.8	-5.4	-3.7	-3.8	-5.6	-3.9	-4.7	-6.3	-3.8	-5.2	-5.1	-4	-4.2	-4.4	-4.5	-3.8	-4.1	-5.1	-5.8	-5.4	-4	-5.6	-4.3	-4.5	-6.4	-4.7	-4.9	-6.6	-4.1	-5.6	-5.4	-4.4	-4.5	-4.7	-4.9	-4.3	-4.5	-5.3	-6.5	-6.1	-5.9	-7.3	-5.4	-5.7	-8	-6.3	-6.6	-8.2	-5.6
102	32	36	39	114	41	45	115	53	83	54	67	75	40	109	92	155	32	80	62	75	58	145	116	183	52	103	25	11	32	24	38	40	8	27	21	49	7	25	15	17	17	43	36	42	23	44	119	33	43	35	103	109	27	81	53
103	0.394	-0.537	-0.869	-1.31	-1.744	-1.591	0	0	0	0	0	0	0	0	0	0	0	0	0	0	0	0	0	0	0	0	-0.187	-0.099	3.336	3.09	-0.879	0	-0.106	-0.818	3.084	-0.784	-0.443	0.088	-1.389	0.112	1.759	0.133	-0.096	-0.536	-0.971	-0.818	-0.899	-0.811	3.643	3.248	-1.592	0	-0.818	-1.53	3.368
104	-0.018	0.357	0.058	-0.288	-0.185	-0.335	0.282	-0.187	-0.336	-0.041	0.457	-0.068	0.127	0.127	0.065	-0.004	0.084	-0.097	0.127	-0.072	-0.037	-0.1	-0.106	0.141	0.036	0.121	-0.079	-0.187	-0.27	0.072	-0.141	0.127	0.392	-0.062	-0.235	0.029	0.293	-0.141	0.037	0.247	0.111	0.08	0.061	0.006	-0.098	-0.305	-0.535	-0.415	0.229	0.385	-0.348	0.127	-0.062	-0.051	-0.058
105	-0.22	0.14	-0.19	-0.78	-0.89	-0.82	-0.09	-0.42	-0.06	0.4	0.21	0.04	0	0.4	0.1	-0.08	0	-0.18	0.4	0.12	-0.15	-0.04	0.13	-0.2	-0.14	-0.32	-0.15	-0.82	-0.06	-0.53	-0.75	0	0.14	-0.52	0.14	-0.36	-0.35	0.06	-0.22	0.05	-0.01	-0.59	-0.27	-0.35	-0.41	-0.67	-0.64	-0.48	0.04	-0.2	-0.66	0.4	-0.52	-0.71	-0.26
106	0.41	0.29	0.31	-0.22	-0.16	0	-0.07	-0.08	-0.22	0.25	-0.38	-0.38	0.2	0.25	0.11	0.23	0.19	-0.14	-0.11	-0.06	-0.04	-0.16	-0.26	0.16	0.18	0.14	0.34	-0.19	-0.39	-0.45	-0.16	0.2	-0.29	0.49	0.22	0.16	0.99	-0.24	-0.21	-0.02	-0.12	-0.05	-0.19	0.19	-0.12	-0.34	-0.22	0.16	0.59	0.35	-0.19	0.25	0.49	-0.22	0.36
107	0.0041	-0.0001	0.0135	-0.0336	0.003	0.0107	0.0025	-0.0184	-0.0646	0.086	-0.1882	-0.1748	0.0876	0.0339	0.0376	0.0932	0.0944	-0.0348	0.0982	0.02	0.0711	-0.0505	-0.0574	0.0406	0.1083	0.0607	0.0092	-0.037	-0.0338	-0.1188	-0.094	0.0876	-0.0265	-0.006	0.0264	0.0136	-0.0151	-0.0009	-0.0737	0.0522	-0.0218	0.0017	-0.005	-0.001	-0.0196	-0.0768	-0.1004	-0.0026	0.0181	0.05	-0.116	0.0339	-0.006	-0.0449	0.0175
108	-0.0904	0.0398	-0.112	-0.4001	-0.3405	-0.4212	0.1844	-0.0982	0.1115	0.2311	0.0789	0.2219	0.0979	0.1174	0.1963	0.0782	-0.0573	0.1196	0.2131	0.171	-0.0251	0.1075	0.1763	0.1859	-0.1567	-0.0176	0.0266	-0.0701	-0.0749	-0.0827	-0.125	0.0979	0.0005	-0.0326	0.039	-0.0169	-0.0345	0.0386	0.0295	-0.0125	-0.0184	-0.0005	0.0681	-0.0039	-0.02	-0.1976	-0.0737	-0.2235	0.1892	0.1103	-0.3791	0.1174	-0.0326	-0.5852	0.0682
109	-3.54	-3.56	-3.76	-5.75	-6.02	-4.95	-2.15	-3.16	-1.62	-1.22	-3.72	-2.17	-1.94	-3.65	-0.84	-3.43	-3.75	-1.56	-1.72	-1.54	-1.68	-1.7	-2.03	-3.06	-3.37	-2.5	-2.09	-3.63	-2.14	-2.27	-4.61	-1.94	-2.78	-3.76	-1.09	-3.84	-3.31	-2.01	-2.17	-1.85	-2.12	-1.94	-2.31	-3.38	-4.02	-3.33	-4.41	-5.03	-2.91	-3.23	-6.39	-3.65	-3.76	-6.22	-2.7
110	0.41	0.28	0.31	-0.23	-0.17	-0.01	-0.07	-0.08	-0.23	0.24	0.37	-0.39	0.19	0.24	0.11	0.22	0.19	-0.14	-0.11	-0.07	-0.04	-0.17	-0.27	0.15	0.17	0.13	0.34	-0.2	-0.4	-0.46	-0.17	0.19	-0.3	0.48	0.21	0.16	0.98	-0.24	-0.21	-0.03	-0.13	-0.06	-0.2	0.18	-0.13	-0.35	-0.22	0.16	0.59	0.34	-0.19	0.24	0.48	-0.22	0.36
111	-3.98	-4.02	-4.28	-6.29	-6.16	-5.66	-2.31	-3.16	-1.59	-1.22	-4.13	-2.24	-2.15	-3.78	-1.15	-4.16	-3.39	-1.74	-1.87	-1.66	-1.72	-1.82	-2.08	-3.38	-3.42	-3.01	-2.41	-3.6	-2.32	-2.15	-4.77	-2.15	-3.05	-4.14	-1.35	-4.54	-3.98	-2.08	-2.25	-1.98	-2.16	-2.11	-2.42	-3.58	-3.98	-3.52	-4.58	-5.5	-3.17	-3.27	-6.84	-3.78	-4.14	-6.54	-3.01
112	0.34	0.17	0.22	-0.19	-0.14	-0.04	-0.1	0.05	-0.11	0.21	0.27	-0.41	0.05	0.28	0.01	0.29	0.17	-0.18	-0.14	-0.02	0.03	-0.2	-0.15	0.15	0.03	0.04	0.17	-0.02	-0.47	-0.35	0	0.05	-0.48	0.29	0.18	0.28	-0.05	-0.15	-0.15	0.03	-0.04	-0.12	-0.12	0.32	-0.16	-0.1	-0.14	-0.06	0.54	0.39	-0.21	0.28	0.29	-0.25	0.38
113	2956	5567	5281	12746	2498	5549	17079	4168	8632	5248	6523	10881	3425	9071	6054	13533	3376	7319	6827	4918	5656	12019	11312	13584	2946	7235	3600	1209	3603	2800	2581	3425	1224	2525	1554	4133	1273	2053	2097	1332	1883	3073	3071	3215	1091	2441	16465	3830	4318	4498	10183	9071	2525	8432	4199
114	-0.05	-0.12	-0.15	-0.67	-0.68	-0.58	-0.08	-0.31	0.11	0.32	-0.19	-0.29	0	-0.13	0.29	-0.16	-0.17	-0.01	0.02	0.13	0.09	-0.01	-0.04	-0.15	-0.25	-0.22	0.07	-0.36	-0.1	0	-0.34	0	-0.4	-0.13	0.38	-0.18	-0.29	0	0.01	0.15	0.05	0.04	-0.03	-0.06	-0.37	-0.3	-0.37	-0.64	0.22	0.17	-0.73	-0.13	-0.13	-0.74	0.24
115	0.2	0.1	0.13	-0.11	-0.08	-0.02	-0.04	0.03	-0.06	0.13	0.16	-0.25	0.04	0.17	0.01	0.18	0.1	-0.09	-0.07	0	0.03	-0.1	-0.07	0.1	0.04	0.03	0.11	-0.02	-0.27	-0.19	0.01	0.04	-0.36	0.17	0.1	0.16	-0.02	-0.08	-0.08	0.02	-0.01	-0.05	-0.06	0.19	-0.08	-0.05	-0.07	-0.04	0.31	0.24	-0.12	0.17	0.17	-0.18	0.22
116	-3.26	-3.46	-3.7	-4.89	-5.07	-4.43	-2.91	-3.68	-2.34	-2.12	-3.52	-2.62	-2.77	-3.61	-2.08	-3.56	-3.4	-2.41	-2.6	-2.39	-2.43	-2.61	-2.78	-3.42	-3.47	-3.12	-2.95	-3.87	-2.7	-2.49	-3.95	-2.77	-3.27	-3.74	-2.02	-3.69	-3.8	-2.6	-2.69	-2.48	-2.6	-2.76	-2.97	-3.44	-3.94	-3.42	-4.18	-4.9	-3.09	-3.11	-5.25	-3.61	-3.74	-5.29	-2.95
117	-4.51	-4.01	-3.85	-5.33	-5.72	-5.33	-2.24	-3.43	-1.84	-1.58	-3.76	-1.77	-2.47	-3.5	-1.32	-3.79	-3.02	-1.63	-1.01	-3.02	-2.71	-1.68	-1.81	-2.77	-3.77	-3.34	-3.29	-4.79	-2.93	-3.15	-3.82	-2.47	-3.08	-4.55	-2.14	-4.85	-4.47	-3.05	-1.8	-4.2	-3.24	-3.12	-2.73	-4.14	-4.5	-3.78	-4.45	-5.53	-3.46	-3.99	-6.11	-3.5	-4.55	-5.97	-2.88
118	0.22	-0.14	-0.02	-0.27	-0.13	-0.07	0.08	-0.26	0.11	0.5	-0.15	-0.27	0.01	0.05	-0.17	0.33	0.31	-0.09	0.13	-0.11	-0.1	0.08	-0.11	0.17	-0.31	-0.21	0.01	-0.64	0	-0.1	0.79	0.01	0.38	-0.02	-0.01	0.25	-0.17	-0.52	0.33	-0.31	0.35	-0.38	-0.05	-0.23	-0.05	0.34	-0.08	-0.3	0.54	0.13	-0.44	0.05	-0.02	-0.37	0.32
119	0.31	0.05	0.03	0.23	0.34	0.43	-0.29	0.11	-0.24	-0.37	-0.12	-0.39	0.08	-0.2	-0.19	-0.13	0.34	-0.18	-0.27	0.05	0.01	-0.19	-0.21	-0.13	0.1	0.07	0.04	0	0.06	0.14	0.34	0.08	0.03	0.24	-0.02	0.13	0	-0.14	-0.22	0.01	0.1	0.08	0.06	-0.1	0	0.47	-0.1	0	-0.24	-0.28	0.59	-0.2	0.24	-0.2	-0.21
120	0.08	0.1	0	-0.67	-0.78	-0.49	-0.03	-0.09	0.17	0.48	0.11	-0.2	0.23	0.21	0.12	0.14	0.08	0.1	-0.01	0.2	0.14	0.1	0	0.04	-0.24	-0.04	0.21	-0.19	-0.22	-0.11	-0.19	0.23	-0.33	0.19	0.26	0.1	-0.17	0.1	-0.05	0.22	0.04	0.15	0	0.18	-0.46	-0.21	-0.35	-0.48	0.54	0.38	-0.65	0.21	0.19	-0.6	0.21
121	-0.89	-0.79	0.32	-0.43	-0.8	-0.79	0.43	0.14	-0.55	0.88	0.9	-0.3	0.19	0.31	0.82	0.23	0.08	0.46	-0.81	-0.08	0.12	-0.05	0.1	-0.12	0.51	-0.56	0.66	-2.34	0.63	-0.17	-1.03	0.19	-3.3	-0.37	0.79	-0.17	-0.52	0.95	-1.01	0.83	-0.62	1.17	0.8	0.67	1.1	-1.02	-0.79	-0.26	0.19	-0.47	-0.72	0.31	-0.37	-0.27	-0.08
122	0.43	-0.01	-0.29	-0.89	0.58	-1.43	0.23	-0.74	0.37	0.48	-0.1	0.01	0.42	-0.04	0.95	-0.2	-0.63	0.12	0.16	-0.36	0.84	-0.09	0.73	-0.43	0.87	-0.62	0.29	-0.66	-0.07	1.27	0.52	0.42	-2.26	-0.31	-0.01	-0.31	0.18	-0.06	0.93	-0.6	0.36	-0.28	-0.27	0.51	0.91	-1.34	-0.85	-1.02	-0.18	0.31	-0.76	-0.04	-0.31	-1.1	-0.01

Table S12 Amino acid descriptors (2D) used in this study.

	F	F	F	F	F	F	G	G	G	G	G	G	G	G	G	G	G	G	G	G	G	G	G	G	G	G	H	H
	R	S	T	V	W	Y	A	C	D	E	F	G	H	I	K	L	M	N	P	Q	R	S	T	V	W	Y	A	C
No.	FR	FS	FT	FV	FW	FY	GA	GC	GD	GE	GF	GG	GH	GI	GK	GL	GM	GN	GP	GQ	GR	GS	GT	GV	GW	GY	HA	HC
123	-0.9	-0.4	-0.7	-1.5	-2	-1.7	0	0	0	0	0	0	0	0	0	0	0	0	0	0	0	0	0	0	0	0	0	-0.6
124	-3	-2.8	-3.4	-4.6	-4.4	-4.1	0	0	0	0	0	0	0	0	0	0	0	0	0	0	0	0	0	0	0	0	-2.6	-3.3
125	-0.4	0	0	-0.8	-1.3	-0.5	0.1	0.1	-0.4	0.6	0.1	0.3	0.3	0.3	1.2	0.5	0.4	-0.1	0.4	-0.1	0.5	0	0	0.2	-0.5	-0.4	-0.1	-1.2
126	0.2	0.1	0.1	0	-0.1	0	0	0	0	0	0	0	0	0	0	0	0	0	0	0	0	0	0	0	0	0	0.2	0
127	-0.4	0.1	-0.2	-1.1	-1.5	-1	1.2	1.3	0.8	1.1	0.3	1.5	0.7	0.4	0.9	0.4	0.5	0.7	0.8	0.8	0.2	0.8	0.5	0.5	0	0.1	0.4	0.1
128	-0.4	0.1	-0.2	-0.9	-1.3	-0.9	1.4	0.9	0.8	1.1	0.4	1.7	0.7	0.5	0.7	0.7	0.6	0.6	1.1	0.8	0.3	0.9	0.6	0.8	0.2	0.3	0.5	-0.2
129	-0.4	0	-0.3	-0.9	-1.4	-1	1.2	0.1	0.7	0.9	0.3	1.1	0.3	0.4	0.6	0.6	0.2	0.4	0.8	0.5	0.2	0.8	0.6	0.7	-0.2	0.2	0.2	-1.4
130	-0.5	-0.7	-0.8	-1.3	-1.2	-1.2	0.1	-0.4	0.5	1	0.3	0.1	0.5	0.2	1	0.3	0.2	0.3	0.4	0.6	0.7	0.1	0.2	0.1	0.3	0.4	0.4	-1.2
131	-0.4	-0.6	-0.6	-1	-1.2	-1	0.4	-0.3	0.5	0.9	0.2	0.1	0.4	0.3	0.7	0.3	0.2	0.2	0.5	0.5	0.4	0.1	0.2	0.3	0.2	0.2	0.5	-1.1
132	-0.6	-0.8	-0.8	-1.4	-1.4	-1.3	0.3	-0.2	0.2	0.4	0.1	0.1	0.3	0.1	0.4	0.2	0.2	0.1	0.4	0.2	0.3	0	0	0.1	0.1	0	0	-1.3
133	5.7	5.4	5.7	5.6	6.2	5.9	4.6	5.5	4.5	4.5	5.4	3.9	4.5	5.5	5.9	5.9	4.7	5.1	5.6	5.1	6.7	4.5	5.9	5.2	5.8	4.3	5.2	5.4
134	6.1	5.6	6.1	6	6.5	6.4	4.7	5.3	4.7	5.6	6	4.5	5.4	5.1	4.7	4.9	5.8	5.6	4	5.9	6.3	4.4	5.5	4.6	5.2	4.8	5.2	5.9
135	5.9	5.5	5.9	6.1	6.3	6.1	4.5	5.2	6.1	5.3	5.3	4.5	5.3	5.2	4.9	5.7	5.3	5.3	4.4	7.2	6.7	5.1	5	4.9	4.5	6.2	5.3	6
136	0.0049	-0.0012	0.0041	8E-06	0.0061	-0.0033	-0.0031	0.0018	0.0015	-0.0001	0.0002	0.001	-0.001	0.0004	-0.0013	-0.0015	0.0023	-0.0016	0.0091	-0.0004	0.0025	-0.0035	0.0011	0.002	-0.0124	-0.0007	-0.0024	0.0546
137	0.0314	0.2801	0.3747	-0.0823	-0.1359	-0.0153	-0.0147	0.2042	-0.2244	0.1492	0.2129	-0.4812	0.086	0.3653	0.005	0.3062	0.1461	-0.3707	-0.1844	-0.0189	-0.0832	-0.2712	-0.2455	0.2311	0.2434	0.1064	0.3241	0.2438
138	0.28	0.0981	0.1481	-0.1227	-0.0572	-0.037	-0.0566	-0.1562	-0.1345	-0.0146	0.0527	-0.1405	-0.1707	0.2764	-0.1311	0.332	0.1519	-0.2072	-0.0569	-0.1651	-0.1149	-0.2097	-0.0864	0.142	-0.0944	-0.0093	0.0214	-0.0895
139	0.1521	-0.0129	0.0311	-0.0486	-0.1111	-0.0217	0.0261	-0.0687	-0.018	0.0457	0.0702	-0.0809	-0.0079	0.0267	0.0022	0.0138	-0.0218	-0.0279	-0.047	-0.0205	-0.0503	0.0087	-0.008	0.0234	0.1041	0.04	0.0646	-0.0512
140	0.0079	-0.0024	0.0108	-0.0063	-0.0297	0.0017	0.0337	-0.0242	-0.021	0.0338	0.0028	-0.0202	-0.0854	0.0007	0.0607	0.0372	0.0003	-0.0053	-0.0151	-0.0266	0.0093	-0.0257	-0.0163	0.0042	-0.0624	-0.0151	0.0165	-0.0659
141	0.009	0.0028	0.0046	-0.0101	-0.0094	-0.004	0.0011	-0.0036	-0.0024	0.0009	0.0038	-0.0053	-0.0039	0.0057	-7E-05	0.0066	0.0018	-0.0034	-0.0024	-0.0024	-0.0019	-0.0032	-0.0022	0.0037	0.0006	0.0011	0.0032	-0.0042
142	-0.39	-1.13	-1.08	-2.39	-2.4	-2.05	-1.74	-2.35	-0.67	0.37	-1.63	-1.68	-0.42	-2.15	-0.16	-1.87	-1.5	-0.62	-0.45	-0.01	-0.11	-1.11	-1.23	-1.98	-1.58	-1.05	-0.48	-1.21
143	-1.44	-1.77	-1.52	-3.21	-2.81	-2.73	-2.84	-3.17	-1.64	-1.07	-3.01	-2.6	-2.08	-2.93	-0.83	-2.98	-2.48	-1.69	-1.85	-1.6	-1.25	-2.03	-1.84	-2.78	-2.58	-2.69	-1.69	-2.31
144	0.24	0.17	0.08	-0.77	-1.02	-0.63	0.11	-0.56	0.75	1.16	0.09	0.35	0.53	0.01	1.08	0.13	0.54	0.79	0.74	0.73	0.65	0.61	0.38	0.04	-0.17	0.28	0.35	-0.83
145	-0.05	-0.92	-0.97	-2.16	-1.59	-1.97	-0.78	-1.67	0.29	1.15	-1.6	-1.32	0.01	-1.54	0.82	-1.27	-1.74	-0.16	0.08	0.92	1.09	0.04	-0.38	-1.29	-0.95	-0.56	0.44	-0.62
146	-0.04	-0.34	-0.22	-1.36	-1.33	-1.16	0.1	-0.26	1.13	1.62	0.12	-0.23	0.86	0.04	1.31	0.28	0.27	0.96	0.69	1.29	1.09	0.57	0.55	-0.06	0.07	0.4	0.69	-0.26
147	-0.07	-0.41	-0.08	-1.55	-1.04	-1.17	-0.24	-1.03	0.56	0.98	-0.11	-0.4	0.41	-0.37	0.61	-0.17	-0.2	-0.07	0.02	0.56	0.52	0.08	0.12	-0.12	-0.12	-0.21	0.07	-0.72
148	50.309	37.197	40.29	42.582	54.909	50.424	20.074	22.974	22.771	25.865	33.301	16.98	28.888	29.355	31.788	29.355	29.161	24.403	24.928	27.496	33.988	20.876	23.97	26.261	38.588	34.103	31.981	34.881
149	0	1	1	1	0	0	1	1	1	1	1	1	1	1	1	1	1	1	1	1	1	1	1	1	1	1	1	1
150	0	0	0	0	0	0	0	0	0	0	0	0	0	0	0	0	0	0	1	0	0	0	0	0	0	0	0	0
151	1	0	0	0	1	1	0	0	0	0	0	0	0	0	0	0	0	0	0	0	0	0	0	0	0	0	0	0
152	3	2	2	2	3	3	1	2	2	2	2	1	2	2	2	2	2	2	0	2	2	2	2	2	2	2	2	2
153	1	2	2	1	1	2	1	1	1	1	1	1	3	1	1	1	1	2	1	2	1	2	2	1	1	2	3	3
154	2	2	2	2	2	2	2	2	4	4	2	2	2	2	2	2	2	2	2	2	2	2	2	2	2	2	2	2
155	6	6	6	6	15	12	0	0	0	0	6	0	5	0	0	0	0	0	0	0	0	0	0	0	9	6	5	5
156	4	1	1	1	1	1	1	1	1	1	1	1	1	1	2	1	1	1	1	1	4	1	1	1	1	1	1	1
157	47	34	37	39	47	44	20	21	22	25	30	17	27	29	32	29	27	24	24	27	34	21	24	26	34	31	30	31
158	1	2	2	1	2	2	1	1	1	1	1	1	3	1	1	1	1	2	0	2	1	2	2	1	2	2	3	3
159	2	4	4	2	3	4	2	2	2	2	2	2	6	2	2	2	2	4	1	4	2	4	4	2	3	4	6	6
160	23	18	19	19	26	24	10	11	13	14	16	9	15	13	14	13	13	13	12	14	16	11	12	12	19	17	16	17
161	12	10	11	12	18	16	3	3	3	4	9	2	5	6	6	6	6	3	5	4	5	3	4	5	11	9	6	6
162	76.059	55.954	59.585	59.634	72.879	68.718	34.855	40.655	40.459	44.782	49.095	30.732	48.608	46.127	51.432	46.127	48.673	43.721	40.613	47.87	57.551	37.41	41.51	42.502	56.755	52.223	52.62	58.993
163	1.6183	1.6457	1.6104	1.5291	1.5506	1.5618	1.7427	1.9359	1.839	1.7913	1.6365	1.8078	1.8003	1.5906	1.6072	1.5906	1.8027	1.8217	1.6922	1.773	1.6927	1.7814	1.7296	1.6347	1.6693	1.6846	1.754	1.903
164	15	12	13	14	20	18	5	5	6	7	11	4	8	8	8	8	7	6	7	7	8	5	6	7	13	11	9	9
165	24	16	18	20	21	20	10	10	9	11	14	8	12	16	18	16	14	11	12	13	18	10	12	14	15	14	14	14
166	5	2	2	2	3	2	2	2	2	2	2	2	4	2	3	2	2	3	2	3	5	2	2	2	3	2	4	4
167	3	4	4	3	3	4	3	3	5	5	3	3	3	3	3	3	3	4	3	4	3	4	4	3	3	4	3	3
168	0	0	0	0	0	0	0	1	0	0	0	0	0	0	0	0	1	0	0	0	0	0	0	0	0	0	0	1
169	2.3208	2.3324	2.4013	2.4013	1.4918	1.7771	3.4944	3.7053	3.9668	3.9139	2.3797	3.0981	2.363	4.109	3.7869	3.9597	3.8113	3.9668	2.5244	3.9139	3.7786	3.7053	3.9772	3.9772	1.9026	2.3907	2.2686	2.3218
170	-2.6323	-2.561	-2.5947	-2.6503	-2.5682	-2.5718	-2.5126	-2.5178	-2.5263	-2.5826	-2.5369	-2.4369	-2.5211	-2.6803	-2.659	-2.6737	-2.5873	-2.5194	-2.5064	-2.5765	-2.6243	-2.5142	-2.575	-2.6431	-2.529	-2.5367	-2.5526	-2.5549
171	-0.5484	-0.5476	-0.5476	-0.53	-0.5577	-0.6144	-0.5193	-0.5694	-0.5497	-0.5498	-0.5816	-0.5315	-0.5668	-0.5077	-0.5173	-0.5166	-0.4872	-0.5546	-0.574	-0.557	-0.5477	-0.5677	-0.5304	-0.5088	-0.5477	-0.6431	-0.5537	-0.5477
172	0.5669	0.5811	0.5806	0.5256	0.5962	0.6586	0.3958	0.4678	0.4196	0.4199	0.5735	0.4051	0.4741	0.3913	0.395	0.3949	0.3702	0.4326	0.4182	0.435	0.4199	0.4702	0.4519	0.3916	0.5809	0.5731	0.4512	0.5392
173	2.6435	2.6179	2.6325	2.6442	2.6187	2.6181	2.5101	2.5222	2.5286	2.5678	2.533	2.4694	2.5378	2.6436	2.642	2.6311	2.5702	2.5508	2.7074	2.5852	2.6167	2.528	2.5811	2.6114	2.5361	2.5334	2.619	2.6217
174	-3.3115	-3.3117	-3.3117	-3.3116	-3.3116	-3.3116	-3.2903	-3.2905	-3.2904	-3.2904	-3.2904	-3.2901	-3.2904	-3.2905	-3.2906	-3.2904	-3.2904	-3.2904	-3.2884	-3.2904	-3.2904	-3.2905	-3.2906	-3.2905	-3.2904	-3.2904	-3.3115	-3.3116
175	-0.5128	-0.5381	-0.5381	-0.4865	-0.5129	-0.5381	-0.4762	-0.5883	-0.5248	-0.5245	-0.5291	-0.498	-0.5374	-0.4629	-0.4737	-0.4718	-0.4233	-0.5427	-0.5221	-0.5382	-0.5244	-0.6054	-0.5499	-0.4648	-0.5246	-0.5381	-0.4988	-0.5631
176	0.6107	0.5998	0.5423	0.5357	0.6974	0.7466	0.3144	0.4039	0.3644	0.3644	0.5506	0.3156	0.4403	0.3142	0.3143	0.3143	0.3756	0.3733	0.3028	0.3739	0.3644	0.4024	0.3889	0.3142	0.6576	0.4868	0.44	0.4778
177	2.5921	2.4179	2.4469	2.6128	2.4593	2.4517	2.356	2.3443	2.3606	2.5337	2.4258	2.1024	2.3805	2.6827	2.6638	2.6888	2.544	2.3707	2.6827	2.5386	2.5911	2.2562	2.4336	2.612	2.4525	2.4428	2.3925	2.3862
178	-2.3929	-2.3535	-2.3694	-2.4019	-2.354	-2.3546	-2.269	-2.2639	-2.274	-2.323	-2.2808	-2.2356	-2.2772	-2.4259	-2.4319	-2.4198	-2.3168	-2.2781	-2.2663	-2.3269	-2.3784	-2.2757	-2.3304	-2.3876	-2.2778	-2.2808	-2.3488	-2.3473
179	-0.412	-0.4175	-0.3902	-0.3762	-0.425	-0.477	-0.1942	-0.3342	-0.2898	-0.2898	-0.4169	-0.2042	-0.3504	-0.1883	-0.1932	-0.193	-0.0966	-0.3096	-0.149	-0.3115	-0.2898	-0.3427	-0.3079	-0.1888	-0.4061	-0.4687	-0.325	-0.4029
180	0.7082	0.7171	0.7128	0.6605	0.7528	0.8103	0.3845	0.598	0.5217	0.5219	0.7326	0.4011	0.6145	0.3772	0.3831	0.3829	0.4768	0.5579	0.3218	0.5613	0.5219	0.594	0.5544	0.3777	0.7431	0.7713	0.5933	0.6896
181	2.8741	2.8224	2.8488	2.8807	2.8348	2.8327	2.7486	2.789	2.7788	2.8223	2.787	2.6729	2.7819	2.8971	2.8745	2.8845	2.8477	2.791	2.966	2.83	2.8592	2.7601	2.8173	2.8646	2.793	2.7874	2.8149	2.8322
182	27.791	19.705	21.892	24.078	25.831	24.078	13.146	15.426	13.969	16.155	17.519	10.96	17.312	19.705	20.572	19.705	19.799	14.537	16.652	16.724	21.232	13.146	15.332	17.519	19.272	17.519	19.499	21.779
183	9	6	6	6	7	7	3	4	5	6	5	3	5	5	7	5	6	5	3	6	7	4	4	4	5	5	5	6
184	0.3913	0.3333	0.3158	0.3158	0.25	0.28	0.3333	0.4	0.4167	0.4615	0.3125	0.375	0.3333	0.4167	0.5385	0.4167	0.5	0.4167	0.25	0.4615	0.4667	0.4	0.3636	0.3636	0.25	0.2941	0.3125	0.3529
185	6	6	6	6	16	12	0	0	0	0	6	0	5	0	0	0	0	0	0	0	0	0	0	0	10	6	5	5
186	47	34	37	39	49	45	19	20	21	24	30	16	27	28	31	28	26	23	24	26	33	20	23	25	35	31	30	31
187	3	2	2	2	2	2	2	2	3	3	2	2	2	2	2	2	2	3	2	3	3	2	2	2	2	2	2	2
188	23	18	19	19	28	25	9	10	12	13	16	8	15	12	13	12	12	12	12	13	15	10	11	11	20	17	16	17
189	7	5	5	5	4	4	4	5	4	5	4	3	4	6	8	6	7	4	3	5	7	5	5	5	4	4	4	5
190	11	7	7	7	8	8	4	5	6	7	6	4	6	6	8	6	7	6	3	7	9	5	5	5	6	6	6	7
191	0.4783	0.3889	0.3684	0.3684	0.2857	0.32	0.4444	0.5	0.5	0.5385	0.375	0.5	0.4	0.5	0.6154	0.5	0.5833	0.5	0.25	0.5385	0.6	0.5	0.4545	0.4545	0.3	0.3529	0.375	0.4118
192	38	26	29	31	31	31	17	18	18	21	22	14	20	26	29	26	24	20	22	23	30	18	21	23	23	23	23	24
193	17.242	13.544	14.414	14.414	18.518	17.527	8.1463	8.8534	10.431	11.138	11.966	7.276	11.259	10.431	10.975	10.431	10.268	10.431	9.1378	11.138	12.552	8.8534	9.7236	9.7236	13.828	12.836	12.129	12.836
194	12.956	9.8347	10.705	11.258	14.274	13.144	5.7163	6.7651	6.7399	7.447	8.8102	4.8461	8.0253	8.0008	8.2518	8.0008	8.3553	6.909	6.7848	7.6161	8.9923	5.8706	6.7409	7.2937	10.31	9.18	8.8956	9.9443
195	8.8699	6.9557	7.8259	8.8259	11.342	10.265	3.2845	2.9916	3.4916	4.1987	6.3783	2.4142	4.6463	5.5689	5.1129	5.5689	4.6987	3.4916	4.4058	4.1987	4.9058	2.9916	3.8618	4.8618	7.3783	6.301	5.5165	5.2236
196	9.8281	7.8365	8.7067	9.7067	13.104	11.82	3.4392	3.1463	3.7236	4.4307	7.2591	2.5689	5.1378	5.7236	5.2676	5.7236	4.8534	3.7236	4.5605	4.4307	5.1378	3.1463	4.0165	5.0165	8.4138	7.1294	6.0081	5.7152
197	10.897	8.5409	8.9136	8.9136	12.542	11.452	4.5746	5.1126	5.9684	6.4684	7.6302	4.1639	7.1302	6.0233	6.6126	5.9684	6.1126	5.9684	5.6639	6.4684	7.4684	5.1126	5.4853	5.4853	9.1134	8.0241	7.5029	8.0409
198	7.3983	5.5336	5.978	6.2972	8.4756	7.6159	2.8706	3.6502	3.4633	3.9633	4.9658	2.423	4.4029	4.3193	4.7015	4.2645	5.2923	3.5479	3.9609	4.0479	4.8824	3.0178	3.4622	3.7813	5.9598	5.1001	4.8236	5.6032
199	4.6581	3.6581	4.1605	4.7379	6.3777	5.833	1.2196	1.0505	1.404	1.904	3.3147	0.7071	1.6927	2.6683	2.5505	2.6134	1.5505	1.404	2.0505	1.904	2.0505	1.0505	1.5529	2.1303	3.7701	3.2254	2.2052	2.0361
200	5.7079	4.7079	5.2103	5.7877	8.5076	7.7491	1.3189	1.1498	1.5581	2.0581	4.3746	0.8165	1.9663	2.7676	2.6498	2.7128	1.6498	1.5581	2.1498	2.0581	2.1498	1.1498	1.6523	2.2296	4.9495	4.1911	2.4688	2.2997
201	2	2	3	2	2	2	1	1	1	1	1	0	1	2	1	1	1	1	1	1	1	1	2	1	1	1	2	2
202	2	2	3	2	2	2	1	1	1	1	1	0	1	2	1	1	1	1	1	1	1	1	2	1	1	1	2	2
203	0.745	0.7755	0.7614	0.7229	0.7366	0.7458	0.824	0.8969	0.9133	0.8775	0.7602	0.8638	0.827	0.7509	0.7587	0.7509	0.8297	0.8691	0.8048	0.8394	0.7918	0.8722	0.8376	0.77	0.7737	0.7919	0.805	0.8543
204	14	10	10	10	14	14	6	6	7	8	9	6	8	7	9	7	8	7	6	8	10	6	6	6	10	10	9	9
205	1	0	0	0	0	0	0	0	-1	-1	0	0	0	0	1	0	0	0	0	0	1	0	0	0	0	0	0	0

	H	H	H	H	H	H	H	H	H	H	H	H	H	H	H	H	H	H	I	I	I	I	I	I	I	I	I
	D	E	F	G	H	I	K	L	M	N	P	Q	R	S	T	V	W	Y	A	C	D	E	F	G	H	I	K
No.	HD	HE	HF	HG	HH	HI	HK	HL	HM	HN	HP	HQ	HR	HS	HT	HV	HW	HY	IA	IC	ID	IE	IF	IG	IH	II	IK
123	-1.1	-1	-1.2	0	-1.6	-0.8	0	-0.7	-0.9	-0.8	-0.4	-0.5	-1	-0.6	-0.7	-0.5	-1.5	-1.5	-0.4	-0.8	0	-0.2	-1.7	0	-0.8	-1.5	0
124	-3.3	-2.8	-3.3	0	-3.5	-3.4	-2.2	-3.1	-3.5	-3.2	-2.6	-2.5	-2.9	-2.7	-3.2	-3.5	-3.6	-3.7	-3.9	-4.2	-2.9	-2.7	-4.5	0	-3.4	-4.9	-2.9
125	-1	-0.9	-1	0.3	-1.4	-0.2	0.3	-0.3	-0.9	-0.5	-0.5	0.9	-0.4	-0.6	-0.6	-0.2	-1.2	-0.8	-0.6	-1.1	0.6	0.4	-0.8	0.3	-0.2	-0.8	0.8
126	-0.7	-0.6	0.1	0	-0.8	0.4	0	0.4	0.2	-0.3	-0.1	-0.2	-0.2	-0.3	-0.1	0.5	0	-0.1	-0.1	0.2	0.5	0.5	0	0	0.4	-0.1	0.2
127	-0.1	0.1	-0.4	0.7	-0.8	-0.1	1	-0.1	-0.4	0.2	0	0.1	-0.1	0	0.1	0	-0.9	-0.8	-0.6	-0.5	0.5	0.4	-1.3	0.4	-0.1	-1.4	0.5
128	-0.2	-0.1	-0.3	0.7	-0.6	0	0.6	0	-0.3	0	0.3	-0.1	-0.1	0.1	0	-0.1	-0.7	-0.7	-0.3	-0.5	0.6	0.3	-1.1	0.5	0	-1.1	0.4
129	-0.4	-0.3	-0.6	0.3	-1.2	-0.3	0.1	-0.2	-0.9	-0.4	-0.2	-0.5	-0.4	-0.2	-0.2	-0.3	-1.2	-0.9	-0.3	-0.8	0.5	0.3	-1.1	0.4	-0.3	-1.1	0.3
130	-0.3	0	-0.8	0.5	-0.9	-0.8	0.3	-0.6	-0.7	-0.2	-0.6	-0.1	0	-0.6	-0.6	-0.7	-0.9	-0.6	-0.4	-0.2	0.9	0.7	-1.4	0.2	-0.8	-0.5	0.7
131	-0.6	-0.4	-0.6	0.4	-1	-0.5	0	-0.5	-0.6	-0.5	-0.5	-0.2	-0.4	-0.6	-0.5	-0.4	-0.8	-0.7	-0.1	-0.2	0.9	0.8	-1.1	0.3	-0.5	-0.3	1
132	-1	-1	-0.9	0.3	-1.5	-0.7	-0.6	-0.8	-0.9	-0.8	-0.6	-0.9	-0.9	-0.9	-0.9	-0.9	-1.1	-1.2	-0.4	-0.8	0.4	0.2	-1.4	0.1	-0.7	-1	0.2
133	5.2	5.5	5.5	4.5	5.3	5.9	5.2	5.7	5	5.4	5.5	5.2	5.7	5.3	5.5	5.4	5.5	5.7	5.2	5.2	5.7	5.6	6	5.5	5.9	5.8	5.8
134	5.8	5.7	6	5.4	6	5.9	5.6	5.8	5.9	5.5	5.5	6.2	5.9	5.4	5.6	5.6	6	6.2	5.5	5.5	5.6	5.9	6.2	5.1	5.9	6.2	5.9
135	5.5	5.8	6.1	5.3	6.2	6.3	5.9	5.8	5.7	5.5	5.4	6.8	5.5	5.5	5.9	5.8	6.6	5.7	5.5	5.9	6	5.9	6.4	5.2	6.3	6.4	5.9
136	-0.0025	-0.0072	-0.0002	-0.001	0.0013	-0.0005	0.0029	-0.0045	0.0318	0.0081	-0.002	0.0058	0.01	0.0099	-0.0059	-0.0069	-0.0067	0.0073	-0.0021	0.003	0.0027	0.0076	-0.0098	0.0004	-0.0005	0.0068	0.0009
137	-0.474	-0.1067	0.0392	0.086	-0.2332	0.1055	0.0036	0.1144	0.0142	-0.0042	-0.2788	-0.0305	-0.1089	-0.113	-0.2562	0.1579	-0.1723	0.0269	-0.2218	0.1253	0.4497	0.2195	-0.1911	0.3653	0.1055	-0.317	0.217
138	-0.5108	-0.3858	0.0943	-0.1707	-0.5506	0.5137	0.1505	0.314	0.0474	-0.221	-0.0532	-0.1302	0.0284	-0.1938	-0.089	0.3418	-0.028	0.0074	-0.0319	0.0828	0.6407	0.517	-0.0925	0.2764	0.5137	-0.3853	0.375
139	-0.1223	-0.0847	0.037	-0.0079	-0.3601	0.1672	0.054	0.1227	0.0377	-0.0712	-0.0585	-0.0724	-0.0224	-0.0645	-0.0351	0.0708	-0.1184	-0.0331	-0.1003	0.0085	0.1705	0.1435	-0.0888	0.0267	0.1672	-0.1275	0.1699
140	0.0135	0.0162	-0.0011	-0.0854	-0.117	0.0612	0.0743	-0.0018	0.0352	-0.0262	0.0233	-0.0011	0.0229	0.0024	0.0119	0.0164	-0.0561	-0.0158	-0.0442	0.0432	0.0087	0.0302	-0.025	0.0007	0.0612	-0.0344	4E-05
141	-0.0069	-0.0035	0.0022	-0.0039	-0.0202	0.0123	0.0029	0.0068	0.0026	-0.0036	-0.0017	-0.0022	7E-05	-0.0033	-0.0021	0.0067	-0.0062	-0.0011	-0.0075	-0.0051	0.0145	0.0141	-0.0138	0.0057	0.0123	-0.027	0.0131
142	-0.09	-0.09	-1.21	-0.42	-0.4	-1.18	0.4	-0.79	-1	0.1	0.31	0.43	0.34	-0.08	-0.4	-0.88	-0.78	-0.59	-2.09	-2.99	-0.65	-0.52	-2.46	-2.15	-1.18	-2.72	-0.56
143	-1.44	-1.17	-1.83	-2.08	-1.69	-1.86	-0.61	-1.86	-1.55	-0.67	-0.81	-1.04	-0.94	-1.53	-1.29	-1.7	-1.5	-1.74	-3.27	-3.73	-1.28	-1.48	-3.5	-2.93	-1.86	-3.5	-1.3
144	0.29	0.74	-0.28	0.53	0.28	-0.21	1.23	-0.03	0.19	0.91	0.59	0.83	0.47	0.46	0.31	0.09	-0.44	-0.18	-0.45	-1.27	0.47	0.7	-1.09	0.01	-0.21	-1.04	0.48
145	0.57	0.97	-0.88	0.13	1.52	-0.05	1.25	-0.46	0.07	0.63	1.11	1.02	0.94	0.75	0.2	-0.24	0.67	-0.57	-1.59	-2.77	0.59	0.2	-2.53	-1.4	-0.83	-2.64	0.1
146	0.69	1.01	-0.01	0.4	0.2	-0.16	1.39	-0.15	0.31	0.88	0.84	1.31	1.16	0.58	0.55	0.28	-0.62	-0.17	-0.81	-1.61	0.43	0.33	-1.64	-0.48	-0.43	-1.64	0.41
147	-0.51	0.58	-0.36	-0.12	-0.64	-0.5	0.95	-0.4	0.9	0.32	-0.45	0.91	0.51	0.06	0.52	-0.26	-0.32	-0.52	-1.64	-1.55	-0.23	0.2	-1.73	-1.58	-0.75	-1.88	0.24
148	34.678	37.772	45.208	28.888	40.795	41.262	43.695	41.262	41.068	36.31	36.835	39.403	45.895	32.783	35.877	38.168	50.495	46.01	32.448	35.348	35.145	38.239	45.675	29.355	41.262	41.729	44.163
149	1	1	0	1	0	1	1	1	1	0	1	0	0	0	0	1	0	0	1	1	1	1	1	1	1	1	1
150	0	0	0	0	0	0	0	0	0	0	0	0	0	0	0	0	0	0	0	0	0	0	0	0	0	0	0
151	0	0	1	0	2	0	0	0	0	2	0	2	1	2	2	0	2	3	0	0	0	0	0	0	0	0	0
152	2	2	3	2	4	2	2	2	2	4	2	4	3	4	4	2	4	5	2	2	2	2	2	2	2	2	2
153	3	3	3	3	5	3	3	3	3	4	3	4	3	4	4	3	3	4	1	1	1	1	1	1	3	1	1
154	4	4	2	2	2	2	2	2	2	2	2	2	2	2	2	2	2	2	2	2	4	4	2	2	2	2	2
155	5	5	11	5	10	5	5	5	5	5	5	5	5	5	5	5	14	11	0	0	0	0	6	0	5	0	0
156	1	1	1	1	1	1	2	1	1	1	1	1	4	1	1	1	1	1	1	1	1	1	1	1	1	1	2
157	32	35	40	27	37	39	42	39	37	34	34	37	44	31	34	36	44	41	32	33	34	37	42	29	39	41	44
158	3	3	3	3	5	3	3	3	3	4	2	4	3	4	4	3	4	4	1	1	1	1	1	1	3	1	1
159	6	6	6	6	10	6	6	6	6	8	5	8	6	8	8	6	7	8	2	2	2	2	2	2	6	2	2
160	19	20	22	15	21	19	20	19	19	19	18	20	22	17	18	18	25	23	14	15	17	18	20	13	19	17	18
161	6	7	12	5	8	9	9	9	9	6	8	7	8	6	7	8	14	12	7	7	7	8	13	6	9	10	10
162	59.065	63.258	66.46	48.608	65.466	63.918	68.986	63.918	66.882	61.541	58.165	65.6	74.682	55.748	59.754	60.25	73.653	69.998	49.657	56.122	56.658	60.376	63.012	46.127	63.918	59.846	65.474
163	1.8458	1.8074	1.6615	1.8003	1.7693	1.6389	1.6425	1.6389	1.8076	1.81	1.7107	1.773	1.6973	1.7983	1.7575	1.6736	1.6739	1.7073	1.5518	1.7007	1.6664	1.6318	1.5003	1.5906	1.6389	1.4597	1.4881
164	10	11	15	8	12	12	12	12	11	10	11	11	12	9	10	11	17	15	9	9	10	11	15	8	12	12	12
165	13	15	18	12	16	20	22	20	18	15	16	17	22	14	16	18	19	18	18	18	17	19	22	16	20	24	26
166	4	4	4	4	6	4	5	4	4	5	4	5	7	4	4	4	5	4	2	2	2	2	2	2	4	2	3
167	5	5	3	3	3	3	3	3	3	4	3	4	3	4	4	3	3	4	3	3	5	5	3	3	3	3	3
168	0	0	0	0	0	0	0	0	1	0	0	0	0	0	0	0	0	0	0	1	0	0	0	0	0	0	0
169	2.3906	2.3696	1.7781	2.1734	1.7722	2.4357	2.3296	2.3894	2.3445	2.3906	1.8674	2.3696	2.3044	2.3218	2.3963	2.3963	1.5013	1.7792	4.015	4.1604	4.3507	4.3147	2.4921	3.7618	2.4779	4.4543	4.2236
170	-2.5594	-2.597	-2.5653	-2.5205	-2.5565	-2.6836	-2.6622	-2.6766	-2.6004	-2.5551	-2.5548	-2.5922	-2.6309	-2.5524	-2.5909	-2.6491	-2.5609	-2.5651	-2.6732	-2.6732	-2.6737	-2.6774	-2.6741	-2.6694	-2.6734	-2.702	-2.6884
171	-0.5506	-0.5493	-0.5629	-0.5672	-0.562	-0.5103	-0.5476	-0.5385	-0.5477	-0.5477	-0.5477	-0.5477	-0.5476	-0.5476	-0.5476	-0.5142	-0.5573	-0.5902	-0.473	-0.5102	-0.5477	-0.5477	-0.5318	-0.5002	-0.5219	-0.434	-0.4654
172	0.4883	0.4927	0.5846	0.5031	0.5515	0.4307	0.4468	0.4444	0.4003	0.4578	0.5318	0.465	0.4934	0.5446	0.5024	0.4322	0.5905	0.6512	0.3726	0.4328	0.3857	0.3858	0.5488	0.3759	0.4394	0.3714	0.3724
173	2.6229	2.6312	2.6235	2.61	2.6246	2.6644	2.6584	2.6543	2.6316	2.6279	2.7256	2.6359	2.6465	2.6233	2.6367	2.6474	2.624	2.6235	2.7061	2.7068	2.7071	2.7087	2.7072	2.7032	2.7075	2.7151	2.7119
174	-3.3116	-3.3116	-3.3116	-3.3113	-3.3116	-3.3117	-3.3116	-3.3116	-3.3116	-3.3116	-3.3095	-3.3116	-3.3116	-3.3117	-3.3117	-3.3116	-3.3116	-3.3116	-3.3149	-3.315	-3.3149	-3.3149	-3.315	-3.3147	-3.315	-3.315	-3.3149
175	-0.5315	-0.5309	-0.5381	-0.5018	-0.5626	-0.4907	-0.4986	-0.4895	-0.4663	-0.5629	-0.5561	-0.5627	-0.5308	-0.5631	-0.5629	-0.4975	-0.5309	-0.5547	-0.3937	-0.461	-0.4578	-0.4483	-0.459	-0.4281	-0.4617	-0.3787	-0.3904
176	0.4565	0.4591	0.6785	0.4753	0.4868	0.4337	0.4387	0.4385	0.4182	0.4182	0.4588	0.4182	0.4593	0.474	0.4362	0.434	0.6912	0.6667	0.2959	0.3833	0.3367	0.3367	0.5411	0.2963	0.438	0.2958	0.2959
177	2.3943	2.5355	2.4333	2.3655	2.4017	2.683	2.6641	2.689	2.5453	2.3988	2.6845	2.5403	2.592	2.3721	2.4405	2.6127	2.4563	2.4475	2.6776	2.6775	2.6776	2.6781	2.6776	2.6773	2.6776	2.6901	2.6819
178	-2.3499	-2.3642	-2.3515	-2.3389	-2.3507	-2.4322	-2.4352	-2.4256	-2.3618	-2.3509	-2.3522	-2.3655	-2.392	-2.3504	-2.3674	-2.4012	-2.3509	-2.3515	-2.467	-2.4668	-2.4672	-2.4686	-2.4673	-2.4651	-2.4673	-2.4762	-2.4734
179	-0.3711	-0.3712	-0.4354	-0.343	-0.4353	-0.2974	-0.3191	-0.3149	-0.2669	-0.3471	-0.3737	-0.3546	-0.3712	-0.4172	-0.3436	-0.2995	-0.4249	-0.4637	-0.1718	-0.2988	-0.2572	-0.2572	-0.3903	-0.1753	-0.3049	-0.1701	-0.1715
180	0.6379	0.639	0.7278	0.6375	0.7015	0.5651	0.5869	0.5822	0.5347	0.6209	0.6577	0.6378	0.6392	0.6845	0.6086	0.5675	0.7496	0.8039	0.3539	0.5348	0.4774	0.4774	0.675	0.3597	0.5728	0.3519	0.3535
181	2.8272	2.8494	2.8302	2.7915	2.8281	2.9063	2.8848	2.8941	2.8655	2.8326	2.9757	2.854	2.8734	2.8193	2.8472	2.88	2.8326	2.8303	2.9088	2.9122	2.9112	2.9152	2.9115	2.9034	2.9112	2.9332	2.9222
182	20.322	22.508	23.872	17.312	23.665	26.058	26.925	26.058	26.152	20.89	23.005	23.077	27.585	19.499	21.685	23.872	25.625	23.872	21.892	24.172	22.715	24.901	26.265	19.705	26.058	28.451	29.317
183	7	8	7	5	7	7	9	7	8	7	5	8	9	6	6	6	7	7	5	6	7	8	7	5	7	7	9
184	0.3684	0.4	0.3043	0.3333	0.3182	0.3684	0.45	0.3684	0.4211	0.3684	0.2632	0.4	0.4091	0.3529	0.3333	0.3333	0.2593	0.2917	0.3846	0.4286	0.4375	0.4706	0.35	0.4167	0.3684	0.4375	0.5294
185	5	5	11	5	10	5	5	5	5	5	5	5	5	5	5	5	15	11	0	0	0	0	6	0	5	0	0
186	32	35	41	27	38	39	42	39	37	34	35	37	44	31	34	36	46	42	31	32	33	36	42	28	39	40	43
187	3	3	2	2	2	2	2	2	2	3	2	3	3	2	2	2	2	2	2	2	3	3	2	2	2	2	2
188	19	20	23	15	22	19	20	19	19	19	19	20	22	17	18	18	27	24	13	14	16	17	20	12	19	16	17
189	4	5	4	4	4	6	8	6	7	4	4	5	7	5	5	5	4	4	6	6	6	6	6	6	6	6	8
190	8	9	8	6	8	8	10	8	9	8	5	9	11	7	7	7	8	8	6	7	8	9	8	6	8	8	10
191	0.4211	0.45	0.3478	0.4	0.3636	0.4211	0.5	0.4211	0.4737	0.4211	0.2632	0.45	0.5	0.4118	0.3889	0.3889	0.2963	0.3333	0.4615	0.5	0.5	0.5294	0.4	0.5	0.4211	0.5	0.5882
192	24	27	28	20	26	32	35	32	30	26	28	29	36	24	27	29	29	29	29	30	30	33	34	26	32	38	41
193	14.414	15.121	15.949	11.259	15.242	14.414	14.958	14.414	14.251	14.414	13.121	15.121	16.535	12.836	13.707	13.707	17.811	16.82	11.301	12.008	13.585	14.293	15.121	10.431	14.414	13.585	14.129
194	9.9192	10.626	11.989	8.0253	11.205	11.18	11.431	11.18	11.535	10.088	9.9641	10.795	12.172	9.0499	9.9201	10.473	13.489	12.359	8.871	9.9198	9.8946	10.602	11.965	8.0008	11.18	11.155	11.407
195	5.7236	6.4307	8.6104	4.6463	6.8783	7.801	7.3449	7.801	6.9307	5.7236	6.6378	6.4307	7.1378	5.2236	6.0939	7.0939	9.6104	8.533	6.4392	6.1463	6.6463	7.3534	9.533	5.5689	7.801	8.7236	8.2676
196	6.2925	6.9996	9.8281	5.1378	7.7067	8.2925	7.8365	8.2925	7.4223	6.2925	7.1294	6.9996	7.7067	5.7152	6.5854	7.5854	10.983	9.6983	6.5939	6.301	6.8783	7.5854	10.414	5.7236	8.2925	8.8783	8.4223
197	8.8968	9.3968	10.559	7.0922	10.059	8.9516	9.5409	8.8968	9.0409	8.8968	8.5922	9.3968	10.397	8.0409	8.4136	8.4136	12.042	10.952	6.396	6.934	7.7898	8.2898	9.4516	5.9853	8.9516	7.8446	8.434
198	5.4163	5.9163	6.9188	4.376	6.3559	6.2723	6.6545	6.2175	7.2453	5.5009	5.9139	6.0009	6.8354	4.9708	5.4152	5.7343	7.9128	7.0531	4.74	5.5196	5.3327	5.8327	6.8352	4.2924	6.2723	6.1887	6.5709
199	2.3896	2.8896	4.3003	1.6927	2.6783	3.6539	3.5361	3.599	2.5361	2.3896	3.0361	2.8896	3.0361	2.0361	2.5385	3.1159	4.7557	4.211	3.1807	3.0116	3.3652	3.8652	5.2759	2.6683	3.6539	4.6294	4.5116
200	2.7079	3.2079	5.5244	1.9663	3.1162	3.9175	3.7997	3.8626	2.7997	2.7079	3.2997	3.2079	3.2997	2.2997	2.8021	3.3794	6.0993	5.3409	3.2701	3.101	3.5092	4.0092	6.3257	2.7676	3.9175	4.7187	4.601
201	2	2	2	1	2	3	2	2	2	2	2	2	2	2	3	2	2	2	3	3	3	3	3	2	3	4	3
202	2	2	2	1	2	3	2	2	2	2	2	2	2	2	3	2	2	2	3	3	3	3	3	2	3	4	3
203	0.8664	0.8451	0.7635	0.827	0.8114	0.7573	0.7626	0.7573	0.8129	0.838	0.7943	0.8194	0.7868	0.8366	0.8162	0.7708	0.7735	0.7869	0.7352	0.7905	0.8046	0.7875	0.7136	0.7509	0.7573	0.7014	0.7096
204	10	11	12	9	11	10	12	10	11	10	9	11	13	9	9	9	13	13	8	8	9	10	11	8	10	9	11
205	-1	-1	0	0	0	0	1	0	0	0	0	0	1	0	0	0	0	0	0	0	-1	-1	0	0	0	0	1

Table S12 Amino acid descriptors (2D) used in this study.

	F	F	F	F	F	F	G	G	G	G	G	G	G	G	G	G	G	G	G	G	G	G	G	G	G	G	H	H	H	H	H	H	H	H	H	H	H	H	H	H	H	H	H	H	H	H	I	I	I	I	I	I	I	I	I
	R	S	T	V	W	Y	A	C	D	E	F	G	H	I	K	L	M	N	P	Q	R	S	T	V	W	Y	A	C	D	E	F	G	H	I	K	L	M	N	P	Q	R	S	T	V	W	Y	A	C	D	E	F	G	H	I	K
No.	FR	FS	FT	FV	FW	FY	GA	GC	GD	GE	GF	GG	GH	GI	GK	GL	GM	GN	GP	GQ	GR	GS	GT	GV	GW	GY	HA	HC	HD	HE	HF	HG	HH	HI	HK	HL	HM	HN	HP	HQ	HR	HS	HT	HV	HW	HY	IA	IC	ID	IE	IF	IG	IH	II	IK
206	-0.8471	-0.8459	-0.8459	-0.8469	-0.863	-0.8468	-0.8494	-0.8489	-0.8506	-0.8543	-0.8491	-0.8498	-0.8495	-0.8491	-0.863	-0.8493	-0.8492	-0.849	-0.8527	-0.8493	-0.8493	-0.8485	-0.8484	-0.8491	-0.863	-0.8491	-0.8485	-0.8484	-0.8506	-0.8543	-0.8485	-0.8487	-0.8489	-0.8485	-0.863	-0.8485	-0.8485	-0.8485	-0.8512	-0.8485	-0.8485	-0.8484	-0.8484	-0.8485	-0.863	-0.8485	-0.8474	-0.8467	-0.8505	-0.8543	-0.847	-0.8481	-0.8488	-0.8471	-0.863
207	-0.3825	-0.39	-0.39	-0.362	-0.388	-0.3878	-0.414	-0.4433	-0.534	-0.5214	-0.4017	-0.4539	-0.4091	-0.3943	-0.398	-0.4025	-0.394	-0.4539	-0.4489	-0.4463	-0.4239	-0.4487	-0.4378	-0.3961	-0.4248	-0.4248	-0.3942	-0.3992	-0.4834	-0.4768	-0.39	-0.4213	-0.399	-0.3597	-0.3784	-0.3722	-0.3786	-0.3993	-0.3992	-0.3993	-0.3887	-0.3992	-0.3992	-0.3602	-0.3941	-0.394	-0.3604	-0.3948	-0.4657	-0.4655	-0.3885	-0.4062	-0.3892	-0.3221	-0.3445
208	0.1555	0.1855	0.1636	0.1011	0.1498	0.1512	0.1551	0.1853	0.131	0.1017	0.1247	0.1671	0.1823	0.1164	0.1849	0.0899	0.1244	0.1276	0.1778	0.1247	0.1853	0.1853	0.1726	0.1293	0.1853	0.1853	0.1776	0.1855	0.1589	0.149	0.1736	0.1783	0.1809	0.1419	0.1849	0.1117	0.1735	0.1737	0.1824	0.1735	0.1814	0.1855	0.1753	0.1546	0.1815	0.1815	0.0874	0.1315	0.1024	0.0981	0.0878	0.1186	0.1301	0.0833	0.1815
209	2.6259	2.4854	2.5363	2.5783	2.6516	2.5975	2.1222	2.1493	2.1732	2.262	2.3651	2.0064	2.3052	2.4198	2.4439	2.3993	2.2883	2.2318	2.4104	2.3103	2.4304	2.1524	2.2568	2.3346	2.4781	2.3801	2.4198	2.4341	2.447	2.4949	2.5524	2.3601	2.5174	2.5933	2.6001	2.5758	2.5097	2.4776	2.6047	2.5215	2.5923	2.4356	2.4925	2.539	2.621	2.561	2.5294	2.5412	2.5519	2.5909	2.6369	2.4792	2.6086	2.6722	2.675
210	-2.2352	-2.2351	-2.2352	-2.2352	-2.2352	-2.2352	-2.2348	-2.2348	-2.2348	-2.2348	-2.2348	-2.2348	-2.2348	-2.2348	-2.2353	-2.2348	-2.2348	-2.2348	-2.2349	-2.2348	-2.2349	-2.2348	-2.2348	-2.2348	-2.2348	-2.2348	-2.2351	-2.2351	-2.2351	-2.2352	-2.2352	-2.2351	-2.2352	-2.2352	-2.2354	-2.2352	-2.2351	-2.2351	-2.2352	-2.2352	-2.2352	-2.2351	-2.2351	-2.2352	-2.2352	-2.2352	-2.2353	-2.2353	-2.2353	-2.2353	-2.2353	-2.2353	-2.2353	-2.2353	-2.2355
211	-0.3871	-0.4294	-0.4206	-0.3424	-0.3782	-0.396	-0.487	-0.4836	-0.562	-0.537	-0.4275	-0.5375	-0.4597	-0.3804	-0.3666	-0.372	-0.4044	-0.5277	-0.4289	-0.4792	-0.4431	-0.5744	-0.509	-0.4182	-0.4024	-0.4228	-0.4117	-0.4486	-0.4994	-0.4535	-0.3988	-0.4644	-0.4122	-0.3635	-0.3535	-0.3588	-0.3514	-0.446	-0.4018	-0.4378	-0.4006	-0.4553	-0.4466	-0.3692	-0.379	-0.4003	-0.3596	-0.4219	-0.4367	-0.4351	-0.3268	-0.3872	-0.3602	-0.2902	-0.3028
212	0.1115	0.0592	0.0588	0.083	0.1411	0.1557	0.0542	0.0555	0.0542	0.0542	0.0849	0.0542	0.0555	0.0542	0.0542	0.0542	0.0555	0.0542	0.0542	0.0542	0.0542	0.0542	0.0542	0.0542	0.1232	0.0581	0.0557	0.058	0.0561	0.0567	0.1383	0.0683	0.0582	0.0546	0.0555	0.0555	0.058	0.0542	0.0542	0.0542	0.0568	0.0542	0.0542	0.0546	0.1361	0.129	0.0542	0.0555	0.0542	0.0542	0.0848	0.0542	0.0555	0.0542	0.0542
213	2.5312	2.3938	2.4407	2.5103	2.6377	2.5559	1.926	1.9615	1.97	2.136	2.3357	1.7255	2.1818	2.3757	2.3657	2.365	2.207	2.0222	2.3184	2.1776	2.298	1.9002	2.0692	2.251	2.5086	2.3708	2.2673	2.2807	2.2871	2.3612	2.4714	2.1989	2.3821	2.5117	2.4977	2.4982	2.3968	2.3102	2.4985	2.3841	2.4563	2.2616	2.3306	2.4285	2.5948	2.4933	2.4416	2.4508	2.4559	2.5051	2.5771	2.3928	2.5181	2.6119	2.5983
214	-0.6409	-0.6409	-0.6409	-0.6409	-0.6409	-0.6409	-0.6412	-0.6412	-0.6412	-0.6412	-0.6412	-0.6412	-0.6412	-0.6412	-0.6419	-0.6412	-0.6412	-0.6412	-0.6413	-0.6412	-0.6412	-0.6412	-0.6412	-0.6412	-0.6412	-0.6412	-0.6409	-0.6409	-0.6409	-0.6409	-0.6409	-0.6409	-0.6409	-0.6409	-0.6419	-0.6409	-0.6409	-0.6409	-0.641	-0.6409	-0.6409	-0.6409	-0.6409	-0.6409	-0.6409	-0.6409	-0.6411	-0.6411	-0.6411	-0.6411	-0.6411	-0.6411	-0.6411	-0.6411	-0.6419
215	-0.2107	-0.2161	-0.2161	-0.1991	-0.2127	-0.2161	-0.1922	-0.1759	-0.2036	-0.2026	-0.2114	-0.2044	-0.2126	-0.1792	-0.1881	-0.1854	-0.1748	-0.2176	-0.1932	-0.2165	-0.2028	-0.2212	-0.2045	-0.1808	-0.2102	-0.2161	-0.209	-0.2033	-0.2134	-0.2131	-0.2161	-0.2163	-0.2199	-0.1983	-0.2058	-0.2036	-0.2005	-0.2201	-0.2196	-0.2201	-0.2132	-0.2201	-0.2198	-0.1997	-0.2153	-0.2188	-0.1806	-0.1704	-0.1891	-0.1886	-0.2005	-0.1835	-0.2012	-0.1745	-0.1789
216	0.1634	0.1656	0.1582	0.1504	0.1712	0.185	0.0645	0.1578	0.1207	0.1208	0.1694	0.0849	0.158	0.0529	0.0624	0.062	0.155	0.1514	0.031	0.1525	0.1208	0.1543	0.1295	0.0536	0.1702	0.1931	0.1321	0.1647	0.1467	0.1467	0.1673	0.1557	0.1654	0.1168	0.1292	0.1275	0.1636	0.1623	0.1582	0.1635	0.1467	0.1629	0.1422	0.118	0.17	0.1841	0.052	0.1157	0.1044	0.1045	0.1454	0.055	0.1162	0.0485	0.0515
217	2.8406	2.7024	2.7534	2.799	2.909	2.8357	2.3272	2.3855	2.4013	2.4916	2.6209	2.192	2.5396	2.6404	2.6396	2.6188	2.5346	2.4474	2.6319	2.5289	2.6382	2.3546	2.4655	2.5533	2.7618	2.6352	2.6205	2.6485	2.6584	2.7085	2.7833	2.5552	2.734	2.8054	2.798	2.7868	2.7321	2.6834	2.8175	2.7301	2.797	2.6348	2.6952	2.7484	2.8761	2.7921	2.7146	2.738	2.7467	2.789	2.8507	2.658	2.8097	2.8722	2.8639
218	21.043	16.056	17.053	17.053	20.727	20.314	10	11	13	14	14.063	9	13.067	13	14	13	13	13	10.083	14	16	11	12	12	15.39	15.059	14.063	15.059	17.053	18.05	18.34	13.067	17.355	17.053	18.05	17.053	17.053	17.053	14.41	18.05	20.045	15.059	16.056	16.056	19.753	19.326	14	15	17	18	18.05	13	17.053	17	18
219	11.536	8.2268	8.3232	8.3232	9.9723	10.222	4.7603	5.625	6.4533	7.3125	7.35	4.8395	6.554	6.4533	8.32	6.4533	7.4082	6.4533	4.2969	7.3125	9.0741	5.625	5.6122	5.6122	7.1358	7.438	6.6667	7.438	8.3232	9.1065	9.3333	6.554	8.585	8.3232	9.8496	8.3232	9.0313	8.3232	6.4379	9.1065	10.714	7.438	7.5556	7.5556	9.2739	9.4746	6.4775	7.3025	8.1633	8.9917	9.1065	6.4533	8.3232	8.1633	9.8685
220	9.1571	5.6805	5.8776	5.8776	5.75	7.0388	4.48	4.4444	6.1224	7.04	5.2645	5.8776	4.5714	4.6875	7.04	6.1224	6.1224	6.1224	2.25	7.04	8.8166	4.4444	4.5918	4.5918	3.9862	5.4444	4.8166	5.0176	6.321	7.0255	6.2041	5.04	5.6055	5.4792	7.0255	6.321	6.321	6.321	3.5262	7.0255	8.4444	5.0176	5.2675	5.2675	5.2585	6.428	4.8889	5.04	6.4793	7.259	6.12	5.3333	5.4792	5.4444	7.259
221	15.438	12.119	13.08	13.08	14.662	14.814	7.7182	10.158	9.8973	10.889	10.211	6.722	9.375	10.711	11.709	10.711	12.164	9.4552	8.1615	10.442	11.976	8.7152	9.7128	9.7128	10.92	11.163	10.323	12.457	12.504	13.461	13.064	9.375	12.246	13.198	14.164	13.198	14.41	12.122	10.981	13.073	14.595	11.276	12.235	12.235	13.859	13.989	11.709	14.167	13.871	14.866	14.045	10.711	13.198	14.705	15.705
222	8.3342	6.0983	6.2827	6.2827	6.9483	7.3438	3.5554	5.1497	4.79	5.5673	5.212	3.4644	4.5793	5.2233	6.8549	5.2233	6.8904	4.5554	3.4044	5.3185	6.6485	4.3415	4.4465	4.4465	4.9576	5.4009	4.7832	6.0744	5.9886	6.6775	6.5312	4.5793	5.9423	6.3426	7.623	6.3426	7.5556	5.7939	4.8208	6.4734	7.6734	5.46	5.6578	5.6578	6.4023	6.7487	5.3369	6.8672	6.5694	7.3365	6.9852	5.2233	6.3426	6.9931	8.5365
223	6.5387	4.1544	4.3811	4.3811	3.9631	5.0025	3.2566	4.0375	4.447	5.2724	3.6669	4.0359	3.125	3.7372	5.7339	4.8812	5.6659	4.2127	1.7528	5.0222	6.3568	3.3505	3.5772	3.5772	2.724	3.8885	3.3967	4.056	4.4809	5.0881	4.2858	3.4453	3.8229	4.125	5.3825	4.7587	5.2469	4.3283	2.6064	4.9262	5.9756	3.625	3.8935	3.8935	3.5863	4.523	3.9819	4.7229	5.1572	5.8697	4.6453	4.2521	4.125	4.628	6.2395
224	5.594	4.106	4.3252	4.3252	3.9183	4.5328	2.7441	4.7557	3.6468	4.3302	3.3262	2.5875	2.8621	4.3036	5.7333	4.3036	6.447	3.3133	2.3154	3.9668	4.9764	3.4397	3.599	3.599	2.8494	3.5464	3.086	4.451	3.9411	4.4942	3.8782	2.8621	3.4652	4.4057	5.3986	4.4057	5.7303	3.6965	2.941	4.2313	5.0908	3.6217	3.8457	3.8457	3.5492	4.1046	4.4636	6.486	5.3601	6.0591	4.9054	4.3036	4.4057	6.0492	7.4479
225	8	6	6	5	6	6	5	5	7	7	5	5	7	5	6	5	5	7	5	7	8	6	6	5	6	6	7	7	9	9	7	7	9	7	8	7	7	9	7	9	10	8	8	7	8	8	5	5	7	7	5	5	7	5	6
226	9	5	5	4	5	5	4	4	4	4	4	4	5	4	7	4	4	6	3	6	9	5	5	4	5	5	5	5	5	5	5	5	6	5	8	5	5	7	4	7	10	6	6	5	6	6	4	4	4	4	4	4	5	4	7
227	1	1	1	1	1	1	1	1	1	1	1	1	1	1	1	1	1	1	1	1	1	1	1	1	1	1	1	1	1	1	1	1	1	1	1	1	1	1	1	1	1	1	1	1	1	1	1	1	1	1	1	1	1	1	1
228	1	0	0	0	0	0	0	0	0	0	0	0	0	0	1	0	0	1	0	1	1	0	0	0	0	0	0	0	0	0	0	0	1	0	1	0	0	1	0	1	1	1	1	0	1	1	0	0	0	0	0	0	0	0	1
229	0.234	-0.449	0.013	1.558	2.487	1.813	-1.411	-1.301	-2.038	-1.596	0.124	-1.873	-2.18	0.003	-1.342	0.003	-0.756	-2.893	-1.023	-2.451	-1.763	-2.446	-1.984	-0.439	0.49	-0.184	-1.718	-1.608	-2.345	-1.903	-0.183	-2.18	-2.487	-0.304	-1.649	-0.304	-1.063	-3.2	-1.33	-2.758	-2.07	-2.753	-2.291	-0.746	0.183	-0.491	0.465	0.575	-0.162	0.28	2	0.003	-0.304	1.879	0.534
230	-2.2154	-1.3132	-1.6404	-2.2465	-3.635	-2.9832	-0.0136	-0.6748	0.1754	-0.0264	-1.5157	0.3136	-0.2729	-0.9323	0.0414	-1.2458	-0.88	0.1532	-0.1328	-0.0486	-0.3861	0.5162	0.189	-0.4171	-1.8056	-1.1538	-0.6001	-1.2613	-0.4111	-0.6129	-2.1023	-0.2729	-0.8594	-1.5189	-0.5452	-1.8323	-1.4665	-0.4334	-0.7193	-0.6351	-0.9726	-0.0703	-0.3975	-1.0036	-2.3921	-1.7403	-1.2596	-1.9208	-1.0706	-1.2723	-2.7617	-0.9323	-1.5189	-2.1783	-1.2046
231	8.7202	6.6361	7.0741	7.389	10.042	9.1286	3.4799	4.2263	4.1172	4.6009	6.0331	3.0252	5.2531	4.8877	5.2393	4.8776	5.1586	4.3061	4.2333	4.7871	5.7177	3.6331	4.0783	4.4079	7.0765	6.1575	5.6983	6.4538	6.3489	6.8305	8.2453	5.2454	7.4574	7.1066	7.4545	7.0965	7.384	6.5255	6.4484	7.005	7.9263	5.8605	6.3043	6.6261	9.2815	8.3761	5.3254	6.0823	5.9846	6.4588	7.8648	4.88	7.1066	6.7164	7.073
232	7	6	6	6	16	12	0	0	0	0	6	0	5	0	0	0	0	0	5	0	1	0	0	0	10	6	5	5	5	5	11	5	10	5	5	5	5	5	10	5	6	5	5	5	15	11	0	0	0	0	6	0	5	0	0
233	0	1	1	1	1	1	1	1	1	1	1	1	1	1	0	1	1	0	1	0	0	1	1	1	1	1	1	1	0	0	1	1	0	1	0	1	1	0	1	0	0	0	0	1	1	1	1	1	1	1	1	1	1	1	1
234	1	1	1	1	3	2	0	0	0	0	1	0	1	0	0	0	0	0	1	0	0	0	0	0	2	1	1	1	1	1	2	1	2	1	1	1	1	1	2	1	1	1	1	1	3	2	0	0	0	0	1	0	1	0	0
235	10	6	6	6	7	7	3	4	5	6	5	3	5	5	7	5	6	5	3	6	8	4	4	4	5	5	5	6	7	8	7	5	7	7	9	7	8	7	5	8	10	6	6	6	7	7	5	6	7	8	7	5	7	7	9
236	2	0	0	0	0	0	1	1	1	1	0	1	1	0	2	0	0	2	0	2	2	1	1	0	0	0	0	0	2	2	0	1	2	0	2	0	0	3	0	3	3	2	2	0	1	1	0	0	1	1	0	0	0	0	1
237	8.625	5.527	5.527	4.847	6.081	6.123	3.953	4.363	4.859	4.859	4.847	3.953	4.82	3.953	5.806	3.953	4.413	5.323	3.883	5.323	7.731	4.633	4.633	3.953	5.187	5.229	4.82	5.23	5.726	5.726	5.714	4.82	5.687	4.82	6.673	4.82	5.28	6.19	4.75	6.19	8.598	5.5	5.5	4.82	6.054	6.096	3.953	4.363	4.859	4.859	4.847	3.953	4.82	3.953	5.806
238	-7.625	-5.527	-5.527	-4.847	-6.082	-6.123	-3.953	-4.363	-5.859	-5.859	-4.847	-3.953	-4.82	-3.953	-4.806	-3.953	-4.413	-5.323	-3.883	-5.323	-6.731	-4.633	-4.633	-3.953	-5.188	-5.229	-4.82	-5.23	-6.726	-6.726	-5.714	-4.82	-5.687	-4.82	-5.673	-4.82	-5.28	-6.19	-4.75	-6.19	-7.598	-5.5	-5.5	-4.82	-6.055	-6.096	-3.953	-4.363	-5.859	-5.859	-4.847	-3.953	-4.82	-3.953	-4.806
239	4.303	2.7888	2.8222	2.5403	2.9897	2.9805	2.0989	2.2343	2.1584	2.1921	2.4077	2.0626	2.6502	2.2537	3.496	2.2539	2.264	2.7019	2.1036	2.7214	3.9637	2.4495	2.4829	2.201	2.6503	2.6411	2.6868	2.8222	2.7462	2.78	2.9956	2.6504	3.2381	2.8416	4.0839	2.8418	2.8519	3.2898	2.6915	3.3093	4.5516	3.0374	3.0708	2.7888	3.2382	3.229	2.2644	2.3997	2.3238	2.3575	2.5731	2.228	2.8157	2.4192	3.6614
240	-3.303	-2.7888	-2.8222	-2.5403	-2.9897	-2.9805	-2.0989	-2.2343	-3.1584	-3.1921	-2.4077	-2.0626	-2.6502	-2.2537	-2.496	-2.2539	-2.264	-2.7019	-2.1036	-2.7214	-2.9637	-2.4495	-2.4829	-2.201	-2.6503	-2.6411	-2.6868	-2.8222	-3.7462	-3.78	-2.9956	-2.6504	-3.2381	-2.8416	-3.0839	-2.8418	-2.8519	-3.2898	-2.6915	-3.3093	-3.5516	-3.0374	-3.0708	-2.7888	-3.2382	-3.229	-2.2644	-2.3997	-3.3238	-3.3575	-2.5731	-2.228	-2.8157	-2.4192	-2.6614
241	0.0803	0.1239	0.1224	0.136	0.1156	0.1159	0.1645	0.1546	0.16	0.1575	0.1434	0.1674	0.1303	0.1532	0.0988	0.1532	0.1525	0.1278	0.1642	0.1269	0.0871	0.141	0.1391	0.1569	0.1303	0.1307	0.1286	0.1224	0.1258	0.1243	0.1154	0.1304	0.1067	0.1216	0.0846	0.1216	0.1212	0.105	0.1284	0.1044	0.0759	0.1138	0.1125	0.1239	0.1067	0.107	0.1526	0.144	0.1487	0.1466	0.1343	0.1551	0.1227	0.1428	0.0944
242	0.1658	0.1964	0.194	0.2156	0.1832	0.1838	0.2609	0.2451	0.1741	0.1722	0.2275	0.2657	0.2067	0.243	0.2194	0.243	0.2419	0.2027	0.2604	0.2013	0.1848	0.2236	0.2205	0.2488	0.2066	0.2074	0.2038	0.1941	0.1467	0.1455	0.1828	0.2068	0.1691	0.1927	0.1776	0.1927	0.192	0.1665	0.2035	0.1655	0.1542	0.1803	0.1783	0.1964	0.1691	0.1696	0.2419	0.2282	0.1654	0.1638	0.2128	0.246	0.1945	0.2264	0.2058
243	34.294	17.232	30.028	47.09	51.417	25.763	12.796	19.208	31.941	31.77	8.5308	0	33.978	38.388	25.592	38.388	29.858	8.5308	38.785	25.763	17.062	0	12.796	29.858	34.185	8.5308	38.073	44.485	57.217	57.047	33.807	25.277	59.255	63.665	50.869	63.665	55.134	33.807	64.061	51.04	42.338	25.277	38.073	55.134	59.461	33.807	51.185	57.597	70.329	70.159	46.919	38.388	72.366	76.777	63.981
244	65.17	66.929	52.617	43.447	64.774	68.967	22.12	22.12	22.12	22.12	43.447	36.432	54.322	22.12	43.843	22.12	22.12	30.821	22.12	22.12	43.843	45.602	31.29	22.12	43.447	47.64	63.023	63.023	63.023	63.023	84.35	77.335	95.225	63.023	84.746	63.023	63.023	71.724	63.023	63.023	84.746	86.505	72.193	63.023	84.35	88.543	22.12	22.12	22.12	22.12	43.447	36.432	54.322	22.12	43.843
245	4.2654	4.2654	4.2654	4.2654	4.2654	4.2654	8.5308	16.312	8.5308	8.5308	8.5308	8.5308	12.796	8.5308	8.5308	8.5308	8.5308	8.5308	8.5308	8.5308	8.5308	8.5308	8.5308	8.5308	8.5308	8.5308	8.5308	16.312	8.5308	8.5308	8.5308	8.5308	12.796	8.5308	8.5308	8.5308	8.5308	8.5308	8.5308	8.5308	8.5308	8.5308	8.5308	8.5308	8.5308	8.5308	4.2654	12.046	4.2654	4.2654	4.2654	4.2654	8.5308	4.2654	4.2654
246	11.765	11.765	11.765	11.765	20.384	11.765	21.811	21.811	21.811	21.811	21.811	21.811	30.43	21.811	21.811	21.811	21.811	39.049	13.192	39.049	21.811	21.811	21.811	21.811	30.43	21.811	20.384	20.384	20.384	20.384	20.384	20.384	29.003	20.384	20.384	20.384	20.384	37.622	11.765	37.622	20.384	20.384	20.384	20.384	29.003	20.384	11.765	11.765	11.765	11.765	11.765	11.765	20.384	11.765	11.765
247	0	10.324	10.324	0	0	10.324	0	0	0	0	0	0	0	0	0	0	0	12.95	0	12.95	0	10.324	10.324	0	0	10.324	0	0	0	0	0	0	0	0	0	0	0	12.95	0	12.95	0	10.324	10.324	0	0	10.324	0	0	0	0	0	0	0	0	0
248	56.045	12.95	12.95	12.95	12.95	12.95	12.95	12.95	12.95	12.95	12.95	12.95	12.95	12.95	12.95	12.95	12.95	12.95	12.95	12.95	56.045	12.95	12.95	12.95	12.95	12.95	12.95	12.95	12.95	12.95	12.95	12.95	12.95	12.95	12.95	12.95	12.95	12.95	12.95	12.95	56.045	12.95	12.95	12.95	12.95	12.95	12.95	12.95	12.95	12.95	12.95	12.95	12.95	12.95	12.95
249	41.539	25.857	25.857	25.857	25.857	25.857	25.857	25.857	25.857	25.857	25.857	25.857	25.857	25.857	51.714	25.857	25.857	25.857	25.857	25.857	41.539	25.857	25.857	25.857	25.857	25.857	25.857	25.857	25.857	25.857	25.857	25.857	25.857	25.857	51.714	25.857	25.857	25.857	25.857	25.857	41.539	25.857	25.857	25.857	25.857	25.857	25.857	25.857	25.857	25.857	25.857	25.857	25.857	25.857	51.714
250	19.611	2.2085	20.956	2.2085	25.373	37.628	18.748	0	0	17.403	10.91	0	0	0	26.104	8.7013	40.102	0	17.403	8.7013	17.403	0	18.748	0	23.165	35.42	18.748	0	0	17.403	10.91	0	0	0	26.104	8.7013	40.102	0	17.403	8.7013	17.403	0	18.748	0	23.165	35.42	27.449	8.7013	8.7013	26.104	19.611	8.7013	8.7013	8.7013	34.805
251	61.275	61.275	61.275	98.77	98.039	85.784	0	0	0	0	61.275	0	0	46.197	0	37.495	0	0	0	0	0	0	0	37.495	36.765	24.51	0	0	0	0	61.275	0	0	46.197	0	37.495	0	0	0	0	0	0	0	37.495	36.765	24.51	37.495	37.495	37.495	37.495	98.77	37.495	37.495	83.692	37.495
252	0	0	0	0	0	0	0	26.363	0	0	0	0	0	0	0	0	19.761	0	0	0	0	0	0	0	0	0	0	26.363	0	0	0	0	0	0	0	0	19.761	0	0	0	0	0	0	0	0	0	0	26.363	0	0	0	0	0	0	0
253	0	0	0	0	0	0	0	0	0	0	0	0	5.6826	0	0	0	0	0	0	0	0	0	0	0	0	0	5.6826	5.6826	5.6826	5.6826	5.6826	5.6826	11.365	5.6826	5.6826	5.6826	5.6826	5.6826	5.6826	5.6826	5.6826	5.6826	5.6826	5.6826	5.6826	5.6826	0	0	0	0	0	0	5.6826	0	0
254	27.006	13.567	13.567	13.567	13.567	13.567	13.567	13.567	13.567	13.567	13.567	13.567	13.567	13.567	13.567	13.567	13.567	27.134	13.567	27.134	27.006	13.567	13.567	13.567	13.567	13.567	13.567	13.567	13.567	13.567	13.567	13.567	13.567	13.567	13.567	13.567	13.567	27.134	13.567	27.134	27.006	13.567	13.567	13.567	13.567	13.567	13.567	13.567	13.567	13.567	13.567	13.567	13.567	13.567	13.567
255	28.376	36.144	36.144	28.376	28.513	36.144	28.376	28.376	55.51	55.51	28.376	28.376	28.513	28.376	29.482	28.376	28.376	35.027	28.239	35.027	28.376	36.144	36.144	28.376	28.513	36.144	28.513	28.513	55.647	55.647	28.513	28.513	28.65	28.513	29.618	28.513	28.513	35.164	28.376	35.164	28.513	36.281	36.281	28.513	28.65	36.281	28.376	28.376	55.51	55.51	28.376	28.376	28.513	28.376	29.482
256	0.5621	0.6235	0.6467	0.7199	0.7656	0.7032	0.5099	0.5672	0.4389	0.4851	0.6568	0.4526	0.6031	0.6292	0.5389	0.6292	0.6378	0.4328	0.5537	0.4776	0.4153	0.4345	0.4852	0.5974	0.6858	0.597	0.6321	0.6633	0.5674	0.594	0.7169	0.6031	0.6799	0.6998	0.6267	0.6998	0.705	0.5589	0.6559	0.5852	0.522	0.5734	0.6014	0.6807	0.7356	0.6686	0.6564	0.6856	0.5891	0.6144	0.7357	0.6292	0.6998	0.7197	0.6455
257	0.3901	0.4312	0.4716	0.4957	0.4795	0.5199	0.3684	0.3661	0.3592	0.4128	0.4851	0.2843	0.219	0.4047	0.296	0.4047	0.4567	0.3095	0.3278	0.3249	0.2782	0.2844	0.3565	0.3961	0.3963	0.447	0.2826	0.2883	0.2849	0.3295	0.3922	0.219	0.1856	0.3258	0.2465	0.3258	0.3667	0.2505	0.2589	0.2657	0.2366	0.2263	0.2829	0.3144	0.3288	0.3655	0.4548	0.4458	0.439	0.4742	0.5247	0.4047	0.3258	0.4663	0.3796
258	0.1585	0.1894	0.1777	0.1455	0.1219	0.1493	0.2546	0.2248	0.3592	0.3297	0.1783	0.2843	0.219	0.1926	0.1843	0.1926	0.1881	0.3095	0.2314	0.285	0.2117	0.2844	0.2589	0.2091	0.1635	0.2027	0.203	0.1857	0.2849	0.2674	0.1562	0.219	0.1856	0.1656	0.1607	0.1656	0.1627	0.2505	0.1896	0.2355	0.1842	0.2263	0.2115	0.1762	0.146	0.1758	0.1785	0.1633	0.2631	0.2469	0.1373	0.1926	0.1656	0.1456	0.1417
259	0.4379	0.3765	0.3533	0.2801	0.2344	0.2968	0.4901	0.4328	0.5611	0.5149	0.3432	0.5474	0.3969	0.3708	0.4611	0.3708	0.3622	0.5672	0.4463	0.5224	0.5847	0.5655	0.5148	0.4026	0.3142	0.403	0.3679	0.3367	0.4326	0.406	0.2831	0.3969	0.3201	0.3002	0.3733	0.3002	0.295	0.4411	0.3441	0.4148	0.478	0.4266	0.3986	0.3193	0.2644	0.3314	0.3436	0.3144	0.4109	0.3856	0.2643	0.3708	0.3002	0.2803	0.3545
260	0.6099	0.5688	0.5284	0.5043	0.5205	0.4801	0.6316	0.6339	0.6408	0.5872	0.5149	0.7157	0.781	0.5953	0.704	0.5953	0.5433	0.6905	0.6722	0.6751	0.7218	0.7156	0.6435	0.6039	0.6037	0.553	0.7174	0.7117	0.7151	0.6705	0.6078	0.781	0.8144	0.6742	0.7535	0.6742	0.6333	0.7495	0.7411	0.7343	0.7634	0.7737	0.7171	0.6856	0.6712	0.6345	0.5452	0.5542	0.561	0.5258	0.4753	0.5953	0.6742	0.5337	0.6204
261	0.2793	0.1872	0.1756	0.1346	0.1124	0.1475	0.2355	0.208	0.2018	0.1852	0.165	0.2631	0.1779	0.1782	0.2768	0.1782	0.1741	0.2577	0.2148	0.2373	0.373	0.2811	0.2559	0.1935	0.1508	0.2003	0.1649	0.1509	0.1476	0.1386	0.1269	0.1779	0.1344	0.1346	0.2126	0.1346	0.1322	0.1907	0.1545	0.1793	0.2938	0.2002	0.1871	0.1431	0.1183	0.1555	0.1651	0.1511	0.1478	0.1387	0.127	0.1782	0.1346	0.1347	0.2128
262	196.38	163.67	180.91	207.55	264.25	234.17	84.006	105.81	84.403	101.63	154.5	66.774	131.53	137.05	125.88	137.05	142.18	86.932	100.03	104.16	108.65	75.944	93.176	119.82	176.52	146.44	148.76	170.57	149.16	166.39	219.26	131.53	196.28	201.8	190.63	201.8	206.94	151.68	164.78	168.92	173.4	140.7	157.93	184.57	241.27	211.19	154.28	176.09	154.68	171.91	224.78	137.05	201.8	207.32	196.15
263	136.27	113.19	131.94	142.92	165.49	173.12	60.691	68.306	69.077	86.48	114.13	41.943	47.763	88.14	69.152	88.14	101.81	62.161	59.209	70.862	72.785	49.711	68.458	79.438	102.01	109.64	66.51	74.126	74.896	92.299	119.95	47.763	53.582	93.959	74.972	93.959	107.63	67.981	65.028	76.682	78.604	55.53	74.278	85.258	107.83	115.46	106.89	114.5	115.27	132.68	160.32	88.14	93.959	134.34	115.35
264	55.382	49.711	49.711	41.943	42.08	49.711	41.943	41.943	69.077	69.077	41.943	41.943	47.763	41.943	43.049	41.943	41.943	62.161	41.806	62.161	55.382	49.711	49.711	41.943	42.08	49.711	47.763	47.763	74.896	74.896	47.763	47.763	53.582	47.763	48.868	47.763	47.763	67.981	47.626	67.981	61.202	55.53	55.53	47.763	47.899	55.53	41.943	41.943	69.077	69.077	41.943	41.943	47.763	41.943	43.049
265	152.97	98.841	98.841	80.75	80.887	98.841	80.75	80.75	107.88	107.88	80.75	80.75	86.569	80.75	107.71	80.75	80.75	113.92	80.613	113.92	152.97	98.841	98.841	80.75	80.887	98.841	86.569	86.569	113.7	113.7	86.569	86.569	92.389	86.569	113.53	86.569	86.569	119.74	86.432	119.74	158.78	104.66	104.66	86.569	86.706	104.66	80.75	80.75	107.88	107.88	80.75	80.75	86.569	80.75	107.71
266	213.08	149.32	147.81	145.37	179.65	159.89	104.06	118.26	123.21	123.04	121.13	105.58	170.33	129.66	164.44	129.66	121.13	138.69	121.43	147.22	188.83	125.07	123.56	121.13	155.4	135.64	168.82	183.01	187.96	187.79	185.88	170.33	235.09	194.41	229.19	194.41	185.88	203.44	186.19	211.97	253.58	189.83	188.31	185.88	220.15	200.4	128.14	142.33	147.29	147.12	145.2	129.66	194.41	153.73	188.52
267	97.583	49.131	49.131	38.807	38.807	49.131	38.807	38.807	38.807	38.807	38.807	38.807	38.807	38.807	64.664	38.807	38.807	51.756	38.807	51.756	97.583	49.131	49.131	38.807	38.807	49.131	38.807	38.807	38.807	38.807	38.807	38.807	38.807	38.807	64.664	38.807	38.807	51.756	38.807	51.756	97.583	49.131	49.131	38.807	38.807	49.131	38.807	38.807	38.807	38.807	38.807	38.807	38.807	38.807	64.664
268	0.5	0.5	0.5	0.5	0.5	0.5	0.5	0.5	0.4286	0.5	0.4444	0.5	0.5	0.4286	0.4444	0.4286	0.5	0.4286	0.5	0.5	0.5	0.5	0.5	0.5	0.5	0.5	0.4444	0.4444	0.5	0.4545	0.5	0.4444	0.4545	0.5	0.5	0.5	0.4545	0.5	0.4444	0.4545	0.4615	0.4444	0.4444	0.4444	0.4615	0.4615	0.5	0.5	0.4444	0.5	0.4545	0.5	0.5	0.4444	0.4545
269	1	1	1	1	1	1	1	1	0.75	1	0.8	1	1	0.75	0.8	0.75	1	0.75	1	1	1	1	1	1	1	1	0.8	0.8	1	0.8333	1	0.8	0.8333	1	1	1	0.8333	1	0.8	0.8333	0.8571	0.8	0.8	0.8	0.8571	0.8571	1	1	0.8	1	0.8333	1	1	0.8	0.8333
270	8.625	5.527	5.527	4.847	6.081	6.123	3.953	4.363	4.859	4.859	4.847	3.953	4.82	3.953	5.806	3.953	4.413	5.323	3.883	5.323	7.731	4.633	4.633	3.953	5.187	5.229	4.82	5.23	5.726	5.726	5.714	4.82	5.687	4.82	6.673	4.82	5.28	6.19	4.75	6.19	8.598	5.5	5.5	4.82	6.054	6.096	3.953	4.363	4.859	4.859	4.847	3.953	4.82	3.953	5.806
271	-7.625	-5.527	-5.527	-4.847	-6.082	-6.123	-3.953	-4.363	-5.859	-5.859	-4.847	-3.953	-4.82	-3.953	-4.806	-3.953	-4.413	-5.323	-3.883	-5.323	-6.731	-4.633	-4.633	-3.953	-5.188	-5.229	-4.82	-5.23	-6.726	-6.726	-5.714	-4.82	-5.687	-4.82	-5.673	-4.82	-5.28	-6.19	-4.75	-6.19	-7.598	-5.5	-5.5	-4.82	-6.055	-6.096	-3.953	-4.363	-5.859	-5.859	-4.847	-3.953	-4.82	-3.953	-4.806
272	0.1391	0.1639	0.1639	0.1869	0.149	0.148	0.2292	0.2077	0.1865	0.1865	0.1869	0.2292	0.188	0.2292	0.156	0.2292	0.2053	0.1702	0.2333	0.1702	0.1552	0.1956	0.1956	0.2292	0.1747	0.1733	0.188	0.1732	0.1582	0.1582	0.1586	0.188	0.1593	0.188	0.1358	0.188	0.1716	0.1464	0.1907	0.1464	0.1396	0.1647	0.1647	0.188	0.1497	0.1486	0.2292	0.2077	0.1865	0.1865	0.1869	0.2292	0.188	0.2292	0.156
273	0.1268	0.1628	0.1628	0.1857	0.148	0.147	0.2277	0.2063	0.1536	0.1536	0.1857	0.2277	0.1867	0.2277	0.1873	0.2277	0.2039	0.1691	0.2318	0.1691	0.1437	0.1943	0.1943	0.2277	0.1735	0.1721	0.1867	0.1721	0.1338	0.1338	0.1575	0.1867	0.1583	0.1867	0.1586	0.1867	0.1705	0.1454	0.1895	0.1454	0.1273	0.1636	0.1636	0.1867	0.1486	0.1476	0.2277	0.2063	0.1536	0.1536	0.1857	0.2277	0.1867	0.2277	0.1873
274	0.4486	0.4657	0.5345	0.6281	0.6158	0.6237	0.2882	0.1729	0.1725	0.2406	0.5015	0.2051	0.3199	0.4616	0.3261	0.4616	0.2445	0.1652	0.3263	0.2311	0.2253	0.14	0.2695	0.4153	0.4459	0.4481	0.4124	0.3182	0.3148	0.3569	0.5479	0.366	0.4134	0.5205	0.4134	0.5205	0.3546	0.3049	0.4319	0.3464	0.3266	0.3018	0.3859	0.49	0.5011	0.505	0.5438	0.4383	0.4323	0.4672	0.6491	0.5077	0.5205	0.6278	0.515
275	0.3403	0.4312	0.4046	0.3657	0.5218	0.4937	0.2546	0.3661	0.4045	0.3712	0.4481	0.2843	0.2952	0.1926	0.1843	0.1926	0.2768	0.3095	0.2314	0.285	0.2117	0.2844	0.2589	0.2091	0.453	0.4115	0.2735	0.3529	0.3812	0.3578	0.4181	0.2952	0.3007	0.2232	0.2153	0.2232	0.2866	0.3117	0.2557	0.293	0.2342	0.294	0.2747	0.2374	0.4239	0.3906	0.1785	0.266	0.2962	0.278	0.3451	0.1926	0.2232	0.1456	0.1417
276	0.1585	0.1894	0.1777	0.1455	0.17	0.1493	0.2546	0.3661	0.3592	0.3297	0.1783	0.2843	0.2952	0.1926	0.1843	0.1926	0.2768	0.3095	0.2314	0.285	0.2117	0.2844	0.2589	0.2091	0.228	0.2027	0.2735	0.3529	0.3481	0.3267	0.2105	0.2952	0.3007	0.2232	0.2153	0.2232	0.2866	0.3117	0.2557	0.293	0.2342	0.294	0.2747	0.2374	0.2473	0.2284	0.1785	0.266	0.2631	0.2469	0.1373	0.1926	0.2232	0.1456	0.1417
277	0.5514	0.5343	0.4655	0.3719	0.3842	0.3763	0.7118	0.8271	0.8275	0.7594	0.4985	0.7949	0.6801	0.5384	0.6739	0.5384	0.7555	0.8348	0.6737	0.7689	0.7747	0.86	0.7305	0.5847	0.5541	0.5519	0.5876	0.6818	0.6852	0.6431	0.4521	0.634	0.5866	0.4795	0.5866	0.4795	0.6454	0.6951	0.5681	0.6536	0.6734	0.6982	0.6141	0.51	0.4989	0.495	0.4562	0.5617	0.5677	0.5328	0.3509	0.4923	0.4795	0.3722	0.485
278	0.6597	0.5688	0.5954	0.6343	0.4782	0.5063	0.7454	0.6339	0.5955	0.6288	0.5519	0.7157	0.7048	0.8074	0.8157	0.8074	0.7232	0.6905	0.7686	0.715	0.7883	0.7156	0.7411	0.7909	0.547	0.5885	0.7265	0.6471	0.6188	0.6422	0.5819	0.7048	0.6993	0.7768	0.7847	0.7768	0.7134	0.6883	0.7443	0.707	0.7658	0.706	0.7253	0.7626	0.5761	0.6094	0.8215	0.734	0.7038	0.722	0.6549	0.8074	0.7768	0.8544	0.8583
279	0.3929	0.345	0.2878	0.2264	0.2141	0.227	0.4572	0.461	0.4682	0.4297	0.3202	0.5106	0.3849	0.3459	0.4896	0.3459	0.4787	0.5253	0.4423	0.4838	0.563	0.5756	0.4716	0.3756	0.3261	0.3492	0.314	0.3289	0.3371	0.3164	0.2416	0.3388	0.2859	0.2563	0.3713	0.2563	0.3588	0.3835	0.3124	0.3606	0.4391	0.4042	0.3394	0.2726	0.2516	0.2667	0.2778	0.2957	0.3046	0.2859	0.2137	0.2997	0.2563	0.2266	0.3433
280	156.71	122.25	149.53	181.07	212.55	207.7	47.486	32.254	33.174	50.406	117.98	30.254	69.778	100.53	76.169	100.53	54.501	33.174	58.937	50.406	58.937	24.473	51.751	83.295	114.77	109.92	97.057	81.825	82.745	99.977	167.56	79.825	119.35	150.1	125.74	150.1	104.07	82.745	108.51	99.977	108.51	74.044	101.32	132.87	164.35	159.49	127.81	112.57	113.49	130.73	198.3	110.57	150.1	180.85	156.49

Table S12 Amino acid descriptors (2D) used in this study.

	F	F	F	F	F	F	G	G	G	G	G	G	G	G	G	G	G	G	G	G	G	G	G	G	G	G	H	H	H	H	H	H	H	H	H	H	H	H	H	H	H	H	H	H	H	H	I	I	I	I	I	I	I	I	I
	R	S	T	V	W	Y	A	C	D	E	F	G	H	I	K	L	M	N	P	Q	R	S	T	V	W	Y	A	C	D	E	F	G	H	I	K	L	M	N	P	Q	R	S	T	V	W	Y	A	C	D	E	F	G	H	I	K
No.	FR	FS	FT	FV	FW	FY	GA	GC	GD	GE	GF	GG	GH	GI	GK	GL	GM	GN	GP	GQ	GR	GS	GT	GV	GW	GY	HA	HC	HD	HE	HF	HG	HH	HI	HK	HL	HM	HN	HP	HQ	HR	HS	HT	HV	HW	HY	IA	IC	ID	IE	IF	IG	IH	II	IK
281	118.87	113.19	113.19	105.43	180.1	164.42	41.943	68.306	77.778	77.778	105.43	41.943	64.372	41.943	43.049	41.943	61.704	62.161	41.806	62.161	55.382	49.711	49.711	41.943	116.62	100.94	64.372	90.735	100.21	100.21	127.85	64.372	86.8	64.372	65.477	64.372	84.132	84.59	64.235	84.59	77.811	72.139	72.139	64.372	139.05	123.37	41.943	68.306	77.778	77.778	105.43	41.943	64.372	41.943	43.049
282	55.382	49.711	49.711	41.943	58.689	49.711	41.943	68.306	69.077	69.077	41.943	41.943	64.372	41.943	43.049	41.943	61.704	62.161	41.806	62.161	55.382	49.711	49.711	41.943	58.689	49.711	64.372	90.735	91.505	91.505	64.372	64.372	86.8	64.372	65.477	64.372	84.132	84.59	64.235	84.59	77.811	72.139	72.139	64.372	81.117	72.139	41.943	68.306	69.077	69.077	41.943	41.943	64.372	41.943	43.049
283	192.63	140.27	130.22	107.22	132.59	125.31	117.27	154.31	159.11	159.11	117.27	117.27	148.32	117.27	157.42	117.27	168.43	167.68	121.71	167.68	202.68	150.31	140.27	117.27	142.63	135.36	138.27	175.31	180.11	180.11	138.27	138.27	169.32	138.27	178.43	138.27	189.43	188.68	142.71	188.68	223.68	171.31	161.27	138.27	163.64	156.36	107.22	144.26	149.07	149.07	107.22	107.22	138.27	107.22	147.38
284	230.48	149.32	166.55	182.87	165.04	168.59	122.81	118.26	114.51	131.74	129.83	105.58	153.72	175.85	190.54	175.85	161.23	138.69	138.84	155.92	206.23	125.07	142.31	158.62	140.79	144.34	170.96	166.4	162.65	179.88	177.97	153.72	201.87	224	238.69	224	209.37	186.83	186.98	204.06	254.38	173.22	190.45	206.77	188.94	192.49	193.09	188.53	184.78	202.01	200.1	175.85	224	246.13	260.82
285	137.25	90.556	80.509	65.28	73.899	75.604	75.326	86.004	90.035	90.035	75.326	75.326	83.945	75.326	114.38	75.326	106.73	105.51	79.9	105.51	147.3	100.6	90.556	75.326	83.945	85.651	73.899	84.576	88.608	88.608	73.899	73.899	82.518	73.899	112.95	73.899	105.3	104.09	78.472	104.09	145.87	99.175	89.128	73.899	82.518	84.223	65.28	75.957	79.989	79.989	65.28	65.28	73.899	65.28	104.33
286	7	5	5	5	7	7	3	3	4	4	5	3	4	4	5	4	4	4	3	4	5	3	3	3	5	5	5	5	5	6	6	5	6	5	6	5	6	5	5	6	7	5	5	5	7	7	4	4	5	5	6	4	5	5	6
287	0	0	0	0	0	0	0	1	0	0	0	0	0	0	0	0	0	0	0	0	0	0	0	0	0	0	0	1	0	0	0	0	0	0	0	0	0	0	0	0	0	0	0	0	0	0	0	1	0	0	0	0	0	0	0
288	1	1	1	1	3	2	0	0	0	0	1	0	1	0	0	0	0	0	1	0	0	0	0	0	2	1	1	1	1	1	2	1	2	1	1	1	1	1	2	1	1	1	1	1	3	2	0	0	0	0	1	0	1	0	0
289	0.1391	0.1639	0.1639	0.1869	0.149	0.148	0.2292	0.2077	0.1865	0.1865	0.1869	0.2292	0.188	0.2292	0.156	0.2292	0.2053	0.1702	0.2333	0.1702	0.1552	0.1956	0.1956	0.2292	0.1747	0.1733	0.188	0.1732	0.1582	0.1582	0.1586	0.188	0.1593	0.188	0.1358	0.188	0.1716	0.1464	0.1907	0.1464	0.1396	0.1647	0.1647	0.188	0.1497	0.1486	0.2292	0.2077	0.1865	0.1865	0.1869	0.2292	0.188	0.2292	0.156
290	0.1268	0.1628	0.1628	0.1857	0.148	0.147	0.2277	0.2063	0.1536	0.1536	0.1857	0.2277	0.1867	0.2277	0.1873	0.2277	0.2039	0.1691	0.2318	0.1691	0.1437	0.1943	0.1943	0.2277	0.1735	0.1721	0.1867	0.1721	0.1338	0.1338	0.1575	0.1867	0.1583	0.1867	0.1586	0.1867	0.1705	0.1454	0.1895	0.1454	0.1273	0.1636	0.1636	0.1867	0.1486	0.1476	0.2277	0.2063	0.1536	0.1536	0.1857	0.2277	0.1867	0.2277	0.1873
291	-4.4821	-2.9334	-2.5449	-1.2697	-0.2018	-0.9775	-3.5171	-3.6072	-5.397	-5.0069	-2.2943	-3.9056	-3.5712	-2.4909	-4.1248	-2.4909	-2.7839	-4.6616	-3.0308	-4.2715	-6.0934	-4.5447	-4.1562	-2.881	-1.813	-2.5887	-3.1827	-3.2728	-5.0626	-4.6725	-1.96	-3.5712	-3.2369	-2.1565	-3.7904	-2.1565	-2.4495	-4.3272	-2.6964	-3.9371	-5.759	-4.2103	-3.8218	-2.5466	-1.4787	-2.2544	-2.1024	-2.1925	-3.9823	-3.5922	-0.8796	-2.4909	-2.1565	-1.0762	-2.7101
292	176.17	118.98	118.98	93.591	93.591	118.98	93.591	93.591	136.27	136.27	93.591	93.591	93.591	93.591	126.49	93.591	93.591	126.49	75.581	126.49	176.17	118.98	118.98	93.591	93.591	118.98	93.591	93.591	136.27	136.27	93.591	93.591	93.591	93.591	126.49	93.591	93.591	126.49	75.581	126.49	176.17	118.98	118.98	93.591	93.591	118.98	93.591	93.591	136.27	136.27	93.591	93.591	93.591	93.591	126.49
293	8.6219	6.7228	6.7228	6.7228	6.7228	6.7228	6.7228	6.7228	8.1868	8.1868	6.7228	6.7228	23.508	6.7228	6.7228	6.7228	6.7228	11.982	9.8471	11.982	8.6219	6.7228	6.7228	6.7228	6.7228	6.7228	23.508	23.508	24.972	24.972	23.508	23.508	40.294	23.508	23.508	23.508	23.508	28.767	26.633	28.767	25.407	23.508	23.508	23.508	23.508	23.508	6.7228	6.7228	8.1868	8.1868	6.7228	6.7228	23.508	6.7228	6.7228
294	55.688	55.688	62.157	55.688	72.474	55.688	51.706	51.706	75.569	75.569	51.706	47.724	68.492	51.706	51.706	51.706	51.706	75.569	51.706	75.569	51.706	51.706	58.175	51.706	68.492	51.706	72.474	72.474	96.336	96.336	72.474	68.492	89.259	72.474	72.474	72.474	72.474	96.336	72.474	96.336	72.474	72.474	78.943	72.474	89.259	72.474	55.688	55.688	79.551	79.551	55.688	51.706	72.474	55.688	55.688
295	18.44	20.926	0	0	0	0	18.44	35.673	18.44	18.44	18.44	36.879	18.44	18.44	36.879	18.44	35.673	18.44	36.879	18.44	36.879	39.366	18.44	18.44	18.44	18.44	0	17.234	0	0	0	18.44	0	0	18.44	0	17.234	0	18.44	0	18.44	20.926	0	0	0	0	0	17.234	0	0	0	18.44	0	0	18.44
296	3.1856	3.1856	3.1856	7.5964	6.3712	6.3712	0	0	0	0	3.1856	0	2.7567	4.4108	0	4.4108	0	0	0	0	0	0	0	4.4108	3.1856	3.1856	2.7567	2.7567	2.7567	2.7567	5.9423	2.7567	5.5135	7.1675	2.7567	7.1675	2.7567	2.7567	2.7567	2.7567	2.7567	2.7567	2.7567	7.1675	5.9423	5.9423	4.4108	4.4108	4.4108	4.4108	7.5964	4.4108	7.1675	8.8216	4.4108
297	18.868	18.868	18.868	18.868	37.737	37.737	0	0	0	0	18.868	0	18.868	0	0	0	31.691	0	0	0	0	0	0	0	18.868	18.868	18.868	18.868	18.868	18.868	37.737	18.868	37.737	18.868	18.868	18.868	50.56	18.868	18.868	18.868	18.868	18.868	18.868	18.868	37.737	37.737	0	0	0	0	18.868	0	18.868	0	0
298	88.216	88.216	88.216	88.216	180.72	158.79	0	0	0	0	88.216	0	34	0	0	0	0	0	0	0	0	0	0	0	92.504	70.573	34	34	34	34	122.22	34	68	34	34	34	34	34	34	34	34	34	34	34	126.5	104.57	0	0	0	0	88.216	0	34	0	0
299	37.737	0	0	0	0	0	0	46.107	18.868	37.737	0	0	0	18.868	56.605	18.868	18.868	18.868	37.737	37.737	37.737	0	0	0	0	0	0	46.107	18.868	37.737	0	0	0	18.868	56.605	18.868	18.868	18.868	37.737	37.737	37.737	0	0	0	0	0	18.868	64.976	37.737	56.605	18.868	18.868	18.868	37.737	75.474
300	0	0	33.326	66.652	0	5.2434	33.326	0	0	0	0	0	0	66.652	0	66.652	32.22	0	0	0	0	0	33.326	66.652	0	5.2434	33.326	0	0	0	0	0	0	66.652	0	66.652	32.22	0	0	0	0	0	33.326	66.652	0	5.2434	99.978	66.652	66.652	66.652	66.652	66.652	66.652	133.3	66.652
301	8.2116	6.0846	6.5441	6.8598	9.5763	8.5571	3.0367	3.8547	3.4317	3.8934	5.4839	2.5772	4.6985	4.4148	4.6299	4.4148	4.7693	3.8504	3.7497	4.3121	5.3049	3.1779	3.6374	3.9531	6.6695	5.6504	5.1579	5.9759	5.5529	6.0146	7.6052	4.6985	6.8197	6.536	6.7512	6.536	6.8906	5.9717	5.871	6.4334	7.4262	5.2991	5.7586	6.0743	8.7908	7.7716	4.8743	5.6923	5.2693	5.731	7.3215	4.4148	6.536	6.2524	6.4675
302	90.408	90.408	90.408	90.408	90.408	90.408	90.408	90.408	156.95	156.95	90.408	90.408	90.408	90.408	90.408	90.408	90.408	114.27	90.408	114.27	90.408	90.408	90.408	90.408	90.408	90.408	90.408	90.408	156.95	156.95	90.408	90.408	90.408	90.408	90.408	90.408	90.408	114.27	90.408	114.27	90.408	90.408	90.408	90.408	90.408	90.408	90.408	90.408	156.95	156.95	90.408	90.408	90.408	90.408	90.408
303	31.672	25.385	25.385	0	0	25.385	0	0	0	0	0	0	16.786	0	0	0	0	0	3.1243	0	31.672	25.385	25.385	0	0	25.385	16.786	16.786	16.786	16.786	16.786	16.786	33.571	16.786	16.786	16.786	16.786	16.786	19.91	16.786	48.457	42.171	42.171	16.786	16.786	42.171	0	0	0	0	0	0	16.786	0	0
304	68.919	50.908	50.908	55.319	67.693	50.908	50.908	50.908	50.908	50.908	50.908	50.908	67.693	55.319	83.805	55.319	50.908	50.908	32.897	50.908	68.919	50.908	50.908	55.319	67.693	50.908	67.693	67.693	67.693	67.693	67.693	67.693	84.479	72.104	100.59	72.104	67.693	67.693	49.683	67.693	85.704	67.693	67.693	72.104	84.479	67.693	55.319	55.319	55.319	55.319	55.319	55.319	72.104	59.73	88.216
305	11.15	11.15	17.618	11.15	14.335	19.579	3.982	3.982	3.982	3.982	7.1675	0	6.7387	3.982	3.982	3.982	3.982	3.982	3.982	3.982	3.982	3.982	10.451	3.982	7.1675	12.411	10.721	10.721	10.721	10.721	13.906	6.7387	13.477	10.721	10.721	10.721	10.721	10.721	10.721	10.721	10.721	10.721	17.189	10.721	13.906	19.15	7.9639	7.9639	7.9639	7.9639	11.15	3.982	10.721	7.9639	7.9639
306	32.897	0	0	0	4.7171	0	0	0	0	0	0	0	0	0	0	0	0	32.897	0	32.897	32.897	0	0	0	4.7171	0	0	0	0	0	0	0	0	0	0	0	0	32.897	0	32.897	32.897	0	0	0	4.7171	0	0	0	0	0	0	0	0	0	0
307	144.82	107.08	107.08	107.08	213.74	196.53	0	0	18.868	37.737	107.08	0	52.868	18.868	56.605	18.868	18.868	18.868	37.737	37.737	37.737	0	0	0	106.66	89.441	52.868	52.868	71.737	90.605	159.95	52.868	105.74	71.737	109.47	71.737	71.737	71.737	90.605	90.605	90.605	52.868	52.868	52.868	159.52	142.31	18.868	18.868	37.737	56.605	125.95	18.868	71.737	37.737	75.474
308	27.061	27.649	6.7228	6.7228	6.7228	6.7228	25.162	42.396	26.626	26.626	25.162	43.602	25.162	25.162	43.602	25.162	42.396	30.421	43.602	30.421	45.501	46.089	25.162	25.162	25.162	25.162	6.7228	23.956	8.1868	8.1868	6.7228	25.162	6.7228	6.7228	25.162	6.7228	23.956	11.982	25.162	11.982	27.061	27.649	6.7228	6.7228	6.7228	6.7228	6.7228	23.956	8.1868	8.1868	6.7228	25.162	6.7228	6.7228	25.162
309	0	0	33.326	66.652	0	0	33.326	46.107	0	0	0	0	0	66.652	0	66.652	63.911	0	0	0	0	0	33.326	66.652	0	0	33.326	46.107	0	0	0	0	0	66.652	0	66.652	63.911	0	0	0	0	0	33.326	66.652	0	0	99.978	112.76	66.652	66.652	66.652	66.652	66.652	133.3	66.652
310	160.51	117.1	117.1	96.87	112.66	117.1	96.87	135.67	137	137	96.87	96.87	125.55	96.87	124.51	96.87	96.87	139.96	88.08	139.96	160.51	117.1	117.1	96.87	112.66	117.1	125.55	164.35	165.68	165.68	125.55	125.55	154.23	125.55	153.19	125.55	125.55	168.64	116.76	168.64	189.19	145.78	145.78	125.55	141.34	145.78	96.87	135.67	137	137	96.87	96.87	125.55	96.87	124.51
311	0.4262	0.5033	0.4855	0.4855	0.4121	0.4257	0.6801	0.6468	0.5895	0.5647	0.5436	0.717	0.5665	0.5895	0.5647	0.5895	0.5895	0.5895	0.65	0.5647	0.5212	0.6468	0.6167	0.6167	0.5023	0.5226	0.5436	0.5226	0.4855	0.469	0.4531	0.5665	0.4683	0.4855	0.469	0.4855	0.4855	0.4855	0.5215	0.469	0.4395	0.5226	0.5033	0.5033	0.4243	0.4389	0.5647	0.542	0.5021	0.4845	0.469	0.5895	0.4855	0.5021	0.4845
312	5.5236	5.1699	5.2479	5.2479	5.8074	5.6439	4.1699	4.3219	4.585	4.7004	5	4	4.9069	4.585	4.7004	4.585	4.585	4.585	4.585	4.7004	4.9069	4.3219	4.4594	4.4594	5.3219	5.0875	5	5.0875	5.2479	5.3219	5.5236	4.9069	5.4594	5.2479	5.3219	5.2479	5.2479	5.2479	5.2479	5.3219	5.4594	5.0875	5.1699	5.1699	5.7549	5.585	4.7004	4.8074	5	5.0875	5.3219	4.585	5.2479	5	5.0875
313	3.5795	3.1732	3.193	3.193	3.5591	3.5312	2.4482	2.4862	2.6548	2.8344	2.9362	2.4644	2.8286	2.5951	2.9319	2.6548	2.765	2.6548	2.3993	2.8344	3.1608	2.4862	2.4992	2.4992	3.0753	3.0596	3.0419	3.0726	3.193	3.3015	3.3838	3.0295	3.3116	3.1586	3.3529	3.193	3.2514	3.193	3.0333	3.3015	3.5134	3.0726	3.0888	3.0888	3.4917	3.464	2.8263	2.8628	2.997	3.1249	3.2118	2.8274	3.1329	2.9534	3.1855
314	8.7342	8.0284	8.1916	8.1916	9.1022	8.8657	6.3065	6.6038	7.1042	7.3111	7.7043	5.9663	7.5174	7.1131	7.2998	7.1042	7.0921	7.1042	6.8787	7.3111	7.6775	6.6038	6.8737	6.8737	8.2072	7.8725	7.6793	7.8642	8.1955	8.3415	8.6198	7.4799	8.4851	8.1988	8.3364	8.1955	8.1909	8.1955	8.0406	8.3415	8.6061	7.8642	8.0374	8.0374	8.9913	8.7444	7.306	7.5168	7.8881	8.0483	8.3555	7.0749	8.2079	7.8932	8.0411
315	349.34	262.52	279.75	288.29	345.14	333.01	164.76	186.56	192.29	209.52	235.25	147.52	218.1	217.8	233.59	217.8	222.93	200.85	180.64	218.08	261.61	174.79	192.02	200.56	257.41	245.28	235.33	257.14	262.86	280.09	305.83	218.1	288.67	288.37	304.17	288.37	293.5	271.42	251.21	288.65	332.19	245.36	262.59	271.14	327.98	315.86	235.03	256.84	262.56	279.79	305.53	217.8	288.37	288.07	303.87
316	432.74	325.3	349.73	365.63	477.1	440.27	177.37	198.69	207.11	231.54	292.35	152.94	256.6	250.66	269.22	250.66	248.61	217.65	213.95	242.08	293.34	185.89	210.32	226.23	337.69	300.87	281.03	302.35	310.77	335.2	396	256.6	360.26	354.32	372.87	354.32	352.27	321.31	317.61	345.74	397	289.55	313.98	329.89	441.35	404.53	275.09	296.41	304.83	329.26	390.06	250.66	354.32	348.38	366.93
317	40.701	40.701	40.701	40.701	40.701	40.701	40.701	40.701	67.835	67.835	40.701	40.701	40.701	40.701	40.701	40.701	40.701	54.268	40.701	54.268	40.701	40.701	40.701	40.701	40.701	40.701	40.701	40.701	67.835	67.835	40.701	40.701	40.701	40.701	40.701	40.701	40.701	54.268	40.701	54.268	40.701	40.701	40.701	40.701	40.701	40.701	40.701	40.701	67.835	67.835	40.701	40.701	40.701	40.701	40.701
318	27.134	27.134	27.134	27.134	27.134	27.134	27.134	27.134	54.268	54.268	27.134	27.134	27.134	27.134	27.134	27.134	27.134	27.134	27.134	27.134	27.134	27.134	27.134	27.134	27.134	27.134	27.134	27.134	54.268	54.268	27.134	27.134	27.134	27.134	27.134	27.134	27.134	27.134	27.134	27.134	27.134	27.134	27.134	27.134	27.134	27.134	27.134	27.134	54.268	54.268	27.134	27.134	27.134	27.134	27.134
319	58.91	17.742	17.742	17.742	17.742	17.742	17.742	17.742	17.742	17.742	17.742	17.742	17.742	17.742	35.485	17.742	17.742	17.742	17.742	17.742	58.91	17.742	17.742	17.742	17.742	17.742	17.742	17.742	17.742	17.742	17.742	17.742	17.742	17.742	35.485	17.742	17.742	17.742	17.742	17.742	58.91	17.742	17.742	17.742	17.742	17.742	17.742	17.742	17.742	17.742	17.742	17.742	17.742	17.742	35.485
320	64.593	23.425	23.425	23.425	29.108	23.425	23.425	23.425	23.425	23.425	23.425	23.425	23.425	23.425	41.168	23.425	23.425	41.168	17.742	41.168	64.593	23.425	23.425	23.425	29.108	23.425	23.425	23.425	23.425	23.425	23.425	23.425	23.425	23.425	41.168	23.425	23.425	41.168	17.742	41.168	64.593	23.425	23.425	23.425	29.108	23.425	23.425	23.425	23.425	23.425	23.425	23.425	23.425	23.425	41.168
321	162.38	132.52	148.33	171.48	220.92	200.17	56.408	44.784	42.808	58.616	124.06	40.599	69.807	103.83	94.724	103.83	111.74	42.808	78.916	58.616	78.916	49.058	64.866	88.024	137.46	116.71	85.615	73.991	72.015	87.824	153.27	69.807	99.014	133.04	123.93	133.04	140.94	72.015	108.12	87.824	108.12	78.265	94.074	117.23	166.67	145.92	119.64	108.02	106.04	121.85	187.29	103.83	133.04	167.07	157.96
322	43.34	27.658	27.658	27.658	27.658	27.658	27.658	59.043	42.367	42.367	27.658	27.658	52.449	27.658	27.658	27.658	27.658	40.608	27.658	40.608	43.34	27.658	27.658	27.658	27.658	27.658	52.449	83.833	67.157	67.157	52.449	52.449	77.239	52.449	52.449	52.449	52.449	65.398	52.449	65.398	68.13	52.449	52.449	52.449	52.449	52.449	27.658	59.043	42.367	42.367	27.658	27.658	52.449	27.658	27.658
323	105.29	77.693	77.693	64.126	69.808	77.693	64.126	64.126	91.26	91.26	64.126	64.126	75.491	64.126	81.868	64.126	64.126	95.435	58.443	95.435	105.29	77.693	77.693	64.126	69.808	77.693	75.491	75.491	102.62	102.62	75.491	75.491	86.856	75.491	93.233	75.491	75.491	106.8	69.808	106.8	116.66	89.058	89.058	75.491	81.174	89.058	64.126	64.126	91.26	91.26	64.126	64.126	75.491	64.126	81.868
324	322.39	252.27	266.3	264.32	351.41	328.37	146.15	178.21	189.15	203.17	222.24	132.12	212.21	188.23	204.25	188.23	206.27	189.17	172.18	203.2	232.26	162.15	176.17	174.2	261.28	238.24	226.24	258.3	269.24	283.26	302.33	212.21	292.3	268.32	284.34	268.32	286.36	269.26	252.27	283.29	312.35	242.24	256.26	254.29	341.37	318.33	202.25	234.32	245.25	259.28	278.35	188.23	268.32	244.33	260.36
325	1416	676	772	772	1813	1503	131	168	266	338	470	104	391	258	349	266	276	266	192	338	525	168	207	207	723	562	489	573	777	914	1164	421	1020	763	931	777	793	777	626	914	1249	573	659	659	1612	1327	329	394	556	668	872	278	753	544	683
326	28	23	25	25	37	32	10	12	14	15	19	7	16	16	15	14	14	14	15	15	17	12	14	14	26	21	18	20	22	23	27	15	24	24	23	22	22	22	23	23	25	20	22	22	34	29	18	20	22	23	27	15	24	24	23
327	104	82	88	88	132	116	40	44	54	58	72	34	68	54	56	54	52	54	56	58	66	44	50	50	94	78	74	78	88	92	106	68	102	88	90	88	86	88	90	92	100	78	84	84	128	112	60	64	74	78	92	54	88	74	76
328	1.9556	3.9467	-1.847	3.1366	-4.1424	-2.4454	5.5214	6.1042	-5.0998	-4.25	-1.0282	6.0219	-2.4492	2.7989	2.1915	-2.8096	5.0941	3.5347	2.5656	-2.2796	0.3929	2.7476	6.0475	5.8119	-2.2006	0.5127	5.8445	3.2786	7.1366	5.1598	1.4335	-3.2666	3.7525	3.9051	-0.2576	1.031	-4.0097	2.9466	1.3106	2.6277	-0.4834	3.1627	4.4007	5.9284	4.2987	-2.1701	-3.3437	3.5763	7.8257	4.9756	-2.2249	-5.4516	-2.0525	-5.3895	3.3399
329	2.9782	2.3111	-1.073	-0.7992	-4.3856	-0.2078	1.7635	-0.2627	0.5283	1.8334	-2.7397	-0.6523	-4.3409	-1.5839	-2.9944	-1.5146	-2.6227	-0.4378	-1.5452	2.4121	0.935	-1.0715	0.1732	-0.5465	1.4247	5.5174	1.9694	-2.0945	0.2647	-0.3223	3.0805	0.6729	-4.0291	-1.4347	2.5249	5.0659	-0.6488	-3.8115	0.3463	2.3746	-2.6777	-0.7248	0.7401	1.5872	-4.2266	3.615	-0.6907	0.1648	0.1976	-0.1223	-1.8338	-1.343	-4.6812	-1.7258	-3.2297
330	0.4462	2.2311	-0.5821	-0.6125	-0.9739	0.6947	0.2343	0.5919	0.434	-0.0757	1.5113	0.1163	0.4743	1.0085	1.3103	1.0544	1.0608	0.8368	-1.3874	0.6831	1.8969	1.1727	0.3576	-0.8389	-2.549	0.0135	0.586	1.1147	1.0353	1.0567	0.3179	-0.4862	0.7591	-1.1101	-0.2505	-1.0523	0.208	0.8165	1.731	-1.069	-0.632	0.1441	-1.1739	0.4655	-0.4597	-0.8062	0.8411	1.0718	-0.7956	-1.3505	1.7916	-1.5648	0.7304	1.2331	-1.1166
331	0	0	0	0	9E-14	0	0	0	0	0	0	0	0	0	0	0	0	0	0	0	0	0	0	0	0	0	0	0	0	0	-3E-14	0	0	0	0	0	0	0	0	0	0	0	0	0	0	0	0	0	0	0	3E-14	0	0	0	-9E-14
332	0	0	0	0	9E-14	0	0	0	0	0	0	0	0	0	0	0	0	0	0	0	0	0	0	0	0	0	0	0	0	0	-3E-14	0	0	0	0	0	0	0	0	0	0	0	0	0	0	0	0	0	0	0	3E-14	0	0	0	-9E-14
333	5015.5	1687.7	1870.4	3076.8	3288.5	4799.1	728.74	973.8	664.48	744.3	1254.8	752.54	705.33	889.17	1513.8	975.97	1350.2	945.01	454.89	1203.9	1676.5	517.1	870.27	803.71	2535.4	1512.9	2295.5	2013	2648.7	2449.8	2733.3	912.05	2539.6	2745.2	2401.4	2057.1	2238.7	2398.8	1637.3	2333.6	5063.8	1777.3	2720.9	2683.7	5851.3	2281.6	1067.3	2060.8	2186.4	1687.9	2339	1129.2	1662.3	1701.1	2919.5
334	528.36	493.15	618.19	312.25	1452.6	768.82	136.46	320.7	339.72	463.39	367.62	95.809	600.05	301.44	318.55	263.04	516.12	270.14	279.8	300.88	385.82	270.92	304.09	275.04	449.63	805.91	375.93	330	366.76	568.27	583.66	423.63	922.91	299.2	866.31	699.72	794.42	604.97	430.94	747.26	429.27	400.49	297.14	352.29	613.08	1215	323.68	283.1	358.43	728.81	854.79	389.88	890.05	484.85	607.88
335	262.25	328.54	248.52	180.32	410.91	263.61	48.489	27.284	127.7	138.71	236.08	5.9502	135.13	74.686	84.591	108.07	93.626	58.17	122.14	115.97	108.53	73.033	37.455	103.55	193.4	123.09	45.106	315.04	289.11	285.39	291.25	183.68	137.18	272.42	100.49	135.15	231.14	108.31	240.8	360.96	255.19	193.93	156.46	222.26	319.38	396.19	127.75	110.25	128.13	190.85	199.08	94.375	207.99	306.49	135.8
336	10.607	6.6295	3.4366	5.4488	23.215	2.0604	22.221	24.324	11.17	9.6996	5.7508	24.54	14.805	3.678	11.246	4.8612	23.744	5.4189	7.1152	4.7774	7.0418	5.6192	23.44	22.272	11.508	21.778	23.06	12.784	17.903	15.124	10.495	6.1232	18.288	9.3005	10.955	18.186	8.7621	16.919	1.8964	9.038	14.407	5.5835	9.8319	24.34	18.002	10.16	6.5946	5.9337	18.295	13.887	5.8127	22.599	20.111	21.659	16.464
337	-97799	-79634	-83220	-79423	-102137	-98608	-49612	-54092	-67135	-70728	-68651	-46018	-68642	-60452	-65713	-60447	-61277	-65112	-56208	-68703	-75263	-57069	-60600	-56794	-79579	-75980	-72224	-76716	-89742	-93322	-91205	-68682	-91238	-83050	-88316	-82990	-83929	-87662	-78805	-91310	-97852	-79671	-83264	-79405	-102178	-98639	-64044	-68524	-81500	-85097	-83020	-60394	-82998	-74759	-80090
338	-721149	-484622	-548096	-515253	-794955	-702553	-219090	-250787	-319992	-362139	-400394	-182941	-389000	-333640	-380941	-329333	-319512	-324466	-288915	-362996	-463429	-259171	-298429	-292604	-498008	-437260	-407568	-470799	-523559	-583403	-646812	-384440	-602673	-542264	-615996	-557673	-548079	-543685	-504715	-589285	-668146	-464053	-495507	-496620	-734222	-691779	-371693	-407565	-481667	-532647	-575510	-327183	-558448	-502047	-557102
339	63.535	-153.8	-153.09	-118.64	12.483	-127.08	-69.979	-62.276	-252.53	-258.55	-107.7	-62.792	-37.137	-148.82	0.7135	-144.31	-73.059	-168.47	-122.39	-172.06	14.638	-177.49	-121.45	-78.308	-25.588	-86.376	-31.925	-37.09	-210.62	-203.97	-12.721	-77.772	15.194	-98.726	45.961	-38.206	-69.042	-57.989	-70.241	-130.43	71.77	-130.79	-136.68	-40.446	41.04	-96.427	-154.79	-145.79	-270.4	-280.15	-129.52	-90.941	-43.902	-108.64	-13.463
340	-11.813	-9.7879	-9.6903	-9.6144	-7.4879	-9.1999	-7.7968	-7.8186	-5.0676	-4.9702	-9.6624	-7.6517	-8.0481	-9.9328	-12.463	-9.9381	-7.7231	-10.41	-9.9766	-10.092	-12.228	-10.35	-7.7413	-7.5698	-7.7684	-7.5973	-7.3967	-8.2838	-5.3112	-5.0572	-8.4376	-9.5384	-7.705	-9.4213	-12.091	-7.7194	-8.3699	-8.1939	-8.9749	-9.1397	-11.441	-9.2307	-9.4028	-7.4594	-7.601	-9.0623	-10.105	-9.2337	-5.1292	-5.1124	-9.6419	-7.8308	-7.5897	-7.5011	-12.322
341	11.813	9.7879	9.6903	9.6144	7.4879	9.1999	7.7968	7.8186	5.0676	4.9702	9.6624	7.6517	8.0481	9.9328	12.463	9.9381	7.7231	10.41	9.9766	10.092	12.228	10.35	7.7413	7.5698	7.7684	7.5973	7.3967	8.2838	5.3112	5.0572	8.4376	9.5384	7.705	9.4213	12.091	7.7194	8.3699	8.1939	8.9749	9.1397	11.441	9.2307	9.4028	7.4594	7.601	9.0623	10.105	9.2337	5.1292	5.1124	9.6419	7.8308	7.5897	7.5011	12.322
342	-3.8578	0.0779	0.212	0.1258	-1.0127	0.114	-1.569	-1.7578	4.1651	4.1521	0.2015	-1.6835	-1.3595	0.9083	-3.6855	0.8494	-1.7265	0.5036	0.8413	0.8271	-3.8437	0.8435	-1.6742	-1.5667	-0.8088	-1.5999	-0.6634	-0.3026	2.4883	2.9331	-0.5561	0.3696	-0.313	0.5303	-3.8096	-1.2671	0.4974	-1.3955	0.9533	0.7721	-3.2718	0.7121	0.5127	-0.7426	-0.1077	0.2413	0.924	0.4131	3.9655	4.0781	0.1868	-1.3386	-0.5249	-1.2552	-3.4923
343	574.91	457.2	475.78	499.47	582.33	568.8	322.95	357.72	345.94	365.85	420.73	307.97	378.35	384.69	419.99	389.8	420.38	354.86	341.73	387.47	430.59	326.04	360.67	374.3	478.84	445.36	443.66	446.94	455.02	469.63	503.67	383.06	497.75	485.84	503.53	482.69	492.16	464.46	440.22	500.68	561.76	432.13	456.15	481.27	584.04	532.82	406.13	431.58	440.83	457.91	517.79	396.31	481.95	476.57	521
344	282.52	188.74	193.86	179.77	284.7	273.03	125.44	134.94	115.43	107.77	191.51	133.6	193.77	105.13	188.89	113.06	145.86	168.84	95.348	182.45	224.36	141.55	123.03	110.3	241.41	221.78	199.73	215.42	196.57	194.83	266.71	200.31	289.98	182.78	251.11	179.89	217.59	251.4	184.47	259.43	309.33	228.43	196.66	193.05	325.21	274.18	61.817	96.314	91.745	82.631	168.12	105.99	155.25	77.505	146.83
345	159.58	172.96	169.83	139.48	233.06	219.58	96.482	140.85	149.47	143.8	150.86	97.709	106.98	64.586	68.199	66.304	127.51	107.52	76.976	108.16	64.975	99.285	96.787	80.528	186.57	154.42	114.54	154.06	169.99	160.47	176.2	115.69	133.08	83.922	71.754	103.13	123.92	137.72	96.279	141.71	110.22	121.53	116.46	109.27	190.16	174.92	74.739	114.07	135.53	128.77	137.66	99.334	109.94	74.014	64.293
346	309.32	269.72	286.76	365.39	411.23	395.38	102.42	93.309	89.522	121.15	257.74	83.285	156.77	215.25	163.72	210.72	147.63	83.524	170.27	103.86	141.81	85.202	141.95	183.76	233.64	205.34	221.99	173.67	184.26	209.74	304.65	157.49	245.73	309.15	254.83	277.1	239.88	156.91	246.17	194.65	233.2	170.02	233.1	268.18	333.31	306.66	270.4	233.25	217.93	255.74	390.55	196.62	293.73	325.33	309.88
347	265.58	187.48	189.03	134.09	171.1	173.42	220.52	264.42	256.42	244.71	162.99	224.69	221.57	169.44	256.27	179.08	272.74	271.34	171.47	283.61	288.78	240.84	218.72	190.54	245.2	240.02	221.67	273.27	270.76	259.9	199.02	225.58	252.02	176.7	248.7	205.59	252.29	307.55	194.05	306.03	328.56	262.11	223.06	213.08	250.73	226.16	135.74	198.33	222.89	202.17	127.24	199.69	188.22	151.24	211.12
348	2436.8	1043.2	1071.5	871.37	1731.2	1671.8	495.88	588.76	560.87	523.67	928.23	528.1	933.95	415.59	1096.7	446.93	643.67	898.72	370.24	971.2	1734.5	655.81	569.98	436.02	1252.2	1159.7	962.68	1126.6	1125.6	1115.6	1524	965.47	1649.1	880.98	1675.7	867.05	1148.9	1556.2	876.24	1605.9	2659.6	1256.4	1081.6	930.48	1968.8	1671.4	244.36	420.22	445.79	401.5	814.89	418.96	748.3	306.38	852.48
349	1216.8	955.97	938.64	676.08	1417.5	1344.5	381.39	614.52	875.73	842.5	731.21	386.25	515.62	255.31	327.76	262.1	562.69	572.33	298.9	575.74	437.35	459.99	448.41	318.33	967.95	807.45	552.08	805.73	1143.4	1079.3	1006.8	557.61	756.83	404.5	407.06	497.06	654.32	852.52	457.32	877.2	837.47	668.4	640.52	526.69	1151.4	1066.3	295.44	497.67	794.05	754.47	667.25	392.67	529.93	292.58	308.99
350	122.94	15.776	24.029	40.29	51.641	53.455	28.962	5.9044	34.038	36.022	40.649	35.886	86.79	40.546	120.69	46.758	18.35	61.317	18.372	74.294	159.39	42.268	26.239	29.772	54.835	67.358	85.187	61.358	26.577	34.36	90.51	84.618	156.9	98.855	179.36	76.761	93.669	113.68	88.194	117.72	199.11	106.9	80.203	83.775	135.05	99.264	12.922	17.753	43.78	46.141	30.461	6.6525	45.305	3.4909	82.534
351	1220	87.195	132.81	195.29	313.79	327.31	114.49	25.761	314.86	318.83	197.03	141.86	418.33	160.28	768.95	184.83	80.98	326.39	71.339	395.46	1297.2	195.83	121.56	117.69	284.24	352.22	410.6	320.9	17.811	36.275	517.17	407.86	892.26	476.48	1268.6	369.99	494.57	703.68	418.92	728.66	1822.1	587.95	441.12	403.79	817.42	605.11	51.081	77.455	348.25	352.97	147.64	26.297	218.37	13.8	543.49
352	1.0263	1.0656	1.0531	1.0036	1.02	1.0416	1.0937	1.1812	1.2233	1.1908	1.0558	1.1414	1.1303	1.0223	1.0322	1.0251	1.0921	1.1982	1.1055	1.1336	1.0937	1.1571	1.1302	1.0361	1.0719	1.0774	1.0936	1.1583	1.1802	1.1574	1.0489	1.1549	1.1221	1.039	1.043	1.0305	1.1056	1.1349	1.0951	1.1247	1.0879	1.1494	1.1258	1.053	1.0743	1.1005	0.9976	1.0543	1.0943	1.0736	0.9827	1.0133	1.0365	0.9498	0.9742
353	3.5907	5.0888	2.214	3.2943	6.1108	2.5507	5.8009	6.1385	5.1454	4.6292	3.2935	6.0582	5.0067	3.3704	3.9352	3.3615	5.827	3.6587	3.3007	3.3885	2.151	3.1738	6.0605	5.8975	3.6565	5.5412	6.1952	4.0471	7.2162	5.2767	3.4125	3.3704	5.558	4.3058	2.5504	5.2758	4.0672	4.8864	2.1986	3.6995	2.7935	3.2479	4.6143	6.1548	6.046	4.2928	3.5164	3.7371	7.8685	5.1571	3.3945	5.8286	5.1633	5.7919	4.7783
354	-48.41	47.053	25.533	32.64	61.349	34.724	24.916	24.074	-7.0293	-11.252	21.209	18.151	10.587	11.757	3.5629	9.1831	26.378	-17.456	10.936	-2.1338	-86.507	3.6375	25.115	27.838	40.767	41.506	12.783	15.109	-8.7086	-8.0939	51.665	-10.919	7.9215	9.2229	-16.198	42.384	8.1999	13.439	10.131	-20.771	-89.146	-11.533	-3.8004	17.05	38.175	13.386	13.512	11.105	16.572	-0.5079	29.574	26.984	21.688	38.049	10.19
355	11.651	9.5589	18.584	7.4215	5.7947	15.08	5.632	4.9922	7.0919	9.8602	4.8916	2.8881	4.2104	6.3604	6.056	5.1343	5.0646	4.9483	10.005	5.3925	9.0887	2.6838	6.4275	5.3989	10.268	5.1083	5.0866	9.8878	7.1947	9.7933	12.506	16.181	9.5719	11.262	8.3299	8.2232	9.3363	7.2911	18.568	10.625	11.333	8.055	4.6187	7.1618	6.8376	6.8567	6.1484	7.1429	9.2924	9.9305	7.4616	7.0535	5.9344	8.6707	9.4053
356	-123.63	-5.9276	-60.4	-50.451	5.3251	-63.528	5.7586	1.5079	-64.502	-64.775	-53.136	7.2933	-19.918	-51.936	-56.175	-52.584	2.7806	-92.376	-54.275	-66.836	-180.2	-53.904	-2.8164	7.3962	-0.7949	4.1186	-18.099	-28.719	-84.203	-72.735	3.449	-74.269	-40.302	-67.547	-77.105	14.872	-69.175	-29.066	-57.933	-87.209	-153.13	-74.468	-76.518	-18.555	-12.14	-75.804	-51.466	-57.111	-53.497	-66.157	-52.793	5.5196	-19.971	8.4432	-54.768
357	-69.837	29.291	-5.1925	3.2838	44.965	5.404	15.294	15.13	-27.055	-26.455	-4.7566	15.174	-1.9794	-11.629	-18.551	-13.696	16.502	-45.852	-19.848	-29.531	-120.02	-19.555	14.187	21.068	23.365	29.813	3.4983	-3.5067	-35.784	-27.76	29.399	-34.464	-10.927	-20.451	-31.456	30.61	-25.664	-4.3389	-18.183	-40.281	-105	-28.882	-28.568	6.4869	23.458	-16.831	-9.8243	-12.841	-9.29	-18.509	3.5543	20.132	6.4842	30.22	-10.691
358	0.5912	0.0288	0.1725	1.3298	0.1245	0.1886	0.021	0.0423	0.983	0.3796	2.0317	4E-05	0.9514	1.7576	1.7739	1.7448	0.0305	1.8674	1.7387	1.7376	2.5797	1.7302	0.1114	0.1162	0.1914	0.0915	0.0262	0.0858	2.3104	0.0233	0.3164	0.0669	0.0299	1.7122	0.0822	0.4245	1.7829	0.5714	0.2722	0.0784	0.0686	0.3894	1.9003	0.0628	0.166	1.0742	1.2136	1.5124	1.7584	0.0473	1.4264	0.0174	0.4011	0.1803	1.9252
359	-99.905	-97.311	-62.947	-56.336	-105.39	-56.435	-100.61	-99.855	-148.26	-139.03	-62.578	-102.16	-94.497	-63.248	-110.46	-63.025	-97.302	-61.597	-60.644	-67.715	-79.624	-65.08	-103.05	-101.21	-84.914	-107.74	-90.763	-78.35	-146.82	-145.48	-100.07	-60.49	-105.44	-71.661	-93.586	-120.63	-70.711	-105.05	-63.599	-71.795	-83.447	-65.348	-60.831	-88.811	-110.31	-75.84	-61.975	-58.173	-164.73	-132.8	-56.406	-95.192	-90.229	-95.965	-109.32
360	-0.1836	-0.059	0.075	0.2398	-0.6846	0.4822	-0.2095	-0.1388	0.0534	0.2651	0.0215	-0.1274	0.0787	0.144	0.2045	0.067	-0.1137	0.1157	-0.2601	0.079	-0.1163	0.076	-0.2767	0.0758	-0.9407	-0.0698	-0.0847	0.3396	0.2478	0.2829	0.049	-0.4511	0.2315	0.3137	0.3485	0.5568	0.3118	0.5036	-0.227	0.2605	-0.4523	0.0902	0.2924	-0.0946	-0.41	0.2685	0.2768	0.3742	0.1933	0.5851	0.3718	0.0481	0.3517	0.2897	0.3344
361	7.7404	4.2783	6.4799	7.4278	5.526	8.1242	1.5842	1.9387	4.0065	3.8191	6.166	0.9507	2.3343	6.6951	5.615	5.6577	1.9847	5.475	4.4944	5.0975	7.6573	4.2231	2.5874	2.3637	4.0606	3.1786	2.7044	3.2819	5.74	5.3634	4.5214	3.7481	3.3264	6.6135	5.2076	3.6191	5.7849	3.8126	4.7761	5.0669	6.2414	4.9264	5.6462	3.7535	5.2221	6.8632	6.249	6.3028	6.5659	5.7023	7.4731	2.6261	3.9383	4.4821	6.4392
362	0.0207	0.0115	0.0172	0.0209	0.0061	0.0127	0.0024	0.0082	0.0027	0.0035	0.0059	0.0029	0.0037	0.0034	0.0113	0.0027	0.0126	0.0044	0.0029	0.0057	0.0052	0.0017	0.0026	0.0024	0.0056	0.0094	0.0035	0.004	0.0052	0.0078	0.0274	0.004	0.0135	0.0056	0.0058	0.0091	0.0043	0.0172	0.0052	0.0145	0.0103	0.009	0.0095	0.0055	0.03	0.0057	0.005	0.0056	0.0038	0.0043	0.0089	0.0048	0.011	0.0141	0.0182
363	1.6276	3.9548	5.4137	12.937	5.624	5.4447	2.5952	2.1101	7.8906	0.8785	12.855	-0.7338	4.9913	8.4292	8.4642	10.275	2.9098	15.99	14.806	15.09	14.309	14.479	2.078	-1.1847	3.8231	3.3849	1.5522	5.0208	11.582	4.2029	4.8731	3.9999	5.6891	9.7728	1.2904	-1.0501	16.648	5.5996	4.9243	3.4786	-1.3329	3.8875	12.311	-0.3204	2.901	15.154	9.4488	8.6138	8.0523	1.7363	9.2865	-2.8933	4.5786	-5.7937	2.7772
364	53.793	35.219	55.207	53.735	39.64	68.932	9.535	13.622	37.448	38.32	48.379	7.8802	17.938	40.307	37.624	38.889	13.722	46.524	34.427	37.306	60.18	34.349	17.004	13.672	24.16	25.694	21.597	25.212	48.419	44.975	25.95	39.805	29.375	47.096	45.649	15.738	43.511	24.727	39.75	46.928	48.125	45.586	47.949	25.042	35.598	58.973	41.642	44.27	44.207	47.648	56.347	14.612	26.455	21.777	44.077

Table S12 Amino acid descriptors (2D) used in this study.

	F	F	F	F	F	F	G	G	G	G	G	G	G	G	G	G	G	G	G	G	G	G	G	G	G	G	H	H
	R	S	T	V	W	Y	A	C	D	E	F	G	H	I	K	L	M	N	P	Q	R	S	T	V	W	Y	A	C
No.	FR	FS	FT	FV	FW	FY	GA	GC	GD	GE	GF	GG	GH	GI	GK	GL	GM	GN	GP	GQ	GR	GS	GT	GV	GW	GY	HA	HC
365	0.4914	0.4128	0.4075	0.3599	0.4889	0.48	0.3884	0.3772	0.3337	0.2946	0.4552	0.4338	0.5121	0.2733	0.4498	0.2901	0.347	0.4758	0.279	0.4709	0.5211	0.4342	0.3411	0.2947	0.5042	0.498	0.4502	0.482
366	0.2776	0.3783	0.3569	0.2793	0.4002	0.386	0.2988	0.3937	0.4321	0.393	0.3586	0.3173	0.2827	0.1679	0.1624	0.1701	0.3033	0.303	0.2253	0.2791	0.1509	0.3045	0.2683	0.2151	0.3896	0.3467	0.2582	0.3447
367	0.538	0.5899	0.6027	0.7315	0.7062	0.6951	0.3172	0.2608	0.2588	0.3311	0.6126	0.2704	0.4144	0.5595	0.3898	0.5406	0.3512	0.2354	0.4982	0.268	0.3293	0.2613	0.3936	0.4909	0.4879	0.4611	0.5004	0.3886
368	0.462	0.4101	0.3973	0.2685	0.2938	0.3049	0.6828	0.7392	0.7412	0.6689	0.3874	0.7296	0.5856	0.4405	0.6102	0.4594	0.6488	0.7646	0.5018	0.732	0.6707	0.7387	0.6064	0.5091	0.5121	0.5389	0.4996	0.6114
369	4.2385	2.2816	2.252	1.7446	2.9729	2.9391	1.5355	1.6459	1.6213	1.4314	2.2062	1.7148	2.4685	1.0803	2.6113	1.1466	1.5312	2.5326	1.0834	2.5065	4.0283	2.0115	1.5803	1.1649	2.6151	2.6039	2.1698	2.5208
370	2.1165	2.0909	1.9728	1.3536	2.4341	2.3637	1.181	1.7179	2.5314	2.3028	1.738	1.2542	1.3628	0.6637	0.7804	0.6724	1.3385	1.6128	0.8747	1.4859	1.0157	1.4108	1.2433	0.8505	2.0215	1.813	1.2444	1.8028
371	0.0585	0.2273	0.1059	0.079	0.1739	0.0838	0.124	0.0687	0.2207	0.2227	0.2451	0.0483	0.2137	0.1009	0.0826	0.139	0.0991	0.0971	0.228	0.1062	0.1013	0.1418	0.0713	0.1812	0.0686	0.1498	0.0448	0.1355
372	13.74	16.407	3.4755	5.0239	24.246	1.6683	18.247	23.868	12.378	10.408	6.8224	25.746	15.491	4.4717	15.124	4.9561	24.613	4.4766	6.1856	4.7	5.3877	6.0072	22.763	21.445	14.484	21.926	22.566	15.611
373	-98084	-79821	-83484	-79661	-102340	-98866	-49798	-55063	-67390	-70995	-68852	-46182	-68854	-60695	-65963	-60691	-62280	-65360	-56421	-68964	-75533	-57312	-60828	-57003	-79764	-76206	-72443	-77718
374	-689668	-481525	-535256	-509328	-761648	-684641	-220554	-250950	-317292	-355774	-394743	-182705	-377336	-329040	-372793	-326735	-315306	-322830	-290620	-353923	-454143	-258264	-293643	-291325	-491588	-433838	-407872	-463369
375	92.381	-66.983	-126.1	-94.082	59.666	-110.5	-44.296	-30.272	-234.41	-235.96	-93.655	-31.268	-29.5	-128.19	36.748	-128.01	-42.291	-154.72	-115.54	-157.37	36.111	-168.93	-81.476	-42.006	4.3874	-56.567	-13.778	-11.736
376	-12.01	-8.1799	-9.6133	-9.6892	-7.2294	-9.1807	-7.6984	-7.5134	-4.9401	-4.8438	-9.6671	-7.2045	-7.8335	-10.372	-12.819	-10.275	-7.4342	-10.523	-10.126	-10.459	-12.961	-10.516	-7.3099	-7.4128	-7.8629	-7.3811	-7.204	-7.8253
377	12.01	8.1799	9.6133	9.6892	7.2294	9.1807	7.6984	7.5134	4.9401	4.8438	9.6671	7.2045	7.8335	10.372	12.819	10.275	7.4342	10.523	10.126	10.459	12.961	10.516	7.3099	7.4128	7.8629	7.3811	7.204	7.8253
378	-4.5197	-0.9645	-0.0782	-0.1894	-1.6569	-0.2085	-1.4939	-2.0092	4.0272	3.7998	-0.1492	-2.0631	-1.6488	0.9044	-4.1513	0.8673	-2.1072	0.5544	0.5497	0.8261	-3.9764	0.878	-1.8848	-1.7213	-1.4073	-1.8425	-1.1612	-1.0734
379	0.1425	0.3763	0.348	0.1453	0.3924	0.1853	0.2137	0.2594	0.4652	0.4985	0.3719	0.1199	0.5601	0.3158	0.2198	0.2994	0.3208	0.2701	0.5467	0.2768	0.2396	0.4351	0.2908	0.3509	0.2152	0.4006	0.1576	0.2688
380	0.9521	0.9238	0.8515	0.9611	0.7806	0.9093	0.8983	0.7828	0.7891	0.7311	0.9188	0.8941	0.6468	0.8097	0.8724	0.875	0.7793	0.8256	0.7853	0.8773	0.8655	0.7502	0.7729	0.8409	0.9142	0.7055	0.8762	0.9879
381	13.69	14.246	3.8554	5.6862	21.685	3.7455	21.547	23.969	10.473	9.6958	5.9287	21.638	17.06	6.068	18.018	5.3956	22.319	7.6108	6.7485	4.9298	9.9861	5.1497	21.849	19.877	7.0302	19.432	24.05	15.578
382	-88404	-73014	-76511	-73181	-93593	-90869	-44980	-49268	-61011	-64458	-62839	-41533	-61369	-55375	-59676	-55381	-56165	-58639	-51215	-62088	-67168	-51810	-55202	-51876	-72383	-69560	-64819	-69114
383	-689620	-494951	-536346	-513657	-775991	-684328	-214220	-244484	-314085	-349417	-395406	-178785	-379249	-330304	-367468	-326888	-310780	-320176	-286238	-354770	-441147	-256904	-290702	-287128	-488196	-427723	-396987	-457014
384	55.858	-96.98	-155.68	-112.12	-10.105	-126.82	-73.544	-64.725	-256.35	-261.31	-100.82	-69.545	-51.067	-136.62	17.75	-142.02	-75.474	-160.9	-129.58	-166.53	19.993	-169.22	-116.08	-82.776	-72.825	-91.382	-58.639	-56.704
385	-11.48	-8.6028	-9.821	-9.9737	-7.7961	-9.1599	-8.048	-7.8762	-5.3893	-5.0842	-9.769	-8.1067	-7.9786	-10.057	-12.528	-10.062	-8.0737	-9.3969	-9.7286	-9.7711	-12.77	-10.096	-8.0253	-8.0564	-8.3985	-8.0977	-7.6371	-8.2041
386	11.48	8.6028	9.821	9.9737	7.7961	9.1599	8.048	7.8762	5.3893	5.0842	9.769	8.1067	7.9786	10.057	12.528	10.062	8.0737	9.3969	9.7286	9.7711	12.77	10.096	8.0253	8.0564	8.3985	8.0977	7.6371	8.2041
387	-4.7203	-1.0857	0.0415	-0.2063	-1.2548	-0.0545	-1.9257	-2.1671	3.7845	3.9008	0.0505	-1.6302	-0.8796	0.2223	-4.7081	0.2114	-2.0292	-0.0762	0.2624	0.2242	-4.4237	0.1757	-1.9452	-1.5937	0.0601	-1.6116	-0.9019	-0.6299
388	5806.1	2509.4	2737.1	3569.4	5152	5831.5	913.69	1321.8	1131.9	1346.4	1858.5	854.3	1440.5	1265.3	1916.9	1347.1	1959.9	1273.3	856.84	1620.8	2170.9	861.05	1211.8	1182.3	3178.5	2441.9	2716.5	2658
389	789.85	821.11	866.2	492.56	1860.6	1031.8	184.89	335.74	467.17	602.1	603.47	101.75	731.17	375.97	402.76	370.98	598.85	328.26	401.76	416.6	494.23	342.85	341.55	378.58	642.56	928.89	420.94	633.24
390	5278.5	2015.7	2119.3	3257.1	3701.6	5063.2	777.24	1013.3	792.39	882.94	1490.9	758.49	844.37	964.01	1598.6	1084.2	1454.5	1003.2	577.08	1320.1	1785.1	591.2	907.69	907.2	2729	1636	2340.7	2327.1
391	5543.9	2181.9	2488.7	3389.1	4741.8	5568.1	865.25	1294.5	1004.2	1207.8	1622.6	848.35	1305.5	1190.6	1832.5	1239	1866.5	1215.2	734.84	1504.8	2062.4	788.05	1174.4	1078.8	2985.3	2318.9	2671.4	2355.7
392	4.2438	3.1539	3.206	3.6747	3.829	4.2142	2.5004	2.7234	2.4463	2.5743	2.8918	2.5429	2.6054	2.5927	3.0635	2.6752	3.0825	2.5944	2.2308	2.8242	3.0572	2.3044	2.6227	2.6052	3.4878	3.2015	3.4652	3.2079
393	4.1498	2.6349	2.8626	3.7007	3.1904	3.9828	2.2482	2.5676	2.0036	2.048	2.5591	2.4233	2.0072	2.6668	3.1605	2.7655	2.9553	2.4644	1.986	2.733	2.9121	2.1204	2.6152	2.3755	3.4165	2.6078	3.2111	2.8451
394	1.5819	1.5661	1.8093	1.2066	1.9925	1.6099	1.0815	1.1367	1.2429	1.411	1.4022	0.9341	1.7335	1.2493	1.1701	1.0749	1.336	1.1631	1.2872	1.1814	1.532	1.1708	1.2296	1.3215	1.446	1.9379	1.3957	1.3965
395	1.0038	1.2561	0.9315	1.0403	1.3306	1.1529	0.7917	0.673	0.9413	0.9665	1.267	0.5327	0.9279	0.847	0.9086	1.031	0.9302	0.7679	0.9482	0.8905	0.927	0.7984	0.6984	1.0111	0.8951	1.0092	0.6793	1.0471
396	314.13	236.75	252.88	263.38	344.5	315.25	133.63	150.88	154.63	170.63	210.5	115.75	187.75	184.13	197.88	183.63	188.88	157.88	155.75	179.25	212.38	140.13	155.88	168.13	243.75	221.13	206.88	223
397	363.86	270.87	289.45	303.55	377.8	348.21	170.16	190.18	186.05	205.88	243.24	151.32	221.8	218.73	241.68	220.92	234.13	191.25	183.8	218.47	254.6	172.39	191.23	209.57	281.89	260.38	246.28	257.75
398	3.4246	4.1931	3.7113	3.5385	3.5708	2.7424	2.8795	1.3858	2.1704	1.0542	5.1818	0.8043	1.4557	4.8424	4.4223	4.908	3.1801	2.0493	4.5358	2.116	2.9044	1.6427	0.9287	2.4656	5.2862	2.683	2.0382	1.2483
399	0.0585	0.056	0.0601	0.0858	0.0946	0.0959	0.0454	0.0512	0.0268	0.0289	0.0803	0.0382	0.0521	0.0632	0.054	0.0705	0.0636	0.0349	0.0666	0.0288	0.0471	0.0262	0.032	0.0526	0.0581	0.0614	0.0525	0.0528
400	2.8053	2.9195	2.8655	2.7997	2.7414	2.7855	3.2749	3.1516	3.2341	3.1605	2.9587	3.3663	3.1116	2.9928	3.0867	2.973	2.9708	3.2784	3.1405	3.1664	3.1022	3.3038	3.2096	3.0804	3.0201	3.0067	2.9968	2.9624
401	2.5181	2.5855	2.4483	2.2398	2.4177	2.4672	2.8039	2.877	2.8867	2.8405	2.5444	3.0326	2.7763	2.217	2.7038	2.1452	2.4176	2.9185	2.6018	2.8339	2.7439	2.9305	2.7805	2.4568	2.6976	2.7462	2.5625	2.6252
402	1.5972	1.701	1.6182	1.3118	1.3416	1.4239	2.1341	2.2429	2.4082	2.3219	1.5393	2.3659	2.0346	1.5853	2.0452	1.6057	1.7001	2.4621	1.7254	2.2571	1.9811	2.3997	2.228	1.8309	1.8779	1.9272	1.8366	1.9205
403	0.8724	1.0496	0.983	0.7304	0.7145	0.7243	1.2453	1.1516	1.6534	1.5334	0.9525	1.3689	1.2232	0.9801	1.1056	0.9659	0.9751	1.525	1.0928	1.4983	1.1861	1.6089	1.394	1.084	1.0594	1.0536	1.0884	1.1071
404	0.4894	0.6071	0.5462	0.4018	0.4149	0.3673	0.7042	0.6106	0.9814	0.9055	0.5159	0.7526	0.6738	0.5378	0.5185	0.5092	0.5327	0.84	0.5951	0.7965	0.6755	0.869	0.8075	0.6043	0.5742	0.5806	0.6008	0.5871
405	0.2244	0.3076	0.2733	0.1867	0.2159	0.1736	0.3637	0.2762	0.522	0.4601	0.2202	0.3287	0.3146	0.2423	0.2092	0.2193	0.231	0.3717	0.2527	0.3478	0.339	0.3806	0.3745	0.2672	0.2589	0.2461	0.2541	0.3017
406	0.0955	0.1023	0.1214	0.0618	0.0958	0.0802	0.18	0.1146	0.2025	0.2061	0.0739	0.132	0.1279	0.0834	0.0744	0.0779	0.098	0.1342	0.0879	0.1157	0.1378	0.1449	0.1236	0.1174	0.0983	0.1016	0.1078	0.1136
407	0.0526	0.0309	0.0675	0.0162	0.0463	0.03	0.0895	0.0402	0.0596	0.0906	0.0225	0.0506	0.0413	0.0214	0.0184	0.0198	0.0323	0.0339	0.0255	0.0259	0.0369	0.0488	0.0359	0.0491	0.029	0.0406	0.0459	0.0377
408	757.5	536.13	558.88	688.88	906.13	877.88	292.13	365	251.75	304.5	531.75	256.88	381.25	406.25	404.25	417.88	479.5	305.38	360.25	309.5	446.75	212.63	285.75	356.88	576	513.75	511	496.88
409	426.13	302.75	312.5	378.5	521	504.5	164.63	192.88	133.5	158.63	299.63	140	212.25	216.63	217.25	225.75	263.13	166.63	197.5	163.75	252.75	110.38	156.88	193.38	337.88	295.13	288.38	268.88
410	293.38	208.5	220.88	271.25	363.13	345.75	118	132.75	92.875	108.75	207.25	98.75	145.38	165.63	156.63	175.63	191.75	113.25	139.75	115.75	179.75	77.875	112.25	142.5	236.88	207.75	203.25	186.75
411	238.13	173	185.75	232.38	300.13	282.38	94.625	99.125	70.125	81	174.5	76.375	115.63	140.5	122.88	149.5	160.88	88.875	116.25	89.25	142.63	60	88.125	118.5	194	158.88	166.25	148.63
412	198.5	147.88	158.5	199	261.25	240	76.125	72.625	55.5	64.75	146.88	63	95.375	117.5	96	121.5	134.75	72.125	98.5	71.375	113.38	44.25	68.25	98.5	164.75	125.5	139.38	120.38
413	168.25	124.5	125.38	157.13	224.88	201.88	59.125	54.75	44.75	49.375	123.88	52.5	77.125	75	72.375	71.875	99.5	54.75	81	51.625	89.125	33.25	47.5	69	141.63	99.75	113.38	97.125
414	103.5	73.875	74.5	83	150.88	135.88	35.875	38.25	28.5	32.125	71	35.625	50.75	30.875	33.5	27.75	54.5	34.375	38.375	28.25	53	15	27	31	101.75	71.125	73.375	58.875
415	45.25	31.125	36.5	33.625	70.75	65.25	18	21.375	17	18.25	29.25	23.25	26.875	14.375	14.875	13.875	24.75	21.125	17	15.25	28.5	7	15.125	14	62.625	38.625	44.125	32
416	6.7082	0.5	0.5	5.6125	0.5	1.118	0.5	1.118	6.8739	0.5	0.5	0.5	0.5	1.2247	1.118	0.5	1.8708	3.5	2.9155	0.5	0.5	6.5955	0.7071	0.7071	0.7071	0.5	0.7071	0.5
417	6.8739	0.866	6.442	5.3619	1.118	0.5	1.2247	7.0887	6.6895	0.7071	0.7071	0.7071	8.2614	0.5	0.5	0.866	1.2247	0.7071	3.3912	1.6583	0.7071	6.5	1.2247	0.866	0.7071	0.866	0.866	0.7071
418	0.5	1.2247	6.8739	0.5	0.7071	0.7071	1.118	7.2801	0.7071	0.5	0.5	1.118	8.3367	1.6583	1.2247	1.2247	0.7071	3.0414	0.7071	1.2247	0.5	3.2016	1.5811	0.5	1.4142	0.7071	0.5	0.5
419	0.7071	4.0311	0.5	0.5	7.3993	0.5	0.866	0.7071	0.5	0.5	0.5	0.7071	0.5	0.866	0.7071	0.7071	7.1589	0.5	0.5	0.5	0.5	1.118	0.5	0.866	0.7071	0.5	0.7071	0.5
420	0.5	4.6368	0.7071	0.7071	0.7071	0.5	0.5	7.433	0.5	0.5	0.5	0.5	1.2247	0.5	1.118	0.5	7.3824	1	0.7071	0.5	0.7071	0.5	0.5	7.1764	5.1478	7.1764	0.7071	0.7071
421	0.5	0.866	0.866	0.866	7.2284	1	0.7071	7.6322	1	1	0.7071	1.118	0.866	0.7071	0.5	0.866	0.5	0.5	0.866	1	0.5	0.7071	0.7071	7.1589	5.244	6.9462	1.2247	0.5
422	-2.6893	-2.7138	-2.6262	-2.2186	-2.6699	-3.0256	-2.2765	-2.2235	-2.2742	-2.2373	-2.6286	-2.1019	-2.2099	-2.0743	-2.1558	-2.1759	-2.3539	-2.2499	-2.1837	-2.5617	-2.9077	-2.0549	-2.2268	-2.4171	-2.5904	-2.4001	-2.4271	-3.1806
423	-2.6391	-2.636	-2.5433	-2.1888	-2.6349	-2.9095	-2.2473	-2.1726	-2.2572	-2.2214	-2.4434	-2.0571	-2.1664	-2.0328	-2.1171	-2.1109	-2.2914	-2.2279	-2.1129	-2.4266	-2.8304	-1.9846	-2.2233	-2.3644	-2.5578	-2.3324	-2.3869	-3.1201
424	-2.5705	-2.489	-2.5237	-2.1574	-2.6094	-2.8192	-2.2207	-2.1637	-2.2469	-2.1239	-2.3992	-2.0162	-2.1591	-2.028	-2.0948	-2.085	-2.2909	-2.1923	-2.0779	-2.4178	-2.7575	-1.98	-2.2058	-2.3244	-2.5427	-2.3068	-2.3278	-3.0089
425	-9.4027	-7.9231	-10.56	-7.1036	-9.0782	-8.0645	-10.007	-7.5502	-7.4998	-8.6413	-7.0345	-7.6569	-8.0971	-7.0246	-6.9245	-7.0907	-7.8048	-7.5101	-7.0861	-7.1727	-7.571	-7.639	-7.4086	-8.0007	-7.538	-8.5169	-8.4831	-8.4292
426	-9.2622	-7.7154	-10.096	-6.9545	-8.8748	-7.8661	-9.556	-7.1171	-7.4015	-8.348	-6.7986	-7.149	-7.668	-6.9192	-6.8993	-6.886	-7.4058	-7.4129	-7.0775	-6.9979	-7.4439	-7.3094	-7.257	-7.6092	-7.5273	-8.158	-8.0215	-8.246
427	-8.5805	-7.6652	-9.9764	-6.7409	-8.6883	-7.6114	-9.3493	-7.0961	-7.3386	-7.9155	-6.7506	-7.1219	-7.4992	-6.8074	-6.8292	-6.7641	-7.3997	-7.1394	-6.8521	-6.8102	-7.1774	-7.2911	-7.2152	-7.5483	-7.1857	-7.9465	-7.9891	-8.0603
428	1.3251	1.2427	1.2416	1.2558	1.2893	1.2971	1.185	1.2121	1.1895	1.2022	1.204	1.1958	1.1803	1.1798	1.2219	1.2049	1.2541	1.1764	1.1476	1.2151	1.2026	1.1875	1.2119	1.2035	1.245	1.2546	1.2666	1.2338
429	204.63	347.25	269.75	237.75	296.88	301.13	248.13	213.63	359.63	349.25	267.13	270.63	273.63	209.63	190.5	216.88	221.25	247.63	242.88	242.38	154	298.88	260	241.13	254.75	279.88	264.63	235
430	262	442.25	361.13	307.75	357.13	384.38	314.13	322.88	480.38	491	335.38	323.75	340.13	262.5	246.5	267.5	284	329.75	319.88	354.88	209.13	396.5	375.75	289.25	334	365.38	342.75	340.63
431	210.88	308	293.63	208.38	253.38	267.13	249.5	266.88	387	390.25	241.38	257.75	270.88	205.75	214	216.63	250.13	303.88	239.88	305.38	177.38	313.25	310.38	238.25	279.88	288.25	271	287.38
432	116.25	155.88	157.63	100.5	131.5	123.88	131	124.88	209.5	208	123.38	126.38	141.13	112.5	104.13	108.38	125.75	164.38	120.25	168	110.5	168.38	166.5	126.5	148.88	145.63	154.63	152.75
433	55	75.125	76.625	46.875	68.25	54.75	66.625	57.375	103	104.88	54.625	60	69.75	53.125	43.875	47.875	61.5	77.5	53	75.625	54.375	76.375	81.5	61	69.875	70.125	74.5	72
434	27	33.75	38.375	20.375	34.25	26.375	36.125	26.75	50.125	47.75	22	27.875	32.125	23.25	20.625	21	24.25	32	22.5	33	24.375	31.625	36	28	31.5	31.25	31.25	35.75
435	12.75	11	17.625	7.375	15.25	11.375	16.25	9.875	17.625	20.625	7.625	9.625	12.125	7.625	7.5	7.75	9.875	12	6.75	11.75	9.625	12	11.375	10.75	12	11.875	11.875	12.875
436	7.5	3.375	9.5	2	6.625	4	8.625	3	5	8	2.25	3.625	3.75	1.625	1.875	1.875	2.875	2.75	1.875	2.5	2.5	3.625	3.25	4.25	3.25	4	4.75	4.25
437	0.3055	0.4335	0.4001	0.23	0.2289	0.196	0.6451	0.5047	1.2073	1.0598	0.3172	0.7038	0.5684	0.3925	0.3982	0.3423	0.3742	0.8212	0.4275	0.8002	0.5188	1.1364	0.8051	0.4965	0.3668	0.3935	0.4176	0.4565
438	0.1627	0.2262	0.2174	0.106	0.1241	0.1023	0.391	0.2762	0.7273	0.6127	0.1361	0.3748	0.2919	0.1824	0.1872	0.1513	0.1593	0.3868	0.2022	0.4062	0.2743	0.5625	0.4213	0.2416	0.1746	0.2046	0.1955	0.2557
439	1.4928	2.1541	2.0059	1.5336	1.497	0.9947	1.2657	0.4069	1.0592	0.4237	2.3899	0.7215	0.5933	1.9403	1.2453	1.896	1.2403	0.7828	2.0708	1.0777	1.2918	0.9258	0.7761	1.2809	2.5534	1.1959	1.0214	0.4747
440	1.3544	2.0409	1.9101	1.5628	1.3277	0.8763	1.1091	0.3071	1.0289	0.4662	2.247	0.5552	0.6387	1.9463	1.2845	1.8376	1.1762	0.8756	1.9389	1.2027	1.2662	1.014	0.735	1.0881	2.4496	1.257	1.0581	0.5429
441	1.2715	2.0112	1.7387	1.2515	1.3312	0.8243	1.2326	0.3068	1.2597	0.3798	2.2124	0.5694	0.6818	2.0844	1.3866	2.0596	1.4568	0.9062	1.9889	1.2693	1.2183	1.2047	0.7604	1.3344	2.3758	1.1652	0.9761	0.6092
442	1.627	2.3371	1.9722	1.2975	1.7125	1.0367	1.4926	0.361	1.4972	0.5483	2.4511	0.6182	0.8558	2.4009	1.5186	2.3876	1.8949	1.0656	2.1946	1.3603	1.3802	1.5418	0.928	1.7394	2.7702	1.1996	1.063	0.7265
443	2.121	2.7159	2.3769	1.5499	1.9358	1.2201	1.6529	0.3978	1.6623	0.6817	2.7436	0.624	0.9695	2.6331	1.4342	2.6718	2.2296	1.1383	2.4488	1.4594	1.4419	1.7376	1.0916	2.0585	3.1155	1.4588	1.2251	0.8593
444	2.464	2.9735	3.0506	2.4505	2.0428	1.395	1.5456	0.6335	1.9298	0.7602	2.9861	0.6637	1.0633	2.4185	1.3811	2.1431	1.9229	1.2604	2.6221	1.582	1.6343	1.7155	1.1509	2.0966	3.3036	1.559	1.6099	1.0311
445	1.9643	2.4047	3.0321	2.9042	1.2172	1.1461	0.8516	0.5941	1.8061	1.098	2.7702	0.9085	1.3366	2.5589	1.2176	1.899	1.2463	1.645	2.174	2.2659	2.0997	1.1487	1.131	1.186	2.8245	2.0012	1.5968	1.1032
446	1.5319	1.4861	2.3879	3.0118	0.9922	1.6963	0.6385	1.2662	1.7104	1.3758	2.234	1.2461	0.8287	2.5304	1.9105	1.8472	0.8027	1.9868	1.6704	2.67	2.0681	1.9832	1.3976	0.6346	2.2124	2.0528	1.2377	1.2109
447	0.3599	0.4394	0.278	0.3565	0.2979	0.3417	0.323	0.4564	0.3631	0.269	0.4999	0.1087	0.2192	0.6958	0.0625	0.6754	0.5779	0.2294	0.6587	0.1324	0.5354	0.287	0.3348	0.5484	0.5295	0.3235	0.3042	0.3594
448	1.1503	1.221	0.978	0.8266	1.2787	0.9302	0.6544	0.3877	0.572	0.3932	1.489	0.2026	0.2513	1.7096	0.3173	1.6934	1.2575	0.3827	1.5206	0.2666	0.8319	0.4361	0.4289	1.1475	1.4254	0.6248	0.5553	0.2818
449	1.7641	1.9553	1.6643	1.6734	2.1301	1.4836	0.9391	0.4875	0.7482	0.4623	2.408	0.2545	0.4552	2.1202	0.7892	1.9717	1.5164	0.5607	2.1212	0.4722	1.1484	0.5199	0.3894	1.2444	2.2529	1.1779	0.9402	0.339
450	1.9054	2.1949	1.8755	2.1213	2.3181	1.852	1.3837	0.8526	0.9213	0.55	2.8147	0.3668	0.6866	2.4473	1.7609	2.4332	1.5669	0.8075	2.3814	0.686	1.5964	0.5554	0.3084	1.1872	2.7606	1.4521	1.0706	0.6904
451	2.3519	2.344	1.9803	2.4052	2.3292	1.9927	1.7046	1.1648	1.0581	0.7471	3.0472	0.5964	0.8785	2.7745	3.0451	2.8706	1.7846	1.1894	2.5851	0.8633	1.8651	0.682	0.3036	1.1975	2.9633	1.5585	1.1194	0.8609
452	3.0854	2.5432	2.2312	2.9554	2.4106	2.1128	2.3217	1.285	1.341	1.2574	3.3364	0.9037	0.9556	3.2256	3.8533	3.4001	2.0199	2.0051	3.0347	1.1725	2.2972	0.9228	0.5316	1.1718	3.463	2.0729	1.2425	0.4108
453	3.6697	3.1204	2.6434	3.9451	1.7711	2.123	2.8093	1.5921	1.9664	1.9442	4.2506	1.0171	1.2205	3.9168	4.2181	3.9268	2.4472	3.046	3.7391	1.9903	2.4471	1.531	0.963	1.3568	4.1885	2.2193	1.0328	1.1342
454	3.9669	3.3964	3.0076	5.1653	1.0777	2.1537	3.1817	2.2082	2.5172	2.3155	4.7765	1.0979	2.1914	4.6271	4.9844	4.641	2.7178	4.1074	4.5672	3.5419	3.2899	1.791	1.8711	1.5184	4.4544	2.5703	1.1575	2.6716
455	1.3903	1.3602	1.3878	1.3907	1.4617	1.424	1.2199	1.237	1.2598	1.2753	1.3583	1.1626	1.3299	1.3295	1.3054	1.303	1.2809	1.2764	1.295	1.281	1.3478	1.2095	1.2345	1.2762	1.3723	1.3262	1.2899	1.346
456	508.62	401.51	416.92	433.16	517.82	500.45	280.04	308.2	302.04	319.62	364.15	261.39	328.89	328.31	351.62	335.86	365.97	299.96	286.68	332.94	357.34	277.08	306.79	321.08	410.99	392.85	382.38	384.85
457	707.13	546.13	578.63	602.38	756.88	712.63	341.63	381.25	380.5	407.63	494.63	303.88	437.38	436.5	459	437.63	468.75	382.88	371.25	426.5	481.63	335.13	378.75	409.75	564	521	493.25	518
458	1395.3	1156.9	1148.3	1117.6	1381.6	1356.9	897.63	946.63	1012.9	1044.8	1038.3	878	1022.3	901.88	1067.8	916.5	1026.3	1025.9	860.88	1081.8	1127.8	929.75	975.5	927.63	1273.9	1172.6	1124.9	1131.3
459	902.13	764	747.75	657.88	821.88	820.63	628.88	674.63	762.25	779.88	656	631.38	709.88	560.25	738.38	568.13	651.38	755.13	558.5	804.5	782.38	695.13	707.38	607.88	855.63	803.13	749.13	773.63
460	479.5	425.25	426.75	342.75	411	404.63	373.75	394.25	492	495.75	350.13	377.38	413.63	327.13	427	337.63	379.88	471.75	316.25	494.5	453.75	439	441	364.88	486.75	444.88	430	450.13
461	201.88	194.13	192.5	144.25	177.88	161.13	165.75	160	240.38	239.75	155	160.63	183.63	148.88	166.5	144.13	161.25	210.88	141.88	225	203	206.25	201.5	161.38	204.63	189.25	193.25	195
462	89.625	90.375	88.375	62.375	83.125	67.75	76.125	67	112.13	115.25	65.75	69.5	82.625	65	62.375	60.125	71.75	93	59.75	92.625	93.25	88.5	90.375	70.75	86.875	81.75	84.875	85.25
463	38.75	39.125	40.375	24.625	37.25	28.875	37	27.375	51	49.625	23.75	28.625	33.75	25.625	23	22.625	25.625	34.375	23.5	36.25	39.125	33.75	37.125	28.625	33.875	32.5	32.5	38
464	16.375	12.25	17.625	7.625	15.25	11.75	16.25	9.875	17.625	20.625	7.625	9.625	12.125	7.625	7.5	7.75	9.875	12	6.75	11.75	14.125	12	11.375	10.75	12	11.875	11.875	12.875
465	8	3.5	9.5	2	6.625	4	8.625	3	5	8	2.25	3.625	3.75	1.625	1.875	1.875	2.875	2.75	1.875	2.5	3.625	3.625	3.25	4.25	3.25	4	4.75	4.25
466	1190.6	809.63	878.5	879.88	1084.8	1055.8	649.5	733	653.25	695.5	771.13	607.38	748.63	692.25	877.25	699.63	805	778.25	618	839.38	973.75	630.88	715.5	686.5	1019.1	892.75	860.25	896.25
467	640.13	321.75	386.63	350.13	464.75	436.25	314.75	351.75	281.88	288.88	320.63	307.63	369.75	297.75	491.88	300.63	367.38	425.38	238.63	449.63	573.25	298.63	331.63	318.63	521.63	437.75	406.38	433
468	268.63	117.25	133.13	134.38	157.63	137.5	124.25	127.38	105	105.5	108.75	119.63	142.75	121.38	213	121	129.75	167.88	76.375	189.13	276.38	125.75	130.63	126.63	206.88	156.63	159	162.75
469	85.625	38.25	34.875	43.75	46.375	37.25	34.75	35.125	30.875	31.75	31.625	34.25	42.5	36.375	62.375	35.75	35.5	46.5	21.625	57	92.5	37.875	35	34.875	55.75	43.625	38.625	42.25
470	34.625	15.25	11.75	15.5	14.875	13	9.5	9.625	9.125	10.375	11.125	9.5	12.875	11.875	18.5	12.25	10.25	15.5	6.75	17	38.875	12.125	8.875	9.75	17	11.625	10.375	13.25
471	11.75	5.375	2	4.25	3	2.5	0.875	0.625	0.875	1.875	1.75	0.75	1.625	2.375	2.375	1.625	1.375	2.375	1	3.25	14.75	2.125	1.125	0.625	2.375	1.25	1.25	2.25
472	3.625	1.25	0	0.25	0	0.375	0	0	0	0	0	0	0	0	0	0	0	0	0	0	4.5	0	0	0	0	0	0	0
473	0.5	0.125	0	0	0	0	0	0	0	0	0	0	0	0	0	0	0	0	0	0	1.125	0	0	0	0	0	0	0

	H	H	H	H	H	H	H	H	H	H	H	H	H	H	H	H	H	H	I	I	I	I	I	I	I	I	I
	D	E	F	G	H	I	K	L	M	N	P	Q	R	S	T	V	W	Y	A	C	D	E	F	G	H	I	K
No.	HD	HE	HF	HG	HH	HI	HK	HL	HM	HN	HP	HQ	HR	HS	HT	HV	HW	HY	IA	IC	ID	IE	IF	IG	IH	II	IK
365	0.432	0.4149	0.5295	0.5229	0.5826	0.3762	0.4987	0.3727	0.4421	0.5413	0.419	0.5182	0.5506	0.5286	0.4311	0.4011	0.5568	0.5146	0.1522	0.2232	0.2081	0.1805	0.3247	0.2674	0.3221	0.1626	0.2818
366	0.3736	0.3417	0.3498	0.302	0.2674	0.1727	0.1425	0.2136	0.2518	0.2965	0.2187	0.283	0.1962	0.2812	0.2553	0.227	0.3256	0.3283	0.184	0.2643	0.3074	0.2812	0.2659	0.2506	0.2281	0.1553	0.1234
367	0.405	0.4466	0.6049	0.4111	0.4937	0.6363	0.5061	0.5741	0.4874	0.3378	0.5592	0.3888	0.4151	0.3934	0.511	0.5572	0.5707	0.5755	0.6658	0.5405	0.4944	0.5585	0.7543	0.4961	0.6095	0.6827	0.5948
368	0.595	0.5534	0.3951	0.5889	0.5063	0.3637	0.4939	0.4259	0.5126	0.6622	0.4408	0.6112	0.5849	0.6066	0.489	0.4428	0.4293	0.4245	0.3342	0.4595	0.5056	0.4415	0.2457	0.5039	0.3905	0.3173	0.4052
369	2.4736	2.3755	3.0258	2.5204	3.3131	1.8133	3.3278	1.7963	2.3344	3.3505	1.9905	3.2074	4.7344	2.9074	2.3712	1.9334	3.3711	3.1369	0.6017	0.9737	1.0113	0.8768	1.5738	1.0572	1.5527	0.6429	1.6362
370	2.5128	2.2982	1.999	1.4557	1.5205	0.8326	0.8084	1.0298	1.3295	1.8355	1.0389	1.752	1.4908	1.5468	1.4042	1.0944	1.9715	2.0013	0.7275	1.1531	1.8013	1.6476	1.2887	0.9908	1.0996	0.6139	0.5931
371	0.1067	0.1662	0.1255	0.3468	0.102	0.1034	0.0751	0.0845	0.1274	0.0753	0.138	0.1288	0.0762	0.1341	0.0927	0.0927	0.0706	0.2344	0.1477	0.1164	0.095	0.1524	0.1203	0.1969	0.1336	0.2086	0.0581
372	18.32	15.572	12.306	6.4187	19.315	10.002	12.263	19.158	5.9859	18.137	1.2561	8.5929	15.749	5.6731	10.015	25.068	21.645	5.333	7.793	6.3578	19.51	15.633	5.4465	24.359	21.611	23.591	24.239
373	-90030	-93634	-91413	-68908	-91478	-83315	-88610	-83250	-85006	-87919	-79069	-91606	-98160	-79949	-83552	-79647	-102404	-98931	-64297	-69565	-81802	-85415	-83268	-60604	-83249	-75025	-80382
374	-518553	-571222	-633828	-374684	-590023	-536963	-599268	-541674	-536470	-535350	-497570	-576003	-655581	-458141	-490699	-490071	-720541	-684338	-365639	-401745	-473981	-516657	-563274	-322027	-546021	-493261	-528793
375	-200.19	-201.26	19.735	-84.146	46.936	-77.536	64.486	-11.943	-93.582	-27.268	-89.061	-124.97	105.64	-132.21	-111.84	-13.959	41.41	-109.24	-131.52	-121.15	-234.27	-243.93	-96.024	-41.719	-12.411	-50.177	29.673
376	-4.911	-4.6912	-7.8535	-9.5644	-7.355	-9.5111	-12.026	-7.3666	-9.3082	-7.6393	-9.0505	-9.3906	-11.951	-9.2303	-9.493	-7.2144	-7.3049	-9.0389	-10.381	-9.8649	-4.7493	-4.8502	-9.4408	-7.3419	-7.2941	-7.2633	-12.644
377	4.911	4.6912	7.8535	9.5644	7.355	9.5111	12.026	7.3666	9.3082	7.6393	9.0505	9.3906	11.951	9.2303	9.493	7.2144	7.3049	9.0389	10.381	9.8649	4.7493	4.8502	9.4408	7.3419	7.2941	7.2633	12.644
378	2.2758	2.7615	-1.2672	0.3174	-0.8458	0.3638	-4.1357	-1.574	0.5877	-1.6609	0.8424	0.4766	-3.7801	0.6547	0.385	-1.2813	-0.7881	-0.0477	1.0397	0.7474	3.938	3.7186	0.0682	-1.8727	-1.435	-1.7326	-4.3361
379	0.2175	0.2828	0.2637	0.4546	0.3061	0.1877	0.2953	0.3028	0.3316	0.2374	0.3246	0.3596	0.1246	0.2729	0.1503	0.1892	0.1442	0.46	0.3245	0.1655	0.1912	0.3805	0.3298	0.3187	0.4301	0.3619	0.2107
380	0.9742	0.9063	0.9119	0.8204	0.7731	0.9911	0.7662	0.7952	0.8205	0.8347	0.9079	0.8747	0.9683	0.9051	0.9534	0.9572	0.9546	0.7666	0.8591	0.9285	0.9095	0.7774	0.7948	0.8055	0.7328	0.9182	0.8663
381	20.504	14.78	8.8918	8.9924	21.841	10.661	11.238	17.35	9.6103	17.288	4.1638	8.5478	13.659	7.4698	11.099	24.657	16.143	10.671	6.7769	7.1088	16.243	15.648	6.1415	22.388	18.977	21.246	24.412
382	-80829	-84279	-82631	-61415	-81201	-75199	-79514	-75152	-76037	-78420	-71040	-81922	-87001	-71641	-75086	-71712	-92183	-89437	-58830	-63116	-74794	-78244	-76629	-55327	-75159	-69116	-73468
383	-518435	-571794	-626281	-372588	-588426	-534960	-599755	-545879	-538213	-532740	-490217	-574351	-648545	-455295	-490049	-484613	-728529	-675744	-367937	-401278	-474616	-526308	-562000	-321586	-546968	-494092	-529331
384	-221.15	-229.28	-40.834	-97.431	-23.188	-106.9	30.363	-60.876	-91.374	-89.05	-101.3	-149.17	37.846	-149.26	-147.53	-66.133	-15.165	-112.29	-148.59	-137.3	-268.17	-274.69	-117.72	-91.351	-68.609	-107.73	-1.9331
385	-5.1124	-5.3109	-8.7153	-9.695	-7.351	-9.773	-12.344	-7.961	-8.703	-8.4789	-9.5314	-9.5412	-11.252	-9.7118	-9.8695	-7.6471	-7.8912	-9.0242	-10.015	-9.2676	-5.0582	-4.9792	-9.5268	-8.0577	-7.8719	-7.8487	-12.338
386	5.1124	5.3109	8.7153	9.695	7.351	9.773	12.344	7.961	8.703	8.4789	9.5314	9.5412	11.252	9.7118	9.8695	7.6471	7.8912	9.0242	10.015	9.2676	5.0582	4.9792	9.5268	8.0577	7.8719	7.8487	12.338
387	2.1152	2.5522	-0.6255	0.0219	-0.304	0.0824	-4.2142	-1.8757	0.1469	-2.0763	0.318	0.4432	-3.3782	0.3032	0.1101	-0.9695	-0.36	-0.0432	0.3463	0.0671	3.6189	3.6943	0.2665	-1.694	-0.7113	-1.6102	-4.7398
388	3304.6	3303.4	3608.2	1519.4	3599.7	3316.8	3368.2	2892	3264.3	3112.1	2309.1	3441.8	5748.3	2371.7	3174.5	3258.2	6783.8	3892.8	1518.7	2454.1	2672.9	2607.6	3392.9	1613.4	2760.3	2492.5	3663.2
389	655.83	853.6	874.57	607.2	1059.8	571.59	965.04	834.76	1006	713.14	671.53	1108	684.43	594.37	453.58	574.4	932.37	1608.3	451.44	388.01	486.5	919.58	1053.4	484.15	1097.7	791.28	743.19
390	2937.8	2735.1	3024.7	1095.8	2676.9	3017.4	2503.6	2192.4	2489.1	2507.2	1878	2694.7	5319	1971.2	2877.4	2906	6170.8	2680.6	1195	2176.4	2314.5	1878.8	2538.5	1223.6	1870.5	2007.4	3055.7
391	3015.5	3018.1	3317.1	1335.7	3462.7	3044.6	3267.7	2756.8	3033.5	3003.8	2068.6	3080.8	5493.1	2178	3018	3036	6464.4	3496.6	1391	2343.9	2544.8	2416.8	3193.8	1519.1	2552.4	2186.2	3527.4
392	3.5034	3.415	3.4546	2.6758	3.5093	3.5159	3.4417	3.283	3.3763	3.3997	3.0254	3.4856	4.2899	3.1291	3.5196	3.5795	4.4578	3.4969	2.7403	3.2363	3.3013	3.1713	3.4913	2.9277	3.2074	3.1939	3.751
393	3.2438	3.0031	3.1574	2.0098	3.0415	3.5008	3.2681	3.0725	3.005	2.9961	2.7335	3.1086	4.1906	2.6877	3.3948	3.3617	4.3727	2.8126	2.4241	3.0276	3.0108	2.8268	3.2933	2.2973	2.902	2.9417	3.8873
394	1.1691	1.3574	1.5617	1.5078	1.7572	1.2685	1.8791	1.6831	1.7581	1.7586	1.5815	1.6847	1.2963	1.6159	1.1595	1.3736	1.4141	1.9494	1.5745	1.1257	1.3205	1.4803	1.7507	1.5399	1.6754	1.4385	1.5627
395	1.0597	1.2244	1.1187	1.1836	0.9713	1.1258	0.8957	0.8932	1.0724	0.8221	1.0156	1.1158	1.1564	0.9844	1.0339	1.0235	1.1615	1.3616	0.9316	1.0329	0.9279	1.1036	1.1425	1.0193	1.0607	1.3435	0.9368
396	228.13	244.75	288.25	183.75	260.5	258.25	272.63	260.38	259	237.25	230.38	251.88	287.13	210.75	227.63	241.5	317.75	289.25	202.75	222.25	224.13	241.5	283.25	185.75	258.88	257.25	267.25
397	261.97	283.49	326.35	217.87	298.48	293.65	313.75	312.1	298.24	280.3	262.34	292.63	334.39	245.4	263.2	284.56	362.1	322.99	238.52	257.62	266.81	281.14	322	226.26	296.67	304.41	316.49
398	3.556	2.9658	3.2186	3.6909	2.572	3.8636	0.8023	1.9866	3.8963	2.0172	3.0687	2.8629	2.0194	4.2227	4.164	2.3221	3.5886	4.029	4.2623	4.1328	4.9251	3.8418	4.9116	4.4401	2.3911	3.3462	4.1558
399	0.0372	0.0363	0.0546	0.0521	0.0701	0.08	0.0704	0.0464	0.0892	0.028	0.0581	0.0434	0.053	0.0374	0.0425	0.0614	0.0694	0.0923	0.0642	0.0789	0.0343	0.0494	0.0979	0.0663	0.0607	0.0739	0.0702
400	3.0136	2.9996	2.8548	3.1454	2.9043	2.8413	2.9411	2.866	2.8493	3.0536	2.9539	2.9479	2.9031	3.0677	2.984	2.8861	2.8559	2.8526	2.8986	2.8882	2.9335	2.9091	2.752	2.9978	2.8292	2.7725	2.8832
401	2.6894	2.687	2.475	2.8113	2.5924	2.234	2.6197	2.291	2.375	2.7323	2.5277	2.6497	2.5785	2.7181	2.5569	2.3501	2.5378	2.5798	2.0776	2.2843	2.2831	2.3299	2.0866	2.3038	2.1991	1.9725	2.3353
402	2.0634	2.1567	1.6227	2.0591	1.7431	1.4641	1.7159	1.6609	1.4679	2.2403	1.8084	1.9736	1.7568	2.0325	1.8453	1.6261	1.6394	1.7364	1.5009	1.7187	1.8358	1.8301	1.2521	1.7438	1.5336	1.3132	1.6192
403	1.3329	1.3104	0.9601	1.1845	0.9733	0.7947	0.9854	1.0542	0.8214	1.4305	1.0388	1.2456	1	1.3043	1.181	0.9617	0.8984	0.9115	0.9657	0.8244	1.2808	1.1001	0.7232	1.0043	0.9297	0.7987	0.8986
404	0.7908	0.7524	0.5758	0.6877	0.5255	0.4423	0.504	0.6053	0.4287	0.8348	0.5548	0.703	0.5799	0.7931	0.7029	0.5334	0.4555	0.4444	0.5231	0.404	0.7663	0.6445	0.3837	0.5539	0.5299	0.4555	0.3989
405	0.4149	0.3715	0.3179	0.3171	0.2857	0.2371	0.2192	0.3027	0.1982	0.391	0.2544	0.3287	0.2936	0.4177	0.3399	0.2345	0.1777	0.1622	0.232	0.1672	0.3771	0.3553	0.1605	0.2823	0.2608	0.2345	0.1526
406	0.1336	0.1678	0.1368	0.115	0.1445	0.1088	0.0956	0.094	0.0913	0.1188	0.0998	0.126	0.1393	0.1631	0.1091	0.0988	0.0642	0.0489	0.0795	0.0519	0.1182	0.1617	0.0438	0.1466	0.1001	0.0969	0.0471
407	0.0372	0.0768	0.0485	0.0401	0.0661	0.0223	0.0339	0.0242	0.0246	0.0286	0.0216	0.0371	0.078	0.0772	0.0241	0.0431	0.0231	0.0159	0.0202	0.0176	0.0255	0.0753	0.014	0.0725	0.0487	0.042	0.0151
408	502.38	490.63	626.38	410.88	664.63	654.13	594.75	501.25	677.25	407.63	495.88	510.5	706.25	403	493	587.13	843.25	695.13	409.25	523.63	433.38	507.88	720.63	422.5	562.63	553.13	576
409	278.75	266.88	355.25	236.63	371.5	362.63	335.75	273.5	379.13	220.25	273.25	291.13	412.88	228.75	269.25	330.75	482.63	411.88	219.75	287.13	228.75	283.13	400.88	238.75	310.25	303.63	321.38
410	191.25	186.25	247.5	163.25	256.25	260.38	238.5	204.63	267.5	155.5	192.13	209.88	290	160.88	188.63	237	338.5	294.63	171.75	212.88	169.38	210.75	292.88	180	229.88	233.13	238.63
411	153.88	140	203.75	127.88	206.5	218.75	199.5	171.75	222.63	117.25	158	167.75	232	130.88	154.25	196	277.38	233.25	150.63	166.25	143	170.75	253.5	152.13	195.13	205.75	196.75
412	126.5	111.38	173.75	104	169.75	184.25	169.5	140	191.63	93.375	131.88	136.25	191.88	106.5	127	166.63	233.38	194.88	126.88	129.5	118.25	139	216.63	127.13	166	174.63	164.5
413	105.25	85.375	146	82	138.25	139.88	137.75	99	151.75	71.5	104.75	108.25	160.13	86.25	101	131.25	195.75	158.38	76.125	81	77.625	89.125	159.5	85.125	116.88	120.13	112
414	73.25	56.5	84.875	56	88.375	77.875	89.875	50.5	92.25	45.875	56.375	66.375	107.25	56.875	65	78.875	129.38	111.63	28.5	40.125	33.125	50.875	86.125	43.25	57.125	54.25	61.625
415	43	31.875	38.25	30.875	48.625	42.375	42.5	24.25	42.75	28.875	27.875	39.5	61.125	29.125	34.75	44.75	69.125	64	8.75	18.625	15.5	29.625	35.125	25.75	27	21.625	25
416	6.2849	0.7071	0.7071	0.5	0.5	0.5	1.118	0.5	0.5	0.7071	0.5	1.118	0.5	0.5	0.5	0.5	0.866	0.5	0.5	7.3824	0.5	1.2247	0.5	1.2247	0.7071	0.866	0.5
417	6.7268	0.5	0.7071	1.2247	1.118	0.7071	0.5	0.5	0.7071	0.5	0.7071	0.7071	0.7071	0.5	0.5	0.5	0.5	2.0616	6.442	7.433	0.5	0.866	1.5	1.8708	0.866	1.5	0.5
418	0.5	0.866	1.2247	1.118	1.2247	0.5	0.7071	0.7071	1.118	0.866	0.5	1.8028	0.5	0.7071	1	0.7071	1.2247	1.5811	6.8739	0.866	0.7071	0.5	1.2247	0.7071	0.5	2.3452	0.7071
419	7.5	0.7071	5.6789	0.5	0.7071	0.5	0.5	8.2158	0.7071	0.5	0.7071	0.5	7.1063	1.118	0.7071	0.5	0.5	0.5	0.5	0.5	0.5	0.7071	0.7071	0.5	0.5	0.5	0.5
420	0.5	0.5	0.7071	7.6974	0.866	0.7071	0.866	0.5	0.5	8.2158	0.866	7.4833	7.433	0.866	0.5	7.0887	0.7071	0.5	0.7071	0.7071	0.7071	0.5	0.5	0.7071	0.7071	0.866	0.7071
421	7.8422	0.5	5.3151	7.8422	1.5	0.866	0.7071	7.7621	0.5	8.3217	1.118	7.7621	0.5	0.7071	0.5	7.1414	0.5	1	0.5	0.5	0.5	0.5	0.5	0.5	0.5	1.2247	0.866
422	-2.5433	-2.7687	-2.7282	-2.3861	-2.5444	-2.4345	-3.0423	-3.1115	-2.6767	-2.8551	-3.2356	-3.0648	-2.863	-2.9148	-2.4277	-2.4166	-2.9375	-3.267	-2.1469	-2.1473	-2.2899	-2.3796	-2.6875	-2.473	-2.5785	-2.4838	-2.2555
423	-2.537	-2.7029	-2.4255	-2.3631	-2.5185	-2.4307	-3.0244	-2.7588	-2.6342	-2.6744	-3.1298	-2.8993	-2.731	-2.8571	-2.397	-2.4049	-2.9101	-3.2206	-2.0905	-2.1057	-2.2368	-2.3684	-2.6871	-2.4412	-2.552	-2.4503	-2.252
424	-2.5214	-2.6569	-2.3953	-2.3585	-2.4873	-2.4018	-2.9718	-2.7396	-2.6196	-2.5607	-3.0027	-2.8742	-2.6285	-2.8535	-2.3674	-2.3784	-2.8872	-3.1769	-2.0777	-2.0995	-2.2037	-2.3452	-2.6672	-2.4028	-2.5069	-2.4502	-2.2329
425	-7.006	-10.605	-7.707	-7.5731	-10.313	-7.3495	-7.7067	-6.8715	-7.1443	-6.8192	-7.0673	-7.3549	-8.1729	-9.4301	-6.9724	-8.4067	-8.3879	-7.1603	-6.8311	-6.9935	-7.0545	-11.408	-7.0613	-9.7833	-10.919	-8.6407	-7.0848
426	-6.9794	-10.514	-7.6829	-7.4131	-9.7312	-6.934	-7.2658	-6.6978	-7.1375	-6.7129	-7.0278	-7.2368	-8.1427	-9.0249	-6.9639	-8.2384	-8.0436	-7.0348	-6.8076	-6.9711	-6.9582	-11.069	-6.8035	-9.5407	-10.407	-8.3124	-6.8153
427	-6.9753	-10.284	-7.5829	-7.1049	-9.6387	-6.8901	-7.16	-6.6844	-6.9462	-6.6819	-6.9501	-7.0119	-8.0951	-9.0089	-6.8225	-7.8132	-7.6501	-6.6809	-6.7413	-6.8333	-6.7602	-10.685	-6.7906	-9.4244	-10.223	-8.1456	-6.798
428	1.2714	1.2551	1.2313	1.1805	1.2633	1.2515	1.2287	1.2447	1.2513	1.2518	1.2135	1.2882	1.3209	1.2317	1.2558	1.2714	1.3071	1.2742	1.2171	1.2263	1.2573	1.2517	1.2665	1.2307	1.2639	1.2568	1.2895
429	359.88	347.75	262.13	288.63	261.75	228.88	172.75	252.63	230.88	278.5	245.25	289.5	190.38	300	302.75	271.38	247.25	322.25	254.13	210.38	332.5	347.25	250.13	268.13	297	241.88	225.88
430	497.88	516.38	352.25	355.5	328.5	277.25	219.13	343.38	288.88	395.63	352.75	374.5	215.38	395.75	403.5	318	317.13	416.63	324.13	316	459	470.75	281.13	312.25	344	286.5	274.88
431	408.63	430.63	294.13	282.63	264.75	221.5	212.25	284.13	228.5	370.88	295.25	328.63	212.25	335.88	321	261.5	267.25	314.25	242.88	244.75	370.13	367.5	216.5	254.75	273.25	221.75	219.25
432	230.63	214.25	163.63	145.75	148.25	123.88	128	155.5	123	207.25	141.13	192.13	148.25	193.25	182.25	150.25	142.63	142.25	119.25	101.13	206.75	184.88	105.88	130	142.75	121.75	100.75
433	115.38	103.13	86.75	72.875	77	62.25	60.75	74.125	56.75	98.75	65.25	94.125	78	102.5	92.875	70.375	59.625	61.125	50.125	43.875	102.75	91	47.875	67.25	68.75	62.25	42.25
434	51.75	46	43.25	30.375	40.75	31.25	29.625	32.125	26.875	39.75	27.125	44	42.5	49.625	40.125	31.125	25	21.75	19.75	16.375	43.125	43.25	20.125	34	29.75	30.75	18
435	15.5	19.25	18.375	11.25	21.25	13.25	10.5	9.375	11.625	10.875	10.625	15.875	20.125	21.25	13	12.875	9.5	6.75	6.5	5.625	12.625	17.875	6	18.375	12.125	12.625	6
436	4.375	9.25	5.5	3.375	9	2.875	3.5	2.625	2.75	2.375	2.375	4.375	10	8.875	2.75	5.125	3.25	2.25	1.625	1.75	2.75	8.125	1.75	8.125	6.25	5.125	1.75
437	0.6484	0.6423	0.4136	0.5161	0.3561	0.2847	0.327	0.474	0.2509	0.8111	0.4008	0.5414	0.3655	0.7265	0.5573	0.3412	0.2345	0.241	0.3872	0.2572	0.6819	0.5089	0.2117	0.4361	0.3725	0.3169	0.2551
438	0.3477	0.3661	0.2276	0.2522	0.21	0.152	0.1566	0.2293	0.1286	0.4147	0.1891	0.2824	0.2139	0.4002	0.2771	0.1645	0.1018	0.0981	0.1643	0.1128	0.3208	0.2972	0.0883	0.2334	0.1864	0.1586	0.1067
439	1.7577	1.098	1.6546	1.5279	0.8007	1.0474	0.2435	0.9566	1.3977	1.2253	0.9829	1.4493	0.8655	2.1397	2.0415	1.1973	1.2	1.1553	1.8654	1.2564	2.344	1.6122	1.6984	1.6648	0.9199	1.4525	1.5442
440	1.8966	1.2145	1.6473	1.4966	0.652	1.1472	0.2107	0.934	1.3356	1.2826	1.0008	1.611	0.7643	2.1549	2.0979	1.2063	1.2734	1.3221	1.7996	1.3768	2.4253	1.5238	1.6169	1.6082	0.7645	1.4155	1.5378
441	1.8909	1.4593	1.6387	1.603	0.7463	1.195	0.3426	1.0203	1.3412	1.3375	1.1443	1.6373	0.6887	2.2609	2.0503	1.1933	1.2718	1.3418	1.9966	1.7029	2.827	1.8828	1.6638	1.9577	1.0908	1.5383	1.7449
442	2.0875	1.7159	1.8627	1.8106	0.8879	1.339	0.4197	1.2278	1.5408	1.5709	1.2051	1.736	0.8066	2.4015	2.2899	1.393	1.4257	1.435	2.2879	2.4018	3.4038	2.4562	1.8315	2.3699	1.4584	1.8238	2.0882
443	2.3061	1.8938	2.0565	2.0866	1.0223	1.493	0.4636	1.2831	1.6838	1.8487	1.2076	1.9263	1.0577	2.5534	2.5369	1.6035	1.629	1.5101	2.5657	3.0929	3.6667	2.7897	2.0728	2.5669	1.6226	2.0663	2.3751
444	2.4285	1.8614	2.3571	2.271	0.9863	1.4227	0.4131	0.7142	1.6895	2.2186	1.2175	2.1062	1.2541	2.6508	2.7812	1.8848	1.7632	1.6374	2.6423	2.6615	3.3787	2.1609	2.4941	1.942	0.8727	1.8979	1.9873
445	2.0609	1.0384	2.0663	2.0676	0.6476	1.9582	0.4553	1.7006	1.5492	1.9275	1.3334	1.8927	1.0708	2.2372	2.6706	1.8238	1.8645	1.5226	1.4823	1.7801	2.6377	1.0781	2.6219	1.1323	0.947	1.7395	1.4871
446	1.5991	1.0986	1.5714	1.4294	0.729	2.0233	1.3272	2.3636	2.095	2.1675	1.5442	1.6469	0.6768	1.7957	2.3081	1.4543	1.5318	1.4851	1.4223	1.8745	3.6654	1.2449	2.2042	1.4817	1.927	1.1905	1.1555
447	0.3791	0.0934	0.1848	0.4146	0.2203	0.4652	0.1507	0.4143	0.4406	0.2641	0.4163	0.1939	0.3244	0.2261	0.2003	0.3785	0.232	0.208	0.6096	0.3543	0.6992	0.6182	0.3071	0.6362	0.3705	0.5307	0.7244
448	0.8481	0.4269	0.8403	0.8895	0.6626	1.0713	0.2641	0.8673	1.1784	0.5907	0.6577	0.3047	0.7357	0.7369	0.7856	0.8098	0.6637	0.3775	1.7149	1.1783	1.7718	1.5598	1.4263	1.4638	1.0727	1.5794	1.5363
449	1.3685	0.7638	1.3316	1.5028	1.1418	1.5656	0.2889	1.0063	1.7082	0.8308	0.9659	0.6838	1.1229	1.3443	1.4153	1.1516	1.2942	1.104	2.0445	1.5175	2.0322	1.781	2.2547	1.7691	1.1654	1.9864	1.6758
450	1.6891	1.1414	1.4809	1.8504	1.5669	2.1839	0.3431	1.0815	2.1683	0.8441	1.4377	0.9823	1.2154	1.7921	1.9891	1.1888	2.0356	1.8804	2.2162	2.1323	2.106	1.8629	2.7442	2.2666	1.3604	1.9554	2.0087
451	1.8211	1.6962	1.6036	2.1052	1.8301	2.733	0.6452	1.0021	2.6089	0.8073	1.9938	1.3017	1.3889	1.983	2.1686	1.1583	2.3266	2.7393	2.3224	2.7299	2.0994	2.0534	3.2848	2.6084	1.6364	1.8638	2.5558
452	2.2341	2.3219	1.6933	2.5583	2.239	3.2913	0.6617	0.8546	3.0599	1.1599	2.6497	1.653	1.8245	2.2562	2.4855	1.088	2.8876	4.4424	2.8262	3.5087	2.2671	2.147	3.8239	3.1436	2.3008	1.73	3.4886
453	2.8705	2.8318	2.1206	3.1382	2.7797	3.7161	0.8469	2.0479	3.3896	2.6445	3.179	2.0849	2.645	2.9399	3.1588	1.1187	3.8037	5.7916	3.7162	4.7502	3.1468	2.8052	5.4602	3.555	3.1443	1.1876	4.7792
454	3.1344	2.9618	2.2304	3.536	3.6551	4.0737	0.6005	2.1733	4.3313	2.5013	4.1684	3.0097	2.4088	3.6872	4.2743	1.0891	4.5829	5.9772	5.1777	4.7578	3.6963	3.1356	5.6387	3.6198	3.3909	0.9943	5.2528
455	1.3213	1.3602	1.4479	1.3305	1.3801	1.3823	1.4195	1.3943	1.3909	1.3494	1.3701	1.3417	1.3597	1.3239	1.3288	1.3457	1.4193	1.4077	1.324	1.3475	1.3318	1.3588	1.4078	1.2899	1.3735	1.3816	1.3595
456	405.76	413.65	442.55	329.38	434.29	423.58	422.77	423.97	428.75	403.97	379.36	435.18	481.82	370.4	395.47	420.89	508.79	463.61	357.44	378.74	398.71	409.54	455.34	350.8	430.75	428.58	448.23
457	536.13	562.63	640.75	438.25	599.38	585.5	600.13	591.13	596.38	545.13	519.75	583.88	655.13	490.38	525.5	566.38	722.13	652.63	473.25	510.38	531	556.5	641	452.5	591.63	592.13	609.38
458	1266.5	1285.6	1250.9	1055.3	1258.8	1132.9	1234.9	1165.9	1172.1	1295.5	1100.3	1311.3	1400.4	1159.5	1175.8	1164.4	1468.4	1323.4	936.38	1009.1	1140.4	1152.4	1148.4	982.25	1140.8	1050.4	1214.3
459	901.88	923.63	814	729.5	814	678.88	817.5	767.88	709.5	954	734.38	928.75	927.38	825.13	804.63	733.63	929.13	874	588.5	651	778.75	773.5	660.25	637.88	711.25	618	789.38
460	549	567.25	460.13	420.88	449.13	376.25	451.38	455.75	379.75	599.75	420.88	555.88	522.88	498.13	473.25	421.25	509.88	464.25	350.5	369.38	498.88	475.63	352.88	382.25	409.63	356.5	434
461	261.25	257.13	212.75	181.25	195.88	161	189.38	213.75	159.63	280.25	177.13	252.75	229.5	239.25	223.88	188.38	204.63	180	158.25	137.13	246.5	220.25	148.13	165.75	186.25	160.63	164.38
462	124	119.63	102.38	84.25	91.25	74.125	78	97	67.125	126.13	77	113.63	106	116.88	105.13	80.875	79.375	71	66.5	54.75	115.5	107.25	62	78.5	85.625	73.875	60.875
463	53.5	51.25	46.375	32.25	43.375	33.25	31.25	39.375	28.625	48.625	29.875	47.375	49.625	52.375	42.75	32.25	28.25	22.875	24.75	18.75	45.875	50.75	22.375	35.5	36.375	32.625	21
464	15.5	21.25	18.375	11.25	21.25	13.25	10.5	11.625	11.625	13.375	10.625	15.875	21.875	21.25	13	12.875	9.5	6.75	7.75	5.625	12.625	20.5	6	18.375	14.125	12.625	6
465	4.375	10.125	5.5	3.375	9	2.875	3.5	3.125	2.75	3.125	2.375	4.375	10.125	8.875	2.75	5.125	3.25	2.25	1.625	1.75	2.75	9.375	1.75	8.125	6.875	5.125	1.75
466	906.63	937.88	988.75	766.63	997	904	1062.1	913.25	941.25	1017	855	1021.8	1210	859.5	873	893	1221.1	1001.1	682.25	798.75	807.88	805.13	898.25	714.13	843.75	808.5	988.38
467	404	407.25	461.75	374	485.5	401.63	598.38	424.5	420.63	558.38	381.63	554.25	712	429.38	401.13	415.63	612	457.38	264.38	335	319.75	302.75	379.13	325.63	367.25	331.5	514.5
468	140.38	136.63	166	138.25	184.38	154.75	239.13	171.63	151.25	228.88	125.63	227.25	310.63	162.25	152.25	159.75	242.63	150	107.63	124.63	128.75	108.13	136.38	127.5	136.38	134.75	214.75
469	30.625	42.875	49.125	35.5	47.625	37.125	61.375	58.25	36.625	73	36	60.625	81.25	46	41.625	38.125	62	37.75	39	36	39.75	35.375	42.25	35.75	43.5	38.875	63.625
470	8.625	16.5	15.625	11.375	14.25	11.875	17.25	22.875	10.375	27.375	11.75	19.5	28	14.375	12.25	10.5	19.75	9.875	16.375	10.875	12.75	16.25	14.125	11.25	16.875	11.625	18.625
471	1.75	5.25	3.125	1.875	2.625	2	1.625	7.25	1.75	8.875	2.75	3.375	7.125	2.75	2.625	1.125	3.25	1.125	5	2.375	2.75	7.5	2.25	1.5	6.625	1.875	3
472	0	2	0	0	0	0	0	2.25	0	2.5	0	0	1.75	0	0	0	0	0	1.25	0	0	2.625	0	0	2	0	0
473	0	0.875	0	0	0	0	0	0.5	0	0.75	0	0	0.125	0	0	0	0	0	0	0	0	1.25	0	0	0.625	0	0

Table S12 Amino acid descriptors (2D) used in this study.

	I	I	I	I	I	I	I	I	I	I	I	K	K	K	K	K	K	K	K	K	K	K	K	K	K	K	K	K	K	K	K	L	L	L	L	L	L	L	L	L	L	L	L	L	L	L	L	L	L	L	L	M	M	M	M
	L	M	N	P	Q	R	S	T	V	W	Y	A	C	D	E	F	G	H	I	K	L	M	N	P	Q	R	S	T	V	W	Y	A	C	D	E	F	G	H	I	K	L	M	N	P	Q	R	S	T	V	W	Y	A	C	D	E
No.	IL	IM	IN	IP	IQ	IR	IS	IT	IV	IW	IY	KA	KC	KD	KE	KF	KG	KH	KI	KK	KL	KM	KN	KP	KQ	KR	KS	KT	KV	KW	KY	LA	LC	LD	LE	LF	LG	LH	LI	LK	LL	LM	LN	LP	LQ	LR	LS	LT	LV	LW	LY	MA	MC	MD	ME
1	-7	-5	0	0	-4	1	-3	-3	3	2	-1	3	-5	-3	-4	0	-7	-2	0	-8	-1	-3	0	-2	-1	0	-1	5	-4	-4	-2	1	-3	-2	-3	1	2	5	-7	-1	1	-4	-5	-2	0	0	-4	-1	-3	0	-2	-5	-5	-4	9
2	-3.7	-1.3	-0.1	0.1	-3.4	1.1	-4.1	-1.6	3.9	1.4	0.1	4.4	-5.3	-4.2	-4.3	0.1	-5.7	-2.8	-0.4	-6.3	-1.2	-3.6	0.1	-1.7	-0.2	0.8	0.9	5.8	-3.2	-1.2	-2.5	1.2	-2.4	-0.9	-1.5	2.5	2.5	5.2	-3.7	-1.2	0.6	-1.2	-4.4	-1.4	-0.6	0.5	-5	0.5	-1.3	0.1	-3.8	-2.3	-1.6	-0.4	12.1
3	-3.7	-1.7	0.4	-0.1	-3.5	1.2	-3.6	-1.4	3.6	1.4	-0.4	3.9	-4.9	-4	-3.9	0.5	-5.4	-1.8	0.1	-6.4	-0.7	-2.4	0.3	-0.7	0.2	0.7	1	6.2	-2.9	-1.5	-2.7	1.1	-2.3	-1.2	-1	2.2	2.4	4.8	-3.7	-0.7	0.6	-1.5	-5.2	-0.6	-0.2	0.5	-4.4	0.5	-1.7	0.3	-3.2	-3	-1.9	-1.6	12.6
4	-3.2	-2	0.4	-0.2	-3.1	1	-2.9	-1.1	3.2	1.2	-0.8	2.9	-4.2	-3.9	-3.2	0.7	-4.7	-1	0.4	-5.5	-0.2	-1.2	0.4	-0.1	0.4	0.2	1.2	6.6	-2.4	-1.3	-2.8	0.9	-2.2	-1.4	-0.5	1.6	2.2	4.8	-3.2	-0.2	0.8	-1.3	-4	0.1	-0.3	0.4	-3.1	-0.5	-1.4	0.4	-2.6	-2.8	-1.8	-2.2	11.8
5	-1.6	1	-2.6	1	-2.2	0.3	-2.3	-2.1	1	1.8	-1.8	3.8	-2.4	-2.1	-2.5	0.4	-1.7	-2.2	-2.1	-2.9	-2.1	-1.9	1.7	-1.7	0.3	1.1	2	4.2	-0.4	-1.6	0.9	-0.3	-2.2	2.5	-2.3	0.3	1.7	4.8	-1.6	-2.1	0.3	-1.6	-3	-2.3	1	0.9	-2.5	1.4	-2.3	1.7	-1.2	2.3	-1.5	0.7	5.5
6	-0.24	0.25	0.22	-0.03	-0.39	0.13	-0.51	-0.13	0.7	0.32	-0.1	0.37	-0.55	-0.54	-0.25	0.2	-0.4	0.09	0.22	-0.41	0.23	0.26	0.22	-0.15	0.18	0.11	0.54	1	-0.2	0.18	-0.37	0.14	-0.46	-0.27	-0.01	0.57	0.56	1	-0.24	0.23	0.19	0.18	-0.39	-0.06	-0.08	0.13	-0.45	-0.23	0.2	0.22	-0.29	-0.34	-0.14	-0.2	1
7	-5	-3	0	0	-3	1	-2	-1	4	2	-1	3	-4	-2	-3	1	-6	-1	0	-7	0	-2	1	-1	0	1	0	5	-3	-3	-2	1	-2	-2	-1	1	2	4	-5	0	2	-3	-4	-1	0	0	-3	0	-2	1	-2	-4	-4	-4	12
8	1	3	3	3	1	3	1	2	5	4	2	5	1	1	0	3	1	2	3	0	2	2	3	3	3	4	4	6	2	2	2	5	2	3	2	3	5	6	1	2	4	2	0	3	4	4	1	4	2	3	2	2	2	2	6
9	2	1	2	1	2	2	3	2	1	1	2	1	2	2	3	2	2	2	2	3	2	2	1	2	2	1	1	0	1	2	1	1	2	1	2	1	1	0	2	2	2	2	3	1	1	1	2	1	2	1	2	1	2	1	0
10	5	8	10	8	7	6	3	10	10	9	8	10	3	3	4	10	2	4	7	1	5	9	5	9	9	6	9	10	3	9	7	10	7	6	9	6	6	10	5	5	6	9	4	10	5	9	3	6	8	5	3	3	8	4	10
11	-3.2	-2	0.4	-0.2	-3	0.9	-2.7	-1	3.1	1.2	-0.8	2.7	-4	-3.8	-3	0.7	-4.5	-0.7	0.5	-5.2	0	-1.1	0.4	0	0.5	0.1	1.2	6.6	-2.2	-1.3	-2.8	0.9	-2.2	-1.4	-0.3	1.5	2.2	4.7	-3.2	0	0.9	-1.3	-3.6	0.2	-0.3	0.5	-2.8	-0.8	-1.2	0.4	-2.4	-2.8	-1.8	-2.2	11.5
12	154	159	80	76	153	42	134	87	29	68	94	45	172	168	160	46	177	108	65	181	85	198	81	42	101	94	56	0	102	174	149	46	133	143	96	43	23	0	154	85	61	174	174	68	126	65	138	98	96	81	97	160	139	180	0
13	-3	-3	0	-1	-3	0	-3	-2	3	1	-1	2	-3	-4	-3	0	-4	-1	0	-4	-1	-3	0	-1	0	-1	1	7	-2	-3	-2	1	-3	-2	-1	1	2	7	-3	-1	0	-3	-4	0	-2	0	-2	-2	-1	0	-3	-2	-2	-3	12
14	-5	-4	-1	-2	-5	0	-5	-3	4	1	-2	2	-5	-5	-5	0	-5	-2	0	-6	-2	-2	-2	-1	-1	-2	1	8	-3	-4	-5	1	-4	-3	-2	1	2	9	-5	-2	0	-4	-6	0	-3	0	-4	-3	-2	0	-4	-5	-4	-5	13
15	-7	-6	-1	-3	-6	-1	-6	-4	4	1	-3	2	-7	-7	-6	0	-6	-3	-1	-8	-2	-2	-2	-1	-2	-3	1	9	-4	-7	-6	1	-5	-4	-3	1	2	10	-7	-2	-1	-7	-7	-1	-3	0	-6	-4	-3	0	-5	-6	-5	-6	13
16	-9.7	-8.2	-1.4	-3.6	-4.8	-0.7	-4.8	-2.8	3.9	1.7	-2.2	2.4	-8	-4.8	-5.9	-0.8	-7	-1	-0.2	-6	-1.8	-7.7	-0.7	-0.4	-1.5	-2.1	1.1	8	-3.7	-8.2	-4.7	1	-5.7	-1.3	-3.4	2.1	2.6	8.5	-9.7	-1.8	-1.1	-8.2	-6.1	-1.2	-1.6	0.1	-5.6	-2.5	-3.3	-0.7	-5.4	-3.8	-7.6	-5.6	16.1
17	-3	-1	0	0	-3	1	-3	-1	4	1	0	4	-4	-3	-3	0	-5	-2	0	-5	-1	-2	0	-1	0	1	1	5	-3	0	-2	1	-2	-1	-1	2	2	5	-3	-1	1	0	-5	-1	0	1	-4	0	-1	0	-3	-2	-1	-1	11
18	-3	-1	-2	0	-4	1	-4	-1	2	3	0	8	-5	-3	-3	3	-6	-2	0	-4	0	-1	3	-2	3	3	1	6	-3	-1	-3	2	-3	-1	1	6	6	12	-3	0	2	-1	-3	-1	0	1	-5	3	-2	3	-3	-2	-1	-1	6
19	0	0	0	0	0	0	0	0	9	5	0	0	0	0	0	0	0	0	6.6	0	0	6.6	5	5	0	0	9	10	6.6	9	0	6.6	0	0	0	9	9	10	0	0	0	9	0	0	0	0	5	0	5	6.6	0	0	0	0	10
20	0	0	0	0	-1	0	-1	0	0	1	0	0	1	-1	-1	-1	1	-1	0	0	-1	0	0	0	0	0	0	2	-1	0	-1	0	-1	-1	0	0	1	2	0	0	0	0	-1	0	0	0	-1	0	0	0	-1	-1	0	0	2
21	-13	-15	-4	-6	-4	2	-3	-8	24	2	1	6	-10	-7	-7	3	-15	-9	2	-10	-2	-13	-5	1	-2	-7	3	39	-4	-23	-3	3	-3	-4	-2	6	4	29	-13	-2	2	-23	-6	2	1	2	-4	-6	1	-5	-4	-10	-7	-6	109
22	-14	-36	5	-7	-10	8	-5	3	17	6	-1	12	-16	-6	-13	6	-25	-7	8	-16	8	-17	5	8	13	6	13	52	-10	-20	-3	8	-3	0	8	6	8	17	-14	8	8	-20	-6	8	6	6	-14	8	-10	5	-4	0	-11	-10	93
23	-1	4	4	1	-12	1	-10	2	18	3	-5	6	-18	-9	-7	2	-18	1	3	-12	1	4	1	2	-4	2	-4	29	-7	0	-6	4	-6	-10	0	2	4	23	-1	1	3	0	-18	2	-2	3	-16	1	-18	1	-3	-14	1	2	9
24	-13	3	7	2	-6	5	-2	5	5	-3	-2	20	-20	-13	-11	4	-26	5	3	-55	1	3	7	3	9	0	14	53	-3	-30	0	4	-1	-4	5	9	11	71	-13	1	10	-30	-34	4	-1	3	-10	8	-14	7	-1	-8	4	-1	50
25	-26	-33	-4	1	-11	0	-7	3	11	14	-2	10	-21	-10	-7	1	-17	-2	0	-40	-1	-26	12	2	-2	3	2	59	-8	-33	-8	0	-9	-2	0	2	9	38	-26	-1	3	-33	-30	-6	-4	-4	-13	4	-7	12	-2	-13	6	-28	156
26	0	-10	6	-17	-15	22	-18	4	11	13	-8	29	-27	-22	5	18	-24	-26	17	-24	2	-29	24	4	30	-4	10	48	-12	-2	-14	13	-17	13	-1	24	18	80	0	2	18	-2	-11	10	4	8	-21	13	-6	24	-14	7	5	-7	66
27	-15	-21	-2	-6	-7	3	-5	-6	24	2	-1	8	-14	-9	-9	4	-20	-8	3	-13	-1	-13	-3	2	1	-5	5	41	-7	-23	-4	4	-4	-2	1	7	5	27	-15	-1	4	-23	-7	3	2	3	-8	-4	-3	-3	-6	-7	-9	-9	108
28	-4	4	6	5	-14	5	-10	3	13	5	-6	10	-21	-12	-9	5	-24	5	5	-35	2	5	3	3	2	4	3	37	-10	-5	-7	5	-8	-7	3	5	7	28	-4	2	6	-5	-38	4	0	4	-18	3	-19	3	-6	-12	1	1	51
29	-16	-18	0	-2	-13	8	-13	4	11	13	-7	17	-22	-17	-5	7	-21	-7	8	-32	2	-30	17	4	8	1	10	54	-10	-12	-12	6	-12	4	2	9	13	49	-16	2	9	-12	-22	2	-1	2	-14	6	-7	17	-9	-5	-5	-19	97
30	1	1	3	3	1	4	1	2	5	4	2	5	1	0	2	4	1	3	3	0	3	1	4	3	3	3	4	8	2	3	1	5	1	2	1	5	5	8	1	3	4	3	0	4	3	3	1	3	2	4	1	1	1	1	9
31	0	0	0	0	0	1	0	0	2	1	0	2	0	0	0	2	0	0	0	0	0	0	0	0	0	0	0	6	0	0	0	1	0	0	0	0	3	5	0	0	1	0	0	1	0	1	0	0	0	0	0	0	0	0	6
32	-0.41	-0.18	-0.11	0.15	-0.9	0.19	-0.86	-0.14	0.41	0.21	-0.45	0.32	-0.75	-0.78	-0.9	0.2	-0.62	-0.15	-0.14	-0.64	-0.1	-0.13	0.18	-0.07	0.16	0.15	0.25	0.43	-0.74	-0.29	-0.83	-0.02	-0.69	0.12	-0.06	0.15	0.2	0.36	-0.41	-0.1	0.16	-0.29	-0.79	-0.2	0.04	0.11	-0.8	0.13	-0.45	0.18	-0.82	0.09	-0.46	-0.35	1.02
33	3.48	2.22	1.96	1.92	3.49	0.85	3.39	2.48	0.85	1.29	2.69	0.9	4.1	3.98	3.69	0.99	4.27	2.4	1.87	4.88	2.05	1.63	1.72	1.12	2.05	2.37	1.14	0	2.62	2.56	3.37	1.31	2.76	3.42	2.34	1.13	0.65	0	3.48	2.05	1.47	2.56	4.39	1.65	2.37	1.4	3.53	2.78	2.76	1.72	2.49	3.95	2.83	3.06	0
34	8	8	10	7	7	10	4	8	12	10	9	11	6	3	5	11	4	8	10	6	9	8	9	10	11	9	11	16	6	10	5	11	5	8	10	10	11	16	8	9	11	10	8	10	9	10	7	6	11	9	4	7	9	8	16
35	-0.1	0.12	0.37	-0.18	-0.23	-0.24	-0.14	-0.28	0.67	0.04	-0.25	0.07	0.01	-0.28	-0.09	-0.14	-0.31	0.02	0.02	-0.19	-0.18	-0.15	-0.01	-0.21	0.13	0.08	0.5	1	0	-0.04	-0.06	0.04	-0.15	0.04	-0.08	0.05	0.3	1	-0.1	-0.18	0.11	-0.04	-0.21	-0.23	0.05	0.01	0.57	-0.45	0.51	-0.01	0.05	-0.19	0.04	-0.17	1
36	-0.1	0.13	0.4	-0.18	-0.24	-0.27	-0.13	-0.32	0.72	0.04	-0.26	0.09	0.01	-0.29	-0.08	-0.16	-0.35	0.01	0.03	-0.22	-0.19	-0.16	0	-0.22	0.14	0.08	0.58	1	0.03	-0.01	-0.07	0.06	-0.16	0.05	-0.08	0.1	0.35	1	-0.1	-0.19	0.13	-0.01	-0.24	-0.22	0.07	0.01	0.62	-0.46	0.54	0	0.06	-0.22	0.04	-0.2	1
37	-7	-6	-1	-2	-3	0	-3	-2	2	2	-2	2	-6	-3	-4	0	-5	-1	0	-6	-1	-4	0	0	-1	-1	1	5	-3	-6	-3	0	-4	-1	-2	1	2	6	-7	-1	0	-6	-5	-1	-1	0	-4	-2	-2	0	-3	-3	-6	-2	11
38	0.035	0.246	0.293	-0.168	0.191	0.206	0.212	0.494	0.641	0.299	0.476	0.137	0.261	0.242	-0.295	0.082	0.475	0.008	0.012	0.578	0.328	-0.029	-0.117	0.344	0.184	0.25	0.247	1	0.446	0.527	0.452	-0.051	0.512	0.387	-0.109	-0.084	0.037	1	0.035	0.328	0.321	0.527	0.277	0.404	0.299	0.076	0.291	0.164	0.555	-0.117	0.305	0.32	0.105	0.273	1
39	0.015	0	0	0.598	-0.172	0.408	-0.135	0	0.946	0.305	0.888	0.851	-0.249	-0.185	-0.109	0.222	-0.107	0.276	0.245	-0.079	0.382	0.717	0.067	0.61	-0.064	0	-0.051	0	0.049	0.425	-0.101	0.661	-0.203	0.338	-0.054	0.243	0.452	1	0.015	0.382	0.257	0.425	0.003	0.357	-0.018	0.519	-0.223	0	0.862	0.067	0.108	0.225	0.032	0.113	1
40	0	1	2	2	0	3	0	2	6	2	5	4	0	0	0	1	0	1	2	0	4	4	0	3	0	0	1	8	0	4	0	3	0	2	-1	0	3	8	0	4	2	4	0	2	0	3	0	-1	5	0	0	1	0	0	8
41	-1.7	-1.7	0.2	-0.6	0.8	1.4	1.5	0.2	2.2	-0.3	1.7	1	-0.2	0	-0.5	1.6	-0.3	-1.2	0.7	-1.4	0	-1.6	-1.3	1.7	0.1	-0.4	1.4	2.2	1.2	-1.8	0.9	1.9	1.1	-0.1	-0.1	2	0.8	2.2	-1.7	0	0.6	-1.8	-1.1	1.8	0.2	1.1	0.9	0.3	1.3	-1.3	1.4	-0.4	-1.6	-1.5	2.2
42	-20	-1	10	0	-35	13	-36	-10	46	8	-17	34	-40	-39	-16	12	-31	1	7	-20	11	12	10	-10	10	8	24	100	-24	3	-32	1	-30	-13	2	26	32	100	-20	11	8	3	-6	-7	3	12	-40	-5	-6	10	-25	-22	-7	-12	100
43	-8	1	8	1	-9	10	-11	0	9	5	-6	15	-11	-14	-7	13	-10	11	8	-5	4	2	8	6	16	7	17	24	-8	-2	-11	7	-11	-3	13	16	16	26	-8	4	16	-2	-5	6	0	4	-8	1	-2	8	-11	-4	-4	-9	18
44	-8	0	7	0	-9	8	-10	0	11	2	-3	10	-10	-12	-6	10	-9	7	6	-4	3	4	4	2	11	8	13	18	-7	-1	-11	6	-11	-4	9	12	12	16	-8	3	13	-1	-4	4	0	2	-7	0	0	4	-10	-5	-5	-7	14
45	30	94	64	76	52	79	29	68	97	89	91	94	25	23	21	96	19	44	39	31	35	93	61	67	98	36	96	100	6	69	51	66	54	66	81	49	73	100	30	35	68	69	58	71	33	63	31	43	93	61	4	38	58	11	100
46	-2	0	0	0	-1	0	-4	0	2	0	0	0	-3	-4	-1	1	-4	-1	-1	-3	-1	0	-1	1	-1	-2	-1	4	-1	0	-1	1	-1	0	-1	0	0	4	-2	-1	-1	0	-1	0	-2	1	-3	-2	0	-1	-2	-3	0	-1	4
47	1	3	0	1	2	0	-1	2	-1	-1	1	-2	1	-1	2	1	-1	0	-1	1	0	1	0	2	0	-1	-2	-2	1	3	2	0	2	2	1	-1	-1	-2	1	0	-1	3	3	0	0	1	0	0	1	0	1	0	3	2	-5
48	1.44	1.36	0.96	0.91	0.98	1.21	0.96	1.11	0.83	1.13	1.08	1.31	1	0.93	0.94	1.2	0.79	0.84	0.97	0.87	1.01	1.24	1.05	1.02	1.18	1.02	1.22	0.86	1.02	1.5	0.89	1.01	0.96	0.83	1.18	1.1	1.08	1.11	1.44	1.01	1.25	1.5	0.88	1.09	1.31	0.93	1.01	1.06	1.19	1.05	0.91	0.73	1.36	1.21	1.04
49	0.67	0.8	0.71	0.83	0.97	0.74	1.15	0.78	1.42	0.78	1.11	0.58	0.96	1.06	0.93	0.71	1.17	0.75	0.77	1.04	0.88	0.96	0.68	1.02	0.64	0.61	0.78	0.97	1.05	0.69	1.12	0.84	1.05	1.08	0.63	0.83	0.69	0.66	0.67	0.88	0.64	0.69	0.95	0.85	0.56	0.99	1.12	0.87	0.97	0.68	1.23	1.12	0.72	0.84	1.07
50	0.6	0.51	1.8	1.63	1.13	1.04	0.74	1.22	0.45	1.18	0.51	1.17	1.09	1.04	1.34	1.13	1.17	2.04	1.63	1.24	1.27	0.43	1.63	0.89	1.38	1.88	0.93	1.5	0.82	0.39	1	1.35	0.99	1.27	1.41	1.13	1.52	1.43	0.6	1.27	1.17	0.39	1.46	1.12	1.23	1.23	0.69	1.15	0.57	1.63	0.69	1.46	0.7	0.82	0.71
51	14	15	18.1	18	11.7	20	14.9	16.3	22.2	18.9	17.2	22.8	12.5	13.7	15.5	18.6	9.04	19.4	19.1	9.08	19	20.9	17.2	17.4	18.2	18.7	19.2	26.8	12.2	13.5	14.8	20.9	14.8	20.1	18.2	19.6	21.4	25.9	14	19	22.2	13.5	7.74	17.6	18.2	20.2	11.4	19.3	13.6	17.2	14.3	14.9	14.7	14.4	31.9
52	2	3.2	5.6	5	2.2	6.1	2.5	4.2	8.3	6.4	4.4	7.9	1.2	1.4	2.2	5.9	0.7	4.5	5.7	0	5.2	4.1	5.6	5.2	5.7	5.3	6.4	11.8	3	3.9	2.4	6.1	3	3.8	4.9	6.7	7.4	9.9	2	5.2	6.1	3.9	1.6	5.4	4.9	5.7	2.4	4.4	4	5.6	2.8	2.4	3.4	3	16.7
53	-13	-15	1	-4	-7	1	-8	-3	8	5	0	5	-10	-9	-7	1	-6	2	0	-7	0	-3	1	2	0	2	6	16	-4	-18	-8	3	-4	1	-2	7	5	17	-13	0	1	-18	-9	0	0	2	-8	0	-2	1	-7	-6	-13	-16	39
54	-2.51	-2.11	0.79	0.24	-2.1	-0.16	-1.81	-0.12	1.94	0.76	-0.97	1.2	-2.65	-2.77	-2.58	0.33	-2.19	0.72	0.71	-1.91	-0.75	0.13	-0.01	0.6	0.66	-1.2	1.11	4.36	-1.19	-1.5	-2.43	0.4	-2.17	-0.36	0.13	0.13	1.78	4.02	-2.51	-0.75	0.34	-1.5	-2.89	1.04	-0.73	0.3	-2.11	-0.41	-0.39	-0.01	-1.4	-1.21	-2.08	-1.3	6.99
55	-6.98	-5.7	1.16	1.3	-5.64	-0.39	-5.89	-0.24	5.23	1.77	-1.72	2.41	-7.41	-6.18	-7.88	1.42	-5.06	0.81	2.34	-5.63	-1.36	-0.13	0.32	1.08	1.53	-3.91	3.08	11.64	-2.83	-5.95	-5.78	1.54	-5.65	-0.93	-0.33	-0.74	4.07	11.01	-6.98	-1.36	1.1	-5.95	-6.29	2.59	-2.38	1.14	-5.62	-1.8	-1.09	0.32	-3.93	-3.85	-6.53	-6.29	19.05
56	-3.1	-1.3	0.7	1	-2.4	2.3	-3.1	-0.3	4.5	1.4	0.3	3.2	-3.4	-2.9	-2.8	1.9	-3.7	-0.2	1.7	-5.7	0.1	-3.2	0.5	-0.1	1.4	0.9	1.2	7.5	-1.5	-1.7	-2.4	1.4	-0.9	-0.8	0.1	2	3.3	5.3	-3.1	0.1	1.1	-1.7	-4	0.9	1	1.4	-1.5	0.5	-0.2	0.5	-1.9	-2.9	-1.9	-1.3	11.5
57	-4	0	5	1	-7	6	-9	0	9	2	-2	7	-8	-9	-4	5	-6	6	4	-4	2	4	3	1	7	4	9	12	-5	-2	-9	4	-8	-2	4	5	8	11	-4	2	6	-2	-4	2	0	2	-6	0	0	3	-7	-3	-5	-5	14
58	0	-1	3	1	0	2	-4	-1	4	7	1	6	-1	-4	1	-2	[illegible]	-3	3	3	-4	3	-1	2	3	0	3	2	0	-2	5	-4	5	-1	-3	3	-2	2	-2	0	3	3	5	-3	0	1	-1	3	3	2	1	0	-4	-3	12
59	-1	-1	1	0	-2	0	-3	0	7	4	0	5	-7	-7	-6	2	-6	1	2	-5	1	1	2	-2	1	1	4	6	-2	1	-8	3	-3	0	0	3	5	5	-1	1	3	1	0	4	-1	4	-2	-1	0	2	0	-2	0	-1	13
60	0	0	2	0	0	0	-3	0	5	3	1	4	-3	-3	-2	3	-4	3	2	-5	2	-4	3	0	2	2	3	1	-2	2	-7	4	0	0	0	1	1	0	0	2	3	2	-1	1	-1	3	0	1	2	3	1	-1	-1	-4	12
61	-8.3	-7.5	-6.8	-8.1	-7.7	-6.5	-8.3	-7.6	-5.7	-6.8	-7.1	-6.4	-7.1	-8.3	-8.2	-7.1	-9.5	-7.3	-5.7	-9.8	-6.5	-9	-8.1	-7	-6.9	-6.3	-6.7	-4.2	-7.9	-9.3	-7.6	-6.5	-7.2	-7	-7.3	-7.5	-6.3	-5	-8.3	-6.5	-7.3	-9.3	-8.6	-7	-6.9	-6.4	-7.4	-7.4	-6.7	-8.1	-7.7	-7.2	-8.6	-8.3	-3.2
62	-30	-29	7	-11	-32	3	-33	-20	35	12	-5	20	-43	-34	-30	2	-33	-7	4	-40	-10	-6	-7	-7	-4	-10	14	67	-20	-29	-32	10	-31	-19	-18	12	18	65	-30	-10	2	-29	-39	0	-20	0	-31	-23	-7	-7	-28	-20	-30	-29	99
63	-7	-2	-1	-3	-3	0	-5	-2	3	4	0	6	-5	-5	-5	2	-5	-5	-4	-7	-5	-3	-1	-3	-5	-2	-4	9	-6	-7	-3	1	-3	2	-7	-2	2	12	-7	-5	0	-7	-5	-1	-5	-1	-5	-3	-1	-1	-6	-4	-2	-8	7
64	-7.5	-1.5	-3	-6	-3.5	-4.5	-7	-3.5	4.5	2.5	0	2	-6	-6.5	-6	-1	-5.5	-7	-5.5	-6.5	-5.5	-0.5	-3.5	-3.5	-5	-4.5	-7.5	7	-4.5	-5	-4	0.5	-4.5	0	-9.5	-2.5	-0.5	9	-7.5	-5.5	-2.5	-5	-4	-2.5	-6.5	-1.5	-7	-6.5	0	-3.5	-5.5	-5.5	-2.5	-5	11
65	-5	-2	0	-1	-4	0	-5	-3	4	1	-1	3	-6	-6	-5	0	-7	-1	0	-7	-1	-1	0	-1	0	-1	1	8	-3	-2	-4	1	-4	-2	-3	2	3	7	-5	-1	1	-2	-5	0	-1	0	-4	-2	-2	0	-4	-5	-3	-3	13
66	-3	-1	0	0	-3	1	-3	-1	3	1	-1	3	-4	-4	-3	1	-5	0	0	-5	0	0	0	0	0	1	1	7	-2	-1	-3	1	-2	-1	-1	2	2	5	-3	0	1	-1	-4	0	-1	0	-3	-1	-1	0	-2	-3	-1	-2	11
67	-4	-3	-1	-1	-4	0	-4	-2	3	0	-2	1	-5	-6	-4	0	-5	-1	0	-5	-1	-1	-1	-1	0	-1	1	7	-3	-3	-5	0	-4	-2	-1	1	1	7	-4	-1	0	-3	-3	0	-2	-1	-4	-2	-2	-1	-3	-3	-1	-4	12
68	0.015	0.007	0.029	0.011	0.002	0.012	0.053	0.005	0.175	0.015	0.034	0.001	0.017	0.015	0.012	0.003	0.004	0.022	0.091	0.015	0.021	0.014	0.004	0.023	0.003	0.134	0.002	0.041	0.054	0.029	0.051	0.026	0.028	0.014	0.064	0.041	0.011	0.021	0.015	0.009	0.029	0.038	0.072	0.045	0.016	0.012	0.019	0.088	0.004	0.046	0.011	0.01	0.032	0.029	[illegible]
69	-1.9	-5.59	-7.13	-7.32	-7.43	-1.92	-0.26	-6.89	8.16	-6.44	-0.47	-9.95	-6.7	-2.2	-8.93	-1.22	-10.05	-2.34	4.09	-3.37	-10.31	-10.75	-7.3	-3.58	-13.72	0.93	-9.45	-1.52	-1.08	-5.77	-0.51	1.4	2.19	-5.02	-4	-6.13	-5.12	-1.42	-1.9	-10.31	-2.38	-5.77	-0.24	0.74	-6.26	-7.74	-1.93	-2.89	0.24	-7.3	0.31	-2.94	-1.44	-6.17	-7.45
70	1.9	1.7	1.8	2.6	-2.4	-1.6	0.5	0.4	11.8	1.7	1.2	-0.6	-0.4	2	-3	0.5	-3.6	2.7	2.4	-0.9	0.2	-0.4	1	-2.2	-1.6	3.6	-1.7	0.2	1	1.5	0.5	0.8	7.4	2	0.9	1.6	0.2	5.1	1.9	0.2	1.8	1.5	0.6	4.1	-1	0.2	-0.4	-2.1	1.6	1	0.6	-1.6	3.9	-1.4	-0.9
71	-2.14	-2.34	-2.76	-2.74	-3.75	-0.6	1.17	-2.93	7.02	-1.64	0.13	-3.62	-3.14	0.53	-3.48	-2.08	-2.65	-0.1	3.37	-1.03	-4.26	-4.39	-3.36	-1.56	-4.98	3.65	-4.55	-0.86	-0.27	-1.9	0.93	0.6	2.88	-1.24	-0.02	-0.96	-2.48	-0.08	-2.14	-4.26	-2.19	-1.9	1.17	2.32	-1.66	-3.19	-0.33	-1.78	1.09	-3.36	1.27	-1.2	-0.84	-1.99	-1.29
72	-0.2	0.1	0.5	-1.4	0	0.9	-2.4	-0.5	-2.1	0.2	-0.8	0.2	0.4	-0.3	-0.2	-0.4	0	0.4	2.7	-0.1	-0.1	-0.2	0.7	-0.8	0	3.4	0.1	2.2	-1.5	-0.5	-0.4	1.9	1.3	-0.5	1.8	0.9	-0.2	-0.8	-0.2	-0.1	-0.4	-0.5	0.1	1.5	-5.7	-0.1	-0.9	0.5	-1.3	0.7	-1.9	0.2	0.2	-1.8	-2
73	-3	-2	-3	-2	-4	-2	0	-3	4	-1	0	-4	-2	1	-4	-3	-4	1	2	-2	-3	-3	-3	-3	-4	3	-4	-1	0	-1	0	0	2	-1	0	-2	-1	-3	-3	-2	-1	-2	-1	0	2	-1	-2	-2	-3	1	-3	0	-2	-1	-1
74	-8	-6	-9	-5	1	-4	-6	-7	-15	-5	8	-5	-2	0	-5	-6	-8	-12	-6	-7	-2	-2	-5	-1	-6	-9	-10	-5	[illegible]	-7	-9	-5	-4	-15	-2	-12	2	-3	8	-2	1	-9	-14	0	7	-6	-6	8	7	-5	-1	-7	-12	-12	-7
75	-12.3	9.8	-7.3	8.9	-4.4	-7.9	39.5	11.1	4.6	13.2	24.1	-2.3	-7.3	-5.7	-9.4	8.9	12.5	-10	11	16.9	-3.8	-10.1	16.1	17	-7.7	8.4	0.5	-6	-3	12.7	13	9.3	24.8	25.1	-7.1	-8.9	-2.7	5.3	-12.3	-3.8	9.2	12.7	-5.3	0.8	-6	-7.4	-8.7	6.8	5.3	16.1	35.5	36.7	27.4	31	26.1
76	1.6	0.1	2.3	0.6	0.1	1.5	0.1	0.4	17.5	7.5	0.1	8.6	0.1	0.1	0.1	2	0.5	3.1	2.7	0.1	0.7	0.1	1.8	0.4	1.3	3.3	5.8	6	0.6	0.1	0.1	1.6	0.7	0.1	1.4	5.7	7.3	52.1	1.6	0.7	4.5	0.1	0.1	0.4	3.9	2.5	0.1	0.1	0.8	1.8	0.1	0.1	1.5	0.1	73.6
77	1.6	0.1	2.3	0.6	0.1	1.5	0.1	0.4	17.5	7.5	0.1	8.6	0.1	0.1	0.1	2	0.5	3.1	2.7	0.1	0.7	0.1	1.8	0.4	1.3	3.3	5.8	6	0.6	0.1	0.1	1.6	0.7	0.1	1.4	5.7	7.3	52.1	1.6	0.7	4.5	0.1	0.1	0.4	3.9	2.5	0.1	0.1	0.8	1.8	0.1	0.1	1.5	0.1	73.6
78	3.4	7.4	0.1	0.8	0.4	0.1	0.6	2.4	17.5	0.1	2.4	4.1	0.1	1.3	5.8	0.1	1.1	1.2	0.3	55.3	1	3	3.5	2.7	3.7	8.3	3.5	1.2	0.1	2.5	0.9	0.8	0.1	0.1	0.1	1.5	0.2	0.2	3.4	1	67.3	9.3	0.1	1.5	1.1	0.6	0.2	4.8	1.9	2.1	6.6	5.2	0.5	0.1	5.4
79	3.4	7.4	0.1	0.8	0.4	0.1	0.6	2.4	17.5	0.1	2.4	4.1	0.1	1.3	5.8	0.1	1.1	1.2	0.3	55.3	1	3	3.5	2.7	3.7	8.3	3.5	1.2	0.1	2.5	0.9	0.8	0.1	0.1	0.1	1.5	0.2	0.2	3.4	1	67.3	9.3	0.1	1.5	1.1	0.6	0.2	4.8	1.9	2.1	6.6	5.2	0.5	0.1	5.4
80	3.4	7.4	0.1	0.8	0.4	0.1	0.6	2.4	17.5	0.1	2.4	4.1	0.1	1.3	5.8	0.1	1.1	1.2	0.3	55.3	1	3	3.5	2.7	3.7	8.3	3.5	1.2	0.1	2.5	0.9	0.8	0.1	0.1	0.1	1.5	0.2	0.2	3.4	1	67.3	9.3	0.1	1.5	1.1	0.6	0.2	4.8	1.9	2.1	6.6	5.2	0.5	0.1	5.4
81	3.4	7.4	0.1	0.8	0.4	0.1	0.6	2.4	17.5	0.1	2.4	4.1	0.1	1.3	5.8	0.1	1.1	1.2	0.3	55.3	1	3	3.5	2.7	3.7	8.3	3.5	1.2	0.1	2.5	0.9	0.8	0.1	0.1	0.1	1.5	0.2	0.2	3.4	1	67.3	9.3	0.1	1.5	1.1	0.6	0.2	4.8	1.9	2.1	6.6	5.2	0.5	0.1	5.4
82	3.4	7.4	0.1	0.8	0.4	0.1	0.6	2.4	17.5	0.1	2.4	4.1	0.1	1.3	5.8	0.1	1.1	1.2	0.3	55.3	1	3	3.5	2.7	3.7	8.3	3.5	1.2	0.1	2.5	0.9	0.8	0.1	0.1	0.1	1.5	0.2	0.2	3.4	1	67.3	9.3	0.1	1.5	1.1	0.6	0.2	4.8	1.9	2.1	6.6	5.2	0.5	0.1	5.4
83	3.4	7.4	0.1	0.8	0.4	0.1	0.6	2.4	17.5	0.1	2.4	4.1	0.1	1.3	5.8	0.1	1.1	1.2	0.3	55.3	1	3	3.5	2.7	3.7	8.3	3.5	1.2	0.1	2.5	0.9	0.8	0.1	0.1	0.1	1.5	0.2	0.2	3.4	1	67.3	9.3	0.1	1.5	1.1	0.6	0.2	4.8	1.9	2.1	6.6	5.2	0.5	0.1	5.4
84	3.4	7.4	0.1	0.8	0.4	0.1	0.6	2.4	17.5	0.1	2.4	4.1	0.1	1.3	5.8	0.1	1.1	1.2	0.3	55.3	1	3	3.5	2.7	3.7	8.3	3.5	1.2	0.1	2.5	0.9	0.8	0.1	0.1	0.1	1.5	0.2	0.2	3.4	1	67.3	9.3	0.1	1.5	1.1	0.6	0.2	4.8	1.9	2.1	6.6	5.2	0.5	0.1	5.4
85	3.4	7.4	0.1	0.8	0.4	0.1	0.6	2.4	17.5	0.1	2.4	4.1	0.1	1.3	5.8	0.1	1.1	1.2	0.3	55.3	1	3	3.5	2.7	3.7	8.3	3.5	1.2	0.1	2.5	0.9	0.8	0.1	0.1	0.1	1.5	0.2	0.2	3.4	1	67.3	9.3	0.1	1.5	1.1	0.6	0.2	4.8	1.9	2.1	6.6	5.2	0.5	0.1	5.4
86	3.4	7.4	0.1	0.8	0.4	0.1	0.6	2.4	17.5	0.1	2.4	4.1	0.1	1.3	5.8	0.1	1.1	1.2	0.3	55.3	1	3	3.5	2.7	3.7	8.3	3.5	1.2	0.1	2.5	0.9	0.8	0.1	0.1	0.1	1.5	0.2	0.2	3.4	1	67.3	9.3	0.1	1.5	1.1	0.6	0.2	4.8	1.9	2.1	6.6	5.2	0.5	0.1	5.4
87	3.4	7.4	0.1	0.8	0.4	0.1	0.6	2.4	17.5	0.1	2.4	4.1	0.1	1.3	5.8	0.1	1.1	1.2	0.3	55.3	1	3	3.5	2.7	3.7	8.3	3.5	1.2	0.1	2.5	0.9	0.8	0.1	0.1	0.1	1.5	0.2	0.2	3.4	1	67.3	9.3	0.1	1.5	1.1	0.6	0.2	4.8	1.9	2.1	6.6	5.2	0.5	0.1	5.4
88	3.4	7.4	0.1	0.8	0.4	0.1	0.6	2.4	17.5	0.1	2.4	4.1	0.1	1.3	5.8	0.1	1.1	1.2	0.3	55.3	1	3	3.5	2.7	3.7	8.3	3.5	1.2	0.1	2.5	0.9	0.8	0.1	0.1	0.1	1.5	0.2	0.2	3.4	1	67.3	9.3	0.1	1.5	1.1	0.6	0.2	4.8	1.9	2.1	6.6	5.2	0.5	0.1	5.4
89	3.4	7.4	0.1	0.8	0.4	0.1	0.6	2.4	17.5	0.1	2.4	4.1	0.1	1.3	5.8	0.1	1.1	1.2	0.3	55.3	1	3	3.5	2.7	3.7	8.3	3.5	1.2	0.1	2.5	0.9	0.8	0.1	0.1	0.1	1.5	0.2	0.2	3.4	1	67.3	9.3	0.1	1.5	1.1	0.6	0.2	4.8	1.9	2.1	6.6	5.2	0.5	0.1	5.4
90	3.4	7.4	0.1	0.8	0.4	0.1	0.6	2.4	17.5	0.1	2.4	4.1	0.1	1.3	5.8	0.1	1.1	1.2	0.3	55.3	1	3	3.5	2.7	3.7	8.3	3.5	1.2	0.1	2.5	0.9	0.8	0.1	0.1	0.1	1.5	0.2	0.2	3.4	1	67.3	9.3	0.1	1.5	1.1	0.6	0.2	4.8	1.9	2.1	6.6	5.2	0.5	0.1	5.4
91	0.026	0.018	0.016	0	0.018	0.053	0.007	0.018	0.004	0.046	0.004	0.336	0.029	0.059	0.073	0.006	0.094	0.163	0.041	0.054	0.026	0.021	0.028	0	0.038	0	0.039	0.021	0.022	0.006	0.001	0.415	0.225	0.033	0.016	0.011	0.028	0.029	0.026	0.026	0.037	0.021	0.036	0.025	0	0.004	0.033	0.069	0.004	0.029	0.002	0.197	0.01	0	0.018
92	0.026	0.018	0.016	0	0.018	0.053	0.007	0.018	0.004	0.046	0.004	0.336	0.029	0.059	0.073	0.006	0.094	0.163	0.041	0.054	0.026	0.021	0.028	0	0.038	0	0.039	0.021	0.022	0.006	0.001	0.415	0.225	0.033	0.016	0.011	0.028	0.029	0.026	0.026	0.037	0.021	0.036	0.025	0	0.004	0.033	0.069	0.004	0.029	0.002	0.197	0.01	0	0.018
93	0.026	0.018	0.016	0	0.018	0.053	0.007	0.018	0.004	0.046	0.004	0.336	0.029	0.059	0.073	0.006	0.094	0.163	0.041	0.054	0.026	0.021	0.028	0	0.038	0	0.039	0.021	0.022	0.006	0.001	0.415	0.225	0.033	0.016	0.011	0.028	0.029	0.026	0.026	0.037	0.021	0.036	0.025	0	0.004	0.033	0.069	0.004	0.029	0.002	0.197	0.01	0	0.018
94	0.026	0.018	0.016	0	0.018	0.053	0.007	0.018	0.004	0.046	0.004	0.336	0.029	0.059	0.073	0.006	0.094	0.163	0.041	0.054	0.026	0.021	0.028	0	0.038	0	0.039	0.021	0.022	0.006	0.001	0.415	0.225	0.033	0.016	0.011	0.028	0.029	0.026	0.026	0.037	0.021	0.036	0.025	0	0.004	0.033	0.069	0.004	0.029	0.002	0.197	0.01	0	0.018
101	-7.5	-7.4	-5.8	-6	-5.9	-6.2	-5.5	-5.9	-7.3	-7.8	-7.4	-3.1	-4.4	-3.2	-3.8	-4.9	-3.8	-4.1	-5.6	-2.7	-4.6	-4.7	-3.3	-3.6	-3.7	-3.6	-3	-3.3	-4.2	-5	-4.9	-4.8	-6.2	-4.3	-4.6	-7	-5.2	-5.6	-7.5	-4.6	-6	-6.3	-4.6	-4.8	-5	-5.1	-4.4	-4.8	-6.2	-6.8	-6.2	-4.6	-6.2	-4.3	-4.6
102	230	44	51	41	29	33	65	67	216	40	81	55	14	54	70	32	92	21	53	20	86	21	39	34	32	20	51	50	67	20	64	174	49	60	53	175	155	49	230	86	167	58	63	46	53	47	93	110	306	68	105	26	11	13	12
103	-1.496	-1.156	-0.577	-2.101	-0.547	1.278	-0.476	-0.808	-1.248	-1.683	-1.53	0.92	8.223	5.256	5.139	1.135	0	3.084	3.368	4.533	7.538	11.998	1.808	0.817	1.91	4.514	1.554	1.786	2	6.663	7.042	-0.865	-0.777	8.76	7.54	-1.557	0	-0.784	-1.496	7.538	-1.461	-1.121	-0.542	-2.066	-0.513	2.952	-0.441	-0.773	-1.214	-1.649	-1.495	-0.525	-0.437	14.958	12.775
104	-0.223	-0.103	0.456	0.382	0.02	-0.014	0.254	0.167	-0.253	-0.505	-0.1	0.063	1.115	-0.759	-0.782	0.259	0.065	-0.235	-0.058	0.567	-0.015	0.347	0.222	0.068	-0.181	0.734	-0.246	-0.17	0.253	-0.822	-0.294	-0.245	-0.061	0.316	0.48	-0.46	-0.004	0.029	-0.223	-0.015	-0.07	-0.253	0.43	-0.17	0.391	0.374	0.39	0.075	-0.454	-0.635	-0.289	-0.364	-0.032	0.097	0.188
105	-1.04	-0.41	0.39	0.25	-0.39	-0.08	-0.13	-0.29	-0.98	-0.89	-0.87	0	0	-0.09	-0.09	-0.17	0.1	0.14	-0.26	1.45	0.1	0.55	0.18	0.51	0.04	0.3	0.18	0.09	-0.08	-0.3	-0.2	-0.57	-0.69	0.5	-0.05	-1.02	-0.08	-0.36	-1.04	0.1	-1.14	-0.31	-0.1	0.09	-0.13	-0.1	-0.07	-0.39	-1.03	-0.97	-0.6	0.05	-1.23	1.07	0.02
106	-0.41	-0.28	0.53	0.25	0.36	0.42	0.21	0.14	-0.25	0.02	0.11	0.14	0.71	-0.76	-0.97	0.44	0.11	0.22	0.36	0.25	0.19	0	-0.33	0.11	-0.38	0.75	-0.13	-0.09	0.44	0.22	-0.21	-0.01	-0.08	0.67	0.43	-0.3	0.23	0.16	-0.41	0.19	-0.27	-0.2	0.3	0.42	0.26	0.35	0.25	0.2	-0.29	-0.09	0.24	0.25	0.19	0.65	0.44
107	-0.1608	-0.0974	0.0425	0.0427	0.0548	0.0338	0.007	0.0158	-0.1095	0.0093	-0.0112	0.0472	0.0114	-0.0931	-0.1536	0.0384	0.0376	0.0264	0.0175	0.1122	0.0509	0.0602	0.0088	0.1598	-0.0489	0.0131	-0.0366	0.0082	0.0331	0.0117	-0.109	-0.0079	0.0747	0.0207	0.0173	-0.0551	0.0932	0.0136	-0.1608	0.0509	-0.098	-0.0624	0.0127	0.0211	0.0215	0.0164	0.0255	0.0169	-0.1274	-0.041	0.054	0.0757	0.1307	0.0611	0.1327
108	-0.5112	-0.2137	0.1485	0.0882	-0.048	-0.0266	0.0332	-0.07	-0.4223	-0.1961	-0.3164	0.0691	-0.033	-0.1154	-0.2403	-0.0416	0.1963	0.039	0.0682	0.1216	0.0543	0.0866	-0.0381	0.1099	-0.0735	0.0839	0.169	0.0467	0.0609	-0.1152	-0.112	-0.1711	-0.1305	0.2673	0.0802	-0.545	0.0782	-0.0169	-0.5112	0.0543	-0.5067	-0.1822	0.089	0.0745	-0.0172	-0.0651	0.0959	-0.0316	-0.4593	-0.2639	-0.2614	-0.0847	-0.0557	-0.0197	0.0637
109	-6.17	-6.33	-2.99	-3.47	-3.22	-3.33	-3.43	-3.74	-5.58	-5.64	-4.63	-1.1	-1.54	-1.32	-1.6	-2.83	-0.84	-1.09	-2.7	0.13	-2.63	-3.11	-0.91	-0.67	-1.02	-0.06	-0.83	-1.02	-1.95	-2.49	-2.01	-3.96	-5.03	-2.59	-2.91	-6.26	-3.43	-3.84	-6.17	-2.63	-5.79	-6.01	-2.99	-3.06	-3.09	-3.15	-3.16	-3.43	-5.38	-5.5	-4.26	-3.99	-5.05	-2.9	-3.19
110	-0.41	-0.28	0.54	0.25	0.36	0.42	0.21	0.13	-0.26	0.01	0.11	0.15	0.71	-0.76	-0.97	0.43	0.11	0.21	0.36	0.25	0.19	0	-0.32	0.11	-0.38	0.75	-0.13	-0.09	0.43	0.22	-0.21	-0.01	-0.08	0.67	0.42	-0.3	0.22	0.16	-0.41	0.19	-0.27	-0.2	0.3	0.42	0.25	0.36	0.24	0.2	-0.3	-0.09	0.24	0.25	0.19	0.65	0.43
111	-7.04	-6.02	-3.24	-3.76	-3.67	-3.63	-3.52	-4.03	-6.05	-5.78	-5.25	-1.31	-1.95	-1.68	-1.8	-3.36	-1.15	-1.35	-3.01	-0.12	-3.37	-2.48	-1.21	-0.97	-1.29	-0.59	-1.05	-1.31	-2.49	-2.69	-2.6	-4.91	-5.83	-3.4	-3.59	-7.28	-4.16	-4.54	-7.04	-3.37	-7.37	-6.41	-3.74	-4.2	-4.04	-4.03	-3.92	-4.34	-6.48	-6.14	-5.67	-3.94	-4.99	-2.57	-2.89
112	-0.36	-0.23	0.55	0.2	0.2	0.35	0.33	0.13	-0.29	-0.1	0.03	0.23	0.59	-0.87	-1.04	0.37	0.01	0.18	0.38	0.37	0.41	0.41	-0.32	0.09	-0.32	0.49	-0.1	-0.05	0.37	0.09	-0.22	-0.08	0	0.7	0.46	-0.26	0.29	0.28	-0.36	0.41	-0.3	-0.23	0.44	0.15	0.22	0.34	0.32	0.21	-0.33	-0.07	0	0	-0.05	0.64	0.27
113	26396	5188	3331	4600	3772	4065	6443	7958	19830	3101	6770	6019	1200	9501	10234	3098	6054	1554	4199	1354	6531	1456	4260	2665	3304	1803	5117	4940	5437	1367	4678	24837	5395	5729	6706	17228	13533	4133	26396	6531	21432	8218	5751	7632	6091	6653	10084	11387	32623	4810	10806	5014	1247	1389	1827
114	-0.81	-0.66	0.14	-0.05	-0.01	0	0.03	-0.15	-0.67	-0.6	-0.49	0.41	0.33	-0.01	-0.06	0.19	0.29	0.38	0.24	0.76	0.22	0.29	0.22	0.47	0.28	0.66	0.36	0.33	0.29	0.09	-0.05	-0.38	-0.65	0.27	0.17	-0.8	-0.16	-0.18	-0.81	0.22	-0.84	-0.7	0.04	-0.12	-0.04	-0.04	-0.02	-0.15	-0.74	-0.62	-0.55	-0.27	-0.61	0.3	0.12
115	-0.21	-0.13	0.32	0.12	0.12	0.2	0.2	0.08	-0.16	-0.05	0.02	0.13	0.35	-0.5	-0.57	0.22	0.01	0.1	0.22	0.16	0.24	0.24	-0.18	0.06	-0.17	0.28	-0.05	-0.02	0.22	0.06	-0.12	-0.04	-0.01	0.4	0.28	-0.15	0.18	0.16	-0.21	0.24	-0.2	-0.13	0.26	0.09	0.13	0.2	0.19	0.12	-0.19	-0.03	0	0	-0.04	0.36	0.16
116	-5.21	-4.95	-3.13	-3.62	-3.3	-3.42	-3.58	-3.87	-5.01	-4.98	-4.51	-2.28	-2.71	-2.22	-2.2	-2.7	-2.08	-2.02	-2.95	-1.14	-2.74	-2.58	-1.95	-1.95	-1.84	-1.47	-2.01	-2.13	-2.69	-2.84	-2.58	-4.06	-4.72	-2.84	-2.85	-5.18	-3.56	-3.69	-5.21	-2.74	-5.03	-4.91	-3.09	-3.55	-3.17	-3.24	-3.47	-3.61	-4.85	-4.87	-4.32	-3.93	-4.72	-2.8	-2.9
117	-6.67	-5.37	-3.42	-3.09	-4.65	-4.29	-3.47	-3.61	-5.31	-5.79	-5.39	-1.91	-2.28	-2.47	-2.83	-2.7	-1.32	-2.14	-2.88	-1.02	-3.24	-2.36	-1.63	-0.5	-2.56	-2.11	-1.91	-1.64	-2.19	-3.12	-2.64	-5.02	-6.13	-4.16	-4.35	-6.65	-3.79	-4.85	-6.67	-3.24	-7.16	-6.24	-3.94	-3.75	-5.36	-5.01	-4.12	-4.28	-6.03	-6.4	-5.67	-4.42	-5.07	-3.69	-3.76
118	-0.5	0	0.16	0.11	0.33	0.37	0.33	0.14	-0.33	-0.26	-0.12	0.06	-0.5	-0.87	-1.11	0.57	-0.17	-0.01	0.32	-0.22	0.52	0.61	-0.44	0.3	0	0.15	-0.5	-0.29	0.4	1	0.14	-0.09	-0.34	0.41	0.34	-0.4	0.33	0.25	-0.5	0.52	-0.42	-0.3	0.23	0.01	0.17	0.21	0.26	0.04	-0.48	-0.31	0.08	-0.27	-0.08	0.09	0.13
119	0.35	0.33	-0.05	-0.43	0.15	0.1	-0.14	-0.05	0.2	0.43	0.34	-0.53	-0.03	0.16	0.16	-0.05	-0.19	-0.02	-0.21	-0.78	-0.07	0.09	-0.17	-0.46	-0.17	-0.01	-0.24	-0.22	-0.2	0.06	0.26	0.01	0.09	-0.21	-0.29	0.41	-0.13	0.13	0.35	-0.07	-0.06	0.44	-0.29	-0.06	0.02	0.07	-0.19	-0.05	0.21	0.25	0.27	0.15	0	0.02	0.1
120	-0.79	-0.6	0.55	0.05	0.14	0.18	0.35	0	-0.68	-0.65	-0.33	0.2	0.35	-0.69	-0.87	0.11	0.12	0.26	0.21	0.38	0.16	0.22	-0.14	0.12	-0.2	0.5	0.1	0	0.16	-0.28	-0.4	-0.37	-0.5	0.62	0.37	-0.78	0.14	0.1	-0.79	0.16	-0.81	-0.68	0.36	-0.08	0.08	0.09	0.26	0	-0.8	-0.7	-0.44	-0.23	-0.49	0.62	0.24
121	-0.58	0.03	0.48	-0.33	0.31	0.31	0.49	-0.29	-0.98	-0.63	-1.34	0.18	0.45	-0.71	-0.34	0.32	0.82	0.79	-0.08	1.01	0.43	0.77	0	0.42	0.86	0.56	-0.04	0.38	0.17	-0.38	-0.2	-0.13	-0.25	0.41	-0.05	-0.89	0.23	-0.17	-0.58	0.43	-1.42	-0.36	1.17	0.08	-0.22	0.28	0.8	0.3	-1.03	-0.44	0.25	-0.82	-1.99	0.08	0.06
122	-1.15	0.07	0.22	0.43	0.66	-0.22	-0.14	-0.18	-1.46	-1.9	-1.42	0.96	-0.38	-0.55	-0.36	-0.01	0.95	-0.01	-0.01	2.28	1.02	1.66	0.38	0.45	-0.04	1.24	0.13	-0.16	0.75	0.33	-1.06	-0.72	-0.24	1.12	0.99	-1.24	-0.2	-0.31	-1.15	1.02	-1.6	-1.39	-0.19	-0.27	0.1	-0.04	1.13	-0.18	-0.77	-0.85	-0.53	-0.85	-1.3	0.42	0

Table S12 Amino acid descriptors (2D) used in this study.

	I	I	I	I	I	I	I	I	I	I	I	K	K	K	K	K	K	K	K	K	K	K	K	K	K	K	K	K
	L	M	N	P	Q	R	S	T	V	W	Y	A	C	D	E	F	G	H	I	K	L	M	N	P	Q	R	S	T
No.	IL	IM	IN	IP	IQ	IR	IS	IT	IV	IW	IY	KA	KC	KD	KE	KF	KG	KH	KI	KK	KL	KM	KN	KP	KQ	KR	KS	KT
123	-1.4	-1.4	-0.1	-0.1	-0.4	-0.7	-0.1	-0.6	-1.2	-1.8	-1.4	1	0.5	-1	-1.1	-0.4	0	0	0	0.7	0.1	-0.1	-0.3	0.6	-0.4	0.1	0.1	0
124	-4.6	-4.7	-3.2	-3	-3.2	-3.3	-3	-3.8	-5	-4.7	-4.4	-1.9	-2.4	-3.5	-3.2	-2.8	0	-2.2	-2.9	-1.7	-2.5	-2.9	-3	-1.8	-2.7	-2	-2.3	-2.8
125	-0.6	-0.7	0.7	0.3	0.3	0.4	0.4	0	-0.7	-0.9	-0.5	1	1.6	-0.6	-0.7	0.3	1.2	0.3	0.8	1.9	1.1	0.5	0.1	1.1	0.4	1.7	0.5	0.6
126	-0.1	0	0.4	0.2	0.2	0.3	0.4	0.1	-0.1	0.1	0.1	0.3	0.4	-0.9	-0.8	0.1	0	0	0.2	0.1	0.3	0.2	-0.5	0.1	-0.3	0.3	-0.3	-0.1
127	-1.3	-1	0.5	0	0.1	-0.2	0.3	-0.3	-1.2	-1.3	-1	1.3	1.3	0.2	0	0.4	0.9	1	0.5	2.1	0.5	0.6	0.7	0.9	0.5	1.1	0.9	0.8
128	-1.2	-0.8	0.4	0.1	0	-0.2	0.4	-0.2	-1	-1.1	-0.8	1	0.9	-0.2	-0.4	0.3	0.7	0.6	0.4	1.6	0.3	0.3	0.3	0.8	0.1	0.6	0.7	0.5
129	-1.2	-0.9	0.2	0	-0.1	-0.2	0.3	-0.2	-1	-1.2	-0.9	0.8	-0.3	-0.2	-0.4	0.1	0.6	0.1	0.3	0.6	0.3	-0.1	0.2	0.5	0	0.4	0.5	0.4
130	-0.4	-0.3	0.7	0.4	0.3	-0.4	0.4	0.3	-0.5	-1.2	-1.1	1	0.4	0.9	1.4	-0.2	1	0.3	0.7	2.5	0.6	0.7	1.4	1.3	1.5	1	1.3	1.2
131	-0.3	-0.2	0.7	0.4	0.7	-0.2	0.4	0.4	-0.2	-1	-0.8	0.8	0.8	0.1	0.2	-0.1	0.7	0	1	1.6	0.8	1	0.6	1.1	0.8	0.2	0.7	0.8
132	-1	-0.8	0.2	0.3	0	-0.6	0	-0.3	-0.9	-1.2	-1.2	0.4	0.5	-0.8	-1	-0.2	0.4	-0.6	0.2	0.3	0.3	0.3	-0.2	0.6	-0.3	-0.4	-0.1	-0.2
133	5.6	5.7	5.3	5.7	5.4	5.9	5.3	5.6	5.6	6.2	6	5.2	5.3	5.3	5.3	5.5	5.9	5.2	5.8	5.7	5.4	5.8	5.3	5.9	5.7	6.5	4.9	5.3
134	6.1	5.9	5.7	5.9	5.7	6.1	5.4	5.8	6	6.3	6.2	5.2	6.2	5.6	5.8	5.5	4.7	5.6	5.9	6.1	5.8	6.5	5.7	5.9	5.8	6.3	5.5	5.7
135	6.3	6.1	5.3	5.9	6	6.2	5.8	6.1	6.1	6.6	6.2	5.4	6.5	5.8	6.3	5.8	4.9	5.9	5.9	5.5	6	5.8	5.9	5.4	5.8	5.3	5.4	5.6
136	-0.0008	-0.0093	0.0023	0.0044	-0.0019	0.001	0.0015	-0.0042	0.0026	0.0027	-0.0048	0.0018	-0.006	-0.0006	-0.0096	-0.0045	-0.0013	0.0029	0.0009	0.0051	0.0021	-0.0047	0.0062	0.0099	0.0023	0.0073	0.005	-0.0059
137	-0.1261	-0.0879	0.3828	0.5603	0.2056	0.3358	0.313	0.2867	-0.1963	-0.1598	-0.1696	0.3989	0.7318	-0.9001	-0.9236	0.0483	0.005	0.0036	0.217	0.5641	0.2129	0.1536	-0.0704	0.0408	-0.248	0.5516	0.0272	-0.1322
138	-0.3571	-0.1536	0.4908	0.1967	0.3146	0.3477	0.352	0.1143	-0.2748	0.134	0.0824	0.1468	0.3018	-0.8377	-0.849	0.3116	-0.1311	0.1505	0.375	0.0691	0.4252	0.3278	-0.2891	-0.0142	-0.2164	0.0572	-0.131	-0.13
139	-0.0864	-0.0063	0.1695	0.1293	0.1356	0.1911	0.0421	0.0042	-0.1361	0.0343	0.0214	0.0617	0.104	-0.2813	-0.3221	0.1406	0.0022	0.054	0.1699	-0.1951	0.1076	0.1502	-0.1759	-0.1263	-0.1615	-0.0335	-0.0377	-0.0157
140	-0.0102	-0.0436	0.0252	0.0791	0.0416	0.0613	0.0059	-0.0215	-0.0423	0.0904	0.072	0.0222	0.0881	-0.0562	-0.0928	0.0152	0.0607	0.0743	4E-05	-0.1234	-0.0186	0.036	-0.0469	-0.0678	0.0131	-0.0384	0.0541	0.0229
141	-0.019	-0.0092	0.0134	0.0119	0.012	0.014	0.0074	0.0023	-0.0216	0.0023	0.0004	0.0037	0.0187	-0.0164	-0.0179	0.0105	-7E-05	0.0029	0.0131	-0.0099	0.01	0.0085	-0.0082	-0.0058	-0.0077	-0.0042	-0.0015	-0.0013
142	-2.93	-2.44	-0.84	-1.14	-1	-0.77	-1.35	-1.52	-2.66	-2.4	-2.01	0.08	-0.46	0.09	0.09	-0.21	-0.16	0.4	-0.56	1.52	-0.2	0.2	0.32	0.64	0.53	1.21	0.37	0.09
143	-3.7	-3.1	-1.41	-1.6	-1.45	-1.41	-1.6	-2	-3.5	-2.9	-2.9	-0.7	-0.74	-1	-1.06	-0.92	-0.83	-0.61	-1.3	0.47	-1.2	-1.17	-0.35	-0.41	-0.42	0.28	-0.83	-1.12
144	-1.15	-0.52	0.4	0.14	0.24	0.05	0.09	-0.28	-0.9	-1.28	-0.74	1.06	0.76	0.58	0.93	0.74	1.08	1.23	0.48	2.23	0.78	1.15	1.11	1.59	1.29	1.65	1.24	1.1
145	-2.53	-2.27	-0.33	-0.2	0.11	-0.01	-0.48	-1.14	-2.46	-1.58	-1.66	1.91	0.19	1.13	1.31	-0.42	1.58	2.26	0.75	3.51	0.67	1.27	2.43	1.94	2.58	3.68	2.28	1.38
146	-1.67	-1.32	0.26	0.05	0.05	-0.04	-0.11	-0.2	-1.25	-1.58	-1.29	1.16	0.8	0.95	1.12	0.58	1.04	1.31	0.87	2.95	0.72	1.02	1.48	1.68	1.5	2.37	1.37	1.42
147	-1.84	-1	-0.31	-1.26	-0.28	-0.3	-0.22	-0.3	-1.64	-1.13	-1.26	0.62	0.27	0.59	0.67	0.71	0.78	1.46	0.84	2.69	0.47	1.55	0.87	1.08	0.98	1.58	0.94	1.11
148	41.729	41.535	36.777	37.302	39.871	46.363	33.25	36.344	38.635	50.962	46.477	34.882	37.782	37.579	40.673	48.109	31.788	43.695	44.163	46.596	44.163	43.969	39.211	39.735	42.304	48.796	35.684	38.777
149	1	1	1	1	1	1	1	1	1	0	1	1	1	1	1	1	1	1	1	1	1	1	1	1	1	0	1	1
150	0	0	0	0	0	0	0	0	0	0	0	0	0	0	0	0	0	0	0	0	0	0	0	0	0	0	0	0
151	0	0	0	0	0	0	0	0	0	1	0	0	0	0	0	0	0	0	0	0	0	0	0	0	0	1	0	0
152	2	2	2	1	2	2	2	2	2	3	2	2	2	2	2	2	2	2	2	2	2	2	2	2	2	3	2	2
153	1	1	2	1	2	1	2	2	1	1	2	1	1	1	1	1	1	3	1	1	1	1	2	1	2	1	2	2
154	2	2	2	2	2	2	2	2	2	2	2	2	2	4	4	2	2	2	2	2	2	2	2	2	2	2	2	2
155	0	0	0	0	0	0	0	0	0	9	6	0	0	0	0	6	0	5	0	0	0	0	0	0	0	0	0	0
156	1	1	1	1	1	4	1	1	1	1	1	2	2	2	2	2	2	2	2	3	2	2	2	2	2	5	2	2
157	41	39	36	36	39	46	33	36	38	46	43	35	36	37	40	45	32	42	44	47	44	42	39	39	42	49	36	39
158	1	1	2	0	2	1	2	2	1	2	2	1	1	1	1	1	1	3	1	1	1	1	2	0	2	1	2	2
159	2	2	4	1	4	2	4	4	2	3	4	2	2	2	2	2	2	6	2	2	2	2	4	1	4	2	4	4
160	17	17	17	16	18	20	15	16	16	23	21	15	16	18	19	21	14	20	18	19	18	18	18	17	19	21	16	17
161	10	10	7	9	8	9	7	8	9	15	13	7	7	7	8	13	6	9	10	10	10	10	7	9	8	9	7	8
162	59.846	63.215	59.433	54.87	63.083	72.488	52.877	56.459	56.505	71.045	66.619	55.047	61.64	62.455	66.22	68.981	51.432	68.986	65.474	70.685	65.474	68.875	64.673	60.46	68.382	77.231	58.395	62.049
163	1.4597	1.6209	1.6509	1.5242	1.6175	1.5758	1.6023	1.5683	1.487	1.5445	1.5493	1.5728	1.7122	1.688	1.6555	1.5329	1.6072	1.6425	1.4881	1.5039	1.4881	1.6399	1.6583	1.5502	1.6281	1.5761	1.6221	1.591
164	12	11	10	11	11	12	9	10	11	17	15	9	9	10	11	15	8	12	12	12	12	11	10	11	11	12	9	10
165	24	22	19	20	21	26	18	20	22	23	22	20	20	19	21	24	18	22	26	28	26	24	21	22	23	28	20	22
166	2	2	3	2	3	5	2	2	2	3	2	3	3	3	3	3	3	5	3	4	3	3	4	3	4	6	3	3
167	3	3	4	3	4	3	4	4	3	3	4	3	3	5	5	3	3	3	3	3	3	3	4	3	4	3	4	4
168	0	1	0	0	0	0	0	0	0	0	0	0	1	0	0	0	0	0	0	0	0	1	0	0	0	0	0	0
169	4.3474	4.2437	4.3507	2.6203	4.3147	4.1955	4.1604	4.351	4.351	1.9136	2.499	3.734	3.8875	4.1093	4.1088	2.3758	3.498	2.3513	4.1933	4.0333	4.1068	4.0224	4.1093	2.4189	4.1088	4.0523	3.8875	4.0755
170	-2.6962	-2.6777	-2.6731	-2.6758	-2.6768	-2.6816	-2.6728	-2.6764	-2.6879	-2.6738	-2.6741	-2.6553	-2.6553	-2.6557	-2.6598	-2.6562	-2.6518	-2.6555	-2.6932	-2.6764	-2.6867	-2.6601	-2.6552	-2.6574	-2.6591	-2.6654	-2.655	-2.6587
171	-0.4614	-0.4336	-0.5095	-0.4638	-0.5137	-0.4975	-0.5074	-0.4632	-0.4374	-0.5413	-0.5477	-0.5075	-0.5341	-0.5477	-0.5477	-0.5494	-0.511	-0.5488	-0.459	-0.4998	-0.4917	-0.4633	-0.53	-0.5031	-0.5384	-0.5366	-0.5308	-0.4854
172	0.3723	0.359	0.4089	0.3978	0.4121	0.3858	0.4345	0.4216	0.3714	0.5552	0.5333	0.3757	0.4394	0.3903	0.3904	0.5568	0.3797	0.446	0.3742	0.3754	0.3754	0.3605	0.4123	0.4061	0.4152	0.3904	0.4414	0.4265
173	2.7121	2.7088	2.7083	2.7514	2.7095	2.7108	2.7074	2.7103	2.7119	2.7073	2.7072	2.6889	2.6897	2.69	2.6918	2.6901	2.6861	2.6904	2.6996	2.696	2.696	2.6918	2.6912	2.7402	2.6927	2.6944	2.6902	2.6934
174	-3.3149	-3.3149	-3.3149	-3.3129	-3.3149	-3.3149	-3.315	-3.3151	-3.315	-3.315	-3.315	-3.3109	-3.311	-3.311	-3.311	-3.311	-3.3107	-3.311	-3.311	-3.311	-3.311	-3.311	-3.311	-3.3092	-3.311	-3.311	-3.3111	-3.3111
175	-0.389	-0.3771	-0.4563	-0.4202	-0.4561	-0.4039	-0.4611	-0.4528	-0.3801	-0.436	-0.5381	-0.4261	-0.5177	-0.4607	-0.4526	-0.4805	-0.4494	-0.4889	-0.4135	-0.4236	-0.4224	-0.3964	-0.4917	-0.4635	-0.4887	-0.4511	-0.5189	-0.4939
176	0.2959	0.3571	0.3548	0.2927	0.3555	0.3367	0.3821	0.3695	0.2958	0.6569	0.4803	0.2968	0.3845	0.3381	0.3381	0.547	0.2973	0.4383	0.2967	0.2968	0.2968	0.358	0.3557	0.2946	0.3564	0.3381	0.3832	0.3706
177	2.6934	2.678	2.6776	2.7042	2.6781	2.6785	2.6774	2.6776	2.679	2.6776	2.6776	2.6593	2.6592	2.6593	2.6599	2.6593	2.659	2.6593	2.6858	2.6704	2.6908	2.6598	2.6593	2.6964	2.6599	2.6604	2.6591	2.6593
178	-2.4736	-2.4684	-2.4673	-2.469	-2.4687	-2.4703	-2.4672	-2.4689	-2.4721	-2.4673	-2.4673	-2.4575	-2.4573	-2.4576	-2.4588	-2.4578	-2.456	-2.4577	-2.4663	-2.4639	-2.4637	-2.4587	-2.4577	-2.459	-2.4589	-2.4604	-2.4577	-2.4591
179	-0.1714	-0.0857	-0.286	-0.1099	-0.2895	-0.2572	-0.301	-0.2808	-0.1702	-0.3955	-0.3958	-0.1765	-0.3095	-0.2642	-0.2642	-0.4023	-0.1812	-0.3205	-0.1742	-0.1761	-0.176	-0.0881	-0.2914	-0.122	-0.2943	-0.2642	-0.314	-0.2875
180	0.3535	0.4619	0.5217	0.2686	0.5292	0.4774	0.534	0.5139	0.352	0.7052	0.657	0.3593	0.5519	0.4853	0.4854	0.7006	0.3668	0.5864	0.3566	0.3588	0.3587	0.4646	0.5297	0.2863	0.536	0.4854	0.5503	0.5228
181	2.9262	2.9182	2.9121	2.9899	2.916	2.9197	2.9097	2.9151	2.9235	2.9119	2.9115	2.8857	2.8899	2.8886	2.894	2.8891	2.8799	2.8886	2.9201	2.9055	2.9112	2.8986	2.8898	2.9826	2.8952	2.9011	2.8868	2.8937
182	28.451	28.545	23.283	25.398	25.469	29.977	21.892	24.078	26.265	28.018	26.265	22.758	25.038	23.581	25.767	27.131	20.572	26.925	29.317	30.184	29.317	29.411	24.149	26.265	26.336	30.844	22.758	24.945
183	7	8	7	5	8	9	6	6	6	7	7	7	8	9	10	9	7	9	9	11	9	10	9	7	10	11	8	8
184	0.4375	0.5	0.4375	0.3125	0.4706	0.4737	0.4286	0.4	0.4	0.2917	0.3333	0.5	0.5333	0.5294	0.5556	0.4286	0.5385	0.45	0.5294	0.6111	0.5294	0.5882	0.5294	0.4118	0.5556	0.55	0.5333	0.5
185	0	0	0	0	0	0	0	0	0	10	6	0	0	0	0	6	0	5	0	0	0	0	0	0	0	0	0	0
186	40	38	35	36	38	45	32	35	37	47	43	34	35	36	39	45	31	42	43	46	43	41	38	39	41	48	35	38
187	2	2	3	2	3	3	2	2	2	2	2	2	2	3	3	2	2	2	2	2	2	2	3	2	3	3	2	2
188	16	16	16	16	17	19	14	15	15	24	21	14	15	17	18	21	13	20	17	18	17	17	17	17	18	20	15	16
189	6	7	6	6	6	7	6	6	6	6	6	8	8	8	8	8	8	8	8	8	8	8	8	8	8	8	8	8
190	8	9	8	5	9	11	7	7	7	8	8	8	9	10	11	10	8	10	10	12	10	11	10	7	11	13	9	9
191	0.5	0.5625	0.5	0.3125	0.5294	0.5789	0.5	0.4667	0.4667	0.3333	0.381	0.5714	0.6	0.5882	0.6111	0.4762	0.6154	0.5	0.5882	0.6667	0.5882	0.6471	0.5882	0.4118	0.6111	0.65	0.6	0.5625
192	38	36	32	34	35	42	30	33	35	35	35	32	33	33	36	37	29	35	41	44	41	39	35	37	38	45	33	36
193	13.585	13.422	13.585	12.293	14.293	15.707	12.008	12.878	12.878	16.983	15.991	11.845	12.552	14.129	14.836	15.665	10.975	14.958	14.129	14.673	14.129	13.966	14.129	12.836	14.836	16.251	12.552	13.422
194	11.155	11.51	10.064	9.9395	10.771	12.147	9.0253	9.8956	10.448	13.465	12.335	9.1221	10.171	10.146	10.853	12.216	8.2518	11.431	11.407	11.658	11.407	11.761	10.315	10.191	11.022	12.398	9.2764	10.147
195	8.7236	7.8534	6.6463	7.5605	7.3534	8.0605	6.1463	7.0165	8.0165	10.533	9.4557	5.9831	5.6902	6.1902	6.8973	9.077	5.1129	7.3449	8.2676	7.8116	8.2676	7.3973	6.1902	7.1044	6.8973	7.6044	5.6902	6.5605
196	8.8783	8.0081	6.8783	7.7152	7.5854	8.2925	6.301	7.1712	8.1712	11.569	10.284	6.1378	5.8449	6.4223	7.1294	9.9578	5.2676	7.8365	8.4223	7.9663	8.4223	7.552	6.4223	7.2591	7.1294	7.8365	5.8449	6.7152
197	7.7898	7.934	7.7898	7.4853	8.2898	9.2898	6.934	7.3066	7.3066	10.935	9.8454	6.9853	7.5233	8.3791	8.8791	10.041	6.5746	9.5409	8.434	9.0233	8.3791	8.5233	8.3791	8.0746	8.8791	9.8791	7.5233	7.896
198	6.1339	7.1617	5.4173	5.8303	5.9173	6.7518	4.8872	5.3316	5.6507	7.8292	6.9695	5.1222	5.9018	5.7149	6.2149	7.2174	4.6747	6.6545	6.5709	6.9531	6.5161	7.5439	5.7995	6.2125	6.2995	7.134	5.2694	5.7138
199	4.5746	3.5116	3.3652	4.0116	3.8652	4.0116	3.0116	3.5141	4.0914	5.7312	5.1866	3.0629	2.8938	3.2474	3.7474	5.1581	2.5505	3.5361	4.5116	4.3938	4.4568	3.3938	3.2474	3.8938	3.7474	3.8938	2.8938	3.3963
200	4.6639	3.601	3.5092	4.101	4.0092	4.101	3.101	3.6034	4.1807	6.9006	6.1422	3.1523	2.9832	3.3914	3.8914	6.2079	2.6498	3.7997	4.601	4.4832	4.5461	3.4832	3.3914	3.9832	3.8914	3.9832	2.9832	3.4856
201	3	3	3	3	3	3	3	4	3	3	3	2	2	2	2	2	1	2	3	2	2	2	2	2	2	2	2	3
202	3	3	3	3	3	3	3	4	3	3	3	2	2	2	2	2	1	2	3	2	2	2	2	2	2	2	2	3
203	0.7014	0.7576	0.7778	0.7325	0.7631	0.7374	0.7696	0.7541	0.7109	0.7289	0.7385	0.7433	0.7948	0.808	0.7915	0.7204	0.7587	0.7626	0.7096	0.717	0.7096	0.763	0.7825	0.7398	0.7683	0.7431	0.7753	0.7603
204	9	10	9	8	10	12	8	8	8	12	12	10	10	11	12	13	10	12	11	13	11	12	11	10	12	14	10	10
205	0	0	0	0	0	1	0	0	0	0	0	1	1	0	0	1	1	1	1	2	1	1	1	1	1	2	1	1

	K	K	K	L	L	L	L	L	L	L	L	L	L	L	L	L	L	L	L	L	L	L	L	M	M	M	M
	V	W	Y	A	C	D	E	F	G	H	I	K	L	M	N	P	Q	R	S	T	V	W	Y	A	C	D	E
No.	KV	KW	KY	LA	LC	LD	LE	LF	LG	LH	LI	LK	LL	LM	LN	LP	LQ	LR	LS	LT	LV	LW	LY	MA	MC	MD	ME
123	0.1	-0.8	-1	-0.4	-0.8	0	-0.1	-1.6	0	-0.7	-1.4	0.1	-1.4	-1.3	-0.1	-0.1	-0.6	-0.6	0	-0.3	-1.2	-1.7	-1.4	-0.5	-0.8	0.1	-0.3
124	-3.1	-3.3	-3.5	-3.7	-4	-2.7	-2.4	-4.2	0	-3.1	-4.6	-2.5	-4.3	-4.4	-3	-2.8	-3.1	-3	-2.6	-3.3	-4.7	-4.4	-4.1	-3.8	-4.2	-2.7	-2.7
125	0.9	0.1	-0.2	-0.4	-0.5	0.8	0.7	-0.9	0.5	-0.3	-0.6	1.1	-0.6	-0.6	0.5	0.5	0.4	0.2	0.4	0.3	-0.6	-0.8	-0.1	0.1	-1.8	1	0
126	0.4	-0.1	-0.3	-0.1	0.1	0.6	0.5	-0.1	0	0.4	-0.1	0.3	-0.2	0	0.3	0.1	0.3	0.3	0.4	0.2	-0.1	0.1	0	0	0.1	0.5	0.3
127	0.7	-0.1	-0.2	-0.3	-0.4	0.7	0.6	-1.3	0.4	-0.1	-1.3	0.5	-1.2	-1	0.4	0	0.2	-0.1	0.4	0	-1.2	-1.4	-0.9	-0.3	-0.3	0.6	0.4
128	0.5	-0.1	-0.2	-0.1	-0.5	0.6	0.4	-1.1	0.7	0	-1.2	0.3	-1.1	-1	0.3	0.1	0	-0.2	0.4	0	-0.9	-1.1	-0.9	-0.2	-0.5	0.4	0.4
129	0.4	-0.5	-0.3	-0.1	-0.6	0.5	0.4	-1.1	0.6	-0.2	-1.2	0.3	-1.1	-1	0.3	0.1	0	-0.2	0.4	0	-0.9	-1.2	-0.9	-0.3	-1.3	0	0
130	0.7	0	0	-0.4	-0.4	1	0.7	-1.4	0.3	-0.6	-0.4	0.6	-0.4	-0.3	0.8	0.4	0.3	-0.4	0.3	0.3	-0.4	-1.1	-1.1	-0.2	-0.4	1.1	1.1
131	1	-0.2	-0.3	-0.2	-0.4	0.8	0.8	-1.2	0.3	-0.5	-0.3	0.8	-0.5	-0.3	0.6	0.4	0.4	-0.4	0.4	0.4	-0.4	-1	-0.9	0	-0.3	0.9	0.9
132	0.1	-0.5	-0.8	-0.4	-0.9	0.5	0.2	-1.4	0.2	-0.8	-1	0.3	-1.2	-0.9	0.2	0.4	-0.2	-0.6	0	-0.3	-1	-1.1	-1.1	-0.3	-0.7	0.4	0.3
133	5.7	5.6	5.6	5.2	5.5	5.5	5.8	5.8	5.9	5.7	5.6	5.4	5.7	5.4	5.7	5.6	5.8	5.8	5.2	5.6	5.6	6.3	5.8	5	5.3	6.1	5.9
134	5.9	5.7	5.7	5.5	5.7	5.6	5.5	6.2	4.9	5.8	6.1	5.8	6.2	6.2	5.9	5.8	5.9	6.1	5.6	5.8	6	6.3	6.1	5.3	5.8	5.9	5.7
135	6.3	5	5.8	5.4	5.7	5.6	5.7	6.3	5.7	5.8	6.3	6	6.2	6.3	5.8	5.9	5.6	6	5.5	5.9	6.1	6.6	6	5.3	5.3	5.5	6.2
136	-0.007	-0.0017	0.008	0.0009	-0.0002	0.0006	-0.0005	-0.0021	-0.0015	-0.0045	-0.0008	0.0021	-0.0007	-0.0025	-0.0006	-0.005	-0.0042	-0.0013	0.001	0.0038	-0.0019	0.0107	0.0035	-0.0015	0.0143	0.0015	0.0032
137	0.2378	-0.2459	-0.169	-0.1503	0.1075	0.382	0.1989	-0.2268	0.3062	0.1144	-0.1261	0.2129	-0.1998	-0.1286	0.3536	0.3522	0.0752	0.1978	0.2714	0.3101	-0.1064	-0.1684	-0.2061	0.0352	0.0446	0.3232	0.1516
138	0.3311	0.1432	-0.0311	0.0187	0.0331	0.6711	0.4759	-0.1269	0.332	0.314	-0.3571	0.4252	-0.3659	-0.141	0.4995	0.2	0.3222	0.3112	0.3421	0.1572	-0.2794	0.1356	0.0359	-0.0863	-0.0316	0.345	0.2154
139	0.1222	0.147	0.0645	-0.0899	-0.0073	0.1488	0.1492	-0.1181	0.0138	0.1227	-0.0864	0.1076	-0.0603	0.0141	0.1282	0.0747	0.1292	0.1851	0.0129	0.0107	-0.067	-0.0601	0.0068	-0.0322	-0.1068	0.0663	0.0714
140	0.0195	0.0335	0.0019	-0.0285	0.0056	0.0075	-0.0286	-0.0042	0.0372	-0.0018	-0.0102	-0.0186	-0.0141	0.0155	0.0228	0.0466	-0.0052	0.0187	-0.0014	0.0053	-0.0072	0.0541	0.0329	0.0046	0.0033	0.025	0.0085
141	0.0116	0.0062	0.0036	-0.0048	-0.0065	0.0123	0.0104	-0.013	0.0066	0.0068	-0.019	0.01	-0.017	-0.0051	0.0111	0.0083	0.0087	0.0105	0.0057	0.0038	-0.0143	-0.0015	-0.002	-0.0018	-0.0092	0.0074	0.0062
142	-0.37	-0.55	-0.41	-1.89	-2.74	-0.31	-0.22	-2.58	-1.87	-0.79	-2.93	-0.2	-2.69	-2.37	-0.63	-0.69	-0.64	-0.63	-1.24	-1.48	-2.57	-2.27	-1.93	-1.28	-2.17	0.14	0.27
143	-1.21	-1.55	-1.42	-3.29	-3.63	-1.26	-1.23	-3.5	-2.98	-1.86	-3.7	-1.2	-3.5	-3.1	-1.45	-1.9	-1.38	-1.18	-1.8	-1.8	-3.5	-2.8	-2.7	-2.63	-3.48	-1.04	-0.64
144	0.81	0.23	0.62	-0.25	-1.25	0.57	0.82	-1.05	0.13	-0.03	-1.15	0.78	-1.04	-0.68	0.37	0.1	0.38	0.26	0.01	-0.05	-0.93	-0.98	-0.56	0.29	-0.74	0.75	1.08
145	0.37	0.61	1.4	-1.64	-2.32	0.79	0.31	-2.44	-1.36	-0.58	-2.33	0.34	-2.44	-2.22	0.2	0.47	0	0.01	-0.67	-1	-2.38	-2.13	-1.38	-0.9	-1.22	1.41	1.25
146	1.14	0.18	0.83	-0.78	-1.77	0.65	0.38	-1.5	-0.81	-0.48	-1.66	0.29	-1.62	-1.04	0.12	0.15	-0.05	-0.09	-0.24	-0.26	-1.5	-1.16	-1.15	-0.22	-1.02	0.75	0.82
147	0.52	0.52	0.6	-1.63	-1.71	0.27	0.5	-1.55	-1.4	0.03	-1.79	0.34	-1.7	-1.12	-0.08	-0.91	-0.11	0.13	-0.29	-0.24	-1.59	-1.01	-1.3	-1.03	-1.24	0.6	1.23
148	41.069	53.396	48.911	32.448	35.348	35.145	38.239	45.675	29.355	41.262	41.729	44.163	41.729	41.535	36.777	37.302	39.871	46.363	33.25	36.344	38.635	50.962	46.477	32.255	35.155	34.952	38.045
149	1	0	0	1	1	1	1	1	1	1	1	1	1	1	1	1	1	1	1	1	1	0	1	1	1	1	1
150	0	0	0	0	0	0	0	0	0	0	0	0	0	0	0	0	0	0	0	0	0	0	0	0	0	0	0
151	0	1	1	0	0	0	0	0	0	0	0	0	0	0	0	0	0	0	0	0	0	1	0	0	0	0	0
152	2	3	3	2	2	2	2	2	2	2	2	2	2	2	2	1	2	2	2	2	2	3	2	2	2	2	2
153	1	1	2	1	1	1	1	1	1	3	1	1	1	1	2	1	2	1	2	2	1	1	2	1	1	1	1
154	2	2	2	2	2	4	4	2	2	2	2	2	2	2	2	2	2	2	2	2	2	2	2	2	2	4	4
155	0	9	6	0	0	0	0	6	0	5	0	0	0	0	0	0	0	0	0	0	0	9	6	0	0	0	0
156	2	2	2	1	1	1	1	1	1	1	1	2	1	1	1	1	1	4	1	1	1	1	1	1	1	1	1
157	41	49	46	32	33	34	37	42	29	39	41	44	41	39	36	36	39	46	33	36	38	46	43	30	31	32	35
158	1	2	2	1	1	1	1	1	1	3	1	1	1	1	2	0	2	1	2	2	1	2	2	1	1	1	1
159	2	3	4	2	2	2	2	2	2	6	2	2	2	2	4	1	4	2	4	4	2	3	4	2	2	2	2
160	17	24	22	14	15	17	18	20	13	19	17	18	17	17	17	16	18	20	15	16	16	23	21	14	15	17	18
161	9	15	13	7	7	7	8	13	6	9	10	10	10	10	7	9	8	9	7	8	9	15	13	7	7	7	8
162	62.057	76.783	72.687	49.657	56.122	56.658	60.376	63.012	46.127	63.918	59.846	65.474	59.846	63.215	59.433	54.87	63.083	72.488	52.877	56.459	56.505	71.045	66.619	52.452	56.825	59.258	63.209
163	1.5136	1.567	1.5801	1.5518	1.7007	1.6664	1.6318	1.5003	1.5906	1.6389	1.4597	1.4881	1.4597	1.6209	1.6509	1.5242	1.6175	1.5758	1.6023	1.5683	1.487	1.5445	1.5493	1.7484	1.8331	1.8518	1.806
164	11	17	15	9	9	10	11	15	8	12	12	12	12	11	10	11	11	12	9	10	11	17	15	8	8	9	10
165	24	25	24	18	18	17	19	22	16	20	24	26	24	22	19	20	21	26	18	20	22	23	22	16	16	15	17
166	3	4	3	2	2	2	2	2	2	4	2	3	2	2	3	2	3	5	2	2	2	3	2	2	2	2	2
167	3	3	4	3	3	5	5	3	3	3	3	3	3	3	4	3	4	3	4	4	3	3	4	3	3	5	5
168	0	0	0	0	1	0	0	0	0	0	0	0	0	1	0	0	0	0	0	0	0	0	0	1	2	1	1
169	4.0755	1.8303	2.3927	3.8933	4.0457	4.2519	4.227	2.4497	3.6383	2.4318	4.3513	4.139	4.2487	4.1489	4.2519	2.5512	4.227	4.1271	4.0457	4.2406	4.2406	1.8876	2.4601	3.7722	3.9325	4.1551	4.1412
170	-2.6735	-2.6559	-2.6562	-2.6681	-2.6682	-2.6686	-2.672	-2.669	-2.6648	-2.6683	-2.6981	-2.6838	-2.6921	-2.6723	-2.6681	-2.6704	-2.6715	-2.6762	-2.6678	-2.6711	-2.6826	-2.6687	-2.669	-2.5946	-2.5953	-2.5972	-2.6163
171	-0.4633	-0.5477	-0.5477	-0.5029	-0.5291	-0.5477	-0.5477	-0.5443	-0.5102	-0.5426	-0.4561	-0.4932	-0.4867	-0.4592	-0.5262	-0.4981	-0.533	-0.5276	-0.5261	-0.4832	-0.4601	-0.5467	-0.5477	-0.4676	-0.5284	-0.5456	-0.5447
172	0.3743	0.5631	0.5456	0.3756	0.4389	0.3901	0.3902	0.5558	0.3795	0.4454	0.3741	0.3753	0.3753	0.3604	0.4121	0.4052	0.4151	0.3902	0.4408	0.4262	0.3742	0.5619	0.544	0.3606	0.3906	0.3754	0.3755
173	2.6955	2.6902	2.6901	2.6881	2.689	2.6893	2.6913	2.6894	2.6849	2.6897	2.6999	2.696	2.696	2.6914	2.6907	2.7417	2.6923	2.6942	2.6895	2.6931	2.6954	2.6895	2.6894	2.6458	2.6475	2.6483	2.6532
174	-3.311	-3.311	-3.311	-3.3092	-3.3093	-3.3092	-3.3092	-3.3093	-3.309	-3.3093	-3.3093	-3.3093	-3.3092	-3.3092	-3.3092	-3.3072	-3.3092	-3.3092	-3.3094	-3.3094	-3.3093	-3.3093	-3.3093	-3.3092	-3.3093	-3.3092	-3.3092
175	-0.4148	-0.4517	-0.5381	-0.4194	-0.4776	-0.459	-0.449	-0.474	-0.4445	-0.4783	-0.4066	-0.4168	-0.4156	-0.3929	-0.4768	-0.4475	-0.4771	-0.4411	-0.4776	-0.4754	-0.4079	-0.4422	-0.5381	-0.401	-0.4528	-0.4566	-0.4515
176	0.2967	0.6576	0.4813	0.2968	0.3845	0.3381	0.3381	0.547	0.2973	0.4383	0.2967	0.2968	0.2968	0.358	0.3557	0.2945	0.3564	0.3381	0.3832	0.3705	0.2967	0.6576	0.4812	0.3581	0.398	0.378	0.378
177	2.6612	2.6593	2.6593	2.6849	2.6848	2.6849	2.6852	2.6848	2.6846	2.6848	2.6923	2.6873	2.6946	2.6851	2.6849	2.7052	2.6852	2.6855	2.6847	2.6848	2.6858	2.6849	2.6848	2.5373	2.5369	2.5374	2.5518
178	-2.462	-2.4577	-2.4578	-2.4554	-2.4552	-2.4556	-2.4571	-2.4557	-2.4535	-2.4557	-2.4662	-2.4634	-2.4632	-2.4569	-2.4557	-2.4572	-2.4572	-2.459	-2.4556	-2.4574	-2.4611	-2.4557	-2.4557	-2.3835	-2.3826	-2.3841	-2.3912
179	-0.1743	-0.4053	-0.4272	-0.1763	-0.3086	-0.2639	-0.2639	-0.4003	-0.1809	-0.3184	-0.174	-0.1759	-0.1758	-0.088	-0.2912	-0.1207	-0.2941	-0.2639	-0.3126	-0.2871	-0.1742	-0.405	-0.4218	-0.0883	-0.264	-0.1761	-0.1761
180	0.3567	0.7144	0.698	0.3591	0.5494	0.485	0.485	0.6953	0.3664	0.5836	0.3564	0.3586	0.3585	0.4645	0.5292	0.2837	0.5357	0.485	0.5481	0.5222	0.3566	0.711	0.6892	0.4648	0.4861	0.4753	0.4753
181	2.9057	2.8895	2.8891	2.8957	2.8994	2.8983	2.9029	2.8987	2.8902	2.8983	2.9249	2.9121	2.9169	2.9067	2.8993	2.985	2.9039	2.9086	2.8966	2.9027	2.9128	2.8991	2.8987	2.8644	2.8713	2.8692	2.8785
182	27.131	28.884	27.131	21.892	24.172	22.715	24.901	26.265	19.705	26.058	28.451	29.317	28.451	28.545	23.283	25.398	25.469	29.977	21.892	24.078	26.265	28.018	26.265	21.985	24.265	22.808	24.995
183	8	9	9	5	6	7	8	7	5	7	7	9	7	8	7	5	8	9	6	6	6	7	7	6	7	8	9
184	0.5	0.36	0.4091	0.3846	0.4286	0.4375	0.4706	0.35	0.4167	0.3684	0.4375	0.5294	0.4375	0.5	0.4375	0.3125	0.4706	0.4737	0.4286	0.4	0.4	0.2917	0.3333	0.4615	0.5	0.5	0.5294
185	0	10	6	0	0	0	0	6	0	5	0	0	0	0	0	0	0	0	0	0	0	10	6	0	0	0	0
186	40	50	46	31	32	33	36	42	28	39	40	43	40	38	35	36	38	45	32	35	37	47	43	29	30	31	34
187	2	2	2	2	2	3	3	2	2	2	2	2	2	2	3	2	3	3	2	2	2	2	2	2	2	3	3
188	16	25	22	13	14	16	17	20	12	19	16	17	16	16	16	16	17	19	14	15	15	24	21	13	14	16	17
189	8	8	8	6	6	6	6	6	6	6	6	8	6	7	6	6	6	7	6	6	6	6	6	7	7	7	7
190	9	10	10	6	7	8	9	8	6	8	8	10	8	9	8	5	9	11	7	7	7	8	8	7	8	9	10
191	0.5625	0.4	0.4545	0.4615	0.5	0.5	0.5294	0.4	0.5	0.4211	0.5	0.5882	0.5	0.5625	0.5	0.3125	0.5294	0.5789	0.5	0.4667	0.4667	0.3333	0.381	0.5385	0.5714	0.5625	0.5882
192	38	38	38	29	30	30	33	34	26	32	38	41	38	36	32	34	35	42	30	33	35	35	35	27	28	28	31
193	13.422	17.527	16.535	11.301	12.008	13.585	14.293	15.121	10.431	14.414	13.585	14.129	13.585	13.422	13.585	12.293	14.293	15.707	12.008	12.878	12.878	16.983	15.991	11.138	11.845	13.422	14.129
194	10.699	13.716	12.586	8.871	9.9198	9.8946	10.602	11.965	8.0008	11.18	11.155	11.407	11.155	11.51	10.064	9.9395	10.771	12.147	9.0253	9.8956	10.448	13.465	12.335	9.2255	10.274	10.249	10.956
195	7.5605	10.077	8.9996	6.4392	6.1463	6.6463	7.3534	9.533	5.5689	7.801	8.7236	8.2676	8.7236	7.8534	6.6463	7.5605	7.3534	8.0605	6.1463	7.0165	8.0165	10.533	9.4557	5.5689	5.276	5.776	6.4831
196	7.7152	11.113	9.8281	6.5939	6.301	6.8783	7.5854	10.414	5.7236	8.2925	8.8783	8.4223	8.8783	8.0081	6.8783	7.7152	7.5854	8.2925	6.301	7.1712	8.1712	11.569	10.284	5.7236	5.4307	6.0081	6.7152
197	7.896	11.524	10.435	6.3411	6.8791	7.735	8.235	9.3968	5.9304	8.8968	7.7898	8.3791	7.735	7.8791	7.735	7.4304	8.235	9.235	6.8791	7.2518	7.2518	10.88	9.7906	6.4853	7.0233	7.8791	8.3791
198	6.0329	8.2114	7.3517	4.6852	5.4648	5.2779	5.7779	6.7803	4.2376	6.2175	6.1339	6.5161	6.079	7.1069	5.3624	5.7754	5.8624	6.697	4.8323	5.2767	5.5959	7.7743	6.9146	5.713	6.4926	6.3057	6.8057
199	3.9736	5.6134	5.0688	3.1259	2.9568	3.3103	3.8103	5.221	2.6134	3.599	4.5746	4.4568	4.5197	3.4568	3.3103	3.9568	3.8103	3.9568	2.9568	3.4592	4.0366	5.6764	5.1317	2.0629	1.8938	2.2474	2.7474
200	4.0629	6.7828	6.0244	3.2152	3.0461	3.4544	3.9544	6.2709	2.7128	3.8626	4.6639	4.5461	4.6091	3.5461	3.4544	4.0461	3.9544	4.0461	3.0461	3.5485	4.1259	6.8458	6.0874	2.1523	1.9832	2.3914	2.8914
201	2	2	2	2	2	2	2	2	1	2	3	2	2	2	2	2	2	2	2	3	2	2	2	2	2	2	2
202	2	2	2	2	2	2	2	2	1	2	3	2	2	2	2	2	2	2	2	3	2	2	2	2	2	2	2
203	0.7192	0.7344	0.7441	0.7352	0.7905	0.8046	0.7875	0.7136	0.7509	0.7573	0.7014	0.7096	0.7014	0.7576	0.7778	0.7325	0.7631	0.7374	0.7696	0.7541	0.7109	0.7289	0.7385	0.8068	0.8573	0.8696	0.8475
204	10	14	14	8	8	9	10	11	8	10	9	11	9	10	9	8	10	12	8	8	8	12	12	9	9	10	11
205	1	1	1	0	0	-1	-1	0	0	0	0	1	0	0	0	0	0	1	0	0	0	0	0	0	0	-1	-1

Table S12 Amino acid descriptors (2D) used in this study.

	I	I	I	I	I	I	I	I	I	I	I	K	K	K	K	K	K	K	K	K	K	K	K	K	K	K	K	K	K	K	K	L	L	L	L	L	L	L	L	L	L	L	L	L	L	L	L	L	L	L	L	M	M	M	M
	L	M	N	P	Q	R	S	T	V	W	Y	A	C	D	E	F	G	H	I	K	L	M	N	P	Q	R	S	T	V	W	Y	A	C	D	E	F	G	H	I	K	L	M	N	P	Q	R	S	T	V	W	Y	A	C	D	E
No.	IL	IM	IN	IP	IQ	IR	IS	IT	IV	IW	IY	KA	KC	KD	KE	KF	KG	KH	KI	KK	KL	KM	KN	KP	KQ	KR	KS	KT	KV	KW	KY	LA	LC	LD	LE	LF	LG	LH	LI	LK	LL	LM	LN	LP	LQ	LR	LS	LT	LV	LW	LY	MA	MC	MD	ME
206	-0.8473	-0.8472	-0.8469	-0.8514	-0.8473	-0.8473	-0.8462	-0.8461	-0.8471	-0.863	-0.847	-0.863	-0.863	-0.863	-0.863	-0.863	-0.863	-0.863	-0.863	-0.8631	-0.863	-0.863	-0.863	-0.863	-0.863	-0.863	-0.863	-0.863	-0.863	-0.863	-0.863	-0.8477	-0.8471	-0.8505	-0.8543	-0.8473	-0.8484	-0.8488	-0.8474	-0.863	-0.8476	-0.8475	-0.8472	-0.8516	-0.8476	-0.8476	-0.8465	-0.8465	-0.8474	-0.863	-0.8473	-0.8475	-0.8469	-0.8505	-0.8543
207	-0.3383	-0.3249	-0.403	-0.3633	-0.4025	-0.3675	-0.3888	-0.3543	-0.3256	-0.3839	-0.3834	-0.3693	-0.3759	-0.4083	-0.4083	-0.3883	-0.3804	-0.389	-0.3315	-0.3536	-0.3475	-0.3432	-0.3838	-0.3532	-0.3834	-0.3674	-0.3697	-0.3531	-0.3349	-0.3837	-0.3832	-0.368	-0.3821	-0.4268	-0.4268	-0.3885	-0.3914	-0.3892	-0.3299	-0.3522	-0.346	-0.3402	-0.3901	-0.3565	-0.3896	-0.3675	-0.376	-0.3563	-0.3334	-0.3838	-0.3833	-0.3519	-0.3599	-0.4739	-0.4667
208	0.0693	0.0691	0.0913	0.0986	0.0822	0.1045	0.1319	0.1241	0.0843	0.1049	0.1048	0.1849	0.1851	0.1849	0.1849	0.1849	0.1849	0.1849	0.1786	0.1849	0.1783	0.1849	0.1849	0.1849	0.1849	0.1849	0.1851	0.1849	0.1789	0.1849	0.1849	0.075	0.1159	0.0807	0.0808	0.0799	0.1079	0.1142	0.0736	0.1814	0.0718	0.0683	0.0818	0.089	0.0745	0.0955	0.116	0.1084	0.0738	0.0812	0.0825	0.1067	0.1855	0.1077	0.0886
209	2.6564	2.6029	2.5767	2.6874	2.6122	2.669	2.5424	2.5891	2.6274	2.6915	2.6436	2.5387	2.5496	2.5594	2.5958	2.6396	2.4936	2.6126	2.6731	2.6762	2.6581	2.6072	2.5825	2.6862	2.6159	2.6705	2.5507	2.594	2.6301	2.6924	2.6461	2.5054	2.5175	2.5283	2.5684	2.6162	2.4549	2.5868	2.6523	2.656	2.6363	2.5808	2.5538	2.6661	2.5905	2.6497	2.5187	2.5665	2.6059	2.6734	2.6233	2.4065	2.4214	2.4348	2.4846
210	-2.2353	-2.2353	-2.2353	-2.2353	-2.2353	-2.2353	-2.2353	-2.2353	-2.2353	-2.2353	-2.2353	-2.2373	-2.2373	-2.2373	-2.2373	-2.2373	-2.2373	-2.2373	-2.2373	-2.2373	-2.2373	-2.2373	-2.2373	-2.2373	-2.2373	-2.2373	-2.2373	-2.2373	-2.2373	-2.2373	-2.2373	-2.2351	-2.2351	-2.2351	-2.2351	-2.2351	-2.2351	-2.2351	-2.2351	-2.2354	-2.2351	-2.2351	-2.2351	-2.2351	-2.2351	-2.2351	-2.2351	-2.2351	-2.2351	-2.2351	-2.2351	-2.2351	-2.2351	-2.2351	-2.2351
211	-0.292	-0.3105	-0.3862	-0.3297	-0.3651	-0.3269	-0.4225	-0.3928	-0.3003	-0.3355	-0.3639	-0.3654	-0.408	-0.406	-0.4054	-0.3398	-0.3737	-0.3559	-0.3032	-0.3115	-0.3045	-0.3244	-0.3759	-0.3407	-0.3555	-0.3276	-0.4083	-0.3825	-0.3099	-0.3355	-0.3638	-0.3599	-0.4188	-0.4291	-0.427	-0.3281	-0.3824	-0.3593	-0.2915	-0.3036	-0.293	-0.312	-0.3835	-0.3305	-0.3623	-0.3268	-0.4194	-0.3902	-0.3011	-0.3355	-0.3638	-0.3724	-0.4167	-0.4649	-0.421
212	0.0542	0.0555	0.0542	0.0542	0.0542	0.0542	0.0542	0.0542	0.0542	0.1232	0.0581	0.0542	0.0555	0.0542	0.0542	0.0848	0.0542	0.0555	0.0542	0.0542	0.0542	0.0555	0.0542	0.0542	0.0542	0.0542	0.0542	0.0542	0.0542	0.1232	0.0581	0.0542	0.0555	0.0542	0.0542	0.0848	0.0542	0.0555	0.0542	0.0542	0.0542	0.0555	0.0542	0.0542	0.0542	0.0542	0.0542	0.0542	0.0542	0.1232	0.0581	0.0557	0.058	0.056	0.0567
213	2.5991	2.528	2.472	2.614	2.5208	2.5707	2.4388	2.4855	2.5518	2.6663	2.5916	2.4273	2.4362	2.4411	2.4896	2.563	2.381	2.5029	2.5972	2.5842	2.5847	2.5128	2.4568	2.5965	2.5053	2.5558	2.4246	2.4701	2.5363	2.6548	2.5779	2.4279	2.4369	2.4419	2.4905	2.5638	2.3809	2.5038	2.5983	2.585	2.5857	2.5137	2.4576	2.5984	2.5063	2.5567	2.4252	2.471	2.5374	2.6553	2.5787	2.2893	2.3019	2.3081	2.3781
214	-0.6411	-0.6411	-0.6411	-0.6412	-0.6411	-0.6411	-0.6411	-0.6411	-0.6411	-0.6411	-0.6411	-0.6433	-0.6433	-0.6433	-0.6433	-0.6433	-0.6433	-0.6433	-0.6433	-0.6433	-0.6433	-0.6433	-0.6433	-0.6433	-0.6433	-0.6433	-0.6433	-0.6433	-0.6433	-0.6433	-0.6433	-0.6411	-0.6411	-0.6411	-0.6411	-0.6411	-0.6411	-0.6411	-0.6411	-0.6419	-0.6411	-0.6411	-0.6411	-0.6412	-0.6411	-0.6411	-0.6411	-0.6411	-0.6411	-0.6411	-0.6411	-0.6408	-0.6408	-0.6408	-0.6408
215	-0.1777	-0.1699	-0.1992	-0.175	-0.1991	-0.1889	-0.2	-0.1895	-0.1755	-0.194	-0.2161	-0.1865	-0.1733	-0.1966	-0.1958	-0.2061	-0.1926	-0.2071	-0.1769	-0.1837	-0.1817	-0.1724	-0.2082	-0.1827	-0.2082	-0.1961	-0.2094	-0.1963	-0.1783	-0.2024	-0.2161	-0.1847	-0.1725	-0.1943	-0.1937	-0.2044	-0.1895	-0.2053	-0.1762	-0.1823	-0.1806	-0.1717	-0.2049	-0.1794	-0.2048	-0.194	-0.2058	-0.1939	-0.1775	-0.1999	-0.2161	-0.1738	-0.16	-0.1853	-0.1848
216	0.0515	0.1155	0.1152	0.0047	0.1155	0.1045	0.1154	0.107	0.0487	0.1571	0.1393	0.057	0.1285	0.1111	0.1112	0.1597	0.0648	0.1291	0.0504	0.056	0.0558	0.128	0.1275	0.0158	0.128	0.1112	0.1278	0.115	0.0509	0.1633	0.1645	0.0568	0.1264	0.1108	0.1109	0.1574	0.0642	0.127	0.0503	0.0558	0.0556	0.1261	0.1257	0.0138	0.1261	0.1109	0.1259	0.1142	0.0508	0.1595	0.1586	0.1307	0.1623	0.1449	0.1449
217	2.855	2.8083	2.7679	2.8882	2.8071	2.863	2.7269	2.7781	2.8233	2.9281	2.858	2.7083	2.7308	2.7392	2.7806	2.8421	2.6551	2.8012	2.8629	2.8552	2.8461	2.7998	2.7598	2.8768	2.7985	2.8544	2.7201	2.7698	2.8142	2.9203	2.8495	2.6922	2.7161	2.7249	2.7683	2.8324	2.6353	2.7899	2.8538	2.8459	2.8365	2.7884	2.7466	2.8683	2.787	2.845	2.7047	2.757	2.8035	2.9132	2.84	2.617	2.6455	2.6555	2.7063
218	17	17	17	14.063	18	20	15	16	16	19.326	19.048	15	16	18	19	19.048	14	18.05	18	19	18	18	18	15.059	19	21	16	17	17	20.314	20.045	14	15	17	18	18.05	13	17.053	17	18	17	17	17	14.063	18	20	15	16	16	19.326	19.048	14	15	17	18
219	8.1633	9	8.1633	6.0744	8.9917	10.688	7.3025	7.35	7.35	8.9091	9.2092	8.1869	9.0741	9.8685	10.748	10.68	8.32	9.8496	9.8685	11.796	9.8685	10.88	9.8685	7.438	10.748	12.535	9.0741	9	9	10.222	10.714	6.4775	7.3025	8.1633	8.9917	9.1065	6.4533	8.3232	8.1633	9.8685	8.1633	9	8.1633	6.0744	8.9917	10.688	7.3025	7.35	7.35	8.9091	9.2092	7.3125	8.1869	9	9.8685
220	6.4793	6.4793	6.4793	3.25	7.259	8.8128	5.04	5.2645	5.2645	4.9887	6.3281	6.9758	7.0582	8.7075	9.5207	7.7051	8.0816	7.0255	7.259	9.5207	8.7075	8.7075	8.7075	4.3018	9.5207	11.25	7.0582	7.1111	7.1111	6.0464	7.9084	6.1875	6.2222	7.84	8.7075	7.0255	7.1006	6.321	6.4793	8.7075	7.84	7.84	7.84	3.7692	8.7075	10.412	6.2222	6.37	6.37	5.5	7.2	6.1875	6.2222	7.84	8.7075
221	14.705	16.17	13.412	12.047	14.404	15.933	12.708	13.707	13.707	14.626	15.013	12.708	15.169	14.866	15.862	15.013	11.709	14.164	15.705	16.704	15.705	17.171	14.404	13.026	15.397	16.925	13.707	14.705	14.705	15.567	15.984	11.709	14.167	13.871	14.866	14.045	10.711	13.198	14.705	15.705	14.705	16.17	13.412	12.047	14.404	15.933	12.708	13.707	13.707	14.626	15.013	13.166	15.735	15.28	16.281
222	6.9931	8.5325	6.337	5.1461	7.0939	8.405	6.1076	6.2268	6.2268	6.6505	7.1659	6.8472	8.5704	8.0519	8.8765	8.3108	6.8549	7.623	8.5365	10.292	8.5365	10.349	7.7856	6.3718	8.6008	9.9859	7.6874	7.7099	7.7099	7.7363	8.4449	5.3369	6.8672	6.5694	7.3365	6.9852	5.2233	6.3426	6.9931	8.5365	6.9931	8.5325	6.337	5.1461	7.0939	8.405	6.1076	6.2268	6.2268	6.6505	7.1659	6.842	8.6184	8.0323	8.8701
223	5.5078	6.1267	4.9653	2.7304	5.6668	6.8653	4.1712	4.4228	4.4228	3.6853	4.8756	5.7733	6.6476	7.041	7.7971	5.9365	6.5823	5.3825	6.2395	8.2573	7.4846	8.2646	6.7976	3.6567	7.544	8.8828	5.9298	6.0448	6.0448	4.5349	6.1811	5.0396	5.8307	6.2402	7.041	5.3327	5.661	4.7587	5.5078	7.4846	6.6644	7.4133	6.008	3.1666	6.7976	8.1112	5.1496	5.3516	5.3516	4.0631	5.5474	5.7671	6.5692	6.9576	7.7904
224	6.0492	8.1159	4.9995	3.8746	5.6767	6.6959	5.1742	5.3342	5.3342	4.2293	5.123	5.8009	8.1251	6.6499	7.4104	5.9415	5.7333	5.3986	7.4479	9.0478	7.4479	9.8724	6.2303	4.8824	6.97	8.0483	6.5855	6.6693	6.6693	5.0179	6.1357	4.4636	6.486	5.3601	6.0591	4.9054	4.3036	4.4057	6.0492	7.4479	6.0492	8.1159	4.9995	3.8746	5.6767	6.6959	5.1742	5.3342	5.3342	4.2293	5.123	6.4342	9.0404	7.2197	8.0229
225	5	5	7	5	7	8	6	6	5	6	6	6	6	8	8	6	6	8	6	7	6	6	8	6	8	9	7	7	6	7	7	5	5	7	7	5	5	7	5	6	5	5	7	5	7	8	6	6	5	6	6	5	5	7	7
226	4	4	6	3	6	9	5	5	4	5	5	7	7	7	7	7	7	8	7	10	7	7	9	6	9	12	8	8	7	8	8	4	4	4	4	4	4	5	4	7	4	4	6	3	6	9	5	5	4	5	5	4	4	4	4
227	1	1	1	1	1	1	1	1	1	1	1	1	1	1	1	1	1	1	1	1	1	1	1	1	1	1	1	1	1	1	1	1	1	1	1	1	1	1	1	1	1	1	1	1	1	1	1	1	1	1	1	1	1	1	1
228	0	0	1	0	1	1	0	0	0	0	0	1	1	1	1	1	1	1	1	1	1	1	1	1	1	1	1	1	1	1	1	0	0	0	0	0	0	0	0	1	0	0	1	0	1	1	0	0	0	0	0	0	0	0	0
229	1.879	1.12	-1.017	0.853	-0.575	0.113	-0.57	-0.108	1.437	2.366	1.692	-0.88	-0.77	-1.507	-1.065	0.655	-1.342	-1.649	0.534	-0.811	0.534	-0.225	-2.362	-0.492	-1.92	-1.232	-1.915	-1.453	0.092	1.021	0.347	0.465	0.575	-0.162	0.28	2	0.003	-0.304	1.879	0.534	1.879	1.12	-1.017	0.853	-0.575	0.113	-0.57	-0.108	1.437	2.366	1.692	-0.294	-0.184	-0.921	-0.479
230	-2.4918	-2.126	-1.0928	-1.3787	-1.2946	-1.632	-0.7298	-1.057	-1.6631	-3.0516	-2.3998	-0.2859	-0.9471	-0.0969	-0.2986	-1.788	0.0414	-0.5452	-1.2046	-0.2309	-1.5181	-1.1523	-0.1191	-0.405	-0.3209	-0.6583	0.2439	-0.0833	-0.6894	-2.0779	-1.4261	-1.573	-2.2343	-1.384	-1.5858	-3.0752	-1.2458	-1.8323	-2.4918	-1.5181	-2.8052	-2.4394	-1.4063	-1.6922	-1.608	-1.9455	-1.0433	-1.3705	-1.9765	-3.3651	-2.7132	-1.2072	-1.8685	-1.0182	-1.22
231	6.7063	7.0001	6.1659	6.0704	6.6391	7.5653	5.489	5.9262	6.241	8.9141	7.9967	5.6784	6.4372	6.3439	6.8189	8.2268	5.2317	7.4545	7.073	7.4232	7.063	7.3573	6.5165	6.4264	6.9906	7.9082	5.844	6.2822	6.5965	9.2725	8.3603	5.3154	6.0722	5.9745	6.4488	7.8548	4.87	7.0965	6.7063	7.063	6.6962	6.99	6.1558	6.0603	6.629	7.5553	5.4789	5.9161	6.231	8.904	7.9867	5.6002	6.3244	6.2564	6.7342
232	0	0	0	5	0	1	0	0	0	10	6	0	0	0	0	6	0	5	0	0	0	0	0	5	0	1	0	0	0	10	6	0	0	0	0	6	0	5	0	0	0	0	0	5	0	1	0	0	0	10	6	0	0	0	0
233	1	1	1	1	1	0	1	1	1	1	1	0	0	0	0	1	0	0	1	0	1	1	0	1	0	0	0	0	1	1	1	1	1	1	1	1	1	1	1	1	1	1	1	1	1	0	1	1	1	1	1	1	1	1	1
234	0	0	0	1	0	0	0	0	0	2	1	0	0	0	0	1	0	1	0	0	0	0	0	1	0	0	0	0	0	2	1	0	0	0	0	1	0	1	0	0	0	0	0	1	0	0	0	0	0	2	1	0	0	0	0
235	7	8	7	5	8	10	6	6	6	7	7	7	8	9	10	9	7	9	9	11	9	10	9	7	10	12	8	8	8	9	9	5	6	7	8	7	5	7	7	9	7	8	7	5	8	10	6	6	6	7	7	6	7	8	9
236	0	0	1	0	1	2	0	0	0	0	0	2	2	2	2	1	2	2	1	3	1	1	2	1	2	4	2	2	1	1	1	0	0	1	1	0	0	0	0	1	0	0	1	0	1	2	0	0	0	0	0	0	0	1	1
237	3.953	4.413	5.323	3.883	5.323	7.731	4.633	4.633	3.953	5.187	5.229	5.806	6.216	6.712	6.712	6.7	5.806	6.673	5.806	7.659	5.806	6.266	7.176	5.736	7.176	9.584	6.486	6.486	5.806	7.04	7.082	3.953	4.363	4.859	4.859	4.847	3.953	4.82	3.953	5.806	3.953	4.413	5.323	3.883	5.323	7.731	4.633	4.633	3.953	5.187	5.229	4.413	4.823	5.319	5.319
238	-3.953	-4.413	-5.323	-3.883	-5.323	-6.731	-4.633	-4.633	-3.953	-5.188	-5.229	-4.806	-5.216	-6.712	-6.712	-5.7	-4.806	-5.673	-4.806	-5.659	-4.806	-5.266	-6.176	-4.736	-6.176	-7.584	-5.486	-5.486	-4.806	-6.041	-6.082	-3.953	-4.363	-5.859	-5.859	-4.847	-3.953	-4.82	-3.953	-4.806	-3.953	-4.413	-5.323	-3.883	-5.323	-6.731	-4.633	-4.633	-3.953	-5.188	-5.229	-4.413	-4.823	-6.319	-6.319
239	2.4193	2.4295	2.8673	2.269	2.8868	4.1291	2.6149	2.6483	2.3664	2.8158	2.8066	3.5017	3.637	3.5611	3.5948	3.8104	3.4653	4.053	3.6565	4.8987	3.6566	3.6668	4.1046	3.5063	4.1241	5.3664	3.8522	3.8856	3.6037	4.0531	4.0439	2.2616	2.397	2.3211	2.3548	2.5704	2.2252	2.8129	2.4164	3.6587	2.4166	2.4267	2.8646	2.2663	2.8841	4.1264	2.6122	2.6456	2.3637	2.813	2.8038	2.2786	2.4139	2.338	2.3717
240	-2.4193	-2.4295	-2.8673	-2.269	-2.8868	-3.1291	-2.6149	-2.6483	-2.3664	-2.8158	-2.8066	-2.5017	-2.637	-3.5611	-3.5948	-2.8104	-2.4653	-3.053	-2.6565	-2.8987	-2.6566	-2.6668	-3.1046	-2.5063	-3.1241	-3.3664	-2.8522	-2.8856	-2.6037	-3.0531	-3.0439	-2.2616	-2.397	-3.3211	-3.3548	-2.5704	-2.2252	-2.8129	-2.4164	-2.6587	-2.4166	-2.4267	-2.8646	-2.2663	-2.8841	-3.1264	-2.6122	-2.6456	-2.3637	-2.813	-2.8038	-2.2786	-2.4139	-3.338	-3.3717
241	0.1428	0.1422	0.1205	0.1523	0.1197	0.0837	0.1321	0.1305	0.146	0.1227	0.1231	0.0987	0.095	0.097	0.0961	0.0907	0.0997	0.0853	0.0945	0.0705	0.0945	0.0942	0.0842	0.0985	0.0838	0.0644	0.0897	0.0889	0.0959	0.0853	0.0854	0.1528	0.1442	0.1489	0.1467	0.1344	0.1553	0.1228	0.143	0.0944	0.143	0.1424	0.1206	0.1525	0.1198	0.0837	0.1323	0.1306	0.1462	0.1228	0.1232	0.1516	0.1431	0.1478	0.1457
242	0.2264	0.2254	0.191	0.2414	0.1897	0.175	0.2094	0.2068	0.2314	0.1945	0.1951	0.2189	0.2077	0.1544	0.1529	0.1949	0.2223	0.1794	0.2062	0.1889	0.2062	0.2054	0.1764	0.2185	0.1753	0.1627	0.192	0.1898	0.2103	0.1794	0.1799	0.2422	0.2285	0.1655	0.1639	0.2131	0.2463	0.1947	0.2266	0.206	0.2266	0.2257	0.1912	0.2417	0.1899	0.1752	0.2096	0.207	0.2317	0.1947	0.1953	0.2404	0.2269	0.1647	0.1631
243	76.777	68.246	46.919	77.173	64.151	55.45	38.388	51.185	68.246	72.573	46.919	47.09	53.502	66.234	66.064	42.824	34.294	68.272	72.682	59.886	72.682	64.151	42.824	73.078	60.056	51.355	34.294	47.09	64.151	68.478	42.824	59.886	66.298	79.03	78.86	55.621	47.09	81.068	85.478	72.682	85.478	76.947	55.621	85.874	72.853	64.151	47.09	59.886	76.947	81.275	55.621	51.355	57.767	70.5	70.329
244	22.12	22.12	30.821	22.12	22.12	43.843	45.602	31.29	22.12	43.447	47.64	43.843	43.843	43.843	43.843	65.17	58.155	76.045	43.843	65.566	43.843	43.843	52.544	43.843	43.843	65.566	67.325	53.013	43.843	65.17	69.363	22.12	22.12	22.12	22.12	43.447	36.432	54.322	22.12	43.843	22.12	22.12	30.821	22.12	22.12	43.843	45.602	31.29	22.12	43.447	47.64	22.12	22.12	22.12	22.12
245	4.2654	4.2654	4.2654	4.2654	4.2654	4.2654	4.2654	4.2654	4.2654	4.2654	4.2654	4.2654	12.046	4.2654	4.2654	4.2654	4.2654	8.5308	4.2654	4.2654	4.2654	4.2654	4.2654	4.2654	4.2654	4.2654	4.2654	4.2654	4.2654	4.2654	4.2654	4.2654	12.046	4.2654	4.2654	4.2654	4.2654	8.5308	4.2654	4.2654	4.2654	4.2654	4.2654	4.2654	4.2654	4.2654	4.2654	4.2654	4.2654	4.2654	4.2654	4.2654	12.046	4.2654	4.2654
246	11.765	11.765	29.003	3.146	29.003	11.765	11.765	11.765	11.765	20.384	11.765	11.765	11.765	11.765	11.765	11.765	11.765	20.384	11.765	11.765	11.765	11.765	29.003	3.146	29.003	11.765	11.765	11.765	11.765	20.384	11.765	11.765	11.765	11.765	11.765	11.765	11.765	20.384	11.765	11.765	11.765	11.765	29.003	3.146	29.003	11.765	11.765	11.765	11.765	20.384	11.765	11.765	11.765	11.765	11.765
247	0	0	12.95	0	12.95	0	10.324	10.324	0	0	10.324	0	0	0	0	0	0	0	0	0	0	0	12.95	0	12.95	0	10.324	10.324	0	0	10.324	0	0	0	0	0	0	0	0	0	0	0	12.95	0	12.95	0	10.324	10.324	0	0	10.324	0	0	0	0
248	12.95	12.95	12.95	12.95	12.95	56.045	12.95	12.95	12.95	12.95	12.95	12.95	12.95	12.95	12.95	12.95	12.95	12.95	12.95	12.95	12.95	12.95	12.95	12.95	12.95	56.045	12.95	12.95	12.95	12.95	12.95	12.95	12.95	12.95	12.95	12.95	12.95	12.95	12.95	12.95	12.95	12.95	12.95	12.95	12.95	56.045	12.95	12.95	12.95	12.95	12.95	12.95	12.95	12.95	12.95
249	25.857	25.857	25.857	25.857	25.857	41.539	25.857	25.857	25.857	25.857	25.857	51.714	51.714	51.714	51.714	51.714	51.714	51.714	51.714	77.571	51.714	51.714	51.714	51.714	51.714	67.396	51.714	51.714	51.714	51.714	51.714	25.857	25.857	25.857	25.857	25.857	25.857	25.857	25.857	51.714	25.857	25.857	25.857	25.857	25.857	41.539	25.857	25.857	25.857	25.857	25.857	25.857	25.857	25.857	25.857
250	17.403	48.804	8.7013	26.104	17.403	26.104	8.7013	27.449	8.7013	31.866	44.121	36.15	17.403	17.403	34.805	28.312	17.403	17.403	17.403	43.507	26.104	57.505	17.403	34.805	26.104	34.805	17.403	36.15	17.403	40.567	52.822	18.748	0	0	17.403	10.91	0	0	0	26.104	8.7013	40.102	0	17.403	8.7013	17.403	0	18.748	0	23.165	35.42	50.149	31.401	31.401	48.804
251	74.991	37.495	37.495	37.495	37.495	37.495	37.495	37.495	74.991	74.26	62.005	0	0	0	0	61.275	0	0	46.197	0	37.495	0	0	0	0	0	0	0	37.495	36.765	24.51	37.495	37.495	37.495	37.495	98.77	37.495	37.495	37.495	83.692	37.495	74.991	37.495	37.495	37.495	37.495	37.495	37.495	74.991	74.26	62.005	0	0	0	0
252	0	19.761	0	0	0	0	0	0	0	0	0	0	26.363	0	0	0	0	0	0	0	0	19.761	0	0	0	0	0	0	0	0	0	0	26.363	0	0	0	0	0	0	0	0	19.761	0	0	0	0	0	0	0	0	0	19.761	46.124	19.761	19.761
253	0	0	0	0	0	0	0	0	0	0	0	0	0	0	0	0	0	5.6826	0	0	0	0	0	0	0	0	0	0	0	0	0	0	0	0	0	0	0	5.6826	0	0	0	0	0	0	0	0	0	0	0	0	0	0	0	0	0
254	13.567	13.567	27.134	13.567	27.134	27.006	13.567	13.567	13.567	13.567	13.567	13.567	13.567	13.567	13.567	13.567	13.567	13.567	13.567	13.567	13.567	13.567	27.134	13.567	27.134	27.006	13.567	13.567	13.567	13.567	13.567	13.567	13.567	13.567	13.567	13.567	13.567	13.567	13.567	13.567	13.567	13.567	27.134	13.567	27.134	27.006	13.567	13.567	13.567	13.567	13.567	13.567	13.567	13.567	13.567
255	28.376	28.376	35.027	28.239	35.027	28.376	36.144	36.144	28.376	28.513	36.144	29.482	29.482	56.615	56.615	29.482	29.482	29.618	29.482	30.587	29.482	29.482	36.133	29.345	36.133	29.482	37.249	37.249	29.482	29.618	37.249	28.376	28.376	55.51	55.51	28.376	28.376	28.513	28.376	29.482	28.376	28.376	35.027	28.239	35.027	28.376	36.144	36.144	28.376	28.513	36.144	28.376	28.376	55.51	55.51
256	0.7197	0.7246	0.5798	0.6787	0.6049	0.5391	0.5967	0.6232	0.7019	0.7532	0.6868	0.5706	0.6049	0.5156	0.5438	0.6648	0.5389	0.6267	0.6455	0.5787	0.6455	0.6514	0.509	0.5967	0.5368	0.4825	0.5177	0.5476	0.6242	0.686	0.6203	0.6564	0.6856	0.5891	0.6144	0.7357	0.6292	0.6998	0.7197	0.6455	0.7197	0.7246	0.5798	0.6787	0.6049	0.5391	0.5967	0.6232	0.7019	0.7532	0.6868	0.6638	0.6918	0.597	0.6214
257	0.4663	0.5048	0.3997	0.4201	0.406	0.3585	0.3914	0.4371	0.4639	0.4523	0.4938	0.3158	0.3184	0.3147	0.3552	0.4128	0.2588	0.2179	0.351	0.2742	0.351	0.3894	0.2812	0.2914	0.2938	0.2626	0.2615	0.3127	0.3417	0.3509	0.3867	0.4178	0.4119	0.4059	0.4431	0.4963	0.3647	0.2957	0.4361	0.351	0.4361	0.4751	0.3676	0.3854	0.3758	0.3323	0.3559	0.404	0.4317	0.4257	0.4663	0.4657	0.456	0.4492	0.4831
258	0.1456	0.1431	0.2293	0.1666	0.2156	0.1669	0.2029	0.1895	0.1549	0.1284	0.1575	0.1716	0.1579	0.2521	0.2374	0.134	0.1843	0.1607	0.1417	0.1381	0.1417	0.1393	0.2205	0.1609	0.208	0.1625	0.1948	0.1827	0.1502	0.1257	0.1534	0.1785	0.1633	0.2631	0.2469	0.1373	0.1926	0.1656	0.1456	0.1417	0.1456	0.1431	0.2293	0.1666	0.2156	0.1669	0.2029	0.1895	0.1549	0.1284	0.1575	0.1746	0.1601	0.258	0.2424
259	0.2803	0.2754	0.4202	0.3213	0.3951	0.4609	0.4033	0.3768	0.2981	0.2468	0.3132	0.4294	0.3951	0.4844	0.4562	0.3352	0.4611	0.3733	0.3545	0.4213	0.3545	0.3486	0.491	0.4033	0.4632	0.5175	0.4823	0.4524	0.3758	0.314	0.3797	0.3436	0.3144	0.4109	0.3856	0.2643	0.3708	0.3002	0.2803	0.3545	0.2803	0.2754	0.4202	0.3213	0.3951	0.4609	0.4033	0.3768	0.2981	0.2468	0.3132	0.3362	0.3082	0.403	0.3786
260	0.5337	0.4952	0.6003	0.5799	0.594	0.6415	0.6086	0.5629	0.5361	0.5477	0.5062	0.6842	0.6816	0.6853	0.6448	0.5872	0.7412	0.7821	0.649	0.7258	0.649	0.6106	0.7188	0.7086	0.7062	0.7374	0.7385	0.6873	0.6583	0.6491	0.6133	0.5822	0.5881	0.5941	0.5569	0.5037	0.6353	0.7043	0.5639	0.649	0.5639	0.5249	0.6324	0.6146	0.6242	0.6677	0.6441	0.596	0.5683	0.5743	0.5337	0.5343	0.544	0.5508	0.5169
261	0.1347	0.1324	0.1909	0.1547	0.1795	0.294	0.2005	0.1873	0.1433	0.1184	0.1557	0.2578	0.2372	0.2323	0.2188	0.2012	0.2768	0.2126	0.2128	0.2832	0.2128	0.2093	0.2705	0.2424	0.2552	0.355	0.2875	0.2697	0.2256	0.1883	0.2263	0.1651	0.1511	0.1478	0.1387	0.127	0.1782	0.1346	0.1347	0.2128	0.1347	0.1324	0.1909	0.1547	0.1795	0.294	0.2005	0.1873	0.1433	0.1184	0.1557	0.1616	0.1481	0.145	0.1362
262	207.32	212.46	157.21	170.3	174.44	178.92	146.22	163.45	190.09	246.8	216.72	143.11	164.92	143.51	160.74	213.61	125.88	190.63	196.15	184.99	196.15	201.29	146.04	159.14	163.27	167.76	135.05	152.28	178.92	235.63	205.55	154.28	176.09	154.68	171.91	224.78	137.05	201.8	207.32	196.15	207.32	212.46	157.21	170.3	174.44	178.92	146.22	163.45	190.09	246.8	216.72	159.41	181.22	159.81	177.04
263	134.34	148	108.36	105.41	117.06	118.98	95.907	114.65	125.64	148.21	155.84	79.199	86.814	87.585	104.99	132.64	60.451	66.271	106.65	87.661	106.65	120.31	80.669	77.717	89.37	91.293	68.219	86.966	97.947	120.52	128.15	98.186	105.8	106.57	123.97	151.62	79.438	85.258	125.64	106.65	125.64	139.3	99.656	96.704	108.36	110.28	87.206	105.95	116.93	139.5	147.14	111.85	119.47	120.24	137.64
264	41.943	41.943	62.161	41.806	62.161	55.382	49.711	49.711	41.943	42.08	49.711	43.049	43.049	70.182	70.182	43.049	43.049	48.868	43.049	44.154	43.049	43.049	63.267	42.912	63.267	56.488	50.816	50.816	43.049	43.185	50.816	41.943	41.943	69.077	69.077	41.943	41.943	47.763	41.943	43.049	41.943	41.943	62.161	41.806	62.161	55.382	49.711	49.711	41.943	42.08	49.711	41.943	41.943	69.077	69.077
265	80.75	80.75	113.92	80.613	113.92	152.97	98.841	98.841	80.75	80.887	98.841	107.71	107.71	134.85	134.85	107.71	107.71	113.53	107.71	134.67	107.71	107.71	140.88	107.58	140.88	179.93	125.8	125.8	107.71	107.85	125.8	80.75	80.75	107.88	107.88	80.75	80.75	86.569	80.75	107.71	80.75	80.75	113.92	80.613	113.92	152.97	98.841	98.841	80.75	80.887	98.841	80.75	80.75	107.88	107.88
266	153.73	145.2	162.76	145.51	171.3	212.91	149.15	147.64	145.2	179.48	159.72	171.63	185.82	190.77	190.6	188.69	173.14	237.89	197.22	232	197.22	188.69	206.25	189	214.78	256.39	192.64	191.12	188.69	222.96	203.21	136.84	151.04	155.99	155.82	153.9	138.36	203.11	162.44	197.22	162.44	153.9	171.47	154.21	180	221.61	157.85	156.34	153.9	188.18	168.42	128.31	142.51	147.46	147.29
267	38.807	38.807	51.756	38.807	51.756	97.583	49.131	49.131	38.807	38.807	49.131	64.664	64.664	64.664	64.664	64.664	64.664	64.664	64.664	90.521	64.664	64.664	77.613	64.664	77.613	123.44	74.988	74.988	64.664	64.664	74.988	38.807	38.807	38.807	38.807	38.807	38.807	38.807	38.807	64.664	38.807	38.807	51.756	38.807	51.756	97.583	49.131	49.131	38.807	38.807	49.131	38.807	38.807	38.807	38.807
268	0.4444	0.5	0.4444	0.5	0.5	0.5	0.5	0.5	0.5	0.5	0.5	0.5	0.5	0.4545	0.5	0.4615	0.5	0.5	0.4545	0.4615	0.4545	0.5	0.4545	0.5	0.5	0.5	0.5	0.5	0.5	0.5	0.5	0.5	0.5	0.4444	0.5	0.4545	0.5	0.5	0.4444	0.4545	0.4444	0.5	0.4444	0.5	0.5	0.5	0.5	0.5	0.5	0.5	0.5	0.4444	0.4444	0.5	0.4545
269	0.8	1	0.8	1	1	1	1	1	1	1	1	1	1	0.8333	1	0.8571	1	1	0.8333	0.8571	0.8333	1	0.8333	1	1	1	1	1	1	1	1	1	1	0.8	1	0.8333	1	1	0.8	0.8333	0.8	1	0.8	1	1	1	1	1	1	1	1	0.8	0.8	1	0.8333
270	3.953	4.413	5.323	3.883	5.323	7.731	4.633	4.633	3.953	5.187	5.229	5.806	6.216	6.712	6.712	6.7	5.806	6.673	5.806	7.659	5.806	6.266	7.176	5.736	7.176	9.584	6.486	6.486	5.806	7.04	7.082	3.953	4.363	4.859	4.859	4.847	3.953	4.82	3.953	5.806	3.953	4.413	5.323	3.883	5.323	7.731	4.633	4.633	3.953	5.187	5.229	4.413	4.823	5.319	5.319
271	-3.953	-4.413	-5.323	-3.883	-5.323	-6.731	-4.633	-4.633	-3.953	-5.188	-5.229	-4.806	-5.216	-6.712	-6.712	-5.7	-4.806	-5.673	-4.806	-5.659	-4.806	-5.266	-6.176	-4.736	-6.176	-7.584	-5.486	-5.486	-4.806	-6.041	-6.082	-3.953	-4.363	-5.859	-5.859	-4.847	-3.953	-4.82	-3.953	-4.806	-3.953	-4.413	-5.323	-3.883	-5.323	-6.731	-4.633	-4.633	-3.953	-5.188	-5.229	-4.413	-4.823	-6.319	-6.319
272	0.2292	0.2053	0.1702	0.2333	0.1702	0.1552	0.1956	0.1956	0.2292	0.1747	0.1733	0.156	0.1458	0.135	0.135	0.1352	0.156	0.1358	0.156	0.1183	0.156	0.1446	0.1263	0.1579	0.1263	0.1252	0.1397	0.1397	0.156	0.1287	0.1279	0.2292	0.2077	0.1865	0.1865	0.1869	0.2292	0.188	0.2292	0.156	0.2292	0.2053	0.1702	0.2333	0.1702	0.1552	0.1956	0.1956	0.2292	0.1747	0.1733	0.2053	0.1878	0.1703	0.1703
273	0.2277	0.2039	0.1691	0.2318	0.1691	0.1437	0.1943	0.1943	0.2277	0.1735	0.1721	0.1873	0.1725	0.1341	0.1341	0.1579	0.1873	0.1586	0.1873	0.159	0.1873	0.1709	0.1457	0.19	0.1457	0.1275	0.1641	0.1641	0.1873	0.149	0.148	0.2277	0.2063	0.1536	0.1536	0.1857	0.2277	0.1867	0.2277	0.1873	0.2277	0.2039	0.1691	0.2318	0.1691	0.1437	0.1943	0.1943	0.2277	0.1735	0.1721	0.2039	0.1866	0.1424	0.1424
274	0.6278	0.4598	0.4186	0.555	0.4534	0.4196	0.4276	0.5035	0.6041	0.5954	0.6029	0.4124	0.3236	0.3202	0.3599	0.5413	0.3691	0.4134	0.515	0.4133	0.515	0.3575	0.3107	0.4308	0.3497	0.3305	0.3083	0.3873	0.4858	0.4971	0.5006	0.5438	0.4383	0.4323	0.4672	0.6491	0.5077	0.5205	0.6278	0.515	0.6278	0.4598	0.4186	0.555	0.4534	0.4196	0.4276	0.5035	0.6041	0.5954	0.6029	0.3405	0.254	0.252	0.2973
275	0.1456	0.2104	0.2293	0.1666	0.2156	0.1669	0.2029	0.1895	0.1549	0.3559	0.3199	0.1716	0.2546	0.2834	0.2669	0.3315	0.1843	0.2153	0.1417	0.1381	0.1417	0.2033	0.2205	0.1609	0.208	0.1625	0.1948	0.1827	0.1502	0.3427	0.308	0.1785	0.266	0.2962	0.278	0.3451	0.1926	0.2232	0.1456	0.1417	0.1456	0.2104	0.2293	0.1666	0.2156	0.1669	0.2029	0.1895	0.1549	0.3559	0.3199	0.2569	0.3362	0.3644	0.3423
276	0.1456	0.2104	0.2293	0.1666	0.2156	0.1669	0.2029	0.1895	0.1549	0.1791	0.1575	0.1716	0.2546	0.2521	0.2374	0.134	0.1843	0.2153	0.1417	0.1381	0.1417	0.2033	0.2205	0.1609	0.208	0.1625	0.1948	0.1827	0.1502	0.1741	0.1534	0.1785	0.266	0.2631	0.2469	0.1373	0.1926	0.2232	0.1456	0.1417	0.1456	0.2104	0.2293	0.1666	0.2156	0.1669	0.2029	0.1895	0.1549	0.1791	0.1575	0.2569	0.3362	0.3319	0.3118
277	0.3722	0.5402	0.5814	0.445	0.5466	0.5804	0.5724	0.4965	0.3959	0.4046	0.3971	0.5876	0.6764	0.6798	0.6401	0.4587	0.6309	0.5866	0.485	0.5867	0.485	0.6425	0.6893	0.5692	0.6503	0.6695	0.6917	0.6127	0.5142	0.5029	0.4994	0.4562	0.5617	0.5677	0.5328	0.3509	0.4923	0.4795	0.3722	0.485	0.3722	0.5402	0.5814	0.445	0.5466	0.5804	0.5724	0.4965	0.3959	0.4046	0.3971	0.6595	0.746	0.748	0.7027
278	0.8544	0.7896	0.7707	0.8334	0.7844	0.8331	0.7971	0.8105	0.8451	0.6441	0.6801	0.8284	0.7454	0.7166	0.7331	0.6685	0.8157	0.7847	0.8583	0.8619	0.8583	0.7967	0.7795	0.8391	0.792	0.8375	0.8052	0.8173	0.8498	0.6573	0.692	0.8215	0.734	0.7038	0.722	0.6549	0.8074	0.7768	0.8544	0.8583	0.8544	0.7896	0.7707	0.8334	0.7844	0.8331	0.7971	0.8105	0.8451	0.6441	0.6801	0.7431	0.6638	0.6356	0.6577
279	0.2266	0.3297	0.3521	0.2784	0.3311	0.4135	0.3695	0.3069	0.241	0.2255	0.2396	0.4159	0.4218	0.4276	0.4027	0.3247	0.4466	0.3713	0.3433	0.4485	0.3433	0.4393	0.4688	0.4083	0.4423	0.5071	0.4968	0.4299	0.364	0.3288	0.346	0.2778	0.2957	0.3046	0.2859	0.2137	0.2997	0.2563	0.2266	0.3433	0.2266	0.3297	0.3521	0.2784	0.3311	0.4135	0.3695	0.3069	0.241	0.2255	0.2396	0.4026	0.4098	0.4161	0.3909
280	180.85	134.82	113.49	139.26	130.73	139.26	104.79	132.07	163.61	195.09	190.24	103.45	88.216	89.136	106.37	173.95	86.216	125.74	156.49	132.13	156.49	110.46	89.136	114.9	106.37	114.9	80.435	107.71	139.26	170.74	165.88	127.81	112.57	113.49	130.73	198.3	110.57	150.1	180.85	156.49	180.85	134.82	113.49	139.26	130.73	139.26	104.79	132.07	163.61	195.09	190.24	81.78	66.548	67.468	84.7

Table S12 Amino acid descriptors (2D) used in this study.

	I	I	I	I	I	I	I	I	I	I	I	K	K	K	K	K	K	K	K	K	K	K	K	K	K	K	K	K	K	K	K	L	L	L	L	L	L	L	L	L	L	L	L	L	L	L	L	L	L	L	L	M	M	M	M
	L	M	N	P	Q	R	S	T	V	W	Y	A	C	D	E	F	G	H	I	K	L	M	N	P	Q	R	S	T	V	W	Y	A	C	D	E	F	G	H	I	K	L	M	N	P	Q	R	S	T	V	W	Y	A	C	D	E
No.	IL	IM	IN	IP	IQ	IR	IS	IT	IV	IW	IY	KA	KC	KD	KE	KF	KG	KH	KI	KK	KL	KM	KN	KP	KQ	KR	KS	KT	KV	KW	KY	LA	LC	LD	LE	LF	LG	LH	LI	LK	LL	LM	LN	LP	LQ	LR	LS	LT	LV	LW	LY	MA	MC	MD	ME
281	41.943	61.704	62.161	41.806	62.161	55.382	49.711	49.711	41.943	116.62	100.94	43.049	69.412	78.884	78.884	106.53	43.049	65.477	43.049	44.154	43.049	62.809	63.267	42.912	63.267	56.488	50.816	50.816	43.049	117.72	102.04	41.943	68.306	77.778	77.778	105.43	41.943	64.372	41.943	43.049	41.943	61.704	62.161	41.806	62.161	55.382	49.711	49.711	41.943	116.62	100.94	61.704	88.067	97.539	97.539
282	41.943	61.704	62.161	41.806	62.161	55.382	49.711	49.711	41.943	58.689	49.711	43.049	69.412	70.182	70.182	43.049	43.049	65.477	43.049	44.154	43.049	62.809	63.267	42.912	63.267	56.488	50.816	50.816	43.049	59.794	50.816	41.943	68.306	69.077	69.077	41.943	41.943	64.372	41.943	43.049	41.943	61.704	62.161	41.806	62.161	55.382	49.711	49.711	41.943	58.689	49.711	61.704	88.067	88.838	88.838
283	107.22	158.38	157.63	111.66	157.63	192.63	140.27	130.22	107.22	132.59	125.31	147.38	184.42	189.22	189.22	147.38	147.38	178.43	147.38	187.53	147.38	198.54	197.78	151.81	197.78	232.79	180.42	170.37	147.38	172.74	165.47	107.22	144.26	149.07	149.07	107.22	107.22	138.27	107.22	147.38	107.22	158.38	157.63	111.66	157.63	192.63	140.27	130.22	107.22	132.59	125.31	158.38	195.43	200.23	200.23
284	246.13	231.5	208.96	209.11	226.19	276.51	195.35	212.58	228.89	211.07	214.62	207.78	203.22	199.47	216.7	214.79	190.54	238.69	260.82	275.51	260.82	246.19	223.65	223.8	240.88	291.2	210.04	227.27	243.59	225.76	229.31	193.09	188.53	184.78	202.01	200.1	175.85	224	246.13	260.82	246.13	231.5	208.96	209.11	226.19	276.51	195.35	212.58	228.89	211.07	214.62	178.46	173.91	170.16	187.39
285	65.28	96.681	95.468	69.853	95.468	137.25	90.556	80.509	65.28	73.899	75.604	104.33	115.01	119.04	119.04	104.33	104.33	112.95	104.33	143.38	104.33	135.73	134.52	108.9	134.52	176.3	129.6	119.56	104.33	112.95	114.65	65.28	75.957	79.989	79.989	65.28	65.28	73.899	65.28	104.33	65.28	96.681	95.468	69.853	95.468	137.25	90.556	80.509	65.28	73.899	75.604	96.681	107.36	111.39	111.39
286	5	5	5	4	5	6	4	4	4	6	6	5	5	6	6	7	5	6	6	7	6	6	6	5	6	7	5	5	5	7	7	4	4	5	5	6	4	5	5	6	5	5	5	4	5	6	4	4	4	6	6	5	5	5	6
287	0	0	0	0	0	0	0	0	0	0	0	0	1	0	0	0	0	0	0	0	0	0	0	0	0	0	0	0	0	0	0	0	1	0	0	0	0	0	0	0	0	0	0	0	0	0	0	0	0	0	0	0	1	0	0
288	0	0	0	1	0	0	0	0	0	2	1	0	0	0	0	1	0	1	0	0	0	0	0	1	0	0	0	0	0	2	1	0	0	0	0	1	0	1	0	0	0	0	0	1	0	0	0	0	0	2	1	0	0	0	0
289	0.2292	0.2053	0.1702	0.2333	0.1702	0.1552	0.1956	0.1956	0.2292	0.1747	0.1733	0.156	0.1458	0.135	0.135	0.1352	0.156	0.1358	0.156	0.1183	0.156	0.1446	0.1263	0.1579	0.1263	0.1252	0.1397	0.1397	0.156	0.1287	0.1279	0.2292	0.2077	0.1865	0.1865	0.1869	0.2292	0.188	0.2292	0.156	0.2292	0.2053	0.1702	0.2333	0.1702	0.1552	0.1956	0.1956	0.2292	0.1747	0.1733	0.2053	0.1878	0.1703	0.1703
290	0.2277	0.2039	0.1691	0.2318	0.1691	0.1437	0.1943	0.1943	0.2277	0.1735	0.1721	0.1873	0.1725	0.1341	0.1341	0.1579	0.1873	0.1586	0.1873	0.159	0.1873	0.1709	0.1457	0.19	0.1457	0.1275	0.1641	0.1641	0.1873	0.149	0.148	0.2277	0.2063	0.1536	0.1536	0.1857	0.2277	0.1867	0.2277	0.1873	0.2277	0.2039	0.1691	0.2318	0.1691	0.1437	0.1943	0.1943	0.2277	0.1735	0.1721	0.2039	0.1866	0.1424	0.1424
291	-1.0762	-1.3692	-3.2469	-1.6161	-2.8568	-4.6787	-3.13	-2.7415	-1.4663	-0.3983	-1.174	-3.7363	-3.8264	-5.6162	-5.2261	-2.5135	-4.1248	-3.7904	-2.7101	-4.344	-2.7101	-3.0031	-4.8808	-3.25	-4.4907	-6.3126	-4.7639	-4.3754	-3.1002	-2.0322	-2.8079	-2.1024	-2.1925	-3.9823	-3.5922	-0.8796	-2.4909	-2.1565	-1.0762	-2.7101	-1.0762	-1.3692	-3.2469	-1.6161	-2.8568	-4.6787	-3.13	-2.7415	-1.4663	-0.3983	-1.174	-2.3954	-2.4855	-4.2753	-3.8852
292	93.591	93.591	126.49	75.581	126.49	176.17	118.98	118.98	93.591	93.591	118.98	126.49	126.49	169.17	169.17	126.49	126.49	126.49	126.49	159.39	126.49	126.49	159.39	108.48	159.39	209.07	151.87	151.87	126.49	126.49	151.87	93.591	93.591	136.27	136.27	93.591	93.591	93.591	93.591	126.49	93.591	93.591	126.49	75.581	126.49	176.17	118.98	118.98	93.591	93.591	118.98	93.591	93.591	136.27	136.27
293	6.7228	6.7228	11.982	9.8471	11.982	8.6219	6.7228	6.7228	6.7228	6.7228	6.7228	6.7228	6.7228	8.1868	8.1868	6.7228	6.7228	23.508	6.7228	6.7228	6.7228	6.7228	11.982	9.8471	11.982	8.6219	6.7228	6.7228	6.7228	6.7228	6.7228	6.7228	6.7228	8.1868	8.1868	6.7228	6.7228	23.508	6.7228	6.7228	6.7228	6.7228	11.982	9.8471	11.982	8.6219	6.7228	6.7228	6.7228	6.7228	6.7228	6.7228	6.7228	8.1868	8.1868
294	55.688	55.688	79.551	55.688	79.551	55.688	55.688	62.157	55.688	72.474	55.688	55.688	55.688	79.551	79.551	55.688	51.706	72.474	55.688	55.688	55.688	55.688	79.551	55.688	79.551	55.688	55.688	62.157	55.688	72.474	55.688	55.688	55.688	79.551	79.551	55.688	51.706	72.474	55.688	55.688	55.688	55.688	79.551	55.688	79.551	55.688	55.688	62.157	55.688	72.474	55.688	55.688	55.688	79.551	79.551
295	0	17.234	0	18.44	0	18.44	20.926	0	0	0	0	18.44	35.673	18.44	18.44	18.44	36.879	18.44	18.44	36.879	18.44	35.673	18.44	36.879	18.44	36.879	39.366	18.44	18.44	18.44	18.44	0	17.234	0	0	0	18.44	0	0	18.44	0	17.234	0	18.44	0	18.44	20.926	0	0	0	0	17.234	34.467	17.234	17.234
296	8.8216	4.4108	4.4108	4.4108	4.4108	4.4108	4.4108	4.4108	8.8216	7.5964	7.5964	0	0	0	0	3.1856	0	2.7567	4.4108	0	4.4108	0	0	0	0	0	0	0	4.4108	3.1856	3.1856	4.4108	4.4108	4.4108	4.4108	7.5964	4.4108	7.1675	8.8216	4.4108	8.8216	4.4108	4.4108	4.4108	4.4108	4.4108	4.4108	4.4108	8.8216	7.5964	7.5964	0	0	0	0
297	0	31.691	0	0	0	0	0	0	0	18.868	18.868	0	0	0	0	18.868	0	18.868	0	0	0	31.691	0	0	0	0	0	0	0	18.868	18.868	0	0	0	0	18.868	0	18.868	0	0	0	31.691	0	0	0	0	0	0	0	18.868	18.868	31.691	31.691	31.691	31.691
298	0	0	0	0	0	0	0	0	0	92.504	70.573	0	0	0	0	88.216	0	34	0	0	0	0	0	0	0	0	0	0	0	92.504	70.573	0	0	0	0	88.216	0	34	0	0	0	0	0	0	0	0	0	0	0	92.504	70.573	0	0	0	0
299	37.737	37.737	37.737	56.605	56.605	56.605	18.868	18.868	18.868	18.868	18.868	56.605	102.71	75.474	94.342	56.605	56.605	56.605	75.474	113.21	75.474	75.474	75.474	94.342	94.342	94.342	56.605	56.605	56.605	56.605	56.605	18.868	64.976	37.737	56.605	18.868	18.868	18.868	37.737	75.474	37.737	37.737	37.737	56.605	56.605	56.605	18.868	18.868	18.868	18.868	18.868	18.868	64.976	37.737	56.605
300	133.3	98.872	66.652	66.652	66.652	66.652	66.652	99.978	133.3	66.652	71.895	33.326	0	0	0	0	0	0	66.652	0	66.652	32.22	0	0	0	0	0	33.326	66.652	0	5.2434	99.978	66.652	66.652	66.652	66.652	66.652	66.652	133.3	66.652	133.3	98.872	66.652	66.652	66.652	66.652	66.652	99.978	133.3	66.652	71.895	65.546	32.22	32.22	32.22
301	6.2524	6.6069	5.688	5.5873	6.1497	7.1425	5.0155	5.475	5.7907	8.5071	7.488	5.0894	5.9074	5.4844	5.9461	7.5366	4.6299	6.7512	6.4675	6.6826	6.4675	6.822	5.9031	5.8024	6.3648	7.3576	5.2306	5.6901	6.0058	8.7223	7.7031	4.8743	5.6923	5.2693	5.731	7.3215	4.4148	6.536	6.2524	6.4675	6.2524	6.6069	5.688	5.5873	6.1497	7.1425	5.0155	5.475	5.7907	8.5071	7.488	5.2288	6.0468	5.6238	6.0855
302	90.408	90.408	114.27	90.408	114.27	90.408	90.408	90.408	90.408	90.408	90.408	90.408	90.408	156.95	156.95	90.408	90.408	90.408	90.408	90.408	90.408	90.408	114.27	90.408	114.27	90.408	90.408	90.408	90.408	90.408	90.408	90.408	90.408	156.95	156.95	90.408	90.408	90.408	90.408	90.408	90.408	90.408	114.27	90.408	114.27	90.408	90.408	90.408	90.408	90.408	90.408	90.408	90.408	156.95	156.95
303	0	0	0	3.1243	0	31.672	25.385	25.385	0	0	25.385	0	0	0	0	0	0	16.786	0	0	0	0	0	3.1243	0	31.672	25.385	25.385	0	0	25.385	0	0	0	0	0	0	16.786	0	0	0	0	0	3.1243	0	31.672	25.385	25.385	0	0	25.385	0	0	0	0
304	59.73	55.319	55.319	37.308	55.319	73.329	55.319	55.319	59.73	72.104	55.319	83.805	83.805	83.805	83.805	83.805	83.805	100.59	88.216	116.7	88.216	83.805	83.805	65.794	83.805	101.82	83.805	83.805	88.216	100.59	83.805	55.319	55.319	55.319	55.319	55.319	55.319	72.104	59.73	88.216	59.73	55.319	55.319	37.308	55.319	73.329	55.319	55.319	59.73	72.104	55.319	50.908	50.908	50.908	50.908
305	7.9639	7.9639	7.9639	7.9639	7.9639	7.9639	7.9639	14.433	7.9639	11.15	16.393	7.9639	7.9639	7.9639	7.9639	11.15	3.982	10.721	7.9639	7.9639	7.9639	7.9639	7.9639	7.9639	7.9639	7.9639	7.9639	14.433	7.9639	11.15	16.393	7.9639	7.9639	7.9639	7.9639	11.15	3.982	10.721	7.9639	7.9639	7.9639	7.9639	7.9639	7.9639	7.9639	7.9639	7.9639	14.433	7.9639	11.15	16.393	7.9639	7.9639	7.9639	7.9639
306	0	0	32.897	0	32.897	32.897	0	0	0	4.7171	0	0	0	0	0	0	0	0	0	0	0	0	32.897	0	32.897	32.897	0	0	0	4.7171	0	0	0	0	0	0	0	0	0	0	0	0	32.897	0	32.897	32.897	0	0	0	4.7171	0	0	0	0	0
307	37.737	37.737	37.737	56.605	56.605	56.605	18.868	18.868	18.868	125.52	108.31	56.605	56.605	75.474	94.342	163.69	56.605	109.47	75.474	113.21	75.474	75.474	75.474	94.342	94.342	94.342	56.605	56.605	56.605	163.26	146.05	18.868	18.868	37.737	56.605	125.95	18.868	71.737	37.737	75.474	37.737	37.737	37.737	56.605	56.605	56.605	18.868	18.868	18.868	125.52	108.31	18.868	18.868	37.737	56.605
308	6.7228	23.956	11.982	25.162	11.982	27.061	27.649	6.7228	6.7228	6.7228	6.7228	25.162	42.396	26.626	26.626	25.162	43.602	25.162	25.162	43.602	25.162	42.396	30.421	43.602	30.421	45.501	46.089	25.162	25.162	25.162	25.162	6.7228	23.956	8.1868	8.1868	6.7228	25.162	6.7228	6.7228	25.162	6.7228	23.956	11.982	25.162	11.982	27.061	27.649	6.7228	6.7228	6.7228	6.7228	23.956	41.19	25.42	25.42
309	133.3	130.56	66.652	66.652	66.652	66.652	66.652	99.978	133.3	66.652	66.652	33.326	46.107	0	0	0	0	0	66.652	0	66.652	63.911	0	0	0	0	0	33.326	66.652	0	0	99.978	112.76	66.652	66.652	66.652	66.652	66.652	133.3	66.652	133.3	130.56	66.652	66.652	66.652	66.652	66.652	99.978	133.3	66.652	66.652	97.237	110.02	63.911	63.911
310	96.87	96.87	139.96	88.08	139.96	160.51	117.1	117.1	96.87	112.66	117.1	124.51	163.31	164.64	164.64	124.51	124.51	153.19	124.51	152.15	124.51	124.51	167.6	115.72	167.6	188.15	144.74	144.74	124.51	140.3	144.74	96.87	135.67	137	137	96.87	96.87	125.55	96.87	124.51	96.87	96.87	139.96	88.08	139.96	160.51	117.1	117.1	96.87	112.66	117.1	96.87	135.67	137	137
311	0.5021	0.5021	0.5021	0.5436	0.4845	0.4529	0.542	0.5212	0.5212	0.4389	0.4537	0.542	0.5212	0.4845	0.4681	0.4537	0.5647	0.469	0.4845	0.4681	0.4845	0.4845	0.4845	0.5226	0.4681	0.4388	0.5212	0.5021	0.5021	0.4257	0.4395	0.5647	0.542	0.5021	0.4845	0.469	0.5895	0.4855	0.5021	0.4845	0.5021	0.5021	0.5021	0.5436	0.4845	0.4529	0.542	0.5212	0.5212	0.4389	0.4537	0.5647	0.542	0.5021	0.4845
312	5	5	5	5	5.0875	5.2479	4.8074	4.9069	4.9069	5.585	5.3923	4.8074	4.9069	5.0875	5.1699	5.3923	4.7004	5.3219	5.0875	5.1699	5.0875	5.0875	5.0875	5.0875	5.1699	5.3219	4.9069	5	5	5.6439	5.4594	4.7004	4.8074	5	5.0875	5.3219	4.585	5.2479	5	5.0875	5	5	5	5	5.0875	5.2479	4.8074	4.9069	4.9069	5.585	5.3923	4.7004	4.8074	5	5.0875
313	2.997	3.0645	2.997	2.7992	3.1249	3.3775	2.8628	2.8826	2.8826	3.3311	3.3057	3.1124	3.1416	3.2438	3.3394	3.4113	3.1063	3.3484	3.2122	3.3849	3.2438	3.292	3.2438	3.0934	3.3394	3.54	3.1416	3.1575	3.1575	3.5102	3.4847	2.8758	2.9058	3.0396	3.1568	3.2484	2.8693	3.1686	2.9996	3.2205	3.0396	3.1044	3.0396	2.8527	3.1568	3.3998	2.9058	2.92	2.92	3.3633	3.3366	2.9643	2.9963	3.1122	3.22
314	7.8881	7.8812	7.8881	7.7182	8.0483	8.3366	7.5168	7.713	7.713	8.7609	8.4907	7.479	7.6768	8.0301	8.186	8.4789	7.2664	8.3366	8.0326	8.1816	8.0301	8.0263	8.0301	7.8612	8.186	8.4674	7.6768	7.8608	7.8608	8.8678	8.6102	7.2974	7.5087	7.8816	8.0431	8.3507	7.0665	8.2025	7.886	8.0365	7.8816	7.8754	7.8816	7.7091	8.0431	8.3336	7.5087	7.7051	7.7051	8.7568	8.4867	7.2869	7.4989	7.8736	8.0368
315	288.07	293.21	271.12	250.92	288.35	331.89	245.06	262.29	270.84	327.68	315.56	250.83	272.63	278.36	295.59	321.32	233.59	304.17	303.87	319.66	303.87	309	286.92	266.71	304.15	347.68	260.86	278.09	286.63	343.48	331.35	235.03	256.84	262.56	279.79	305.53	217.8	288.37	288.07	303.87	288.07	293.21	271.12	250.92	288.35	331.89	245.06	262.29	270.84	327.68	315.56	240.16	261.97	267.7	284.93
316	348.38	346.33	315.37	311.67	339.8	391.06	283.61	308.04	323.95	435.41	398.58	293.64	314.97	323.38	347.81	408.62	269.22	372.87	366.93	385.49	366.93	364.89	333.92	330.22	358.35	409.61	302.17	326.6	342.5	453.97	417.14	275.09	296.41	304.83	329.26	390.06	250.66	354.32	348.38	366.93	348.38	346.33	315.37	311.67	339.8	391.06	283.61	308.04	323.95	435.41	398.58	273.04	294.36	302.78	327.21
317	40.701	40.701	54.268	40.701	54.268	40.701	40.701	40.701	40.701	40.701	40.701	40.701	40.701	67.835	67.835	40.701	40.701	40.701	40.701	40.701	40.701	40.701	54.268	40.701	54.268	40.701	40.701	40.701	40.701	40.701	40.701	40.701	40.701	67.835	67.835	40.701	40.701	40.701	40.701	40.701	40.701	40.701	54.268	40.701	54.268	40.701	40.701	40.701	40.701	40.701	40.701	40.701	40.701	67.835	67.835
318	27.134	27.134	27.134	27.134	27.134	27.134	27.134	27.134	27.134	27.134	27.134	27.134	27.134	54.268	54.268	27.134	27.134	27.134	27.134	27.134	27.134	27.134	27.134	27.134	27.134	27.134	27.134	27.134	27.134	27.134	27.134	27.134	27.134	54.268	54.268	27.134	27.134	27.134	27.134	27.134	27.134	27.134	27.134	27.134	27.134	27.134	27.134	27.134	27.134	27.134	27.134	27.134	27.134	54.268	54.268
319	17.742	17.742	17.742	17.742	17.742	58.91	17.742	17.742	17.742	17.742	17.742	35.485	35.485	35.485	35.485	35.485	35.485	35.485	35.485	53.227	35.485	35.485	35.485	35.485	35.485	76.653	35.485	35.485	35.485	35.485	35.485	17.742	17.742	17.742	17.742	17.742	17.742	17.742	17.742	35.485	17.742	17.742	17.742	17.742	17.742	58.91	17.742	17.742	17.742	17.742	17.742	17.742	17.742	17.742	17.742
320	23.425	23.425	41.168	17.742	41.168	64.593	23.425	23.425	23.425	29.108	23.425	41.168	41.168	41.168	41.168	41.168	41.168	41.168	41.168	58.91	41.168	41.168	58.91	35.485	58.91	82.335	41.168	41.168	41.168	46.85	41.168	23.425	23.425	23.425	23.425	23.425	23.425	23.425	23.425	41.168	23.425	23.425	41.168	17.742	41.168	64.593	23.425	23.425	23.425	29.108	23.425	23.425	23.425	23.425	23.425
321	167.07	174.97	106.04	142.15	121.85	142.15	112.29	128.1	151.26	200.69	179.94	110.53	98.909	96.933	112.74	178.18	94.724	123.93	157.96	148.85	157.96	165.86	96.933	133.04	112.74	133.04	103.18	118.99	142.15	191.58	170.83	119.64	108.02	106.04	121.85	187.29	103.83	133.04	167.07	157.96	167.07	174.97	106.04	142.15	121.85	142.15	112.29	128.1	151.26	200.69	179.94	127.55	115.92	113.95	129.75
322	27.658	27.658	40.608	27.658	40.608	43.34	27.658	27.658	27.658	27.658	27.658	27.658	59.043	42.367	42.367	27.658	27.658	52.449	27.658	27.658	27.658	27.658	40.608	27.658	40.608	43.34	27.658	27.658	27.658	27.658	27.658	27.658	59.043	42.367	42.367	27.658	27.658	52.449	27.658	27.658	27.658	27.658	40.608	27.658	40.608	43.34	27.658	27.658	27.658	27.658	27.658	27.658	59.043	42.367	42.367
323	64.126	64.126	95.435	58.443	95.435	105.29	77.693	77.693	64.126	69.808	77.693	81.868	81.868	109	109	81.868	81.868	93.233	81.868	99.611	81.868	81.868	113.18	76.186	113.18	123.04	95.435	95.435	81.868	87.551	95.435	64.126	64.126	91.26	91.26	64.126	64.126	75.491	64.126	81.868	64.126	64.126	95.435	58.443	95.435	105.29	77.693	77.693	64.126	69.808	77.693	64.126	64.126	91.26	91.26
324	244.33	262.37	245.28	228.29	259.31	288.37	218.25	232.28	230.31	317.39	294.35	218.28	250.34	261.28	275.3	294.38	204.25	284.34	260.36	276.38	260.36	278.4	261.3	244.32	275.33	304.39	234.28	248.3	246.33	333.41	310.37	202.25	234.32	245.25	259.28	278.35	188.23	268.32	244.33	260.36	244.33	262.37	245.28	228.29	259.31	288.37	218.25	232.28	230.31	317.39	294.35	220.29	252.36	263.29	277.32
325	556	570	556	434	668	947	394	461	461	1245	1008	436	513	701	828	1060	374	926	688	844	701	716	701	562	828	1140	513	592	592	1478	1212	338	404	568	681	887	286	767	556	696	568	582	568	445	681	962	404	472	472	1263	1024	349	416	582	696
326	22	22	22	23	23	25	20	22	22	34	29	17	19	21	22	26	14	23	23	22	21	21	21	22	22	24	19	21	21	33	28	16	18	20	21	25	13	22	22	21	20	20	20	21	21	23	18	20	20	32	27	16	18	20	21
327	74	72	74	76	78	86	64	70	70	114	98	62	66	76	80	94	56	90	76	78	76	74	76	78	80	88	66	72	72	116	100	60	64	74	78	92	54	88	74	76	74	72	74	76	78	86	64	70	70	114	98	58	62	72	76
328	3.3773	-4.3175	3.9277	2.553	-3.4536	2.7596	4.0629	4.7043	-2.9905	0.9937	-3.3743	8.2752	4.7421	14.714	4.4362	3.1263	-2.0934	8.4058	7.7419	-1.6118	5.7674	6.1786	9.5636	7.449	11.415	4.7517	9.7363	9.4255	9.2649	11.61	5.1204	3.1158	-4	-6.3803	4.1507	-2.2118	-4.7189	-3.6809	1.7315	0.3166	4.7418	-3.6716	-2.782	4.7468	2.5814	0.2188	3.0143	5.1041	5.1195	2.4066	-6.0134	2.6214	4.4757	5.7816	3.7563
329	3.288	3.4888	-3.2145	-1.5446	-0.2252	0.135	3.8064	2.764	-0.8526	-4.5171	1.7392	0.0306	1.2487	-2.9741	2.4994	0.7202	1.0363	2.38	-1.2515	-2.8012	-0.8094	1.3221	-0.6198	-2.1304	-2.2207	1.2417	0.7496	-2.4085	0.1955	-4.2651	0.3188	-0.7988	-1.9207	0.1482	2.8333	1.4658	0.4488	-3.8484	2.0395	2.2819	-3.5684	4.4301	-0.5431	0.2317	0.2515	-5.3282	-0.0913	2.9802	3.0389	-2.4291	-1.4676	0.1009	0.6675	-0.0511	1.9914
330	2.206	1.5716	-2.3344	1.5085	1.0126	3.6809	0.0498	-1.1715	-0.5586	-1.491	1.301	1.2169	-1.1828	-1.1166	-0.5298	-0.7644	-0.2169	0.301	1.7935	1.5949	-0.0586	-0.3261	-0.916	0.9489	-1.43	-3.1881	-2.4084	0.0162	1.3723	-1.0216	-0.1062	-1.0472	-2.6054	-0.4871	0.0037	-0.784	1.4737	-1.5598	-2.3079	0.9342	-0.5855	0.296	-0.7136	1.5422	0.2927	-1.3104	-1.3141	-0.5037	-0.6589	-0.5032	-0.6876	1.1761	-2.4163	-0.0032	0.5542
331	0	0	0	0	0	0	0	0	0	0	0	0	0	0	-6E-14	-3E-14	0	0	0	0	0	1E-13	0	0	-1E-13	-6E-14	0	0	0	-3E-13	0	0	0	0	0	-1E-14	0	0	0	9E-14	-1E-14	0	0	0	0	0	0	0	0	0	0	0	0	0	0
332	0	0	0	0	0	0	0	0	0	0	0	0	0	0	-6E-14	-3E-14	0	0	0	0	0	1E-13	0	0	-1E-13	-6E-14	0	0	0	-3E-13	0	0	0	0	0	-1E-14	0	0	0	9E-14	-1E-14	0	0	0	0	0	0	0	0	0	0	0	0	0	0
333	1608.2	2590.7	1633.5	1331.1	2832	2698.6	1170.9	1523.6	1160	2156.4	3510.1	2140.3	1197.7	3201.2	1419.2	2670.8	862.67	2440.5	2361.2	4029.3	1120.7	2075.4	2523.8	2319.8	3726.8	5187.8	2167	1920.2	2908.4	5673	3097.8	1354	1422.7	2005.9	1502.7	2322.8	1370.7	1935.4	1831.8	2740.3	2238.1	2352.1	1433.9	1483.1	2093.1	3005.1	1670	1954.5	1752	3537.5	1702	2154.8	2520.8	2766.2	2478.7
334	559.7	581.24	376.59	427.03	320.6	505.9	398.82	322.09	387.52	1265.3	638.1	252.19	718.07	505.19	888.12	601.1	348.82	1253	544.72	557.82	747.56	839.44	573.21	344.01	604.8	496.94	493.25	679.74	368.87	930.86	804.57	273.44	537.77	333.68	791.74	772.55	269.71	711.04	361	583.55	547.45	695.79	538.49	352.18	624.79	752.12	319.62	428.01	439.42	836.64	1320.9	210.88	365.71	381.21	656.37
335	272.59	172.12	298.11	124.19	186.03	362.28	224.46	244	338	409.13	162.95	127.79	140.01	137.38	142.57	280.26	157.27	150.82	266.6	128.23	377.06	295.32	281.26	178.59	111.14	176.84	196.68	288.31	98.539	261.3	247.09	68.671	227.11	248.18	200.57	271.52	79.08	196.76	190.08	141.45	87.65	134.13	209.8	174.56	203.77	342.68	114.9	152.95	175.93	375.28	271.52	107.83	163.9	205.63	291.09
336	20.661	22.966	18.098	7.0103	5.1889	7.7849	20.445	20.389	3.8559	15.016	6.9759	26.373	13.604	23.688	5.5481	13.466	11.655	28.845	20.379	13.065	13.462	16.88	39.351	5.1849	27.352	17.736	18.754	14.456	32.759	50.366	12.129	5.53	17.939	13.969	12.447	5.3644	14.126	19.619	6.3384	4.6639	23.099	21.339	2.8812	13.074	4.2105	8.1846	5.4108	23.137	19.886	8.0496	23.203	5.0034	19.195	15.246	8.6671
337	-74758	-75648	-79426	-70578	-83080	-89618	-71383	-74975	-71225	-93943	-90408	-69295	-73780	-86815	-90477	-88290	-65716	-88311	-80079	-85307	-80079	-80975	-84670	-75857	-88291	-94858	-76672	-80287	-76475	-99177	-95689	-64040	-68470	-81503	-85101	-83015	-60413	-82986	-74812	-80047	-74757	-75650	-79487	-70535	-83074	-89603	-71435	-74973	-71171	-93992	-90350	-64931	-69353	-82398	-85990
338	-502793	-488927	-500507	-451589	-524425	-661583	-425552	-470842	-462403	-708345	-621838	-402142	-472356	-541101	-610224	-647900	-392252	-596726	-561749	-580485	-556690	-556188	-531548	-514597	-585718	-669126	-481720	-521029	-487883	-718070	-682234	-369099	-412623	-491966	-534119	-579202	-332350	-563264	-503081	-548391	-494757	-491960	-498922	-458133	-530107	-640855	-409613	-454030	-458073	-681980	-641809	-354372	-403247	-471625	-521429
339	-106.91	-97.426	-134.21	-145.28	-201.75	3.4639	-143.48	-149.36	-161.8	-42.798	-168.38	5.5275	27.338	-173.56	-248.98	11.203	-1.7516	51.183	-15.745	163.7	-17.245	-13.167	42.966	-13.552	-1.4851	173.87	-21.694	-50.901	19.84	133.38	-38.226	-150.38	-83.821	-273.75	-284.32	-124.52	-108.54	-34.382	-155.77	13.536	-105.34	-98.605	-195.94	-101.73	-195.64	18.739	-196.39	-148.25	-102.38	-78.159	-110.46	-140.02	-74.209	-267.23	-272.04
340	-7.6973	-7.7308	-8.4919	-9.9531	-10.342	-12.509	-7.762	-8.106	-9.9557	-7.992	-9.0706	-12.083	-11.874	-7.5332	-9.8515	-11.772	-13.199	-11.12	-12.552	-14.603	-12.648	-10.676	-10.321	-12.951	-10.759	-14.809	-11.755	-12.376	-12.17	-8.7928	-11.338	-10.261	-8.1183	-5.3018	-5.1423	-9.6601	-8.4325	-7.2913	-9.8147	-10.696	-7.5564	-7.6851	-10.222	-8.2247	-9.8676	-11.597	-10.234	-7.7258	-7.6369	-8.3114	-7.7294	-8.7969	-8.0972	-5.4053	-5.0262
341	7.6973	7.7308	8.4919	9.9531	10.342	12.509	7.762	8.106	9.9557	7.992	9.0706	12.083	11.874	7.5332	9.8515	11.772	13.199	11.12	12.552	14.603	12.648	10.676	10.321	12.951	10.759	14.809	11.755	12.376	12.17	8.7928	11.338	10.261	8.1183	5.3018	5.1423	9.6601	8.4325	7.2913	9.8147	10.696	7.5564	7.6851	10.222	8.2247	9.8676	11.597	10.234	7.7258	7.6369	8.3114	7.7294	8.7969	8.0972	5.4053	5.0262
342	-1.4835	-1.4166	-1.5148	0.9298	0.5653	-3.2408	-1.2311	-1.2878	0.9356	-0.7417	0.2624	-4.2885	-4.1998	-1.1952	0.6911	-3.2879	-3.4388	-4.1268	-3.9139	-5.4651	-4.1433	-3.4472	-4.9002	-2.8224	-3.6252	-5.6103	-3.8946	-4.1769	-4.4223	-4.9158	-3.0709	0.8245	-1.0966	4.0073	4.083	0.1621	-0.4893	-0.5176	0.9434	-3.2583	-1.2247	-1.3577	0.7463	-0.1036	0.9184	-3.56	0.7145	-1.3747	-1.0318	0.3153	-1.3762	0.5223	-1.2959	2.4673	2.9216
343	485.97	505.27	445.93	430.4	479.05	513	424.5	435.8	449.92	544.29	526.75	449.33	437.83	495.2	444.85	521.37	380.42	535.64	507.96	554.86	469.08	506.4	495.96	475.93	524.98	577.78	465.4	490.02	495.75	623.31	533.32	411.41	448.42	443.86	454.32	517.12	400.35	483.27	470.57	514.12	486.92	500.11	439.03	440.81	486.08	545.03	428.84	467.1	462.75	574.87	504.23	427.93	454.48	458.69	479.97
344	67.833	112.11	117.87	55.324	120.74	198.6	118.13	87.445	62.806	184.28	172.86	166.28	157.63	192.27	124.42	223.24	152.66	277.13	155.13	240.06	139.42	158.26	214.26	145.31	217.73	275.4	194.13	179.43	156.33	285.28	239.77	83.426	89.276	92.462	72.536	176.65	82.648	163.18	56.351	153.27	74.325	102.41	120.33	74.952	144.95	207.82	117.66	84.361	67.608	197.43	167.77	110.61	127.05	116.11	125.78
345	77.474	116.23	130.14	66.3	100.36	60.912	95.398	102.82	66.729	160.75	147.77	67.225	96.394	146.03	101.81	130.72	64.316	105.29	48.824	50.76	54.736	96.467	126.44	68.742	110.53	64.991	99.047	101.47	57.946	169.95	139.94	63.348	137.72	147.29	138.04	147.04	99.71	94.278	57.179	67.415	63.564	121.44	103.01	73.616	103.54	93.88	91.401	103.98	75.313	171.7	147.08	117.84	168.82	185.92	177.35
346	340.66	277.04	202.63	309.06	263.45	254.01	210.97	245.54	320.39	362.74	352.83	216.38	193.86	161.11	227.03	353.44	173.01	235.79	304	264.6	275.49	251.96	160.36	262.72	204.06	238.24	172.97	210.43	281.48	347.92	292.95	265.93	226.3	208.75	251.78	372.13	224.27	296.23	357.04	293.43	349.88	276.26	223.4	292.36	242.61	243.9	220.34	279.05	320.4	389.16	302.97	200.01	173.04	164.19	184.18
347	145.31	228.22	243.3	121.34	215.6	258.99	213.53	190.27	129.53	181.55	173.92	232.95	243.97	334.09	217.82	167.93	207.42	299.85	203.96	290.26	193.59	254.44	335.6	213.21	320.92	339.54	292.43	279.59	214.27	275.39	240.38	145.48	222.11	235.1	202.54	145	176.09	187.03	113.53	220.68	137.04	223.85	215.63	148.45	243.47	301.12	208.49	188.06	142.35	185.71	201.25	227.92	281.44	294.51	295.79
348	268.14	494.75	627.44	214.82	642.68	1535.4	547.31	405.13	248.27	955.88	903.91	965.4	979.83	1290.5	835.09	1495.7	886.34	1849.3	900.71	1838.6	809.49	991.66	1537.5	833.5	1562.4	2639.4	1259.1	1163.8	907.62	2008.4	1698.1	329.78	389.51	449.27	352.45	856.24	326.71	786.51	222.76	889.87	293.81	451.93	640.52	291.04	771.55	1606.6	545.13	390.84	267.26	1024.1	877.26	488.11	612.74	617.56	669
349	306.26	512.94	692.75	257.44	534.23	410	441.98	476.37	263.78	833.95	772.7	323.08	502.79	980.13	683.38	745.13	309.1	597.29	234.65	287.25	263.06	508	780.87	325.56	682.64	492.89	543.37	556.66	278.49	1026.7	851.09	250.41	600.88	862.96	808.78	712.68	394.15	454.42	226.03	324	251.27	535.92	548.32	285.85	551.12	631.91	423.46	481.73	297.71	890.8	769.08	520.03	814.23	1174.8	1120.7
350	9.6414	4.1219	12.269	10.976	20.374	137.69	22.735	15.376	3.9235	23.538	25.091	99.051	61.237	46.247	22.602	92.518	88.343	171.85	106.31	189.3	84.688	61.793	87.824	76.569	107.2	210.41	95.082	77.964	98.379	115.33	99.836	20.078	48.446	54.826	65.505	29.619	17.062	68.897	0.8277	85.852	10.761	19.033	17.322	1.3362	41.411	113.94	26.261	19.618	7.7042	25.725	20.688	7.2328	41.778	69.817	51.57
351	38.112	18.19	65.31	42.62	108.45	1125.4	105.33	71.235	15.51	121.93	131.2	642.32	477.05	310.41	151.71	750.6	577.23	1252	666.06	1551.3	546.43	483.66	756.66	507.94	879.78	2146.5	715.75	607.15	629.13	981.71	846.97	79.367	211.37	413.69	456.33	143.56	67.446	332.08	3.2718	565.87	42.538	83.994	92.206	5.1885	220.43	974.74	121.67	90.889	30.455	133.26	108.18	31.919	201.49	557.28	451.65
352	0.9484	1.0091	1.046	1.0068	1.0419	1.0083	1.0307	1.0255	0.9728	1.0036	1.0247	1.007	1.0721	1.0802	1.0996	1.0077	1.0468	1.0396	0.963	0.9719	0.9747	1.0254	1.0505	1.0101	1.0429	1.0232	1.0395	1.0351	0.9785	1.0088	1.035	1.0013	1.0508	1.0961	1.0731	0.9901	1.0133	1.037	0.9624	0.9541	0.9447	1.0024	1.0705	0.9947	1.031	0.9974	1.0499	1.0244	0.9636	1.0148	1.0172	1.0812	1.1197	1.1695	1.1319
353	5.2042	5.769	5.5865	3.3435	3.606	4.6025	5.5676	5.5805	3.1595	4.8595	4.0129	8.3642	5.0444	15.053	5.1194	3.298	2.3459	8.7414	8.0448	3.6039	5.8242	6.3268	9.6273	7.8055	11.717	5.8553	10.058	9.7284	9.368	12.41	5.1314	3.3827	5.1456	6.4006	5.0256	2.7668	4.964	5.5491	3.5333	2.4859	5.9633	5.7615	2.923	4.9964	2.6101	5.4914	3.2896	5.9319	5.9898	3.4562	6.228	2.8749	5.1299	5.7818	4.2875
354	39.704	33.599	36.091	21.581	-7.6337	-55.216	36.667	35.18	19.701	49.778	33.103	14.857	10.863	46.152	-53.535	-0.4101	-34.566	-2.59	18.984	9.0261	11.889	0.4114	30.65	18.249	19.072	-75.34	37.306	29.483	21.113	54.908	14.865	10.088	33.738	6.2178	0.1648	21.168	30.492	32.055	25.092	28.623	40.148	35.397	-6.1948	43.607	3.5031	-40.706	12.085	32.734	37.24	27.884	48.629	8.6487	34.653	0.1984	-5.2254
355	8.9799	7.5914	15.544	12.794	7.9923	18.276	5.0413	11.713	11.338	10.189	8.2403	6.148	17.159	8.8007	16.178	7.9281	19.906	9.6199	10.016	7.8622	20.934	19.019	10.986	12.533	10.355	13.133	7.1774	8.073	7.8187	8.3565	13.86	5.8162	8.3913	15.507	9.0913	12.064	7.6056	9.2385	12.652	8.6411	11.513	8.7885	9.8998	13.535	8.7851	11.447	5.9785	5.3616	9.8816	7.2708	10.983	4.8418	7.138	7.996	11.345
356	7.4431	1.5081	-5.9145	-54.579	-90.916	-124.22	10.839	-7.7057	-53.485	4.8436	-56.366	-48.271	-55.157	17.342	-156.58	-114.59	-122.94	-59.611	-52.43	-57.172	-55.492	-81.785	-5.3638	-52.455	-29.429	-172.6	1.6373	-2.1108	-48.545	14.203	-69.549	-52.35	-0.0998	-65.687	-64.425	-57.591	7.1417	-4.7759	-50.556	-1.6864	3.4619	4.9122	-87.571	7.4707	-68.59	-72.428	-51.968	-4.4194	5.1778	-53.036	2.4312	-53.627	3.3751	-79.545	-70.649
357	29.618	22.42	11	-12.908	-34.981	-81.194	27.437	18.061	-8.0449	34.321	3.4184	-5.2354	-12.883	36.073	-81.751	-36.933	-60.482	-16.788	-5.7471	-12.544	-11.777	-27.853	14.143	-11.104	2.8505	-107.86	27.765	19.246	-1.8358	44.05	-13.008	-12.188	20.51	-20.327	-18.246	-0.6396	21.825	16.329	-4.3421	20.734	26.717	24.25	-37.95	25.405	-23.729	-50.174	-10.767	22.859	26.532	1.8018	34.23	-15.437	20.098	-29.381	-27.91
358	0.1927	0.0513	0.2198	1.6151	1.9691	0.5691	0.0665	0.0763	1.9355	0.3327	1.177	1.1649	0.0531	0.4658	0.3616	1.589	1.338	0.0834	0.8668	1.4093	0.0686	0.3038	0.2683	0.7055	0.1352	2.8222	0.2197	0.1003	1.7157	0.0699	0.9026	1.5937	0.1084	0.3389	0.1077	0.1924	0.0627	1.0351	0.6805	0.3374	0.0913	0.1184	2.1994	0.2137	1.7153	0.084	1.6643	0.1124	0.14	1.9323	0.1899	1.7283	0.2181	2.2339	0.1562
359	-94.057	-90.67	-111.83	-55.797	-57.205	-102.39	-103.89	-89.224	-56.887	-97.947	-71.159	-156.82	-129.07	-302.08	-92.001	-82.582	-84.438	-143.85	-129.15	-182.2	-130.49	-124.84	-195.29	-130.95	-187.42	-186.65	-195.77	-176.21	-160.9	-217.68	-106.76	-60.041	-94.787	-146.28	-134.37	-54.976	-93.661	-109.34	-62.126	-119.13	-93.353	-94.473	-54.115	-92.984	-59.694	-121.86	-64.03	-97.009	-95.263	-65.46	-102.24	-60.289	-93.913	-127.26	-123.98
360	0.2551	0.1013	0.307	0.1092	0.2566	0.3376	0.5426	0.3526	0.3823	-0.508	0.2292	0.0919	0.7887	0.2145	-0.2266	0.5697	0.2428	0.1358	0.6191	0.1837	0.2405	0.1601	0.022	0.025	0.1683	-0.2459	0.4937	0.0571	0.2869	-0.935	0.2126	0.1005	-0.0301	0.2067	0.3678	0.1224	0.2144	0.583	0.5725	0.7317	0.065	0.2955	0.1772	-0.0017	0.2237	0.0575	0.2368	0.2752	0.255	-0.6314	0.2678	0.0501	0.4919	0.2363	0.2797
361	4.2423	3.624	3.4787	6.0016	7.2535	7.3438	2.9123	4.3555	7.5046	5.5473	8.1824	5.1202	6.7186	2.8271	7.8856	9.0239	5.8097	5.5042	7.792	6.4779	6.1202	6.0603	2.9681	5.9247	4.3215	8.8041	3.8012	3.5875	6.4639	5.2279	6.5703	5.6317	3.2939	5.5058	5.2612	7.2069	2.255	3.4124	7.6576	3.4863	4.0924	3.1868	6.2964	3.1466	6.2335	4.4782	5.5712	4.087	3.7052	7.7971	4.368	5.1825	2.4806	6.1485	5.1312
362	0.0062	0.0156	0.0053	0.014	0.0054	0.0104	0.0044	0.0063	0.0168	0.0066	0.0124	0.0437	0.0046	0.0149	0.0035	0.0098	0.0024	0.0093	0.0193	2.4927	0.0059	0.0079	0.0081	0.0043	0.0096	0.0181	0.02	0.0157	0.0228	0.0083	0.0068	0.0074	-0.1033	0.0072	0.0878	0.0134	0.0032	0.0236	0.005	5.7397	0.0075	0.0076	0.0038	0.0078	0.0112	0.0059	3.7979	0.9198	0.0058	0.041	0.0146	0.0052	0.0047	0.0062	0.0029
363	-3.5845	-0.1886	5.5413	13.97	9.8755	-0.5491	0.6681	0.6216	6.5858	-0.1033	11.856	7.567	-0.9736	-2.2292	4.0171	17.413	-1.3808	-1.1459	5.4372	5.6372	-3.697	2.7207	2.2621	10.164	1.242	8.0054	-2.1513	-1.5805	6.6639	-1.8617	6.3262	9.1336	1.4645	4.9867	3.5827	2.2219	-1.4702	1.4567	7.8713	-5.3079	-2.3306	-1.2421	13.182	1.3089	10.274	-6.5976	9.401	0.0389	-3.2737	9.713	-1.41	12.283	4.2268	12.965	5.7727
364	22.175	20.911	16.915	41.671	55.936	43.024	16.598	25.767	45.44	29.477	59.785	43.035	42.274	18.731	74.83	77.657	62.46	42.823	46.683	44.627	43.714	53.932	19.507	41.351	32.279	64.744	26.128	21.357	46.709	29.847	56.542	40.162	20.61	45.36	46.179	56.952	14.683	21.105	46.214	22.42	23.255	19.338	49.621	17.934	44.861	22.254	41.201	27.278	21.354	54.837	31.799	38.19	16.723	50.165	42.739

Table S12 Amino acid descriptors (2D) used in this study.

	I	I	I	I	I	I	I	I	I	I	I	K	K	K	K	K	K	K	K	K	K	K	K	K	K	K	K	K
	L	M	N	P	Q	R	S	T	V	W	Y	A	C	D	E	F	G	H	I	K	L	M	N	P	Q	R	S	T
No.	IL	IM	IN	IP	IQ	IR	IS	IT	IV	IW	IY	KA	KC	KD	KE	KF	KG	KH	KI	KK	KL	KM	KN	KP	KQ	KR	KS	KT
365	0.1396	0.2219	0.2643	0.1285	0.252	0.3871	0.2783	0.2007	0.1396	0.3386	0.3282	0.3701	0.36	0.3883	0.2797	0.4282	0.4013	0.5174	0.3054	0.4326	0.2972	0.3125	0.432	0.3053	0.4147	0.4767	0.4171	0.3662
366	0.1594	0.23	0.2918	0.154	0.2095	0.1187	0.2247	0.2359	0.1483	0.2953	0.2805	0.1496	0.2202	0.2949	0.2289	0.2507	0.1691	0.1966	0.0961	0.0915	0.1167	0.1905	0.2549	0.1444	0.2105	0.1125	0.2128	0.2071
367	0.701	0.5483	0.4544	0.7181	0.5499	0.4951	0.497	0.5634	0.7121	0.6664	0.6698	0.4816	0.4428	0.3253	0.5103	0.6779	0.4548	0.4402	0.5985	0.4769	0.5873	0.4975	0.3233	0.552	0.3887	0.4123	0.3717	0.4294
368	0.299	0.4517	0.5456	0.2819	0.4501	0.5049	0.503	0.4366	0.2879	0.3336	0.3302	0.5184	0.5572	0.6747	0.4897	0.3221	0.5452	0.5598	0.4015	0.5231	0.4127	0.5025	0.6767	0.448	0.6113	0.5877	0.6283	0.5706
369	0.5518	0.9792	1.407	0.4991	1.3416	2.993	1.2893	0.9296	0.5518	1.7562	1.716	2.1485	2.2379	2.6061	1.8772	2.8689	2.3299	3.4525	1.7732	3.3136	1.7257	1.9583	3.1001	1.7513	2.9762	4.5683	2.7054	2.375
370	0.6302	1.0152	1.5535	0.5981	1.1152	0.7992	1.0412	1.0931	0.5863	1.5322	1.4669	0.719	1.1484	1.9793	1.5362	1.4292	0.8125	1.1151	0.4619	0.5177	0.5608	1.0032	1.5745	0.6841	1.3003	0.8531	1.1675	1.136
371	0.1952	0.0984	0.1943	0.1114	0.0703	0.1679	0.2017	0.1511	0.2245	0.2039	0.0632	0.0725	0.1883	0.0554	0.1463	0.1163	0.2344	0.0622	0.1211	0.0422	0.3404	0.2136	0.1106	0.0833	0.0486	0.0476	0.0991	0.1371
372	21.845	23.772	20.733	7.2728	7.8643	9.652	22.074	19.753	3.9192	18.618	5.8848	27.768	12.871	40.873	6.5318	6.087	12.929	31.412	22.591	10.923	13.949	21.27	40.471	27.667	31.737	18.413	14.108	13.846
373	-75027	-76703	-79688	-70851	-83382	-89916	-71647	-75248	-71509	-94180	-90709	-69565	-74838	-87025	-90827	-88547	-65964	-88604	-80387	-85631	-80388	-82054	-84962	-76123	-88580	-95188	-77004	-80606
374	-493276	-479144	-493088	-443580	-516043	-628278	-415406	-460862	-452778	-690813	-609512	-397410	-453626	-537837	-580095	-620952	-380319	-583610	-544192	-568240	-542144	-539538	-527375	-483011	-570472	-656092	-456265	-504940
375	-50.901	-52.836	-58.732	-128.97	-163.27	65.162	-91.505	-89.454	-137.4	1.2567	-147.84	53.382	43.142	-19.15	-218.46	60.966	36.553	70.241	25.365	219.19	25.75	33.095	95.329	33.256	78.941	230.29	13.86	-10.101
376	-7.4248	-7.4174	-7.9433	-10.329	-10.584	-12.165	-7.3438	-7.7123	-10.35	-7.6592	-8.8897	-12.079	-12.623	-6.13	-10.416	-12.124	-13.296	-11.045	-12.599	-14.686	-13.828	-11.511	-10.189	-11.867	-10.217	-14.866	-13.281	-12.9
377	7.4248	7.4174	7.9433	10.329	10.584	12.165	7.3438	7.7123	10.35	7.6592	8.8897	12.079	12.623	6.13	10.416	12.124	13.296	11.045	12.599	14.686	13.828	11.511	10.189	11.867	10.217	14.866	13.281	12.9
378	-1.888	-1.8896	-1.8854	0.8349	0.7444	-4.4064	-1.652	-1.7503	0.8566	-1.4852	0.1345	-4.5463	-4.5373	-2.3907	0.7971	-5.5732	-3.9793	-4.4682	-4.1977	-5.6489	-4.3195	-4.023	-5.1301	-4.4583	-4.0954	-5.8292	-4.4784	-4.3463
379	0.3839	0.2349	0.3356	0.3135	0.1607	0.2709	0.3969	0.3066	0.4686	0.4891	0.1931	0.1587	0.4378	0.1733	0.4462	0.2692	0.4174	0.3795	0.279	0.1495	0.6013	0.3825	0.2758	0.1961	0.1653	0.1185	0.2593	0.3722
380	0.8676	0.8731	0.9609	0.8277	0.9573	0.9552	0.8888	0.9576	0.9671	0.7499	0.8855	0.9479	0.7053	0.9008	0.6773	0.9017	0.842	0.7022	0.9043	0.9064	0.802	0.8197	0.9056	0.9379	0.886	0.9437	0.8883	0.8496
381	19.304	20.589	16.114	6.3272	7.493	10.224	19.668	21.298	5.9036	13.413	7.8099	27.245	12.561	54.821	6.6025	15.813	10.63	31.12	22.327	5.4679	16.019	17.219	34.624	24.613	25.271	17.823	17.042	19.095
382	-69118	-69962	-72384	-65008	-75882	-80944	-65552	-68999	-65722	-86145	-83403	-63125	-67409	-79047	-82663	-80921	-59686	-79504	-73468	-77741	-73460	-74316	-76689	-69299	-80161	-85223	-69888	-73315
383	-492418	-479727	-493564	-442712	-518710	-630927	-417666	-462725	-456217	-693415	-609543	-396490	-450495	-517296	-602190	-635534	-385285	-584751	-545604	-573296	-574688	-545149	-530473	-487121	-585867	-649501	-477522	-524600
384	-108.82	-99.897	-135.56	-149.88	-188.89	14.992	-139.76	-145.43	-154.6	-38.866	-160.07	9.2884	26.746	-64.832	-239.89	47.052	7.3863	39.954	-4.7539	176.54	3.0249	1.4029	18.341	13.663	-13.418	193.09	-16.588	-5.61
385	-8.0145	-8.1952	-8.5688	-9.8648	-9.3788	-12.34	-7.9306	-8.2385	-9.9671	-8.5793	-8.9639	-11.963	-11.845	-5.7039	-9.871	-11.469	-13.117	-11.449	-12.313	-14.371	-12.957	-11.633	-10.442	-11.669	-10.655	-14.65	-12.401	-11.877
386	8.0145	8.1952	8.5688	9.8648	9.3788	12.34	7.9306	8.2385	9.9671	8.5793	8.9639	11.963	11.845	5.7039	9.871	11.469	13.117	11.449	12.313	14.371	12.957	11.633	10.442	11.669	10.655	14.65	12.401	11.877
387	-1.8056	-1.4916	-1.9652	0.0861	0.013	-3.809	-1.655	-1.8313	0.3059	-0.8342	0.3176	-4.9352	-4.6901	-3.4559	0.3284	-3.7936	-3.9379	-4.8933	-4.5827	-6.0228	-4.5254	-3.9255	-5.3797	-4.8469	-4.5305	-6.2635	-4.3873	-4.3144
388	2440.5	3344	2308.2	1882.3	3338.7	3566.7	1794.1	2089.7	1885.5	3830.9	4311.2	2520.3	2055.7	3843.8	2449.9	3552.1	1368.8	3844.3	3172.6	4715.3	2245.3	3210.2	3378.3	2842.4	4442.7	5861.6	2856.9	2888.3
389	832.22	745.29	674.64	551.22	506.62	867.99	623.13	565.89	725.49	1673.8	800.97	379.82	839.93	642.39	1029.6	880.93	505.76	1401.6	810.89	685.81	1123.5	1115.2	854.23	522.48	715.85	673.45	689.85	967.63
390	1880.8	2770	1931.7	1455.3	3018.1	3061	1395.3	1767.6	1497.3	2566	3673.1	2268.1	1353.1	3338.7	1562.8	2950.9	1020.2	2593.5	2628	4157.7	1498.6	2389.7	2805	2498.4	3838	5364.8	2363.3	2209
391	2167.9	3172.7	2010.2	1758.2	3152.6	3204.5	1569.9	1845.8	1548.2	3421.9	4148.2	2392.7	1918.4	3706.6	2307.4	3272.4	1211.6	3693.5	2906.2	4587.2	1868.5	2915.4	3097.3	2663.9	4331.7	5684.9	2660.6	2600
392	3.1604	3.5701	3.0677	2.8715	3.5882	3.5169	2.8671	2.9994	2.8613	3.4742	3.8271	3.398	2.8656	3.8356	2.9831	3.4737	2.5887	3.677	3.4908	4.1305	2.9366	3.3957	3.5956	3.4109	4.017	4.3882	3.4921	3.4106
393	3.0178	3.4514	2.6961	2.7726	3.5379	3.3007	2.5477	2.7327	2.6595	2.9516	3.6608	3.3698	2.2835	3.5665	2.4062	3.2787	2.1501	3.2567	3.4421	4.3449	2.3311	2.8786	3.2122	3.5093	3.8673	4.5119	3.1344	2.929
394	1.398	1.4119	1.3572	1.4869	1.2747	1.5411	1.4749	1.4082	1.4733	1.9495	1.6371	1.2962	1.8783	1.521	1.9197	1.6889	1.4892	2.1711	1.6101	1.5793	1.8517	1.7338	1.6689	1.2444	1.5593	1.51	1.6566	1.857
395	1.3333	1.0829	1.1886	0.9256	0.9378	1.3526	1.1442	1.0623	1.26	1.3327	0.92	0.9072	0.9908	0.8396	0.9204	1.118	1.041	0.8123	1.198	0.8926	1.3601	1.3303	1.0683	1.0129	0.853	0.9844	0.9866	1.0844
396	257.63	260	234.5	226.75	248.88	286	211.75	226.5	236.75	316.25	287.25	216.75	233.5	241.88	250.38	292.13	195.13	273.5	270.38	284.38	267.13	271.5	248.75	241.88	264	297.5	225.38	239.88
397	303.74	307.59	275.06	259.24	285.8	333.49	255.3	265.1	275.77	359.35	325.87	262.16	270.39	296.48	282.85	324.15	222.95	323.9	315.14	341.46	308.85	312.55	303.28	281.6	315.4	352.65	273.85	293.2
398	2.6973	0.9286	4.781	4.5712	4.2874	0.4074	3.5629	3.1838	4.1248	2.8767	3.8555	3.6631	2.138	3.9952	3.2652	2.6933	3.5181	4.0411	4.0923	3.3255	2.451	3.7737	4.1207	3.3292	2.3324	3.6974	4.3246	3.7738
399	0.082	0.0782	0.0375	0.085	0.0466	0.0611	0.0399	0.0451	0.0916	0.0874	0.08	0.0531	0.048	0.0272	0.0422	0.0711	0.0486	0.055	0.0914	0.0717	0.0754	0.0749	0.0347	0.0734	0.0414	0.0475	0.0345	0.0417
400	2.7235	2.7643	2.9131	2.8352	2.9183	2.8741	2.9715	2.8812	2.7776	2.7607	2.8269	3.0514	2.9546	3.0503	2.9654	2.8009	3.0997	2.9514	2.8842	2.9965	2.8955	2.7944	2.9638	2.9618	2.9676	2.9555	3.046	3.0281
401	1.9074	2.007	2.3829	2.0396	2.2728	2.3794	2.3889	2.2641	1.886	2.1931	2.2762	2.7326	2.6469	2.8099	2.6351	2.4397	2.6905	2.6803	2.3823	2.7702	2.3772	2.2034	2.7068	2.6093	2.6959	2.6207	2.7274	2.7424
402	1.282	1.3478	1.8326	1.2683	1.8033	1.6696	1.8623	1.69	1.3295	1.4532	1.5227	1.9288	1.8665	2.2667	1.9386	1.3579	1.9485	1.9304	1.6145	1.9423	1.611	1.3961	2.1161	1.6981	2.1345	1.8587	2.1584	2.0634
403	0.7643	0.7824	1.2306	0.793	1.1361	0.9409	1.2003	1.1179	0.774	0.7931	0.8343	1.0531	1.0506	1.3884	1.2324	0.8121	1.1759	1.0556	0.8113	0.9064	0.9304	0.8516	1.2928	0.906	1.1873	1.0311	1.2871	1.1851
404	0.4196	0.4098	0.7028	0.4408	0.5919	0.5413	0.6444	0.6218	0.408	0.4311	0.4094	0.5005	0.6373	0.7213	0.7494	0.4755	0.7108	0.5127	0.3445	0.3602	0.4893	0.4887	0.671	0.4034	0.5817	0.5395	0.6693	0.6111
405	0.1906	0.1738	0.3734	0.1832	0.2742	0.3132	0.2862	0.326	0.2088	0.2103	0.1792	0.2188	0.2711	0.3777	0.4075	0.2458	0.3787	0.2323	0.1619	0.1386	0.2103	0.2578	0.3007	0.1694	0.2425	0.2652	0.2898	0.2827
406	0.0741	0.0717	0.1697	0.0569	0.1049	0.1543	0.1156	0.1489	0.0797	0.0859	0.0679	0.0704	0.1012	0.1554	0.19	0.1176	0.1515	0.0944	0.0638	0.0403	0.0902	0.1255	0.1175	0.0503	0.0976	0.112	0.073	0.1069
407	0.0342	0.0271	0.0602	0.0185	0.0309	0.0451	0.04	0.0591	0.022	0.0257	0.0157	0.0153	0.0453	0.031	0.0862	0.0403	0.0607	0.0321	0.0171	0.0149	0.037	0.0463	0.0419	0.0164	0.0288	0.0286	0.0175	0.0352
408	561.13	631.63	397	530.88	520.88	585.25	354.25	407.13	530.13	707.5	698.38	432.63	446.13	410.13	490.25	687.63	365.63	553.75	602	602.5	512.5	621.38	430.25	535.25	551.38	630.75	382.63	427.5
409	307.75	349.75	216.75	292.25	284.38	336	193.25	220	284.75	396.5	399.5	243.13	231.75	224	263	380.25	199.38	318.75	333.38	341.75	281.38	343	238.25	301.13	310.75	360.38	211.5	246.63
410	241.25	266.88	164.75	221.88	211	252.75	144.75	169.13	224.13	294.13	287.13	170.13	159.38	155.5	177.13	260.25	137.63	223.88	243.63	241	206.25	251.75	165.88	210.75	215.13	256.5	149.63	175.5
411	207.38	233.63	139.25	192.5	178.25	211.5	116.75	144.5	196.5	253.88	237.75	129.13	127.38	116.13	144.63	220.75	116	173.13	202.13	189.38	171.75	218.75	129.38	171.88	163.5	205.63	120	134.88
412	172.25	201.75	116.13	163	149.13	172.75	95.75	119.88	164.88	216.5	202.38	100.25	101.38	89.25	121.38	190.88	95.5	140.25	162.63	146.13	144	190.25	96.125	142.63	132	166.38	91.125	103.75
413	109.25	139.88	78.75	117.25	97.875	122.25	58.75	78.5	101.63	160.63	149.88	71.875	75.375	68.375	95.25	158	77.625	111.88	111.75	108	105.13	143.25	70.125	115.5	104	135.25	71.375	76.375
414	46.75	67.625	37	58.5	47.25	65.625	22.75	34.625	39	88.25	92.125	45	38.875	42.875	57.875	94.75	43.5	73.125	60.75	67.875	56.375	77.625	37.125	63.5	68.875	76.375	34.75	49.875
415	21.875	30.125	14.125	25.375	22.125	34.375	8.75	11.125	16.875	45	47.875	19	11.625	23.125	26.25	33.625	19.5	37.75	23.5	28.875	22.625	26.375	20.125	25.375	39.375	35.75	19	26.875
416	1.5811	0.7071	0.5	0.5	2.5495	0.5	0.5	0.5	1.8708	8.9582	0.5	0.5	0.5	0.5	0.7071	0.7071	0.5	0.5	0.5	6.8374	7.9687	0.5	0.5	0.5	0.7071	0.5	0.5	0.7071
417	0.5	0.7071	1.118	0.5	2.2361	0.7071	6.5955	1.118	1.118	0.7071	0.7071	0.5	1	2.2913	1.8708	0.5	1.5	0.5	6.7454	6.8007	7.8262	0.7071	1	0.7071	0.5	0.5	0.866	1.118
418	1.118	1.4142	0.7071	1	0.7071	1.118	6.3443	1.5811	0.866	8.6458	0.5	0.7071	0.5	2.4495	1.2247	0.5	1.2247	0.7071	6.6895	8.3367	0.5	0.5	0.5	0.5	0.5	0.7071	0.7071	0.5
419	0.7071	0.7071	0.5	0.5	0.5	8.5147	0.5	0.5	0.5	1.118	0.5	0.5	0.5	1.2247	0.866	0.7071	0.7071	0.866	0.5	0.5	0.5	0.5	6.8191	0.5	0.7071	0.5	0.5	0.5
420	1.118	0.5	0.5	0.7071	0.5	9.1241	0.866	0.5	0.7071	0.866	0.5	0.7071	0.7071	0.7071	0.7071	0.5	0.7071	1.118	0.7071	0.5	0.7071	0.5	0.5	0.5	0.866	0.5	0.7071	0.5
421	0.5	0.5	0.7071	0.5	0.7071	5.5	0.7071	0.7071	1.118	0.7071	1	0.5	0.866	0.7071	0.5	0.5	0.7071	0.7071	0.5	0.7071	0.866	0.7071	6.9821	1	0.5	0.7071	1.118	0.7071
422	-2.6073	-2.8147	-2.5594	-2.374	-2.6119	-3.235	-2.1696	-2.2009	-2.4584	-2.6227	-2.6221	-2.4413	-2.2547	-2.2268	-2.4366	-2.9364	-2.3168	-2.8308	-2.3891	-2.4214	-2.3843	-2.6824	-2.7525	-2.484	-2.5263	-2.82	-2.1752	-2.42
423	-2.5944	-2.7871	-2.5328	-2.2987	-2.5522	-3.1427	-2.0496	-2.184	-2.4108	-2.5283	-2.6034	-2.2012	-2.178	-2.2004	-2.4179	-2.6627	-2.1757	-2.8034	-2.3524	-2.2517	-2.3055	-2.6806	-2.7486	-2.4089	-2.5083	-2.786	-2.1664	-2.3364
424	-2.5617	-2.7038	-2.5152	-2.2343	-2.4517	-3.0201	-2.0111	-2.1777	-2.4097	-2.5085	-2.5921	-2.0404	-2.0204	-2.1895	-2.3865	-2.5233	-2.1648	-2.7413	-2.2683	-2.2045	-2.2219	-2.5482	-2.7103	-2.3478	-2.4733	-2.7684	-2.1468	-2.2956
425	-9.8149	-8.3173	-11.493	-6.8072	-7.7797	-7.1393	-7.9433	-9.5127	-7.1784	-7.2815	-6.9596	-7.0562	-8.5783	-7.6718	-9.3744	-8.8361	-8.5135	-7.3506	-7.0266	-7.1168	-8.3784	-7.9063	-8.0533	-7.2195	-7.9593	-7.284	-7.0935	-8.3159
426	-9.6085	-8.2516	-10.941	-6.8058	-7.5531	-7.1245	-7.5575	-9.4853	-6.9779	-7.1756	-6.9111	-6.9119	-8.2724	-7.1001	-9.1347	-8.8024	-8.2608	-7.2313	-6.8714	-6.8909	-8.308	-7.9045	-7.9304	-6.7509	-7.2421	-7.2593	-6.8397	-8.1461
427	-9.344	-7.5379	-10.772	-6.7779	-7.3803	-7.1218	-7.4466	-9.3376	-6.9536	-7.1185	-6.612	-6.7331	-7.6578	-7.0791	-9.0689	-8.717	-8.1226	-6.9772	-6.8474	-6.8893	-7.9597	-7.6874	-7.8244	-6.6818	-7.0988	-7.0216	-6.7492	-8.0925
428	1.2758	1.2844	1.2484	1.2058	1.2634	1.2501	1.2328	1.2429	1.2353	1.2575	1.2738	1.2443	1.1854	1.3052	1.192	1.2489	1.1615	1.2993	1.26	1.2954	1.1911	1.2604	1.3152	1.2444	1.323	1.3132	1.2833	1.2759
429	269.13	229.13	321.5	258.75	278.75	169.25	303.5	292	242.88	276.63	286.88	213.88	148.88	314.13	249.88	178	190.38	240.25	182.75	162.5	191.75	185.5	261.88	235.75	253.38	150.88	283.25	286.38
430	317.63	280.25	398.75	302.25	357.88	200.13	379.63	380.13	285.25	351.75	358.5	290.38	221.25	460.25	355.63	239	228.25	307.13	236	215	231.88	219.88	355.5	289.88	346.25	194.75	379.75	361.63
431	221.5	239.13	320.38	222.13	317	179	295.75	301.63	219.63	255.25	280.75	208.38	191.25	382.75	313.5	191.13	219.63	270.88	187.5	185.25	197.25	188.75	305.88	221.13	324.13	183.5	303.88	289.13
432	109.25	121.5	184.25	110.75	165.38	107.13	161.25	163.75	106.5	123.88	122.88	106	120.25	205.25	183	115.5	132.25	137.63	81.375	78.25	107.38	118.63	168.38	103.25	159.13	104.25	156.75	156.63
433	50.25	54.5	91.375	50.5	74.875	57.75	73.5	80.75	48.5	58.625	52.375	48.125	66.5	99.875	99.375	59	75.375	62.875	33.5	31	54.625	64.75	82.625	42.25	68.75	51	73.625	76.875
434	21.375	24.125	47.75	18.875	33.625	33.375	31.5	39.75	22.5	27.875	20.5	22	30.625	51.125	51.625	28.375	39.125	30.875	16.125	17.125	26.75	35.625	37	18	32.875	27.625	31.5	37.25
435	8.625	8.875	21.5	5.875	13.375	15.5	13.5	18	9.625	12.625	9.625	7.5	11.25	18.125	23.75	13	14.875	12.625	7.625	5.5	10	16.375	14.875	6.375	12.5	10.75	9.125	13.5
436	4.5	3.5	8.75	1.875	3.125	4.5	3.875	6.875	2.5	3.375	2.5	1.375	4.25	3.75	10.125	4.25	5.5	4.25	2	1.625	4	5	5.5	1.5	3.75	3	1.75	4
437	0.271	0.2412	0.6662	0.262	0.4336	0.3863	0.601	0.5514	0.2649	0.2639	0.2429	0.399	0.5506	0.783	0.6366	0.3208	0.663	0.3808	0.2283	0.236	0.3491	0.3386	0.6526	0.2675	0.4259	0.3899	0.6433	0.5349
438	0.132	0.1102	0.3761	0.1065	0.209	0.2222	0.2901	0.2907	0.1279	0.1305	0.1057	0.1936	0.265	0.4607	0.3717	0.171	0.3578	0.1957	0.1082	0.1023	0.1674	0.1794	0.3227	0.1164	0.2217	0.2091	0.2885	0.2864
439	0.5297	0.2993	2.253	1.6635	1.5871	0.5152	2.0556	1.6129	1.5649	0.9491	1.2475	1.3104	0.6003	1.7112	1.7261	1.5201	1.7355	1.6328	1.1445	0.6922	1.0177	1.3081	1.5939	0.9983	0.8902	1.0144	1.8942	1.6416
440	0.4508	0.2458	2.2389	1.6174	1.5746	0.6397	2.0147	1.6089	1.4175	0.9518	1.2978	1.3556	0.7206	1.8384	1.6342	1.5499	1.7057	1.627	1.0186	0.7207	1.0494	1.4176	1.648	0.9635	0.9274	0.8926	1.8743	1.7001
441	0.6357	0.1472	2.5974	1.6298	2.0958	0.4481	2.3	1.5855	1.5931	0.9359	1.3288	1.3939	0.7614	1.987	1.5941	1.594	1.8998	1.6382	1.1792	0.7691	1.2376	1.6372	1.7866	0.8793	1.0239	0.9684	2.033	1.7996
442	0.7519	0.1843	2.9891	1.8153	2.6457	0.4466	2.8756	1.8016	1.864	1.178	1.4954	1.5777	1.0026	2.2414	1.8264	1.7088	2.1804	1.7934	1.3892	0.7533	1.6376	1.9255	2.0951	1.026	1.2241	1.0909	2.1034	1.8628
443	0.7582	0.2344	3.2294	2.0181	2.9679	0.4943	3.3003	2.0862	2.0396	1.3067	1.6236	1.8006	1.2114	2.44	2.0573	1.8418	2.474	1.939	1.5922	0.827	1.8956	2.0948	2.3297	1.2877	1.3855	1.1233	2.0983	1.891
444	0.7664	0.4922	2.9354	2.3531	2.3989	0.6151	2.6836	2.2576	1.8108	1.2701	1.8579	2.1542	1.4782	2.4946	2.1432	2.1385	2.6943	2.0815	1.3412	0.9948	1.8698	1.8397	2.3434	1.4706	1.4942	1.1824	2.0081	1.9546
445	1.1894	0.845	2.359	2.3873	1.6897	1.9328	1.7803	2.6207	1.0619	2.6682	2.1114	2.4814	1.363	2.068	1.5714	1.9271	2.3309	1.8824	1.0617	1.1673	1.8644	1.5795	1.697	1.6965	0.8208	1.1568	1.1375	1.8149
446	1.7486	0.7554	1.9967	2.2763	1.9193	2.6975	1.3529	2.7677	0.3029	3.2204	2.7145	2.7132	2.0926	1.916	2.0029	2.8351	2.7002	1.62	1.0309	1.6032	1.0747	1.7638	1.6818	1.7042	0.6951	2.1147	1.6576	1.1927
447	0.3886	0.1855	0.6813	0.5484	0.7324	0.5444	0.3783	0.3995	0.4693	0.3106	0.5001	0.5422	0.2407	0.2482	0.4895	0.1736	0.3188	0.4328	0.6408	0.2313	0.406	0.2125	0.3324	0.803	0.3512	0.423	0.22	0.3584
448	0.8891	0.2374	1.5336	1.6584	1.7115	0.8575	1.0862	1.0493	1.6213	0.9489	1.251	0.7569	0.3991	0.4233	1.1078	0.7476	0.8806	0.6261	1.2604	0.3138	0.9986	0.9655	0.6511	1.3097	0.4879	0.7149	0.2972	0.1024
449	1.1607	0.3913	1.9389	2.4033	1.9712	0.6763	1.2246	1.3039	1.9745	1.3255	1.6853	1.0435	0.7485	0.9501	1.4746	1.2929	1.3016	1.0051	1.3477	0.6429	1.2694	1.4639	1.1093	1.5136	0.8171	1.0029	0.7971	0.5508
450	1.5258	0.5463	2.224	2.689	2.1454	0.3975	1.3203	1.5768	2.2913	1.6425	2.1177	1.6134	1.1813	1.5993	1.6294	1.3116	1.558	1.6996	1.9547	1.3892	1.2488	1.8303	1.7883	1.7432	1.0708	1.6908	1.6378	1.2901
451	2.0723	0.8619	2.2679	3.0159	2.2709	0.6836	1.3888	1.7102	2.5802	1.9612	2.5962	2.318	1.4299	2.0285	1.7111	1.1809	1.6191	2.4256	3.0271	2.5723	1.2675	2.1508	2.3713	2.495	1.569	2.8207	2.3374	2.0543
452	2.9157	1.1005	2.4563	3.6426	2.8088	0.6687	1.2939	2.0572	2.9785	2.4445	3.433	3.1565	1.8439	2.3473	1.7475	0.9581	1.8456	2.9619	3.799	3.4042	1.1243	2.5032	3.0161	3.4662	2.1403	3.6681	2.8845	2.2739
453	3.6957	0.9485	2.7117	4.7105	3.6064	0.588	1.1306	2.639	3.5866	3.2971	4.078	4.4612	2.6797	2.3687	2.2756	1.3343	1.9527	3.4672	4.2733	4.9561	0.4538	2.5259	2.9915	4.6479	2.8128	4.7181	3.1859	1.86
454	3.554	1.9961	3.6818	4.9993	3.8595	1.5878	0.7892	3.651	4.5333	3.283	5.0589	5.145	3.0237	2.1765	2.907	2.7364	1.9566	4.0265	5.0544	5.2216	1.9784	2.4148	2.2576	4.8916	0.8175	5.0331	3.2704	2.0637
455	1.365	1.3702	1.3538	1.3795	1.3555	1.4178	1.3232	1.3411	1.3676	1.4643	1.4088	1.3133	1.3944	1.3128	1.4122	1.4178	1.3398	1.3584	1.3864	1.3706	1.4442	1.3904	1.3088	1.3553	1.32	1.3732	1.2943	1.3332
456	437.63	449.92	403.3	377.31	419.11	444.16	370.97	390.55	398.7	482.22	463.97	375.77	366.28	433.43	382.04	442.85	318.14	457.72	434.86	461.85	398.59	437.82	440.77	400.36	456.35	482.92	400.4	417.59
457	597.38	616.5	546	520.5	568.13	629.75	490.88	523.75	545.25	706.13	653.63	493.5	510.75	569	539.5	627.88	426.25	621.75	602.88	633	575.63	608.75	576.88	542.63	602.38	663.13	518.25	556.75
458	1031.5	1114.3	1134.5	952.88	1188.8	1229.5	1041.8	1044.9	970.25	1253.1	1248.1	1115.8	1046.8	1367.4	1138.3	1179	966.13	1356.4	1182.3	1368.8	1081.8	1149.9	1319.9	1137	1385.5	1436.5	1220.5	1260.5
459	602.25	650	785.25	553	791.75	805.75	720.25	696.75	561.38	769.63	773.63	783.13	717.25	1030.6	785.25	723	673.63	949.38	752.75	946.25	695.38	708.25	965.25	750.5	985.63	980.13	890.25	904.88
460	337	373.5	489.25	301.38	488.13	470.25	441.88	426.5	324.38	429.88	423	427.75	420.13	632.5	478	378.38	399.88	533.63	401	500	391.38	399	585.63	398.25	585.38	558.25	537.13	521.5
461	149.38	157.5	237.75	132.5	214.63	204.75	204	202.38	138.63	177.13	169.13	173.38	188.75	278.38	227.63	174.63	186.88	213.38	149.38	169.88	168.75	181	253.25	152.75	236.25	229.75	232.88	221.13
462	65.375	64.375	109.75	58.125	91.5	97.625	87	93.25	59.375	77.625	69.75	67.875	87.75	121.75	112.75	83.5	91.25	85.25	55.625	56.875	72	85.25	108.25	56.375	91.625	100	96.25	93.875
463	27.375	25.75	52.375	20.5	37.25	47	33.875	42	25.125	33.125	25.125	25	33.75	53.5	53.75	37.75	41.5	33.875	21.875	19.375	28.75	39.25	41.75	20	36.25	43	34.625	38.625
464	10.375	8.875	21.875	5.875	13.375	19.625	13.5	18	9.75	12.75	9.75	7.5	11.25	18.125	23.75	16.5	14.875	12.625	8.25	5.5	10	16.375	15.375	6.375	12.5	15.5	9.25	13.5
465	4.75	3.5	8.75	1.875	3.125	5	3.875	6.875	2.5	3.375	2.5	1.375	4.25	3.75	10.125	5.375	5.5	4.25	2	1.625	4	5	5.5	1.5	3.75	3.875	1.75	4
466	762.38	885.13	813	694.13	910	1060.3	738.25	752.88	727.38	976.5	961.25	901.88	897.88	1053.3	888.38	1001	775.75	1116.1	999.5	1206.3	890	964.38	1058	901.25	1132.1	1285.6	937.25	974.13
467	284.63	369.75	386.5	250.75	433.88	605.63	340.63	316.63	276.13	417.88	415.13	492.75	496	570.38	429.63	484	445.38	642.25	516.75	731.25	463.5	488.38	609.75	460.63	639.38	785.38	510.5	543.25
468	115.5	134.38	168.88	79.25	171.13	291.25	146.13	124.88	104.75	174.63	142.25	219.38	228.88	249.75	164.5	187.25	180.25	262.75	213.5	314.75	194.13	210.25	279.75	177.13	261.25	374.75	233.25	232.38
469	40.125	36	53.5	21.75	49.25	97.625	42.75	38.625	32.125	53.25	46.25	67.375	68.5	73.125	44.625	59.125	54.625	75.75	68	91.625	61.375	62.375	84.875	49.5	77.125	125.5	76.125	64.5
470	15.125	9.875	18.375	7.625	16.625	39.875	13.5	12.5	10.875	19	17.375	19.75	21.25	21.875	13.375	24.5	15.875	22.375	22.125	25.875	17.375	20.5	25.625	14.125	22.875	49	22.625	17
471	6	1.625	4.625	1.625	3.625	13.625	2.375	2.25	2.625	5.25	4.625	3	3.125	2.375	2.125	9.375	2.375	3	5.75	2.25	2	3.625	4.75	2	3.375	15.375	3.125	1.375
472	1.75	0	0.375	0	0	4.125	0	0	0.125	0.125	0.125	0	0	0	0	3.5	0	0	0.625	0	0	0	0.5	0	0	4.75	0.125	0
473	0.25	0	0	0	0	0.5	0	0	0	0	0	0	0	0	0	1.125	0	0	0	0	0	0	0	0	0	0.875	0	0

	K	K	K	L	L	L	L	L	L	L	L	L	L	L	L	L	L	L	L	L	L	L	L	M	M	M	M
	V	W	Y	A	C	D	E	F	G	H	I	K	L	M	N	P	Q	R	S	T	V	W	Y	A	C	D	E
No.	KV	KW	KY	LA	LC	LD	LE	LF	LG	LH	LI	LK	LL	LM	LN	LP	LQ	LR	LS	LT	LV	LW	LY	MA	MC	MD	ME
365	0.3153	0.4577	0.4496	0.2028	0.1991	0.2083	0.1597	0.3416	0.2064	0.3377	0.1198	0.2981	0.1526	0.2048	0.2741	0.17	0.2982	0.3813	0.2744	0.1806	0.1461	0.3434	0.3327	0.2585	0.2795	0.2531	0.2621
366	0.1169	0.2727	0.2624	0.154	0.3071	0.3318	0.3038	0.2843	0.2491	0.1951	0.1215	0.1311	0.1305	0.2428	0.2346	0.167	0.213	0.1722	0.2131	0.2226	0.1627	0.2987	0.2917	0.2754	0.3715	0.4053	0.3695
367	0.5678	0.5582	0.5493	0.6464	0.5047	0.4703	0.5542	0.7196	0.5602	0.613	0.7587	0.5708	0.7186	0.5524	0.5089	0.6632	0.4991	0.4475	0.5138	0.5974	0.6924	0.677	0.6009	0.4674	0.3807	0.3579	0.3837
368	0.4322	0.4418	0.4507	0.3536	0.4953	0.5297	0.4458	0.2804	0.4398	0.387	0.2413	0.4292	0.2814	0.4476	0.4911	0.3368	0.5009	0.5525	0.4862	0.4026	0.3076	0.323	0.3991	0.5326	0.6193	0.6421	0.6163
369	1.8308	3.2222	3.1839	0.8016	0.8686	1.0122	0.7758	1.6558	0.816	1.6275	0.4734	1.7309	0.6034	0.9037	1.459	0.6602	1.5873	2.9478	1.2712	0.8367	0.5775	1.7814	1.7398	1.1406	1.3482	1.3464	1.3939
370	0.5618	1.6472	1.5958	0.6087	1.34	1.9442	1.7802	1.3782	0.9845	0.9403	0.4803	0.6302	0.516	1.0716	1.2489	0.6485	1.1338	1.1594	0.9875	1.0313	0.6433	1.5496	1.5253	1.2152	1.7916	2.5613	2.3348
371	0.0564	0.0635	0.1185	0.0924	0.2231	0.1279	0.126	0.1508	0.1352	0.1297	0.1158	0.0752	0.0696	0.0869	0.1606	0.1754	0.1085	0.143	0.1131	0.0698	0.1335	0.1098	0.1985	0.0906	0.0926	0.0999	0.1142
372	33.693	42.825	13.056	7.302	19.696	18.582	12.686	5.9016	15.878	21.385	4.6326	8.4794	22.781	22.312	3.5677	14.831	3.9061	20.597	6.274	22.839	23.015	7.7436	23.182	6.4056	22.996	16.601	12.006
373	-76770	-99447	-95984	-64299	-69485	-81799	-85420	-83273	-60623	-83246	-75110	-80324	-75031	-76706	-79780	-70781	-83388	-89874	-71733	-75254	-71425	-94259	-90623	-65975	-71156	-83486	-87094
374	-482899	-717446	-660289	-364249	-406155	-476165	-525889	-566278	-326646	-546378	-491335	-534676	-485795	-482190	-487716	-445507	-524068	-615353	-406766	-448786	-447659	-673032	-626175	-351904	-394271	-465797	-511299
375	39.887	174.01	13.863	-133.59	-39.97	-230.64	-247.41	-102.67	-58.065	-7.9398	-134.87	90.126	-54.563	-35.604	-164.01	-63.009	-169.02	107.08	-177.79	-95.355	-51.89	-77.779	-59.284	-134	-37.101	-242.17	-242.15
376	-12.134	-9.0812	-11.103	-10.527	-7.7983	-4.7728	-4.5803	-9.6718	-8.0301	-7.0814	-10.261	-10.135	-7.34	-7.4136	-10.601	-7.9502	-10.563	-9.9117	-10.492	-7.423	-7.3011	-8.4019	-7.403	-9.901	-7.5819	-4.9591	-4.8296
377	12.134	9.0812	11.103	10.527	7.7983	4.7728	4.5803	9.6718	8.0301	7.0814	10.261	10.135	7.34	7.4136	10.601	7.9502	10.563	9.9117	10.492	7.423	7.3011	8.4019	7.403	9.901	7.5819	4.9591	4.8296
378	-4.6927	-4.5816	-4.2779	0.9779	-1.7513	3.5145	3.6738	-0.1665	-1.0094	-1.3848	0.881	-3.2986	-1.7214	-1.7578	0.7994	-0.7415	0.8662	-3.9841	1.0137	-1.7983	-1.7099	0.1012	-1.7135	0.8679	-2.0152	3.1637	3.5213
379	0.1426	0.1803	0.2694	0.2102	0.3813	0.2487	0.4325	0.3372	0.2126	0.3429	0.2513	0.218	0.2279	0.2694	0.3793	0.287	0.3048	0.2914	0.2183	0.2437	0.2807	0.2771	0.5266	0.1345	0.182	0.1862	0.3021
380	0.9175	0.8986	0.8572	0.8742	0.8446	0.9631	0.7423	0.8382	0.8837	0.8058	0.9221	0.8671	0.835	0.8183	0.8333	0.9031	0.8451	0.891	0.8972	0.8845	0.8798	0.8945	0.6528	0.9564	0.9279	0.944	0.8836
381	33.417	21.521	13.668	6.1384	18.275	17.549	11.07	5.2002	12.258	19.754	6.6707	2.7332	20.463	20.064	2.9139	8.7993	4.6755	14.457	6.2083	23.369	20.038	7.2206	21.875	4.4115	19.054	13.965	10.89
382	-70008	-90465	-87712	-58829	-63066	-74796	-78252	-76637	-55355	-75148	-69166	-73451	-69122	-69962	-72435	-64976	-75882	-80918	-65602	-68999	-65675	-86188	-83353	-59672	-63900	-75651	-79094
383	-482654	-742295	-690052	-363752	-400149	-476011	-523807	-573840	-325935	-548690	-496752	-542869	-486301	-483562	-490687	-456519	-522898	-618869	-405200	-444728	-448292	-670496	-631353	-350821	-388206	-462525	-513057
384	14.416	72.942	-16.293	-147.9	-88.34	-271.27	-283	-128.1	-114.82	-57.446	-152.19	13.852	-112.96	-98.875	-184.86	-117.99	-188.02	40.576	-177.09	-144.82	-109.2	-104.06	-103.71	-137.32	-68.392	-268.02	-269.2
385	-11.956	-9.7787	-11.331	-10.059	-8.2389	-5.1636	-5.3187	-9.7254	-8.8987	-7.2536	-9.9599	-11.319	-8.0486	-8.0608	-9.9194	-8.841	-9.632	-11.129	-10.056	-7.8215	-8.0424	-8.4036	-7.9917	-9.3439	-8.2723	-5.7856	-5.0729
386	11.956	9.7787	11.331	10.059	8.2389	5.1636	5.3187	9.7254	8.8987	7.2536	9.9599	11.319	8.0486	8.0608	9.9194	8.841	9.632	11.129	10.056	7.8215	8.0424	8.4036	7.9917	9.3439	8.2723	5.7856	5.0729
387	-5.137	-4.2344	-4.0908	0.2787	-1.8369	3.4964	3.961	0.0614	-0.6496	-0.8256	0.4423	-3.7947	-1.287	-1.7229	0.3119	0.2631	0.2613	-3.8017	0.3	-1.9299	-1.2737	0.0791	-1.6953	-0.0658	-1.9801	2.0092	2.222
388	3375.8	6865.2	4149.4	1696.1	2187.6	2587.7	2495	3366.9	1719.5	2843.2	2382.9	3465.3	2873.2	3182	2182.2	2009.8	2921.7	4099.9	2104.5	2535.5	2367.4	4749.4	3294.4	2473.5	3050.5	3353.1	3426.2
389	467.23	1191	1051.3	342.1	749.44	581.85	992.28	1043.8	348.74	907.38	551.02	724.72	634.88	821.18	748.15	526.72	828.45	1094.8	434.34	580.76	615.15	1211.9	1592.1	318.23	526.31	586.1	947.16
390	3007	5935	3345.2	1422.7	1660.1	2253.6	1703.3	2594.5	1449.8	2132.5	2021.9	2882	2325.9	2494.5	1643.7	1657.5	2297	3347.6	1785	2107.6	1928.1	3912.8	1973.6	2262.8	2683	2971.9	2770.1
391	3277.4	6604.4	3902.4	1627.4	1965.6	2340	2294.5	3095.5	1640.5	2646.5	2192.8	3323.8	2785.6	3048.4	1972.6	1835.4	2717.9	3757.3	1989.6	2382.6	2191.5	4374.2	3023.1	2366	2891.6	3148.2	3135.1
392	3.7019	4.5377	3.6564	2.8959	3.0555	3.2482	3.1021	3.4779	3.0225	3.2552	3.1229	3.6482	3.4292	3.4825	2.9828	2.9671	3.3567	3.7706	3.1052	3.3039	3.2061	3.8683	3.3454	3.3509	3.4767	3.5686	3.5149
393	3.8671	4.583	3.3338	2.7577	2.6037	2.9291	2.7582	3.1009	2.6607	3.0495	3.2624	3.6639	3.4852	3.3354	2.6697	2.7065	3.2157	3.5737	3.0642	3.2865	3.0578	3.743	2.5893	3.1542	3.1644	3.1971	3.1709
394	1.2565	1.6331	1.7548	1.4359	1.4177	1.2469	1.5978	1.7782	1.1983	1.6007	1.2955	1.5383	1.448	1.4971	1.5447	1.4443	1.595	1.5277	1.1633	1.4398	1.395	1.6908	2.1053	1.0677	1.1687	1.2278	1.3709
395	0.9188	1.1547	1.1477	0.8384	1.2299	1.0476	0.9792	1.2043	0.9782	1.0983	1.1101	1.0051	0.9197	0.9835	1.0698	1.1335	1.0592	1.3515	1.0304	0.8682	1.1173	1.2405	1.1536	0.9492	0.9631	1.0103	1.0714
396	251.75	330.5	299.88	202	223	223.75	241.63	281.13	185.75	258.75	253.88	272.88	258.63	261.75	229.13	229.5	251.5	289.13	207.88	226.75	239	312.75	289.38	203.75	225.38	225.13	245
397	298.82	385.16	343.12	239.71	264.74	262.92	281.17	319.05	227.74	302.71	294.31	323.93	303.27	308.66	265.29	268.68	293.18	345.09	251.22	273.9	282.8	355.14	334.47	246.95	271.98	268.86	289.54
398	3.392	2.1832	2.8146	4.718	3.3288	5.3112	3.0194	5.2766	4.2787	2.4691	1.3232	4.6064	1.1295	1.006	3.6767	5.2997	3.2377	2.1114	4.3497	2.8186	2.341	4.1079	3.2745	4.1572	4.7391	4.9045	3.2654
399	0.0646	0.0754	0.0527	0.066	0.0694	0.037	0.0383	0.1013	0.0584	0.0616	0.0814	0.0583	0.0983	0.0691	0.041	0.072	0.0484	0.0487	0.0412	0.0425	0.073	0.0866	0.0721	0.0658	0.0627	0.0396	0.049
400	2.913	2.8651	2.8552	2.9333	2.8357	2.949	2.9004	2.7576	2.9608	2.8474	2.7524	2.8574	2.7513	2.7598	2.9273	2.8518	2.8725	2.8286	2.9517	2.8594	2.7812	2.7257	2.8412	2.8954	2.8552	2.9334	2.9094
401	2.3403	2.5937	2.6132	2.1637	2.2179	2.364	2.3172	2.1689	2.3107	2.1967	1.9498	2.3253	2.0134	2.0241	2.3734	2.1141	2.2547	2.3474	2.2407	2.1364	1.9898	2.1061	2.4115	2.2454	2.372	2.426	2.4053
402	1.649	1.6923	1.6815	1.5472	1.6468	1.8704	1.8211	1.2732	1.664	1.5594	1.2351	1.6643	1.3125	1.3476	1.8316	1.3589	1.708	1.6327	1.7808	1.6426	1.4035	1.3654	1.6973	1.4721	1.6324	1.7891	1.852
403	0.9074	0.8355	0.9597	0.9383	0.8882	1.2675	1.2043	0.7522	0.9978	0.9401	0.7209	0.952	0.6949	0.7949	1.1879	0.8369	1.0985	0.9634	1.1912	1.1139	0.8101	0.7517	0.942	0.912	0.8732	1.2167	1.1541
404	0.4414	0.4063	0.5619	0.4884	0.473	0.7689	0.7141	0.4035	0.5358	0.5388	0.4101	0.4749	0.3601	0.455	0.6971	0.4816	0.6016	0.5941	0.6459	0.6203	0.4705	0.4127	0.4988	0.496	0.4838	0.7119	0.6297
405	0.1891	0.1759	0.2917	0.2057	0.2046	0.4125	0.406	0.2049	0.2312	0.2332	0.2175	0.2061	0.1877	0.2274	0.3371	0.2235	0.2923	0.3175	0.2467	0.322	0.2209	0.1811	0.2038	0.2103	0.2393	0.3572	0.3249
406	0.069	0.0755	0.1421	0.0737	0.0717	0.1445	0.1591	0.0832	0.0875	0.0689	0.1054	0.072	0.0731	0.0831	0.1137	0.0838	0.0875	0.1371	0.0691	0.1162	0.0802	0.0513	0.0824	0.0589	0.1028	0.0976	0.1459
407	0.0227	0.0304	0.0486	0.0192	0.0297	0.0438	0.0674	0.0306	0.0386	0.0228	0.0546	0.0181	0.0249	0.0221	0.0284	0.0278	0.0195	0.0375	0.0182	0.0267	0.0279	0.0131	0.0305	0.0166	0.0408	0.0283	0.0453
408	544	835.5	628.75	446.5	490	421.63	459.5	706.75	414.5	550	554.75	528.88	646.5	612.38	432	523.38	526.5	619.63	384.75	422.5	533	796	583.13	503.38	564.5	508.75	537
409	297.25	493.75	358.25	238.13	274.25	233.88	245.13	406.88	228.75	297.63	302	291.5	360.13	342.25	232.75	287	289.38	352	209.25	224.38	294.63	454.88	335.63	274.88	308.38	283.38	306.5
410	221.63	344.13	245	181	203.38	176.13	175.25	301	172.25	224.5	233.13	218.5	269.75	257.5	167.75	214.88	213.25	255.63	158.5	181.88	228.38	338.25	243.25	209	217.88	200.38	225.38
411	188.13	272.25	198	155.88	167.88	147.5	149	260	143.13	189.5	207	184.63	226.63	224	144.25	186	175.5	209.38	136.63	158.25	198.88	290.13	197.25	179	179.5	170.38	186.13
412	154.13	225.5	163.25	130.63	136.25	116.25	126.25	225.38	120	158.25	174.38	150.75	191.5	190.38	117.25	162.5	144.75	173	110.63	132.25	168.5	248.63	165.38	151.63	147.25	145	154.5
413	110.5	186.88	133.75	83.625	86.5	75.25	83.875	178.13	80.5	100.13	117.5	100.5	130.25	132.25	78.625	122.25	96.125	127.38	65.875	72.5	110.38	178.25	124.38	106.88	101.63	106.63	107
414	52	130.13	96.25	37.875	39.375	42	40.25	101.63	44.375	47.875	50.25	47	71.5	69.25	36.375	57.375	47.125	76.625	24.625	28.375	51.375	100.25	82.5	57.625	58.375	64.125	71
415	23.125	72.375	41.5	13.5	18	22.5	18.25	48	21.5	18.125	18	17.125	32.75	28	13	22.375	25.125	40.25	11.5	12.625	23.75	53.25	44.375	22.875	24.125	26.375	35.75
416	6.7823	0.5	0.5	1.2247	0.5	6.8191	7.2284	0.7071	0.5	0.5	0.7071	0.5	0.5	0.866	1.118	0.7071	0.5	0.7071	0.5	0.5	6.7823	0.5	0.5	0.5	0.5	0.7071	0.7071
417	0.5	0.7071	1.2247	0.5	1.5811	0.7071	6.9282	0.5	0.5	0.7071	0.5	6.1033	1.2247	7.3824	0.7071	1.118	0.7071	0.5	1.118	0.5	0.5	0.7071	0.5	0.5	1.118	1.8028	0.7071
418	7.0178	0.5	1.6583	1.118	1.118	6.7454	0.5	0.5	1	0.866	0.5	6.364	1.6583	7.433	0.866	0.5	0.5	0.5	0.7071	0.7071	6.5765	0.5	0.7071	0.7071	0.7071	1.118	1.2247
419	0.5	0.5	0.5	0.7071	0.7071	7.3144	0.5	0.5	0.5	0.5	0.7071	0.5	7.1414	0.5	0.5	0.5	0.5	0.7071	1.2247	0.7071	0.5	0.5	7.6322	0.866	0.5	0.5	8.4558
420	0.7071	0.5	0.7071	0.5	0.7071	0.5	0.5	7.3144	0.5	0.866	0.7071	0.7071	7.2284	0.5	0.7071	0.5	0.5	4.5277	0.5	0.5	0.866	0.7071	7.3993	0.5	0.866	8.0623	8.3367
421	0.5	0.7071	1.118	0.5	1.4142	7.7942	1	7.433	0.7071	0.7071	0.7071	0.866	0.5	0.7071	1.118	0.7071	0.7071	4.3012	0.866	1.118	0.7071	1.118	0.7071	0.7071	1.2247	8.3217	0.7071
422	-2.4224	-2.6085	-2.3217	-2.125	-2.7477	-2.4138	-2.4263	-3.147	-2.2983	-2.1192	-2.4781	-2.6527	-2.5209	-2.5937	-2.7862	-2.4183	-2.7355	-2.3468	-1.9966	-2.52	-2.5153	-3.1454	-2.8553	-2.0216	-2.3151	-2.4411	-2.9018
423	-2.3222	-2.5752	-2.3165	-2.0981	-2.7368	-2.3551	-2.3567	-3.1445	-2.2041	-2.0795	-2.3692	-2.4771	-2.5075	-2.4602	-2.6475	-2.4051	-2.7046	-2.3051	-1.957	-2.3216	-2.503	-3.1007	-2.7367	-2.0095	-2.2534	-2.4247	-2.8652
424	-2.2816	-2.5615	-2.295	-2.0964	-2.653	-2.3379	-2.3458	-3.0826	-2.1918	-2.0368	-2.3496	-2.4703	-2.4341	-2.3249	-2.6402	-2.3301	-2.6747	-2.3044	-1.9402	-2.309	-2.4989	-3.0747	-2.7102	-1.9974	-2.2484	-2.4098	-2.8582
425	-7.1921	-8.3113	-8.0519	-7.2088	-9.2386	-7.2522	-11.252	-7.3682	-9.8782	-7.8437	-8.6042	-7.4274	-7.5714	-7.9428	-7.5753	-8.507	-7.0552	-7.019	-6.9984	-7.2713	-8.3142	-7.249	-7.6625	-6.6924	-9.718	-7.1632	-7.2879
426	-7.1076	-8.1386	-7.6961	-6.8901	-8.3929	-7.177	-10.867	-7.3652	-9.6762	-7.5129	-8.0255	-7.1184	-7.1511	-7.5052	-7.2191	-8.3148	-7.0195	-6.9836	-6.8997	-7.0566	-7.8875	-6.8227	-7.5813	-6.6429	-9.1762	-6.942	-7.1786
427	-6.8803	-7.7783	-7.5014	-6.7975	-8.2707	-7.1682	-10.33	-7.0324	-9.4308	-7.1605	-7.9096	-6.9546	-7.0684	-7.4641	-7.2173	-7.7101	-6.9134	-6.9758	-6.8897	-7.0378	-7.4105	-6.7032	-7.5386	-6.6241	-9.0949	-6.8723	-7.1324
428	1.2625	1.3399	1.2606	1.2033	1.262	1.2605	1.2496	1.27	1.2408	1.2412	1.2273	1.2939	1.2692	1.2894	1.2316	1.2173	1.2773	1.3186	1.2446	1.2843	1.2677	1.3185	1.241	1.2521	1.2716	1.2784	1.2815
429	217.38	218.63	222.13	227.63	256.88	365.13	360.25	241.75	325.63	232.13	225.13	200.13	230.5	265.25	267.88	235.25	249.88	203.13	285.63	314.5	247.5	279.75	298.25	246.5	260.88	348.63	384.75
430	266.5	261.75	283.63	296	365.25	494	491.38	296.13	359.88	307.5	285	256.13	257.88	296	356.38	294.38	322.13	216.63	378.25	398.5	290.5	309	388.63	323.75	351.88	488.13	496.5
431	196.5	212.63	249.13	219.5	260.88	390.13	390	227.25	247.75	242.5	207.5	222.75	199.63	236.88	314.63	231.25	288.75	190.25	298	308	229.25	239.75	299.38	231.88	253.88	392.63	382
432	96.125	116.75	152.5	109.88	119	213.25	205.38	120.5	121.25	134.88	99.375	116.25	100.38	131.25	175.38	121.88	164.5	122.88	158.5	171.25	123	124.63	143.13	116.75	123.25	212.75	195.13
433	43.375	55.875	84.625	48.5	52.625	106.88	103	56.5	53.375	66	49	57.125	47.375	65.125	84.5	59.75	76.75	66.5	67.625	80.25	60.875	57.625	65	51.5	59.625	99.875	93
434	21.25	28	42.75	20.75	21.125	50.375	49.5	27.5	21.375	26.625	23.25	25.25	23.625	28.75	37.625	25.125	34.625	34	25.25	38.625	26.625	25.25	28	21.625	25.75	41.375	43.75
435	8.125	12	19	7.375	7.75	17.375	17.125	11.25	8.375	8.375	9.75	9.25	8.625	10.5	12	9.5	10.875	14.5	8	12.625	9.5	7.25	11.125	7	12.25	12.625	18
436	2.5	4.25	6.875	1.625	3.625	4.375	7.375	3.75	3.875	2.625	5.125	2.5	3	2.875	3.375	2.75	2.375	4.125	1.875	3.25	3	1.625	3.875	1.75	5.125	3	5.125
437	0.3063	0.2263	0.4184	0.3425	0.3325	0.6806	0.6805	0.2297	0.3984	0.3653	0.282	0.3541	0.2095	0.2951	0.5812	0.3194	0.449	0.4244	0.5166	0.523	0.3087	0.2177	0.3176	0.3134	0.3442	0.5446	0.4698
438	0.1369	0.1107	0.2254	0.15	0.1512	0.3593	0.3842	0.1144	0.186	0.1596	0.1522	0.155	0.1098	0.1362	0.2747	0.1452	0.2144	0.2364	0.2059	0.2646	0.1439	0.0974	0.1515	0.1369	0.1762	0.2531	0.2525
439	1.1337	0.8567	1.2284	1.7884	1.248	2.7639	1.3377	2.1197	1.9769	1.0148	0.4168	1.8547	0.0915	0.4862	1.6904	1.976	1.4124	0.868	2.3472	1.6189	0.9423	1.3948	1.2513	1.7097	1.6098	2.3491	1.7323
440	1.0296	0.7933	1.3173	1.7186	1.2333	2.6712	1.3183	2.1026	1.8813	1.0326	0.4183	1.8981	0.1268	0.5309	1.6663	1.9994	1.3389	0.7673	2.2726	1.6625	0.9358	1.4163	1.3524	1.639	1.5716	2.3193	1.7097
441	1.1048	0.7444	1.256	2.0541	1.6626	2.9304	1.867	2.0898	2.0915	1.4659	0.5684	2.1199	0.2447	0.3435	1.9745	2.1682	1.7079	0.9517	2.8044	1.7708	1.0716	1.3659	1.4539	1.5853	1.8833	2.6237	1.9978
442	1.2336	0.9763	1.1766	2.5055	2.1941	3.3975	2.3879	2.2871	2.5077	1.9433	0.6624	2.4696	0.3606	0.2684	2.4702	2.423	2.1874	1.2805	3.3495	2.0389	1.2706	1.4034	1.7303	1.8724	2.3881	3.094	2.5616
443	1.3663	1.2892	1.2947	2.8391	2.6977	3.7238	2.8226	2.4574	2.6799	2.2047	0.7835	2.8527	0.3606	0.2905	2.9234	2.6203	2.4699	1.5702	3.8803	2.2921	1.4153	1.4619	1.8983	2.2869	2.846	3.409	2.946
444	1.5016	1.6289	1.3999	2.5194	2.334	3.2348	2.4534	2.6754	2.3564	1.47	0.5013	2.5714	0.3685	0.728	2.8333	2.5698	1.7373	1.2005	3.2069	2.4153	1.1192	1.8314	1.6526	2.8334	2.5868	3.0333	2.4936
445	1.2069	1.5777	1.8189	1.7552	1.296	2.654	1.1017	2.3543	2.0878	1.1623	1.212	2.1199	0.8814	1.6418	1.9002	2.5892	0.9824	1.4675	1.5974	2.0218	0.8745	2.6376	1.6274	2.6684	1.6271	2.1589	1.6106
446	1.0953	1.4394	2.9427	1.9506	2.022	2.4068	0.8992	2.2645	2.3174	2.089	2.1248	2.161	1.13	2.1178	0.3843	2.6269	1.4287	1.9633	1.7021	1.8016	0.9498	2.3655	2.0174	2.1951	2.1709	2.1338	0.7109
447	0.7209	0.6975	0.4062	0.5694	0.4032	0.7254	0.4455	0.3555	0.7234	0.3442	0.2105	0.6204	0.2629	0.2029	0.3856	0.5878	0.5187	0.5853	0.4595	0.2113	0.356	0.1225	0.3758	0.542	0.6184	0.6632	0.41
448	1.2231	1.1757	0.7766	1.7252	1.1539	1.7129	1.1908	1.6166	1.7055	1.0366	0.5055	1.4813	0.4692	0.3055	1.0989	1.9344	1.2868	1.1676	1.5465	0.7731	1.0488	0.8694	0.9237	1.5074	1.6578	1.7182	1.1903
449	1.3478	1.3739	1.217	2.0562	1.2898	2.0732	1.3319	2.6353	2.0838	1.1749	0.6749	1.83	0.5757	0.5769	1.3527	2.7188	1.4136	1.2805	1.6934	0.9868	1.2572	1.5822	1.3959	2.2315	2.2295	2.1479	1.4383
450	1.7753	1.3974	1.4677	2.3587	1.5743	2.2741	1.2936	3.0705	2.2219	1.1601	0.9289	2.1903	0.7994	0.7544	1.4542	3.0194	1.4681	1.2899	1.6628	1.153	1.4091	2.3906	1.6845	2.4069	2.6163	2.2798	1.3761
451	2.3009	1.4621	1.5988	2.7315	2.1225	2.3856	1.3284	3.2024	2.4359	1.1827	1.1376	2.5337	1.0541	0.8183	1.8028	3.1323	1.6167	1.2473	1.6291	1.1367	1.3669	2.7952	1.9157	2.6025	2.9369	2.3719	1.3294
452	3.1431	1.7702	1.7917	3.27	3.0153	2.4255	1.5694	3.3037	2.9506	1.4735	1.674	2.8669	1.0331	0.9276	2.3913	3.2999	1.7685	1.263	2.261	1.0451	1.238	3.643	2.3045	3.0445	3.3299	2.7767	1.0596
453	3.4898	1.5275	1.7805	4.0128	3.8848	2.6953	2.547	3.4831	3.7306	2.6537	2.279	3.2095	1.8195	1.3528	3.5048	3.4447	2.3443	0.7541	3.9148	1.1479	1.628	4.8308	2.2013	4.381	3.6163	3.7155	1.3388
454	4.4921	2.0665	2.3887	5.222	3.7156	2.7043	3.0388	3.4349	3.0037	2.7216	2.3689	3.3116	0.6295	1.9581	4.4827	3.3843	4.8657	0.1174	4.6828	1.6576	0.6686	5.8788	1.9934	5.3822	3.7409	3.9067	1.569
455	1.3587	1.4019	1.4253	1.3408	1.329	1.3285	1.3648	1.4058	1.2816	1.3962	1.4	1.3541	1.3656	1.3612	1.3555	1.3738	1.3495	1.3631	1.3102	1.3108	1.3428	1.4023	1.4446	1.3071	1.3253	1.3255	1.3454
456	420.17	534.72	460.33	354.18	401.44	399.8	411.06	457.83	354.84	421.58	409.82	449.19	431.21	449.25	388.23	385.06	429.24	481.72	374.36	411.67	415.47	509.77	451.15	379.28	408.49	415.14	430.9
457	570.88	749.63	656.13	474.88	533.5	531.13	561	643.63	454.75	588.63	573.75	608.25	588.88	611.5	526.25	529	579.25	656.63	490.5	539.63	557.88	714.88	651.75	495.75	541.38	550.25	579.75
458	1154	1529.4	1288.4	952.13	1042.6	1160.8	1162.6	1183	969.5	1118.8	977.75	1205.3	1050.9	1118.9	1103.1	990.25	1187.6	1312	1035.8	1088.1	1020.6	1307.9	1230.6	1015.5	1084.5	1202.5	1246.4
459	746.38	993.5	863.88	596.25	678.25	814.88	785.75	690.63	626.13	708.5	565.25	793.25	595.75	661.38	752.13	588.5	780	848.25	703.88	728.63	605.63	769.63	821	639.5	694.88	816.13	852
460	422.5	515.5	474.38	345.5	387.38	514.38	493.75	365.63	355.5	411.13	307.38	453.88	325.13	372.63	461.25	328.25	477.13	489.25	436.75	445.63	351.25	418.75	451.63	355.25	383.13	501.75	519.13
461	169.38	195.5	214.88	148.5	157.63	249.75	241.13	158.25	158.88	185.88	136.75	188.13	134.25	167.63	215.5	150.25	218.5	224.5	200.75	214.75	155.13	172.38	187	157.25	162.88	241.38	234.88
462	67.875	77.875	102.5	62	67.625	119.88	119.25	69.125	68.625	82	65.75	77.375	56.5	76	97.5	68.625	95.75	108.5	81.875	95.125	70.5	73.625	77.25	65.5	75	109.13	105.88
463	25.75	30.125	44.625	23.375	25.375	53	57.25	29.75	26.625	30.25	31.5	28.625	24.875	30.5	39.625	27	37.625	49.5	28.125	41.875	28.625	28.25	29.875	24.5	31.625	43.125	47
464	8.25	12	19	7.375	8.625	17.375	20.75	11.25	10.25	8.625	13.625	9.25	8.625	10.5	12	9.5	10.875	19.875	8	12.875	9.5	7.5	11.125	7	13.625	12.625	18
465	2.5	4.25	6.875	1.625	3.75	4.375	8.875	3.75	4.375	2.625	6.375	2.5	3	2.875	3.375	2.75	2.375	4.75	1.875	3.25	3	1.625	3.875	1.75	5.125	3	5.125
466	936.63	1310.8	1066.3	724.5	785.75	795.63	802.38	941.25	643.88	886.63	752.63	1005.1	820.38	853.63	835.25	755	937.75	1108.9	750.13	773.63	773.13	1028.1	932.38	769	823.63	853.88	861.63
467	479.88	731.75	580.25	300.25	313	320.88	294.38	394.5	266.25	401	280.25	537.13	337.88	365.38	395.75	294.13	457.88	631.63	325.63	330.13	315.13	460.63	432.38	315.75	343	328	355.5
468	226	302.88	225.25	126	126.5	124.25	103.75	138.38	107.75	168.63	99.875	231.13	125.5	135.75	146.63	97	188.38	299	138.75	137.63	122	179	152.25	123.38	129.25	109.13	137.13
469	73.25	78.75	62.375	38.625	38.625	36.5	35.75	37.75	37.625	51	37.375	71.875	33.875	36.375	40.125	28.375	54	101.63	42.25	43.5	32.125	47.75	43.875	40.5	39.625	28.625	39.75
470	24.5	22	17.875	13.5	15	13	16.25	12.625	15.25	16	16.75	20.25	9.125	10.875	13	8.875	19	42	14.25	14.875	9.625	16	12.25	14	15.375	9.25	12.875
471	4.5	2.125	1.875	2.625	4.25	2.625	7.75	2.25	5.25	3.625	8.25	3.375	1.25	1.75	2	1.875	3	15.5	2.875	3.25	2	3	1.875	2.875	5.875	1.75	3.25
472	0.125	0	0	0	0.875	0	3.625	0	1.875	0.25	3.875	0	0	0	0	0	0	5.375	0	0.25	0	0.25	0	0	1.375	0	0
473	0	0	0	0	0.125	0	1.5	0	0.5	0	1.25	0	0	0	0	0	0	0.625	0	0	0	0	0	0	0	0	0

Table S12 Amino acid descriptors (2D) used in this study.

	M	M	M	M	M	M	M	M	M	M	M	M	M	M	M	M	N	N	N	N	N	N	N	N	N	N	N	N	N	N	N	N	N	N	N	N	P	P	P	P	P	P	P	P	P	P	P	P	P	P	P	P	P	P	P
	F	G	H	I	K	L	M	N	P	Q	R	S	T	V	W	Y	A	C	D	E	F	G	H	I	K	L	M	N	P	Q	R	S	T	V	W	Y	A	C	D	E	F	G	H	I	K	L	M	N	P	Q	R	S	T	V	W
No.	MF	MG	MH	MI	MK	ML	MM	MN	MP	MQ	MR	MS	MT	MV	MW	MY	NA	NC	ND	NE	NF	NG	NH	NI	NK	NL	NM	NN	NP	NQ	NR	NS	NT	NV	NW	NY	PA	PC	PD	PE	PF	PG	PH	PI	PK	PL	PM	PN	PP	PQ	PR	PS	PT	PV	PW
1	1	-7	-7	-5	-3	-4	-7	-3	-6	-7	-3	-4	-4	-4	3	-3	0	-2	-3	-3	-6	-4	-6	0	0	-5	-3	-1	-3	-7	-3	1	-4	1	1	-2	6	-3	3	0	-5	-1	-6	0	-2	-2	-6	-3	-3	-2	2	-1	-5	-7	-4
2	0.6	-3.2	-4.7	-1.3	-3.6	-1.2	-3.8	-1.7	-2.6	-3.7	-3.3	-4.1	-2.1	-3.4	3.2	-4.2	-0.6	-0.2	-2.1	-1.5	-0.1	-3.2	-2.6	-0.1	0.1	-4.4	-1.7	-1.7	-1.6	-4.3	-1.7	1.1	-4.1	4	0.8	0.1	5.3	-3.8	3.2	2.5	-1.4	-3.1	-4.4	0.1	-1.7	-1.4	-2.6	-1.6	-3.5	-2.4	2.1	-2.6	-4.6	-3.8	-2.1
3	0.6	-3.3	-4.3	-1.7	-2.4	-1.5	-2.6	-1.2	-3.3	-3.7	-3	-3.4	-2	-3.5	2.4	-4	-0.2	-0.8	-1.6	-1	-0.1	-2.9	-2.7	0.4	0.3	-5.2	-1.2	-0.9	-1.4	-5.5	-1.2	1.2	-4	3.1	0.6	0.3	4.2	-2.7	2.4	2.2	-1.9	-1.7	-3.6	-0.1	-0.7	-0.6	-3.3	-1.4	-2.4	-2	1.7	-1.7	-4.9	-2.6	-2
4	0.8	-2.6	-3.2	-2	-1.2	-1.3	-1.6	0.3	-2.8	-3.2	-2.9	-2.2	-1.6	-3.1	1.4	-3.9	-0.3	-0.8	-1	-0.5	-0.7	-2.4	-2	0.4	0.4	-4	0.3	-0.3	-1.1	-4.1	0.3	1	-3	2.6	0.4	0	3	-2	1.4	1.7	-1.8	-1	-2.8	-0.2	-0.1	0.1	-2.8	-1.1	-1.7	-1.7	1.7	-0.7	-4.6	-1.6	-1.6
5	0.3	-3.1	-3	1	-1.9	-1.6	-1.3	-1.9	-2.3	-1.6	1	-1.8	-2.2	-2.2	3.6	-2.1	1	-2	-2.1	-2.3	1.8	-0.4	-1.9	-2.6	1.7	-3	-1.9	-2.1	-2.1	-2.2	-1.9	0.3	-0.9	3.3	-0.6	-1.1	5.5	-1.9	3.6	2.2	-0.8	-1.2	-2.1	1	-1.7	-2.3	-2.3	-2.1	-2	0.1	2	-2.1	-2.2	-1.3	-2.2
6	0.19	-0.29	-0.51	0.25	0.26	0.18	0.23	0.04	-0.17	-0.24	-0.48	-0.14	0.18	-0.39	0.05	-0.54	-0.08	0.15	-0.11	-0.01	0.3	-0.2	-0.03	0.22	0.22	-0.39	0.04	-0.01	-0.13	-0.25	0.04	0.13	-0.32	0.36	0.29	-0.06	1	-0.43	0.05	0.52	-0.28	-0.18	-0.44	-0.03	-0.15	-0.06	-0.17	-0.13	-0.38	-0.3	0.51	-0.11	-0.14	0.23	0.18
7	2	-5	-5	-3	-2	-3	-6	-2	-5	-5	-2	-2	-2	-3	3	-2	0	-1	-1	-1	-4	-3	-4	0	1	-4	-2	0	-1	-6	-2	1	-4	2	1	-2	4	-2	3	1	-4	-1	-5	0	-1	-1	-5	-1	-2	-2	2	-1	-4	-6	-2
8	4	1	0	3	2	2	2	2	1	1	3	1	1	1	4	1	4	2	2	2	3	2	1	3	3	0	2	3	2	2	2	3	1	4	5	2	6	1	4	4	2	2	1	3	3	3	1	2	2	2	4	3	2	2	1
9	2	2	3	1	2	2	2	2	1	2	1	2	2	2	1	2	1	2	2	2	1	1	2	2	1	3	2	2	2	2	2	2	2	1	1	2	0	3	1	1	1	2	2	1	2	1	1	2	2	1	1	2	1	2	2
10	6	8	5	8	9	9	9	9	4	5	4	4	9	7	9	3	5	9	10	9	8	3	2	10	5	4	9	9	10	5	9	6	3	9	10	7	10	7	9	6	6	8	6	8	9	10	4	10	7	7	6	4	7	9	9
11	0.9	-2.4	-3	-2	-1.1	-1.3	-1.5	0.5	-2.7	-3.2	-2.9	-2	-1.4	-3	1.2	-3.8	-0.3	-0.7	-0.8	-0.3	-0.8	-2.2	-1.8	0.4	0.4	-3.6	0.5	-0.2	-1	-3.6	0.5	0.9	-2.7	2.5	0.4	-0.1	2.7	-1.9	1.2	1.5	-1.7	-1	-2.6	-0.2	0	0.2	-2.7	-1	-1.5	-1.6	1.7	-0.5	-4.4	-1.5	-1.4
12	61	154	170	159	198	174	198	195	130	154	152	126	98	153	24	168	126	84	86	96	205	102	77	80	81	174	195	91	87	148	195	42	122	10	56	100	0	109	24	53	99	101	116	76	42	68	130	87	96	113	29	93	138	198	98
13	0	-3	-3	-3	-3	-3	-2	-1	-2	-3	-3	-2	-2	-3	1	-4	-2	-1	-2	-1	-2	-2	-1	0	0	-4	-1	-1	-2	-2	-1	0	-2	2	0	-2	6	-2	1	1	-1	0	-4	-1	-1	0	-2	-2	-3	-2	2	0	-3	-2	-2
14	0	-4	-5	-4	-2	-4	-2	-1	-3	-5	-5	-3	-3	-5	1	-5	-3	-1	-2	-2	-4	-3	-3	-1	-2	-6	-1	-1	-3	-4	-1	0	-3	2	0	-2	8	-4	1	2	-2	-1	-5	-2	-1	0	-3	-3	-3	-3	3	-2	-5	-2	-3
15	-1	-5	-7	-6	-2	-7	-3	-1	-4	-7	-6	-4	-4	-6	1	-7	-3	-2	-3	-3	-4	-4	-4	-1	-2	-7	-1	-2	-4	-5	-1	-1	-5	2	-1	-2	9	-5	1	2	-3	-1	-5	-3	-1	-1	-4	-4	-4	-4	3	-2	-7	-3	-4
16	-1.1	-6.9	-6.9	-8.2	-7.7	-8.2	-8.7	-3.4	-8.2	-9.7	-5.2	-2.8	-3.2	-4.8	1.4	-4.8	-1.6	-1.5	-3.1	-3.4	-4.4	-3.7	-2.1	-1.4	-0.7	-6.1	-3.4	-0.6	-2.8	-5.8	-3.4	-0.7	-3.7	2.6	-1.3	-1.7	9	-7	1.4	1.1	-5.1	-0.6	-6.4	-3.6	-0.4	-1.2	-8.2	-2.8	-3.6	-4.4	2.4	-1.5	-7.2	-8.7	-3.2
17	1	-3	-4	-1	-2	0	-3	-1	-3	-3	-2	-3	-2	-3	2	-3	0	-1	-2	-1	0	-3	-2	0	0	-5	-1	-1	-1	-4	-1	1	-4	3	1	0	5	-3	2	2	-2	-2	-4	0	-1	-1	-3	-1	-3	-2	2	-2	-4	-3	-2
18	2	-3	-3	-1	-1	-1	-1	0	0	-3	-3	-3	-3	-4	7	-3	0	-1	-3	1	1	-3	-1	-2	3	-3	0	-2	-1	-4	0	1	-5	1	1	-3	11	-4	7	6	0	-2	-4	0	-2	-1	0	-1	-4	-2	7	-3	-4	-1	-3
19	0	0	0	0	0	9	0	0	5	0	0	0	0	0	5	0	0	5	0	0	5	6.6	5	0	6.6	0	0	6.6	0	0	0	0	0	0	0	5	10	0	5	9	5	5	6.6	0	5	0	5	0	0	9	6.6	0	0	0	0
20	0	0	0	0	0	0	0	0	-1	0	-1	-1	0	-1	0	-1	0	0	0	0	-1	-1	-1	0	0	-1	0	0	0	-1	0	0	-1	0	0	-1	2	-1	0	0	-1	-1	-1	0	0	0	-1	0	-1	-1	1	-1	-1	0	0
21	2	-10	-12	-15	-13	-23	-1	-5	-13	-13	-7	-4	-13	-4	2	-7	1	1	-4	-2	-12	-4	-3	-4	-5	-6	-5	2	-8	-6	-5	2	-10	20	-6	-6	16	-3	2	5	-9	0	-15	-6	1	2	-13	-8	-2	-2	5	-3	-17	-1	-13
22	8	-5	-16	-36	-17	-20	-35	1	-2	-14	-4	-12	-14	-10	4	-6	6	-6	-2	8	-36	-10	3	5	5	-6	1	6	3	-20	1	8	-3	18	7	-10	7	-4	4	10	0	-5	-20	-7	8	8	-2	3	-3	-12	9	-7	-32	-35	-14
23	3	2	-3	4	4	0	-3	2	-3	-1	-4	-5	0	-12	2	-9	-2	-3	-5	0	4	-7	-10	4	1	-18	2	-1	2	-20	2	1	-21	17	5	-9	13	-4	2	-1	-12	0	-13	1	2	2	-3	2	-5	-13	3	-1	-15	-3	0
24	10	0	-2	3	3	-30	-11	8	-15	-13	-15	-3	-32	-6	13	-13	-1	-2	-30	5	-12	-3	-3	7	7	-34	8	2	5	-50	8	5	-8	3	7	-7	6	0	13	9	0	-1	-14	2	3	4	-15	5	-2	-7	7	4	-21	-11	-32
25	3	-29	-30	-33	-26	-33	-34	-22	-34	-26	-13	-5	-4	-11	4	-10	-4	2	-11	0	12	-8	-13	-4	12	-30	-22	-3	3	-2	-22	0	-13	10	0	-3	30	-7	4	4	-14	-5	-9	1	2	-6	-34	3	-8	-13	6	-10	-21	-34	-4
26	18	7	-1	-10	-29	-2	-29	0	-14	0	-30	5	-3	-15	13	-22	4	1	2	-1	-40	-12	7	6	24	-11	0	6	4	-23	0	22	14	3	16	6	42	-14	13	22	9	4	-16	-17	4	10	-14	4	-15	-9	15	-19	-9	-29	-3
27	4	-11	-15	-21	-13	-23	-10	-5	-13	-15	-7	-7	-12	-7	2	-9	2	-1	-3	1	-19	-7	-1	-2	-3	-7	-5	3	-6	-9	-5	3	-9	22	-4	-9	14	-4	2	6	-6	-2	-18	-6	2	3	-13	-6	-3	-6	6	-3	-22	-10	-12
28	6	1	-5	4	5	-5	-8	4	-19	-4	-9	-7	-1	-14	4	-12	0	-4	-6	3	-4	-10	-8	6	3	-38	4	1	3	-43	4	5	-16	12	6	-13	11	-6	4	4	-8	-2	-19	5	3	4	-19	3	-7	-14	7	6	-19	-8	-1
29	9	-11	-21	-18	-30	-12	-34	-5	-26	-16	-18	-3	-5	-13	8	-17	-1	1	-4	2	-31	-10	-5	0	17	-22	-5	0	4	-17	-5	8	-3	8	8	3	31	-13	8	12	-4	-3	-12	-2	4	2	-26	4	-11	-12	11	-13	-15	-34	-5
30	4	0	0	1	1	3	0	1	2	1	1	1	2	1	4	0	3	3	3	1	0	2	2	3	4	0	1	3	2	1	1	4	2	5	3	4	8	0	4	4	1	3	0	3	3	4	2	2	2	3	5	4	1	0	2
31	1	0	0	0	0	0	0	0	0	0	0	0	0	0	2	0	0	0	0	0	0	0	0	0	0	0	0	0	0	0	0	1	0	3	0	2	5	0	2	1	0	0	0	0	0	1	0	0	0	0	3	0	0	0	0
32	0.16	-0.74	-0.64	-0.18	-0.13	-0.29	-0.02	-0.51	-0.83	-0.41	-0.39	-0.87	-0.19	-0.9	0.37	-0.78	0.04	-0.47	-0.21	-0.06	0.39	-0.74	-0.31	-0.11	0.18	-0.79	-0.51	-0.16	-0.14	-0.51	-0.51	0.19	-0.11	0.67	0	-0.43	0.48	-0.95	0.37	0.3	-0.02	-0.97	-0.85	0.15	-0.07	-0.2	-0.83	-0.14	-0.68	-0.74	0.25	-0.13	-0.56	-0.02	-0.19
33	1.47	2.48	3.26	2.22	1.63	2.56	1.65	1.39	3.42	3.48	3.4	3.13	2.78	3.49	0.32	3.98	2.37	2.42	2.17	2.34	2.24	2.62	2.02	1.96	1.72	4.39	1.39	1.92	2.48	4.23	1.39	0.85	3.22	0.29	0.85	2.63	0	2.57	0.32	1.06	2.48	2.3	2.81	1.92	1.12	1.65	3.42	2.48	2.13	2.7	0.84	2.48	3.67	1.65	2.78
34	11	10	9	8	8	10	11	11	9	8	3	8	7	7	11	3	9	11	11	10	10	6	7	10	9	8	11	11	8	11	11	10	6	9	11	9	16	6	11	12	8	9	7	7	10	10	9	8	6	9	11	5	6	11	7
35	0.11	-0.16	-0.34	0.12	-0.15	-0.04	-0.27	-0.35	-0.17	-0.1	-0.22	0.36	0.06	-0.23	0.08	-0.28	0.05	0.39	-0.18	-0.08	-0.05	0	-0.01	0.37	-0.01	-0.21	-0.35	0.32	-0.28	-0.11	-0.35	-0.24	-0.57	0.15	0.28	0.17	1	-0.13	0.08	0.16	-0.28	0.22	-0.25	-0.18	-0.21	-0.23	-0.17	-0.28	-0.03	0.29	0.39	-0.08	-0.54	-0.27	0.06
36	0.13	-0.16	-0.36	0.13	-0.16	-0.01	-0.29	-0.38	-0.18	-0.1	-0.25	0.38	0.09	-0.24	0.12	-0.29	0.07	0.43	-0.2	-0.08	-0.07	0.03	-0.01	0.4	0	-0.24	-0.38	0.36	-0.32	-0.11	-0.38	-0.27	-0.62	0.18	0.28	0.2	1	-0.15	0.12	0.23	-0.32	0.22	-0.26	-0.18	-0.22	-0.22	-0.18	-0.32	-0.04	0.35	0.42	0.07	-0.56	-0.29	0.09
37	0	-3	-3	-6	-4	-6	-6	-2	-5	-7	-4	-2	-3	-3	0	-3	-1	0	-2	-2	-2	-3	-1	-1	0	-5	-2	0	-2	-3	-2	0	-2	1	-1	-2	6	-5	0	1	-3	1	-4	-2	0	-1	-5	-2	-2	-3	2	-1	-5	-6	-3
38	0.321	0.191	0.341	0.246	-0.029	0.527	0.501	0.27	0.515	0.035	0.149	0.128	0.497	0.191	0.63	0.242	0.299	0.207	0.356	-0.109	0.55	0.446	0.201	0.293	-0.117	0.277	0.27	0.261	0.494	0.081	0.27	0.206	0.273	0.44	0.015	0.405	1	0.372	0.63	0.323	0.207	0.211	0.364	-0.168	0.344	0.404	0.515	0.494	0.334	0.251	0.228	-0.216	0.327	0.501	0.497
39	0.257	0.418	-0.167	0	0.717	0.425	0.787	0.799	0.478	0.015	-0.196	-0.18	0	-0.172	0.614	-0.185	-0.018	0.858	0.523	-0.054	0.857	0.049	0.266	0	0.067	0.003	0.799	0.55	0	0.825	0.799	0.408	0.036	0.842	0	0.511	1	0.511	0.614	0.377	0.55	0.45	0.482	0.598	0.61	0.357	0.478	0	0.458	0.439	0.254	0.097	0	0.787	0
40	2	3	0	1	4	4	5	5	2	0	0	0	0	0	4	0	0	5	4	-1	6	0	0	2	0	0	5	2	2	4	5	3	1	5	1	4	8	2	4	1	2	2	3	2	3	2	2	2	3	3	2	0	1	5	0
41	0.6	-1.4	-1.5	-1.7	-1.6	-1.8	-1.5	-1.5	-1	-1.7	0	0.6	-1.2	0.8	-0.5	0	0.2	1	-0.6	-0.1	-1.6	1.2	0.8	0.2	-1.3	-1.1	-1.5	1.8	0.2	-0.9	-1.5	1.4	0.2	0.9	0.7	-1.1	2.2	1.4	-0.5	1.7	0.5	1.2	0.7	-0.6	1.7	1.8	-1	0.2	1.5	1.1	2.1	-0.1	-0.2	-1.5	-1.2
42	8	-8	-18	-1	12	3	15	4	-3	-20	-32	-17	-8	-35	-4	-39	3	-2	-10	2	11	-24	0	10	10	-6	4	0	-10	-12	4	13	-28	18	9	-10	100	-20	-4	26	-23	-12	-22	0	-10	-7	-3	-10	-12	-26	37	-7	-16	15	-8
43	16	-10	-8	1	2	-2	1	5	-3	-8	-13	1	6	-9	6	-14	0	2	-2	13	2	-8	-1	8	8	-5	5	0	0	-2	5	10	-1	2	8	-1	21	-12	6	22	-3	-2	-10	1	6	6	-3	0	-12	-9	17	5	-9	1	6
44	13	-9	-6	0	4	-1	2	2	-5	-8	-12	0	5	-9	2	-12	0	1	-2	9	3	-7	-3	7	4	-4	2	1	0	-1	2	8	-1	3	6	-1	17	-12	2	16	-4	-3	-10	0	2	4	-5	0	-12	-9	13	0	-8	2	5
45	68	62	36	94	93	69	95	98	63	30	26	27	75	52	93	23	33	89	71	81	89	6	19	64	61	58	98	64	68	98	98	79	44	98	98	58	100	55	93	71	70	53	51	76	67	71	63	68	58	57	74	50	89	95	75
46	-1	0	-2	0	0	0	0	0	0	-2	-3	-1	-1	-1	0	-4	-2	1	0	-1	0	-1	-1	0	-1	-1	0	0	0	0	0	0	-4	2	0	0	4	0	0	0	1	1	0	0	1	0	0	0	-1	0	0	-1	-1	0	-1
47	-1	3	2	3	1	3	1	0	2	1	0	1	1	2	0	-1	0	2	2	1	2	1	1	0	0	3	0	1	2	3	0	0	-2	1	0	1	-1	3	0	-1	2	1	3	1	2	0	2	2	1	2	-2	0	3	1	1
48	1.25	1.49	1.43	1.36	1.24	1.5	1.41	1.41	0.95	1.44	1.01	1.02	1.1	0.98	1.22	0.93	1.31	1.2	1.17	1.18	1.16	1.02	0.75	0.96	1.05	0.88	1.41	1.31	1.11	0.9	1.41	1.21	0.76	1.01	0.94	0.94	1.16	1.02	1.22	1.21	0.85	1.1	0.88	0.91	1.02	1.09	0.95	1.11	1.06	1.01	1.26	0.96	0.88	1.41	1.1
49	0.64	0.65	0.69	0.8	0.96	0.69	0.84	0.75	1.17	0.67	0.99	0.96	0.71	0.97	0.79	1.06	0.56	0.97	0.79	0.63	1.04	1.05	1.25	0.71	0.68	0.95	0.75	0.68	0.78	1.18	0.75	0.74	1.31	1.16	0.91	1.05	0.8	1.1	0.79	0.75	1.11	0.97	1.07	0.83	1.02	0.85	1.17	0.78	1.03	1.07	0.73	0.75	1.09	0.84	0.71
50	1.17	0.51	0.57	0.51	0.43	0.39	0.28	0.49	0.73	0.6	0.99	1.02	1.4	1.13	0.92	1.04	1.23	0.53	1.04	1.41	0.46	0.82	1.1	1.8	1.63	1.46	0.49	0.92	1.22	0.86	0.49	1.04	0.91	0.59	1.37	1.05	0.96	0.7	0.92	1.02	1.14	0.79	1.16	1.63	0.89	1.12	0.73	1.22	0.77	0.8	0.96	1.71	1.1	0.28	1.4
51	22.2	13.8	12.2	15	20.9	13.5	15.5	21.5	16.6	14	15.1	15.2	12.2	11.7	21.2	13.7	18.2	16.2	11.5	18.2	13.5	12.2	13.8	18.1	17.2	7.74	21.5	17.2	16.3	10.1	21.5	20	12.7	20.9	20.2	10.7	96.5	15.8	21.2	22	14.6	17.8	11.5	18	17.4	17.6	16.6	16.3	15.2	13.3	20.4	19.1	11.5	15.5	12.2
52	6.1	2.8	2.2	3.2	4.1	3.9	3.7	5.7	2.5	2	2.3	3.2	3.8	2.2	6.4	1.4	4.9	4.5	4.4	4.9	4.4	3	3.4	5.6	5.6	1.6	5.7	5	4.2	1.6	5.7	6.1	2.5	7.7	5.6	5.1	7.9	3.3	6.4	6.7	3.5	4.2	2.6	5	5.2	5.4	2.5	4.2	3.7	3.6	6.9	4.7	0.8	3.7	3.8
53	1	-14	-20	-15	-3	-18	-11	-5	-12	-13	-10	-6	-2	-7	2	-9	0	-2	-2	-2	-12	-4	-1	1	1	-9	-5	1	-3	-11	-5	1	-5	10	0	-1	13	-7	2	4	-5	-8	-4	-4	2	0	-12	-3	-2	-10	5	-1	-6	-11	-2
54	0.34	-0.83	-1.97	-2.11	0.13	-1.5	-0.31	0.33	-0.81	-2.51	-2.02	-1.86	-0.4	-2.1	-0.46	-2.77	-0.73	-0.04	-1.42	0.13	1.13	-1.19	-0.75	0.79	-0.01	-2.89	0.33	-0.75	-0.12	-0.66	0.33	-0.16	-1.64	0.99	0.29	-0.22	2.6	-1.47	-0.46	0.92	-0.61	-0.13	-2.31	0.24	0.6	1.04	-0.81	-0.12	-1.38	-1.49	1.23	-0.26	-1.98	-0.31	-0.4
55	1.1	-2.47	-4.7	-5.7	-0.13	-5.95	-0.82	0.45	-4.3	-6.98	-6.1	-3.94	-1.24	-5.64	-2.24	-6.18	-2.38	0.16	-3.01	-0.33	1.72	-2.83	-1.01	1.16	0.32	-6.29	0.45	-2.16	-0.24	-2.61	0.45	-0.39	-4.5	2.94	0.63	0.25	7.85	-3.26	-2.24	3.24	-1.73	-1.76	-5.54	1.3	1.08	2.59	-4.3	-0.24	-4.97	-4.56	3.16	-0.43	-6.55	-0.82	-1.24
56	1.1	-2.5	-2.4	-1.3	-3.2	-1.7	-1.6	1.2	-2.9	-3.1	-2.5	-2	-1.7	-2.4	1.4	-2.9	1	-0.2	-1.4	0.1	-0.8	-1.5	-0.9	0.7	0.5	-4	1.2	-0.1	-0.3	-3.3	1.2	2.3	-2.2	2.2	1.6	-0.9	3.6	-0.9	1.4	2.2	-1.5	-0.6	-1.7	1	-0.1	0.9	-2.9	-0.3	-1.5	-1.2	1.7	0	-3.8	-1.6	-1.7
57	6	-4	-6	0	4	-2	2	2	-2	-4	-9	-2	3	-7	1	-9	0	0	-1	4	3	-5	-2	5	3	-4	2	0	0	0	2	6	-2	3	4	-2	7	-7	1	7	-1	-2	-5	1	1	2	-2	0	-8	-5	8	0	-6	2	3
58	3	2	-8	-1	-1	5	4	2	-1	0	-2	-3	-5	0	0	-4	0	4	-1	3	-1	-2	3	3	2	-3	2	1	-1	-4	2	2	-2	1	4	0	0	1	0	3	2	-1	-6	1	3	0	-1	-1	2	-2	6	1	-2	4	-5
59	3	-6	-4	-1	1	1	2	2	-3	-1	-3	-3	-1	-2	4	-7	-1	0	-2	0	3	-2	2	1	2	0	2	-1	0	-1	2	0	-1	5	0	0	4	-3	4	4	1	0	-2	0	-2	4	-3	0	-1	-5	2	2	-4	2	-1
60	3	-4	-8	0	-4	2	2	2	-1	0	-1	-1	-3	0	0	-3	-1	2	0	0	3	-2	3	2	3	-1	2	0	0	0	2	0	-1	4	0	0	0	0	0	4	0	0	-3	0	0	1	-1	0	0	-2	4	1	-2	2	-3
61	-7.3	-8.3	-10.2	-7.5	-9	-9.3	-7.7	-7.7	-8.3	-8.3	-7.1	-8.1	-7.7	-7.7	-7.7	-8.3	-6.9	-8	-7.7	-7.3	-8.9	-7.9	-8.4	-6.8	-8.1	-8.6	-7.7	-7	-7.6	-9	-7.7	-6.5	-7.5	-7.4	-6.3	-8.2	-6.5	-7.9	-7.7	-6.7	-7.3	-8.5	-8.6	-8.1	-7	-7	-8.3	-7.6	-7.5	-7.4	-6.8	-7.8	-8	-7.7	-7.7
62	2	-30	-39	-29	-6	-29	-6	6	-19	-30	-31	-21	-18	-32	3	-34	-20	-9	-19	-18	-18	-20	-15	7	-7	-39	6	-3	-20	-29	6	3	-21	12	2	-8	46	-30	3	15	-9	1	-29	-11	-7	0	-19	-20	-19	-23	19	-7	-42	-6	-18
63	0	-5	-7	-2	-3	-7	-2	1	1	-7	-5	-5	-4	-3	2	-5	-5	-1	-3	-7	0	-6	-6	-1	-1	-5	1	-3	-2	-4	1	0	-2	3	1	-2	9	-3	2	-1	0	-1	-2	-3	-3	-1	1	-2	-3	-3	1	-5	-2	-2	-4
64	-2.5	-3.5	-7	-1.5	-0.5	-5	1	2.5	1	-7.5	-6	-6	-5.5	-3.5	-0.5	-6.5	-6.5	0	-3	-9.5	2.5	-4.5	-5.5	-3	-3.5	-4	2.5	-4	-3.5	-2.5	2.5	-4.5	-5.5	4	0.5	-1	7	-3.5	-0.5	-2	-2	-1.5	-2	-6	-3.5	-2.5	1	-3.5	-3.5	-3.5	-2.5	-7	-2	1	-5.5
65	1	-4	-5	-2	-1	-2	-4	1	-6	-5	-4	-3	-3	-4	2	-6	-1	-1	-2	-3	-4	-3	-3	0	0	-5	1	-1	-3	-5	1	0	-3	2	0	0	6	-4	2	2	-4	-1	-4	-1	-1	0	-6	-3	-3	-2	2	-1	-6	-4	-3
66	1	-2	-3	-1	0	-1	-2	1	-4	-3	-3	-2	-1	-3	1	-4	0	-1	-1	-1	-2	-2	-2	0	0	-4	1	0	-1	-3	1	1	-2	2	0	0	3	-2	1	2	-2	-1	-2	0	0	0	-4	-1	-2	-2	2	0	-4	-2	-1
67	0	-3	-4	-3	-1	-3	-2	0	-2	-4	-5	-3	-2	-4	0	-6	-2	-1	-2	-1	-2	-3	-2	-1	-1	-3	0	-1	-2	-3	0	0	-3	1	-1	-1	6	-3	0	1	-2	-2	-3	-1	-1	0	-2	-2	-3	-3	1	-1	-5	-2	-2
68	0.009	0.055	0.047	0.007	0.014	0.029	0.025	0.043	0.002	0.015	0.016	0.757	0.097	0.002	0.036	0.015	0.045	0.011	0.013	0.064	0.023	0.054	0.006	0.029	0.004	0.038	0.043	0.069	0.005	0.001	0.043	0.012	0.001	0.012	0.021	0.019	0.034	0.021	0.036	0.036	0.011	0.006	0.023	0.011	0.023	0.072	0.002	0.005	0.008	0.039	0.033	0.092	0.07	0.025	0.097
69	-2.38	-2.26	-0.76	-5.59	-10.75	-5.77	-9.36	-0.71	-6.74	-1.9	3.12	8.91	3.2	-7.43	-1.19	-2.2	-6.26	-7.82	-2.29	-4	-8.27	-1.08	-5.32	-7.13	-7.3	-0.24	-0.71	-5.11	-6.89	-7.17	-0.71	-1.92	-5.59	-8.6	-0.89	4.11	-4.35	-9.48	-1.19	-0.39	-3.13	-12.59	-7.21	-7.32	-3.58	0.74	-6.74	-6.89	-11.11	-5.3	-1.47	0.13	3.82	-9.36	3.2
70	1.8	5.5	2.9	1.7	-0.4	1.5	-0.6	4.5	-1.3	1.9	6.3	16.3	1.9	-2.4	0.3	2	-1	-1.4	0.7	0.9	-0.4	1	2.1	1.8	1	0.6	4.5	1.8	0.4	-1.9	4.5	-1.6	-0.6	-1.6	-1.9	4.6	2.3	-1.1	0.3	-0.1	-0.4	-4.9	0.5	2.6	-2.2	4.1	-1.3	0.4	-2.2	0.7	1	3.1	2.2	-0.6	1.9
71	-2.19	0.48	1.22	-2.34	-4.39	-1.9	-4.39	-0.7	-2.9	-2.14	2.56	13.84	3.48	-3.75	-0.19	0.53	-1.66	-3.58	-1.15	-0.02	-0.95	-0.27	-1.9	-2.76	-3.36	1.17	-0.7	0.01	-2.93	-3.47	-0.7	-0.6	-2.46	-1.2	-2.17	0.87	-1.67	-5.08	-0.19	1.13	-2.21	-3.34	-0.79	-2.74	-1.56	2.32	-2.9	-2.93	-3.78	-2.11	1.1	2.36	3.25	-4.39	3.48
72	-0.4	-3.9	2	0.1	-0.2	-0.5	-0.9	0.5	0.3	-0.2	-0.2	0	-1.3	0	-2.1	-0.3	-5.7	-0.1	1.3	1.8	0	-1.5	0	0.5	0.7	0.1	0.5	-0.4	-0.5	-0.5	0.5	0.9	-0.3	-0.3	0	-0.6	2.4	-0.5	-2.1	-0.7	0	0.2	-0.9	-1.4	-0.8	1.5	0.3	-0.5	0.3	1	0.9	-1.9	1.3	-0.9	-1.3
73	-2	0	1	-2	-3	-1	-3	-2	-3	-3	0	13	2	-4	-1	1	-1	-3	-1	0	-1	0	-1	-3	-3	0	-2	1	-3	-4	-2	-2	-3	-3	-3	-1	-2	-4	-1	-1	-2	-4	-1	-2	-3	2	-3	-3	-3	-2	0	2	2	-3	2
74	1	-7	-8	-6	-2	-9	-6	9	-4	-8	-15	-10	-5	1	-5	0	7	11	9	-12	-4	-7	-6	-9	-5	-14	9	8	-7	13	9	-4	-5	-8	-1	-3	-4	-7	-5	-2	-7	-11	2	-5	-1	0	-4	-7	-15	-7	-4	0	-5	-6	-5
75	9.2	-9	-5.8	9.8	-10.1	12.7	20.3	11.4	24.6	-12.3	-4.6	23.2	-15	-4.4	-5.6	-5.7	-6	39.2	-5.7	-7.1	9.1	-3	22.1	-7.3	16.1	-5.3	11.4	10	11.1	-7.7	11.4	-7.9	-10.4	21.5	9	2	-2.8	-13.3	-5.6	-10.3	-7.1	27.2	-10.4	8.9	17	0.8	24.6	11.1	-1.4	-5.7	6.8	-7.1	-7.3	20.3	-15
76	4.5	0.3	0.1	0.1	0.1	0.1	0.1	1.5	1.8	1.6	0.1	5.4	0.1	0.1	4.4	0.1	3.9	5.2	0.3	1.4	1	0.6	0.1	2.3	1.8	0.1	1.5	8.4	0.4	0.3	1.5	1.5	1.1	7.4	5	0.1	47.5	0.4	4.4	3.7	0.1	2	0.8	0.6	0.4	0.4	1.8	0.4	0.1	1.1	4.9	0.6	0.2	0.1	0.1
77	4.5	0.3	0.1	0.1	0.1	0.1	0.1	1.5	1.8	1.6	0.1	5.4	0.1	0.1	4.4	0.1	3.9	5.2	0.3	1.4	1	0.6	0.1	2.3	1.8	0.1	1.5	8.4	0.4	0.3	1.5	1.5	1.1	7.4	5	0.1	47.5	0.4	4.4	3.7	0.1	2	0.8	0.6	0.4	0.4	1.8	0.4	0.1	1.1	4.9	0.6	0.2	0.1	0.1
78	0.4	1.5	0.1	7.4	3	9.3	44	3.2	0.1	2	4.3	0.1	1	0.1	0.1	0.2	0.2	1.5	7.3	1.5	0.3	2.3	7.5	0.1	3.5	0.1	3.2	48.2	0.5	2	0.5	1.6	2.5	0.7	0.1	0.1	1.7	0.1	3.1	0.6	0.1	0.1	1	0.8	2.7	1.5	0.1	0.5	65.9	0.6	1	0.5	0.2	0.2	0.1
79	0.4	1.5	0.1	7.4	3	9.3	44	3.2	0.1	2	4.3	0.1	1	0.1	0.1	0.2	0.2	1.5	7.3	1.5	0.3	2.3	7.5	0.1	3.5	0.1	3.2	48.2	0.5	2	0.5	1.6	2.5	0.7	0.1	0.1	1.7	0.1	3.1	0.6	0.1	0.1	1	0.8	2.7	1.5	0.1	0.5	65.9	0.6	1	0.5	0.2	0.2	0.1
80	0.4	1.5	0.1	7.4	3	9.3	44	3.2	0.1	2	4.3	0.1	1	0.1	0.1	0.2	0.2	1.5	7.3	1.5	0.3	2.3	7.5	0.1	3.5	0.1	3.2	48.2	0.5	2	0.5	1.6	2.5	0.7	0.1	0.1	1.7	0.1	3.1	0.6	0.1	0.1	1	0.8	2.7	1.5	0.1	0.5	65.9	0.6	1	0.5	0.2	0.2	0.1
81	0.4	1.5	0.1	7.4	3	9.3	44	3.2	0.1	2	4.3	0.1	1	0.1	0.1	0.2	0.2	1.5	7.3	1.5	0.3	2.3	7.5	0.1	3.5	0.1	3.2	48.2	0.5	2	0.5	1.6	2.5	0.7	0.1	0.1	1.7	0.1	3.1	0.6	0.1	0.1	1	0.8	2.7	1.5	0.1	0.5	65.9	0.6	1	0.5	0.2	0.2	0.1
82	0.4	1.5	0.1	7.4	3	9.3	44	3.2	0.1	2	4.3	0.1	1	0.1	0.1	0.2	0.2	1.5	7.3	1.5	0.3	2.3	7.5	0.1	3.5	0.1	3.2	48.2	0.5	2	0.5	1.6	2.5	0.7	0.1	0.1	1.7	0.1	3.1	0.6	0.1	0.1	1	0.8	2.7	1.5	0.1	0.5	65.9	0.6	1	0.5	0.2	0.2	0.1
83	0.4	1.5	0.1	7.4	3	9.3	44	3.2	0.1	2	4.3	0.1	1	0.1	0.1	0.2	0.2	1.5	7.3	1.5	0.3	2.3	7.5	0.1	3.5	0.1	3.2	48.2	0.5	2	0.5	1.6	2.5	0.7	0.1	0.1	1.7	0.1	3.1	0.6	0.1	0.1	1	0.8	2.7	1.5	0.1	0.5	65.9	0.6	1	0.5	0.2	0.2	0.1
84	0.4	1.5	0.1	7.4	3	9.3	44	3.2	0.1	2	4.3	0.1	1	0.1	0.1	0.2	0.2	1.5	7.3	1.5	0.3	2.3	7.5	0.1	3.5	0.1	3.2	48.2	0.5	2	0.5	1.6	2.5	0.7	0.1	0.1	1.7	0.1	3.1	0.6	0.1	0.1	1	0.8	2.7	1.5	0.1	0.5	65.9	0.6	1	0.5	0.2	0.2	0.1
85	0.4	1.5	0.1	7.4	3	9.3	44	3.2	0.1	2	4.3	0.1	1	0.1	0.1	0.2	0.2	1.5	7.3	1.5	0.3	2.3	7.5	0.1	3.5	0.1	3.2	48.2	0.5	2	0.5	1.6	2.5	0.7	0.1	0.1	1.7	0.1	3.1	0.6	0.1	0.1	1	0.8	2.7	1.5	0.1	0.5	65.9	0.6	1	0.5	0.2	0.2	0.1
86	0.4	1.5	0.1	7.4	3	9.3	44	3.2	0.1	2	4.3	0.1	1	0.1	0.1	0.2	0.2	1.5	7.3	1.5	0.3	2.3	7.5	0.1	3.5	0.1	3.2	48.2	0.5	2	0.5	1.6	2.5	0.7	0.1	0.1	1.7	0.1	3.1	0.6	0.1	0.1	1	0.8	2.7	1.5	0.1	0.5	65.9	0.6	1	0.5	0.2	0.2	0.1
87	0.4	1.5	0.1	7.4	3	9.3	44	3.2	0.1	2	4.3	0.1	1	0.1	0.1	0.2	0.2	1.5	7.3	1.5	0.3	2.3	7.5	0.1	3.5	0.1	3.2	48.2	0.5	2	0.5	1.6	2.5	0.7	0.1	0.1	1.7	0.1	3.1	0.6	0.1	0.1	1	0.8	2.7	1.5	0.1	0.5	65.9	0.6	1	0.5	0.2	0.2	0.1
88	0.4	1.5	0.1	7.4	3	9.3	44	3.2	0.1	2	4.3	0.1	1	0.1	0.1	0.2	0.2	1.5	7.3	1.5	0.3	2.3	7.5	0.1	3.5	0.1	3.2	48.2	0.5	2	0.5	1.6	2.5	0.7	0.1	0.1	1.7	0.1	3.1	0.6	0.1	0.1	1	0.8	2.7	1.5	0.1	0.5	65.9	0.6	1	0.5	0.2	0.2	0.1
89	0.4	1.5	0.1	7.4	3	9.3	44	3.2	0.1	2	4.3	0.1	1	0.1	0.1	0.2	0.2	1.5	7.3	1.5	0.3	2.3	7.5	0.1	3.5	0.1	3.2	48.2	0.5	2	0.5	1.6	2.5	0.7	0.1	0.1	1.7	0.1	3.1	0.6	0.1	0.1	1	0.8	2.7	1.5	0.1	0.5	65.9	0.6	1	0.5	0.2	0.2	0.1
90	0.4	1.5	0.1	7.4	3	9.3	44	3.2	0.1	2	4.3	0.1	1	0.1	0.1	0.2	0.2	1.5	7.3	1.5	0.3	2.3	7.5	0.1	3.5	0.1	3.2	48.2	0.5	2	0.5	1.6	2.5	0.7	0.1	0.1	1.7	0.1	3.1	0.6	0.1	0.1	1	0.8	2.7	1.5	0.1	0.5	65.9	0.6	1	0.5	0.2	0.2	0.1
91	0.009	0.003	0.01	0.018	0.021	0.021	0.018	0.031	0.011	0.057	0.035	0.02	0.022	0.007	0.014	0.002	0.195	0	0.089	0.053	0.009	0.049	0.017	0.016	0.028	0.036	0.031	0	0.045	0.023	0.005	0.038	0.044	0.006	0.007	0.002	0.424	0.013	0.016	0.039	0.011	0.009	0.002	0	0	0.025	0.011	0.045	0.039	0.011	0.021	0.014	0.178	0.012	0.013
92	0.009	0.003	0.01	0.018	0.021	0.021	0.018	0.031	0.011	0.057	0.035	0.02	0.022	0.007	0.014	0.002	0.195	0	0.089	0.053	0.009	0.049	0.017	0.016	0.028	0.036	0.031	0	0.045	0.023	0.005	0.038	0.044	0.006	0.007	0.002	0.424	0.013	0.016	0.039	0.011	0.009	0.002	0	0	0.025	0.011	0.045	0.039	0.011	0.021	0.014	0.178	0.012	0.013
93	0.009	0.003	0.01	0.018	0.021	0.021	0.018	0.031	0.011	0.057	0.035	0.02	0.022	0.007	0.014	0.002	0.195	0	0.089	0.053	0.009	0.049	0.017	0.016	0.028	0.036	0.031	0	0.045	0.023	0.005	0.038	0.044	0.006	0.007	0.002	0.424	0.013	0.016	0.039	0.011	0.009	0.002	0	0	0.025	0.011	0.045	0.039	0.011	0.021	0.014	0.178	0.012	0.013
94	0.009	0.003	0.01	0.018	0.021	0.021	0.018	0.031	0.011	0.057	0.035	0.02	0.022	0.007	0.014	0.002	0.195	0	0.089	0.053	0.009	0.049	0.017	0.016	0.028	0.036	0.031	0	0.045	0.023	0.005	0.038	0.044	0.006	0.007	0.002	0.424	0.013	0.016	0.039	0.011	0.009	0.002	0	0	0.025	0.011	0.045	0.039	0.011	0.021	0.014	0.178	0.012	0.013
101	-6.6	-5.1	-5.4	-7.4	-4.7	-6.3	-5.8	-4.2	-5.1	-3.5	-5	-4.1	-4.6	-6	-6.9	-6.1	-3.1	-4.9	-3.1	-3.4	-5	-4	-4.4	-5.8	-3.3	-4.6	-4.2	-3.2	-3.6	-3.8	-4.1	-3.1	-3.5	-4.3	-5.3	-5	-3.4	-5.3	-3.3	-3.5	-5.2	-4.2	-4.5	-6	-3.6	-4.8	-5.1	-3.6	-3.5	-4	-4.2	-3.4	-3.6	-4.7	-5.6
102	20	32	7	44	21	58	6	7	16	1	9	14	16	52	17	19	39	21	31	27	27	80	25	51	39	63	7	22	22	30	34	42	46	50	21	50	33	24	23	17	19	62	15	41	34	46	16	22	11	23	20	38	32	58	19
103	-1.217	0	-0.443	-1.156	11.998	-1.121	-0.781	-0.202	-1.726	-0.173	6.049	-0.101	-0.433	-0.874	-1.308	-1.155	0.01	0.245	1.921	1.835	-0.638	0	0.088	-0.577	1.808	-0.542	-0.202	0.13	-1.148	0.149	1.153	0.134	0.053	-0.295	-0.73	-0.577	-1.47	-1.382	0.322	0.449	-2.162	0	-1.389	-2.101	0.817	-2.066	-1.726	-1.148	-2.672	-1.118	0.099	-1.047	-1.379	-1.819	-2.254
104	-0.501	0.084	0.293	-0.103	0.347	-0.253	-0.006	0.405	-0.104	0.013	0.509	0.259	0.087	-0.265	-0.783	-0.218	0.159	0.105	-0.534	-0.262	0.336	-0.097	-0.141	0.456	0.222	0.43	0.405	-0.206	0.109	-0.187	-0.303	-0.334	-0.018	0.158	0.538	-0.491	0.022	-0.199	0.175	-0.064	-0.083	0.127	0.037	0.382	0.068	-0.17	-0.104	0.109	0.033	-0.545	-0.176	0.057	0.108	0.358	-0.312
105	-1.03	0	-0.35	-0.41	0.55	-0.31	0.36	0.31	-0.25	-0.54	-0.43	-0.47	0.06	-0.94	-0.07	-1.1	-0.14	-0.32	-0.12	-0.25	-0.02	-0.18	0.06	0.39	0.18	-0.1	0.31	-0.86	0.11	-0.05	0.04	0.37	-0.27	-0.24	-0.68	-0.74	0.41	0.07	0.84	0.33	-0.43	0.4	-0.22	0.25	0.51	0.09	-0.25	0.11	0.28	-0.21	-0.02	0.44	0.36	-0.08	-0.44
106	-0.42	0.19	0.99	-0.28	0	-0.2	0.04	0.08	-0.34	0.46	0.31	0.14	0.19	-0.14	-0.67	-0.13	0.28	0.13	-0.3	-0.32	0.18	-0.14	-0.24	0.53	-0.33	0.3	0.08	-0.53	-0.18	-0.25	-0.14	-0.14	-0.11	0.5	0.06	-0.2	0.1	0	0.04	-0.1	0.2	-0.11	-0.21	0.25	0.11	0.42	-0.34	-0.18	0.26	-0.42	-0.38	0.01	-0.07	0.09	-0.28
107	-0.0876	0.0944	-0.0151	-0.0974	0.0602	-0.0624	0.014	0.0373	-0.0395	0.0304	0.0583	0.0551	0.0894	-0.0634	-0.2448	0.0085	0.0908	0.0094	0.0758	-0.0072	0.0029	-0.0348	-0.0009	0.0425	0.0088	0.0127	0.0373	-0.0773	0.0125	0.0439	-0.0321	-0.0005	0.003	0.0147	-0.0179	-0.0725	0.0782	-0.0151	0.0438	0.0724	0.066	0.0982	-0.0737	0.0427	0.1598	0.0211	-0.0395	0.0125	0.013	-0.0837	-0.062	0.112	0.062	0.027	-0.174
108	-0.1785	-0.0573	-0.0345	-0.2137	0.0866	-0.1822	-0.1059	0.0124	-0.0069	-0.1038	-0.0163	0.0185	0.0018	-0.2127	-0.0775	-0.1621	0.1049	-0.0844	0.0192	0.1146	0.0018	0.1196	0.0386	0.1485	-0.0381	0.089	0.0124	-0.0917	0.156	-0.005	-0.015	0.1452	0.0155	0.118	-0.025	-0.1149	0.1462	0.0545	0.2386	0.2241	-0.0604	0.2131	0.0295	0.0882	0.1099	0.0745	-0.0069	0.156	0.1077	0.1127	-0.0614	0.1626	0.1908	0.0868	-0.091
109	-6.68	-3.75	-3.31	-6.33	-3.11	-6.01	-6.06	-3.5	-4.11	-3.17	-3.49	-3.55	-3.73	-5.52	-6.37	-4.92	-1.44	-2.59	-1.33	-1.43	-3.55	-1.56	-2.01	-2.99	-0.91	-2.99	-3.5	-1.59	-1.43	-1.36	-1.41	-1.31	-1.51	-2.36	-3.11	-2.47	-1.81	-2.92	-1.19	-1.4	-3.73	-1.72	-2.17	-3.47	-0.67	-3.06	-4.11	-1.43	-1.18	-1.73	-1.85	-1.35	-1.66	-2.96	-3.66
110	-0.43	0.19	0.98	-0.28	0	-0.2	0.04	0.08	-0.34	0.46	0.31	0.14	0.19	-0.15	-0.67	-0.13	0.28	0.13	-0.3	-0.33	0.18	-0.14	-0.24	0.54	-0.32	0.3	0.08	-0.53	-0.18	-0.25	-0.13	-0.14	-0.11	0.49	0.07	-0.2	0.1	-0.01	0.03	-0.11	0.19	-0.11	-0.21	0.25	0.11	0.42	-0.34	-0.18	0.26	-0.43	-0.38	0.01	-0.07	0.08	-0.29
111	-6.56	-3.39	-3.98	-6.02	-2.48	-6.41	-5.46	-2.95	-3.45	-3.3	-3.12	-3.03	-3.51	-5.32	-5.55	-4.91	-1.84	-2.59	-1.68	-1.51	-3.75	-1.74	-2.08	-3.24	-1.21	-3.74	-2.95	-1.68	-1.53	-1.71	-1.64	-1.58	-1.88	-2.83	-3.07	-2.76	-2.03	-3.07	-1.33	-1.26	-4.25	-1.87	-2.25	-3.76	-0.97	-4.2	-3.45	-1.53	-1.75	-1.73	-1.7	-1.57	-1.9	-3.32	-3.73
112	-0.43	0.17	-0.05	-0.23	0.41	-0.23	-0.17	0.34	0.01	0.07	0.36	0.32	0.15	-0.06	-0.37	-0.13	0.1	0.35	-0.47	-0.35	0.38	-0.18	-0.15	0.55	-0.32	0.44	0.34	-0.39	-0.07	-0.34	-0.16	-0.23	-0.22	0.43	0.11	0.02	0.08	0.04	0.05	0.07	0.05	-0.14	-0.15	0.2	0.09	0.15	0.01	-0.07	-0.12	-0.19	-0.05	-0.05	-0.07	0.11	-0.38
113	4546	3376	1273	5188	1456	8218	1125	1410	1916	1490	1463	2205	2595	5343	1393	2765	6465	1549	6075	4550	2940	7319	2053	3331	4260	5751	1410	2605	3077	3368	3325	5920	5790	4792	1381	3792	6368	1983	3483	2989	3930	6827	2097	4600	2665	7632	1916	3077	1824	2779	3036	4843	4806	6518	2253
114	-0.83	-0.17	-0.29	-0.66	0.29	-0.7	-0.7	0.04	-0.13	-0.06	0.03	0.05	-0.11	-0.51	-0.73	-0.56	0.15	-0.01	0.02	0.12	-0.01	-0.01	0	0.14	0.22	0.04	0.04	-0.06	0.18	0.06	0.1	0.09	0.04	0.12	-0.1	-0.11	0.15	-0.18	0.33	0.37	-0.19	0.02	0.01	-0.05	0.47	-0.12	-0.13	0.18	0.11	0.17	0.17	0.2	0.13	-0.05	-0.37
115	-0.25	0.1	-0.02	-0.13	0.24	-0.13	-0.2	0.19	0.01	0.04	0.2	0.19	0.09	-0.03	-0.21	-0.08	0.07	0.21	-0.27	-0.19	0.22	-0.09	-0.08	0.32	-0.18	0.26	0.19	-0.26	-0.03	-0.19	-0.08	-0.12	-0.11	0.25	0.07	0.02	0.06	0.03	0.03	0.05	0.03	-0.07	-0.08	0.12	0.06	0.09	0.01	-0.03	-0.11	-0.09	-0.02	-0.02	-0.03	0.07	-0.21
116	-5.03	-3.4	-3.8	-4.95	-2.58	-4.91	-4.8	-3.04	-3.43	-3.24	-3.15	-3.35	-3.62	-4.59	-4.87	-4.3	-2.59	-3.14	-2.21	-2.14	-3.17	-2.41	-2.6	-3.13	-1.95	-3.09	-3.04	-2.24	-2.28	-2.26	-2.19	-2.36	-2.43	-3.02	-3.23	-2.82	-2.81	-3.5	-2.16	-2.09	-3.54	-2.6	-2.69	-3.62	-1.95	-3.55	-3.43	-2.28	-2.53	-2.35	-2.29	-2.57	-2.68	-3.39	-3.6
117	-5.58	-3.02	-4.47	-5.37	-2.36	-6.24	-5.89	-3.05	-3.11	-4.46	-4.13	-3.33	-3.33	-5.16	-5.49	-5.01	-2.06	-1.76	-2.5	-2.43	-3.89	-1.63	-3.05	-3.42	-1.63	-3.94	-3.05	-1.99	-1.01	-3	-2.67	-2.13	-2.05	-2.76	-3.31	-3.14	-1.78	-2.97	-1.24	-1.7	-3.46	-1.01	-1.8	-3.09	-0.5	-3.75	-3.11	-1.01	-0.83	-2.67	-2.43	-1.56	-1.24	-2.43	-3.96
118	-0.14	0.31	-0.17	0	0.61	-0.3	-0.74	0.32	-0.14	0.27	0.3	0.25	0.19	-0.4	-0.2	-0.05	0.31	1.46	-0.51	-0.3	-0.22	-0.09	-0.52	0.16	-0.44	0.23	0.32	-0.4	0.19	-0.05	-0.02	-0.32	-0.3	0.22	0.2	0.04	0.19	-0.15	0.36	0.01	-0.19	0.13	0.33	0.11	0.3	0.01	-0.14	0.19	-0.03	-0.11	-0.17	-0.15	0.11	0.16	-0.85
119	0.58	0.34	0	0.33	0.09	0.44	0	-0.01	0.39	0.23	0.37	-0.01	0.1	0.38	0	0.47	-0.21	-0.3	-0.12	-0.01	-0.1	-0.18	-0.14	-0.05	-0.17	-0.29	-0.01	-0.18	0.02	0.08	0.24	-0.16	-0.23	-0.12	0.04	0.26	-0.56	-0.28	-0.22	-0.3	0.08	-0.27	-0.22	-0.43	-0.46	-0.06	0.39	0.02	-0.57	-0.14	0.03	-0.13	-0.13	-0.1	0.35
120	-0.89	0.08	-0.17	-0.6	0.22	-0.68	-0.56	0.32	-0.16	-0.01	0.17	0.32	0	-0.47	-0.94	-0.51	0.24	0.28	-0.12	-0.01	0.29	0.1	0.1	0.55	-0.14	0.36	0.32	-0.04	0.13	-0.05	0.02	0.14	0	0.39	-0.09	0.01	0.07	-0.18	0.25	0.26	-0.19	-0.01	-0.05	0.05	0.12	-0.08	-0.16	0.13	-0.07	-0.05	-0.02	0.17	0	-0.08	-0.73
121	-1.37	0.08	-0.52	0.03	0.77	-0.36	-1.08	1.92	-0.75	0.16	0.4	-0.2	-0.4	-0.93	-0.33	-0.19	0.33	-1.89	0.28	0.78	0.36	0.46	0.95	0.48	0	1.17	1.92	-0.68	0.08	0.87	0.82	-0.78	0.13	0.71	1.45	0.43	0.87	-0.19	0.2	-0.18	1.41	-0.81	-1.01	-0.33	0.42	0.08	-0.75	0.08	0.06	0.24	-0.59	0.6	0.47	0.32	-2.24
122	-1.04	-0.63	0.18	0.07	1.66	-1.39	-1.89	0.66	-0.22	0.41	0.67	-0.59	-0.62	-1.22	-0.3	0.02	1.01	0.18	-0.53	-0.19	0.5	0.12	-0.06	0.22	0.38	-0.19	0.66	-0.45	-0.25	0.57	0.14	-0.17	1.23	0.54	-0.04	0.22	0.08	-0.45	0.83	0.24	-0.09	0.16	0.93	0.43	0.45	-0.27	-0.22	-0.25	0.41	-0.07	-0.31	0.48	0.34	0.75	-2.51

Table S12 Amino acid descriptors (2D) used in this study.

	M	M	M	M	M	M	M	M	M	M	M	M	M	M	M	M	N	N	N	N	N	N	N	N	N	N	N	N	N	N	N	N	N	N	N	N	P	P	P	P	P	P	P	P	P	P	P	P	P	P	P	P	P	P	P
	F	G	H	I	K	L	M	N	P	Q	R	S	T	V	W	Y	A	C	D	E	F	G	H	I	K	L	M	N	P	Q	R	S	T	V	W	Y	A	C	D	E	F	G	H	I	K	L	M	N	P	Q	R	S	T	V	W
No.	MF	MG	MH	MI	MK	ML	MM	MN	MP	MQ	MR	MS	MT	MV	MW	MY	NA	NC	ND	NE	NF	NG	NH	NI	NK	NL	NM	NN	NP	NQ	NR	NS	NT	NV	NW	NY	PA	PC	PD	PE	PF	PG	PH	PI	PK	PL	PM	PN	PP	PQ	PR	PS	PT	PV	PW
123	-1.9	0	-0.9	-1.4	-0.1	-1.3	-1.5	-0.3	-0.5	-0.6	-0.5	-0.1	-0.6	-1	-2	-1.5	0.3	0	-0.6	-0.6	-0.6	0	-0.8	-0.1	-0.3	-0.1	-0.3	-0.7	-0.1	-0.7	-0.8	-0.1	-0.4	0	-0.8	-0.8	0.6	0	0.1	0.1	-0.7	0	-0.4	-0.1	0.6	-0.1	-0.5	-0.1	0.1	-0.3	-0.2	0.2	0	0	-1.3
124	-4.6	0	-3.5	-4.7	-2.9	-4.4	-4.8	-3.3	-3.4	-3.3	-3.1	-3	-3.8	-4.7	-4.8	-4.4	-2.8	-3.2	-3.3	-2.8	-3.2	0	-3.2	-3.2	-3	-3	-3.3	-3.6	-2.7	-3.2	-3.2	-2.8	-3.4	-3.5	-3.5	-3.5	-2.3	-2.8	-2.3	-1.8	-3.1	0	-2.6	-3	-1.8	-2.8	-3.4	-2.7	-2.2	-2.5	-2.4	-2.2	-2.8	-3.2	-3.7
125	-1.1	0.4	-0.9	-0.7	0.5	-0.6	-1.1	0.2	-0.2	0.2	0.1	0	0.2	-0.5	-1.3	-0.6	0.1	0.3	-0.6	-0.4	0.2	-0.1	-0.5	0.7	0.1	0.5	0.2	-0.4	0.4	-0.4	0	-0.3	-0.3	0.2	-0.1	-0.5	0.3	0	0.6	0.6	-0.2	0.4	-0.5	0.3	1.1	0.5	-0.2	0.4	0.1	-0.1	0.5	0.4	0.3	0.1	-0.7
126	-0.1	0	0.2	0	0.2	0	-0.2	0.1	0	0.1	0.3	0.3	0.1	0.1	-0.1	-0.1	0.1	-0.1	-0.6	-0.5	0.1	0	-0.3	0.4	-0.5	0.3	0.1	-0.7	-0.3	-0.6	-0.5	-0.4	-0.3	0.3	0	-0.2	0	-0.1	0	-0.1	-0.1	0	-0.1	0.2	0.1	0.1	0	-0.3	-0.3	-0.4	-0.3	-0.3	-0.2	0.1	-0.4
127	-1.3	0.5	-0.4	-1	0.6	-1	-1.1	0.3	-0.2	0.1	0.2	0.4	0	-0.8	-1.5	-1.1	0.9	0.6	0.4	0.5	0.1	0.7	0.2	0.5	0.7	0.4	0.3	0.1	0.5	0.2	0	0.7	0.3	0.5	0	-0.2	0.6	0.4	0.9	0.7	-0.1	0.8	0	0	0.9	0	-0.2	0.5	0.4	0.2	0.1	0.6	0.2	-0.1	-0.8
128	-1.1	0.6	-0.3	-0.8	0.3	-1	-1	0.1	0	-0.1	0	0.3	-0.1	-0.8	-1.2	-0.8	0.8	0.5	0	0.3	0	0.6	0	0.4	0.3	0.3	0.1	0.1	0.6	0	0	0.4	0.2	0.5	-0.2	-0.2	0.8	0.4	0.9	0.5	-0.2	1.1	0.3	0.1	0.8	0.1	0	0.6	0.9	0.4	0.1	0.6	0.5	0.2	-0.7
129	-1.2	0.2	-0.9	-0.9	-0.1	-1	-1.4	-0.3	-0.4	-0.5	-0.4	-0.1	-0.3	-0.8	-1.6	-1	0.7	-0.5	0	0.2	-0.2	0.4	-0.4	0.2	0.2	0.3	-0.3	-0.2	0.3	-0.2	-0.2	0.2	0.1	0.3	-0.6	-0.3	0.7	-0.4	0.6	0.4	-0.3	0.8	-0.2	0	0.5	0.1	-0.4	0.3	0.3	0	0	0.4	0.3	0.2	-0.9
130	-1.4	0.2	-0.7	-0.3	0.7	-0.3	-0.7	0.5	0.3	0.3	-0.3	0.6	0.5	-0.2	-1.1	-0.9	0.7	0.2	0.8	1.2	-0.4	0.3	-0.2	0.7	1.4	0.8	0.5	0.6	0.6	0.8	0.1	0.5	0.5	0.7	-0.4	-0.3	0.4	-0.1	1	1	-0.7	0.4	-0.6	0.4	1.3	0.4	0.3	0.6	0.4	0.6	-0.1	0.5	0.6	0.3	-0.8
131	-1.2	0.2	-0.6	-0.2	1	-0.3	-0.5	0.6	0.3	0.5	-0.4	0.4	0.3	-0.2	-1	-1	0.4	0.3	0.2	0.6	-0.4	0.2	-0.5	0.7	0.6	0.6	0.6	0.1	0.6	0.3	-0.3	0.3	0.4	0.7	-0.5	-0.6	0.5	0.1	0.8	0.7	-0.6	0.5	-0.5	0.4	1.1	0.4	0.3	0.6	0.5	0.5	-0.4	0.5	0.5	0.3	-0.8
132	-1.4	0.2	-0.9	-0.8	0.3	-0.9	-1	0.2	0.2	0	-0.6	0	-0.2	-0.8	-1.1	-1.1	0.1	-0.1	-0.6	-0.2	-0.6	0.1	-0.8	0.2	-0.2	0.2	0.2	-0.6	0.3	-0.3	-0.8	-0.4	-0.4	0.1	-0.8	-0.8	0.4	-0.1	0.5	0.4	-0.6	0.4	-0.6	0.3	0.6	0.4	0.2	0.3	0.8	0.3	-0.5	0.3	0.2	0.2	-0.9
133	5.8	4.7	5	5.7	5.8	5.4	5.9	5.2	5.4	4.9	5.8	5.5	5.5	5.5	6.1	6.3	5	5.7	5.3	5.3	5.8	5.1	5.4	5.3	5.3	5.7	5.2	5.4	5.3	5.3	5.5	5.2	5.3	5.4	5.7	5.7	5.2	5.9	5.2	5.5	5.5	5.6	5.5	5.7	5.9	5.6	5.4	5.3	4.9	5.7	5.7	5.4	5.4	5.8	5.4
134	6.2	5.8	5.9	5.9	6.5	6.2	5.9	6.3	5.8	5.6	6.3	5.2	5.9	5.9	6.5	6.1	5.2	5.7	5.2	5.6	6.1	5.6	5.5	5.7	5.7	5.9	6.3	5.6	5.5	5.9	6.1	5.1	5.5	5.6	6.5	5.9	5.3	5.7	5.2	5.3	5.8	4	5.5	5.9	5.9	5.8	5.8	5.5	5.3	5.4	6.1	5.2	5.5	5.7	5.8
135	6	5.3	5.7	6.1	5.8	6.3	5.5	5.7	6.1	5.2	6.1	5.5	5.7	5.9	6.6	6	4.9	5.2	5	5	6.1	5.3	5.5	5.3	5.9	5.8	5.7	6.1	5.1	5.9	6	5.2	5.2	5.4	6.2	6.1	4.9	5.2	5.4	5.6	5.6	4.4	5.4	5.9	5.4	5.9	6.1	5.1	5.2	5.8	5.3	5.1	5.3	5.5	5.9
136	0.001	0.0023	0.0318	-0.0093	-0.0047	-0.0025	0.0317	0.0184	-0.0087	-0.0029	-0.0047	0.002	-0.0022	-0.0053	0.9849	-0.0033	0.0033	0.0061	0.0079	-0.0032	0.0035	-0.0016	0.0081	0.0023	0.0062	-0.0006	0.0184	-0.002	0.0037	0.0045	-0.0067	0.0062	-0.0059	-0.001	-0.003	-0.007	-0.0051	-0.0017	0.0008	0.0054	-0.007	0.0091	-0.002	0.0044	0.0099	-0.005	-0.0087	0.0037	-0.0036	0.0005	-7E-05	-0.0031	0.0054	0.0014	0.0139
137	-0.2336	0.1461	0.0142	-0.0879	0.1536	-0.1286	-0.14	0.0288	0.1129	-0.0354	0.13	0.197	0.3009	0.0064	-0.1	-0.2292	0.2594	0.3248	-0.4183	-0.1801	0.2661	-0.3707	-0.0042	0.3828	-0.0704	0.3536	0.0288	-0.6146	-0.2018	-0.2614	-0.2705	-0.2883	-0.2853	0.4745	0.286	0.1264	0.0362	-0.0448	-0.2145	-0.1419	-0.1048	-0.1844	-0.2788	0.5603	0.0408	0.3522	0.1129	-0.2018	-0.0417	-0.1657	-0.07	-0.1079	0.0284	0.3976	-0.5591
138	-0.12	0.1519	0.0474	-0.1536	0.3278	-0.141	-0.1651	0.1345	0.0819	0.1375	0.2375	0.1082	0.1044	-0.0739	0.001	-0.0112	0.0296	0.0506	-0.3678	-0.2905	0.2704	-0.2072	-0.221	0.4908	-0.2891	0.4995	0.1345	-0.4372	-0.2224	-0.29	-0.2048	-0.2376	-0.1505	0.4019	0.0369	0.058	0.08	-0.0959	-0.108	-0.0851	0.1612	-0.0569	-0.0532	0.1967	-0.0142	0.2	0.0819	-0.2224	-0.2054	-0.188	-0.1175	-0.112	-0.1338	0.1512	-0.1359
139	-0.114	-0.0218	0.0377	-0.0063	0.1502	0.0141	-0.0096	0.1145	0.0936	0.0196	0.0707	-0.0241	-0.0095	-0.0151	-0.0664	-0.001	0.0926	0.0203	-0.1502	-0.0928	0.0974	-0.0279	-0.0712	0.1695	-0.1759	0.1282	0.1145	-0.1797	-0.0911	-0.1179	-0.1577	-0.0751	-0.0265	0.108	0.0099	-0.0166	0.0457	0.0197	-0.0557	-0.1063	0.1237	-0.047	-0.0585	0.1293	-0.1263	0.0747	0.0936	-0.0911	-0.1365	-0.0886	-0.1662	-0.0211	0.0245	0.1117	0.0105
140	0.0074	0.0003	0.0352	-0.0436	0.036	0.0155	-0.0856	-0.0015	0.0212	0.0128	0.0438	-0.0269	0.0057	0.0343	-0.0187	-0.0128	0.0385	0.0009	-0.0157	-0.0179	0.0071	-0.0053	-0.0262	0.0252	-0.0469	0.0228	-0.0015	-0.0301	-0.0751	-0.0251	-0.0501	-0.0099	0.0057	0.0499	-0.0182	-0.0226	0.0226	-0.022	-0.0888	-0.0533	0.0582	-0.0151	0.0233	0.0791	-0.0678	0.0466	0.0212	-0.0751	-0.0832	-0.0632	-0.0134	0.0047	0.0143	0.0616	-0.0197
141	-0.0098	0.0018	0.0026	-0.0092	0.0085	-0.0051	-0.0088	0.0051	0.005	0.0039	0.0067	0.0011	0.0024	-0.0033	-0.0048	-0.0039	0.0039	0.008	-0.0083	-0.0068	0.0084	-0.0034	-0.0036	0.0134	-0.0082	0.0111	0.0051	-0.01	-0.0066	-0.0069	-0.0074	-0.0046	-0.0025	0.0117	0.0026	0.0018	0.0026	0.003	-0.0059	-0.0059	0.0081	-0.0024	-0.0017	0.0119	-0.0058	0.0083	0.005	-0.0066	-0.0071	-0.0061	-0.0055	-0.0019	-0.0005	0.0097	-0.0026
142	-2.25	-1.5	-1	-2.44	0.2	-2.37	-1.85	-0.46	0.07	-0.23	-0.1	-0.57	-1.12	-2.05	-2.2	-1.69	-0.25	-0.95	0.09	0.52	-0.43	-0.62	0.1	-0.84	0.32	-0.63	-0.46	0.14	0.59	0.2	0.25	-0.25	-0.23	-0.71	-0.48	-0.28	-0.26	-1.12	0.44	0.87	-0.42	-0.45	0.31	-1.14	0.64	-0.69	0.07	0.59	1.19	0.94	0.45	0.32	0	-0.97	-0.75
143	-3.13	-2.48	-1.55	-3.1	-1.17	-3.1	-1.95	-1.32	-1.43	-1.66	-0.98	-1.72	-1.4	-2.96	-2.31	-2.26	-1.22	-1.72	-1.17	-0.67	-1.41	-1.69	-0.67	-1.41	-0.35	-1.45	-1.32	-0.48	-0.78	-0.74	-0.8	-1.16	-0.9	-1.33	-1.13	-1.47	-1.64	-1.92	-0.27	0.38	-1.75	-1.85	-0.81	-1.6	-0.41	-1.9	-1.43	-0.78	0.2	-0.58	-0.84	-0.48	-0.72	-1.53	-2.05
144	-0.6	0.54	0.19	-0.52	1.15	-0.68	-0.12	0.68	0.54	0.65	0.58	0.56	0.46	-0.31	-0.5	-0.26	0.59	-0.07	0.61	1.09	0.46	0.79	0.91	0.4	1.11	0.37	0.68	0.83	1.09	0.87	0.83	0.81	0.54	0.54	0.13	0.4	0.48	-0.23	1.26	1.25	-0.04	0.74	0.59	0.14	1.59	0.1	0.54	1.09	1.11	1.09	0.82	0.94	0.67	0.17	-0.33
145	-1.83	-0.9	-0.82	-1.85	0.98	-1.8	-1.77	0.99	-0.34	0.79	0.89	0.45	-0.35	-1.21	-1.75	-1.6	0.55	-1	0.76	2.18	-0.7	0.03	0.13	-0.2	2.23	-0.04	-0.63	1.82	-0.19	1.22	2.31	1.02	0.35	0.24	-0.03	-0.29	0.28	-1.62	1.7	2	-1.4	0.82	0.53	-1.06	1.15	-1.29	0.34	0.54	0.57	0.53	1.37	0.75	-0.05	-1	-1.07
146	-1.36	-0.62	-0.63	-0.89	0.98	-0.96	-0.57	0.32	0.38	0.41	0.47	0.08	0.5	-0.93	-1.03	-0.98	0.84	0.4	1.06	1.33	0.42	0.27	0.95	0.61	1.75	0.5	0.76	1.16	1.31	1.28	1.16	1.04	0.75	0.48	-0.19	0.52	0.61	-0.31	1.41	1.54	-0.3	0.41	0.56	-0.27	1.63	0.02	0.07	1.14	1.05	1.14	1.22	0.76	0.88	-0.04	-0.56
147	-0.97	-0.46	-0.16	-0.99	0.97	-1.06	-0.31	0.29	0.83	0.15	0.88	-0.08	0.01	-0.7	-1.08	-0.76	0.3	0.21	0.46	1.37	0.63	0.27	0.68	0.31	1.18	0.43	0.47	0.57	0.6	0.92	0.76	0.73	0.7	0.48	0.27	0.2	-0.11	-0.5	1.02	1.26	-0.28	-0.21	0.52	-0.47	1.23	-0.39	-0.01	0.57	0.63	0.64	0.6	0.67	0.65	-0.28	-0.32
148	45.482	29.161	41.068	41.535	43.969	41.535	41.342	36.583	37.108	39.677	46.169	33.057	36.15	38.442	50.769	46.284	27.496	30.396	30.194	33.287	40.723	24.403	36.31	36.777	39.211	36.777	36.583	31.825	32.35	34.919	41.411	28.298	31.392	33.683	46.01	41.525	28.021	30.921	30.718	33.812	41.248	24.928	36.835	37.302	39.735	37.302	37.108	32.35	32.875	35.443	41.935	28.823	31.917	34.208	46.535
149	1	1	1	1	1	1	1	1	1	1	0	1	1	1	0	0	1	1	1	1	1	1	0	1	1	1	1	1	1	1	1	1	1	1	0	1	1	1	1	1	1	1	1	1	1	1	1	1	1	1	1	1	1	1	0
150	0	0	0	0	0	0	0	0	0	0	0	0	0	0	0	0	0	0	0	0	0	0	0	0	0	0	0	0	0	0	0	0	0	0	0	0	0	0	0	0	0	0	0	0	0	0	0	0	0	0	0	0	0	0	0
151	0	0	0	0	0	0	0	0	0	0	1	0	0	0	1	1	0	0	0	0	0	0	2	0	0	0	0	0	0	0	0	0	0	0	1	0	0	0	0	0	0	0	0	0	0	0	0	0	0	0	0	0	0	0	1
152	2	2	2	2	2	2	2	2	1	2	3	2	2	2	3	3	2	2	2	2	2	2	4	2	2	2	2	2	2	2	2	2	2	2	3	2	1	1	2	2	1	1	2	1	2	1	1	2	1	2	2	2	2	1	2
153	1	1	3	1	1	1	1	2	1	2	1	2	2	1	1	2	2	2	2	2	2	2	4	2	2	2	2	3	2	3	2	3	3	2	2	3	1	1	1	1	1	1	3	1	1	1	1	2	1	2	1	2	2	1	1
154	2	2	2	2	2	2	2	2	2	2	2	2	2	2	2	2	2	2	4	4	2	2	2	2	2	2	2	2	2	2	2	2	2	2	2	2	2	2	4	4	2	2	2	2	2	2	2	2	2	2	2	2	2	2	2
155	6	0	5	0	0	0	0	0	0	0	0	0	0	0	9	6	0	0	0	0	6	0	5	0	0	0	0	0	0	0	0	0	0	0	9	6	0	0	0	0	6	0	5	0	0	0	0	0	0	0	0	0	0	0	9
156	1	1	1	1	2	1	1	1	1	1	4	1	1	1	1	1	1	1	1	1	1	1	1	1	2	1	1	1	1	1	4	1	1	1	1	1	1	1	1	1	1	1	1	1	2	1	1	1	1	1	4	1	1	1	1
157	40	27	37	39	42	39	37	34	34	37	44	31	34	36	44	41	27	28	29	32	37	24	34	36	39	36	34	31	31	34	41	28	31	33	41	38	27	28	29	32	37	24	34	36	39	36	34	31	31	34	41	28	31	33	41
158	1	1	3	1	1	1	1	2	0	2	1	2	2	1	2	2	2	2	2	2	2	2	4	2	2	2	2	3	1	3	2	3	3	2	3	3	1	1	1	1	1	1	3	1	1	1	1	2	0	2	1	2	2	1	2
159	2	2	6	2	2	2	2	4	1	4	2	4	4	2	3	4	4	4	4	4	4	4	8	4	4	4	4	6	3	6	4	6	6	4	5	6	2	2	2	2	2	2	6	2	2	2	2	4	1	4	2	4	4	2	3
160	20	13	19	17	18	17	17	17	16	18	20	15	16	16	23	21	14	15	17	18	20	13	19	17	18	17	17	17	16	18	20	15	16	16	23	21	13	14	16	17	19	12	18	16	17	16	16	16	15	17	19	14	15	15	22
161	13	6	9	10	10	10	10	7	9	8	9	7	8	9	15	13	4	4	4	5	10	3	6	7	7	7	7	4	6	5	6	4	5	6	12	10	6	6	6	7	12	5	8	9	9	9	9	6	8	7	8	6	7	8	14
162	66.381	48.673	66.882	63.215	68.875	63.215	64.337	62.203	57.941	66.065	75.758	55.58	59.386	59.705	74.467	69.918	47.87	54.094	53.597	57.903	62.402	43.721	61.541	59.433	64.673	59.433	62.203	56.667	53.693	60.835	70.692	50.484	54.612	55.694	69.998	65.464	44.324	50.548	50.637	54.568	57.633	40.613	58.165	54.87	60.46	54.87	57.941	53.693	49.606	57.513	67.134	47.303	51.051	51.432	65.439
163	1.6595	1.8027	1.8076	1.6209	1.6399	1.6209	1.7388	1.8295	1.7041	1.7855	1.7218	1.7929	1.7466	1.6585	1.6924	1.7053	1.773	1.9319	1.8482	1.8095	1.6865	1.8217	1.81	1.6509	1.6583	1.6509	1.8295	1.828	1.732	1.7893	1.7242	1.803	1.7617	1.6877	1.7073	1.7227	1.6416	1.8053	1.7461	1.7052	1.5577	1.6922	1.7107	1.5242	1.5502	1.5242	1.7041	1.732	1.6002	1.6916	1.6374	1.6894	1.6468	1.5586	1.5961
164	14	7	11	11	11	11	10	9	10	10	11	8	9	10	16	14	7	7	8	9	13	6	10	10	10	10	9	8	9	9	10	7	8	9	15	13	8	8	9	10	14	7	11	11	11	11	10	9	10	10	11	8	9	10	16
165	20	14	18	22	24	22	20	17	18	19	24	16	18	20	21	20	13	13	12	14	17	11	15	19	21	19	17	14	15	16	21	13	15	17	18	17	14	14	13	15	18	12	16	20	22	20	18	15	16	17	22	14	16	18	19
166	2	2	4	2	3	2	2	3	2	3	5	2	2	2	3	2	3	3	3	3	3	3	5	3	4	3	3	4	3	4	6	3	3	3	4	3	2	2	2	2	2	2	4	2	3	2	2	3	2	3	5	2	2	2	3
167	3	3	3	3	3	3	3	4	3	4	3	4	4	3	3	4	4	4	6	6	4	4	4	4	4	4	4	5	4	5	4	5	5	4	4	5	3	3	5	5	3	3	3	3	3	3	3	4	3	4	3	4	4	3	3
168	1	1	1	1	1	1	2	1	1	1	1	1	1	1	1	1	0	1	0	0	0	0	0	0	0	0	1	0	0	0	0	0	0	0	0	0	0	1	0	0	0	0	0	0	0	0	1	0	0	0	0	0	0	0	0
169	2.4083	3.5136	2.3868	4.2506	4.0561	4.152	4.0559	4.1551	2.4839	4.1412	4.06	3.9325	4.1324	4.1324	1.8624	2.4221	3.898	4.0497	4.2546	4.2293	2.4506	3.6439	2.4328	4.3541	4.1412	4.2515	4.1516	4.2546	2.5531	4.2293	4.1287	4.0497	4.2439	4.2439	1.8881	2.4609	2.2743	2.3369	2.407	2.367	1.8088	2.1449	1.808	2.4692	2.3128	2.4049	2.3424	2.407	1.9593	2.367	2.2692	2.3369	2.4292	2.4292	1.5485
170	-2.5997	-2.5811	-2.5959	-2.6867	-2.6657	-2.6795	-2.6182	-2.5951	-2.5984	-2.6134	-2.6394	-2.594	-2.6124	-2.6557	-2.5979	-2.5996	-2.5512	-2.5535	-2.5582	-2.5962	-2.5641	-2.5189	-2.5552	-2.6834	-2.662	-2.6764	-2.5997	-2.5538	-2.5533	-2.5914	-2.6305	-2.5511	-2.5901	-2.6487	-2.5597	-2.564	-2.5468	-2.5493	-2.5543	-2.5944	-2.5607	-2.5127	-2.5511	-2.6831	-2.6616	-2.6761	-2.598	-2.5497	-2.5483	-2.5894	-2.6298	-2.5468	-2.5881	-2.6481	-2.5559
171	-0.5477	-0.4743	-0.5477	-0.437	-0.4633	-0.4588	-0.4259	-0.5219	-0.4821	-0.5348	-0.5005	-0.5234	-0.4548	-0.4399	-0.5431	-0.5472	-0.54	-0.5477	-0.5491	-0.5491	-0.5477	-0.5564	-0.5477	-0.5004	-0.5323	-0.5267	-0.5258	-0.5477	-0.5477	-0.5477	-0.5399	-0.5476	-0.5476	-0.5039	-0.5466	-0.5889	-0.5362	-0.5477	-0.5491	-0.5491	-0.5477	-0.5687	-0.5477	-0.4932	-0.527	-0.5213	-0.5179	-0.5477	-0.5477	-0.5477	-0.5373	-0.5476	-0.5476	-0.4967	-0.5464
172	0.5433	0.3626	0.3944	0.3599	0.3605	0.3605	0.3455	0.3898	0.3455	0.3904	0.3755	0.3909	0.3895	0.3599	0.5488	0.4941	0.4168	0.4715	0.4438	0.4439	0.5487	0.4214	0.4721	0.4139	0.4164	0.4163	0.389	0.4549	0.4619	0.4643	0.4439	0.4717	0.4689	0.4142	0.552	0.5251	0.4437	0.5368	0.4834	0.4877	0.5843	0.4955	0.5489	0.4232	0.4393	0.437	0.3903	0.4572	0.5261	0.4649	0.4884	0.542	0.5005	0.4247	0.5898
173	2.6486	2.6396	2.6493	2.6747	2.6687	2.6668	2.6534	2.6514	2.7308	2.6559	2.6618	2.6487	2.6569	2.6633	2.6489	2.6486	2.6332	2.6354	2.6364	2.6429	2.6368	2.6255	2.6378	2.6699	2.6637	2.6609	2.6432	2.6404	2.7291	2.6465	2.6545	2.6368	2.6475	2.6558	2.6372	2.6368	2.785	2.7854	2.7855	2.786	2.7855	2.7836	2.7856	2.7876	2.7865	2.7867	2.786	2.7859	2.8036	2.7862	2.7864	2.7856	2.7866	2.7869	2.7855
174	-3.3093	-3.309	-3.3093	-3.3093	-3.3093	-3.3093	-3.3093	-3.3092	-3.3072	-3.3092	-3.3092	-3.3094	-3.3094	-3.3093	-3.3093	-3.3093	-3.3089	-3.309	-3.309	-3.309	-3.309	-3.3087	-3.309	-3.3091	-3.309	-3.309	-3.309	-3.309	-3.307	-3.309	-3.309	-3.3091	-3.3092	-3.3091	-3.309	-3.309	-3.3807	-3.3808	-3.3808	-3.3807	-3.3808	-3.3806	-3.3808	-3.3808	-3.3808	-3.3808	-3.3808	-3.3808	-3.3794	-3.3807	-3.3808	-3.3808	-3.3808	-3.3808	-3.3808
175	-0.4532	-0.4371	-0.4535	-0.3792	-0.3966	-0.3948	-0.3691	-0.4523	-0.4283	-0.4525	-0.4246	-0.4528	-0.451	-0.3813	-0.4474	-0.5097	-0.493	-0.5843	-0.5435	-0.5395	-0.5381	-0.5028	-0.5626	-0.4757	-0.4919	-0.4758	-0.4462	-0.5692	-0.5082	-0.5633	-0.5378	-0.5845	-0.5709	-0.483	-0.5389	-0.5692	-0.5168	-0.6472	-0.5922	-0.5821	-0.5381	-0.5339	-0.5627	-0.4749	-0.5082	-0.4749	-0.445	-0.573	-0.5202	-0.5641	-0.574	-0.6512	-0.5812	-0.4835	-0.5806
176	0.4812	0.3585	0.4143	0.358	0.358	0.358	0.3979	0.391	0.3979	0.393	0.378	0.398	0.398	0.358	0.5985	0.4535	0.3563	0.3937	0.375	0.375	0.4816	0.3568	0.4143	0.3562	0.3563	0.3563	0.3937	0.3908	0.3912	0.393	0.375	0.3937	0.3937	0.3562	0.5986	0.4523	0.3862	0.4772	0.4293	0.4319	0.6785	0.4216	0.4861	0.38	0.3849	0.3847	0.3979	0.391	0.4538	0.393	0.432	0.4734	0.4356	0.3802	0.6903
177	2.5377	2.5357	2.5373	2.6834	2.6645	2.6893	2.5557	2.5375	2.6869	2.5541	2.5944	2.5363	2.5379	2.6143	2.538	2.5378	2.389	2.3813	2.3912	2.5358	2.4333	2.352	2.4	2.683	2.6641	2.6891	2.5455	2.3965	2.6849	2.5405	2.5921	2.3616	2.4406	2.6128	2.4565	2.4477	2.6236	2.6234	2.6236	2.6254	2.6236	2.6228	2.6235	2.6849	2.6667	2.6904	2.6253	2.6237	2.6945	2.6255	2.6285	2.6232	2.6237	2.6333	2.6237
178	-2.3849	-2.3775	-2.3845	-2.4371	-2.438	-2.4306	-2.39	-2.3845	-2.3867	-2.3918	-2.4055	-2.3843	-2.3929	-2.413	-2.3846	-2.3849	-2.3493	-2.3479	-2.3504	-2.3645	-2.352	-2.3395	-2.3512	-2.4322	-2.4352	-2.4256	-2.3622	-2.3513	-2.3527	-2.3658	-2.3921	-2.3509	-2.3676	-2.4012	-2.3513	-2.352	-2.3328	-2.3312	-2.3342	-2.3522	-2.3361	-2.3221	-2.3351	-2.43	-2.434	-2.4236	-2.3492	-2.3353	-2.3356	-2.354	-2.3871	-2.3347	-2.3562	-2.3965	-2.3353
179	-0.3877	-0.0906	-0.2652	-0.0871	-0.0881	-0.088	3E-17	-0.2638	-3E-17	-0.2642	-0.1761	-0.2641	-0.2603	-0.0872	-0.3586	-0.3732	-0.2939	-0.353	-0.3235	-0.3235	-0.3883	-0.2993	-0.3542	-0.289	-0.2933	-0.2932	-0.2632	-0.3435	-0.3363	-0.3519	-0.3235	-0.3534	-0.3355	-0.2897	-0.3884	-0.4088	-0.3185	-0.3974	-0.3639	-0.364	-0.4258	-0.3548	-0.4255	-0.2934	-0.3133	-0.3099	-0.2626	-0.3462	-0.358	-0.3544	-0.364	-0.4093	-0.3402	-0.2954	-0.4185
180	0.6892	0.4686	0.5312	0.4634	0.4646	0.4645	0.4541	0.4855	0.4541	0.4861	0.4753	0.486	0.4822	0.4635	0.7031	0.6289	0.5344	0.6311	0.582	0.5822	0.7024	0.5448	0.6336	0.5237	0.5331	0.5325	0.4842	0.6119	0.6046	0.6277	0.5822	0.6306	0.5969	0.5251	0.7138	0.6941	0.5545	0.6776	0.6127	0.6137	0.7228	0.5995	0.6888	0.5269	0.5485	0.5445	0.4833	0.6178	0.634	0.6368	0.6138	0.6734	0.6024	0.5292	0.7468
181	2.8702	2.8549	2.8694	2.9151	2.8972	2.9045	2.8862	2.8713	2.981	2.8805	2.8904	2.8661	2.8778	2.8962	2.871	2.8702	2.8216	2.8372	2.8326	2.8529	2.8353	2.8005	2.8334	2.9074	2.8861	2.8953	2.8679	2.8375	2.9766	2.8571	2.8753	2.8256	2.8509	2.8819	2.8374	2.8354	2.9683	2.97	2.9695	2.971	2.9696	2.9653	2.9694	2.977	2.9729	2.9742	2.972	2.9698	3.0136	2.9713	2.9723	2.9688	2.9711	2.9741	2.9697
182	26.358	19.799	26.152	28.545	29.411	28.545	28.638	23.377	25.492	25.563	30.071	21.985	24.172	26.358	28.111	26.358	16.724	19.004	17.546	19.733	21.097	14.537	20.89	23.283	24.149	23.283	23.377	18.115	20.23	20.301	24.809	16.724	18.91	21.097	22.85	21.097	18.839	21.119	19.662	21.848	23.212	16.652	23.005	25.398	26.265	25.398	25.492	20.23	22.345	22.417	26.925	18.839	21.025	23.212	24.965
183	8	6	8	8	10	8	9	8	6	9	10	7	7	7	8	8	5	6	7	8	7	5	7	7	9	7	8	7	5	8	9	6	6	6	7	7	3	4	5	6	5	3	5	5	7	5	6	5	3	6	7	4	4	4	5
184	0.4	0.5	0.4211	0.5	0.5882	0.5	0.5625	0.5	0.375	0.5294	0.5263	0.5	0.4667	0.4667	0.3333	0.381	0.3846	0.4286	0.4375	0.4706	0.35	0.4167	0.3684	0.4375	0.5294	0.4375	0.5	0.4375	0.3125	0.4706	0.4737	0.4286	0.4	0.4	0.2917	0.3333	0.2308	0.2857	0.3125	0.3529	0.25	0.25	0.2632	0.3125	0.4118	0.3125	0.375	0.3125	0.1875	0.3529	0.3684	0.2857	0.2667	0.2667	0.2083
185	6	0	5	0	0	0	0	0	0	0	0	0	0	0	10	6	0	0	0	0	6	0	5	0	0	0	0	0	0	0	0	0	0	0	10	6	0	0	0	0	6	0	5	0	0	0	0	0	0	0	0	0	0	0	10
186	40	26	37	38	41	38	36	33	34	36	43	30	33	35	45	41	26	27	28	31	37	23	34	35	38	35	33	30	31	33	40	27	30	32	42	38	27	28	29	32	38	24	35	36	39	36	34	31	32	34	41	28	31	33	43
187	2	2	2	2	2	2	2	3	2	3	3	2	2	2	2	2	3	3	4	4	3	3	3	3	3	3	3	4	3	4	4	3	3	3	3	3	2	2	3	3	2	2	2	2	2	2	2	3	2	3	3	2	2	2	2
188	20	12	19	16	17	16	16	16	16	17	19	14	15	15	24	21	13	14	16	17	20	12	19	16	17	16	16	16	16	17	19	14	15	15	24	21	13	14	16	17	20	12	19	16	17	16	16	16	16	17	19	14	15	15	24
189	7	7	7	7	8	7	7	7	7	7	7	7	7	7	7	7	4	5	4	5	4	4	4	6	8	6	7	4	4	5	7	5	5	5	4	4	4	5	4	5	4	3	4	6	8	6	7	4	2	5	7	5	5	5	4
190	9	7	9	9	11	9	10	9	6	10	12	8	8	8	9	9	6	7	8	9	8	6	8	8	10	8	9	8	5	9	11	7	7	7	8	8	4	5	6	7	6	4	6	6	8	6	7	6	3	7	9	5	5	5	6
191	0.45	0.5833	0.4737	0.5625	0.6471	0.5625	0.625	0.5625	0.375	0.5882	0.6316	0.5714	0.5333	0.5333	0.375	0.4286	0.4615	0.5	0.5	0.5294	0.4	0.5	0.4211	0.5	0.5882	0.5	0.5625	0.5	0.3125	0.5294	0.5789	0.5	0.4667	0.4667	0.3333	0.381	0.3077	0.3571	0.375	0.4118	0.3	0.3333	0.3158	0.375	0.4706	0.375	0.4375	0.375	0.1875	0.4118	0.4737	0.3571	0.3333	0.3333	0.25
192	32	24	30	36	39	36	34	30	32	33	40	28	31	33	33	33	23	24	24	27	28	20	26	32	35	32	30	26	28	29	36	24	27	29	29	29	25	26	26	29	30	22	28	34	37	34	32	28	30	31	38	26	29	31	31
193	14.958	10.268	14.251	13.422	13.966	13.422	13.259	13.422	12.129	14.129	15.544	11.845	12.715	12.715	16.82	15.828	11.301	12.008	13.585	14.293	15.121	10.431	14.414	13.585	14.129	13.585	13.422	13.585	12.293	14.293	15.707	12.008	12.878	12.878	16.983	15.991	9.8449	10.552	12.129	12.836	13.665	8.9747	12.958	12.129	12.673	12.129	11.966	12.129	10.836	12.836	14.251	10.552	11.422	11.422	15.527
194	12.319	8.3553	11.535	11.51	11.761	11.51	11.864	10.418	10.294	11.125	12.502	9.3798	10.25	10.803	13.819	12.689	7.7793	8.828	8.8029	9.51	10.873	6.909	10.088	10.064	10.315	10.064	10.418	8.972	8.8478	9.6791	11.055	7.9336	8.8038	9.3566	12.373	11.243	7.5781	8.6269	8.6017	9.3088	10.672	6.7079	9.8871	9.8626	10.114	9.8626	10.217	8.7708	8.6467	9.4779	10.854	7.7324	8.6027	9.1555	12.172
195	8.6628	4.6987	6.9307	7.8534	7.3973	7.8534	6.9831	5.776	6.6902	6.4831	7.1902	5.276	6.1463	7.1463	9.6628	8.5854	4.3618	4.0689	4.5689	5.276	7.4557	3.4916	5.7236	6.6463	6.1902	6.6463	5.776	4.5689	5.4831	5.276	5.9831	4.0689	4.9392	5.9392	8.4557	7.3783	5.276	4.9831	5.4831	6.1902	8.3699	4.4058	6.6378	7.5605	7.1044	7.5605	6.6902	5.4831	6.3973	6.1902	6.8973	4.9831	5.8534	6.8534	9.3699
196	9.5436	4.8534	7.4223	8.0081	7.552	8.0081	7.1378	6.0081	6.8449	6.7152	7.4223	5.4307	6.301	7.301	10.698	9.4138	4.5939	4.301	4.8783	5.5854	8.4138	3.7236	6.2925	6.8783	6.4223	6.8783	6.0081	4.8783	5.7152	5.5854	6.2925	4.301	5.1712	6.1712	9.5685	8.2841	5.4307	5.1378	5.7152	6.4223	9.2507	4.5605	7.1294	7.7152	7.2591	7.7152	6.8449	5.7152	6.552	6.4223	7.1294	5.1378	6.0081	7.0081	10.405
197	9.5409	6.0746	9.0409	7.934	8.5233	7.8791	8.0233	7.8791	7.5746	8.3791	9.3791	7.0233	7.396	7.396	11.024	9.9348	6.3411	6.8791	7.735	8.235	9.3968	5.9304	8.8968	7.7898	8.3791	7.735	7.8791	7.735	7.4304	8.235	9.235	6.8791	7.2518	7.2518	10.88	9.7906	6.1091	6.6471	7.5029	8.0029	9.1647	5.6984	8.6647	7.5577	8.1471	7.5029	7.6471	7.5029	7.1984	8.0029	9.0029	6.6471	7.0197	7.0197	10.648
198	7.8081	5.2654	7.2453	7.1617	7.5439	7.1069	8.1347	6.3903	6.8033	6.8903	7.7248	5.8601	6.3045	6.6237	8.8022	7.9424	3.9686	4.7482	4.5613	5.0613	6.0637	3.521	5.5009	5.4173	5.7995	5.3624	6.3903	4.6458	5.0589	5.1458	5.9804	4.1157	4.5601	4.8793	7.0577	6.198	4.4556	5.2352	5.0483	5.5483	6.5507	4.008	5.9879	5.9043	6.2865	5.8494	6.8772	5.1328	5.5458	5.6328	6.4674	4.6027	5.0471	5.3663	7.5447
199	4.1581	1.5505	2.5361	3.5116	3.3938	3.4568	2.3938	2.2474	2.8938	2.7474	2.8938	1.8938	2.3963	2.9736	4.6134	4.0688	1.9165	1.7474	2.101	2.601	4.0116	1.404	2.3896	3.3652	3.2474	3.3103	2.2474	2.101	2.7474	2.601	2.7474	1.7474	2.2498	2.8272	4.467	3.9223	2.5629	2.3938	2.7474	3.2474	4.6581	2.0505	3.0361	4.0116	3.8938	3.9568	2.8938	2.7474	3.3938	3.2474	3.3938	2.3938	2.8963	3.4736	5.1134
200	5.2079	1.6498	2.7997	3.601	3.4832	3.5461	2.4832	2.3914	2.9832	2.8914	2.9832	1.9832	2.4856	3.0629	5.7828	5.0244	2.0605	1.8914	2.2997	2.7997	5.1162	1.5581	2.7079	3.5092	3.3914	3.4544	2.3914	2.2997	2.8914	2.7997	2.8914	1.8914	2.3938	2.9712	5.6911	4.9327	2.6523	2.4832	2.8914	3.3914	5.7079	2.1498	3.2997	4.101	3.9832	4.0461	2.9832	2.8914	3.4832	3.3914	3.4832	2.4832	2.9856	3.5629	6.2828
201	2	1	2	3	2	2	2	2	2	2	2	2	3	2	2	2	2	2	2	2	2	1	2	3	2	2	2	2	2	2	2	2	3	2	2	2	2	2	2	2	2	1	2	3	2	2	2	2	2	2	2	2	3	2	2
202	2	1	2	3	2	2	2	2	2	2	2	2	3	2	2	2	2	2	2	2	2	1	2	3	2	2	2	2	2	2	2	2	3	2	2	2	2	2	2	2	2	1	2	3	2	2	2	2	2	2	2	2	3	2	2
203	0.7639	0.8297	0.8129	0.7576	0.763	0.7576	0.8145	0.8404	0.7956	0.8212	0.7877	0.8392	0.8181	0.7715	0.774	0.7878	0.8394	0.8932	0.9057	0.8784	0.7822	0.8691	0.838	0.7778	0.7825	0.7778	0.8404	0.872	0.8226	0.8483	0.808	0.8747	0.848	0.7948	0.7911	0.8078	0.7812	0.8405	0.8549	0.8315	0.7423	0.8048	0.7943	0.7325	0.7398	0.7325	0.7956	0.8226	0.7719	0.8026	0.7685	0.819	0.7969	0.746	0.7558
204	12	9	11	10	12	10	11	10	9	11	13	9	9	9	13	13	8	8	9	10	11	8	10	9	11	9	10	9	8	10	12	8	8	8	12	12	7	7	8	9	10	7	9	8	10	8	9	8	7	9	11	7	7	7	11
205	0	0	0	0	1	0	0	0	0	0	1	0	0	0	0	0	0	0	-1	-1	0	0	0	0	1	0	0	0	0	0	1	0	0	0	0	0	0	0	-1	-1	0	0	0	0	1	0	0	0	0	0	1	0	0	0	0

Table S12 Amino acid descriptors (2D) used in this study.

	M	M	M	M	M	M	M	M	M	M	M	M	M	M	M	M	N	N	N	N	N	N	N	N	N	N	N	N	N	N	N	N	N	N	N	N	P	P	P	P	P	P	P	P	P	P	P	P	P	P	P	P	P	P	P
	F	G	H	I	K	L	M	N	P	Q	R	S	T	V	W	Y	A	C	D	E	F	G	H	I	K	L	M	N	P	Q	R	S	T	V	W	Y	A	C	D	E	F	G	H	I	K	L	M	N	P	Q	R	S	T	V	W
No.	MF	MG	MH	MI	MK	ML	MM	MN	MP	MQ	MR	MS	MT	MV	MW	MY	NA	NC	ND	NE	NF	NG	NH	NI	NK	NL	NM	NN	NP	NQ	NR	NS	NT	NV	NW	NY	PA	PC	PD	PE	PF	PG	PH	PI	PK	PL	PM	PN	PP	PQ	PR	PS	PT	PV	PW
206	-0.8472	-0.8483	-0.8488	-0.8473	-0.863	-0.8475	-0.8473	-0.8471	-0.8515	-0.8475	-0.8474	-0.8464	-0.8463	-0.8472	-0.863	-0.8472	-0.847	-0.8463	-0.8505	-0.8543	-0.8467	-0.8478	-0.8487	-0.8467	-0.863	-0.847	-0.8468	-0.8465	-0.8511	-0.8469	-0.8469	-0.8458	-0.8457	-0.8467	-0.863	-0.8467	-0.8636	-0.8635	-0.8635	-0.8635	-0.8635	-0.8636	-0.8635	-0.8635	-0.8636	-0.8635	-0.8635	-0.8634	-0.8645	-0.8635	-0.8635	-0.8634	-0.8633	-0.8635	-0.8635
207	-0.3878	-0.3764	-0.3832	-0.3326	-0.344	-0.3408	-0.3321	-0.3719	-0.3325	-0.3711	-0.3557	-0.3506	-0.332	-0.3345	-0.3714	-0.3829	-0.4302	-0.479	-0.5219	-0.5155	-0.39	-0.4799	-0.4014	-0.3839	-0.3831	-0.3866	-0.3787	-0.5041	-0.4963	-0.488	-0.4053	-0.4929	-0.4632	-0.3921	-0.4399	-0.4654	-0.4085	-0.4421	-0.499	-0.4924	-0.39	-0.45	-0.4013	-0.3703	-0.383	-0.386	-0.3786	-0.4421	-0.442	-0.4421	-0.4053	-0.4421	-0.4419	-0.3738	-0.4227
208	0.0793	0.1424	0.1798	0.0849	0.1849	0.0702	0.0666	0.0864	0.1126	0.0719	0.1555	0.1855	0.163	0.0913	0.1455	0.1512	0.1194	0.1855	0.1147	0.0952	0.0862	0.1442	0.1804	0.0988	0.1849	0.0813	0.0861	0.0904	0.1313	0.0862	0.1555	0.1855	0.1643	0.1053	0.1523	0.1524	0.103	0.1737	0.1075	0.0885	0.0793	0.1393	0.1735	0.0847	0.1804	0.0701	0.0664	0.0863	0.1093	0.0718	0.1478	0.1748	0.1609	0.0909	0.1384
209	2.5442	2.344	2.508	2.5861	2.5933	2.5682	2.4999	2.4667	2.5973	2.5122	2.5853	2.423	2.4822	2.5302	2.6148	2.553	2.356	2.3725	2.3873	2.4426	2.5085	2.2867	2.4687	2.5532	2.5626	2.5345	2.4594	2.4229	2.5627	2.4731	2.5536	2.3743	2.4398	2.4925	2.5863	2.5183	2.4979	2.5097	2.5204	2.5599	2.6069	2.448	2.578	2.643	2.6465	2.6271	2.572	2.5455	2.6578	2.5816	2.6401	2.5109	2.5581	2.597	2.6639
210	-2.2351	-2.2351	-2.2351	-2.2351	-2.2354	-2.2351	-2.2351	-2.2351	-2.2351	-2.2351	-2.2351	-2.2351	-2.2351	-2.2351	-2.2351	-2.2351	-2.2351	-2.2351	-2.2351	-2.2351	-2.2351	-2.2351	-2.2351	-2.2351	-2.2354	-2.2351	-2.2351	-2.2351	-2.2351	-2.2351	-2.2351	-2.2351	-2.2351	-2.2351	-2.2351	-2.2351	-2.2465	-2.2465	-2.2465	-2.2465	-2.2465	-2.2465	-2.2465	-2.2465	-2.2465	-2.2465	-2.2465	-2.2465	-2.2465	-2.2465	-2.2465	-2.2465	-2.2465	-2.2465	-2.2465
211	-0.3607	-0.406	-0.3608	-0.3114	-0.326	-0.3139	-0.3492	-0.3676	-0.3625	-0.3626	-0.3501	-0.4175	-0.375	-0.3234	-0.3524	-0.3635	-0.4457	-0.4512	-0.5496	-0.5139	-0.4031	-0.5196	-0.4194	-0.3904	-0.3797	-0.3855	-0.3733	-0.5155	-0.4095	-0.4512	-0.4203	-0.5567	-0.4898	-0.4072	-0.3932	-0.4073	-0.4195	-0.4506	-0.5037	-0.4637	-0.4009	-0.4773	-0.4165	-0.3583	-0.3485	-0.3535	-0.3529	-0.4662	-0.4045	-0.4475	-0.4045	-0.4714	-0.4657	-0.3701	-0.3804
212	0.1383	0.0683	0.0582	0.0546	0.0555	0.0555	0.058	0.0542	0.0542	0.0542	0.0567	0.0542	0.0542	0.0546	0.1361	0.129	0.0542	0.0542	0.0542	0.0542	0.0581	0.0542	0.0542	0.0542	0.0542	0.0542	0.0542	0.0542	0.0542	0.0542	0.0542	0.0542	0.0542	0.0542	0.1015	0.0568	0.0557	0.058	0.0561	0.0567	0.1383	0.0683	0.0582	0.0546	0.0555	0.0555	0.058	0.0542	0.0542	0.0542	0.0568	0.0542	0.0542	0.0546	0.1361
213	2.4826	2.2241	2.3977	2.5224	2.5083	2.5089	2.4116	2.33	2.5117	2.3999	2.4687	2.284	2.3492	2.4423	2.6018	2.5035	2.1439	2.1636	2.1716	2.2758	2.4189	2.0408	2.3047	2.4617	2.4478	2.4482	2.3238	2.204	2.4371	2.3057	2.3964	2.1334	2.2328	2.3618	2.5626	2.4459	2.3341	2.3455	2.3514	2.414	2.5071	2.2738	2.4311	2.5458	2.5315	2.5323	2.4437	2.3712	2.5411	2.4337	2.4958	2.3296	2.3884	2.4724	2.6169
214	-0.6408	-0.6408	-0.6408	-0.6409	-0.6419	-0.6408	-0.6408	-0.6408	-0.641	-0.6409	-0.6409	-0.6408	-0.6409	-0.6409	-0.6408	-0.6408	-0.6409	-0.6409	-0.6409	-0.6409	-0.6409	-0.6409	-0.6409	-0.6409	-0.6419	-0.6409	-0.6409	-0.6409	-0.641	-0.6409	-0.6409	-0.6409	-0.6409	-0.6409	-0.6409	-0.6409	-0.639	-0.639	-0.639	-0.639	-0.639	-0.639	-0.639	-0.639	-0.6418	-0.639	-0.639	-0.639	-0.6391	-0.639	-0.639	-0.639	-0.639	-0.639	-0.639
215	-0.2025	-0.177	-0.202	-0.1687	-0.1723	-0.1712	-0.16	-0.1966	-0.16	-0.1964	-0.1849	-0.1978	-0.1836	-0.1694	-0.1961	-0.2115	-0.212	-0.1997	-0.2214	-0.2207	-0.2161	-0.222	-0.2201	-0.1985	-0.2079	-0.2048	-0.1971	-0.2315	-0.2246	-0.2319	-0.2207	-0.2323	-0.2213	-0.2002	-0.2245	-0.2291	-0.2103	-0.1973	-0.2199	-0.2191	-0.2161	-0.2234	-0.2201	-0.1967	-0.2061	-0.2029	-0.1949	-0.2301	-0.2207	-0.2305	-0.2191	-0.231	-0.2185	-0.1985	-0.2234
216	0.165	0.1531	0.163	0.116	0.128	0.1265	0.1613	0.16	0.1553	0.1613	0.1449	0.1607	0.1406	0.1171	0.1692	0.1833	0.1299	0.1606	0.1437	0.1437	0.1634	0.1506	0.1613	0.1156	0.1273	0.1259	0.1596	0.1586	0.1529	0.1596	0.1437	0.1591	0.1395	0.1166	0.1684	0.1827	0.1259	0.1533	0.1382	0.1382	0.1592	0.1464	0.1541	0.1129	0.1235	0.1225	0.1525	0.1516	0.1426	0.1525	0.1382	0.152	0.1336	0.1139	0.167
217	2.7818	2.5504	2.732	2.804	2.7966	2.7853	2.7301	2.6809	2.8163	2.7281	2.7955	2.6315	2.6928	2.7465	2.8751	2.7906	2.5433	2.578	2.5895	2.6494	2.7376	2.4625	2.6801	2.7605	2.7537	2.7404	2.6776	2.6195	2.7701	2.6749	2.7525	2.5606	2.6332	2.6954	2.8435	2.7478	2.6737	2.6984	2.7073	2.7516	2.8172	2.6151	2.7737	2.839	2.8308	2.8213	2.7722	2.7294	2.8543	2.7707	2.8298	2.6865	2.7401	2.7876	2.9002
218	18.05	13	17.053	17	18	17	17	17	14.063	18	20	15	16	16	19.326	19.048	14	15	17	18	18.05	13	17.053	17	18	17	17	17	14.063	18	20	15	16	16	19.326	19.048	11.077	12.071	14.063	15.059	15.39	10.083	14.41	14.063	15.059	14.063	14.063	14.063	11.484	15.059	17.053	12.071	13.067	13.067	16.844
219	9.8496	7.4082	9.0313	9	10.88	9	9.9723	9	6.6667	9.8685	11.637	8.1869	8.144	8.144	9.4746	9.904	6.4775	7.3025	8.1633	8.9917	9.1065	6.4533	8.3232	8.1633	9.8685	8.1633	9	8.1633	6.0744	8.9917	10.688	7.3025	7.35	7.35	8.9091	9.2092	5.0242	5.7778	6.6667	7.438	7.6953	4.8889	6.9632	6.6667	8.1633	6.6667	7.35	6.6667	4.8884	7.438	9.0313	5.7778	5.915	5.915	7.7135
220	7.0255	7.1006	6.321	6.4793	8.7075	7.84	7.84	7.84	3.7692	8.7075	10.412	6.2222	6.37	6.37	5.5	7.2	6.1875	6.2222	7.84	8.7075	7.0255	7.1006	6.321	6.4793	8.7075	7.84	7.84	7.84	3.7692	8.7075	10.412	6.2222	6.37	6.37	5.5	7.2	3.3241	3.5918	4.8166	5.4444	4.795	3.5156	4.2667	4.0768	5.4444	4.8166	4.8166	4.8166	2.3971	5.4444	6.8166	3.5918	3.811	3.811	4.1103
221	15.264	12.164	14.41	16.17	17.171	16.17	17.729	14.791	13.332	15.79	17.305	14.167	15.169	15.169	15.732	16.245	10.442	12.792	12.627	13.613	12.96	9.4552	12.122	13.412	14.404	13.412	14.791	12.196	10.916	13.178	14.712	11.43	12.421	12.421	13.636	13.914	9.1264	11.352	11.317	12.287	11.805	8.1615	10.981	12.047	13.026	12.047	13.332	10.916	9.7294	11.88	13.411	10.096	11.07	11.07	12.574
222	8.2513	6.8904	7.5556	8.5325	10.349	8.5325	10.428	7.7577	6.2966	8.586	9.9877	7.6989	7.692	7.692	7.6355	8.3755	4.7112	6.1515	5.9405	6.6788	6.4142	4.5554	5.7939	6.337	7.7856	6.337	7.7577	5.7233	4.6289	6.451	7.7235	5.4465	5.6009	5.6009	6.1774	6.6122	4.0666	5.406	5.2809	5.9857	5.8087	3.8734	5.2142	5.6479	6.9931	5.6479	6.942	5.0802	4.0885	5.774	7.0003	4.7604	4.9471	4.9471	5.6714
223	5.845	6.5711	5.2469	6.1267	8.2646	7.4133	8.2161	6.7078	3.55	7.5299	8.8835	5.8307	5.9994	5.9994	4.3989	6.0493	4.414	5.1889	5.6123	6.3818	4.8792	4.8858	4.3283	4.9653	6.7976	6.008	6.7078	5.3961	2.8324	6.1539	7.4272	4.5596	4.7868	4.7868	3.7634	5.1015	2.6502	3.3478	3.7704	4.3335	3.5742	2.7388	3.1537	3.425	4.628	4.0466	4.5364	3.6195	1.9839	4.1722	5.2241	2.9255	3.154	3.154	2.989
224	6.2976	6.447	5.7303	8.1159	9.8724	8.1159	10.875	6.7495	5.2466	7.5319	8.642	7.2715	7.2924	7.2924	5.2226	6.4791	3.5139	5.2461	4.4124	5.051	4.1564	3.3133	3.6965	4.9995	6.2303	4.9995	6.7495	4.1061	3.1582	4.7227	5.6812	4.1504	4.3479	4.3479	3.6624	4.3809	2.8549	4.3835	3.7352	4.3262	3.609	2.6344	3.1809	4.2524	5.3585	4.2524	5.7844	3.4661	2.6519	4.0352	4.941	3.433	3.6509	3.6509	3.2414
225	5	5	7	5	6	5	5	7	5	7	8	6	6	5	6	6	7	7	9	9	7	7	9	7	8	7	7	9	7	9	10	8	8	7	8	8	5	5	7	7	5	5	7	5	6	5	5	7	5	7	8	6	6	5	6
226	4	4	5	4	7	4	4	6	3	6	9	5	5	4	5	5	6	6	6	6	6	6	7	6	9	6	6	8	5	8	11	7	7	6	7	7	3	3	3	3	3	3	4	3	6	3	3	5	2	5	8	4	4	3	4
227	1	1	1	1	1	1	1	1	1	1	1	1	1	1	1	1	1	1	1	1	1	1	1	1	1	1	1	1	1	1	1	1	1	1	1	1	1	1	1	1	1	1	1	1	1	1	1	1	1	1	1	1	1	1	1
228	0	0	0	0	1	0	0	1	0	1	1	0	0	0	0	0	1	1	1	1	1	1	1	1	1	1	1	1	0	1	1	1	1	1	1	1	0	0	0	0	0	0	0	0	1	0	0	0	0	0	1	0	0	0	0
229	1.241	-0.756	-1.063	1.12	-0.225	1.12	0.361	-1.776	0.094	-1.334	-0.646	-1.329	-0.867	0.678	1.607	0.933	-2.431	-2.321	-3.058	-2.616	-0.896	-2.893	-3.2	-1.017	-2.362	-1.017	-1.776	-3.913	-2.043	-3.471	-2.783	-3.466	-3.004	-1.459	-0.53	-1.204	-0.233	-0.123	-0.86	-0.418	1.302	-0.695	-1.002	1.181	-0.164	1.181	0.422	-1.715	0.155	-1.273	-0.585	-1.268	-0.806	0.739	1.668
230	-2.7094	-0.88	-1.4665	-2.126	-1.1523	-2.4394	-2.0737	-1.0405	-1.3264	-1.2423	-1.5797	-0.6775	-1.0047	-1.6108	-2.9993	-2.3474	-0.1741	-0.8353	0.0149	-0.1868	-1.6762	0.1532	-0.4334	-1.0928	-0.1191	-1.4063	-1.0405	-0.0073	-0.2932	-0.2091	-0.5465	0.3557	0.0285	-0.5776	-1.9661	-1.3143	-0.6415	-1.3028	-0.4525	-0.6543	-2.1437	-0.3143	-0.9008	-1.5603	-0.5866	-1.8738	-1.508	-0.4748	-0.7607	-0.6766	-1.014	-0.1118	-0.439	-1.0451	-2.4336
231	8.1486	5.1509	7.384	7.0001	7.3573	6.99	7.2488	6.4403	6.3494	6.9168	7.8475	5.7624	6.203	6.5221	9.1986	8.2794	4.7536	5.5066	5.3929	5.8763	7.3114	4.2985	6.5255	6.1659	6.5165	6.1558	6.4403	5.5788	5.508	6.06	6.9933	4.9077	5.3534	5.6844	8.3538	7.4347	4.7007	5.4538	5.3492	5.8267	7.2394	4.2525	6.4753	6.0972	6.4533	6.0872	6.3763	5.5349	5.4467	6.0107	6.9403	4.8606	5.3003	5.6204	8.2851
232	6	0	5	0	0	0	0	0	5	0	1	0	0	0	10	6	0	0	0	0	6	0	5	0	0	0	0	0	5	0	1	0	0	0	10	6	5	5	5	5	11	5	10	5	5	5	5	5	10	5	6	5	5	5	15
233	1	1	1	1	1	1	1	0	1	1	0	1	1	1	1	1	0	0	0	0	1	0	0	1	0	1	0	0	1	0	0	0	0	0	1	1	1	1	1	1	1	1	1	1	0	1	1	1	1	1	0	1	1	1	1
234	1	0	1	0	0	0	0	0	1	0	0	0	0	0	2	1	0	0	0	0	1	0	1	0	0	0	0	0	1	0	0	0	0	0	2	1	1	1	1	1	2	1	2	1	1	1	1	1	2	1	1	1	1	1	3
235	8	6	8	8	10	8	9	8	6	9	11	7	7	7	8	8	5	6	7	8	7	5	7	7	9	7	8	7	5	8	10	6	6	6	7	7	4	5	6	7	6	4	6	6	8	6	7	6	4	7	9	5	5	5	6
236	0	0	0	0	1	0	0	2	0	1	3	0	0	0	0	0	2	2	3	3	1	2	3	1	2	1	2	3	1	3	3	2	2	2	1	1	0	0	1	1	0	0	0	0	2	0	0	1	0	1	2	1	1	0	0
237	5.307	4.413	5.28	4.413	6.266	4.413	4.873	5.783	4.343	5.783	8.191	5.093	5.093	4.413	5.647	5.689	5.323	5.733	6.229	6.229	6.217	5.323	6.19	5.323	7.176	5.323	5.783	6.693	5.253	6.693	9.101	6.003	6.003	5.323	6.557	6.599	4.006	4.416	4.912	4.912	4.9	4.006	4.873	4.006	5.859	4.006	4.466	5.376	3.936	5.376	7.784	4.686	4.686	4.006	5.24
238	-5.307	-4.413	-5.28	-4.413	-5.266	-4.413	-4.873	-5.783	-4.343	-5.783	-7.191	-5.093	-5.093	-4.413	-5.648	-5.689	-5.323	-5.733	-7.229	-7.229	-6.217	-5.323	-6.19	-5.323	-6.176	-5.323	-5.783	-6.693	-5.253	-6.693	-8.101	-6.003	-6.003	-5.323	-6.558	-6.599	-4.006	-4.416	-5.912	-5.912	-4.9	-4.006	-4.873	-4.006	-4.859	-4.006	-4.466	-5.376	-3.936	-5.376	-6.784	-4.686	-4.686	-4.006	-5.241
239	2.5873	2.2422	2.8299	2.4334	3.6756	2.4335	2.4437	2.8815	2.2832	2.901	4.1433	2.6291	2.6625	2.3806	2.83	2.8208	2.7384	2.8737	2.7978	2.8315	3.0471	2.702	3.2897	2.8932	4.1354	2.8933	2.9035	3.3413	2.743	3.3608	4.6031	3.0889	3.1223	2.8404	3.2897	3.2806	2.084	2.2193	2.1434	2.1771	2.3927	2.0476	2.6353	2.2388	3.481	2.2389	2.2491	2.6869	2.0886	2.7064	3.9487	2.4345	2.4679	2.186	2.6354
240	-2.5873	-2.2422	-2.8299	-2.4334	-2.6756	-2.4335	-2.4437	-2.8815	-2.2832	-2.901	-3.1433	-2.6291	-2.6625	-2.3806	-2.83	-2.8208	-2.7384	-2.8737	-3.7978	-3.8315	-3.0471	-2.702	-3.2897	-2.8932	-3.1354	-2.8933	-2.9035	-3.3413	-2.743	-3.3608	-3.6031	-3.0889	-3.1223	-2.8404	-3.2897	-3.2806	-2.084	-2.2193	-3.1434	-3.1771	-2.3927	-2.0476	-2.6353	-2.2388	-2.481	-2.2389	-2.2491	-2.6869	-2.0886	-2.7064	-2.9487	-2.4345	-2.4679	-2.186	-2.6354
241	0.1335	0.1541	0.1221	0.142	0.094	0.142	0.1414	0.1199	0.1513	0.1191	0.0834	0.1314	0.1298	0.1451	0.1221	0.1225	0.1262	0.1202	0.1235	0.122	0.1134	0.1279	0.105	0.1194	0.0836	0.1194	0.119	0.1034	0.126	0.1028	0.0751	0.1119	0.1107	0.1217	0.105	0.1053	0.1666	0.1565	0.162	0.1595	0.1451	0.1696	0.1318	0.1551	0.0998	0.1551	0.1544	0.1292	0.1663	0.1283	0.0879	0.1426	0.1407	0.1589	0.1318
242	0.2117	0.2444	0.1935	0.2251	0.2047	0.2251	0.2241	0.1901	0.2399	0.1888	0.1742	0.2083	0.2057	0.2301	0.1935	0.1942	0.2	0.1906	0.1448	0.1435	0.1797	0.2028	0.1665	0.1893	0.1747	0.1893	0.1886	0.1639	0.1997	0.163	0.152	0.1773	0.1754	0.1928	0.1665	0.1669	0.2628	0.2468	0.1749	0.1731	0.2289	0.2676	0.2078	0.2446	0.2208	0.2446	0.2435	0.2038	0.2622	0.2024	0.1857	0.2249	0.2219	0.2505	0.2078
243	47.09	38.559	72.537	76.947	64.151	76.947	68.417	47.09	77.344	64.322	55.621	38.559	51.355	68.417	72.744	47.09	21.327	27.739	40.471	40.301	17.062	8.5308	42.509	46.919	34.123	46.919	38.388	17.062	47.315	34.294	25.592	8.5308	21.327	38.388	42.716	17.062	47.26	53.672	66.405	66.234	42.995	34.464	68.442	72.853	60.056	72.853	64.322	42.995	73.249	60.227	51.526	34.464	47.26	64.322	68.649
244	43.447	36.432	54.322	22.12	43.843	22.12	22.12	30.821	22.12	22.12	43.843	45.602	31.29	22.12	43.447	47.64	30.821	30.821	30.821	30.821	52.148	45.133	63.023	30.821	52.544	30.821	30.821	39.522	30.821	30.821	52.544	54.303	39.991	30.821	52.148	56.341	43.843	43.843	43.843	43.843	65.17	58.155	76.045	43.843	65.566	43.843	43.843	52.544	43.843	43.843	65.566	67.325	53.013	43.843	65.17
245	4.2654	4.2654	8.5308	4.2654	4.2654	4.2654	4.2654	4.2654	4.2654	4.2654	4.2654	4.2654	4.2654	4.2654	4.2654	4.2654	4.2654	12.046	4.2654	4.2654	4.2654	4.2654	8.5308	4.2654	4.2654	4.2654	4.2654	4.2654	4.2654	4.2654	4.2654	4.2654	4.2654	4.2654	4.2654	4.2654	4.2654	12.046	4.2654	4.2654	4.2654	4.2654	8.5308	4.2654	4.2654	4.2654	4.2654	4.2654	4.2654	4.2654	4.2654	4.2654	4.2654	4.2654	4.2654
246	11.765	11.765	20.384	11.765	11.765	11.765	11.765	29.003	3.146	29.003	11.765	11.765	11.765	11.765	20.384	11.765	29.003	29.003	29.003	29.003	29.003	29.003	37.622	29.003	29.003	29.003	29.003	46.241	20.384	46.241	29.003	29.003	29.003	29.003	37.622	29.003	11.765	11.765	11.765	11.765	11.765	11.765	20.384	11.765	11.765	11.765	11.765	29.003	3.146	29.003	11.765	11.765	11.765	11.765	20.384
247	0	0	0	0	0	0	0	12.95	0	12.95	0	10.324	10.324	0	0	10.324	12.95	12.95	12.95	12.95	12.95	12.95	12.95	12.95	12.95	12.95	12.95	25.899	12.95	25.899	12.95	23.274	23.274	12.95	12.95	23.274	0	0	0	0	0	0	0	0	0	0	0	12.95	0	12.95	0	10.324	10.324	0	0
248	12.95	12.95	12.95	12.95	12.95	12.95	12.95	12.95	12.95	12.95	56.045	12.95	12.95	12.95	12.95	12.95	12.95	12.95	12.95	12.95	12.95	12.95	12.95	12.95	12.95	12.95	12.95	12.95	12.95	12.95	56.045	12.95	12.95	12.95	12.95	12.95	12.95	12.95	12.95	12.95	12.95	12.95	12.95	12.95	12.95	12.95	12.95	12.95	12.95	12.95	56.045	12.95	12.95	12.95	12.95
249	25.857	25.857	25.857	25.857	51.714	25.857	25.857	25.857	25.857	25.857	41.539	25.857	25.857	25.857	25.857	25.857	25.857	25.857	25.857	25.857	25.857	25.857	25.857	25.857	51.714	25.857	25.857	25.857	25.857	25.857	41.539	25.857	25.857	25.857	25.857	25.857	17.238	17.238	17.238	17.238	17.238	17.238	17.238	17.238	43.095	17.238	17.238	17.238	17.238	17.238	32.92	17.238	17.238	17.238	17.238
250	42.311	31.401	31.401	31.401	57.505	40.102	71.504	31.401	48.804	40.102	48.804	31.401	50.149	31.401	54.566	66.821	18.748	0	0	17.403	10.91	0	0	0	26.104	8.7013	40.102	0	17.403	8.7013	17.403	0	18.748	0	23.165	35.42	18.748	0	0	17.403	10.91	0	0	0	26.104	8.7013	40.102	0	17.403	8.7013	17.403	0	18.748	0	23.165
251	61.275	0	0	46.197	0	37.495	0	0	0	0	0	0	0	37.495	36.765	24.51	0	0	0	0	61.275	0	0	46.197	0	37.495	0	0	0	0	0	0	0	37.495	36.765	24.51	0	0	0	0	61.275	0	0	46.197	0	37.495	0	0	0	0	0	0	0	37.495	36.765
252	19.761	19.761	19.761	19.761	19.761	19.761	39.521	19.761	19.761	19.761	19.761	19.761	19.761	19.761	19.761	19.761	0	26.363	0	0	0	0	0	0	0	0	19.761	0	0	0	0	0	0	0	0	0	0	26.363	0	0	0	0	0	0	0	0	19.761	0	0	0	0	0	0	0	0
253	0	0	5.6826	0	0	0	0	0	0	0	0	0	0	0	0	0	0	0	0	0	0	0	5.6826	0	0	0	0	0	0	0	0	0	0	0	0	0	0	0	0	0	0	0	5.6826	0	0	0	0	0	0	0	0	0	0	0	0
254	13.567	13.567	13.567	13.567	13.567	13.567	13.567	27.134	13.567	27.134	27.006	13.567	13.567	13.567	13.567	13.567	27.134	27.134	27.134	27.134	27.134	27.134	27.134	27.134	27.134	27.134	27.134	40.701	27.134	40.701	40.573	27.134	27.134	27.134	27.134	27.134	13.567	13.567	13.567	13.567	13.567	13.567	13.567	13.567	13.567	13.567	13.567	27.134	13.567	27.134	27.006	13.567	13.567	13.567	13.567
255	28.376	28.376	28.513	28.376	29.482	28.376	28.376	35.027	28.239	35.027	28.376	36.144	36.144	28.376	28.513	36.144	35.027	35.027	62.161	62.161	35.027	35.027	35.164	35.027	36.133	35.027	35.027	41.678	34.89	41.678	35.027	42.795	42.795	35.027	35.164	42.795	27.271	27.271	54.405	54.405	27.271	27.271	27.408	27.271	28.376	27.271	27.271	33.922	27.134	33.922	27.271	35.038	35.038	27.271	27.408
256	0.7401	0.6378	0.705	0.7246	0.6514	0.7246	0.7293	0.5876	0.6852	0.6119	0.5461	0.6049	0.6304	0.7074	0.757	0.6918	0.4776	0.5251	0.4257	0.4634	0.6052	0.4328	0.5589	0.5798	0.509	0.5798	0.5876	0.4213	0.5137	0.4581	0.409	0.4213	0.4619	0.5513	0.633	0.5579	0.6393	0.6753	0.5626	0.5938	0.7344	0.6047	0.6929	0.7158	0.6313	0.7158	0.7216	0.5528	0.6669	0.5836	0.5124	0.5694	0.6025	0.6948	0.7542
257	0.5321	0.4176	0.337	0.4751	0.3894	0.4751	0.5127	0.4102	0.431	0.4158	0.3678	0.4032	0.4473	0.4732	0.4602	0.5014	0.371	0.369	0.3636	0.4059	0.4655	0.3095	0.2505	0.3997	0.3115	0.3997	0.4417	0.3241	0.3395	0.3356	0.2953	0.3066	0.3614	0.3925	0.3934	0.4349	0.3026	0.3073	0.3029	0.3533	0.4227	0.2273	0.1864	0.3482	0.2561	0.3482	0.3948	0.262	0.273	0.2788	0.244	0.2349	0.3005	0.3366	0.3485
258	0.135	0.1881	0.1627	0.1431	0.1393	0.1431	0.1406	0.225	0.1633	0.2118	0.1643	0.1987	0.1859	0.152	0.1264	0.155	0.285	0.2591	0.3636	0.3397	0.2154	0.3095	0.2505	0.2293	0.2205	0.2293	0.225	0.3241	0.2651	0.3035	0.24	0.3066	0.285	0.2448	0.2005	0.2342	0.2074	0.1867	0.3029	0.2813	0.1527	0.2273	0.1864	0.1634	0.1578	0.1634	0.1601	0.262	0.1913	0.244	0.1848	0.2349	0.2168	0.1755	0.1415
259	0.2599	0.3622	0.295	0.2754	0.3486	0.2754	0.2707	0.4124	0.3148	0.3881	0.4539	0.3951	0.3696	0.2926	0.243	0.3082	0.5224	0.4749	0.5743	0.5366	0.3948	0.5672	0.4411	0.4202	0.491	0.4202	0.4124	0.5787	0.4863	0.5419	0.591	0.5787	0.5381	0.4487	0.367	0.4421	0.3607	0.3247	0.4374	0.4062	0.2656	0.3953	0.3071	0.2842	0.3687	0.2842	0.2784	0.4472	0.3331	0.4164	0.4876	0.4306	0.3975	0.3052	0.2458
260	0.4679	0.5824	0.663	0.5249	0.6106	0.5249	0.4873	0.5898	0.569	0.5842	0.6322	0.5968	0.5527	0.5268	0.5398	0.4986	0.629	0.631	0.6364	0.5941	0.5345	0.6905	0.7495	0.6003	0.6885	0.6003	0.5583	0.6759	0.6605	0.6644	0.7047	0.6934	0.6386	0.6075	0.6066	0.5651	0.6974	0.6927	0.6971	0.6467	0.5773	0.7727	0.8136	0.6518	0.7439	0.6518	0.6052	0.738	0.727	0.7212	0.756	0.7651	0.6995	0.6634	0.6515
261	0.1249	0.1741	0.1322	0.1324	0.2093	0.1324	0.1301	0.1873	0.1516	0.1763	0.2895	0.1964	0.1837	0.1406	0.1166	0.1532	0.2373	0.2157	0.2107	0.1969	0.1793	0.2577	0.1907	0.1909	0.2705	0.1909	0.1873	0.2546	0.2212	0.2384	0.351	0.2721	0.253	0.2039	0.1666	0.2079	0.1533	0.138	0.1345	0.1249	0.1129	0.168	0.1206	0.1208	0.2109	0.1208	0.1183	0.1851	0.1419	0.1724	0.3028	0.1958	0.1807	0.1297	0.1043
262	229.91	142.18	206.94	212.46	201.29	212.46	217.59	162.34	175.44	179.57	184.06	151.35	168.59	195.22	251.93	221.85	104.16	125.97	104.56	121.79	174.66	86.932	151.68	157.21	146.04	157.21	162.34	107.09	120.19	124.32	128.81	96.102	113.33	139.97	196.68	166.6	125.88	147.69	126.28	143.51	196.38	108.65	173.4	178.92	167.76	178.92	184.06	128.81	141.91	146.04	150.52	117.82	135.05	161.69	218.4
263	165.29	93.105	98.924	139.3	120.31	139.3	152.97	113.32	110.37	122.02	123.95	100.87	119.62	130.6	153.17	160.8	80.909	88.524	89.295	106.7	134.35	62.161	67.981	108.36	89.37	108.36	122.02	82.379	79.427	91.08	93.003	69.929	88.676	99.656	122.23	129.86	59.585	67.201	67.971	85.374	113.02	40.838	46.657	87.034	68.047	87.034	100.7	61.056	58.103	69.757	71.679	48.605	67.353	78.333	100.9
264	41.943	41.943	47.763	41.943	43.049	41.943	41.943	62.161	41.806	62.161	55.382	49.711	49.711	41.943	42.08	49.711	62.161	62.161	89.295	89.295	62.161	62.161	67.981	62.161	63.267	62.161	62.161	82.379	62.024	82.379	75.6	69.929	69.929	62.161	62.298	69.929	40.838	40.838	67.971	67.971	40.838	40.838	46.657	40.838	41.943	40.838	40.838	61.056	40.701	61.056	54.277	48.605	48.605	40.838	40.975
265	80.75	80.75	86.569	80.75	107.71	80.75	80.75	113.92	80.613	113.92	152.97	98.841	98.841	80.75	80.887	98.841	113.92	113.92	141.05	141.05	113.92	113.92	119.74	113.92	140.88	113.92	113.92	147.08	113.78	147.08	186.13	132.01	132.01	113.92	114.05	132.01	71.025	71.025	98.159	98.159	71.025	71.025	76.845	71.025	97.988	71.025	71.025	104.19	70.888	104.19	143.24	89.117	89.117	71.025	71.162
266	145.37	129.83	194.58	153.9	188.69	153.9	145.37	162.94	145.68	171.47	213.08	149.32	147.81	145.37	179.65	159.89	137.17	151.37	156.32	156.15	154.23	138.69	203.44	162.76	197.55	162.76	154.23	171.8	154.54	180.33	221.94	158.18	156.67	154.23	188.51	168.75	137.32	151.51	156.47	156.3	154.38	138.84	203.59	162.91	197.7	162.91	154.38	171.94	154.69	180.48	222.09	158.33	156.82	154.38	188.66
267	38.807	38.807	38.807	38.807	64.664	38.807	38.807	51.756	38.807	51.756	97.583	49.131	49.131	38.807	38.807	49.131	51.756	51.756	51.756	51.756	51.756	51.756	51.756	51.756	77.613	51.756	51.756	64.706	51.756	64.706	110.53	62.08	62.08	51.756	51.756	62.08	30.188	30.188	30.188	30.188	30.188	30.188	30.188	30.188	56.045	30.188	30.188	43.137	30.188	43.137	88.964	40.512	40.512	30.188	30.188
268	0.5	0.4444	0.4545	0.5	0.5	0.5	0.4545	0.5	0.4444	0.4545	0.4615	0.4444	0.4444	0.4444	0.4615	0.4615	0.5	0.5	0.4444	0.5	0.4545	0.5	0.5	0.4444	0.4545	0.4444	0.5	0.4444	0.5	0.5	0.5	0.5	0.5	0.5	0.5	0.5	0.4286	0.4286	0.5	0.4444	0.5	0.4286	0.4444	0.5	0.5	0.5	0.4444	0.5	0.4286	0.4444	0.4545	0.4286	0.4286	0.4286	0.4545
269	1	0.8	0.8333	1	1	1	0.8333	1	0.8	0.8333	0.8571	0.8	0.8	0.8	0.8571	0.8571	1	1	0.8	1	0.8333	1	1	0.8	0.8333	0.8	1	0.8	1	1	1	1	1	1	1	1	0.75	0.75	1	0.8	1	0.75	0.8	1	1	1	0.8	1	0.75	0.8	0.8333	0.75	0.75	0.75	0.8333
270	5.307	4.413	5.28	4.413	6.266	4.413	4.873	5.783	4.343	5.783	8.191	5.093	5.093	4.413	5.647	5.689	5.323	5.733	6.229	6.229	6.217	5.323	6.19	5.323	7.176	5.323	5.783	6.693	5.253	6.693	9.101	6.003	6.003	5.323	6.557	6.599	4.006	4.416	4.912	4.912	4.9	4.006	4.873	4.006	5.859	4.006	4.466	5.376	3.936	5.376	7.784	4.686	4.686	4.006	5.24
271	-5.307	-4.413	-5.28	-4.413	-5.266	-4.413	-4.873	-5.783	-4.343	-5.783	-7.191	-5.093	-5.093	-4.413	-5.648	-5.689	-5.323	-5.733	-7.229	-7.229	-6.217	-5.323	-6.19	-5.323	-6.176	-5.323	-5.783	-6.693	-5.253	-6.693	-8.101	-6.003	-6.003	-5.323	-6.558	-6.599	-4.006	-4.416	-5.912	-5.912	-4.9	-4.006	-4.873	-4.006	-4.859	-4.006	-4.466	-5.376	-3.936	-5.376	-6.784	-4.686	-4.686	-4.006	-5.241
272	0.1707	0.2053	0.1716	0.2053	0.1446	0.2053	0.1859	0.1567	0.2086	0.1567	0.1465	0.1779	0.1779	0.2053	0.1604	0.1593	0.1702	0.158	0.1454	0.1454	0.1457	0.1702	0.1464	0.1702	0.1263	0.1702	0.1567	0.1354	0.1725	0.1354	0.1319	0.1509	0.1509	0.1702	0.1382	0.1373	0.2262	0.2052	0.1844	0.1844	0.1849	0.2262	0.1859	0.2262	0.1546	0.2262	0.2029	0.1685	0.2302	0.1685	0.1542	0.1933	0.1933	0.2262	0.1729
273	0.1696	0.2039	0.1705	0.2039	0.1709	0.2039	0.1847	0.1556	0.2072	0.1556	0.1345	0.1767	0.1767	0.2039	0.1593	0.1582	0.1691	0.157	0.1245	0.1245	0.1448	0.1691	0.1454	0.1691	0.1457	0.1691	0.1556	0.1345	0.1713	0.1345	0.1194	0.1499	0.1499	0.1691	0.1372	0.1364	0.2262	0.2052	0.1532	0.1532	0.1849	0.2262	0.1859	0.2262	0.1865	0.2262	0.2029	0.1685	0.2302	0.1685	0.1425	0.1933	0.1933	0.2262	0.1729
274	0.4902	0.2895	0.3546	0.4598	0.3575	0.4598	0.2976	0.2442	0.3641	0.2886	0.2766	0.2349	0.3218	0.4261	0.4479	0.4497	0.2772	0.1885	0.1879	0.2411	0.4538	0.2152	0.3049	0.4186	0.3107	0.4186	0.2442	0.1815	0.3073	0.2335	0.2283	0.1641	0.2638	0.3791	0.4111	0.4115	0.4378	0.3245	0.3204	0.3688	0.5861	0.3839	0.4336	0.5571	0.4324	0.5571	0.3655	0.3086	0.459	0.3562	0.3325	0.3054	0.4036	0.5244	0.5301
275	0.403	0.2768	0.2866	0.2104	0.2033	0.2104	0.2731	0.2965	0.2404	0.2791	0.223	0.2777	0.2598	0.2236	0.4098	0.3764	0.285	0.369	0.399	0.3728	0.4354	0.3095	0.3117	0.2293	0.2205	0.2293	0.2965	0.3241	0.2651	0.3035	0.24	0.3066	0.285	0.2448	0.4404	0.4057	0.2074	0.3073	0.3416	0.3173	0.3901	0.2273	0.2528	0.1634	0.1578	0.1634	0.2376	0.262	0.1913	0.244	0.1848	0.2349	0.2168	0.1755	0.3989
276	0.1986	0.2768	0.2866	0.2104	0.2033	0.2104	0.2731	0.2965	0.2404	0.2791	0.223	0.2777	0.2598	0.2236	0.2357	0.2166	0.285	0.369	0.3636	0.3397	0.2154	0.3095	0.3117	0.2293	0.2205	0.2293	0.2965	0.3241	0.2651	0.3035	0.24	0.3066	0.285	0.2448	0.2539	0.2342	0.2074	0.3073	0.3029	0.2813	0.1527	0.2273	0.2528	0.1634	0.1578	0.1634	0.2376	0.262	0.1913	0.244	0.1848	0.2349	0.2168	0.1755	0.1989
277	0.5098	0.7105	0.6454	0.5402	0.6425	0.5402	0.7024	0.7558	0.6359	0.7114	0.7234	0.7651	0.6782	0.5739	0.5521	0.5503	0.7228	0.8115	0.8121	0.7589	0.5462	0.7848	0.6951	0.5814	0.6893	0.5814	0.7558	0.8185	0.6927	0.7665	0.7717	0.8359	0.7362	0.6209	0.5889	0.5885	0.5622	0.6755	0.6796	0.6312	0.4139	0.6161	0.5664	0.4429	0.5676	0.4429	0.6345	0.6914	0.541	0.6438	0.6675	0.6946	0.5964	0.4756	0.4699
278	0.597	0.7232	0.7134	0.7896	0.7967	0.7896	0.7269	0.7035	0.7596	0.7209	0.777	0.7223	0.7402	0.7764	0.5902	0.6236	0.715	0.631	0.601	0.6272	0.5646	0.6905	0.6883	0.7707	0.7795	0.7707	0.7035	0.6759	0.7349	0.6965	0.76	0.6934	0.715	0.7552	0.5596	0.5943	0.7926	0.6927	0.6584	0.6827	0.6099	0.7727	0.7472	0.8366	0.8422	0.8366	0.7624	0.738	0.8087	0.756	0.8152	0.7651	0.7832	0.8245	0.6011
279	0.3112	0.4337	0.3588	0.3297	0.4393	0.3297	0.4293	0.4592	0.3954	0.4323	0.5004	0.4874	0.4185	0.3503	0.3164	0.3337	0.4378	0.4425	0.4486	0.4192	0.3308	0.4753	0.3835	0.3521	0.4688	0.3521	0.4592	0.4944	0.4276	0.463	0.5316	0.5293	0.4512	0.376	0.335	0.3543	0.3548	0.3682	0.3768	0.3499	0.2612	0.3888	0.3136	0.2795	0.4098	0.2795	0.3969	0.4294	0.3498	0.3998	0.4828	0.4597	0.3795	0.3002	0.271
280	152.28	64.548	104.07	134.82	110.46	134.82	88.795	67.468	93.231	84.7	93.231	58.767	86.045	117.59	149.07	144.22	60.453	45.221	46.141	63.373	130.95	43.221	82.745	113.49	89.136	113.49	67.468	46.141	71.904	63.373	71.904	37.44	64.718	96.262	127.74	122.89	86.216	70.984	71.904	89.136	156.71	68.983	108.51	139.26	114.9	139.26	93.231	71.904	97.667	89.136	97.667	63.202	90.481	122.02	153.5

Table S12 Amino acid descriptors (2D) used in this study.

	M	M	M	M	M	M	M	M	M	M	M	M	M	M	M	M	N	N	N	N	N	N	N	N	N	N	N	N	N	N	N	N	N	N	N	N	P	P	P	P	P	P	P	P	P	P	P	P	P	P	P	P	P	P	P
	F	G	H	I	K	L	M	N	P	Q	R	S	T	V	W	Y	A	C	D	E	F	G	H	I	K	L	M	N	P	Q	R	S	T	V	W	Y	A	C	D	E	F	G	H	I	K	L	M	N	P	Q	R	S	T	V	W
No.	MF	MG	MH	MI	MK	ML	MM	MN	MP	MQ	MR	MS	MT	MV	MW	MY	NA	NC	ND	NE	NF	NG	NH	NI	NK	NL	NM	NN	NP	NQ	NR	NS	NT	NV	NW	NY	PA	PC	PD	PE	PF	PG	PH	PI	PK	PL	PM	PN	PP	PQ	PR	PS	PT	PV	PW
281	125.19	61.704	84.132	61.704	62.809	61.704	81.464	81.922	61.567	81.922	75.143	69.471	69.471	61.704	136.38	120.7	62.161	88.524	97.996	97.996	125.64	62.161	84.59	62.161	63.267	62.161	81.922	82.379	62.024	82.379	75.6	69.929	69.929	62.161	136.83	121.16	40.838	67.201	76.673	76.673	104.32	40.838	63.266	40.838	41.943	40.838	60.598	61.056	40.701	61.056	54.277	48.605	48.605	40.838	115.51
282	61.704	61.704	84.132	61.704	62.809	61.704	81.464	81.922	61.567	81.922	75.143	69.471	69.471	61.704	78.45	69.471	62.161	88.524	89.295	89.295	62.161	62.161	84.59	62.161	63.267	62.161	81.922	82.379	62.024	82.379	75.6	69.929	69.929	62.161	78.907	69.929	40.838	67.201	67.971	67.971	40.838	40.838	63.266	40.838	41.943	40.838	60.598	61.056	40.701	61.056	54.277	48.605	48.605	40.838	57.584
283	158.38	158.38	189.43	158.38	198.54	158.38	209.55	208.79	162.82	208.79	243.79	191.43	181.38	158.38	183.75	176.48	157.63	194.67	199.47	199.47	157.63	157.63	188.68	157.63	197.78	157.63	208.79	208.03	162.07	208.03	243.04	190.67	180.63	157.63	182.99	175.72	110.69	147.73	152.53	152.53	110.69	110.69	141.74	110.69	150.85	110.69	161.85	161.1	115.13	161.1	196.1	143.73	133.69	110.69	136.06
284	185.48	161.23	209.37	231.5	246.19	231.5	216.88	194.34	194.49	211.57	261.88	180.72	197.96	214.27	196.44	199.99	155.92	151.37	147.62	164.85	162.94	138.69	186.83	208.96	223.65	208.96	194.34	171.8	171.94	189.03	239.34	158.18	175.41	191.73	173.9	177.45	156.07	151.51	147.76	165	163.08	138.84	186.98	209.11	223.8	209.11	194.49	171.94	172.09	189.18	239.49	158.33	175.56	191.88	174.05
285	96.681	96.681	105.3	96.681	135.73	96.681	128.08	126.87	101.25	126.87	168.65	121.96	111.91	96.681	105.3	107.01	95.468	106.14	110.18	110.18	95.468	95.468	104.09	95.468	134.52	95.468	126.87	125.66	100.04	125.66	167.44	120.74	110.7	95.468	104.09	105.79	69.853	80.531	84.562	84.562	69.853	69.853	78.472	69.853	108.9	69.853	101.25	100.04	74.427	100.04	141.82	95.129	85.082	69.853	78.472
286	6	5	6	5	6	5	6	5	5	6	7	5	5	5	7	7	4	4	5	5	6	4	5	5	6	5	5	5	4	5	6	4	4	4	6	6	4	4	4	5	5	4	5	4	5	4	5	4	4	5	6	4	4	4	6
287	0	0	0	0	0	0	0	0	0	0	0	0	0	0	0	0	0	1	0	0	0	0	0	0	0	0	0	0	0	0	0	0	0	0	0	0	0	1	0	0	0	0	0	0	0	0	0	0	0	0	0	0	0	0	0
288	1	0	1	0	0	0	0	0	1	0	0	0	0	0	2	1	0	0	0	0	1	0	1	0	0	0	0	0	1	0	0	0	0	0	2	1	1	1	1	1	2	1	2	1	1	1	1	1	2	1	1	1	1	1	3
289	0.1707	0.2053	0.1716	0.2053	0.1446	0.2053	0.1859	0.1567	0.2086	0.1567	0.1465	0.1779	0.1779	0.2053	0.1604	0.1593	0.1702	0.158	0.1454	0.1454	0.1457	0.1702	0.1464	0.1702	0.1263	0.1702	0.1567	0.1354	0.1725	0.1354	0.1319	0.1509	0.1509	0.1702	0.1382	0.1373	0.2262	0.2052	0.1844	0.1844	0.1849	0.2262	0.1859	0.2262	0.1546	0.2262	0.2029	0.1685	0.2302	0.1685	0.1542	0.1933	0.1933	0.2262	0.1729
290	0.1696	0.2039	0.1705	0.2039	0.1709	0.2039	0.1847	0.1556	0.2072	0.1556	0.1345	0.1767	0.1767	0.2039	0.1593	0.1582	0.1691	0.157	0.1245	0.1245	0.1448	0.1691	0.1454	0.1691	0.1457	0.1691	0.1556	0.1345	0.1713	0.1345	0.1194	0.1499	0.1499	0.1691	0.1372	0.1364	0.2262	0.2052	0.1532	0.1532	0.1849	0.2262	0.1859	0.2262	0.1865	0.2262	0.2029	0.1685	0.2302	0.1685	0.1425	0.1933	0.1933	0.2262	0.1729
291	-1.1726	-2.7839	-2.4495	-1.3692	-3.0031	-1.3692	-1.6622	-3.5399	-1.9091	-3.1498	-4.9717	-3.423	-3.0345	-1.7593	-0.6913	-1.467	-4.2731	-4.3632	-6.153	-5.7629	-3.0503	-4.6616	-4.3272	-3.2469	-4.8808	-3.2469	-3.5399	-5.4176	-3.7868	-5.0275	-6.8494	-5.3007	-4.9122	-3.637	-2.569	-3.3447	-3.0332	-3.1233	-4.9131	-4.523	-1.8104	-3.4217	-3.0873	-2.007	-3.6409	-2.007	-2.3	-4.1777	-2.5469	-3.7876	-5.6095	-4.0608	-3.6723	-2.3971	-1.3291
292	93.591	93.591	93.591	93.591	126.49	93.591	93.591	126.49	75.581	126.49	176.17	118.98	118.98	93.591	93.591	118.98	126.49	126.49	169.17	169.17	126.49	126.49	126.49	126.49	159.39	126.49	126.49	159.39	108.48	159.39	209.07	151.87	151.87	126.49	126.49	151.87	78.705	78.705	121.39	121.39	78.705	78.705	78.705	78.705	111.6	78.705	78.705	111.6	60.694	111.6	161.28	104.09	104.09	78.705	78.705
293	6.7228	6.7228	23.508	6.7228	6.7228	6.7228	6.7228	11.982	9.8471	11.982	8.6219	6.7228	6.7228	6.7228	6.7228	6.7228	11.982	11.982	13.446	13.446	11.982	11.982	28.767	11.982	11.982	11.982	11.982	17.24	15.106	17.24	13.881	11.982	11.982	11.982	11.982	11.982	6.7228	6.7228	8.1868	8.1868	6.7228	6.7228	23.508	6.7228	6.7228	6.7228	6.7228	11.982	9.8471	11.982	8.6219	6.7228	6.7228	6.7228	6.7228
294	55.688	51.706	72.474	55.688	55.688	55.688	55.688	79.551	55.688	79.551	55.688	55.688	62.157	55.688	72.474	55.688	79.551	79.551	103.41	103.41	79.551	75.569	96.336	79.551	79.551	79.551	79.551	103.41	79.551	103.41	79.551	79.551	86.019	79.551	96.336	79.551	55.688	55.688	79.551	79.551	55.688	51.706	72.474	55.688	55.688	55.688	55.688	79.551	55.688	79.551	55.688	55.688	62.157	55.688	72.474
295	17.234	35.673	17.234	17.234	35.673	17.234	34.467	17.234	35.673	17.234	35.673	38.16	17.234	17.234	17.234	17.234	0	17.234	0	0	0	18.44	0	0	18.44	0	17.234	0	18.44	0	18.44	20.926	0	0	0	0	18.44	35.673	18.44	18.44	18.44	36.879	18.44	18.44	36.879	18.44	35.673	18.44	36.879	18.44	36.879	39.366	18.44	18.44	18.44
296	3.1856	0	2.7567	4.4108	0	4.4108	0	0	0	0	0	0	0	4.4108	3.1856	3.1856	0	0	0	0	3.1856	0	2.7567	4.4108	0	4.4108	0	0	0	0	0	0	0	4.4108	3.1856	3.1856	0	0	0	0	3.1856	0	2.7567	4.4108	0	4.4108	0	0	0	0	0	0	0	4.4108	3.1856
297	50.56	31.691	50.56	31.691	31.691	31.691	63.382	31.691	31.691	31.691	31.691	31.691	31.691	31.691	50.56	50.56	0	0	0	0	18.868	0	18.868	0	0	0	31.691	0	0	0	0	0	0	0	18.868	18.868	0	0	0	0	18.868	0	18.868	0	0	0	31.691	0	0	0	0	0	0	0	18.868
298	88.216	0	34	0	0	0	0	0	0	0	0	0	0	0	92.504	70.573	0	0	0	0	88.216	0	34	0	0	0	0	0	0	0	0	0	0	0	92.504	70.573	0	0	0	0	88.216	0	34	0	0	0	0	0	0	0	0	0	0	0	92.504
299	18.868	18.868	18.868	37.737	75.474	37.737	37.737	37.737	56.605	56.605	56.605	18.868	18.868	18.868	18.868	18.868	18.868	64.976	37.737	56.605	18.868	18.868	18.868	37.737	75.474	37.737	37.737	37.737	56.605	56.605	56.605	18.868	18.868	18.868	18.868	18.868	37.737	83.844	56.605	75.474	37.737	37.737	37.737	56.605	94.342	56.605	56.605	56.605	75.474	75.474	75.474	37.737	37.737	37.737	37.737
300	32.22	32.22	32.22	98.872	32.22	98.872	64.439	32.22	32.22	32.22	32.22	32.22	65.546	98.872	32.22	37.463	33.326	0	0	0	0	0	0	66.652	0	66.652	32.22	0	0	0	0	0	33.326	66.652	0	5.2434	33.326	0	0	0	0	0	0	66.652	0	66.652	32.22	0	0	0	0	0	33.326	66.652	0
301	7.676	4.7693	6.8906	6.6069	6.822	6.6069	6.9614	6.0425	5.9418	6.5042	7.497	5.37	5.8295	6.1452	8.8617	7.8425	4.3099	5.1279	4.7049	5.1666	6.7571	3.8504	5.9717	5.688	5.9031	5.688	6.0425	5.1237	5.023	5.5854	6.5782	4.4511	4.9106	5.2263	7.9428	6.9236	4.2426	5.0606	4.6376	5.0993	6.6898	3.7831	5.9044	5.6207	5.8358	5.6207	5.9752	5.0564	4.9556	5.5181	6.5109	4.3838	4.8433	5.159	7.8755
302	90.408	90.408	90.408	90.408	90.408	90.408	90.408	114.27	90.408	114.27	90.408	90.408	90.408	90.408	90.408	90.408	114.27	114.27	180.82	180.82	114.27	114.27	114.27	114.27	114.27	114.27	114.27	138.13	114.27	138.13	114.27	114.27	114.27	114.27	114.27	114.27	90.408	90.408	156.95	156.95	90.408	90.408	90.408	90.408	90.408	90.408	90.408	114.27	90.408	114.27	90.408	90.408	90.408	90.408	90.408
303	0	0	16.786	0	0	0	0	0	3.1243	0	31.672	25.385	25.385	0	0	25.385	0	0	0	0	0	0	16.786	0	0	0	0	0	3.1243	0	31.672	25.385	25.385	0	0	25.385	18.011	18.011	18.011	18.011	18.011	18.011	34.796	18.011	18.011	18.011	18.011	18.011	21.135	18.011	49.683	43.396	43.396	18.011	18.011
304	50.908	50.908	67.693	55.319	83.805	55.319	50.908	50.908	32.897	50.908	68.919	50.908	50.908	55.319	67.693	50.908	50.908	50.908	50.908	50.908	50.908	50.908	67.693	55.319	83.805	55.319	50.908	50.908	32.897	50.908	68.919	50.908	50.908	55.319	67.693	50.908	18.011	18.011	18.011	18.011	18.011	18.011	34.796	22.422	50.908	22.422	18.011	18.011	0	18.011	36.022	18.011	18.011	22.422	34.796
305	11.15	3.982	10.721	7.9639	7.9639	7.9639	7.9639	7.9639	7.9639	7.9639	7.9639	7.9639	14.433	7.9639	11.15	16.393	7.9639	7.9639	7.9639	7.9639	11.15	3.982	10.721	7.9639	7.9639	7.9639	7.9639	7.9639	7.9639	7.9639	7.9639	7.9639	14.433	7.9639	11.15	16.393	7.9639	7.9639	7.9639	7.9639	11.15	3.982	10.721	7.9639	7.9639	7.9639	7.9639	7.9639	7.9639	7.9639	7.9639	7.9639	14.433	7.9639	11.15
306	0	0	0	0	0	0	0	32.897	0	32.897	32.897	0	0	0	4.7171	0	32.897	32.897	32.897	32.897	32.897	32.897	32.897	32.897	32.897	32.897	32.897	65.794	32.897	65.794	65.794	32.897	32.897	32.897	37.614	32.897	0	0	0	0	0	0	0	0	0	0	0	32.897	0	32.897	32.897	0	0	0	4.7171
307	125.95	18.868	71.737	37.737	75.474	37.737	37.737	37.737	56.605	56.605	56.605	18.868	18.868	18.868	125.52	108.31	18.868	18.868	37.737	56.605	125.95	18.868	71.737	37.737	75.474	37.737	37.737	37.737	56.605	56.605	56.605	18.868	18.868	18.868	125.52	108.31	37.737	37.737	56.605	75.474	144.82	37.737	90.605	56.605	94.342	56.605	56.605	56.605	75.474	75.474	75.474	37.737	37.737	37.737	144.39
308	23.956	42.396	23.956	23.956	42.396	23.956	41.19	29.215	42.396	29.215	44.295	44.883	23.956	23.956	23.956	23.956	11.982	29.215	13.446	13.446	11.982	30.421	11.982	11.982	30.421	11.982	29.215	17.24	30.421	17.24	32.32	32.908	11.982	11.982	11.982	11.982	25.162	42.396	26.626	26.626	25.162	43.602	25.162	25.162	43.602	25.162	42.396	30.421	43.602	30.421	45.501	46.089	25.162	25.162	25.162
309	63.911	63.911	63.911	130.56	63.911	130.56	127.82	63.911	63.911	63.911	63.911	63.911	97.237	130.56	63.911	63.911	33.326	46.107	0	0	0	0	0	66.652	0	66.652	63.911	0	0	0	0	0	33.326	66.652	0	0	33.326	46.107	0	0	0	0	0	66.652	0	66.652	63.911	0	0	0	0	0	33.326	66.652	0
310	96.87	96.87	125.55	96.87	124.51	96.87	96.87	139.96	88.08	139.96	160.51	117.1	117.1	96.87	112.66	117.1	139.96	178.76	180.09	180.09	139.96	139.96	168.64	139.96	167.6	139.96	139.96	183.05	131.17	183.05	203.6	160.19	160.19	139.96	155.75	160.19	85.84	124.64	125.97	125.97	85.84	85.84	114.52	85.84	113.48	85.84	85.84	128.93	77.05	128.93	149.48	106.07	106.07	85.84	101.63
311	0.469	0.5895	0.4855	0.5021	0.4845	0.5021	0.5021	0.5021	0.5436	0.4845	0.4529	0.542	0.5212	0.5212	0.4389	0.4537	0.5647	0.542	0.5021	0.4845	0.469	0.5895	0.4855	0.5021	0.4845	0.5021	0.5021	0.5021	0.5436	0.4845	0.4529	0.542	0.5212	0.5212	0.4389	0.4537	0.6194	0.5917	0.5436	0.5226	0.5023	0.65	0.5215	0.5436	0.5226	0.5436	0.5436	0.5436	0.59	0.5226	0.4855	0.5917	0.5665	0.5665	0.4664
312	5.3219	4.585	5.2479	5	5.0875	5	5	5	5	5.0875	5.2479	4.8074	4.9069	4.9069	5.585	5.3923	4.7004	4.8074	5	5.0875	5.3219	4.585	5.2479	5	5.0875	5	5	5	5	5.0875	5.2479	4.8074	4.9069	4.9069	5.585	5.3923	4.7004	4.8074	5	5.0875	5.3219	4.585	5.2479	5	5.0875	5	5	5	5	5.0875	5.2479	4.8074	4.9069	4.9069	5.585
313	3.2993	2.9612	3.2289	3.0756	3.2729	3.1122	3.1674	3.1122	2.94	3.22	3.4471	2.9963	3.0134	3.0134	3.4076	3.3815	2.8758	2.9058	3.0396	3.1568	3.2484	2.8693	3.1686	2.9996	3.2205	3.0396	3.1044	3.0396	2.8527	3.1568	3.3998	2.9058	2.92	2.92	3.3633	3.3366	2.7068	2.7417	2.8937	3.0337	3.1303	2.7049	3.0395	2.8475	3.1073	2.8937	2.9747	2.8937	2.6783	3.0337	3.3103	2.7417	2.7573	2.7573	3.2571
314	8.345	7.0565	8.1961	7.8773	8.031	7.8736	7.8683	7.8736	7.6976	8.0368	8.33	7.4989	7.6955	7.6955	8.7519	8.4818	7.2974	7.5087	7.8816	8.0431	8.3507	7.0665	8.2025	7.886	8.0365	7.8816	7.8754	7.8816	7.7091	8.0431	8.3336	7.5087	7.7051	7.7051	8.7568	8.4867	7.0794	7.3082	7.7067	7.8766	8.2026	6.8257	8.0466	7.7127	7.8683	7.7067	7.6983	7.7067	7.5264	7.8766	8.1813	7.3082	7.52	7.52	8.6298
315	310.66	222.93	293.5	293.21	309	293.21	298.34	276.26	256.05	293.49	337.02	250.19	267.43	275.97	332.82	320.69	218.08	239.89	245.61	262.84	288.58	200.85	271.42	271.12	286.92	271.12	276.26	254.18	233.97	271.41	314.94	228.11	245.34	253.89	310.73	298.61	196.91	218.71	224.44	241.67	267.4	179.67	250.25	249.95	265.74	249.95	255.08	233	212.79	250.23	293.77	206.94	224.17	232.72	289.56
316	388.02	248.61	352.27	346.33	364.89	346.33	344.28	313.32	309.62	337.75	389.01	281.56	305.99	321.9	433.36	396.54	242.08	263.4	271.82	296.25	357.05	217.65	321.31	315.37	333.92	315.37	313.32	282.36	278.66	306.79	358.05	250.6	275.03	290.94	402.4	365.58	238.38	259.7	268.12	292.55	353.35	213.95	317.61	311.67	330.22	311.67	309.62	278.66	274.96	303.09	354.35	246.9	271.33	287.24	398.7
317	40.701	40.701	40.701	40.701	40.701	40.701	40.701	54.268	40.701	54.268	40.701	40.701	40.701	40.701	40.701	40.701	54.268	54.268	81.402	81.402	54.268	54.268	54.268	54.268	54.268	54.268	54.268	67.835	54.268	67.835	54.268	54.268	54.268	54.268	54.268	54.268	40.701	40.701	67.835	67.835	40.701	40.701	40.701	40.701	40.701	40.701	40.701	54.268	40.701	54.268	40.701	40.701	40.701	40.701	40.701
318	27.134	27.134	27.134	27.134	27.134	27.134	27.134	27.134	27.134	27.134	27.134	27.134	27.134	27.134	27.134	27.134	27.134	27.134	54.268	54.268	27.134	27.134	27.134	27.134	27.134	27.134	27.134	27.134	27.134	27.134	27.134	27.134	27.134	27.134	27.134	27.134	27.134	27.134	54.268	54.268	27.134	27.134	27.134	27.134	27.134	27.134	27.134	27.134	27.134	27.134	27.134	27.134	27.134	27.134	27.134
319	17.742	17.742	17.742	17.742	35.485	17.742	17.742	17.742	17.742	17.742	58.91	17.742	17.742	17.742	17.742	17.742	17.742	17.742	17.742	17.742	17.742	17.742	17.742	17.742	35.485	17.742	17.742	17.742	17.742	17.742	58.91	17.742	17.742	17.742	17.742	17.742	5.6826	5.6826	5.6826	5.6826	5.6826	5.6826	5.6826	5.6826	23.425	5.6826	5.6826	5.6826	5.6826	5.6826	46.85	5.6826	5.6826	5.6826	5.6826
320	23.425	23.425	23.425	23.425	41.168	23.425	23.425	41.168	17.742	41.168	64.593	23.425	23.425	23.425	29.108	23.425	41.168	41.168	41.168	41.168	41.168	41.168	41.168	41.168	58.91	41.168	41.168	58.91	35.485	58.91	82.335	41.168	41.168	41.168	46.85	41.168	11.365	11.365	11.365	11.365	11.365	11.365	11.365	11.365	29.108	11.365	11.365	29.108	5.6826	29.108	52.533	11.365	11.365	11.365	17.048
321	195.2	111.74	140.94	174.97	165.86	174.97	182.88	113.95	150.05	129.75	150.05	120.2	136	159.16	208.6	187.85	58.616	46.992	45.016	60.825	126.27	42.808	72.015	106.04	96.933	106.04	113.95	45.016	81.124	60.825	81.124	51.266	67.075	90.233	139.67	118.92	94.724	83.1	81.124	96.933	162.38	78.916	108.12	142.15	133.04	142.15	150.05	81.124	117.23	96.933	117.23	87.374	103.18	126.34	175.77
322	27.658	27.658	52.449	27.658	27.658	27.658	27.658	40.608	27.658	40.608	43.34	27.658	27.658	27.658	27.658	27.658	40.608	71.992	55.316	55.316	40.608	40.608	65.398	40.608	40.608	40.608	40.608	53.557	40.608	53.557	56.289	40.608	40.608	40.608	40.608	40.608	27.658	59.043	42.367	42.367	27.658	27.658	52.449	27.658	27.658	27.658	27.658	40.608	27.658	40.608	43.34	27.658	27.658	27.658	27.658
323	64.126	64.126	75.491	64.126	81.868	64.126	64.126	95.435	58.443	95.435	105.29	77.693	77.693	64.126	69.808	77.693	95.435	95.435	122.57	122.57	95.435	95.435	106.8	95.435	113.18	95.435	95.435	126.74	89.753	126.74	136.6	109	109	95.435	101.12	109	52.066	52.066	79.2	79.2	52.066	52.066	63.431	52.066	69.808	52.066	52.066	83.375	46.383	83.375	93.233	65.633	65.633	52.066	57.748
324	296.39	206.27	286.36	262.37	278.4	262.37	280.41	263.32	246.33	277.35	306.41	236.29	250.32	248.35	335.43	312.39	203.2	235.26	246.2	260.23	279.3	189.17	269.26	245.28	261.3	245.28	263.32	246.22	229.24	260.25	289.32	219.2	233.22	231.25	318.33	295.29	186.21	218.28	229.21	243.24	262.31	172.18	252.27	228.29	244.32	228.29	246.33	229.24	212.25	243.26	272.33	202.21	216.24	214.27	301.35
325	904	296	783	570	711	582	596	582	458	696	979	416	485	485	1283	1042	338	404	568	681	887	286	767	556	696	568	582	568	445	681	962	404	472	472	1263	1024	263	320	464	565	749	219	641	453	579	464	477	464	355	565	819	320	379	379	1089
326	25	13	22	22	21	20	20	20	21	21	23	18	20	20	32	27	16	18	20	21	25	13	22	22	21	20	20	20	21	21	23	18	20	20	32	27	14	16	18	19	23	11	20	20	19	18	18	18	19	19	21	16	18	18	30
327	90	52	86	72	74	72	70	72	74	76	84	62	68	68	112	96	60	64	74	78	92	54	88	74	76	74	72	74	76	78	86	64	70	70	114	98	60	64	74	78	92	54	88	74	76	74	72	74	76	78	86	64	70	70	114
328	4.4591	4.6576	2.6061	3.0612	0.2758	5.0708	1.8842	3.4762	-0.4798	-1.0468	-0.1068	4.9154	5.2783	2.2497	2.4579	3.0063	4.8552	3.5249	-6.5678	-8.2689	2.4911	-6.283	2.2376	3.974	2.8046	5.106	4.5395	3.5255	2.2018	4.3791	2.4067	5.0903	5.1258	4.1665	3.0569	0.3528	4.81	-5.7764	-3.7463	-9.4006	-5.7462	-2.493	2.6911	4.3179	1.0228	3.501	-5.3803	-5.3792	4.4849	-3.8463	-1.7074	5.2388	5.3315	-5.5119	-2.4965
329	-0.4179	-3.5177	1.462	0.1155	5.4714	-1.8303	0.5436	2.9209	3.7302	1.0445	0.8661	-3.4468	-2.9074	-2.6825	2.2369	-0.7231	2.7734	-1.1562	-0.1487	3.2102	-0.6117	-1.744	-1.7298	1.3908	-4.2068	1.9684	2.4456	0.5341	-4.8418	1.859	-5.35	0.7087	0.9015	-1.9697	-1.805	6.1617	-0.4528	-1.3211	0.7965	-1.6774	-2.177	-0.0159	-0.7216	1.3586	-0.2423	-4.0445	-0.882	-0.2294	-2.9909	-3.2235	-2.4586	-2.0634	-0.4655	-2.2321	2.3908
330	2.3263	-0.3586	0.7336	-1.0989	2.3102	-1.854	-0.9178	-1.3029	1.3274	-0.5483	0.694	1.6317	-1.2158	0.4904	-0.9073	-0.6269	1.0889	2.2314	0.0637	-0.0311	-0.3594	-0.2163	-1.0599	0.0193	-1.6517	-0.2497	0.5342	-2.0208	-0.5668	-1.2933	1.1608	1.6462	0.9403	0.7083	1.1519	-0.9302	1.2343	0.4539	-1.5602	0.3456	-0.5434	-1.2486	0.8485	-2.0489	-3.2798	-0.7377	0.6545	-0.4507	0.5653	-0.6617	0.2711	-0.8659	-0.5225	0.806	0.6391
331	0	0	0	0	0	0	0	0	0	0	-1E-13	0	0	0	0	0	0	0	0	0	0	0	0	0	0	0	0	0	0	0	0	0	0	0	3E-14	0	0	0	0	0	0	0	0	0	0	0	0	0	0	0	-9E-14	0	0	0	-1E-14
332	0	0	0	0	0	0	0	0	0	0	-1E-13	0	0	0	0	0	0	0	0	0	0	0	0	0	0	0	0	0	0	0	0	0	0	0	3E-14	0	0	0	0	0	0	0	0	0	0	0	0	0	0	0	-9E-14	0	0	0	-1E-14
333	3689.4	1295.4	2082.1	2301.8	3170.2	1404.7	2936.7	2523.8	1464.9	2456.5	4387.9	1549.6	2254.4	1950.9	3281.4	5501.9	1298.7	1910.1	1892.4	2101	3070.9	929.58	1768.1	1328.7	2255.1	2252	2994.3	2151.3	1003.6	2226.1	3587.2	1266.1	1213.1	1795.9	3506.1	2058	957.64	1405.2	909.39	2313.3	1629.4	607.67	2307.5	1146.7	1925.3	1441	1423.9	1483.3	1075.4	1929.8	2840.8	1221.4	1283.4	1472.8	3384.6
334	930.08	545.95	1170.4	494.15	826.37	770.3	741.41	485.33	609.28	527.71	471.93	579.11	443.51	341.26	1602.9	516.16	232.67	268.33	300.64	521.97	668.03	444.67	866.06	557.74	424.7	450.06	577.68	464.02	487.16	776.37	725.55	322	414.61	361.68	996.68	1062.5	297.3	479.57	426.28	562.77	785.38	315.61	352.82	415.34	340.24	478.34	744.05	349.8	334.33	502.23	408.2	233.95	353.46	371.26	644.82
335	165.93	126.78	178.48	283.9	315.99	382.28	101.48	313.54	136.5	301.29	280.24	230.19	295.24	198.71	280.04	163.93	156.5	138.78	227.45	269.73	105.74	59.735	191.26	230.56	277.04	190.44	104.77	153.44	167.08	250.32	222.96	165.4	269.58	171.31	171.37	226.26	88.2	72.026	212.15	96.175	232.32	63.564	154.04	263.16	271.66	167.75	227.65	199.76	149.26	195.02	270.31	105.94	139.09	93.062	201.49
336	5.6751	22.825	7.5952	5.5152	8.9298	21.772	4.0046	18.14	11.503	1.5851	6.7094	23.817	23.06	12.369	7.4024	2.0824	15.435	4.252	12.069	13.173	2.6722	16.677	7.2553	8.5228	10.318	20.674	19.299	10.856	15.859	17.807	20.059	12.489	15.423	3.241	6.5917	17.636	5.9795	20.052	12.521	25.463	23.876	4.635	4.5927	5.1148	12.83	13.737	20.414	20.831	16.631	7.0385	15.013	15.568	20.736	22.792	5.1422
337	-83904	-61278	-83940	-75708	-80960	-75646	-76595	-80313	-71425	-83959	-90502	-72265	-75859	-72071	-94883	-91299	-68664	-73187	-86172	-89734	-87677	-65065	-87699	-79484	-84727	-79427	-80322	-84109	-75183	-87691	-94253	-76062	-79644	-75891	-98654	-95021	-59805	-64234	-77263	-80790	-78722	-56213	-78810	-70580	-75806	-70537	-71410	-75198	-66286	-78839	-85373	-67159	-70732	-66930	-89755
338	-554235	-319721	-544239	-495313	-541840	-506312	-488048	-477900	-455605	-534487	-628026	-408157	-451757	-465897	-688120	-595125	-378395	-399104	-485467	-557971	-556249	-329566	-539459	-499496	-558146	-483624	-471366	-485153	-454112	-514868	-610578	-423754	-461874	-440149	-676183	-619827	-322603	-358316	-435920	-475315	-523815	-287543	-485213	-445170	-503563	-452994	-443617	-445078	-405462	-468180	-584305	-375650	-411369	-399284	-619605
339	-112.21	-74.089	-87.053	-157.14	1.7615	-94.953	-143.4	-121.33	-90.581	-180.27	20.401	-125.54	-132.41	-107.04	-81.637	-156.22	-131.3	-168.8	-301.39	-275.72	-144.89	-119.64	-106.02	-177.69	-21.282	-136.2	-129.32	-175.14	-108.33	-171.55	10.804	-180.06	-177.06	-186.53	-110.24	-139.37	-132.12	-54.081	-249.81	-190.53	-47.552	-127.04	-75.606	-146.43	38.332	-101.47	-75.9	-123.71	-67.724	-177.51	31.961	-134.63	-123.25	-83.29	-70.812
340	-8.629	-7.7183	-8.6027	-8.7424	-11.671	-7.7233	-8.6529	-8.3468	-8.2872	-8.8635	-11.532	-7.8982	-7.7412	-8.3562	-8.4798	-8.6836	-8.197	-9.2103	-5.3869	-4.8602	-9.609	-8.0581	-8.9869	-9.9721	-11.895	-7.6506	-7.7624	-9.0337	-8.1803	-7.8404	-10.454	-8.5523	-8.0896	-10.333	-8.3433	-8.0129	-10.163	-7.7323	-5.1198	-4.3927	-7.6938	-10.256	-8.9854	-10.109	-11.082	-8.1508	-7.9055	-7.9921	-7.8056	-10.268	-11.657	-8.4043	-7.9871	-7.511	-8.3147
341	8.629	7.7183	8.6027	8.7424	11.671	7.7233	8.6529	8.3468	8.2872	8.8635	11.532	7.8982	7.7412	8.3562	8.4798	8.6836	8.197	9.2103	5.3869	4.8602	9.609	8.0581	8.9869	9.9721	11.895	7.6506	7.7624	9.0337	8.1803	7.8404	10.454	8.5523	8.0896	10.333	8.3433	8.0129	10.163	7.7323	5.1198	4.3927	7.6938	10.256	8.9854	10.109	11.082	8.1508	7.9055	7.9921	7.8056	10.268	11.657	8.4043	7.9871	7.511	8.3147
342	0.3448	-1.5623	0.5875	0.5659	-3.2216	-1.4879	0.3544	-1.7435	-0.7012	0.4733	-3.3409	-1.6616	-1.4922	-0.4187	0.1211	0.0614	-0.8184	0.3701	3.4955	3.0533	0.2337	-1.3132	0.9287	0.8037	-3.0617	-1.0954	-1.1029	-1.0596	-1.2626	-1.1029	-3.7847	-0.3859	-0.9388	0.8534	0.273	-1.4736	0.8921	-1.1397	4.0022	1.5017	-1.3121	0.6983	0.9624	0.8236	-3.3899	-0.3794	-1.4279	-1.1468	-0.5502	0.6215	-3.5662	-0.5064	-1.4614	-1.2598	0.266
343	545.77	417.24	508.7	500	547.12	468.46	500.33	487.58	435.86	487.83	544.74	433.07	470.07	453.25	575.2	558.86	398.75	410.79	414.41	454.27	503.36	376.21	474.76	452.51	481.4	465.42	487.66	438.29	396.61	477.83	536.31	393.4	416.78	435.4	540.56	498.26	381.72	408.41	387.93	452.47	473.19	343.59	459.17	431.32	464.23	448.47	449.22	415.19	399.02	455.29	499.35	380.76	404.11	428.24	523.01
344	209.24	135.27	201.33	84.243	187.44	110.72	129.69	165.49	107.62	169.38	210.01	139.65	121.67	104.81	228.16	222.58	156.27	163.49	155.06	167.25	245.41	177.65	263.37	130.29	227.94	155.04	183.54	200.2	128.25	226.36	275.64	173.93	149.55	137.74	276.21	252.04	71.038	89.043	64.538	96.222	160.07	84.884	178.41	51.974	139.59	75.84	88.105	131.9	69.286	137.52	179.06	108.47	70.469	68.672	192.44
345	193.79	138.03	147.8	113.64	113.44	120.88	142.15	163.79	101.57	149.57	119.88	145.93	141.54	98.874	200.7	193.18	110.95	154.93	179.22	180.19	183.34	121.05	138.36	96.435	106.11	99.867	157.7	154.71	112.02	159.83	132.06	123.03	120.17	115.54	193.54	175.56	86.374	132.6	129.72	152.46	147.92	87.179	104.54	75.18	87.319	67.117	125.73	108.15	74.79	123.41	81.118	87.489	99.78	75.435	158.03
346	319.96	150.81	240.44	302.4	247.26	237.8	228.75	163.03	227.72	173.16	215.36	148.63	207.43	249.85	323.21	290.1	138.7	108.58	90.266	118.19	266.59	88.228	166.32	231.08	149.01	213.62	150.65	95.2	161.61	98.04	134.85	102.88	153.2	185.23	253.84	204.67	225.4	199.66	200.56	208.78	327.76	181.25	259.28	304.73	237.32	305.51	236.23	182.96	254.94	200.05	240.13	186.21	234.42	284.41	351.45
347	225.81	266.43	268.26	197.6	299.86	230.66	271.59	324.55	208.15	314.67	329.37	284.45	262.64	203.4	251.99	268.76	260.06	302.22	324.15	336.08	236.77	287.98	308.43	221.43	332.39	251.8	337.01	343.09	234.99	379.79	401.46	290.51	263.58	250.17	286.72	293.59	156.32	208.75	187.37	243.69	145.42	162.33	199.89	126.58	226.91	142.96	212.99	232.23	144.08	255.24	259.22	194.55	169.69	143.83	171.57
348	1110.5	596.93	1063	371.76	1174.5	488.6	631.99	957.04	467.38	979.51	1720.2	711.25	619.68	462.54	1288.4	1266.2	831.82	937.27	965.87	1041.8	1525.7	945.65	1630.3	693.54	1635.7	825.28	1061.4	1340	673.69	1515	2508.6	1044.1	897.74	733.22	1811.1	1663.2	284.58	393.22	317.01	472.64	784.34	340.05	869.4	208.21	817.87	303.81	393.48	709.09	272.71	739.32	1393.8	508.27	330.22	275.1	1008.4
349	1028.4	609.14	780.36	501.49	597.39	533.43	692.68	947.19	441.11	864.97	862.09	743.23	720.85	436.33	1133.5	1099	590.57	888.2	1295.6	1302.6	1139.8	644.35	856.43	513.32	655.34	531.59	911.99	1035.5	588.46	1069.8	1069.8	738.57	721.37	615.01	1269.2	1158.5	346.01	585.55	766.89	901.33	724.79	349.24	509.45	301.17	424.28	268.87	561.51	581.43	294.37	663.47	550.3	409.97	467.57	302.19	828.22
350	15.455	2.7669	53.533	29.395	74.002	10.159	12.455	1.7028	6.0486	19.807	90.127	6.2808	19.863	5.9399	27.46	29.392	45.323	8.5583	24.158	12.935	62.069	56.605	125.01	33.857	121.83	55.174	25.84	45.494	16.224	66.522	143.58	50.901	29.381	22.206	82.664	76.472	15.335	43.554	65.179	56.237	12.153	2.2949	73.866	23.206	52.273	8.7228	37.626	23.746	5.5041	14.108	97.942	20.977	29.311	6.7623	34.416
351	82.018	12.21	282.65	129.72	577.14	44.833	60.695	9.8473	26.269	114.54	858.11	31.988	101.16	26.213	154.87	167.21	241.26	49.065	329.7	260.75	385.89	301.31	773.83	180.22	980.35	293.69	149.43	304.49	85.224	445.23	1438.8	305.56	176.37	118.2	541.83	504.64	61.434	192.33	449.88	428.69	59.552	9.1934	359.95	92.963	393.59	34.944	168.04	127.66	21.664	75.846	843.5	98.297	137.35	27.09	180.18
352	1.035	1.0892	1.103	1.0184	1.0174	1.0121	1.0785	1.1211	1.0584	1.0968	1.0589	1.1232	1.083	1.031	1.0627	1.0703	1.1265	1.2073	1.2403	1.1903	1.0799	1.1606	1.1421	1.0723	1.059	1.0607	1.1205	1.1781	1.1237	1.1408	1.098	1.1873	1.1517	1.0825	1.0972	1.1112	1.0476	1.1137	1.1807	1.1203	1.0246	1.0906	1.0927	0.9893	1.0033	0.9931	1.0681	1.1135	1.052	1.0842	1.0591	1.111	1.0825	1.0125	1.0578
353	5.0468	5.8478	3.077	3.2545	5.9455	5.7009	2.1652	4.7237	3.9883	1.5771	1.115	6.2212	6.1475	3.5351	3.4451	3.155	5.6965	4.329	6.5698	8.8702	2.5901	6.5241	3.0204	4.2104	5.3189	5.478	5.184	4.0985	5.3491	4.9301	5.9801	5.3966	5.2887	4.6627	3.7322	6.2415	4.9864	5.9429	4.1356	9.5553	6.1688	2.7882	2.9125	4.9687	3.4442	5.3999	5.4913	5.4029	5.4203	5.0619	3.0056	5.6967	5.3772	6.0011	3.5152
354	51.33	24.926	7.5642	15.677	25.618	34.421	1.8568	11.078	34.218	-7.3379	-58.12	27.604	34.292	35.461	28.516	28.498	17.072	-10.185	-10.87	26.646	11.929	8.1315	-5.7249	-5.9824	10.102	14.188	10.431	-8.6388	20.194	0.1812	-64.901	11.006	11.53	14.877	8.7696	29.771	33.485	31.825	-3.977	41.431	48.76	6.702	16.062	40.666	37.903	39.835	36.995	12.352	43.069	28.692	-72.4	29.808	31.584	36.706	34.914
355	7.3116	4.2538	4.2624	7.6062	8.4697	9.2713	14.717	7.8242	12.654	19.008	13.069	6.439	8.2101	17.307	6.9192	6.9292	5.7399	5.6209	18.405	8.5	5.2572	3.8345	8.1809	7.8296	12.382	8.6879	6.3558	8.2169	13.588	7.0724	8.8686	7.2318	9.6439	9.2964	7.5464	5.1526	8.2201	7.5191	12.309	10.973	9.2258	14.584	7.4859	12.397	16.912	9.0541	10.255	9.2277	13.434	9.7653	15.12	7.5268	10.298	8.3339	9.5114
356	1.8656	4.8473	-63.547	-54.415	-3.4203	3.4363	-66.333	-38.03	-1.8797	-85.099	-131.57	-5.3897	-1.9974	-8.2503	-54.635	-57.268	-15.86	-84.963	-86.976	-14.002	-75.371	-12.683	-79.426	-81.781	-35.202	-28.403	-29.14	-62.413	-33.624	-42.339	-105.38	-33.108	-37.837	-26.029	-75.073	-17.559	5.4832	2.9973	-100.64	9.4788	5.5973	-47.197	-47.939	4.9433	-3.1947	11.225	4.2065	-28.165	8.4441	-8.6848	-135.26	-5.4922	-4.6065	7.1999	-44.519
357	33.705	17.288	-22.08	-11.799	16.591	21.943	-22.614	-9.1187	14.435	-37.432	-89.071	14.49	19.398	10.605	-3.0348	-1.6188	4.3861	-38.311	-43.966	8.9507	-20.043	0.5277	-38.182	-34.307	-9.7312	-2.3211	-4.8967	-30.421	-7.6273	-18.142	-77.407	-4.4457	-9.0958	-1.0619	-21.838	13.934	19.058	17.625	-45.501	26.349	32.698	-16.398	-11.967	23.311	12.656	28.715	20.494	-5.33	22.223	8.2562	-93.344	16.421	15.552	24.158	3.8171
358	0.1583	0.0089	0.7007	0.9674	0.1087	0.0797	0.1022	0.2155	0.0817	0.1769	2.0842	0.0119	0.1025	0.2061	0.7997	1.825	0.3527	1.4561	1.6255	0.1968	1.3352	0.0044	2.11	1.1287	0.1439	0.1291	0.0656	0.203	0.0865	0.1722	0.1506	0.1633	0.1907	0.1864	1.5636	0.0313	0.0769	0.0696	1.2908	0.1541	0.0455	0.1807	1.4342	0.2323	0.2017	0.1397	0.0693	0.3663	0.0112	0.1405	0.574	0.1642	0.0424	0.1438	1.3938
359	-88.264	-100.56	-67.519	-63.45	-133.43	-98.097	-54.262	-87.392	-79.225	-57.561	-100.16	-104.39	-106.62	-72.259	-72.115	-65.467	-112.44	-67.831	-146.58	-204.54	-62.131	-113.02	-72.276	-65.816	-136.38	-85.891	-92.884	-76.12	-101.92	-83.01	-111.17	-104.78	-95.895	-96.942	-82.513	-120.06	-88.129	-91.523	-118.98	-198.83	-90.331	-53.366	-63.24	-86.389	-117.87	-95.931	-90.908	-85.049	-92.917	-92.904	-75.28	-91.438	-90.291	-91.777	-67.356
360	-0.1266	-0.0466	0.2353	0.348	0.3558	0.0677	0.6294	-0.0009	-0.2007	0.2202	0.221	-0.0996	-0.0477	0.0942	-0.5521	-0.1982	0.4297	0.0572	-0.0037	0.5295	-0.1172	0.0348	0.1322	0.1889	0.2958	-0.0505	-0.0963	-0.0551	0.1948	0.0186	-0.39	0.3611	-0.0989	-0.1368	-0.8101	0.1444	-0.412	-0.2662	-0.0101	-0.5053	-0.3797	-0.0965	-0.2567	-0.1825	-0.0073	0.0171	-0.2525	-0.2879	-0.5912	-0.432	-0.7788	-0.042	-0.2095	-0.4025	-1.0138
361	4.5792	1.7086	5.5368	6.571	3.0959	3.2201	5.2787	3.5609	3.0387	5.3523	6.8259	2.5732	3.3557	3.6308	7.4267	6.9896	2.7072	5.485	4.9018	3.1932	7.0184	1.6123	5.5601	6.4301	3.2599	3.9634	2.6449	3.9797	3.6128	3.1525	4.1754	3.2953	3.4937	3.3722	7.0641	3.7312	2.5071	2.362	7.1502	3.1955	3.9417	3.4618	5.8113	3.9836	2.8499	3.5668	3.0511	3.1313	2.8	2.925	6.1729	3.2959	3.1981	3.4195	7.4812
362	0.0112	0.0133	0.0202	0.0135	0.0205	0.007	0.0113	0.0038	0.0111	0.0057	0.0122	0.0057	0.2897	0.8177	0.0179	0.0226	0.0029	0.0038	0.0033	0.0039	0.0338	0.0192	1.5869	0.0067	0.0096	0.021	0.0107	0.0037	0.0048	0.004	0.0075	0.0058	0.0032	0.009	0.0144	0.0349	0.0025	0.003	0.0033	0.0045	0.0163	0.0072	0.0073	0.0089	0.0054	0.0085	0.016	0.0063	0.0039	1.6167	0.0122	0.0094	0.0099	0.0119	0.0133
363	5.703	1.714	18.909	11.982	-3.0037	-0.1609	3.7434	8.5969	4.209	5.3366	8.7522	4.1892	3.2732	3.6172	16.957	14.571	3.4566	15.507	8.1677	5.2762	18.478	2.1179	16.474	12.747	3.7514	3.7795	6.3573	9.4372	10.339	7.9072	-0.2986	4.4003	7.3968	3.2208	15.244	6.7772	4.0347	4.5158	20.785	1.2642	3.228	4.9702	13.554	0.9257	5.2908	-1.6579	3.3785	5.2449	5.1926	8.0372	-0.1447	2.4416	2.7022	1.0533	13.725
364	31.839	12.44	41.466	42.616	20.012	18.507	43.719	28.911	16.315	47.668	42.502	19.88	21.396	18.855	51.6	55.649	20.246	46.653	43.01	22.952	55.328	13.211	41.243	47.474	25.471	26.082	24.243	31.992	25.997	24.198	27.974	28.662	28.741	24.967	53.235	31.494	13.575	14.628	55.137	16.87	27.101	30.799	35.973	18.367	15.85	17.491	16.288	22.835	13.779	16.941	41.918	21.914	20.159	16.958	48.336

Table S12 Amino acid descriptors (2D) used in this study.

	M	M	M	M	M	M	M	M	M	M	M	M	M	M	M	M	N	N	N	N	N	N	N	N	N	N	N	N	N	N	N	N	N	N	N	N	P	P	P	P	P	P	P	P	P	P	P	P	P	P	P	P	P	P	P
	F	G	H	I	K	L	M	N	P	Q	R	S	T	V	W	Y	A	C	D	E	F	G	H	I	K	L	M	N	P	Q	R	S	T	V	W	Y	A	C	D	E	F	G	H	I	K	L	M	N	P	Q	R	S	T	V	W
No.	MF	MG	MH	MI	MK	ML	MM	MN	MP	MQ	MR	MS	MT	MV	MW	MY	NA	NC	ND	NE	NF	NG	NH	NI	NK	NL	NM	NN	NP	NQ	NR	NS	NT	NV	NW	NY	PA	PC	PD	PE	PF	PG	PH	PI	PK	PL	PM	PN	PP	PQ	PR	PS	PT	PV	PW
365	0.3834	0.3242	0.3958	0.1685	0.3426	0.2363	0.2592	0.3394	0.2469	0.3472	0.3855	0.3225	0.2588	0.2313	0.3967	0.3983	0.3919	0.398	0.3742	0.3682	0.4875	0.4722	0.5547	0.2879	0.4735	0.3331	0.3764	0.4568	0.3234	0.4737	0.514	0.4421	0.3588	0.3164	0.511	0.5058	0.1861	0.218	0.1664	0.2127	0.3383	0.2471	0.3886	0.1205	0.3007	0.1691	0.1961	0.3177	0.1736	0.3021	0.3586	0.2849	0.1744	0.1604	0.3679
366	0.3551	0.3308	0.2905	0.2273	0.2073	0.258	0.2841	0.3359	0.233	0.3066	0.2201	0.337	0.3011	0.2181	0.3489	0.3457	0.2782	0.3771	0.4325	0.3966	0.3642	0.3218	0.2914	0.2131	0.2204	0.2146	0.3234	0.353	0.2825	0.3345	0.2462	0.3127	0.2883	0.2654	0.358	0.3524	0.2263	0.3247	0.3344	0.3369	0.3126	0.2537	0.2277	0.1743	0.1881	0.1497	0.2799	0.2605	0.1874	0.2711	0.1624	0.2298	0.2469	0.1761	0.3021
367	0.5863	0.3615	0.4727	0.6048	0.4519	0.5076	0.4572	0.3344	0.5224	0.355	0.3954	0.3432	0.4413	0.5512	0.5619	0.5191	0.3478	0.2643	0.2178	0.2602	0.5296	0.2345	0.3503	0.5107	0.3095	0.459	0.3089	0.2172	0.4075	0.2052	0.2514	0.2615	0.3676	0.4254	0.4696	0.4108	0.5905	0.4889	0.517	0.4614	0.6927	0.5275	0.5647	0.7065	0.5112	0.6812	0.5259	0.4407	0.6389	0.4394	0.4809	0.489	0.5801	0.6641	0.672
368	0.4137	0.6385	0.5273	0.3952	0.5481	0.4924	0.5428	0.6656	0.4776	0.645	0.6046	0.6568	0.5587	0.4488	0.4381	0.4809	0.6522	0.7357	0.7822	0.7398	0.4704	0.7655	0.6497	0.4893	0.6905	0.541	0.6911	0.7828	0.5925	0.7948	0.7486	0.7385	0.6324	0.5746	0.5304	0.5892	0.4095	0.5111	0.483	0.5386	0.3073	0.4725	0.4353	0.2935	0.4888	0.3188	0.4741	0.5593	0.3611	0.5606	0.5191	0.511	0.4199	0.3359	0.328
369	2.0346	1.4307	2.0897	0.7435	2.1467	1.043	1.2631	1.9628	1.0723	2.0079	3.1579	1.6423	1.3183	1.0205	2.2399	2.2658	2.0861	2.2816	2.3307	2.2933	3.0311	2.5136	3.4339	1.5327	3.3978	1.7732	2.1766	3.0572	1.6986	3.1706	4.6775	2.6541	2.154	1.684	3.3504	3.338	0.7455	0.9628	0.8172	1.0446	1.6576	0.9897	1.8934	0.4827	1.7618	0.6774	0.8759	1.7079	0.6834	1.6238	2.7912	1.3349	0.8172	0.6424	1.9281
370	1.8844	1.4599	1.534	1.003	1.0919	1.1387	1.3844	1.9426	1.012	1.7731	1.5826	1.7162	1.5335	0.9627	1.9707	1.9666	1.481	2.1622	3.1263	2.8673	2.2644	1.7127	1.8039	1.1344	1.3613	1.1422	1.8701	2.3625	1.4837	2.2388	1.9948	1.8774	1.7308	1.4125	2.348	2.3252	0.9064	1.4337	1.9769	1.992	1.5317	1.0164	1.1095	0.6983	0.9139	0.5995	1.25	1.4004	0.7377	1.4572	1.102	1.0767	1.157	0.7057	1.5836
371	0.0659	0.1704	0.1086	0.1526	0.0848	0.2833	0.066	0.1136	0.1482	0.1091	0.1025	0.1457	0.1438	0.1011	0.1025	0.0536	0.1818	0.1326	0.1521	0.1343	0.0587	0.1174	0.1065	0.157	0.1215	0.136	0.0661	0.112	0.2048	0.1472	0.0889	0.1923	0.2129	0.1117	0.0608	0.1431	0.1723	0.0833	0.2012	0.0703	0.1593	0.164	0.0778	0.2207	0.1282	0.1405	0.1831	0.157	0.1604	0.1137	0.1167	0.1444	0.188	0.0861	0.064
372	5.0247	24.546	7.552	3.8712	12.596	22.363	2.6714	18.964	13.994	2.4377	13.574	20.136	20.041	13.884	6.4806	5.3926	18.973	5.3354	16.396	9.9476	3.5631	19.007	8.0069	7.2765	9.1012	21.306	18.753	6.2227	18.233	18.737	24.488	13.941	14.063	19.095	6.4733	18.392	5.2383	22.958	3.9944	14.553	25.139	4.7739	4.8143	20.964	12.635	16.326	17.589	23.072	14.13	4.8827	18.167	17.084	16.322	23.857	4.6262
373	-84947	-62279	-85009	-76787	-82063	-76701	-78460	-81369	-72451	-85052	-91598	-73324	-76927	-73103	-95936	-92387	-68886	-74234	-86469	-90084	-87939	-65279	-87989	-79764	-85045	-79695	-81376	-84442	-75428	-87970	-94543	-76328	-79929	-76088	-98926	-95299	-60039	-65228	-77531	-81158	-78925	-56430	-79079	-70762	-76056	-70780	-72456	-75439	-66515	-79127	-85669	-67396	-71001	-67162	-90001
374	-544705	-316355	-535587	-486050	-524716	-486664	-478129	-472573	-443666	-522385	-591738	-403513	-442577	-450053	-675322	-587546	-367709	-394646	-473567	-526595	-549366	-321652	-538416	-490544	-536347	-475066	-468218	-469523	-440759	-506799	-595362	-412481	-457046	-437449	-674146	-600347	-315427	-354111	-425765	-471123	-513663	-281252	-479339	-447292	-490989	-442803	-440862	-433674	-400984	-460507	-556488	-368233	-412473	-395262	-614377
375	-101.69	-40.902	-88.673	-136.38	24.704	-51.382	-134.8	-77.411	-56.677	-157.38	70.561	-90.667	-91.267	-55.907	-79.989	-151.2	-77.162	-149.07	-259.48	-270.91	-127.12	-72.774	-110.11	-146.88	8.388	-79.264	-83.786	-180.32	-68.363	-109.54	80.398	-127.78	-127.27	-73.832	-104.1	-96.48	-130.37	-39.767	-219.52	-243.26	-7.5014	-123.73	-98.917	-43.641	101	-58.494	-59.005	-79.323	-52.758	-164.99	55.749	-93.672	-94.959	-43.843	-76.954
376	-9.4819	-7.2824	-9.4382	-9.8099	-12.44	-7.4295	-9.7454	-7.9029	-7.9067	-9.8337	-12.339	-7.6032	-7.4518	-8.0445	-8.5449	-9.0046	-7.5942	-10.144	-4.9355	-4.8108	-9.5033	-7.5596	-9.2081	-10.361	-12.755	-7.4149	-7.6437	-10.669	-7.5695	-7.6034	-9.8463	-8.2685	-8.202	-7.5955	-8.3531	-7.5317	-10.492	-7.3844	-3.1398	-4.4773	-7.3569	-10.553	-9.1786	-7.5592	-10.582	-7.8431	-8.0888	-7.5707	-7.9017	-10.593	-11.753	-7.9676	-8.1116	-7.166	-8.3981
377	9.4819	7.2824	9.4382	9.8099	12.44	7.4295	9.7454	7.9029	7.9067	9.8337	12.339	7.6032	7.4518	8.0445	8.5449	9.0046	7.5942	10.144	4.9355	4.8108	9.5033	7.5596	9.2081	10.361	12.755	7.4149	7.6437	10.669	7.5695	7.6034	9.8463	8.2685	8.202	7.5955	8.3531	7.5317	10.492	7.3844	3.1398	4.4773	7.3569	10.553	9.1786	7.5592	10.582	7.8431	8.0888	7.5707	7.9017	10.593	11.753	7.9676	8.1116	7.166	8.3981
378	0.0246	-2.0522	0.4504	0.8843	-4.2667	-1.8869	0.6658	-2.0448	-1.4093	0.6439	-4.4436	-1.691	-1.6192	-1.0518	-0.0485	-0.0197	-1.5756	0.5721	3.0237	3.6077	0.0189	-1.7691	0.6448	0.6396	-4.4513	-1.7846	-1.6986	0.3516	-1.7351	-1.78	-4.2097	-0.9217	-1.0924	-1.8522	0.1212	-1.6561	0.7736	-1.6504	3.1542	3.6954	-1.7281	0.7258	0.7002	-1.6911	-3.4801	-0.972	-1.0311	-1.654	-0.8073	0.6445	-4.2835	-1.0621	-0.9691	-1.6755	0.0966
379	0.2327	0.3579	0.4092	0.2761	0.2852	0.5195	0.2276	0.264	0.3579	0.2749	0.1547	0.374	0.2703	0.235	0.3768	0.1125	0.2541	0.1847	0.2408	0.3018	0.2068	0.3669	0.4014	0.4178	0.2617	0.237	0.189	0.2361	0.4387	0.3419	0.2199	0.3069	0.4202	0.247	0.2594	0.4127	0.3072	0.2891	0.4779	0.2291	0.4207	0.4107	0.1905	0.4343	0.27	0.3366	0.4391	0.2996	0.3429	0.2866	0.2087	0.2335	0.3009	0.2517	0.21
380	0.8382	0.7792	0.6999	0.9253	0.8718	0.823	0.8265	0.9434	0.7723	0.9223	0.9605	0.8372	0.9459	0.9373	0.7364	0.9419	0.9502	0.9401	0.9666	0.9038	0.8497	0.72	0.7438	0.8265	0.9446	0.9037	0.8697	0.881	0.7855	0.8248	0.8834	0.9013	0.9108	0.9117	0.8167	0.7321	0.8334	0.786	0.8396	0.8378	0.7715	0.727	0.9253	0.9025	0.9697	0.8382	0.7686	0.918	0.8687	0.8737	0.9574	0.912	0.869	0.8492	0.89
381	15.493	20.689	7.2193	5.7231	9	20.243	5.0331	17.673	10.557	2.2093	2.0608	22.972	22.777	11.339	6.4249	4.5781	15.854	7.8983	13.034	11.737	3.294	19.117	3.6794	8.6989	6.0274	19.527	19.562	15.332	16.706	17.451	14.511	18.969	13.741	17.128	5.4073	16.955	12.255	20.767	8.3399	21.226	21.3	5.1682	3.1298	10.794	14.314	17.318	13.724	19.237	17.283	12.349	15.478	19.168	19.276	20.503	4.7737
382	-77415	-56169	-76048	-70013	-74297	-69959	-70858	-73222	-65814	-76725	-81767	-66386	-69831	-66529	-87023	-84248	-62046	-66377	-78073	-81482	-79891	-58588	-78460	-72433	-76731	-72386	-73231	-75639	-68217	-79099	-84180	-68814	-72280	-68936	-89445	-86620	-54632	-58901	-70637	-74025	-72413	-51217	-71041	-64969	-69273	-64958	-65813	-68222	-60792	-71684	-76785	-61392	-64828	-61506	-82020
383	-545740	-318665	-527035	-486113	-532368	-499111	-475024	-466238	-440908	-521805	-609678	-401270	-440202	-449485	-668362	-585823	-363645	-392836	-469354	-541156	-544809	-314625	-527846	-493881	-545972	-472347	-459463	-465546	-445480	-500427	-592153	-406554	-454398	-437624	-661595	-607532	-321480	-346351	-442662	-474750	-516496	-279784	-477956	-452561	-493773	-438427	-439826	-432105	-394786	-469128	-550860	-364328	-404777	-392851	-613541
384	-49.589	-78.404	-104.45	-148.42	20.235	-97.003	-141.84	-119.2	-102.72	-178.98	86.81	-121.18	-123.59	-109.15	-84.606	-151.15	-124.15	-158.67	-306.28	-271.57	-140.51	-111.78	-128.68	-182.13	-28.311	-137.39	-128.65	-151.1	-118.93	-167.47	18.757	-160	-182.28	-130.95	-120.63	-138.1	-99.124	-74.667	-257.63	-206.54	-41.143	-131.63	-99.681	-106.42	41.19	-97.036	-97.173	-123.34	-84.823	-138.73	23.719	-127.66	-109.48	-90.262	-85.835
385	-8.3143	-8.1753	-9.0869	-9.2794	-11.984	-8.0715	-9.1695	-8.4918	-8.8684	-9.3385	-11.771	-8.2026	-8.1773	-8.899	-8.4777	-8.9779	-8.0162	-9.3134	-5.6428	-5.2133	-9.5626	-8.2345	-9.4195	-9.9498	-12.086	-7.9625	-8.0982	-8.671	-8.4433	-8.0775	-10.64	-7.9492	-8.5746	-8.0635	-8.4302	-8.2269	-8.824	-8.1524	-5.6144	-4.9776	-8.0805	-9.9565	-9.2204	-8.8495	-11.535	-7.9256	-8.4981	-8.5162	-7.8654	-9.0062	-11.432	-8.2388	-8.2972	-7.9512	-8.2902
386	8.3143	8.1753	9.0869	9.2794	11.984	8.0715	9.1695	8.4918	8.8684	9.3385	11.771	8.2026	8.1773	8.899	8.4777	8.9779	8.0162	9.3134	5.6428	5.2133	9.5626	8.2345	9.4195	9.9498	12.086	7.9625	8.0982	8.671	8.4433	8.0775	10.64	7.9492	8.5746	8.0635	8.4302	8.2269	8.824	8.1524	5.6144	4.9776	8.0805	9.9565	9.2204	8.8495	11.535	7.9256	8.4981	8.5162	7.8654	9.0062	11.432	8.2388	8.2972	7.9512	8.2902
387	-1.6697	-1.8459	0.1523	-0.0666	-3.6831	-1.8437	0.0488	-2.1999	-0.8195	-0.0305	-3.8911	-2.0486	-2.0284	-0.5874	-0.0472	-0.0715	-1.0744	-0.0248	3.2331	2.4447	0.0305	-1.8462	0.1246	0.2648	-3.273	-1.0909	-1.2836	-2.3828	-1.9142	-1.2394	-4.4778	-1.3068	-0.5068	-1.3256	0.0446	-1.8844	-0.5359	-1.4029	3.713	2.0243	-1.3215	0.4922	0.4395	-0.5323	-4.1447	-1.0996	-0.7219	-1.312	-1.0067	-0.6554	-4.1106	-1.279	-1.8032	-1.3152	0.1705
388	4785.4	1968.2	3430.9	3079.8	4312.5	2557.3	3779.6	3322.7	2210.7	3285.5	5140	2358.9	2993.2	2490.8	5164.3	6181.9	1687.9	2317.2	2420.5	2892.7	3844.7	1434	2825.4	2117	2956.8	2892.5	3676.8	2768.8	1657.9	3252.8	4535.7	1753.5	1897.3	2328.9	4674.2	3346.7	1343.1	1956.8	1547.8	2972.3	2647.1	986.85	2814.4	1825.2	2537.2	2087	2395.6	2032.9	1559	2627.1	3519.3	1561.3	1776	1937.1	4230.9
389	1075.5	659.22	1331.3	772.62	1140.4	1134.3	837.65	794.73	742.8	822.01	751.79	798.01	730.05	538.92	1841.5	677.08	389.14	402.79	528.07	791.62	773.38	504.19	1057.3	788.21	701.43	640.47	675.04	617.45	653.96	1026.7	948.5	487.39	684.02	532.96	1167.9	1287.9	385.48	545.32	638.36	658.93	1016.1	379.15	506.84	678.45	611.78	646.07	953	549.3	483.46	697.12	678.25	339.88	492.53	464.19	846.24
390	3873.8	1435.2	2277.2	2589.1	3486.1	1796.9	3041.5	2840.1	1603	2758.3	4668.1	1786.2	2555.1	2149.5	3599.4	5668.4	1455.1	2050.5	2119.8	2370.8	3177.1	989.53	1959.3	1559.3	2531.8	2442.2	3106.5	2304.5	1171	2476.4	3810	1431.4	1482.7	1967.2	3677.6	2284.8	1045.8	1482.4	1121.5	2409.5	1863.1	671.25	2461.5	1409.9	2197	1608.7	1667.9	1683.1	1224.7	2124.9	3111	1327.4	1422.5	1565.9	3586.1
391	4621.4	1841.9	3253.4	2798	3998.5	2183.4	3680	3010.6	2075.6	2990.7	4860.2	2133.6	2701.3	2293.3	4887.8	6018.4	1531.5	2181.2	2193	2623.1	3739	1374.3	2634.3	1886.5	2680.4	2702.3	3572	2615.7	1490.8	3002.5	4312.8	1588.2	1627.9	2157.8	4502.8	3120.7	1255	1886	1335.8	2876.1	2414.9	923.29	2660.3	1562.1	2265.6	1919.3	2170.2	1833.4	1409.8	2432.1	3249.4	1455.4	1636.9	1844.1	4029.5
392	4.0181	3.089	3.4614	3.4261	3.9358	3.122	3.6713	3.5523	2.9958	3.4418	4.0957	3.1596	3.458	3.167	3.9238	4.4485	2.8821	3.1384	3.1355	3.3341	3.7102	2.7533	3.2393	2.9379	3.3639	3.434	3.7367	3.3534	2.6893	3.5353	3.9595	2.8283	2.8522	3.1735	3.8319	3.3665	2.6857	2.9941	2.5986	3.4956	3.1767	2.394	3.3401	2.8276	3.2226	3.0236	3.1185	2.9779	2.7102	3.2862	3.5949	2.7787	2.8658	3.0068	3.747
393	3.7745	2.3167	2.8289	3.0396	3.6592	2.3357	3.2851	3.1075	2.5556	3.133	3.8382	2.6305	3.0546	3.1535	3.3501	4.2092	2.5654	2.8303	2.7716	2.8781	3.472	2.199	2.87	2.7048	3.208	3.1843	3.512	2.9194	2.1825	3.054	3.7305	2.3835	2.5404	3.0133	3.6517	2.7892	2.4443	2.7656	2.2987	3.1969	2.7432	2.0939	3.3643	2.5558	3.2264	3.0031	2.5604	2.7802	2.5879	3.0934	3.4953	2.637	2.5869	2.9698	3.6966
394	1.7013	1.7857	2.1412	1.653	1.8525	1.8747	1.8003	1.6867	1.7407	1.4987	1.4177	1.6575	1.4664	1.2825	2.235	1.4178	1.3196	1.1231	1.1005	1.4324	1.6505	1.7013	1.82	1.6046	1.4823	1.3408	1.4	1.4244	1.621	1.5402	1.5396	1.497	1.4131	1.2643	1.8114	1.9442	1.1811	1.3291	1.2756	1.3618	1.7863	1.2798	1.2117	1.4095	1.1671	1.3576	1.6892	1.247	1.2871	1.3215	1.3493	1.1536	1.2219	1.3696	1.6154
395	0.9691	0.9563	0.9323	1.1872	1.0657	1.2433	0.844	1.0475	0.9837	1.0347	1.2289	1.0042	1.1582	1.0027	1.0724	0.9745	1.094	1.0307	1.0809	1.0546	0.8412	0.7536	0.9365	1.0717	1.1183	1.1742	0.903	0.9768	0.9878	1.1717	1.1122	1.0453	1.1722	1.0073	0.9006	1.0553	1.0146	0.7983	1.031	0.8475	1.095	0.848	0.9381	1.2007	1.1553	1.1256	1.0955	1.1015	1.0365	1.043	1.1942	1.0019	1.1217	0.8715	0.9354
396	286.38	189.38	259.63	257.63	273.63	259.25	260	234.88	232.75	252.88	289.38	210.38	231.13	240.88	315.63	291.88	180.38	194.88	198.5	218.63	258.63	163	235.75	228.75	246.75	231.25	235	209	204	228.13	263.5	184.63	202.5	213.63	290.13	265.75	177.75	196	194.13	217.13	256	157.88	230.88	230.75	243.5	229.88	230.63	205.88	201.75	224.38	257.13	182	199.75	211.63	284.88
397	338.52	235.01	302.5	300.27	329.96	308.39	303.53	280.3	269.04	290.41	338.35	254.56	274.41	279.45	360.34	334.69	222.53	232.27	239.77	265.55	296.73	203.78	276.7	267.86	292.31	279.69	284.14	248.16	233.47	275.28	318.41	226	242.33	259.29	332.15	312.42	212.69	231.17	219.6	259.44	291.56	185.11	266.41	266.25	281.05	272.28	275.11	241.44	232.85	266.36	299.9	216.18	232.36	250.29	318.52
398	4.3571	3.7575	4.5927	4.7604	3.4885	3.6143	4.3328	4.1075	1.8477	4.1522	3.8059	4.3122	3.6055	1.2835	4.618	2.9961	0.6341	3.338	1.4358	1.1902	4.7246	1.4365	2.831	2.6535	3.7409	3.6339	3.6723	0.3506	3.9764	0.8737	1.5749	3.1356	1.5748	2.6211	4.5205	2.2832	3.5244	3.8708	2.919	4.3091	4.3295	3.3394	1.611	3.4783	3.8199	3.9238	4.3831	3.9487	5.1691	3.467	4.1736	5.0702	4.3032	3.3291	3.9768
399	0.1031	0.0643	0.072	0.0842	0.0686	0.0802	0.1218	0.0492	0.0963	0.0582	0.0576	0.047	0.0505	0.0641	0.1007	0.078	0.03	0.0381	0.0187	0.0221	0.049	0.0324	0.0385	0.0613	0.0304	0.0539	0.0442	0.0269	0.0409	0.0268	0.0302	0.023	0.0291	0.0391	0.0493	0.0429	0.1097	0.0893	0.0458	0.0474	0.1289	0.0839	0.0808	0.1288	0.0726	0.1154	0.1272	0.0519	0.1315	0.0609	0.083	0.0624	0.0677	0.0982	0.1499
400	2.763	2.9873	2.8195	2.7577	2.8648	2.8446	2.764	2.9157	2.8612	2.8796	2.8097	2.9684	2.8771	2.8221	2.7635	2.82	3.1134	3.0622	3.1502	3.0804	2.9356	3.2277	2.9928	2.9209	3.0109	2.9452	2.9102	3.1108	3.0976	3.0334	3.0105	3.1384	3.0722	2.9709	2.9043	2.9338	2.9393	2.8946	3.0256	2.927	2.7813	3.0798	2.9097	2.8017	2.8858	2.7651	2.7752	2.939	2.8934	2.9161	2.8339	3.0359	2.9277	2.8139	2.8112
401	2.2222	2.4621	2.3035	1.9541	2.4075	2.2299	2.0695	2.3998	2.3782	2.4314	2.3307	2.452	2.2908	2.0605	2.2674	2.3717	2.7737	2.8308	2.9083	2.8615	2.6178	2.9534	2.6825	2.3482	2.748	2.4898	2.4442	2.863	2.719	2.8036	2.7904	2.8491	2.7152	2.4875	2.6186	2.7075	2.2304	2.3568	2.5566	2.4339	2.2589	2.4651	2.3246	2.0289	2.4125	1.8922	2.0621	2.4207	2.2205	2.4353	2.3478	2.4951	2.2509	2.0026	2.2865
402	1.2673	1.7331	1.4836	1.2578	1.6728	1.441	1.2199	1.7909	1.535	1.6755	1.5683	1.7965	1.6422	1.4341	1.3913	1.4834	2.1555	2.1475	2.4868	2.4294	1.7654	2.4381	2.0714	1.7451	2.0454	1.8302	1.8236	2.4815	2.1325	2.3503	2.1768	2.2346	2.1918	1.955	1.8426	2.0028	1.4491	1.5803	1.9511	1.8518	1.2592	1.7525	1.6067	1.184	1.5761	1.1748	1.2422	1.7997	1.2343	1.7415	1.5888	1.7438	1.5789	1.2291	1.4033
403	0.6888	1.0022	0.8828	0.7707	0.8953	0.8757	0.6553	1.1132	0.828	0.985	0.9007	1.1325	1.0561	0.8833	0.7496	0.787	1.4328	1.2436	1.7391	1.5927	1.0869	1.4543	1.2986	1.0461	1.3377	1.1286	1.1345	1.535	1.3526	1.5363	1.3589	1.5429	1.4401	1.2118	1.0957	1.1356	0.7862	0.8058	1.2801	1.1438	0.686	0.9547	0.8581	0.6609	0.8477	0.69	0.6755	1.1287	0.7022	1.0247	0.8252	1.1059	0.9756	0.7162	0.6666
404	0.3425	0.5405	0.4776	0.4096	0.4298	0.4671	0.3353	0.6244	0.3876	0.549	0.5176	0.6411	0.6165	0.4658	0.3889	0.4063	0.7903	0.712	1.0321	0.8742	0.6294	0.8385	0.7527	0.5779	0.776	0.588	0.6402	0.8973	0.7215	0.8894	0.7943	0.9802	0.8403	0.683	0.6184	0.6726	0.3868	0.4295	0.6933	0.6315	0.3548	0.4908	0.436	0.3302	0.4404	0.3409	0.3465	0.6324	0.3384	0.5479	0.4259	0.5573	0.5319	0.3927	0.2823
405	0.1595	0.2314	0.2241	0.1909	0.2017	0.2047	0.156	0.2973	0.1466	0.2812	0.2559	0.3016	0.3043	0.2294	0.1808	0.1758	0.3238	0.3171	0.5826	0.4519	0.2957	0.4187	0.3709	0.2807	0.4502	0.2817	0.288	0.4723	0.3288	0.4374	0.3812	0.454	0.4194	0.2875	0.2873	0.3282	0.1372	0.2012	0.3202	0.2978	0.1564	0.2289	0.1964	0.1209	0.2257	0.1366	0.1391	0.2934	0.1485	0.2298	0.1861	0.2097	0.2471	0.1974	0.1144
406	0.0622	0.0957	0.0946	0.0658	0.0823	0.0805	0.069	0.118	0.0574	0.1252	0.1212	0.1151	0.108	0.1063	0.0689	0.0558	0.0937	0.1128	0.2904	0.1868	0.0922	0.171	0.1451	0.1138	0.1988	0.1337	0.1156	0.1802	0.1097	0.1889	0.1556	0.1407	0.1506	0.1092	0.1126	0.1468	0.0363	0.0758	0.1141	0.0941	0.0622	0.0977	0.0461	0.0343	0.117	0.0382	0.0435	0.1093	0.0406	0.0784	0.0736	0.049	0.0752	0.0773	0.0363
407	0.0229	0.0373	0.0471	0.0209	0.0315	0.0319	0.0203	0.0399	0.0215	0.0602	0.0627	0.0365	0.0331	0.0428	0.0167	0.0164	0.0135	0.0251	0.1015	0.0317	0.0153	0.054	0.0348	0.0322	0.0693	0.0471	0.0364	0.0451	0.0193	0.068	0.0364	0.0427	0.0371	0.0412	0.035	0.0512	0.0075	0.0235	0.0308	0.0254	0.0205	0.0293	0.0091	0.0049	0.0644	0.0073	0.0061	0.025	0.0068	0.0126	0.033	0.0102	0.0104	0.0267	0.0078
408	842.13	464.38	636.38	605.5	627	558.13	726.88	543.13	548.38	588.13	662.38	459.25	512.25	520.63	822.13	783	325.63	422.5	315.38	365	628.75	299.25	454.25	473.63	403.38	537.88	551.88	410.5	359.88	414	478.75	296.63	314.25	419.13	664.88	535.38	485.63	540.13	399	541	704.25	411.5	598.13	587.38	542	585.88	642.88	460.13	562.88	538.25	655	422.5	485.25	563.25	827.75
409	472.25	263.75	364.38	335.63	357.25	308.25	409.25	308.25	300.13	341.88	371.75	257.13	284.38	283.5	481.38	449.88	167.88	227.88	164.25	191.38	354	170.88	253.38	267.25	229.13	294.63	306.13	223.63	201.38	231.13	278.75	161.75	171	228.5	384.38	307.13	267.63	290.88	208.38	289.25	393.5	219.5	322.88	325	305.25	316.63	359.38	241.13	307	301	362.63	223.25	265.5	305	470.5
410	336.75	190.75	265.25	255.75	262.5	226	303.25	225.5	214.88	243.5	270.5	185.38	207.63	213.25	352	320.38	118	160.25	111	127.88	244.25	118.88	175.88	200.63	160	211.25	221.5	151	142.75	161	196.88	109.5	126.13	169.13	269.88	212.88	195.13	210.38	144.63	208.5	280.75	156	234.75	239.88	219.63	243.25	260.75	174.25	221.13	216.25	259.63	158.38	193.25	227.63	338.5
411	282.25	157.75	221.63	225.38	214.5	195.88	261.13	181.63	177	202.63	227.25	154.13	175	185.5	298.25	258.25	91.25	116.25	81.875	94.625	196.25	87.625	137.63	165	125.88	168.38	184.88	107.25	112.63	119.13	150.38	85.375	97.75	135.75	215.5	164	167.88	173.88	118.25	168.75	234.5	131	192.88	209	184.5	213.5	223.13	143.38	191.13	176.88	212.25	131.38	159.5	204	280.13
412	244	132.25	191.13	193.75	175	167.63	227.13	153.75	145.38	171.38	191.38	128.75	146.38	157.5	260.75	218.5	69.25	86.5	61.75	68.625	168.38	65.75	108.25	134.38	101.13	136.38	156	77.625	87.125	90.875	117.88	70.375	76	106.75	178.75	127.63	142.25	141.38	96.75	140.75	208.25	109.13	163.88	180.63	157.88	181.13	195.25	121.5	169.38	150	177.5	107.63	135.38	176.13	242.5
413	190.63	97.625	145	124.63	122	129.5	165.25	106.63	116.88	130.5	143.38	90.25	101.63	102.13	200.63	166.25	50.375	65.75	45.25	49	138	50	86.375	90.25	80.625	104.25	118.13	57	70.125	72	95.5	56.5	51.875	76.875	148.5	102.88	114.38	113.63	73.25	112.38	177	89.5	132.13	144.75	128.75	124.13	155.63	93.5	141.88	124.13	141.5	84.25	110	136.13	199.38
414	112.38	60	93.75	60.375	79.625	67.5	100.5	64.375	61	83.875	83.75	55.875	58	48.625	124.38	108.5	28.125	44.875	31	31.625	86.25	37.25	53.875	47.125	51.125	64.375	72	42.375	38.125	50	69.375	36.875	32.25	40.75	101.5	74.375	57.375	60.75	33.375	59.375	105.5	43.625	69.125	70.125	67.25	52.75	84.5	45.5	71.875	68.875	81.875	42	56.375	60.375	118.38
415	55.25	30.5	49.5	24.125	36.75	27.25	44.75	36.5	26.875	41	38.875	29	27	17.625	66.625	55.125	14.125	23.25	16.375	20	40.75	26.125	29	24.75	21.5	37.625	39	31	19.75	33	45.625	20.5	16.625	23.375	56.75	42.625	30.5	30.625	17	32.5	53.375	23	36.75	31	29.625	24	42.25	22.875	34.625	33.5	43.125	20.125	30.875	26.875	65.625
416	0.5	0.5	0.5	0.5	0.7071	0.5	0.5	0.5	0.5	0.5	0.5	0.7071	1.2247	6.265	0.5	0.7071	0.5	0.7071	0.5	8.2462	1.8708	0.5	2.2913	0.5	0.5	0.7071	0.7071	0.5	6.2249	0.5	0.7071	0.5	1.2247	0.7071	0.5	0.5	0.5	0.7071	0.5	0.7071	5.3852	0.5	6.0208	0.5	1.118	1.5811	0.5	0.5	7.2457	6.7082	0.5	0.5	0.5	0.7071	0.5
417	1.6583	1.8708	0.5	0.7071	1.4142	0.7071	0.5	0.5	0.7071	0.5	0.7071	0.866	1.5	0.7071	0.5	1.118	0.866	0.5	1.2247	0.5	2.5495	1.2247	0.5	0.866	6.8007	6.7268	0.7071	2.5495	6.7268	1.118	0.7071	7.5333	0.7071	0.5	0.7071	0.5	6.1033	1.118	1.2247	0.7071	4.9497	1.5	0.5	1.118	6.4807	2.0616	1.2247	0.5	0.5	0.7071	0.5	6.6708	0.7071	0.7071	0.5
418	1.5811	2.2913	0.7071	0.5	0.7071	0.866	1	1	0.5	0.7071	1.118	0.5	0.5	6.1033	0.7071	0.5	1.2247	0.5	1.118	8.5	0.7071	1.5	2.7386	1.2247	6.7454	6.9821	0.7071	2.1794	7.1063	0.7071	1.4142	7.5829	0.7071	0.5	0.5	0.7071	6.0828	0.866	1.6583	1.4142	0.7071	1.2247	6.0828	0.7071	6.8007	0.5	0.866	0.7071	7.0887	6.6708	0.7071	6.5383	1.118	1.4142	0.7071
419	1.8028	0.7071	0.7071	0.5	0.7071	0.5	0.5	0.7071	0.5	0.5	0.7071	0.5	0.5	0.7071	0.5	0.7071	0.5	0.7071	0.5	5.7009	0.5	8.9722	0.5	0.5	5.6125	0.7071	7.1414	1.2247	0.5	7.1764	0.7071	0.7071	0.5	0.7071	0.5	9.2736	0.7071	0.866	0.7071	0.5	0.5	0.5	0.5	0.5	0.7071	0.7071	5.3151	5.099	0.5	8.3066	0.5	0.7071	0.7071	0.5	0.7071
420	1.4142	0.5	0.5	0.7071	7.0178	0.5	0.5	0.7071	0.5	1	0.5	0.7071	0.7071	0.5	0.7071	0.5	10.735	0.5	0.7071	10.977	0.5	0.5	5.5902	8.6168	7.6322	1.118	7.5	0.7071	0.5	0.7071	0.5	0.866	4.1533	0.7071	0.5	0.866	0.5	0.7071	7.2801	0.7071	0.5	8.6313	0.5	0.7071	0.7071	0.866	0.5	0.5	0.7071	10.173	0.5	0.5	0.5	0.7071	1.118
421	0.5	1.118	0.5	0.5	7.0887	0.7071	0.7071	1.4142	0.7071	0.5	1.118	0.866	0.5	0.5	0.5	0.866	10.886	0.5	0.5	5.8737	0.7071	9.3941	5.1478	9.083	7.5333	0.5	0.5	1.8708	0.7071	7.4498	0.5	0.5	4.1231	1.4142	0.7071	8.9861	0.5	0.5	6.8191	0.5	0.7071	8.4113	1	0.5	0.7071	0.5	5.4314	5.0249	0.5	6.2849	0.7071	0.866	1.118	0.5	0.5
422	-2.4671	-2.6528	-3.1691	-2.6999	-2.6657	-2.597	-2.9224	-3.0035	-2.9729	-3.286	-2.8128	-2.5057	-2.3378	-2.1833	-2.9111	-2.8613	-2.3968	-2.4775	-2.3423	-2.4206	-2.58	-2.7907	-2.4428	-2.7997	-2.6858	-2.5075	-2.6236	-2.4909	-2.323	-2.3527	-2.9953	-2.4243	-2.6095	-2.5757	-2.8847	-3.125	-2.5627	-2.6185	-2.6791	-2.6182	-2.483	-2.4146	-2.4563	-2.5471	-2.5437	-2.4557	-2.4711	-2.4776	-2.3414	-2.6938	-3.2268	-2.1523	-2.5965	-2.8602	-2.9842
423	-2.4442	-2.6062	-3.0265	-2.6677	-2.4978	-2.3963	-2.8756	-2.9529	-2.9628	-3.1231	-2.6993	-2.499	-2.3218	-2.1261	-2.8674	-2.6911	-2.2943	-2.4747	-2.2344	-2.3671	-2.5768	-2.7667	-2.3651	-2.7554	-2.634	-2.4631	-2.5262	-2.4843	-2.3058	-2.3399	-2.884	-2.1528	-2.5396	-2.5463	-2.7669	-2.9888	-2.4197	-2.5774	-2.5228	-2.5526	-2.4269	-2.4016	-2.4391	-2.5293	-2.4999	-2.4381	-2.4446	-2.4067	-2.2872	-2.5502	-3.0121	-2.1367	-2.3857	-2.8032	-2.9806
424	-2.3986	-2.5702	-3.0113	-2.6618	-2.3683	-2.2378	-2.815	-2.893	-2.8903	-3.0259	-2.6912	-2.455	-2.2932	-2.0823	-2.8592	-2.541	-2.2696	-2.4532	-2.1778	-2.2417	-2.4945	-2.7203	-2.2982	-2.6959	-2.5652	-2.4489	-2.5181	-2.472	-2.2852	-2.3387	-2.8499	-2.1251	-2.5177	-2.4688	-2.7609	-2.9211	-2.3347	-2.5586	-2.4867	-2.5488	-2.2875	-2.401	-2.437	-2.4887	-2.4826	-2.4085	-2.3483	-2.3924	-2.2823	-2.5473	-2.8721	-2.094	-2.2963	-2.7691	-2.9153
425	-7.4896	-8.5421	-10.119	-6.9985	-8.2227	-7.8823	-7.3486	-9.3822	-8.1456	-7.8525	-9.8374	-8.4918	-7.6735	-8.2477	-7.155	-7.2824	-6.6216	-7.1288	-8.5545	-6.729	-6.9425	-7.622	-7.2863	-7.1095	-7.2104	-8.1966	-7.9228	-8.4531	-7.2479	-8.4865	-7.6779	-7.5806	-7.2023	-9.5573	-7.9671	-7.3565	-6.3744	-7.9803	-6.7479	-7.5549	-7.5977	-7.3934	-6.8674	-6.3653	-10.944	-6.6673	-6.6258	-7.0104	-6.421	-6.485	-8.5108	-6.4598	-6.6861	-8.3667	-6.4692
426	-7.4539	-7.994	-9.7314	-6.9269	-8.0405	-7.5916	-7.0081	-8.9378	-8.0739	-7.726	-9.5272	-8.1845	-7.4531	-7.8025	-7.1097	-6.8747	-6.3304	-6.9838	-7.956	-6.6156	-6.8846	-7.2224	-7.0922	-6.9542	-7.1631	-7.9819	-7.5542	-8.1984	-6.8534	-8.3456	-7.6541	-7.4846	-6.7155	-9.3041	-7.8643	-7.3183	-6.3477	-7.7622	-6.6988	-7.4937	-7.1047	-7.1618	-6.7117	-6.3436	-10.857	-6.3964	-6.4795	-6.9233	-6.3962	-6.4641	-8.3499	-6.4152	-6.5274	-8.1669	-6.4394
427	-7.2856	-7.5884	-9.5841	-6.8271	-7.633	-7.4428	-6.8595	-8.6433	-7.6585	-7.3145	-9.1758	-7.9098	-7.2818	-7.775	-6.7151	-6.8325	-6.3127	-6.7623	-7.7099	-6.4951	-6.8197	-7.1304	-6.8585	-6.8412	-7.1613	-7.8797	-7.269	-7.6152	-6.8427	-8.3335	-7.4445	-7.0437	-6.5972	-9.1615	-7.699	-7.2993	-6.2946	-7.7029	-6.6013	-7.3458	-6.9016	-6.6761	-6.5307	-6.2532	-10.057	-6.201	-6.3552	-6.8951	-6.3783	-6.4482	-7.6363	-6.2119	-6.4794	-7.4729	-6.4344
428	1.3123	1.2515	1.2918	1.2783	1.3281	1.2139	1.2761	1.296	1.2015	1.268	1.3084	1.2581	1.2937	1.2304	1.3105	1.3112	1.2313	1.2258	1.2478	1.2771	1.272	1.2207	1.2686	1.2325	1.2624	1.266	1.3039	1.276	1.1837	1.3161	1.3242	1.2262	1.2277	1.2547	1.2927	1.2609	1.1936	1.2262	1.193	1.2932	1.2265	1.1643	1.2349	1.1975	1.2398	1.2297	1.2375	1.2205	1.171	1.2698	1.2656	1.1864	1.2088	1.2328	1.2542
429	245.25	263	270.63	272.5	246.88	232.63	237	287.25	196	256.88	203.75	300.63	311.75	228.25	268	288.88	247	221.13	358.13	379.88	266.63	258.5	258.38	269.5	217.5	250.88	266.5	306.25	290	307.25	248.88	272.13	311.88	297.25	243	305.5	312.5	296.38	340.75	396.13	280.13	292	279.63	285.75	258.38	255	294.38	269.5	276.63	322.75	195.75	295.25	318.63	266	266.63
430	287.5	333.75	356.63	323.38	298.38	302.38	278.75	371.38	290.25	328.25	227	395.13	389.13	285.25	327	361.63	341.25	341.63	511.75	567.75	352.63	360.13	349.25	338.88	273.25	298.38	333.13	409.88	390.63	393.5	314.25	380	434	370.88	336.88	394.75	379.13	396.38	501.13	563.25	372.25	363.63	369.5	355.25	320.13	323.88	351.25	384.63	351.75	435.75	259.75	432.5	423.13	349.25	329.75
431	214.88	254.63	273.13	245.25	246.88	241.13	208.25	313.75	228.5	281.63	190.38	311.13	301.13	241.13	249.38	266.13	281.5	289.25	441.5	453.63	294.5	300.75	324	267.75	264.63	272.13	305	379.25	321.38	389.13	294.5	316.5	340.13	300.25	298.25	326.25	248.13	273.63	386.13	435.88	259.75	259.75	292.5	232.13	246.88	231.63	252.75	328.38	242.38	334.75	208.38	309.75	317.13	245.13	244.63
432	106.13	127.88	138.63	116.5	125.5	129.5	103.13	170.63	99.5	152.13	115.13	171.38	171.88	127.75	110	123.88	159.13	160	261.5	236.75	165.63	164.38	181.25	144.25	185.88	160.88	176.25	218.63	167.38	246.63	180.13	201	191.88	163.5	168.63	178.63	110.38	122.63	186.25	208.88	119.25	115	126.38	106.25	124.63	101.75	114.25	169	101.38	161.63	105.13	144.13	153.75	117.38	96.125
433	47	58.5	67.375	50.625	59.625	59.375	46.375	82.25	40.375	76.625	61.375	81.125	85.25	61.125	48.625	53.875	74.125	77	138.38	118	81.5	84.25	92.25	73.75	103.75	76.5	87.875	111.75	79	123.88	93.375	106.63	98.375	78.75	86.875	94.625	43.625	55.5	82.5	93.25	50.25	50.25	53.5	42.375	62.375	42.25	47.375	78.25	40.625	71.75	48	59	68.375	54.875	34.875
434	21.25	27	31.75	20.25	29.375	26.25	20.125	36.125	16.375	38.625	30.75	36.375	38	29.125	20.375	22	28.125	34.375	72.25	55.875	34.375	41.75	44.25	34.75	59.5	37.375	39.125	53.375	33.25	58.875	42.375	45.625	43.75	31.125	38.375	47.75	14.25	22.375	33.25	39.125	21.75	22	21	14.375	33.25	14.375	17.75	33.125	15	28.375	20.875	18.625	27.375	24	13.875
435	8.25	10.125	13.375	7.125	12	9.625	9.375	13.5	6.625	17.25	15.375	13.125	12.875	12.625	8.625	7.875	10	11.875	33.25	20.875	11.75	15.75	16.5	13	24.125	16.75	14.75	19.625	10.25	24.375	15.25	14	15.375	11.625	15.375	19.5	3.25	8.125	10.25	10.875	7.125	7.75	4.625	3.5	17.375	4.625	4.75	11.75	3.875	9.125	8.5	4.5	7.25	8.125	4.875
436	3	3.625	6.5	2.75	3.875	3.375	2.5	4.375	2.125	7.125	8.375	3.875	3.75	4.75	2.25	2.375	1.375	2.25	9.5	3.75	2.125	4.375	3.875	3.5	7.75	4.75	4.375	5	2.125	8.75	4	3.875	3.5	4.75	4.25	6.875	0.75	2.625	3.125	2.625	2.5	2.125	1	0.5	9.125	0.875	0.875	2.375	0.625	1.5	3.875	1	1	3.125	1
437	0.1797	0.3644	0.312	0.26	0.2876	0.3037	0.1826	0.444	0.2339	0.3742	0.3581	0.499	0.4678	0.3394	0.2042	0.2204	0.804	0.578	1.4032	1.0616	0.4048	0.858	0.6532	0.4262	0.8078	0.4254	0.4678	0.875	0.6436	0.9068	0.7079	1.1393	0.9049	0.5706	0.4136	0.5232	0.2236	0.2715	0.6016	0.4544	0.1857	0.3462	0.262	0.1767	0.3301	0.1963	0.1836	0.4778	0.1837	0.3659	0.2513	0.4025	0.3558	0.2504	0.1285
438	0.0881	0.1791	0.1619	0.1148	0.1463	0.1404	0.0843	0.2264	0.0989	0.2011	0.1892	0.2466	0.2286	0.1664	0.0918	0.0983	0.3644	0.3161	0.9221	0.6222	0.1917	0.505	0.3551	0.2205	0.5025	0.2324	0.2197	0.5396	0.313	0.5247	0.3857	0.5622	0.4847	0.2652	0.199	0.3041	0.0849	0.1301	0.2907	0.2319	0.0928	0.1708	0.1199	0.0688	0.185	0.0749	0.0796	0.2363	0.0785	0.1668	0.1243	0.1494	0.1716	0.1189	0.0571
439	1.2564	1.7035	1.7967	1.9107	1.5452	1.5485	1.187	1.7761	0.6324	1.7655	1.2404	2.0409	1.9925	0.2757	1.627	0.9412	0.1935	0.9942	0.3566	0.5392	2.4069	0.5451	1.4838	1.2551	1.7387	1.2322	1.2693	0.4089	1.9673	0.5381	0.8897	1.5677	0.6787	0.538	2.23	1.1168	1.3381	1.4071	1.4911	1.6323	1.2486	1.358	0.6418	0.9784	1.4553	1.1479	1.1614	1.5592	1.5425	1.437	1.1169	2.0502	1.756	0.9096	1.1244
440	1.1339	1.6163	1.8026	1.9075	1.4919	1.5373	1.1569	1.7102	0.5942	1.6582	1.2741	1.984	1.8892	0.3286	1.6145	0.8656	0.2573	1.0671	0.4505	0.6532	2.3305	0.6237	1.5305	1.2934	1.7583	1.2506	1.1661	0.3166	1.9365	0.6871	0.9535	1.7076	0.8038	0.5332	2.1438	1.0456	1.1445	1.2097	1.4153	1.5025	1.2023	1.1217	0.6197	0.9537	1.284	1.2334	1.0763	1.4712	1.5743	1.3006	1.1098	2.0339	1.6324	0.8543	1.1716
441	1.0477	1.8496	1.9909	1.9338	1.7138	1.6587	1.3568	1.9822	0.7097	1.6652	1.4706	2.2854	1.9483	0.2985	1.6895	0.9228	0.2863	1.0486	0.5763	0.7202	2.3561	0.8758	1.5216	1.5787	1.6954	1.3729	1.4511	0.2989	2.0111	0.7438	1.0122	1.7325	0.8813	0.725	2.1605	0.9372	1.2115	1.375	1.6115	1.7506	1.3438	1.3104	0.7493	0.9346	1.3434	1.2673	1.3305	1.7498	1.5077	1.3642	1.4023	2.1298	1.7843	0.8294	1.1697
442	1.2848	2.3089	2.3113	2.1184	2.1264	2.0125	1.5777	2.5244	0.8599	1.9557	1.7792	2.7577	2.2977	0.3327	1.9374	1.2585	0.2982	1.2146	0.79	0.8152	2.7274	0.9491	1.8568	1.8726	1.947	1.7274	1.9042	0.2761	2.2558	0.7396	1.1548	1.8267	0.9836	0.9593	2.4315	0.9381	1.4025	1.7929	1.9184	2.3034	1.6321	1.5839	0.9406	1.125	1.6063	1.355	1.6567	2.162	1.5956	1.74	1.7123	2.4392	2.1386	0.9047	1.354
443	1.4347	2.6613	2.4413	2.3178	2.5251	2.2947	1.6854	2.9251	1.0265	2.2542	2.0125	3.1099	2.6256	0.3829	2.0804	1.5241	0.4642	1.5716	0.7124	0.8641	3.0451	1.0643	2.0271	2.0692	2.0784	1.9559	2.1892	0.472	2.5706	0.7245	1.2378	1.9038	1.1746	1.1355	2.7258	1.1254	1.6992	2.253	2.2082	2.6681	1.8042	1.7669	1.0395	1.3318	1.8979	1.4545	1.8572	2.4283	1.771	2.0913	2.0094	2.8077	2.3088	1.0599	1.521
444	1.6846	2.5242	2.1593	2.2059	1.9114	2.298	1.363	2.5978	0.9339	2.1985	1.6599	2.6888	2.6996	0.3524	2.0986	1.5545	0.6672	1.7979	0.8762	0.9796	3.4688	1.0584	2.0503	1.5294	2.1302	1.7957	1.8091	0.5918	2.7926	0.8099	1.4607	1.966	1.6537	0.6189	2.9621	1.3283	1.9406	2.5936	2.444	2.888	1.862	1.7863	1.1231	1.5616	2.2404	1.4977	1.8186	2.7068	1.9705	2.1732	2.146	2.9231	2.445	1.4368	1.6673
445	1.3229	1.7317	1.8237	2.3687	1.3811	1.6855	0.9893	1.8844	0.835	1.8356	1.3447	1.9523	2.1505	0.934	1.6516	1.4691	1.1222	1.7518	0.8046	0.928	2.6696	0.9964	1.6986	1.0827	1.9028	1.4385	0.6619	0.7594	2.3081	0.7311	1.2231	1.8051	1.8335	1.0855	2.5967	1.7246	1.033	1.354	1.9883	1.3685	1.0964	0.4868	0.7742	1.3861	1.8633	1.9191	0.9495	1.7082	2.2551	1.4875	0.9329	2.4942	1.4136	1.471	1.8677
446	1.2293	0.8641	1.2095	2.3294	0.7314	1.0702	1.6293	0.7234	0.4974	1.5822	2.2091	0.872	1.4636	1.4283	1.2192	2.1984	0.9747	1.1194	1.5531	1.6715	2.1828	1.1676	1.0562	1.2952	0.3635	0.9668	1.022	1.1404	2.2612	0.9853	1.3024	1.4075	1.7491	2.0971	2.1186	1.7056	0.2888	1.1444	1.6918	0.7153	0.4155	0.4313	1.2571	1.1535	1.6234	1.686	0.9306	1.7129	2.186	0.5656	0.4833	2.2133	1.3034	1.1655	1.7429
447	0.3643	0.6201	0.3681	0.4615	0.7498	0.5	0.4517	0.5492	0.241	0.6345	0.4116	0.4616	0.3453	0.2173	0.2011	0.4625	0.2928	0.5252	0.2475	0.2789	0.4626	0.2249	0.273	0.3879	0.2787	0.6538	0.7279	0.1144	0.5487	0.2757	0.331	0.1977	0.2689	0.616	0.4257	0.3845	0.5348	0.5941	0.5427	0.8861	0.4631	0.529	0.5085	0.3986	0.6904	0.4281	0.4909	0.5987	0.5778	0.705	0.6481	0.7025	0.5659	0.5821	0.3401
448	1.297	1.4926	1.3688	1.6781	1.5621	1.5558	1.5907	1.4647	0.6457	1.5129	1.1241	1.3649	1.2066	0.3328	1.1157	1.171	0.3597	0.6987	0.3304	0.4025	1.4882	0.2398	0.4771	0.9708	0.6491	1.337	1.4401	0.0999	1.0853	0.1858	0.1959	0.4206	0.2426	1.0078	1.1576	0.6409	1.5076	1.5168	1.177	1.949	1.5352	1.3155	1.0686	1.2434	1.5838	1.3618	1.6533	1.3363	1.7137	1.6033	1.4603	1.7204	1.6799	1.4194	1.0724
449	2.0887	1.9267	2.1001	2.3099	1.8247	2.0958	2.2589	1.9253	0.9253	2.045	1.5309	1.7782	1.6178	0.488	1.903	1.6348	0.3997	1.1148	0.4904	0.5615	2.2001	0.3796	0.815	1.0863	1.098	1.7619	1.7427	0.1766	1.4427	0.1258	0.158	0.7592	0.3486	1.1179	1.7419	1.0387	2.0166	1.9569	1.4606	2.3572	2.4464	1.596	1.188	1.9933	2.0412	2.0717	2.3208	1.6924	2.9315	2.0026	1.9751	2.3796	2.1648	2.038	1.7107
450	2.5952	2.0765	2.4528	2.6789	1.924	2.1058	2.7204	2.111	1.0574	2.3099	1.8938	1.967	1.7154	0.8694	2.5419	1.8428	0.4904	1.8893	0.6617	0.6924	2.4432	0.6783	1.1305	1.2491	1.5925	2.0222	2.006	0.2551	1.844	0.2242	0.459	1.1368	0.6577	1.3943	2.2203	1.1727	2.3011	2.3294	1.4955	2.4355	2.7419	1.8543	1.1144	2.2607	2.3035	2.4816	2.7757	1.924	3.3754	2.075	2.4048	2.773	2.3524	2.3506	2.3053
451	3.3298	2.0329	2.6151	2.8369	1.8869	2.0225	2.9794	2.2397	1.2003	2.4951	2.5163	2.0668	1.6987	1.1605	2.9704	2.1602	0.4484	2.3163	0.8872	0.8681	2.3814	1.0145	1.3889	1.4145	2.0515	2.302	2.3121	0.4688	2.1335	0.143	0.6704	1.4394	1.171	1.9324	2.381	1.5458	2.5266	2.6829	1.4776	2.5806	3.1688	2.0443	0.903	2.7298	2.4937	2.6877	3.2071	2.3722	3.687	2.1138	3.0017	2.9603	2.5847	2.531	2.8851
452	3.867	1.7819	2.8555	3.3325	1.8122	1.8964	3.3555	2.5956	1.2391	2.813	3.174	2.1462	1.9412	1.2921	3.8278	2.9876	0.4359	2.7906	0.9304	1.1115	2.1666	1.4391	1.7271	1.7985	2.1663	2.1466	2.4691	0.829	2.6344	0.1942	0.9233	1.7609	1.9254	2.5399	2.6524	2.3435	3.784	3.2059	1.6249	2.9815	3.6921	2.0794	1.4135	3.9352	2.8845	3.2265	3.9134	2.9639	4.0898	2.5527	3.6166	3.3576	2.9391	2.7549	3.9282
453	4.6524	1.3156	3.4088	4.0688	1.3786	1.4111	3.5487	2.9823	2.0759	3.1527	3.927	1.7179	1.2593	1.2587	4.5571	4.0365	1.126	3.69	1.0304	1.2161	2.0749	1.8818	2.219	2.2617	1.8281	1.9078	2.5457	1.6316	3.288	0.478	1.1788	2.5303	3.3213	3.228	2.5774	2.7933	5.5486	3.2959	2.2821	2.8699	4.1554	2.0864	3.6088	5.3533	3.2846	4.7863	4.9938	3.4851	4.7431	2.7843	4.1988	4.6132	3.4751	3.4187	4.8614
454	4.4647	1.5614	2.9909	4.372	1.068	1.829	4.4638	3.3658	4.4527	3.0731	4.1262	2.1552	0.5634	1.7127	4.8214	4.4697	2.3558	3.7535	0.8571	1.892	3.797	1.8444	2.7516	2.9168	1.6098	1.8373	2.3589	2.9189	4.159	0.7122	1.7868	3.176	3.8744	3.0422	2.6213	3.245	5.4561	3.2668	2.9359	2.6777	3.6098	2.8718	4.785	5.3291	3.574	5.2513	4.2029	4.6283	5.1831	2.3033	4.3629	4.9099	4.0901	3.4645	5.4833
455	1.3809	1.2846	1.3623	1.3672	1.3426	1.4326	1.3759	1.3225	1.389	1.3672	1.3765	1.3148	1.3072	1.3839	1.4171	1.3879	1.2697	1.3085	1.2965	1.3098	1.3667	1.2503	1.3312	1.3597	1.3493	1.3301	1.3132	1.28	1.352	1.2812	1.3266	1.2829	1.3157	1.3177	1.3923	1.3916	1.309	1.3111	1.3296	1.29	1.4109	1.2807	1.3606	1.3969	1.3687	1.3684	1.3727	1.3283	1.3719	1.3195	1.3641	1.3127	1.3325	1.3307	1.4239
456	487.38	365.86	452.46	441.52	477.62	415.23	444.89	430.62	378.51	430.99	479.94	389.31	418.44	403.4	511.1	491.1	340.34	356.62	369.35	404.17	434.8	321.62	409.22	391.44	414.28	406	432.42	384.97	342.91	423.23	462.09	343.16	362.27	387.9	473.67	439.05	329.54	358.4	339.47	407.07	415.34	292.72	394.23	378.97	403.76	393.79	403.85	362.78	341.77	403.1	426.65	325.47	354.68	375.18	452.37
457	673	470	616.38	603.63	641.25	594.88	612.13	569.5	525.75	589.25	660.63	511.88	547	558.25	724.25	681.63	432.13	466.63	478.88	529.38	594.25	402.13	544.75	532.25	559	540	567.88	492.75	463.63	542.25	613	440.25	476.63	511.13	659.5	611	431.38	469.88	451.38	525.13	586	374.88	536.38	529.38	552.63	538.88	554.38	481.88	468.88	531.88	582	427.25	472.63	499.25	644.13
458	1263.5	1042.8	1224.5	1088	1308.1	1088.5	1122	1234.3	993.38	1212.9	1291	1123.8	1148.5	1022.4	1365.3	1333.6	1065.3	1107	1247	1319.3	1298	1076.4	1258.9	1095.5	1274.1	1174.6	1241.8	1276.8	1071.5	1354.3	1446.1	1106.8	1125.9	1124.8	1415.5	1306.9	878.38	962	1015.6	1179.8	1073.8	854.5	1102.8	931.13	1097	942.88	1009.3	1025.4	892.88	1145.6	1145.6	942	969.63	934.5	1224.4
459	712.63	680.13	774.88	631.63	860	667.38	624.38	828.25	617.13	788.88	822.88	753.88	746.38	628.88	825.5	820.38	778.13	798.38	964.88	1012.1	861.38	807.63	898.88	728.63	931.63	780	832.13	957.38	773.88	1018.9	1068.1	826.63	836.38	780.13	964.13	927.75	512.13	594.38	710.75	784.75	611.38	540.75	685.25	508	697.13	515.13	556.5	686.63	497	747.88	713.75	614.75	596.5	519	701.88
460	360.5	391.75	416.5	354.75	476.63	374.75	319.13	485.5	339.38	457.25	461.63	451.5	448	369.25	436.5	424	477	469.13	631.75	645.88	495.5	492.13	547.25	429.5	565.13	471.13	507.63	610.38	471.63	661.63	657.88	518.38	520.75	474.38	558.38	533.75	269.5	318	431.38	476.38	299.88	303.25	376.5	253.25	382.63	276.88	285.75	410.13	249.25	431.38	386	351.38	346	283	353.88
461	145.38	165.25	180.25	154.13	186.75	165.25	129	220.75	129.75	198.63	203.38	208.5	211.13	163.5	170.63	170.13	219.63	208.88	315.88	298.75	220.88	217.88	249.38	186.75	271.13	212.5	231.38	281	210.88	319	299.63	259.25	244	213	246.38	235.5	111.25	129.5	198.63	215.5	127.88	126.63	149.25	106.25	162.13	115.88	117.88	189.13	101.38	183.88	154.38	157	157.88	124.63	123.38
462	60.5	69.5	82.75	66.5	75.5	68.625	55.375	100.13	50.25	91.125	96.875	92.5	97.125	72.375	71.875	70.625	94.875	92.625	155.75	135.75	98.875	102	114.88	85.5	129.25	89.875	103.63	132.13	91.875	146	139.38	124.75	114.13	96.5	111.63	111.38	43.625	57.125	87	94.75	52.125	54	61.5	42.375	72.5	47.75	47.875	83.25	40.625	79.125	65.25	63.75	68.75	57	43.5
463	24.875	28.25	35.875	25.875	31.375	27.5	22	41.125	17.5	40.75	43	38	40	30.875	27.375	25.375	33.25	36.75	75.5	58.875	37.625	44.25	48.875	36.375	63.25	39.125	40.625	57.875	35.25	62.5	58	48	47.375	36	42.875	49.875	14.25	22.625	34.375	39.125	21.75	22.375	23.125	14.375	34.125	16	17.75	33.875	15	29.5	26.375	19.625	27.375	24.25	16
464	8.375	10.125	14.25	8.125	12	9.625	9.375	14.75	6.625	17.25	19.125	13.125	12.875	12.625	10	7.875	10	11.875	33.25	21	11.75	15.75	16.75	13	24.125	16.75	14.75	20.25	10.25	24.375	19.5	14	15.375	12.75	15.375	19.5	3.25	8.125	10.25	10.875	7.125	7.75	4.625	3.5	17.375	4.625	4.75	11.75	3.875	9.125	9.875	4.5	7.25	8.125	4.875
465	3	3.625	6.5	2.75	3.875	3.375	2.5	4.375	2.125	7.125	8.375	3.875	3.75	4.75	2.25	2.375	1.375	2.25	9.5	3.75	2.125	4.375	3.875	3.5	7.75	4.75	4.375	5	2.125	8.75	4	3.875	3.5	4.75	4.25	6.875	0.75	2.625	3.125	2.625	2.5	2.125	1	0.5	9.125	0.875	0.875	2.375	0.625	1.5	3.875	1	1	3.125	1
466	1078.3	779.75	953.88	815.5	1061.3	855.88	885	947	797.38	956	1087.3	823.13	836.75	794.13	1097.3	1044.8	818.25	885.88	888.88	939.38	1071.1	817.88	1000.5	826	1056.6	923.75	975.25	970.7	891.38	1047	1197.3	834.63	814	827.5	1172.5	1001.4	565.88	665.63	674.88	783.63	793.63	562.5	823.13	645.38	838.63	687.88	714.88	755.88	616.25	822.88	949.88	646.75	661	663.25	957.75
467	425.13	346.38	418.25	308.25	561.63	365	345.63	456.88	326.88	460.63	595.88	358.75	357.25	343.63	498.5	458.75	436.88	466.75	453.13	444.38	508.75	447.5	549.63	389.75	658.38	481.63	499	547.5	383.25	625.38	712.38	437.13	475.13	393.75	708.63	617.13	319.38	338.75	375.38	508.13	502.63	272.13	433.25	338.5	485.88	393.75	388.88	408.88	312.13	454	[illegible]	182.25	173.38	169.75	372.13
468	145.63	137.13	143.38	109.5	229.75	133.63	110.88	171.75	110.88	178.38	229.5	138.13	127.38	140.38	157.88	195.5	179.88	157.63	185.5	182.5	197.25	168	212.63	130.38	278.75	177.63	202.38	225.25	133.88	216	301.13	147.5	184.75	139.88	246.5	209.5	114.13	104.88	138	209.63	119.5	73.125	166.5	118.5	235.25	146.63	135.13	140.13	84.625	193	[illegible]	96.625	177.63	68.375	136.13
469	39.25	37.375	41.625	37.625	61.25	35.75	25.875	50.125	30.25	46.5	88.25	37.125	39.25	35.75	60.625	46.25	60.5	48.875	54.375	62	55.25	53.5	61.25	38.25	68.125	42.5	85.25	50.5	51.625	73.5	83.25	45.375	58.5	52.125	49.5	77.75	56.875	0.875	6.875	12.375	6.25	6.625	8.625	11.625	22.875	[illegible]	[illegible]	[illegible]	[illegible]	[illegible]	[illegible]	[illegible]	[illegible]	[illegible]	[illegible]
470	1.75	11	15.375	15.875	15.875	9.25	9	17.875	9.875	14.5	35	11.375	17.875	14.25	2.875	20.75	[illegible]	[illegible]	[illegible]	[illegible]	[illegible]	[illegible]	[illegible]	[illegible]	[illegible]	[illegible]	[illegible]	[illegible]	[illegible]	[illegible]	[illegible]	[illegible]	[illegible]	[illegible]	[illegible]	[illegible]	[illegible]	[illegible]	[illegible]	[illegible]	[illegible]	[illegible]	[illegible]	[illegible]	[illegible]	[illegible]	[illegible]	[illegible]	[illegible]	[illegible]	[illegible]	[illegible]	[illegible]	[illegible]	[illegible]
471	3.625	1.25	4.125	5.625	5	1.25	1.875	5	1.125	2.125	12	1.25	1.625	2	1.75	7	[illegible]	[illegible]	[illegible]	[illegible]	[illegible]	[illegible]	[illegible]	[illegible]	[illegible]	[illegible]	[illegible]	[illegible]	[illegible]	[illegible]	[illegible]	[illegible]	[illegible]	[illegible]	[illegible]	[illegible]	[illegible]	[illegible]	[illegible]	[illegible]	[illegible]	[illegible]	[illegible]	[illegible]	[illegible]	[illegible]	[illegible]	[illegible]	[illegible]	[illegible]	[illegible]	[illegible]	[illegible]	[illegible]	[illegible]
472	0.125	0	0.875	[illegible]	[illegible]	[illegible]	[illegible]	[illegible]	[illegible]	[illegible]	[illegible]	[illegible]	[illegible]	[illegible]	[illegible]	[illegible]	[illegible]	[illegible]	[illegible]	[illegible]	[illegible]	[illegible]	[illegible]	[illegible]	[illegible]	[illegible]	[illegible]	[illegible]	[illegible]	[illegible]	[illegible]	[illegible]	[illegible]	[illegible]	[illegible]	[illegible]	[illegible]	[illegible]	[illegible]	[illegible]	[illegible]	[illegible]	[illegible]	[illegible]	[illegible]	[illegible]	[illegible]	[illegible]	[illegible]	[illegible]	[illegible]	[illegible]	[illegible]	[illegible]	[illegible]
473	0	0	0	[illegible]	[illegible]	[illegible]	[illegible]	[illegible]	[illegible]	[illegible]	[illegible]	[illegible]	[illegible]	[illegible]	[illegible]	[illegible]	[illegible]	[illegible]	[illegible]	[illegible]	[illegible]	[illegible]	[illegible]	[illegible]	[illegible]	[illegible]	[illegible]	[illegible]	[illegible]	[illegible]	[illegible]	[illegible]	[illegible]	[illegible]	[illegible]	[illegible]	[illegible]	[illegible]	[illegible]	[illegible]	[illegible]	[illegible]	[illegible]	[illegible]	[illegible]	[illegible]	[illegible]	[illegible]	[illegible]	[illegible]	[illegible]	[illegible]	[illegible]	[illegible]	[illegible]

Table S12 Amino acid descriptors (2D) used in this study.

No.	PY	QA	QC	QD	QE	QF	QG	QH	QI	QK	QL	QM	QN	QP	QQ	QR	QS	QT	QV	QW	QY	RA	RC	RD	RE	RF
	P	Q	Q	Q	Q	Q	Q	Q	Q	Q	Q	Q	Q	Q	Q	Q	Q	Q	Q	Q	Q	R	R	R	R	R
	Y	A	C	D	E	F	G	H	I	K	L	M	N	P	Q	R	S	T	V	W	Y	A	C	D	E	F
1	-3	1	0	-4	-3	-3	-4	-1	-4	-1	0	-7	-7	-2	-3	1	2	-4	-2	-6	-3	-3	-2	1	-1	-6
2	-3.8	1.4	0.7	-4	-0.4	-1.5	-4.9	-0.1	-3.4	-0.2	-0.6	-3.7	-4.3	-2.4	-1.7	1.7	2.1	-0.1	-1.2	-4.9	-1.7	-1.7	-1.9	0.8	-0.1	-3.7
3	-2.7	1.3	0.4	-3.5	-0.1	-1.1	-4.3	0.4	-3.5	0.2	-0.2	-3.7	-5.5	-2	-1.2	1.4	1.7	-0.7	-1.4	-4.8	-1.1	-1.2	-1	0.8	0.4	-2.5
4	-2	1.1	0.1	-2.6	0.3	-0.6	-3.5	0.3	-3.1	0.4	-0.3	-3.2	-4.1	-1.7	-0.8	0.7	1.7	-1	-1.8	-4.3	-0.5	-0.8	-0.4	0.6	0.3	-1.2
5	-1.9	0.1	1	-2.4	-2	-1.9	-1.5	-1.5	-2.2	0.3	1	-1.6	-2.2	0.1	-1.6	0.9	2	0.8	-0.5	-1.8	-1.8	-1.6	-1.9	1.2	-1.5	-2.7
6	-0.43	0.14	0.06	-0.36	0.34	0.14	-0.31	0.12	-0.39	0.18	-0.08	-0.24	-0.25	-0.3	-0.07	0.11	0.51	-0.11	-0.27	-0.24	0.08	-0.07	-0.07	0.11	0.12	0.1
7	-2	1	0	-3	-1	-2	-4	0	-3	0	0	-5	-6	-2	-2	1	2	-3	-1	-5	-1	-2	-1	1	0	-5
8	1	5	5	2	2	2	1	2	1	3	4	1	2	2	2	5	4	3	2	2	2	2	3	5	2	2
9	3	1	1	2	2	2	2	2	2	2	1	2	2	1	2	1	1	1	1	2	2	2	2	1	2	3
10	7	9	8	7	9	9	1	6	7	9	5	5	5	7	5	10	6	5	7	6	10	5	5	9	6	10
11	-1.9	1.1	0.1	-2.2	0.4	-0.5	-3.2	0.3	-3	0.5	-0.3	-3.2	-3.6	-1.6	-0.6	0.6	1.7	-1	-1.8	-4	-0.3	-0.6	-0.4	0.5	0.3	-0.9
12	109	99	64	112	54	149	97	86	153	101	126	154	148	113	112	58	29	125	142	147	47	112	106	60	86	196
13	-2	1	0	-2	0	-1	-2	0	-3	0	-2	-3	-2	-2	-2	0	2	-2	-2	-3	-2	-2	-1	0	0	-2
14	-4	2	0	-3	0	-1	-4	-1	-5	-1	-3	-5	-4	-3	-2	0	3	-3	-4	-5	-3	-2	-1	0	-1	-2
15	-5	2	-1	-4	-1	-2	-5	-1	-6	-2	-3	-7	-5	-4	-3	0	3	-4	-4	-6	-3	-3	-1	0	-1	-3
16	-7	0	-0.5	-4	-0.2	-6	-6	-1.5	-4.8	-1.5	-1.6	-9.7	-5.8	-4.4	-1.6	-0.8	2.4	-2.8	-4.7	-5.4	-3	-1.6	-0.9	-0.5	-1.5	-4.4
17	-3	1	1	-3	0	-1	-4	0	-3	0	0	-3	-4	-2	-1	2	2	0	-1	-4	-1	-1	-1	1	0	-2
18	-4	2	0	-3	2	0	-4	2	-4	3	0	-3	-4	-2	-1	1	7	0	-1	-5	-2	-1	-2	1	2	-1
19	0	0	0	0	6.6	6.6	9	0	0	5	0	0	0	9	6.6	0	6.6	0	0	0	5	6.6	6.6	0	0	0
20	-1	1	0	-1	0	0	-1	0	-1	0	0	0	-1	-1	0	0	1	0	-1	-1	0	0	0	0	0	0
21	-3	4	3	-6	2	-7	-14	2	-4	-2	1	-13	-6	-2	0	3	5	-7	-5	-16	-5	0	-1	-5	2	-3
22	-4	9	0	-4	8	1	-11	5	-10	13	6	-14	-20	-12	5	8	9	-2	-11	-16	-5	5	6	5	5	-17
23	-4	1	-5	-21	-1	4	-18	2	-12	-4	-2	-1	-20	-13	-2	1	3	2	-4	-14	1	-2	-10	1	2	4
24	0	9	-1	-15	12	8	-14	5	-6	9	-1	-13	-50	-7	-2	6	7	0	1	-17	-16	-2	1	5	5	0
25	-7	5	-2	-10	-1	-7	-9	0	-11	-2	-4	-26	-2	-13	-3	3	6	6	-10	-14	-19	-3	-6	9	0	-17
26	-14	12	-7	0	-1	-11	-10	10	-15	30	4	0	-23	-9	1	6	15	-8	-12	-9	5	1	4	14	10	-23
27	-4	4	2	-5	3	-6	-15	3	-7	1	2	-15	-9	-6	1	3	6	-6	-8	-16	-5	1	1	-4	3	-7
28	-6	3	-6	-19	4	6	-22	5	-14	2	0	-4	-43	-14	-1	2	7	3	-5	-14	0	-1	-6	2	5	3
29	-13	7	-5	-5	2	-18	-11	4	-13	8	-1	-16	-17	-12	-2	4	11	2	-13	-13	-4	-2	-3	12	4	-28
30	0	4	3	1	3	2	1	3	1	3	3	1	1	3	2	3	5	3	2	0	4	2	3	3	3	3
31	0	1	0	0	0	0	0	0	0	0	0	0	0	0	0	1	3	0	0	0	0	0	0	3	0	0
32	-0.95	0.16	-0.06	-0.34	-0.02	-0.62	-0.79	0.01	-0.9	0.16	0.04	-0.41	-0.51	-0.74	-0.08	0.18	0.25	0.2	-0.68	-0.35	-0.13	-0.08	-0.2	0.19	0.01	-0.29
33	2.57	0.51	1.85	3.18	1.45	1.45	2.47	2.04	3.49	2.05	2.37	3.48	4.23	2.7	2.92	0.9	0.84	3.58	2.95	4.08	1.32	2.92	2.96	0.91	2.04	1.46
34	6	10	9	9	9	10	5	10	7	11	9	8	11	9	8	10	11	7	8	10	10	8	10	8	10	10
35	-0.13	-0.38	-0.23	-0.59	0.09	0.12	0.15	-0.1	-0.23	0.13	0.05	-0.1	-0.11	0.29	0.09	-0.59	0.39	-0.18	-0.34	0.19	0.1	0.09	0.55	-0.4	-0.1	-0.25
36	-0.15	-0.4	-0.25	-0.63	0.11	0.15	0.2	-0.12	-0.24	0.14	0.07	-0.1	-0.11	0.35	0.11	-0.63	0.42	-0.21	-0.37	0.2	0.09	0.11	0.6	-0.41	-0.12	-0.29
37	-5	0	0	-3	0	-5	-4	-1	-3	-1	-1	-7	-3	-3	-1	-1	2	-2	-3	-3	-2	-1	-1	0	-1	-5
38	0.372	0.193	0.565	0.683	0.375	0.087	0.552	0.266	0.191	0.184	0.299	0.035	0.081	0.251	0.39	-0.083	0.228	0.394	0.166	0.372	0.259	0.39	0.398	0.407	0.266	-0.006
39	0.511	0.281	0.85	0.772	0.037	0.326	0.108	0.558	-0.172	-0.064	-0.018	0.015	0.825	0.439	0.25	0.574	0.254	0	0.083	0	0.399	0.25	0.281	0	0.558	0.912
40	2	2	5	4	0	1	0	2	0	0	0	0	4	3	2	3	2	2	0	0	2	2	2	2	2	5
41	1.4	2	2	0.2	1.9	-1.4	0.4	1.2	0.8	0.1	0.2	-1.7	-0.9	1.1	1.5	1.9	2.1	0.1	1.6	-0.2	-0.9	1.5	1.4	0.6	1.2	-1.6
42	-20	10	-12	-18	11	-4	-11	11	-35	10	3	-20	-12	-26	-2	-3	37	-6	-17	-18	-7	-2	2	-3	11	2
43	-12	1	-5	-2	13	-5	-8	10	-9	16	0	-8	-2	-9	1	-2	17	0	-8	-2	4	1	1	5	10	0
44	-12	0	-4	-1	10	-3	-8	7	-9	11	0	-8	-1	-9	1	-2	13	0	-7	-1	3	1	1	1	7	1
45	55	94	94	94	74	95	0	53	52	98	33	30	98	57	13	98	74	17	84	98	74	13	35	96	53	91
46	0	0	1	0	-1	0	-2	0	-1	-1	-2	-2	0	0	-1	0	0	-1	-1	-2	-1	-1	-1	-1	0	1
47	3	-1	1	2	-1	1	1	0	2	0	0	1	3	2	0	0	-2	1	1	1	1	0	0	-1	0	2
48	1.02	1.17	1.09	0.86	1.15	1.18	0.74	1.03	0.98	1.18	1.31	1.44	0.9	1.01	1.17	1.06	1.26	0.99	0.89	0.58	0.95	1.17	1.29	1.11	1.03	1.36
49	1.1	0.8	1.05	1.23	0.82	0.93	1.26	0.88	0.97	0.64	0.56	0.67	1.18	1.07	0.8	0.97	0.73	0.84	1.24	1.27	0.91	0.8	0.65	0.83	0.88	0.97
50	0.7	1.04	0.65	0.84	1.02	0.68	1.09	1.19	1.13	1.38	1.23	0.6	0.86	0.8	1.02	0.92	0.96	1.41	0.74	1.51	1.33	1.02	1.06	1.11	1.19	0.1
51	15.8	20.4	16.7	15.3	18.7	16.2	10.1	17.1	11.7	18.2	18.2	14	10.1	13.3	16.8	18.1	20.4	16.1	14.6	13.7	14.8	16.8	17.1	19.9	17.1	19.8
52	3.3	6.3	5.3	3	5.6	4.7	2	5.5	2.2	5.7	4.9	2	1.6	3.6	4.6	5.8	6.9	4.2	3.4	1.2	4.9	4.6	4.8	5.7	5.5	4.3
53	-7	0	0	-3	3	-19	-6	1	-7	0	0	-13	-11	-10	0	-3	5	-2	-6	-10	0	0	0	1	1	-9
54	-1.47	0.34	0.02	-1.25	0.99	-0.59	-1.4	0.6	-2.1	0.66	-0.73	-2.51	-0.66	-1.49	-0.5	0.13	1.23	-0.96	-1.75	-1.62	-0.52	-0.5	-0.38	0.27	0.6	1.04
55	-3.26	1.27	0.16	-4.22	2.83	-1.89	-4.36	0.24	-5.64	1.53	-2.38	-6.98	-2.61	-4.56	-2.24	0.6	3.16	-3.39	-4.67	-4.34	-1.14	-2.24	-1.22	0.63	0.24	3.5
56	-0.9	1.7	1.9	-1.8	1.9	0.3	-3.2	0.7	-2.4	1.4	1	-3.1	-3.3	-1.2	0.2	1.7	1.7	-0.2	-1.1	-4.8	-0.2	0.2	-0.2	1.4	0.7	-1.3
57	-7	0	-2	0	8	-2	-5	4	-7	7	0	-4	0	-5	0	0	8	0	-5	-1	0	0	0	0	4	2
58	1	0	-1	1	2	2	5	2	0	0	0	0	-4	-2	1	1	6	2	-1	1	5	1	1	1	2	3
59	-3	2	1	0	2	-2	-3	0	-2	1	-1	-1	-1	-5	0	0	2	0	-4	-1	1	0	1	1	0	1
60	0	0	0	0	2	0	-1	0	0	2	-1	0	0	-2	1	0	4	1	-3	0	3	1	1	0	0	1
61	-7.9	-5.6	-5.9	-7.5	-7.5	-7.5	-8.1	-7.3	-7.7	-6.9	-6.9	-8.3	-9	-7.4	-7	-6.2	-6.8	-7.5	-7.2	-7.5	-8	-7	-6.7	-5.8	-7.3	-8.7
62	-30	12	6	-19	3	-7	-30	4	-32	-4	-20	-30	-29	-23	-9	0	19	-18	-20	-29	-20	-9	-10	4	4	-8
63	-3	2	1	-3	-6	-1	-7	-3	-3	-5	-5	-7	-4	-3	-6	0	1	-5	-2	-3	-4	-6	-7	1	-3	-2
64	-3.5	2	1.5	-3.5	-9	1	-5.5	-4.5	-3.5	-5	-6.5	-7.5	-2.5	-3.5	-5.5	1.5	-2.5	-5.5	-1.5	-3.5	-3.5	-5.5	-5.5	0.5	-4.5	1
65	-4	1	0	-3	-1	0	-5	0	-4	0	-1	-5	-5	-2	-2	1	2	-3	-3	-5	-1	-2	-1	0	0	-1
66	-2	1	0	-2	0	0	-3	0	-3	0	0	-3	-3	-2	-1	1	2	-1	-2	-4	0	-1	-1	1	0	0
67	-3	0	-1	-2	0	-1	-3	-1	-4	0	-2	-4	-3	-3	-2	-1	1	-3	-4	-4	-1	-2	-2	-1	-1	-1
68	0.021	0.056	0.157	0.044	0.026	0.06	0.003	0	0.002	0.003	0.045	0.015	0.001	0.039	0.03	0.036	0.033	0.02	0.009	0.047	0.001	0.03	0.102	0.014	0	0.007
69	-9.48	-4.66	6.53	-2.36	-0.16	-2.72	2.17	0.83	-7.43	-13.72	-6.26	-1.9	-7.17	-5.3	-5.1	-1.81	-1.47	-8.02	-10.89	-4.71	-2.34	-5.1	3.96	-2.33	0.83	-11.04
70	-1.1	3	9.8	-0.2	-0.4	2	2.2	3.8	-2.4	-1.6	-1	1.9	-1.9	0.7	-0.2	0.7	1	0.5	-0.3	4.1	-1	-0.2	2.7	1.8	3.8	-2.4
71	-5.08	-1.02	4.84	-0.08	-0.49	-0.26	-1.22	0.01	-3.75	-4.98	-1.66	-2.14	-3.47	-2.11	-2.01	-1.28	1.1	-3.98	-4.95	0.77	-2.34	-2.01	3.93	-0.2	0.01	-1.98
72	-0.5	5.7	-0.3	-0.4	2.1	-1.8	0	-0.4	0	0	-5.7	-0.2	-0.5	1	-2.4	2.2	0.9	0.2	0.5	-0.3	0.3	-2.4	-2.7	0.1	-0.4	-0.2
73	-4	-1	2	0	-1	0	-2	-1	-4	-4	-1	-3	-4	-2	-2	-1	0	-4	-4	2	-3	-2	2	0	-1	-2
74	-7	6	7	10	-3	-12	-9	2	1	-6	7	-8	13	-7	8	7	-4	-8	-7	-6	-14	8	6	6	2	-3
75	-13.3	10.9	24.9	-3.9	7.4	-5.5	-6.2	-7.2	-4.4	-7.7	-6	-12.3	-7.7	-5.7	-5.3	16.2	6.8	-1.5	16.2	-13.9	10.2	-5.3	-8.8	-7.1	-7.2	0.4
76	0.4	18.1	6.2	0.5	1	0.1	0.1	0.5	0.1	1.3	3.9	1.6	0.3	1.1	0.9	7.5	4.9	1.2	0.6	0.1	0.8	0.9	4.1	2	0.5	0.5
77	0.4	18.1	6.2	0.5	1	0.1	0.1	0.5	0.1	1.3	3.9	1.6	0.3	1.1	0.9	7.5	4.9	1.2	0.6	0.1	0.8	0.9	4.1	2	0.5	0.5
78	0.3	8.4	0.3	4.5	4.9	0.8	0.4	4.4	0.4	3.7	1.1	2	2	0.6	47.5	5.7	0.4	0.4	0.1	1.8	0.1	0.9	0.1	1.4	1	0.1
79	0.3	8.4	0.3	4.5	4.9	0.8	0.4	4.4	0.4	3.7	1.1	2	2	0.6	47.5	5.7	0.4	0.4	0.1	1.8	0.1	0.9	0.1	1.4	1	0.1
80	0.3	8.4	0.3	4.5	4.9	0.8	0.4	4.4	0.4	3.7	1.1	2	2	0.6	47.5	5.7	0.4	0.4	0.1	1.8	0.1	0.9	0.1	1.4	1	0.1
81	0.3	8.4	0.3	4.5	4.9	0.8	0.4	4.4	0.4	3.7	1.1	2	2	0.6	47.5	5.7	0.4	0.4	0.1	1.8	0.1	0.9	0.1	1.4	1	0.1
82	0.3	8.4	0.3	4.5	4.9	0.8	0.4	4.4	0.4	3.7	1.1	2	2	0.6	47.5	5.7	0.4	0.4	0.1	1.8	0.1	0.9	0.1	1.4	1	0.1
83	0.3	8.4	0.3	4.5	4.9	0.8	0.4	4.4	0.4	3.7	1.1	2	2	0.6	47.5	5.7	0.4	0.4	0.1	1.8	0.1	0.9	0.1	1.4	1	0.1
84	0.3	8.4	0.3	4.5	4.9	0.8	0.4	4.4	0.4	3.7	1.1	2	2	0.6	47.5	5.7	0.4	0.4	0.1	1.8	0.1	0.9	0.1	1.4	1	0.1
85	0.3	8.4	0.3	4.5	4.9	0.8	0.4	4.4	0.4	3.7	1.1	2	2	0.6	47.5	5.7	0.4	0.4	0.1	1.8	0.1	0.9	0.1	1.4	1	0.1
86	0.3	8.4	0.3	4.5	4.9	0.8	0.4	4.4	0.4	3.7	1.1	2	2	0.6	47.5	5.7	0.4	0.4	0.1	1.8	0.1	0.9	0.1	1.4	1	0.1
87	0.3	8.4	0.3	4.5	4.9	0.8	0.4	4.4	0.4	3.7	1.1	2	2	0.6	47.5	5.7	0.4	0.4	0.1	1.8	0.1	0.9	0.1	1.4	1	0.1
88	0.3	8.4	0.3	4.5	4.9	0.8	0.4	4.4	0.4	3.7	1.1	2	2	0.6	47.5	5.7	0.4	0.4	0.1	1.8	0.1	0.9	0.1	1.4	1	0.1
89	0.3	8.4	0.3	4.5	4.9	0.8	0.4	4.4	0.4	3.7	1.1	2	2	0.6	47.5	5.7	0.4	0.4	0.1	1.8	0.1	0.9	0.1	1.4	1	0.1
90	0.3	8.4	0.3	4.5	4.9	0.8	0.4	4.4	0.4	3.7	1.1	2	2	0.6	47.5	5.7	0.4	0.4	0.1	1.8	0.1	0.9	0.1	1.4	1	0.1
91	0.004	0.183	0.029	0.03	0.045	0.041	0.009	0.037	0.018	0.038	0	0.057	0.023	0.011	0.008	0.019	0.026	0.025	0.006	0.031	0	0.348	0.004	0.048	0.017	0.053
92	0.004	0.183	0.029	0.03	0.045	0.041	0.009	0.037	0.018	0.038	0	0.057	0.023	0.011	0.008	0.019	0.026	0.025	0.006	0.031	0	0.348	0.004	0.048	0.017	0.053
93	0.004	0.183	0.029	0.03	0.045	0.041	0.009	0.037	0.018	0.038	0	0.057	0.023	0.011	0.008	0.019	0.026	0.025	0.006	0.031	0	0.348	0.004	0.048	0.017	0.053
94	0.004	0.183	0.029	0.03	0.045	0.041	0.009	0.037	0.018	0.038	0	0.057	0.023	0.011	0.008	0.019	0.026	0.025	0.006	0.031	0	0.348	0.004	0.048	0.017	0.053
101	-5.2	-3.5	-5	-3.2	-3.6	-5.3	-4.4	-4.7	-5.9	-3.7	-5	-3.5	-3.8	-4	-3.4	-4.5	-3.6	-3.7	-4.7	-5.8	-5.2	-3.4	-5.3	-3.9	-4.2	-5.8
102	42	34	13	17	19	21	75	17	29	32	53	1	30	23	7	29	44	28	46	22	32	20	13	35	34	32
103	-2.101	0.017	0.296	2.021	1.926	-0.609	0	0.112	-0.547	1.91	-0.513	-0.173	0.149	-1.118	0.169	1.212	0.153	0.067	-0.265	-0.7	-0.547	0.529	5.014	5.172	4.911	0.394
104	0.071	-0.008	0.487	-0.128	-0.728	0.283	-0.072	0.247	0.02	-0.181	0.391	0.013	-0.187	-0.545	-0.43	0.222	0.298	0.06	-0.191	0.459	-0.036	0.237	0.174	-0.527	-0.717	-0.018
105	-0.45	-0.11	-0.49	0.46	0.62	0.1	0.12	0.05	-0.39	0.04	-0.13	-0.54	-0.05	-0.21	-0.08	0.62	0.4	-0.17	-0.09	0.4	-0.39	0.07	-0.4	-0.15	-0.26	-0.22
106	-0.33	0.08	0.05	-0.17	-0.17	-0.29	-0.06	-0.02	0.36	-0.38	0.26	0.46	-0.25	-0.42	0.29	-0.52	-0.14	-0.14	0.24	0.08	-0.2	0.43	0.24	-0.72	-0.74	0.41
107	-0.091	0.023	0.0358	-0.0876	-0.0372	-0.1336	0.02	0.0522	0.0548	-0.0489	0.0215	0.0304	0.0439	-0.0837	0.0197	-0.1314	-0.0284	0.0725	0.0097	-0.0157	-0.029	0.0144	0.1005	-0.1196	-0.1873	0.0041
108	-0.1326	-0.0457	0.0715	-0.0728	-0.0413	-0.0171	0.171	-0.0125	-0.048	-0.0735	-0.0172	-0.1038	-0.005	0.1127	-0.055	0.0059	0.1169	-0.0243	0.1061	-0.054	-0.1431	0.1049	0.0544	-0.1859	-0.3511	-0.0904
109	-2.8	-1.7	-2.73	-1.26	-1.33	-3.3	-1.54	-1.85	-3.22	-1.02	-3.09	-3.17	-1.36	-1.73	-0.89	-1.85	-1.37	-1.59	-2.67	-3.16	-2.53	-1.5	-2.7	-1.98	-2.07	-3.54
110	-0.34	0.07	0.04	-0.18	-0.18	0.48	-0.07	-0.03	0.36	-0.38	0.25	0.46	-0.25	-0.43	0.27	-0.52	-0.15	-0.14	0.23	0.07	-0.21	0.44	0.24	-0.73	-0.75	0.41
111	-3.19	-1.89	-2.85	-1.46	-1.42	-4.1	-1.66	-1.98	-3.67	-1.29	-4.04	-3.3	-1.71	-1.73	-1.54	-1.8	-1.49	-1.9	-3.07	-3.11	-2.97	-1.83	-2.57	-2.29	-2.27	-3.98
112	-0.24	0.13	0.17	-0.17	-0.18	0.11	-0.02	0.03	0.2	-0.32	0.22	0.07	-0.34	-0.19	-0.09	-0.24	-0.06	-0.16	0.27	0.15	-0.11	0.3	0.56	-0.89	-0.92	0.34
113	4653	5037	1375	3550	3096	3056	4918	1332	3772	3304	6091	1490	3368	2779	1253	3062	3957	4412	4541	1057	3350	5205	1120	8981	8570	2956
114	-0.25	0.22	-0.07	0.24	0.27	-0.11	0.13	0.15	-0.01	0.28	-0.04	-0.06	0.06	0.17	0.2	0.09	0.22	0.12	0.08	-0.02	-0.14	0.24	0.08	-0.24	-0.22	-0.05
115	-0.13	0.09	0.1	-0.1	-0.09	0.07	0	0.02	0.12	-0.17	0.13	0.04	-0.19	-0.09	-0.1	-0.14	-0.04	-0.08	0.16	0.1	-0.06	0.18	0.32	-0.51	-0.51	0.2
116	-3.23	-2.67	-3.31	-2.09	-1.93	-3.25	-2.39	-2.48	-3.3	-1.84	-3.17	-3.24	-2.26	-2.35	-1.94	-2.25	-2.34	-2.46	-3.05	-3.25	-2.9	-2.56	-3.02	-2.67	-2.61	-3.26
117	-2.92	-3.65	-4.37	-3.15	-3.45	-4.89	-3.02	-4.2	-4.65	-2.56	-5.36	-4.46	-3	-2.67	-4.71	-4.13	-3.06	-3.44	-4.29	-4.87	-4.75	-3.26	-4.41	-3.92	-4.05	-4.51
118	-0.13	0.08	0.21	0.21	0.03	0.14	-0.11	-0.31	0.33	0	0.17	0.27	-0.05	-0.11	-0.38	-0.11	0.1	-0.32	0.05	0.01	-0.21	0.16	-0.12	-0.87	-0.87	0.22
119	0.24	-0.16	0.23	-0.01	-0.05	0.16	0.05	0.01	0.15	-0.17	0.02	0.23	0.08	-0.14	-0.29	0.24	-0.1	-0.17	0.08	0.22	0.26	-0.2	0.17	0.45	0.37	0.31
120	-0.4	0.21	0.04	0.12	0.1	-0.04	0.2	0.22	0.14	-0.2	0.08	-0.01	-0.05	-0.05	0.14	-0.12	0.25	0	0.17	-0.11	-0.18	0.27	0.32	-0.71	-0.75	0.08
121	-0.68	-0.14	-0.84	-0.26	0.48	-0.65	-0.08	0.83	0.31	0.86	-0.22	0.16	0.87	0.24	0.73	0.5	0.18	-0.2	0.37	-1.14	-0.64	0.03	0.8	-0.4	-0.91	-0.89
122	-0.17	0.16	-0.54	0.78	0.28	-0.1	-0.36	-0.6	0.66	-0.04	0.1	0.41	0.57	-0.07	0.41	1.24	0.27	-0.28	-0.06	0	-0.79	-0.16	-0.25	-0.57	-0.7	0.43

No.	RG	RH	RI	RK	RL	RM	RN	RP	RQ	RR	RS	RT	RV	RW	RY	SA	SC	SD	SE	SF	SG	SH	SI	SK	SL	SM	SN	SP	SQ
	R	R	R	R	R	R	R	R	R	R	R	R	R	R	R	S	S	S	S	S	S	S	S	S	S	S	S	S	S
	G	H	I	K	L	M	N	P	Q	R	S	T	V	W	Y	A	C	D	E	F	G	H	I	K	L	M	N	P	Q
1	-2	1	1	0	0	-3	-3	2	1	0	-3	-8	-2	-2	-4	0	-7	3	5	-6	-1	-1	-3	-1	-4	-4	1	-1	2
2	-3.8	2	1.1	0.8	0.5	-3.3	-1.7	2.1	1.7	0	-0.4	-5.6	-3.8	-1.7	-3.4	-0.7	-4.7	4.4	5.2	-6.7	0.5	-0.2	-4.1	0.9	-5	-4.1	1.1	-2.6	2.1
3	-3.2	-1.1	1.2	0.7	0.5	-3	-1.2	1.7	1.4	0	-0.1	-6.3	-3	-1.8	-2.4	-0.3	-4.3	3.9	4.6	-5.7	0.5	-0.2	-3.6	1	-4.4	-3.4	1.2	-1.7	1.7
4	-2.6	-1.6	1	0.2	0.4	-2.9	0.3	1.7	0.7	-0.2	0.3	-4.7	-2.3	-1.7	-1.5	-0.1	-3.2	2.9	3.7	-4.3	-0.5	0.2	-2.9	1.2	-3.1	-2.2	1	-0.7	1.7
5	-1.2	1.8	0.3	1.1	0.9	1	-1.9	2	0.9	-1.7	-2	-3.2	0.7	-1.8	-1.8	1.3	-3	3.8	5.7	-2.9	1.4	0.3	-2.3	2	-2.5	-1.8	0.3	-2.1	2
6	-0.29	-0.03	0.13	0.11	0.13	-0.48	0.04	0.51	0.11	-0.14	0.34	-0.22	-0.4	0.25	-0.19	0.08	-0.51	0.37	1	-0.5	-0.23	-0.19	-0.51	0.54	-0.45	-0.14	0.13	-0.11	0.51
7	-2	2	1	1	0	-2	-2	2	1	0	-1	-7	-2	-1	-2	0	-5	3	4	-5	0	1	-2	0	-3	-2	1	-1	2
8	2	2	3	4	4	3	2	4	5	3	2	1	2	3	1	4	0	5	6	0	4	2	1	4	1	1	3	3	4
9	2	1	2	1	1	1	2	1	1	2	2	2	2	2	3	1	3	1	0	3	1	2	3	1	2	2	2	2	1
10	3	0	6	6	9	4	9	6	10	9	9	1	3	8	9	5	5	10	10	2	6	5	3	9	3	4	6	4	6
11	-2.4	-1.6	0.9	0.1	0.5	-2.9	0.5	1.7	0.6	-0.3	0.4	-4.3	-2.1	-1.6	-1.3	0	-3	2.7	3.6	-3.9	-0.8	0.4	-2.7	1.2	-2.8	-2	0.9	-0.5	1.7
12	97	101	42	94	65	152	195	29	58	111	54	152	102	42	87	107	170	45	0	140	98	40	134	56	138	126	42	93	29
13	-3	-2	0	-1	0	-3	-1	2	0	-1	0	-3	-3	-2	0	-1	-3	2	6	-3	-2	0	-3	1	-2	-2	0	0	2
14	-4	-4	0	-2	0	-5	-1	3	0	-2	0	-4	-4	-3	-2	-1	-5	2	7	-5	-3	0	-5	1	-4	-3	0	-2	3
15	-5	-5	-1	-3	0	-6	-1	3	0	-3	-1	-6	-5	-5	-4	-2	-7	2	8	-6	-4	0	-6	1	-6	-4	-1	-2	3
16	-5.4	-3.8	-0.7	-2.1	0.1	-5.2	-3.4	2.4	-0.8	-1.4	-0.2	-7.6	-4.7	-2.5	-2.3	-0.7	-6.9	2.4	8.6	-6.4	-2.5	-2.3	-4.8	1.1	-5.6	-2.8	-0.7	-1.5	2.4
17	-3	0	1	1	1	-2	-1	2	2	0	0	-5	-3	-1	-2	-1	-4	4	5	-5	0	0	-3	1	-4	-3	1	-2	2
18	-3	5	1	3	1	-3	0	7	1	-1	2	-3	-4	-2	-3	0	-3	8	13	-6	3	2	-4	1	-5	-3	1	-3	7
19	0	6.6	0	0	0	0	0	6.6	0	0	6.6	0	0	0	0	9	0	0	10	0	0	0	0	9	5	0	0	0	6.6
20	-1	0	0	0	0	-1	0	1	0	0	0	-1	-1	0	-1	1	0	1	2	-1	0	0	-1	0	-1	-1	0	-1	1
21	-4	3	2	-7	2	-7	-5	5	3	2	2	-14	-10	-19	-3	3	-12	6	15	-18	-6	-3	-3	3	-4	-4	2	-3	5
22	-4	2	8	6	6	-4	1	9	8	5	8	-18	-5	-11	-5	7	-16	12	15	-30	8	-3	-5	13	-14	-12	8	-7	9
23	-3	-8	1	2	3	-4	2	3	1	-1	-1	-16	-11	0	-5	-2	-3	6	24	-24	1	-1	-10	-4	-16	-5	1	-1	3
24	-1	-35	5	0	3	-15	8	7	6	4	12	-40	-1	6	-8	4	-2	20	12	-21	8	-3	-2	14	-10	-3	5	4	7
25	-2	-31	0	3	-4	-13	-22	6	3	-2	-1	-35	-9	-10	-11	-3	-30	10	25	-12	4	-5	-7	2	-13	-5	0	-10	6
26	-14	16	22	-4	8	-30	0	15	6	6	-1	-19	-13	-14	3	8	-1	29	59	-7	13	16	-18	10	-21	5	22	-19	15
27	-6	2	3	-5	3	-7	-5	6	3	2	3	-17	-11	-17	-4	3	-15	8	15	-22	-4	-2	-5	5	-8	-7	3	-3	6
28	-6	-29	5	4	4	-9	4	7	2	1	4	-38	-12	3	-7	0	-5	10	23	-28	3	1	-10	3	-18	-7	5	6	7
29	-9	-5	8	1	2	-18	-5	11	4	1	2	-29	-14	-13	-3	0	-21	17	31	-13	6	5	-13	10	-14	-3	8	-13	11
30	1	3	4	3	3	1	1	5	3	3	3	1	1	3	3	4	0	5	8	0	3	2	1	4	1	1	4	4	5
31	0	0	1	0	1	0	0	3	1	0	0	0	0	0	0	0	0	2	5	0	0	0	0	0	0	0	1	0	3
32	-0.82	-0.26	0.19	0.15	0.11	-0.39	-0.51	0.25	0.18	-0.16	-0.02	-0.66	-1.01	-0.14	-0.86	0.03	-0.64	0.32	0.36	-0.81	0.13	0.13	-0.86	0.25	-0.8	-0.87	0.19	-0.13	0.25
33	2.49	2.72	0.85	2.37	1.4	3.4	1.39	0.84	0.9	1.78	1.45	4.08	2.84	0.97	2.19	2.46	3.26	0.9	0	3.59	2.78	0.96	3.39	1.14	3.53	3.13	0.85	2.48	0.84
34	4	7	10	9	10	3	11	11	10	9	9	7	4	11	10	10	9	11	16	6	6	11	4	11	7	8	10	5	11
35	0.05	0	-0.24	0.08	0.01	-0.22	-0.35	0.39	-0.59	-0.29	0.09	-0.12	-0.17	0.1	-0.08	0.7	-0.34	0.07	1	-0.24	-0.45	-0.14	-0.14	0.5	0.57	0.36	-0.24	-0.08	0.39
36	0.06	0.03	-0.27	0.08	0.01	-0.25	-0.38	0.42	-0.63	-0.31	0.11	-0.12	-0.18	0.09	-0.06	0.74	-0.36	0.09	1	-0.27	-0.46	-0.14	-0.13	0.58	0.62	0.38	-0.27	0.07	0.42
37	-3	-2	0	-1	0	-4	-2	2	-1	-1	0	-6	-3	-2	-2	0	-3	2	5	-4	-2	-2	-3	1	-4	-2	0	-1	2
38	0.305	-0.059	0.206	0.25	0.076	0.149	0.27	0.228	-0.083	0.37	0.375	0.149	0.088	-0.023	0.461	0.3	0.341	0.137	1	0.433	0.164	0.121	0.212	0.247	0.291	0.128	0.206	-0.216	0.228
39	0.108	0.169	0.408	0	0.519	-0.196	0.799	0.254	0.574	0.138	0.037	-0.216	0.099	0	0.441	-0.116	-0.167	0.851	1	-0.187	0	-0.102	-0.135	-0.051	-0.223	-0.18	0.408	0.097	0.254
40	0	0	3	0	3	0	5	2	3	1	0	0	1	0	3	0	0	4	8	0	-1	0	0	1	0	0	3	0	2
41	1.4	-0.8	1.4	-0.4	1.1	0	-1.5	2.1	1.9	1.3	1.9	-1	1	-1.2	-1.2	1.7	-1.5	1	2.2	0.6	0.3	-0.6	1.5	1.4	0.9	0.6	1.4	-0.1	2.1
42	-25	-7	13	8	12	-32	4	37	-3	-8	11	-10	-17	4	-8	12	-18	34	100	-40	-5	-8	-36	24	-40	-17	13	-7	37
43	-11	-3	10	7	4	-13	5	17	-2	1	13	-6	-13	13	-1	2	-8	15	11	-7	1	4	-11	17	-8	1	10	5	17
44	-10	-4	8	8	2	-12	2	13	-2	0	10	-5	-12	10	-2	1	-6	10	8	-7	0	1	-10	13	-7	0	8	0	13
45	4	11	79	36	63	26	98	74	98	60	74	37	26	93	60	39	36	94	100	26	43	68	29	96	31	27	79	50	74
46	-2	0	0	-2	1	-3	0	0	0	0	-1	-2	-2	0	0	-3	-2	0	4	-4	-2	-1	-4	-1	-3	-1	0	-1	0
47	1	3	0	-1	1	0	0	-2	0	2	-1	1	1	2	2	-2	2	-2	-1	-1	0	-1	-1	-2	0	1	0	0	-2
48	0.91	0.63	1.21	1.02	0.93	1.01	1.41	1.26	1.06	1.28	1.15	0.86	0.94	0.92	1.08	1.34	1.43	1.31	1.19	0.91	1.06	1.23	0.96	1.22	1.01	1.02	1.21	0.96	1.26
49	1.23	1.33	0.74	0.61	0.99	0.99	0.75	0.73	0.97	0.71	0.82	1.33	1.11	0.75	0.96	0.63	0.69	0.58	0.74	1.17	0.87	0.78	1.15	0.78	1.12	0.96	0.74	0.75	0.73
50	0.69	1.22	1.04	1.88	1.23	0.99	0.49	0.96	0.92	0.95	1.02	0.61	0.91	1.8	0.88	0.96	0.57	1.17	1.01	0.85	1.15	0.91	0.74	0.93	0.69	1.02	1.04	1.71	0.96
51	14.3	9.94	20	18.7	20.2	15.1	21.5	20.4	18.1	17.1	18.7	15.3	14.2	17	11.4	18.1	12.2	22.8	26.5	9.24	19.3	15.7	14.9	19.2	11.4	15.2	20	19.1	20.4
52	2.8	3.6	6.1	5.3	5.7	2.3	5.7	6.9	5.8	4.9	5.6	0.9	3.1	3.6	3.9	5.2	2.2	7.9	8.8	1.3	4.4	5.6	2.5	6.4	2.4	3.2	6.1	4.7	6.9
53	-7	-6	1	2	2	-10	-5	5	-3	0	3	-3	-7	0	-5	0	-20	5	12	-10	0	0	-8	6	-8	-6	1	-1	5
54	-1.4	-0.04	-0.16	-1.2	0.3	-2.02	0.33	1.23	0.13	-0.57	0.99	-2.7	-1.81	-0.04	-1.04	-0.12	-1.97	1.2	2.97	-1.61	-0.41	-0.62	-1.81	1.11	-2.11	-1.86	-0.16	-0.26	1.23
55	-3.93	1.02	-0.39	-3.91	1.14	-6.1	0.45	3.16	0.6	-1.77	2.83	-6.28	-4.82	-1.79	-3.66	-0.47	-4.7	2.41	8.43	-4.44	-1.8	-1.29	-5.89	3.08	-5.62	-3.94	-0.39	-0.43	3.16
56	-1.9	-1.5	2.3	0.9	1.4	-2.5	1.2	1.7	1.7	1.1	1.9	-4.7	-1.5	-0.8	-0.8	1.2	-2.4	3.2	3.7	-13.7	0.5	0.3	-3.1	1.2	-1.5	-2	2.3	0	1.7
57	-7	-3	6	4	2	-9	2	8	0	0	8	-3	-9	6	-1	0	-6	7	11	-6	0	2	-9	9	-6	-2	6	0	8
58	1	-5	2	3	1	-2	2	6	1	0	2	-1	-4	0	3	2	-8	6	-2	-4	3	-11	-4	2	-1	-3	2	1	6
59	0	2	0	1	4	-3	2	2	0	0	2	-2	-6	0	1	0	-4	5	3	-5	-1	3	-3	4	-2	-3	0	2	2
60	1	0	0	2	3	-1	2	4	0	0	2	-3	-5	1	3	1	-8	4	0	-5	1	-1	-3	3	0	-1	0	1	4
61	-7.7	-9.4	-6.5	-6.3	-6.4	-7.1	-7.7	-6.8	-6.2	-7.1	-7.5	-7.9	-8.1	-7.2	-9.7	-6.7	-10.2	-6.4	-5.4	-8.2	-7.4	-7.8	-8.3	-6.7	-7.4	-8.1	-6.5	-7.8	-6.8
62	-28	-29	3	-10	0	-31	6	19	0	-19	3	-29	-31	-18	-19	-10	-39	20	40	-32	-23	2	-33	14	-31	-21	3	-7	19
63	-6	-7	0	-2	-1	-5	1	1	0	-2	-6	-7	-7	-3	-4	-5	-7	6	12	-5	-3	-1	-5	-4	-5	-5	0	-5	1
64	-5.5	-4.5	-4.5	-4.5	-1.5	-6	2.5	-2.5	1.5	-3	-9	-6	-5.5	-4.5	-4	-7	-7	2	7	-5.5	-6.5	-3	-7	-7.5	-7	-6	-4.5	-7	-2.5
65	-4	-4	0	-1	0	-4	1	2	1	-1	-1	-7	-4	-3	-3	-1	-5	3	6	-6	-2	-1	-5	1	-4	-3	0	-1	2
66	-2	-3	1	0	0	-3	1	2	1	0	0	-5	-2	-1	-2	0	-3	3	4	-4	-1	0	-3	1	-3	-2	1	0	2
67	-3	-2	0	-1	-1	-5	0	1	-1	-2	0	-3	-4	-2	-2	-1	-4	1	5	-4	-2	-1	-4	1	-4	-3	0	-1	1
68	0.046	0.017	0.012	0.134	0.016	0.016	0.043	0.033	0.036	0.033	0.026	0.012	0.047	0.013	0.003	0.519	0.047	0.001	0.022	0.001	0.019	0.396	0.053	0.002	0.012	0.757	0.012	0.092	0.033
69	0.31	-0.52	-1.92	0.93	-7.74	3.12	-0.71	-1.47	-1.81	-7.73	-0.16	-10.01	1.79	-6	-10.67	0.78	-0.76	-9.95	-2.26	1.05	-2.89	2.69	-0.26	-9.45	-1.93	8.91	-1.92	0.13	-1.47
70	0.6	-1.5	-1.6	3.6	0.2	6.3	4.5	1	0.7	-2.8	-0.4	-1.3	0.9	3.4	-4.1	8.1	2.9	-0.6	-1.9	-1.2	-2.1	6.4	0.5	-1.7	-0.4	16.3	-1.6	3.1	1
71	1.27	0.51	-0.6	3.65	-3.19	2.56	-0.7	1.1	-1.28	-2.43	-0.49	-3.91	0.9	-1.42	-3.46	7.03	1.22	-3.62	-2.07	-2.5	-1.78	6.86	1.17	-4.55	-0.33	13.84	-0.6	2.36	1.1
72	-1.9	-0.2	0.9	3.4	-0.1	-0.2	0.5	0.9	2.2	-1	2.1	0.4	-0.3	0.5	-0.3	0	2	0.2	0.8	-0.1	0.5	0	-2.4	0.1	-0.9	0	0.9	-1.9	0.9
73	0	-2	-2	3	-2	0	-2	0	-1	-2	-1	-4	0	0	-3	7	1	-4	-3	-5	-3	7	0	-4	-2	13	-2	2	0
74	-1	-4	-4	-9	-6	-15	9	-4	7	7	-3	-10	-4	-2	-7	7	-8	-5	-6	-7	-8	-3	-6	-10	-6	-10	-4	0	-4
75	35.5	-3.9	-7.9	8.4	-7.4	-4.6	11.4	6.8	16.2	23.5	7.4	13.4	-11.2	3	18.9	6.7	-5.8	-2.3	8.7	8.2	6.8	-11.3	39.5	0.5	-8.7	23.2	-7.9	-7.1	6.8
76	0.1	0.1	1.5	3.3	2.5	0.1	1.5	4.9	7.5	0.2	1	0.1	0.3	0.1	0.1	5.3	0.1	8.6	56.8	0.5	0.1	0.7	0.1	5.8	0.1	5.4	1.5	0.6	4.9
77	0.1	0.1	1.5	3.3	2.5	0.1	1.5	4.9	7.5	0.2	1	0.1	0.3	0.1	0.1	5.3	0.1	8.6	56.8	0.5	0.1	0.7	0.1	5.8	0.1	5.4	1.5	0.6	4.9
78	1.2	4.7	0.1	8.3	0.6	4.3	0.5	1	5.7	62.7	1.2	0.3	1.4	0.1	0.1	18.1	0.9	2.7	2.4	0.1	5	1.1	0.6	3.5	0.2	0.1	1.6	0.5	0.4
79	1.2	4.7	0.1	8.3	0.6	4.3	0.5	1	5.7	62.7	1.2	0.3	1.4	0.1	0.1	18.1	0.9	2.7	2.4	0.1	5	1.1	0.6	3.5	0.2	0.1	1.6	0.5	0.4
80	1.2	4.7	0.1	8.3	0.6	4.3	0.5	1	5.7	62.7	1.2	0.3	1.4	0.1	0.1	18.1	0.9	2.7	2.4	0.1	5	1.1	0.6	3.5	0.2	0.1	1.6	0.5	0.4
81	1.2	4.7	0.1	8.3	0.6	4.3	0.5	1	5.7	62.7	1.2	0.3	1.4	0.1	0.1	18.1	0.9	2.7	2.4	0.1	5	1.1	0.6	3.5	0.2	0.1	1.6	0.5	0.4
82	1.2	4.7	0.1	8.3	0.6	4.3	0.5	1	5.7	62.7	1.2	0.3	1.4	0.1	0.1	18.1	0.9	2.7	2.4	0.1	5	1.1	0.6	3.5	0.2	0.1	1.6	0.5	0.4
83	1.2	4.7	0.1	8.3	0.6	4.3	0.5	1	5.7	62.7	1.2	0.3	1.4	0.1	0.1	18.1	0.9	2.7	2.4	0.1	5	1.1	0.6	3.5	0.2	0.1	1.6	0.5	0.4
84	1.2	4.7	0.1	8.3	0.6	4.3	0.5	1	5.7	62.7	1.2	0.3	1.4	0.1	0.1	18.1	0.9	2.7	2.4	0.1	5	1.1	0.6	3.5	0.2	0.1	1.6	0.5	0.4
85	1.2	4.7	0.1	8.3	0.6	4.3	0.5	1	5.7	62.7	1.2	0.3	1.4	0.1	0.1	18.1	0.9	2.7	2.4	0.1	5	1.1	0.6	3.5	0.2	0.1	1.6	0.5	0.4
86	1.2	4.7	0.1	8.3	0.6	4.3	0.5	1	5.7	62.7	1.2	0.3	1.4	0.1	0.1	18.1	0.9	2.7	2.4	0.1	5	1.1	0.6	3.5	0.2	0.1	1.6	0.5	0.4
87	1.2	4.7	0.1	8.3	0.6	4.3	0.5	1	5.7	62.7	1.2	0.3	1.4	0.1	0.1	18.1	0.9	2.7	2.4	0.1	5	1.1	0.6	3.5	0.2	0.1	1.6	0.5	0.4
88	1.2	4.7	0.1	8.3	0.6	4.3	0.5	1	5.7	62.7	1.2	0.3	1.4	0.1	0.1	18.1	0.9	2.7	2.4	0.1	5	1.1	0.6	3.5	0.2	0.1	1.6	0.5	0.4
89	1.2	4.7	0.1	8.3	0.6	4.3	0.5	1	5.7	62.7	1.2	0.3	1.4	0.1	0.1	18.1	0.9	2.7	2.4	0.1	5	1.1	0.6	3.5	0.2	0.1	1.6	0.5	0.4
90	1.2	4.7	0.1	8.3	0.6	4.3	0.5	1	5.7	62.7	1.2	0.3	1.4	0.1	0.1	18.1	0.9	2.7	2.4	0.1	5	1.1	0.6	3.5	0.2	0.1	1.6	0.5	0.4
91	0.021	0.009	0.053	0	0.004	0.035	0.005	0.021	0.019	0.02	0.032	0.111	0.02	0.033	0	0.264	0.096	0.028	0.005	0.02	0.054	0.043	0.007	0.039	0.033	0.02	0.038	0.014	0.026
92	0.021	0.009	0.053	0	0.004	0.035	0.005	0.021	0.019	0.02	0.032	0.111	0.02	0.033	0	0.264	0.096	0.028	0.005	0.02	0.054	0.043	0.007	0.039	0.033	0.02	0.038	0.014	0.026
93	0.021	0.009	0.053	0	0.004	0.035	0.005	0.021	0.019	0.02	0.032	0.111	0.02	0.033	0	0.264	0.096	0.028	0.005	0.02	0.054	0.043	0.007	0.039	0.033	0.02	0.038	0.014	0.026
94	0.021	0.009	0.053	0	0.004	0.035	0.005	0.021	0.019	0.02	0.032	0.111	0.02	0.033	0	0.264	0.096	0.028	0.005	0.02	0.054	0.043	0.007	0.039	0.033	0.02	0.038	0.014	0.026
101	-4.5	-4.9	-6.2	-3.6	-5.1	-5	-4.1	-4.2	-4.5	-4.3	-3.8	-4	-4.9	-5.8	-5.6	-2.9	-4.6	-2.7	-3.2	-4.7	-3.8	-4.3	-5.5	-3	-4.4	-4.1	-3.1	-3.4	-3.6
102	58	17	33	20	47	9	34	20	29	14	44	36	44	15	45	64	28	37	41	36	145	43	65	51	93	14	42	38	44
103	0	1.759	1.278	4.514	2.952	6.049	1.153	0.099	1.212	3.717	1.048	1.054	0.944	2.069	2.657	0.022	0.376	1.66	1.603	-0.537	0	0.133	-0.476	1.554	-0.441	-0.101	0.134	-1.047	0.153
104	-0.037	0.111	-0.014	0.734	0.374	0.509	-0.303	-0.176	0.222	-0.145	-0.263	0.25	-0.011	-0.244	0.044	0.115	-0.164	-0.565	-0.207	0.357	-0.1	0.08	0.254	-0.246	0.39	0.259	-0.334	0.057	0.298
105	-0.15	-0.01	-0.08	0.3	-0.1	-0.43	0.04	-0.02	0.62	0.23	0.61	-0.17	-0.48	-0.78	0.21	-0.01	-0.2	-0.09	0.3	0.14	-0.04	-0.59	-0.13	0.18	-0.07	-0.47	0.37	0.44	0.4
106	-0.04	-0.12	0.42	0.75	0.35	0.31	-0.14	-0.38	-0.52	0.11	0.17	-0.35	0.3	-0.16	-0.25	-0.06	-0.02	-0.31	-0.26	0.29	-0.16	-0.05	0.21	-0.13	0.25	0.14	-0.14	0.01	-0.14
107	0.0711	-0.0218	0.0338	0.0131	0.0164	0.0583	-0.0321	-0.062	-0.1314	0.0543	0.0856	-0.093	0.023	-0.0219	-0.0652	0.0065	0.0158	0.0006	-0.1229	-0.0001	-0.0505	0.0017	0.007	-0.0366	0.0255	0.0551	-0.0005	0.112	-0.0284
108	-0.0251	-0.0184	-0.0266	0.0839	-0.0651	-0.0163	-0.015	-0.0614	0.0059	0.0306	0.0442	-0.021	0.0475	-0.207	-0.1369	0.0464	-0.0132	0.0424	0.0823	0.0398	0.1075	-0.0005	0.0332	0.169	0.0959	0.0185	0.1452	0.1626	0.1169
109	-1.68	-2.12	-3.33	-0.06	-3.15	-3.49	-1.41	-1.85	-1.85	-1.39	-1.22	-1.97	-2.78	-3.56	-2.75	-1.89	-2.86	-1.46	-1.48	-3.56	-1.7	-1.94	-3.43	-0.83	-3.16	-3.55	-1.31	-1.35	-1.37
110	-0.04	-0.13	0.42	0.75	0.36	0.31	-0.13	-0.38	-0.52	0.11	0.17	-0.35	0.29	-0.16	-0.26	-0.06	-0.03	-0.32	-0.27	0.28	-0.17	-0.06	0.21	-0.13	0.24	0.14	-0.14	0.01	-0.15
111	-1.72	-2.16	-3.63	-0.59	-4.03	-3.12	-1.64	-1.7	-1.8	-1.55	-1.62	-1.9	-3.07	-3.41	-3.16	-2.01	-2.86	-1.63	-1.48	-4.02	-1.82	-2.11	-3.52	-1.05	-3.92	-3.03	-1.58	-1.57	-1.49
112	0.03	-0.04	0.35	0.49	0.34	0.36	-0.16	-0.05	-0.24	0.12	-0.08	-0.05	0.38	-0.04	-0.19	-0.01	0.14	-0.36	-0.26	0.17	-0.2	-0.12	0.33	-0.1	0.32	0.32	-0.23	-0.05	-0.06
113	5656	1883	4065	1803	6653	1463	3325	3036	3062	1558	4790	4720	4867	1588	4433	11447	3012	8327	6343	5567	12019	3073	6443	5117	10084	2205	5920	4843	3957
114	0.09	0.05	0	0.66	-0.04	0.03	0.1	0.17	0.09	0.19	0.16	0.11	0.08	-0.21	-0.25	0.1	-0.13	0.1	0.18	-0.12	-0.01	0.04	0.03	0.36	-0.02	0.05	0.09	0.2	0.22
115	0.03	-0.01	0.2	0.28	0.2	0.2	-0.08	-0.02	-0.14	0.03	-0.03	-0.02	0.23	-0.02	-0.1	0.01	0.08	-0.2	-0.14	0.1	-0.1	-0.05	0.2	-0.05	0.19	0.19	-0.12	-0.02	-0.04
116	-2.43	-2.6	-3.42	-1.47	-3.24	-3.15	-2.19	-2.29	-2.25	-2.03	-2.46	-2.55	-3	-3.42	-3.01	-2.76	-3.56	-2.31	-2.22	-3.46	-2.61	-2.76	-3.58	-2.01	-3.47	-3.35	-2.36	-2.57	-2.34
117	-2.71	-3.24	-4.29	-2.11	-5.01	-4.13	-2.67	-2.43	-4.13	-3.98	-2.78	-2.43	-3.7	-4.54	-4.32	-2.49	-3.41	-2.34	-2.57	-4.01	-1.68	-3.12	-3.47	-1.91	-4.12	-3.33	-2.13	-1.56	-3.06
118	-0.1	0.35	0.37	0.15	0.21	0.3	-0.02	-0.17	-0.11	-0.26	0.08	0.38	0.35	0.04	-0.07	0.08	0.02	-0.13	-0.25	-0.14	0.08	-0.38	0.33	-0.5	0.26	0.25	-0.32	-0.15	0.1
119	0.01	0.1	0.1	-0.01	0.07	0.37	0.24	0.03	0.24	-0.11	0.19	0	-0.04	0.37	0.42	-0.15	0.15	0	-0.1	0.05	-0.19	0.08	-0.14	-0.24	-0.19	-0.01	-0.16	-0.13	-0.1
120	0.14	0.04	0.18	0.5	0.09	0.17	0.02	-0.02	-0.12	0.13	0.12	0	0.17	-0.41	-0.37	0.15	0.09	0.01	0.1	0.1	0.1	0.15	0.35	0.1	0.26	0.32	0.14	0.17	0.25
121	0.12	-0.62	0.31	0.56	0.28	0.4	0.82	-0.59	0.5	1.01	0.84	-0.19	0.41	0.23	-1.64	0.18	0.47	0.32	0.15	-0.79	-0.05	1.17	0.49	-0.04	0.8	-0.2	-0.78	0.6	0.18
122	0.84	0.36	-0.22	1.24	-0.04	0.67	0.14	-0.31	1.24	0.65	0.91	-0.44	0.53	-0.14	0.54	0.24	-0.41	0.29	1.14	-0.01	-0.09	-0.28	-0.14	0.13	1.13	-0.59	-0.17	0.48	0.27

Table S12 Amino acid descriptors (2D) used in this study.

	P	Q	Q	Q	Q	Q	Q	Q	Q	Q	Q	Q	Q	Q	Q	Q	Q	Q	Q	Q	Q	R	R	R	R	R	R	R
	Y	A	C	D	E	F	G	H	I	K	L	M	N	P	Q	R	S	T	V	W	Y	A	C	D	E	F	G	H
No.	PY	QA	QC	QD	QE	QF	QG	QH	QI	QK	QL	QM	QN	QP	QQ	QR	QS	QT	QV	QW	QY	RA	RC	RD	RE	RF	RG	RH
123	-1	0	-0.2	-0.3	-0.4	-0.8	0	-0.5	-0.4	-0.4	-0.6	-0.6	-0.7	-0.3	-0.5	-0.9	0	-0.5	-0.4	-1	-1.1	0.2	0	-1.4	-1.5	-0.9	0	-1
124	-3.5	-2.7	-2.9	-2.7	-2.2	-3	0	-2.5	-3.2	-2.7	-3.1	-3.3	-3.2	-2.5	-2.6	-2.9	-2.3	-3.1	-3.4	-3.3	-3.4	-2.4	-2.6	-3.7	-3.2	-3	0	-2.9
125	-0.9	0	0.1	0	0.2	0	-0.1	0.9	0.3	0.4	0.4	0.2	-0.4	-0.1	0	-0.5	0	-0.2	0.2	-0.2	-0.5	0.5	0.4	-1.1	-0.9	-0.4	0.5	-0.4
126	-0.4	0	0.1	-0.3	-0.3	0.1	0	-0.2	0.2	-0.3	0.3	0.1	-0.6	-0.4	-0.4	-0.4	-0.3	-0.2	0.2	-0.1	-0.2	0.3	0.4	-1	-1	0.2	0	-0.2
127	-0.5	0.6	0.3	0.6	0.8	-0.1	0.8	0.1	0.1	0.5	0.2	0.1	0.2	0.2	0.3	0.2	0.6	0.4	0.2	-0.4	-0.3	0.6	0.7	-0.5	-0.4	-0.4	0.2	-0.1
128	-0.5	0.6	0.2	0.2	0.2	-0.1	0.8	-0.1	0	0.1	0	-0.1	0	0.4	0	0	0.2	0.1	0.3	-0.5	-0.3	0.4	0.2	-0.6	-0.5	-0.4	0.3	-0.1
129	-0.6	0.4	-0.7	0	0.1	-0.3	0.5	-0.5	-0.1	0	0	-0.5	-0.2	0	-0.5	-0.2	0.1	0	0.1	-0.9	-0.4	0.3	-0.6	-0.6	-0.5	-0.4	0.2	-0.4
130	-0.8	0.6	0.1	1.2	1.6	-0.4	0.6	-0.1	0.3	1.5	0.3	0.3	0.8	0.6	0.8	0.2	0.7	0.6	0.4	-0.4	-0.3	0.6	-0.7	-0.2	-0.1	-0.5	0.7	0
131	-0.8	0.4	0.3	0.6	0.7	-0.3	0.5	-0.2	0.7	0.8	0.4	0.5	0.3	0.5	0.6	-0.4	0.3	0.4	0.6	-0.5	-0.4	0.5	-0.6	-0.8	-0.7	-0.4	0.4	-0.4
132	-0.8	0.2	0	-0.3	-0.2	-0.6	0.2	-0.9	0	-0.3	-0.2	0	-0.3	0.3	-0.3	-1	-0.2	-0.4	0	-0.9	-0.8	0.3	-0.6	-1.2	-1.4	-0.6	0.3	-0.9
133	5.8	5	5.5	5.3	5.4	5.7	5.1	5.2	5.4	5.7	5.8	4.9	5.3	5.7	5.8	6.1	5.4	5.6	5.6	5.5	5.6	5.5	5.9	5.6	5.7	5.7	6.7	5.7
134	5.6	5.4	5.4	5.5	5.7	6.1	5.9	6.2	5.7	5.8	5.9	5.6	5.9	5.4	5.4	6	5.3	5.3	5.4	6.2	6.7	5.5	5.6	5.6	5.9	6.1	6.3	5.9
135	6	5.3	6.1	5.5	6.5	6.1	7.2	6.8	6	5.8	5.6	5.2	5.9	5.8	6.1	6.4	5.3	6.3	6	6.7	5.5	5.5	6.1	6	6.1	5.9	6.7	5.5
136	0.001	0.005	0.0014	-0.0015	-0.0052	0.0038	-0.0004	0.0058	-0.0019	0.0023	-0.0042	-0.0029	0.0045	0.0005	0.0084	-0.0012	-0.009	0.001	3E-05	0.0121	-0.0051	-0.0025	-0.0061	0.002	-0.0046	0.0049	0.0025	0.01
137	-0.4161	0.2658	0.252	-0.0006	-0.0584	0.1434	-0.0189	-0.0305	0.2056	-0.248	0.0752	-0.0354	-0.2614	-0.1657	-0.2407	-0.2531	-0.0614	-0.2301	0.0858	-0.0457	-0.0378	0.3712	0.491	-0.7824	-0.7861	0.0314	-0.0832	-0.1089
138	-0.1066	0.0889	0.0607	-0.2211	-0.2264	0.1169	-0.1651	-0.1302	0.3146	-0.2164	0.3222	0.1375	-0.29	-0.188	-0.254	-0.1085	-0.1934	-0.16	0.2812	-0.0303	-0.0242	0.0952	0.2119	-0.7576	-0.7311	0.28	-0.1149	0.0284
139	-0.0559	0.0719	0.027	-0.122	-0.1063	0.0641	-0.0205	-0.0724	0.1356	-0.1615	0.1292	0.0196	-0.1179	-0.0886	-0.0969	-0.1349	-0.0542	-0.0182	0.1181	-0.0687	-0.0471	0.0615	0.1034	-0.3095	-0.2898	0.1521	-0.0503	-0.0224
140	-0.0477	0.0202	0.0267	-0.0043	-0.0266	0.0194	-0.0266	-0.0011	0.0416	0.0131	-0.0052	0.0128	-0.0251	-0.0632	-0.066	-0.0221	0.0225	0.0165	0.0177	0.0015	-0.0333	0.0032	-0.0216	-0.0461	-0.0543	0.0079	0.0093	0.0229
141	-0.0038	0.0034	0.0099	-0.0064	-0.0073	0.007	-0.0024	-0.0022	0.012	-0.0077	0.0087	0.0039	-0.0069	-0.0061	-0.0075	-0.0061	-0.0026	-0.0019	0.0095	0.0005	0.0001	0.0034	0.012	-0.016	-0.0152	0.009	-0.0019	7E-05
142	-0.41	0	-0.41	0.15	0.47	-0.48	-0.01	0.43	-1	0.53	-0.64	-0.23	0.2	0.94	0.59	0.41	0.24	-0.03	-0.32	-0.88	-0.33	0.02	-0.54	-0.16	-0.11	-0.39	-0.11	0.34
143	-1.69	-1.17	-1.97	-0.71	-0.47	-1.59	-1.6	-1.04	-1.45	-0.42	-1.38	-1.66	-0.74	-0.58	-0.1	-0.68	-1.1	-1.03	-1.33	-1.72	-1.31	-1.24	-0.78	-1.51	-1.24	-1.44	-1.25	-0.94
144	0.04	0.7	-0.05	0.96	1.11	0.39	0.73	0.83	0.24	1.29	0.38	0.65	0.87	1.09	1.22	0.94	0.75	0.81	0.23	-0.01	0.09	0.56	-0.25	0.33	0.54	0.24	0.65	0.47
145	-0.73	1.14	0.48	1.66	2.42	-0.59	0.34	1.98	-0.12	1.8	0.24	0.14	0.46	1.46	1.17	2.54	1.27	0.38	0.45	-0.23	0.09	1.26	0.78	0.76	1.05	0.33	0.95	2.19
146	-0.16	1.16	-0.24	1.88	1.6	0.25	0.42	0.9	0.24	1.68	0.11	0.68	1.23	1.44	1.62	1.26	1.47	1.03	0.66	-0.14	0.38	1.17	0.1	0.4	0.91	-0.06	0.72	0.81
147	-0.42	0.45	0.26	0.41	1.25	0.29	0.38	0.73	0.07	1.1	0.22	0.36	0.52	0.5	0.93	0.92	0.85	0.58	0.17	0.65	0.16	0.47	-0.46	-0.25	0.89	0.03	0.12	0.75
148	42.05	30.59	33.49	33.287	36.381	43.817	27.496	39.403	39.871	42.304	39.871	39.677	34.919	35.443	38.012	44.504	31.392	34.485	36.777	49.104	44.619	37.082	39.982	39.779	42.873	50.309	33.988	45.895
149	1	1	1	1	1	1	1	0	1	1	1	1	1	1	1	0	1	1	1	0	0	1	1	1	0	0	1	0
150	0	0	0	0	0	0	0	0	0	0	0	0	0	0	0	0	0	0	0	0	0	0	0	0	0	0	0	0
151	0	0	0	0	0	0	0	2	0	0	0	0	0	0	0	1	0	0	0	1	1	0	0	0	1	1	0	1
152	2	2	2	2	2	2	2	4	2	2	2	2	2	2	2	3	2	2	2	3	3	2	2	2	3	3	2	3
153	2	2	2	2	2	2	2	4	2	2	2	2	3	2	3	2	3	3	2	2	3	1	1	1	1	1	1	3
154	2	2	2	4	4	2	2	2	2	2	2	2	2	2	2	2	2	2	2	2	2	2	2	4	4	2	2	2
155	6	0	0	0	0	6	0	5	0	0	0	0	0	0	0	0	0	0	0	9	6	0	0	0	0	6	0	5
156	1	1	1	1	1	1	1	1	1	2	1	1	1	1	1	4	1	1	1	1	1	4	4	4	4	4	4	4
157	38	30	31	32	35	40	27	37	39	42	39	37	34	34	37	44	31	34	36	44	41	37	38	39	42	47	34	44
158	2	2	2	2	2	2	2	4	2	2	2	2	3	1	3	2	3	3	2	3	3	1	1	1	1	1	1	3
159	4	4	4	4	4	4	4	8	4	4	4	4	6	3	6	4	6	6	4	5	6	2	2	2	2	2	2	6
160	20	15	16	18	19	21	14	20	18	19	18	18	18	17	19	21	16	17	17	24	22	17	18	20	21	23	16	22
161	12	5	5	5	6	11	4	7	8	8	8	8	5	7	6	7	5	6	7	13	11	6	6	6	7	12	5	8
162	61.06	51.848	58.222	57.903	62.041	66.109	47.87	65.6	63.083	68.382	63.083	66.065	60.835	57.513	64.862	74.61	54.612	58.593	59.433	73.776	69.282	61.417	68.089	68.981	72.966	76.059	57.551	74.682
163	1.6068	1.7283	1.8781	1.8095	1.7726	1.6527	1.773	1.773	1.6175	1.6281	1.6175	1.7855	1.7893	1.6916	1.753	1.6957	1.7617	1.7233	1.6509	1.6767	1.6898	1.6599	1.7918	1.7688	1.7373	1.6183	1.6927	1.6973
164	14	8	8	9	10	14	7	11	11	11	11	10	9	10	10	11	8	9	10	16	14	9	9	10	11	15	8	12
165	18	15	15	14	16	19	13	17	21	23	21	19	16	17	18	23	15	17	19	20	19	20	20	19	21	24	18	22
166	2	3	3	3	3	3	3	5	3	4	3	3	4	3	4	6	3	3	3	4	3	5	5	5	5	5	5	7
167	4	4	4	6	6	4	4	4	4	4	4	4	5	4	5	4	5	5	4	4	5	3	3	5	5	3	3	3
168	0	0	1	0	0	0	0	0	0	0	0	1	0	0	0	0	0	0	0	0	0	0	1	0	0	0	0	0
169	1.8051	3.8384	3.9854	4.1939	4.1846	2.4125	3.606	2.3909	4.2809	4.1069	4.1913	4.1041	4.1939	2.4761	4.1846	4.1128	3.9854	4.1692	4.1692	1.8528	2.4265	3.7113	3.8469	4.0545	4.0693	2.3259	3.5158	2.2986
170	-2.5605	-2.5869	-2.5878	-2.5901	-2.6122	-2.593	-2.571	-2.5886	-2.6861	-2.665	-2.679	-2.6144	-2.5877	-2.5907	-2.609	-2.6376	-2.5864	-2.6079	-2.6544	-2.5909	-2.5929	-2.6239	-2.6242	-2.6251	-2.6349	-2.6263	-2.6168	-2.6245
171	-0.6113	-0.5482	-0.5477	-0.5491	-0.5491	-0.5488	-0.5576	-0.5477	-0.5031	-0.5389	-0.5316	-0.536	-0.5477	-0.5477	-0.5477	-0.5432	-0.5476	-0.5476	-0.5069	-0.5495	-0.5724	-0.5477	-0.5408	-0.5484	-0.5484	-0.5509	-0.548	-0.5498
172	0.6503	0.4159	0.4662	0.4409	0.4409	0.5437	0.4199	0.4662	0.4143	0.4156	0.4155	0.3903	0.457	0.4654	0.4647	0.4409	0.4662	0.466	0.4144	0.5491	0.5196	0.3908	0.4883	0.437	0.4392	0.5706	0.3967	0.4977
173	2.7855	2.6576	2.6591	2.6597	2.6635	2.6599	2.6524	2.6605	2.6807	2.6751	2.674	2.6637	2.6622	2.7337	2.6657	2.6701	2.66	2.6666	2.6716	2.6601	2.6599	2.6763	2.6773	2.6777	2.6802	2.6778	2.6725	2.6783
174	-3.3808	-3.3083	-3.3084	-3.3083	-3.3083	-3.3083	-3.3081	-3.3083	-3.3084	-3.3083	-3.3083	-3.3083	-3.3083	-3.3063	-3.3083	-3.3083	-3.3084	-3.3085	-3.3084	-3.3083	-3.3083	-3.3086	-3.3087	-3.3086	-3.3086	-3.3087	-3.3084	-3.3087
175	-0.6151	-0.49	-0.5682	-0.5324	-0.5295	-0.5381	-0.4976	-0.5626	-0.4787	-0.4895	-0.4785	-0.4503	-0.5655	-0.5222	-0.5629	-0.5288	-0.5682	-0.5653	-0.4855	-0.5292	-0.5582	-0.4518	-0.5884	-0.5306	-0.5214	-0.5093	-0.4624	-0.5258
176	0.6317	0.3564	0.3931	0.3747	0.3747	0.4812	0.3569	0.4143	0.3564	0.3564	0.3564	0.3931	0.391	0.393	0.393	0.3747	0.3931	0.3931	0.3564	0.5984	0.4519	0.3381	0.431	0.3838	0.3851	0.6128	0.3388	0.4623
177	2.6237	2.5306	2.53	2.5306	2.5502	2.5311	2.5283	2.5305	2.6835	2.6646	2.6894	2.5548	2.5308	2.6873	2.5528	2.5946	2.5292	2.5314	2.6145	2.5316	2.5313	2.5845	2.5843	2.5845	2.5879	2.5846	2.5835	2.5844
178	-2.3361	-2.391	-2.3903	-2.3916	-2.3977	-2.3923	-2.3857	-2.392	-2.4387	-2.439	-2.4322	-2.3967	-2.392	-2.3941	-2.3982	-2.4096	-2.3918	-2.3991	-2.4165	-2.392	-2.3923	-2.4243	-2.4239	-2.4245	-2.4274	-2.4249	-2.4212	-2.4247
179	-0.4737	-0.2949	-0.3552	-0.3249	-0.3249	-0.3878	-0.2997	-0.3553	-0.2921	-0.2945	-0.2944	-0.2641	-0.3463	-0.3516	-0.3541	-0.3249	-0.3552	-0.3412	-0.2924	-0.3886	-0.404	-0.2647	-0.355	-0.3159	-0.316	-0.4168	-0.2718	-0.3759
180	0.7994	0.5375	0.6397	0.5882	0.5882	0.6909	0.5453	0.6401	0.5286	0.5368	0.5366	0.4856	0.6185	0.6299	0.6356	0.5882	0.6395	0.6044	0.5305	0.7041	0.6816	0.4861	0.6184	0.5519	0.5524	0.7132	0.4973	0.6416
181	2.9696	2.8506	2.8598	2.857	2.8694	2.8584	2.8382	2.8573	2.9122	2.8929	2.901	2.8794	2.8598	2.9796	2.8721	2.8846	2.8529	2.8684	2.8909	2.8596	2.8585	2.8733	2.879	2.8772	2.8847	2.878	2.8656	2.8774
182	23.212	18.91	21.19	19.733	21.919	23.283	16.724	23.077	25.469	26.336	25.469	25.563	20.301	22.417	22.488	26.996	18.91	21.097	23.283	25.036	23.283	23.418	25.698	24.241	26.427	27.791	21.232	27.585
183	5	6	7	8	9	8	6	8	8	10	8	9	8	6	9	10	7	7	7	8	8	7	8	9	10	9	7	9
184	0.2381	0.4286	0.4667	0.4706	0.5	0.381	0.4615	0.4	0.4706	0.5556	0.4706	0.5294	0.4706	0.3529	0.5	0.5	0.4667	0.4375	0.4375	0.32	0.3636	0.4375	0.4706	0.4737	0.5	0.3913	0.4667	0.4091
185	6	0	0	0	0	6	0	5	0	0	0	0	0	0	0	0	0	0	0	10	6	0	0	0	0	6	0	5
186	39	29	30	31	34	40	26	37	38	41	38	36	33	34	36	43	30	33	35	45	41	36	37	38	41	47	33	44
187	2	3	3	4	4	3	3	3	3	3	3	3	4	3	4	4	3	3	3	3	3	3	3	4	4	3	3	3
188	21	14	15	17	18	21	13	20	17	18	17	17	17	17	18	20	15	16	16	25	22	16	17	19	20	23	15	22
189	4	5	5	5	5	5	5	5	6	8	6	7	5	5	5	7	5	5	5	5	5	7	7	7	7	7	7	7
190	6	7	8	9	10	9	7	9	9	11	9	10	9	6	10	12	8	8	8	9	9	9	10	11	12	11	9	11
191	0.2857	0.5	0.5333	0.5294	0.5556	0.4286	0.5385	0.45	0.5294	0.6111	0.5294	0.5882	0.5294	0.3529	0.5556	0.6	0.5333	0.5	0.5	0.36	0.4091	0.5625	0.5882	0.5789	0.6	0.4783	0.6	0.5
192	31	26	27	27	30	31	23	29	35	38	35	33	29	31	32	39	27	30	32	32	32	33	34	34	37	38	30	36
193	14.535	12.008	12.715	14.293	15	15.828	11.138	15.121	14.293	14.836	14.293	14.129	14.293	13	15	16.414	12.715	13.585	13.585	17.69	16.698	13.422	14.129	15.707	16.414	17.242	12.552	16.535
194	11.042	8.4864	9.5351	9.51	10.217	11.58	7.6161	10.795	10.771	11.022	10.771	11.125	9.6791	9.5549	10.386	11.762	8.6407	9.5109	10.064	13.08	11.95	9.8626	10.911	10.886	11.593	12.956	8.9923	12.172
195	8.2925	5.0689	4.776	5.276	5.9831	8.1628	4.1987	6.4307	7.3534	6.8973	7.3534	6.4831	5.276	6.1902	5.9831	6.6902	4.776	5.6463	6.6463	9.1628	8.0854	5.776	5.4831	5.9831	6.6902	8.8699	4.9058	7.1378
196	9.121	5.301	5.0081	5.5854	6.2925	9.121	4.4307	6.9996	7.5854	7.1294	7.5854	6.7152	5.5854	6.4223	6.2925	6.9996	5.0081	5.8783	6.8783	10.276	8.9912	6.0081	5.7152	6.2925	6.9996	9.8281	5.1378	7.7067
197	9.5586	6.8411	7.3791	8.235	8.735	9.8968	6.4304	9.3968	8.2898	8.8791	8.235	8.3791	8.235	7.9304	8.735	9.735	7.3791	7.7518	7.7518	11.38	10.291	7.8411	8.3791	9.235	9.735	10.897	7.4304	10.397
198	6.685	4.4686	5.2482	5.0613	5.5613	6.5637	4.021	6.0009	5.9173	6.2995	5.8624	6.8903	5.1458	5.5589	5.6458	6.4804	4.6157	5.0601	5.3793	7.5577	6.698	5.3031	6.0827	5.8958	6.3958	7.3983	4.8556	6.8354
199	4.5688	2.4165	2.2474	2.601	3.101	4.5116	1.904	2.8896	3.8652	3.7474	3.8103	2.7474	2.601	3.2474	3.101	3.2474	2.2474	2.7498	3.3272	4.967	4.4223	2.5629	2.3938	2.7474	3.2474	4.6581	2.0505	3.0361
200	5.5244	2.5605	2.3914	2.7997	3.2997	5.6162	2.0581	3.2079	4.0092	3.8914	3.9544	2.8914	2.7997	3.3914	3.2997	3.3914	2.3914	2.8938	3.4712	6.1911	5.4327	2.6523	2.4832	2.8914	3.3914	5.7079	2.1498	3.2997
201	2	2	2	2	2	2	1	2	3	2	2	2	2	2	2	2	2	3	2	2	2	2	2	2	2	2	1	2
202	2	2	2	2	2	2	1	2	3	2	2	2	2	2	2	2	2	3	2	2	2	2	2	2	2	2	1	2
203	0.7691	0.8151	0.8661	0.8784	0.8552	0.7689	0.8394	0.8194	0.7631	0.7683	0.7631	0.8212	0.8483	0.8026	0.8281	0.7931	0.848	0.8257	0.7778	0.7787	0.7931	0.7751	0.8209	0.8325	0.8155	0.745	0.7918	0.7868
204	11	9	9	10	11	12	9	11	10	12	10	11	10	9	11	13	9	9	9	13	13	11	11	12	13	14	11	13
205	0	0	0	-1	-1	0	0	0	0	1	0	0	0	0	0	1	0	0	0	0	0	1	1	0	0	1	1	1

	R	R	R	R	R	R	R	R	R	R	R	R	R	S	S	S	S	S	S	S	S	S	S	S	S	S	S
	I	K	L	M	N	P	Q	R	S	T	V	W	Y	A	C	D	E	F	G	H	I	K	L	M	N	P	Q
No.	RI	RK	RL	RM	RN	RP	RQ	RR	RS	RT	RV	RW	RY	SA	SC	SD	SE	SF	SG	SH	SI	SK	SL	SM	SN	SP	SQ
123	-0.7	0.1	-0.6	-0.5	-0.8	-0.2	-0.9	-0.9	-0.4	-0.6	-0.5	-1.3	-1.4	0.5	-0.1	-0.3	-0.2	-0.4	0	-0.6	-0.1	0.1	0	-0.1	-0.1	0.2	0
124	-3.3	-2	-3	-3.1	-3.2	-2.4	-2.9	-2.9	-2.6	-3.1	-3.5	-3.5	-3.6	-2.4	-3	-2.8	-2.3	-2.8	0	-2.7	-3	-2.3	-2.6	-3	-2.8	-2.2	-2.3
125	0.4	1.7	0.2	0.1	0	0.5	-0.5	0.2	0	0.1	0.3	-0.9	-0.4	-0.1	-0.4	-0.9	-0.2	0	0	-0.6	0.4	0.5	0.4	0	-0.3	0.4	0
126	0.3	0.3	0.3	0.3	-0.5	-0.3	-0.4	-0.4	-0.3	-0.1	0.4	-0.1	-0.3	0.1	0.1	-0.5	-0.5	0.1	0	-0.3	0.4	-0.3	0.4	0.3	-0.4	-0.3	-0.3
127	-0.2	1.1	-0.1	0.2	0	0.1	0.2	-0.1	0.2	0	0	-0.6	-0.7	0.9	0.6	0.7	0.6	0.1	0.8	0	0.3	0.9	0.4	0.4	0.7	0.6	0.6
128	-0.2	0.6	-0.2	0	0	0.1	0	-0.1	0.3	0	0.1	-0.6	-0.7	0.9	0.4	0.3	0.4	0.1	0.9	0.1	0.4	0.7	0.4	0.3	0.4	0.6	0.2
129	-0.2	0.4	-0.2	-0.4	-0.2	0	-0.2	-0.3	0.2	0	0	-0.9	-0.7	0.8	-0.4	0.2	0.3	0	0.8	-0.2	0.3	0.5	0.4	-0.1	0.2	0.4	0.1
130	-0.4	1	-0.4	-0.3	0.1	-0.1	0.2	0.4	-0.2	-0.1	-0.3	-0.5	-0.3	0.3	-0.1	0.7	0.9	-0.7	0.1	-0.6	0.4	1.3	0.3	0.6	0.5	0.5	0.7
131	-0.2	0.2	-0.4	-0.4	-0.3	-0.4	-0.4	-0.2	-0.4	-0.3	-0.3	-0.6	-0.5	0.3	-0.1	0.2	0.5	-0.6	0.1	-0.6	0.4	0.7	0.4	0.4	0.3	0.5	0.3
132	-0.6	-0.4	-0.6	-0.6	-0.8	-0.5	-1	-0.8	-0.8	-0.9	-0.6	-0.7	-1	-0.2	-0.5	-0.5	-0.2	-0.8	0	-0.9	0	-0.1	0	0	-0.4	0.3	-0.2
133	5.9	6.5	5.8	5.8	5.5	5.7	6.1	6.5	5.6	5.9	5.6	6	6	4.7	5.1	5.2	5.1	5.4	4.5	5.3	5.3	4.9	5.2	5.5	5.2	5.4	5.4
134	6.1	6.3	6.1	6.3	6.1	6.1	6	6.6	5.4	6	6	5.9	6.3	4.7	5.3	4.7	5.2	5.6	4.4	5.4	5.4	5.5	5.6	5.2	5.1	5.2	5.3
135	6.2	5.3	6	6.1	6	5.3	6.4	6.7	5.7	6	6	6.3	6.5	4.9	5.5	5.4	4.9	5.5	5.1	5.5	5.8	5.4	5.5	5.5	5.2	5.1	5.3
136	0.001	0.0073	-0.0013	-0.0047	-0.0067	-7E-05	-0.0012	0.0099	-0.0012	0.004	-0.0052	-0.0148	0.0042	-0.0015	0.0018	-0.0016	0.0029	-0.0012	-0.0035	0.0099	0.0015	0.005	0.001	0.002	0.0062	-0.0031	-0.009
137	0.3358	0.5516	0.1978	0.13	-0.2705	-0.07	-0.2531	0.2325	-0.0255	-0.0531	0.3328	-0.3312	-0.1449	0.1048	0.1128	-0.5029	-0.3531	0.2801	-0.2712	-0.113	0.313	0.0272	0.2714	0.197	-0.2883	-0.1079	-0.0614
138	0.3477	0.0572	0.3112	0.2375	-0.2048	-0.1175	-0.1085	0.0755	-0.1882	-0.058	0.3325	0.0402	0.0155	0.0035	-0.062	-0.1746	-0.2114	0.0981	-0.2097	-0.1938	0.352	-0.131	0.3421	0.1082	-0.2376	-0.112	-0.1934
139	0.1911	-0.0335	0.1851	0.0707	-0.1577	-0.1662	-0.1349	-0.0847	-0.0918	-0.0465	0.1729	0.0774	0.0223	0.0536	-0.0084	9E-05	0.007	-0.0129	0.0087	-0.0645	0.0421	-0.0377	0.0129	-0.0241	-0.0751	-0.0211	-0.0542
140	0.0613	-0.0384	0.0187	0.0438	-0.0501	-0.0134	-0.0221	-0.074	-0.0008	0.0352	0.0362	-0.0337	-0.0466	0.0028	-0.016	0.0167	0.0472	-0.0024	-0.0257	0.0024	0.0059	0.0541	-0.0014	-0.0269	-0.0099	0.0047	0.0225
141	0.014	-0.0042	0.0105	0.0067	-0.0074	-0.0055	-0.0061	-0.0041	-0.0036	-0.0008	0.0121	0.0014	0.0006	0.0017	0.001	-0.0031	-0.0023	0.0028	-0.0032	-0.0033	0.0074	-0.0015	0.0057	0.0011	-0.0046	-0.0019	-0.0026
142	-0.77	1.21	-0.63	-0.1	0.25	0.45	0.41	1.08	0.24	0.09	-0.47	-0.65	-0.49	-0.83	-1.36	0.11	0.5	-1.13	-1.11	-0.08	-1.35	0.37	-1.24	-0.57	-0.25	0.32	0.24
143	-1.41	0.28	-1.18	-0.98	-0.8	-0.84	-0.68	0.03	-0.81	-0.89	-1.26	-1.39	-1.57	-1.74	-2.23	-1.09	-0.85	-1.77	-2.03	-1.53	-1.6	-0.83	-1.8	-1.72	-1.16	-0.48	-1.1
144	0.05	1.65	0.26	0.58	0.83	0.82	0.94	1.18	0.55	0.8	0.43	-0.04	0.22	0.45	-0.47	0.54	0.87	0.17	0.61	0.46	0.09	1.24	0.01	0.56	0.81	0.94	0.75
145	0.25	3.11	0.34	-0.06	2.21	2.43	2.15	1.79	0.88	1.48	0.85	0.45	-0.2	0.2	-0.83	1.03	1.09	-0.82	-0.27	-0.17	-0.59	2.09	-0.4	-0.02	0.24	0	1.19
146	0.17	2.48	0.2	0.65	1.16	1.3	1.1	1.65	1.07	0.9	0.49	-0.06	-0.07	0.47	-0.31	0.39	0.78	0.02	0.23	0.4	0.02	1.51	0.19	0.47	0.73	1.19	0.86
147	-0.14	1.99	0.32	0.83	0.74	0.5	1.18	0.79	0.99	0.93	0.2	-0.11	0.2	-0.1	-0.55	0.08	0.95	-0.09	0.05	0.31	-0.05	1.07	0.09	0.34	0.11	0.31	0.88
148	46.363	48.796	46.363	46.169	41.411	41.935	44.504	50.996	37.884	40.977	43.269	55.596	51.111	23.97	26.87	26.667	29.76	37.197	20.876	32.783	33.25	35.684	33.25	33.057	28.298	28.823	31.392
149	1	0	1	0	1	1	0	0	1	1	1	0	0	1	1	1	1	1	1	0	1	1	1	1	1	1	1
150	0	0	0	0	0	0	0	0	0	0	0	0	0	0	0	0	0	0	0	0	0	0	0	0	0	0	0
151	0	1	0	1	0	0	1	1	0	0	0	1	1	0	0	0	0	0	0	2	0	0	0	0	0	0	0
152	2	3	2	3	2	2	3	3	2	2	2	3	3	2	2	2	2	2	2	4	2	2	2	2	2	2	2
153	1	1	1	1	2	1	2	1	2	2	1	1	2	2	2	2	2	2	2	4	2	2	2	2	3	2	3
154	2	2	2	2	2	2	2	2	2	2	2	2	2	2	2	4	4	2	2	2	2	2	2	2	2	2	2
155	0	0	0	0	0	0	0	0	0	0	0	9	6	0	0	0	0	6	0	5	0	0	0	0	0	0	0
156	4	5	4	4	4	4	4	7	4	4	4	4	4	1	1	1	1	1	1	1	1	2	1	1	1	1	1
157	46	49	46	44	41	41	44	51	38	41	43	51	48	24	25	26	29	34	21	31	33	36	33	31	28	28	31
158	1	1	1	1	2	0	2	1	2	2	1	2	2	2	2	2	2	2	2	4	2	2	2	2	3	1	3
159	2	2	2	2	4	1	4	2	4	4	2	3	4	4	4	4	4	4	4	8	4	4	4	4	6	3	6
160	20	21	20	20	20	19	21	23	18	19	19	26	24	12	13	15	16	18	11	17	15	16	15	15	15	14	16
161	9	9	9	9	6	8	7	8	6	7	8	14	12	4	4	4	5	10	3	6	7	7	7	7	4	6	5
162	72.488	77.231	72.488	75.758	70.692	67.134	74.61	82.913	64.844	68.723	68.872	83.446	79.826	41.51	47.567	46.996	51.266	55.954	37.41	55.748	52.877	58.395	52.877	55.58	50.484	47.303	54.612
163	1.5758	1.5761	1.5758	1.7218	1.7242	1.6374	1.6957	1.6258	1.7064	1.6762	1.6017	1.6362	1.663	1.7296	1.9027	1.8076	1.7678	1.6457	1.7814	1.7983	1.6023	1.6221	1.6023	1.7929	1.803	1.6894	1.7617
164	12	12	12	11	10	11	11	12	9	10	11	17	15	6	6	7	8	12	5	9	9	9	9	8	7	8	8
165	26	28	26	24	21	22	23	28	20	22	24	25	24	12	12	11	13	16	10	14	18	20	18	16	13	14	15
166	5	6	5	5	6	5	6	8	5	5	5	6	5	2	2	2	2	2	2	4	2	3	2	2	3	2	3
167	3	3	3	3	4	3	4	3	4	4	3	3	4	4	4	6	6	4	4	4	4	4	4	4	5	4	5
168	0	0	0	1	0	0	0	0	0	0	0	0	0	0	1	0	0	0	0	0	0	0	0	1	0	0	0
169	4.1213	4.0085	4.0527	3.9858	4.0545	2.3289	4.0693	4.0458	3.8469	4.0106	4.0106	1.7768	2.3461	3.7702	3.9466	4.1724	4.1301	2.4398	3.4549	2.4241	4.2935	4.0233	4.1677	4.0446	4.1724	2.5798	4.1301
170	-2.6892	-2.6692	-2.6821	-2.6358	-2.624	-2.6271	-2.6333	-2.6487	-2.6234	-2.6326	-2.6621	-2.6254	-2.6263	-2.5484	-2.5508	-2.5557	-2.5951	-2.562	-2.5139	-2.5526	-2.6832	-2.6617	-2.6762	-2.5987	-2.5512	-2.5501	-2.5902
171	-0.4813	-0.5367	-0.5249	-0.5008	-0.5388	-0.5253	-0.543	-0.5402	-0.5391	-0.5165	-0.487	-0.5495	-0.5795	-0.5436	-0.5477	-0.5491	-0.5491	-0.5477	-0.5745	-0.5477	-0.4984	-0.5335	-0.5269	-0.5279	-0.5477	-0.5477	-0.5477
172	0.3885	0.3904	0.3903	0.3754	0.4348	0.4666	0.4401	0.4396	0.492	0.4636	0.3886	0.5766	0.598	0.4453	0.5381	0.4848	0.4892	0.5844	0.4967	0.5503	0.4246	0.4409	0.4385	0.392	0.4575	0.5294	0.465
173	2.6914	2.6869	2.6866	2.6803	2.6794	2.7377	2.6816	2.6841	2.678	2.6824	2.6855	2.678	2.6779	2.6114	2.6145	2.616	2.6256	2.6166	2.6009	2.618	2.6626	2.6567	2.652	2.6262	2.6217	2.725	2.6312
174	-3.3087	-3.3087	-3.3086	-3.3086	-3.3086	-3.3066	-3.3086	-3.3086	-3.3088	-3.3088	-3.3087	-3.3087	-3.3087	-3.3174	-3.3175	-3.3175	-3.3174	-3.3175	-3.3172	-3.3175	-3.3175	-3.3175	-3.3175	-3.3175	-3.3175	-3.3154	-3.3174
175	-0.4484	-0.4511	-0.4505	-0.4238	-0.5326	-0.4994	-0.5265	-0.5162	-0.592	-0.5385	-0.4489	-0.5193	-0.5804	-0.5412	-0.6759	-0.6258	-0.6072	-0.5381	-0.5658	-0.5627	-0.4831	-0.5206	-0.4824	-0.456	-0.574	-0.5514	-0.5643
176	0.338	0.3381	0.3381	0.3779	0.3733	0.3749	0.3747	0.3852	0.4284	0.4031	0.338	0.6744	0.5744	0.3863	0.4771	0.4292	0.4319	0.5801	0.4168	0.486	0.38	0.3849	0.3847	0.3979	0.391	0.4537	0.393
177	2.6837	2.665	2.6896	2.588	2.5846	2.689	2.5883	2.6017	2.584	2.5847	2.6175	2.5847	2.5846	2.3636	2.3521	2.3677	2.5345	2.4279	2.2358	2.3851	2.6829	2.6639	2.6889	2.5446	2.3768	2.6837	2.5394
178	-2.4484	-2.4462	-2.4432	-2.427	-2.4247	-2.4266	-2.4276	-2.4321	-2.4246	-2.4281	-2.436	-2.4247	-2.4249	-2.3471	-2.3457	-2.3484	-2.363	-2.35	-2.337	-2.3492	-2.432	-2.4351	-2.4254	-2.3606	-2.3493	-2.3505	-2.3644
179	-0.2613	-0.2642	-0.2641	-0.1761	-0.319	-0.2441	-0.3244	-0.316	-0.3639	-0.3147	-0.2615	-0.4138	-0.4514	-0.3205	-0.3994	-0.3663	-0.3664	-0.4289	-0.3574	-0.4288	-0.2947	-0.3151	-0.3115	-0.2639	-0.3465	-0.3636	-0.3545
180	0.4821	0.4854	0.4852	0.4751	0.5746	0.5125	0.5867	0.5525	0.6152	0.5641	0.4823	0.7315	0.7501	0.5574	0.6818	0.6163	0.6172	0.7239	0.5979	0.6931	0.5291	0.5513	0.5471	0.4854	0.6191	0.6434	0.6372
181	2.9169	2.9002	2.907	2.891	2.8789	2.9816	2.8864	2.8945	2.8747	2.8842	2.8998	2.8786	2.878	2.8006	2.8223	2.8162	2.8431	2.8201	2.7698	2.8174	2.9045	2.8825	2.8921	2.8613	2.8229	2.9743	2.8484
182	29.977	30.844	29.977	30.071	24.809	26.925	26.996	31.504	23.418	25.605	27.791	29.544	27.791	15.332	17.612	16.155	18.342	19.705	13.146	19.499	21.892	22.758	21.892	21.985	16.724	18.839	18.91
183	9	11	9	10	9	7	10	11	8	8	8	9	9	4	5	6	7	6	4	6	6	8	6	7	6	4	7
184	0.4737	0.55	0.4737	0.5263	0.4737	0.3684	0.5	0.5	0.4706	0.4444	0.4444	0.3333	0.375	0.3636	0.4167	0.4286	0.4667	0.3333	0.4	0.3529	0.4286	0.5333	0.4286	0.5	0.4286	0.2857	0.4667
185	0	0	0	0	0	0	0	0	0	0	0	10	6	0	0	0	0	6	0	5	0	0	0	0	0	0	0
186	45	48	45	43	40	41	43	50	37	40	42	52	48	23	24	25	28	34	20	31	32	35	32	30	27	28	30
187	3	3	3	3	4	3	4	4	3	3	3	3	3	2	2	3	3	2	2	2	2	2	2	2	3	2	3
188	19	20	19	19	19	19	20	22	17	18	18	27	24	11	12	14	15	18	10	17	14	15	14	14	14	14	15
189	7	8	7	7	7	7	7	7	7	7	7	7	7	5	5	5	5	5	5	5	6	8	6	7	5	5	5
190	11	13	11	12	11	8	12	14	10	10	10	11	11	5	6	7	8	7	5	7	7	9	7	8	7	4	8
191	0.5789	0.65	0.5789	0.6316	0.5789	0.4211	0.6	0.6364	0.5882	0.5556	0.5556	0.4074	0.4583	0.4545	0.5	0.5	0.5333	0.3889	0.5	0.4118	0.5	0.6	0.5	0.5714	0.5	0.2857	0.5333
192	42	45	42	40	36	38	39	46	34	37	39	39	39	21	22	22	25	26	18	24	30	33	30	28	24	26	27
193	15.707	16.251	15.707	15.544	15.707	14.414	16.414	17.828	14.129	15	15	19.104	18.113	9.7236	10.431	12.008	12.715	13.544	8.8534	12.836	12.008	12.552	12.008	11.845	12.008	10.715	12.715
194	12.147	12.398	12.147	12.502	11.055	10.931	11.762	13.139	10.017	10.887	11.44	14.456	13.326	6.7409	7.7896	7.7645	8.4716	9.8347	5.8706	9.0499	9.0253	9.2764	9.0253	9.3798	7.9336	7.8094	8.6407
195	8.0605	7.6044	8.0605	7.1902	5.9831	6.8973	6.6902	7.3973	5.4831	6.3534	7.3534	9.8699	8.7925	3.8618	3.5689	4.0689	4.776	6.9557	2.9916	5.2236	6.1463	5.6902	6.1463	5.276	4.0689	4.9831	4.776
196	8.2925	7.8365	8.2925	7.4223	6.2925	7.1294	6.9996	7.7067	5.7152	6.5854	7.5854	10.983	9.6983	4.0165	3.7236	4.301	5.0081	7.8365	3.1463	5.7152	6.301	5.8449	6.301	5.4307	4.301	5.1378	5.0081
197	9.2898	9.8791	9.235	9.3791	9.235	8.9304	9.735	10.735	8.3791	8.7518	8.7518	12.38	11.291	5.4853	6.0233	6.8791	7.3791	8.5409	5.0746	8.0409	6.934	7.5233	6.8791	7.0233	6.8791	6.5746	7.3791
198	6.7518	7.134	6.697	7.7248	5.9804	6.3934	6.4804	7.315	5.4503	5.8947	6.2138	8.3923	7.5326	3.4385	4.2181	4.0312	4.5312	5.5336	2.9909	4.9708	4.8872	5.2694	4.8323	5.8601	4.1157	4.5287	4.6157
199	4.0116	3.8938	3.9568	2.8938	2.7474	3.3938	3.2474	3.3938	2.3938	2.8963	3.4736	5.1134	4.5688	1.5629	1.3938	1.7474	2.2474	3.6581	1.0505	2.0361	3.0116	2.8938	2.9568	1.8938	1.7474	2.3938	2.2474
200	4.101	3.9832	4.0461	2.9832	2.8914	3.4832	3.3914	3.4832	2.4832	2.9856	3.5629	6.2828	5.5244	1.6523	1.4832	1.8914	2.3914	4.7079	1.1498	2.2997	3.101	2.9832	3.0461	1.9832	1.8914	2.4832	2.3914
201	3	2	2	2	2	2	2	2	2	3	2	2	2	2	2	2	2	2	1	2	3	2	2	2	2	2	2
202	3	2	2	2	2	2	2	2	2	3	2	2	2	2	2	2	2	2	1	2	3	2	2	2	2	2	2
203	0.7374	0.7431	0.7374	0.7877	0.808	0.7685	0.7931	0.7664	0.8039	0.7879	0.7483	0.756	0.7669	0.8376	0.899	0.913	0.8817	0.7755	0.8722	0.8366	0.7696	0.7753	0.7696	0.8392	0.8747	0.819	0.848
204	12	14	12	13	12	11	13	15	11	11	11	15	15	7	7	8	9	10	7	9	8	10	8	9	8	7	9
205	1	2	1	1	1	1	1	2	1	1	1	1	1	0	0	-1	-1	0	0	0	0	1	0	0	0	0	0

Table S12 Amino acid descriptors (2D) used in this study.

	P	Q	Q	Q	Q	Q	Q	Q	Q	Q	Q	Q	Q	Q	Q	Q	Q	Q	Q	Q	Q	R	R	R	R	R	R	R	R	R	R	R	R	R	R	R	R	R	R	R	R	S	S	S	S	S	S	S	S	S	S	S	S	S	S
	Y	A	C	D	E	F	G	H	I	K	L	M	N	P	Q	R	S	T	V	W	Y	A	C	D	E	F	G	H	I	K	L	M	N	P	Q	R	S	T	V	W	Y	A	C	D	E	F	G	H	I	K	L	M	N	P	Q
No.	PY	QA	QC	QD	QE	QF	QG	QH	QI	QK	QL	QM	QN	QP	QQ	QR	QS	QT	QV	QW	QY	RA	RC	RD	RE	RF	RG	RH	RI	RK	RL	RM	RN	RP	RQ	RR	RS	RT	RV	RW	RY	SA	SC	SD	SE	SF	SG	SH	SI	SK	SL	SM	SN	SP	SQ
206	-0.8635	-0.8476	-0.847	-0.8505	-0.8543	-0.8473	-0.8483	-0.8488	-0.8474	-0.863	-0.8476	-0.8474	-0.8472	-0.8516	-0.8476	-0.8475	-0.8465	-0.8464	-0.8473	-0.863	-0.8473	-0.8476	-0.847	-0.8505	-0.8543	-0.8473	-0.8483	-0.8488	-0.8474	-0.863	-0.8476	-0.8474	-0.8471	-0.8516	-0.8476	-0.8475	-0.8465	-0.8464	-0.8473	-0.863	-0.8473	-0.8455	-0.8445	-0.8505	-0.8543	-0.8451	-0.8466	-0.8486	-0.8452	-0.863	-0.8456	-0.8452	-0.8449	-0.8503	-0.8455
207	-0.4225	-0.4221	-0.4781	-0.5113	-0.5051	-0.39	-0.4712	-0.4014	-0.3831	-0.3831	-0.3866	-0.3787	-0.4826	-0.4821	-0.4818	-0.4053	-0.4821	-0.4631	-0.3873	-0.4399	-0.4496	-0.3916	-0.3982	-0.4525	-0.4496	-0.3892	-0.4173	-0.3953	-0.3349	-0.368	-0.3588	-0.3608	-0.4022	-0.3867	-0.4019	-0.3841	-0.3951	-0.3867	-0.34	-0.395	-0.3947	-0.4212	-0.4746	-0.514	-0.5067	-0.39	-0.4701	-0.4013	-0.3815	-0.383	-0.3865	-0.3787	-0.4797	-0.4792	-0.4789
208	0.144	0.1102	0.1855	0.1098	0.0905	0.0793	0.1427	0.18	0.0894	0.1849	0.0744	0.0722	0.0866	0.1152	0.0735	0.1555	0.1855	0.1635	0.0958	0.1474	0.1512	0.1767	0.1822	0.1448	0.1253	0.1495	0.1788	0.1797	0.1253	0.1849	0.0955	0.1494	0.1497	0.1607	0.1494	0.179	0.1825	0.1736	0.1439	0.1792	0.1795	0.1856	0.1856	0.1667	0.157	0.1856	0.1856	0.1856	0.1458	0.1852	0.1158	0.1856	0.1856	0.1856	0.1856
209	2.6139	2.426	2.4402	2.453	2.5005	2.5574	2.3664	2.5227	2.5981	2.6045	2.5806	2.5151	2.4834	2.61	2.5268	2.5968	2.4416	2.4982	2.5443	2.6252	2.5658	2.5274	2.5386	2.5488	2.5864	2.6317	2.4807	2.6038	2.6659	2.6693	2.6506	2.5982	2.5726	2.6788	2.6072	2.6635	2.5397	2.5845	2.6217	2.686	2.6383	2.2811	2.3006	2.3179	2.3825	2.4593	2.1995	2.4134	2.5079	2.5211	2.4882	2.4021	2.3597	2.5134	2.4182
210	-2.2465	-2.2351	-2.2351	-2.2351	-2.2351	-2.2351	-2.2351	-2.2351	-2.2351	-2.2354	-2.2351	-2.2351	-2.2351	-2.2351	-2.2351	-2.2351	-2.2351	-2.2351	-2.2351	-2.2351	-2.2351	-2.2352	-2.2352	-2.2352	-2.2352	-2.2352	-2.2351	-2.2352	-2.2352	-2.2354	-2.2352	-2.2352	-2.2352	-2.2352	-2.2352	-2.2352	-2.2352	-2.2352	-2.2352	-2.2352	-2.2352	-2.2351	-2.2351	-2.2351	-2.2351	-2.2351	-2.2351	-2.2351	-2.2351	-2.2354	-2.2351	-2.2351	-2.2351	-2.2352	-2.2351
211	-0.4031	-0.4202	-0.4507	-0.5044	-0.4647	-0.4011	-0.4785	-0.4168	-0.3658	-0.3556	-0.361	-0.3535	-0.4679	-0.4051	-0.448	-0.407	-0.4728	-0.4671	-0.3778	-0.3806	-0.4033	-0.3949	-0.4384	-0.4815	-0.4532	-0.3774	-0.458	-0.3884	-0.3313	-0.3317	-0.3312	-0.3453	-0.428	-0.3804	-0.4095	-0.3695	-0.449	-0.4311	-0.3317	-0.358	-0.3835	-0.4888	-0.4583	-0.5973	-0.5335	-0.4486	-0.5521	-0.4554	-0.4327	-0.4122	-0.4239	-0.4455	-0.529	-0.4607	-0.4625
212	0.129	0.0542	0.0542	0.0542	0.0542	0.0581	0.0542	0.0542	0.0542	0.0542	0.0542	0.0542	0.0542	0.0542	0.0542	0.0542	0.0542	0.0542	0.0542	0.1015	0.0568	0.0542	0.0568	0.0551	0.0555	0.1116	0.0542	0.0569	0.0542	0.0542	0.0542	0.0567	0.0542	0.0542	0.0542	0.0555	0.0542	0.0542	0.0542	0.1296	0.0936	0.0542	0.0542	0.0542	0.0542	0.0581	0.0542	0.0542	0.0542	0.0542	0.0542	0.0542	0.0542	0.0542	0.0542
213	2.5259	2.2676	2.2813	2.288	2.3634	2.474	2.1958	2.3844	2.5146	2.5003	2.501	2.3993	2.3116	2.5028	2.3866	2.459	2.2616	2.3323	2.4314	2.5969	2.4958	2.3699	2.3806	2.3862	2.4441	2.5307	2.3147	2.4601	2.5679	2.5541	2.5547	2.4717	2.4047	2.5636	2.4625	2.5211	2.3663	2.4206	2.4985	2.6343	2.5482	2.0524	2.0778	2.0865	2.2157	2.3829	1.9158	2.2513	2.4257	2.4129	2.4129	2.2733	2.1268	2.3893	2.2507
214	-0.639	-0.6409	-0.6409	-0.6409	-0.6409	-0.6409	-0.6409	-0.6409	-0.6409	-0.6419	-0.6409	-0.6409	-0.6409	-0.641	-0.6409	-0.6409	-0.6409	-0.6409	-0.6409	-0.6409	-0.6409	-0.641	-0.641	-0.641	-0.641	-0.641	-0.641	-0.641	-0.641	-0.6419	-0.641	-0.641	-0.641	-0.6411	-0.641	-0.641	-0.641	-0.641	-0.641	-0.641	-0.641	-0.6407	-0.6407	-0.6407	-0.6407	-0.6407	-0.6407	-0.6407	-0.6407	-0.6418	-0.6407	-0.6407	-0.6407	-0.6408	-0.6407
215	-0.2287	-0.212	-0.1995	-0.2217	-0.221	-0.2161	-0.2209	-0.2201	-0.1985	-0.208	-0.2048	-0.1969	-0.232	-0.2251	-0.2324	-0.221	-0.2328	-0.2215	-0.2002	-0.2247	-0.2294	-0.2003	-0.1869	-0.2105	-0.2097	-0.2112	-0.2095	-0.2137	-0.1857	-0.196	-0.193	-0.1851	-0.2213	-0.2057	-0.2215	-0.2098	-0.2225	-0.2098	-0.1878	-0.2149	-0.2238	-0.2138	-0.2007	-0.2234	-0.2225	-0.2161	-0.2281	-0.2201	-0.1994	-0.2093	-0.2058	-0.1981	-0.2333	-0.2281	-0.2337
216	0.1803	0.1307	0.1622	0.1449	0.1449	0.165	0.152	0.163	0.116	0.128	0.1265	0.1612	0.16	0.1552	0.1612	0.1449	0.1607	0.1406	0.1171	0.1691	0.1833	0.1127	0.1457	0.1282	0.1283	0.1627	0.1244	0.1464	0.1027	0.1112	0.1109	0.145	0.1441	0.1197	0.145	0.1283	0.1445	0.1279	0.1034	0.1664	0.1741	0.1304	0.1614	0.1443	0.1443	0.1642	0.1523	0.1622	0.1159	0.1277	0.1262	0.1605	0.1593	0.1543	0.1605
217	2.825	2.6131	2.6421	2.6523	2.7039	2.7803	2.5449	2.73	2.8027	2.7952	2.7838	2.728	2.6781	2.815	2.726	2.7941	2.6279	2.6901	2.7446	2.8743	2.7892	2.7068	2.7293	2.7378	2.7793	2.8411	2.6535	2.8	2.8618	2.8543	2.8451	2.7986	2.7585	2.8755	2.7973	2.8535	2.7186	2.7684	2.813	2.9196	2.8485	2.465	2.5074	2.5205	2.5908	2.6934	2.3668	2.6273	2.716	2.7106	2.6949	2.624	2.5559	2.7211	2.6204
218	16.372	15	16	18	19	19.048	14	18.05	18	19	18	18	18	15.059	19	21	16	17	17	20.314	20.045	17	18	20	21	21.043	16	20.045	20	21	20	20	20	17.053	21	23	18	19	19	22.291	22.042	12	13	15	16	16.056	11	15.059	15	16	15	15	15	12.071	16
219	7.852	7.3025	8.144	8.9917	9.8336	9.904	7.3125	9.1065	8.9917	10.748	8.9917	9.8685	8.9917	6.8053	9.8336	11.552	8.144	8.1633	8.1633	9.6298	9.9881	9	9.8685	10.688	11.552	11.536	9.0741	10.714	10.688	12.535	10.688	11.637	10.688	8.3232	11.552	13.309	9.8685	9.8336	9.8336	11.111	11.584	5.6122	6.4533	7.3025	8.144	8.2268	5.625	7.438	7.3025	9.0741	7.3025	8.1869	7.3025	5.1856	8.144
220	5.0579	6.9758	7.0582	8.7075	9.5207	7.7051	8.0816	7.0255	7.259	9.5207	8.7075	8.7075	8.7075	4.3018	9.5207	11.25	7.0582	7.1111	7.1111	6.0464	7.9084	8.687	8.7075	10.412	11.25	9.1571	9.9531	8.4444	8.8128	11.25	10.412	10.412	10.412	5.4792	11.25	13.018	8.7075	8.7108	8.7108	7.165	9.3333	4.5918	4.6875	6.2222	7.0582	5.6805	5.2893	5.0176	5.04	7.0582	6.2222	6.2222	6.2222	2.75	7.0582
221	12.739	11.43	13.791	13.613	14.6	13.914	10.442	13.073	14.404	15.397	14.404	15.79	13.178	11.88	14.161	15.694	12.421	13.412	13.412	14.561	14.871	12.963	15.309	15.146	16.132	15.438	11.976	14.595	15.933	16.925	15.933	17.305	14.712	13.411	15.694	17.228	13.952	14.942	14.942	16.063	16.397	9.7128	12.164	11.882	12.876	12.119	8.7152	11.276	12.708	13.707	12.708	14.167	11.43	10.096	12.421
222	6.023	5.4465	6.9454	6.6788	7.4369	7.1111	5.3185	6.4734	7.0939	8.6008	7.0939	8.586	6.451	5.2828	7.1991	8.4974	6.206	6.337	6.337	6.7943	7.2959	6.7365	8.308	7.9655	8.7483	8.3342	6.6485	7.6734	8.405	9.9859	8.405	9.9877	7.7235	6.4515	8.4974	9.8273	7.5252	7.6222	7.6222	7.8935	8.498	4.4465	6.0023	5.6798	6.4512	6.0983	4.3415	5.46	6.1076	7.6874	6.1076	7.6989	5.4465	4.2725	6.206
223	3.8391	5.1118	5.9713	6.3818	7.1108	5.4594	5.7653	4.9262	5.6668	7.544	6.7976	7.5299	6.1539	3.2966	6.8729	8.1746	5.304	5.4494	5.4494	4.2176	5.7121	6.4062	7.2753	7.67	8.4262	6.5387	7.1762	5.9756	6.8653	8.8828	8.1112	8.8835	7.4272	4.1991	8.1746	9.5088	6.5598	6.6758	6.6758	5.0383	6.7784	3.5772	4.3379	4.7675	5.525	4.1544	3.9874	3.625	4.1712	5.9298	5.1496	5.8307	4.5596	2.2398	5.304
224	3.8365	4.1504	5.9867	5.051	5.7148	4.7115	3.9668	4.2313	5.6767	6.97	5.6767	7.5319	4.7227	3.6919	5.3656	6.3505	4.8176	4.9995	4.9995	4.1222	4.9317	5.1369	7.0661	6.0323	6.7205	5.594	4.9764	5.0908	6.6959	8.0483	6.6959	8.642	5.6812	4.5536	6.3505	7.3609	5.8329	5.9944	5.9944	4.8768	5.8061	3.599	5.6161	4.4992	5.1916	4.106	3.4397	3.6217	5.1742	6.5855	5.1742	7.2715	4.1504	3.0811	4.8176
225	6	7	7	9	9	7	7	9	7	8	7	7	9	7	9	10	8	8	7	8	8	8	8	10	10	8	8	10	8	9	8	8	10	8	10	11	9	9	8	9	9	6	6	8	8	6	6	8	6	7	6	6	8	6	8
226	4	6	6	6	6	6	6	7	6	9	6	6	8	5	8	11	7	7	6	7	7	9	9	9	9	9	9	10	9	12	9	9	11	8	11	14	10	10	9	10	10	5	5	5	5	5	5	6	5	8	5	5	7	4	7
227	1	1	1	1	1	1	1	1	1	1	1	1	1	1	1	1	1	1	1	1	1	1	1	1	1	1	1	1	1	1	1	1	1	1	1	0	1	1	1	1	1	1	1	1	1	1	1	1	1	1	1	1	1	1	1
228	0	1	1	1	1	1	1	1	1	1	1	1	1	0	1	1	1	1	1	1	1	1	1	1	1	1	1	1	1	1	1	1	1	1	1	2	1	1	1	1	1	0	0	0	0	0	0	1	0	1	0	0	1	0	1
229	0.994	-1.989	-1.879	-2.616	-2.174	-0.454	-2.451	-2.758	-0.575	-1.92	-0.575	-1.334	-3.471	-1.601	-3.029	-2.341	-3.024	-2.562	-1.017	-0.088	-0.762	-1.301	-1.191	-1.928	-1.486	0.234	-1.763	-2.07	0.113	-1.232	0.113	-0.646	-2.783	-0.913	-2.341	-1.653	-2.336	-1.874	-0.329	0.6	-0.074	-1.984	-1.874	-2.611	-2.169	-0.449	-2.446	-2.753	-0.57	-1.915	-0.57	-1.329	-3.466	-1.596	-3.024
230	-1.7818	-0.3758	-1.0371	-0.1868	-0.3886	-1.878	-0.0486	-0.6351	-1.2946	-0.3209	-1.608	-1.2423	-0.2091	-0.495	-0.4109	-0.7483	0.1539	-0.1733	-0.7794	-2.1679	-1.516	-0.7133	-1.3745	-0.5243	-0.726	-2.2154	-0.3861	-0.9726	-1.632	-0.6583	-1.9455	-1.5797	-0.5465	-0.8324	-0.7483	-1.0858	-0.1835	-0.5107	-1.1168	-2.5053	-1.8535	0.189	-0.4723	0.378	0.1762	-1.3132	0.5162	-0.0703	-0.7298	0.2439	-1.0433	-0.6775	0.3557	0.0698	0.1539
231	7.3685	5.2319	5.9873	5.8763	6.3571	7.7854	4.7795	7.005	6.6391	6.9906	6.629	6.9168	6.06	5.9838	6.5391	7.4707	5.3883	5.8317	6.1589	8.8289	7.9105	6.1607	6.9207	6.8286	7.307	8.7202	5.71	7.9263	7.5653	7.9082	7.5553	7.8475	6.9933	6.9134	7.4707	8.3797	6.3275	6.7693	7.0857	9.7593	8.8545	4.0797	4.8302	4.7153	5.1981	6.6361	3.6254	5.8605	5.489	5.844	5.4789	5.7624	4.9077	4.8337	5.3883
232	11	0	0	0	0	6	0	5	0	0	0	0	0	5	0	1	0	0	0	10	6	1	1	1	1	7	1	6	1	1	1	1	1	6	1	2	1	1	1	11	7	0	0	0	0	6	0	5	0	0	0	0	0	5	0
233	1	0	0	0	0	1	0	0	1	0	1	1	0	1	0	0	0	0	1	1	1	0	0	0	0	0	0	0	0	0	0	0	0	0	0	0	0	0	0	0	0	1	1	1	1	1	1	0	1	0	1	1	0	1	0
234	2	0	0	0	0	1	0	1	0	0	0	0	0	1	0	0	0	0	0	2	1	0	0	0	0	1	0	1	0	0	0	0	0	1	0	0	0	0	0	2	1	0	0	0	0	1	0	1	0	0	0	0	0	1	0
235	6	6	7	8	9	8	6	8	8	10	8	9	8	6	9	11	7	7	7	8	8	8	9	10	11	10	8	10	10	12	10	11	10	8	11	13	9	9	9	10	10	4	5	6	7	6	4	6	6	8	6	7	6	4	7
236	0	2	2	3	3	1	2	3	1	2	1	1	3	0	3	4	2	2	1	1	1	2	2	3	4	2	2	3	2	4	2	3	3	2	4	4	3	3	2	3	3	1	1	1	1	0	1	2	0	2	0	0	2	1	2
237	5.282	5.323	5.733	6.229	6.229	6.217	5.323	6.19	5.323	7.176	5.323	5.783	6.693	5.253	6.693	9.101	6.003	6.003	5.323	6.557	6.599	7.731	8.141	8.637	8.637	8.625	7.731	8.598	7.731	9.584	7.731	8.191	9.101	7.661	9.101	11.509	8.411	8.411	7.731	8.965	9.007	4.633	5.043	5.539	5.539	5.527	4.633	5.5	4.633	6.486	4.633	5.093	6.003	4.563	6.003
238	-5.282	-5.323	-5.733	-7.229	-7.229	-6.217	-5.323	-6.19	-5.323	-6.176	-5.323	-5.783	-6.693	-5.253	-6.693	-8.101	-6.003	-6.003	-5.323	-6.558	-6.599	-6.731	-7.141	-8.637	-8.637	-7.625	-6.731	-7.598	-6.731	-7.584	-6.731	-7.191	-8.101	-6.661	-8.101	-9.509	-7.411	-7.411	-6.731	-7.966	-8.007	-4.633	-5.043	-6.539	-6.539	-5.527	-4.633	-5.5	-4.633	-5.486	-4.633	-5.093	-6.003	-4.563	-6.003
239	2.6262	2.7392	2.8745	2.7986	2.8324	3.048	2.7028	3.2905	2.894	4.1362	2.8942	2.9043	3.3422	2.7439	3.3617	4.6039	3.0897	3.1231	2.8412	3.2906	3.2814	3.9722	4.1075	4.0316	4.0653	4.281	3.9358	4.5235	4.127	5.3692	4.1271	4.1373	4.5751	3.9768	4.5946	5.8369	4.3227	4.3561	4.0742	4.5236	4.5144	2.4825	2.6178	2.5419	2.5756	2.7912	2.4461	3.0338	2.6373	3.8795	2.6374	2.6476	3.0854	2.4871	3.1049
240	-2.6262	-2.7392	-2.8745	-3.7986	-3.8324	-3.048	-2.7028	-3.2905	-2.894	-3.1362	-2.8942	-2.9043	-3.3422	-2.7439	-3.3617	-3.6039	-3.0897	-3.1231	-2.8412	-3.2906	-3.2814	-2.9722	-3.1075	-4.0316	-4.0653	-3.281	-2.9358	-3.5235	-3.127	-3.3692	-3.1271	-3.1373	-3.5751	-2.9768	-3.5946	-3.8369	-3.3227	-3.3561	-3.0742	-3.5236	-3.5144	-2.4825	-2.6178	-3.5419	-3.5756	-2.7912	-2.4461	-3.0338	-2.6373	-2.8795	-2.6374	-2.6476	-3.0854	-2.4871	-3.1049
241	0.1322	0.1261	0.1202	0.1235	0.122	0.1134	0.1278	0.105	0.1194	0.0835	0.1194	0.119	0.1034	0.1259	0.1028	0.0751	0.1118	0.1106	0.1216	0.105	0.1053	0.087	0.0841	0.0857	0.085	0.0807	0.0878	0.0764	0.0837	0.0644	0.0837	0.0835	0.0755	0.0869	0.0752	0.0592	0.0799	0.0793	0.0848	0.0764	0.0765	0.1392	0.132	0.136	0.1342	0.1238	0.1413	0.1139	0.131	0.0891	0.131	0.1305	0.112	0.139	0.1113
242	0.2085	0.1999	0.1905	0.1447	0.1435	0.1797	0.2027	0.1664	0.1892	0.1746	0.1892	0.1886	0.1639	0.1996	0.1629	0.152	0.1772	0.1753	0.1928	0.1664	0.1669	0.1843	0.1762	0.1364	0.1352	0.1669	0.1867	0.1554	0.1751	0.1626	0.1751	0.1746	0.1532	0.184	0.1524	0.1427	0.1648	0.1632	0.1782	0.1554	0.1558	0.2206	0.2092	0.1552	0.1538	0.1962	0.224	0.1805	0.2077	0.1902	0.2077	0.2069	0.1775	0.2202	0.1764
243	42.995	47.26	53.672	66.405	66.234	42.995	34.464	68.442	72.853	60.056	72.853	64.322	42.995	73.249	60.227	51.526	34.464	47.26	64.322	68.649	42.995	38.559	44.971	57.703	57.533	34.294	25.763	59.741	64.151	51.355	64.151	55.621	34.294	64.548	51.526	42.824	25.763	38.559	55.621	59.948	34.294	12.796	19.208	31.941	31.77	8.5308	0	33.978	38.388	25.592	38.388	29.858	8.5308	38.785	25.763
244	69.363	22.12	22.12	22.12	22.12	43.447	36.432	54.322	22.12	43.843	22.12	22.12	30.821	22.12	22.12	43.843	45.602	31.29	22.12	43.447	47.64	43.843	43.843	43.843	43.843	65.17	58.155	76.045	43.843	65.566	43.843	43.843	52.544	43.843	43.843	65.566	67.325	53.013	43.843	65.17	69.363	30.651	30.651	30.651	30.651	51.978	44.962	62.852	30.651	52.374	30.651	30.651	39.352	30.651	30.651
245	4.2654	4.2654	12.046	4.2654	4.2654	4.2654	4.2654	8.5308	4.2654	4.2654	4.2654	4.2654	4.2654	4.2654	4.2654	4.2654	4.2654	4.2654	4.2654	4.2654	4.2654	4.2654	12.046	4.2654	4.2654	4.2654	4.2654	8.5308	4.2654	4.2654	4.2654	4.2654	4.2654	4.2654	4.2654	4.2654	4.2654	4.2654	4.2654	4.2654	4.2654	19.217	26.998	19.217	19.217	19.217	19.217	23.482	19.217	19.217	19.217	19.217	19.217	19.217	19.217
246	11.765	29.003	29.003	29.003	29.003	29.003	29.003	37.622	29.003	29.003	29.003	29.003	46.241	20.384	46.241	29.003	29.003	29.003	29.003	37.622	29.003	11.765	11.765	11.765	11.765	11.765	11.765	20.384	11.765	11.765	11.765	11.765	29.003	3.146	29.003	11.765	11.765	11.765	11.765	20.384	11.765	11.765	11.765	11.765	11.765	11.765	11.765	20.384	11.765	11.765	11.765	11.765	29.003	3.146	29.003
247	10.324	12.95	12.95	12.95	12.95	12.95	12.95	12.95	12.95	12.95	12.95	12.95	25.899	12.95	25.899	12.95	23.274	23.274	12.95	12.95	23.274	0	0	0	0	0	0	0	0	0	0	0	12.95	0	12.95	0	10.324	10.324	0	0	10.324	10.324	10.324	10.324	10.324	10.324	10.324	10.324	10.324	10.324	10.324	10.324	23.274	10.324	23.274
248	12.95	12.95	12.95	12.95	12.95	12.95	12.95	12.95	12.95	12.95	12.95	12.95	12.95	12.95	12.95	56.045	12.95	12.95	12.95	12.95	12.95	56.045	56.045	56.045	56.045	56.045	56.045	56.045	56.045	56.045	56.045	56.045	56.045	56.045	56.045	99.14	56.045	56.045	56.045	56.045	56.045	12.95	12.95	12.95	12.95	12.95	12.95	12.95	12.95	12.95	12.95	12.95	12.95	12.95	12.95
249	17.238	25.857	25.857	25.857	25.857	25.857	25.857	25.857	25.857	51.714	25.857	25.857	25.857	25.857	25.857	41.539	25.857	25.857	25.857	25.857	25.857	41.539	41.539	41.539	41.539	41.539	41.539	41.539	41.539	67.396	41.539	41.539	41.539	41.539	41.539	57.22	41.539	41.539	41.539	41.539	41.539	25.857	25.857	25.857	25.857	25.857	25.857	25.857	25.857	51.714	25.857	25.857	25.857	25.857	25.857
250	35.42	18.748	0	0	17.403	10.91	0	0	0	26.104	8.7013	40.102	0	17.403	8.7013	17.403	0	18.748	0	23.165	35.42	27.449	8.7013	8.7013	26.104	19.611	8.7013	8.7013	8.7013	34.805	17.403	48.804	8.7013	26.104	17.403	26.104	8.7013	27.449	8.7013	31.866	44.121	18.748	0	0	17.403	10.91	0	0	0	26.104	8.7013	40.102	0	17.403	8.7013
251	24.51	0	0	0	0	61.275	0	0	46.197	0	37.495	0	0	0	0	0	0	0	37.495	36.765	24.51	0	0	0	0	61.275	0	0	46.197	0	37.495	0	0	0	0	0	0	0	37.495	36.765	24.51	0	0	0	0	61.275	0	0	46.197	0	37.495	0	0	0	0
252	0	0	26.363	0	0	0	0	0	0	0	0	19.761	0	0	0	0	0	0	0	0	0	0	26.363	0	0	0	0	0	0	0	0	19.761	0	0	0	0	0	0	0	0	0	0	26.363	0	0	0	0	0	0	0	0	19.761	0	0	0
253	0	0	0	0	0	0	0	5.6826	0	0	0	0	0	0	0	0	0	0	0	0	0	0	0	0	0	0	0	5.6826	0	0	0	0	0	0	0	0	0	0	0	0	0	0	0	0	0	0	0	5.6826	0	0	0	0	0	0	0
254	13.567	27.134	27.134	27.134	27.134	27.134	27.134	27.134	27.134	27.134	27.134	27.134	40.701	27.134	40.701	40.573	27.134	27.134	27.134	27.134	27.134	27.006	27.006	27.006	27.006	27.006	27.006	27.006	27.006	27.006	27.006	27.006	40.573	27.006	40.573	40.445	27.006	27.006	27.006	27.006	27.006	13.567	13.567	13.567	13.567	13.567	13.567	13.567	13.567	13.567	13.567	13.567	27.134	13.567	27.134
255	35.038	35.027	35.027	62.161	62.161	35.027	35.027	35.164	35.027	36.133	35.027	35.027	41.678	34.89	41.678	35.027	42.795	42.795	35.027	35.164	42.795	28.376	28.376	55.51	55.51	28.376	28.376	28.513	28.376	29.482	28.376	28.376	35.027	28.239	35.027	28.376	36.144	36.144	28.376	28.513	36.144	36.144	36.144	63.278	63.278	36.144	36.144	36.281	36.144	37.249	36.144	36.144	42.795	36.007	42.795
256	0.6788	0.5159	0.557	0.4634	0.4964	0.6275	0.4776	0.5852	0.6049	0.5368	0.6049	0.6119	0.4581	0.5471	0.4904	0.4396	0.4619	0.4973	0.5798	0.6522	0.582	0.4514	0.4912	0.4122	0.4435	0.5621	0.4153	0.522	0.5391	0.4825	0.5391	0.5461	0.409	0.4815	0.4396	0.4006	0.4079	0.4412	0.5139	0.5879	0.524	0.4852	0.5377	0.4262	0.468	0.6235	0.4345	0.5734	0.5967	0.5177	0.5967	0.6049	0.4213	0.5252	0.4619
257	0.3912	0.3438	0.3443	0.3397	0.381	0.4393	0.285	0.2355	0.3758	0.2938	0.3758	0.4158	0.3035	0.3162	0.3156	0.28	0.285	0.3377	0.3676	0.3727	0.4111	0.297	0.3008	0.2977	0.3357	0.3901	0.245	0.2104	0.3323	0.2626	0.3323	0.3678	0.2677	0.276	0.28	0.2527	0.2487	0.296	0.3228	0.3342	0.3667	0.3565	0.3558	0.35	0.398	0.4643	0.2844	0.2263	0.3914	0.2949	0.3914	0.438	0.3066	0.3221	0.3205
258	0.1752	0.2642	0.2418	0.3397	0.3188	0.2033	0.285	0.2355	0.2156	0.208	0.2156	0.2118	0.3035	0.2469	0.2854	0.2276	0.285	0.2663	0.2293	0.19	0.2214	0.1986	0.1842	0.2693	0.255	0.1585	0.2117	0.1842	0.1669	0.1625	0.1669	0.1643	0.24	0.1874	0.2276	0.1832	0.2186	0.2063	0.176	0.1494	0.1757	0.2589	0.2325	0.35	0.3245	0.1894	0.2844	0.2263	0.2029	0.1948	0.2029	0.1987	0.3066	0.2384	0.285
259	0.3212	0.4841	0.443	0.5366	0.5036	0.3725	0.5224	0.4148	0.3951	0.4632	0.3951	0.3881	0.5419	0.4529	0.5096	0.5604	0.5381	0.5027	0.4202	0.3478	0.418	0.5486	0.5088	0.5878	0.5565	0.4379	0.5847	0.478	0.4609	0.5175	0.4609	0.4539	0.591	0.5185	0.5604	0.5994	0.5921	0.5588	0.4861	0.4121	0.476	0.5148	0.4623	0.5738	0.532	0.3765	0.5655	0.4266	0.4033	0.4823	0.4033	0.3951	0.5787	0.4748	0.5381
260	0.6088	0.6562	0.6557	0.6603	0.619	0.5607	0.715	0.7645	0.6242	0.7062	0.6242	0.5842	0.6965	0.6838	0.6844	0.72	0.715	0.6623	0.6324	0.6273	0.5889	0.703	0.6992	0.7023	0.6643	0.6099	0.755	0.7896	0.6677	0.7374	0.6677	0.6322	0.7323	0.724	0.72	0.7473	0.7513	0.704	0.6772	0.6658	0.6333	0.6435	0.6442	0.65	0.602	0.5357	0.7156	0.7737	0.6086	0.7051	0.6086	0.562	0.6934	0.6779	0.6795
261	0.146	0.2199	0.2013	0.1969	0.1848	0.1692	0.2373	0.1793	0.1795	0.2552	0.1795	0.1763	0.2384	0.206	0.2242	0.3328	0.253	0.2364	0.1909	0.1578	0.1966	0.35	0.3246	0.3185	0.3015	0.2793	0.373	0.2938	0.294	0.355	0.294	0.2895	0.351	0.3311	0.3328	0.4162	0.3735	0.3525	0.3101	0.2627	0.3003	0.2559	0.2298	0.2238	0.2075	0.1872	0.2811	0.2002	0.2005	0.2875	0.2005	0.1964	0.2721	0.2363	0.253
262	188.32	121.4	143.2	121.79	139.03	191.89	104.16	168.92	174.44	163.27	174.44	179.57	124.32	137.42	141.55	146.04	113.33	130.57	157.21	213.91	183.83	125.88	147.69	126.28	143.51	196.38	108.65	173.4	178.92	167.76	178.92	184.06	128.81	141.91	146.04	150.52	117.82	135.05	161.69	218.4	188.32	93.176	114.98	93.573	110.8	163.67	75.944	140.7	146.22	135.05	146.22	151.35	96.102	109.2	113.33
263	108.53	80.909	88.524	89.295	106.7	134.35	62.161	67.981	108.36	89.37	108.36	122.02	82.379	79.427	91.08	93.003	69.929	88.676	99.656	122.23	129.86	82.831	90.447	91.217	108.62	136.27	64.084	69.903	110.28	91.293	110.28	123.95	84.302	81.349	93.003	94.925	71.851	90.599	101.58	124.15	131.78	68.458	76.074	76.844	94.247	121.9	49.711	55.53	95.907	76.92	95.907	109.57	69.929	66.976	78.63
264	48.605	62.161	62.161	89.295	89.295	62.161	62.161	67.981	62.161	63.267	62.161	62.161	82.379	62.024	82.379	75.6	69.929	69.929	62.161	62.298	69.929	55.382	55.382	82.516	82.516	55.382	55.382	61.202	55.382	56.488	55.382	55.382	75.6	55.245	75.6	68.821	63.15	63.15	55.382	55.519	63.15	49.711	49.711	76.844	76.844	49.711	49.711	55.53	49.711	50.816	49.711	49.711	69.929	49.574	69.929
265	89.117	113.92	113.92	141.05	141.05	113.92	113.92	119.74	113.92	140.88	113.92	113.92	147.08	113.78	147.08	186.13	132.01	132.01	113.92	114.05	132.01	152.97	152.97	180.1	180.1	152.97	152.97	158.78	152.97	179.93	152.97	152.97	186.13	152.83	186.13	225.18	171.06	171.06	152.97	153.1	171.06	98.841	98.841	125.98	125.98	98.841	98.841	104.66	98.841	125.8	98.841	98.841	132.01	98.704	132.01
266	168.9	154.4	168.6	173.55	173.38	171.47	155.92	220.67	180	214.78	180	171.47	189.03	171.77	197.56	239.17	175.41	173.9	171.47	205.74	185.98	196.02	210.21	215.16	214.99	213.08	197.53	262.28	221.61	256.39	221.61	213.08	230.64	213.38	239.17	280.78	217.03	215.51	213.08	247.35	227.59	123.56	137.75	142.7	142.53	140.62	125.07	189.83	149.15	183.94	149.15	140.62	158.18	140.93	166.71
267	40.512	51.756	51.756	51.756	51.756	51.756	51.756	51.756	51.756	77.613	51.756	51.756	64.706	51.756	64.706	110.53	62.08	62.08	51.756	51.756	62.08	97.583	97.583	97.583	97.583	97.583	97.583	97.583	97.583	123.44	97.583	97.583	110.53	97.583	110.53	156.36	107.91	107.91	97.583	97.583	107.91	49.131	49.131	49.131	49.131	49.131	49.131	49.131	49.131	74.988	49.131	49.131	62.08	49.131	62.08
268	0.4545	0.4444	0.4444	0.5	0.4545	0.5	0.4444	0.4545	0.5	0.5	0.5	0.4545	0.5	0.4444	0.4545	0.4615	0.4444	0.4444	0.4444	0.4615	0.4615	0.4545	0.4545	0.5	0.4615	0.5	0.4545	0.4615	0.5	0.5	0.5	0.4615	0.5	0.4545	0.4615	0.4667	0.4545	0.4545	0.4545	0.4667	0.4667	0.4286	0.4286	0.5	0.4444	0.5	0.4286	0.4444	0.5	0.5	0.5	0.4444	0.5	0.4286	0.4444
269	0.8333	0.8	0.8	1	0.8333	1	0.8	0.8333	1	1	1	0.8333	1	0.8	0.8333	0.8571	0.8	0.8	0.8	0.8571	0.8571	0.8333	0.8333	1	0.8571	1	0.8333	0.8571	1	1	1	0.8571	1	0.8333	0.8571	0.875	0.8333	0.8333	0.8333	0.875	0.875	0.75	0.75	1	0.8	1	0.75	0.8	1	1	1	0.8	1	0.75	0.8
270	5.282	5.323	5.733	6.229	6.229	6.217	5.323	6.19	5.323	7.176	5.323	5.783	6.693	5.253	6.693	9.101	6.003	6.003	5.323	6.557	6.599	7.731	8.141	8.637	8.637	8.625	7.731	8.598	7.731	9.584	7.731	8.191	9.101	7.661	9.101	11.509	8.411	8.411	7.731	8.965	9.007	4.633	5.043	5.539	5.539	5.527	4.633	5.5	4.633	6.486	4.633	5.093	6.003	4.563	6.003
271	-5.282	-5.323	-5.733	-7.229	-7.229	-6.217	-5.323	-6.19	-5.323	-6.176	-5.323	-5.783	-6.693	-5.253	-6.693	-8.101	-6.003	-6.003	-5.323	-6.558	-6.599	-6.731	-7.141	-8.637	-8.637	-7.625	-6.731	-7.598	-6.731	-7.584	-6.731	-7.191	-8.101	-6.661	-8.101	-9.509	-7.411	-7.411	-6.731	-7.966	-8.007	-4.633	-5.043	-6.539	-6.539	-5.527	-4.633	-5.5	-4.633	-5.486	-4.633	-5.093	-6.003	-4.563	-6.003
272	0.1715	0.1702	0.158	0.1454	0.1454	0.1457	0.1702	0.1464	0.1702	0.1263	0.1702	0.1567	0.1354	0.1725	0.1354	0.1319	0.1509	0.1509	0.1702	0.1382	0.1373	0.1552	0.1474	0.1389	0.1389	0.1391	0.1552	0.1396	0.1552	0.1252	0.1552	0.1465	0.1319	0.1566	0.1319	0.1043	0.1427	0.1427	0.1552	0.1339	0.1332	0.1956	0.1797	0.1636	0.1636	0.1639	0.1956	0.1647	0.1956	0.1397	0.1956	0.1779	0.1509	0.1986	0.1509
273	0.1715	0.1691	0.157	0.1245	0.1245	0.1448	0.1691	0.1454	0.1691	0.1457	0.1691	0.1556	0.1345	0.1713	0.1345	0.1194	0.1499	0.1499	0.1691	0.1372	0.1364	0.1437	0.1354	0.112	0.112	0.1268	0.1437	0.1273	0.1437	0.1275	0.1437	0.1345	0.1194	0.1452	0.1194	0.1017	0.1305	0.1305	0.1437	0.1214	0.1208	0.1943	0.1785	0.1376	0.1376	0.1628	0.1943	0.1636	0.1943	0.1641	0.1943	0.1767	0.1499	0.1972	0.1499
274	0.5358	0.3301	0.2429	0.2411	0.2878	0.4846	0.2772	0.3464	0.4534	0.3497	0.4534	0.2886	0.2335	0.3548	0.2793	0.2683	0.2228	0.3121	0.4186	0.442	0.4436	0.3092	0.2361	0.2347	0.2754	0.4486	0.2637	0.3266	0.4196	0.3305	0.4196	0.2766	0.2283	0.3314	0.2683	0.26	0.2188	0.2956	0.3878	0.4132	0.4136	0.2695	0.1708	0.1705	0.2309	0.4657	0.1975	0.3018	0.4276	0.3083	0.4276	0.2349	0.1641	0.304	0.2228
275	0.3598	0.2642	0.3443	0.3728	0.3499	0.4109	0.285	0.293	0.2156	0.208	0.2156	0.2791	0.3035	0.2469	0.2854	0.2276	0.285	0.2663	0.2293	0.4172	0.3836	0.1986	0.2719	0.2977	0.2819	0.3403	0.2117	0.2342	0.1669	0.1625	0.1669	0.223	0.24	0.1874	0.2276	0.1832	0.2186	0.2063	0.176	0.3501	0.3183	0.2589	0.3558	0.3896	0.3613	0.4312	0.2844	0.294	0.2029	0.1948	0.2029	0.2777	0.3066	0.2384	0.285
276	0.1752	0.2642	0.3443	0.3397	0.3188	0.2033	0.285	0.293	0.2156	0.208	0.2156	0.2791	0.3035	0.2469	0.2854	0.2276	0.285	0.2663	0.2293	0.2406	0.2214	0.1986	0.2719	0.2693	0.255	0.1585	0.2117	0.2342	0.1669	0.1625	0.1669	0.223	0.24	0.1874	0.2276	0.1832	0.2186	0.2063	0.176	0.1942	0.1757	0.2589	0.3558	0.35	0.3245	0.1894	0.2844	0.294	0.2029	0.1948	0.2029	0.2777	0.3066	0.2384	0.285
277	0.4642	0.6699	0.7571	0.7589	0.7122	0.5154	0.7228	0.6536	0.5466	0.6503	0.5466	0.7114	0.7665	0.6452	0.7207	0.7317	0.7772	0.6879	0.5814	0.558	0.5564	0.6908	0.7639	0.7653	0.7246	0.5514	0.7363	0.6734	0.5804	0.6695	0.5804	0.7234	0.7717	0.6686	0.7317	0.74	0.7812	0.7044	0.6122	0.5868	0.5864	0.7305	0.8292	0.8295	0.7691	0.5343	0.8025	0.6982	0.5724	0.6917	0.5724	0.7651	0.8359	0.696	0.7772
278	0.6402	0.7358	0.6557	0.6272	0.6501	0.5891	0.715	0.707	0.7844	0.792	0.7844	0.7209	0.6965	0.7531	0.7146	0.7724	0.715	0.7337	0.7707	0.5828	0.6164	0.8014	0.7281	0.7023	0.7181	0.6597	0.7883	0.7658	0.8331	0.8375	0.8331	0.777	0.76	0.8126	0.7724	0.8168	0.7814	0.7937	0.824	0.6499	0.6817	0.7411	0.6442	0.6104	0.6387	0.5688	0.7156	0.706	0.7971	0.8052	0.7971	0.7223	0.6934	0.7616	0.715
279	0.289	0.4057	0.4128	0.4192	0.3934	0.3122	0.4378	0.3606	0.3311	0.4423	0.3311	0.4323	0.463	0.3983	0.4353	0.5041	0.4921	0.4216	0.3521	0.3174	0.335	0.4922	0.492	0.496	0.4696	0.3929	0.5246	0.4391	0.4135	0.5071	0.4135	0.5004	0.5316	0.4812	0.5041	0.5569	0.5626	0.4981	0.4362	0.3926	0.4106	0.4716	0.4734	0.4795	0.4446	0.345	0.5181	0.4042	0.3695	0.4968	0.3695	0.4874	0.5293	0.4576	0.4921
280	148.65	77.685	62.453	63.373	80.605	148.18	60.453	99.977	130.73	106.37	130.73	84.7	63.373	89.136	80.605	89.136	54.672	81.95	113.49	144.97	140.12	86.216	70.984	71.904	89.136	156.71	68.983	108.51	139.26	114.9	139.26	93.231	71.904	97.667	89.136	97.667	63.202	90.481	122.02	153.5	148.65	51.751	36.519	37.44	54.672	122.25	34.519	74.044	104.79	80.435	104.79	58.767	37.44	63.202	54.672

Table S12 Amino acid descriptors (2D) used in this study.

	P	Q	Q	Q	Q	Q	Q	Q	Q	Q	Q	Q	Q	Q	Q	Q	Q	Q	Q	Q	Q
	Y	A	C	D	E	F	G	H	I	K	L	M	N	P	Q	R	S	T	V	W	Y
No.	PY	QA	QC	QD	QE	QF	QG	QH	QI	QK	QL	QM	QN	QP	QQ	QR	QS	QT	QV	QW	QY
281	99.833	62.161	88.524	97.996	97.996	125.64	62.161	84.59	62.161	63.267	62.161	81.922	82.379	62.024	82.379	75.6	69.929	69.929	62.161	136.83	121.16
282	48.605	62.161	88.524	89.295	89.295	62.161	62.161	84.59	62.161	63.267	62.161	81.922	82.379	62.024	82.379	75.6	69.929	69.929	62.161	78.907	69.929
283	128.78	157.63	194.67	199.47	199.47	157.63	157.63	188.68	157.63	197.78	157.63	208.79	208.03	162.07	208.03	243.04	190.67	180.63	157.63	182.99	175.72
284	177.6	173.15	168.6	164.85	182.08	180.17	155.92	204.06	226.19	240.88	226.19	211.57	189.03	189.18	206.26	256.57	175.41	192.65	208.96	191.13	194.68
285	80.178	95.468	106.14	110.18	110.18	95.468	95.468	104.09	95.468	134.52	95.468	126.87	125.66	100.04	125.66	167.44	120.74	110.7	95.468	104.09	105.79
286	6	5	5	5	6	6	5	6	5	6	5	6	5	5	6	7	5	5	5	7	7
287	0	0	1	0	0	0	0	0	0	0	0	0	0	0	0	0	0	0	0	0	0
288	2	0	0	0	0	1	0	1	0	0	0	0	0	1	0	0	0	0	0	2	1
289	0.1715	0.1702	0.158	0.1454	0.1454	0.1457	0.1702	0.1464	0.1702	0.1263	0.1702	0.1567	0.1354	0.1725	0.1354	0.1319	0.1509	0.1509	0.1702	0.1382	0.1373
290	0.1715	0.1691	0.157	0.1245	0.1245	0.1448	0.1691	0.1454	0.1691	0.1457	0.1691	0.1556	0.1345	0.1713	0.1345	0.1194	0.1499	0.1499	0.1691	0.1372	0.1364
291	-2.1048	-3.883	-3.9731	-5.7629	-5.3728	-2.6602	-4.2715	-3.9371	-2.8568	-4.4907	-2.8568	-3.1498	-5.0275	-3.3967	-4.6374	-6.4593	-4.9106	-4.5221	-3.2469	-2.1789	-2.9546
292	104.09	126.49	126.49	169.17	169.17	126.49	126.49	126.49	126.49	159.39	126.49	126.49	159.39	108.48	159.39	209.07	151.87	151.87	126.49	126.49	151.87
293	6.7228	11.982	11.982	13.446	13.446	11.982	11.982	28.767	11.982	11.982	11.982	11.982	17.24	15.106	17.24	13.881	11.982	11.982	11.982	11.982	11.982
294	55.688	79.551	79.551	103.41	103.41	79.551	75.569	96.336	79.551	79.551	79.551	79.551	103.41	79.551	103.41	79.551	79.551	86.019	79.551	96.336	79.551
295	18.44	0	17.234	0	0	0	18.44	0	0	18.44	0	17.234	0	18.44	0	18.44	20.926	0	0	0	0
296	3.1856	0	0	0	0	3.1856	0	2.7567	4.4108	0	4.4108	0	0	0	0	0	0	0	4.4108	3.1856	3.1856
297	18.868	0	0	0	0	18.868	0	18.868	0	0	0	31.691	0	0	0	0	0	0	0	18.868	18.868
298	70.573	0	0	0	0	88.216	0	34	0	0	0	0	0	0	0	0	0	0	0	92.504	70.573
299	37.737	37.737	83.844	56.605	75.474	37.737	37.737	37.737	56.605	94.342	56.605	56.605	56.605	75.474	75.474	75.474	37.737	37.737	37.737	37.737	37.737
300	5.2434	33.326	0	0	0	0	0	0	66.652	0	66.652	32.22	0	0	0	0	0	33.326	66.652	0	5.2434
301	6.8563	4.7716	5.5896	5.1666	5.6283	7.2188	4.3121	6.4334	6.1497	6.3648	6.1497	6.5042	5.5854	5.4847	6.0471	7.0399	4.9128	5.3723	5.688	8.4045	7.3853
302	90.408	114.27	114.27	180.82	180.82	114.27	114.27	114.27	114.27	114.27	114.27	114.27	138.13	114.27	138.13	114.27	114.27	114.27	114.27	114.27	114.27
303	43.396	0	0	0	0	0	0	16.786	0	0	0	0	0	3.1243	0	31.672	25.385	25.385	0	0	25.385
304	18.011	50.908	50.908	50.908	50.908	50.908	50.908	67.693	55.319	83.805	55.319	50.908	50.908	32.897	50.908	68.919	50.908	50.908	55.319	67.693	50.908
305	16.393	7.9639	7.9639	7.9639	7.9639	11.15	3.982	10.721	7.9639	7.9639	7.9639	7.9639	7.9639	7.9639	7.9639	7.9639	7.9639	14.433	7.9639	11.15	16.393
306	0	32.897	32.897	32.897	32.897	32.897	32.897	32.897	32.897	32.897	32.897	32.897	65.794	32.897	65.794	65.794	32.897	32.897	32.897	37.614	32.897
307	127.18	37.737	37.737	56.605	75.474	144.82	37.737	90.605	56.605	94.342	56.605	56.605	56.605	75.474	75.474	75.474	37.737	37.737	37.737	144.39	127.18
308	25.162	11.982	29.215	13.446	13.446	11.982	30.421	11.982	11.982	30.421	11.982	29.215	17.24	30.421	17.24	32.32	32.908	11.982	11.982	11.982	11.982
309	0	33.326	46.107	0	0	0	0	0	66.652	0	66.652	63.911	0	0	0	0	0	33.326	66.652	0	0
310	106.07	139.96	178.76	180.09	180.09	139.96	139.96	168.64	139.96	167.6	139.96	139.96	183.05	131.17	183.05	203.6	160.19	160.19	139.96	155.75	160.19
311	0.4846	0.542	0.5212	0.4845	0.4681	0.4537	0.5647	0.469	0.4845	0.4681	0.4845	0.4845	0.4845	0.5226	0.4681	0.4388	0.5212	0.5021	0.5021	0.4257	0.4395
312	5.3923	4.8074	4.9069	5.0875	5.1699	5.3923	4.7004	5.3219	5.0875	5.1699	5.0875	5.0875	5.0875	5.0875	5.1699	5.3219	4.9069	5	5	5.6439	5.4594
313	3.2336	3.0302	3.0576	3.1778	3.2811	3.3642	3.0203	3.293	3.1423	3.3343	3.1778	3.2339	3.1778	3.0181	3.2811	3.4959	3.0576	3.0711	3.0711	3.4701	3.4432
314	8.3451	7.4883	7.6856	8.0375	8.1921	8.4845	7.2753	8.3428	8.0406	8.1871	8.0375	8.033	8.0375	7.8714	8.1921	8.4712	7.6856	7.8695	7.8695	8.8727	8.6151
315	277.43	235.31	257.12	262.84	280.08	305.81	218.08	288.65	288.35	304.15	288.35	293.49	271.41	251.2	288.64	332.17	245.34	262.58	271.12	327.97	315.84
316	361.88	266.51	287.83	296.25	320.68	381.48	242.08	345.74	339.8	358.35	339.8	337.75	306.79	303.09	331.22	382.48	275.03	299.46	315.37	426.83	390.01
317	40.701	54.268	54.268	81.402	81.402	54.268	54.268	54.268	54.268	54.268	54.268	54.268	67.835	54.268	67.835	54.268	54.268	54.268	54.268	54.268	54.268
318	27.134	27.134	27.134	54.268	54.268	27.134	27.134	27.134	27.134	27.134	27.134	27.134	27.134	27.134	27.134	27.134	27.134	27.134	27.134	27.134	27.134
319	5.6826	17.742	17.742	17.742	17.742	17.742	17.742	17.742	17.742	35.485	17.742	17.742	17.742	17.742	17.742	58.91	17.742	17.742	17.742	17.742	17.742
320	11.365	41.168	41.168	41.168	41.168	41.168	41.168	41.168	41.168	58.91	41.168	41.168	58.91	35.485	58.91	82.335	41.168	41.168	41.168	46.85	41.168
321	155.03	74.425	62.801	60.825	76.633	142.08	58.616	87.824	121.85	112.74	121.85	129.75	60.825	96.933	76.633	96.933	67.075	82.883	106.04	155.47	134.73
322	27.658	40.608	71.992	55.316	55.316	40.608	40.608	65.398	40.608	40.608	40.608	40.608	53.557	40.608	53.557	56.289	40.608	40.608	40.608	40.608	40.608
323	65.633	95.435	95.435	122.57	122.57	95.435	95.435	106.8	95.435	113.18	95.435	95.435	126.74	89.753	126.74	136.6	109	109	95.435	101.12	109
324	278.31	217.22	249.29	260.23	274.25	293.32	203.2	283.29	259.31	275.33	259.31	277.35	260.25	243.26	274.28	303.34	233.22	247.25	245.28	332.36	309.32
325	873	424	500	686	812	1042	363	909	673	828	686	701	686	548	812	1122	500	578	578	1457	1193
326	25	17	19	21	22	26	14	23	23	22	21	21	21	22	22	24	19	21	21	33	28
327	98	64	68	78	82	96	58	92	78	80	78	76	78	80	82	90	68	74	74	118	102
328	-3.7972	4.9981	4.0971	5.3918	5.2624	1.2306	-3.9074	5.0068	5.4828	-3.612	3.3503	0.9592	2.8064	1.5068	-0.5731	-3.1994	5.214	4.3308	3.242	4.008	-1.5757
329	4.2941	1.8912	0.5034	-2.8185	-0.0846	-3.2311	0.4815	0.9747	-0.0074	-4.6341	0.0174	-5.1836	1.2703	4.7744	-2.2948	-1.5811	1.6475	1.2479	-1.9502	0.3627	-0.4954
330	0.6641	0.6723	-1.2517	0.2483	3.3771	-2.6481	-0.2776	0.3767	-0.9159	0.1929	-0.1853	0.9297	0.5405	0.8326	-0.8157	2.1626	-0.1476	-0.4204	-0.4676	-0.9571	-3.1994
331	0	0	0	0	0	0	0	0	0	-3E-14	0	0	0	0	0	-3E-13	0	0	0	0	0
332	0	0	0	0	0	0	0	0	0	-3E-14	0	0	0	0	0	-3E-13	0	0	0	0	0
333	2492.2	1394.4	2641.3	1434.1	2687.6	2384.1	1626.9	2082.6	1921.1	3520.4	2029.5	2506	2990.1	1116	2455.6	3745.6	1429.6	1309.3	1618.1	5592.3	4357.9
334	995.88	379.06	284.15	738.1	492.28	538.06	177.57	805.64	413.64	577.76	582.38	621.98	394.64	592.39	597.72	811.57	394.5	591.01	518.67	786.98	494.73
335	127.88	159.44	192.04	169.78	286.85	424.04	122.54	181.22	281.02	115.68	243.28	151.42	131.02	254.52	267.63	209.18	154.37	180.89	312.28	166.46	289.19
336	23.492	16.342	8.0594	17.356	21.736	14.691	7.4749	18.346	21.043	26.668	5.3495	17.09	6.0134	15.651	7.7867	12.427	19.152	8.9651	10.076	2.2582	8.5782
337	-86114	-72256	-76779	-89725	-93294	-91219	-68712	-91260	-83019	-88334	-83074	-83909	-87740	-78784	-91290	-97882	-79642	-83288	-79451	-102257	-98662
338	-554118	-411549	-432434	-542110	-565794	-628760	-353383	-601041	-546049	-568360	-537918	-540680	-517187	-498363	-578428	-674716	-462278	-509945	-507913	-703411	-652769
339	-90.274	-137.32	-173.24	-266.71	-243.72	-99.613	-181.82	-81.086	-140.99	-45.693	-196.64	-128.35	-220.55	-123.17	-184.22	-30.631	-174.85	-232.99	-157.61	-128.53	-194.2
340	-7.6691	-7.7515	-9.1991	-4.8976	-4.6329	-8.411	-10.214	-7.689	-7.7726	-12.011	-10.257	-7.9131	-10.517	-8.1999	-8.6116	-12.748	-8.1229	-10.069	-8.5424	-8.4607	-9.1684
341	7.6691	7.7515	9.1991	4.8976	4.6329	8.411	10.214	7.689	7.7726	12.011	10.257	7.9131	10.517	8.1999	8.6116	12.748	8.1229	10.069	8.5424	8.4607	9.1684
342	-1.331	-0.6616	0.4397	2.2136	1.4834	-1.4274	0.8936	-0.666	-0.9459	-4.1143	0.9563	-1.2895	0.6493	-1.009	-0.8466	-4.2289	-1.2061	0.8535	-0.2556	0.1334	0.1609
343	501.94	415.74	443.45	415.57	472.63	500.42	392.06	474.59	460.94	519.31	478.36	488.02	459.15	427.77	481.61	534.62	404.73	425.79	457.87	589.61	539.13
344	177.64	142.21	173.58	154.53	173.16	215.28	174.37	243.74	127.21	220.14	133.21	169.12	206.97	132.7	232.46	266.48	154.87	150.19	141.76	278.27	250.6
345	147.74	110.14	163.8	175.14	175.86	186.58	115.92	130.15	95.438	97.579	112.23	132.82	149.12	106.88	152.95	108.27	116.27	108.97	117.62	212.91	196
346	315.9	167	122.13	100.72	133.28	281.74	117.47	175.72	243.96	206.81	235.59	191.27	112.42	192.18	98.77	164.98	141.82	172.46	199.85	278.68	243.49
347	186.04	248.74	321.32	314.85	339.35	218.68	274.59	298.87	216.98	312.5	242.77	296.75	346.73	235.59	382.84	369.64	262.9	253.33	258.02	310.93	295.64
348	938.31	756.99	995.15	962.54	1078.6	1338.4	928.15	1508.7	677.13	1579.7	709.06	978.01	1385.3	697.09	1555.9	2425.2	929.68	901.58	754.58	1824.6	1653.7
349	780.35	586.26	939.04	1266.1	1271.3	1160	617.04	805.6	508.02	602.65	597.38	768.07	998.05	561.43	1023.7	877.12	697.95	654.15	626.11	1396.3	1293.4
350	29.905	32.074	9.7862	20.614	2.703	28.699	58.447	113.59	31.77	122.56	20.98	36.302	57.854	25.825	79.513	158.2	38.603	41.219	24.134	65.363	54.6
351	157.96	170.73	56.104	303.54	192.7	178.42	311.11	703.13	169.11	977.08	111.68	209.94	387.22	135.66	532.18	1548.1	231.73	247.44	128.47	428.37	360.31
352	1.0592	1.1062	1.1649	1.1951	1.1517	1.0647	1.1368	1.1203	1.0377	1.0504	1.0367	1.1094	1.1547	1.1026	1.1184	1.0877	1.1546	1.1387	1.0493	1.0683	1.0988
353	5.7706	5.386	4.3135	6.0891	6.2534	4.3551	3.9467	5.1147	5.5588	5.8786	3.3555	5.3529	3.1276	5.0753	2.502	4.1728	5.4701	4.5266	3.8121	4.1366	3.6006
354	48.307	3.9032	0.0104	1.0228	17.791	38.195	-11.245	0.6281	15.694	-10.908	7.0199	16.187	-32.192	16.729	11.865	-72.468	8.7815	-6.7519	26.009	39.139	16.07
355	8.9647	8.6074	7.1748	10.325	14.801	18.921	4.5771	8.6442	15.827	7.7589	11.907	8.5604	13.111	11.474	13.161	11.642	11.137	8.7476	12.185	6.7522	17.833
356	0.0928	-35.338	-71.38	-61.14	-35.535	-33.199	-69.37	-39.863	-35.688	-86.977	-66.72	-33.974	-110.99	-33.785	-28.636	-152.3	-41.172	-90.544	-11.32	-4.1132	-74.622
357	31.112	-9.1662	-29.372	-26.633	-5.3777	6.1678	-32.818	-14.043	-3.7783	-36.029	-24.281	-4.7652	-58.88	-6.2631	-10.438	-105.2	-10.49	-38.078	8.9591	23.383	-16.44
358	0.0442	0.0938	1.6986	1.4512	0.5423	0.4805	1.6211	0.079	0.4329	1.6401	1.4432	0.1578	0.1186	0.0685	0.1969	1.4363	0.0919	1.7715	0.1473	0.5435	0.3389
359	-96.504	-89.078	-61.484	-152.35	-168.17	-91.78	-58.343	-85.453	-87.366	-127.71	-63.213	-98.651	-44.577	-95.08	-89.332	-86.031	-92.345	-65.366	-96.392	-92.444	-70.145
360	-0.5281	0.0499	0.2433	-0.0003	0.507	0.3085	0.0327	0.1451	0.5609	0.3928	0.4006	0.435	0.4163	0.0553	-0.0988	0.2615	0.0756	0.3821	-0.0737	-0.7761	0.0613
361	4.1123	2.8057	5.7899	4.2667	4.367	5.6741	4.9034	3.2005	4.338	6.0399	6.1586	3.7998	5.6607	3.6319	3.0617	7.2567	3.4927	6.0505	3.5982	4.1839	6.6056
362	1.44	0.0042	0.0034	0.0041	0.0047	0.0125	0.0037	0.0056	0.0272	0.0126	0.0209	0.0112	0.006	0.0035	0.0109	0.0063	0.0034	0.0067	0.012	0.0185	0.02
363	4.6019	1.5127	14.475	11.614	2.9506	6.6426	10.439	2.6019	-1.6872	9.289	11.392	7.999	7.3806	7.7631	5.9826	12.14	4.4743	14.374	1.193	5.0519	7.6717
364	31.019	26.172	42.009	34.506	30.157	39.367	36.551	25.821	31.91	50.948	42.439	29.209	52.108	27.522	18.198	47.099	30.682	52.466	20.279	27.496	58.182

	R	R	R	R	R	R	R	R	R	R	R	R	R	R	R	R	R	R	R	R
	A	C	D	E	F	G	H	I	K	L	M	N	P	Q	R	S	T	V	W	Y
No.	RA	RC	RD	RE	RF	RG	RH	RI	RK	RL	RM	RN	RP	RQ	RR	RS	RT	RV	RW	RY
281	55.382	81.745	91.217	91.217	118.87	55.382	77.811	55.382	56.488	55.382	75.143	75.6	55.245	75.6	68.821	63.15	63.15	55.382	130.06	114.38
282	55.382	81.745	82.516	82.516	55.382	55.382	77.811	55.382	56.488	55.382	75.143	75.6	55.245	75.6	68.821	63.15	63.15	55.382	72.128	63.15
283	192.63	229.67	234.47	234.47	192.63	192.63	223.68	192.63	232.79	192.63	243.79	243.04	197.07	243.04	278.04	225.67	215.63	192.63	218	210.72
284	223.46	218.91	215.16	232.39	230.48	206.23	254.38	276.51	291.2	276.51	261.88	239.34	239.49	256.57	306.88	225.73	242.96	259.27	241.44	245
285	137.25	147.93	151.96	151.96	137.25	137.25	145.87	137.25	176.3	137.25	168.65	167.44	141.82	167.44	209.22	162.52	152.48	137.25	145.87	147.57
286	6	6	6	7	7	6	7	6	7	6	7	6	6	7	8	6	6	6	8	8
287	0	1	0	0	0	0	0	0	0	0	0	0	0	0	0	0	0	0	0	0
288	0	0	0	0	1	0	1	0	0	0	0	0	1	0	0	0	0	0	2	1
289	0.1552	0.1474	0.1389	0.1389	0.1391	0.1552	0.1396	0.1552	0.1252	0.1552	0.1465	0.1319	0.1566	0.1319	0.1043	0.1427	0.1427	0.1552	0.1339	0.1332
290	0.1437	0.1354	0.112	0.112	0.1268	0.1437	0.1273	0.1437	0.1275	0.1437	0.1345	0.1194	0.1452	0.1194	0.1017	0.1305	0.1305	0.1437	0.1214	0.1208
291	-5.7049	-5.795	-7.5848	-7.1947	-4.4821	-6.0934	-5.759	-4.6787	-6.3126	-4.6787	-4.9717	-6.8494	-5.2186	-6.4593	-8.2812	-6.7325	-6.344	-5.0688	-4.0008	-4.7765
292	176.17	176.17	218.85	218.85	176.17	176.17	176.17	176.17	209.07	176.17	176.17	209.07	158.16	209.07	258.75	201.56	201.56	176.17	176.17	201.56
293	8.6219	8.6219	10.086	10.086	8.6219	8.6219	25.407	8.6219	8.6219	8.6219	8.6219	13.881	11.746	13.881	10.521	8.6219	8.6219	8.6219	8.6219	8.6219
294	55.688	55.688	79.551	79.551	55.688	51.706	72.474	55.688	55.688	55.688	55.688	79.551	55.688	79.551	55.688	55.688	62.157	55.688	72.474	55.688
295	18.44	35.673	18.44	18.44	18.44	36.879	18.44	18.44	36.879	18.44	35.673	18.44	36.879	18.44	36.879	39.366	18.44	18.44	18.44	18.44
296	0	0	0	0	3.1856	0	2.7567	4.4108	0	4.4108	0	0	0	0	0	0	0	4.4108	3.1856	3.1856
297	0	0	0	0	18.868	0	18.868	0	0	0	31.691	0	0	0	0	0	0	0	18.868	18.868
298	0	0	0	0	88.216	0	34	0	0	0	0	0	0	0	0	0	0	0	92.504	70.573
299	37.737	83.844	56.605	75.474	37.737	37.737	37.737	56.605	94.342	56.605	56.605	56.605	75.474	75.474	75.474	37.737	37.737	37.737	37.737	37.737
300	33.326	0	0	0	0	0	0	66.652	0	66.652	32.22	0	0	0	0	0	33.326	66.652	0	5.2434
301	5.7644	6.5824	6.1594	6.6211	8.2116	5.3049	7.4262	7.1425	7.3576	7.1425	7.497	6.5782	6.4775	7.0399	8.0327	5.9056	6.3651	6.6808	9.3973	8.3781
302	90.408	90.408	156.95	156.95	90.408	90.408	90.408	90.408	90.408	90.408	90.408	114.27	90.408	114.27	90.408	90.408	90.408	90.408	90.408	90.408
303	31.672	31.672	31.672	31.672	31.672	31.672	48.457	31.672	31.672	31.672	31.672	31.672	34.796	31.672	63.344	57.057	57.057	31.672	31.672	57.057
304	68.919	68.919	68.919	68.919	68.919	68.919	85.704	73.329	101.82	73.329	68.919	68.919	50.908	68.919	86.929	68.919	68.919	73.329	85.704	68.919
305	7.9639	7.9639	7.9639	7.9639	11.15	3.982	10.721	7.9639	7.9639	7.9639	7.9639	7.9639	7.9639	7.9639	7.9639	7.9639	14.433	7.9639	11.15	16.393
306	32.897	32.897	32.897	32.897	32.897	32.897	32.897	32.897	32.897	32.897	32.897	65.794	32.897	65.794	65.794	32.897	32.897	32.897	37.614	32.897
307	37.737	37.737	56.605	75.474	144.82	37.737	90.605	56.605	94.342	56.605	56.605	56.605	75.474	75.474	75.474	37.737	37.737	37.737	144.39	127.18
308	27.061	44.295	28.525	28.525	27.061	45.501	27.061	27.061	45.501	27.061	44.295	32.32	45.501	32.32	47.4	47.988	27.061	27.061	27.061	27.061
309	33.326	46.107	0	0	0	0	0	66.652	0	66.652	63.911	0	0	0	0	0	33.326	66.652	0	0
310	160.51	199.31	200.64	200.64	160.51	160.51	189.19	160.51	188.15	160.51	160.51	203.6	151.72	203.6	224.15	180.74	180.74	160.51	176.3	180.74
311	0.5021	0.4845	0.4529	0.4388	0.4262	0.5212	0.4395	0.4529	0.4388	0.4529	0.4529	0.4529	0.4855	0.4388	0.4133	0.4845	0.4681	0.4681	0.4018	0.4138
312	5	5.0875	5.2479	5.3219	5.5236	4.9069	5.4594	5.2479	5.3219	5.2479	5.2479	5.2479	5.2479	5.3219	5.4594	5.0875	5.1699	5.1699	5.7549	5.585
313	3.3165	3.3398	3.4348	3.5172	3.5856	3.3059	3.5276	3.4075	3.5591	3.4348	3.4788	3.4348	3.3074	3.5172	3.6889	3.3398	3.3516	3.3516	3.6736	3.65
314	7.8333	8.0072	8.3229	8.4651	8.7311	7.649	8.6009	8.324	8.4623	8.3229	8.3208	8.3229	8.1688	8.4651	8.7241	8.0072	8.17	8.17	9.0881	8.8523
315	278.85	300.66	306.38	323.61	349.34	261.61	332.19	331.89	347.68	331.89	337.02	314.94	294.73	332.17	375.71	288.88	306.11	314.66	371.5	359.37
316	317.77	339.09	347.51	371.93	432.74	293.34	397	391.06	409.61	391.06	389.01	358.05	354.35	382.48	433.73	326.29	350.72	366.63	478.09	441.26
317	40.701	40.701	67.835	67.835	40.701	40.701	40.701	40.701	40.701	40.701	40.701	54.268	40.701	54.268	40.701	40.701	40.701	40.701	40.701	40.701
318	27.134	27.134	54.268	54.268	27.134	27.134	27.134	27.134	27.134	27.134	27.134	27.134	27.134	27.134	27.134	27.134	27.134	27.134	27.134	27.134
319	58.91	58.91	58.91	58.91	58.91	58.91	58.91	58.91	76.653	58.91	58.91	58.91	58.91	58.91	100.08	58.91	58.91	58.91	58.91	58.91
320	64.593	64.593	64.593	64.593	64.593	64.593	64.593	64.593	82.335	64.593	64.593	82.335	58.91	82.335	105.76	64.593	64.593	64.593	70.275	64.593
321	94.724	83.1	81.124	96.933	162.38	78.916	108.12	142.15	133.04	142.15	150.05	81.124	117.23	96.933	117.23	87.374	103.18	126.34	175.77	155.03
322	43.34	74.724	58.048	58.048	43.34	43.34	68.13	43.34	43.34	43.34	43.34	56.289	43.34	56.289	59.021	43.34	43.34	43.34	43.34	43.34
323	105.29	105.29	132.43	132.43	105.29	105.29	116.66	105.29	123.04	105.29	105.29	136.6	99.611	136.6	146.46	118.86	118.86	105.29	110.98	118.86
324	246.29	278.36	289.29	303.32	322.39	232.26	312.35	288.37	304.39	288.37	306.41	289.32	272.33	303.34	332.41	262.29	276.32	274.35	361.43	338.39
325	642	741	977	1132	1416	560	1254	962	1150	977	994	977	806	1132	1506	741	842	842	1918	1598
326	19	21	23	24	28	16	25	25	24	23	23	23	24	24	26	21	23	23	35	30
327	72	76	86	90	104	66	100	86	88	86	84	86	88	90	98	76	82	82	126	110
328	5.8995	7.771	-5.5499	11.8	10.126	6.872	1.0477	7.0993	1.8999	7.9088	7.0984	6.8203	5.9692	8.1998	1.5349	7.5381	8.8418	7.2907	10.577	8.3258
329	-3.7934	0.0279	1.4543	0.7932	2.4137	0.1759	-2.7447	-4.0985	2.9093	1.3526	-0.1718	0.1439	-5.3048	-5.1555	-6.9996	-0.7565	2.5024	-4.7084	1.8615	-1.3822
330	-1.1889	0.6913	0.7654	-0.6869	2.8117	-0.0152	0.2304	-1.3116	3.5306	-0.959	1.981	-1.2368	0.9502	-0.5618	-2.4219	0.3327	-0.2101	1.6013	0.4994	-0.7546
331	0	0	0	0	-1E-13	0	0	0	3E-13	0	0	0	3E-14	0	2E-13	0	0	0	-2E-13	0
332	0	0	0	0	-1E-13	0	0	0	3E-13	0	0	0	3E-14	0	2E-13	0	0	0	-2E-13	0
333	1979.1	2593.4	1287.9	3984.6	3955.3	2400.3	2782.1	2870.3	4113	3878.1	3179.5	2800.2	2502.5	4663.5	5078.2	3419.9	3678.2	3180.9	4397.9	5196.5
334	391.78	735.04	614.27	718.27	1060	291.42	777.72	594.49	985.59	527.33	1150.5	897.08	608.02	814.54	922.77	469.86	443.11	616.21	1463.9	613.01
335	263.78	264.93	360.5	251.49	540.04	140.5	171.43	327.78	313.51	259.41	298.63	199.1	243.31	217.7	476.37	233.74	212.71	187.61	426.8	527.77
336	10.155	24.761	10.217	28.531	40.386	20.829	8.6264	25.698	11.134	22.635	25.712	15.684	15.449	16.435	14.167	23.906	31.776	25.145	34.72	27.728
337	-78822	-83319	-96393	-99960	-97749	-75256	-97846	-89564	-94829	-89624	-90472	-94305	-85346	-97856	-104348	-86240	-89775	-85973	-108749	-105210
338	-522378	-526317	-689262	-671527	-696253	-437620	-723503	-646907	-695634	-615046	-637208	-637815	-602277	-693695	-749555	-529153	-595834	-593101	-840200	-754953
339	41.3	30.064	-190.76	-171.84	114.27	18.987	77.249	57.1	204.29	-0.5066	71.277	-42.524	58.901	-4.7163	247.84	-29.831	22.937	67.392	126.34	1.3629
340	-12.291	-11.186	-8.8094	-7.4367	-9.42	-12.537	-11.412	-10.299	-13.612	-12.147	-9.9905	-12.609	-11.202	-11.26	-12.201	-12.347	-10.037	-10.174	-9.7852	-10.859
341	12.291	11.186	8.8094	7.4367	9.42	12.537	11.412	10.299	13.612	12.147	9.9905	12.609	11.202	11.26	12.201	12.347	10.037	10.174	9.7852	10.859
342	-3.2814	-4.5693	-0.2323	-1.9669	-4.8724	-4.4333	-2.8286	-4.1319	-5.4503	-4.4292	-4.2777	-3.6933	-3.6893	-3.5398	-5.642	-4.4071	-4.4142	-3.9941	-4.741	-4.4281
343	461.27	501.86	422.8	532.23	588.64	447.97	512.52	533.7	590.59	550.8	563.47	513.87	497.87	577.04	623.69	492.21	513.64	529.67	622.94	603.16
344	199.05	231.16	152.52	202.54	316.4	234.66	283.98	197.23	282.93	198.88	222.93	245.33	211.02	284.66	339.07	239.35	214.92	215.78	317.83	309.62
345	98.237	143.5	119.01	161.7	163.88	98.844	104.41	83.952	91.506	89.096	120.2	122.97	90.984	144.37	110.36	119.36	105.49	89.673	181.82	171.98
346	163.98	150.25	157.7	175.24	269.75	125.11	204.43	253.07	216.71	262.82	220.92	153.55	196.43	149.91	174.27	134.58	195.06	224.22	289.56	264.39
347	297.29	351.61	265.1	356.99	318.89	322.87	308.1	280.64	373.87	287.97	342.55	360.32	301.44	427.13	449.43	357.63	318.58	305.45	333.38	338.77
348	1538.9	1881.9	1317.3	1749.4	2728.9	1814.2	2441.7	1524.8	2711.6	1537.5	1826	2232.8	1616.6	2590.7	3902.4	2013.2	1807.7	1668.2	2849.4	2788.7
349	661.24	1024.8	1027.9	1396.6	1249.6	665.32	793.28	565.08	693.98	599.71	864.36	996.2	606.05	1169.5	1049.4	884.6	781.8	603.59	1448.4	1377
350	100.82	87.662	33.516	40.848	152.51	135.82	179.58	113.27	191.42	109.78	102.73	122.36	120.03	140.29	228.72	119.99	109.43	126.1	136.01	137.64
351	877.65	857.16	289.48	352.8	1479.3	1148.8	1648.4	959.67	2017.6	937.81	961.63	1236.6	1010.6	1421.2	2853	1128.6	1025.9	1064.6	1401	1411.7
352	1.0565	1.1002	1.1659	1.1146	1.0174	1.079	1.0813	1.0022	0.9976	1.0009	1.0543	1.1128	1.0434	1.0757	1.0351	1.098	1.0726	1.0128	1.0375	1.0604
353	7.1139	7.8017	5.7881	11.847	10.783	6.8743	2.9469	8.3017	4.9537	8.0808	7.3716	6.933	8.0421	9.7021	7.5641	7.5832	9.1915	8.8254	10.752	8.4734
354	-35.539	-48.35	-113.54	-72.112	-16.601	-71.335	-81.183	-31.573	-38.642	-50.227	-40.918	-74.884	-18.495	-40.811	-93.435	-69.269	-32.88	-24.556	-15.392	-48.383
355	15.207	8.3075	26.938	12.648	7.8872	10.377	20.297	15.526	12.44	9.017	11.967	7.2959	14.923	9.7589	10.632	13.96	8.4214	8.3153	10.831	15.351
356	-78.915	-117.53	-240.27	-140.42	-61.409	-124.9	-150.13	-76.768	-71.387	-118.3	-89.291	-153.46	-56.437	-76.09	-127.96	-141.27	-81.41	-54.06	-59.35	-130.79
357	-57.369	-75.342	-160.38	-93.399	-30.116	-85.389	-108.93	-50.037	-49.231	-72.472	-62.04	-104.66	-39.507	-54.208	-108.28	-91.061	-55.165	-34.633	-29.634	-71.964
358	0.328	1.1419	2.8649	0.0295	0.0944	0.107	0.0562	0.0674	0.1034	1.7079	0.1682	1.3917	0.1362	0.1889	0.3749	0.6697	1.2942	0.1217	0.3513	0.2909
359	-150.62	-163.26	-100	-169.08	-160.84	-143.47	-88.515	-166.99	-192.95	-147.78	-126.57	-116.38	-155.9	-168.62	-198.81	-103.9	-162.98	-167.27	-176.76	-137.04
360	-0.514	0.1069	-0.5779	0.0146	-0.2118	-0.0506	-0.2743	-0.3515	-0.0869	-0.0268	-0.3883	-0.004	0.1793	-0.0439	0.2509	0.2474	0.0047	0.2122	-0.9051	0.1327
361	4.2079	6.5243	9.2246	6.4332	5.2256	5.2372	6.5263	5.541	4.7921	7.302	4.6905	6.8709	3.9201	4.2347	5.7552	6.4052	5.1013	4.8488	5.898	8.008
362	0.0039	0.0043	0.0043	0.0131	0.009	0.0038	21.054	0.0135	0.0271	0.0181	0.0071	0.0063	0.0549	0.0079	0.0121	0.0048	0.0061	0.0208	0.0087	0.0073
363	2.6006	10.911	8.3818	2.1619	0.5194	-1.617	1.1384	-2.3197	-6.6592	4.2445	4.6848	14.22	1.8525	-0.742	-2.1648	0.5096	7.4633	-3.4205	-1.9331	-0.2005
364	21.546	42.191	79.898	47.022	31.294	39.511	41.201	26.731	22.156	45.832	27.251	48.798	16.931	21.882	19.68	50.208	26.245	19.427	29.716	58.824

	S	S	S	S	S	S	S	S	S	S	S	S	S	S
	A	C	D	E	F	G	H	I	K	L	M	N	P	Q
No.	SA	SC	SD	SE	SF	SG	SH	SI	SK	SL	SM	SN	SP	SQ
281	49.711	76.074	85.546	85.546	113.19	49.711	72.139	49.711	50.816	49.711	69.471	69.929	49.574	69.929
282	49.711	76.074	76.844	76.844	49.711	49.711	72.139	49.711	50.816	49.711	69.471	69.929	49.574	69.929
283	140.27	177.31	182.11	182.11	140.27	140.27	171.31	140.27	180.42	140.27	191.43	190.67	144.7	190.67
284	142.31	137.75	134	151.23	149.32	125.07	173.22	195.35	210.04	195.35	180.72	158.18	158.33	175.41
285	90.556	101.23	105.26	105.26	90.556	90.556	99.175	90.556	129.6	90.556	121.96	120.74	95.129	120.74
286	4	4	4	5	5	4	5	4	5	4	5	4	4	5
287	0	1	0	0	0	0	0	0	0	0	0	0	0	0
288	0	0	0	0	1	0	1	0	0	0	0	0	1	0
289	0.1956	0.1797	0.1636	0.1636	0.1639	0.1956	0.1647	0.1956	0.1397	0.1956	0.1779	0.1509	0.1986	0.1509
290	0.1943	0.1785	0.1376	0.1376	0.1628	0.1943	0.1636	0.1943	0.1641	0.1943	0.1767	0.1499	0.1972	0.1499
291	-4.1562	-4.2463	-6.0361	-5.646	-2.9334	-4.5447	-4.2103	-3.13	-4.7639	-3.13	-3.423	-5.3007	-3.6699	-4.9106
292	118.98	118.98	161.66	161.66	118.98	118.98	118.98	118.98	151.87	118.98	118.98	151.87	100.97	151.87
293	6.7228	6.7228	8.1868	8.1868	6.7228	6.7228	23.508	6.7228	6.7228	6.7228	6.7228	11.982	9.8471	11.982
294	55.688	55.688	79.551	79.551	55.688	51.706	72.474	55.688	55.688	55.688	55.688	79.551	55.688	79.551
295	20.926	38.16	20.926	20.926	20.926	39.366	20.926	20.926	39.366	20.926	38.16	20.926	39.366	20.926
296	0	0	0	0	3.1856	0	2.7567	4.4108	0	4.4108	0	0	0	0
297	0	0	0	0	18.868	0	18.868	0	0	0	31.691	0	0	0
298	0	0	0	0	88.216	0	34	0	0	0	0	0	0	0
299	0	46.107	18.868	37.737	0	0	0	18.868	56.605	18.868	18.868	18.868	37.737	37.737
300	33.326	0	0	0	0	0	0	66.652	0	66.652	32.22	0	0	0
301	3.6374	4.4554	4.0324	4.4941	6.0846	3.1779	5.2991	5.0155	5.2306	5.0155	5.37	4.4511	4.3504	4.9128
302	90.408	90.408	156.95	156.95	90.408	90.408	90.408	90.408	90.408	90.408	90.408	114.27	90.408	114.27
303	25.385	25.385	25.385	25.385	25.385	25.385	42.171	25.385	25.385	25.385	25.385	25.385	28.51	25.385
304	50.908	50.908	50.908	50.908	50.908	50.908	67.693	55.319	83.805	55.319	50.908	50.908	32.897	50.908
305	7.9639	7.9639	7.9639	7.9639	11.15	3.982	10.721	7.9639	7.9639	7.9639	7.9639	7.9639	7.9639	7.9639
306	0	0	0	0	0	0	0	0	0	0	0	32.897	0	32.897
307	0	0	18.868	37.737	107.08	0	52.868	18.868	56.605	18.868	18.868	18.868	37.737	37.737
308	27.649	44.883	29.113	29.113	27.649	46.089	27.649	27.649	46.089	27.649	44.883	32.908	46.089	32.908
309	33.326	46.107	0	0	0	0	0	66.652	0	66.652	63.911	0	0	0
310	117.1	155.9	157.23	157.23	117.1	117.1	145.78	117.1	144.74	117.1	117.1	160.19	108.31	160.19
311	0.6167	0.5895	0.542	0.5212	0.5033	0.6468	0.5226	0.542	0.5212	0.542	0.542	0.542	0.5917	0.5212
312	4.4594	4.585	4.8074	4.9069	5.1699	4.3219	5.0875	4.8074	4.9069	4.8074	4.8074	4.8074	4.8074	4.9069
313	2.6517	2.6888	2.8374	2.9843	3.0781	2.6622	2.9874	2.7867	3.0608	2.8374	2.9203	2.8374	2.6127	2.9843
314	6.8502	7.0973	7.524	7.7045	8.0494	6.5745	7.8846	7.5306	7.6956	7.524	7.5149	7.524	7.3304	7.7045
315	192.02	213.83	219.55	236.78	262.52	174.79	245.36	245.06	260.86	245.06	250.19	228.11	207.91	245.34
316	210.32	231.64	240.06	264.49	325.3	185.89	289.55	283.61	302.17	283.61	281.56	250.6	246.9	275.03
317	40.701	40.701	67.835	67.835	40.701	40.701	40.701	40.701	40.701	40.701	40.701	54.268	40.701	54.268
318	27.134	27.134	54.268	54.268	27.134	27.134	27.134	27.134	27.134	27.134	27.134	27.134	27.134	27.134
319	17.742	17.742	17.742	17.742	17.742	17.742	17.742	17.742	35.485	17.742	17.742	17.742	17.742	17.742
320	23.425	23.425	23.425	23.425	23.425	23.425	23.425	23.425	41.168	23.425	23.425	41.168	17.742	41.168
321	64.866	53.242	51.266	67.075	132.52	49.058	78.265	112.29	103.18	112.29	120.2	51.266	87.374	67.075
322	27.658	59.043	42.367	42.367	27.658	27.658	52.449	27.658	27.658	27.658	27.658	40.608	27.658	40.608
323	77.693	77.693	104.83	104.83	77.693	77.693	89.058	77.693	95.435	77.693	77.693	109	72.01	109
324	176.17	208.24	219.17	233.2	252.27	162.15	242.24	218.25	234.28	218.25	236.29	219.2	202.21	233.22
325	216	266	394	485	651	178	553	384	498	394	406	394	297	485
326	14	16	18	19	23	11	20	20	19	18	18	18	19	19
327	50	54	64	68	82	44	78	64	66	64	62	64	66	68
328	-5.0499	4.9373	5.0768	-2.5123	4.6018	-2.5058	0.8455	2.9329	0.8438	2.9585	3.4592	-5.1554	3.6329	-1.8212
329	0.9449	0.6658	-2.3666	-1.3208	3.3566	-0.2338	5.0929	-2.3664	-3.8755	1.366	-1.6314	-2.2098	1.1656	4.6811
330	0.2984	-0.9115	-1.0452	-1.072	0.103	0.1485	1.1701	-1.1007	0.6908	-0.9399	1.1109	0.4442	0.2719	1.5638
331	0	0	0	0	0	0	0	0	0	0	0	0	0	0
332	0	0	0	0	0	0	0	0	0	0	0	0	0	0
333	1067	1383.4	883.71	1100.7	2145.7	508	1107.5	1463.1	1741	1626.7	1844.8	1313.5	733.08	1658
334	178.22	358.14	432.15	404.7	669.87	271.72	535.26	363.86	647.74	317.06	335.94	493.41	286.39	540.85
335	69.361	72.836	145.79	205.88	118.54	97.094	261.98	113.89	196.96	110.49	222.6	98.472	201.12	119.34
336	5.2467	5.5765	16.447	7.4013	21.704	5.8337	17.213	12.625	10.706	5.5151	7.0557	3.3674	8.2334	14.383
337	-60660	-65143	-78080	-81719	-79576	-57069	-79613	-71389	-76669	-71433	-72329	-76103	-67195	-79649
338	-298214	-336335	-425703	-450730	-486119	-261688	-478538	-424060	-475592	-413265	-409700	-412043	-372725	-450586
339	-182.21	-177.24	-260.21	-313.62	-96.515	-177.22	-71.331	-150.59	-18.085	-188.96	-189.28	-222.74	-173.09	-182.68
340	-10.356	-9.2623	-4.5526	-5.306	-7.8865	-10.313	-7.8624	-8.1925	-11.28	-10.252	-8.5516	-10.193	-9.9209	-8.3626
341	10.356	9.2623	4.5526	5.306	7.8865	10.313	7.8624	8.1925	11.28	10.252	8.5516	10.193	9.9209	8.3626
342	0.6276	0.3217	2.7066	3.8397	-1.3634	0.7428	-1.3326	-0.6082	-3.1771	0.7165	0.7414	0.721	0.9515	-1.0052
343	361.79	385.6	366.07	388.73	471.27	321.96	418.63	426.04	472.5	428.2	435.97	401	369.11	422.57
344	125.99	138.13	121.48	99.745	220.82	146.96	206.2	127.68	198.15	126.86	144.93	175.22	105.83	191.23
345	99.06	151.58	146.01	148.2	165.96	104.22	124.46	83.644	104.19	79.554	124.94	138.51	88.632	133.64
346	138.15	107.46	104.55	147.33	262.45	80.622	170.28	214.99	170.16	221.78	166.84	94.37	175.48	99.69
347	223.64	278.15	261.52	241.4	208.82	241.33	248.35	211.04	302.33	206.41	269.13	306.63	193.63	322.88
348	583.73	696.57	672.86	552.49	1220.5	680.85	1134.1	591.55	1285.2	587.74	738.15	1051.9	482.92	1148
349	458.95	764.41	954.75	969.1	917.27	482.83	684.52	387.52	571.57	368.57	636.34	831.46	404.43	802.24
350	26.935	13.451	24.532	48.458	54.859	42.741	81.744	44.039	93.96	47.305	19.99	36.716	17.201	57.595
351	124.79	67.834	281.89	416.61	303.2	198.02	449.59	204.03	713.61	219.16	101.81	220.41	78.487	345.74
352	1.1257	1.1781	1.2427	1.2225	1.0544	1.1728	1.1419	1.0412	1.0401	1.0525	1.1139	1.1873	1.108	1.1468
353	5.1462	5.0647	5.698	3.034	5.6968	2.5211	5.2936	3.926	4.026	3.3914	3.9827	5.6267	3.8249	5.2607
354	29.058	31.545	23.833	-10.78	46.86	-2.9671	27.986	30.645	35.118	16.132	11.756	18.376	20.28	17.755
355	6.0829	5.8502	12.798	9.6686	6.9951	13.201	4.1981	12.219	7.4816	5.0332	4.2227	8.3728	11.078	16.408
356	-3.007	0.2913	-34.251	-111.12	-7.3527	-57.107	-16.811	-19.309	8.9997	-48.041	-56.121	-30.123	-56.406	-38.711
357	17.001	19.357	0.8347	-46.71	29.056	-22.83	10.768	9.9999	25.637	-7.104	-14.274	-4.0781	-16.509	-6.4419
358	0.0583	0.1337	0.3942	1.9358	0.0815	0.059	0.5322	0.1675	0.4928	1.5046	1.3379	0.0992	0.7796	0.2096
359	-93.815	-93.702	-173.34	-108.11	-90.292	-55.953	-106.21	-72.424	-130.44	-61.498	-63.928	-100.04	-61.787	-96.333
360	-0.132	-0.0988	0.2633	0.4643	-0.3394	-0.1352	0.5065	-0.0516	0.1664	0.2115	0.1079	-0.1871	-0.0582	-0.9882
361	2.4698	2.6733	4.6201	7.3372	4.4781	3.1867	4.0384	4.7748	2.8204	5.8219	5.4702	2.891	4.8475	5.131
362	0.0021	1.1364	0.0019	0.0038	0.0063	0.003	0.0043	0.0035	0.0051	0.0039	0.0044	0.0035	0.0034	0.0042
363	3.5778	3.6292	4.9229	16.524	6.5889	3.5518	7.9426	3.5356	-1.48	10.665	14.891	11.279	20.142	3.437
364	20.008	19.066	35.086	64.415	36.408	34.277	27.579	29.308	16.637	40.937	41.847	26.044	39.897	32.269

Table S12 Amino acid descriptors (2D) used in this study.

	P	Q	Q	Q	Q	Q	Q	Q	Q	Q	Q	Q	Q	Q	Q	Q	Q	Q	Q	Q	Q	R	R	R	R	R	R	R	R	R	R	R	R	R	R	R	R	R	R	R	R	S	S	S	S	S	S	S	S	S	S	S	S	S	S
	Y	A	C	D	E	F	G	H	I	K	L	M	N	P	Q	R	S	T	V	W	Y	A	C	D	E	F	G	H	I	K	L	M	N	P	Q	R	S	T	V	W	Y	A	C	D	E	F	G	H	I	K	L	M	N	P	Q
No.	PY	QA	QC	QD	QE	QF	QG	QH	QI	QK	QL	QM	QN	QP	QQ	QR	QS	QT	QV	QW	QY	RA	RC	RD	RE	RF	RG	RH	RI	RK	RL	RM	RN	RP	RQ	RR	RS	RT	RV	RW	RY	SA	SC	SD	SE	SF	SG	SH	SI	SK	SL	SM	SN	SP	SQ
365	0.3539	0.3421	0.3914	0.3718	0.3664	0.4302	0.4447	0.5136	0.276	0.4239	0.2785	0.3465	0.4508	0.3102	0.4827	0.4984	0.3827	0.3527	0.3096	0.472	0.4648	0.4315	0.4606	0.3607	0.3806	0.5375	0.5238	0.5541	0.3695	0.4791	0.3611	0.3956	0.4774	0.4238	0.4933	0.5437	0.4863	0.4184	0.4074	0.5102	0.5133	0.3482	0.3582	0.3318	0.2566	0.4686	0.4565	0.4926	0.2997	0.4194	0.2963	0.3324	0.437	0.2867	0.4525
366	0.2943	0.2649	0.3694	0.4214	0.3721	0.3728	0.2957	0.2742	0.2071	0.1879	0.2346	0.2721	0.3248	0.2499	0.3176	0.2025	0.2873	0.2559	0.2569	0.3611	0.3636	0.213	0.2859	0.2815	0.3038	0.2784	0.2206	0.2037	0.1573	0.1549	0.1618	0.2133	0.2393	0.1827	0.2502	0.1769	0.2425	0.2054	0.1693	0.2919	0.2851	0.2738	0.3931	0.3989	0.3813	0.3522	0.3237	0.2973	0.1963	0.2205	0.1858	0.2866	0.3454	0.2401	0.3162
367	0.6294	0.4017	0.2754	0.2424	0.282	0.563	0.2996	0.3702	0.5293	0.3982	0.4925	0.3919	0.2448	0.4493	0.2051	0.3086	0.3504	0.405	0.4365	0.4727	0.4516	0.3555	0.2994	0.373	0.3293	0.4583	0.2793	0.3989	0.4742	0.3669	0.4772	0.3921	0.2988	0.3945	0.2598	0.2794	0.2734	0.3798	0.4233	0.4648	0.4383	0.3818	0.2787	0.2856	0.379	0.5569	0.2504	0.4067	0.5046	0.3601	0.5179	0.3827	0.2353	0.4754	0.2359
368	0.3706	0.5983	0.7246	0.7576	0.718	0.437	0.7004	0.6298	0.4707	0.6018	0.5075	0.6081	0.7552	0.5507	0.7949	0.6914	0.6496	0.595	0.5635	0.5273	0.5484	0.6445	0.7006	0.627	0.6707	0.5417	0.7207	0.6011	0.5258	0.6331	0.5228	0.6079	0.7012	0.6055	0.7402	0.7206	0.7266	0.6202	0.5767	0.5352	0.5617	0.6182	0.7213	0.7144	0.621	0.4431	0.7496	0.5933	0.4954	0.6399	0.4821	0.6173	0.7647	0.5246	0.7641
369	1.8694	1.8208	2.2441	2.3162	2.2822	2.6745	2.3674	3.179	1.469	3.042	1.4823	2.004	3.0171	1.6296	3.2305	4.5364	2.2971	2.1174	1.648	3.0947	3.0674	3.3362	3.7498	3.1158	3.2869	4.636	4.0497	4.7641	2.8569	4.5913	2.7914	3.2406	4.345	3.2471	4.4896	6.2569	4.0901	3.5194	3.1494	4.5741	4.6235	1.6134	1.8065	1.838	1.4213	2.5897	2.1147	2.7091	1.3885	2.72	1.3726	1.6931	2.6231	1.3083	2.7166
370	1.5547	1.4102	2.1176	3.0467	2.6899	2.318	1.5738	1.6975	1.1021	1.1605	1.2488	1.5738	2.1737	1.3125	2.1256	1.6407	1.7245	1.5363	1.3674	2.3681	2.3991	1.4335	2.0419	2.4311	2.624	2.1229	1.4852	1.5478	1.0588	1.1751	1.0888	1.534	1.9386	1.2173	2.0267	1.6825	1.7972	1.5221	1.1395	2.3251	2.283	1.2685	1.9824	2.6081	2.493	1.9464	1.4997	1.6351	0.9096	1.2097	0.8608	1.4596	2.0735	1.0957	1.8984
371	0.0907	0.1781	0.0817	0.237	0.1372	0.1699	0.105	0.1398	0.228	0.0466	0.1592	0.1271	0.0778	0.2385	0.1258	0.0953	0.1487	0.1549	0.1765	0.0456	0.0922	0.1781	0.1212	0.2661	0.0775	0.1659	0.1181	0.0737	0.1351	0.0714	0.0985	0.0879	0.0969	0.1155	0.0618	0.1074	0.0874	0.085	0.0787	0.1084	0.1191	0.1324	0.1218	0.2486	0.1838	0.0755	0.4088	0.2318	0.0991	0.0919	0.1069	0.1292	0.1588	0.2291	0.1056
372	22.058	19.267	5.35	8.9613	13.753	14.963	9.2326	20.079	16.692	29.367	4.7011	18.949	7.3771	18.494	10.006	22.223	16.552	6.2227	11.378	12.388	8.6215	12.595	21.768	7.2594	34.841	36.711	24.481	9.9314	28.546	10.504	27.757	28.196	18.56	14.014	27.445	12.812	26.117	39.459	17.23	39.977	27.062	13.807	15.366	20.705	8.6139	23.022	5.9059	19.495	15.33	17.784	3.137	4.9814	5.6142	7.4918	19.829
373	-86368	-72492	-77843	-90085	-93627	-91459	-68966	-91520	-83309	-88650	-83384	-84977	-88029	-79042	-91583	-98206	-79931	-83606	-79706	-102460	-98976	-79065	-84403	-96739	-100290	-98012	-75522	-98155	-89857	-95129	-89943	-91544	-94619	-85620	-98141	-104687	-86557	-90069	-86279	-109010	-105537	-60848	-66120	-78344	-82023	-79804	-57309	-79868	-71653	-76936	-71736	-73409	-76398	-67465	-79924
374	-550609	-404294	-425765	-522422	-561789	-613948	-347280	-584723	-534072	-558143	-524107	-520837	-510420	-483464	-558860	-640675	-451108	-498098	-486953	-697740	-641136	-512802	-524899	-648334	-639101	-691501	-426394	-676182	-625952	-684518	-600525	-625140	-618887	-594384	-648685	-764404	-517622	-565939	-603937	-819341	-743271	-300555	-336459	-412495	-449630	-475958	-257373	-467209	-417383	-454438	-406059	-403232	-403418	-369746	-443829
375	-50.702	-81.385	-154.77	-272.37	-211.61	-43.143	-159.52	-26.556	-86.409	6.282	-164.99	-81.411	-168.68	-74.096	-119.84	18.952	-128.22	-203.34	-87.521	-37.58	-171.42	108.25	49.546	-163.74	-112.36	171.74	47.98	98.381	124.16	291.04	37.611	115.09	3.202	104.18	85.852	301.72	4.0267	96.315	100.25	185.24	29.074	-97.925	-92.108	-194.97	-259.29	-52.675	-165.94	-49.032	-95.736	59.872	-180.45	-178.05	-201.65	-166.05	-136.84
376	-7.3221	-7.3909	-9.9093	-5.0424	-4.1857	-8.0488	-10.466	-7.3109	-8.0524	-12.426	-10.516	-7.6954	-10.66	-7.5463	-8.2193	-12.101	-8.1452	-10.565	-8.2397	-7.9166	-9.2693	-11.149	-12.198	-9.738	-6.5891	-9.3731	-12.494	-12.023	-9.8629	-12.977	-12.203	-10.355	-12.813	-10.995	-10.018	-12.859	-12.367	-9.1709	-11.023	-9.7475	-10.695	-8.2234	-8.3043	-4.2722	-4.9984	-7.4018	-10.512	-7.4415	-7.8672	-10.244	-10.341	-9.668	-10.675	-10.249	-7.9579
377	7.3221	7.3909	9.9093	5.0424	4.1857	8.0488	10.466	7.3109	8.0524	12.426	10.516	7.6954	10.66	7.5463	8.2193	12.101	8.1452	10.565	8.2397	7.9166	9.2693	11.149	12.198	9.738	6.5891	9.3731	12.494	12.023	9.8629	12.977	12.203	10.355	12.813	10.995	10.018	12.859	12.367	9.1709	11.023	9.7475	10.695	8.2234	8.3043	4.2722	4.9984	7.4018	10.512	7.4415	7.8672	10.244	10.341	9.668	10.675	10.249	7.9579
378	-1.6598	-1.4862	0.6948	3.5763	2.2574	-1.7809	0.8577	-1.3033	-0.6912	-4.4598	0.7823	-1.8549	0.4589	-1.674	-1.553	-4.4945	-0.9364	0.6959	-1.149	-0.9962	-0.2831	-4.1947	-4.7325	0.2938	-2.8269	-4.8811	-4.7577	-3.8069	-4.5096	-5.8323	-4.7505	-4.7189	-4.3616	-4.289	-4.4203	-5.9696	-4.7415	-5.0244	-4.1436	-5.131	-4.687	-0.7976	-0.9872	1.9294	3.5944	-1.8446	0.7192	-1.6916	-1.4384	-4.0448	0.8285	0.7986	0.5615	0.5593	-1.4663
379	0.3221	0.3036	0.1604	0.4178	0.245	0.3291	0.1663	0.3416	0.2974	0.1691	0.316	0.2472	0.1553	0.4955	0.2834	0.2239	0.3008	0.4062	0.3888	0.1494	0.1615	0.2765	0.2966	0.5121	0.2062	0.3188	0.1604	0.2665	0.2662	0.2548	0.1786	0.331	0.2964	0.2737	0.1884	0.2331	0.1809	0.1591	0.2117	0.3222	0.1963	0.1987	0.2407	0.4392	0.4056	0.2792	0.4724	0.4849	0.2615	0.3535	0.22	0.2533	0.3274	0.4776	0.3002
380	0.7511	0.8762	0.9678	0.7384	0.9354	0.9606	0.9694	0.7838	0.9432	0.8873	0.8702	0.8496	0.9221	0.802	0.8918	0.8679	0.8679	0.7841	0.9033	0.9028	0.9576	0.946	0.8594	0.8663	0.9007	0.8964	0.9439	0.8298	0.923	0.8682	0.9391	0.8047	0.8113	0.8827	0.891	0.9256	0.9393	0.9441	0.8871	0.8235	0.9853	0.9125	0.8427	0.7824	0.8679	0.8048	0.7766	0.8339	0.8631	0.8113	0.8937	0.95	0.7815	0.9164	0.8083
381	20.635	15.965	8.5233	16.642	17.873	14.881	7.4976	16.786	20.455	27.646	5.8544	17.954	7.4472	16.943	7.8168	24.833	16.154	8.5448	6.8622	10.006	8.0932	18.195	21.786	12.222	31.499	40.39	21.5	7.9294	13.44	13.623	26.277	24.69	16.348	25.3	14.288	15.371	26.788	26.801	21.868	31.25	30.485	10.576	13.325	18.14	8.2258	19.752	5.6227	18.435	11.196	18.186	5.7787	7.0485	9.8302	5.9818	17.524
382	-79189	-65500	-69823	-81483	-84933	-83289	-62093	-81874	-75833	-80178	-75882	-76671	-79147	-71669	-82559	-87657	-72280	-75758	-72403	-92865	-90115	-70554	-74896	-86655	-90071	-88343	-67162	-86980	-80929	-85197	-80951	-81739	-84223	-76730	-87645	-92654	-77378	-80768	-77459	-97924	-95183	-55229	-59515	-71179	-74672	-73007	-51813	-71583	-65565	-69868	-65602	-66441	-68835	-61433	-72271
383	-543750	-401014	-424892	-531729	-553141	-615501	-348179	-587102	-534238	-559905	-526374	-527016	-504009	-488101	-560621	-641811	-452842	-496602	-492335	-689739	-640310	-492911	-521250	-665786	-652206	-678417	-427309	-703012	-656549	-681116	-595471	-622089	-618219	-570798	-678591	-735244	-514860	-582882	-574283	-834383	-740794	-296955	-332046	-415317	-458742	-475700	-256452	-466708	-415363	-452025	-407345	-401453	-403336	-370160	-441102
384	-98.695	-136.43	-148.34	-272.79	-282.13	-96.517	-171.83	-102.04	-141.8	-2.868	-190.57	-123.28	-215.23	-127.88	-183.82	-12.481	-181.88	-218.25	-150.39	-96.07	-190.45	76.285	30.489	-179.11	-152.09	128.3	24.675	59.986	29.87	216.49	7.7685	73.662	-24.342	77.91	-2.3014	259.83	-15.882	37.535	58.853	113.24	8.2875	-143.58	-133.04	-249.44	-296.75	-95.748	-174.3	-88.451	-153.58	2.157	-187.95	-174.12	-180.85	-172.56	-173.66
385	-8.0688	-8.0667	-9.2655	-5.2678	-4.9855	-8.8194	-10.122	-7.9451	-7.9828	-11.781	-9.9997	-8.2707	-9.6503	-8.1491	-9.0749	-11.151	-8.7353	-10.092	-9.0682	-8.1965	-9.3172	-10.841	-12.314	-8.8986	-7.4412	-9.8031	-12.249	-11.741	-11.942	-13.941	-11.913	-10.334	-12.3	-10.168	-11.707	-12.617	-12.139	-10.48	-10.496	-10.263	-10.722	-8.9063	-8.3975	-4.4769	-5.1355	-8.2529	-10.024	-7.9565	-8.7884	-11.118	-10.081	-8.9461	-9.0373	-9.7709	-8.5632
386	8.0688	8.0667	9.2655	5.2678	4.9855	8.8194	10.122	7.9451	7.9828	11.781	9.9997	8.2707	9.6503	8.1491	9.0749	11.151	8.7353	10.092	9.0682	8.1965	9.3172	10.841	12.314	8.8986	7.4412	9.8031	12.249	11.741	11.942	13.941	11.913	10.334	12.3	10.168	11.707	12.617	12.139	10.48	10.496	10.263	10.722	8.9063	8.3975	4.4769	5.1355	8.2529	10.024	7.9565	8.7884	11.118	10.081	8.9461	9.0373	9.7709	8.5632
387	-1.368	-0.7226	-0.0015	2.2097	2.795	-1.9731	0.2084	-0.5605	-1.1543	-4.7716	0.5941	-1.9643	0.2506	-1.7769	-0.9943	-5.1838	-0.6169	0.0476	-0.7766	-0.8008	0.0155	-4.5673	-5.2675	-1.157	-3.1517	-5.635	-5.2736	-3.4461	-4.5091	-6.0881	-5.2147	-4.6387	-4.5666	-4.7153	-3.9573	-6.7635	-5.3358	-4.9955	-4.4421	-5.449	-5.3111	-0.7178	-1.0556	2.2542	3.8481	-1.4519	0.3814	-1.8425	-0.7815	-4.5724	0.1972	0.1807	-0.7794	0.2023	-1.0953
388	3616	1932.9	3117.5	2342	3466.7	3346.2	1927.1	3069.5	2615.8	4213.8	2855.1	3279.4	3515.8	1962.9	3320.9	4766.3	1978.5	2081.2	2449.1	6545.7	5141.8	2634.7	3593.3	2262.7	4954.4	5555.3	2832.3	3731.2	3792.5	5412.1	4664.9	4628.6	3896.4	3353.8	5695.7	6477.3	4123.5	4334	3984.7	6288.6	6337.3	1314.6	1814.4	1461.6	1711.2	2934.1	876.82	1904.7	1940.9	2585.7	2054.2	2403.3	1905.4	1220.6	2318.2
389	1123.5	538.37	469.91	907.7	779.11	961.91	300.1	986.65	694.47	693.11	825.4	773.11	525.61	846.72	865.31	1020.5	548.85	771.86	830.93	953.11	783.82	655.46	988.58	974.43	969.66	1598.8	431.8	948.56	922.17	1298.9	786.67	1434.7	1095.9	851.33	1032.1	1399.1	703.55	655.69	803.77	1888.5	1140.3	247.49	419.19	577.89	610.55	786.25	368.33	796.66	477.75	844.46	427.54	552.69	591.61	487.04	660.2
390	2620.1	1553.9	2835.4	1604	2974.4	2807.7	1749.4	2263.9	2202.2	3636.3	2272.9	2657.6	3121.2	1370.4	2723	3954.9	1583.6	1490.1	1930.3	5759.1	4647	2243	2864.8	1648.3	4236.1	4496.2	2540.8	2954	3198.1	4426.5	4137.4	3487.9	2999.5	2745.6	4881.2	5554.5	3653.7	3891	3368.6	4826.9	5724.3	1136.3	1467.8	1029.5	1306.5	2266.2	605.57	1369.9	1577	1938.1	1737.2	2072.4	1412.2	934.44	1777.4
391	3488.3	1773.5	2929.6	2172.3	3179.9	2922.8	1804.6	2888.4	2334.9	4098.2	2612	3128.1	3384.8	1708.7	3053.5	4557.2	1824.5	1900.4	2136.9	6379.3	4852.8	2370.9	3333.3	1902.7	4703	5015.7	2691.9	3559.8	3464.8	5098.7	4405.6	4334.6	3697.4	3110.7	5478.1	6001.1	3889.8	4121.3	3797.1	5861.8	5809.9	1245.3	1741.8	1315.9	1505.4	2815.8	779.73	1642.8	1827	2388.9	1943.8	2181.6	1807	1019.7	2198.9
392	3.6045	2.983	3.5363	3	3.5554	3.3775	3.0796	3.2917	3.1761	3.9121	3.3182	3.4386	3.6755	2.8406	3.4796	3.9639	2.9126	2.9013	3.1599	4.4379	4.0771	3.2707	3.5929	2.7967	4.0415	4.1511	3.492	3.4562	3.6265	4.2166	4.022	3.8866	3.6698	3.5093	4.3332	4.4143	3.965	3.9604	3.8111	4.1713	4.3276	2.7316	2.9518	2.5824	2.7089	3.4104	2.3254	2.8041	2.9821	3.3222	3.0679	3.1892	2.9483	2.4569	3.1528
393	3.2964	2.5337	3.1709	2.2071	3.1108	3.0484	2.8383	2.7095	2.6876	4.0076	2.8699	2.9659	3.4363	2.3087	3.0483	3.6602	2.4549	2.4251	2.6345	4.2529	3.885	2.9055	3.0577	2.2194	3.7474	3.5878	3.0941	3.1698	3.3872	4.0489	3.9505	3.3985	3.2043	3.2383	4.0664	4.1679	3.6787	3.7174	3.6925	3.6724	4.0193	2.4525	2.5767	2.1519	2.3359	3.2605	1.7505	2.3626	2.9864	3.2141	3.1295	2.9936	2.5201	2.1197	2.7831
394	1.7768	1.5129	1.156	1.5975	1.3629	1.5295	1.0569	1.8515	1.5068	1.415	1.6397	1.7058	1.1536	1.6623	1.6339	1.6008	1.5268	1.7848	1.6926	1.7198	1.3581	1.4018	1.7329	1.6903	1.458	1.7937	1.2825	1.8187	1.5591	1.9218	1.3882	2.1556	1.8946	1.5803	1.6347	1.6578	1.3152	1.4128	1.6055	2.156	1.491	1.1651	1.1882	1.301	1.3026	1.5732	1.1914	1.5802	1.2795	1.5425	1.1781	1.2115	1.3772	1.3653	1.4872
395	0.993	1.0693	0.9061	1.0744	1.1522	1.2565	0.9197	1.013	1.2833	0.8647	1.1452	1.0572	0.9584	1.1276	1.081	1.1299	0.9468	0.9544	1.1067	0.9085	1.1797	1.2261	1.0643	1.1449	1.0429	1.4613	1.0632	0.8603	1.2451	1.0822	1.2399	1.0073	0.9976	1.1007	1.0112	1.3662	1.0876	1.084	1.0359	1.2089	1.3871	0.8924	0.8992	1.0729	1.0016	0.8957	1.1193	1.1375	0.9403	0.9741	1.0232	1.0759	1.0044	1.0145	0.9045
396	262.75	196.38	214	217.75	238.13	275.5	178.75	252.88	249.88	262.13	250.13	250	225.38	220.63	245.25	278.88	202	217.13	233.75	311.13	281.5	233.13	253	248.13	272.13	316.88	215.25	288.88	287.75	305.13	288.13	290.63	260	261	282	321.13	238.88	257.63	270.88	348.38	319.13	156.5	176.75	176.38	190.75	239.25	138.25	212.13	209.63	225.25	207.38	212.13	184.63	182.5	203.38
397	303.18	237.95	256.3	251.37	281.53	310.05	218.24	297.98	290.26	310.62	292.26	298.53	265.15	254.85	293.11	329.25	238.83	253.65	281.74	365.12	325.06	278.98	307.07	272.98	328.34	373.37	261.73	326.91	337.04	370.07	342.9	341.65	310.98	309.98	346.06	396.63	286.91	309.34	330.34	407.71	369.49	195.31	215.33	208.49	218.15	278.49	168.51	246.27	247.86	277.99	250.85	250.12	222.88	207.69	242.81
398	2.9955	1.1204	2.4634	2.2542	1.4631	3.5602	2.136	2.0982	2.2008	4.5397	2.9682	4.1322	2.7586	3.8675	1.7727	1.1533	2.8072	3.3557	2.9878	4.4337	2.9682	2.1703	2.5838	1.3624	1.7706	2.7526	3.1714	1.5062	2.9691	1.2666	3.5051	2.105	2.9367	2.1797	1.8164	1.8344	3.0851	2.0322	1.5579	3.3618	3.0275	2.0733	2.1052	2.9787	2.8193	4.0546	2.8778	3.8151	4.0929	2.8467	5.5142	4.0452	1.9211	3.8339	3.1257
399	0.1276	0.041	0.0397	0.023	0.0294	0.0481	0.0297	0.0401	0.0544	0.0439	0.0434	0.049	0.0286	0.0484	0.0239	0.0417	0.0296	0.035	0.0457	0.0435	0.0367	0.0457	0.0419	0.0314	0.0312	0.0551	0.0383	0.0506	0.0587	0.0528	0.0478	0.0623	0.0309	0.0432	0.0297	0.0389	0.0309	0.0359	0.0407	0.0465	0.0471	0.032	0.037	0.0214	0.023	0.0629	0.0286	0.0406	0.0504	0.032	0.0449	0.0589	0.0311	0.0504	0.031
400	2.8172	3.0593	3.0507	3.0954	3.0303	2.8849	3.1867	3.0043	2.9219	3.0219	2.9305	2.9179	3.0953	3.0479	3.0202	3.0121	3.0532	3.0858	2.9351	2.8866	2.9112	2.9599	2.9296	3.0281	2.9788	2.8564	3.0847	2.9436	2.8344	2.8602	2.8267	2.8414	2.9167	2.9973	2.9619	2.8893	3.0945	2.958	2.9084	2.7991	2.869	3.1038	3.0532	3.1761	3.1469	2.9053	3.3358	3.0655	2.9356	3.0308	2.9432	2.9118	3.1317	3.0869	3.144
401	2.34	2.6905	2.8174	2.8656	2.8075	2.5726	2.9156	2.7644	2.5034	2.771	2.4664	2.4388	2.8376	2.6389	2.7976	2.7929	2.7325	2.7722	2.5095	2.6194	2.6862	2.6556	2.6585	2.7421	2.7515	2.5979	2.7776	2.7057	2.3409	2.6461	2.3155	2.3883	2.6996	2.6687	2.754	2.6824	2.8474	2.6254	2.4376	2.5699	2.6563	2.6643	2.7554	2.8906	2.8922	2.5508	3.0373	2.7825	2.279	2.7823	2.2298	2.301	2.8274	2.6164	2.8828
402	1.4398	2.0624	2.3096	2.4041	2.3706	1.7218	2.3102	2.1028	1.7578	2.0135	1.8273	1.8042	2.3669	1.9542	2.3108	2.1978	2.1428	2.1528	1.9585	1.8334	1.9219	1.8992	2.0076	2.1432	2.1602	1.7775	2.0587	1.9629	1.6313	1.927	1.6031	1.6122	2.0427	1.9588	2.1515	1.9161	2.263	1.9632	1.8062	1.7553	1.8468	2.1228	2.2482	2.3658	2.3896	1.7077	2.3754	2.1072	1.7983	2.1655	1.7264	1.6198	2.3669	1.9172	2.3736
403	0.6936	1.2801	1.1829	1.6658	1.4453	1.0995	1.4114	1.2889	1.0644	1.0991	1.1301	1.0855	1.4514	1.1987	1.4886	1.2319	1.415	1.4169	1.1568	1.0832	1.1976	1.0932	1.1796	1.3577	1.3417	0.9751	1.2557	1.1078	0.9331	0.9725	0.9931	0.9135	1.2589	1.1403	1.2939	1.0992	1.3798	1.1734	1.0834	1.0245	1.0354	1.3415	1.2868	1.6745	1.6577	0.9622	1.5282	1.3407	1.0344	1.2793	1.0922	1.0211	1.4908	1.1996	1.4873
404	0.3294	0.7259	0.6441	1.0385	0.8245	0.62	0.7931	0.7481	0.5852	0.5698	0.6491	0.5916	0.8659	0.6847	0.8837	0.671	0.8966	0.7999	0.6127	0.6197	0.6584	0.6399	0.6124	0.8886	0.7688	0.5571	0.701	0.5711	0.5379	0.4988	0.5797	0.5311	0.7943	0.5979	0.7532	0.6276	0.8089	0.7034	0.616	0.5791	0.551	0.7779	0.6881	1.0717	0.9535	0.5588	0.9109	0.7211	0.6043	0.6245	0.6384	0.5493	0.8423	0.6684	0.9127
405	0.1263	0.3496	0.3134	0.5682	0.4175	0.3152	0.3608	0.3354	0.2606	0.274	0.3153	0.2676	0.4502	0.3256	0.4828	0.3011	0.5042	0.3863	0.2681	0.2856	0.3158	0.3584	0.2986	0.5189	0.3939	0.2738	0.3483	0.2606	0.2967	0.2675	0.2831	0.2638	0.4131	0.2785	0.3579	0.3266	0.4184	0.3144	0.3304	0.2729	0.2562	0.3523	0.2978	0.5493	0.4081	0.2696	0.4619	0.3207	0.3103	0.2494	0.3058	0.2599	0.4278	0.3065	0.4915
406	0.0503	0.141	0.1196	0.2504	0.1806	0.1285	0.1212	0.1346	0.1004	0.0879	0.0855	0.1082	0.2063	0.1144	0.2272	0.0926	0.2009	0.1343	0.0915	0.1034	0.1293	0.17	0.1153	0.2866	0.1706	0.1072	0.1375	0.1191	0.1511	0.13	0.1019	0.0995	0.1802	0.0794	0.1273	0.1548	0.1581	0.1135	0.1473	0.0927	0.1026	0.097	0.1258	0.2381	0.1563	0.1096	0.1833	0.109	0.1463	0.0801	0.1022	0.1236	0.1635	0.0833	0.219
407	0.0159	0.0439	0.0428	0.1031	0.0658	0.0472	0.0308	0.0435	0.0341	0.0207	0.0191	0.0336	0.0666	0.0314	0.0983	0.0216	0.0861	0.0329	0.0154	0.0295	0.0371	0.0822	0.0185	0.1489	0.0707	0.0359	0.0342	0.0483	0.0615	0.0439	0.0164	0.0224	0.077	0.0081	0.0314	0.0692	0.0434	0.0364	0.0338	0.0241	0.0321	0.0129	0.0358	0.1044	0.0574	0.0354	0.083	0.025	0.0659	0.0218	0.0208	0.0494	0.052	0.0259	0.0765
408	768.75	405	465.13	344.63	426.88	567.75	328.13	495.13	504.13	554.63	479	554.25	444.38	442.75	398.13	555.13	365.88	372.13	427	683.88	580.25	461.25	492.38	426.88	506.75	715.63	428.75	578.13	599.88	653.75	590.63	694.75	481.63	479.5	542.13	638.13	430.75	502.25	518.13	702.5	692.13	286.38	349.25	281.13	296.63	630.63	234.5	375.13	434.88	366.13	428	505	342.25	361.38	349.13
409	441.38	224.63	260.25	192.63	245.25	315.38	181.38	275.13	277.25	320.38	269.13	307.75	252.88	250.63	221.13	317.38	193.75	210.5	239.25	395.5	328.75	260.5	264.25	229.25	286.88	416.38	243.75	325.38	349.13	380.75	325.38	397.13	271.88	271.38	316.5	369.13	246.13	280.63	285.13	408.25	413.5	148.38	182.5	143	150.88	354.5	132.38	205.75	241.75	200.75	231	267.75	189.13	192.13	198.75
410	310.88	159.25	178.88	127	170.5	217.75	123.38	189.13	199.13	223.13	194.38	218.38	176.75	175.63	152.13	221.75	133.25	146.63	178.5	276.88	226.25	186	187.38	149.25	201.5	293.25	173.5	220.13	259	268.88	243.88	286.13	185.25	190.63	221	262	176.75	202.38	209	288.38	288.25	108.5	125.38	96.125	97.75	236.38	92.25	142.25	186.5	138.13	178.13	195.5	132	137.63	142.63
411	247.25	124	126	97.375	128	177	95.5	144.63	162.75	172.5	159.5	179.5	132.25	140.75	117.5	166.5	101.63	112.75	139.5	222.88	176	148.75	145.13	119	151.75	235.38	136.75	172.13	212.63	205.38	207.88	235.38	141	151.63	166	201.88	132.88	158.63	172.38	233.25	229.13	84.125	92.5	70.125	75.125	191.63	67.125	110.63	157.63	104.5	149.5	169.25	101	114.38	108.13
412	208.63	99.25	90.5	74.875	97.75	152.75	72.625	118.25	134.25	138	132.75	150.13	104	114.5	88.125	129.13	83.375	88.25	109.75	184.38	140.5	117.38	106.38	95.125	114.63	192	110.13	143	177.63	156.75	169.25	194.5	112	123.5	126.75	161.38	104.25	126.5	138.63	191.63	186.5	65.625	67.125	55.5	53.5	160.25	53	89.5	130.25	74.375	121.75	143.38	77	92.375	84.25
413	173.5	76.375	69.875	57.5	75.875	126.88	54.875	97.25	104.88	110.75	98	104.13	83.75	99.625	72.75	97.75	67.625	65.875	78	153.13	112	89.875	78.125	74	89.75	163	88	115.63	131.13	121.63	122.5	146	87.25	97.625	100.88	134.25	86	92.875	104.5	157.25	149.5	48.375	52.25	42.125	38	137.88	40.875	69	83.5	52.375	71.875	106.5	57.75	73	68.75
414	114.5	49.25	48	43.375	55.25	77.5	36	69.75	58.125	74.375	51.25	65.375	61.375	60.75	49.625	68.25	42	40.75	43.375	99.625	77.625	52.75	41.875	50.125	63.5	110.63	55.5	80.875	79.875	84.125	62.5	96	64.25	53.375	70.5	97.375	62.875	66.375	56.125	107.88	108.25	25.625	34.5	29.75	20.75	88.375	31	46.375	43.625	28.875	29.125	50.875	38.375	37.625	49.875
415	64.75	27.625	30.375	27.125	36.375	31.25	16.75	39.875	29.125	40.125	23.375	31.625	42.5	34.875	32.5	41.125	23.75	19.75	23.625	55.875	35.5	26	17.875	25.875	38	59.125	32.125	41.5	40.375	55.125	27.75	44.75	35.25	26.625	45.625	53.375	40.25	38	32.5	56.125	61.875	15.375	21.625	15.5	7.875	50	18	23.5	23.25	11.625	15	18.75	25.25	17.875	35.625
416	0.5	0.866	6.8191	3.1623	0.5	0.7071	1.118	0.7071	7.6322	0.5	0.5	0.5	1.118	0.5	0.5	0.866	2.4495	0.866	1.2247	0.5	0.5	0.5	6.5955	0.7071	6.8739	7.8899	5.8949	0.5	0.5	0.5	0.5	0.7071	0.7071	0.7071	6.5383	1.2247	0.5	5.6789	0.7071	0.5	6.8191	0.5	0.5	0.5	6.2048	0.5	0.5	0.5	0.5	0.7071	6.265	0.5	0.5	0.7071	7.1063
417	0.5	1.5	0.866	1.2247	0.7071	0.7071	0.5	0.5	7.4498	0.5	0.866	8.3815	0.7071	0.866	0.866	6.9462	0.7071	1.2247	0.866	0.7071	0.5	0.866	6.8739	1.4142	6.5955	7.9687	0.5	1	1.5	6.5765	1.5811	0.5	0.5	1.118	6.7454	2.0616	0.5	4.7697	0.5	0.7071	6.7454	0.5	0.7071	1.2247	6.5765	7.1589	0.5	1.118	0.5	0.7071	6.8191	0.866	6.5192	0.5	3.0822
418	1	0.7071	7.3655	4.3012	0.5	0.7071	0.7071	0.5	0.5	1	0.7071	8.6603	0.5	0.7071	0.7071	6.9642	2.3452	0.5	0.5	0.866	0.7071	0.7071	0.5	0.7071	0.5	0.5	5.5227	0.5	1.2247	6.6708	1.118	0.5	1.118	0.5	0.5	0.866	0.7071	1.2247	0.866	0.5	0.7071	0.7071	0.5	1.118	0.5	6.9642	0.7071	0.7071	1	1.4142	0.866	0.7071	6.5765	0.5	6.5955
419	0.5	0.866	0.5	0.5	0.5	0.7071	0.5	1.118	0.7071	0.5	0.5	0.866	0.7071	0.866	8.5147	0.7071	0.7071	9.9373	0.7071	0.866	0.7071	0.5	0.5	0.7071	0.5	0.5	7.433	0.866	0.5	0.5	0.5	0.7071	0.5	0.7071	0.7071	0.7071	8.441	6.7268	0.7071	1.2247	0.5	0.5	2.4495	0.5	0.5	0.5	0.7071	1.118	0.7071	0.7071	0.5	3.3541	0.5	4.9244	0.5
420	0.5	0.5	0.7071	0.5	0.5	0.7071	7.6485	0.5	0.5	0.5	0.866	0.5	0.7071	1.2247	8.5	1.118	6.7454	0.5	0.866	1.2247	0.5	0.5	1.118	0.7071	1.118	7.2284	0.5	5.6125	0.5	0.7071	0.7071	0.5	0.5	0.866	7.8102	0.5	8.3815	6.4031	0.5	0.7071	8.0467	5.5227	0.5	0.5	0.866	1	8.7464	0.7071	0.5	0.7071	0.5	0.7071	0.866	0.7071	6.4031
421	0.7071	0.7071	0.5	0.7071	1	0.7071	7.6974	0.7071	1.118	1	0.7071	1.2247	1.4142	0.5	0.5	0.866	7.1414	10.173	0.5	0.5	0.5	0.7071	0.7071	1.4142	0.7071	7.4833	7.6485	5.0249	0.7071	0.5	0.5	0.5	0.7071	0.5	7.7782	0.5	0.7071	0.5	1.118	0.7071	7.9057	5.5	2.8723	1	0.7071	0.5	8.7178	0.5	0.5	1.4142	0.7071	3.9051	1.2247	5.4083	6.3443
422	-2.7223	-2.9336	-2.1425	-2.4342	-3.2492	-2.6246	-2.1123	-2.3497	-2.4895	-2.4444	-2.4777	-2.2984	-2.8838	-2.5817	-2.5058	-2.6181	-2.1754	-2.8005	-2.6368	-2.5486	-2.6001	-2.8065	-2.296	-2.2255	-2.538	-2.4356	-2.3706	-2.5006	-2.8591	-2.3892	-2.2165	-2.7211	-2.2484	-2.4703	-2.633	-2.5958	-2.8266	-2.3367	-2.8855	-2.8475	-3.1576	-2.22	-2.2707	-2.0823	-2.4314	-2.2913	-2.4269	-2.9774	-2.6064	-2.2452	-2.2004	-2.392	-2.5255	-2.4006	-2.3623
423	-2.6565	-2.8804	-2.129	-2.4332	-3.1457	-2.4651	-2.0684	-2.2872	-2.4348	-2.3975	-2.4574	-2.2266	-2.7691	-2.5412	-2.4398	-2.5883	-2.1614	-2.5438	-2.6082	-2.5241	-2.5094	-2.5856	-2.1183	-2.1923	-2.5175	-2.3779	-2.3606	-2.4308	-2.7995	-2.3482	-2.1942	-2.7079	-2.2396	-2.4376	-2.6113	-2.5557	-2.7653	-2.3019	-2.5188	-2.7998	-3.108	-2.2132	-2.255	-2.0418	-2.2266	-2.2752	-2.3776	-2.8599	-2.5794	-2.2096	-2.1966	-2.3866	-2.4719	-2.3459	-2.3588
424	-2.6066	-2.845	-2.0991	-2.422	-2.8688	-2.4535	-2.0534	-2.2633	-2.3897	-2.3346	-2.3866	-2.2034	-2.7145	-2.4734	-2.3885	-2.5315	-2.1482	-2.5335	-2.5962	-2.5019	-2.4748	-2.5567	-2.0759	-2.1692	-2.4467	-2.3723	-2.3397	-2.3798	-2.7934	-2.3325	-2.1573	-2.6934	-2.196	-2.4327	-2.5894	-2.4552	-2.658	-2.2899	-2.4384	-2.6257	-2.9823	-2.1871	-2.2499	-2.037	-2.1605	-2.2582	-2.3769	-2.7393	-2.5514	-2.1957	-2.185	-2.3532	-2.4634	-2.3407	-2.3397
425	-7.5612	-7.8882	-7.9393	-10.082	-8.3325	-7.9075	-7.0984	-7.8965	-7.929	-7.1989	-6.9235	-8.4547	-8.0397	-7.6273	-8.922	-6.9555	-8.944	-7.1942	-7.5641	-8.4771	-7.609	-11.459	-6.9422	-9.7574	-11.393	-8.1897	-7.1803	-7.9173	-10.578	-9.7948	-7.1958	-7.8365	-10.751	-6.518	-7.5196	-9.7291	-7.2329	-8.1146	-8.3035	-7.881	-8.0365	-6.8481	-7.0414	-8.4405	-7.7804	-7.6108	-8.0104	-7.6228	-8.8461	-8.2734	-7.0425	-7.2555	-10.159	-7.0014	-7.6129
426	-7.2851	-7.6156	-7.4185	-9.5715	-8.1213	-7.683	-6.8027	-7.8122	-7.8242	-6.9167	-6.7342	-7.7237	-7.8665	-7.6177	-8.889	-6.9073	-8.6637	-6.8466	-7.114	-7.8424	-7.4401	-11.303	-6.9345	-9.596	-11.385	-8.1244	-7.133	-7.6353	-9.9852	-9.54	-7.1762	-7.6324	-10.294	-6.3668	-7.4401	-9.0537	-7.2047	-7.9099	-7.9466	-7.7081	-7.641	-6.472	-6.9405	-8.0375	-7.1855	-7.57	-7.8326	-7.3798	-8.404	-7.5663	-6.969	-7.25	-9.7462	-6.9	-7.4837
427	-6.9974	-7.3803	-7.4097	-9.4604	-7.795	-7.6725	-6.7684	-7.7675	-7.6948	-6.8377	-6.6998	-7.5677	-7.6425	-7.4844	-8.6459	-6.8066	-8.6413	-6.8226	-6.6032	-7.7409	-7.2668	-11.187	-6.8186	-9.3262	-11.336	-7.8064	-7.1307	-7.5779	-9.9041	-9.4207	-6.7942	-7.5336	-10.072	-6.3229	-7.291	-8.7939	-7.1333	-7.6619	-7.8849	-7.5492	-7.2611	-6.4694	-6.9368	-7.8252	-7.128	-7.2105	-7.4994	-7.1337	-8.312	-7.5071	-6.7943	-7.1693	-9.5838	-6.8025	-7.4274
428	1.2707	1.2321	1.2565	1.2283	1.2881	1.2507	1.2254	1.24	1.2234	1.2921	1.2577	1.2546	1.2775	1.1894	1.286	1.293	1.2214	1.2115	1.2574	1.3425	1.3176	1.266	1.2939	1.1685	1.3292	1.3372	1.2569	1.2346	1.2949	1.3748	1.3288	1.3218	1.3224	1.2577	1.3724	1.3726	1.3012	1.3157	1.3004	1.3364	1.3472	1.2208	1.2407	1.1915	1.2069	1.2633	1.1718	1.2134	1.232	1.3012	1.2407	1.2453	1.2426	1.1744	1.2414
429	309	275.13	261.25	365.63	383.13	285.63	300.5	268.25	265.5	247.63	296.5	239.25	304.25	279.13	260.13	238.63	306.63	305.25	310.63	271.38	308	209.75	235.88	243.5	364.25	261.38	226.25	175.63	211.13	179.25	211.75	214.38	254.25	242.88	270.75	190.75	296.38	271.13	185.13	236.25	268	304	277.75	328.75	416.5	245.75	311.5	301.75	250.63	278.38	258.75	270.25	327.38	286.63	286.25
430	379.88	341.13	402.63	507	546.38	403.88	383.75	338.75	352.13	302.38	395	314.63	409.25	357.63	380.25	330.5	387.5	421.88	417.63	389.13	440.5	255.38	339.5	337.38	495.25	312.25	282.38	223.13	254.88	228.13	273.13	259.13	328.75	311.88	356.25	231.5	387.88	339	252.5	320.13	364.38	388.75	410.75	463.63	577.38	307.5	386.63	432.38	317.38	381.63	339	329.63	429.13	380.25	403.75
431	283.38	282.75	337.13	454.25	449.25	315.38	298.75	319.75	272.5	263.88	315.75	281.13	383.13	300	374.75	276.88	329.88	344.25	314.88	333.13	364.88	204.13	262.25	296.88	384.13	236.13	224.88	217	192	200.63	210.25	205.5	301.13	240.75	315.88	195.13	322.75	270.38	218.88	247	278.5	296.38	318.25	393.75	458.63	275	312.75	338.5	282	317.63	281	281	362.25	303.25	364.38
432	120.75	166.25	157.25	269.88	233	168.25	158.88	189	146.38	145.5	169.25	151.75	217.75	164.88	233.75	140.38	194	195.5	154.13	186.75	199.25	121.63	119.5	180.63	201.63	124.5	122.13	129.88	110.38	102.63	114	110.13	172.88	120.25	184.13	113.63	179.13	154.88	122.38	121.75	138	158.63	156.5	231	240.88	158	167.88	174.75	151.38	153.38	153.5	142.88	199.13	153.88	208.13
433	47.25	82.25	76.375	142	115.88	85.625	75.25	92.5	71.125	72.625	85.25	71.75	114.63	80.75	122.38	61.125	107.13	96.125	72	94.25	95.125	69.5	48	106.13	94	65.875	57.75	62.75	60	53.375	59.25	54.5	91.25	53.625	89.375	56.75	88.25	76.5	61	59.875	63.125	80.5	70.875	124.38	113.63	76.875	84.75	79.125	78.5	69.375	74.875	68.25	100.88	68.875	117.13
434	18.375	38.75	35.625	69.75	54.875	42.125	34	42.375	31.375	35.875	37.75	33.625	57.125	35.25	67	25.875	56	41	29.625	42.75	45.25	42.25	19.375	58.625	42.375	28.125	28.125	31.75	34.25	30.125	24.375	23.625	41.25	22.75	38.5	30.5	40.375	29.625	30.125	30.125	28.125	31.875	31	57.5	43.5	35.25	40.125	32.125	39	30.125	32.25	30.875	47.375	27.5	56.625
435	5.625	14.25	14.25	30.25	22.625	16.875	12.125	16.875	11.875	11.375	10	13.125	26.125	12.375	29.875	7.875	22.75	13.75	9.625	15.25	17.75	20.875	8.125	31.625	18.75	10.875	10.75	13.875	19.75	14.75	9	8	17.75	5.125	13	12.5	14.5	10.125	14.625	10.625	10.5	8.875	11.625	23.875	15.75	12.5	14.75	10.75	16.875	9.25	10.375	14	17.25	7.625	23.375
436	2	4.25	4.75	12.25	7.5	5.375	2.625	4.125	3.75	2.25	2.375	4	6.875	2.875	12.5	1.5	10.125	3.25	2.25	4.125	5	10.75	2.125	16.875	7.625	3.75	2.875	5.875	9.625	5.125	1.5	2.625	8.875	0.375	3.625	7.75	4.5	3.625	3.875	3.25	4.375	1.25	3.625	8.75	5.25	3.75	6.375	2	7	2.625	2.25	4.75	5.75	2.25	7.375
437	0.1612	0.595	0.5311	1.2244	0.7918	0.4811	0.7862	0.581	0.4244	0.4235	0.517	0.4139	0.7709	0.5352	0.9803	0.4983	0.9447	0.7528	0.5175	0.4293	0.5177	0.5618	0.5183	0.9179	0.6948	0.3674	0.5829	0.4276	0.3933	0.3728	0.4352	0.3403	0.7557	0.4898	0.6425	0.5038	0.7532	0.5757	0.5311	0.4049	0.3669	0.8088	0.6411	1.3836	1.2174	0.3485	1.0393	0.6599	0.4739	0.6443	0.4933	0.399	0.8343	0.5386	0.9334
438	0.0748	0.3216	0.3085	0.7381	0.4639	0.2535	0.3992	0.3103	0.2097	0.2203	0.2484	0.2047	0.4641	0.2647	0.6043	0.2598	0.5904	0.3847	0.2482	0.2109	0.2805	0.3555	0.273	0.5788	0.4077	0.1917	0.3208	0.2324	0.2352	0.224	0.2032	0.1726	0.4477	0.2457	0.3381	0.3152	0.428	0.2774	0.2857	0.2031	0.1909	0.3878	0.3541	0.8414	0.589	0.1879	0.622	0.322	0.2554	0.305	0.2308	0.1891	0.479	0.2448	0.5491
439	0.6208	0.7766	1.013	1.3989	0.352	2.016	1.356	1.0262	0.552	1.4092	1.6862	1.4876	1.5244	1.7525	1.0622	0.7874	1.5541	1.9892	1.2781	2.0997	1.2871	0.7053	1.2399	0.4658	1.2647	1.3692	1.6508	0.3884	0.6858	0.3724	1.4682	0.8568	1.4736	1.0471	1.0509	0.9403	1.8358	1.1811	0.6782	1.8469	1.3911	0.8029	0.8058	1.7869	1.7644	1.9128	1.8109	2.2225	1.9143	1.3776	2.4398	1.9032	1.0414	1.7783	1.719
440	0.4635	0.6411	1.0254	1.4165	0.4021	2.0044	1.3654	1.1644	0.6272	1.4207	1.6369	1.4453	1.5821	1.7542	1.1138	0.9032	1.4481	2.0484	1.3093	2.1628	1.4096	0.8052	1.2921	0.5509	1.3228	1.3536	1.5918	0.3606	0.6076	0.5997	1.358	0.7257	1.6369	1.0642	1.0326	0.9403	2.0273	1.243	0.5607	1.6952	1.3002	0.8986	0.8141	1.9144	1.6849	1.8165	1.693	2.1957	1.7439	1.5656	2.2655	1.8673	1.0649	1.6806	1.8177
441	0.5441	0.4789	1.0629	1.4211	0.4155	1.934	1.3607	1.1981	0.6912	1.476	1.6139	1.6194	1.5572	1.9055	1.0938	0.9266	1.4844	2.0063	1.4377	2.082	1.389	0.7398	1.4481	0.6689	1.4442	1.2968	1.6257	0.3293	0.7595	0.6784	1.4756	0.9541	1.754	1.0105	1.0458	0.9825	2.2005	1.1419	0.8009	1.6942	1.2062	0.7719	0.7794	1.8341	1.6475	1.795	1.7652	2.2023	2.0613	1.634	2.5566	1.8904	1.0692	1.6578	1.8472
442	0.8213	0.5692	1.1728	1.4713	0.393	2.1605	1.4279	1.3723	0.77	1.5535	1.9232	2.1895	1.5862	2.2859	1.0513	0.9632	1.8129	2.1719	1.8137	2.3854	1.4146	0.759	1.7723	1.005	1.6628	1.4493	1.8186	0.3963	0.9934	0.7236	1.7577	1.2317	1.9499	1.1379	1.1083	0.9882	2.2502	1.2318	1.0813	1.8866	1.2639	0.6904	0.8165	1.8941	1.7282	2.1435	1.9649	2.4293	2.4933	1.8357	2.9999	2.16	1.2581	1.8736	1.9268
443	1.0089	0.7383	1.4268	1.9264	0.4786	2.4821	1.3409	1.3563	0.8582	1.7009	2.1158	2.5656	1.7332	2.6118	1.1446	1.023	1.9631	2.3618	2.0341	2.7134	1.6752	0.8788	2.1616	1.296	1.8941	1.6359	2.0421	0.4919	1.096	0.8846	2.0017	1.3797	2.278	1.3317	1.1288	1.0733	2.4567	1.3482	1.3728	2.2616	1.3516	0.827	1.1638	2.0925	2.219	2.553	2.1751	2.8118	2.8688	2.13	3.357	2.4765	1.4026	2.0298	2.0125
444	1.0942	0.8704	1.6749	2.1057	0.511	2.7805	1.4724	1.4015	0.9106	1.8235	2.101	2.4354	1.8806	2.8016	1.2681	1.2989	2.0276	2.4965	2.0051	2.9383	1.85	1.1103	2.5462	1.4075	2.1524	1.8349	2.2177	0.5105	0.784	1.2346	1.8854	0.9291	2.6582	1.3848	1.04	1.0662	2.6425	2.2743	1.012	2.5233	1.4584	1.3894	1.5074	2.2001	2.6794	2.7347	2.3962	3.2181	2.2552	2.1237	3.1905	2.4579	1.6499	2.2623	2.0976
445	0.8416	0.9822	1.7801	2.1704	0.6167	2.4799	1.5263	1.2377	0.6379	1.7553	1.691	2.1721	1.7708	2.3037	1.3629	1.2372	1.2167	2.8493	1.3148	2.8972	2.2594	1.8206	2.6989	1.0084	1.8474	1.5556	1.9482	0.4113	1.2929	1.5056	1.7672	0.5904	2.7226	1.5133	1.2434	1.0841	2.5318	2.7871	1.6131	2.3486	1.4835	2.7617	1.9913	2.704	2.4742	2.3155	2.3045	3.0706	1.1967	2.3652	2.0727	2.6277	1.7283	2.1187	2.0057
446	1.1564	0.6059	2.0993	1.578	0.9151	2.0722	2.2124	0.7172	1.2881	1.6075	2.8548	1.7086	1.8562	1.874	1.3266	1.4918	1.3309	2.5036	1.1113	2.4678	2.4394	1.8764	2.389	1.2685	1.4675	1.4186	1.7446	0.5655	1.7352	1.7192	2.5243	0.2336	2.8171	1.9724	1.185	1.4476	2.7675	2.4983	2.4707	2.2793	1.3001	3.1832	2.722	3.3133	1.6838	1.6721	1.9589	2.9483	1.1176	2.6541	1.8866	1.5937	1.6589	1.555	1.9216
447	0.3812	0.3964	0.7035	0.3581	0.3622	0.3094	0.2909	0.1282	0.2514	0.1849	0.53	0.7309	0.2845	0.5496	0.1843	0.3308	0.4349	0.0993	0.5498	0.6271	0.4257	0.2054	0.3944	0.3319	0.3041	0.7275	0.4512	0.2373	0.5866	0.2756	0.7671	0.314	0.4617	0.7019	0.2599	0.4189	0.501	0.4174	0.8311	0.6969	0.4095	0.4287	0.4632	0.2627	0.3999	0.2628	0.3285	0.3068	0.4319	0.2606	0.6412	0.5048	0.2351	0.5562	0.2165
448	1.1105	0.5922	0.7047	0.5456	0.4155	1.0162	0.3626	0.2665	0.3441	0.4844	1.0078	1.5847	0.4654	1.191	0.2844	0.3042	0.8835	0.4965	0.9388	1.3877	0.7259	0.2837	0.4379	0.5902	0.21	1.0744	0.8983	0.3551	1.0722	0.2428	1.5332	0.7662	0.4677	0.9652	0.3356	0.4557	0.2665	0.3481	1.2937	1.1144	0.7373	0.5312	0.5348	0.5505	0.6407	1.1074	0.5632	0.7061	1.3503	0.1672	1.7395	1.3571	0.4166	1.1551	0.3022
449	1.4377	0.5901	0.8446	0.7231	0.5254	1.4863	0.551	0.698	0.6372	1.0546	1.1861	2.0368	0.7311	1.6864	0.4803	0.3464	1.1238	0.9007	1.0642	2.0356	1.1867	0.6243	0.6978	0.7877	0.3301	1.4647	1.2496	0.4827	1.3279	0.2813	1.8086	0.8074	0.7805	1.0072	0.5907	0.5905	0.506	0.5276	1.1653	1.5053	1.2164	0.7003	0.6894	0.8238	0.7588	1.8509	0.8847	1.1916	1.574	0.298	1.9914	1.8671	0.6163	1.6229	0.6338
450	1.7954	0.6168	1.4982	0.815	0.8787	1.7264	0.7945	0.9269	1.079	1.9934	1.3529	2.2265	1.0947	1.8948	0.6688	0.4026	1.2227	1.3125	1.3696	2.3333	1.5464	1.1049	1.0631	0.8679	0.4182	1.6283	1.4817	0.7725	1.818	0.6545	1.9331	0.8958	1.1499	1.3001	0.7579	0.8459	0.8992	0.9314	0.9599	1.76	1.5963	1.0619	1.1221	1.0229	0.9679	2.0205	1.0615	1.515	1.8256	0.7846	2.5143	2.1749	0.7876	1.8955	1.0824
451	2.5056	0.6913	1.7764	0.854	1.0941	1.8578	1.0537	1.0138	1.6568	3.2544	1.5647	2.6117	1.2356	2.0645	0.6895	0.2828	1.3448	1.3719	1.9456	2.3871	1.612	1.5866	1.6368	0.893	0.5864	1.6472	1.5737	1.2298	2.2635	1.4715	2.0977	1.2453	1.467	1.6602	0.8101	1.0087	1.0599	1.0864	0.9007	1.9935	1.8889	1.407	1.4334	1.2361	1.3231	2.2644	1.1927	1.682	2.0674	1.4317	2.9805	2.3173	0.9895	2.2421	1.307
452	4.1291	0.9512	1.8286	0.8321	1.4039	2.2174	1.348	1.1089	2.3181	4.1402	1.9733	2.9945	1.3874	2.3272	0.6685	0.1685	1.4296	1.4685	2.6438	2.1655	2.0044	2.1018	2.0764	1.1771	0.9804	1.8197	1.8126	1.9151	2.7458	1.9574	2.2979	1.5599	1.7928	2.1685	0.9433	1.1589	1.326	1.524	0.7526	2.3439	2.2179	1.9431	1.7092	1.7563	2.057	2.2113	1.5126	1.8016	2.2698	1.9181	3.4666	2.5358	1.1743	2.7992	1.6067
453	5.0195	0.9634	2.0135	1.2171	2.0634	2.5921	1.8682	1.5172	2.5724	4.8547	2.599	3.4272	1.7344	3.0373	0.6459	1.0769	1.7923	1.3521	3.3907	2.2895	2.0971	2.9277	2.6839	1.4367	1.7301	1.7928	2.0904	2.567	2.814	2.3498	2.3395	2.4058	1.8611	2.8947	0.5847	1.169	1.8707	2.1526	0.6133	2.7488	2.2632	3.0256	1.9509	2.3332	2.8975	1.7357	1.8507	1.613	2.6891	2.7619	3.9738	3.0042	1.9464	3.7127	1.6294
454	4.742	1.5546	2.0664	1.9243	2.9288	2.3208	2.3186	1.5686	2.7449	5.0866	3.71	3.0874	1.8896	3.1154	0.8916	2.8046	1.856	1.6123	3.3029	3.2372	1.7966	3.5721	4.1852	1.5011	2.5606	1.7328	3.4793	3.5386	2.7563	2.3928	3.4475	4.0041	1.6001	4.0801	0.7679	1.3529	2.855	2.1935	2.3062	2.8665	2.442	3.9302	2.4115	2.802	2.7589	2.2271	1.6113	1.9856	3.2761	3.8267	4.4935	2.9519	2.9622	4.1644	2.2674
455	1.3787	1.3027	1.3129	1.3354	1.3235	1.4088	1.2733	1.3821	1.3957	1.3476	1.3637	1.3761	1.3095	1.3803	1.3342	1.3692	1.3111	1.3484	1.3452	1.3755	1.3561	1.3282	1.333	1.425	1.3321	1.3834	1.3104	1.4289	1.3802	1.3267	1.3485	1.3705	1.3168	1.3732	1.311	1.3562	1.3003	1.3192	1.3558	1.4237	1.3771	1.233	1.2515	1.2956	1.3035	1.3417	1.2294	1.3408	1.3247	1.2818	1.3129	1.3211	1.2725	1.3107	1.3
456	441.08	358.97	386.75	373.76	423.39	439.2	337.41	411.87	403.38	443.02	418.45	422.94	404.36	362.52	428.17	458.38	354.27	365.63	406.91	517.73	475.8	404.79	435.31	366.39	471.34	517.57	385.67	434.53	467.79	517.34	482.49	490.6	453.48	424.29	502.48	537.9	421.33	448.2	457.19	547.16	524.46	312.86	338.3	321.09	337.76	410.48	275.03	363.22	371.13	409.39	372.27	381.22	351.38	314.71	365.67
457	608.13	467.63	507.75	499.13	560.38	618.75	429.63	569.25	563	597	570.63	582	529.5	500.38	571.25	627.63	464.5	493	547.38	712.13	645.25	537.63	580.25	522.13	627.88	716	505.38	620.88	645.63	686.38	650.63	672.38	597.13	582.63	658.75	729.5	547.88	591.25	619.88	779	722.25	385.75	423.38	416	440.25	550.75	338.13	487	491.63	524.75	488.75	503.63	447.13	412.5	475.38
458	1177.8	1095.4	1203.5	1230.9	1347.5	1266.1	1105.4	1273	1137	1362.8	1207	1210.5	1327.9	1110.4	1351.6	1447	1099.1	1161.9	1166	1519.5	1411.3	1177.9	1287.4	1139.1	1463.8	1486.4	1216.1	1296.5	1270.4	1480.8	1308.4	1355.8	1363.4	1281.5	1556.9	1577.1	1372.9	1327	1295.4	1543.1	1524.5	934.25	1007.8	1057.1	1109.4	1157.6	934.75	1129.9	1017.6	1234.3	1029	1047.6	1132.6	941.5	1196.8
459	690.88	772.88	883.38	945.63	1019.6	856	819.38	903	754.75	958.13	822.88	814.13	986.88	769.38	1022.6	1049.5	787.38	846.63	821.75	1047	1008.5	818.88	922.5	827	1077.5	999.63	869	903	828.63	1031.6	863.25	871.5	989.88	899.75	1141	1116.6	1018.8	938.88	886.25	1065	1061.3	666.25	738.38	800.5	842.63	733.63	698.75	818	671.13	909.75	675.75	654.63	841.75	641	903
460	347.25	465.38	528.63	622.25	641.13	491.38	490.88	540.75	432.63	538.75	491.25	485	618.38	456.13	656.88	621.13	499.75	514.25	489.13	599.13	583	475.25	552.5	518.5	656.13	556.63	514.13	519.38	473.88	568.38	499.38	484.38	595	523.13	693	641.13	618.13	555.13	533.75	600.75	588.75	410.13	445.88	523.88	542.63	412.38	430	482.75	416.38	543.38	416.63	393	521	380.88	571.13
461	131.13	213	214.25	313.25	290.63	225.38	221.25	248.63	191.38	220.88	221.63	210	285	207.88	315.63	257	247	240.13	211.75	261	264.88	213.63	236	254.13	300	235.25	227.13	217.13	213.88	225.88	226.38	207.25	282.38	220.25	313	286.25	282.63	250.5	237.75	260.75	248.88	190.13	191.38	265.88	259.75	184.63	201.75	220.5	183.13	220.25	193.38	174.25	238.75	173.5	267
462	50.125	94.75	95	155.5	135	104.75	97	109.88	84.5	94.5	100.5	90.375	136.25	94	149.13	110.5	125.88	110.38	92.375	118.88	117.13	104.5	97.125	137	140	107.75	101.13	94.125	101.88	100.25	106.13	97.375	140	93.375	142	132	133.13	116.5	111	116.75	105.75	87.75	80.375	133	119	82.375	95.875	93.875	88.375	89	87.875	78	110.13	74.125	133.13
463	18.5	39.875	38.875	71.875	59.375	44.875	38.125	44.875	34.125	38	39.625	36.75	61.375	37.25	71	43.25	60	43.375	34.625	47	49.375	52.875	39.625	68.875	61.875	45.125	43.875	40	50	46	42.25	40.625	63.125	37.25	56.125	63.625	56.875	44	49.25	47.375	43.75	32.625	32.75	59	44.25	36	41.75	35.625	40.25	31.875	34.5	32	48.375	28	59.375
464	5.625	14.25	14.25	30.25	22.75	17.125	12.125	16.875	11.875	11.375	10	13.125	26.125	12.375	29.875	12.25	23.25	13.75	9.875	15.25	17.875	23	13.875	34.375	25.625	15.5	15	15	24	19.25	13.375	13.625	26.25	9.25	18.625	25.125	18.75	14.5	19.375	15.125	15.25	8.875	11.75	23.875	15.75	12.5	14.75	11.25	16.875	9.25	10.375	14	17.25	7.625	23.375
465	2	4.25	4.75	12.25	7.5	5.375	2.625	4.125	3.75	2.25	2.375	4	6.875	2.875	12.5	2.5	10.125	3.25	2.25	4.125	5	10.75	2.625	17.25	9.625	4.375	3.375	5.875	10.25	6.5	2	3.125	10.875	1	4.25	11.375	4.875	4.375	4.25	3.875	4.75	1.25	3.625	8.75	5.25	3.75	6.375	2	7	2.625	2.25	4.75	5.75	2.25	7.375
466	868.75	820.25	942.25	865.25	964.38	980.5	804.88	1004.8	871.5	1115.1	910.5	971.25	1023.6	831.25	1091.5	1208.4	792.5	856.63	855.38	1248.1	1103.3	968.13	1051.5	895.63	1099.5	1225	989.88	1120.9	1059.3	1301.5	1096.6	1141.4	1109.1	1038.6	1286.1	1386.4	1076.5	1055.9	1110.3	1306.9	1256.5	630.25	730	728.38	692.88	911.88	623.25	828.13	767	955.88	770.25	777.38	805.25	654.88	910.5
467	311	431.75	480.75	438.63	473.25	452.13	435.63	564.25	402.63	655.75	427.88	499.5	577.63	411.75	642.38	719	399.88	424.75	404.13	657.88	568	563.5	583	489.63	582.25	687.38	586.63	679.88	573.75	803.5	590.13	612.38	661.13	587.88	784.75	885.13	630.88	599.88	633.75	744.88	696.88	277.5	327.63	336.88	265.25	426.13	312.13	385.63	353.75	528.13	336.75	325	412.63	260.75	499.25
468	63.875	182.63	191.5	168	191.88	176	192.13	221	160.13	274.88	175.5	203.88	235.25	156.13	282.13	344.25	169.88	170	174.25	266	218.13	271.13	290.25	221.63	272	320.5	289.25	302.38	281.88	367.75	289.13	278.88	293.88	282.38	377.13	446	295.38	284.75	314.88	353.75	310.25	113.75	127.63	130.13	84	137.38	117.25	144.25	134.38	225.75	135.63	112	158.75	77.625	206.75
469	10.375	46.75	57	43.375	57.625	57.125	62.375	59.625	45	75.375	52.375	58.25	67.25	43	81.875	116.63	53	44.625	57.625	74.25	65.625	92	116.5	73.5	98.375	110.75	105	87.25	103.5	123.25	112.38	97.125	109.5	100	128.88	172.63	103.5	95.625	115.38	139	110.88	31.5	34.875	34.875	18.875	26.625	33.875	45.75	31.75	66.875	39.875	31.375	39.625	19.625	58.875
470	[illegible]	[illegible]	[illegible]	[illegible]	[illegible]	[illegible]	[illegible]	[illegible]	[illegible]	[illegible]	[illegible]	[illegible]	[illegible]	[illegible]	[illegible]	[illegible]	[illegible]	[illegible]	[illegible]	[illegible]	[illegible]	[illegible]	[illegible]	[illegible]	[illegible]	[illegible]	[illegible]	[illegible]	[illegible]	[illegible]	[illegible]	[illegible]	[illegible]	[illegible]	[illegible]	[illegible]	[illegible]	[illegible]	[illegible]	[illegible]	[illegible]	[illegible]	[illegible]	[illegible]	[illegible]	[illegible]	[illegible]	[illegible]	[illegible]	[illegible]	[illegible]	[illegible]	[illegible]	[illegible]	[illegible]
471	[illegible]	[illegible]	[illegible]	[illegible]	[illegible]	[illegible]	[illegible]	[illegible]	[illegible]	[illegible]	[illegible]	[illegible]	[illegible]	[illegible]	[illegible]	[illegible]	[illegible]	[illegible]	[illegible]	[illegible]	[illegible]	[illegible]	[illegible]	[illegible]	[illegible]	[illegible]	[illegible]	[illegible]	[illegible]	[illegible]	[illegible]	[illegible]	[illegible]	[illegible]	[illegible]	[illegible]	[illegible]	[illegible]	[illegible]	[illegible]	[illegible]	[illegible]	[illegible]	[illegible]	[illegible]	[illegible]	[illegible]	[illegible]	[illegible]	[illegible]	[illegible]	[illegible]	[illegible]	[illegible]	[illegible]
472	[illegible]	[illegible]	[illegible]	[illegible]	[illegible]	[illegible]	[illegible]	[illegible]	[illegible]	[illegible]	[illegible]	[illegible]	[illegible]	[illegible]	[illegible]	[illegible]	[illegible]	[illegible]	[illegible]	[illegible]	[illegible]	[illegible]	[illegible]	[illegible]	[illegible]	[illegible]	[illegible]	[illegible]	[illegible]	[illegible]	[illegible]	[illegible]	[illegible]	[illegible]	[illegible]	[illegible]	[illegible]	[illegible]	[illegible]	[illegible]	[illegible]	[illegible]	[illegible]	[illegible]	[illegible]	[illegible]	[illegible]	[illegible]	[illegible]	[illegible]	[illegible]	[illegible]	[illegible]	[illegible]	[illegible]
473	[illegible]	[illegible]	[illegible]	[illegible]	[illegible]	[illegible]	[illegible]	[illegible]	[illegible]	[illegible]	[illegible]	[illegible]	[illegible]	[illegible]	[illegible]	[illegible]	[illegible]	[illegible]	[illegible]	[illegible]	[illegible]	[illegible]	[illegible]	[illegible]	[illegible]	[illegible]	[illegible]	[illegible]	[illegible]	[illegible]	[illegible]	[illegible]	[illegible]	[illegible]	[illegible]	[illegible]	[illegible]	[illegible]	[illegible]	[illegible]	[illegible]	[illegible]	[illegible]	[illegible]	[illegible]	[illegible]	[illegible]	[illegible]	[illegible]	[illegible]	[illegible]	[illegible]	[illegible]	[illegible]	[illegible]

Table S12 Amino acid descriptors (2D) used in this study.

	S	S	S	S	S	S	T	T	T	T	T	T	T	T	T	T	T	T	T	T	T	T	T	T	T	T
	R	S	T	V	W	Y	A	C	D	E	F	G	H	I	K	L	M	N	P	Q	R	S	T	V	W	Y
No.	SR	SS	ST	SV	SW	SY	TA	TC	TD	TE	TF	TG	TH	TI	TK	TL	TM	TN	TP	TQ	TR	TS	TT	TV	TW	TY
1	-3	-1	-3	-2	7	-3	-2	1	-3	0	-4	0	-5	-3	5	-1	-4	-4	-5	-4	-8	-3	-4	-4	-4	-4
2	-0.4	-1.2	-1.4	-2.4	6.1	-4.1	-1.6	0.8	-3	-0.1	-3.4	0.8	-1.3	-1.6	5.8	0.5	-2.1	-4.1	-4.6	-0.1	-5.6	-1.4	-3.7	-5	-3.7	-3.4
3	-0.1	-0.5	-1	-2	6.1	-3.6	-0.9	0.8	-2.7	0.4	-3.8	0.7	-1.7	-1.4	6.2	0.5	-2	-4	-4.9	-0.7	-6.3	-1	-3.8	-4.4	-3.2	-3.8
4	0.3	0.1	-1.1	-1.7	6.1	-2.9	-0.2	0.6	-2.1	0.4	-4.3	0.2	-2	-1.1	6.6	-0.5	-1.6	-3	-4.6	-1	-4.7	-1.1	-3.5	-3.1	-2.3	-4.3
5	-2	-2.8	-2.2	0.1	4.7	-2.3	-2.1	1.2	1.3	-2.6	-2.5	1.1	1	-2.1	4.2	1.4	-2.2	-0.9	-2.2	0.8	-3.2	-2.2	-2.3	-2.5	-1.8	-2.5
6	0.34	-0.17	-0.23	-0.3	1	-0.51	-0.27	0.11	-0.44	0.22	-0.22	0.11	0.25	-0.13	1	-0.23	0.18	-0.32	-0.14	-0.11	-0.22	-0.23	-0.01	-0.45	-0.14	-0.22
7	-1	0	-2	-2	6	-2	0	1	-2	0	-3	1	-3	-1	5	0	-2	-4	-4	-3	-7	-2	-3	-3	-2	-3
8	2	3	2	2	6	1	3	5	4	3	2	4	3	2	6	4	1	1	2	3	1	2	1	1	1	2
9	2	2	2	1	0	3	2	1	1	2	2	1	1	2	0	1	2	2	1	1	2	2	2	2	2	2
10	9	7	5	7	10	3	5	9	4	10	7	6	8	10	10	6	9	3	7	5	1	5	8	3	8	7
11	0.4	0.2	-1.1	-1.6	6	-2.7	-0.1	0.5	-1.9	0.4	-4.5	0.1	-2	-1	6.6	-0.8	-1.4	-2.7	-4.4	-1	-4.3	-1.1	-3.5	-2.8	-2.2	-4.5
12	54	80	127	113	0	134	65	60	121	80	135	94	159	87	0	98	98	122	138	125	152	127	127	138	94	135
13	0	0	-2	-2	10	-3	-1	0	-3	0	-4	-1	-3	-2	7	-2	-2	-2	-3	-2	-3	-2	-2	-2	-3	-4
14	0	0	-2	-3	11	-5	-1	0	-4	-1	-6	-2	-4	-3	8	-3	-3	-3	-5	-3	-4	-2	-4	-4	-5	-6
15	-1	-1	-3	-4	12	-6	-2	0	-4	-1	-7	-3	-6	-4	9	-4	-4	-5	-7	-4	-6	-3	-5	-6	-6	-7
16	-0.2	-2.2	-3.5	-4.4	12.7	-4.8	-0.5	-0.5	-4.2	-1.4	-5.5	-2.1	-8.2	-2.8	8	-2.5	-3.2	-3.7	-7.2	-2.8	-7.6	-3.5	-5.2	-5.6	-5.1	-5.5
17	0	-1	-1	-2	6	-3	-1	1	-2	0	-3	1	-1	-1	5	0	-2	-4	-4	0	-5	-1	-3	-4	-3	-3
18	2	0	-1	-2	11	-4	-1	1	-2	-2	-2	3	-1	-1	6	3	-3	-5	-4	0	-3	-1	-3	-5	-4	-2
19	6.6	0	0	9	10	0	0	0	0	0	0	0	0	0	10	0	0	0	0	0	0	0	0	5	0	0
20	0	0	0	-1	2	-1	0	0	-1	0	-1	0	0	0	2	0	0	-1	-1	0	-1	0	-1	-1	-1	-1
21	2	2	-15	-2	54	-3	1	-5	-2	-4	-16	-7	-15	-8	39	-6	-13	-10	-17	-7	-14	-15	-11	-4	-10	-16
22	8	9	2	-12	65	-5	10	5	-4	5	-20	6	-36	3	52	8	-14	-3	-32	-2	-18	2	-32	-14	-10	-20
23	-1	1	-4	-13	43	-10	0	1	-6	4	-5	2	4	2	29	1	0	-21	-15	2	-16	-4	-5	-16	-4	-5
24	12	5	2	-7	95	-2	4	5	-3	7	-6	0	3	5	53	8	-32	-8	-21	0	-40	2	-6	-10	-17	-6
25	-1	-3	-1	-13	84	-7	0	9	-8	-4	-8	3	-33	3	59	4	-4	-13	-21	6	-35	-1	-2	-13	-13	-8
26	-1	13	-2	-9	18	-18	9	14	-24	6	-9	-4	-10	4	48	13	-3	14	-9	-8	-19	-2	4	-21	-15	-9
27	3	3	-12	-6	55	-5	2	-4	-3	-2	-18	-5	-21	-6	41	-4	-12	-9	-22	-6	-17	-12	-15	-8	-12	-18
28	4	4	-1	-14	52	-10	2	2	-8	6	-8	4	4	3	37	3	-1	-16	-19	3	-38	-1	-6	-18	-9	-8
29	2	5	0	-12	55	-13	4	12	-12	0	-11	1	-18	4	54	6	-5	-3	-15	2	-29	0	2	-14	-15	-11
30	3	4	3	3	8	1	4	3	2	3	1	3	1	2	8	3	2	2	1	3	1	3	1	1	2	1
31	0	0	0	0	6	0	0	3	0	0	0	0	0	0	6	0	0	0	0	0	0	0	0	0	0	0
32	-0.02	-0.19	-0.16	-0.74	0.54	-0.86	-0.09	0.19	-0.42	-0.11	-0.58	0.15	-0.18	-0.14	0.43	0.13	-0.19	-0.11	-0.56	0.2	-0.66	-0.16	-0.61	-0.8	-0.69	-0.58
33	1.45	2.06	3.54	2.7	0	3.39	1.83	0.91	2.97	1.96	3.6	2.37	2.22	2.48	0	2.78	2.78	3.22	3.67	3.58	4.08	3.54	3.34	3.53	2.45	3.6
34	9	9	7	9	16	4	8	8	4	10	6	9	8	8	16	6	7	6	6	7	7	7	4	7	8	6
35	0.09	-0.41	-0.34	0.29	1	-0.14	-0.5	-0.4	-0.09	0.37	-0.23	0.08	0.12	-0.28	1	-0.45	0.06	-0.57	-0.54	-0.18	-0.12	-0.34	-0.29	0.57	0.01	-0.23
36	0.11	-0.43	-0.38	0.35	1	-0.13	-0.54	-0.41	-0.09	0.4	-0.24	0.08	0.13	-0.32	1	-0.46	0.09	-0.62	-0.56	-0.21	-0.12	-0.38	-0.3	0.62	0	-0.24
37	0	-1	-3	-3	8	-3	0	0	-2	-1	-5	-1	-6	-2	5	-2	-3	-2	-5	-2	-6	-3	-4	-4	-5	-5
38	0.375	-0.081	0.006	0.251	1	0.212	0.22	0.407	0.444	0.293	0.429	0.25	0.246	0.494	1	0.164	0.497	0.273	0.327	0.394	0.149	0.006	0.326	0.291	0.406	0.429
39	0.037	0.204	0	0.439	1	-0.135	0.287	0	-0.186	0	0	0	0	0	0	0	0	0.036	0	0	-0.216	0	0	-0.223	0.378	0
40	0	2	-1	3	8	0	3	2	0	2	0	0	1	2	8	-1	0	1	1	2	0	-1	2	0	3	0
41	1.9	1.8	-0.1	1.1	2.2	1.5	1.6	0.6	1.6	0.2	0	-0.4	-1.7	0.2	2.2	0.3	-1.2	0.2	-0.2	0.1	-1	-0.1	-0.4	0.9	-0.8	0
42	11	-10	-11	-26	100	-36	-12	-3	-20	10	-21	8	-1	-10	100	-5	-8	-28	-16	-6	-10	-11	-10	-40	3	-21
43	13	6	-1	-9	7	-11	3	5	-12	8	-10	7	1	0	24	1	6	-1	-9	0	-6	-1	-3	-8	-4	-10
44	10	3	0	-9	5	-10	1	1	-11	7	-8	8	0	0	18	0	5	-1	-8	0	-5	0	-5	-7	-3	-8
45	74	45	39	57	100	29	41	96	33	64	87	36	94	68	100	43	75	44	89	17	37	39	85	31	62	87
46	-1	-2	-1	0	4	-4	-1	-1	-4	0	-2	-2	0	0	4	-2	-1	-4	-1	-1	-2	-1	0	-3	-1	-2
47	-1	-2	1	2	-4	-1	0	-1	-2	0	2	-1	3	2	-2	0	1	-2	3	1	1	1	3	0	2	2
48	1.15	1.02	1.05	1.01	0.99	0.96	0.94	1.11	1.04	0.96	0.79	1.02	1.36	1.11	0.86	1.06	1.1	0.76	0.88	0.99	0.86	1.05	0.81	1.01	0.86	0.79
49	0.82	0.92	0.76	1.07	0.99	1.15	1.11	0.83	1.08	0.71	1.25	0.61	0.8	0.78	0.97	0.87	0.71	1.31	1.09	0.84	1.33	0.76	1.18	1.12	1.24	1.25
50	1.02	1.13	1.43	0.8	1.05	0.74	0.89	1.11	0.71	1.8	0.98	1.88	0.51	1.22	1.5	1.15	1.4	0.91	1.1	1.41	0.61	1.43	1.07	0.69	0.81	0.98 -
51	18.7	17.7	15.6	13.3	30.8	14.9	18.8	19.9	15.1	18.1	14.7	18.7	15	16.3	26.8	19.3	12.2	12.7	11.5	16.1	15.3	15.6	15	11.4	12.4	14.7
52	5.6	5.4	4.1	3.6	11.2	2.5	5.1	5.7	3.3	5.6	0.7	5.3	3.2	4.2	11.8	4.4	3.8	2.5	0.8	4.2	0.9	4.1	1.7	2.4	3	0.7
53	3	0	0	-10	23	-8	3	1	-6	1	-3	2	-15	-3	16	0	-2	-5	-6	-2	-3	0	-7	-8	-4	-3
54	0.99	0.31	-0.71	-1.49	5.89	-1.81	-0.21	0.27	-1.84	0.79	-2.93	-1.2	-2.11	-0.12	4.36	-0.41	-0.4	-1.64	-1.98	-0.96	-2.7	-0.71	-1.86	-2.11	-1.76	-2.93
55	2.83	0.42	-1.82	-4.56	15.72	-5.89	-0.61	0.63	-4.23	1.16	-8.58	-3.91	-5.7	-0.24	11.64	-1.8	-1.24	-4.5	-6.55	-3.39	-6.28	-1.82	-5.29	-5.62	-4.44	-8.58
56	1.9	1.1	0.4	-1.2	6.8	-3.1	1.6	1.4	-1.3	0.7	-3.7	0.9	-1.3	-0.3	7.5	0.5	-1.7	-2.2	-3.8	-0.2	-4.7	0.4	-3.8	-1.5	-1.8	-3.7
57	8	3	0	-5	5	-9	3	0	-9	5	-7	4	0	0	12	0	3	-2	-6	0	-3	0	-3	-6	-3	-7
58	2	2	2	-2	-3	-4	2	1	-3	3	0	3	-1	-1	0	3	-5	-2	-2	2	-1	2	1	-1	1	0
59	2	1	0	-5	11	-3	0	1	-2	1	-4	1	-1	0	6	-1	-1	-1	-4	0	-2	0	-3	-2	-4	-4
60	2	2	1	-2	1	-3	1	0	-2	2	-1	2	0	0	1	1	-3	-1	-2	1	-3	1	0	0	-1	-1
61	-7.5	-6.6	-6.8	-7.4	-6.1	-8.3	-6.8	-5.8	-7.3	-6.8	-8	-6.3	-7.5	-7.6	-4.2	-7.4	-7.7	-7.5	-8	-7.5	-7.9	-6.8	-8.2	-7.4	-8.2	-8
62	3	-1	-18	-23	86	-33	-6	4	-28	7	-41	-10	-29	-20	67	-23	-18	-21	-42	-18	-29	-18	-30	-31	-28	-41
63	-6	-3	-5	-3	11	-5	-5	1	-5	-1	-2	-2	-2	-2	9	-3	-4	-2	-2	-5	-7	-5	-1	-5	-5	-2
64	-9	-5	-4.5	-3.5	10	-7	-6.5	0.5	-6.5	-3	-2.5	-4.5	-1.5	-3.5	7	-6.5	-5.5	-5.5	-2	-5.5	-6	-4.5	-1.5	-7	-4	-2.5
65	-1	0	-2	-2	9	-5	-1	0	-3	0	-7	-1	-2	-3	8	-2	-3	-3	-6	-3	-7	-2	-5	-4	-4	-7
66	0	0	-1	-2	6	-3	0	1	-2	0	-4	0	-1	-1	7	-1	-1	-2	-4	-1	-5	-1	-3	-3	-2	-4
67	0	-1	-2	-3	9	-4	-1	-1	-4	-1	-6	-1	-3	-2	7	-2	-2	-3	-5	-3	-3	-2	-4	-4	-3	-6
68	0.026	0.474	0.007	0.039	0.001	0.053	0.027	0.014	0.039	0.029	0.006	0.134	0.007	0.005	0.041	0.019	0.097	0.001	0.07	0.02	0.012	0.007	0.007	0.012	0.008	0.006
69	-0.16	3.35	-9.67	-5.3	-9.21	-0.26	-1.07	-2.33	-6.99	-7.13	-8	0.93	-5.59	-6.89	-1.52	-2.89	3.2	-5.59	3.82	-8.02	-10.01	-9.67	-9.31	-1.93	-7.97	-8
70	-0.4	7	-3	0.7	-0.3	0.5	-0.1	1.8	1.1	1.8	0.2	3.6	1.7	0.4	0.2	-2.1	1.9	-0.6	2.2	0.5	-1.3	-3	-2.4	-0.4	-1.9	0.2
71	-0.49	6.97	-5.14	-2.11	-2.83	1.17	1.55	-0.2	-1.2	-2.76	-3.28	3.65	-2.34	-2.93	-0.86	-1.78	3.48	-2.46	3.25	-3.98	-3.91	-5.14	-3.45	-0.33	-4.64	-3.28
72	2.1	0	-0.1	1	0.2	-2.4	0.4	0.1	0.8	0.5	-0.2	3.4	0.1	-0.5	2.2	0.5	-1.3	-0.3	1.3	0.2	0.4	-0.1	0.1	-0.9	-0.4	-0.2
73	-1	6	-5	-2	-2	0	0	0	-1	-3	-2	3	-2	-3	-1	-3	2	-3	2	-4	-4	-5	-3	-2	-4	-2
74	-3	-5	-8	-7	-6	-6	-7	6	-15	-9	-2	-9	-6	-7	-5	-8	-5	-5	-5	-8	-10	-8	-1	-6	-12	-2
75	7.4	-2.6	25.5	-5.7	39.5	39.5	19.1	-7.1	166.7	-7.3	-8.2	8.4	9.8	11.1	-6	6.8	-15	-10.4	-7.3	-1.5	13.4	25.5	-12.2	-8.7	-12.4	-8.2
76	1	2.4	1.1	1.1	56.9	0.1	0.2	2	0.3	2.3	0.1	3.3	0.1	0.4	6	0.1	0.1	1.1	0.2	1.2	0.1	1.1	1.5	0.1	0.1	0.1
77	1	2.4	1.1	1.1	56.9	0.1	0.2	2	0.3	2.3	0.1	3.3	0.1	0.4	6	0.1	0.1	1.1	0.2	1.2	0.1	1.1	1.5	0.1	0.1	0.1
78	1.2	46.8	6.3	0.1	0.1	0.9	7.5	0.1	0.7	0.2	0.1	1.8	0.8	2.4	1.2	4.8	1	2.5	0.2	0.4	0.3	6.3	48.8	0.6	0.6	1.5
79	1.2	46.8	6.3	0.1	0.1	0.9	7.5	0.1	0.7	0.2	0.1	1.8	0.8	2.4	1.2	4.8	1	2.5	0.2	0.4	0.3	6.3	48.8	0.6	0.6	1.5
80	1.2	46.8	6.3	0.1	0.1	0.9	7.5	0.1	0.7	0.2	0.1	1.8	0.8	2.4	1.2	4.8	1	2.5	0.2	0.4	0.3	6.3	48.8	0.6	0.6	1.5
81	1.2	46.8	6.3	0.1	0.1	0.9	7.5	0.1	0.7	0.2	0.1	1.8	0.8	2.4	1.2	4.8	1	2.5	0.2	0.4	0.3	6.3	48.8	0.6	0.6	1.5
82	1.2	46.8	6.3	0.1	0.1	0.9	7.5	0.1	0.7	0.2	0.1	1.8	0.8	2.4	1.2	4.8	1	2.5	0.2	0.4	0.3	6.3	48.8	0.6	0.6	1.5
83	1.2	46.8	6.3	0.1	0.1	0.9	7.5	0.1	0.7	0.2	0.1	1.8	0.8	2.4	1.2	4.8	1	2.5	0.2	0.4	0.3	6.3	48.8	0.6	0.6	1.5
84	1.2	46.8	6.3	0.1	0.1	0.9	7.5	0.1	0.7	0.2	0.1	1.8	0.8	2.4	1.2	4.8	1	2.5	0.2	0.4	0.3	6.3	48.8	0.6	0.6	1.5
85	1.2	46.8	6.3	0.1	0.1	0.9	7.5	0.1	0.7	0.2	0.1	1.8	0.8	2.4	1.2	4.8	1	2.5	0.2	0.4	0.3	6.3	48.8	0.6	0.6	1.5
86	1.2	46.8	6.3	0.1	0.1	0.9	7.5	0.1	0.7	0.2	0.1	1.8	0.8	2.4	1.2	4.8	1	2.5	0.2	0.4	0.3	6.3	48.8	0.6	0.6	1.5
87	1.2	46.8	6.3	0.1	0.1	0.9	7.5	0.1	0.7	0.2	0.1	1.8	0.8	2.4	1.2	4.8	1	2.5	0.2	0.4	0.3	6.3	48.8	0.6	0.6	1.5
88	1.2	46.8	6.3	0.1	0.1	0.9	7.5	0.1	0.7	0.2	0.1	1.8	0.8	2.4	1.2	4.8	1	2.5	0.2	0.4	0.3	6.3	48.8	0.6	0.6	1.5
89	1.2	46.8	6.3	0.1	0.1	0.9	7.5	0.1	0.7	0.2	0.1	1.8	0.8	2.4	1.2	4.8	1	2.5	0.2	0.4	0.3	6.3	48.8	0.6	0.6	1.5
90	1.2	46.8	6.3	0.1	0.1	0.9	7.5	0.1	0.7	0.2	0.1	1.8	0.8	2.4	1.2	4.8	1	2.5	0.2	0.4	0.3	6.3	48.8	0.6	0.6	1.5
91	0.032	0.015	0.016	0.001	0.003	0.004	0.266	0.037	0.016	0.034	0	0.055	0	0.018	0.021	0.069	0.022	0.044	0.178	0.025	0.111	0.016	0.018	0.001	0.02	0.003
92	0.032	0.015	0.016	0.001	0.003	0.004	0.266	0.037	0.016	0.034	0	0.055	0	0.018	0.021	0.069	0.022	0.044	0.178	0.025	0.111	0.016	0.018	0.001	0.02	0.003
93	0.032	0.015	0.016	0.001	0.003	0.004	0.266	0.037	0.016	0.034	0	0.055	0	0.018	0.021	0.069	0.022	0.044	0.178	0.025	0.111	0.016	0.018	0.001	0.02	0.003
94	0.032	0.015	0.016	0.001	0.003	0.004	0.266	0.037	0.016	0.034	0	0.055	0	0.018	0.021	0.069	0.022	0.044	0.178	0.025	0.111	0.016	0.018	0.001	0.02	0.003
101	-3.8	-2.5	-3.3	-4.2	-5	-4.7	-3.3	-4.8	-3.1	-3.3	-5.1	-4.1	-4.5	-5.9	-3.3	-4.8	-4.6	-3.5	-3.6	-3.7	-4	-3.3	-3.1	-4.4	-5.1	-4.9
102	44	34	69	106	31	71	70	22	38	28	39	116	36	67	50	110	16	46	32	28	36	69	29	79	20	53
103	1.048	0.128	0.072	-0.194	-0.629	-0.476	-0.177	-0.089	1.89	1.792	-0.869	0	-0.096	-0.808	1.786	-0.773	-0.433	0.053	-1.379	0.067	1.054	0.072	-0.085	-0.526	-0.961	-0.807
104	-0.263	-0.15	-0.43	0.316	0.298	0.442	0.002	0.021	-0.433	-0.006	0.058	-0.106	0.061	0.167	-0.17	0.075	0.087	-0.018	0.108	0.06	0.25	-0.43	-0.315	0.251	0.04	0.492
105	0.61	-0.13	0.05	-0.31	-0.2	0.25	-0.22	-0.38	-0.03	0.15	-0.19	0.13	-0.27	-0.29	0.09	-0.39	0.06	-0.27	0.36	-0.17	-0.17	0.05	0.26	0.06	0.07	-0.23
106	0.17	-0.2	-0.08	0.18	0.34	0.09	-0.09	0.19	-0.29	0	0.31	-0.26	-0.19	0.14	-0.09	0.2	0.19	-0.11	-0.07	-0.14	-0.35	-0.08	0.03	0.25	0.22	0.13
107	0.0856	-0.0667	0.0026	0.0312	0.07	0.068	-0.0383	0.1422	-0.0422	0.0788	0.0135	-0.0574	-0.005	0.0158	0.0082	0.0169	0.0894	0.003	0.062	0.0725	-0.093	0.0026	0.0664	-0.0002	0.0656	0.0568
108	0.0442	0.0941	0.0228	0.1766	-0.0802	0.0214	0.031	-0.0013	0.1043	0.0675	-0.112	0.1763	0.0681	-0.07	0.0467	-0.0316	0.0018	0.0155	0.1908	-0.0243	-0.021	0.0228	0.015	0.0119	0.0052	-0.1445
109	-1.22	-1.48	-1.59	-2.79	-2.95	-2.3	-2.15	-2.88	-1.66	-1.45	-3.76	-2.03	-2.31	-3.74	-1.02	-3.43	-3.73	-1.51	-1.66	-1.59	-1.97	-1.59	-1.72	-2.95	-3.31	-2.48
110	0.17	-0.2	-0.08	0.17	0.34	0.08	-0.09	0.18	-0.29	-0.01	0.31	-0.27	-0.2	0.13	-0.09	0.2	0.19	-0.11	-0.07	-0.14	-0.35	-0.08	0.02	0.24	0.21	0.13
111	-1.62	-1.67	-1.96	-3.05	-2.99	-2.78	-2.32	-3.11	-1.8	-1.74	-4.28	-2.08	-2.42	-4.03	-1.31	-4.34	-3.51	-1.88	-1.9	-1.9	-1.9	-1.96	-2.12	-3.46	-3.22	-3.01
112	-0.08	-0.26	-0.24	0.27	0.25	0.06	-0.01	0.2	-0.22	-0.21	0.22	-0.15	-0.12	0.13	-0.05	0.21	0.15	-0.22	-0.07	-0.16	-0.05	-0.24	-0.09	0.17	0.33	0.14
113	4790	5458	9465	9059	1889	5537	11323	2475	7362	6172	5281	11312	3071	7958	4940	11387	2595	5790	4806	4412	4720	9465	4387	9906	1708	5102
114	0.16	0.05	0.04	0.04	-0.01	-0.08	0.04	-0.15	0.11	0.16	-0.15	-0.04	-0.03	-0.15	0.33	-0.15	-0.11	0.04	0.13	0.12	0.11	0.04	0.03	-0.07	-0.02	-0.09
115	-0.03	-0.17	-0.12	0.16	0.15	0.04	0.01	0.12	-0.13	-0.1	0.13	-0.07	-0.06	0.08	-0.02	0.12	0.09	-0.11	-0.03	-0.08	-0.02	-0.12	-0.07	0.11	0.2	0.09
116	-2.46	-2.47	-2.74	-3.35	-3.3	-2.97	-2.97	-3.73	-2.43	-2.33	-3.7	-2.78	-2.97	-3.87	-2.13	-3.61	-3.62	-2.43	-2.68	-2.46	-2.55	-2.74	-2.81	-3.58	-3.44	-3.16
117	-2.78	-2.14	-2.22	-2.95	-2.86	-3.43	-2.38	-2.92	-2.41	-2.34	-3.85	-1.81	-2.73	-3.61	-1.64	-4.28	-3.33	-2.05	-1.24	-3.44	-2.43	-2.22	-2.12	-2.87	-3.48	-3.33
118	0.08	-0.14	-0.27	0.25	0.86	-0.03	0.14	0.46	-0.26	-0.07	-0.02	-0.11	-0.05	0.14	-0.29	0.04	0.19	-0.3	0.11	-0.32	0.38	-0.27	-0.22	0.27	0.18	0
119	0.19	-0.63	-0.05	-0.19	0.05	0.12	-0.43	-0.17	-0.24	-0.23	0.03	-0.21	0.06	-0.05	-0.22	-0.05	0.1	-0.23	-0.13	-0.17	0	-0.05	-0.58	-0.13	0.03	0.15
120	0.12	0.13	0	0.25	0.07	0.07	0	0	0	0	0	0	0	0	0	0	0	0	0	0	0	0	0	0	0	0
121	0.84	0.03	0.32	0.52	-0.08	-0.83	-0.45	-1.38	0.49	0.81	0.32	0.1	0.8	-0.29	0.38	0.3	-0.4	0.13	0.47	-0.2	-0.19	0.32	-0.38	0.24	-0.61	0.67
122	0.91	1.31	0.03	0.14	-0.77	-0.86	0.33	0	-0.35	-0.11	-0.29	0.73	-0.27	-0.18	-0.16	-0.18	-0.62	1.23	0.34	-0.28	-0.44	0.03	-0.41	0.29	0.32	-0.19

	V	V	V	V	V	V	V	V	V	V	V	V	V	V	V	V	V	V	V	V	W	W	W	W	W	W	W	W	W
	A	C	D	E	F	G	H	I	K	L	M	N	P	Q	R	S	T	V	W	Y	A	C	D	E	F	G	H	I	K
No.	VA	VC	VD	VE	VF	VG	VH	VI	VK	VL	VM	VN	VP	VQ	VR	VS	VT	VV	VW	VY	WA	WC	WD	WE	WF	WG	WH	WI	WK
1	1	-3	0	0	-5	-7	-2	3	-4	-3	-4	1	-7	-2	-2	-2	-4	1	-5	-3	-5	-8	-1	-2	-1	-3	1	2	-4
2	2.4	-2	0	0.7	-5.3	-5	-3.3	3.9	-3.2	-1.3	-3.4	4	-3.8	-1.2	-3.8	-2.4	-5	2.4	-4.6	-2.2	-5.7	-1.7	-0.5	-2.3	-0.2	-2.1	1.8	1.4	-1.2
3	2.7	-2.3	0.5	0.3	-4.9	-4.4	-2.2	3.6	-2.9	-1.7	-3.5	3.1	-2.6	-1.4	-3	-2	-4.4	2.7	-4.9	-2.1	-5.8	-4.5	-0.7	-2.5	-0.2	-1.6	1.5	1.4	-1.5
4	2.8	-2.6	0.9	-0.3	-4.2	-2.3	-1	3.2	-2.4	-1.4	-3.1	2.6	-1.6	-1.8	-2.3	-1.7	-3.1	2.8	-4.6	-1.9	-5.4	-4.1	-1	-3.1	0.2	-1	1	1.2	-1.3
5	1.2	-1.6	1.3	0.8	-2.4	-2.2	-1.6	1	-0.4	-2.3	-2.2	3.3	-1.3	-0.5	0.7	0.1	-2.5	1.2	-2.2	-0.1	-1.9	1.4	-2.1	1.1	0.3	-2.1	3.6	1.8	-1.6
6	0.6	-0.19	0.6	0	-0.55	0.36	0.43	0.7	-0.2	0.2	-0.39	0.36	0.23	-0.27	-0.4	-0.3	-0.45	0.6	-0.14	-0.12	-0.09	-0.08	-0.02	-0.12	-0.19	-0.11	0.13	0.32	0.18
7	2	-2	1	0	-4	-5	-1	4	-3	-2	-3	2	-6	-1	-2	-2	-3	2	-4	-2	-5	-7	0	-1	1	-1	2	2	-3
8	5	2	4	3	1	2	3	5	2	2	1	4	2	2	2	2	1	5	2	3	1	3	2	4	2	2	4	4	2
9	1	2	1	1	2	3	2	1	1	2	2	1	2	1	2	1	2	1	1	1	2	1	2	1	2	2	1	1	2
10	10	9	9	8	3	8	9	10	3	8	7	9	9	7	3	7	3	10	7	8	6	5	9	8	5	10	5	9	9
11	2.8	-2.6	1	-0.6	-4	-1.8	-0.7	3.1	-2.2	-1.2	-3	2.5	-1.5	-1.8	-2.1	-1.6	-2.8	2.8	-4.4	-1.9	-5.2	-4	-1.1	-3.3	0.4	-0.8	0.6	1.2	-1.3
12	5	95	21	89	172	61	33	29	102	96	153	10	198	142	102	113	138	5	138	99	153	184	59	109	40	86	29	68	174
13	2	-2	0	-1	-3	-2	0	3	-2	-1	-3	2	-2	-2	-3	-2	-2	2	-3	-2	-3	-2	-2	-3	0	-2	0	1	-3
14	2	-4	0	-1	-5	-4	-2	4	-3	-2	-5	2	-2	-4	-4	-3	-4	2	-5	-4	-5	-4	-2	-5	0	-2	0	1	-4
15	2	-5	-1	-2	-7	-5	-3	4	-4	-3	-6	2	-3	-4	-5	-4	-6	2	-7	-5	-6	-6	-3	-6	0	-3	0	1	-7
16	2.6	-5.7	0.5	-3.2	-8	-3.3	-2.5	3.9	-3.7	-3.3	-4.8	2.6	-8.7	-4.7	-4.7	-4.4	-5.6	2.6	-7.2	-4.2	-8.6	-6.3	-3.8	-5.6	-2.3	-3.1	0.1	1.7	-8.2
17	2	-2	0	1	-4	-4	-2	4	-3	-1	-3	3	-3	-1	-3	-2	-4	2	-4	-2	-5	-2	-1	-2	0	-2	2	1	0
18	1	-3	-1	0	-5	-3	-4	2	-3	-2	-4	1	-1	-1	-4	-2	-5	1	-4	-4	-4	-2	0	-1	2	-3	5	3	-1
19	0	0	0	0	0	0	0	9	6.6	5	0	0	0	0	0	9	5	0	0	0	0	0	0	0	0	0	5	5	9
20	0	-1	1	0	-1	0	0	1	-1	0	-1	0	0	-1	-1	-1	-1	0	-1	-1	-1	-1	0	-1	0	0	0	0	0
21	19	-11	7	0	-10	1	-3	24	-4	1	-4	20	-1	-5	-10	-2	-4	19	-17	-6	-24	-12	-12	-12	-3	-4	-1	2	-23
22	9	-28	-4	-1	-16	-19	0	17	-10	-10	-10	18	-35	-11	-5	-12	-14	9	-32	-18	-52	-23	0	-20	-3	-2	5	6	-20
23	13	-8	10	3	-18	-14	-14	18	-7	-18	-12	17	-3	-4	-11	-13	-16	13	-15	-12	-21	-21	3	-5	-1	-5	0	3	0
24	0	1	-4	2	-20	-27	-4	5	-3	-14	-6	3	-11	1	-1	-7	-10	0	-21	-10	-25	-57	4	-10	-3	-30	4	-3	-30
25	16	8	7	-3	-21	-25	-10	11	-8	-7	-11	10	-34	-10	-9	-13	-13	16	-21	-9	-10	-33	-1	-14	-5	-11	-7	14	-33
26	11	-9	-10	-7	-27	-8	-16	11	-12	-6	-15	3	-29	-12	-13	-9	-21	11	-9	-15	-17	-32	8	-9	16	2	11	13	-2
27	19	-15	5	-1	-14	-5	-2	24	-7	-3	-7	22	-10	-8	-11	-6	-8	19	-22	-10	-30	-15	-10	-14	-2	-3	0	2	-23
28	7	-7	3	1	-21	-25	-11	13	-10	-19	-14	12	-8	-5	-12	-14	-18	7	-19	-14	-26	-46	4	-9	1	-6	2	5	-5
29	13	-1	-3	-7	-22	-12	-15	11	-10	-7	-13	8	-34	-13	-14	-12	-14	13	-15	-13	-15	-33	3	-12	5	-4	2	13	-12
30	5	1	3	3	1	3	3	5	2	2	1	5	0	2	1	3	1	5	1	2	0	1	2	2	2	3	5	4	3
31	3	0	1	1	0	1	1	2	0	0	0	3	0	0	0	0	0	3	0	0	0	0	0	0	0	0	0	1	0
32	0.48	-0.78	0.45	-0.59	-0.75	-0.23	-0.35	0.41	-0.74	-0.45	-0.9	0.67	-0.02	-0.68	-1.01	-0.74	-0.8	0.48	-0.56	-0.5	-0.52	-0.15	-0.09	-0.15	0.13	-0.21	0.11	0.21	-0.29
33	0.14	2.62	0.61	2.14	4.1	1.72	0.86	0.85	2.62	2.76	3.49	0.29	1.65	2.95	2.84	2.7	3.53	0.14	3.67	2.59	4.14	5.13	1.7	2.76	0.96	2.17	0.82	1.29	2.56
34	10	3	12	10	6	11	10	12	6	11	7	9	11	8	4	9	7	10	6	10	7	8	10	6	11	11	10	10	10
35	0.25	-0.53	0.27	0.3	0.01	0.03	0.13	0.67	0	0.51	-0.23	0.15	-0.27	-0.34	-0.17	0.29	0.57	0.25	-0.54	-0.09	-0.05	-0.1	0.1	-0.24	-0.14	-0.18	0.15	0.04	-0.04
36	0.27	-0.55	0.32	0.32	0.01	0.04	0.15	0.72	0.03	0.54	-0.24	0.18	-0.29	-0.37	-0.18	0.35	0.62	0.27	-0.56	-0.13	-0.05	-0.11	0.11	-0.25	-0.14	-0.2	0.19	0.04	-0.01
37	2	-4	1	-2	-6	-2	-1	2	-3	-2	-3	1	-6	-3	-3	-3	-4	2	-5	-3	-6	-4	-3	-4	-2	-2	0	2	-6
38	0.57	0.065	0.532	0.385	0.261	0.357	0.333	0.641	0.446	0.555	0.191	0.44	0.501	0.166	0.088	0.251	0.291	0.57	0.327	0.457	0.455	0.136	0.335	0.639	0.121	0.356	0.093	0.299	0.527
39	0.96	0.572	0.893	0.382	-0.249	0.792	0.573	0.946	0.049	0.862	-0.172	0.842	0.787	0.083	0.099	0.439	-0.223	0.96	0	0.289	0	0	0	0	-0.102	0.523	0.392	0.305	0.425
40	6	3	5	1	0	4	4	6	0	5	0	5	5	0	1	3	0	6	1	2	0	-1	1	2	0	4	2	2	4
41	2.1	-0.6	1	1.6	-0.2	-0.7	0.4	2.2	1.2	1.3	0.8	0.9	-1.5	1.6	1	1.1	0.9	2.1	-0.2	-0.9	-0.4	-1.3	0.2	0.1	-0.6	-0.6	-0.4	-0.3	-1.8
42	46	-18	32	-5	-40	12	29	46	-24	-6	-35	18	15	-17	-17	-26	-40	46	-16	-9	-12	-13	-13	-14	-8	-10	0	8	3
43	8	-14	6	-4	-11	4	2	9	-8	-2	-9	2	1	-8	-13	-9	-8	8	-9	-5	-4	-1	1	-8	4	-2	4	5	-2
44	8	-9	7	-2	-10	4	3	11	-7	0	-9	3	2	-7	-12	-9	-7	8	-8	-4	-3	-1	1	-7	1	-2	2	2	-1
45	98	79	96	88	25	93	85	97	6	93	52	98	95	84	26	57	31	98	89	64	83	94	99	90	68	71	42	89	69
46	1	0	0	0	-3	0	0	2	-1	0	-1	2	0	-1	-2	0	-3	1	-1	-1	-2	-2	0	-2	-1	0	0	0	0
47	-1	3	0	1	1	3	1	-1	1	1	2	1	1	1	1	2	0	-1	3	2	1	0	2	1	-1	2	0	-1	3
48	0.99	0.67	0.74 -		1	0.53	0.68	0.83	1.02	1.19	0.98	1.01	1.41	0.89	0.94	1.01	1.01	0.99	0.88	1.08	0.68	0.58	0.86	0.95	1.23	1.17	1.06	1.13	1.5
49	1.21	1.3	1.54 -	1.27	0.96	1.71	1.51	1.42	1.05	0.97	0.97	1.16	0.84	1.24	1.11	1.07	1.12	1.21	1.09	1.07	1.3	1.28	1.03	1.09	0.78	0.79	0.92	0.78	0.69
50		1.18	0.42	0.79	1.09	0.56	0.65	0.45	0.82	0.57	1.13	0.59	0.28	0.74	0.91	0.8	0.69 -		1.1	0.62	1.16	1.46	1.3	0.9	0.91	1.04	1.02	1.18	0.39
51	21	9.35	18.4	17.5	12.5	10.4	17.6	22.2	12.2	13.6	11.7	20.9	15.5	14.6	14.2	13.3	11.4	21	11.5	9.07	9.84	7.2	18.2	14.5	15.7	11.5	13.7	18.9	13.5
52	8	2.6	6.2	4.6	1.2	3.4	4.5	8.3	3	4	2.2	7.7	3.7	3.4	3.1	3.6	2.4	8	0.8	3.3	0	1.2	4.1	1.9	5.6	4.4	5.8	6.4	3.9
53	7	-3	3	-3	-10	-6	-2	8	-4	-2	-7	10	-11	-6	-7	-10	-8	7	-6	-4	-8	-10	-4	-5	0	-2	2	5	-18
54	1.56	-2	0.76	-0.96	-2.65	0.25	0.08	1.94	-1.19	-0.39	-2.1	0.99	-0.31	-1.75	-1.81	-1.49	-2.11	1.56	-1.98	-0.93	-2.67	-1.21	-0.81	-1.96	-0.62	-1.42	0.54	0.76	-1.5
55	3.86	-4.04	2.3	-3.03	-7.41	-0.26	-0.08	5.23	-2.83	-1.09	-5.64	2.94	-0.82	-4.67	-4.82	-4.56	-5.62	3.86	-6.55	-2.49	-7.46	-4.77	-2.24	-5.32	-1.29	-3.01	0.7	1.77	-5.95
56	3.4	-2.4	2.2	0	-3.4	-1.8	0.2	4.5	-1.5	-0.2	-2.4	2.2	-1.6	-1.1	-1.5	-1.2	-1.5	3.4	-3.8	-2.4	-4.7	-4.2	-0.6	-1.4	0.3	-1.4	1.5	1.4	-1.7
57	7	-8	6	-3	-8	4	2	9	-5	0	-7	3	2	-5	-9	-5	-6	7	-6	-3	-3	-1	0	-5	2	-1	3	2	-2
58	3	2	5	0	-1	2	3	4	-2	3	0	1	4	-1	-4	-2	-1	3	-2	3	-1	0	0	-3	-11	-1	2	7	5
59	7	-2	4	0	-7	3	0	7	-2	0	-2	5	2	-4	-6	-5	-2	7	-4	0	-6	-2	0	-2	3	-2	2	4	1
60	5	0	4	0	-3	4	2	5	-2	2	0	4	2	-3	-5	-2	0	5	-2	2	-3	-1	0	-1	-1	0	3	3	2
61	-5.5	-7.2	-7.2	-7.1	-7.1	-7.7	-7.4	-5.7	-7.9	-6.7	-7.7	-7.4	-7.7	-7.2	-8.1	-7.4	-7.4	-5.5	-8	-8.1	-8.1	-7.2	-6.7	-7.3	-7.8	-7.7	-8.1	-6.8	-9.3
62	28	-30	8	-8	-43	-28	-8	35	-20	-7	-32	12	-6	-20	-31	-23	-31	28	-42	-27	-32	-19	-17	-30	2	-19	3	12	-29
63	2	-4	0	-1	-5	-4	-3	3	-6	-1	-3	3	-2	-2	-7	-3	-5	2	-2	-4	-2	-5	-1	-2	-1	-3	-4	4	-7
64	3.5	-4	1.5	0.5	-6	-1.5	-2.5	4.5	-4.5	0	-3.5	4	1	-1.5	-5.5	-3.5	-7	3.5	-2	-4	-1	-4	-1.5	-2.5	-3	-3	-4.5	2.5	-5
65	3	-4	0	-1	-6	-2	-2	4	-3	-2	-4	2	-4	-3	-4	-2	-4	3	-6	-3	-6	-5	-2	-5	-1	-2	1	1	-2
66	3	-3	1	0	-4	-1	-1	3	-2	-1	-3	2	-2	-2	-2	-2	-3	3	-4	-2	-4	-4	-1	-3	0	-1	1	1	-1
67	2	-4	0	-2	-5	-1	-1	3	-3	-2	-4	1	-2	-4	-4	-3	-4	2	-5	-3	-5	-4	-2	-5	-1	-2	0	0	-3
68	0	0.013	0.055	0.016	0.017	0.041	0.024	0.175	0.054	0.088	0.002	0.012	0.025	0.009	0.047	0.039	0.012	0	0.07	0.522	0.015	0.019	0.036	0.001	0.396	0.013	0.004	0.015	0.029
69	-10.18	-2.89	-4.22	-2.6	-6.7	-1.65	-3.54	8.16	-1.08	0.24	-7.43	-8.6	-9.36	-10.89	1.79	-5.3	-1.93	-10.18	3.82	8.58	1.86	-4.62	-0.67	1.12	2.69	-2.29	-6.2	-6.44	-5.77
70	-2.3	-0.2	3.5	-1.3	-0.4	0.8	4.1	11.8	1	1.6	-2.4	-1.6	-0.6	-0.3	0.9	0.7	-0.4	-2.3	2.2	7.3	1	1.4	-1.3	1.6	6.4	0.7	-0.5	1.7	1.5
71	-4.24	-0.67	-1.3	-1.25	-3.14	0.68	-0.31	7.02	-0.27	1.09	-3.75	-1.2	-4.39	-4.95	0.9	-2.11	-0.33	-4.24	3.25	9.44	-1.78	-2.56	1.48	-0.79	6.86	-1.15	-2.34	-1.64	-1.9
72	-0.2	-0.3	0.1	0.5	0.4	0.3	0.8	-2.1	-1.5	-1.3	0	-0.3	-0.9	0.5	-0.3	1	-0.9	-0.2	1.3	0	0.1	0.2	1	-0.5	0	1.3	0.2	0.2	-0.5
73	-4	-1	-1	-2	-2	1	-1	4	0	1	-4	-3	-3	-4	0	-2	-2	-4	2	8	-3	-2	-1	-3	7	-1	-3	-1	-1
74	-2	-3	-5	-11	-2	-8	-5	-15	-7	7	1	-8	-6	-7	-4	-7	-6	-2	-5	-6	-1	-3	-13	-15	-3	9	-8	-5	-9
75	8.5	7.6	-2.1	-6.5	-7.3	-14.8	-9.3	4.6	-3	5.3	-4.4	21.5	20.3	16.2	-11.2	-5.7	-8.7	8.5	-7.3	-1.5	10.1	-5.4	23.8	-1.7	-11.3	-5.7	9.8	13.2	12.7
76	3.4	0.8	0.7	2.4	0.1	0.1	2.4	17.5	0.6	0.8	0.1	7.4	0.1	0.6	0.3	1.1	0.1	3.4	0.2	0.2	0.1	0.1	1.8	0.3	0.7	0.3	4.7	7.5	0.1
77	3.4	0.8	0.7	2.4	0.1	0.1	2.4	17.5	0.6	0.8	0.1	7.4	0.1	0.6	0.3	1.1	0.1	3.4	0.2	0.2	0.1	0.1	1.8	0.3	0.7	0.3	4.7	7.5	0.1
78	6.2	0.1	0.1	0.3	1.6	0.3	0.1	17.5	0.1	1.9	0.1	0.7	0.2	0.1	1.4	0.1	0.6	53.9	0.3	1.2	0.3	0.1	0.1	0.1	4.3	0.1	0.1	0.1	2.5
79	6.2	0.1	0.1	0.3	1.6	0.3	0.1	17.5	0.1	1.9	0.1	0.7	0.2	0.1	1.4	0.1	0.6	53.9	0.3	1.2	0.3	0.1	0.1	0.1	4.3	0.1	0.1	0.1	2.5
80	6.2	0.1	0.1	0.3	1.6	0.3	0.1	17.5	0.1	1.9	0.1	0.7	0.2	0.1	1.4	0.1	0.6	53.9	0.3	1.2	0.3	0.1	0.1	0.1	4.3	0.1	0.1	0.1	2.5
81	6.2	0.1	0.1	0.3	1.6	0.3	0.1	17.5	0.1	1.9	0.1	0.7	0.2	0.1	1.4	0.1	0.6	53.9	0.3	1.2	0.3	0.1	0.1	0.1	4.3	0.1	0.1	0.1	2.5
82	6.2	0.1	0.1	0.3	1.6	0.3	0.1	17.5	0.1	1.9	0.1	0.7	0.2	0.1	1.4	0.1	0.6	53.9	0.3	1.2	0.3	0.1	0.1	0.1	4.3	0.1	0.1	0.1	2.5
83	6.2	0.1	0.1	0.3	1.6	0.3	0.1	17.5	0.1	1.9	0.1	0.7	0.2	0.1	1.4	0.1	0.6	53.9	0.3	1.2	0.3	0.1	0.1	0.1	4.3	0.1	0.1	0.1	2.5
84	6.2	0.1	0.1	0.3	1.6	0.3	0.1	17.5	0.1	1.9	0.1	0.7	0.2	0.1	1.4	0.1	0.6	53.9	0.3	1.2	0.3	0.1	0.1	0.1	4.3	0.1	0.1	0.1	2.5
85	6.2	0.1	0.1	0.3	1.6	0.3	0.1	17.5	0.1	1.9	0.1	0.7	0.2	0.1	1.4	0.1	0.6	53.9	0.3	1.2	0.3	0.1	0.1	0.1	4.3	0.1	0.1	0.1	2.5
86	6.2	0.1	0.1	0.3	1.6	0.3	0.1	17.5	0.1	1.9	0.1	0.7	0.2	0.1	1.4	0.1	0.6	53.9	0.3	1.2	0.3	0.1	0.1	0.1	4.3	0.1	0.1	0.1	2.5
87	6.2	0.1	0.1	0.3	1.6	0.3	0.1	17.5	0.1	1.9	0.1	0.7	0.2	0.1	1.4	0.1	0.6	53.9	0.3	1.2	0.3	0.1	0.1	0.1	4.3	0.1	0.1	0.1	2.5
88	6.2	0.1	0.1	0.3	1.6	0.3	0.1	17.5	0.1	1.9	0.1	0.7	0.2	0.1	1.4	0.1	0.6	53.9	0.3	1.2	0.3	0.1	0.1	0.1	4.3	0.1	0.1	0.1	2.5
89	6.2	0.1	0.1	0.3	1.6	0.3	0.1	17.5	0.1	1.9	0.1	0.7	0.2	0.1	1.4	0.1	0.6	53.9	0.3	1.2	0.3	0.1	0.1	0.1	4.3	0.1	0.1	0.1	2.5
90	6.2	0.1	0.1	0.3	1.6	0.3	0.1	17.5	0.1	1.9	0.1	0.7	0.2	0.1	1.4	0.1	0.6	53.9	0.3	1.2	0.3	0.1	0.1	0.1	4.3	0.1	0.1	0.1	2.5
91	0.35	0.012	0.043	0.162	0.009	0	0.003	0.004	0.022	0.004	0.007	0.006	0.012	0.006	0.02	0.001	0.001	0.006	0.004	0.002	0.588	0.064	0	0.009	0	0.006	0.006	0.046	0.006
92	0.35	0.012	0.043	0.162	0.009	0	0.003	0.004	0.022	0.004	0.007	0.006	0.012	0.006	0.02	0.001	0.001	0.006	0.004	0.002	0.588	0.064	0	0.009	0	0.006	0.006	0.046	0.006
93	0.35	0.012	0.043	0.162	0.009	0	0.003	0.004	0.022	0.004	0.007	0.006	0.012	0.006	0.02	0.001	0.001	0.006	0.004	0.002	0.588	0.064	0	0.009	0	0.006	0.006	0.046	0.006
94	0.35	0.012	0.043	0.162	0.009	0	0.003	0.004	0.022	0.004	0.007	0.006	0.012	0.006	0.02	0.001	0.001	0.006	0.004	0.002	0.588	0.064	0	0.009	0	0.006	0.006	0.046	0.006
101	-4.3	-6	-4	-4.2	-6.5	-5.1	-5.3	-7.3	-4.2	-6.2	-6	-4.3	-4.7	-4.7	-4.9	-4.2	-4.4	-5.5	-6.5	-5.9	-5.2	-6.9	-5.1	-5.2	-7.4	-5.8	-6.5	-7.8	-5
102	101	53	53	39	114	183	42	216	67	306	52	50	58	46	44	106	79	152	62	85	38	17	24	15	41	52	23	40	20
103	-0.617	-0.529	2.1	1.944	-1.31	0	-0.536	-1.248	2	-1.214	-0.874	-0.295	-1.819	-0.265	0.944	-0.194	-0.526	-0.966	-1.401	-1.248	-1.052	-0.964	7.299	6.288	-1.744	0	-0.971	-1.683	6.663
104	-0.359	-0.026	0.442	0.255	-0.288	0.141	0.006	-0.253	0.253	-0.454	-0.265	0.158	0.358	-0.191	-0.011	0.316	0.251	-0.641	-0.034	0.075	0.117	0.08	1.287	-0.089	-0.185	0.036	-0.098	-0.505	-0.822
105	-0.6	-0.94	0.25	-0.02	-0.78	-0.2	-0.35	-0.98	-0.08	-1.03	-0.94	-0.24	-0.08	-0.09	-0.48	-0.31	0.06	-1.15	-0.6	-0.7	-0.08	-0.3	0.24	0.32	-0.89	-0.14	-0.41	-0.89	-0.3
106	-0.1	0.06	0.58	0.34	-0.22	0.16	0.19	-0.25	0.44	-0.29	-0.14	0.5	0.09	0.24	0.3	0.18	0.25	-0.29	-0.07	0.02	-0.09	0.08	0.24	0.29	-0.16	0.18	-0.12	0.02	0.22
107	-0.0852	-0.0607	0.0684	0.0595	-0.0336	0.0406	-0.001	-0.1095	0.0331	-0.1274	-0.0634	0.0147	0.027	0.0097	0.023	0.0312	-0.0002	-0.0718	0.026	-0.0303	0.0095	0.0489	-0.0061	0.1134	0.003	0.1083	-0.0196	0.0093	0.0117
108	-0.1431	-0.2349	0.2728	0.101	-0.4001	0.1859	-0.0039	-0.4223	0.0609	-0.4593	-0.2127	0.118	0.0868	0.1061	0.0475	0.1766	0.0119	-0.5193	-0.2898	-0.2792	-0.088	-0.1176	-0.0124	-0.0967	-0.3405	-0.1567	-0.02	-0.1961	-0.1152
109	-3.62	-4.46	-2.25	-2.56	-5.75	-3.06	-3.38	-5.58	-1.95	-5.38	-5.52	-2.36	-2.96	-2.67	-2.78	-2.79	-2.95	-4.94	-5.05	-4.05	-3.93	-4.76	-2.91	-2.94	-6.02	-3.37	-4.02	-5.64	-2.49
110	-0.11	0.05	0.57	0.33	-0.23	0.15	0.18	-0.26	0.43	-0.3	-0.15	0.49	0.08	0.23	0.29	0.17	0.24	-0.3	-0.08	0.01	-0.09	0.08	0.24	0.28	-0.17	0.17	-0.13	0.01	0.22
111	-4.04	-4.96	-2.48	-2.67	-6.29	-3.38	-3.58	-6.05	-2.49	-6.48	-5.32	-2.83	-3.32	-3.07	-3.07	-3.05	-3.46	-5.52	-5.18	-4.62	-3.82	-4.95	-2.84	-2.99	-6.16	-3.42	-3.98	-5.78	-2.69
112	-0.13	-0.05	0.7	0.46	-0.19	0.15	0.32	-0.29	0.37	-0.33	-0.06	0.43	0.11	0.27	0.38	0.27	0.17	-0.29	-0.03	0.13	0.01	-0.12	0.26	0.06	-0.14	0.03	-0.16	-0.1	0.09
113	21733	4768	4606	5241	12746	13584	3215	19830	5437	32623	5343	4792	6518	4541	4867	9059	9906	14091	3723	7777	3453	1168	1444	1521	2498	2946	1091	3101	1367
114	-0.32	-0.59	0.36	0.26	-0.67	-0.15	-0.06	-0.67	0.29	-0.74	-0.51	0.12	-0.05	0.08	0.08	0.04	-0.07	-0.65	-0.51	-0.38	-0.27	-0.66	0.07	0	-0.68	-0.25	-0.37	-0.6	0.09
115	-0.07	-0.04	0.4	0.28	-0.11	0.1	0.19	-0.16	0.22	-0.19	-0.03	0.25	0.07	0.16	0.23	0.16	0.11	-0.19	-0.01	0.08	0.02	-0.07	0.15	0.06	-0.08	0.04	-0.08	-0.05	0.06
116	-3.93	-4.53	-2.82	-2.84	-4.89	-3.42	-3.44	-5.01	-2.69	-4.85	-4.59	-3.02	-3.39	-3.05	-3	-3.35	-3.58	-4.64	-4.63	-4.04	-3.81	-4.6	-3.12	-3.04	-5.07	-3.47	-3.94	-4.98	-2.84
117	-3.86	-4.82	-3.28	-3.2	-5.33	-2.77	-4.14	-5.31	-2.19	-6.03	-5.16	-2.76	-2.43	-4.29	-3.7	-2.95	-2.87	-4.86	-4.7	-4.54	-4.66	-4.7	-3.81	-3.84	-5.72	-3.77	-4.5	-5.79	-3.12
118	-0.11	-0.22	0.11	0.3	-0.27	0.17	-0.23	-0.33	0.4	-0.48	-0.4	0.22	0.16	0.05	0.35	0.25	0.27	-0.49	0.2	0.03	-0.38	0.44	0.11	0.2	-0.13	-0.31	-0.05	-0.26	1
119	-0.01	-0.09	-0.29	-0.13	0.23	-0.13	-0.1	0.2	-0.2	0.21	0.38	-0.12	-0.1	0.08	-0.04	-0.19	-0.13	0	0.4	0.27	0.08	0	0.02	0	0.34	0.1	0	0.43	0.06
120	-0.38	-0.51	0.66	0.41	-0.67	0.04	0.18	-0.68	0.16	-0.8	-0.47	0.39	-0.08	0.17	0.17	0.25	0	-0.72	-0.62	-0.27	-0.4	-0.74	0.06	-0.15	-0.78	-0.24	-0.46	-0.65	-0.28
121	-0.54	-0.46	0.31	0.34	-0.43	-0.12	0.67	-0.98	0.17	-1.03	-0.93	0.71	0.32	0.37	0.41	0.52	0.24	-0.53	-0.41	-0.5	-0.04	-2.09	-0.05	-0.41	-0.8	0.51	1.1	-0.63	-0.38
122	-0.36	-0.58	0.2	0.47	-0.89	-0.43	0.51	-1.46	0.75	-0.77	-1.22	0.54	0.75	-0.06	0.53	0.14	0.29	-1.32	-0.18	-0.3	0.24	-0.32	-1.41	1.13	0.58	0.87	0.91	-1.9	0.33

Table S12 Amino acid descriptors (2D) used in this study.

	S	S	S	S	S	S	T	T	T	T	T	T	T	T	T	T	T	T	T	T	T	T	T	T	T	T
	R	S	T	V	W	Y	A	C	D	E	F	G	H	I	K	L	M	N	P	Q	R	S	T	V	W	Y
No.	SR	SS	ST	SV	SW	SY	TA	TC	TD	TE	TF	TG	TH	TI	TK	TL	TM	TN	TP	TQ	TR	TS	TT	TV	TW	TY
123	-0.4	0	-0.2	0	-0.6	-0.6	0	-0.3	-0.3	-0.3	-0.7	0	-0.7	-0.6	0	-0.3	-0.6	-0.4	0	-0.5	-0.6	-0.2	-0.5	-0.3	-0.9	-0.8
124	-2.6	-2.4	-2.9	-3.1	-3.1	-3.1	-3.2	-3.5	-3.2	-2.7	-3.4	0	-3.2	-3.8	-2.8	-3.3	-3.8	-3.4	-2.8	-3.1	-3.1	-2.9	-3.5	-3.9	-3.7	-3.6
125	0	-0.6	-0.5	0.3	-0.1	-0.1	-0.3	0	-0.6	-0.2	0	0	-0.6	0	0.6	0.3	0.2	-0.3	0.3	-0.2	0.1	-0.5	-0.3	0.2	0	-0.2
126	-0.3	-0.4	-0.2	0.3	0.2	-0.1	0	0	-0.3	-0.2	0.1	0	-0.1	0.1	-0.1	0.2	0.1	-0.3	-0.2	-0.2	-0.1	-0.2	0	0.2	0.2	0
127	0.2	0.6	0.4	0.4	-0.1	0.1	0.5	0.5	0.5	0.4	-0.2	0.5	0.1	-0.3	0.8	0	0	0.3	0.2	0.4	0	0.4	0.1	-0.2	-0.2	-0.2
128	0.3	0.5	0.2	0.4	-0.2	0.1	0.6	0.1	0.1	0.2	-0.2	0.6	0	-0.2	0.5	0	-0.1	0.2	0.5	0.1	0	0.2	0.2	-0.1	-0.3	-0.2
129	0.2	0.2	0.2	0.4	-0.5	0	0.5	-0.5	0.1	0.1	-0.3	0.6	-0.2	-0.2	0.4	0	-0.3	0.1	0.3	0	0	0.2	0.2	-0.1	-0.6	-0.2
130	-0.2	0.3	0.5	0.3	-0.5	-0.6	0.2	-0.1	0.9	1	-0.8	0.2	-0.6	0.3	1.2	0.3	0.5	0.5	0.6	0.6	-0.1	0.5	0.6	0.3	-0.8	-0.6
131	-0.4	0.1	0.2	0.4	-0.7	-0.6	0.2	0	0.3	0.5	-0.6	0.2	-0.5	0.4	0.8	0.4	0.3	0.4	0.5	0.4	-0.3	0.2	0.3	0.3	-0.6	-0.5
132	-0.8	-0.5	-0.5	-0.2	-0.8	-0.8	-0.1	-0.4	-0.4	-0.4	-0.8	0	-0.9	-0.3	-0.2	-0.3	-0.2	-0.4	0.2	-0.4	-0.9	-0.5	-0.5	-0.3	-0.8	-0.8
133	5.6	4.9	4.8	5.1	5.8	5.8	5.2	5.6	5.5	5.4	5.7	5.9	5.5	5.6	5.3	5.6	5.5	5.3	5.4	5.6	5.9	4.8	5.4	5.6	6.1	5.9
134	5.4	5	5.2	5.4	5.7	5.9	5.1	5.5	5.3	5.2	6.1	5.5	5.6	5.8	5.7	5.8	5.9	5.5	5.5	5.3	6	5.2	5.4	5.7	6.5	6.1
135	5.7	5	5.2	5.4	5.6	6.1	4.9	6	5.2	5.3	5.9	5	5.9	6.1	5.6	5.9	5.7	5.2	5.3	6.3	6	5.2	5.5	5.8	6.5	6.5
136	-0.0012	-0.0008	-0.0024	-0.0004	-0.0023	-0.0019	-0.0002	0.0026	0.0022	0.0009	0.0041	0.0011	-0.0059	-0.0042	-0.0059	0.0038	-0.0022	-0.0059	0.0054	0.001	0.004	-0.0024	0.0033	0.0041	0.0038	-0.0012
137	-0.0255	-0.2096	-0.3238	0.1537	0.3655	0.3161	-0.0468	0.2754	-0.3658	-0.2914	0.3747	-0.2455	-0.2562	0.2867	-0.1322	0.3101	0.3009	-0.2853	0.0284	-0.2301	-0.0531	-0.3238	-0.1955	0.1476	0.3361	0.3658
138	-0.1882	-0.1893	-0.0778	0.2547	-0.0315	-0.0216	0.0053	-0.0289	-0.0437	-0.1265	0.1481	-0.0864	-0.089	0.1143	-0.13	0.1572	0.1044	-0.1505	-0.1338	-0.16	-0.058	-0.0778	-0.0822	0.0831	0.038	0.0679
139	-0.0918	0.0117	-0.0139	0.0375	0.0189	0.0014	0.0474	-0.044	0.0076	-0.0213	0.0311	-0.008	-0.0351	0.0042	-0.0157	0.0107	-0.0095	-0.0265	0.0245	-0.0182	-0.0465	-0.0139	-0.0161	0.0102	0.0165	0.0063
140	-0.0008	-0.0144	0.0067	-0.0001	-0.031	-0.0309	0.0064	-0.0133	-0.0128	-0.0025	0.0108	-0.0163	0.0119	-0.0215	0.0229	0.0053	0.0057	0.0057	0.0143	0.0165	0.0352	0.0067	-0.0056	-0.0145	0.0839	-0.0271
141	-0.0036	-0.0025	-0.0019	0.0052	0.0011	0.0007	0.0012	-5E-05	-0.0017	-0.0022	0.0046	-0.0022	-0.0021	0.0023	-0.0013	0.0038	0.0024	-0.0025	-0.0005	-0.0019	-0.0008	-0.0019	-0.0019	0.0018	0.0047	0.0016
142	0.24	-0.36	-0.4	-1.19	-0.72	-0.62	-0.78	-1.6	0.08	0.31	-1.08	-1.23	-0.4	-1.52	0.09	-1.48	-1.12	-0.23	0	-0.03	0.09	-0.4	-0.37	-1.38	-1.22	-0.74
143	-0.81	-1.03	-1.31	-1.83	-1.5	-1.61	-1.87	-2.39	-1	-1.17	-1.52	-1.84	-1.29	-2	-1.12	-1.8	-1.4	-0.9	-0.72	-1.03	-0.89	-1.31	-1.29	-1.82	-1	-1.46
144	0.55	0.87	0.54	0.2	0.14	0.23	0.3	-0.37	0.51	0.81	0.08	0.38	0.31	-0.28	1.1	-0.05	0.46	0.54	0.67	0.81	0.8	0.54	0.69	-0.1	-0.19	0.27
145	0.83	0.5	-0.16	-0.1	-0.34	-0.32	-0.04	-1.14	1.19	1.13	-0.61	0.21	0.02	-0.65	1.3	-0.34	-0.21	0.63	1.15	1.11	1.35	0.96	0.29	-0.57	-0.4	-0.37
146	1.05	0.51	0.69	0.1	0.15	0.34	0.36	-0.49	0.54	0.54	0.04	-0.04	0.28	-0.32	1.48	-0.09	0.04	0.62	0.83	0.62	0.92	0.48	0.56	-0.01	-0.19	-0.12
147	0.65	0.33	0.47	0.12	-0.03	0.05	-0.34	-0.97	0.05	1.06	-0.32	-0.26	-0.11	-0.54	0.83	-0.42	0.2	0.19	0.26	0.68	0.37	0.13	0.41	-0.17	-0.1	-0.27
148	37.884	24.772	27.865	30.157	42.483	37.999	27.063	29.963	29.76	32.854	40.29	23.97	35.877	36.344	38.777	36.344	36.15	31.392	31.917	34.485	40.977	27.865	30.959	33.25	45.577	41.092
149	1	1	1	1	1	1	1	1	1	1	1	1	0	1	1	1	1	1	1	1	1	1	1	1	0	1
150	0	0	0	0	0	0	0	0	0	0	0	0	0	0	0	0	0	0	0	0	0	0	0	0	0	0
151	0	0	0	0	0	0	0	0	0	0	0	0	2	0	0	0	0	0	0	0	0	0	0	0	1	0
152	2	2	2	2	2	2	2	2	2	2	2	2	4	2	2	2	2	2	2	2	2	2	2	2	3	2
153	2	3	3	2	2	3	2	2	2	2	2	2	4	2	2	2	2	3	2	3	2	3	3	2	2	3
154	2	2	2	2	2	2	2	2	4	4	2	2	2	2	2	2	2	2	2	2	2	2	2	2	2	2
155	0	0	0	0	9	6	0	0	0	0	6	0	5	0	0	0	0	0	0	0	0	0	0	0	9	6
156	4	1	1	1	1	1	1	1	1	1	1	1	1	1	2	1	1	1	1	1	4	1	1	1	1	1
157	38	25	28	30	38	35	27	28	29	32	37	24	34	36	39	36	34	31	31	34	41	28	31	33	41	38
158	2	3	3	2	3	3	2	2	2	2	2	2	4	2	2	2	2	3	1	3	2	3	3	2	3	3
159	4	6	6	4	5	6	4	4	4	4	4	4	8	4	4	4	4	6	3	6	4	6	6	4	5	6
160	18	13	14	14	21	19	13	14	16	17	19	12	18	16	17	16	16	16	15	17	19	14	15	15	22	20
161	6	4	5	6	12	10	5	5	5	6	11	4	7	8	8	8	8	5	7	6	7	5	6	7	13	11
162	64.844	43.957	48.042	49.207	63.877	58.896	45.428	51.651	51.266	55.358	59.585	41.51	59.754	56.459	62.049	56.459	59.386	54.612	51.051	58.593	68.723	48.042	51.97	52.877	67.591	62.647
163	1.7064	1.7583	1.7158	1.6402	1.681	1.6827	1.6825	1.8447	1.7678	1.7299	1.6104	1.7296	1.7575	1.5683	1.591	1.5683	1.7466	1.7617	1.6468	1.7233	1.6762	1.7158	1.6765	1.6023	1.6486	1.6486
164	9	6	7	8	14	12	7	7	8	9	13	6	10	10	10	10	9	8	9	9	10	7	8	9	15	13
165	20	12	14	16	17	16	14	14	13	15	18	12	16	20	22	20	18	15	16	17	22	14	16	18	19	18
166	5	2	2	2	3	2	2	2	2	2	2	2	4	2	3	2	2	3	2	3	5	2	2	2	3	2
167	4	5	5	4	4	5	4	4	6	6	4	4	4	4	4	4	4	5	4	5	4	5	5	4	4	5
168	0	0	0	0	0	0	0	1	0	0	0	0	0	0	0	0	1	0	0	0	0	0	0	0	0	0
169	4.0026	3.9466	4.1748	4.1748	1.9079	2.4485	3.9368	4.0938	4.295	4.2523	2.4794	3.6564	2.4656	4.4083	4.1529	4.2911	4.177	4.295	2.6231	4.2523	4.1226	4.0938	4.3002	4.3002	1.9182	2.4859
170	-2.63	-2.5483	-2.5889	-2.6483	-2.5573	-2.5618	-2.5853	-2.5863	-2.5886	-2.6112	-2.5916	-2.5693	-2.5871	-2.6858	-2.6646	-2.6787	-2.6134	-2.5862	-2.5891	-2.6079	-2.6369	-2.5848	-2.6068	-2.6538	-2.5894	-2.5915
171	-0.5405	-0.5476	-0.5476	-0.502	-0.5474	-0.6113	-0.5058	-0.5477	-0.5491	-0.5491	-0.5477	-0.5355	-0.5477	-0.4752	-0.5004	-0.4979	-0.4774	-0.5477	-0.5477	-0.5477	-0.524	-0.5476	-0.5464	-0.478	-0.5452	-0.6113
172	0.4899	0.5434	0.5015	0.4261	0.5902	0.6508	0.4387	0.5284	0.4779	0.4814	0.5828	0.4666	0.5388	0.4206	0.4351	0.4332	0.3883	0.4564	0.5078	0.4648	0.4819	0.5329	0.4961	0.422	0.5869	0.6087
173	2.6434	2.6163	2.6318	2.644	2.6173	2.6167	2.6611	2.6626	2.6633	2.6672	2.6635	2.6555	2.6641	2.6841	2.6783	2.6774	2.6674	2.6659	2.737	2.6694	2.6736	2.6636	2.6704	2.6753	2.6638	2.6635
174	-3.3175	-3.3176	-3.3176	-3.3175	-3.3175	-3.3175	-3.3209	-3.321	-3.3209	-3.3209	-3.321	-3.3207	-3.321	-3.321	-3.3209	-3.3209	-3.3209	-3.3209	-3.3189	-3.3209	-3.3209	-3.321	-3.3211	-3.321	-3.321	-3.321
175	-0.591	-0.6896	-0.5848	-0.4921	-0.5921	-0.642	-0.4845	-0.6039	-0.558	-0.5519	-0.5381	-0.5087	-0.5626	-0.4575	-0.4811	-0.4589	-0.4215	-0.5695	-0.4738	-0.5632	-0.5412	-0.6047	-0.5721	-0.4649	-0.5492	-0.5832
176	0.432	0.4733	0.4356	0.3802	0.6318	0.547	0.3808	0.468	0.4232	0.4246	0.4937	0.3844	0.4725	0.377	0.3802	0.3801	0.3979	0.3909	0.4343	0.393	0.4246	0.466	0.4346	0.3772	0.6028	0.4858
177	2.5915	2.2849	2.4356	2.6123	2.4537	2.4442	2.431	2.4287	2.4315	2.5362	2.4449	2.4221	2.4327	2.683	2.6641	2.6891	2.5457	2.4327	2.6847	2.5409	2.5921	2.4248	2.4499	2.6128	2.4608	2.4539
178	-2.3916	-2.3488	-2.3663	-2.4007	-2.3493	-2.35	-2.3951	-2.3944	-2.3956	-2.4013	-2.3962	-2.39	-2.3959	-2.4398	-2.4397	-2.4334	-2.4004	-2.396	-2.3982	-2.4018	-2.4122	-2.3958	-2.4027	-2.4188	-2.396	-2.3962
179	-0.3664	-0.4121	-0.3415	-0.2967	-0.4206	-0.4748	-0.2961	-0.3676	-0.3333	-0.3334	-0.3918	-0.3076	-0.3768	-0.2834	-0.2942	-0.2933	-0.2551	-0.3419	-0.3157	-0.3535	-0.3334	-0.3708	-0.3291	-0.2846	-0.3963	-0.4467
180	0.6174	0.6774	0.6048	0.5315	0.7471	0.8009	0.5311	0.6323	0.5803	0.5807	0.7123	0.549	0.6385	0.5159	0.5285	0.5272	0.4765	0.6063	0.5796	0.626	0.5807	0.631	0.5877	0.5174	0.734	0.7348
181	2.8701	2.8063	2.8403	2.8766	2.8233	2.8203	2.8417	2.853	2.8496	2.8645	2.8514	2.8264	2.8501	2.9111	2.891	2.8995	2.876	2.8531	2.9793	2.8676	2.8819	2.8446	2.8632	2.8884	2.8528	2.8514
182	23.418	15.332	17.519	19.705	21.459	19.705	17.519	19.799	18.342	20.528	21.892	15.332	21.685	24.078	24.945	24.078	24.172	18.91	21.025	21.097	25.605	17.519	19.705	21.892	23.645	21.892
183	8	5	5	5	6	6	4	5	6	7	6	4	6	6	8	6	7	6	4	7	8	5	5	5	6	6
184	0.4706	0.4167	0.3846	0.3846	0.2727	0.3158	0.3333	0.3846	0.4	0.4375	0.3158	0.3636	0.3333	0.4	0.5	0.4	0.4667	0.4	0.2667	0.4375	0.4444	0.3846	0.3571	0.3571	0.2609	0.3
185	0	0	0	0	10	6	0	0	0	0	6	0	5	0	0	0	0	0	0	0	0	0	0	0	10	6
186	37	24	27	29	39	35	26	27	28	31	37	23	34	35	38	35	33	30	31	33	40	27	30	32	42	38
187	3	2	2	2	2	2	2	2	3	3	2	2	2	2	2	2	2	3	2	3	3	2	2	2	2	2
188	17	12	13	13	22	19	12	13	15	16	19	11	18	15	16	15	15	15	15	16	18	13	14	14	23	20
189	7	5	5	5	5	5	5	5	5	5	5	5	5	6	8	6	7	5	5	5	7	5	5	5	5	5
190	10	6	6	6	7	7	5	6	7	8	7	5	7	7	9	7	8	7	4	8	10	6	6	6	7	7
191	0.5882	0.5	0.4615	0.4615	0.3182	0.3684	0.4167	0.4615	0.4667	0.5	0.3684	0.4545	0.3889	0.4667	0.5625	0.4667	0.5333	0.4667	0.2667	0.5	0.5556	0.4615	0.4286	0.4286	0.3043	0.35
192	34	22	25	27	27	27	24	25	25	28	29	21	27	33	36	33	31	27	29	30	37	25	28	30	30	30
193	14.129	10.431	11.301	11.301	15.405	14.414	10.594	11.301	12.878	13.585	14.414	9.7236	13.707	12.878	13.422	12.878	12.715	12.878	11.585	13.585	15	11.301	12.171	12.171	16.276	15.284
194	10.017	6.8952	7.7654	8.3182	11.335	10.205	7.6111	8.6599	8.6347	9.3418	10.705	6.7409	9.9201	9.8956	10.147	9.8956	10.25	8.8038	8.6797	9.5109	10.887	7.7654	8.6357	9.1885	12.205	11.075
195	5.4831	3.5689	4.4392	5.4392	7.9557	6.8783	4.7321	4.4392	4.9392	5.6463	7.8259	3.8618	6.0939	7.0165	6.5605	7.0165	6.1463	4.9392	5.8534	5.6463	6.3534	4.4392	5.3094	6.3094	8.8259	7.7486
196	5.7152	3.7236	4.5939	5.5939	8.9912	7.7067	4.8868	4.5939	5.1712	5.8783	8.7067	4.0165	6.5854	7.1712	6.7152	7.1712	6.301	5.1712	6.0081	5.8783	6.5854	4.5939	5.4641	6.4641	9.8614	8.577
197	8.3791	6.0233	6.396	6.396	10.024	8.9348	5.8579	6.396	7.2518	7.7518	8.9136	5.4473	8.4136	7.3066	7.896	7.2518	7.396	7.2518	6.9473	7.7518	8.7518	6.396	6.7686	6.7686	10.397	9.3074
198	5.4503	3.5856	4.03	4.3492	6.5276	5.6679	3.8829	4.6625	4.4756	4.9756	5.978	3.4353	5.4152	5.3316	5.7138	5.2767	6.3045	4.5601	4.9731	5.0601	5.8947	4.03	4.4744	4.7936	6.972	6.1123
199	2.3938	1.3938	1.8963	2.4736	4.1134	3.5688	2.0654	1.8963	2.2498	2.7498	4.1605	1.5529	2.5385	3.5141	3.3963	3.4592	2.3963	2.2498	2.8963	2.7498	2.8963	1.8963	2.3987	2.9761	4.6159	4.0712
200	2.4832	1.4832	1.9856	2.5629	5.2828	4.5244	2.1547	1.9856	2.3938	2.8938	5.2103	1.6523	2.8021	3.6034	3.4856	3.5485	2.4856	2.3938	2.9856	2.8938	2.9856	1.9856	2.488	3.0654	5.7853	5.0268
201	2	2	3	2	2	2	3	3	3	3	3	2	3	4	3	3	3	3	3	3	3	3	4	3	3	3
202	2	2	3	2	2	2	3	3	3	3	3	2	3	4	3	3	3	3	3	3	3	3	4	3	3	3
203	0.8039	0.8781	0.8476	0.788	0.7859	0.8036	0.8102	0.868	0.8817	0.8557	0.7614	0.8376	0.8162	0.7541	0.7603	0.7541	0.8181	0.848	0.7969	0.8257	0.7879	0.8476	0.8226	0.7696	0.7729	0.788
204	11	7	7	7	11	11	7	7	8	9	10	7	9	8	10	8	9	8	7	9	11	7	7	7	11	11
205	1	0	0	0	0	0	0	0	-1	-1	0	0	0	0	1	0	0	0	0	0	1	0	0	0	0	0

	V	V	V	V	V	V	V	V	V	V	V	V	V	V	V	V	V	V	V	V	W	W	W	W	W	W	W	W	W
	A	C	D	E	F	G	H	I	K	L	M	N	P	Q	R	S	T	V	W	Y	A	C	D	E	F	G	H	I	K
No.	VA	VC	VD	VE	VF	VG	VH	VI	VK	VL	VM	VN	VP	VQ	VR	VS	VT	VV	VW	VY	WA	WC	WD	WE	WF	WG	WH	WI	WK
123	-0.3	-0.5	0.4	0	-1.5	0	-0.5	-1.2	0.1	-1.2	-1	0	0	-0.4	-0.5	0	-0.3	-1.1	-1.6	-1.2	-0.8	-1.3	-0.6	-0.8	-2	0	-1.5	-1.8	-0.8
124	-4.1	-4.3	-2.8	-2.8	-4.6	0	-3.5	-5	-3.1	-4.7	-4.7	-3.5	-3.2	-3.4	-3.5	-3.1	-3.9	-5.1	-4.8	-4.5	-3.8	-4.3	-3.2	-2.8	-4.4	0	-3.6	-4.7	-3.3
125	-0.6	-0.8	0.7	0.4	-0.8	0.2	-0.2	-0.7	0.9	-0.6	-0.5	0.2	0.1	0.2	0.3	0.3	0.2	-0.9	-0.8	-0.3	-0.7	-0.5	-0.2	0.1	-1.3	-0.5	-1.2	-0.9	0.1
126	-0.1	0	0.6	0.4	0	0	0.5	-0.1	0.4	-0.1	0.1	0.3	0.1	0.2	0.4	0.3	0.2	-0.2	0.1	0.1	0	0.2	0	0	-0.1	0	0	0.1	-0.1
127	-0.4	-0.6	1	0.5	-1.1	0.5	0	-1.2	0.7	-1.2	-0.8	0.5	-0.1	0.2	0	0.4	-0.2	-1.2	-1.1	-0.8	-0.6	-0.7	0	-0.1	-1.5	0	-0.9	-1.3	-0.1
128	-0.1	-0.5	0.8	0.5	-0.9	0.8	-0.1	-1	0.5	-0.9	-0.8	0.5	0.2	0.3	0.1	0.4	-0.1	-0.8	-0.9	-0.7	-0.5	-0.7	0	-0.2	-1.3	0.2	-0.7	-1.1	-0.1
129	0	-0.7	0.6	0.4	-0.9	0.7	-0.3	-1	0.4	-0.9	-0.8	0.3	0.2	0.1	0	0.4	-0.1	-0.7	-1	-0.7	-0.6	-1.5	-0.4	-0.5	-1.4	-0.2	-1.2	-1.2	-0.5
130	-0.5	-0.3	1	0.8	-1.3	0.1	-0.7	-0.5	0.7	-0.4	-0.2	0.7	0.3	0.4	-0.3	0.3	0.3	-0.6	-1.1	-1	0.1	-1.2	-0.2	-0.2	-1.2	0.3	-0.9	-1.2	0
131	-0.2	-0.3	0.8	0.9	-1	0.3	-0.4	-0.2	1	-0.4	-0.2	0.7	0.3	0.6	-0.3	0.4	0.3	-0.2	-0.9	-0.7	0.3	-1.1	-0.5	-0.3	-1.2	0.2	-0.8	-1	-0.2
132	-0.4	-0.9	0.4	0.2	-1.4	0.1	-0.9	-0.9	0.1	-1	-0.8	0.1	0.2	0	-0.6	-0.2	-0.3	-1	-1.1	-1.1	0.2	-1.3	-0.5	-0.6	-1.4	0.1	-1.1	-1.2	-0.5
133	4.9	5.4	5.5	5.3	5.6	5.2	5.4	5.6	5.7	5.6	5.5	5.4	5.8	5.6	5.6	5.1	5.6	5.5	6.3	5.9	5.8	5.3	6.6	6.3	6.2	5.8	5.5	6.2	5.6
134	5.4	5.7	5.6	5.4	6	4.6	5.6	6	5.9	6	5.9	5.6	5.7	5.4	6	5.4	5.7	5.8	6.3	6	5.9	6.1	6	6.2	6.5	5.2	6	6.3	5.7
135	5.2	5.8	5.6	5.5	6.1	4.9	5.8	6.1	6.3	6.1	5.9	5.4	5.5	6	6	5.4	5.8	5.8	6.4	6.1	5.8	6.5	6.3	6.8	6.3	4.5	6.6	6.6	5
136	0.0036	0.0003	9E-05	0.0014	8E-06	0.002	-0.0069	0.0026	-0.007	-0.0019	-0.0053	-0.001	0.0014	3E-05	-0.0052	-0.0004	0.0041	0.0014	-0.0015	-0.0022	-0.0097	-0.0352	-0.0078	-0.0094	0.0061	-0.0124	-0.0067	0.0027	-0.0017
137	-0.2713	0.0978	0.4466	0.2276	-0.0823	0.2311	0.1579	-0.1963	0.2378	-0.1064	0.0064	0.4745	0.3976	0.0858	0.3328	0.1537	0.1476	-0.2973	0.1217	0.0206	0.2	0.094	0.5038	0.1607	-0.1359	0.2434	-0.1723	-0.1598	-0.2459
138	-0.0235	-0.0185	0.4962	0.4169	-0.1227	0.142	0.3418	-0.2748	0.3311	-0.2794	-0.0739	0.4019	0.1512	0.2812	0.3325	0.2547	0.0831	-0.2593	0.075	0.041	-0.1126	-0.1733	-0.0089	-0.098	-0.0572	-0.0944	-0.028	0.134	0.1432
139	-0.0728	0.0077	0.1478	0.119	-0.0486	0.0234	0.0708	-0.1361	0.1222	-0.067	-0.0151	0.108	0.1117	0.1181	0.1729	0.0375	0.0102	-0.1076	0.0159	0.0332	0.0873	-0.0257	0.0226	-0.0492	-0.1111	0.1041	-0.1184	0.0343	0.147
140	-0.0201	-0.0156	0.0341	0.0478	-0.0063	0.0042	0.0164	-0.0423	0.0195	-0.0072	0.0343	0.0499	0.0616	0.0177	0.0362	-0.0001	-0.0145	-0.0668	0.0379	0.0335	0.0105	-0.0989	-0.0321	-0.0116	-0.0297	-0.0624	-0.0561	0.0904	0.0335
141	-0.0059	-0.0093	0.013	0.0125	-0.0101	0.0037	0.0067	-0.0216	0.0116	-0.0143	-0.0033	0.0117	0.0097	0.0095	0.0121	0.0052	0.0018	-0.0208	0.0008	0.0001	0.0019	-0.0129	0.0034	0.002	-0.0094	0.0006	-0.0062	0.0023	0.0062
142	-1.84	-2.72	-0.37	-0.23	-2.39	-1.98	-0.88	-2.66	-0.37	-2.57	-2.05	-0.71	-0.97	-0.32	-0.47	-1.19	-1.38	-2.35	-1.82	-1.76	-1.4	-2.02	-0.04	-0.43	-2.4	-1.58	-0.78	-2.4	-0.55
143	-3.07	-3.33	-1.08	-1.07	-3.21	-2.78	-1.7	-3.5	-1.21	-3.5	-2.96	-1.33	-1.53	-1.33	-1.26	-1.83	-1.82	-3.1	-2.4	-2.4	-2.2	-3.03	-1	-1.21	-2.81	-2.58	-1.5	-2.9	-1.55
144	-0.18	-1.22	0.59	0.74	-0.77	0.04	0.09	-0.9	0.81	-0.93	-0.31	0.54	0.17	0.23	0.43	0.2	-0.1	-0.31	-0.57	-0.38	-0.28	-0.63	0.43	0.44	-1.02	-0.17	-0.44	-1.28	0.23
145	-1.45	-2.35	0.86	0.39	-2.25	-1.09	-0.56	-2.45	0.13	-2.26	-1.81	0.23	-0.06	0.15	0.44	-0.54	-0.73	-2.31	-1.79	-1.38	-0.92	-0.52	1.85	0.58	-1.63	-0.59	-49	-1.82	-0.14
146	-0.43	-1.62	0.77	0.86	-1.34	-0.35	-0.21	-1.36	0.64	-1.32	-0.82	0.23	0.12	0.48	0.32	0.18	-0.15	-1.14	-0.92	-0.78	-0.72	-1.3	-0.1	0.23	-1.41	-0.71	-0.2	-1.4	0.32
147	-1.55	-1.62	0.34	0.48	-1.3	-1.13	0.03	-1.64	0.36	-1.64	-0.79	-0.23	-0.79	0.15	0.17	-0.24	-0.32	-1.42	-0.64	-0.84	-1.44	-0.67	-0.48	0.04	-1.23	-1.41	-1	-1.65	0
148	29.355	32.255	32.052	35.145	42.582	26.261	38.168	38.635	41.069	38.635	38.442	33.683	34.208	36.777	43.269	30.157	33.25	35.542	47.869	43.384	41.681	44.581	44.379	47.472	54.909	38.588	50.495	50.962	53.396
149	1	1	1	1	1	1	1	1	1	1	1	1	1	1	1	1	1	1	0	1	1	0	0	0	0	1	0	0	0
150	0	0	0	0	0	0	0	0	0	0	0	0	0	0	0	0	0	0	0	0	0	0	0	0	0	0	0	0	0
151	0	0	0	0	0	0	0	0	0	0	0	0	0	0	0	0	0	0	1	0	0	1	1	1	1	0	2	1	1
152	2	2	2	2	2	2	2	2	2	2	2	2	1	2	2	2	2	2	3	2	2	3	3	3	3	2	4	3	3
153	1	1	1	1	1	1	3	1	1	1	1	2	1	2	1	2	2	1	1	2	1	1	1	1	1	1	3	1	1
154	2	2	4	4	2	2	2	2	2	2	2	2	2	2	2	2	2	2	2	2	2	2	4	4	2	2	2	2	2
155	0	0	0	0	6	0	5	0	0	0	0	0	0	0	0	0	0	0	9	6	9	9	9	9	15	9	14	9	9
156	1	1	1	1	1	1	1	1	2	1	1	1	1	1	4	1	1	1	1	1	1	1	1	1	1	1	1	1	2
157	29	30	31	34	39	26	36	38	41	38	36	33	33	36	43	30	33	35	43	40	37	38	39	42	47	34	44	46	49
158	1	1	1	1	1	1	3	1	1	1	1	2	0	2	1	2	2	1	2	2	2	2	2	2	2	2	4	2	2
159	2	2	2	2	2	2	6	2	2	2	2	4	1	4	2	4	4	2	3	4	3	3	3	3	3	3	7	3	3
160	13	14	16	17	19	12	18	16	17	16	16	16	15	17	19	14	15	15	22	20	20	21	23	24	26	19	25	23	24
161	6	6	6	7	12	5	8	9	9	9	9	6	8	7	8	6	7	8	14	12	12	12	12	13	18	11	14	15	15
162	46.127	52.452	52.838	56.658	59.634	42.502	60.25	56.505	62.057	56.505	59.705	55.694	51.432	59.433	68.872	49.207	52.877	53.112	67.581	63.136	60.45	67.122	67.163	71.054	72.879	56.755	73.653	71.045	76.783
163	1.5906	1.7484	1.7044	1.6664	1.5291	1.6347	1.6736	1.487	1.5136	1.487	1.6585	1.6877	1.5586	1.6509	1.6017	1.6402	1.6023	1.5175	1.5716	1.5784	1.6338	1.7664	1.7221	1.6918	1.5506	1.6693	1.6739	1.5445	1.567
164	8	8	9	10	14	7	11	11	11	11	10	9	10	10	11	8	9	10	16	14	14	14	15	16	20	13	17	17	17
165	16	16	15	17	20	14	18	22	24	22	20	17	18	19	24	16	18	20	21	20	17	17	16	18	21	15	19	23	25
166	2	2	2	2	2	2	4	2	3	2	2	3	2	3	5	2	2	2	3	2	3	3	3	3	3	3	5	3	4
167	3	3	5	5	3	3	3	3	3	3	3	4	3	4	3	4	4	3	3	4	3	3	5	5	3	3	3	3	3
168	0	1	0	0	0	0	0	0	0	0	1	0	0	0	0	0	0	0	0	0	0	1	0	0	0	0	0	0	0
169	3.9368	4.0938	4.295	4.2523	2.4794	3.6564	2.4656	4.4083	4.1529	4.2911	4.177	4.295	2.6231	4.2523	4.1226	4.0938	4.3002	4.3002	1.9182	2.4859	1.8055	1.8193	1.8321	1.8102	1.4749	1.772	1.4774	1.857	1.7881
170	-2.6397	-2.6399	-2.6407	-2.6487	-2.6417	-2.6329	-2.6402	-2.6933	-2.6745	-2.6863	-2.6493	-2.6397	-2.6432	-2.6474	-2.6588	-2.6392	-2.6468	-2.6702	-2.641	-2.6417	-2.557	-2.5591	-2.5633	-2.5987	-2.5687	-2.5277	-2.5605	-2.684	-2.6625
171	-0.4772	-0.5127	-0.5477	-0.5477	-0.5331	-0.5012	-0.525	-0.4363	-0.4691	-0.4647	-0.4367	-0.5118	-0.4672	-0.5164	-0.5017	-0.5098	-0.4652	-0.4399	-0.5418	-0.5477	-0.5477	-0.5469	-0.5508	-0.5502	-0.5578	-0.548	-0.5573	-0.5407	-0.5477
172	0.3727	0.4335	0.386	0.386	0.5501	0.3761	0.4402	0.3715	0.3725	0.3725	0.359	0.4091	0.3989	0.4123	0.386	0.4352	0.422	0.3716	0.5566	0.5352	0.5562	0.5834	0.5629	0.565	0.5961	0.6013	0.5863	0.5387	0.5472
173	2.6826	2.6837	2.6841	2.6867	2.6842	2.6786	2.6847	2.6973	2.6927	2.6927	2.6868	2.6859	2.7425	2.688	2.6904	2.6844	2.6889	2.6918	2.6844	2.6843	2.6161	2.6189	2.6202	2.6287	2.6207	2.6068	2.6219	2.6632	2.6574
174	-3.3144	-3.3145	-3.3145	-3.3144	-3.3145	-3.3142	-3.3145	-3.3145	-3.3145	-3.3145	-3.3145	-3.3145	-3.3125	-3.3144	-3.3145	-3.3146	-3.3146	-3.3145	-3.3145	-3.3145	-3.3114	-3.3115	-3.3115	-3.3115	-3.3115	-3.3112	-3.3115	-3.3116	-3.3115
175	-0.3983	-0.472	-0.4579	-0.4484	-0.4596	-0.431	-0.4649	-0.3837	-0.3951	-0.3937	-0.3801	-0.4625	-0.4272	-0.4612	-0.4105	-0.4723	-0.4603	-0.3851	-0.436	-0.5381	-0.4625	-0.5935	-0.5311	-0.5283	-0.5129	-0.4684	-0.5294	-0.4602	-0.4621
176	0.2959	0.3833	0.3367	0.3367	0.5453	0.2963	0.438	0.2958	0.2959	0.2959	0.3571	0.3548	0.2928	0.3555	0.3367	0.3821	0.3696	0.2958	0.6574	0.4803	0.6535	0.6878	0.632	0.622	0.6962	0.6537	0.6878	0.6534	0.6535
177	2.605	2.6048	2.605	2.6072	2.605	2.6041	2.6049	2.6841	2.6655	2.6898	2.6072	2.605	2.6908	2.6074	2.6131	2.6045	2.6051	2.6227	2.6051	2.6051	2.4491	2.4479	2.4493	2.5364	2.4549	2.4446	2.4497	2.683	2.6641
178	-2.4395	-2.4392	-2.4398	-2.4423	-2.4401	-2.4366	-2.4399	-2.4577	-2.4545	-2.4533	-2.4419	-2.4399	-2.442	-2.4424	-2.4458	-2.4399	-2.4428	-2.449	-2.44	-2.4401	-2.3493	-2.3479	-2.3504	-2.3645	-2.352	-2.3395	-2.3512	-2.4322	-2.4352
179	-0.1721	-0.3001	-0.2577	-0.2577	-0.3924	-0.1757	-0.307	-0.1704	-0.1718	-0.1717	-0.0859	-0.2864	-0.1111	-0.2898	-0.2577	-0.3026	-0.2814	-0.1705	-0.3994	-0.4008	-0.4062	-0.4056	-0.4117	-0.4118	-0.4255	-0.4065	-0.4255	-0.3641	-0.4059
180	0.3542	0.5368	0.4778	0.4779	0.6792	0.3601	0.5748	0.3521	0.3538	0.3538	0.462	0.5224	0.2704	0.5299	0.4779	0.536	0.5147	0.3523	0.7064	0.6632	0.717	0.7432	0.7259	0.7262	0.7512	0.7543	0.745	0.7027	0.7091
181	2.8807	2.8865	2.8847	2.892	2.8854	2.8723	2.8848	2.9215	2.9057	2.912	2.8977	2.8864	2.9851	2.8935	2.9007	2.8822	2.8916	2.906	2.8861	2.8854	2.822	2.8372	2.8327	2.8526	2.8353	2.8021	2.8334	2.9071	2.8859
182	19.705	21.985	20.528	22.715	24.078	17.519	23.872	26.265	27.131	26.265	26.358	21.097	23.212	23.283	27.791	19.705	21.892	24.078	25.831	24.078	21.459	23.739	22.281	24.468	25.831	19.272	25.625	28.018	28.884
183	4	5	6	7	6	4	6	6	8	6	7	6	4	7	8	5	5	5	6	6	5	6	7	8	7	5	7	7	9
184	0.3333	0.3846	0.4	0.4375	0.3158	0.3636	0.3333	0.4	0.5	0.4	0.4667	0.4	0.2667	0.4375	0.4444	0.3846	0.3571	0.3571	0.2609	0.3	0.2381	0.2727	0.2917	0.32	0.25	0.25	0.2593	0.2917	0.36
185	0	0	0	0	6	0	5	0	0	0	0	0	0	0	0	0	0	0	10	6	10	10	10	10	16	10	15	10	10
186	28	29	30	33	39	25	36	37	40	37	35	32	33	35	42	29	32	34	44	40	38	39	40	43	49	35	46	47	50
187	2	2	3	3	2	2	2	2	2	2	2	3	2	3	3	2	2	2	2	2	2	2	3	3	2	2	2	2	2
188	12	13	15	16	19	11	18	15	16	15	15	15	15	16	18	13	14	14	23	20	21	22	24	25	28	20	27	24	25
189	5	5	5	5	5	5	5	6	8	6	7	5	5	5	7	5	5	5	5	5	4	5	4	5	4	4	4	6	8
190	5	6	7	8	7	5	7	7	9	7	8	7	4	8	10	6	6	6	7	7	6	7	8	9	8	6	8	8	10
191	0.4167	0.4615	0.4667	0.5	0.3684	0.4545	0.3889	0.4667	0.5625	0.4667	0.5333	0.4667	0.2667	0.5	0.5556	0.4615	0.4286	0.4286	0.3043	0.35	0.2857	0.3182	0.3333	0.36	0.2857	0.3	0.2963	0.3333	0.4
192	26	27	27	30	31	23	29	35	38	35	33	29	31	32	39	27	30	32	32	32	26	27	27	30	31	23	29	35	38
193	10.594	11.301	12.878	13.585	14.414	9.7236	13.707	12.878	13.422	12.878	12.715	12.878	11.585	13.585	15	11.301	12.171	12.171	16.276	15.284	14.698	15.405	16.983	17.69	18.518	13.828	17.811	16.983	17.527
194	8.1639	9.2127	9.1875	9.8946	11.258	7.2937	10.473	10.448	10.699	10.448	10.803	9.3566	9.2324	10.064	11.44	8.3182	9.1885	9.7413	12.758	11.628	11.18	12.229	12.204	12.911	14.274	10.31	13.489	13.465	13.716
195	5.7321	5.4392	5.9392	6.6463	8.8259	4.8618	7.0939	8.0165	7.5605	8.0165	7.1463	5.9392	6.8534	6.6463	7.3534	5.4392	6.3094	7.3094	9.8259	8.7486	8.2486	7.9557	8.4557	9.1628	11.342	7.3783	9.6104	10.533	10.077
196	5.8868	5.5939	6.1712	6.8783	9.7067	5.0165	7.5854	8.1712	7.7152	8.1712	7.301	6.1712	7.0081	6.8783	7.5854	5.5939	6.4641	7.4641	10.861	9.577	9.2841	8.9912	9.5685	10.276	13.104	8.4138	10.983	11.569	11.113
197	5.8579	6.396	7.2518	7.7518	8.9136	5.4473	8.4136	7.3066	7.896	7.2518	7.396	7.2518	6.9473	7.7518	8.7518	6.396	6.7686	6.7686	10.397	9.3074	9.4861	10.024	10.88	11.38	12.542	9.0754	12.042	10.935	11.524
198	4.202	4.9816	4.7947	5.2947	6.2972	3.7544	5.7343	5.6507	6.0329	5.5959	6.6237	4.8793	5.2923	5.3793	6.2138	4.3492	4.7936	5.1127	7.2912	6.4315	6.3805	7.1601	6.9732	7.4732	8.4756	5.9329	7.9128	7.8292	8.2114
199	2.6427	2.4736	2.8272	3.3272	4.7379	2.1303	3.1159	4.0914	3.9736	4.0366	2.9736	2.8272	3.4736	3.3272	3.4736	2.4736	2.9761	3.5534	5.1932	4.6486	4.2825	4.1134	4.467	4.967	6.3777	3.7701	4.7557	5.7312	5.6134
200	2.7321	2.5629	2.9712	3.4712	5.7877	2.2296	3.3794	4.1807	4.0629	4.1259	3.0629	2.9712	3.5629	3.4712	3.5629	2.5629	3.0654	3.6427	6.3626	5.6042	5.4519	5.2828	5.6911	6.1911	8.5076	4.9495	6.0993	6.9006	6.7828
201	2	2	2	2	2	1	2	3	2	2	2	2	2	2	2	2	3	2	2	2	2	2	2	2	2	1	2	3	2
202	2	2	2	2	2	1	2	3	2	2	2	2	2	2	2	2	3	2	2	2	2	2	2	2	2	1	2	3	2
203	0.7509	0.81	0.8246	0.8046	0.7229	0.77	0.7708	0.7109	0.7192	0.7109	0.7715	0.7948	0.746	0.7778	0.7483	0.788	0.7696	0.7221	0.7381	0.7492	0.7603	0.8016	0.8123	0.7983	0.7366	0.7737	0.7735	0.7289	0.7344
204	7	7	8	9	10	7	9	8	10	8	9	8	7	9	11	7	7	7	11	11	11	11	12	13	14	11	13	12	14
205	0	0	-1	-1	0	0	0	0	1	0	0	0	0	0	1	0	0	0	0	0	0	0	-1	-1	0	0	0	0	1

Table S12 Amino acid descriptors (2D) used in this study.

	S	S	S	S	S	S	T	T	T	T	T	T	T	T	T	T	T	T	T	T	T	T	T	T	T	T
	R	S	T	V	W	Y	A	C	D	E	F	G	H	I	K	L	M	N	P	Q	R	S	T	V	W	Y
No.	SR	SS	ST	SV	SW	SY	TA	TC	TD	TE	TF	TG	TH	TI	TK	TL	TM	TN	TP	TQ	TR	TS	TT	TV	TW	TY
206	-0.8454	-0.8437	-0.8436	-0.8451	-0.863	-0.8451	-0.8454	-0.8444	-0.8505	-0.8543	-0.8449	-0.8465	-0.8486	-0.845	-0.863	-0.8454	-0.8451	-0.8448	-0.8502	-0.8453	-0.8453	-0.8435	-0.8434	-0.8449	-0.863	-0.8449
207	-0.4053	-0.478	-0.462	-0.3859	-0.4396	-0.4477	-0.4055	-0.4329	-0.4971	-0.4899	-0.39	-0.444	-0.4013	-0.3672	-0.383	-0.3833	-0.3787	-0.4331	-0.4329	-0.4331	-0.4053	-0.4329	-0.4327	-0.3708	-0.4167	-0.4165
208	0.1856	0.1856	0.1856	0.1585	0.1854	0.1856	0.1522	0.1856	0.135	0.1234	0.1352	0.1583	0.1807	0.127	0.1849	0.0984	0.135	0.1359	0.1776	0.1351	0.1687	0.1856	0.1678	0.1364	0.1687	0.1688
209	2.5107	2.3027	2.3792	2.4395	2.5482	2.4706	2.3811	2.3969	2.4111	2.4636	2.5263	2.3147	2.4883	2.5697	2.5778	2.5514	2.4797	2.4448	2.5808	2.4927	2.5693	2.3986	2.4611	2.5115	2.6004	2.5355
210	-2.2352	-2.2351	-2.2351	-2.2351	-2.2351	-2.2351	-2.2353	-2.2353	-2.2353	-2.2353	-2.2353	-2.2352	-2.2353	-2.2353	-2.2355	-2.2353	-2.2353	-2.2353	-2.2353	-2.2353	-2.2353	-2.2353	-2.2353	-2.2353	-2.2353	-2.2353
211	-0.4505	-0.6252	-0.4966	-0.4574	-0.4319	-0.4427	-0.4373	-0.4511	-0.5257	-0.4926	-0.4046	-0.5036	-0.4203	-0.3984	-0.3875	-0.3934	-0.3851	-0.5024	-0.4102	-0.4511	-0.4242	-0.5164	-0.4875	-0.4146	-0.3976	-0.4113
212	0.0542	0.0542	0.0542	0.0542	0.1015	0.0568	0.0542	0.0542	0.0542	0.0542	0.0581	0.0542	0.0542	0.0542	0.0542	0.0542	0.0542	0.0542	0.0542	0.0542	0.0542	0.0542	0.0542	0.0542	0.1015	0.0568
213	2.3544	2.0367	2.1628	2.3147	2.5398	2.4135	2.18	2.1977	2.2053	2.3003	2.4339	2.0878	2.3268	2.4764	2.4622	2.4627	2.3446	2.2348	2.4556	2.3282	2.4136	2.1711	2.2611	2.3811	2.572	2.4594
214	-0.6407	-0.6407	-0.6407	-0.6407	-0.6407	-0.6407	-0.6408	-0.6408	-0.6408	-0.6408	-0.6408	-0.6408	-0.6408	-0.6408	-0.6418	-0.6408	-0.6408	-0.6408	-0.6409	-0.6408	-0.6408	-0.6408	-0.6408	-0.6408	-0.6408	-0.6408
215	-0.2225	-0.2345	-0.2228	-0.2012	-0.2266	-0.231	-0.1995	-0.1886	-0.2075	-0.2068	-0.2161	-0.208	-0.2196	-0.1893	-0.1965	-0.1943	-0.1873	-0.2159	-0.1982	-0.2161	-0.2069	-0.2167	-0.2032	-0.1907	-0.2116	-0.2196
216	0.1443	0.1599	0.1402	0.117	0.1689	0.183	0.1161	0.1357	0.1249	0.125	0.1579	0.1293	0.1366	0.107	0.1145	0.114	0.1353	0.1346	0.1202	0.1353	0.125	0.135	0.1207	0.1076	0.1661	0.1743
217	2.7093	2.4857	2.5713	2.6428	2.8123	2.7051	2.5601	2.5937	2.605	2.6632	2.7487	2.4813	2.6928	2.7716	2.7645	2.7517	2.6904	2.6342	2.7825	2.6879	2.7633	2.577	2.6475	2.7081	2.8516	2.7586
218	18	13	14	14	17.355	17.053	13	14	16	17	17.053	12	16.056	16	17	16	16	16	13.067	17	19	14	15	15	18.34	18.05
219	9.8685	6.4533	6.4775	6.4775	8.0222	8.3232	5.6719	6.4775	7.35	8.1633	8.3232	5.6122	7.5556	7.35	9	7.35	8.144	7.35	5.3651	8.1633	9.8336	6.4775	6.554	6.554	8.2031	8.4444
220	8.7075	4.6875	4.8889	4.8889	4.4875	5.8776	4.6875	4.8889	6.37	7.1111	5.8776	5.3254	5.2675	5.2645	7.1111	6.37	6.37	6.37	2.9822	7.1111	8.7108	4.8889	5.04	5.04	4.75	6.12
221	13.952	10.711	11.709	11.709	12.761	13.08	10.711	13.166	12.876	13.871	13.08	9.7128	12.235	13.707	14.705	13.707	15.169	12.421	11.07	13.412	14.942	11.709	12.708	12.708	13.691	14.045
222	7.5252	5.2233	5.3369	5.3369	5.8	6.2827	4.5908	6.0608	5.8222	6.5694	6.2827	4.4465	5.6578	6.2268	7.7099	6.2268	7.692	5.6009	4.4872	6.337	7.6222	5.3369	5.4816	5.4816	6.0314	6.4774
223	6.5598	3.7372	3.9819	3.9819	3.2027	4.3811	3.7372	4.5567	4.9863	5.66	4.3811	4.1487	3.8935	4.4228	6.0448	5.3516	5.9994	4.7868	2.4682	5.4494	6.6758	3.9819	4.1712	4.1712	3.4542	4.6453
224	5.8329	4.3036	4.4636	4.4636	3.5244	4.3252	3.7824	5.6995	4.6854	5.3601	4.3252	3.599	3.8457	5.3342	6.6693	5.3342	7.2924	4.3479	3.3115	4.9995	5.9944	4.4636	4.6439	4.6439	3.7534	4.5488
225	9	7	7	6	7	7	6	6	8	8	6	6	8	6	7	6	6	8	6	8	9	7	7	6	7	7
226	10	6	6	5	6	6	5	5	5	5	5	5	6	5	8	5	5	7	4	7	10	6	6	5	6	6
227	1	1	1	1	1	1	1	1	1	1	1	1	1	1	1	1	1	1	1	1	1	1	1	1	1	1
228	1	1	1	0	1	1	0	0	0	0	0	0	1	0	1	0	0	1	0	1	1	1	1	0	1	1
229	-2.336	-3.019	-2.557	-1.012	-0.083	-0.757	-1.522	-1.412	-2.149	-1.707	0.013	-1.984	-2.291	-0.108	-1.453	-0.108	-0.867	-3.004	-1.134	-2.562	-1.874	-2.557	-2.095	-0.55	0.379	-0.295
230	-0.1835	0.7187	0.3915	-0.2146	-1.6031	-0.9513	-0.1382	-0.7995	0.0507	-0.151	-1.6404	0.189	-0.3975	-1.057	-0.0833	-1.3705	-1.0047	0.0285	-0.2574	-0.1733	-0.5107	0.3915	0.0643	-0.5418	-1.9303	-1.2785
231	6.3275	4.2312	4.6762	5.0085	7.6836	6.7576	4.5221	5.2752	5.1632	5.6432	7.0741	4.0707	6.3043	5.9262	6.2822	5.9161	6.203	5.3534	5.2734	5.8317	6.7693	4.6762	5.1188	5.4471	8.1229	7.1974
232	1	0	0	0	10	6	0	0	0	0	6	0	5	0	0	0	0	0	5	0	1	0	0	0	10	6
233	0	0	0	1	1	1	1	1	1	1	1	1	0	1	0	1	1	0	1	0	0	0	0	1	1	1
234	0	0	0	0	2	1	0	0	0	0	1	0	1	0	0	0	0	0	1	0	0	0	0	0	2	1
235	9	5	5	5	6	6	4	5	6	7	6	4	6	6	8	6	7	6	4	7	9	5	5	5	6	6
236	3	2	2	1	1	1	1	1	1	1	0	1	2	0	2	0	0	2	0	2	3	2	2	0	1	1
237	8.411	5.313	5.313	4.633	5.867	5.909	4.633	5.043	5.539	5.539	5.527	4.633	5.5	4.633	6.486	4.633	5.093	6.003	4.563	6.003	8.411	5.313	5.313	4.633	5.867	5.909
238	-7.411	-5.313	-5.313	-4.633	-5.868	-5.909	-4.633	-5.043	-6.539	-6.539	-5.527	-4.633	-5.5	-4.633	-5.486	-4.633	-5.093	-6.003	-4.563	-6.003	-7.411	-5.313	-5.313	-4.633	-5.868	-5.909
239	4.3472	2.833	2.8664	2.5845	3.0339	3.0247	2.5121	2.6474	2.5715	2.6052	2.8208	2.4757	3.0634	2.6668	3.9091	2.667	2.6771	3.115	2.5167	3.1345	4.3768	2.8626	2.896	2.6141	3.0634	3.0543
240	-3.3472	-2.833	-2.8664	-2.5845	-3.0339	-3.0247	-2.5121	-2.6474	-3.5715	-3.6052	-2.8208	-2.4757	-3.0634	-2.6668	-2.9091	-2.667	-2.6771	-3.115	-2.5167	-3.1345	-3.3768	-2.8626	-2.896	-2.6141	-3.0634	-3.0543
241	0.0795	0.122	0.1206	0.1337	0.1139	0.1143	0.1376	0.1305	0.1344	0.1327	0.1225	0.1396	0.1128	0.1296	0.0884	0.1296	0.1291	0.1109	0.1373	0.1103	0.079	0.1207	0.1193	0.1322	0.1128	0.1132
242	0.1636	0.1933	0.191	0.2119	0.1805	0.1811	0.218	0.2069	0.1539	0.1525	0.1942	0.2214	0.1788	0.2054	0.1883	0.2054	0.2046	0.1758	0.2176	0.1747	0.1622	0.1913	0.1891	0.2095	0.1788	0.1793
243	17.062	0	12.796	29.858	34.185	8.5308	25.592	32.004	44.737	44.566	21.327	12.796	46.774	51.185	38.388	51.185	42.654	21.327	51.581	38.559	29.858	12.796	25.592	42.654	46.981	21.327
244	52.374	54.133	39.821	30.651	51.978	56.171	26.385	26.385	26.385	26.385	47.712	40.697	58.587	26.385	48.108	26.385	26.385	35.087	26.385	26.385	48.108	49.867	35.556	26.385	47.712	51.905
245	19.217	19.217	19.217	19.217	19.217	19.217	9.1703	16.951	9.1703	9.1703	9.1703	9.1703	13.436	9.1703	9.1703	9.1703	9.1703	9.1703	9.1703	9.1703	9.1703	9.1703	9.1703	9.1703	9.1703	9.1703
246	11.765	11.765	11.765	11.765	20.384	11.765	11.765	11.765	11.765	11.765	11.765	11.765	20.384	11.765	11.765	11.765	11.765	29.003	3.146	29.003	11.765	11.765	11.765	11.765	20.384	11.765
247	10.324	20.648	20.648	10.324	10.324	20.648	10.324	10.324	10.324	10.324	10.324	10.324	10.324	10.324	10.324	10.324	10.324	23.274	10.324	23.274	10.324	20.648	20.648	10.324	10.324	20.648
248	56.045	12.95	12.95	12.95	12.95	12.95	12.95	12.95	12.95	12.95	12.95	12.95	12.95	12.95	12.95	12.95	12.95	12.95	12.95	12.95	56.045	12.95	12.95	12.95	12.95	12.95
249	41.539	25.857	25.857	25.857	25.857	25.857	25.857	25.857	25.857	25.857	25.857	25.857	25.857	25.857	51.714	25.857	25.857	25.857	25.857	25.857	41.539	25.857	25.857	25.857	25.857	25.857
250	17.403	0	18.748	0	23.165	35.42	37.495	18.748	18.748	36.15	29.658	18.748	18.748	18.748	44.852	27.449	58.85	18.748	36.15	27.449	36.15	18.748	37.495	18.748	41.912	54.167
251	0	0	0	37.495	36.765	24.51	0	0	0	0	61.275	0	0	46.197	0	37.495	0	0	0	0	0	0	0	37.495	36.765	24.51
252	0	0	0	0	0	0	0	26.363	0	0	0	0	0	0	0	0	19.761	0	0	0	0	0	0	0	0	0
253	0	0	0	0	0	0	0	0	0	0	0	0	5.6826	0	0	0	0	0	0	0	0	0	0	0	0	0
254	27.006	13.567	13.567	13.567	13.567	13.567	13.567	13.567	13.567	13.567	13.567	13.567	13.567	13.567	13.567	13.567	13.567	27.134	13.567	27.134	27.006	13.567	13.567	13.567	13.567	13.567
255	36.144	43.911	43.911	36.144	36.281	43.911	36.144	36.144	63.278	63.278	36.144	36.144	36.281	36.144	37.249	36.144	36.144	42.795	36.007	42.795	36.144	43.911	43.911	36.144	36.281	43.911
256	0.4079	0.4213	0.4667	0.5662	0.6523	0.571	0.5276	0.5722	0.468	0.5041	0.6467	0.4852	0.6014	0.6232	0.5476	0.6232	0.6304	0.4619	0.5616	0.4973	0.4412	0.4667	0.5056	0.5967	0.6722	0.5965
257	0.2788	0.2845	0.3476	0.3828	0.3856	0.4308	0.4168	0.4104	0.4037	0.4448	0.5027	0.3565	0.2829	0.4371	0.344	0.4371	0.4798	0.3614	0.3808	0.3709	0.3244	0.3476	0.4016	0.4324	0.4257	0.4699
258	0.2186	0.2845	0.2621	0.2182	0.1751	0.2109	0.2376	0.2151	0.3245	0.3025	0.1777	0.2589	0.2115	0.1895	0.1827	0.1895	0.1859	0.285	0.2202	0.2663	0.2063	0.2621	0.243	0.2029	0.1651	0.1984
259	0.5921	0.5787	0.5333	0.4338	0.3477	0.429	0.4724	0.4278	0.532	0.4959	0.3533	0.5148	0.3986	0.3768	0.4524	0.3768	0.3696	0.5381	0.4384	0.5027	0.5588	0.5333	0.4944	0.4033	0.3278	0.4035
260	0.7212	0.7155	0.6524	0.6172	0.6144	0.5692	0.5832	0.5896	0.5963	0.5552	0.4973	0.6435	0.7171	0.5629	0.656	0.5629	0.5202	0.6386	0.6192	0.6291	0.6756	0.6524	0.5984	0.5676	0.5743	0.5301
261	0.3735	0.2943	0.2711	0.2156	0.1726	0.2181	0.2348	0.2126	0.2075	0.1934	0.1756	0.2559	0.1871	0.1873	0.2697	0.1873	0.1837	0.253	0.2182	0.2364	0.3525	0.2711	0.2514	0.2005	0.1627	0.2052
262	117.82	85.114	102.35	128.99	185.69	155.61	110.41	132.22	110.8	128.04	180.91	93.176	157.93	163.45	152.28	163.45	168.59	113.33	126.43	130.57	135.05	102.35	119.58	146.22	202.92	172.84
263	80.552	57.478	76.226	87.206	109.78	117.41	87.206	94.821	95.592	112.99	140.64	68.458	74.278	114.65	95.668	114.65	128.32	88.676	85.724	97.378	99.3	76.226	94.974	105.95	128.52	136.16
264	63.15	57.478	57.478	49.711	49.848	57.478	49.711	49.711	76.844	76.844	49.711	49.711	55.53	49.711	50.816	49.711	49.711	69.929	49.574	69.929	63.15	57.478	57.478	49.711	49.848	57.478
265	171.06	116.93	116.93	98.841	98.978	116.93	98.841	98.841	125.98	125.98	98.841	98.841	104.66	98.841	125.8	98.841	98.841	132.01	98.704	132.01	171.06	116.93	116.93	98.841	98.978	116.93
266	208.32	144.57	143.05	140.62	174.89	155.14	122.04	136.24	141.19	141.02	139.11	123.56	188.31	147.64	182.42	147.64	139.11	156.67	139.41	165.2	206.81	143.05	141.54	139.11	173.38	153.62
267	107.91	59.455	59.455	49.131	49.131	59.455	49.131	49.131	49.131	49.131	49.131	49.131	49.131	49.131	74.988	49.131	49.131	62.08	49.131	62.08	107.91	59.455	59.455	49.131	49.131	59.455
268	0.4545	0.4286	0.4286	0.4286	0.4545	0.4545	0.4286	0.4286	0.5	0.4444	0.5	0.4286	0.4444	0.5	0.5	0.5	0.4444	0.5	0.4286	0.4444	0.4545	0.4286	0.4286	0.4286	0.4545	0.4545
269	0.8333	0.75	0.75	0.75	0.8333	0.8333	0.75	0.75	1	0.8	1	0.75	0.8	1	1	1	0.8	1	0.75	0.8	0.8333	0.75	0.75	0.75	0.8333	0.8333
270	8.411	5.313	5.313	4.633	5.867	5.909	4.633	5.043	5.539	5.539	5.527	4.633	5.5	4.633	6.486	4.633	5.093	6.003	4.563	6.003	8.411	5.313	5.313	4.633	5.867	5.909
271	-7.411	-5.313	-5.313	-4.633	-5.868	-5.909	-4.633	-5.043	-6.539	-6.539	-5.527	-4.633	-5.5	-4.633	-5.486	-4.633	-5.093	-6.003	-4.563	-6.003	-7.411	-5.313	-5.313	-4.633	-5.868	-5.909
272	0.1427	0.1705	0.1705	0.1956	0.1544	0.1533	0.1956	0.1797	0.1636	0.1636	0.1639	0.1956	0.1647	0.1956	0.1397	0.1956	0.1779	0.1509	0.1986	0.1509	0.1427	0.1705	0.1705	0.1956	0.1544	0.1533
273	0.1305	0.1694	0.1694	0.1943	0.1534	0.1523	0.1943	0.1785	0.1376	0.1376	0.1628	0.1943	0.1636	0.1943	0.1641	0.1943	0.1767	0.1499	0.1972	0.1499	0.1305	0.1694	0.1694	0.1943	0.1534	0.1523
274	0.2188	0.1422	0.2555	0.3843	0.4182	0.419	0.3777	0.2761	0.2733	0.3226	0.5345	0.3218	0.3859	0.5035	0.3873	0.5035	0.3218	0.2638	0.4019	0.3121	0.2956	0.2555	0.3522	0.4686	0.4847	0.4882
275	0.2186	0.2845	0.2621	0.2182	0.4369	0.3989	0.2376	0.3292	0.3613	0.3368	0.4046	0.2589	0.2747	0.1895	0.1827	0.1895	0.2598	0.285	0.2202	0.2663	0.2063	0.2621	0.243	0.2029	0.412	0.3751
276	0.2186	0.2845	0.2621	0.2182	0.2335	0.2109	0.2376	0.3292	0.3245	0.3025	0.1777	0.2589	0.2747	0.1895	0.1827	0.1895	0.2598	0.285	0.2202	0.2663	0.2063	0.2621	0.243	0.2029	0.2201	0.1984
277	0.7812	0.8578	0.7445	0.6157	0.5818	0.581	0.6223	0.7239	0.7267	0.6774	0.4655	0.6782	0.6141	0.4965	0.6127	0.4965	0.6782	0.7362	0.5981	0.6879	0.7044	0.7445	0.6478	0.5314	0.5153	0.5118
278	0.7814	0.7155	0.7379	0.7818	0.5631	0.6011	0.7624	0.6708	0.6387	0.6632	0.5954	0.7411	0.7253	0.8105	0.8173	0.8105	0.7402	0.715	0.7798	0.7337	0.7937	0.7379	0.757	0.7971	0.588	0.6249
279	0.5626	0.5733	0.4824	0.3975	0.3484	0.3701	0.3848	0.3946	0.4021	0.3749	0.2878	0.4193	0.3394	0.3069	0.4299	0.3069	0.4185	0.4512	0.3779	0.4216	0.4981	0.4824	0.4048	0.3285	0.2952	0.3135
280	63.202	28.738	56.017	87.561	119.04	114.19	79.03	63.798	64.718	81.95	149.53	61.798	101.32	132.07	107.71	132.07	86.045	64.718	90.481	81.95	90.481	56.017	83.295	114.84	146.32	141.47

	V	V	V	V	V	V	V	V	V	V	V	V	V	V	V	V	V	V	V	V	W	W	W	W	W	W	W	W	W
	A	C	D	E	F	G	H	I	K	L	M	N	P	Q	R	S	T	V	W	Y	A	C	D	E	F	G	H	I	K
No.	VA	VC	VD	VE	VF	VG	VH	VI	VK	VL	VM	VN	VP	VQ	VR	VS	VT	VV	VW	VY	WA	WC	WD	WE	WF	WG	WH	WI	WK
206	-0.8474	-0.8467	-0.8505	-0.8543	-0.847	-0.8481	-0.8488	-0.8471	-0.863	-0.8473	-0.8471	-0.8469	-0.8514	-0.8473	-0.8472	-0.8461	-0.846	-0.847	-0.863	-0.847	-0.8628	-0.8628	-0.8628	-0.8628	-0.8628	-0.8628	-0.8628	-0.8628	-0.863
207	-0.3613	-0.3986	-0.4871	-0.4864	-0.3885	-0.4092	-0.3893	-0.323	-0.3455	-0.3392	-0.3267	-0.41	-0.37	-0.4085	-0.3675	-0.3947	-0.3557	-0.3266	-0.3839	-0.3834	-0.3919	-0.4294	-0.485	-0.4816	-0.3893	-0.4619	-0.3954	-0.354	-0.3682
208	0.0949	0.1382	0.111	0.0989	0.0912	0.1237	0.1368	0.0891	0.1816	0.0703	0.074	0.0949	0.1057	0.0855	0.1145	0.1386	0.1308	0.091	0.1149	0.1149	0.1795	0.1834	0.1458	0.1263	0.1461	0.1803	0.1806	0.1258	0.1849
209	2.4528	2.4665	2.4789	2.5244	2.5784	2.3948	2.5454	2.618	2.6231	2.6008	2.5383	2.508	2.6316	2.5494	2.6159	2.4679	2.5222	2.5664	2.6426	2.5863	2.5663	2.5764	2.5855	2.6193	2.6602	2.5245	2.6349	2.6917	2.6943
210	-2.2353	-2.2353	-2.2353	-2.2353	-2.2353	-2.2352	-2.2353	-2.2353	-2.2355	-2.2353	-2.2353	-2.2353	-2.2353	-2.2353	-2.2353	-2.2353	-2.2353	-2.2353	-2.2353	-2.2353	-2.2352	-2.2352	-2.2352	-2.2352	-2.2352	-2.2351	-2.2352	-2.2352	-2.2354
211	-0.3665	-0.427	-0.4973	-0.4489	-0.3401	-0.4263	-0.3607	-0.3024	-0.3109	-0.3037	-0.3236	-0.4069	-0.3426	-0.3704	-0.3278	-0.4485	-0.4092	-0.3092	-0.3355	-0.3639	-0.3922	-0.4209	-0.4402	-0.4292	-0.3773	-0.4181	-0.3843	-0.3358	-0.3358
212	0.0542	0.0555	0.0542	0.0542	0.0848	0.0542	0.0555	0.0542	0.0542	0.0542	0.0555	0.0542	0.0542	0.0542	0.0542	0.0542	0.0542	0.0542	0.1232	0.0581	0.123	0.136	0.1117	0.1088	0.1411	0.1231	0.136	0.123	0.123
213	2.3316	2.3434	2.3495	2.414	2.509	2.2693	2.4316	2.5481	2.5337	2.5345	2.4445	2.37	2.5428	2.4341	2.4976	2.327	2.3877	2.4736	2.6196	2.528	2.5507	2.5566	2.5602	2.5921	2.6413	2.5199	2.6011	2.6678	2.6573
214	-0.6409	-0.6409	-0.6409	-0.6409	-0.6409	-0.6409	-0.6409	-0.641	-0.6419	-0.6409	-0.6409	-0.6409	-0.6411	-0.641	-0.641	-0.6409	-0.641	-0.641	-0.6409	-0.6409	-0.6409	-0.6409	-0.6409	-0.6409	-0.6409	-0.6409	-0.6409	-0.6409	-0.6419
215	-0.1821	-0.1711	-0.191	-0.1904	-0.2018	-0.1854	-0.2026	-0.1752	-0.1802	-0.1788	-0.1705	-0.2011	-0.1763	-0.201	-0.1907	-0.2019	-0.1909	-0.1763	-0.1959	-0.2161	-0.2049	-0.1969	-0.2147	-0.2139	-0.2126	-0.2159	-0.2152	-0.19	-0.2004
216	0.0525	0.1169	0.1051	0.1052	0.1468	0.0558	0.1174	0.0487	0.052	0.052	0.1166	0.1164	0.0056	0.1167	0.1052	0.1165	0.1078	0.049	0.1572	0.1415	0.1664	0.1703	0.1668	0.1668	0.1714	0.1704	0.1706	0.1575	0.1635
217	2.6373	2.6653	2.6752	2.7249	2.7977	2.5706	2.7497	2.8203	2.8123	2.8016	2.7479	2.7	2.8345	2.746	2.8112	2.6517	2.7118	2.7643	2.8877	2.8063	2.8133	2.8292	2.8357	2.8663	2.9122	2.7749	2.8813	2.9299	2.9229
218	13	14	16	17	17.053	12	16.056	16	17	16	16	16	13.067	17	19	14	15	15	18.34	18.05	16.372	17.355	19.326	20.314	20.727	15.39	19.753	19.326	20.314
219	5.6719	6.4775	7.35	8.1633	8.3232	5.6122	7.5556	7.35	9	7.35	8.144	7.35	5.3651	8.1633	9.8336	6.4775	6.554	6.554	8.2031	8.4444	7.3199	8.0222	8.9091	9.6298	9.9723	7.1358	9.2739	8.9091	10.222
220	4.6875	4.8889	6.37	7.1111	5.8776	5.3254	5.2675	5.2645	7.1111	6.37	6.37	6.37	2.9822	7.1111	8.7108	4.8889	5.04	5.04	4.75	6.12	4.25	4.4875	5.5	6.0464	5.75	4.2314	5.2585	4.9887	6.0464
221	10.711	13.166	12.876	13.871	13.08	9.7128	12.235	13.707	14.705	13.707	15.169	12.421	11.07	13.412	14.942	11.709	12.708	12.708	13.691	14.045	11.837	13.833	13.989	14.92	14.662	10.92	13.859	14.626	15.567
222	4.5908	6.0608	5.8222	6.5694	6.2827	4.4465	5.6578	6.2268	7.7099	6.2268	7.692	5.6009	4.4872	6.337	7.6222	5.3369	5.4816	5.4816	6.0314	6.4774	5.1938	6.3164	6.3459	6.9702	6.9483	4.9576	6.4023	6.6505	7.7363
223	3.7372	4.5567	4.9863	5.66	4.3811	4.1487	3.8935	4.4228	6.0448	5.3516	5.9994	4.7868	2.4682	5.4494	6.6758	3.9819	4.1712	4.1712	3.4542	4.6453	2.9743	3.5002	3.87	4.3306	3.9631	2.8915	3.5863	3.6853	4.5349
224	3.7824	5.6995	4.6854	5.3601	4.3252	3.599	3.8457	5.3342	6.6693	5.3342	7.2924	4.3479	3.3115	4.9995	5.9944	4.4636	4.6439	4.6439	3.7534	4.5488	3.0739	4.1607	3.8596	4.3331	3.9183	2.8494	3.5492	4.2293	5.0179
225	5	5	7	7	5	5	7	5	6	5	5	7	5	7	8	6	6	5	6	6	6	6	8	8	6	6	8	6	7
226	4	4	4	4	4	4	5	4	7	4	4	6	3	6	9	5	5	4	5	5	5	5	5	5	5	5	6	5	8
227	1	1	1	1	1	1	1	1	1	1	1	1	1	1	1	1	1	1	1	1	1	1	1	1	1	1	1	1	1
228	0	0	0	0	0	0	0	0	1	0	0	1	0	1	1	0	0	0	0	0	0	0	0	0	0	0	1	0	1
229	0.023	0.133	-0.604	-0.162	1.558	-0.439	-0.746	1.437	0.092	1.437	0.678	-1.459	0.411	-1.017	-0.329	-1.012	-0.55	0.995	1.924	1.25	0.952	1.062	0.325	0.767	2.487	0.49	0.183	2.366	1.021
230	-0.7443	-1.4056	-0.5553	-0.7571	-2.2465	-0.4171	-1.0036	-1.6631	-0.6894	-1.9765	-1.6108	-0.5776	-0.8635	-0.7794	-1.1168	-0.2146	-0.5418	-1.1479	-2.5364	-1.8845	-2.1328	-2.7941	-1.9439	-2.1456	-3.635	-1.8056	-2.3921	-3.0516	-2.0779
231	4.8471	5.6018	5.5018	5.9776	7.389	4.4003	6.6261	6.241	6.5965	6.231	6.5221	5.6844	5.5935	6.1589	7.0857	5.0085	5.4471	5.7649	8.4368	7.5192	7.5168	8.2769	8.1715	8.6485	10.042	7.0689	9.2815	8.9141	9.2725
232	0	0	0	0	6	0	5	0	0	0	0	0	5	0	1	0	0	0	10	6	10	10	10	10	16	10	15	10	10
233	1	1	1	1	1	1	1	1	1	1	1	0	1	1	0	1	1	1	1	1	1	1	1	1	1	1	1	1	1
234	0	0	0	0	1	0	1	0	0	0	0	0	1	0	0	0	0	0	2	1	2	2	2	2	3	2	3	2	2
235	4	5	6	7	6	4	6	6	8	6	7	6	4	7	9	5	5	5	6	6	5	6	7	8	7	5	7	7	9
236	0	0	1	1	0	0	0	0	1	0	0	2	0	1	2	1	0	0	0	0	0	0	0	0	0	0	1	0	1
237	3.953	4.363	4.859	4.859	4.847	3.953	4.82	3.953	5.806	3.953	4.413	5.323	3.883	5.323	7.731	4.633	4.633	3.953	5.187	5.229	5.187	5.597	6.093	6.093	6.081	5.187	6.054	5.187	7.04
238	-3.953	-4.363	-5.859	-5.859	-4.847	-3.953	-4.82	-3.953	-4.806	-3.953	-4.413	-5.323	-3.883	-5.323	-6.731	-4.633	-4.633	-3.953	-5.188	-5.229	-5.188	-5.598	-7.094	-7.094	-6.082	-5.188	-6.055	-5.188	-6.041
239	2.2092	2.3445	2.2686	2.3023	2.5179	2.1728	2.7605	2.3639	3.6062	2.3641	2.3742	2.8121	2.2138	2.8316	4.0739	2.5597	2.5931	2.3112	2.7605	2.7514	2.6832	2.8185	2.7426	2.7763	2.9919	2.6468	3.2345	2.8379	4.0802
240	-2.2092	-2.3445	-3.2686	-3.3023	-2.5179	-2.1728	-2.7605	-2.3639	-2.6062	-2.3641	-2.3742	-2.8121	-2.2138	-2.8316	-3.0739	-2.5597	-2.5931	-2.3112	-2.7605	-2.7514	-2.6832	-2.8185	-3.7426	-3.7763	-2.9919	-2.6468	-3.2345	-2.8379	-3.0802
241	0.1564	0.1474	0.1523	0.1501	0.1372	0.159	0.1252	0.1462	0.0958	0.1462	0.1455	0.1229	0.1561	0.122	0.0848	0.135	0.1333	0.1495	0.1252	0.1256	0.1288	0.1226	0.126	0.1245	0.1155	0.1306	0.1068	0.1218	0.0847
242	0.2479	0.2336	0.1682	0.1665	0.2175	0.2522	0.1984	0.2317	0.2102	0.2317	0.2307	0.1948	0.2474	0.1934	0.1782	0.2139	0.2112	0.237	0.1984	0.1991	0.2041	0.1943	0.1469	0.1456	0.1831	0.207	0.1693	0.193	0.1778
243	42.654	49.066	61.798	61.628	38.388	29.858	63.836	68.246	55.45	68.246	59.715	38.388	68.642	55.621	46.919	29.858	42.654	59.715	64.042	38.388	55.682	62.094	74.827	74.656	51.417	42.886	76.864	81.275	68.478
244	22.12	22.12	22.12	22.12	43.447	36.432	54.322	22.12	43.843	22.12	22.12	30.821	22.12	22.12	43.843	45.602	31.29	22.12	43.447	47.64	43.447	43.447	43.447	43.447	64.774	57.759	75.649	43.447	65.17
245	4.2654	12.046	4.2654	4.2654	4.2654	4.2654	8.5308	4.2654	4.2654	4.2654	4.2654	4.2654	4.2654	4.2654	4.2654	4.2654	4.2654	4.2654	4.2654	4.2654	4.2654	12.046	4.2654	4.2654	4.2654	4.2654	8.5308	4.2654	4.2654
246	11.765	11.765	11.765	11.765	11.765	11.765	20.384	11.765	11.765	11.765	11.765	29.003	3.146	29.003	11.765	11.765	11.765	11.765	20.384	11.765	20.384	20.384	20.384	20.384	20.384	20.384	29.003	20.384	20.384
247	0	0	0	0	0	0	0	0	0	0	0	12.95	0	12.95	0	10.324	10.324	0	0	10.324	0	0	0	0	0	0	0	0	0
248	12.95	12.95	12.95	12.95	12.95	12.95	12.95	12.95	12.95	12.95	12.95	12.95	12.95	12.95	56.045	12.95	12.95	12.95	12.95	12.95	12.95	12.95	12.95	12.95	12.95	12.95	12.95	12.95	12.95
249	25.857	25.857	25.857	25.857	25.857	25.857	25.857	25.857	51.714	25.857	25.857	25.857	25.857	25.857	41.539	25.857	25.857	25.857	25.857	25.857	25.857	25.857	25.857	25.857	25.857	25.857	25.857	25.857	51.714
250	18.748	0	0	17.403	10.91	0	0	0	26.104	8.7013	40.102	0	17.403	8.7013	17.403	0	18.748	0	23.165	35.42	33.211	14.463	14.463	31.866	25.373	14.463	14.463	14.463	40.567
251	37.495	37.495	37.495	37.495	98.77	37.495	37.495	83.692	37.495	74.991	37.495	37.495	37.495	37.495	37.495	37.495	37.495	74.991	74.26	62.005	36.765	36.765	36.765	36.765	98.039	36.765	36.765	82.961	36.765
252	0	26.363	0	0	0	0	0	0	0	0	19.761	0	0	0	0	0	0	0	0	0	0	26.363	0	0	0	0	0	0	0
253	0	0	0	0	0	0	5.6826	0	0	0	0	0	0	0	0	0	0	0	0	0	0	0	0	0	0	0	5.6826	0	0
254	13.567	13.567	13.567	13.567	13.567	13.567	13.567	13.567	13.567	13.567	13.567	27.134	13.567	27.134	27.006	13.567	13.567	13.567	13.567	13.567	13.567	13.567	13.567	13.567	13.567	13.567	13.567	13.567	13.567
255	28.376	28.376	55.51	55.51	28.376	28.376	28.513	28.376	29.482	28.376	28.376	35.027	28.239	35.027	28.376	36.144	36.144	28.376	28.513	36.144	28.513	28.513	55.647	55.647	28.513	28.513	28.65	28.513	29.618
256	0.6292	0.663	0.5602	0.5891	0.7199	0.5974	0.6807	0.7019	0.6242	0.7019	0.7074	0.5513	0.655	0.5798	0.5139	0.5662	0.5967	0.6816	0.7395	0.6687	0.7055	0.7271	0.6425	0.6618	0.7656	0.6858	0.7356	0.7532	0.686
257	0.4508	0.4416	0.4344	0.4722	0.5259	0.3961	0.3144	0.4639	0.3721	0.4639	0.5048	0.3925	0.4138	0.3997	0.3505	0.3828	0.4324	0.4611	0.4494	0.4932	0.408	0.4037	0.3986	0.4316	0.4795	0.3625	0.3022	0.4257	0.3509
258	0.1926	0.1751	0.2816	0.2631	0.1455	0.2091	0.1762	0.1549	0.1502	0.1549	0.152	0.2448	0.1789	0.2293	0.176	0.2182	0.2029	0.1654	0.1355	0.1666	0.1532	0.1419	0.2291	0.2167	0.1219	0.1635	0.146	0.1284	0.1257
259	0.3708	0.337	0.4398	0.4109	0.2801	0.4026	0.3193	0.2981	0.3758	0.2981	0.2926	0.4487	0.345	0.4202	0.4861	0.4338	0.4033	0.3184	0.2605	0.3313	0.2945	0.2729	0.3575	0.3382	0.2344	0.3142	0.2644	0.2468	0.314
260	0.5492	0.5584	0.5656	0.5278	0.4741	0.6039	0.6856	0.5361	0.6279	0.5361	0.4952	0.6075	0.5862	0.6003	0.6495	0.6172	0.5676	0.5389	0.5506	0.5068	0.592	0.5963	0.6014	0.5684	0.5205	0.6375	0.6978	0.5743	0.6491
261	0.1782	0.162	0.1582	0.1478	0.1346	0.1935	0.1431	0.1433	0.2256	0.1433	0.1406	0.2039	0.1661	0.1909	0.3101	0.2156	0.2005	0.153	0.125	0.1647	0.1413	0.1309	0.1284	0.1215	0.1124	0.1508	0.1183	0.1184	0.1883
262	137.05	158.86	137.44	154.68	207.55	119.82	184.57	190.09	178.92	190.09	195.22	139.97	153.07	157.21	161.69	128.99	146.22	172.86	229.56	199.48	193.75	215.56	194.15	211.38	264.25	176.52	241.27	246.8	235.63
263	98.186	105.8	106.57	123.97	151.62	79.438	85.258	125.64	106.65	125.64	139.3	99.656	96.704	108.36	110.28	87.206	105.95	116.93	139.5	147.14	112.06	119.67	120.44	137.84	165.49	93.308	99.128	139.5	120.52
264	41.943	41.943	69.077	69.077	41.943	41.943	47.763	41.943	43.049	41.943	41.943	62.161	41.806	62.161	55.382	49.711	49.711	41.943	42.08	49.711	42.08	42.08	69.214	69.214	42.08	42.08	47.899	42.08	43.185
265	80.75	80.75	107.88	107.88	80.75	80.75	86.569	80.75	107.71	80.75	80.75	113.92	80.613	113.92	152.97	98.841	98.841	80.75	80.887	98.841	80.887	80.887	108.02	108.02	80.887	80.887	86.706	80.887	107.85
266	119.61	133.8	138.76	138.58	136.67	121.13	185.88	145.2	179.99	145.2	136.67	154.23	136.98	162.76	204.38	140.62	139.11	136.67	170.95	151.19	162.58	176.78	181.73	181.56	179.65	164.1	228.85	188.18	222.96
267	38.807	38.807	38.807	38.807	38.807	38.807	38.807	38.807	64.664	38.807	38.807	51.756	38.807	51.756	97.583	49.131	49.131	38.807	38.807	49.131	38.807	38.807	38.807	38.807	38.807	38.807	38.807	38.807	64.664
268	0.4286	0.4286	0.5	0.4444	0.5	0.4286	0.4444	0.5	0.5	0.5	0.4444	0.5	0.4286	0.4444	0.4545	0.4286	0.4286	0.4286	0.4545	0.4545	0.4545	0.4545	0.5	0.4615	0.5	0.4545	0.4615	0.5	0.5
269	0.75	0.75	1	0.8	1	0.75	0.8	1	1	1	0.8	1	0.75	0.8	0.8333	0.75	0.75	0.75	0.8333	0.8333	0.8333	0.8333	1	0.8571	1	0.8333	0.8571	1	1
270	3.953	4.363	4.859	4.859	4.847	3.953	4.82	3.953	5.806	3.953	4.413	5.323	3.883	5.323	7.731	4.633	4.633	3.953	5.187	5.229	5.187	5.597	6.093	6.093	6.081	5.187	6.054	5.187	7.04
271	-3.953	-4.363	-5.859	-5.859	-4.847	-3.953	-4.82	-3.953	-4.806	-3.953	-4.413	-5.323	-3.883	-5.323	-6.731	-4.633	-4.633	-3.953	-5.188	-5.229	-5.188	-5.598	-7.094	-7.094	-6.082	-5.188	-6.055	-5.188	-6.041
272	0.2292	0.2077	0.1865	0.1865	0.1869	0.2292	0.188	0.2292	0.156	0.2292	0.2053	0.1702	0.2333	0.1702	0.1552	0.1956	0.1956	0.2292	0.1747	0.1733	0.1747	0.1619	0.1487	0.1487	0.149	0.1747	0.1497	0.1747	0.1287
273	0.2277	0.2063	0.1536	0.1536	0.1857	0.2277	0.1867	0.2277	0.1873	0.2277	0.2039	0.1691	0.2318	0.1691	0.1437	0.1943	0.1943	0.2277	0.1735	0.1721	0.1735	0.1608	0.1269	0.1269	0.148	0.1735	0.1486	0.1735	0.149
274	0.5077	0.3979	0.3924	0.4323	0.6281	0.4654	0.49	0.6041	0.4858	0.6041	0.4261	0.3791	0.5222	0.4186	0.3878	0.3843	0.4686	0.5772	0.5729	0.5799	0.5172	0.4278	0.4227	0.4539	0.6158	0.4849	0.5011	0.5954	0.4971
275	0.1926	0.2851	0.317	0.2962	0.3657	0.2091	0.2374	0.1549	0.1502	0.1549	0.2236	0.2448	0.1789	0.2293	0.176	0.2182	0.2029	0.1654	0.3756	0.3384	0.4246	0.4823	0.5045	0.4773	0.5218	0.453	0.4239	0.3559	0.3427
276	0.1926	0.2851	0.2816	0.2631	0.1455	0.2091	0.2374	0.1549	0.1502	0.1549	0.2236	0.2448	0.1789	0.2293	0.176	0.2182	0.2029	0.1654	0.189	0.1666	0.2137	0.2869	0.284	0.2687	0.17	0.228	0.2473	0.1791	0.1741
277	0.4923	0.6021	0.6076	0.5677	0.3719	0.5346	0.51	0.3959	0.5142	0.3959	0.5739	0.6209	0.4778	0.5814	0.6122	0.6157	0.5314	0.4228	0.4271	0.4201	0.4828	0.5722	0.5773	0.5461	0.3842	0.5151	0.4989	0.4046	0.5029
278	0.8074	0.7149	0.683	0.7038	0.6343	0.7909	0.7626	0.8451	0.8498	0.8451	0.7764	0.7552	0.8211	0.7707	0.824	0.7818	0.7971	0.8346	0.6244	0.6616	0.5754	0.5177	0.4955	0.5227	0.4782	0.547	0.5761	0.6441	0.6573
279	0.2997	0.317	0.326	0.3046	0.2264	0.3255	0.2726	0.241	0.364	0.241	0.3503	0.376	0.2989	0.3521	0.4362	0.3975	0.3285	0.2574	0.238	0.2534	0.2691	0.2853	0.2932	0.2774	0.2141	0.2871	0.2516	0.2255	0.3288
280	110.57	95.342	96.262	113.49	181.07	93.342	132.87	163.61	139.26	163.61	117.59	96.262	122.02	113.49	122.02	87.561	114.84	146.38	177.86	173.01	142.05	126.82	127.74	144.97	212.55	124.82	164.35	195.09	170.74

Table S12 Amino acid descriptors (2D) used in this study.

	S	S	S	S	S	S	T	T	T	T	T	T	T	T	T	T	T	T	T	T	T	T	T	T	T	T	V	V	V	V	V	V	V	V	V	V	V	V	V	V	V	V	V	V	V	V	W	W	W	W	W	W	W	W	W
	R	S	T	V	W	Y	A	C	D	E	F	G	H	I	K	L	M	N	P	Q	R	S	T	V	W	Y	A	C	D	E	F	G	H	I	K	L	M	N	P	Q	R	S	T	V	W	Y	A	C	D	E	F	G	H	I	K
No.	SR	SS	ST	SV	SW	SY	TA	TC	TD	TE	TF	TG	TH	TI	TK	TL	TM	TN	TP	TQ	TR	TS	TT	TV	TW	TY	VA	VC	VD	VE	VF	VG	VH	VI	VK	VL	VM	VN	VP	VQ	VR	VS	VT	VV	VW	VY	WA	WC	WD	WE	WF	WG	WH	WI	WK
281	63.15	57.478	57.478	49.711	124.38	108.71	49.711	76.074	85.546	85.546	113.19	49.711	72.139	49.711	50.816	49.711	69.471	69.929	49.574	69.929	63.15	57.478	57.478	49.711	124.38	108.71	41.943	68.306	77.778	77.778	105.43	41.943	64.372	41.943	43.049	41.943	61.704	62.161	41.806	62.161	55.382	49.711	49.711	41.943	116.62	100.94	116.62	142.98	152.45	152.45	180.1	116.62	139.05	116.62	117.72
282	63.15	57.478	57.478	49.711	66.457	57.478	49.711	76.074	76.844	76.844	49.711	49.711	72.139	49.711	50.816	49.711	69.471	69.929	49.574	69.929	63.15	57.478	57.478	49.711	66.457	57.478	41.943	68.306	69.077	69.077	41.943	41.943	64.372	41.943	43.049	41.943	61.704	62.161	41.806	62.161	55.382	49.711	49.711	41.943	58.689	49.711	58.689	85.052	85.823	85.823	58.689	58.689	81.117	58.689	59.794
283	225.67	173.31	163.26	140.27	165.63	158.36	130.22	167.26	172.06	172.06	130.22	130.22	161.27	130.22	170.37	130.22	181.38	180.63	134.66	180.63	215.63	163.26	153.22	130.22	155.58	148.31	107.22	144.26	149.07	149.07	107.22	107.22	138.27	107.22	147.38	107.22	158.38	157.63	111.66	157.63	192.63	140.27	130.22	107.22	132.59	125.31	132.59	169.63	174.43	174.43	132.59	132.59	163.64	132.59	172.74
284	225.73	144.57	161.8	178.12	160.29	163.84	159.54	154.98	151.23	168.47	166.55	142.31	190.45	212.58	227.27	212.58	197.96	175.41	175.56	192.65	242.96	161.8	179.03	195.35	177.52	181.07	175.85	171.3	167.55	184.78	182.87	158.62	206.77	228.89	243.59	228.89	214.27	191.73	191.88	208.96	259.27	178.12	195.35	211.66	193.83	197.39	158.02	153.47	149.72	166.95	165.04	140.79	188.94	211.07	225.76
285	162.52	115.83	105.78	90.556	99.175	100.88	80.509	91.187	95.218	95.218	80.509	80.509	89.128	80.509	119.56	80.509	111.91	110.7	85.082	110.7	152.48	105.78	95.738	80.509	89.128	90.833	65.28	75.957	79.989	79.989	65.28	65.28	73.899	65.28	104.33	65.28	96.681	95.468	69.853	95.468	137.25	90.556	80.509	65.28	73.899	75.604	73.899	84.576	88.608	88.608	73.899	73.899	82.518	73.899	112.95
286	6	4	4	4	6	6	4	4	4	5	5	4	5	4	5	4	5	4	4	5	6	4	4	4	6	6	4	4	4	5	5	4	5	4	5	4	5	4	4	5	6	4	4	4	6	6	6	6	6	7	7	6	7	6	7
287	0	0	0	0	0	0	0	1	0	0	0	0	0	0	0	0	0	0	0	0	0	0	0	0	0	0	0	1	0	0	0	0	0	0	0	0	0	0	0	0	0	0	0	0	0	0	0	1	0	0	0	0	0	0	0
288	0	0	0	0	2	1	0	0	0	0	1	0	1	0	0	0	0	0	1	0	0	0	0	0	2	1	0	0	0	0	1	0	1	0	0	0	0	0	1	0	0	0	0	0	2	1	2	2	2	2	3	2	3	2	2
289	0.1427	0.1705	0.1705	0.1956	0.1544	0.1533	0.1956	0.1797	0.1636	0.1636	0.1639	0.1956	0.1647	0.1956	0.1397	0.1956	0.1779	0.1509	0.1986	0.1509	0.1427	0.1705	0.1705	0.1956	0.1544	0.1533	0.2292	0.2077	0.1865	0.1865	0.1869	0.2292	0.188	0.2292	0.156	0.2292	0.2053	0.1702	0.2333	0.1702	0.1552	0.1956	0.1956	0.2292	0.1747	0.1733	0.1747	0.1619	0.1487	0.1487	0.149	0.1747	0.1497	0.1747	0.1287
290	0.1305	0.1694	0.1694	0.1943	0.1534	0.1523	0.1943	0.1785	0.1376	0.1376	0.1628	0.1943	0.1636	0.1943	0.1641	0.1943	0.1767	0.1499	0.1972	0.1499	0.1305	0.1694	0.1694	0.1943	0.1534	0.1523	0.2277	0.2063	0.1536	0.1536	0.1857	0.2277	0.1867	0.2277	0.1873	0.2277	0.2039	0.1691	0.2318	0.1691	0.1437	0.1943	0.1943	0.2277	0.1735	0.1721	0.1735	0.1608	0.1269	0.1269	0.148	0.1735	0.1486	0.1735	0.149
291	-6.7325	-5.1838	-4.7953	-3.5201	-2.4521	-3.2278	-3.7677	-3.8578	-5.6476	-5.2575	-2.5449	-4.1562	-3.8218	-2.7415	-4.3754	-2.7415	-3.0345	-4.9122	-3.2814	-4.5221	-6.344	-4.7953	-4.4068	-3.1316	-2.0636	-2.8393	-2.4925	-2.5826	-4.3724	-3.9823	-1.2697	-2.881	-2.5466	-1.4663	-3.1002	-1.4663	-1.7593	-3.637	-2.0062	-3.2469	-5.0688	-3.5201	-3.1316	-1.8564	-0.7884	-1.5641	-1.4245	-1.5146	-3.3044	-2.9143	-0.2018	-1.813	-1.4787	-0.3983	-2.0322
292	201.56	144.36	144.36	118.98	118.98	144.36	118.98	118.98	161.66	161.66	118.98	118.98	118.98	118.98	151.87	118.98	118.98	151.87	100.97	151.87	201.56	144.36	144.36	118.98	118.98	144.36	93.591	93.591	136.27	136.27	93.591	93.591	93.591	93.591	126.49	93.591	93.591	126.49	75.581	126.49	176.17	118.98	118.98	93.591	93.591	118.98	93.591	93.591	136.27	136.27	93.591	93.591	93.591	93.591	126.49
293	8.6219	6.7228	6.7228	6.7228	6.7228	6.7228	6.7228	6.7228	8.1868	8.1868	6.7228	6.7228	23.508	6.7228	6.7228	6.7228	6.7228	11.982	9.8471	11.982	8.6219	6.7228	6.7228	6.7228	6.7228	6.7228	6.7228	6.7228	8.1868	8.1868	6.7228	6.7228	23.508	6.7228	6.7228	6.7228	6.7228	11.982	9.8471	11.982	8.6219	6.7228	6.7228	6.7228	6.7228	6.7228	6.7228	6.7228	8.1868	8.1868	6.7228	6.7228	23.508	6.7228	6.7228
294	55.688	55.688	62.157	55.688	72.474	55.688	62.157	62.157	86.019	86.019	62.157	58.175	78.943	62.157	62.157	62.157	62.157	86.019	62.157	86.019	62.157	62.157	68.626	62.157	78.943	62.157	55.688	55.688	79.551	79.551	55.688	51.706	72.474	55.688	55.688	55.688	55.688	79.551	55.688	79.551	55.688	55.688	62.157	55.688	72.474	55.688	72.474	72.474	96.336	96.336	72.474	68.492	89.259	72.474	72.474
295	39.366	41.853	20.926	20.926	20.926	20.926	0	17.234	0	0	0	18.44	0	0	18.44	0	17.234	0	18.44	0	18.44	20.926	0	0	0	0	0	17.234	0	0	0	18.44	0	0	18.44	0	17.234	0	18.44	0	18.44	20.926	0	0	0	0	0	17.234	0	0	0	18.44	0	0	18.44
296	0	0	0	4.4108	3.1856	3.1856	0	0	0	0	3.1856	0	2.7567	4.4108	0	4.4108	0	0	0	0	0	0	0	4.4108	3.1856	3.1856	4.4108	4.4108	4.4108	4.4108	7.5964	4.4108	7.1675	8.8216	4.4108	8.8216	4.4108	4.4108	4.4108	4.4108	4.4108	4.4108	4.4108	8.8216	7.5964	7.5964	3.1856	3.1856	3.1856	3.1856	6.3712	3.1856	5.9423	7.5964	3.1856
297	0	0	0	0	18.868	18.868	0	0	0	0	18.868	0	18.868	0	0	0	31.691	0	0	0	0	0	0	0	18.868	18.868	0	0	0	0	18.868	0	18.868	0	0	0	31.691	0	0	0	0	0	0	0	18.868	18.868	18.868	18.868	18.868	18.868	37.737	18.868	37.737	18.868	18.868
298	0	0	0	0	92.504	70.573	0	0	0	0	88.216	0	34	0	0	0	0	0	0	0	0	0	0	0	92.504	70.573	0	0	0	0	88.216	0	34	0	0	0	0	0	0	0	0	0	0	0	92.504	70.573	92.504	92.504	92.504	92.504	180.72	92.504	126.5	92.504	92.504
299	37.737	0	0	0	0	0	0	46.107	18.868	37.737	0	0	0	18.868	56.605	18.868	18.868	18.868	37.737	37.737	37.737	0	0	0	0	0	0	46.107	18.868	37.737	0	0	0	18.868	56.605	18.868	18.868	18.868	37.737	37.737	37.737	0	0	0	0	0	0	46.107	18.868	37.737	0	0	0	18.868	56.605
300	0	0	33.326	66.652	0	5.2434	66.652	33.326	33.326	33.326	33.326	33.326	33.326	99.978	33.326	99.978	65.546	33.326	33.326	33.326	33.326	33.326	66.652	99.978	33.326	38.569	99.978	66.652	66.652	66.652	66.652	66.652	66.652	133.3	66.652	133.3	98.872	66.652	66.652	66.652	66.652	66.652	99.978	133.3	66.652	71.895	33.326	0	0	0	0	0	0	66.652	0
301	5.9056	3.7785	4.238	4.5538	7.2702	6.251	4.0969	4.9149	4.4919	4.9536	6.5441	3.6374	5.7586	5.475	5.6901	5.475	5.8295	4.9106	4.8099	5.3723	6.3651	4.238	4.6975	5.0133	7.7297	6.7105	4.4126	5.2306	4.8076	5.2693	6.8598	3.9531	6.0743	5.7907	6.0058	5.7907	6.1452	5.2263	5.1256	5.688	6.6808	4.5538	5.0133	5.329	8.0455	7.0263	7.129	7.9471	7.5241	7.9858	9.5763	6.6695	8.7908	8.5071	8.7223
302	90.408	90.408	90.408	90.408	90.408	90.408	90.408	90.408	156.95	156.95	90.408	90.408	90.408	90.408	90.408	90.408	90.408	114.27	90.408	114.27	90.408	90.408	90.408	90.408	90.408	90.408	90.408	90.408	156.95	156.95	90.408	90.408	90.408	90.408	90.408	90.408	90.408	114.27	90.408	114.27	90.408	90.408	90.408	90.408	90.408	90.408	90.408	90.408	156.95	156.95	90.408	90.408	90.408	90.408	90.408
303	57.057	50.77	50.77	25.385	25.385	50.77	25.385	25.385	25.385	25.385	25.385	25.385	42.171	25.385	25.385	25.385	25.385	25.385	28.51	25.385	57.057	50.77	50.77	25.385	25.385	50.77	0	0	0	0	0	0	16.786	0	0	0	0	0	3.1243	0	31.672	25.385	25.385	0	0	25.385	0	0	0	0	0	0	16.786	0	0
304	68.919	50.908	50.908	55.319	67.693	50.908	50.908	50.908	50.908	50.908	50.908	50.908	67.693	55.319	83.805	55.319	50.908	50.908	32.897	50.908	68.919	50.908	50.908	55.319	67.693	50.908	55.319	55.319	55.319	55.319	55.319	55.319	72.104	59.73	88.216	59.73	55.319	55.319	37.308	55.319	73.329	55.319	55.319	59.73	72.104	55.319	67.693	67.693	67.693	67.693	67.693	67.693	84.479	72.104	100.59
305	7.9639	7.9639	14.433	7.9639	11.15	16.393	14.433	14.433	14.433	14.433	17.618	10.451	17.189	14.433	14.433	14.433	14.433	14.433	14.433	14.433	14.433	14.433	20.901	14.433	17.618	22.862	7.9639	7.9639	7.9639	7.9639	11.15	3.982	10.721	7.9639	7.9639	7.9639	7.9639	7.9639	7.9639	7.9639	7.9639	7.9639	14.433	7.9639	11.15	16.393	11.15	11.15	11.15	11.15	14.335	7.1675	13.906	11.15	11.15
306	32.897	0	0	0	4.7171	0	0	0	0	0	0	0	0	0	0	0	0	32.897	0	32.897	32.897	0	0	0	4.7171	0	0	0	0	0	0	0	0	0	0	0	0	32.897	0	32.897	32.897	0	0	0	4.7171	0	4.7171	4.7171	4.7171	4.7171	4.7171	4.7171	4.7171	4.7171	4.7171
307	37.737	0	0	0	106.66	89.441	0	0	18.868	37.737	107.08	0	52.868	18.868	56.605	18.868	18.868	18.868	37.737	37.737	37.737	0	0	0	106.66	89.441	0	0	18.868	37.737	107.08	0	52.868	18.868	56.605	18.868	18.868	18.868	37.737	37.737	37.737	0	0	0	106.66	89.441	106.66	106.66	125.52	144.39	213.74	106.66	159.52	125.52	163.26
308	47.988	48.575	27.649	27.649	27.649	27.649	6.7228	23.956	8.1868	8.1868	6.7228	25.162	6.7228	6.7228	25.162	6.7228	23.956	11.982	25.162	11.982	27.061	27.649	6.7228	6.7228	6.7228	6.7228	6.7228	23.956	8.1868	8.1868	6.7228	25.162	6.7228	6.7228	25.162	6.7228	23.956	11.982	25.162	11.982	27.061	27.649	6.7228	6.7228	6.7228	6.7228	6.7228	23.956	8.1868	8.1868	6.7228	25.162	6.7228	6.7228	25.162
309	0	0	33.326	66.652	0	0	66.652	79.433	33.326	33.326	33.326	33.326	33.326	99.978	33.326	99.978	97.237	33.326	33.326	33.326	33.326	33.326	66.652	99.978	33.326	33.326	99.978	112.76	66.652	66.652	66.652	66.652	66.652	133.3	66.652	133.3	130.56	66.652	66.652	66.652	66.652	66.652	99.978	133.3	66.652	66.652	33.326	46.107	0	0	0	0	0	66.652	0
310	180.74	137.33	137.33	117.1	132.89	137.33	117.1	155.9	157.23	157.23	117.1	117.1	145.78	117.1	144.74	117.1	117.1	160.19	108.31	160.19	180.74	137.33	137.33	117.1	132.89	137.33	96.87	135.67	137	137	96.87	96.87	125.55	96.87	124.51	96.87	96.87	139.96	88.08	139.96	160.51	117.1	117.1	96.87	112.66	117.1	112.66	151.46	152.79	152.79	112.66	112.66	141.34	112.66	140.3
311	0.4845	0.5895	0.5647	0.5647	0.4683	0.4855	0.5895	0.5647	0.5212	0.5021	0.4855	0.6167	0.5033	0.5212	0.5021	0.5212	0.5212	0.5212	0.5665	0.5021	0.4681	0.5647	0.542	0.542	0.4531	0.469	0.5895	0.5647	0.5212	0.5021	0.4855	0.6167	0.5033	0.5212	0.5021	0.5212	0.5212	0.5212	0.5665	0.5021	0.4681	0.5647	0.542	0.542	0.4531	0.469	0.4846	0.4683	0.4389	0.4257	0.4121	0.5023	0.4243	0.4389	0.4257
312	5.0875	4.585	4.7004	4.7004	5.4594	5.2479	4.585	4.7004	4.9069	5	5.2479	4.4594	5.1699	4.9069	5	4.9069	4.9069	4.9069	4.9069	5	5.1699	4.7004	4.8074	4.8074	5.5236	5.3219	4.585	4.7004	4.9069	5	5.2479	4.4594	5.1699	4.9069	5	4.9069	4.9069	4.9069	4.9069	5	5.1699	4.7004	4.8074	4.8074	5.5236	5.3219	5.3923	5.4594	5.585	5.6439	5.8074	5.3219	5.7549	5.585	5.6439
313	3.2738	2.6888	2.7059	2.7059	3.207	3.1854	2.7052	2.7385	2.8921	3.0324	3.1291	2.7082	3.0398	2.8436	3.1055	2.8921	2.9726	2.8921	2.6758	3.0324	3.3094	2.7385	2.7539	2.7539	3.2557	3.2323	2.7052	2.7385	2.8921	3.0324	3.1291	2.7082	3.0398	2.8436	3.1055	2.8921	2.9726	2.8921	2.6758	3.0324	3.3094	2.7385	2.7539	2.7539	3.2557	3.2323	3.2643	3.2996	3.4038	3.4967	3.5701	3.2485	3.5091	3.3752	3.5367
314	8.0269	7.0973	7.3248	7.3248	8.4983	8.1993	7.0896	7.3171	7.7137	7.8829	8.2078	6.8379	8.0523	7.7197	7.8747	7.7137	7.7055	7.7137	7.5338	7.8829	8.1865	7.3171	7.5278	7.5278	8.6337	8.3497	7.0896	7.3171	7.7137	7.8829	8.2078	6.8379	8.0523	7.7197	7.8747	7.7137	7.7055	7.7137	7.5338	7.8829	8.1865	7.3171	7.5278	7.5278	8.6337	8.3497	8.3303	8.477	8.7455	8.8662	9.0991	8.1743	8.9859	8.7479	8.8624
315	288.88	202.05	219.28	227.83	284.67	272.55	209.25	231.06	236.78	254.01	279.75	192.02	262.59	262.29	278.09	262.29	267.43	245.34	225.14	262.58	306.11	219.28	236.51	245.06	301.9	289.78	217.8	239.61	245.33	262.56	288.29	200.56	271.14	270.84	286.63	270.84	275.97	253.89	233.68	271.12	314.66	227.83	245.06	253.61	310.45	298.32	274.64	296.45	302.17	319.4	345.14	257.41	327.98	327.68	343.48
316	326.29	218.85	243.27	259.18	370.65	333.82	234.75	256.07	264.49	288.92	349.73	210.32	313.98	308.04	326.6	308.04	305.99	275.03	271.33	299.46	350.72	243.27	267.7	283.61	395.07	358.25	250.66	271.98	280.4	304.83	365.63	226.23	329.89	323.95	342.5	323.95	321.9	290.94	287.24	315.37	366.63	259.18	283.61	299.52	410.98	374.16	362.12	383.44	391.86	416.29	477.1	337.69	441.35	435.41	453.97
317	40.701	40.701	40.701	40.701	40.701	40.701	40.701	40.701	67.835	67.835	40.701	40.701	40.701	40.701	40.701	40.701	40.701	54.268	40.701	54.268	40.701	40.701	40.701	40.701	40.701	40.701	40.701	40.701	67.835	67.835	40.701	40.701	40.701	40.701	40.701	40.701	40.701	54.268	40.701	54.268	40.701	40.701	40.701	40.701	40.701	40.701	40.701	40.701	67.835	67.835	40.701	40.701	40.701	40.701	40.701
318	27.134	27.134	27.134	27.134	27.134	27.134	27.134	27.134	54.268	54.268	27.134	27.134	27.134	27.134	27.134	27.134	27.134	27.134	27.134	27.134	27.134	27.134	27.134	27.134	27.134	27.134	27.134	27.134	54.268	54.268	27.134	27.134	27.134	27.134	27.134	27.134	27.134	27.134	27.134	27.134	27.134	27.134	27.134	27.134	27.134	27.134	27.134	27.134	54.268	54.268	27.134	27.134	27.134	27.134	27.134
319	58.91	17.742	17.742	17.742	17.742	17.742	17.742	17.742	17.742	17.742	17.742	17.742	17.742	17.742	35.485	17.742	17.742	17.742	17.742	17.742	58.91	17.742	17.742	17.742	17.742	17.742	17.742	17.742	17.742	17.742	17.742	17.742	17.742	17.742	35.485	17.742	17.742	17.742	17.742	17.742	58.91	17.742	17.742	17.742	17.742	17.742	17.742	17.742	17.742	17.742	17.742	17.742	17.742	17.742	35.485
320	64.593	23.425	23.425	23.425	29.108	23.425	23.425	23.425	23.425	23.425	23.425	23.425	23.425	23.425	41.168	23.425	23.425	41.168	17.742	41.168	64.593	23.425	23.425	23.425	29.108	23.425	23.425	23.425	23.425	23.425	23.425	23.425	23.425	23.425	41.168	23.425	23.425	41.168	17.742	41.168	64.593	23.425	23.425	23.425	29.108	23.425	29.108	29.108	29.108	29.108	29.108	29.108	29.108	29.108	46.85
321	87.374	57.516	73.325	96.483	145.92	125.17	80.675	69.051	67.075	82.883	148.33	64.866	94.074	128.1	118.99	128.1	136	67.075	103.18	82.883	103.18	73.325	89.133	112.29	161.72	140.98	103.83	92.209	90.233	106.04	171.48	88.024	117.23	151.26	142.15	151.26	159.16	90.233	126.34	106.04	126.34	96.483	112.29	135.45	184.88	164.13	153.27	141.64	139.67	155.47	220.92	137.46	166.67	200.69	191.58
322	43.34	27.658	27.658	27.658	27.658	27.658	27.658	59.043	42.367	42.367	27.658	27.658	52.449	27.658	27.658	27.658	27.658	40.608	27.658	40.608	43.34	27.658	27.658	27.658	27.658	27.658	27.658	59.043	42.367	42.367	27.658	27.658	52.449	27.658	27.658	27.658	27.658	40.608	27.658	40.608	43.34	27.658	27.658	27.658	27.658	27.658	27.658	59.043	42.367	42.367	27.658	27.658	52.449	27.658	27.658
323	118.86	91.26	91.26	77.693	83.375	91.26	77.693	77.693	104.83	104.83	77.693	77.693	89.058	77.693	95.435	77.693	77.693	109	72.01	109	118.86	91.26	91.26	77.693	83.375	91.26	64.126	64.126	91.26	91.26	64.126	64.126	75.491	64.126	81.868	64.126	64.126	95.435	58.443	95.435	105.29	77.693	77.693	64.126	69.808	77.693	69.808	69.808	96.942	96.942	69.808	69.808	81.174	69.808	87.551
324	262.29	192.17	206.2	204.23	291.31	268.27	190.2	222.27	233.2	247.23	266.3	176.17	256.26	232.28	248.3	232.28	250.32	233.22	216.24	247.25	276.32	206.2	220.22	218.25	305.33	282.3	188.23	220.29	231.23	245.25	264.32	174.2	254.29	230.31	246.33	230.31	248.35	231.25	214.27	245.28	274.35	204.23	218.25	216.28	303.36	280.32	275.31	307.37	318.31	332.34	351.41	261.28	341.37	317.39	333.41
325	716	266	318	318	961	764	266	323	467	568	752	222	644	456	582	467	480	467	358	568	822	323	382	382	1092	876	266	323	467	568	752	222	644	456	582	467	480	467	358	568	822	323	382	382	1092	876	877	1001	1293	1482	1828	773	1632	1275	1503
326	21	16	18	18	30	25	16	18	20	21	25	13	22	22	21	20	20	20	21	21	23	18	20	20	32	27	16	18	20	21	25	13	22	22	21	20	20	20	21	21	23	18	20	20	32	27	28	30	32	33	37	25	34	34	33
327	76	54	60	60	104	88	56	60	70	74	88	50	84	70	72	70	68	70	72	74	82	60	66	66	110	94	56	60	70	74	88	50	84	70	72	70	68	70	72	74	82	60	66	66	110	94	100	104	114	118	132	94	128	114	116
328	0.5908	3.8709	2.9946	5.3755	-4.4328	-3.048	6.173	-3.9483	-5.3478	7.3279	-2.2004	-3.2484	1.2497	4.6766	2.3676	-4.2568	-2.1863	-2.6204	2.4487	-3.2233	-0.0635	-5.5518	3.2359	4.9135	0.313	1.8314	-5.0815	3.5546	5.4762	-8.0486	3.7802	-3.0142	-2.2794	1.5415	3.9994	-4.8645	4.1413	4.5063	-2.8732	2.9826	-0.168	2.851	2.1804	2.3652	2.3328	2.4792	-2.7326	5.0542	7.0942	-8.475	3.3286	-4.7252	5.946	1.3767	-2.8618
329	-1.0319	1.2434	-0.9669	1.3767	-2.9327	-2.8798	1.4666	-2.9756	2.266	-1.695	-5.1512	-0.101	-2.8232	2.8622	-2.6669	0.4397	2.5733	-0.6265	0.8642	-3.4566	-2.014	-0.6552	0.3764	0.3064	-6.2379	-3.1229	2.6999	-0.0919	-1.2321	0.3143	4.0755	-0.5311	5.5786	3.6549	7.2366	3.5325	3.9836	0.4189	-0.9714	2.2754	-3.8336	0.292	-0.1777	-3.2222	1.8416	5.3377	-0.9724	-0.1695	1.2036	-1.1706	-0.36	-3.9142	0.1481	-1.9691	4.1033
330	1.53	1.325	-1.8366	-0.7373	0.4663	0.4688	-0.3748	1.2067	-1.0112	-1.0784	-0.5273	0.9078	-0.3101	-0.9999	-1.7327	-1.9778	1.2279	-0.0471	-1.2908	0.89	-0.6618	-0.204	-0.8641	0.2851	0.2514	-0.972	-1.957	-1.1363	-0.8707	1.3049	0.4161	-1.3254	0.2471	-1.1701	-0.5025	0.6293	-0.2562	1.6265	-1.6004	1.2779	0.4559	-0.263	-0.3743	1.9197	0.9361	0.6376	0.3868	0.9522	-1.1848	2.5087	0.5229	1.2914	1.7236	1.7309	4.8752
331	0	0	0	0	0	0	0	0	0	0	0	0	0	0	0	0	0	0	0	0	0	0	0	0	0	0	0	0	0	0	0	0	0	0	0	0	0	0	0	0	0	0	0	0	3E-14	0	0	0	0	1E-13	0	0	0	0	6E-14
332	0	0	0	0	0	0	0	0	0	0	0	0	0	0	0	0	0	0	0	0	0	0	0	0	0	0	0	0	0	0	0	0	0	0	0	0	0	0	0	0	0	0	0	0	3E-14	0	0	0	0	1E-13	0	0	0	0	6E-14
333	2846	720.43	1182.1	984.06	3483.2	3014.1	1081.1	1144.8	1025.4	1851.4	1614.2	899.71	1943.3	1513.7	2412	1651.7	2392.8	1623.8	1110.5	1298.7	3089	1249.7	1374.2	1078.7	1703.2	2375.5	997.48	1581.4	969.23	1389.4	2828.9	873.93	1792.8	1367.4	2139.6	1531.8	2039.3	1796.1	979.02	2223.3	2904.7	1226.3	1266.3	1167.8	3783.6	1705.6	2618.6	3770.4	3829.3	4850.6	5245	2601.1	3613.2	3639.7	5116.4
334	393.74	262.99	296.1	383.25	511.85	357.65	318.13	393.92	431.88	720.94	1017.4	174.73	648.5	488.31	375.45	495.08	513.46	417.46	415.25	733.64	347.06	268.99	356.98	352.95	1180.6	832.2	256.05	275.79	441.42	865.34	413.32	174.88	673.55	421.57	740.25	495.04	681.31	410.2	338.77	441.47	365.4	267.21	391.85	393.92	616.98	1112.7	512.36	428.93	587.46	634.68	1068.7	752.18	1121.2	758.61	934.81
335	154.6	185.66	85.361	147.41	230.65	140.01	58.919	108.01	247.56	84.288	188.24	88.106	157	190.92	159.96	176.75	128.93	101.72	82.826	160.53	227.23	151.94	115.57	229.32	333.62	202.57	112.14	104.49	274.89	233.97	190.54	84.168	253.81	199.8	144.05	186.54	124.71	112.11	181.74	147.42	288.02	119.86	131.23	182.33	182.52	199.99	338.58	391	311.43	308.76	297.5	84.205	254.31	177.99	415.74
336	5.1818	7.6977	5.4247	12.784	12.592	12.143	18.753	17.836	17.37	16.743	18.989	5.3151	6.1403	20.646	11.56	11.619	4.4818	4.1819	5.1498	17.874	12.923	21.646	4.0751	12.883	20.286	7.1934	21.25	5.6466	12.763	26.269	19.671	5.8766	18.24	14.042	27.713	23.348	22.129	14.835	6.8234	2.9518	11.782	3.9698	2.539	6.8227	3.4782	20.637	6.6746	19.849	26.736	29.568	4.9362	25.776	12.222	5.1441	17.503
337	-86229	-68018	-71649	-67803	-90573	-86988	-64199	-68676	-81679	-85279	-83174	-60657	-83258	-74975	-80298	-74988	-75921	-79693	-70784	-83243	-89818	-71596	-75240	-71398	-94145	-90623	-60386	-64931	-77909	-81433	-79367	-56861	-79400	-71172	-76430	-71166	-72058	-75853	-66984	-79485	-86002	-67845	-71433	-67600	-90397	-86755	-83219	-87655	-100631	-104251	-102188	-79570	-102188	-93991	-99224
338	-556325	-358622	-382364	-384248	-598838	-539108	-346963	-389163	-472762	-506161	-536659	-296713	-505984	-464195	-516026	-467810	-447163	-457655	-414665	-506015	-592959	-388218	-423637	-429966	-669572	-587039	-336290	-369939	-451472	-492901	-530330	-293239	-506184	-471683	-501756	-455004	-444428	-452896	-412691	-483316	-594468	-378837	-425020	-424539	-633212	-594566	-539618	-589391	-678564	-709783	-758369	-486908	-738641	-697785	-734885
339	-18.206	-187.21	-235.05	-149.51	-81.623	-157.09	-132.52	-111.41	-272.75	-287.1	-106.76	-177.79	-130.95	-148.99	-61.871	-160.45	-194.44	-226.69	-175.44	-189.37	-17.947	-179.06	-239.13	-157.37	-69.128	-207.83	-81.811	-140	-266.28	-188.74	-61.675	-138.04	-34.493	-108.26	44.783	-103.35	-93.778	-146.6	-137.67	-193.78	34.604	-193.15	-194.45	-118.94	-83.481	-100.73	-78.525	-27.268	-151.33	-176.86	-46.823	-16.581	16.39	-90.358	87.409
340	-11.619	-9.3093	-10.261	-8.2868	-7.9178	-8.4064	-7.8342	-7.8011	-4.6468	-4.5631	-7.8595	-10.391	-9.3021	-7.6839	-12.984	-8.4898	-8.7537	-10.538	-9.9353	-7.7627	-11.558	-7.8846	-10.288	-8.4362	-7.7616	-9.1837	-7.565	-9.2526	-5.053	-4.3143	-7.733	-10.326	-7.4302	-8.2719	-9.7322	-7.6024	-7.6705	-8.7805	-9.9016	-10.385	-10.863	-10.413	-10.243	-9.2417	-8.3947	-7.5527	-8.7056	-8.2704	-4.7868	-4.2564	-8.3917	-7.4884	-7.9463	-8.4726	-10.711
341	11.619	9.3093	10.261	8.2868	7.9178	8.4064	7.8342	7.8011	4.6468	4.5631	7.8595	10.391	9.3021	7.6839	12.984	8.4898	8.7537	10.538	9.9353	7.7627	11.558	7.8846	10.288	8.4362	7.7616	9.1837	7.565	9.2526	5.053	4.3143	7.733	10.326	7.4302	8.2719	9.7322	7.6024	7.6705	8.7805	9.9016	10.385	10.863	10.413	10.243	9.2417	8.3947	7.5527	8.7056	8.2704	4.7868	4.2564	8.3917	7.4884	7.9463	8.4726	10.711
342	-3.2372	-0.489	0.4488	-0.3502	-0.5035	-0.5775	-1.0961	-1.0967	2.7759	2.9309	-1.0107	0.5156	0.6437	-1.1417	-3.5267	-0.6868	0.59	0.6137	0.735	-0.869	-3.7072	-1.247	0.7502	-0.4511	-1.3342	0.1358	-1.4365	0.3975	4.225	1.3183	-1.0581	0.7568	-0.8209	-0.7326	-3.9153	-1.338	-1.3708	-0.668	0.9568	0.6505	-3.1793	0.677	0.9219	-0.0804	0.2016	-1.3055	-0.1189	-1.0574	1.2748	1.5436	0.1663	-1.0948	-0.6056	0.123	-2.8812
343	472.8	349.1	378.71	404.42	518.95	464.94	388.36	387.69	385	444.52	486.3	348.76	463.56	450.85	474.65	454.35	467.52	411.92	396.83	438.93	499.91	383.35	412.1	413.69	504.83	493.48	391.08	409.33	399.16	451.7	503.51	361.33	481.58	442.12	504.61	467.92	482.96	434.3	399.7	462.02	509.7	393.14	419.06	429.73	540.42	481.89	498.73	515.25	513.66	552.45	610.05	483.36	560.75	550.73	618.53
344	248.22	138.68	123.38	127.76	256.52	224.48	112.26	117.9	104.76	129.71	203.96	126.15	210.77	105.11	187.54	94.986	122.37	152.17	87.94	154.93	230.6	137.07	123.2	96.404	211.67	199.14	81.209	95.484	77.888	103.71	182.94	96.307	192.1	82.227	165.36	69.967	103.13	124.42	54.488	132.66	198.6	104.03	98.602	73.914	200.63	156.29	213.74	216.4	217.87	218.87	308.67	218.49	286.38	178.21	289.79
345	84.263	115.78	98.629	88.44	179.64	156.03	94.732	119.72	151.24	157.01	156.79	93.221	118.62	85.171	71.969	89.391	123.15	121.73	79.562	114.36	78.128	116.66	98.821	86.373	164.41	158.75	80.946	117.02	135.08	154.34	147.46	74.881	111.43	64.094	71.843	74.483	123.3	119.05	76.357	111.74	70.077	89.322	83.95	78.284	154.94	148.15	172.59	232.58	233.83	228.31	237.46	191.2	201.29	158.81	189.2
346	140.32	97.138	156.7	188.22	262.22	231.02	182.49	161.68	135.81	164.2	299.11	140.06	222.34	261.09	215.14	270.82	222.28	146.27	230.41	178.63	191.46	131.13	190.09	230.91	289.66	278.4	230.05	208.53	193.38	195.92	356.25	201.5	265.92	296.08	267.69	324.04	256.54	196.03	268.85	222.44	241.3	200.65	236.51	277.54	367.85	302.01	298.22	262.96	242.52	291.29	430.48	259.96	312.37	385.7	317.32
347	332.48	251.96	222.01	216.2	256.72	233.91	205.87	226.01	249.19	280.32	187.19	208.7	241.22	189.76	259.51	183.53	245.24	265.65	166.42	260.3	308.44	252.23	222.02	182.78	215.18	215.08	161.03	200.8	205.78	255.78	147.27	159.83	215.65	146.04	236.93	143.88	226.43	238.28	130.84	239.58	268.4	192.5	182.55	152.2	172.58	179.89	200.51	252.3	271.14	261.17	179.58	223.4	248.38	165.03	301.2
348	2087.8	736.81	655.52	591.91	1505	1326.4	520.1	594.58	580.29	718.47	1127.3	584.46	1159.3	486.99	1216.4	440.07	623.22	913.49	401.27	930.06	1939.5	728.23	654.54	446.64	1241.9	1176.7	321.02	416.6	378.46	503.94	886.71	380.7	925.93	325.04	960.06	276.58	455.13	662.27	211.58	706.16	1535.3	481.96	456.82	292.18	1040.7	817.23	1108.7	1211.2	1327.5	1333.6	1877	1133.3	1733.7	924.4	2040.1
349	624.47	615.12	524.01	409.74	1054.1	921.99	438.89	603.76	988.98	1026.7	866.57	431.89	652.4	394.6	394.82	414.15	627.2	730.75	363.04	686.52	579	619.8	525.04	400.17	964.78	938.06	319.98	510.55	791.43	904.3	714.74	296	537.09	253.36	345.28	294.43	544.1	633.71	296.49	594.8	471.69	413.83	388.94	309.45	803.85	774.67	895.37	1302	1658.8	1619.6	1444.2	991.93	1218.8	823.9	1143
350	163.95	22.905	24.752	39.32	76.882	68.447	17.528	1.8214	46.479	27.301	47.168	32.931	92.156	19.943	115.58	5.5946	0.7819	30.441	8.3778	40.57	152.47	20.409	24.375	10.031	47.26	40.391	0.2631	21.534	57.191	50.63	35.48	21.426	80.674	18.133	93.513	4.5163	20.161	5.3655	21.869	20.922	128.52	14.707	14.652	4.37	45.689	8.1392	41.152	16.183	15.959	9.4408	71.215	27.297	85.082	19.406	100.59
351	1463.3	121.69	131.51	182.17	450.89	404.45	81.207	9.1854	408.69	308.23	260.7	152.57	506.86	92.396	821.6	25.92	3.9821	182.74	38.228	243.54	1360.5	108.43	129.51	46.472	277.11	238.67	1.04	93.954	412.97	400.35	171.97	84.696	388.85	71.68	614.78	17.853	88.971	28.561	84.916	111.37	1063.7	68.136	67.884	17.275	236.83	42.56	213.28	90.809	331.3	286.06	432.82	141.4	514.89	100.5	897.19
352	1.0963	1.1983	1.1633	1.0616	1.0799	1.1103	1.0761	1.1554	1.2005	1.1426	1.0412	1.1284	1.1215	1.0193	1.0292	1.0216	1.0961	1.161	1.0866	1.1251	1.0752	1.1408	1.1151	1.0338	1.063	1.0847	0.9999	1.0832	1.1307	1.0745	0.9877	1.0385	1.0497	0.9662	0.9627	0.9537	1.03	1.0882	1.0185	1.0653	1.0175	1.0742	1.0468	0.9693	1.0336	1.0325	1.0568	1.1032	1.1213	1.1014	1.0305	1.0703	1.0739	1.0144	1.0184
353	1.9377	4.2762	3.6436	5.5978	5.3355	4.2194	6.3559	5.0892	5.8955	7.5983	5.6262	3.3744	3.103	5.5734	3.9648	4.7144	3.593	2.6946	2.8999	4.8093	2.1209	5.594	3.3704	4.9313	6.2508	3.7485	6.078	3.7329	5.6802	8.1598	5.5743	3.3353	6.0314	4.1356	8.2835	6.0447	5.752	4.8092	3.4293	3.9632	3.8643	2.878	2.2194	4.4341	3.1161	5.9198	2.9261	5.1459	7.2925	8.9157	3.3886	6.2702	6.1925	2.9612	6.9853
354	-77.013	30.849	6.2275	46.078	52.592	38.132	38.073	36.546	18.732	39.668	48.509	7.5019	11.08	33.615	15.844	46.568	13.867	-11.492	15.494	12.333	-67.47	26.969	22.592	36.285	54.205	26.929	43.873	10.124	3.2044	37.682	55.439	3.6021	34.959	45.369	40.39	34.37	33.543	12.369	25.432	4.441	-61.409	-4.1589	2.8405	37.894	28.878	55.811	11.538	47.773	44.281	34.245	44.648	37.411	47.304	27.663	44.692
355	13.382	4.1426	2.8847	5.5906	7.6934	6.7114	4.9385	9.1022	11.465	8.1707	5.8412	3.8783	5.2057	7.7222	8.9882	9.6769	3.3869	11.97	19.162	6.2188	12.104	4.3502	6.5228	8.3481	7.7102	5.6166	7.5832	6.8064	13.407	8.3087	6.7158	5.0894	4.6025	17.536	6.3196	9.2917	6.7939	10.299	13.682	8.7377	11.001	11.014	12.774	8.4123	8.3894	7.3544	10.347	10.531	10.782	9.7053	6.8029	5.0598	5.2751	22.478	9.4717
356	-151.81	-25.278	-64.857	21.118	0.855	-33.859	14.514	-10.477	-41.153	0.6315	-6.5312	-47.36	-54.894	-7.5788	-51.433	11.339	-52.497	-88.779	-54.032	-31.97	-137.79	-8.6055	-48.482	1.1304	15.201	-57.614	20.664	-57.026	-65.363	9.1496	13.189	-52.158	10.789	3.1365	15.275	6.3836	4.622	-37.121	-55.798	-65.242	-119.05	-72.939	-60.348	6.1862	-52.368	11.653	-57.427	1.0473	-9.5435	-50.44	-51.438	6.1187	10.489	-58.794	2.0231
357	-101.95	5.6397	-16.38	36.961	34.656	14.381	30.012	15.895	-5.1542	22.517	30.654	-12.197	-14.514	19.91	-9.5213	33.937	-10.319	-36.74	-11.403	-3.1972	-89.768	15.73	-6.578	22.335	42.153	-1.4703	33.96	-14.6	-22.479	28.272	43.786	-15.569	26.696	23.311	35.388	26.701	23.25	-5.8269	-16.671	-22.798	-76.932	-23.089	-16.451	24.369	2.0378	41.793	-6.8	28.774	20.865	3.2625	13.814	30.015	35.298	-3.1858	30.636
358	0.1279	2.2881	1.6429	0.0465	0.2529	0.4451	0.0393	0.5955	0.0282	0.079	0.063	1.5799	1.3197	0.115	1.5486	0.1088	1.6473	0.0567	0.2522	0.2084	0.1905	0.064	2.0376	0.0998	0.0667	1.1677	0.2716	1.5543	1.0992	0.1169	0.1562	1.7663	0.159	0.3509	0.2161	0.0748	0.1071	0.2021	0.441	1.8834	0.3264	0.2386	0.1353	0.3412	1.5716	0.6485	0.0647	0.263	0.6264	1.5246	1.8694	0.0072	0.2898	0.1722	0.0135
359	-64.023	-79.944	-59.611	-110.4	-106.25	-86.592	-106.02	-93.278	-155.65	-175.58	-89.028	-60.657	-66.207	-88.207	-115.51	-96.153	-57.392	-55.868	-53.967	-87.188	-85.17	-92.3	-64.5	-90.314	-113.02	-66.741	-116.27	-58.428	-144.02	-190.86	-101.51	-59.091	-113.83	-83.552	-181.19	-95.68	-93.804	-85.43	-58.71	-66.57	-100.56	-49.955	-53.707	-86.512	-65.026	-118.86	-64.816	-100.68	-171.85	-160.75	-63.333	-98.214	-116.7	-63.469	-141.17
360	-0.6755	-0.1589	0.1729	0.5195	-0.7713	0.0437	0.2906	0.5638	-0.2521	0.0486	-0.0491	0.2055	0.379	0.0495	0.5275	0.1289	0.1459	0.4101	-0.0357	0.2405	-0.2262	0.1037	0.3586	0.0622	-0.3685	0.252	0.7564	0.3205	0.3209	0.323	0.442	0.1297	0.6037	0.5095	0.6226	0.3025	0.2978	0.1608	0.2439	0.2581	0.2895	0.6923	0.6728	0.2032	-0.6163	0.6014	-0.7542	-0.6101	-0.6333	-0.3936	-0.8073	-0.864	-0.4045	-1.0148	-0.9216
361	6.3881	4.8413	5.7807	3.3316	4.9326	6.5279	2.3719	4.1644	4.7092	3.1801	4.3257	4.4065	5.0454	4.0137	5.4964	3.9555	5.6625	5.576	4.9502	3.3228	6.7597	3.0779	5.6492	3.5291	4.4852	6.946	2.8145	5.8868	5.2089	3.0849	4.7868	4.8789	2.8648	4.7227	3.7218	3.5176	2.9973	3.9735	5.0013	5.9742	6.4374	5.6613	5.5889	4.1075	7.8161	4.7556	6.1617	4.2384	4.6115	7.3414	8.8715	3.3766	4.1814	7.3072	4.8366
362	0.0045	0.0019	0.006	0.0408	2.7544	0.0197	0.0046	0.0045	0.0042	41.88	0.0694	0.003	0.0166	0.0174	0.0082	0.0255	0.0034	0.0035	0.0026	0.0096	0.0076	0.0038	0.007	0.0073	0.4137	0.0042	0.0038	0.0031	0.0095	0.005	0.0159	0.0048	4.3851	0.0052	0.0051	0.0119	0.0142	0.0096	0.0064	0.0044	0.0071	0.003	0.0057	0.0102	0.0404	0.0105	0.0121	0.0165	0.007	0.0424	0.0109	0.0081	0.0875	0.0089	4.1174
363	5.7181	14.096	12.127	-0.3712	5.8285	10.023	0.421	6.2253	7.9358	5.6726	7.6737	9.6289	13.645	1.8046	8.8043	-1.2399	13.344	7.2349	2.5687	5.5396	3.4702	3.6429	14.602	1.9116	0.1583	14.417	-1.5132	10.156	5.6475	-2.4233	-0.447	7.307	0.0336	-1.0611	-5.8778	-5.5176	0.0973	3.5606	22.735	10.386	-2.5311	1.3232	0.1212	0.4611	9.6794	0.658	2.519	4.576	8.0298	12.804	14.098	-0.1835	2.6638	1.9062	0.6558
364	49.851	30.918	48.477	15.843	33.801	48.241	15.498	26.372	35.998	21.885	37.185	35.162	40.38	27.489	41.911	22.598	42.178	52.039	42.629	28.773	48.018	24.335	41.904	21.204	26.951	56.144	13.296	42.426	42.884	19.123	30.597	36.589	15.907	20.174	20.113	20.318	18.628	31.294	39.127	42.444	42.114	49.85	43.896	18.183	54.406	30.141	50.627	27.727	30.408	53.703	65.251	23.897	24.81	55.608	28.613

Table S12 Amino acid descriptors (2D) used in this study.

	S	S	S	S	S	S	T	T	T	T	T	T	T	T	T	T	T	T	T	T	T	T	T	T	T	T	V	V	V	V	V	V	V	V	V	V	V	V	V	V	V	V	V	V	V	V	W	W	W	W	W	W	W	W	W
	R	S	T	V	W	Y	A	C	D	E	F	G	H	I	K	L	M	N	P	Q	R	S	T	V	W	Y	A	C	D	E	F	G	H	I	K	L	M	N	P	Q	R	S	T	V	W	Y	A	C	D	E	F	G	H	I	K
No.	SR	SS	ST	SV	SW	SY	TA	TC	TD	TE	TF	TG	TH	TI	TK	TL	TM	TN	TP	TQ	TR	TS	TT	TV	TW	TY	VA	VC	VD	VE	VF	VG	VH	VI	VK	VL	VM	VN	VP	VQ	VR	VS	VT	VV	VW	VY	WA	WC	WD	WE	WF	WG	WH	WI	WK
365	0.525	0.3973	0.3258	0.3159	0.4943	0.4828	0.2891	0.3041	0.2721	0.2918	0.4194	0.3617	0.4547	0.2331	0.3951	0.2091	0.2617	0.3694	0.2216	0.353	0.4613	0.3575	0.2989	0.233	0.4193	0.4035	0.2077	0.2333	0.1951	0.2296	0.3633	0.2665	0.3989	0.186	0.3277	0.1495	0.2135	0.2865	0.1363	0.2871	0.3896	0.2646	0.2353	0.172	0.3713	0.3243	0.4286	0.42	0.4241	0.3962	0.506	0.452	0.5107	0.3236	0.4685
366	0.1782	0.3316	0.2604	0.2187	0.3462	0.3356	0.2439	0.3088	0.3928	0.3532	0.3224	0.2673	0.2559	0.1889	0.1516	0.1967	0.2634	0.2955	0.2005	0.2605	0.1563	0.3043	0.2398	0.2088	0.3257	0.3217	0.207	0.2859	0.3384	0.3417	0.2929	0.2072	0.2314	0.145	0.1424	0.1592	0.2553	0.2741	0.191	0.2419	0.1375	0.2272	0.2003	0.1822	0.2867	0.3074	0.3461	0.4514	0.4552	0.4133	0.3892	0.3956	0.359	0.2884	0.3059
367	0.2968	0.2783	0.4138	0.4654	0.5053	0.4969	0.4699	0.417	0.3527	0.3694	0.6151	0.4016	0.4796	0.5791	0.4533	0.5961	0.4754	0.3551	0.5806	0.407	0.383	0.3421	0.4613	0.5582	0.5738	0.5642	0.5882	0.5094	0.4845	0.4337	0.7075	0.5577	0.5522	0.6697	0.5305	0.6925	0.5312	0.4514	0.6726	0.4814	0.4734	0.5104	0.5644	0.6458	0.6807	0.6267	0.598	0.5103	0.4721	0.5273	0.7056	0.5378	0.5571	0.7003	0.513
368	0.7032	0.7217	0.5862	0.5346	0.4947	0.5031	0.5301	0.583	0.6473	0.6306	0.3849	0.5984	0.5204	0.4209	0.5467	0.4039	0.5246	0.6449	0.4194	0.593	0.617	0.6579	0.5387	0.4418	0.4262	0.4358	0.4118	0.4906	0.5155	0.5663	0.2925	0.4423	0.4478	0.3303	0.4695	0.3075	0.4688	0.5486	0.3274	0.5186	0.5266	0.4896	0.4356	0.3542	0.3193	0.3733	0.402	0.4897	0.5279	0.4727	0.2944	0.4622	0.4429	0.2997	0.487
369	4.4158	2.1106	1.731	1.4636	2.9001	2.853	1.3392	1.5336	1.5072	1.6163	2.318	1.6758	2.5008	1.0802	2.5627	0.9686	1.333	2.2176	1.0112	2.1189	3.8798	1.8996	1.5883	1.0796	2.46	2.3846	0.8209	1.0178	0.9481	1.1156	1.7611	1.0536	1.9227	0.7352	1.9026	0.5911	0.9424	1.5249	0.5293	1.5284	3.0122	1.2259	1.0901	0.6799	1.9257	1.6959	2.223	2.3506	2.5843	2.4139	3.0768	2.3447	3.0918	1.6785	3.2984
370	1.3208	1.762	1.3837	1.0131	2.0313	1.9831	1.1301	1.5573	2.5688	2.3097	1.782	1.2383	1.4074	0.8752	0.8318	0.9115	1.3416	1.774	0.9148	1.5641	1.1582	1.6168	1.274	0.9673	1.9111	1.9009	0.8182	1.2473	1.9827	2.002	1.4195	0.8192	1.1153	0.5731	0.6842	0.6292	1.1266	1.4591	0.7418	1.2874	0.9254	1.0526	0.9281	0.7201	1.4874	1.6076	1.7953	2.5269	3.2293	2.9317	2.3674	2.0522	2.1736	1.496	1.8479
371	0.066	0.2548	0.1121	0.1406	0.0615	0.071	0.1181	0.1356	0.3028	0.0758	0.1587	0.1722	0.0914	0.1589	0.093	0.1477	0.0725	0.0999	0.1204	0.1475	0.1044	0.17	0.1025	0.1919	0.2168	0.1039	0.1486	0.1413	0.2767	0.2052	0.0824	0.1642	0.1663	0.1408	0.078	0.1493	0.0845	0.1126	0.2059	0.088	0.1156	0.1257	0.12	0.1475	0.0598	0.1738	0.1195	0.1116	0.0953	0.09	0.0786	0.069	0.1102	0.0764	0.0679
372	15.242	4.1643	7.0248	15.096	15.257	14.735	21.138	20.736	24.411	21.414	21.756	6.3243	6.7757	21.482	24.03	14.174	6.9156	5.4851	3.1003	19.579	15.196	22.671	6.2369	15.458	20.351	7.3691	22.541	6.5828	14.294	25.385	21.605	6.7632	22.433	16.744	29.53	23.562	22.392	16.078	5.8367	8.2667	16.949	2.2753	2.3272	16.527	3.3587	22.302	6.987	22.772	31.655	28.626	2.8568	26.461	18.998	5.0278	11.837
373	-86540	-68347	-71953	-68050	-90810	-87256	-64438	-69705	-81941	-85564	-83414	-60913	-83553	-75255	-80600	-75263	-77013	-79999	-71069	-83534	-90144	-71869	-75555	-71659	-94402	-90931	-60601	-65962	-78203	-81717	-79580	-57087	-79641	-71418	-76696	-71423	-73100	-76102	-67241	-79776	-86309	-68121	-71725	-67821	-90654	-87015	-83449	-88640	-100874	-104555	-102426	-79758	-102410	-94259	-99488
374	-530354	-337986	-376697	-378801	-587189	-530865	-337918	-380255	-453290	-495359	-530060	-290304	-498533	-457163	-492334	-457120	-440542	-449745	-409474	-495342	-576419	-379120	-418776	-422343	-651561	-580407	-332123	-364810	-442137	-486353	-518607	-287300	-499170	-458939	-486016	-447613	-439354	-445048	-406873	-474516	-572141	-373593	-418334	-415830	-623739	-579325	-533008	-578064	-668902	-698471	-748692	-485824	-733626	-680116	-729827
375	21.465	-211.18	-213.8	-94.344	-42.76	-112.43	-87.644	-76.299	-189.24	-209.53	-58.941	-166.94	-132.92	-94.514	-4.2349	-101.14	-179.61	-199.7	-166.84	-113.65	20.245	-130.48	-200.51	-101.18	-36.264	-187.13	-37.283	-121.53	-236.8	-148.91	-11.588	-127.23	-6.5476	-45.573	115.26	-50.445	-51.907	-85.925	-125.78	-143.13	68.411	-169.31	-169.53	-51.782	-76.059	-56.094	-77.061	9.6818	-98.248	-177.91	-49.244	11.058	35.644	-78.167	130.93
376	-11.869	-10.662	-10.548	-7.9809	-7.9358	-8.0639	-7.3721	-7.6762	-4.2042	-4.5614	-7.4277	-10.526	-9.3439	-7.4339	-12.628	-8.1327	-9.9101	-10.627	-10.273	-7.4116	-12.019	-7.4763	-10.363	-8.0239	-7.4818	-9.0406	-7.3332	-9.8939	-4.9787	-4.3182	-7.3513	-10.468	-7.094	-7.8871	-9.358	-7.3534	-7.4075	-8.3303	-10.348	-10.596	-11.77	-10.59	-10.452	-7.7934	-8.5517	-7.3404	-8.754	-7.6784	-4.3093	-4.2925	-8.4644	-7.0981	-7.5254	-8.5688	-10.833
377	11.869	10.662	10.548	7.9809	7.9358	8.0639	7.3721	7.6762	4.2042	4.5614	7.4277	10.526	9.3439	7.4339	12.628	8.1327	9.9101	10.627	10.273	7.4116	12.019	7.4763	10.363	8.0239	7.4818	9.0406	7.3332	9.8939	4.9787	4.3182	7.3513	10.468	7.094	7.8871	9.358	7.3534	7.4075	8.3303	10.348	10.596	11.77	10.59	10.452	7.7934	8.5517	7.3404	8.754	7.6784	4.3093	4.2925	8.4644	7.0981	7.5254	8.5688	10.833
378	-4.3194	0.6904	0.7466	-1.1224	-0.8239	-0.8928	-1.4845	-1.6316	1.415	1.9056	-1.581	0.8355	0.5239	-1.6042	-4.5072	-1.0074	0.7597	0.5387	0.3265	-1.3512	-4.2557	-1.7065	1.0687	-0.9101	-1.6919	-0.0449	-1.7929	0.8085	4.0783	0.9657	-1.5685	0.8652	-1.4345	-1.4443	-4.1282	-1.7679	-1.7576	-1.1257	0.3467	0.6908	-4.4978	0.7553	0.896	-1.5277	-0.0521	-1.7703	-0.2507	-1.5039	0.9308	1.4131	0.0156	-1.6284	-0.8946	-0.0622	-3.3702
379	0.1692	0.4561	0.258	0.388	0.1857	0.1476	0.2695	0.3196	0.4661	0.313	0.4569	0.2446	0.3106	0.3392	0.192	0.3129	0.2194	0.2543	0.3264	0.4398	0.1671	0.2771	0.273	0.4065	0.5246	0.3226	0.2936	0.2015	0.5077	0.4875	0.1861	0.247	0.3759	0.3473	0.307	0.3362	0.292	0.2367	0.3949	0.2209	0.1998	0.2591	0.3153	0.3689	0.1816	0.4652	0.2717	0.1932	0.2035	0.172	0.2163	0.2492	0.2905	0.2129	0.2229
380	0.9261	0.9212	0.8574	0.8271	0.9295	0.9354	0.8148	0.8167	0.8734	0.7525	0.6861	0.9194	0.8104	0.8514	0.9227	0.8515	0.8686	0.8453	0.7821	0.7179	0.9651	0.9228	0.8606	0.9137	0.7066	0.8036	0.8851	0.911	0.882	0.7199	0.9314	0.9135	0.8299	0.876	0.793	0.8478	0.7987	0.8648	0.8808	0.8896	0.9762	0.9013	0.8429	0.8644	0.9013	0.6767	0.9445	0.9835	0.9375	0.9406	0.8778	0.8009	0.817	0.868	0.9145
381	10.157	15.01	6.4439	7.8741	9.3924	13.829	20.175	18.28	17.482	16.194	19.151	6.1134	5.497	19.373	22.21	9.901	5.7017	5.3033	5.8673	16.067	10.889	22.672	3.9041	8.9538	19.298	7.6117	21.697	7.0329	12.643	30.268	9.9527	6.2341	19.149	8.5572	25.122	22.035	20.824	19.582	6.4484	8.558	13.852	4.9966	3.8318	10.002	4.2867	21.52	6.811	19.47	27.241	30.163	6.1249	24.458	21.295	5.7505	8.23
382	-77381	-61985	-65480	-62120	-82583	-79797	-58652	-62942	-74639	-78098	-76459	-55260	-75081	-69001	-73344	-69016	-69892	-72316	-64885	-75720	-80833	-65428	-68928	-65572	-86009	-83283	-55319	-59668	-71353	-74725	-73150	-51935	-71703	-65706	-69980	-65670	-66514	-68936	-61560	-72433	-77496	-62159	-65605	-62240	-82735	-79898	-75850	-80087	-91772	-95241	-93642	-72343	-92165	-86186	-90478
383	-526349	-343161	-376724	-381186	-588290	-526229	-330331	-377514	-465553	-500203	-527507	-292209	-492763	-455788	-496873	-458203	-438043	-447511	-405276	-498056	-577534	-377396	-418304	-430829	-656661	-572175	-330267	-363888	-444320	-475522	-530543	-288950	-491536	-463607	-495536	-447222	-434521	-436327	-404095	-475420	-573150	-372937	-417050	-414043	-623792	-582857	-534107	-574071	-665293	-697228	-747943	-479443	-738389	-680850	-734199
384	-20.011	-167.64	-221.39	-143.28	-90.408	-144.3	-127.01	-117.9	-265.7	-282.29	-91.784	-175.48	-144.97	-146.29	-30.251	-156.9	-181.85	-221.15	-180.58	-182.35	-28.081	-170.73	-225.69	-156.03	-81.674	-193.36	-80.006	-132.34	-269.62	-199.82	-79.81	-139.45	-55.934	-132.62	45.106	-104.95	-94.696	-129.75	-144.97	-182.18	20.434	-190.74	-190.5	-118.04	-94.045	-97.826	-97.456	-37.325	-173.16	-197.46	-57.196	-33.56	-0.8156	-103.68	59.253
385	-11.511	-8.6053	-10.333	-9.3875	-8.1242	-8.4126	-8.2242	-8.2936	-4.6749	-4.7246	-8.0705	-10.179	-9.4599	-8.0827	-12.461	-9.0569	-9.0221	-9.6604	-9.8641	-8.1513	-11.461	-7.9151	-10.151	-9.1546	-8.0827	-9.0206	-7.4891	-9.2691	-5.3887	-4.0665	-9.0042	-10.117	-7.3619	-8.7199	-10.337	-7.911	-8.0355	-8.4222	-9.8963	-9.8514	-11.509	-10.095	-9.8751	-8.8417	-8.2985	-7.5058	-8.8482	-8.3901	-4.8847	-4.1938	-8.6702	-7.8205	-7.2919	-8.6395	-11.159
386	11.511	8.6053	10.333	9.3875	8.1242	8.4126	8.2242	8.2936	4.6749	4.7246	8.0705	10.179	9.4599	8.0827	12.461	9.0569	9.0221	9.6604	9.8641	8.1513	11.461	7.9151	10.151	9.1546	8.0827	9.0206	7.4891	9.2691	5.3887	4.0665	9.0042	10.117	7.3619	8.7199	10.337	7.911	8.0355	8.4222	9.8963	9.8514	11.509	10.095	9.8751	8.8417	8.2985	7.5058	8.8482	8.3901	4.8847	4.1938	8.6702	7.8205	7.2919	8.6395	11.159
387	-3.9203	-1.6409	-0.1145	0.0051	0.0684	-1.0312	-1.4286	-1.6331	2.565	2.949	-1.1496	0.1539	0.5069	-1.1393	-4.7895	-0.8372	0.0061	0.2053	0.0391	-1.1201	-3.8188	-1.6814	0.0906	-0.8057	-1.6686	0.3138	-1.9196	0.0562	3.8502	0.6269	-0.6294	0.2349	-1.3202	0.449	-4.6146	-1.6498	-1.7185	-1.9799	0.0841	0.133	-4.3677	0.3766	0.6055	-0.6566	0.1723	-1.8336	-0.3816	-1.0968	1.2015	1.3312	-0.2096	-1.4059	-1.0886	-0.1665	-3.6057
388	3394.3	1169.1	1563.6	1514.7	4225.7	3511.7	1458.1	1646.8	1704.8	2656.6	2819.8	1162.5	2748.8	2193	2947.4	2323.5	3035.2	2143	1608.5	2192.9	3663.3	1670.6	1846.7	1661	3217.5	3410.3	1365.7	1961.7	1685.5	2488.7	3432.7	1133	2720.2	1988.7	3023.9	2213.4	2845.3	2318.4	1499.5	2812.2	3558.1	1613.4	1789.4	1744	4583.1	3018.3	3469.5	4590.3	4728.2	5794	6611.2	3437.5	4988.6	4576.3	6466.9
389	548.24	448.57	381.43	530.62	741.79	497.57	377.03	492.78	679.29	805.11	1202.4	262.78	805.13	679.21	535.14	671.8	637.8	519.13	498.01	893.99	574.28	420.87	472.53	582.05	1513.1	1034.7	368.05	374.19	716.21	1099.2	603.54	259.04	927.14	621.34	884.12	681.39	794.68	522.16	520.41	588.73	653.34	386.98	522.77	576.18	799.37	1311.1	850.71	814.68	898.78	943.21	1365.9	835.72	1375.2	936.3	1349
390	3000.6	906.04	1267.5	1131.1	3714	3154.1	1140	1259.1	1272.9	1935.8	1805.6	987.84	2100.5	1704.6	2572	1828.2	2525.3	1725.5	1193.3	1459.2	3316.2	1401.6	1489.8	1308.2	2037.8	2578	1109.6	1691.9	1244.2	1623.4	3019.7	958.1	2046.8	1567.1	2283.8	1718.5	2173.9	1908.2	1160.8	2370.7	3192.6	1346.2	1397.8	1350.1	3966.2	1907.1	2957.3	4148.1	4140.8	5159.5	5542.6	2685.8	3867.7	3817.9	5533.6
391	3239.8	983.57	1478.3	1367.7	3995.5	3371.8	1399.2	1541.6	1457.4	2572.4	2631.7	1074.5	2592	2002.1	2787.6	2147.1	2907.4	2041.3	1525.7	2032.6	3436.1	1518.8	1731.2	1431.8	2884	3207.9	1253.7	1857.3	1410.7	2254.9	3242.2	1048.8	2466.4	1789	2879.9	2026.9	2722	2206.4	1317.9	2664.9	3270.4	1493.6	1658.2	1561.8	4400.6	2818.3	3131	4217.8	4416.8	5485.4	6313.9	3353.5	4734.3	4398.4	6051.2
392	3.5974	2.4665	2.7537	2.7234	3.8087	3.6181	2.7688	2.7219	2.7038	3.2781	3.2541	2.5688	3.2752	3.0726	3.4453	3.1628	3.4822	3.0313	2.7274	2.9781	3.6411	2.8464	2.8958	2.7587	3.2462	3.4757	2.6936	2.9841	2.6999	3.1855	3.6037	2.5503	3.2707	2.9386	3.5037	3.1001	3.3848	3.1663	2.6455	3.3861	3.6013	2.8107	2.8633	2.8397	3.8869	3.2813	3.55	3.8644	3.8541	4.1754	4.3375	3.6272	3.8228	3.7972	4.4041
393	3.4976	1.9753	2.5949	2.5644	3.8119	3.5477	2.5294	2.5392	2.1472	2.9007	2.7832	2.3228	3.1017	2.8526	3.5499	2.9322	3.3494	2.8721	2.5938	2.5533	3.635	2.4483	2.9106	2.4941	2.6598	3.1646	2.5223	2.7409	2.2002	2.5568	3.6999	2.3022	2.9925	2.8933	3.4826	2.9925	3.2847	3.0528	2.3869	3.2049	3.6595	2.6254	2.8118	2.7918	3.9605	2.6373	3.1576	3.4159	3.47	3.8015	4.031	3.0626	3.2771	3.7778	4.2905
394	1.4443	1.3308	1.2976	1.4362	1.4772	1.2316	1.3646	1.32	1.3482	1.5739	1.8596	1.0692	1.5771	1.4684	1.2018	1.4876	1.4758	1.2709	1.3743	1.6768	1.32	1.3036	1.2549	1.4408	2.0281	1.7393	1.2937	1.1477	1.4247	1.5855	1.3159	1.2485	1.5833	1.3555	1.6287	1.4401	1.4399	1.1839	1.4021	1.4299	1.3391	1.3014	1.369	1.377	1.5426	1.9876	1.694	1.3999	1.4263	1.4206	1.9478	1.8513	1.9575	1.6037	1.7208
395	0.8983	0.9971	0.8688	0.9614	0.9455	0.9455	0.8691	0.9351	1.1815	0.7984	1.1089	0.9638	0.9376	1.1372	1.0828	1.1268	0.9021	0.9077	0.8998	0.9805	1.1743	1.0093	0.9316	1.0925	1.2383	1.0203	0.9725	1.0304	1.1573	1.1583	1.0619	0.933	1.2204	1.0858	0.9723	1.1564	0.9549	1.0244	1.083	0.9508	1.2442	0.9309	0.974	1.0722	0.9682	1.0994	1.0916	1.1409	1.071	1.1403	1.1304	0.8045	1.0878	1.0439	1.1176
396	239.25	160.38	177.25	192.38	269.75	241.63	176.75	192.38	194.25	216.38	255.75	156.13	228.5	227.88	241.25	227.38	228.38	200.88	199	219.75	257	180.75	197.5	211.13	287.25	260.25	188.25	203.38	204.5	228.25	267.63	167.75	242.25	238.38	255.88	241.5	241.13	212.5	210.38	230.25	269.63	190.13	208.5	223.13	293.5	271.5	260.5	278.63	283.88	301.75	341	244.13	317.88	312.88	327.38
397	282.35	193.94	212.12	239.29	311.93	276.24	217.82	229.15	226.18	264.05	296.05	192.23	268.56	273.37	287.42	272.3	271.92	235.96	232.21	259.06	306.73	221.93	237.33	253.32	328.46	298.95	226.77	238.25	238.71	273.35	310.39	201.01	291.6	280.19	310.98	285.4	288.86	253.34	238.45	272.85	320.2	224.45	246.55	264.8	334.85	315.5	297.44	317.2	319.58	340.5	378.5	283.58	366.51	352.68	381.7
398	3.5959	0.3336	3.8001	4.0415	5.2044	5.1535	1.8843	1.8803	2.636	2.1676	2.9929	3.0722	3.5522	2.6755	4.1751	3.7135	5.1239	2.6474	3.9132	0.9763	3.5402	1.3967	1.2377	4.0607	2.6474	3.5334	2.1776	3.8068	3.4091	2.7166	3.6495	4.6694	3.4024	1.7279	3.2658	1.2484	1.3522	3.2016	4.2615	4.0521	2.7439	4.1139	2.3274	1.6239	4.6769	2.1516	5.1252	5.4825	5.1241	5.0893	4.3289	4.0792	4.9096	1.6372	3.8604
399	0.0443	0.0175	0.027	0.0391	0.0553	0.0522	0.0442	0.0407	0.0285	0.0244	0.0589	0.0298	0.0405	0.0508	0.0429	0.0613	0.0499	0.0251	0.0545	0.0305	0.0425	0.021	0.0264	0.0542	0.0664	0.058	0.0603	0.0743	0.0381	0.0361	0.0837	0.0581	0.0583	0.0823	0.0605	0.0724	0.0694	0.0463	0.0797	0.0387	0.0494	0.0483	0.0507	0.0556	0.0966	0.0719	0.0888	0.084	0.0421	0.0499	0.1025	0.0763	0.0586	0.0978	0.0685
400	3.0401	3.2633	3.1278	3.0011	2.9364	3.0178	3.0595	3.0258	3.0938	2.9908	2.8263	3.1907	2.95	2.875	2.9945	2.8505	2.8586	3.0703	2.9651	3.0115	2.9422	3.1473	3.0138	2.921	2.8559	2.8822	2.9535	2.9207	3.0121	2.9407	2.8136	3.0671	2.8673	2.8248	2.9107	2.7729	2.7683	2.9538	2.9234	2.9386	2.9066	3.0414	2.9096	2.8227	2.8031	2.8345	2.877	2.8587	2.8817	2.8945	2.778	2.9228	2.8295	2.7161	2.8514
401	2.7907	2.9478	2.628	2.4037	2.6249	2.7398	2.5115	2.6462	2.7546	2.6573	2.3923	2.8152	2.558	2.1874	2.673	2.1859	2.1567	2.6977	2.4628	2.615	2.6038	2.8092	2.5804	2.2858	2.4863	2.459	2.3156	2.3563	2.4644	2.4732	2.2689	2.3869	2.3264	2.0532	2.4624	1.9191	2.0343	2.3995	2.2235	2.4055	2.4393	2.4245	2.2508	1.9876	2.2671	2.3406	2.4875	2.5699	2.6078	2.6072	2.4379	2.6363	2.5317	2.1571	2.6028
402	2.1158	2.4377	2.1682	1.803	1.8134	1.9893	2.0087	2.0255	2.3244	2.2019	1.5894	2.1861	1.869	1.681	2.0061	1.72	1.5483	2.2561	1.8467	2.074	1.9184	2.3528	2.0636	1.69	1.7438	1.7953	1.6911	1.7962	1.9731	2.0476	1.3712	1.7671	1.6399	1.4676	1.7518	1.3933	1.3825	1.9374	1.496	1.8591	1.6831	1.9206	1.7233	1.4733	1.453	1.6486	1.6213	1.7285	1.9023	1.8702	1.3764	1.7708	1.6727	1.3965	1.7607
403	1.1949	1.6877	1.5272	1.1903	1.0274	1.0871	1.212	1.1947	1.5247	1.4499	0.9683	1.4101	1.1823	1.0384	1.151	1.021	1.0212	1.526	1.0724	1.383	1.1057	1.586	1.3946	1.0543	0.9995	1.0285	1.0238	0.8782	1.3279	1.311	0.7723	1.1001	1.0004	0.8442	0.9678	0.8524	0.8148	1.1655	0.8717	1.2149	0.9963	1.2133	1.0795	0.9366	0.7623	0.9431	0.8474	0.8318	1.1807	1.0992	0.7241	0.9281	0.9819	0.7067	0.8609
404	0.669	0.9873	0.8956	0.6851	0.5836	0.6014	0.6729	0.661	0.9469	0.9118	0.5802	0.7845	0.6972	0.6124	0.5692	0.5369	0.5997	0.894	0.6045	0.8497	0.645	0.8791	0.7651	0.5682	0.5396	0.5468	0.4965	0.4327	0.7624	0.7522	0.4134	0.571	0.5534	0.4108	0.4387	0.4678	0.4644	0.6439	0.4758	0.6649	0.5751	0.665	0.5741	0.5382	0.3675	0.4731	0.4648	0.4436	0.6659	0.5753	0.3574	0.4942	0.5467	0.3587	0.4267
405	0.3601	0.3925	0.3781	0.3582	0.2997	0.2788	0.2724	0.3318	0.527	0.4964	0.2921	0.3201	0.3445	0.2757	0.2259	0.2068	0.2536	0.397	0.307	0.4237	0.3292	0.4198	0.3269	0.2447	0.2406	0.2419	0.2158	0.1803	0.3831	0.3792	0.2148	0.2086	0.3113	0.193	0.1903	0.218	0.2269	0.3317	0.204	0.293	0.3036	0.3159	0.2755	0.2846	0.1536	0.2094	0.2197	0.2295	0.37	0.2653	0.1623	0.1997	0.2901	0.1797	0.213
406	0.1787	0.1119	0.1156	0.1514	0.1279	0.1125	0.0707	0.1492	0.2448	0.1917	0.1113	0.1031	0.1334	0.1069	0.0823	0.0754	0.0791	0.1591	0.1292	0.1842	0.1496	0.1433	0.1162	0.0988	0.0908	0.1154	0.0774	0.0555	0.1443	0.145	0.0829	0.059	0.1536	0.0844	0.0682	0.0852	0.0858	0.1304	0.0628	0.0742	0.1224	0.1175	0.135	0.1271	0.0417	0.0661	0.0899	0.1153	0.1774	0.0912	0.0551	0.0644	0.1459	0.0767	0.1071
407	0.0909	0.0235	0.0369	0.0328	0.039	0.0333	0.0203	0.0681	0.0993	0.0557	0.0438	0.0379	0.0579	0.0385	0.0271	0.0302	0.0314	0.059	0.036	0.08	0.0787	0.0452	0.0572	0.037	0.0206	0.0493	0.0286	0.0196	0.0367	0.0454	0.0169	0.0208	0.0467	0.0302	0.0247	0.0316	0.0342	0.0329	0.0217	0.0176	0.0325	0.0424	0.0674	0.0418	0.0132	0.0133	0.0298	0.0509	0.0811	0.0287	0.014	0.0215	0.0618	0.0268	0.0557
408	510.5	192.25	278.13	343.13	635.38	565.88	340.13	375.88	326.88	374.63	583.25	256.38	460.63	457.75	434.25	469.25	524.25	330.63	406.5	373.63	522.13	244.5	296.88	405.13	586.25	580	368.75	469.38	372.25	408	666.88	344.63	507.88	510.5	509.88	509	583.63	458.25	473.88	435.13	564.63	367.25	392.25	419.75	775.25	570.25	664.38	735.38	631.5	735	967	648.88	700.5	800.38	801.25
409	298.5	98.375	149.25	177.75	366.5	323	186.63	199.88	181.25	201.13	328.5	138.88	255.5	256.25	239.25	254.75	288.63	175.88	219.38	203.75	294.25	127.5	156.63	213	338.13	328.63	196.88	253.88	198.63	223.63	371.38	181.75	281.5	274.5	281.5	279.38	327.25	251.25	258.75	234.88	316.13	201.75	207.75	218.63	443.38	311.13	399.75	419.38	346.5	415.88	561.25	381.38	400.63	449	473.75
410	205.13	66.125	111	131.38	261.75	221	139.75	137.5	125.13	143.25	229.75	97.125	183.25	197.38	172.75	198.25	218.25	128.5	159.13	147.63	209	92.25	112.5	158.25	242.25	236.5	150.88	186.5	144.38	163.88	273.88	139.25	213	212.75	207	219.63	249	186.13	192	172.88	229.75	154.38	162	175.25	319.13	224.75	290.25	291	236.75	283.63	387.13	266.88	281.63	325.63	332.88
411	160.5	47.875	89.875	109.38	215.13	175.13	107.88	104.5	91.625	111.38	191.5	75.75	143.13	165.88	133.5	164.75	187.38	99.5	129.75	118.38	168.75	69.375	87.875	132.38	198.13	190.13	123.5	142.25	119.75	130.13	231.38	117.88	177.63	184	163.38	192.25	215.88	148.75	166.5	141.88	186.88	129.25	138	155	266	184.38	235.75	234.5	188.63	229.5	322.5	218	231.38	277.5	266.88
412	130.25	31.25	70.875	88.375	180.5	143.5	85.75	77	72.375	86.5	162.25	56.25	114.88	137.13	101.13	129.25	161.63	72.875	100.5	94.375	135.13	48.75	62.875	109.25	164	152.38	97	108.5	96.625	102.25	199	99.625	147.63	152.25	125.75	162.25	182.63	120.75	140.88	115.5	152.75	105.75	110.13	128.63	222.38	152.13	198.63	192.88	157.5	193.88	280.75	182.25	195.38	236	221.5
413	107.25	21.5	46.875	61.5	152.5	117.25	52.75	48.875	56.25	62.5	125.75	36.625	89.375	88.25	72.125	75.125	105.38	44.875	70.875	65.75	103.25	33.25	33.125	73	128.38	107.5	60.625	67.875	69.125	68.875	155	66	105.63	92.125	88.625	100.13	124.38	80.75	104.38	75.75	116.63	69.875	66.5	77.875	173.63	110.38	154.25	159.13	131.5	160.13	240.25	157.5	162.88	175.5	185.63
414	78	9.75	25	24.875	102.88	88.625	31.375	27.125	39.375	42	77.75	22.375	52.5	46.625	45.75	32.875	58.25	27.375	35.125	38.375	66	17.375	14.875	29.125	79.25	70.25	23.875	31.75	34.75	37.25	86.75	30	51.875	32.75	43.75	36.25	62.5	42.5	48.125	37.25	67.375	35.25	26.625	19.5	108.5	63.875	105.25	107.75	86.25	107.5	156.25	112.13	105.38	103.5	127.25
415	44.375	4.5	14.5	11	61.75	51.875	21	14.375	26.75	24.25	38.5	9.25	28.75	23.5	22	18.75	25.375	16.5	19.125	19.375	38.75	8.625	5.75	7.625	41.125	36.25	11.875	16.25	16.75	24.75	44.625	9.375	29.875	12.5	22.25	16.75	31.375	24	17.25	15.75	34.5	21.75	14.5	6	59.25	30.5	60.75	57.125	47.375	58.125	78.125	68.5	55.625	53.625	73
416	0.5	0.866	0.5	1.2247	0.7071	0.5	0.5	6.6895	0.5	0.866	0.866	6.8374	1.2247	0.866	0.7071	6.8739	0.5	0.7071	0.5	0.5	0.7071	0.7071	6.7823	0.5	6.9642	0.5	0.7071	2.7386	6.442	0.5	0.5	0.5	0.5	0.5	7.0356	0.7071	0.866	1.2247	6.5955	0.5	1.118	6.3443	0.5	0.5	0.5	0.5	3.1623	6.892	0.5	0.7071	0.5	0.5	2.1213	0.5	0.866
417	0.5	0.5	2.5	1.5	0.5	0.7071	6.7454	6.3443	0.5	0.5	0.7071	6.5192	0.5	0.7071	0.5	6.9642	0.5	0.5	0.7071	0.5	1.5	1.5	0.866	1.2247	6.6895	0.5	0.5	6.9642	7.1239	0.7071	0.5	0.5	0.7071	0.5	7.2973	0.7071	1.5	0.7071	1.5	0.5	0.5	0.5	0.5	0.7071	0.5	9.6047	1.2247	3.2016	0.7071	0.5	0.7071	0.5	2.5981	0.7071	6.4807
418	0.7071	1.2247	3	0.5	0.5	1.118	6.8739	1.2247	0.7071	0.7071	1.5	0.5	1.5	0.5	0.5	0.866	0.7071	0.5	0.5	1	2.1794	1.118	6.5383	1.5	0.5	0.7071	0.5	8.124	1.118	0.5	0.7071	1	1.118	0.7071	0.5	1.4142	0.7071	0.7071	6.3443	1	0.7071	6.3246	0.7071	0.866	0.7071	9.6954	4.062	6.5	0.5	1.118	1.118	1	0.5	0.5	6.5383
419	0.5	0.5	0.7071	0.5	0.7071	0.5	0.5	7.1764	0.5	0.7071	0.5	6.0622	0.5	0.5	0.7071	1.2247	0.5	0.5	6.2849	6.9821	0.5	0.7071	0.866	0.5	0.7071	4.1533	0.5	0.5	0.5	7.2801	0.7071	0.5	7.0887	0.7071	0.5	0.5	7.1764	0.5	0.5	0.5	0.5	8.0467	0.7071	5.3151	0.5	7.4498	0.5	0.7071	0.866	0.5	0.5	0.7071	7.5829	0.7071	0.5
420	0.7071	0.5	0.5	0.5	0.5	0.5	0.5	0.7071	0.5	0.5	0.866	5.8523	0.866	0.7071	6.0208	0.866	5.0249	0.7071	0.5	7.0887	0.7071	0.5	1.118	1.2247	0.5	3.3912	0.5	0.7071	0.7071	0.866	0.5	0.7071	6.5955	1.118	0.5	7.4833	0.5	0.5	0.5	0.5	0.5	7.7782	0.7071	5.5227	0.5	8.3217	7.7942	1.118	0.5	1.118	0.5	0.5	7.5	0.5	0.5
421	0.5	1	1.118	1	0.5	1	1	7.4498	0.7071	0.866	0.7071	0.7071	0.7071	0.866	6.3443	0.5	4.6904	0.866	6.5765	0.7071	0.5	0.5	0.5	0.7071	0.866	0.866	0.7071	0.866	0.5	7.1239	0.866	1.118	0.5	0.5	0.7071	7.2284	7.1239	1	1	0.7071	0.7071	0.5	0.7071	0.5	0.7071	1.5	7.7136	0.5	0.7071	0.7071	1	0.5	0.5	0.866	0.7071
422	-2.7249	-2.0119	-2.2689	-2.02	-3.1404	-2.8054	-2.2901	-2.2065	-2.1907	-2.8033	-2.7569	-1.9953	-2.7927	-2.6716	-2.4865	-2.3396	-2.2046	-2.9869	-2.8206	-2.6223	-2.4147	-2.2521	-2.0877	-2.1438	-2.5724	-2.57	-2.4026	-2.0829	-2.4924	-2.7224	-2.8013	-1.963	-2.9147	-2.5076	-2.3838	-2.5607	-2.6238	-2.4684	-2.47	-2.127	-2.5585	-2.4444	-2.4253	-2.5377	-2.9064	-2.2623	-2.7325	-2.5987	-2.5273	-2.5272	-2.9846	-2.821	-2.6261	-2.9275	-2.6755
423	-2.6953	-2.0012	-2.1556	-2.0116	-3.1328	-2.6705	-2.2823	-2.1648	-2.17	-2.6823	-2.6981	-1.9632	-2.6065	-2.6236	-2.2939	-2.2792	-2.1982	-2.9067	-2.8159	-2.58	-2.4141	-2.2369	-2.0835	-2.025	-2.5443	-2.5262	-2.3958	-2.0796	-2.4663	-2.6943	-2.7652	-1.9588	-2.9072	-2.4568	-2.2846	-2.5499	-2.5695	-2.43	-2.445	-2.1141	-2.5359	-2.3765	-2.4242	-2.4217	-2.8701	-2.2365	-2.7218	-2.5168	-2.5133	-2.5212	-2.929	-2.8124	-2.5467	-2.8383	-2.6391
424	-2.6092	-1.9507	-2.1327	-1.9986	-3.1155	-2.6685	-2.2424	-2.1465	-2.1675	-2.6472	-2.6865	-1.941	-2.5714	-2.603	-2.2863	-2.2514	-2.1957	-2.9031	-2.7781	-2.5655	-2.3579	-2.226	-2.0807	-1.9808	-2.5443	-2.4813	-2.1796	-2.0423	-2.3406	-2.5596	-2.7222	-1.9301	-2.7644	-2.3058	-2.2334	-2.5054	-2.5681	-2.423	-2.4255	-2.0903	-2.3775	-2.3312	-2.4014	-2.376	-2.8451	-2.2306	-2.6729	-2.5126	-2.5074	-2.4891	-2.9167	-2.7695	-2.5406	-2.8076	-2.6355
425	-10.11	-7.2178	-7.5759	-7.4393	-7.8739	-7.699	-7.3885	-8.677	-8.5207	-8.2072	-8.0945	-6.9219	-8.3799	-8.1317	-7.2455	-8.6301	-7.5664	-7.828	-7.1342	-8.5613	-8.7872	-7.4935	-8.6685	-8.6379	-7.0498	-7.4286	-8.2387	-7.2129	-7.369	-8.5348	-7.3192	-7.0113	-7.2579	-7.0159	-8.3007	-7.8522	-8.1539	-9.3595	-7.3102	-7.1297	-8.3795	-7.4672	-8.7148	-7.37	-7.0626	-6.4558	-7.0434	-10.433	-9.7849	-7.8256	-7.0682	-7.7759	-8.9652	-8.0246	-9.9704
426	-10.064	-7.1018	-7.5372	-7.0533	-7.5101	-7.4803	-7.2388	-8.4522	-8.4619	-8.1549	-7.6771	-6.8804	-8.2313	-7.6724	-7.2416	-8.4776	-7.2964	-7.7432	-7.0372	-8.5166	-8.5495	-7.4554	-8.324	-8.0787	-6.9096	-7.3237	-7.4911	-6.9784	-7.184	-7.7685	-7.1645	-6.8602	-7.0235	-6.8647	-7.7153	-7.6598	-7.9081	-9.0507	-6.9068	-6.9879	-7.7945	-7.2809	-8.4231	-7.3627	-7.0279	-6.4435	-7.0026	-10.053	-9.7624	-7.5833	-6.9843	-7.7118	-8.4802	-7.95	-9.4355
427	-9.5048	-6.7792	-7.2839	-6.946	-7.3197	-7.4722	-6.8786	-8.4368	-8.3558	-8.0075	-7.6021	-6.7107	-8.2099	-7.6537	-7.116	-8.2515	-7.1836	-7.5882	-6.9243	-8.467	-8.4927	-7.4424	-8.2058	-7.7749	-6.8815	-7.3224	-7.4392	-6.7974	-7.0587	-7.7413	-7.1105	-6.6092	-6.9999	-6.7861	-7.507	-7.6557	-7.7253	-7.9048	-6.9041	-6.9361	-7.5486	-7.1149	-8.3231	-7.2104	-6.9887	-6.4366	-6.9726	-9.6838	-9.4595	-7.4609	-6.8897	-7.4036	-8.1176	-7.3996	-9.2309
428	1.2605	1.1976	1.2068	1.2157	1.2736	1.2425	1.2152	1.1951	1.2054	1.2795	1.2652	1.1877	1.2678	1.2553	1.257	1.2772	1.2791	1.2461	1.1997	1.2437	1.2695	1.2212	1.2408	1.2083	1.2445	1.2753	1.2058	1.207	1.2044	1.2827	1.2572	1.1768	1.2721	1.2133	1.2816	1.266	1.2936	1.2584	1.1801	1.2713	1.2588	1.2032	1.2308	1.2337	1.2771	1.2424	1.2633	1.2773	1.2888	1.3114	1.3066	1.2726	1.2715	1.2925	1.3499
429	197.88	372.88	312.38	249.25	267.75	277.75	284.88	226.25	336.13	384.5	296.38	312.75	283	264.63	232	297.13	268.88	321.13	277	313.63	176.5	328.5	300.63	271.88	279	312.5	244.38	216.88	344.25	385.63	222.88	266	267.25	227.25	217	254.63	240.5	285	249	285.88	154.38	282	260.38	244.75	227.38	319.63	286.38	329.63	361	362	243.13	312.88	306.5	241.25	270.38
430	239.5	478.13	439.13	322	336.38	379.25	351.38	346.63	465.38	527	351.38	398.63	397.88	341.5	316.25	384.88	357	425.5	375.38	392.88	223.63	464.38	438.25	353.38	360.38	409.63	336.75	313.25	467.75	511.63	286.63	310	332	284.88	265.63	288.75	291.88	351.75	332.63	382.13	192.38	378.13	345.13	293	279.13	404	341.38	391.75	490.88	503.63	303	363.75	383.25	288.25	326.13
431	250.5	380.88	356.13	267.25	299.63	320.88	280.25	281.75	415.63	452	288.88	295.63	321.63	305.13	259.38	299.25	292.75	381.38	295.75	331.5	222.13	376.63	343.13	262	290.63	331.13	234.25	239.75	376.5	416.75	217.5	225	266.5	217.13	214.75	239.13	237.63	308.88	248	315.25	171	295.75	279.25	237.25	213.63	304.5	243.38	287	392.5	400.13	233.88	257.75	290.13	219	264.63
432	148.63	202.38	200.5	156.88	165.38	161.5	148.63	149.38	235.5	255.75	168.13	158.13	189.63	165.25	138.25	140.25	161.75	219.25	146.63	207	140.38	204.88	189.88	131.63	154.13	156.75	104.88	95.75	199.75	218.63	114.13	114.63	146.63	107	106.13	127	125.38	160.5	112.38	163.63	106.5	152.88	147.38	128.13	102.75	136.5	117.88	133.75	210.88	198	110.88	126.25	159	107.88	130.25
433	79.625	94.25	93.25	78.375	84.625	80.875	68	71.5	128.25	134.25	86.25	71	93.75	83.25	63	58.875	77.375	108.5	71.375	109.5	78.5	95.125	88.5	62.125	74.625	74.375	41.375	40.875	94.125	107.5	55	48.5	75.875	48.625	48	59.75	62.375	75.5	51.875	75	53.5	72.75	70.25	65.875	42.875	56.875	56.375	67.125	109	85.75	50.625	56.625	84.375	49.375	66.25
434	43.75	32.625	38.75	38.375	42	33.5	26.25	35.25	63.125	63.375	40	26.875	43.125	33.375	25.75	21.75	29.875	44.125	33.25	51.625	42.125	41.875	36.5	25.625	32.875	34.75	15.625	16	41.75	47.125	26.25	16.25	40.375	22	22.5	27.125	28.75	37	19.75	29.75	27.25	30.625	34.25	33.125	19.125	21.25	27	33.5	57	36.875	22.25	25	43.125	23.75	37.875
435	21.75	8.625	11	14.375	15.875	13.5	7.125	15.75	26.25	21.625	13.75	8.625	16.875	12.625	9.375	8.125	9.875	18.5	11.75	21.125	20.75	14.5	13.25	10.625	10.75	15.375	3.875	6.375	14.625	18.25	10.625	5.375	18.75	9	9.75	10.25	10.875	13.5	6.875	9.5	11.25	11.5	17.625	13.75	5.75	5.875	11.375	16.625	27.375	14.375	8.25	7.625	23.375	11.125	19.125
436	11.375	2	3	3.875	4.75	3.625	1.75	5.75	9.875	6.125	5.25	3.125	6.625	4.375	2.875	3.375	3.625	6.125	3.375	8.75	9.625	4.25	5.5	3.75	2.5	5.875	1.25	1.875	3.75	4.875	1.875	1.625	5.375	3.875	2.875	3.25	3.5	3.75	2	2	3.5	3.625	7.375	3.875	1.625	1.375	3.75	8.125	11.875	3.5	2.375	2.875	8.75	3.625	9.25
437	0.5271	1.6011	0.9302	0.6993	0.3749	0.4129	0.5617	0.6227	1.0609	0.9895	0.4081	0.8649	0.5873	0.449	0.4776	0.3745	0.4072	0.9446	0.4973	0.8577	0.5126	1.1653	0.9078	0.4684	0.3607	0.3668	0.401	0.2815	0.7065	0.7239	0.251	0.4336	0.4372	0.285	0.3321	0.3204	0.2967	0.4943	0.3073	0.5611	0.4195	0.5498	0.5062	0.4551	0.1861	0.3304	0.2537	0.2762	0.528	0.364	0.1792	0.2628	0.3866	0.2019	0.2677
438	0.324	0.7337	0.452	0.368	0.204	0.202	0.2549	0.36	0.6917	0.5746	0.2128	0.3845	0.3205	0.2042	0.2172	0.1616	0.1701	0.4673	0.2688	0.4562	0.2993	0.6342	0.4381	0.2096	0.1785	0.188	0.1893	0.1292	0.3643	0.3775	0.1248	0.1591	0.2491	0.1304	0.1599	0.1508	0.1413	0.2815	0.1261	0.252	0.2328	0.2544	0.2618	0.2331	0.0813	0.1492	0.1288	0.1567	0.3227	0.1792	0.0818	0.1216	0.2129	0.1027	0.1522
439	1.5382	0.6143	1.7848	2.3618	2.3982	1.9114	0.8134	0.7832	1.5169	1.1816	1.5919	1.2595	1.7606	1.2729	1.5052	1.5716	2.246	1.5074	1.4467	0.2944	1.4421	0.7559	0.3586	1.6824	1.3088	1.431	0.837	1.2115	1.6609	1.415	1.716	1.9139	1.5618	0.4385	1.3036	0.5677	0.5369	1.0362	1.5086	1.7736	0.7591	1.7656	0.994	0.6769	1.5196	0.7736	2.1549	1.8414	2.2208	2.0441	1.286	1.8002	1.9967	0.7924	1.5702
440	1.5714	0.5089	1.8299	2.238	2.4215	1.9825	0.8052	0.6782	1.447	1.0869	1.5972	1.2417	1.8316	1.2313	1.4365	1.5481	2.1806	1.4268	1.3793	0.3584	1.5479	0.6696	0.405	1.6722	1.3598	1.5322	0.7721	1.2173	1.6847	1.3656	1.7544	1.8435	1.5596	0.4123	1.2706	0.5569	0.5352	1.0126	1.3772	1.6831	0.7302	1.7303	0.9528	0.6544	1.543	0.862	2.0599	1.8976	2.2993	2.1013	1.2691	1.6732	1.9953	0.7769	1.4353
441	1.6627	0.4545	2.158	2.4377	2.3211	2.0159	1.0334	0.5862	1.5213	1.136	1.3927	1.3766	1.7725	1.3681	1.3503	1.8355	2.2914	1.3997	1.4338	0.5492	1.5315	0.8771	0.5289	1.9107	1.2145	1.4675	1.0098	1.4294	1.9628	1.7123	1.462	2.1972	1.5643	0.6077	1.3437	0.6208	0.3334	1.4384	1.5848	2.0017	0.7771	2.0204	1.0985	0.6792	1.3646	0.8158	1.9919	1.9053	2.4147	2.21	1.3441	1.6682	2.0739	0.687	1.3342
442	1.9018	0.5032	2.3573	2.8184	2.5304	2.0563	1.347	0.6258	1.6044	1.2588	1.5017	1.5098	1.9566	1.7194	1.3916	2.2127	2.585	1.5039	1.581	0.7737	1.5806	1.0265	0.5681	2.2919	1.3522	1.422	1.3886	1.9909	2.4244	2.1114	1.537	2.6051	1.6812	0.7695	1.5618	0.7021	0.3928	2.0305	1.8568	2.4266	0.9721	2.492	1.3528	0.6851	1.5266	1.0281	2.2469	2.2204	2.7771	2.4836	1.5096	1.9114	2.3361	0.7606	1.5887
443	2.0709	0.8988	2.4825	3.2157	2.8417	2.1572	1.42	0.7052	1.5997	1.4065	1.7069	1.5671	2.2344	2.0281	1.5259	2.6207	2.8275	1.6568	1.8999	0.8302	1.8547	1.1865	0.5138	2.5502	1.5756	1.484	1.4991	2.6013	2.7189	2.3549	1.8301	2.8764	1.7924	0.8341	1.8595	0.731	0.5158	2.2752	2.0444	2.7263	1.0553	2.7913	1.6631	0.7251	1.8033	1.1062	2.4677	2.5087	3.0729	2.771	1.6421	2.0861	2.5506	0.746	1.9201
444	2.178	1.4636	2.3141	3.1268	3.0468	2.2509	0.9726	1.3746	1.4674	1.3411	2.4788	1.6575	2.8031	1.743	1.6993	2.4395	3.048	1.7878	2.2219	0.4209	2.3275	1.1069	0.6267	2.4982	2.1374	2.0894	1.3367	2.4135	2.4576	1.811	2.6326	2.5186	1.8118	0.6093	1.9234	0.8419	0.9285	1.7096	2.1006	2.2387	1.1804	2.3398	1.7991	0.7657	2.4612	0.9021	2.8819	2.802	3.2923	3.0781	1.7806	2.1632	2.6906	1.2655	2.2096
445	2.0627	3.4697	1.5085	2.6423	3.1179	2.4398	0.7814	2.0377	1.1498	1.62	2.5738	2.077	2.9662	1.2623	1.8527	1.9364	2.8083	2.269	1.9026	0.6591	2.4884	2.2166	1.5881	2.0156	2.4055	2.5574	1.6713	1.9431	1.29	0.723	3.2268	1.7457	2.756	0.7313	2.0494	0.9528	1.5974	0.7212	1.3522	1.691	2.0965	1.4484	1.7076	1.4318	2.9668	2.5997	2.5566	2.4848	2.6246	2.5567	1.4876	1.6573	2.2946	2.0641	1.8731
446	1.6816	4.8401	1.1491	3.0983	2.6703	2.3145	1.2091	2.5094	1.4456	1.5285	2.2893	2.3483	2.7157	0.7424	1.5329	2.0754	1.5864	2.1272	1.3294	1.2517	2.3583	2.7437	3.5459	2.2076	2.3141	2.1927	2.5486	2.1187	1.3196	0.6084	2.7366	1.9179	2.7989	1.7584	2.3814	1.2966	1.5716	1.8001	0.6198	2.2795	2.2817	1.3019	1.9363	2.1729	2.7126	3.4588	1.9508	2.4151	2.2327	2.0822	1.175	1.2512	2.0682	2.2986	1.6122
447	0.1429	0.1003	0.4108	0.6286	0.3455	0.1362	0.2906	0.2573	0.2456	0.3517	0.2259	0.4751	0.1906	0.2219	0.4744	0.438	0.4525	0.3693	0.4431	0.328	0.3372	0.3614	0.3207	0.5328	0.3173	0.1329	0.2593	0.3677	0.5067	0.412	0.1376	0.6836	0.2205	0.1181	0.6085	0.2014	0.2471	0.5493	0.5853	0.7561	0.5621	0.585	0.3552	0.1502	0.3505	0.1998	0.3597	0.3387	0.3377	0.673	0.0749	0.4337	0.3297	0.3131	0.7103
448	0.5387	0.0694	0.8242	1.4289	1.2211	0.5887	0.5497	0.3727	0.5878	0.6238	0.8739	0.6923	0.5873	0.8613	0.7078	1.2324	1.5262	0.6114	1.0095	0.3877	0.7111	0.4972	0.3709	1.3542	0.6147	0.4825	0.7463	0.9598	1.1743	0.8801	0.8714	1.5239	0.7197	0.4139	1.155	0.449	0.4761	1.2332	1.6132	1.4534	1.0116	1.3113	0.8267	0.4338	1.052	0.6152	1.3102	1.1871	1.1346	1.6303	0.8022	1.1068	1.1086	0.8053	1.4129
449	1.1774	0.1114	1.1023	1.641	2.0419	1.4379	0.7176	0.6227	0.7273	0.7305	1.486	0.9669	1.1246	1.0438	1.0324	1.4027	2.1224	0.8232	1.4313	0.3564	1.2283	0.5365	0.4783	1.6582	1.0356	1.1	0.9223	1.2822	1.3717	1.0633	1.6998	1.8316	1.2322	0.6057	1.2873	0.6051	0.8101	1.4597	2.1361	1.7066	1.3167	1.615	0.977	0.6026	1.8135	0.7983	2.2503	2.1133	1.8468	2.4807	1.6771	1.8612	1.8661	0.7839	1.7987
450	1.7489	0.189	1.3792	1.7265	2.7009	2.3823	0.8331	1.0417	0.9505	0.9253	1.5683	1.3004	1.5623	1.1589	1.7787	1.5885	2.5674	1.0622	1.9742	0.4295	1.7439	0.4554	0.5459	1.9097	1.412	1.7542	1.137	2.0739	1.5291	1.1518	2.1363	2.1183	1.6346	0.8401	1.5687	0.7038	1.0247	1.6516	2.4339	1.9149	1.702	1.8864	1.0686	0.7692	2.6245	1.1697	3.0198	3.0944	2.3926	2.8727	2.3897	2.4233	2.4919	0.8239	2.2562
451	1.9544	0.3687	1.6465	1.6522	2.9042	3.161	0.9849	1.3948	1.1722	1.0346	1.6136	1.781	1.8839	1.3102	2.8544	2.0427	2.836	1.3079	2.5437	0.6051	2.0941	0.5304	0.767	2.295	1.4876	2.1287	1.2981	2.7087	1.5401	1.0552	2.2132	2.5363	1.8461	1.1678	2.0619	0.8333	1.1313	1.8767	2.698	2.13	2.0105	2.1031	1.2351	0.9686	3.3601	1.4299	3.1482	3.6186	2.7213	2.8962	3.0054	2.5082	2.8381	1.0702	2.6216
452	2.2691	0.8526	2.0944	1.5884	2.9489	4.0091	1.3596	1.5234	1.4114	1.2327	1.7036	2.6153	2.3422	1.5487	3.8733	3.0489	3.3812	1.9332	3.2088	0.9402	2.5282	0.4502	1.1342	2.98	1.6083	3.2567	1.8215	3.5205	1.8249	1.0142	2.3248	3.4859	2.126	1.6476	2.7199	0.9034	1.3737	2.3146	3.2483	2.6762	2.3304	2.3497	1.614	1.5451	4.2743	2.2698	3.6082	4.1441	3.1356	3.1358	3.701	2.7275	3.1471	1.3148	3.5266
453	2.6954	1.7355	3.5776	1.2928	2.9687	4.4049	1.6175	1.1873	1.8509	1.167	1.6339	3.7281	2.7572	2.0067	4.5321	4.2777	4.5509	2.5814	3.4266	1.3923	3.1134	0.6427	1.9654	3.7821	1.6668	3.643	2.3718	4.5016	2.3068	0.8299	2.8619	4.9125	2.238	1.8688	3.4529	1.5704	1.8718	2.5017	4.6276	3.5085	2.5337	2.4569	1.9441	1.9661	4.8093	3.2093	4.2279	4.7744	3.2607	3.5111	4.7205	2.7429	3.0497	1.9892	4.3249
454	2.7142	2.7329	3.9793	1.7343	3.9772	4.3434	2.4084	1.4529	2.4209	1.6449	2.0688	3.676	2.5378	1.8365	4.6408	4.1297	4.8099	2.469	3.6655	2.0154	3.2539	1.5738	2.2231	3.7577	1.4189	3.5907	2.4536	4.6629	3.0165	0.8061	4.0293	4.9966	2.1983	2.5123	3.9159	1.2401	1.7328	2.723	5.1019	4.2595	2.831	2.4155	2.3195	2.5417	5.3832	3.1821	4.5866	4.9812	3.4444	3.2056	5.9333	3.3978	2.3774	2.7037	4.618
455	1.3358	1.246	1.2801	1.309	1.3813	1.3614	1.28	1.3205	1.3194	1.2974	1.3693	1.2522	1.3206	1.334	1.3372	1.3111	1.3233	1.2873	1.3192	1.3245	1.3591	1.2731	1.2836	1.3518	1.4278	1.3623	1.31	1.3356	1.347	1.3138	1.3986	1.2936	1.3483	1.3911	1.3452	1.3463	1.3337	1.3093	1.3687	1.325	1.3874	1.3118	1.32	1.3462	1.4118	1.416	1.3803	1.397	1.3893	1.3915	1.4469	1.3534	1.4553	1.4203	1.3909
456	404.24	301.57	325.76	348.18	445.78	402.08	332.52	336.62	344.85	398.79	429.51	297.09	401.91	398.14	401.67	405.1	414.41	362.59	339.88	381.74	427.48	333.84	355.94	364.53	444.4	435.39	340.27	354.72	358.49	411.96	439.64	308.43	423.23	390.87	430.81	415.95	435.53	386.36	348.23	408.02	434.26	339.03	367.42	384.88	469.61	434.85	434.4	459.92	467.24	493.89	528.12	426.9	492.42	492.66	538.24
457	540	375.75	417	455.75	615.75	547.38	425.63	444.5	455	517.38	588.13	372	530.75	531.13	537.13	531.13	548.38	466.75	448.38	505.63	581	425	456.88	492.75	634.5	593.13	445.75	473.75	482.88	541.25	614.88	399	570.63	543.75	579.5	560	580.88	505.88	476.63	540.63	602.5	444.75	485	518.13	663	615.75	599.63	642.5	649.13	687.25	764.13	577.75	716.63	699.75	748.63
458	1251.4	1009.5	1010.4	983.25	1313	1225.5	966.63	989.63	1088.4	1208.1	1152.6	935	1168.6	1047.6	1181.3	1065	1096	1140.4	950.63	1151.4	1232.8	1054.4	1038	978.63	1245	1224.6	911.5	957.63	1066.9	1203.6	1152.1	888.75	1158.4	986.75	1191.5	1011.6	1079.3	1114.3	935	1169.1	1220	997.75	990	959.5	1261.6	1169.5	1224.1	1306.1	1376.8	1477.8	1459.8	1249.9	1401.8	1243	1545
459	883.88	776.88	743.25	664.38	875.63	846.13	675.25	705.38	811.5	897.13	737.5	692.75	817.88	688.25	845.75	692	703.63	856	644.75	829	855	802.5	767.25	638.75	831.25	822.13	602.88	634.75	742.75	863.25	691	588.5	762.88	597.38	802.25	604.63	647.13	754	575.5	813.5	803	684.63	662.75	597	759.38	758.75	789.88	841	953	995.5	863.88	815	933.25	732.88	1025.3
460	526.13	491.75	475.88	408.63	500.25	482	406.5	426	525.38	580.13	421.75	419.63	484.38	425.63	490	420	418.5	545.75	383.5	523.63	504	513.63	484	378.38	466.13	469.13	350.38	361.38	467.13	546.88	367.25	342.63	437.25	347.63	448.88	362.38	367.88	461	323.88	500.13	461.88	418.5	403	368.25	402.63	432.5	423.63	455	570.5	577.88	437.88	437.13	524.75	389.75	546.38
461	229.63	235.88	234.75	198.63	212.75	202.25	181.13	192.38	260	287.13	198.75	196.75	232.63	191.63	203.63	179.13	198.88	263.13	170.88	261.75	224.5	242.75	232.5	167.88	199.13	198.5	150.38	132.25	223.13	254.63	157.5	150.75	199.63	145.38	177.38	162.75	163	206.25	133.88	226.25	207.13	186.75	182.25	170.25	154.38	180.5	168.38	175.88	262.13	247	171.13	172.38	229.38	153.88	207.88
462	108.13	105.88	103.25	91.875	98.125	91.25	78.5	85.625	132.75	141.75	93.75	84	107.63	88.625	82.5	74.25	88.875	121.38	79.125	126.63	107.13	107.5	102.13	74.125	87.375	86.75	60.5	52.5	102	118.63	68.75	60.375	93.125	60.625	68.75	70.375	73.875	92	59	97	96.375	84.875	82	79.75	59.375	74.25	73.625	80.375	125	103.25	69.375	70.125	108.88	65.75	89.125
463	52	35.125	40.625	40.25	43.875	35.375	27.5	37.625	63.375	64	40.75	29.125	45.875	33.875	29	26.625	31.875	46.5	34.875	54	50.5	44	38.5	27.75	35.375	35.75	23.375	18.375	43.625	49.125	28.875	18.75	44.25	24	26.125	29	30.5	41.875	21	35.75	43.5	32.875	36.125	36.125	21.625	27.5	30.375	36.75	60.875	41.125	26.375	26.5	49.25	28.5	40.625
464	23.375	8.625	11	14.375	15.875	13.5	7.125	15.75	26.25	21.625	13.75	8.625	16.875	12.625	9.375	9.5	9.875	18.5	11.875	21.125	22.5	14.5	13.25	10.875	10.75	15.375	7.5	6.375	14.625	18.25	10.625	5.375	19.25	9	9.75	10.25	10.875	14.75	6.875	9.5	15.75	11.5	17.625	13.75	5.75	8	11.625	17	27.5	14.375	8.25	7.625	23.75	11.375	19.125
465	11.375	2	3	3.875	4.75	3.625	1.75	5.75	9.875	6.125	5.25	3.125	6.625	4.375	2.875	3.375	3.625	6.125	3.375	8.75	9.625	4.25	5.5	3.75	2.5	5.875	3	1.875	3.75	4.875	1.875	1.625	5.375	3.875	2.875	3.25	3.5	4.125	2	2	4	3.625	7.375	3.875	1.625	1.625	3.75	8.125	11.875	3.5	2.375	2.875	8.75	3.625	9.25
466	1053.5	636.63	698	734	1045.3	947.75	681.75	763.38	752.25	823.63	856.25	622.25	885.63	783	949.25	767.88	827.13	819.25	673.63	837.75	1056.3	725.88	737.38	706.75	966	912.13	667.13	740.75	722.63	818	929.25	622.75	891.13	759.5	974.5	757	838.75	829.25	686	883.25	1065.6	715.75	729.63	714.75	1034.3	849.88	937.75	976.5	1015.8	1115.8	1216.6	937	1095.3	1001.8	1274.6
467	644.38	298.75	304.13	342.38	539.25	466.88	323.88	358.75	346.13	370.13	386.13	294.13	420	346.75	529.5	307.13	346.63	430.5	269.38	436.13	631.38	338.13	329	285.38	470.88	412.5	266.13	321.5	275	351.63	404.38	278.5	430.88	312.5	536.63	315.88	355.25	402.25	242.88	431.38	610.63	306.5	317.63	304	480.25	354.75	448.5	449.25	462.13	491.88	560.88	451.25	550	444.63	699.13
468	275.63	110.88	119.75	141.38	200.63	161.13	126.25	144.25	109.75	128.13	132.88	124	162.75	120.5	230.63	120.75	125.75	164.38	87.75	192.13	281.88	137	140.88	116.38	175.5	138	116.13	121.63	90.625	130.13	149.75	117.63	170.75	130.5	234.13	123.25	130.25	152.13	75.875	184.88	290.88	122.75	123.75	131	189	128	180.25	168	178	177.75	204	179.38	234.63	170.75	281.75
469	81	33.5	34.25	41.75	47.375	40.75	32.5	43	24.5	31.375	30.625	38.625	43	26.375	65.375	38.875	37.125	43.875	24.25	54.75	84.125	37.875	42.625	36.25	45	41.75	45.5	36.5	23.375	36	43.375	36.125	53	38.375	71.25	35.75	37.625	45.75	21.5	62.625	100.63	33.875	34.875	42.125	51.625	44	50.5	42.125	51.25	49	60.25	46.125	70.375	46	77.625
470	28.5	11.625	10	13.5	13.5	10.375	10.5	14.125	4.5	7.5	7.5	13	13.875	5.375	19.5	15.375	11.5	12.875	7.75	17.125	28.625	12.375	13.625	12	12.75	12.375	19.125	11.625	7.875	11.125	13.75	11.875	17.25	12	20.75	10.625	11.5	16.5	7.125	22	42.875	12.125	11.75	13.875	16.5	17.375	17.25	13.25	16	17.5	18.75	13.5	24.5	16.375	22.875
471	8.25	2.5	1.875	1.875	1.875	1.875	1.25	2.375	0.25	0.625	0.75	2.25	2.75	0.5	3.25	4.875	2	2.375	1.625	2.375	8.375	2.125	2	2.125	2.5	1	7.75	2.375	1.875	2	2.625	2.5	3.875	2	3.625	1.875	1.75	4.875	1.25	6	16.25	2.25	1.875	3	2.5	6.25	3.375	3.25	3.875	4.25	4.125	1.5	6.125	4.75	2.75
472	1.625	0	0	0	0	0	0	0	0	0	0	0	0	0	0	1.375	0	0	0.125	0	1.75	0	0	0.25	0	0	3.625	0	0	0	0	0	0.5	0	0	0	0	1.25	0	0	4.5	0	0	0	0	2.125	0.25	0.375	0.125	0	0	0	0.375	0.25	0
473	0	0	0	0	0	0	0	0	0	0	0	0	0	0	0	0	0	0	0	0	0	0	0	0	0	0	1.75	0	0	0	0	0	0	0	0	0	0	0.375	0	0	0.5	0	0	0	0	0.25	0	0	0	0	0	0	0	0	0

Table S12 Amino acid descriptors (2D) used in this study.

	W	W	W	W	W	W	W	W	W	W	W	Y	Y	Y	Y	Y	Y	Y	Y	Y	Y	Y	Y	Y	Y	Y	Y	Y	Y	Y	Y
	L	M	N	P	Q	R	S	T	V	W	Y	A	C	D	E	F	G	H	I	K	L	M	N	P	Q	R	S	T	V	W	Y
No.	WL	WM	WN	WP	WQ	WR	WS	WT	WV	WW	WY	YA	YC	YD	YE	YF	YG	YH	YI	YK	YL	YM	YN	YP	YQ	YR	YS	YT	YV	YW	YY
1	0	3	1	-4	-6	-2	7	-4	-5	-3	-4	-2	-2	-1	-5	-3	-1	-3	-1	-2	-2	-3	-2	-3	-3	-4	-3	-4	-3	-4	6
2	0.1	3.2	0.8	-2.1	-4.9	-1.7	6.1	-3.7	-4.6	-2.2	-3.7	0.9	-0.9	-0.4	-2.8	-3.6	4.4	-3.8	0.1	-2.5	-3.8	-4.2	0.1	-3.8	-1.7	-3.4	-4.1	-3.4	-2.2	-3.7	4.4
3	0.3	2.4	0.6	-2	-4.8	-1.8	6.1	-3.2	-4.9	-2.1	-3.2	0.8	-0.5	-0.4	-2.7	-2.4	3.7	-3	-0.4	-2.7	-3.2	-4	0.3	-2.7	-1.1	-2.4	-3.6	-3.8	-2.1	-3.2	4.2
4	0.4	1.4	0.4	-1.6	-4.3	-1.7	6.1	-2.3	-4.6	-1.9	-2.3	0.6	-0.3	-1	-1	-1.2	2.5	-2.1	-0.8	-2.8	-2.6	-3.9	0	-2	-0.5	-1.5	-2.9	-4.3	-1.9	-2.3	4
5	1.7	3.6	-0.6	-2.2	-1.8	-1.8	4.7	-1.8	-2.2	-0.1	-1.8	0.6	-1.6	0.7	-2.1	-1.9	2.3	-2.1	-1.8	0.9	-1.2	-2.1	-1.1	-1.9	-1.8	-1.8	-2.3	-2.5	-0.1	-1.8	4.1
6	0.22	0.05	0.29	0.18	-0.24	0.25	1	-0.14	-0.14	-0.12	-0.14	-0.09	0.14	0.05	-0.11	0.26	0.08	-0.18	-0.1	-0.37	-0.29	-0.54	-0.06	-0.43	0.08	-0.19	-0.51	-0.22	-0.12	-0.14	1
7	1	3	1	-2	-5	-1	6	-2	-4	-2	-2	0	-1	0	-3	-2	0	-2	-1	-2	-2	-2	-2	-2	-1	-2	-2	-3	-2	-2	5
8	3	4	5	1	2	3	6	1	2	3	1	3	3	3	1	2	3	1	2	2	2	1	2	1	2	1	1	2	3	1	6
9	1	1	1	2	2	2	0	2	1	1	2	2	2	1	3	2	1	2	2	1	2	2	2	3	2	3	3	2	1	2	0
10	5	9	10	9	6	8	10	8	7	8	8	5	9	9	5	9	7	8	8	7	3	3	7	7	10	9	3	7	8	8	10
11	0.4	1.2	0.4	-1.4	-4	-1.6	6	-2.2	-4.4	-1.9	-2.2	0.6	-0.2	-1.1	-0.8	-1.1	2.2	-2	-0.8	-2.8	-2.4	-3.8	-0.1	-1.9	-0.3	-1.3	-2.7	-4.5	-1.9	-2.2	4
12	81	24	56	98	147	42	0	94	138	99	94	32	89	77	115	198	83	84	94	149	97	168	100	109	47	87	134	135	99	94	0
13	0	1	0	-2	-3	-2	10	-3	-3	-2	-3	-1	-1	-2	-3	-3	2	-3	-1	-2	-3	-4	-2	-2	-2	0	-3	-4	-2	-3	5
14	-2	1	0	-3	-5	-3	11	-5	-5	-4	-5	-1	-1	-3	-4	-2	3	-5	-2	-5	-4	-5	-2	-4	-3	-2	-5	-6	-4	-5	6
15	-2	1	-1	-4	-6	-5	12	-6	-7	-5	-6	-1	-2	-4	-4	-2	3	-5	-3	-6	-5	-7	-2	-5	-3	-4	-6	-7	-5	-6	7
16	-0.7	1.4	-1.3	-3.2	-5.4	-2.5	12.7	-5.1	-7.2	-4.2	-5.1	0.1	-2.6	-4.3	-4	-7.7	-0.4	-3.9	-2.2	-4.7	-5.4	-4.8	-1.7	-7	-3	-2.3	-4.8	-5.5	-4.2	-5.1	8.1
17	0	2	1	-2	-4	-1	6	-3	-4	-2	-3	1	-1	0	-3	-2	4	-3	0	-2	-3	-3	0	-3	-1	-2	-3	-3	-2	-3	4
18	3	7	1	-3	-5	-2	11	-4	-4	-4	-4	4	-2	-4	-1	-1	6	-4	0	-3	-3	-3	-3	-4	-2	-3	-4	-2	-4	-4	2
19	6.6	5	0	0	0	0	10	0	0	0	0	0	9	0	5	0	6.6	0	0	0	0	0	5	0	5	0	0	0	0	0	10
20	0	0	0	0	-1	0	2	-1	-1	-1	-1	0	0	0	-1	0	0	-1	0	-1	-1	-1	-1	-1	0	-1	-1	-1	-1	-1	2
21	-5	2	-6	-13	-16	-19	54	-10	-17	-6	-10	-8	-4	-12	-3	-13	2	-9	1	-3	-4	-7	-6	-3	-5	-3	-3	-16	-6	-10	50
22	5	4	7	-14	-16	-11	65	-10	-32	-18	-10	6	-1	-23	-21	-17	5	-6	-1	-3	-4	-6	-10	-4	-5	-5	-5	-20	-18	-10	31
23	1	2	5	0	-14	0	43	-4	-15	-12	-4	-6	0	-7	7	4	-1	-8	-5	-6	-3	-9	-9	-4	1	-5	-10	-5	-12	-4	41
24	7	13	7	-32	-17	6	95	-17	-21	-10	-17	-9	-19	-14	-41	3	23	-27	-2	0	-1	-13	-7	0	-16	-8	-2	-6	-10	-17	17
25	12	4	0	-4	-14	-10	84	-13	-21	-9	-13	-19	-11	-9	-36	-26	18	-19	-2	-8	-2	-10	-3	-7	-19	-11	-7	-8	-9	-13	29
26	24	13	16	-3	-9	-14	18	-15	-9	-15	-15	14	10	-15	18	-29	20	-19	-8	-14	-14	-22	6	-14	5	3	-18	-9	-15	-15	20
27	-3	2	-4	-12	-16	-17	55	-12	-22	-10	-12	-6	-3	-12	-7	-13	3	-9	-1	-4	-6	-9	-9	-4	-5	-4	-5	-18	-10	-12	49
28	3	4	6	-1	-14	3	52	-9	-19	-14	-9	-2	0	-4	-17	5	4	-14	-6	-7	-6	-12	-13	-6	0	-7	-10	-8	-14	-9	38
29	17	8	8	-5	-13	-13	55	-15	-15	-13	-15	-1	3	-10	7	-30	21	-18	-7	-12	-9	-17	3	-13	-4	-3	-13	-11	-13	-15	28
30	4	4	3	2	0	3	8	2	1	2	2	4	3	3	3	1	4	2	2	1	1	0	4	0	4	3	1	1	2	2	8
31	0	2	0	0	0	0	6	0	0	0	0	0	0	0	1	0	2	0	0	0	0	0	2	0	0	0	0	0	0	0	5
32	0.18	0.37	0	-0.19	-0.35	-0.14	0.54	-0.69	-0.56	-0.5	-0.69	0.14	-0.15	0.14	-0.7	-0.13	0.35	-0.59	-0.45	-0.83	-0.82	-0.78	-0.43	-0.95	-0.13	-0.86	-0.86	-0.58	-0.5	-0.69	0.75
33	1.72	0.32	0.85	2.78	4.08	0.97	0	2.45	3.67	2.59	2.45	0.79	1.94	2.15	3.16	1.63	2.27	2.11	2.69	3.37	2.49	3.98	2.63	2.57	1.32	2.19	3.39	3.6	2.59	2.45	0
34	9	11	11	7	10	11	16	8	6	10	8	11	10	5	10	8	9	9	9	5	4	3	9	6	10	10	4	6	10	8	16
35	-0.01	0.08	0.28	0.06	0.19	0.1	1	0.01	-0.54	-0.09	0.01	-0.23	-0.05	-0.32	-0.01	-0.15	0.13	-0.11	-0.25	-0.06	0.05	-0.28	0.17	-0.13	0.1	-0.08	-0.14	-0.23	-0.09	0.01	1
36	0	0.12	0.28	0.09	0.2	0.09	1	0	-0.56	-0.13	0	-0.26	-0.02	-0.34	-0.02	-0.16	0.14	-0.13	-0.26	-0.07	0.06	-0.29	0.2	-0.15	0.09	-0.06	-0.13	-0.24	-0.13	0	1
37	0	0	-1	-3	-3	-2	8	-5	-5	-3	-5	0	-2	-3	-3	-4	0	-2	-2	-3	-3	-3	-2	-5	-2	-2	-3	-5	-3	-5	6
38	-0.117	0.63	0.015	0.497	0.372	-0.023	1	0.406	0.327	0.457	0.406	0.072	0.144	0.304	0.232	-0.029	0.083	0.534	0.476	0.452	0.305	0.242	0.405	0.372	0.259	0.461	0.212	0.429	0.457	0.406	1
39	0.067	0.614	0	0	0	0	1	0.378	0	0.289	0.378	0.488	0.568	0.651	0.46	0.717	0.492	0.284	0.888	-0.101	0.108	-0.185	0.511	0.511	0.399	0.441	-0.135	0	0.289	0.378	1
40	0	4	1	0	0	0	8	3	1	2	3	3	3	4	3	4	3	2	5	0	0	0	4	2	2	3	0	0	2	3	8
41	-1.3	-0.5	0.7	-1.2	-0.2	-1.2	2.2	-0.8	-0.2	-0.9	-0.8	-1	-0.4	-1.6	-1.7	-1.6	-0.8	-0.7	1.7	0.9	1.4	0	-1.1	1.4	-0.9	-1.2	1.5	0	-0.9	-0.8	2.2
42	10	-4	9	-8	-18	4	100	3	-16	-9	3	-4	-3	-2	-6	12	12	-10	-17	-32	-25	-39	-10	-20	-7	-8	-36	-21	-9	3	100
43	8	6	8	6	-2	13	7	-4	-9	-5	-4	3	3	4	3	2	0	-4	-6	-11	-11	-14	-1	-12	4	-1	-11	-10	-5	-4	10
44	4	2	6	5	-1	10	5	-3	-8	-4	-3	2	2	3	-1	4	0	-3	-3	-11	-10	-12	-1	-12	3	-2	-10	-8	-4	-3	11
45	61	93	98	75	98	93	100	62	89	64	62	64	78	83	69	93	77	65	91	51	4	23	58	55	74	60	29	87	64	62	100
46	-1	0	0	-1	-2	0	4	-1	-1	-1	-1	0	-1	0	0	0	0	-1	0	-1	-2	-4	0	0	-1	0	-4	-2	-1	-1	4
47	0	0	0	1	1	2	-4	2	3	2	2	1	0	2	2	1	-2	2	1	2	1	-1	1	3	1	2	-1	2	2	2	0
48	1.05	1.22	0.94	1.1	0.58	0.92	0.99	0.86	0.88	1.08	0.86	1.1	1.03	0.8	0.92	1.24	0.81	1.01	1.08	0.89	0.91	0.93	0.94	1.02	0.95	1.08	0.96	0.79	1.08	0.86	0.9
49	0.68	0.79	0.91	0.71	1.27	0.75	0.99	1.24	1.09	1.07	1.24	0.82	0.85	0.83	1.18	0.96	1.23	1.13	1.11	1.12	1.23	1.06	1.05	1.1	0.91	0.96	1.15	1.25	1.07	1.24	1.33
50	1.63	0.92	1.37	1.4	1.51	1.8	1.05	0.81	1.1	0.62	0.81	1.15	1.29	1.96	0.78	0.43	0.97	0.66	0.51	1	0.69	1.04	1.05	0.7	1.33	0.88	0.74	0.98	0.62	0.81	0.58
51	17.2	21.2	20.2	12.2	13.7	17	30.8	12.4	11.5	9.07	12.4	15.5	13.8	11.7	0	20.9	19.4	12.9	17.2	14.8	14.3	13.7	10.7	15.8	14.8	11.4	14.9	14.7	9.07	12.4	24.7
52	5.6	6.4	5.6	3.8	1.2	3.6	11.2	3	0.8	3.3	3	5.8	5	4.1	4.4	4.1	7.4	3.2	4.4	2.4	2.8	1.4	5.1	3.3	4.9	3.9	2.5	0.7	3.3	3	9.2
53	1	2	0	-2	-10	0	23	-4	-6	-4	-4	1	0	-4	-9	-3	1	-6	0	-8	-7	-9	-1	-7	0	-5	-8	-3	-4	-4	10
54	-0.01	-0.46	0.29	-0.4	-1.62	-0.04	5.89	-1.76	-1.98	-0.93	-1.76	0.31	-0.74	-1.44	-1.48	0.13	-0.12	-0.35	-0.97	-2.43	-1.4	-2.77	-0.22	-1.47	-0.52	-1.04	-1.81	-2.93	-0.93	-1.76	2.76
55	0.32	-2.24	0.63	-1.24	-4.34	-1.79	15.72	-4.44	-6.55	-2.49	-4.44	-0.17	-2.38	-3.55	-5.71	-0.13	1.17	-1.63	-1.72	-5.78	-3.93	-6.18	0.25	-3.26	-1.14	-3.66	-5.89	-8.58	-2.49	-4.44	6.74
56	0.5	1.4	1.6	-1.7	-4.8	-0.8	6.8	-1.8	-3.8	-2.4	-1.8	1.1	-0.1	-2.1	-1.2	-3.2	2.9	-2.5	0.3	-2.4	-1.9	-2.9	-0.9	-0.9	-0.2	-0.8	-3.1	-3.7	-2.4	-1.8	4.5
57	3	1	4	3	-1	6	5	-3	-6	-3	-3	1	2	2	-1	4	0	-2	-2	-9	-7	-9	-2	-7	0	-1	-9	-7	-3	-3	10
58	2	0	4	-5	1	0	-3	1	-2	3	1	2	1	2	2	-1	3	0	1	-4	1	-4	0	1	5	3	-4	0	3	1	2
59	2	4	0	-1	-1	0	11	-4	-4	0	-4	1	-4	1	0	1	2	-3	0	-8	0	-7	0	-3	1	1	-3	-4	0	-4	1
60	3	0	0	-3	0	1	1	-1	-2	2	-1	3	-1	2	0	-4	1	-1	1	-7	1	-3	0	0	3	3	-3	-1	2	-1	0
61	-8.1	-7.7	-6.3	-7.7	-7.5	-7.2	-6.1	-8.2	-8	-8.1	-8.2	-7.6	-7	-7.5	-9.7	-9	-6.8	-7.5	-7.1	-7.6	-7.7	-8.3	-8.2	-7.9	-8	-9.7	-8.3	-8	-8.1	-8.2	-5.5
62	-7	3	2	-18	-29	-18	86	-28	-42	-27	-28	-7	-9	-18	-18	-6	20	-29	-5	-32	-28	-34	-8	-30	-20	-19	-33	-41	-27	-28	35
63	-1	2	1	-4	-3	-3	11	-5	-2	-4	-5	-5	-2	-6	-3	-3	3	-5	0	-3	-6	-5	-2	-3	-4	-4	-5	-2	-4	-5	5
64	-3.5	-0.5	0.5	-5.5	-3.5	-4.5	10	-4	-2	-4	-4	-5.5	-2.5	-8	-1	-0.5	2.5	-4.5	0	-4	-5.5	-6.5	-1	-3.5	-3.5	-4	-7	-2.5	-4	-4	6
65	0	2	0	-3	-5	-3	9	-4	-6	-3	-4	0	-1	-2	-1	-1	3	-3	-1	-4	-4	-6	0	-4	-1	-3	-5	-7	-3	-4	6
66	0	1	0	-1	-4	-1	6	-2	-4	-2	-2	0	0	-1	0	0	2	-2	-1	-3	-2	-4	0	-2	0	-2	-3	-4	-2	-2	4
67	-1	0	-1	-2	-4	-2	9	-3	-5	-3	-3	-1	-1	-2	0	-1	0	-3	-2	-5	-3	-6	-1	-3	-1	-2	-4	-6	-3	-3	5
68	0.004	0.036	0.021	0.097	0.047	0.013	0.001	0.008	0.07	0.522	0.008	0.014	0.01	0.005	0.038	0.014	0.015	0.009	0.034	0.051	0.046	0.015	0.019	0.021	0.001	0.003	0.053	0.006	0.522	0.008	0.009
69	-7.3	-1.19	-0.89	3.2	-4.71	-6	-9.21	-7.97	3.82	8.58	-7.97	-0.94	-2.27	-7.28	-2.7	-10.75	-6.44	1.5	-0.47	-0.51	0.31	-2.2	4.11	-9.48	-2.34	-10.67	-0.26	-8	8.58	-7.97	-1.81
70	1	0.3	-1.9	1.9	4.1	3.4	-0.3	-1.9	2.2	7.3	-1.9	-0.4	1.6	-0.6	1.1	-0.4	0.3	4	1.2	0.5	0.6	2	4.6	-1.1	-1	-4.1	0.5	0.2	7.3	-1.9	-1.3
71	-3.36	-0.19	-2.17	3.48	0.77	-1.42	-2.83	-4.64	3.25	9.44	-4.64	1.12	-0.47	-4.69	0.01	-4.39	-4.03	0.26	0.13	0.93	1.27	0.53	0.87	-5.08	-2.34	-3.46	1.17	-3.28	9.44	-4.64	-0.37
72	0.7	-2.1	0	-1.3	-0.3	0.5	0.2	-0.4	1.3	0	-0.4	-0.6	-0.4	-0.3	-0.1	-0.2	0.1	0	-0.8	-0.4	-1.9	-0.3	-0.6	-0.5	0.3	-0.3	-2.4	-0.2	0	-0.4	-0.8
73	-3	-1	-3	2	2	0	-2	-4	2	8	-4	-1	0	-4	0	-3	-4	-1	0	0	0	1	-1	-4	-3	-3	0	-2	8	-4	-2
74	-5	-5	-1	-5	-6	-2	-6	-12	-5	-6	-12	-8	-4	0	-10	-2	-6	-15	8	-5	-1	0	-3	-7	-14	-7	-6	-2	-6	-12	-5
75	16.1	-5.6	9	-15	-13.9	3	39.5	-12.4	-7.3	-1.5	-12.4	22.4	12.3	-3	-10.7	-10.1	48.4	-10.1	24.1	13	35.5	-5.7	2	-13.3	10.2	18.9	39.5	-8.2	-1.5	-12.4	-11.5
76	1.8	4.4	5	0.1	0.1	0.1	56.9	0.1	0.2	0.2	0.1	1.2	1.1	1	0.1	0.1	3.4	0.1	0.1	0.1	0.1	0.1	0.1	0.4	0.8	0.1	0.1	0.1	0.2	0.1	5
77	1.8	4.4	5	0.1	0.1	0.1	56.9	0.1	0.2	0.2	0.1	1.2	1.1	1	0.1	0.1	3.4	0.1	0.1	0.1	0.1	0.1	0.1	0.4	0.8	0.1	0.1	0.1	0.2	0.1	5
78	2.1	0.1	0.1	0.1	1.8	0.1	0.1	0.6	0.3	64.6	1	0.5	0.1	0.1	1.1	8.7	0.1	3.4	2.4	0.9	6.6	0.2	0.1	0.3	0.1	0.1	0.9	1.5	1.2	1	60.8
79	2.1	0.1	0.1	0.1	1.8	0.1	0.1	0.6	0.3	64.6	1	0.5	0.1	0.1	1.1	8.7	0.1	3.4	2.4	0.9	6.6	0.2	0.1	0.3	0.1	0.1	0.9	1.5	1.2	1	60.8
80	2.1	0.1	0.1	0.1	1.8	0.1	0.1	0.6	0.3	64.6	1	0.5	0.1	0.1	1.1	8.7	0.1	3.4	2.4	0.9	6.6	0.2	0.1	0.3	0.1	0.1	0.9	1.5	1.2	1	60.8
81	2.1	0.1	0.1	0.1	1.8	0.1	0.1	0.6	0.3	64.6	1	0.5	0.1	0.1	1.1	8.7	0.1	3.4	2.4	0.9	6.6	0.2	0.1	0.3	0.1	0.1	0.9	1.5	1.2	1	60.8
82	2.1	0.1	0.1	0.1	1.8	0.1	0.1	0.6	0.3	64.6	1	0.5	0.1	0.1	1.1	8.7	0.1	3.4	2.4	0.9	6.6	0.2	0.1	0.3	0.1	0.1	0.9	1.5	1.2	1	60.8
83	2.1	0.1	0.1	0.1	1.8	0.1	0.1	0.6	0.3	64.6	1	0.5	0.1	0.1	1.1	8.7	0.1	3.4	2.4	0.9	6.6	0.2	0.1	0.3	0.1	0.1	0.9	1.5	1.2	1	60.8
84	2.1	0.1	0.1	0.1	1.8	0.1	0.1	0.6	0.3	64.6	1	0.5	0.1	0.1	1.1	8.7	0.1	3.4	2.4	0.9	6.6	0.2	0.1	0.3	0.1	0.1	0.9	1.5	1.2	1	60.8
85	2.1	0.1	0.1	0.1	1.8	0.1	0.1	0.6	0.3	64.6	1	0.5	0.1	0.1	1.1	8.7	0.1	3.4	2.4	0.9	6.6	0.2	0.1	0.3	0.1	0.1	0.9	1.5	1.2	1	60.8
86	2.1	0.1	0.1	0.1	1.8	0.1	0.1	0.6	0.3	64.6	1	0.5	0.1	0.1	1.1	8.7	0.1	3.4	2.4	0.9	6.6	0.2	0.1	0.3	0.1	0.1	0.9	1.5	1.2	1	60.8
87	2.1	0.1	0.1	0.1	1.8	0.1	0.1	0.6	0.3	64.6	1	0.5	0.1	0.1	1.1	8.7	0.1	3.4	2.4	0.9	6.6	0.2	0.1	0.3	0.1	0.1	0.9	1.5	1.2	1	60.8
88	2.1	0.1	0.1	0.1	1.8	0.1	0.1	0.6	0.3	64.6	1	0.5	0.1	0.1	1.1	8.7	0.1	3.4	2.4	0.9	6.6	0.2	0.1	0.3	0.1	0.1	0.9	1.5	1.2	1	60.8
89	2.1	0.1	0.1	0.1	1.8	0.1	0.1	0.6	0.3	64.6	1	0.5	0.1	0.1	1.1	8.7	0.1	3.4	2.4	0.9	6.6	0.2	0.1	0.3	0.1	0.1	0.9	1.5	1.2	1	60.8
90	2.1	0.1	0.1	0.1	1.8	0.1	0.1	0.6	0.3	64.6	1	0.5	0.1	0.1	1.1	8.7	0.1	3.4	2.4	0.9	6.6	0.2	0.1	0.3	0.1	0.1	0.9	1.5	1.2	1	60.8
91	0.029	0.014	0.007	0.013	0.031	0.033	0.003	0.02	0.004	0.007	0.004	0.377	0.027	0.009	0	0.001	0	0.001	0.004	0.001	0.002	0.002	0.002	0.004	0	0	0.004	0.003	0.002	0.004	0.01
92	0.029	0.014	0.007	0.013	0.031	0.033	0.003	0.02	0.004	0.007	0.004	0.377	0.027	0.009	0	0.001	0	0.001	0.004	0.001	0.002	0.002	0.002	0.004	0	0	0.004	0.003	0.002	0.004	0.01
93	0.029	0.014	0.007	0.013	0.031	0.033	0.003	0.02	0.004	0.007	0.004	0.377	0.027	0.009	0	0.001	0	0.001	0.004	0.001	0.002	0.002	0.002	0.004	0	0	0.004	0.003	0.002	0.004	0.01
94	0.029	0.014	0.007	0.013	0.031	0.033	0.003	0.02	0.004	0.007	0.004	0.377	0.027	0.009	0	0.001	0	0.001	0.004	0.001	0.002	0.002	0.002	0.004	0	0	0.004	0.003	0.002	0.004	0.01
101	-6.8	-6.9	-5.3	-5.6	-5.8	-5.8	-5	-5.1	-6.5	-6.8	-6.8	-4.7	-6.6	-4.7	-4.9	-6.6	-5.4	-6.1	-7.4	-4.9	-6.2	-6.1	-5	-5.2	-5.2	-5.6	-4.7	-4.9	-5.9	-6.8	-6
102	68	17	21	19	22	15	31	20	62	7	32	65	43	52	40	45	103	44	81	64	105	19	50	42	32	45	71	53	85	32	33
103	-1.649	-1.308	-0.73	-2.254	-0.7	2.069	-0.629	-0.961	-1.401	-1.836	-1.683	-0.899	-0.811	8.069	6.961	-1.591	0	-0.818	-1.53	7.042	-1.495	-1.155	-0.577	-2.101	-0.547	2.657	-0.476	-0.807	-1.248	-1.683	-1.53
104	-0.635	-0.783	0.538	-0.312	0.459	-0.244	0.298	0.04	-0.034	0.707	-0.105	-0.03	0.069	0.694	-0.004	-0.335	0.121	-0.305	-0.1	-0.294	-0.289	-0.218	-0.491	0.071	-0.036	0.044	0.442	0.492	0.075	-0.105	0.211
105	-0.97	-0.07	-0.68	-0.44	0.4	-0.78	-0.2	0.07	-0.6	0.02	-0.99	-0.37	-0.96	0.11	0.22	-0.82	-0.32	-0.67	-0.87	-0.2	-0.6	-1.1	-0.74	-0.45	-0.39	0.21	0.25	-0.23	-0.7	-0.99	0.35
106	-0.09	-0.67	0.06	-0.28	0.08	-0.16	0.34	0.22	-0.07	-0.12	-0.04	0.09	0.04	0	-0.1	0	0.14	-0.34	0.11	-0.21	0.24	-0.13	-0.2	-0.33	-0.2	-0.25	0.09	0.13	0.02	-0.04	0.06
107	-0.041	-0.2448	-0.0179	-0.174	-0.0157	-0.0219	0.07	0.0656	0.026	-0.0382	0.061	0.0159	0.0217	0.0017	-0.0183	0.0107	0.0607	-0.0768	-0.0112	-0.109	0.054	0.0085	-0.0725	-0.091	-0.029	-0.0652	0.068	0.0568	-0.0303	0.061	-0.0484
108	-0.2639	-0.0775	-0.025	-0.091	-0.054	-0.207	-0.0802	0.0052	-0.2898	-0.1066	-0.3209	-0.1408	-0.2444	-0.1165	-0.0522	-0.4212	-0.0176	-0.1976	-0.3164	-0.112	-0.2614	-0.1621	-0.1149	-0.1326	-0.1431	-0.1369	0.0214	-0.1445	-0.2792	-0.3209	-0.2793
109	-5.5	-6.37	-3.11	-3.66	-3.16	-3.56	-2.95	-3.31	-5.05	-5.42	-4.44	-2.85	-3.89	-2.25	-2.42	-4.95	-2.5	-3.33	-4.63	-2.01	-4.26	-4.92	-2.47	-2.8	-2.53	-2.75	-2.3	-2.48	-4.05	-4.44	-3.55
110	-0.09	-0.67	0.07	-0.29	0.07	-0.16	0.34	0.21	-0.08	-0.12	-0.05	0.08	0.04	-0.01	-0.11	-0.01	0.13	-0.35	0.11	-0.21	0.24	-0.13	-0.2	-0.34	-0.21	-0.26	0.08	0.13	0.01	-0.05	-0.07
111	-6.14	-5.55	-3.07	-3.73	-3.11	-3.41	-2.99	-3.22	-5.18	-5.06	-4.66	-3.36	-4.16	-2.76	-2.79	-5.66	-3.01	-3.52	-5.25	-2.6	-5.67	-4.91	-2.76	-3.19	-2.97	-3.16	-2.78	-3.01	-4.62	-4.66	-4.17
112	-0.07	-0.37	0.11	-0.38	0.15	-0.04	0.25	0.33	-0.03	0.01	0.01	0.07	0.27	-0.06	-0.14	-0.04	0.04	-0.1	0.03	-0.22	0	-0.13	0.02	-0.24	-0.11	-0.19	0.06	0.14	0.13	0.01	0.1
113	4810	1393	1381	2253	1057	1588	1889	1708	3723	519	1975	7942	2045	5105	4601	5549	7235	2441	6770	4678	10806	2765	3792	4653	3350	4433	5537	5102	7777	1975	2491
114	-0.62	-0.73	-0.1	-0.37	-0.02	-0.21	-0.01	-0.02	-0.51	-0.64	-0.49	-0.2	-0.39	-0.07	-0.08	-0.58	-0.22	-0.3	-0.49	-0.05	-0.55	-0.56	-0.11	-0.25	-0.14	-0.25	-0.08	-0.09	-0.38	-0.49	-0.45
115	-0.03	-0.21	0.07	-0.21	0.1	-0.02	0.15	0.2	-0.01	-0.1	0.01	0.05	0.16	-0.03	-0.06	-0.02	0.03	-0.05	0.02	-0.12	0	-0.08	0.02	-0.13	-0.06	-0.1	0.04	0.09	0.08	0.01	0.01
116	-4.87	-4.87	-3.23	-3.6	-3.25	-3.42	-3.3	-3.44	-4.63	-4.74	-4.2	-3.41	-4.08	-2.84	-2.71	-4.43	-3.12	-3.42	-4.51	-2.58	-4.32	-4.3	-2.82	-3.23	-2.9	-3.01	-2.97	-3.16	-4.04	-4.2	-3.69
117	-6.4	-5.49	-3.31	-3.96	-4.87	-4.54	-2.86	-3.48	-4.7	-5.16	-5.12	-3.96	-4.71	-3.63	-3.62	-5.33	-3.34	-3.78	-5.39	-2.64	-5.67	-5.01	-3.14	-2.92	-4.75	-4.32	-3.43	-3.33	-4.54	-5.12	-4.58
118	-0.31	-0.2	0.2	-0.85	0.01	0.04	0.86	0.18	0.2	0.27	-0.02	0	0.1	-0.04	0.08	-0.07	-0.21	0.34	-0.12	0.14	0.08	-0.05	0.04	-0.13	-0.21	-0.07	-0.03	0	0.03	-0.02	0.2
119	0.25	0	0.04	0.35	0.22	0.37	0.05	0.03	0.4	0	0.59	0.15	0.25	0.25	0.08	0.43	0.07	0.47	0.34	0.26	0.27	0.47	0.26	0.24	0.26	0.42	0.12	0.15	0.27	0.59	0.09
120	-0.7	-0.94	-0.09	-0.73	-0.11	-0.41	0.07	0	-0.62	-0.74	-0.55	-0.15	-0.16	-0.07	-0.16	-0.49	-0.04	-0.21	-0.33	-0.4	-0.44	-0.51	0.01	-0.4	-0.18	-0.37	0.07	0	-0.27	-0.55	-0.27
121	-0.44	-0.33	1.45	-2.24	-1.14	0.23	-0.08	-0.61	-0.41	-0.97	-0.85	-0.08	-0.65	0.04	-0.16	-0.79	-0.56	-1.02	-1.34	-0.2	0.25	-0.19	0.43	-0.68	-0.64	-1.64	-0.83	0.67	-0.5	-0.85	-2.22
122	-0.85	-0.3	-0.04	-2.51	0	-0.14	-0.77	0.32	-0.18	-1.35	-1.96	-0.5	-0.23	-0.27	-0.84	-1.43	-0.62	-1.34	-1.42	-1.06	-0.53	0.02	0.22	-0.17	-0.79	0.54	-0.86	-0.19	-0.3	-1.96	-1.35

Table S12 Amino acid descriptors (2D) used in this study.

	W	W	W	W	W	W	W	W	W	W	W	Y	Y	Y	Y	Y	Y	Y	Y	Y	Y	Y	Y	Y	Y	Y	Y	Y	Y	Y	Y
	L	M	N	P	Q	R	S	T	V	W	Y	A	C	D	E	F	G	H	I	K	L	M	N	P	Q	R	S	T	V	W	Y
No.	WL	WM	WN	WP	WQ	WR	WS	WT	WV	WW	WY	YA	YC	YD	YE	YF	YG	YH	YI	YK	YL	YM	YN	YP	YQ	YR	YS	YT	YV	YW	YY
123	-1.7	-2	-0.8	-1.3	-1	-1.3	-0.6	-0.9	-1.6	-2.2	-1.8	-0.7	-0.8	-1	-1	-1.7	0	-1.5	-1.4	-1	-1.4	-1.5	-0.8	-1	-1.1	-1.4	-0.6	-0.8	-1.2	-1.8	-1.6
124	-4.4	-4.8	-3.5	-3.7	-3.3	-3.5	-3.1	-3.7	-4.8	-4.7	-4.3	-3.7	-3.8	-3.5	-3	-4.1	0	-3.7	-4.4	-3.5	-4.1	-4.4	-3.5	-3.5	-3.4	-3.6	-3.1	-3.6	-4.5	-4.3	-4.1
125	-0.8	-1.3	-0.1	-0.7	-0.2	-0.9	-0.1	0	-0.8	-0.8	-0.5	-0.5	-0.1	-0.3	0	-0.5	-0.4	-0.8	-0.5	-0.2	-0.1	-0.6	-0.5	-0.9	-0.5	-0.4	-0.1	-0.2	-0.3	-0.5	-0.8
126	0.1	-0.1	0	-0.4	-0.1	-0.1	0.2	0.2	0.1	0	0.1	-0.1	0.1	-0.2	-0.3	0	0	-0.1	0.1	-0.3	0	-0.1	-0.2	-0.4	-0.2	-0.3	-0.1	0	0.1	0.1	-0.1
127	-1.4	-1.5	0	-0.8	-0.4	-0.6	-0.1	-0.2	-1.1	-1.2	-1.2	-0.4	-0.1	-0.2	-0.2	-1	0.1	-0.8	-1	-0.2	-0.9	-1.1	-0.2	-0.5	-0.3	-0.7	0.1	-0.2	-0.8	-1.2	-0.8
128	-1.1	-1.2	-0.2	-0.7	-0.5	-0.6	-0.2	-0.3	-0.9	-1.1	-1.1	-0.2	-0.3	-0.1	-0.2	-0.9	0.3	-0.7	-0.8	-0.2	-0.9	-0.8	-0.2	-0.5	-0.3	-0.7	0.1	-0.2	-0.7	-1.1	-0.7
129	-1.2	-1.6	-0.6	-0.9	-0.9	-0.9	-0.5	-0.6	-1	-1.7	-1.3	-0.3	-1	-0.2	-0.2	-1	0.2	-0.9	-0.9	-0.3	-0.9	-1	-0.3	-0.6	-0.4	-0.7	0	-0.2	-0.7	-1.3	-0.9
130	-1.1	-1.1	-0.4	-0.8	-0.4	-0.5	-0.5	-0.8	-1.1	-1.4	-1.1	0	-1.2	-0.2	-0.1	-1.2	0.4	-0.6	-1.1	0	-1.1	-0.9	-0.3	-0.8	-0.3	-0.3	-0.6	-0.6	-1	-1.1	-0.9
131	-1	-1	-0.5	-0.8	-0.5	-0.6	-0.7	-0.6	-0.9	-1.1	-1	0.2	-1	-0.5	-0.4	-1	0.2	-0.7	-0.8	-0.3	-0.9	-1	-0.6	-0.8	-0.4	-0.5	-0.6	-0.5	-0.7	-1	-0.8
132	-1.1	-1.1	-0.8	-0.9	-0.9	-0.7	-0.8	-0.8	-1.1	-1.6	-1.3	-0.1	-1.1	-0.7	-0.7	-1.3	0	-1.2	-1.2	-0.8	-1.1	-1.1	-0.8	-0.8	-0.8	-1	-0.8	-0.8	-1.1	-1.3	-1.1
133	6.3	6.1	5.7	5.4	5.5	6	5.8	6.1	6.3	6.4	6.5	5.5	6.1	5.7	5.8	5.9	4.3	5.7	6	5.6	5.8	6.3	5.7	5.8	5.6	6	5.8	5.9	5.9	6.5	6.3
134	6.3	6.5	6.5	5.8	6.2	5.9	5.7	6.5	6.3	7.5	6.9	5.6	6.1	6.2	6.1	6.4	4.8	6.2	6.2	5.7	6.1	6.1	5.9	5.6	6.7	6.3	5.9	6.1	6	6.9	6.5
135	6.6	6.6	6.2	5.9	6.7	6.3	5.6	6.5	6.4	7	6.1	5.7	5.9	6.2	6.5	6.1	6.2	5.7	6.2	5.8	6	6	6.1	6	5.5	6.5	6.1	6.5	6.1	6.1	6.2
136	0.0107	0.9849	-0.003	0.0139	0.0121	-0.0148	-0.0023	0.0038	-0.0015	0.1318	0.0037	-0.0007	0.0026	0.0002	0.0033	-0.0033	-0.0007	0.0073	-0.0048	0.008	0.0035	-0.0033	-0.007	0.001	-0.0051	0.0042	-0.0019	-0.0012	-0.0022	0.0037	-0.0077
137	-0.1684	-0.1	0.286	-0.5591	-0.0457	-0.3312	0.3655	0.3361	0.1217	0.0546	-0.0328	0.1284	0.1646	0.4647	0.1888	-0.0153	0.1064	0.0269	-0.1696	-0.169	-0.2061	-0.2292	0.1264	-0.4161	-0.0378	-0.1449	0.3161	0.3658	0.0206	-0.0328	-0.0167
138	0.1356	0.001	0.0369	-0.1359	-0.0303	0.0402	-0.0315	0.038	0.075	-0.051	-0.0089	-0.0531	0.002	-0.1298	-0.0382	-0.037	-0.0093	0.0074	0.0824	-0.0311	0.0359	-0.0112	0.058	-0.1066	-0.0242	0.0155	-0.0216	0.0679	0.041	-0.0089	0.0016
139	-0.0601	-0.0664	0.0099	0.0105	-0.0687	0.0774	0.0189	0.0165	0.0159	-0.0752	-0.0907	0.039	-0.0139	-0.0585	-0.0819	-0.0217	0.04	-0.0331	0.0214	0.0645	0.0068	-0.001	-0.0166	-0.0559	-0.0471	0.0223	0.0014	0.0063	0.0332	-0.0907	-0.0393
140	0.0541	-0.0187	-0.0182	-0.0197	0.0015	-0.0337	-0.031	0.0839	0.0379	-0.1115	-0.012	0.0121	0.0344	-0.0087	0.0011	0.0017	-0.0151	-0.0158	0.072	0.0019	0.0329	-0.0128	-0.0226	-0.0477	-0.0333	-0.0466	-0.0309	-0.0271	0.0335	-0.012	-0.0583
141	-0.0015	-0.0048	0.0026	-0.0026	0.0005	0.0014	0.0011	0.0047	0.0008	-0.0084	-0.0051	0.0011	-0.0026	0.0016	0.0022	-0.004	0.0011	-0.0011	0.0004	0.0036	-0.002	-0.0039	0.0018	-0.0038	0.0001	0.0006	0.0007	0.0016	0.0001	-0.0051	-0.005
142	-2.27	-2.2	-0.48	-0.75	-0.88	-0.65	-0.72	-1.22	-1.82	-1.21	-1.82	-1.1	-2.05	-0.22	-0.12	-2.05	-1.05	-0.59	-2.01	-0.41	-1.93	-1.69	-0.28	-0.41	-0.33	-0.49	-0.62	-0.74	-1.76	-1.82	-1.13
143	-2.8	-2.31	-1.13	-2.05	-1.72	-1.39	-1.5	-1	-2.4	-1.6	-2	-2.57	-2.92	-1.5	-1.27	-2.73	-2.69	-1.74	-2.9	-1.42	-2.7	-2.26	-1.47	-1.69	-1.31	-1.57	-1.61	-1.46	-2.4	-2	-1.6
144	-0.98	-0.5	0.13	-0.33	-0.01	-0.04	0.14	-0.19	-0.57	-0.46	-0.87	-0.07	-0.58	0.38	0.47	-0.63	0.28	-0.18	-0.74	0.62	-0.56	-0.26	0.4	0.04	0.09	0.22	0.23	0.27	-0.38	-0.87	-0.18
145	-1.76	-0.93	0.11	0.06	0.21	0	0.22	-0.53	-1.34	-1.29	-1.4	-0.75	-1.94	0.18	0.31	-1.83	-1.27	-0.61	-1.88	0.28	-1.66	-1.54	-0.19	0.26	0.39	0.33	-0.19	-0.76	-1.52	-1.27	-0.96
146	-1.46	-1.29	0.62	-0.24	0.45	-0.09	-0.04	0.41	-1.11	-1.83	-0.79	-0.37	-0.98	0.12	0.52	-1.36	0.08	-0.22	-1.13	0.6	-0.95	-0.85	0.53	0.23	0.31	0.06	0.47	0.11	-0.82	-0.67	-0.77
147	-1.81	-1.7	0.04	-0.5	-0.44	0.14	-1.01	-0.31	-1.16	-0.67	-1.2	-0.39	-1.26	0.02	0.87	-0.91	-0.61	-0.58	-1.09	0.26	-1.15	-0.6	0.24	-0.55	0.66	0.32	0.13	0.12	-0.85	-0.73	-0.75
148	50.962	50.769	46.01	46.535	49.104	55.596	42.483	45.577	47.869	60.195	55.711	37.197	40.097	39.894	42.987	50.424	34.103	46.01	46.477	48.911	46.477	46.284	41.525	42.05	44.619	51.111	37.999	41.092	43.384	55.711	51.226
149	0	0	0	0	0	0	1	0	0	0	0	1	1	1	0	0	1	0	1	0	1	0	1	1	0	0	1	1	1	0	0
150	0	0	0	0	0	0	0	0	0	0	0	0	0	0	0	0	0	0	0	0	0	0	0	0	0	0	0	0	0	0	0
151	1	1	1	1	1	1	0	1	1	1	1	0	0	0	1	1	0	3	0	1	0	1	0	0	1	1	0	0	0	1	1
152	3	3	3	3	3	3	2	3	3	3	3	2	2	2	3	3	2	5	2	3	2	3	2	2	3	3	2	2	2	3	3
153	1	1	2	1	2	1	2	2	1	1	2	2	2	2	2	2	2	4	2	2	2	2	3	2	3	2	3	3	2	2	3
154	2	2	2	2	2	2	2	2	2	2	2	2	2	4	4	2	2	2	2	2	2	2	2	2	2	2	2	2	2	2	2
155	9	9	9	9	9	9	9	9	9	18	15	6	6	6	6	12	6	11	6	6	6	6	6	6	6	6	6	6	6	15	12
156	1	1	1	1	1	4	1	1	1	1	1	1	1	1	1	1	1	1	1	2	1	1	1	1	1	4	1	1	1	1	1
157	46	44	41	41	44	51	38	41	43	51	48	34	35	36	39	44	31	41	43	46	43	41	38	38	41	48	35	38	40	48	45
158	2	2	3	1	3	2	3	3	2	3	3	2	2	2	2	2	2	4	2	2	2	2	3	1	3	2	3	3	2	3	3
159	3	3	5	2	5	3	5	5	3	4	5	4	4	4	4	4	4	8	4	4	4	4	6	3	6	4	6	6	4	5	6
160	23	23	23	22	24	26	21	22	22	29	27	18	19	21	22	24	17	23	21	22	21	21	21	20	22	24	19	20	20	27	25
161	15	15	12	14	13	14	12	13	14	20	18	10	10	10	11	16	9	12	13	13	13	13	10	12	11	12	10	11	12	18	16
162	71.045	74.467	69.998	65.439	73.776	83.446	63.877	67.591	67.581	80.324	76.646	55.954	62.505	61.898	65.831	68.718	52.223	69.998	66.619	72.687	66.619	69.918	65.464	61.06	69.282	79.826	58.896	62.647	63.136	76.646	72.027
163	1.5445	1.6924	1.7073	1.5961	1.6767	1.6362	1.681	1.6486	1.5716	1.575	1.5968	1.6457	1.7859	1.7194	1.688	1.5618	1.6846	1.7073	1.5493	1.5801	1.5493	1.7053	1.7227	1.6068	1.6898	1.663	1.6827	1.6486	1.5784	1.5968	1.6006
164	17	16	15	16	16	17	14	15	16	22	20	12	12	13	14	18	11	15	15	15	15	14	13	14	14	15	12	13	14	20	18
165	23	21	18	19	20	25	17	19	21	22	21	16	16	15	17	20	14	18	22	24	22	20	17	18	19	24	16	18	20	21	20
166	3	3	4	3	4	6	3	3	3	4	3	2	2	2	2	2	2	4	2	3	2	2	3	2	3	5	2	2	2	3	2
167	3	3	4	3	4	3	4	4	3	3	4	4	4	6	6	4	4	4	4	4	4	4	5	4	5	4	5	5	4	4	5
168	0	1	0	0	0	0	0	0	0	0	0	0	1	0	0	0	0	0	0	0	0	1	0	0	0	0	0	0	0	0	0
169	1.8316	1.8071	1.8321	1.5611	1.8102	1.7544	1.8193	1.8465	1.8465	1.2844	1.4697	2.2939	2.3392	2.4005	2.3852	1.7697	2.2164	1.763	2.4381	2.3516	2.3997	2.3623	2.4005	1.8379	2.3852	2.332	2.3392	2.4025	2.4025	1.4803	1.772
170	-2.6769	-2.602	-2.5592	-2.5597	-2.5941	-2.6317	-2.5568	-2.5928	-2.6498	-2.5647	-2.5685	-2.5612	-2.563	-2.5669	-2.6003	-2.5718	-2.5347	-2.5643	-2.6842	-2.6628	-2.6771	-2.6034	-2.5631	-2.5641	-2.5958	-2.6323	-2.5609	-2.5946	-2.6503	-2.5681	-2.5717
171	-0.5439	-0.5433	-0.5459	-0.5458	-0.5476	-0.5486	-0.5464	-0.5451	-0.5409	-0.5525	-0.5856	-0.5609	-0.6113	-0.5808	-0.5809	-0.6149	-0.6431	-0.591	-0.5477	-0.5477	-0.5477	-0.5467	-0.5931	-0.6113	-0.5734	-0.5795	-0.6112	-0.6112	-0.5477	-0.5866	-0.6431
172	0.5435	0.542	0.5603	0.5831	0.5432	0.5654	0.5845	0.5806	0.5389	0.6023	0.6355	0.5516	0.6481	0.5887	0.5931	0.6597	0.7006	0.6522	0.4939	0.5391	0.5319	0.4907	0.533	0.6294	0.5168	0.5938	0.6499	0.6358	0.4982	0.6366	0.7029
173	2.653	2.6292	2.6253	2.7247	2.6337	2.6448	2.6205	2.6344	2.6456	2.6213	2.6208	2.6136	2.6164	2.6178	2.6268	2.6184	2.6039	2.6196	2.6625	2.6567	2.6521	2.6273	2.6232	2.7243	2.632	2.6437	2.6181	2.6327	2.6443	2.619	2.6184
174	-3.3115	-3.3115	-3.3115	-3.3095	-3.3115	-3.3115	-3.3116	-3.3117	-3.3116	-3.3115	-3.3115	-3.3115	-3.3116	-3.3115	-3.3115	-3.3116	-3.3113	-3.3116	-3.3116	-3.3116	-3.3115	-3.3115	-3.3115	-3.3095	-3.3115	-3.3115	-3.3117	-3.3117	-3.3116	-3.3116	-3.3116
175	-0.4617	-0.4528	-0.5368	-0.5199	-0.5303	-0.5257	-0.5947	-0.5441	-0.4606	-0.5272	-0.5792	-0.5381	-0.6319	-0.5959	-0.5874	-0.5381	-0.5381	-0.5545	-0.5381	-0.5381	-0.5367	-0.5117	-0.5622	-0.5523	-0.5556	-0.579	-0.6417	-0.5697	-0.5381	-0.5789	-0.5913
176	0.6535	0.5956	0.5975	0.6364	0.5955	0.6706	0.6356	0.6164	0.6534	0.706	0.73	0.4835	0.6738	0.524	0.5217	0.7408	0.589	0.6768	0.4647	0.4795	0.4789	0.4526	0.4541	0.5475	0.4508	0.577	0.5578	0.5068	0.4654	0.7293	0.7833
177	2.6891	2.5458	2.4499	2.6848	2.5411	2.5922	2.446	2.4578	2.6128	2.4645	2.4597	2.4399	2.4384	2.4402	2.5362	2.4487	2.4342	2.4408	2.683	2.6641	2.6891	2.5457	2.4409	2.6847	2.5409	2.5921	2.4359	2.4526	2.6128	2.4617	2.4557
178	-2.4256	-2.3622	-2.3513	-2.3527	-2.3658	-2.3922	-2.3509	-2.3677	-2.4013	-2.3513	-2.352	-2.352	-2.3507	-2.3531	-2.3664	-2.3546	-2.3427	-2.3539	-2.4325	-2.4354	-2.4259	-2.3642	-2.354	-2.3554	-2.3677	-2.3929	-2.3535	-2.3694	-2.4019	-2.354	-2.3546
179	-0.4051	-0.3597	-0.3865	-0.3953	-0.3889	-0.4117	-0.4135	-0.3854	-0.37	-0.4158	-0.454	-0.4386	-0.4672	-0.4479	-0.448	-0.4779	-0.4999	-0.4657	-0.3637	-0.4221	-0.4102	-0.3705	-0.4146	-0.4566	-0.4019	-0.448	-0.4719	-0.4475	-0.3696	-0.4555	-0.4999
180	0.7054	0.7016	0.7241	0.7427	0.7037	0.7263	0.7422	0.7376	0.703	0.7617	0.7927	0.7113	0.7995	0.7447	0.7458	0.8124	0.8502	0.8036	0.6272	0.6919	0.6774	0.6263	0.7019	0.7788	0.6798	0.746	0.7978	0.7589	0.6344	0.796	0.8543
181	2.895	2.8675	2.8374	2.9763	2.8568	2.875	2.8259	2.8507	2.8815	2.8374	2.8354	2.8184	2.8347	2.8299	2.851	2.8327	2.7967	2.8307	2.9067	2.8853	2.8946	2.8665	2.835	2.976	2.8554	2.8742	2.8226	2.8489	2.8808	2.835	2.8328
182	28.018	28.111	22.85	24.965	25.036	29.544	21.459	23.645	25.831	27.585	25.831	19.705	21.985	20.528	22.715	24.078	17.519	23.872	26.265	27.131	26.265	26.358	21.097	23.212	23.283	27.791	19.705	21.892	24.078	25.831	24.078
183	7	8	7	5	8	9	6	6	6	7	7	5	6	7	8	7	5	7	7	9	7	8	7	5	8	9	6	6	6	7	7
184	0.2917	0.3333	0.2917	0.2083	0.32	0.3333	0.2727	0.2609	0.2609	0.2188	0.2414	0.2778	0.3158	0.3333	0.3636	0.28	0.2941	0.2917	0.3333	0.4091	0.3333	0.381	0.3333	0.2381	0.3636	0.375	0.3158	0.3	0.3	0.2414	0.2692
185	10	10	10	10	10	10	10	10	10	20	16	6	6	6	6	12	6	11	6	6	6	6	6	6	6	6	6	6	6	16	12
186	47	45	42	43	45	52	39	42	44	54	50	34	35	36	39	45	31	42	43	46	43	41	38	39	41	48	35	38	40	50	46
187	2	2	3	2	3	3	2	2	2	2	2	2	2	3	3	2	2	2	2	2	2	2	3	2	3	3	2	2	2	2	2
188	24	24	24	24	25	27	22	23	23	32	29	18	19	21	22	25	17	24	21	22	21	21	21	21	22	24	19	20	20	29	26
189	6	7	4	4	5	7	5	5	5	4	4	4	5	4	5	4	4	4	6	8	6	7	4	4	5	7	5	5	5	4	4
190	8	9	8	5	9	11	7	7	7	8	8	6	7	8	9	8	6	8	8	10	8	9	8	5	9	11	7	7	7	8	8
191	0.3333	0.375	0.3333	0.2083	0.36	0.4074	0.3182	0.3043	0.3043	0.25	0.2759	0.3333	0.3684	0.381	0.4091	0.32	0.3529	0.3333	0.381	0.4545	0.381	0.4286	0.381	0.2381	0.4091	0.4583	0.3684	0.35	0.35	0.2759	0.3077
192	35	33	29	31	32	39	27	30	32	32	32	26	27	27	30	31	23	29	35	38	35	33	29	31	32	39	27	30	32	32	32
193	16.983	16.82	16.983	15.69	17.69	19.104	15.405	16.276	16.276	20.38	19.389	13.707	14.414	15.991	16.698	17.527	12.836	16.82	15.991	16.535	15.991	15.828	15.991	14.698	16.698	18.113	14.414	15.284	15.284	19.389	18.397
194	13.465	13.819	12.373	12.249	13.08	14.456	11.335	12.205	12.758	15.774	14.644	10.05	11.099	11.074	11.781	13.144	9.18	12.359	12.335	12.586	12.335	12.689	11.243	11.119	11.95	13.326	10.205	11.075	11.628	14.644	13.514
195	10.533	9.6628	8.4557	9.3699	9.1628	9.8699	7.9557	8.8259	9.8259	12.342	11.265	7.1712	6.8783	7.3783	8.0854	10.265	6.301	8.533	9.4557	8.9996	9.4557	8.5854	7.3783	8.2925	8.0854	8.7925	6.8783	7.7486	8.7486	11.265	10.188
196	11.569	10.698	9.5685	10.405	10.276	10.983	8.9912	9.8614	10.861	14.259	12.974	7.9996	7.7067	8.2841	8.9912	11.82	7.1294	9.6983	10.284	9.8281	10.284	9.4138	8.2841	9.121	8.9912	9.6983	7.7067	8.577	9.577	12.974	11.69
197	10.88	11.024	10.88	10.575	11.38	12.38	10.024	10.397	10.397	14.025	12.936	8.3968	8.9348	9.7906	10.291	11.452	7.9861	10.952	9.8454	10.435	9.7906	9.9348	9.7906	9.4861	10.291	11.291	8.9348	9.3074	9.3074	12.936	11.846
198	7.7743	8.8022	7.0577	7.4708	7.5577	8.3923	6.5276	6.972	7.2912	9.4697	8.6099	5.5208	6.3004	6.1135	6.6135	7.6159	5.0732	7.0531	6.9695	7.3517	6.9146	7.9424	6.198	6.611	6.698	7.5326	5.6679	6.1123	6.4315	8.6099	7.7502
199	5.6764	4.6134	4.467	5.1134	4.967	5.1134	4.1134	4.6159	5.1932	6.833	6.2883	3.7379	3.5688	3.9223	4.4223	5.833	3.2254	4.211	5.1866	5.0688	5.1317	4.0688	3.9223	4.5688	4.4223	4.5688	3.5688	4.0712	4.6486	6.2883	5.7437
200	6.8458	5.7828	5.6911	6.2828	6.1911	6.2828	5.2828	5.7853	6.3626	9.0825	8.3241	4.6935	4.5244	4.9327	5.4327	7.7491	4.1911	5.3409	6.1422	6.0244	6.0874	5.0244	4.9327	5.5244	5.4327	5.5244	4.5244	5.0268	5.6042	8.3241	7.5656
201	2	2	2	2	2	2	2	3	2	2	2	2	2	2	2	2	1	2	3	2	2	2	2	2	2	2	2	3	2	2	2
202	2	2	2	2	2	2	2	3	2	2	2	2	2	2	2	2	1	2	3	2	2	2	2	2	2	2	2	3	2	2	2
203	0.7289	0.774	0.7911	0.7558	0.7787	0.756	0.7859	0.7729	0.7381	0.7473	0.7566	0.7755	0.8203	0.8317	0.8151	0.7458	0.7919	0.7869	0.7385	0.7441	0.7385	0.7878	0.8078	0.7691	0.7931	0.7669	0.8036	0.788	0.7492	0.7566	0.7673
204	12	13	12	11	13	15	11	11	11	15	15	11	11	12	13	14	11	13	12	14	12	13	12	11	13	15	11	11	11	15	15
205	0	0	0	0	0	1	0	0	0	0	0	0	0	-1	-1	0	0	0	0	1	0	0	0	0	0	1	0	0	0	0	0

Table S12 Amino acid descriptors (2D) used in this study.

	W	W	W	W	W	W	W	W	W	W	W	Y	Y	Y	Y	Y	Y	Y	Y	Y	Y	Y	Y	Y	Y	Y	Y	Y	Y	Y	Y
	L	M	N	P	Q	R	S	T	V	W	Y	A	C	D	E	F	G	H	I	K	L	M	N	P	Q	R	S	T	V	W	Y
No.	WL	WM	WN	WP	WQ	WR	WS	WT	WV	WW	WY	YA	YC	YD	YE	YF	YG	YH	YI	YK	YL	YM	YN	YP	YQ	YR	YS	YT	YV	YW	YY
206	-0.8628	-0.8628	-0.8628	-0.8628	-0.8628	-0.8628	-0.8628	-0.8628	-0.8628	-0.863	-0.8628	-0.8472	-0.8465	-0.8505	-0.8543	-0.8468	-0.848	-0.8488	-0.8469	-0.863	-0.8471	-0.847	-0.8467	-0.8513	-0.8471	-0.8471	-0.8459	-0.8458	-0.8469	-0.863	-0.8468
207	-0.359	-0.3705	-0.4335	-0.4275	-0.4332	-0.3865	-0.4275	-0.4257	-0.354	-0.4107	-0.4104	-0.3918	-0.4466	-0.5273	-0.5211	-0.3893	-0.4652	-0.3953	-0.3818	-0.3818	-0.3818	-0.3808	-0.4663	-0.4586	-0.4528	-0.396	-0.4562	-0.4361	-0.3818	-0.4119	-0.4279
208	0.0807	0.1423	0.1486	0.1627	0.1436	0.1812	0.1835	0.1747	0.1448	0.1812	0.1815	0.1855	0.1855	0.1477	0.1283	0.1512	0.1855	0.1837	0.1257	0.1849	0.0825	0.1512	0.1526	0.1625	0.1512	0.1855	0.1855	0.1782	0.1447	0.1852	0.1855
209	2.6775	2.63	2.6069	2.7045	2.6381	2.6891	2.5774	2.6176	2.6514	2.7095	2.6662	2.4837	2.4961	2.5072	2.5488	2.5988	2.4322	2.5681	2.6357	2.6403	2.6194	2.5617	2.5337	2.6481	2.5718	2.6338	2.4973	2.5467	2.5875	2.6587	2.6062
210	-2.2352	-2.2352	-2.2352	-2.2352	-2.2352	-2.2352	-2.2352	-2.2352	-2.2352	-2.2352	-2.2352	-2.2351	-2.2351	-2.2352	-2.2352	-2.2352	-2.2351	-2.2352	-2.2352	-2.2354	-2.2352	-2.2352	-2.2352	-2.2352	-2.2352	-2.2352	-2.2352	-2.2352	-2.2352	-2.2352	-2.2352
211	-0.3358	-0.3463	-0.4	-0.3788	-0.3909	-0.3632	-0.4214	-0.4036	-0.3358	-0.3572	-0.3801	-0.3935	-0.4362	-0.4656	-0.446	-0.3886	-0.4429	-0.3979	-0.3667	-0.3666	-0.3666	-0.3617	-0.4204	-0.391	-0.4151	-0.3898	-0.44	-0.4219	-0.3667	-0.3782	-0.3845
212	0.123	0.136	0.1015	0.1082	0.1014	0.1295	0.1017	0.1016	0.123	0.1425	0.1498	0.0588	0.129	0.0734	0.0726	0.1557	0.0966	0.129	0.0554	0.0581	0.0581	0.129	0.057	0.0699	0.0568	0.0935	0.0575	0.0573	0.0554	0.1498	0.1645
213	2.6576	2.6075	2.5709	2.6708	2.603	2.6381	2.5495	2.5797	2.6241	2.7077	2.6514	2.4265	2.4347	2.4393	2.485	2.5567	2.3844	2.498	2.59	2.5774	2.5779	2.5074	2.454	2.5878	2.5002	2.5494	2.424	2.4665	2.5301	2.6485	2.5715
214	-0.6409	-0.6409	-0.6409	-0.641	-0.6409	-0.6409	-0.6409	-0.6409	-0.6409	-0.6409	-0.6409	-0.6409	-0.6409	-0.6409	-0.6409	-0.6409	-0.6409	-0.6409	-0.6409	-0.6419	-0.6409	-0.6409	-0.6409	-0.641	-0.6409	-0.6409	-0.6409	-0.6409	-0.6409	-0.6409	-0.6409
215	-0.1973	-0.1951	-0.2252	-0.2186	-0.2252	-0.214	-0.2267	-0.2147	-0.1918	-0.2187	-0.225	-0.2161	-0.212	-0.2246	-0.2242	-0.2161	-0.2161	-0.2188	-0.2161	-0.2161	-0.2161	-0.211	-0.2305	-0.2298	-0.2306	-0.2242	-0.2317	-0.2239	-0.2161	-0.225	-0.2252
216	0.1607	0.1699	0.1693	0.1688	0.1699	0.1668	0.1697	0.1666	0.1577	0.1718	0.1805	0.1709	0.184	0.1746	0.1745	0.1849	0.1931	0.1842	0.1449	0.1653	0.1603	0.1836	0.1832	0.182	0.1836	0.1746	0.1834	0.1763	0.147	0.18	0.1931
217	2.916	2.8804	2.8509	2.9434	2.8797	2.9224	2.8219	2.8585	2.892	2.9727	2.9178	2.7025	2.7248	2.7331	2.7743	2.836	2.6502	2.795	2.8566	2.8491	2.8399	2.7935	2.7536	2.8701	2.7922	2.8483	2.7142	2.7635	2.8079	2.9149	2.8434
218	19.326	19.326	19.326	16.844	20.314	22.291	17.355	18.34	18.34	22.203	21.703	16.056	17.053	19.048	20.045	20.314	15.059	19.326	19.048	20.045	19.048	19.048	19.048	16.372	20.045	22.042	17.053	18.05	18.05	21.703	21.302
219	8.9091	9.4746	8.9091	7.2664	9.6298	11.111	8.0222	8.2031	8.2031	10.08	10.156	7.5556	8.3232	9.2092	9.9881	10.222	7.438	9.4746	9.2092	10.714	9.2092	9.904	9.2092	7.3199	9.9881	11.584	8.3232	8.4444	8.4444	10.156	10.364
220	5.5	5.5	5.5	3.5917	6.0464	7.165	4.4875	4.75	4.75	5.2578	5.9904	5.6805	5.8776	7.2	7.9084	7.0388	5.9282	6.428	6.3281	7.9084	7.2	7.2	7.2	4.25	7.9084	9.3333	5.8776	6.12	6.12	5.9904	7.26
221	14.626	15.732	13.636	12.574	14.561	16.063	12.761	13.691	13.691	15.515	15.567	12.119	14.286	14.305	15.267	14.814	11.163	13.989	15.013	15.984	15.013	16.245	13.914	12.739	14.871	16.397	13.08	14.045	14.045	15.567	15.748
222	6.6505	7.6355	6.1774	5.3427	6.7943	7.8935	5.8	6.0314	6.0314	6.9458	7.1843	5.6007	6.8994	6.8091	7.5011	7.3438	5.4009	6.7487	7.1659	8.4449	7.1659	8.3755	6.6122	5.6148	7.2959	8.498	6.2827	6.4774	6.4774	7.1843	7.5579
223	4.0631	4.3989	3.7634	2.6119	4.2176	5.0383	3.2027	3.4542	3.4542	3.5857	4.1938	4.1544	4.8321	5.2599	5.8789	5.0025	4.234	4.523	4.8756	6.1811	5.5474	6.0493	5.1015	3.2259	5.7121	6.7784	4.3811	4.6453	4.6453	4.1938	5.2402
224	4.2293	5.2226	3.6624	3.0535	4.1222	4.8768	3.5244	3.7534	3.7534	3.7159	4.1422	3.7709	5.1875	4.6382	5.2056	4.5328	3.5464	4.1046	5.123	6.1357	5.123	6.4791	4.3809	3.5764	4.9317	5.8061	4.3252	4.5488	4.5488	4.1422	4.7609
225	6	6	8	6	8	9	7	7	6	7	7	6	6	8	8	6	6	8	6	7	6	6	8	6	8	9	7	7	6	7	7
226	5	5	7	4	7	10	6	6	5	6	6	5	5	5	5	5	5	6	5	8	5	5	7	4	7	10	6	6	5	6	6
227	1	1	1	1	1	1	1	1	1	1	1	1	1	1	1	1	1	1	1	1	1	1	1	1	1	1	1	1	1	1	1
228	0	0	1	0	1	1	1	1	0	1	1	0	0	0	0	0	0	1	0	1	0	0	1	0	1	1	1	1	0	1	1
229	2.366	1.607	-0.53	1.34	-0.088	0.6	-0.083	0.379	1.924	2.853	2.179	0.278	0.388	-0.349	0.093	1.813	-0.184	-0.491	1.692	0.347	1.692	0.933	-1.204	0.666	-0.762	-0.074	-0.757	-0.295	1.25	2.179	1.505
230	-3.3651	-2.9993	-1.9661	-2.252	-2.1679	-2.5053	-1.6031	-1.9303	-2.5364	-3.9249	-3.2731	-1.481	-2.1423	-1.292	-1.4938	-2.9832	-1.1538	-1.7403	-2.3998	-1.4261	-2.7132	-2.3474	-1.3143	-1.6002	-1.516	-1.8535	-0.9513	-1.2785	-1.8845	-3.2731	-2.6212
231	8.904	9.1986	8.3538	8.2583	8.8289	9.7593	7.6836	8.1229	8.4368	11.082	10.176	6.5984	7.3566	7.2411	7.7187	9.1286	6.1499	8.3761	7.9967	8.3603	7.9867	8.2794	7.4347	7.3416	7.9105	8.8545	6.7576	7.1974	7.5192	10.176	9.2558
232	10	10	10	15	10	11	10	10	10	20	16	6	6	6	6	12	6	11	6	6	6	6	6	11	6	7	6	6	6	16	12
233	1	1	1	1	1	0	1	1	1	1	1	1	1	1	1	1	1	1	1	1	1	1	1	1	1	0	1	1	1	1	1
234	2	2	2	3	2	2	2	2	2	4	3	1	1	1	1	2	1	2	1	1	1	1	1	2	1	1	1	1	1	3	2
235	7	8	7	5	8	10	6	6	6	7	7	5	6	7	8	7	5	7	7	9	7	8	7	5	8	10	6	6	6	7	7
236	0	0	1	0	1	3	1	1	0	1	1	0	0	1	1	0	0	1	0	1	0	0	1	0	1	3	1	1	0	1	1
237	5.187	5.647	6.557	5.117	6.557	8.965	5.867	5.867	5.187	6.421	6.463	5.229	5.639	6.135	6.135	6.123	5.229	6.096	5.229	7.082	5.229	5.689	6.599	5.159	6.599	9.007	5.909	5.909	5.229	6.463	6.505
238	-5.188	-5.648	-6.558	-5.118	-6.558	-7.966	-5.868	-5.868	-5.188	-6.423	-6.464	-5.229	-5.639	-7.135	-7.135	-6.123	-5.229	-6.096	-5.229	-6.082	-5.229	-5.689	-6.599	-5.159	-6.599	-8.007	-5.909	-5.909	-5.229	-6.464	-6.505
239	2.8381	2.8483	3.2861	2.6878	3.3056	4.5479	3.0337	3.0671	2.7852	3.2345	3.2254	2.6717	2.807	2.7311	2.7649	2.9805	2.6353	3.223	2.8265	4.0688	2.8267	2.8368	3.2747	2.6764	3.2942	4.5364	3.0223	3.0557	2.7737	3.2231	3.2139
240	-2.8381	-2.8483	-3.2861	-2.6878	-3.3056	-3.5479	-3.0337	-3.0671	-2.7852	-3.2345	-3.2254	-2.6717	-2.807	-3.7311	-3.7649	-2.9805	-2.6353	-3.223	-2.8265	-3.0688	-2.8267	-2.8368	-3.2747	-2.6764	-3.2942	-3.5364	-3.0223	-3.0557	-2.7737	-3.2231	-3.2139
241	0.1218	0.1213	0.1052	0.1286	0.1045	0.076	0.1139	0.1127	0.1241	0.1068	0.1071	0.1293	0.1231	0.1265	0.125	0.1159	0.1311	0.1072	0.1223	0.0849	0.1222	0.1218	0.1055	0.1291	0.1049	0.0762	0.1143	0.1131	0.1246	0.1072	0.1075
242	0.193	0.1923	0.1667	0.2038	0.1657	0.1544	0.1805	0.1785	0.1966	0.1693	0.1698	0.205	0.1951	0.1473	0.146	0.1838	0.2079	0.1699	0.1938	0.1785	0.1938	0.1931	0.1672	0.2046	0.1663	0.1549	0.1812	0.1792	0.1975	0.1699	0.1704
243	81.275	72.744	51.417	81.671	68.649	59.948	42.886	55.682	72.744	77.071	51.417	30.028	36.44	49.173	49.002	25.763	17.232	51.21	55.621	42.824	55.621	47.09	25.763	56.017	42.995	34.294	17.232	30.028	47.09	51.417	25.763
244	43.447	43.447	52.148	43.447	43.447	65.17	66.929	52.617	43.447	64.774	68.967	47.64	47.64	47.64	47.64	68.967	61.952	79.842	47.64	69.363	47.64	47.64	56.341	47.64	47.64	69.363	71.122	56.81	47.64	68.967	73.16
245	4.2654	4.2654	4.2654	4.2654	4.2654	4.2654	4.2654	4.2654	4.2654	4.2654	4.2654	4.2654	12.046	4.2654	4.2654	4.2654	4.2654	8.5308	4.2654	4.2654	4.2654	4.2654	4.2654	4.2654	4.2654	4.2654	4.2654	4.2654	4.2654	4.2654	4.2654
246	20.384	20.384	37.622	11.765	37.622	20.384	20.384	20.384	20.384	29.003	20.384	11.765	11.765	11.765	11.765	11.765	11.765	20.384	11.765	11.765	11.765	11.765	29.003	3.146	29.003	11.765	11.765	11.765	11.765	20.384	11.765
247	0	0	12.95	0	12.95	0	10.324	10.324	0	0	10.324	10.324	10.324	10.324	10.324	10.324	10.324	10.324	10.324	10.324	10.324	10.324	23.274	10.324	23.274	10.324	20.648	20.648	10.324	10.324	20.648
248	12.95	12.95	12.95	12.95	12.95	56.045	12.95	12.95	12.95	12.95	12.95	12.95	12.95	12.95	12.95	12.95	12.95	12.95	12.95	12.95	12.95	12.95	12.95	12.95	12.95	56.045	12.95	12.95	12.95	12.95	12.95
249	25.857	25.857	25.857	25.857	25.857	41.539	25.857	25.857	25.857	25.857	25.857	25.857	25.857	25.857	25.857	25.857	25.857	25.857	25.857	51.714	25.857	25.857	25.857	25.857	25.857	41.539	25.857	25.857	25.857	25.857	25.857
250	23.165	54.566	14.463	31.866	23.165	31.866	14.463	33.211	14.463	37.628	49.883	45.466	26.718	26.718	44.121	37.628	26.718	26.718	26.718	52.822	35.42	66.821	26.718	44.121	35.42	44.121	26.718	45.466	26.718	49.883	62.138
251	74.26	36.765	36.765	36.765	36.765	36.765	36.765	36.765	74.26	73.529	61.275	24.51	24.51	24.51	24.51	85.784	24.51	24.51	70.706	24.51	62.005	24.51	24.51	24.51	24.51	24.51	24.51	24.51	62.005	61.275	49.02
252	0	19.761	0	0	0	0	0	0	0	0	0	0	26.363	0	0	0	0	0	0	0	0	19.761	0	0	0	0	0	0	0	0	0
[truncated: 156,485 more chars]
